# Supplementary material for: Silver-assisted gold-catalyzed formal synthesis of the anticoagulant Fondaparinux pentasaccharide
Source: Commun Chem. 2021 Feb 15;4:15. doi: 10.1038/s42004-021-00452-y (PMC9814392; doi:10.1038/s42004-021-00452-y)

Supporting Information for

Silver-assisted gold-catalyzed formal synthesis of the anticoagulant Fondaparinux pentasaccharide

Gulab Walke, Niteshlal Kasdekar, Yogesh Sutar, Srinivas Hotha*

Department of Chemistry, Indian Institute of Science Education and Research (IISER), Pune – 411 008, MH, India

s.hotha@iiserpune.ac.in

Contents

S. No.	Description	Page Number
1.	Schemes for supplementary information	S2-S5
2.	Experimental Procedures and characterization data	S6-S82
3.	Spectral charts	S83-S519

Supplimentary Figures Required for the Experimental Procedures

Scheme S1. Preparation of Iduronic acid donor

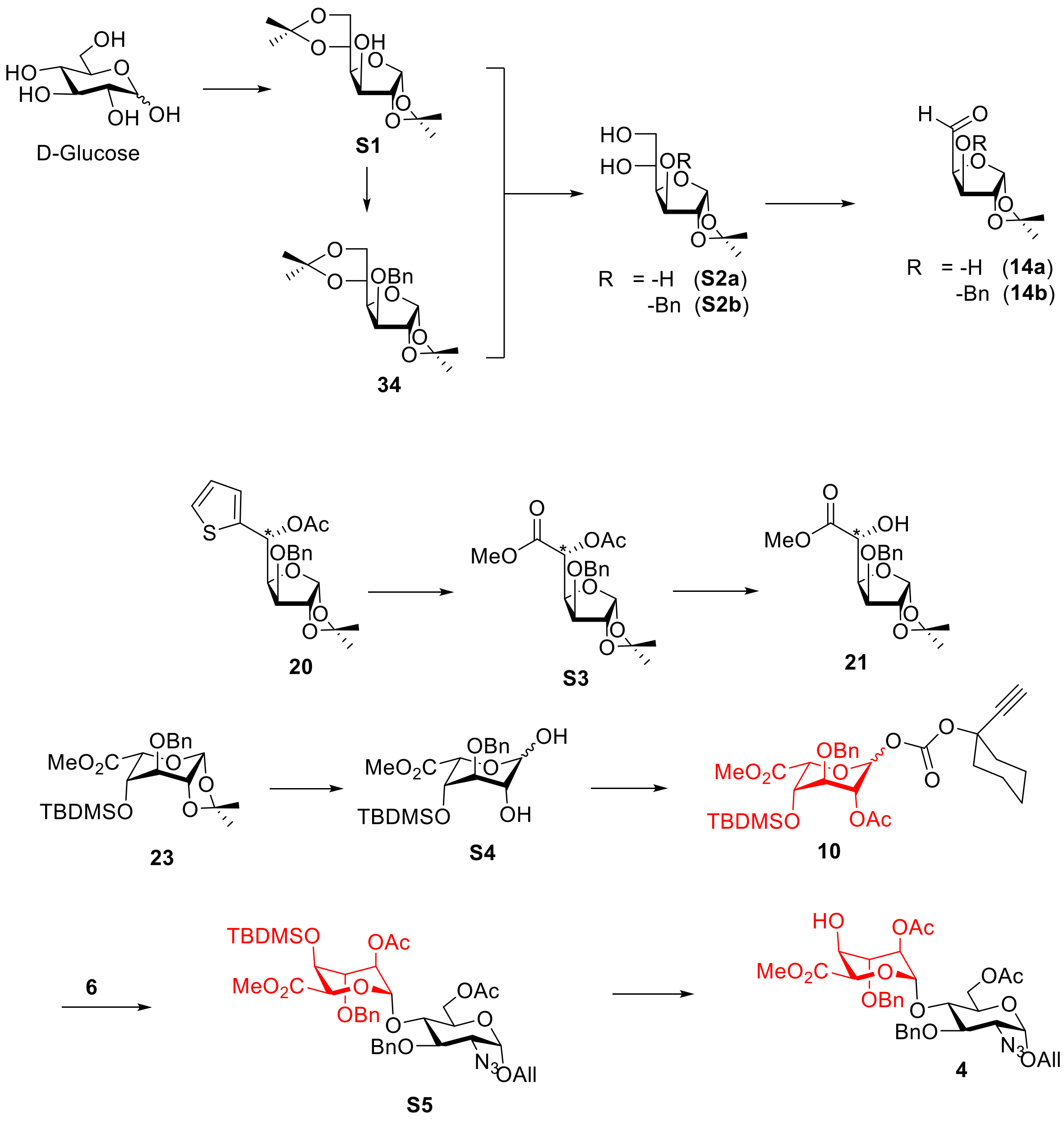

Scheme S2. Synthesis of Glucosamine Building blocks

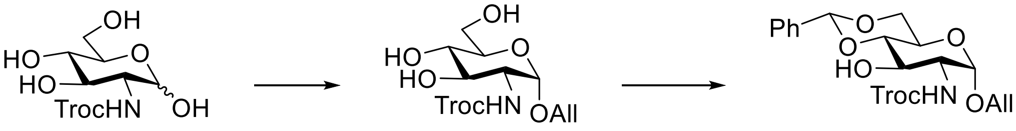

26 **S6** **27**

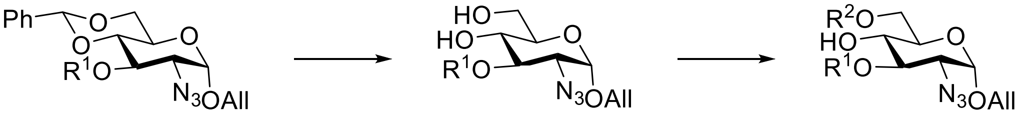

30a R1 = PMB
30b R1 = Bn
30c R1 = Nap
30d R1=TBDPS
30e R1= Ac

S7a R1 = PMB
S7b R1 = Bn
S7c R1 = Nap
S7d R1=TBDPS
S7e R1= Ac

31a R1 = PMB, R2 = Bz
6 R1 = Bn, R2 = Ac
31b R1 = Bn, R2 = TBDPS
31c R1 = PMB, R2 = TBDPS
31d R1 = Nap, R2 = TBDPS
31e R1 = Ac, R2 = TBDPS
31f R1, R2 = TBDPS

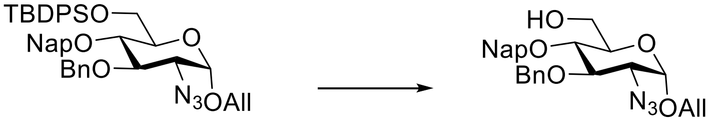

32a **S8**

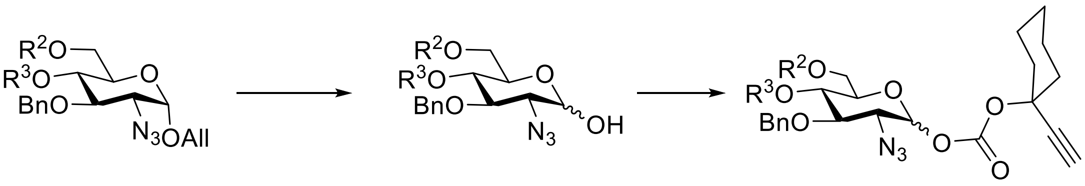

R2 = TBDPS, R3 = Nap (**32a**)
R2 = TBDPS, R3 = PMB (**32b**)
R2 = TBDPS, R3 = Bn (**32c**)

R2 = Ac, R3 = Nap (**32d**)
R2 = Lev, R3 = Nap (**32e**)

R2 = TBDPS, R3 = Nap (**S9a**)
R2 = TBDPS, R3 = PMB (**S9b**)
R2 = TBDPS, R3 = Bn (**S9c**)

R2 = Ac, R3 = Nap (**S9d**)
R2 = Lev, R3 = Nap (**S9e**)

R2 = TBDPS, R3 = Nap (**33a**)
R2 = TBDPS, R3 = PMB (**33b**)
R2 = TBDPS, R3 = Bn (**33c**)

R2 = Ac, R3 = Nap (**33d**)
R2 = Lev, R3 = Nap (**33e**)

Scheme S3. Synthesis of Glucuronic acid Building Blocks

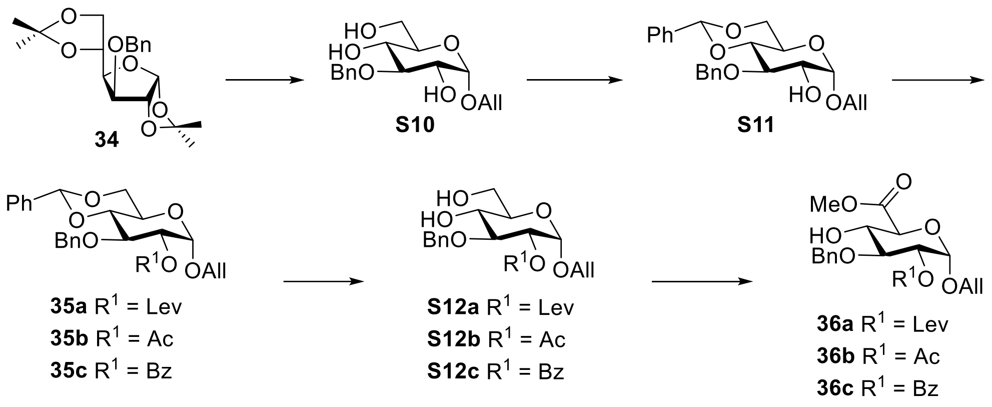

Scheme S4. Synthesis of DEFG Tetrasaccharide 49

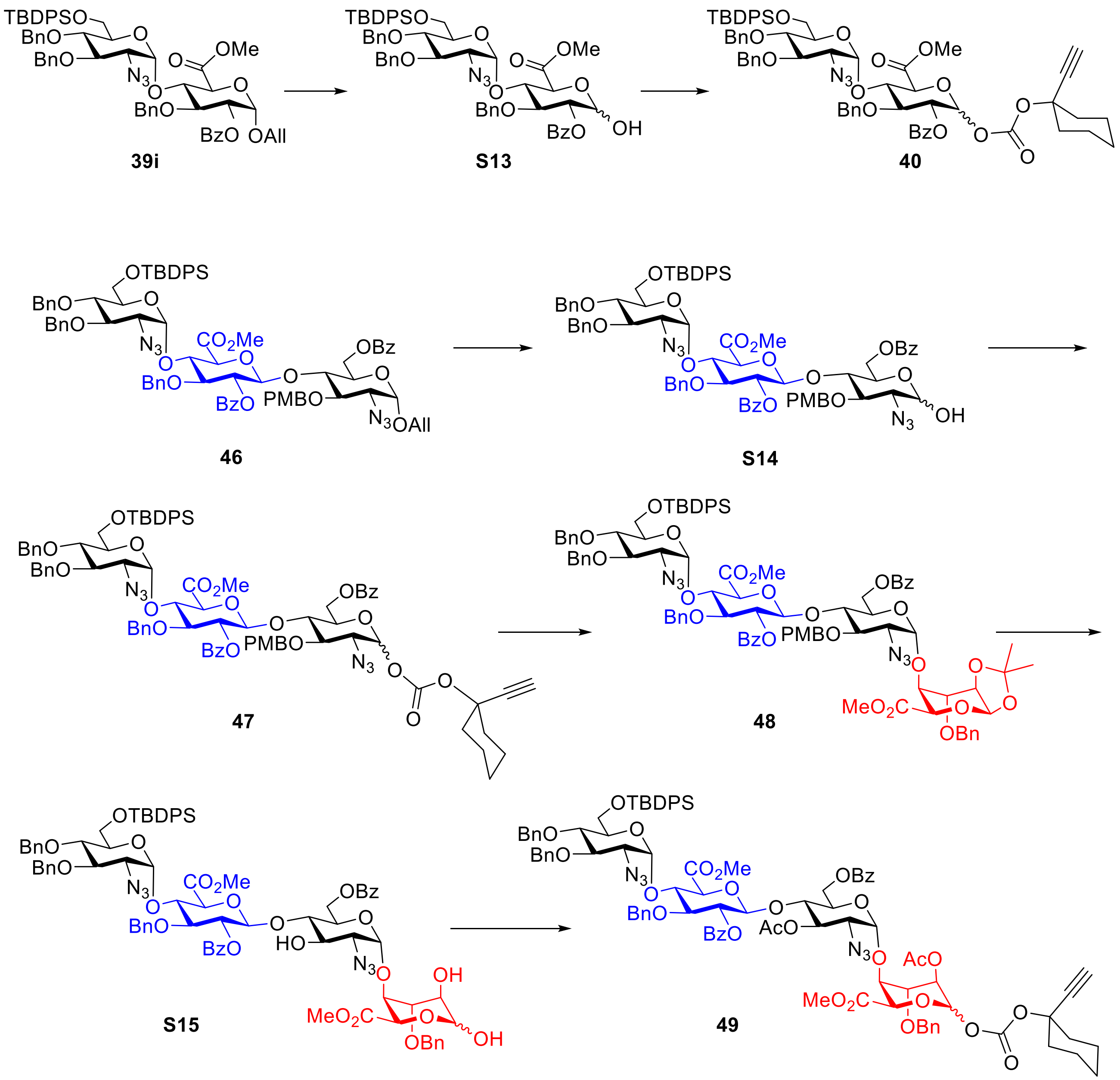

Supplementary methods

Experimental Procedures and Characterization Data

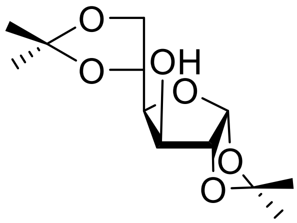

1,2:5,6 Di-*O*-isopropylidene-α-D-glucofuranose (S1): To a solution of D-glucose (40 g, 222 mmol) and 2,2-dimethoxy propane (2.5 eq) in dry acetone (600 mL) was added 50 g of anhydrous $CuSO_4$. After vigorously stirring for 30 min, 2 mL of concentrated H_2SO_4 was added dropwise under argon atmosphere and the reaction mixture was stirred at 25 °C for 24 h. After completion, the reaction was neutralized with saturated aq. $NaHCO_3$ solution, acetone was removed at reduced pressure *in vacuo* and the product was extracted with CH_2Cl_2. The organic layer was dried over anhydrous Na_2SO_4 and concentrated *in vacuo*. The crude product was precipitated by the addition of CH_2Cl_2 and hexane to afford 1,2:5,6-Di-*O*-isopropylidene-α-D-glucofuranose **S1** (37.6 g, 65%) as a fluffy white solid. mp 105.2 °C; $[\alpha]^{25}_D$ ($CHCl_3$, *c*1.0): -11.8°; IR (cm^{-1}, $CHCl_3$) : 3429, 2987, 2945, 1377, 1220, 1069, 1025, 851, 786, 754; ^1H NMR (400.31 MHz, $CDCl_3$): δ 5.93 (d, *J* = 3.4 Hz, 1H), 4.52 (d, *J* = 3.5 Hz, 1H), 4.35 – 4.30 (m, 2H), 4.16 (dd, *J* = 8.5, 6.3 Hz, 1H), 4.05 (dd, *J* = 7.7, 2.5 Hz, 1H), 3.98 (dd, *J* = 8.6, 5.4 Hz, 1H), 2.69 – 2.63 (m, 1H), 1.49 (s, 3H), 1.43 (s, 3H), 1.35 (s, 3H), 1.31 (s, 3H); ^{13}C NMR (100.67 MHz, $CDCl_3$): δ 111.8, 109.7, 105.3, 85.1, 81.1, 75.2, 73.4, 67.6, 26.8, 26.8, 26.2, 25.1; HRMS (ESI-MS): m/z calcd for $C_{12}H_{20}O_6$ $[M+H]^+$: 261.1338, Found: 261.1336.

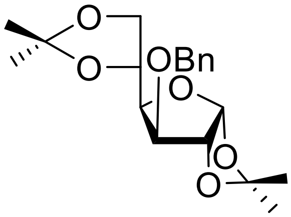

1,2:5,6 Di-*O*-isopropylidene-3-*O*-benzyl-α-D-glucofuranose (34): To a solution of compound **S1** (60 g, 231 mmol) in anhydrous THF (300 mL), NaH (60% in mineral oil, 1.2 eq) was added in portions under argon atmosphere. After evolution of the hydrogen ceased, benzyl bromide (1.1 eq) was

added dropwise, followed by the addition of catalytic amount of tetrabutylammonium iodide (0.05 eq) and the mixture was stirred at 25 °C for 3 h. After completion, the reaction mixture was quenched by the addition of ice-cold water (500 mL) and extracted with EtOAc (3 x 300 mL). The combined organic phases were washed with brine, dried over anhydrous Na_2SO_4 and concentrated *in vacuo*.The crude residue was purified by silica gel column chromatography using 8% ethyl acetate in *n*-hexane as a mobile phase to afford compound **34** (74g, 92%) as a thick syrup. $[\alpha]^{25}_D$ ($CHCl_3$, *c*1.0): -30.0º; IR (cm^{-1}, $CHCl_3$): 2986, 2936, 1377, 1255, 1076, 1023, 851, 748;^1H NMR (400.31 MHz, $CDCl_3$): δ 7.37 – 7.27 (m, 5H), 5.90 (d, *J* = 3.7 Hz, 1H), 4.71 – 4.62 (m, 2H), 4.59 (d, *J* = 3.7 Hz, 1H), 4.37 (dt, *J* = 7.7, 6.0 Hz, 1H), 4.15 (dd, *J* = 7.8, 3.1 Hz, 1H), 4.12 (dd, *J* = 8.6, 6.2 Hz, 1H), 4.04 – 3.99 (m, 2H), 1.50 (s, 3H), 1.43 (s, 3H), 1.38 (s, 3H), 1.31 (s, 3H);^{13}C NMR (100.67 MHz, $CDCl_3$): δ 137.7, 128.4, 128.4, 127.8, 127.7, 127.7, 111.8, 109.0, 105.3, 82.7, 81.7, 81.3, 72.5, 72.4, 67.4, 26.9, 26.8, 26.3, 25.5; HRMS (ESI-MS): m/z calcd for $C_{19}H_{26}O_6$ $[M+Na]^+$: 373.1627, Found: 373.1638.

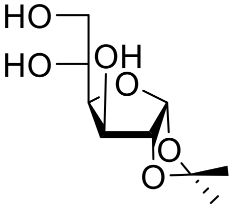

1,2-*O*-isopropylidene-α-D-glucofuranose (S2a): Diacetonide **S1** (20 g, 76.84 mmol) was dissolved in 120 mL of the 66.6% aqueous acetic acid and the mixture was stirred at 50 ºC for 8 h. After removal of the solvent under reduced pressure, the crude residue was co-evaporated twice with water and twice with toluene and the resulting residue was purified by silica gel column chromatography to afford 14 g (83%) of the diol compound **S2a** as a white solid. Eluent for purification: 10% MeOH in CH_2Cl_2; mp 158.7 ^{0}C; $[\alpha]^{25}_D$ ($CHCl_3$, *c*0.5): -14.4^0; IR (cm^{-1}, $CHCl_3$): 3691, 3023, 1595, 1426, 1216, 1023, 928, 770, 672;^1H NMR (399.78 MHz, CD_3OD): δ 5.86 (d, *J* = 3.7 Hz, 1H), 4.47 (d, *J* = 3.7 Hz, 1H), 4.20 (d, *J* = 2.6 Hz, 1H), 4.01 (dd, *J* = 8.4, 2.7 Hz, 1H), 3.88 (ddd, *J* = 8.7, 6.0, 3.1 Hz, 1H), 3.76 (dd, *J* = 11.6, 3.1 Hz, 1H), 3.59 (dd, *J* = 11.5, 6.0 Hz, 1H), 1.45 (s, 3H), 1.29 (s, 3H); ^{13}C

NMR (100.53 MHz, CD_3OD): δ 112.7, 106.4, 86.5, 81.3, 75.4, 70.4, 65.3, 27.0, 26.4; HRMS (ESI-MS): m/z calcd for $C_9H_{16}O_6$ $[M+Na]^+$: 243.0845, Found: 243.0851.

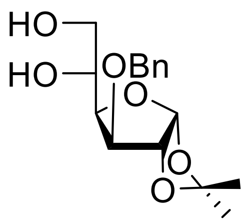

1,2-*O*-isopropylidene-3-*O*-benzyl-α-D-glucofuranose (S2b): This compound was prepared from **34** following the procedure invoked for the preparation of compound **S2a**. Eluent for purification: 40% ethyl acetate in *n*-hexane; thick syrup; yield 30 g (89%) from 38 g; $[\alpha]^{25}_D$ ($CHCl_3$, *c*1.0): -55.8º; IR (cm^{-1}, $CHCl_3$): 3387, 2932, 1639, 1457, 1253, 1216, 1077, 1019, 894, 858, 742, 700; ^1H NMR (399.78 MHz, $CDCl_3$): δ 7.38 – 7.27 (m, 5H), 5.91 (d, *J* = 3.8 Hz, 1H), 4.70 (d, *J* = 11.8 Hz, 1H), 4.60 (d, *J* = 3.8 Hz, 1H), 4.56 (d, *J* = 11.8 Hz, 1H), 4.14 – 4.08 (m, 2H), 4.02 (m, 1H), 3.79 (d, *J* = 9.3 Hz, 1H), 3.67 (dd, *J* = 11.5, 5.4 Hz, 1H), 2.94 (d, *J* = 5.6 Hz, 1H), 2.84 (s, 1H), 1.47 (s, 3H), 1.30 (s, 3H); ^{13}C NMR (100.53 MHz, $CDCl_3$): δ 137.3, 128.7, 128.7, 128.2, 127.9, 127.9, 111.9, 105.2, 82.2, 82.0, 80.0, 72.2, 69.2, 64.4, 26.8, 26.3; HRMS (ESI-MS): m/z calcd for $C_{16}H_{22}O_6$ $[M+Na]^+$: 333.1314, Found: 333.1320.

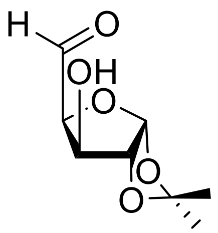

1,2-*O*-(isopropylidene)-α-D-xylo-pentodialdo-1,4-furanose (14a): Compound **S2a** (12g, 54.49 mmol) was dissolved in 360 mL of methanol and the mixture was cooled to 0 ºC. $NaIO_4$ (1.2 eq) was dissolved in 120 mL of water, slowly added to the reaction mixture and stirred for 45 min. After completion, the reaction mixture was filtered through a bed of Celite®, the filtrate was concentrated *in vacuo* and extracted with CH_2Cl_2 (3 x 200 mL). The combined organic layers were washed with brine solution (200 mL), dried over anhydrous Na_2SO_4 and concentrated *in vacuo*. The crude residue was passed through a bed of silica gel to obtain the desired aldehyde **14a** (6.7g, 65%).

1,2-*O*-(isopropylidene)-3-*O*-benzyl-α-D-xylo-pentodialdo-1,4-furanose (14b): This compound was prepared from **S2b** by following the procedure adopted for the preparation of compound **14a**. Eluent for purification: 40% ethyl acetate in *n*-hexane; thick syrup; yield 26 g (quantitative) from 29 g; $[\alpha]^{25}_{D}$ ($CHCl_3$, *c*1.0): -45.2^0; IR (cm^{-1}, $CHCl_3$): 3485, 2988, 2938, 1737, 1456, 1380, 1260, 1078, 1021, 752; ^{1}H NMR (399.78 MHz, $CDCl_3$): δ 9.67 (d, *J* = 1.5 Hz, 1H), 7.37 – 7.29 (m, 4H), 7.25 – 7.23 (m, 1H), 6.12 (d, *J* = 3.5 Hz, 1H), 4.65 (d, *J* = 3.5 Hz, 1H), 4.61 (d, *J* = 11.8 Hz, 1H), 4.57 (dd, *J* = 3.8, 1.5 Hz, 1H), 4.48 (d, *J* = 11.9 Hz, 1H), 4.34 (d, *J* = 3.8 Hz, 1H), 1.47 (s, 3H), 1.33 (s, 3H).; ^{13}C NMR (100.53 MHz, $CDCl_3$):δ 200.0, 136.7, 128.7, 128.7, 128.3 127.8, 127.8, 112.7, 106.3, 84.7, 83.8, 82.3, 72.4, 27.1, 26.4; HRMS (ESI-MS): m/z calcd for $C_{15}H_{18}O_5$ $[M+H]^+$: 279.1232, Found: 279.1233.

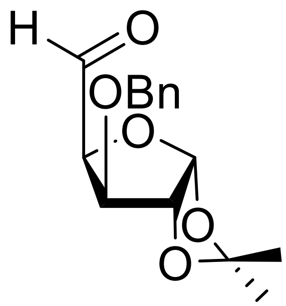

15a:16a Grignard Reaction: Aldehyde **14a** (3.5 g, 18.6 mmol) was dissolved in 50 mL of anhydrous THF and added slowly to the freshly prepared Grignard reagent (1.5 eq) at 0 ºC under argon atmosphere. The reaction mixture was allowed to stir at 25 ºC for 3 h. After completion, the reaction was quenched by the addition of saturated aqueous ammonium chloride solution (20 mL) and extracted with EtOAc (3 x 30 mL). The combined organic phases were washed with brine solution (100 mL), dried over anhydrous Na_2SO_4 and concentrated *in vacuo*. The crude residue was purified by silica gel column chromatography using EtOAc and hexane (30-35%) as a mobile phase to afford **15a**:**16a** (4.21 g, 85%, 10:1) as corresponding Ido:Glc isomers. **Grignard Reagent Preparation:** Mg-metal turnings (1.5 eq) were freshly activatived and suspended in anhydrous THF (50 mL) in a two neck round bottom flask equipped with a reflux condenser, added phenyl bromide (1.5 eq) slowly under argon atmosphere and the mixture was stirred at 70 ºC for 1h.

5*S*-1,2-O-(isopropylidene)-5-*C*-phenyl-α-D-xylofuranose (15a): White solid; mp 156.8 ºC; $[\alpha]^{25}_{D}$ ($CHCl_3$, *c*1.0): +16.0^0; IR (cm^{-1}, $CHCl_3$): 3386, 3020, 1589, 1424, 1218, 772; ^1H NMR (400.31 MHz, $CDCl_3$): δ 7.49 – 7.46 (m, 2H), 7.40 – 7.30 (m, 3H), 6.03 (d, *J* = 3.6 Hz, 1H), 5.05 (t, *J* = 4.9 Hz, 1H), 4.51 (d, *J* = 3.6 Hz, 1H), 4.34 (dd, *J* = 4.7, 2.8 Hz, 1H), 4.13 – 4.09 (m, 1H), 3.18 (d, *J* = 4.8 Hz, 1H), 2.85 (d, *J* = 5.4 Hz, 1H), 1.46 (s, 3H), 1.31 (s, 3H); ^{13}C NMR (100.67 MHz, $CDCl_3$): δ 140.2, 128.8, 128.8, 128.5, 126.9, 126.9, 112.2, 105.2, 85.6, 82.8, 76.3, 72.8, 27.0, 26.4; HRMS (ESI-MS): m/z calcd for $C_{14}H_{18}O_5$ $[M+Na]^+$: 289.1052, Found: 289.1053.

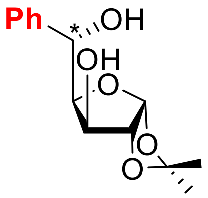

5*R*-1,2-*O*-(isopropylidene)-5-*C*-phenyl-α-D-xylofuranose (16a): Thick syrup; $[\alpha]^{25}_{D}$ ($CHCl_3$, *c* 0.8): -23.0^0; IR (cm^{-1}, $CHCl_3$): 3778, 3022, 1595, 1426, 1216, 1021, 929, 761, 670; ^1H NMR (400.31 MHz, $CDCl_3$): δ 7.46 – 7.30 (m, 5H), 6.03 (d, *J* = 3.6 Hz, 1H), 5.27 (d, *J* = 4.0 Hz, 1H), 4.49 (d, *J* = 3.6 Hz, 1H), 4.29 (s, 1H), 4.20 (dd, *J* = 4.0, 2.6 Hz, 1H), 4.15 (d, *J* = 2.1 Hz, 1H), 3.24 (s, 1H), 1.46 (s, 3H), 1.30 (s, 3H); ^{13}C NMR (100.67 MHz, $CDCl_3$): δ 139.1, 128.9, 128.4, 126.1, 111.9, 105.1, 85.3, 82.2, 75.5, 73.8, 26.9, 26.2; HRMS (ESI-MS): m/z calcd for $C_{14}H_{18}O_5$ $[M+Na]^+$: 289.1052, Found: 289.1054.

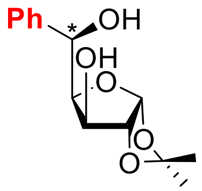

(15b:**16b)**:These compounds were prepared from **14b** following the procedure identified for the compounds **15a**:**16a**. Eluent for purification: 18-20% ethyl acetate in *n*-hexane; yield 530 mg (83%, 12:1) from 500 mg;

5*S*-1,2-*O*-(isopropylidene)-3-*O*-benzyl-5-*C*-phenyl-α-D-xylofuranose (15b): Thick syrup; $[\alpha]^{25}_{D}$ ($CHCl_3$, *c*1.0): -44.6º; IR (cm^{-1}, $CHCl_3$): 3779, 3022, 1593, 1427, 1216, 930, 766, 670; ^1H NMR (399.78 MHz, $CDCl_3$): δ 7.31 – 7.28 (m, 2H), 7.27 – 7.19 (m, 8H), 5.92 (d, *J* = 3.8 Hz, 1H), 4.96 (d, *J* = 7.6 Hz, 1H), 4.49

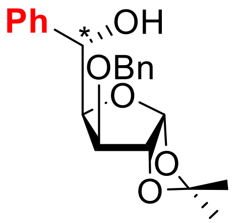

(d, J = 3.8 Hz, 1H), 4.43 (d, J = 11.5 Hz, 1H), 4.23 (dd, J = 7.6, 3.2 Hz, 1H), 4.18 (d, J = 11.5 Hz, 1H), 3.51 (d, J = 3.2 Hz, 1H), 2.93 (s, 1H), 1.39 (s, 3H), 1.20 (s, 3H); ^{13}C NMR (100.53 MHz, $CDCl_3$): δ 139.8, 137.0, 128.6, 128.6, 128.4, 128.4, 128.1, 128.0, 127.7, 127.7, 127.1, 127.1, 111.9, 105.2, 84.6, 82.2, 82.1, 72.4, 71.8, 26.8, 26.3; HRMS (ESI-MS): m/z calcd for $C_{21}H_{24}O_5$ $[M+Na]^+$: 379.1521, Found: 379.1523.

5*S*-1,2-*O*-(isopropylidene)-3-*O*-benzyl-5-*C*-phenyl-α-D-xylofuranose (16b): Thick syrup; $[\alpha]^{25}_D$ ($CHCl_3$, *c*1.0): -77.2º; IR (cm^{-1}, $CHCl_3$): 3688, 3022, 2929, 1595, 1427, 1216, 1079, 1026, 928, 767, 671;^{1}H NMR (399.78 MHz, $CDCl_3$): δ 7.39 – 7.28 (m, 10H), 6.03 (d, J = 3.9 Hz, 1H), 5.10 (t, J = 6.4 Hz, 1H), 4.69 (d, J = 11.5 Hz, 1H), 4.63 (d, J = 3.9 Hz, 1H), 4.46 (d, J = 11.5 Hz, 1H), 4.33 (dd, J = 6.2, 3.2 Hz, 1H), 4.00 (d, J = 3.2 Hz, 1H), 3.26 (d, J = 6.9 Hz, 1H), 1.47 (s, 3H), 1.31 (s, 3H); ^{13}C NMR (100.53 MHz, $CDCl_3$): δ 141.4, 136.8, 128.9, 128.9, 128.5 (3C), 128.2, 128.2, 127.8, 126.2, 126.2, 111.8, 105.3, 82.9, 82.6, 81.8, 72.4, 72.2, 26.9, 26.3; HRMS (ESI-MS): m/z calcd for $C_{21}H_{24}O_5$ $[M+Na]^+$: 379.1521, Found: 379.1515.

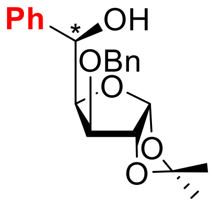

5*S*-1,2,3,4-tetra-*O*-acetyl-5-*C*-Phenyl xylopyranoside (17a): Compound **15a** (440 mg, 1.30 mmol) was dissolved in 90% aqueous trifluoroacetic acid (3 mL) and stirred for 15 min at room temperature. The solvent was evaporated and the residue was co-evaporated twice with water and twice with toluene to afford the crude product which was redissolved in anhydrous pyridine (5 mL), acetic anhydride (1.2 eq) was added dropwise at 0 ºC under argon atmosphere, followed by the addition of a catalytic amount of DMAP. The reaction mixture was gradually warmed up to 25 °C and stirred for 3 h. After completion, ice-cooled water (50mL) was added and extracted with EtOAc (2 x 25 mL). The organic layer was washed with aqueous 1*N* HCl (2 x 50 mL) and

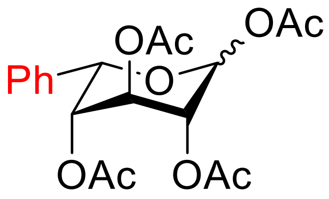

brine solution (1x50 mL), dried over anhydrous Na_2SO_4, concentrated *in vacuo* and the crude residue was purified by silica gel column chromatography using 25% ethyl acetate in *n*-hexane as a mobile phase to obtain **17a** (410 mg, 74%, over two steps) as a thick syrup. $[\alpha]^{25}_{D}$ ($CHCl_3$, *c*2.0): +23.0^0; IR (cm^{-1}, $CHCl_3$): 3022, 1756, 1594, 1427, 1216, 1044, 929, 761, 670;^{1}H NMR (400.31 MHz, $CDCl_3$): δ 7.40 – 7.37 (m, 2H), 7.35 – 7.32 (m, 2H), 7.31 – 7.29 (m, 1H), 6.18 (d, *J* = 1.8 Hz, 1H), 5.24 (t, *J* = 3.1 Hz, 1H), 5.17 (d, *J* = 1.8 Hz, 1H), 5.08 – 5.06 (m, 1H), 5.05 – 5.03 (m, 1H), 2.20 (s, 3H), 2.18 (s, 3H), 2.09 (s, 3H), 1.88 (s, 3H); ^{13}C NMR (100.53 MHz, $CDCl_3$): δ 169.7, 169.3, 168.9, 168.5, 135.7, 128.3 (3C), 126.5, 126.5, 90.6, 76.1, 68.4, 67.7, 65.7, 21.0 (3C), 20.5; HRMS (ESI-MS): m/z calcd for $C_{19}H_{22}O_9$ [M+Na]$^+$: 417.1162, Found: 417.1165.

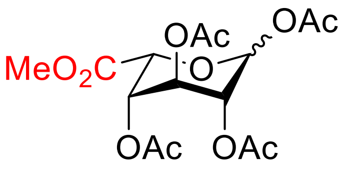

5*S*-1,2,4-tri-*O*-acetyl-3-*O*-benzyl-5-*C*-Phenyl xylopyranoside (17b): This compound was prepared from **15b**, following the procedure developed for compound **17a**. Eluent for purification: 20% ethyl acetate in *n*-hexane; Thick syrup; yield 190 mg (69%) from 120 mg; $[\alpha]^{25}_{D}$ ($CHCl_3$, *c*2.0): +10.9^o; IR (cm^{-1}, $CHCl_3$): 3024, 1755, 1599, 1431, 1214, 1110, 1050, 931, 758, 669;^{1}H NMR (400.31 MHz, $CDCl_3$): δ 7.40 (s, 1H), 7.39 (s, 2H), 7.36 – 7.32 (m, 5H), 7.31 – 7.30 (m, 2H), 6.25 (d, *J* = 1.7 Hz, 1H), 5.22 (d, *J* = 1.3 Hz, 1H), 5.11 (ddd, *J* = 2.7, 1.7, 0.9 Hz, 1H), 5.06 – 5.04 (m, 1H), 4.82 (d, *J* = 11.8 Hz, 1H), 4.76 (d, *J* = 11.8 Hz, 1H), 3.95 (t, *J* = 2.8 Hz, 1H), 2.16 (s, 3H), 2.14 (s, 3H), 1.86 (s, 3H); ^{13}C NMR (100.67 MHz, $CDCl_3$): δ 170.2, 169.9, 168.9, 137.2, 136.3, 128.8, 128.8, 128.4, 128.2, 128.2, 128.0(3C), 126.5, 126.5, 90.9, 75.8, 74.3, 73.1, 68.5, 66.7, 21.1, 21.1, 20.7; HRMS (ESI-MS): m/z calcd for $C_{24}H_{26}O_8$[M+Na]$^+$: 465.1525, Found: 465.1531.

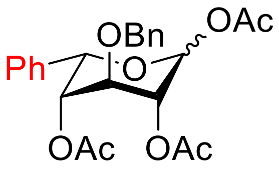

Methyl 1,2,3,4-tetra-O-acetyl-L-idopyranuronate (18a): To a solution of **17a** (200 mg, 0.507 mmol) in CCl_4:CH_3CN (20 mL, 1:1) was added solution of $NaIO_4$ (8 eq) in H_2O (15 mL) at 25 °C

and stirred for 10 min. $RuCl_3{\bullet}3H_2O$ (5 mol%) was added and the mixture was stirred vigorously for 24 h. NaCl was added to saturate the aqueous layer and extracted with CH_2Cl_2 (3 × 50 mL). Combined organic phases were washed with brine solution (50 mL), dried over Na_2SO_4 and concentrated *in vacuo*. To the solution of crude product in anhydrous DMF (5 mL) was added 1.5 equivalent of K_2CO_3. After stirring for 15 min, iodomethane (2 eq) was added under argon atmosphere and the mixture was stirred at 25 °C for 8 h in a dark place. After complete consumption, the reaction was quenched by the addition of excess amount of saturated aqueous solution of Na_2SO_3, followed by water (10 mL) and extracted with EtOAc (2 x 20 mL). The combined organic phases were washed with water, dried over anhydrous Na_2SO_4 and concentrated under reduced pressure. The crude residue was purified by column chromatography using 35% ethyl acetate in *n*-hexane as a mobile phase to obtain the **18a** (138 mg, 72%). Thick syrup; $[\alpha]^{25}_D$ ($CHCl_3$, *c*0.8): -2.0°; IR (cm^{-1}, $CHCl_3$): 3022, 2926, 1761, 1594, 1427, 1216, 1056, 929, 766, 670; ^1H NMR (400.31 MHz, $CDCl_3$): δ 6.05 (d, *J* = 1.8 Hz, 1H), 5.28 (t, *J* = 3.5 Hz, 1H), 5.10 (t, *J* = 2.5 Hz, 1H), 5.03 – 4.99 (m, 1H), 4.71 (d, *J* = 2.3 Hz, 1H), 3.78 (s, 3H), 2.16 (s, 3H), 2.14 (s, 3H), 2.11 (s, 3H), 2.08 (s, 3H); ^{13}C NMR (100.53 MHz, $CDCl_3$): δ 169.6, 169.4, 168.7, 168.3, 166.9, 89.9, 73.5, 67.3, 66.4, 65.4, 52.9, 20.9 (3C), 20.7; HRMS (ESI-MS): m/z calcd for $C_{15}H_{20}O_{11}$ $[M+Na]^+$: 399.0903, Found: 399.0909.

19: Grignard Reaction: Aldehyde **14b** (10 mmol) was dissolved in 30 mL of the anhydrous Et_2O or THF and added dropwise to the freshly prepared Grignard reagent (1.5 eq) at 0°C under argon atmosphere. Reaction mixture was allowed to stir at 25 °C for 3 h. After completion, the reaction was quenched by the addition of excess amount of saturated aqueous ammonium chloride solution, water and extracted with EtOAc (3 x 100 mL). The combined organic phases were washed with brine solution (100 mL), dried over Na_2SO_4 and concentrated *in vacuo*. The crude residue was purified by silica gel column chromatography using 10-15% ethyl acetate in

n-hexane as mobile phase to afford the compound **19** [89% (10:1) when Et_2O as a solvent whereas 82% (5:1) when THF as a solvent] as Ido:Glc isomers. **2-Thienylmagnesium bromide Preparation:** To freshly activated Mg-metal turnings (1.5 eq) suspended in 30 mL of anhydrous Et_2O or THF in a two neck flask equipped with a reflux condenser was added 2-thienyl bromide (1.5 eq) slowly under argon atmosphere and mixture was stirred at 45 °C for 1 h.

5*R*-1,2-*O*-isopropylidene-3-*O*-benzyl-5-*C*-[2-thienyl]-α-D-xylofuranose 19 (Ido isomer):

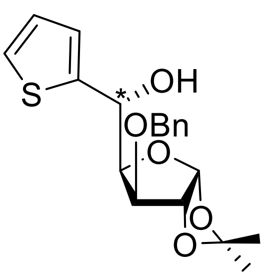

Thick syrup; $[\alpha]^{25}_D$ ($CHCl_3$, *c*1.0): -46.3°; IR (cm^{-1}, $CHCl_3$): 3496, 3010, 2923, 1456, 1379, 122, 1215, 1075, 1018, 746, 703; ^1H NMR (399.78 MHz, $CDCl_3$): δ 7.38 – 7.25 (m, 6H), 7.01 (d, *J* = 3.4 Hz, 1H), 6.95 (dd, *J* = 5.0, 3.5 Hz, 1H), 6.02 (d, *J* = 3.8 Hz, 1H), 5.35 (d, *J* = 7.6 Hz, 1H), 4.63 (d, *J* = 3.8 Hz, 1H), 4.58 (d, *J* = 11.5 Hz, 1H), 4.41 – 4.35 (m, 2H), 3.80 (d, *J* = 3.3 Hz, 1H), 2.99 (s, 1H), 1.50 (s, 3H), 1.32 (s, 3H); ^{13}C NMR (100.53 MHz, $CDCl_3$): δ 142.6, 137.1, 128.7, 128.7, 128.2, 127.7, 127.7, 126.7, 125.3, 125.2, 112.2, 105.4, 84.6, 82.3, 82.3, 72.0, 68.7, 27.0, 26.4; HRMS (ESI-MS): m/z calcd for $C_{19}H_{22}O_5S[M+Na]^+$: 385.1086, Found: 385.1086.

5*S*-1,2-O-isopropylidene-3-*O*-benzyl-5-*C*-[2-thienyl]-α-D-xylofuranose 19 (Glc isomer):

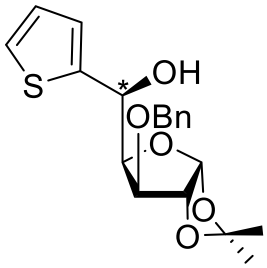

Thick syrup; $[\alpha]^{25}_D$ ($CHCl_3$, *c*0.45): -61.4°; IR (cm^{-1}, $CHCl_3$): 3489, 3010, 2929, 1498, 1379, 1261, 1217, 1077, 1025, 753, 703; ^1H NMR (400.31 MHz, $CDCl_3$): δ 7.39 – 7.30 (m, 5H), 7.26 (dd, *J* = 5.0, 1.2 Hz, 1H), 7.02 (dt, *J* = 2.5, 1.0 Hz, 1H), 6.98 (dd, *J* = 5.0, 3.5 Hz, 1H), 6.03 (d, *J* = 3.8 Hz, 1H), 5.31 (t, *J* = 6.4 Hz, 1H), 4.68 (d, *J* = 11.4 Hz, 1H), 4.64 (d, *J* = 3.8 Hz, 1H), 4.52 (d, *J* = 11.4 Hz, 1H), 4.38 (dd, *J* = 6.2, 3.3 Hz, 1H), 4.16 (d, *J* = 3.3 Hz, 1H), 3.38 (d, *J* = 7.1 Hz, 1H), 1.49 (s, 3H), 1.32 (s, 3H); ^{13}C NMR (100.67 MHz, $CDCl_3$): δ 145.4, 136.8, 128.9, 128.9, 128.5, 128.1, 128.1, 127.0, 125.0, 124.5, 112.0, 105.4, 83.1, 82.6, 81.9, 72.5, 69.0, 27.0, 26.4; HRMS (ESI-MS): m/z calcd for $C_{19}H_{22}O_5S[M+Na]^+$: 385.1086, Found: 385.1083.

5*R*-1,2-*O*-isopropylidene-3-*O*-benzyl-5-*C*-[2-thienyl]-5-*O*-acetyl-α-Dxylofuranose 20 (Ido isomer): To a solution of 13.0 g (35.9 mmol) of the compound **19** in anhydrous CH_2Cl_2 (65 mL) was added DMAP (1.2 eq) under argon atmosphere, followed by dropwise addition of the acetic anhydride (1.5 eq). The reaction mixture was stirred at 25 °C for 1 h. After completion, the mixture was purified by silica gel column chromatography using 18% ethyl acetate in *n*-hexane as a mobile phase to obtain compound **20** (13.8 g, 95%) as a thick syrup. $[\alpha]^{25}_D$ ($CHCl_3$, *c*1.0): +20.8º; IR (cm^{-1}, $CHCl_3$): 2988, 2933, 1742, 1453, 1375, 1226, 1076, 1018, 752, 705; ^1H NMR (400.31 MHz, $CDCl_3$): δ 7.35 – 7.21 (m, 6H), 7.10 – 7.05 (m, 1H), 6.94 (dd, *J* = 5.1, 3.5 Hz, 1H), 6.40 (d, *J* = 9.3 Hz, 1H), 6.00 (d, *J* = 3.8 Hz, 1H), 4.62 (dd, *J* = 9.3, 3.3 Hz, 1H), 4.56 (d, *J* = 3.8 Hz, 1H), 4.43 (d, *J* = 11.5 Hz, 1H), 4.21 (d, *J* = 11.4 Hz, 1H), 3.67 (d, *J* = 3.3 Hz, 1H), 2.05 (s, 3H), 1.54 (s, 3H), 1.31 (s, 3H); ^{13}C NMR (100.67 MHz, $CDCl_3$): δ 170.0, 139.1, 137.1, 128.5, 128.5, 128.0, 127.7, 127.7, 127.2, 126.7, 125.9, 112.0, 105.7, 82.2, 82.2, 81.8, 72.1, 69.7, 27.0, 26.4, 21.4; HRMS (ESI-MS): m/z calcd for $C_{21}H_{24}O_6S[M+Na]^+$: 427.1191, Found: 427.1187.

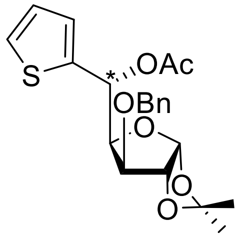

5*R*-1,2-*O*-isopropylidene-3-*O*-benzyl-5-*C*-[methoxycarbonyl]-5-*O*-acetyl-α-D-xylofuranose (S3): To a solution of compound **20** (13.2 g, 32.63 mmol) in hexane–EtOAc (240 mL, 1:3) was added a solution of $NaIO_4$ (8 eq) in H_2O (240 mL) at 25 °C and stirred for 10 min. Catalyst $RuCl_3$•$3H_2O$ (5 mol%) was added and the mixture was stirred vigorously for 24 h. NaCl was added to saturate the aqueous layer, two phases were separated and the aqueous layer was extracted with EtOAc (2 × 100 mL). The combined organic phases were washed with brine solution (100 mL), dried over Na_2SO_4 and concentrated *in vacuo*. The crude product was redissolved in anhydrous DMF (100 mL) and added 1.5 equivalent of K_2CO_3. After stirring for

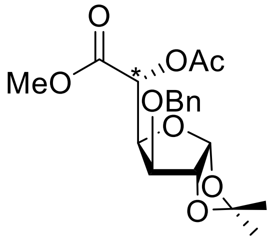

15 min, iodomethane (2 eq) was added under argon atmosphere and the mixture was stirred at 25 °C for 8 h in a dark place. After complete consumption, the reaction was quenched by adding excess of saturated aqueous solution of Na_2SO_3, water (200 mL) and extracted by EtOAc (2 x 200 mL). The combined organic phases washed with brine, dried over anhydrous Na_2SO_4 and concentrated under reduced pressure. The crude residue was purified by column chromatography using 18% ethyl acetate in *n*-hexane as a mobile phase to obtain the desired compound **S3** (9.2 g, 74%) over two steps. Thick syrup; $[\alpha]^{25}_D$ ($CHCl_3$, *c*1.8): -3.4º; IR (cm^{-1}, $CHCl_3$): 2956, 1747, 1446, 1376, 1216, 1076, 1024, 745, 700;H NMR (400.31 MHz, $CDCl_3$): δ 7.38 – 7.27 (m, 5H), 5.97 (d, *J* = 3.6 Hz, 1H), 5.52 (d, *J* = 7.1 Hz, 1H), 4.66 – 4.58 (m, 3H), 4.51 (d, *J* = 11.4 Hz, 1H), 4.16 (d, *J* = 3.8 Hz, 1H), 3.69 (s, 3H), 2.11 (s, 3H), 1.50 (s, 3H), 1.34 (s, 3H);^{13}C NMR (100.67 MHz, $CDCl_3$): δ 170.0, 168.5, 137.2, 128.6, 128.6, 128.1, 127.9, 127.9, 112.6, 105.3, 83.0, 82.7, 78.8, 72.5, 70.8, 52.7, 27.2, 26.7, 20.9; HRMS (ESI-MS): m/z calcd for $C_{19}H_{24}O_8$ $[M+Na]^+$: 403.1369, Found: 403.1369.

5*R*-1,2-*O*-isopropylidene-3-*O*-benzyl-5-*C*-[methoxycarbonyl]-α-D-xylofuranose (21): To a solution of the compound **S3** (9.18 g, 24.13 mmol) in anhydrous CH_3OH (30 mL, 1:1), freshly prepared 1*M* NaOMe in MeOH (0.3 eq) was added and the reaction mixture was stirred at 25 °C for 30 minutes. After complete consumption of the starting material, Amberlite IR 120® (H^+) (1.0 g per mol) was added to the reaction mixture to quench the NaOMe. After complete neutralization, the resin was filtered off and the filtrate was concentrated *in vacuo*, the crude product was purified by silica gel column chromatography to obtain the compound **21** (7.7 g, 94%). Eluent for purification: 30% ethyl acetate in *n*-hexane; thick syrup; $[\alpha]^{25}_D$ ($CHCl_3$, *c*1.0): -39.8º; IR (cm^{-1}, $CHCl_3$): 3448, 2988, 2954, 1740, 1451, 1378, 1255, 1214, 1075, 1025, 861, 743, 701;^{1}H NMR (399.78 MHz, $CDCl_3$): δ 7.36 – 7.27 (m, 5H), 5.99 (d, *J* = 3.9 Hz, 1H), 4.70 (d, *J* =

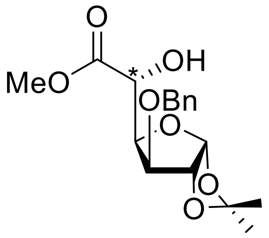

11.6 Hz, 1H), 4.66 (d, *J* = 3.8 Hz, 1H), 4.54 – 4.48 (m, 3H), 4.17 (d, *J* = 3.4 Hz, 1H), 3.71 (s, 3H), 3.40 (d, 1H), 1.46 (s, 3H), 1.32 (s, 3H); ^{13}C NMR (100.53 MHz, $CDCl_3$): δ 172.0, 136.8, 128.6, 128.6, 128.2, 128.0, 128.0, 112.5, 105.2, 83.0, 83.0, 80.3, 72.4, 69.9, 52.7, 27.1, 26.7; HRMS (ESI-MS): m/z calcd for $C_{17}H_{22}O_7$ $[M+Na]^+$: 361.1263, Found: 361.1266.

Methyl 1,2-O-isopropylidene-3 -O-benzyl-β-L-idopyranuronate 5: Compound **21** (1.54 g, 4.55 mmol) was dissolved in 90% aqueous trifluoroacetic acid (10 mL) and stirred for 15 min at room temperature. The solvent was evaporated and the residue was co-evaporated twice with water and twice with toluene. 2-Methoxypropene (8.7 mL, 20 eq) was added to the crude product in anhydrous THF (6 mL). The reaction mixture was cooled to 0 °C and PTSA (0.1 eq) in THF (0.5 mL) was added dropwise with stirring under argon atmosphere. Resulting solution was brought to room temperature and stirred for 4 h. The reaction was quenched by the addition of Et_3N, water (50 mL) and extracted with EtOAc (3 x 20 mL). The combined organic phases were washed with saturated aqueous $NaHCO_3$ and brine solution, dried over anhydrous Na_2SO_4 and concentrated *in vacuo*. The crude residue was purified by column chromatography using 20% ethyl acetate in *n*-hexane as a mobile phase to obtain 850 mg (55%, over two steps) of the desired compound **5** as athick syrup; $[\alpha]^{25}_D$ ($CHCl_3$, *c*1.0): -15.8º; IR (cm^{-1}, $CHCl_3$): 3535, 2924, 2857, 1763, 1737, 1458, 1379, 1229, 1119, 1061, 846, 703; ^1H NMR (400.31 MHz, $CDCl_3$): δ 7.40 – 7.30 (m, 5H), 5.36 (d, *J* = 1.9 Hz, 1H), 4.71 (d, *J* = 11.7 Hz, 1H), 4.64 (d, *J* = 11.7 Hz, 1H), 4.49 (s, 1H), 4.12 – 4.06 (m, 2H), 3.97 (q, *J* = 2.0 Hz, 1H), 3.81 (s, 3H), 3.12 (d, *J* = 11.3 Hz, 1H), 1.63 (s, 3H), 1.38 (s, 3H); ^{13}C NMR (100.67 MHz, $CDCl_3$): δ 169.4, 137.1, 128.8, 128.8, 128.4, 127.9, 127.9, 112.0, 96.5, 75.5, 73.5, 72.9, 72.2, 67.4, 52.5, 28.4, 25.7; HRMS (ESI-MS): m/z calcd for $C_{17}H_{22}O_7$ $[M+Na]^+$: 361.1263, Found: 361.1261.

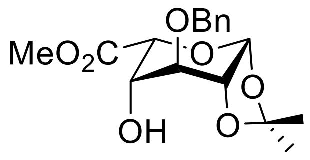

Methyl 1,2-*O*-isopropylidene-3-*O*-benzyl-4-*O*-$^{t-}$butyldimethylsilyl-β-L-idopyranuronate 23: To a solution of alcohol **5** (280 mg, 0.828 mmol) in anhydrous CH_2Cl_2 (5 mL) was added 5.0 equivalents of 2,6-lutidine. TBDMSOTf (1.2 eq) was added dropwise at 0 °C under argon atmosphere and the resulting reaction mixture was stirred at 25 °C. After 15 min, ice-cold water (10 mL) was added and extracted with CH_2Cl_2 (2 x 10 mL). The combined organic phases were washed with brine, dried over anhydrous Na_2SO_4 and concentrated *in vacuo*, crude residue was purified by silica gel column chromatography using 10% ethyl acetate in *n*-hexane as a mobile phase to obtain 350 mg (93%) of the compound **23** as a thick syrup; $[\alpha]^{25}_{D}$ ($CHCl_3$, *c*0.8): -61.4º; IR (cm^{-1}, $CHCl_3$): 2926, 2858, 1770, 1461, 1376, 1238, 1144, 1099, 1068, 835, 776; ^{1}H NMR (400.31 MHz, $CDCl_3$): δ 7.40 – 7.30 (m, 5H), 5.32 (d, *J* = 2.5 Hz, 1H), 4.65 (q, *J* = 12.0 Hz, 2H), 4.38 (d, *J* = 1.3 Hz, 1H), 4.07 (dt, *J* = 2.5, 1.2 Hz, 1H), 3.95 (t, *J* = 2.7 Hz, 1H), 3.83 (t, 1H), 3.76 (s, 3H), 1.59 (s, 3H), 1.38 (s, 3H), 0.82 (s, 9H), -0.05 (s, 3H), -0.07 (s, 3H); ^{13}C NMR (100.67 MHz, $CDCl_3$): δ 169.8, 137.4, 128.8, 128.3, 128.0, 112.3, 96.9, 75.3, 75.1, 72.8, 72.6, 68.0, 52.2, 28.3, 26.6, 25.6, 18.0, -4.5, -5.3; HRMS (ESI-MS): m/z calcd for $C_{23}H_{36}O_7Si[M+Na]^+$: 475.2128, Found: 475.2130.

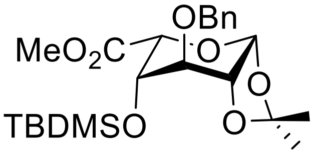

Methyl 3-O-benzyl-4-O-$^{t-}$butyldimethylsilyl-L-idopyranuronate (S4): To the compound **23** (310 mg, 684.9 µmol) in a 50 mL round bottom flask was added 75% aq. dichloroacetic acid (7 mL) at 0 °C and the solution was stirred at 0-25 °C for 1-2 h. After completion, the reaction was diluted with ice-cold water (30 mL), neutralized with $NaHCO_3$ (4 g) and extracted with CH_2Cl_2 (3 x 15 mL). The combined organic phases were washed with saturated aqueous $NaHCO_3$ and brine solution, dried over anhydrous Na_2SO_4 and concentrated *in vacuo*. The crude residue was further purified by silica gel column chromatography using 30% ethyl acetate in *n*-hexane as mobile phase to afford 230

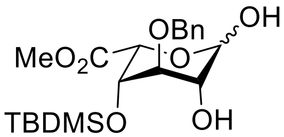

mg (81%) of the compound **S4** as a thick syrup; $[\alpha]^{25}_{D}$ ($CHCl_3$, *c* 0.7): -7.7°; IR (cm^{-1}, $CHCl_3$): 3504, 2956, 2925, 2858, 1740, 1461, 1373, 1214, 1140, 1082, 916, 839, 777; ^{1}H NMR (400.31 MHz, $CDCl_3$): δ 7.43 – 7.30 (m, 5H), 4.98 (d, *J* = 12.4 Hz, 1H), 4.68 (d, *J* = 12.1 Hz, 1H), 4.66 – 4.55 (m, 2H), 4.09 – 4.05 (m, 1H), 3.81 – 3.71 (m, 4H), 3.70 – 3.62 (m, 1H), 3.47 (d, *J* = 11.7 Hz, 1H), 0.83 (s, 9H), -0.03 (d, *J* = 2.7 Hz, 6H); ^{13}C NMR (100.67 MHz, $CDCl_3$): δ 169.4, 137.2, 128.9, 128.9, 128.5, 128.1, 128.1, 93.1, 75.4, 74.6, 72.6, 69.2, 68.5, 52.5, 25.6 (3C), 18.0, -4.8, -5.4; HRMS (ESI-MS): m/z calcd for $C_{20}H_{32}O_7Si[M+Na]^+$: 435.1815, Found: 435.1818.

Methyl-1-*O*-(((1-ethynylcyclohexyl)oxy)carbonyl)-2-*O*-acetyl-3-*O*-benzyl-4-*O*-^{t}butyldimethylsilyl-L-idopyranuronate (10): To a solution of **S4** (155 mg, 375.71 µmol) in anhydrous CH_2Cl_2 (2 mL), DMAP (138 mg, 3.0 eq) and ethynyl cyclohexyl (4-nitrophenyl) carbonate **24** (130 mg, 1.2 eq) were added and the reaction mixture was stirred at 25 °C for 3 h. After complete consumption of the starting hemiacetal, acetic anhydride (71 µL, 2.0 eq) was added and the stirring continued for another 2 h. The reaction mixture was concentrated *in vacuo* and purified by silica gel column chromatography using 15% ethyl acetate in *n*-hexane as mobile phase to obtain **10** (220 mg, 97%) of the desired carbonate donor as a thick syrup; $[\alpha]^{25}_{D}$ ($CHCl_3$, *c*0.8): -24.7°; IR (cm^{-1}, $CHCl_3$): 3289, 2929, 2860, 1764, 1742, 1455, 1370, 1223, 1147, 1075, 1012, 915, 835, 755; ^{1}H NMR (399.78 MHz, $CDCl_3$): δ 7.37 – 7.30 (m, 5H), 5.91 (d, *J* = 1.8 Hz, 1H), 5.06 – 5.03 (m, 1H), 4.76 (d, *J* = 12.1 Hz, 1H), 4.60 (d, *J* = 7.0 Hz, 1H), 4.59 (d, *J* = 2.8 Hz, 1H), 3.94 (t, *J* = 2.3 Hz, 1H), 3.75 – 3.73 (m, 4H), 2.62 (s, 1H), 2.21 – 2.11 (m, 2H), 2.08 (s, 3H), 1.90 – 1.81 (m, 2H), 1.69 – 1.50 (m, 5H), 1.34 – 1.29 (m, 1H), 0.78 (s, 9H), -0.12 (s, 3H), -0.23 (s, 3H); ^{13}C NMR (100.53 MHz, $CDCl_3$): δ 170.8, 168.4, 151.1, 137.0, 128.7, 128.7, 128.4, 128.3, 128.3, 93.1, 82.6, 78.6, 76.4, 75.4, 75.0, 72.6, 68.2, 66.3, 52.3, 36.9, 36.7, 25.6 (3C), 25.0, 22.6,

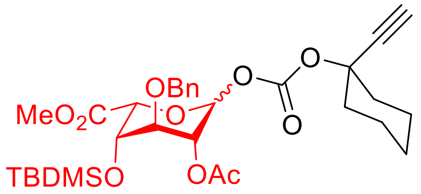

22.6, 21.2, 17.9, -4.5, -5.7; HRMS (ESI-MS): m/z calcd for $C_{31}H_{44}O_{10}Si[M+Na]^+$: 627.2601, Found: 627.2605.

Allyl 2-*deoxy*-2-azido-3-*O*-benzyl-6-*O*-acetyl-4-*O*-(2-*O*-acetyl-3-*O*-benzyl-4-*O*-^{t}-butyldimethylsilyl-5*R*-5-methoxycarbonyl-β-D-xylopyranosyl)-α-D-glucopyranoside (S5):

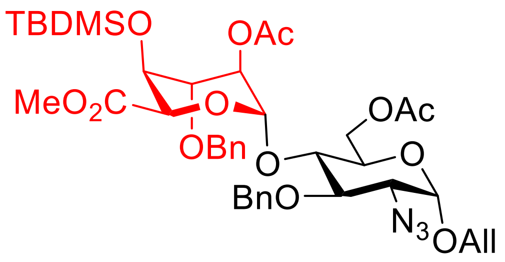

Freshly activated 4Å MS powder (50 mg) was added to a solution of glycosyl donor (256 mg, 0.423 mmol) and acceptor (120 mg, 0.318 mmol) in anhydrous CH_2Cl_2 (1.0 mL) at 25 °C under argon atmosphere. After 15 min of vigorous stirring at 25 °C, chloro[tris(2,4-di-t-butyl phenyl)phosphite]gold(I) **25** (8mol%) and AgOTf (8mol%) were added simultaneously to the reaction mixture and stirred for 15 min. After completion, the reaction mixture was quenched adding excess of Et_3N and filtered through a bed of Celite®, the filtrate was concentrated *in vacuo* and the crude residue was purified by silica gel column chromatography using 20% ethyl acetate and hexane as a mobile phase to afford 230 mg (89%) of the glycosylated compound **S5**. Thick syrup; $[\alpha]^{25}_{D}$ ($CHCl_3$, *c*1.0): +17.0º; IR (cm^{-1}, $CHCl_3$): 2955, 2860, 2109, 1742, 1460, 1371, 1237, 1091, 1040, 837, 701; ^{1}H NMR (400.31 MHz, $CDCl_3$): δ 7.39 – 7.36 (m, 2H), 7.35 – 7.27 (m, 7H), 7.27 – 7.24 (m, 1H), 5.94 (dddd, *J* = 16.6, 10.4, 6.1, 5.3 Hz, 1H), 5.35 (dq, *J* = 17.2, 1.5 Hz, 1H), 5.30 (d, *J* = 5.0 Hz, 1H), 5.25 (dq, *J* = 10.4, 1.2 Hz, 1H), 4.94 (d, *J* = 10.4 Hz, 1H), 4.92 (d, *J* = 3.7 Hz, 1H), 4.89 (t, *J* = 4.7 Hz, 1H), 4.76 – 4.70 (m, 2H), 4.67 (d, *J* = 11.8 Hz, 1H), 4.59 (d, *J* = 4.5 Hz, 1H), 4.43 (dd, *J* = 12.1, 2.0 Hz, 1H), 4.22 – 4.16 (m, 2H), 4.04 (ddt, 1H), 4.01 – 3.98 (m, 1H), 3.97 – 3.85 (m, 3H), 3.67 – 3.63 (m, 1H), 3.56 (s, 3H), 3.40 (dd, *J* = 9.8, 3.7 Hz, 1H), 2.12 (s, 3H), 2.00 (s, 3H), 0.83 (s, 9H), -0.04 (s, 3H), -0.09 (s, 3H); ^{13}C NMR (100.67 MHz, $CDCl_3$): δ 170.8, 170.2, 170.1, 138.2, 137.9, 133.3, 128.6, 128.6, 128.3, 128.3, 128.2, 128.2, 128.0 (3C), 127.6, 118.4, 98.0, 96.7, 78.3, 77.2, 76.3, 75.0, 73.3, 72.4, 70.9, 69.4, 69.3, 68.8, 63.2, 62.2, 51.8, 25.7 (3C), 21.1,

21.0, 17.9, -4.6, -5.3; HRMS (ESI-MS): m/z calcd for $C_{40}H_{55}N_3O_{13}Si[M+Na]^+$: 836.3402, Found: 836.3409.

Allyl 2-*deoxy*-2-azido-3-*O*-benzyl-6-*O*-acetyl-4-*O*-(2-*O*-acetyl-3-*O*-benzyl-5*R*-5-methoxycarbonyl-β-D-xylopyranosyl)-α-D-glucopyranoside 4: To a solution of the compound **S5** (230 mg, 0.283 mmol) in anhydrous pyridine (2 mL) was added dropwise 2 mL of the 70% HF•py at 0 °C and the reaction mixture was allowed to stir at 25 °C for 5 h. After completion, ice-cold water (20 ml) was added and extracted with EtOAc (2 x 10 mL). The organic layer was washed with aqueous 1*N* HCl (25 mL), saturated aq. $NaHCO_3$ (25 mL) and brine solution (50 mL), dried over anhydrous Na_2SO_4, concentrated *in vacuo* and the crude residue was purified by silica gel column chromatography using 60% ethyl acetate in *n*-hexane as mobile phase to furnish the corresponding disaccharide acceptor **4** (170 mg, 86%) as a thick syrup; $[\alpha]^{25}_D$ ($CHCl_3$, *c*1.2): +13.0°; IR (cm^{-1}, $CHCl_3$) : 3446, 3024, 2956, 2925, 2109, 1739, 1451, 1371, 1225, 1093, 1035, 752, 700; ^1H NMR (400.31 MHz, $CDCl_3$): δ 7.43 – 7.38 (m, 4H), 7.38 – 7.34 (m, 1H), 7.33 – 7.30 (m, 4H), 7.28 – 7.23 (m, 1H), 5.98 (dddd, *J* = 16.6, 10.4, 6.1, 5.4 Hz, 1H), 5.39 (dq, *J* = 17.2, 1.5 Hz, 1H), 5.30 (dq, *J* = 10.4, 1.2 Hz, 1H), 5.09 (s, 1H), 4.98 (d, *J* = 3.7 Hz, 1H), 4.96 (dt, *J* = 2.5, 1.2 Hz, 1H), 4.93 (d, *J* = 2.0 Hz, 1H), 4.77 (d, *J* = 11.3 Hz, 2H), 4.68 (t, *J* = 11.0 Hz, 2H), 4.44 (dd, *J* = 12.3, 1.1 Hz, 1H), 4.28 – 4.21 (m, 2H), 4.09 (ddt, *J* = 12.9, 6.2, 1.3 Hz, 1H), 4.01 – 3.96 (m, 1H), 3.93 – 3.84 (m, 3H), 3.76 – 3.73 (m, 1H), 3.50 (s, 3H), 3.42 (dd, *J* = 9.7, 3.6 Hz, 1H), 2.64 (d, *J* = 11.3 Hz, 1H), 2.12 (s, 3H), 2.10 (s, 3H); ^{13}C NMR (100.67 MHz, $CDCl_3$): δ 170.7, 169.6, 169.3, 137.9, 137.3, 133.2, 128.7, 128.7, 128.3 (3C), 128.2, 128.2, 127.6, 127.6, 127.5, 118.5, 98.2, 96.7, 78.7, 75.2, 74.7, 74.5, 72.5, 69.4, 68.9, 68.6, 67.8, 67.2, 63.7, 62.3, 52.2, 21.1, 21.0; HRMS (ESI-MS): m/z calcd for $C_{34}H_{41}N_3O_{13}$ $[M+Na]^+$: 722.2537, Found: 722.2545.

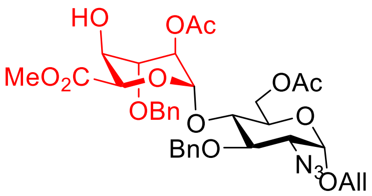

26: Troc Protection: To a solution of glucosamine hydrochloride (50 g, 231.88 mmol) in water (500 mL) was added $NaHCO_3$ (38.96 g, 463.76 mmol). After stirring for 30 min TrocCl (46.34 mL, 347.82 mmol) was added drop-wise and the mixture was stirred at 25 °C. After 16 h, the reaction mixture was filtered through Whatman filter paper, residue was washed with water and CH_2Cl_2-hexane (1:4), dried under vacuum to get 70 g (85%) of the compound **26**.

Allyl 2-*deoxy*-2-[[(2,2,2-trichloroethoxy)carbonyl]amino]-α-D-glucopyranoside (S6): A solution of the compound **26** (115 g, 324.34 mmol) in anhydrous allyl alcohol (1.1 L, 16.22 mol) was cooled to 0 °C and acetyl chloride (57.86 mL, 810.86 mmol) was added dropwise under argon atmosphere. After stirring at 0 °C for 1 h, the reaction mixture was refluxed at 105 °C for 15 h, the reaction was quenched by adding Et_3N and the volatile components were removed under reduced pressure. The product was precipitated by stirring crude compound in methanol-CH_2Cl_2-hexane (1:2:4) solvent mixture. The residue was filtered through Whatman filter, washed with CH_2Cl_2-Hexane (1:4) and dried over high vacuum; successfully afforded 100 g of the pure white solid. The filtrate was evaporated and purified by column chromatography to get overall 120 g (94%) of the compound **S6**. mp 121.3 °C; $[\alpha]^{25}_{D}$ ($CHCl_3$, *c* 1.0): +89.7°; IR (cm^{-1}, $CHCl_3$): 3320, 2961, 2920, 1712, 1543, 1272, 1034, 755; ^1H NMR (399.78 MHz, CD_3OD): δ 5.99 – 5.88 (m, 1H), 5.33 (dq, *J* = 17.4, 1.6 Hz, 1H), 5.16 (dq, *J* = 10.5, 1.4 Hz, 1H), 4.88 (d, *J* = 2.3 Hz, 1H), 4.85 (s, 1H), 4.70 (d, *J* = 12.1 Hz, 1H), 4.21 (ddt, *J* = 13.2, 5.0, 1.4 Hz, 1H), 4.01 (ddt, *J* = 13.3, 6.0, 1.2 Hz, 1H), 3.82 (dd, *J* = 11.8, 2.2 Hz, 1H), 3.72 – 3.57 (m, 4H), 3.36 (dd, *J* = 9.7, 8.3 Hz, 1H); ^{13}C NMR (100.53 MHz, CD_3OD): δ 156.8, 135.4, 117.6, 97.8, 97.1, 75.5, 73.9, 72.7, 72.2, 69.1, 62.6, 57.3; HRMS (ESI-MS): m/z calcd for $C_{12}H_{18}Cl_3NO_7$ $[M+Na]^+$: 416.0047, Found: 416.0046.

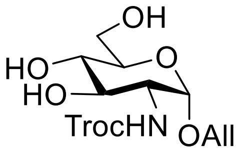

Allyl 2-*deoxy*-2-[[(2,2,2-trichloroethoxy)carbonyl]amino]-4,6-*O*-benzylidene-α-D-glucopyranoside 27: To a solution of the compound **S6** (120 g, 304.09 mmol) and benzaldehyde dimethyl acetal (68.46 mL, 456.13 mmol) in anhydrous DMF (500 mL) was added camphorsulfonic acid (14.13 g, 60.82 mmol) portion-wise under argon atmosphere and the mixture was stirred at 25 °C for 4 h. After completion, the reaction was quenched by dropwise addition of the ice-cold saturated aq $NaHCO_3$ solution (500 mL) and extracted with EtOAc (3 x 300 mL). The organic layers were washed with brine (3 x 300 mL), dried over Na_2SO_4 and concentrated *in vacuo*. The product was precipitated by stirring crude residue in the combination of methanol-water (1:1) solvent mixture and after filtration, residue was washed with methanol-water (1:1) followed by CH_2Cl_2-hexane (1:4), dried under vacuum to afford 98 g of the pure white solid. Further the filtrate was evaporated and purified by column chromatography to get an overall 108 g (74%) of the compound **27**. mp 172.5 °C; $[\alpha]^{25}_D$ ($CHCl_3$, *c*1.0): +66.9^0; IR (cm^{-1}, $CHCl_3$): 3336, 2955, 2870, 1710, 1541, 1375, 1269, 1086, 754; ^1H NMR (399.78 MHz, $CDCl_3$): δ 7.54 – 7.47 (m, 2H), 7.42 – 7.35 (m, 3H), 5.88 (ddt, *J* = 16.6, 10.8, 5.8 Hz, 1H), 5.52 (s, 1H), 5.39 (d, *J* = 9.2 Hz, 1H), 5.30 (d, *J* = 17.1 Hz, 1H), 5.23 (d, *J* = 10.3 Hz, 1H), 4.90 (d, *J* = 3.5 Hz, 1H), 4.83 (d, *J*= 12.1 Hz, 1H), 4.65 (d, *J* = 12.1 Hz, 1H), 4.25 (dd, *J* = 10.0, 4.6 Hz, 1H), 4.18 (dd, *J* = 12.8, 5.3 Hz, 1H), 4.02 – 3.89 (m, 2H), 3.88 – 3.79 (m, 2H), 3.72 (t, *J* = 10.2 Hz, 1H), 3.54 (t, *J* = 9.1 Hz, 1H), 3.11 (d, *J* = 3.3 Hz, 1H); ^{13}C NMR (100.53 MHz, $CDCl_3$): δ 155.0, 137.1, 133.3, 129.4, 128.4, 128.4, 126.4, 126.4, 118.4, 102.0, 97.1, 95.5, 81.9, 74.8, 69.8, 68.8, 68.7, 62.7, 55.8; HRMS (ESI-MS): m/z calcd for $C_{19}H_{22}Cl_3NO_7$ $[M+Na]^+$: 504.0360, Found: 504.0360 & 506.0321.

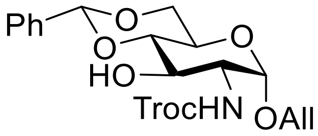

Allyl 2-*deoxy*-2-amino-4,6-*O*-benzylidene-α-D-glucopyranoside (28): To a solution of the compound **27** (98 g, 203.01 mmol) in 1:2 CH_2Cl_2-MeOH (60 mL) was added 20 mL of glacial acetic acid. Zinc powder (239 g, 3.65 mol) was added portion-wise at 0 °C and the mixture was

stirred at 25 °C for 1 h, filtered through a bed of Celite®, the residue was washed with excess CH_2Cl_2 and filtrate was concentrated under reduced pressure. The resulting crude residue was treated with saturated aq $NaHCO_3$ solution (300 mL) and extracted with CH_2Cl_2 (3 x 200 mL). The combined organic phases were washed with brine (200 mL), dried over Na_2SO_4 and concentrated *in vacuo*. The crude residue was passed through a small bed of silica gel using 2% methanol in CH_2Cl_2 as the mobile phase to afford 48 g (77%) of the compound **28** as a thick syrup; $[\alpha]^{25}_{D}$ ($CHCl_3$, *c*1.0): +108.3°; IR (cm^{-1},$CHCl_3$): 3275, 2917, 2865, 1604, 1571, 1457, 1376, 1082, 1016, 929, 754, 698; ^1H NMR (400.31 MHz, $CDCl_3$): δ 7.52 – 7.45 (m, 2H), 7.40 – 7.32 (m, 3H), 5.91 (dddd, *J* = 16.5, 10.4, 6.1, 5.4 Hz, 1H), 5.52 (s, 1H), 5.30 (dq, *J* = 17.2, 1.6 Hz, 1H), 5.21 (dq, *J* = 10.4, 1.3 Hz, 1H), 4.83 (d, *J* = 3.6 Hz, 1H), 4.25 (dd, *J* = 10.1, 4.8 Hz, 1H), 4.20 (ddt, *J* = 12.8, 5.3, 1.4 Hz, 1H), 4.00 (ddt, *J* = 12.8, 6.1, 1.3 Hz, 1H), 3.84 (td, *J* = 9.9, 4.7 Hz, 1H), 3.73 (td, *J* = 10.3, 9.8, 5.4 Hz, 2H), 3.45 (t, *J* = 9.3 Hz, 1H), 2.80 (dd, *J* = 9.7, 3.6 Hz, 1H), 2.68 (s, 3H); ^{13}C NMR (100.67 MHz, $CDCl_3$): δ 137.4, 133.8, 129.3, 128.4, 128.4, 126.4, 126.4, 117.8, 102.0, 99.3, 82.2, 71.7, 69.2, 68.8, 62.9, 56.6; HRMS (ESI-MS): m/z calcd for $C_{16}H_{21}NO_5$ $[M+H]^+$: 308.1498, Found: 308.1501.

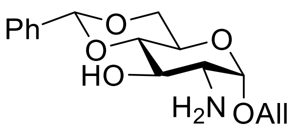

Allyl 2-*deoxy*-2-azido-4,6-*O*-benzylidene-α-D-glucopyranoside (29): To a solution of the compound **28** (50g, 162.68 mmol) in MeOH:THF (750 mL, 4:1) was added K_2CO_3 (53.96 g, 390.44 mmol) and $CuSO_4$•$5H_2O$ (406 mg, 1.63 mmol). Freshly prepared imidazole-1-sulfonyl azide hydrochloride (47.74 g, 227.76 mmol) was added portion-wise and the mixture was stirred at 25 °C for 2 h. The reaction mixture was concentrated, diluted with ice-cold water (1 L) and extracted with EtOAc (2 × 500 mL). The combined organic layers were washed with brine solution (300 mL), dried over Na_2SO_4 and concentrated *in vacuo*. The crude was purified by silica gel column chromatography to afford

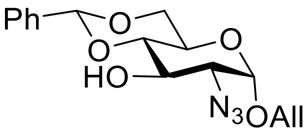

44.5 g (82 %) of the compound **29**. Eluent for purification: 15% ethyl acetate in *n*-hexane; white solid; mp 164.0 °C; $[\alpha]^{25}_{D}$ ($CHCl_3$, *c*1.0): +89.6°; IR (cm^{-1}, $CHCl_3$): 3390, 2920, 2868, 2109, 1648, 1459, 1381, 1267, 1092, 1032, 756; ^1H NMR (399.78 MHz, $CDCl_3$): δ 7.51 – 7.46 (m, 2H), 7.40 – 7.35 (m, 3H), 5.98 – 5.86 (m, 1H), 5.51 (s, 1H), 5.35 (dq, *J* = 17.3, 1.5 Hz, 1H), 5.28 – 5.22 (m, 1H), 4.92 (d, *J* = 3.6 Hz, 1H), 4.28 – 4.14 (m, 3H), 4.03 (ddt, *J* = 12.9, 6.0, 1.3 Hz, 1H), 3.86 (td, *J* = 10.0, 4.8 Hz, 1H), 3.70 (t, *J* = 10.3 Hz, 1H), 3.47 (t, *J* = 9.3 Hz, 1H), 3.25 (dd, *J* = 10.1, 3.7 Hz, 1H), 3.08 (s, 1H); ^{13}C NMR (100.53 MHz, $CDCl_3$): δ 137.0, 133.2, 129.5, 128.5, 128.5, 126.4, 126.4, 118.2, 102.1, 97.6, 81.9, 68.9, 68.8, 68.8, 63.1, 62.6; HRMS (ESI-MS): m/z calcd for $C_{16}H_{19}N_3O_5$ $[M+Na]^+$: 356.1222, Found: 356.1209.

30 (a-c):Benzyl/PMB/Nap protection: To a solution of alcohol (1 mmol) in anhydrous THF (2.5 mL) was added NaH (60% in mineral oil, 1.2 eq per -OH) in portions under argon atmosphere. After the evolution of hydrogen ceased, BnBr/PMB-Cl or Nap-Br (1.1 eq per OH) were added dropwise, followed by the addition of catalytic amount of tetrabutylammonium iodide and the mixture was stirred at 25 °C for 3 h. After completion, ice cold water (10 mL) was added slowly to the reaction mixture and extracted with EtOAc (2 x 10 mL), combined organic phases were dried over anhydrous Na_2SO_4, concentrated *in vacuo* to obtain a crude residue that was purified by silica gel column chromatography using EtOAc and hexane as mobile phase to afford the corresponding product.

Allyl 2-*deoxy*-2-azido-3-*O*-(4-methoxybenzyl)-4,6-*O*-benzylidene-α-D-glucopyranoside (30a): Eluent for purification: 12% ethyl acetate in *n*-hexane as a thick syrup; yield 7.85 g (81%) from 7.12 g; $[\alpha]^{25}_{D}$ ($CHCl_3$, *c*1.0): +47.8º; IR (cm^{-1}, $CHCl_3$) : 2922, 2866, 2107, 1612, 1513, 1460, 1374, 1248, 1089, 1036, 999, 823, 755, 700;^1H NMR (399.78 MHz, $CDCl_3$): δ 7.55 – 7.49 (m, 2H), 7.45 – 7.37 (m, 3H), 7.35 – 7.30 (m,

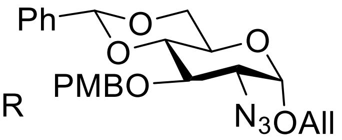

2H), 6.90 – 6.85 (m, 2H), 6.00 – 5.89 (m, 1H), 5.60 (s, 1H), 5.37 (dq, *J* = 17.3, 1.5 Hz, 1H), 5.27 (dq, *J* = 10.6, 1.3 Hz, 1H), 4.95 (d, *J* = 3.7 Hz, 1H), 4.90 (d, *J* = 10.6 Hz, 1H), 4.76 (d, *J* = 10.6 Hz, 1H), 4.30 (dd, *J* = 10.2, 4.8 Hz, 1H), 4.24 (ddt, *J* = 12.9, 5.1, 1.4 Hz, 1H), 4.14 – 4.04 (m, 2H), 3.94 (td, *J* = 10.0, 4.8 Hz, 1H), 3.80 (s, 3H), 3.79 – 3.74 (m, 1H), 3.71 (t, *J* = 9.3 Hz, 1H), 3.42 (dd, *J* = 10.0, 3.7 Hz, 1H); ^{13}C NMR (100.53 MHz, $CDCl_3$): δ 159.5, 137.3, 133.2, 130.0 (3C), 129.2, 128.4, 128.4, 126.1, 126.1, 118.3, 113.9, 113.9, 101.5, 97.5, 82.9, 76.0, 74.8, 69.0, 68.8, 63.1, 62.9, 55.3; HRMS (ESI-MS): m/z calcd for $C_{24}H_{27}N_3O_6$ $[M+Na]^+$: 476.1798, Found: 476.1791.

Allyl 2-*deoxy*-2-azido-3-*O*-benzyl-4,6-*O*-benzylidene-α-D-glucopyranoside (30b): Eluent for purification: 10% ethyl acetate in *n*-hexane; white solid; yield 9.9 g (87%) from 9.0 g as a solid; mp = 174.5 °C; $[\alpha]^{25}_D$ ($CHCl_3$, *c*1.0): +53.3°;

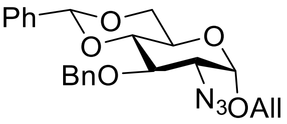

IR (cm^{-1}, $CHCl_3$): 2919, 2868, 2107, 1457, 1375, 1268, 1088, 997, 752, 698; ^{1}H NMR (399.78 MHz, $CDCl_3$): δ 7.57 – 7.52 (m, 2H), 7.46 – 7.40 (m, 5H), 7.39 – 7.30 (m, 3H), 5.97 (ddt, *J* = 16.3, 10.7, 5.7 Hz, 1H), 5.62 (s, 1H), 5.43 – 5.36 (m, 1H), 5.29 (d, *J* = 10.4 Hz, 1H), 5.00 (d, *J* = 11.0 Hz, 1H), 4.98 (d, *J* = 3.7 Hz, 1H), 4.85 (d, *J* = 11.0 Hz, 1H), 4.32 (dd, *J* = 10.2, 4.8 Hz, 1H), 4.26 (dd, *J* = 12.9, 5.2 Hz, 1H), 4.16 (t, *J* = 9.5 Hz, 1H), 4.09 (dd, *J* = 12.9, 6.1 Hz, 1H), 3.97 (td, *J* = 10.0, 4.8 Hz, 1H), 3.82 – 3.77 (m, 1H), 3.77 – 3.72 (m, 1H), 3.45 (dd, *J* = 9.9, 3.6 Hz, 1H); ^{13}C NMR (100.53 MHz, $CDCl_3$): δ 137.9, 137.3, 133.2, 129.1, 128.5, 128.5, 128.4, 128.4, 128.3, 128.3, 127.9, 126.1, 126.1, 118.3, 101.5, 97.5, 82.9, 76.3, 75.1, 68.9, 68.8, 63.1, 62.9; HRMS (ESI-MS): m/z calcd for $C_{23}H_{25}N_3O_5$ $[M+Na]^+$: 446.1692, Found: 446.1688.

Allyl 2-*deoxy*-2-azido-3-*O*-(^{t}butyldiphenylsilyl)-4,6-*O*-benzylidene-α-D-glucopyranoside 30d: To a solution of the compound **29** (1.5 g, 4.5 mmol) and Et_3N (5 eq) in anhydrous CH_2Cl_2 (20 mL) was added 0.5 equivalents of DMAP.

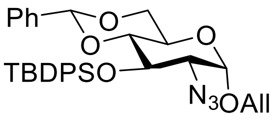

TBDPSCl (1.2 eq) was added drop-wise with stirring under argon atmosphere and the reaction mixture was stirred at 25 °C for 15 h. Ice-cold water (150 mL) was added and extracted with CH_2Cl_2 (3x100 mL), organic layer was washed with brine solution (2 x 200 mL), dried over Na_2SO_4 and concentrated *in vacuo* and the crude product was purified by flash silica gel column chromatography using EtOAc and hexane as a mobile phase to obtain the compound **30d** (2.0 g, 78%) as a thick syrup; ^{1}H NMR (400.31 MHz, $CDCl_3$): δ 7.59 – 7.53 (m, 4H), 7.39 – 7.33 (m, 2H), 7.30 – 7.24 (m, 3H), 7.17 (dt, *J* = 15.2, 7.6 Hz, 4H), 6.76 (dd, *J* = 8.1, 1.0 Hz, 2H), 5.87 (dddd, 1H), 5.27 (dq, *J* = 17.3, 1.6 Hz, 1H), 5.20 (dq, *J* = 10.5, 1.3 Hz, 1H), 4.99 – 4.93 (m, 2H), 4.19 (d, *J* = 9.8 Hz, 1H), 4.17 – 4.10 (m, 2H), 4.00 (ddt, *J* = 13.1, 6.1, 1.4 Hz, 1H), 3.66 (dd, *J* = 9.6, 4.5 Hz, 1H), 3.58 (t, *J* = 10.1 Hz, 1H), 3.50 (t, *J* = 9.1 Hz, 1H), 3.29 (dd, *J* = 9.8, 3.7 Hz, 1H), 0.99 (s, 9H); ^{13}C NMR (100.67 MHz, $CDCl_3$): δ 136.7, 136.5, 136.5, 135.6, 135.6, 134.8, 133.3, 132.2, 129.7, 129.4, 128.8, 127.8, 127.8, 127.4, 127.4, 127.3, 127.3, 126.5, 126.5, 118.0, 101.8, 98.3, 82.2, 70.3, 68.8, 68.8, 64.7, 62.7, 26.8 (3C), 19.7.

Allyl 2-*deoxy*-2-azido-3-*O*-acetyl-4,6-*O*-benzylidene-α-D-glucopyranoside (30e): This compound was prepared from compound **29** following the procedure adopted for the preparation of compound **20**. Eluent for purification: 13% ethyl acetate in *n*-hexane; thick syrup; yield 9.5 g (84%) from 10 g; $[\alpha]^{25}_{D}$ ($CHCl_3$, *c*1.0): +146.7°; IR (cm^{-1}, $CHCl_3$): 2959, 2912, 2105, 1732, 1460, 1377, 1267, 1230, 1090, 1040, 990, 757, 701; ^{1}H NMR (399.78 MHz, $CDCl_3$): δ 7.48 – 7.43 (m, 2H), 7.39 – 7.34 (m, 3H), 6.02 – 5.89 (m, 1H), 5.68 – 5.61 (m, 1H), 5.51 (s, 1H), 5.37 (dq, *J* = 17.3, 1.5 Hz, 1H), 5.27 (dq, *J* = 10.3, 1.1 Hz, 1H), 5.04 (d, *J* = 3.6 Hz, 1H), 4.31 – 4.24 (m, 2H), 4.09 (ddt, *J* = 12.8, 6.1, 1.2 Hz, 1H), 4.00 (td, *J* = 10.0, 4.9 Hz, 1H), 3.75 (t, *J* = 10.3 Hz, 1H), 3.63 (t, *J* = 9.6 Hz, 1H), 3.24 (dd, *J* = 10.3, 3.6 Hz, 1H), 2.13 (s, 3H); ^{13}C NMR (100.53 MHz, $CDCl_3$): δ 169.9,

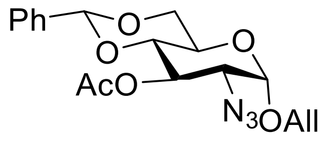

137.0, 133.0, 129.2, 128.3, 128.3, 126.3, 126.3, 118.5, 101.8, 98.0, 79.6, 69.1, 69.1, 68.8, 63.0, 61.7, 21.0; HRMS (ESI-MS): m/z calcd for $C_{18}H_{21}N_3O_6$ [M+Na]$^+$: 398.1328, Found: 398.1334.

S7 (a-e): Benzylidene Deprotection: To a solution of benzylidene protected compound (1.0 mmol) in 1:1 CH_3OH:CH_2Cl_2 (2 mL) was added 0.3 equivalent of the *p*-toluenesulfonic acid monohydrate and the mixture was stirred at 25 °C for 1 h. After completion, the reaction was quenched with Et_3N and volatiles were evaporated under reduced pressure, the crude residue was purified by silica gel column chromatography using EtOAc and hexane as mobile phase to obtain the desired product.

Allyl 2-*deoxy*-2-azido-3-*O*-(4-methoxybenzyl)-α-D-glucopyranoside (S7a): This compound was prepared from **30a,** following the above delineated procedure for **S7** (**a-e**). Eluent for purification: 70% ethyl acetate in *n*-hexane; Thick syrup;

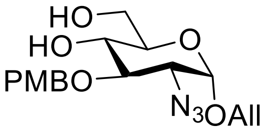

yield 5.45 g (86%) from 7.85 g; $[\alpha]^{25}_D$ ($CHCl_3$, *c*1.0): +74.0°; IR (cm^{-1}, $CHCl_3$): 3388, 2923, 2109, 1612, 1513, 1247, 1093, 1034, 822, 759;^{1}H NMR (400.31 MHz, $CDCl_3$): δ 7.34 – 7.29 (m, 2H), 6.91 – 6.87 (m, 2H), 5.93 (dddd, *J* = 17.1, 10.4, 6.1, 5.3 Hz, 1H), 5.34 (dq, *J* = 17.2, 1.6 Hz, 1H), 5.24 (dq, *J* = 10.4, 1.3 Hz, 1H), 4.93 (d, *J* = 3.5 Hz, 1H), 4.88 (d, *J* = 10.9 Hz, 1H), 4.68 (d, *J* = 10.9 Hz, 1H), 4.20 (ddt, *J* = 13.0, 5.2, 1.5 Hz, 1H), 4.03 (ddt, *J* = 13.0, 6.2, 1.3 Hz, 1H), 3.83 (dd, *J* = 10.2, 8.5 Hz, 1H), 3.79 (s, 3H), 3.79 – 3.76 (m, 2H), 3.67 (dt, *J* = 9.7, 3.6 Hz, 1H), 3.63 – 3.57 (m, 1H), 3.28 (dd, *J* = 10.2, 3.5 Hz, 1H), 2.78 (s, 1H), 2.23 (s, 1H); ^{13}C NMR (100.67 MHz, $CDCl_3$): δ 159.6, 133.3, 130.2, 129.9, 129.9, 118.3, 114.2, 114.2, 97.0, 79.8, 74.9, 71.5, 70.9, 68.7, 63.2, 62.1, 55.4; HRMS (ESI-MS): m/z calcd for $C_{17}H_{23}N_3O_6$ [M+Na]$^+$: 388.1485, Found: 388.1485.

Allyl 2-*deoxy*-2-azido-3-*O*-benzyl-α-D-glucopyranoside (S7b): This compound was prepared from compound **30b** following the above delineated procedure of **S7** (**a-e**). Eluent for purification: 40% ethyl acetate in *n*-hexane; thick syrup; yield 7.0 g (89%) from 9.9 g; $[\alpha]^{25}_D$

(CHCl3, *c*1.0): +54.0°; IR (cm^{-1}, $CHCl_3$): 3383, 2923, 2108, 1454, 1361, 1267, 1032, 754, 699; ^{1}H NMR (399.78 MHz, $CDCl_3$): δ 7.40 – 7.26 (m, 5H), 5.96 – 5.84 (m, 1H), 5.33 (dq, *J* = 17.3, 1.6 Hz, 1H), 5.25 – 5.19 (m, 1H), 4.91 – 4.86 (m, 2H), 4.80 (d, *J* = 11.1 Hz, 1H), 4.19 – 4.12 (m, 1H), 4.02 – 3.95 (m, 1H), 3.86 – 3.81 (m, 1H), 3.79 – 3.65 (m, 3H), 3.62 (d, *J* = 6.0 Hz, 2H), 3.26 (dd, *J* = 10.2, 3.6 Hz, 1H), 3.02 (s, 1H); ^{13}C NMR (100.53 MHz, $CDCl_3$): δ 137.9, 133.2, 128.6, 128.6, 128.1(3C), 118.1, 96.9, 79.9, 75.2, 71.6, 70.4, 68.5, 62.9, 61.4.

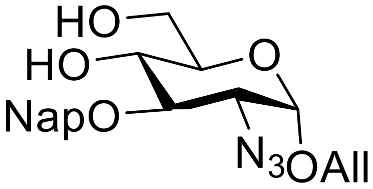

Allyl 2-*deoxy*-2-azido-3-*O*-naphthyl-α-D-glucopyranoside (S7c): This compound was prepared from **30c,**following the above procedure of **S7 (a-e)**. Eluent for purification: 40% ethyl acetate in n-hexane; thick syrup; yield 510 mg (87%) from 720 mg; $[\alpha]^{25}_{D}$ ($CHCl_3$, *c* 1.4): +87.9^{0}; IR (cm^{-1}, $CHCl_3$): 3349, 2921, 2865, 2106, 1339, 1266, 1110, 1033, 755; ^{1}H NMR (400.31 MHz, $CDCl_3$) δ 7.87 – 7.81 (m, 4H), 7.53 – 7.46 (m, 3H), 5.93 (dddd, *J* = 16.7, 10.4, 6.2, 5.3 Hz, 1H), 5.35 (dq, *J* = 17.2, 1.6 Hz, 1H), 5.25 (dq, *J* = 10.4, 1.3 Hz, 1H), 5.12 (d, *J* = 11.4 Hz, 1H), 4.95 (d, *J* = 3.5 Hz, 1H), 4.92 (d, *J* = 11.4 Hz, 1H), 4.21 (ddt, *J* = 12.9, 5.3, 1.5 Hz, 1H), 4.04 (ddt, *J* = 12.9, 6.2, 1.4 Hz, 1H), 3.91 (dd, *J* = 10.2, 8.4 Hz, 1H), 3.80 (d, *J* = 3.2 Hz, 2H), 3.73 – 3.64 (m, 2H), 3.33 (dd, *J* = 10.2, 3.5 Hz, 1H), 2.70 (s, 1H), 2.07 (s, 1H); ^{13}C NMR (100.67 MHz, $CDCl_3$) δ 135.5, 133.4, 133.3, 133.2, 128.7, 128.1, 127.9, 127.1, 126.4, 126.3, 125.9, 118.4, 97.1, 80.2, 75.3, 71.4, 71.1, 68.8, 63.2, 62.3; HRMS (ESI-MS): m/z calcd for $C_{20}H_{23}N_3O_5$ $[M+Na]^+$: 408.1535, Found: 408.1526.

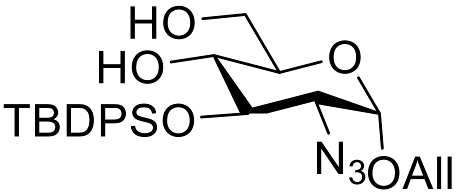

Allyl 2-*deoxy*-2-azido-3-*O*-($^{t-}$butyldiphenylsilyl)-α-D-glucopyranoside (S7d):This compound was prepared from **30d,**following the above procedure of **S7 (a-e)**. Eluent for purification: 35% ethyl acetate in n-hexane; thick syrup; yield 1.24 g (81%) from 1.8 g; ^{1}H NMR (400.31 MHz, $CDCl_3$) δ 7.83 – 7.79 (m,

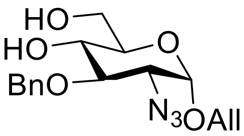

2H), 7.71 (dt, J = 6.1, 1.9 Hz, 2H), 7.47 – 7.40 (m, 6H), 5.83 (dddd, J = 17.2, 10.6, 5.8, 5.0 Hz, 1H), 5.20 (dq, J = 17.2, 1.6 Hz, 1H), 5.15 (dq, J = 10.5, 1.4 Hz, 1H), 4.96 (d, J = 3.5 Hz, 1H), 4.13 (ddt, J = 13.2, 4.9, 1.6 Hz, 1H), 4.03 (dd, J = 10.1, 8.3 Hz, 1H), 3.96 (ddt, J = 13.3, 5.8, 1.4 Hz, 1H), 3.76 – 3.68 (m, 2H), 3.64 (ddd, J = 9.8, 8.4, 4.3 Hz, 1H), 3.51 (dt, J = 9.8, 3.7 Hz, 1H), 3.16 (dd, J = 10.1, 3.5 Hz, 1H), 1.89 (s, 1H), 1.85 (d, J = 4.3 Hz, 1H), 1.10 (s, 9H); ^{13}C NMR (100.67 MHz, $CDCl_3$) δ 136.4, 136.4, 135.5, 135.5, 134.7, 133.3, 132.0, 130.2, 130.1, 128.3, 128.3, 128.0, 128.0, 117.5, 97.5, 73.9, 72.2, 71.0, 68.4, 63.9, 62.2, 26.9 (3C), 19.7.

Allyl 2-*deoxy*-2-azido-3-*O*-acetyl-α-D-glucopyranoside (S7e):This compound was prepared from compound **30e**, following the above described procedure of **S7** (**a**-**e**). Eluent for purification: 40% ethyl acetate in *n*-hexane; thick syrup; yield 1.95 g (85%) from 3 g; ^{1}H NMR (400.31 MHz, $CDCl_3$): δ 5.91 (dddd, J = 17.3, 10.4, 6.1, 5.2 Hz, 1H), 5.38 – 5.28 (m, 2H), 5.23 (dq, J = 10.4, 1.4 Hz, 1H), 4.98 (d, J = 3.5 Hz, 1H), 4.22 (ddt, J = 13.0, 5.3, 1.5 Hz, 1H), 4.04 (ddt, J = 13.0, 6.1, 1.4 Hz, 1H), 3.82 (d, J = 3.4 Hz, 2H), 3.72 (dt, J = 9.9, 3.4 Hz, 1H), 3.65 (t, J = 9.4 Hz, 2H), 3.23 (dd, J = 10.5, 3.5 Hz, 1H), 2.71 (s, 1H), 2.15 (s, 3H); ^{13}C NMR (100.67 MHz, $CDCl_3$): δ 171.8, 133.1, 118.4, 96.9, 73.5, 71.9, 69.5, 68.8, 61.7, 61.0, 21.0.

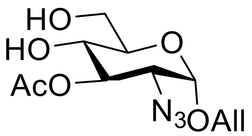

Allyl 2-*deoxy*-2-azido-3-*O*-(4-methoxybenzyl)-6-O-benzoyl-α-D-glucopyranoside (31a): A solution of the 4,6-diol **30a** (5.45 g, 31.15 mmol) in anhydrous CH_2Cl_2 (50 mL) was added 9.0 eq of Et_3N and the benzoic anhydride (1.2 eq) was added slowly at 0 °C under argon atmosphere. The reaction mixture was stirred at 25 °C for 12 h, quenched by adding methanol (5 mL), volatiles were evaporated under vacuum and the residue was purified by silica gel column chromatography using 12% ethyl acetate in *n*-hexane as a mobile phase to afford **31a** (5.86 g, 86%) as a thick syrup; $[\alpha]^{25}_D$ ($CHCl_3$, *c*1.0): +74.0°; IR (cm^{-1}, $CHCl_3$): 3465, 2919, 2106, 1717, 1611, 1514, 1455, 1271, 1109, 1033, 930, 822, 756,

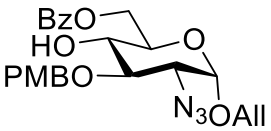

713; ^{1}H NMR (399.78 MHz, $CDCl_3$): δ 8.04 (dt, *J* = 8.4, 1.6 Hz, 2H), 7.60 – 7.55 (m, 1H), 7.46 – 7.41 (m, 2H), 7.37 – 7.31 (m, 2H), 6.92 – 6.87 (m, 2H), 5.94 (dddd, *J* = 16.8, 10.3, 6.1, 5.4 Hz, 1H), 5.34 (dq, *J* = 17.1, 1.5 Hz, 1H), 5.24 (dq, *J* = 10.5, 1.4 Hz, 1H), 4.97 (d, *J* = 3.6 Hz, 1H), 4.87 (d, *J* = 10.7 Hz, 1H), 4.76 (d, *J* = 10.7 Hz, 1H), 4.71 (dd, *J* = 12.2, 4.4 Hz, 1H), 4.47 (dd, *J* = 12.2, 2.1 Hz, 1H), 4.23 (ddt, *J* = 12.9, 5.2, 1.4 Hz, 1H), 4.08 (ddt, *J* = 13.1, 6.3, 1.3 Hz, 1H), 3.94 (ddd, *J* = 10.0, 4.3, 2.1 Hz, 1H), 3.88 (dd, *J* = 10.2, 8.7 Hz, 1H), 3.79 (s, 3H), 3.57 (td, *J* = 9.9, 3.6 Hz, 1H), 3.34 (dd, *J* = 10.2, 3.5 Hz, 1H), 2.91 (d, *J* = 3.4 Hz, 1H); ^{13}C NMR (100.53 MHz, $CDCl_3$): δ 167.2, 159.7, 133.5, 133.3, 130.1, 130.0, 130.0, 129.9, 129.9, 129.6, 128.6, 128.6, 118.4, 114.2, 114.2, 97.0, 79.5, 75.1, 70.8, 70.4, 68.8, 63.5, 63.1, 55.4; HRMS (ESI-MS): m/z calcd for $C_{24}H_{27}N_3O_7$ $[M+Na]^+$: 492.1746, Found: 492.1743.

Allyl 2-*deoxy*-2-azido-3-*O*-(4-methoxybenzyl)-6-O-acetyl-α-D-glucopyranoside (6): A solution of the 4,6-diol **30b** (2.32 g, 6.92 mmol) in anhydrous CH_2Cl_2 (40 mL) was added 9.0 eq of Et_3N and the acetic anhydride (1.2 eq) dropwise at 0 °C under argon atmosphere. The reaction mixture was stirred at 25 °C for 12 h, quenched by adding methanol (4 mL), volatiles were evaporatedand the residue was purified by silica gel column chromatography using 10% ethyl acetate in *n*-hexane as mobile phase to afford compound **6** (2.2 g, 84%) as a thick syrup; $[\alpha]^{25}_D$ ($CHCl_3$, *c*1.0): +60.8°; IR (cm^{-1}, $CHCl_3$): 3452, 2920, 2108, 1737, 1455, 1367, 1245, 1097, 1037, 921, 747, 701;^{1}H NMR (400.31 MHz, $CDCl_3$): δ 7.42 – 7.29 (m, 5H), 5.94 (dddd, *J* = 17.1, 10.4, 6.2, 5.3 Hz, 1H), 5.35 (dq, *J* = 17.2, 1.6 Hz, 1H), 5.25 (dq, *J* = 10.4, 1.3 Hz, 1H), 4.96 (d, *J* = 3.6 Hz, 1H), 4.93 (d, *J* = 11.0 Hz, 1H), 4.82 (d, *J* = 11.0 Hz, 1H), 4.51 (dd, *J* = 12.3, 4.1 Hz, 1H), 4.23 – 4.16 (m, 2H), 4.06 (ddt, *J* = 12.9, 6.2, 1.3 Hz, 1H), 3.87 (dd, *J* = 10.2, 8.7 Hz, 1H), 3.81 (ddd, *J* = 10.0, 4.0, 2.3 Hz, 1H), 3.49 (td, *J* = 9.9, 3.4 Hz, 1H), 3.33 (dd, *J* = 10.2, 3.6 Hz, 1H), 2.75 (d, *J* = 3.5 Hz, 1H), 2.11 (s, 3H); ^{13}C NMR (100.53 MHz, $CDCl_3$): δ 171.9, 138.0, 133.2, 128.8, 128.8, 128.3

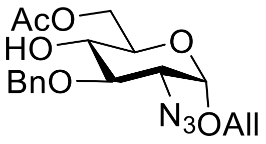

(3C), 118.4, 97.0, 79.7, 75.4, 70.7, 70.2, 68.8, 63.0, 63.0, 21.0; HRMS (ESI-MS): m/z calcd for $C_{18}H_{23}N_3O_6$ $[M+Na]^+$: 400.1485, Found: 400.1489.

31(b-f): Selective TBDPS Protection: To a solution of the 4,6-diol compound (1.0 mmol) and Et_3N (5 eq) in anhydrous CH_2Cl_2 (5 mL) was added 0.5 equivalent of DMAP. The TBDPSCl (1.2 eq) was added drop-wise with stirring under argon atmosphere and the reaction mixture was stirred at 25 °C for 15 h. Ice-cold water (30 mL) was added to the reaction mixture and extracted with CH_2Cl_2 (3x20 mL), organic layer was washed with brine solution (2 x 50 mL), dried over Na_2SO_4 and concentrated *in vacuo*. The crude product was purified by flash silica gel column chromatography using EtOAc and hexane as a mobile phase to obtain the desired product.

Allyl 2-*deoxy*-2-azido-3-*O*-benzyl-6-O-^{t}butyldiphenylsilyl-α-D-glucopyranoside (31b): This compound was prepared from compound **S7b,** following the above described procedure for **31** (**b**-**f**). Eluent for purification: 8% ethyl acetate in *n*-hexane; Thick syrup; yield 10.03 g (98%) from 6.0 g; $[\alpha]^{25}_D$ ($CHCl_3$, *c*1.0): +40.5°; IR (cm^{-1}, $CHCl_3$): 3382, 3069, 2932, 2861, 2106, 1465, 1355, 1268, 1103, 1049,752, 701; ^{1}H NMR (399.78 MHz, $CDCl_3$): δ 7.73 – 7.69 (m, 4H), 7.47 – 7.31 (m, 11H), 6.01 – 5.85 (m, 1H), 5.34 (dq, *J* = 17.3, 1.5 Hz, 1H), 5.29 – 5.18 (m, 1H), 4.96 – 4.91 (m, 2H), 4.87 (d, *J* = 11.1 Hz, 1H), 4.19 (ddt, *J* = 12.9, 5.1, 1.4 Hz, 1H), 4.07 – 4.00 (m, 1H), 3.92 – 3.87 (m, 3H), 3.76 – 3.71 (m, 2H), 3.34 (dd, *J* = 10.2, 3.6 Hz, 1H), 2.55 (d, *J* = 1.9 Hz, 1H), 1.09 (s, 9H); ^{13}C NMR (100.53 MHz, $CDCl_3$): δ 138.3, 135.8, 135.8, 135.7, 135.7, 133.4, 133.2, 133.0, 130.0, 130.0, 128.7, 128.7, 128.3, 128.3, 128.1, 127.9(4C), 118.1, 96.7, 80.1, 75.3, 72.7, 71.2, 68.4, 64.4, 63.0, 27.0(3C), 19.4; HRMS (ESI-MS): m/z calcd for $C_{32}H_{39}N_3O_5Si[M+H]^+$: 574.2737, Found: 574.2726.

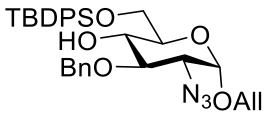

Allyl 2-*deoxy*-2-azido-3-*O*-(4-methoxybenzyl)-6-O-^{t}butyldiphenylsilyl-α-D-glucopyranoside (31c): This compound was prepared from compound **S7a** following the above delineated procedure for **31** (**b**-**f**).

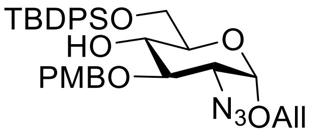

Eluent for purification: 7% ethyl acetate in *n*-hexane; Thick syrup; yield 1.18 g (83%) from 0.865 g; $[\alpha]^{25}_{D}$ ($CHCl_3$, *c*1.0): +30.3°; IR (cm^{-1}, $CHCl_3$): 3488, 2930, 2869, 2107, 1613, 1514, 1465, 1257, 1109, 1043, 754, 704; ^{1}H NMR (399.78 MHz, $CDCl_3$): δ 7.76 – 7.70 (m, 4H), 7.50 – 7.37 (m, 8H), 6.96 – 6.92 (m, 2H), 6.01 – 5.90 (m, 1H), 5.36 (dq, *J* = 17.1, 1.5 Hz, 1H), 5.28 – 5.23 (m, 1H), 4.96 (d, *J* = 3.5 Hz, 1H), 4.88 (d, *J* = 10.7 Hz, 1H), 4.80 (d, *J* = 10.7 Hz, 1H), 4.21 (ddt, *J* = 13.0, 5.3, 1.4 Hz, 1H), 4.05 (ddt, *J* = 13.2, 6.3, 1.3 Hz, 1H), 3.92 – 3.87 (m, 3H), 3.82 (s, 3H), 3.78 – 3.69 (m, 2H), 3.35 (dd, *J* = 10.2, 3.5 Hz, 1H), 2.57 (d, *J* = 2.2 Hz, 1H), 1.11 (s, 9H); ^{13}C NMR (100.53 MHz, $CDCl_3$): δ 159.5, 135.7(4C), 133.4, 133.2, 133.0, 130.4, 129.9(4C), 127.9, 127.9, 127.8, 127.8, 118.1, 114.1, 114.1, 96.7, 79.8, 74.9, 72.5, 71.3, 68.3, 64.2, 63.0, 55.3, 26.9(3C), 19.3; HRMS (ESI-MS): m/z calcd for $C_{33}H_{41}N_3O_6Si[M+H]^+$: 604.2843, Found: 604.2844.

Allyl 2-*deoxy*-2-azido-3-*O*-naphthyl-6-O-^{t}butyldiphenylsilyl-α-D-glucopyranoside (31d): This compound was prepared from compound **S7c** following the above described procedure of **31** (**b**-**f**). Eluent for purification: 7% ethyl acetate

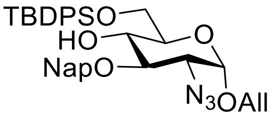

in *n*-hexane; thick syrup; yield 660 mg (91%) from 450 mg; $[\alpha]^{25}_{D}$ ($CHCl_3$, *c*1.0): +36.5°; IR (cm^{-1}, $CHCl_3$): 3492, 3055, 2928, 2862, 2106, 1692, 1466, 1339, 1267, 1106, 1048, 817, 753, 702; ^{1}H NMR (400.31 MHz, $CDCl_3$): δ 7.92 – 7.85 (m, 4H), 7.76 – 7.72 (m, 4H), 7.59 (dd, *J* = 8.4, 1.7 Hz, 1H), 7.52 – 7.40 (m, 8H), 5.96 (dddd, *J* = 16.6, 10.4, 6.1, 5.2 Hz, 1H), 5.37 (dq, *J* = 17.2, 1.6 Hz, 1H), 5.26 (dq, *J* = 10.4, 1.3 Hz, 1H), 5.12 (d, *J* = 11.3 Hz, 1H), 5.06 (d, *J* = 11.4 Hz, 1H), 4.98 (d, *J* = 3.6 Hz, 1H), 4.21 (ddt, *J* = 13.0, 5.2, 1.5 Hz, 1H), 4.06 (ddt, *J* = 13.0, 6.2, 1.3 Hz, 1H), 4.01 – 3.96 (m, 1H), 3.92 (d, *J* = 3.7 Hz, 2H), 3.80 – 3.75 (m, 2H), 3.39 (dd, *J* = 10.2, 3.6

Hz, 1H), 2.65 (s, 1H), 1.12 (s, 9H); ^{13}C NMR (100.67 MHz, $CDCl_3$): δ 135.8, 135.8, 135.7(3C), 133.5, 133.5, 133.2, 133.1, 133.0, 130.0, 130.0, 128.5, 128.1, 127.9(4C), 127.8, 127.0, 126.2, 126.1, 126.1, 118.1, 96.8, 80.1, 75.3, 72.8, 71.3, 68.4, 64.4, 63.0, 27.0(3C), 19.4; HRMS (ESI-MS): m/z calcd for $C_{36}H_{41}N_3O_5Si[M+Na]^+$: 646.2712, Found: 646.2718.

Allyl 2-*deoxy*-2-azido-3-*O*-acetyl-6-O-^{t}butyldiphenylsilyl-α-D-glucopyranoside (31e): This compound was prepared from compound **S7e** following the above delineated procedure for**31** (**b**-**f**). Eluent for purification: 12% ethyl acetate in *n*-hexane; Thick syrup; yield 10.1 g (89%) from 6.2 g; $[\alpha]^{25}_D$ ($CHCl_3$, *c* 1.0): +10.9°; IR (cm^{-1}, $CHCl_3$): 3384, 2931, 2862, 2109, 1740, 1467, 1371, 1235, 1096, 1047, 754, 703; ^{1}H NMR (400.31 MHz, $CDCl_3$): δ 7.73 – 7.68 (m, 4H), 7.47 – 7.38 (m, 6H), 5.98 – 5.86 (m, 1H), 5.41 – 5.30 (m, 2H), 5.23 (dq, *J* = 10.4, 1.3 Hz, 1H), 4.98 (d, *J* = 3.5 Hz, 1H), 4.20 (ddt, *J* = 12.9, 5.3, 1.4 Hz, 1H), 4.03 (ddt, *J* = 12.9, 6.2, 1.3 Hz, 1H), 3.91 (d, *J* = 4.2 Hz, 2H), 3.82 – 3.69 (m, 2H), 3.24 (dd, *J* = 10.6, 3.5 Hz, 1H), 2.84 (d, *J* = 4.1 Hz, 1H), 2.19 (s, 3H), 1.08 (s, 9H); ^{13}C NMR (100.67 MHz, $CDCl_3$): δ 171.5, 135.7(4C), 133.2, 133.1, 133.0, 130.0, 130.0, 127.9(4C), 118.2, 96.7, 73.6, 71.7, 71.0, 68.6, 64.1, 61.0, 26.9(3C), 21.1, 19.4; HRMS (ESI-MS): m/z calcd for $C_{27}H_{35}N_3O_6Si[M+Na]^+$: 548.2193, Found: 548.2192.

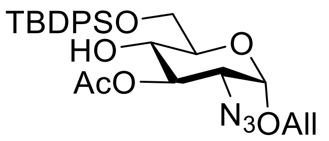

Allyl 2-*deoxy*-2-azido-3,6-di-*O*-^{t}butyldiphenylsilyl-α-D-glucopyranoside (31f): This compound was prepared from compound **S7d** following the above mentioned procedure for **31** (**b**-**f**). Eluent for purification: 7% ethyl acetate in *n*-hexane; Thick syrup; yield 680 mg (91%) from 500 mg; $[\alpha]^{25}_D$ ($CHCl_3$, *c*1.0): +76.6°; IR (cm^{-1}, $CHCl_3$): 3110, 2930, 2861, 2107, 1457, 1268, 1102, 1036, 752, 702; ^{1}H NMR (399.78 MHz, $CDCl_3$)P: δ 7.88 – 7.83 (m, 2H), 7.77 – 7.73 (m, 2H), 7.70 – 7.66 (m, 4H), 7.51 – 7.42 (m, 8H), 7.41 – 7.35 (m, 4H), 5.92 – 5.81 (m, 1H), 5.23 (dq, *J* = 17.3, 1.6 Hz, 1H), 5.17 (dq, *J* = 10.4,

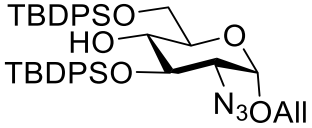

1.4 Hz, 1H), 5.01 (d, *J* = 3.6 Hz, 1H), 4.16 (ddt, *J* = 13.3, 4.9, 1.6 Hz, 1H), 4.07 (dd, *J* = 10.1, 8.4 Hz, 1H), 3.99 (ddt, *J* = 13.3, 5.9, 1.3 Hz, 1H), 3.86 – 3.71 (m, 3H), 3.55 (ddd, *J* = 9.8, 4.2, 2.8 Hz, 1H), 3.23 (dd, *J* = 10.1, 3.5 Hz, 1H), 1.67 (d, *J* = 4.5 Hz, 1H), 1.13 (s, 9H), 1.09 (s, 9H); ^{13}C NMR (100.53 MHz, $CDCl_3$): δ 136.3, 136.3, 135.8, 135.8, 135.7, 135.7, 135.4, 135.4, 134.9, 133.5, 133.5, 133.4, 132.3, 130.1, 129.9, 129.8, 129.8, 128.1, 128.1, 127.9, 127.9, 127.8(4C), 117.4, 97.2, 74.1, 71.9, 71.7, 68.0, 64.0, 63.4, 26.9(6C), 19.7, 19.4.

Allyl 2-*deoxy*-2-azido-3-*O*-benzyl-4-*O*-naphthyl-6-O-^{t}butyldiphenylsilyl-α-D-glucopyranoside (32a): This compound was prepared from compound **31b** following the above mentioned procedure for **30** (**a**-**c**).

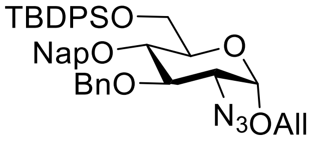

Eluent for purification: 5% ethyl acetate in *n*-hexane; Thick syrup; yield 6.2 g (92%) from 5.4 g; $[\alpha]^{25}_D$ ($CHCl_3$, *c*1.0): +22.7°; IR (cm^{-1}, $CHCl_3$): 3040, 2932, 2860, 2107, 1596, 1464, 1356, 1268, 1153, 1089, 1059, 815, 751, 700; ^{1}H NMR (399.78 MHz, $CDCl_3$): δ 7.90 – 7.86 (m, 1H), 7.82 – 7.77 (m, 4H), 7.74 (dd, *J* = 8.1, 1.3 Hz, 2H), 7.69 (s, 1H), 7.55 – 7.51 (m, 2H), 7.48 – 7.34 (m, 12H), 5.98 (dddd, *J* = 16.8, 10.5, 6.2, 5.4 Hz, 1H), 5.38 (dq, *J* = 17.5, 1.5 Hz, 1H), 5.30 – 5.25 (m, 1H), 5.10 (d, *J* = 11.1 Hz, 1H), 5.06 (d, *J* = 3.6 Hz, 1H), 4.99 (s, 2H), 4.91 (d, *J* = 11.1 Hz, 1H), 4.25 (ddt, *J* = 12.7, 4.9, 1.2 Hz, 1H), 4.19 – 4.13 (m, 1H), 4.09 (ddt, *J* = 12.7, 6.3, 1.2 Hz, 1H), 4.05 – 3.95 (m, 2H), 3.93 – 3.85 (m, 2H), 3.52 (dd, *J* = 10.2, 3.6 Hz, 1H), 1.13 (s, 9H; ^{13}C NMR (100.53 MHz, $CDCl_3$): δ 138.0, 136.0, 136.0, 135.7, 135.7, 135.6, 133.6, 133.5, 133.4, 133.2, 133.1, 129.8, 129.8, 128.6, 128.6, 128.3(3C), 128.0, 128.0, 127.8(3C), 127.7, 127.7, 126.5, 126.2, 126.1, 125.8, 118.1, 96.6, 80.7, 78.5, 75.8, 75.3, 72.2, 68.3, 63.8, 62.7, 26.9(3C), 19.4; HRMS (ESI-MS): m/z calcd for $C_{43}H_{47}N_3O_5Si[M+Na]^+$: 714.3363, Found: 714.3353.

Allyl 2-*deoxy*-2-azido-3-*O*-benzyl-4-*O*-(4-methoxylbenzyl)-6-O-^{t}butyldiphenylsilyl-α-D-glucopyranoside (32b): This compound was prepared from compound **31b** following the above described procedure for **30** (**a**-**c**). Eluent for purification: 5% ethyl acetate in *n*-hexane; Thick syrup; yield 525 mg (87%) from 500 mg; $[\alpha]^{25}_D$ ($CHCl_3$, *c*1.0): +71.6º; IR (cm^{-1}, $CHCl_3$): 2954, 2924, 2859, 2106, 1612, 1513, 1462, 1363, 1249, 1111, 1045, 822, 743, 703, 611; ^{1}H NMR (400.31 MHz, $CDCl_3$): δ 7.73 – 7.68 (m, 4H), 7.46 – 7.41 (m, 4H), 7.40 – 7.35 (m, 6H), 7.34 – 7.29 (m, 1H), 7.09 – 7.05 (m, 2H), 6.81 – 6.77 (m, 2H), 5.91 (dddd, *J* = 16.8, 10.4, 6.2, 5.2 Hz, 1H), 5.30 (dq, *J* = 17.2, 1.6 Hz, 1H), 5.21 (dq, *J* = 10.4, 1.3 Hz, 1H), 4.98 (d, *J* = 3.6 Hz, 1H), 4.91 (s, 2H), 4.79 (d, *J* = 10.4 Hz, 1H), 4.58 (d, *J* = 10.4 Hz, 1H), 4.17 (ddt, *J* = 12.9, 5.3, 1.5 Hz, 1H), 4.06 – 3.99 (m, 2H), 3.92 – 3.85 (m, 2H), 3.79 (s, 3H), 3.75 – 3.69 (m, 2H), 3.41 (dd, *J* = 10.3, 3.6 Hz, 1H), 1.07 (s, 9H); ^{13}C NMR (100.67 MHz, $CDCl_3$): δ 159.4, 138.1, 136.0, 136.0, 135.8, 135.8, 133.7, 133.5, 133.3, 130.3, 129.8, 129.8, 129.6, 129.6, 128.7, 128.7, 128.3, 128.3, 128.1, 127.9, 127.9, 127.7, 127.7, 118.1, 114.0, 114.0, 96.6, 80.7, 78.3, 75.7, 75.0, 72.2, 68.2, 63.8, 62.7, 55.4, 26.9 (3C), 19.4; HRMS (ESI-MS): m/z calcd for $C_{40}H_{47}N_3O_6Si[M+Na]^+$: 716.3132, Found: 716.3127.

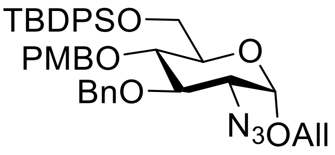

Allyl 2-*deoxy*-2-azido-3,4-di-*O*-benzyl-6-O-^{t}butyldiphenylsilyl-α-D-glucopyranoside (32c): This compound was prepared from compound **31b** following the above described procedure for **30** (**a**-**c**). Eluent for purification: 6% ethyl acetate in *n*-hexane; Thick syrup; yield 8.6 g (93%) from 8.0 g; $[\alpha]^{25}_D$ ($CHCl_3$, *c*1.0): +55.9°; IR (cm^{-1}, $CHCl_3$): 3029, 2932, 2861, 2107, 1461, 1268, 1151, 1093, 1060, 752, 700; ^{1}H NMR (399.78 MHz, $CDCl_3$): δ 7.76 – 7.70 (m, 4H), 7.48 – 7.41 (m, 5H), 7.41 – 7.39 (m, 2H), 7.39 – 7.37 (m, 2H), 7.37 – 7.32 (m, 3H), 7.32 – 7.30 (m, 2H), 7.21 (dd, *J* = 6.7, 2.9 Hz, 2H), 5.94 (dddd, *J* = 16.7, 10.3, 6.2, 5.3 Hz, 1H), 5.34 (dq, *J* = 17.2, 1.6 Hz, 1H), 5.24 (dq, *J* = 10.5, 1.1

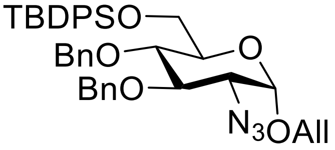

Hz, 1H), 5.02 (d, *J* = 3.6 Hz, 1H), 4.94 – 4.89 (m, 3H), 4.71 (d, *J* = 10.8 Hz, 1H), 4.21 (ddt, *J* = 12.9, 5.1, 1.4 Hz, 1H), 4.11 – 4.03 (m, 2H), 3.96 (dd, *J* = 11.2, 2.9 Hz, 1H), 3.91 (dd, *J* = 11.2, 0.8 Hz, 1H), 3.82 – 3.78 (m, 2H), 3.46 (dd, *J* = 10.2, 3.6 Hz, 1H), 1.10 (s, 9H); ^{13}C NMR (100.53 MHz, $CDCl_3$): δ 138.1, 138.0(3C), 135.7, 135.7, 133.6, 133.5, 133.2, 129.8, 129.8, 128.6(4C), 128.4, 128.4, 128.1, 127.9(3C), 127.8, 127.8, 127.7, 127.7, 118.1, 96.6, 80.6, 78.5, 75.8, 75.3, 72.2, 68.3, 63.8, 62.6, 26.9(3C), 19.4; HRMS (ESI-MS): m/z calcd for $C_{39}H_{45}N_3O_5Si[M+Na]^+$: 664.3206, Found: 664.3215.

Allyl 2-*deoxy*-2-azido-3-*O*-benzyl-4-*O*-naphthyl-α-D-glucopyranoside (S8): To a solution of the silyl ether **32a** (500 mg, 0.7 mmol) in 4:1 anhydrous pyridine-THF (5 mL) was added dropwise 1.0 mL of the 70% HF•py solution at 0 °C and the reaction mixture was stirred at 25 °C for 5 h. Reaction mixture was diluted with ice-cold water (20 mL) and extracted with EtOAc (2 x 20 mL),the organic layer was washed with aqueous 1*N* HCl (25 mL), saturated aq. $NaHCO_3$ (25 mL), brine solution (50 mL), dried over anhydrous Na_2SO_4, concentrated *in vacuo* and the crude residue was purified by silica gel column chromatography using 20% ethyl acetate in *n*-hexane as mobile phase to furnish 295 mg (89%) of the compound **S8** as a thick syrup;

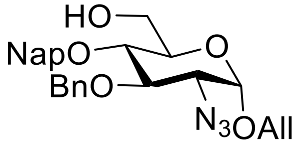

$[\alpha]^{25}_D$ ($CHCl_3$, *c*1.0): +60.6°; IR (cm^{-1}, $CHCl_3$): 3438, 3031, 2921, 2107, 1458, 1361, 1127, 1088, 1036, 818, 752;^{1}H NMR (400.31 MHz, $CDCl_3$): δ 7.87 – 7.80 (m, 3H), 7.75 (s, 1H), 7.52 – 7.47 (m, 2H), 7.44 (dd, *J* = 8.4, 1.6 Hz, 1H), 7.42 – 7.38 (m, 2H), 7.38 – 7.31 (m, 3H), 6.01 – 5.90 (m, 1H), 5.37 (dq, *J* = 17.2, 1.5 Hz, 1H), 5.26 (dq, *J* = 10.4, 1.2 Hz, 1H), 5.04 (d, *J* = 11.2 Hz, 1H), 4.97 (d, *J* = 3.5 Hz, 1H), 4.95 – 4.91 (m, 2H), 4.86 (d, *J* = 11.2 Hz, 1H), 4.22 (ddt, *J* = 12.9, 5.2, 1.4 Hz, 1H), 4.13 – 4.03 (m, 2H), 3.87 – 3.78 (m, 3H), 3.71 (t, *J* = 9.2 Hz, 1H), 3.40 (dd, *J* = 10.2, 3.6 Hz, 1H), 1.87 (s, 1H); ^{13}C NMR (100.66 MHz, $CDCl_3$): δ 138.0, 135.4, 133.4, 133.3, 133.1,

128.6, 128.6, 128.4, 128.2, 128.2, 128.0, 128.0, 127.8, 126.7, 126.3, 126.2, 125.8, 118.2, 96.9, 80.3, 78.1, 75.6, 75.3, 71.6, 68.7, 63.6, 61.6; HRMS (ESI-MS): m/z calcd for $C_{27}H_{29}N_3O_5$ $[M+Na]^+$: 498.2005, Found: 498.1999.

Allyl 2-*deoxy*-2-azido-3-*O*-benzyl-4-*O*-naphthyl-6-*O*-acetyl-α-D-glucopyranoside (32d): This compound was prepared from compound **S8** following the above described procedure for the compound **20**. Eluent for purification: 10% ethyl acetate in *n*-hexane; Thick syrup; yield 112 mg (86%) from 120 mg; $[\alpha]^{25}_D$ ($CHCl_3$, *c*1.0): +95.0°; IR (cm^{-1}, $CHCl_3$): 3026, 2919, 2107, 1741, 1456, 1366, 1238, 1087, 1037, 820, 754, 700; ^1H NMR (400.31 MHz, $CDCl_3$): δ 7.86 – 7.80 (m, 3H), 7.71 (s, 1H), 7.52 – 7.47 (m, 2H), 7.44 – 7.40 (m, 3H), 7.39 – 7.30 (m, 3H), 5.95 (dddd, *J* = 17.2, 10.4, 6.1, 5.3 Hz, 1H), 5.36 (dq, *J* = 17.2, 1.6 Hz, 1H), 5.26 (dq, *J* = 10.4, 1.3 Hz, 1H), 5.02 (d, *J* = 11.1 Hz, 1H), 4.98 (dd, *J* = 7.1, 3.6 Hz, 2H), 4.91 (d, *J* = 10.6 Hz, 1H), 4.78 (d, *J* = 11.1 Hz, 1H), 4.35 – 4.26 (m, 2H), 4.22 (ddt, *J* = 12.9, 5.3, 1.5 Hz, 1H), 4.13 – 4.04 (m, 2H), 3.98 – 3.93 (m, 1H), 3.64 (dd, *J* = 10.1, 8.7 Hz, 1H), 3.43 (dd, *J* = 10.3, 3.5 Hz, 1H), 1.92 (s, 3H); ^{13}C NMR (100.66 MHz, $CDCl_3$): δ 170.7, 137.9, 135.0, 133.3, 133.2, 133.2, 128.7, 128.7, 128.5, 128.2, 128.2, 128.1, 128.1, 127.8, 127.0, 126.3, 126.2, 126.0, 118.4, 96.8, 80.7, 77.7, 75.7, 75.2, 69.3, 68.8, 63.6, 62.8, 20.7; HRMS (ESI-MS): m/z calcd for $C_{29}H_{31}N_3O_6$ $[M+Na]^+$: 540.2111, Found: 540.2128.

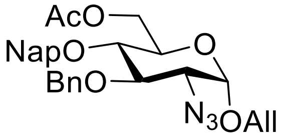

Allyl 2-*deoxy*-2-azido-3-*O*-benzyl-4-*O*-naphthyl-6-*O*-levulinoyl-α-D-glucopyranoside (32e): To a solution of **S8** (250 mg, 0.526 mmol) and levulinic acid (1.5 eq) in anhydrous CH_2Cl_2 (3 mL) was added 0.2 equivalent of DMAP and reaction was cooled to 0 °C, *N*, *N'*-diisopropyl carbodiimide (1.2 eq) was added drop-wise under argon atmosphere and the mixture was stirred at 25 °C for 2 h. After complete consumption of the starting alcohol, the reaction mixture was concentrated *in vacuo* and the crude residue was

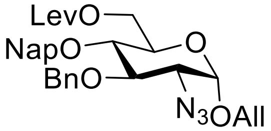

purified by silica gel column chromatography using 20% ethyl acetate in *n*-hexane as mobile phase to afford the levulinate ester **32e** (280 mg, 93%) as a thick syrup; $[\alpha]^{25}_D$ ($CHCl_3$, *c*1.0): +73.8^0; IR (cm^{-1}, $CHCl_3$): 3028, 2919, 2107, 1734, 1360, 1156, 1087, 1062, 920, 820, 751, 700;^{1}H NMR (400.31 MHz, $CDCl_3$): δ 7.85 – 7.79 (m, 3H), 7.72 (s, 1H), 7.51 – 7.46 (m, 2H), 7.44 – 7.39 (m, 3H), 7.38 – 7.31 (m, 3H), 5.95 (dddd, *J* = 17.2, 10.4, 6.1, 5.3 Hz, 1H), 5.37 (dq, *J* = 17.2, 1.6 Hz, 1H), 5.26 (dq, *J* = 10.4, 1.4 Hz, 1H), 5.03 (d, *J* = 11.1 Hz, 1H), 4.99 – 4.95 (m, 2H), 4.91 (d, *J* = 10.7 Hz, 1H), 4.79 (d, *J* = 11.1 Hz, 1H), 4.36 – 4.30 (m, 2H), 4.22 (ddt, *J* = 12.9, 5.3, 1.5 Hz, 1H), 4.12 – 4.04 (m, 2H), 3.95 (dt, *J* = 10.0, 3.2 Hz, 1H), 3.64 (dd, *J* = 10.1, 8.8 Hz, 1H), 3.43 (dd, *J* = 10.2, 3.6 Hz, 1H), 2.65 (t, *J* = 6.2 Hz, 2H), 2.56 – 2.40 (m, 2H), 2.15 (s, 3H); ^{13}C NMR (100.66 MHz, $CDCl_3$): δ 206.3, 172.5, 137.9, 135.2, 133.3, 133.2, 133.1, 128.6, 128.6, 128.4, 128.2, 128.2, 128.1, 128.1, 127.8, 127.0, 126.3, 126.1, 126.0, 118.3, 96.8, 80.6, 77.9, 75.6, 75.2, 69.4, 68.7, 63.5, 62.9, 37.8, 29.9, 27.8; HRMS (ESI-MS): m/z calcd for $C_{32}H_{35}N_3O_7$ $[M+Na]^+$: 596.2373, Found: 596.2354.

S9 (a-e): Allyl Deprotection: To a biphasic solution of the allyl glycoside (1.0 mmol) in 3:1 CH_3OH:CH_2Cl_2 (20 mL) was added 0.15 equivalent of $PdCl_2$ and the reaction mixture was stirred for 4-8 h at 25 °C, the reaction was quenched by adding excess of Et_3N and filtered through a bed of Celite®. The filtrate was concentrated *in vacuo* and the crude residue was purified by silica gel column chromatography using ethyl acetate and *n*-hexane as a mobile phase to obtain the desired hemiacetal.

2-*deoxy*-2-azido-3-*O*-benzyl-4-*O*-naphthyl-6-*O*-(^{t}butyl diphenylsilyl)-D-glucopyranoside (S9a): This compound was prepared from compound **32a** following the above delineated procedure for **S9** (**a-e**). Eluent for purification: 10% ethyl acetate in *n*-hexane; Thick syrup; yield 1.55 g (82%) from 2.0 g; $[\alpha]^{25}_D$ ($CHCl_3$, *c*1.0): -20.8°; IR (cm^{-1}, $CHCl_3$): 3390, 3033, 2930, 2860, 2108, 1463, 1359, 1267, 1108, 1051, 817, 752,

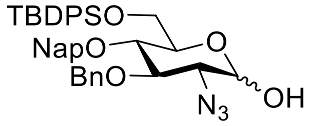

701; ^{1}H NMR (399.78 MHz, $CDCl_3$): δ 7.90 – 7.85 (m, 1H), 7.82 – 7.66 (m, 7H), 7.52 (dt, *J* = 6.0, 3.2 Hz, 2H), 7.48 – 7.30 (m, 12H), 5.34 (t, *J* = 3.0 Hz, 1H), 5.09 (dd, *J* = 17.9, 11.1 Hz, 1H), 5.00 – 4.86 (m, 3H), 4.19 – 3.84 (m, 4H), 3.56 – 3.50 (m, 1H), 3.45 – 3.37 (m, 1H), 3.11 (s, 1H), 1.12 (s, 9H); ^{13}C NMR (100.53 MHz, $CDCl_3$): δ 137.9(2C), 136.1(2C), 136.0(2C), 135.7(4C), 135.5, 135.4, 133.7, 133.7, 133.4, 133.4, 133.2, 133.1(3C), 129.8(4C), 128.6(4C), 128.4(2C), 128.3(4C), 128.1(4C), 127.8(6C), 127.7(4C), 126.6, 126.4, 126.3, 126.2, 126.1, 126.0, 125.8, 125.7, 96.2, 92.2, 83.2, 80.3, 78.4, 77.6, 76.2, 75.8, 75.8, 75.3, 75.3, 72.1, 67.8, 64.3, 62.8, 62.7, 27.0(6C), 19.5, 19.4; HRMS (ESI-MS): m/z calcd for $C_{40}H_{43}N_3O_5Si[M+Na]^+$: 696.2869, Found: 696.2878.

2-*deoxy*-2-azido-3-*O*-benzyl-4-*O*-benzyl-6-*O*-($^{t-}$butyl diphenylsilyl)-D-glucopyranoside (S9c): This compound was prepared from compound **32c** following the above delineated procedure for **S9** (**a-e**). Eluent for purification: 12% ethyl acetate in *n*-hexane; Thick syrup; yield 6.8 g (85%) from

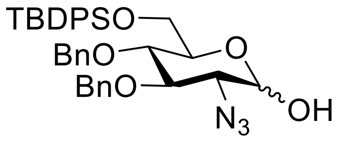

8.5 g; $[\alpha]^{25}_{D}$ ($CHCl_3$, *c*1.0): -8.7°; IR (cm^{-1}, $CHCl_3$): 3045, 2936, 2859, 2106, 1471, 1268, 1105, 754, 702;^{1}H NMR (400.31 MHz, $CDCl_3$)α:β isomers(1.5:1): δ 7.73 – 7.64 (m, 8H), 7.42 – 7.26 (m, 28H), 7.22 – 7.15 (m, 4H), 5.26 (t, *J* = 3.3 Hz, 1H), 4.93 – 4.79 (m, 6H), 4.70 (dd, *J* = 28.2, 10.9 Hz, 2H), 4.45 (dd, *J* = 7.9, 5.2 Hz, 1H), 4.06 – 3.82 (m, 8H), 3.77 – 3.72 (m, 1H), 3.47 – 3.40 (m, 2H), 3.36 – 3.26 (m, 2H), 2.96 (d, *J* = 3.0 Hz, 1H), 1.06 (s, 18H); ^{13}C NMR (100.67 MHz, $CDCl_3$) (α:β isomers(1.5:1)): δ 138.2, 138.0(3C), 136.1, 136.1, 136.0, 136.0, 135.7(4C), 133.8, 133.7, 133.2, 133.2, 129.8(4C), 128.6(8C), 128.4(2C), 128.3(2C), 128.1(2C), 127.9(3C), 127.8(5C), 127.7(6C), 96.2, 92.2, 83.2, 80.2, 78.4, 77.6, 76.2, 75.8, 75.8, 75.2, 75.2, 72.1, 67.8, 64.4, 62.9, 62.7, 27.0(6C), 19.5, 19.4;

2-*deoxy*-2-azido-3-*O*-benzyl-4-*O*-naphthyl-6-*O*-acetyl-D-glucopyranoside (S9d): This compound was prepared from compound **32d** following the above described procedure of **S9**

(**a-e**). Eluent for purification: 25% ethyl acetate in *n*-hexane; White solid; yield 81 mg (80%) from 110 mg; mp 123.4 °C; $[\alpha]^{25}_{D}$ ($CHCl_3$, *c*1.0): -20.0°; IR (cm^{-1}, $CHCl_3$): 3437, 3033, 2927, 2109, 1739, 1454,

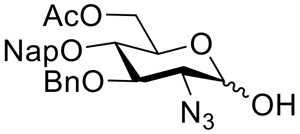

1365, 1240, 1127, 1077, 1037, 821, 752, 699; ^1H NMR (400.31 MHz, $CDCl_3$) [α:β isomers(1:2)]: δ 7.87 – 7.79 (m, 6H), 7.74 – 7.69 (m, 2H), 7.53 – 7.47 (m, 4H), 7.44 – 7.31 (m, 12H), 5.29 (t, *J* = 2.7 Hz, 1H), 5.02 (t, *J* = 10.8 Hz, 2H), 4.99 – 4.93 (m, 3H), 4.92 – 4.84 (m, 1H), 4.78 (dd, *J* = 11.2, 9.7 Hz, 2H), 4.59 (d, *J* = 7.9 Hz, 1H), 4.38 (ddd, *J* = 12.0, 7.6, 2.2 Hz, 2H), 4.24 – 4.07 (m, 5H), 3.67 – 3.38 (m, 7H), 1.90 (d, *J* = 1.5 Hz, 6H); ^{13}C NMR (100.67 MHz, $CDCl_3$)[α:β isomers(1:2)]: δ 171.0, 171.0, 137.7, 137.7, 135.0, 134.8, 133.3, 133.3, 133.2 (2C), 128.7 (4C), 128.5, 128.5, 128.3 (4C), 128.2, 128.1, 128.0 (2C), 127.8 (2C), 127.1, 127.1, 126.4, 126.4, 126.3, 126.2, 126.0, 126.0, 96.3, 92.1, 83.3, 80.4, 77.7, 76.9, 75.8, 75.7, 75.2, 75.2, 73.3, 69.2, 67.5, 64.1, 62.9 (C), 20.7 (2C); HRMS (ESI-MS): m/z calcd for $C_{26}H_{27}N_3O_6$ $[M+Na]^+$: 500.1798, Found: 500.1801.

2-*deoxy*-2-azido-3-*O*-benzyl-4-*O*-naphthyl-6-*O*-(levulinoyl)-D-glucopyranoside (S9e): This compound was prepared from compound **32e** following the above described procedure of **S9** (**a**-**e**). Eluent for purification: 40% ethyl

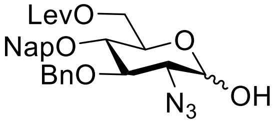

acetate in *n*-hexane; Thick syrup; yield 123 mg (85%) from 155 mg; $[\alpha]^{25}_{D}$ ($CHCl_3$, *c*1.0): +33.2°; IR (cm^{-1}, $CHCl_3$): 3391, 2921, 2107, 1716, 1361, 1153, 1131, 1075, 820, 750, 699; ^1H NMR (400.31 MHz, $CDCl_3$) [α:β isomers(2:1)]: δ 7.84 – 7.78 (m, 6H), 7.74 – 7.69 (m, 2H), 7.50 – 7.45 (m, 4H), 7.44 – 7.30 (m, 12H), 5.31 (t, *J* = 3.3 Hz, 1H), 5.01 (dd, *J* = 11.2, 8.3 Hz, 2H), 4.97 – 4.92 (m, 3H), 4.90 – 4.83 (m, 1H), 4.78 (dd, *J* = 11.2, 5.6 Hz, 2H), 4.60 (dd, *J* = 8.0, 4.2 Hz, 1H), 4.38 (dt, *J* = 11.8, 2.4 Hz, 2H), 4.25 – 4.13 (m, 4H), 4.08 (dd, *J* = 10.2, 8.8 Hz, 1H), 3.71 (dd, *J* = 3.2, 1.5 Hz, 1H), 3.63 – 3.48 (m, 4H), 3.47 – 3.38 (m, 2H), 2.69 – 2.62 (m, 4H), 2.55 – 2.37 (m, 4H), 2.15 (d, *J* = 2.5 Hz, 6H); ^{13}C NMR (100.66 MHz, $CDCl_3$) [α:β isomers(2:1)]: δ 207.3,

207.2, 172.6, 172.6, 137.8, 137.8, 135.2, 135.0, 133.3, 133.3, 133.2, 133.2, 128.7 (4C), 128.5, 128.5, 128.3 (4C), 128.1 (4C), 127.8, 127.8, 127.1, 127.0, 126.3, 126.3, 126.2, 126.2, 126.0, 126.0, 96.3, 92.1, 83.3, 80.4, 78.2, 77.3, 75.8, 75.7, 75.2, 75.1, 73.3, 69.2, 67.6, 64.2, 63.1, 63.0, 38.1, 37.9, 30.0, 29.9, 28.0, 27.9; HRMS (ESI-MS): m/z calcd for $C_{29}H_{31}N_3O_7$ $[M+Na]^+$: 556.2060, Found: 556.2059.

33(a-e): Synthesis of Ethynyl Cyclohexyl Glycosyl Carbonate Donor: To a solution of glycosyl hemiacetal (1.0 mmol) in anhydrous CH_2Cl_2 (5 mL) was added DMAP (1.5 eq) and ethynyl cyclohexyl (4-nitrophenyl) carbonate **24** (1.2 eq), the reaction mixture was stirred at 25 °C for 3 h. After complete consumption of the hemiacetal, the reaction mixture was concentrated *in vacuo* and purified by silica gel column chromatography using EtOAc and hexane as a mobile phase to obtain the desired carbonate donor.

1-*O*-(((1-ethynylcyclohexyl)oxy)carbonyl)-2-*deoxy*-2-azido-3-*O*-benzyl-4-*O*-naphthyl-6-O-($^{t-}$butyldiphenylsilyl)-D-glucopyranoside (33a): This compound was prepared from compound **S9a** following the above mentioned procedure for **33** (**a**-**e**). Eluent for purification: 8% ethyl acetate in *n*-hexane; yield 530 mg (96%) from 450 mg; Thick syrup; $[\alpha]^{25}_D$ ($CHCl_3$, *c*1.0): +24.3°; IR (cm^{-1}, $CHCl_3$): 3035, 2932, 2860, 2110, 1763, 1458, 1361, 1268, 1112, 1030, 753, 700; ^1H NMR (399.78 MHz, $CDCl_3$) [α-isomer]: δ 7.87 – 7.82 (m, 1H), 7.80 – 7.75 (m, 2H), 7.71 – 7.63 (m, 5H), 7.50 (dt, *J* = 6.3, 3.6 Hz, 2H), 7.44 – 7.33 (m, 9H), 7.32 – 7.27 (m, 3H), 6.17 (d, *J* = 3.6 Hz, 1H), 5.07 (d, *J* = 11.1 Hz, 1H), 4.95 (s, 2H), 4.90 (d, *J* = 11.1 Hz, 1H), 4.10 – 4.05 (m, 1H), 4.02 – 3.97 (m, 2H), 3.92 (dd, *J* = 10.0, 7.2 Hz, 2H), 3.67 (dd, *J* = 9.9, 3.6 Hz, 1H), 2.64 (s, 1H), 2.23 – 2.14 (m, 2H), 1.90 (q, *J* = 9.2 Hz, 2H), 1.74 – 1.60 (m, 4H), 1.57 – 1.50 (m, 1H), 1.37 – 1.31 (m, 1H), 1.08 (s, 9H); ^{13}C NMR (100.53 MHz, $CDCl_3$) [α-isomer]:δ 151.0, 137.8, 136.0, 136.0,

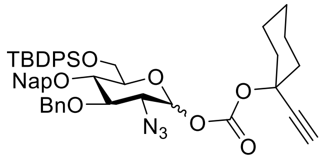

135.7, 135.7, 135.5, 133.5, 133.4, 133.1, 133.0, 129.8, 129.8, 128.7, 128.7, 128.4(3C), 128.2, 128.1, 127.9, 127.9 127.8(3C), 126.4, 126.3, 126.1, 125.6, 94.3, 82.7, 80.7, 78.4, 77.6, 76.0, 75.3, 75.3, 74.4, 63.2, 62.2, 36.8, 36.8, 27.0(3C), 25.0, 22.7, 22.6, 19.5; HRMS (ESI-MS): m/z calcd for $C_{49}H_{53}N_3O_7Si[M+Na]^+$: 846.3550, Found: 846.3548.

β-isomer: thick syrup; $[\alpha]^{25}_D$ ($CHCl_3$, *c*1.0): -35.5°; IR (cm^{-1}, $CHCl_3$): 3277, 3035, 2934, 2861, 2111, 1765, 1458, 1270, 1084, 1017, 909, 754, 701; ^{1}H NMR (399.78 MHz, $CDCl_3$)[β-isomer]: δ 7.87 – 7.82 (m, 1H), 7.81 – 7.76 (m, 2H), 7.72 (dd, *J* = 7.8, 1.6 Hz, 2H), 7.68 (dd, *J* = 8.0, 1.2 Hz, 3H), 7.51 (dt, *J* = 6.2, 3.3 Hz, 2H), 7.43 – 7.33 (m, 11H), 7.31 (d, *J* = 7.0 Hz, 1H), 5.40 – 5.36 (m, 1H), 5.07 (d, *J* = 11.0 Hz, 1H), 4.97 – 4.90 (m, 3H), 4.03 – 3.97 (m, 3H), 3.68 – 3.60 (m, 2H), 3.49 (dt, *J* = 9.7, 2.2 Hz, 1H), 2.67 (s, 1H), 2.28 – 2.20 (m, 2H), 2.05 – 1.95 (m, 2H), 1.78 – 1.64 (m, 4H), 1.57 – 1.53 (m, 1H), 1.46 – 1.36 (m, 1H), 1.08 (s, 9H); ^{13}C NMR (100.53 MHz, $CDCl_3$) [β-isomer]:δ 151.1, 137.7, 136.1, 136.1, 135.7, 135.7, 135.5, 133.6, 133.4, 133.1, 132.8, 129.8, 129.7, 128.7, 128.7, 128.5, 128.5, 128.4, 128.2, 128.1, 127.9, 127.9, 127.8, 127.7, 127.7, 126.6, 126.3, 126.2, 125.8, 96.0, 83.4, 82.6, 78.8, 77.3, 76.6, 76.1, 75.5, 75.4, 65.1, 62.1, 37.0, 36.7, 26.9(3C), 25.1, 22.6, 22.6, 19.4; HRMS (ESI-MS): m/z calcd for $C_{49}H_{53}N_3O_7Si[M+Na]^+$: 846.3550, Found: 846.3545.

1-*O*-(((1-ethynylcyclohexyl)oxy)carbonyl)-2-*deoxy*-2-azido-3-*O*-benzyl-4-*O*-(4-methoxy benzyl)-6-O-(^{t}butyldiphenylsilyl)-D-glucopyranoside (33b): α:β (5:1): This compound was prepared from compound **S9b** following the above mentioned procedure of **33** (**a**-**e**). Eluent for purification:5% ethyl acetate in *n*-hexane; Thick syrup; yield 155 mg (84%) from 150 mg; $[\alpha]^{25}_D$ ($CHCl_3$, *c*1.0): -30.6°; IR (cm^{-1}, $CHCl_3$): 3289, 2925, 2860, 2111, 1765, 1613, 1513, 1459, 1361, 1241, 1081, 1019, 911, 822, 745, 702, 613;^{1}H NMR (400.31 MHz, $CDCl_3$): δ 7.69 (td, *J* = 8.1, 1.4 Hz, 4H), 7.45 – 7.33 (m, 11H), 7.10

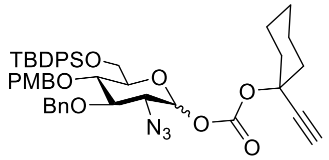

(d, J = 8.7 Hz, 2H), 6.82 (d, J = 8.6 Hz, 2H), 5.60 (d, 1H), 4.90 (s, 2H), 4.82 (d, J = 10.3 Hz, 1H), 4.67 (d, J = 10.4 Hz, 1H), 3.94 – 3.84 (m, 3H), 3.80 (s, 3H), 3.64 – 3.52 (m, 2H), 3.41 (dt, J = 9.6, 2.2 Hz, 1H), 2.64 (d, J = 11.9 Hz, 1H), 2.26 – 2.17 (m, 2H), 2.01 – 1.86 (m, 2H), 1.76 – 1.62 (m, 4H), 1.56 – 1.51 (m, 1H), 1.42 – 1.36 (m, 1H), 1.06 (s, 9H); ^{13}C NMR (100.67 MHz, $CDCl_3$): δ 159.5, 151.1, 137.7, 136.1, 136.1, 135.7, 135.7, 133.7, 132.9, 130.1, 129.8, 129.7 (3C), 128.7, 128.7, 128.4, 128.4, 128.2, 127.9, 127.9, 127.7, 127.7, 114.1, 114.1, 96.0, 83.4, 82.6, 78.8, 77.0, 76.7, 76.1, 75.5, 75.0, 65.1, 62.0, 55.4, 37.0, 36.7, 27.0 (3C), 25.1, 22.6, 22.6, 19.4; HRMS (ESI-MS): m/z calcd for $C_{46}H_{53}N_3O_8Si[M+Na]^+$: 826.3500, Found: 826.3499.

1-*O*-(((1-ethynylcyclohexyl)oxy)carbonyl)-2-*deoxy*-2-azido-3-*O*-benzyl-4-*O*-benzyl-6-O-(^{t}-butyldiphenylsilyl)-D-glucopyranoside (33c): α:β (2.3:1): This compound was prepared from compound **S9c** following the above procedure for **33** (**a**-**e**).

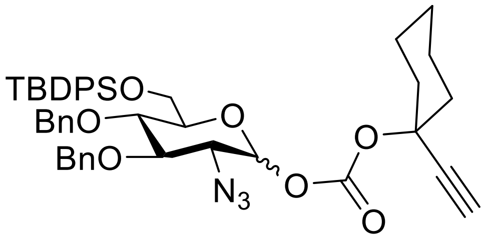

Eluent for purification: 8% ethyl acetate in *n*-hexane; Thick syrup; yield 6.2 g (92%) from 6.7 g; $[\alpha]^{25}_D$ ($CHCl_3$, *c*1.0): +5.3°; IR (cm^{-1}, $CHCl_3$): 3280, 3032, 2936, 2862, 2111, 1765, 1765, 1457, 1357, 1269, 1096, 1018, 909, 754, 700; ^{1}H NMR (400.31 MHz, $CDCl_3$): δ 7.70 (dd, J = 16.0, 6.8 Hz, 8H), 7.45 – 7.30 (m, 28H), 7.26 – 7.18 (m, 4H), 6.16 (d, J = 3.4 Hz, 1H), 5.36 (d, J = 7.6 Hz, 1H), 4.92 (d, J = 9.6 Hz, 6H), 4.77 (t, J = 11.1 Hz, 2H), 4.05 (t, J = 9.4 Hz, 1H), 4.00 – 3.84 (m, 7H), 3.67 – 3.56 (m, 3H), 3.45 (d, J = 9.6 Hz, 1H), 2.67 (s, 1H), 2.64 (s, 1H), 2.28 – 2.14 (m, 4H), 2.04 – 1.84 (m, 4H), 1.78 – 1.62 (m, 8H), 1.58 – 1.48 (m, 2H), 1.45 – 1.34 (m, 2H), 1.07 (s, 18H); ^{13}C NMR (100.67 MHz, $CDCl_3$): δ 151.1, 151.0, 138.0, 138.0, 137.8, 137.7, 136.0(4C), 135.7(4C), 133.6, 133.5, 133.0, 132.9, 129.8(3C), 129.7, 128.7(4C), 128.6(4C), 128.5(2C), 128.4(2C), 128.2(2C), 128.0, 127.9(9C), 127.8, 127.7(3C), 96.0, 94.3, 83.3, 82.7, 82.6, 80.7, 78.8, 78.4, 77.6, 77.3, 76.6, 76.1, 76.0, 75.5, 75.3(3C), 74.3, 65.1, 63.2, 62.2, 62.0, 37.0, 36.8(2C), 36.7, 27.0(3C),

26.9(3C), 25.1(2C), 22.6(4C), 19.5, 19.4; HRMS (ESI-MS): m/z calcd for $C_{45}H_{51}N_3O_7Si[M+Na]^+$: 796.3394, Found: 796.3386.

1-*O*-(((1-ethynylcyclohexyl)oxy)carbonyl)-2-*deoxy*-2-azido-3-*O*-benzyl-4-*O*-naphthyl-6-O-acetyl-D-glucopyranoside (33d): α:β (5:1): This compound was prepared from compound **S9d** following the above mentioned procedure for **33** (**a**-**e**). Eluent for purification: 12% ethyl acetate in *n*-hexane; thick syrup; yield 90 mg (85%) from 81 mg; $[\alpha]^{25}_D$ ($CHCl_3$, *c*1.0): +22.0º; IR (cm^{-1}, $CHCl_3$): 3294, 2927, 2863, 2111, 1764, 1455, 1367, 1271, 1237, 1088, 1016, 906, 755; ^{1}H NMR (399.78 MHz, $CDCl_3$): δ 7.84 – 7.78 (m, 3H), 7.70 – 7.68 (m, 1H), 7.51 – 7.46 (m, 2H), 7.41 – 7.33 (m, 6H), 5.60 (d, 1H), 5.03 – 4.94 (m, 2H), 4.90 (d, 1H), 4.76 (d, *J* = 11.1 Hz, 1H), 4.36 – 4.30 (m, 1H), 4.27 – 4.22 (m, 1H), 3.71 – 3.56 (m, 4H), 2.67 (s, 1H), 2.23 – 2.15 (m, 2H), 1.96 – 1.89 (m, 2H), 1.87 (s, 3H), 1.74 – 1.61 (m, 4H), 1.56 – 1.50 (m, 1H), 1.39 – 1.30 (m, 1H); ^{13}C NMR (100.53 MHz, $CDCl_3$): δ 170.7, 150.9, 137.5, 134.7, 133.3, 133.2, 128.7, 128.7, 128.6, 128.3, 128.3, 128.3, 128.1, 127.9, 127.2, 126.4, 126.3, 126.0, 95.8, 83.5, 82.4, 79.1, 76.6, 76.0, 75.6, 75.2, 73.9, 65.0, 62.4, 36.9, 36.7, 25.0, 22.6, 22.6, 20.7; HRMS (ESI-MS): m/z calcd for $C_{35}H_{37}N_3O_8$ $[M+Na]^+$: 650.2478, Found: 650.2472.

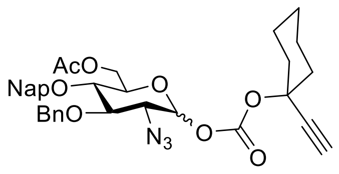

1-*O*-(((1-ethynylcyclohexyl)oxy)carbonyl)-2-*deoxy*-2-azido-3-*O*-benzyl-4-*O*-naphthyl-6-O-levulinoyl-D-glucopyranoside (33e): This compound was prepared from compound **S9e** following the above described procedure for **33** (**a**-**e**). Eluent for purification: 30% ethyl acetate in *n*-hexane; Thick syrup; yield 130 mg (83%) from 123 mg; $[\alpha]^{25}_D$ ($CHCl_3$, *c*1.0): -5.6°; IR (cm^{-1}, $CHCl_3$): 3291, 2938, 2865, 2113, 1765, 1740, 1452, 1362, 1272, 1239, 1091, 1017, 910, 759; ^{1}H NMR (399.78 MHz, $CDCl_3$): δ 7.85 – 7.79 (m, 3H), 7.72 – 7.69 (m, 1H), 7.50 – 7.46 (m, 2H), 7.41 – 7.33 (m, 6H), 5.39 – 5.32

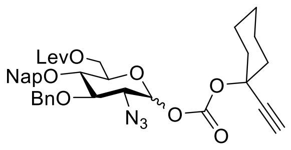

(m, 1H), 5.02 – 4.94 (m, 2H), 4.89 (d, *J* = 10.7 Hz, 1H), 4.78 (d, *J* = 11.1 Hz, 1H), 4.32 (d, *J* = 2.6 Hz, 2H), 3.71 – 3.59 (m, 4H), 2.68 (s, 1H), 2.63 (td, *J* = 6.7, 6.1, 3.4 Hz, 2H), 2.53 – 2.38 (m, 2H), 2.23 – 2.16 (m, 2H), 2.15 (s, 3H), 1.97 – 1.87 (m, 2H), 1.75 – 1.67 (m, 2H), 1.66 – 1.51 (m, 3H), 1.40 – 1.29 (m, 1H); ^{13}C NMR (100.53 MHz, $CDCl_3$): δ 206.5, 172.4, 150.9, 137.5, 134.9, 133.3, 133.2, 128.7, 128.7, 128.5, 128.3, 128.3, 128.2, 128.1, 127.8, 127.1, 126.3, 126.2, 126.0, 95.8, 83.4, 82.4, 79.1, 76.7, 76.0, 75.6, 75.2, 73.9, 64.9, 62.4, 37.8, 36.9, 36.7, 29.9, 27.8, 25.0, 22.6, 22.6; HRMS (ESI-MS): m/z calcd for $C_{38}H_{41}N_3O_9$ [M+Na]$^+$: 706.2740, Found: 706.2751.

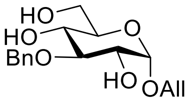

Allyl 3-*O*-benzyl α-D-glucopyranoside (S10): α:β (3:1): To the compound **34** (74 g, 211.18 mmol) in a 1L round bottom flask was added 90% aqueous trifluoroacetic acid (320 mL) and the reaction mixture was stirred for 15 min at 25 °C. The solvent was evaporated under reduced pressure and the residue was co-evaporated twice with water and twice with toluene. The crude was triturated in minimum quantity of the 10% ethyl acetate in hexane, washed with hexane and the trace solvent was removed by applying high vacuum. The crude was redissolved in anhydrous allyl alcohol (50 eq) and the mixture was cooled to 0 °C, acetyl chloride (2.5 eq) was added dropwise under argon atmosphere, the reaction mixture was stirred at 0 °C for 1h, refluxed at 105 °C for 12-16 h. After complete conversion, the reaction was quenched by adding excess of Et_3N and the volatile components were removed under vacuum. The crude residue was purified by silica gel column chromatography using 50% ethyl acetate in *n*-hexane as mobile phase to afford compound **S10** (49.5 g, 76%, over two steps) as a thick syrup; $[\alpha]^{25}_D$ ($CHCl_3$, *c*1.0): +63.1º; IR (cm^{-1}, $CHCl_3$): 3394, 1923, 1643, 1362, 1216, 1028, 927, 746, 700; ^{1}H NMR (399.78 MHz, $CDCl_3$): δ 7.38 – 7.25 (m, 10H), 5.97 – 5.85 (m, 2H), 5.30 (ddq, *J* = 17.3, 3.0, 1.5 Hz, 2H), 5.24 – 5.19 (m, 2H), 5.0-4.93 (d, *J* = 11.5 Hz, 2H), 4.87 (d, *J* = 3.5 Hz, 1H), 4.77-4.71 (d, *J* = 11.6 Hz, 2H), 4.35 (dt, *J* = 5.4, 1.4 Hz, 1H), 4.32 (d, *J* = 7.7, 1H), 4.19 (ddt, *J* = 12.8, 5.3, 1.4 Hz, 1H), 4.13 – 4.07 (ddt, 1H), 4.04 – 3.98 (m, 1H), 3.85 – 3.81 (m,

1H), 3.76 (d, *J* = 3.5 Hz, 2H), 3.72 (m, 1H), 3.66 – 3.54 (m, 5H), 3.53 – 3.47 (m, 1H), 3.39 (t, *J* = 9.0 Hz, 1H), 3.32 – 3.27 (m, 1H), 2.91 (s, 1H), 2.71 (s, 1H), 2.47 – 2.28 (m, 2H), 2.26 (m, 2H);^{13}C NMR (100.53 MHz, $CDCl_3$): δ 138.6, 138.6, 133.7, 133.6, 128.7(4C), 128.1(5C), 128.0, 118.3, 118.3, 102.0, 97.8, 83.8, 82.9, 75.4, 75.1, 74.9, 74.4, 72.8, 71.4, 70.6, 70.0, 70.0, 68.7, 62.4, 62.2; HRMS (ESI-MS): m/z calcd for $C_{16}H_{22}O_6$ $[M+Na]^+$: 333.1314, Found: 333.1316.

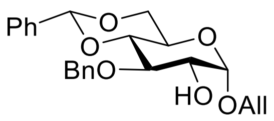

Allyl 3-*O*-benzyl-4,6-*O*-benzylidene-α-D-glucopyranoside (S11): To a solution of the compound **S10** (30 g, 96.67 mmol) in anhydrous DMF (150 mL) was added benzaldehyde dimethyl acetal (17.41 mL, 116 mmol) followed by the camphorsulfonic acid (4.49 g, 19.33 mmol) in a portion-wise manner under argon atmosphere and the reaction mixture stirred at 25 °C for 4 h. Ice-cold saturated aq $NaHCO_3$ solution (150 mL) and water (150 mL) were added to neutralize the reaction and extracted with EtOAc (3 x 100 mL), organic layer was washed with brine (3 x 100 mL), dried over anhydrous Na_2SO_4 and concentrated in vacuo. The product was precipitated by stirring crude residue in the combination of CH_2Cl_2-hexane solvent mixture and the residue was washed with hexane, dried by applying high vacuum to afford 25.5 g of white solid. The filtrate was evaporated and purified by column chromatography resulting in the overall 28.9 g (75%) of the compound **S11**. Eluent for purification: 25% ethyl acetate in *n*-hexane; white solid; mp 143.1 °C; $[\alpha]^{25}_D$ ($CHCl_3$, *c*1.0): +90.4º; IR (cm^{-1}, $CHCl_3$): 3432, 3025, 2925, 2869, 1449, 1367, 1217, 1076, 1032, 748, 694;^{1}H NMR (400.31 MHz, $CDCl_3$): δ 7.54 – 7.48 (m, 2H), 7.44 – 7.36 (m, 5H), 7.36 – 7.27 (m, 3H), 5.94 (dddd, *J* = 16.8, 10.4, 6.3, 5.4 Hz, 1H), 5.58 (s, 1H), 5.33 (dq, *J* = 17.2, 1.5 Hz, 1H), 5.25 (dd, *J* = 10.4, 1.3 Hz, 1H), 5.00 – 4.95 (m, 2H), 4.82 (d, *J* = 11.6 Hz, 1H), 4.30 (dd, *J* = 10.2, 4.8 Hz, 1H), 4.24 (ddt, *J* = 12.8, 5.4, 1.4 Hz, 1H), 4.08 (ddt, *J* = 12.8, 6.4, 1.2 Hz, 1H), 3.94 – 3.88 (m, 1H), 3.86 (d, *J* = 9.2 Hz, 1H), 3.76 (t, *J* = 10.3 Hz, 2H), 3.66 (t, *J* = 9.3 Hz, 1H), 2.38 (s, 1H);^{13}C NMR (100.67 MHz, $CDCl_3$): δ 138.6, 137.4, 133.5, 129.1, 128.5, 128.5, 128.4, 128.4,

128.1, 128.1, 127.8, 126.1, 126.1, 118.4, 101.4, 98.1, 82.0, 79.1, 75.0, 72.5, 69.1, 68.9, 62.9; HRMS (ESI-MS): m/z calcd for $C_{23}H_{26}O_6$ $[M+Na]^+$: 421.1627, Found: 421.1628.

Allyl 2-*O*-levulinoyl-3-*O*-benzyl-4,6-*O*-benzylidene-α-D-glucopyranoside (35a): This compound was prepared from compound **S11** following procedure described for the compound **32e**. Eluent for purification: 25% ethyl acetate

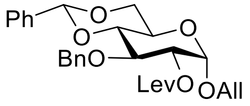

in *n*-hexane; Thick syrup; yield 3.95 g (92%) from 4.28 g; $[\alpha]^{25}_D$ ($CHCl_3$, *c*1.0): +67.3º; IR (cm^{-1}, $CHCl_3$): 3020, 2923, 2869, 1738, 1453, 1368, 1214, 1089, 1038, 751, 699; ^{1}H NMR (399.78 MHz, $CDCl_3$): δ 7.51 – 7.47 (m, 2H), 7.40 – 7.34 (m, 3H), 7.34 – 7.22 (m, 5H), 5.96 – 5.84 (m, 1H), 5.58 (s, 1H), 5.30 (dq, *J* = 17.3, 1.5 Hz, 1H), 5.21 (dd, *J* = 10.4, 1.3 Hz, 1H), 5.05 (d, *J* = 3.8 Hz, 1H), 4.89 (dd, *J* = 10.5, 4.2 Hz, 2H), 4.72 (d, *J* = 11.8 Hz, 1H), 4.28 (dd, *J* = 10.2, 4.8 Hz, 1H), 4.18 (ddt, *J* = 12.9, 5.2, 1.3 Hz, 1H), 4.10 – 3.98 (m, 3H), 3.92 (td, *J* = 10.0, 4.8 Hz, 1H), 3.81 – 3.67 (m, 2H), 2.76 – 2.49 (m, 4H), 2.14 (s, 3H); ^{13}C NMR (100.53 MHz, $CDCl_3$): δ 206.2, 172.2, 138.7, 137.4, 133.6, 129.1, 128.4, 128.4, 128.3, 128.3, 127.8, 127.8, 127.7, 126.1, 126.1, 118.1, 101.4, 96.0, 82.2, 76.3, 74.9, 73.2, 69.0, 68.9, 62.6, 37.9, 29.9, 28.1; HRMS (ESI-MS): m/z calcd for $C_{28}H_{32}O_8$ $[M+Na]^+$: 519.1995, Found: 519.1995.

35b: This compound was prepared from compound **S11** following procedure described for the compound **20**. Eluent for purification: 10% ethyl acetate in *n*-hexane; Thick syrup; yield 3.35 g (95%) from 3.2 g, forwarded as such for the next step.

Allyl 2-*O*-benzoyl-3-*O*-benzyl-4,6-*O*-benzylidene-α-D-glucopyranoside 35c: To a solution of the compound **S11** (20 g, 50.19 mmol) in anhydrous pyridine (100 mL) was cooled to 0 °C and the benzoyl chloride (1.2 eq) was added drop-wise

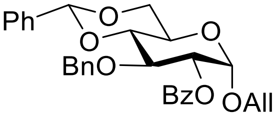

under argon atmosphere, catalytic amount of DMAP was added. The reaction mixture was gradually warmed up to 25 °C and stirred for 2 h. After completion, ice cooled water (500mL)

was added and extracted with EtOAc (2 x 250 mL). The organic layer was washed with aqueous 1*N* HCl (3 x 300 mL) and brine solution (500 mL), dried over anhydrous Na_2SO_4, concentrated *in vacuo* and the crude residue was purified by silica gel column chromatography using 10% ethyl acetate in *n*-hexane as mobile phase to afford 23.2 g (92%) of the compound **35c** as a white solid. mp 93.6 °C; $[\alpha]^{25}_{D}$ ($CHCl_3$, *c*1.0): +146.6º; IR (cm^{-1}, $CHCl_3$): 3024, 2930, 2867, 1727, 1601, 1453, 1370, 1270, 1217, 1096, 997, 752, 702;^{1}H NMR (400.31 MHz, $CDCl_3$): δ 8.16 – 8.09 (m, 2H), 7.66 (tt, *J* = 7.0, 1.3 Hz, 1H), 7.62 – 7.57 (m, 2H), 7.55 – 7.43 (m, 5H), 7.34 – 7.29 (m, 2H), 7.29 – 7.20 (m, 3H), 5.88 (dddd, *J* = 17.1, 10.5, 5.9, 5.2 Hz, 1H), 5.69 (s, 1H), 5.36 – 5.27 (m, 2H), 5.26 – 5.17 (m, 2H), 4.97 (d, *J* = 11.8 Hz, 1H), 4.85 (d, *J* = 11.8 Hz, 1H), 4.39 (dd, *J* = 10.2, 4.8 Hz, 1H), 4.32 (t, *J* = 9.4 Hz, 1H), 4.25 (ddt, *J* = 13.2, 5.1, 1.5 Hz, 1H), 4.11 – 4.03 (m, 2H), 3.88 (td, *J* = 9.9, 9.4, 2.4 Hz, 2H);^{13}C NMR (100.67 MHz, $CDCl_3$): δ 166.0, 138.4, 137.5, 133.6, 133.3, 130.0, 130.0, 129.8, 129.1, 128.5, 128.5, 128.4, 128.4, 128.3, 128.3, 128.0, 128.0, 127.6, 126.2, 126.2, 117.7, 101.5, 96.3, 82.4, 76.1, 74.9, 73.6, 69.1, 68.8, 62.7; HRMS (ESI-MS): m/z calcd for $C_{30}H_{30}O_7$ $[M+Na]^+$: 525.1889, Found: 525.1879.

Allyl 2-*O*-levulinoyl-3-*O*-benzyl-α-D-glucopyranoside (S12a): This compound was prepared from compound **35a** following the above described procedure of **S7** (**a**-**e**). Eluent for purification: 60% ethyl acetate in *n*-hexane; Thick syrup; yield 3.0 g (91%) from 4.0 g; $[\alpha]^{25}_{D}$ ($CHCl_3$, *c*1.0): +85.6º; IR (cm^{-1}, $CHCl_3$): 3463, 3015, 2963, 1738, 1424, 1366, 1216, 1035, 752;^{1}H NMR (399.78 MHz, $CDCl_3$): δ 7.37 – 7.26 (m, 5H), 5.94 – 5.84 (m, 1H), 5.29 (dd, *J* = 17.4, 1.8 Hz, 1H), 5.20 (dd, *J* = 10.4, 1.3 Hz, 1H), 5.01 (d, *J* = 3.7 Hz, 1H), 4.84 – 4.76 (m, 2H), 4.73 (d, *J* = 11.8 Hz, 1H), 4.16 (dd, *J* = 13.1, 5.2 Hz, 1H), 3.99 (dd, *J* = 13.1, 6.1 Hz, 1H), 3.92 – 3.86 (m, 1H), 3.78 (s, 2H), 3.72 – 3.63 (m, 2H), 3.17 (s, 1H), 2.71 – 2.45 (m, 5H), 2.15 (s, 3H);^{13}C NMR (100.53 MHz, $CDCl_3$): δ 206.4, 172.3, 138.6, 133.9, 128.6,

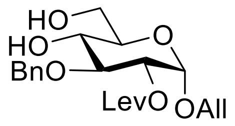

128.6, 127.9, 127.7, 127.7, 117.9, 95.3, 79.8, 75.2, 73.6, 71.2, 70.3, 68.6, 62.0, 37.9, 29.9, 28.0; HRMS (ESI-MS): m/z calcd for $C_{21}H_{28}O_8$ $[M+Na]^+$: 431.1682, Found: 431.1682.

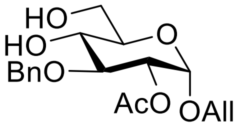

Allyl 2-*O*-acetyl-3-*O*-benzyl-α-D-glucopyranoside (S12b): This compound was prepared from compound **35b** following the above described procedure for **S7** (**a**-**e**). Eluent for purification: 60% ethyl acetate in *n*-hexane; thick syrup; yield 0.79 g (89%) from 1.11 g; $[\alpha]^{25}_D$ ($CHCl_3$, *c*1.0): +121.6º; IR (cm^{-1}, $CHCl_3$): 3383, 2922, 1737, 1455, 1369, 1241, 1035, 750; ^1H NMR (400.31 MHz, $CDCl_3$): δ 7.38 – 7.26 (m, 5H), 5.87 (dddd, *J* = 16.9, 10.3, 6.1, 5.1 Hz, 1H), 5.29 (dq, *J* = 17.2, 1.6 Hz, 1H), 5.21 (dq, *J* = 10.4, 1.4 Hz, 1H), 5.05 (d, *J* = 3.7 Hz, 1H), 4.84 – 4.71 (m, 3H), 4.17 (ddt, *J* = 13.1, 5.2, 1.5 Hz, 1H), 3.99 (ddt, *J* = 13.1, 6.2, 1.4 Hz, 1H), 3.89 (dd, *J* = 9.9, 8.0 Hz, 1H), 3.80 (d, *J* = 3.2 Hz, 2H), 3.72 – 3.64 (m, 2H), 2.96 (s, 1H), 2.36 (s, 1H), 2.04 (s, 3H); ^{13}C NMR (100.67 MHz, $CDCl_3$): δ 170.5, 138.6, 133.6, 128.7, 128.7, 128.0, 127.7, 127.7, 118.0, 95.3, 79.9, 75.3, 73.6, 71.2, 70.4, 68.5, 62.2, 21.0; HRMS (ESI-MS): m/z calcd for $C_{18}H_{24}O_7$ $[M+Na]^+$: 375.1420, Found: 375.1417.

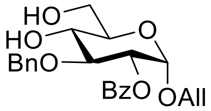

Allyl 2-*O*-benzoyl-3-*O*-benzyl-α-D-glucopyranoside (S12c): This compound was prepared from compound **35c** following the above mentioned procedure for **S7** (**a**-**e**). Eluent for purification: 50% ethyl acetate in *n*-hexane; Thick syrup; yield 17.5 g (91%) from 23.2 g; $[\alpha]^{25}_D$ ($CHCl_3$, *c*1.0): +145.8º; IR (cm^{-1}, $CHCl_3$): 3436, 3022, 2929, 1726, 1601, 1451, 1365, 1272, 1218, 1103, 1033, 749, 708; ^1H NMR (400.31 MHz, $CDCl_3$): δ 8.10 – 8.03 (m, 2H), 7.58 (tt, *J* = 7.0, 1.3 Hz, 1H), 7.48 – 7.42 (m, 2H), 7.27 – 7.17 (m, 5H), 5.90 – 5.72 (m, 1H), 5.25 (dq, *J* = 17.2, 1.6 Hz, 1H), 5.18 (d, *J* = 3.7 Hz, 1H), 5.11 (dq, *J* = 10.4, 1.3 Hz, 1H), 5.07 (dd, *J* = 9.9, 3.7 Hz, 1H), 4.85 (d, *J* = 11.5 Hz, 1H), 4.74 (d, *J* = 11.5 Hz, 1H), 4.18 (ddt, *J* = 13.3, 5.1, 1.5 Hz, 1H), 4.11 – 4.04 (m, 1H), 3.98 (ddt, *J* = 13.3, 5.9, 1.4 Hz, 1H), 3.87 – 3.81 (m, 2H), 3.81 – 3.72 (m, 2H), 3.16 (s, 1H), 2.55 (s, 1H); ^{13}C NMR (100.67 MHz, $CDCl_3$):

δ 166.0, 138.2, 133.6, 133.3, 129.8, 129.8, 129.7, 128.5(4C), 127.9, 127.9, 127.7, 117.5, 95.5, 79.8, 75.3, 74.0, 71.3, 70.4, 68.5, 62.1; HRMS (ESI-MS): m/z calcd for $C_{23}H_{26}O_7$ $[M+Na]^+$: 437.1576, Found: 437.1569.

36 (a-c): Preparation of Methyl Carboxylate via Selective 1°OH Oxidation: To a biphasic solution of the 4,6-diol (1 mmol) in 2:1 CH_2Cl_2−water (10 mL) was added BAIB (2.5 eq) and TEMPO (0.2 eq) simultaneously, stirred vigorously at 25 °C. After 3 h, the reaction was quenched by the addition of a saturated aqueous solution of Na_2SO_3 and extracted by CH_2Cl_2 (3 x 10 mL), the combined organic phases were washed with brine (10 mL), dried over Na_2SO_4 and concentrated *in vacuo*. The crude product was redissolved in anhydrous DMF (5 mL) and added 1.5 equivalent of K_2CO_3. After stirring for 15 min, iodomethane (2 eq) was added dropwise under argon atmosphere and stirred for 8 h at 25 °C in a dark place. After complete consumption, the reaction was arrested by adding saturated aqueous solution of Na_2SO_3, brine (5 mL) and water (10 mL); and extracted with EtOAc (2 x 10 mL). The combined organic phases were washed with brine, dried over anhydrous Na_2SO_4, concentrated under reduced pressure. The crude residue was purified by silica gel column chromatography using ethyl acetate and *n*-hexane as mobile phase to obtain the desired product.

Allyl 2-*O*-levulinoyl-3-*O*-benzyl-5*S*-5-methoxycarbonyl-α-D-xylopyranoside (36a): Eluent for purification: 42% ethyl acetate in *n*-hexane; Thick syrup; yield 1.3 g (72%, over two steps) from 1.7 g; $[\alpha]^{25}_D$ ($CHCl_3$, *c*1.0): +107.2º; IR (cm^{-1}, $CHCl_3$): 3467, 3023, 2958, 1741, 1442, 1366, 1213, 1050, 750;^1H NMR (399.78 MHz, $CDCl_3$): δ 7.36 – 7.24 (m, 5H), 5.96 – 5.84 (m, 1H), 5.31 (dq, *J* = 17.4, 1.5 Hz, 1H), 5.22 (dd, *J* = 10.5, 1.2 Hz, 1H), 5.10 (d, *J* = 3.6 Hz, 1H), 4.86 – 4.81 (m, 2H), 4.77 (d, *J* = 11.7 Hz, 1H), 4.26 – 4.18 (m, 2H), 4.04 (ddt, *J* = 12.6, 5.8, 1.2 Hz, 1H), 3.94 – 3.85 (m, 2H), 3.80 (s, 3H), 3.35 (s, 1H), 2.73 – 2.44 (m, 4H), 2.14 (s, 3H);^{13}C NMR (100.53 MHz, $CDCl_3$): δ 206.4, 172.1, 170.6, 138.6, 133.3, 128.5,

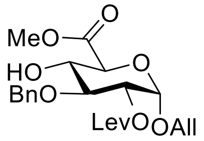

128.5, 127.8 127.7, 127.7, 118.2, 95.5, 78.7, 75.2, 72.5, 72.1, 70.6, 69.0, 52.8, 37.8, 29.9, 27.9; HRMS (ESI-MS): m/z calcd for $C_{22}H_{28}O_9$ $[M+Na]^+$: 459.1631, Found: 459.1648.

Allyl 2-*O*-acetyl-3-*O*-benzyl-5*S*-5-methoxycarbonyl-α-D-xylopyranoside (36b): Eluent for purification: 30% ethyl acetate in *n*-hexane; Thick syrup; yield 320 mg (74%, over two steps) from 400 mg; $[\alpha]^{25}_D$ ($CHCl_3$, *c*1.0): +110.0°; IR (cm^{-1}, $CHCl_3$): 3452, 2954, 2920, 1740, 1448, 1369, 1236, 1042, 747, 699; ^{1}H NMR (399.78 MHz, $CDCl_3$): δ 7.39 – 7.25 (m, 5H), 5.88 (dddd, *J* = 16.9, 10.9, 6.0, 5.1 Hz, 1H), 5.36 – 5.28 (m, 1H), 5.23 (dd, *J* = 10.4, 1.2 Hz, 1H), 5.14 (d, *J* = 3.6 Hz, 1H), 4.88 – 4.82 (m, 2H), 4.78 (d, *J* = 11.7 Hz, 1H), 4.28 – 4.19 (m, 2H), 4.04 (dd, *J* = 13.1, 6.2 Hz, 1H), 3.95 – 3.88 (m, 2H), 3.83 (s, 3H), 3.06 (d, *J* = 2.1 Hz, 1H), 2.05 (s, 3H); ^{13}C NMR (100.53 MHz, $CDCl_3$): δ 170.7, 170.3, 138.6, 133.2, 128.6, 128.6, 127.9, 127.7, 127.7, 118.3, 95.6, 78.8, 75.4, 72.5, 72.2, 70.5, 69.0, 52.9, 21.0; HRMS (ESI-MS): m/z calcd for $C_{19}H_{24}O_8$ $[M+Na]^+$: 403.1369, Found: 403.1362.

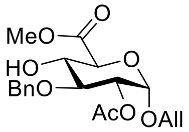

Allyl 2-*O*-benzoyl-3-*O*-benzyl-5*S*-5-methoxycarbonyl-α-D-xylopyranoside (36c): Eluent for purification: 20% ethyl acetate in *n*-hexane; thick syrup; yield 6.35 g (74%, over two steps) from 8.0 g; $[\alpha]^{25}_D$ ($CHCl_3$, *c*1.0): +143.6°; IR (cm^{-1}, $CHCl_3$): 3467, 3017, 2956, 1731, 1602, 1448, 1364, 1269, 1213, 1101, 1050, 925, 748, 709; ^{1}H NMR (400.31 MHz, $CDCl_3$): δ 8.11 – 8.05 (m, 2H), 7.61 (t, *J* = 7.4 Hz, 1H), 7.48 (t, *J* = 7.7 Hz, 2H), 7.31 – 7.22 (m, 5H), 5.91 – 5.78 (m, 1H), 5.34 – 5.27 (m, 2H), 5.17 (dd, *J* = 10.5, 1.3 Hz, 1H), 5.13 (dd, *J* = 9.9, 3.7 Hz, 1H), 4.86 (s, 2H), 4.33 (d, *J* = 9.8 Hz, 1H), 4.26 (ddt, *J* = 13.2, 5.0, 1.4 Hz, 1H), 4.15 – 4.09 (m, 1H), 4.09 – 3.98 (m, 2H), 3.87 (s, 3H), 3.16 (s, 1H); ^{13}C NMR (100.67 MHz, $CDCl_3$): δ 170.7, 165.9, 138.3, 133.4, 133.2, 129.9, 129.9, 129.7, 128.6, 128.6, 128.5, 128.5, 128.0, 128.0, 127.8, 118.0, 95.9, 78.6, 75.4, 72.9, 72.4, 70.6, 69.1, 52.9; HRMS (ESI-MS): m/z calcd for $C_{24}H_{26}O_8$ $[M+Na]^+$: 465.1525, Found: 465.1519.

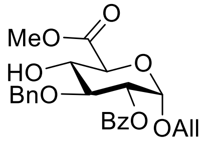

Allyl 2-*O*-levulinoyl-3-*O*-benzyl-4-*O*-acetyl-5*S*-5-methoxycarbonyl-α-D-xylopyranoside (37a): This compound was prepared from compound **36a** following a procedure described for the preparation of compound **20**. Eluent for purification: 30% ethyl acetate in *n*-hexane; Thick syrup; yield 335 mg (87%) from 350 mg; $[\alpha]^{25}_{D}$ ($CHCl_3$, *c*1.0): +79.0°; IR (cm^{-1}, $CHCl_3$): 3020, 1747, 1718, 1521, 1214, 1052, 744, 668; ^1H NMR (400.31 MHz, $CDCl_3$): δ 7.36 – 7.31 (m, 2H), 7.30 – 7.25 (m, 3H), 5.90 (dddd, *J* = 16.7, 10.4, 6.2, 5.3 Hz, 1H), 5.32 (dq, *J* = 17.2, 1.5 Hz, 1H), 5.27 – 5.22 (m, 1H), 5.19 – 5.12 (m, 2H), 4.92 (dd, *J* = 9.8, 3.7 Hz, 1H), 4.74 (d, *J* = 11.9 Hz, 1H), 4.66 (d, *J* = 11.9 Hz, 1H), 4.29 (d, *J* = 10.0 Hz, 1H), 4.22 (ddt, *J* = 13.0, 5.2, 1.4 Hz, 1H), 4.09 – 4.02 (m, 2H), 3.74 (s, 3H), 2.79 – 2.47 (m, 4H), 2.17 (s, 3H), 1.97 (s, 3H); ^{13}C NMR (100.67 MHz, $CDCl_3$): δ 206.2, 171.9, 169.6, 168.6, 138.2, 133.2, 128.4, 128.4, 127.8, 127.6, 127.6, 118.4, 95.3, 76.7, 75.0, 72.6, 71.1, 69.3, 69.0, 52.9, 37.8, 29.9, 27.9, 20.7; HRMS (ESI-MS): m/z calcd for $C_{24}H_{30}O_{10}$ $[M+Na]^+$: 501.1737, Found: 501.1741.

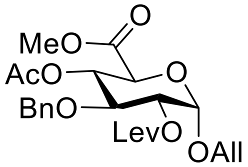

Allyl 2-*O*-benzoyl-3-*O*-benzyl-4-*O*-levulinoyl-5*S*-5-methoxycarbonyl-α-D-xylopyranoside (37b): This compound was prepared from compound **36c** following the procedure described for the preparation of compound **32e**. Eluent for purification: 20% ethyl acetate in *n*-hexane; Thick syrup; yield 527 mg (86%) from 500 mg; $[\alpha]^{25}_{D}$ ($CHCl_3$, *c*1.0): +95.8°; IR (cm^{-1}, $CHCl_3$): 3020, 2922, 2853, 1748, 1719, 1540, 1360, 1267, 1213, 1154, 1106, 1067, 928, 749, 668; ^1H NMR (400.31 MHz, $CDCl_3$): δ 8.07 – 8.00 (m, 2H), 7.59 (tt, *J* = 7.0, 1.3 Hz, 1H), 7.49 – 7.42 (m, 2H), 7.25 – 7.15 (m, 5H), 5.80 (dddd, *J* = 16.5, 10.5, 6.0, 5.2 Hz, 1H), 5.31 (d, *J* = 3.6 Hz, 1H), 5.30 – 5.20 (m, 2H), 5.19 – 5.12 (m, 2H), 4.77 – 4.68 (m, 2H), 4.35 (d, *J* = 10.0 Hz, 1H), 4.26 – 4.19 (m, 2H), 4.02 (ddt, *J* = 13.2, 6.1, 1.3 Hz, 1H), 3.75 (s, 3H), 2.75 – 2.38 (m, 4H), 2.16 (s, 3H); ^{13}C NMR (100.37 MHz, $CDCl_3$): δ 206.2, 171.6, 168.7, 165.7, 138.0, 133.5, 133.1, 129.9, 129.9, 129.6, 128.6, 128.6, 128.4,

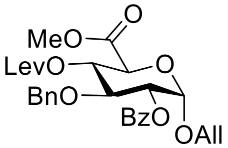

128.4, 127.9, 127.9, 127.8, 118.2, 95.6, 76.8, 75.1, 72.9, 71.6, 69.3, 69.0, 53.1, 37.8, 30.0, 27.8; HRMS (ESI-MS): m/z calcd for $C_{29}H_{32}O_{10}$ [M+Na]$^+$: 563.1893, Found: 563.1884.

Allyl 2-*O*-benzoyl-3-*O*-benzyl-4-*O*-^{t}-butyldimethylsilyl-5*S*-5-methoxycarbonyl-α-D-xylopyranoside (37c): This compound was prepared from compound **36c** following the procedure described for the compound **23**. Eluent for purification: 5% ethyl acetate in *n*-hexane; Thick syrup; yield 4.1 g (93%) from 3.5 g; $[\alpha]^{25}_{D}$ ($CHCl_3$, *c*1.0): +145.7º; IR (cm^{-1}, $CHCl_3$): 2938, 2860, 1753, 1725, 1601, 1453, 1328, 1264, 1102, 1051, 842, 777, 744, 710; ^{1}H NMR (399.78 MHz, $CDCl_3$): δ 7.91 – 7.86 (m, 2H), 7.45 – 7.40 (m, 1H), 7.32 – 7.26 (m, 2H), 7.09 – 7.01 (m, 5H), 5.77 – 5.65 (m, 1H), 5.17 (dq, *J* = 17.3, 1.6 Hz, 1H), 5.14 – 5.08 (m, 2H), 5.03 (dq, *J* = 10.6, 1.3 Hz, 1H), 4.74 (d, *J* = 11.2 Hz, 1H), 4.66 (d, *J* = 11.3 Hz, 1H), 4.24 – 4.18 (m, 1H), 4.13 (ddt, *J* = 13.2, 5.1, 1.5 Hz, 1H), 3.99 – 3.87 (m, 3H), 3.69 (s, 3H), 0.78 (s, 9H), -0.09 (s, 3H), -0.10 (s, 3H); ^{13}C NMR (100.53 MHz, $CDCl_3$): δ 169.8, 165.8, 138.2, 133.4, 133.3, 129.8, 129.8, 129.6, 128.5, 128.5, 128.2, 128.2, 127.4, 127.3, 127.3, 118.0, 99.0, 78.0, 75.3, 73.9, 72.6, 72.6, 69.0, 52.5, 25.9(3C), 18.0, -3.9, -5.1; HRMS (ESI-MS): m/z calcd for $C_{30}H_{40}O_8Si$[M+Na]$^+$: 579.2390, Found: 579.2394.

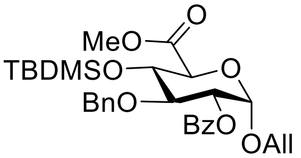

1-*O*-(((1-ethynylcyclohexyl)oxy)carbonyl)-2-*O*-levulinoyl-3-*O*-benzyl-4-*O*-acetyl-5*S*-5-methoxycarbonyl-α-D-xylopyranoside (38a): This compound was prepared from compound **37a** following the above described procedure for compound **S9** (**a**-**e**) and **33** (**a**-**e**). Eluent for purification: 25% ethyl acetate in *n*-hexane; white solid; yield 370 mg (72%) from 420 mg (over two steps); mp 92.1 ºC; $[\alpha]^{25}_{D}$ ($CHCl_3$, *c*1.0): +31.1º; IR (cm^{-1}, $CHCl_3$): 3277, 3018, 2942, 2863, 2115, 1751, 1444, 1367, 1224, 1152, 1040, 903, 752, 700; ^{1}H NMR (400.31 MHz, $CDCl_3$): δ 7.35 – 7.22 (m, 10H), 6.23 (d, *J* = 3.6 Hz, 1H), 5.61 (d, *J* = 7.2 Hz, 1H), 5.26 (t, *J* = 8.8 Hz, 1H), 5.20

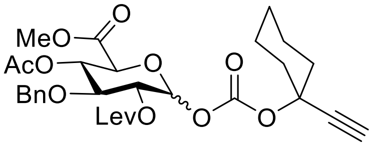

– 5.13 (m, 2H), 5.08 (dd, *J* = 9.8, 3.6 Hz, 1H), 4.74 (d, *J* = 11.9 Hz, 1H), 4.69 – 4.60 (m, 3H), 4.38 (d, *J* = 10.1 Hz, 1H), 4.09 (d, *J* = 8.9 Hz, 1H), 4.05 (t, *J* = 9.5 Hz, 1H), 3.82 – 3.77 (m, 1H), 3.70 (s, 3H), 3.69 (s, 3H), 2.79 – 2.59 (m, 6H), 2.54 – 2.48 (m, 4H), 2.24 – 2.08 (m, 10H), 1.97 (s, 3H), 1.95 (s, 3H), 1.93 – 1.73 (m, 4H), 1.72 – 1.54 (m, 10H), 1.35 – 1.28 (m, 2H); ^{13}C NMR (100.67 MHz, $CDCl_3$): δ 206.1, 205.9, 171.5, 171.0, 169.4, 169.4, 167.6, 167.3, 151.0, 150.5, 137.9, 137.6, 128.4(4C), 127.8(4C), 127.6(2C), 94.4, 92.3, 82.4(2C), 78.9, 78.8, 78.2, 76.1, 75.6, 75.4, 75.0, 74.0, 73.3, 71.1, 71.1, 70.9, 70.4, 70.1, 52.9, 52.9, 37.8, 37.7, 37.0, 36.8, 36.5, 36.4, 29.8, 29.8, 27.8, 27.7, 24.9, 24.9, 22.5(4C), 20.6, 20.5; HRMS (ESI-MS): m/z calcd for $C_{30}H_{36}O_{12}$ $[M+Na]^+$: 611.2104, Found: 611.2097.

1-*O*-(((1-ethynylcyclohexyl)oxy)carbonyl)-2-*O*-benzoyl-3-*O*-benzyl-4-*O*- levulinoyl-5*S*-5-methoxycarbonyl-α-D-xylopyranoside (38b) α:β (6:1): This compound was prepared from compound **37b** following the above described procedure for compound **S9** (**a**-**e**)and **33** (**a**-**e**). Eluent for purification: 35% ethyl acetate in *n*-hexane; white solid; yield 705 mg (72%) from 810 mg (over two steps); mp 86.1 ºC; $[\alpha]^{25}_D$ ($CHCl_3$, *c*1.0): +74.0º; IR (cm^{-1}, $CHCl_3$): 3284, 3012, 2940, 2862, 2117, 1747, 1447, 1366, 1228, 1152, 1101, 1015, 900, 753, 709; ^{1}H NMR (400.31 MHz, $CDCl_3$): δ 8.02 – 7.95 (m, 2H), 7.60 – 7.53 (m, 1H), 7.46 – 7.38 (m, 2H), 7.24 – 7.14 (m, 5H), 6.41 (d, *J* = 3.6 Hz, 1H), 5.40 – 5.34 (m, 1H), 5.27 (dd, *J* = 10.1, 9.2 Hz, 1H), 4.73 (s, 2H), 4.48 (d, *J* = 10.1 Hz, 1H), 4.25 (t, *J* = 9.5 Hz, 1H), 3.73 (s, 3H), 2.75 – 2.62 (m, 2H), 2.61 – 2.52 (m, 1H), 2.50 – 2.41 (m, 1H), 2.39 (s, 1H), 2.16 (s, 3H), 2.15 – 2.05 (m, 1H), 2.05 – 1.96 (m, 1H), 1.88 – 1.74 (m, 2H), 1.67 – 1.43 (m, 5H), 1.29 (dq, *J* = 9.6, 4.8 Hz, 1H); ^{13}C NMR (100.67 MHz, $CDCl_3$): δ 206.1, 171.5, 167.8, 165.2, 150.4, 137.8, 133.5, 130.0, 130.0, 129.20, 128.5, 128.5, 128.4, 128.4, 127.9, 127.8, 127.8, 92.6, 82.2, 79.0, 76.4, 75.4, 75.2, 71.4, 71.0, 71.0, 53.1, 37.7, 36.7, 36.6, 29.9, 27.7, 24.9, 22.6, 22.6; HRMS (ESI-MS): m/z calcd for $C_{35}H_{38}O_{12}$ $[M+Na]^+$: 673.2260, Found: 673.2263.

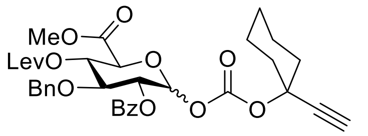

(38c): This compound was prepared from compound **37c** following the above described procedures for compound **S9** (**a**-**e**) and **33** (**a**-**e**). Hemiacetal intermediate: Eluent for purification: 20% ethyl acetate in *n*-hexane; Thick syrup; yield 1.9 g (82%) from 2.5 g;

2-*O*-benzoyl-3-*O*-benzyl-4-*O*- t-butyldimethylsilyl-5*S*-5-methoxycarbonyl-D-xylopyranoside: $[\alpha]^{25}_{D}$ ($CHCl_3$, *c*1.0): +118.2º; IR (cm^{-1}, $CHCl_3$): 3480, 2948, 2859, 1726, 1602, 1451, 1266, 1102, 1061, 841, 751, 711; ^1H NMR (399.78 MHz, $CDCl_3$): δ 7.98 (d, *J* = 7.4 Hz, 2H), 7.53 (t, *J* = 7.4 Hz, 1H), 7.38 (t, *J* = 7.7 Hz, 2H), 7.21 – 7.11 (m, 5H), 5.58 (t, *J* = 3.9 Hz, 1H), 5.19 – 5.11 (m, 1H), 4.85 – 4.73 (m, 2H), 4.51 (d, *J* = 8.3 Hz, 1H), 4.04 (p, *J* = 8.1 Hz, 2H), 3.78 – 3.71 (m, 4H), 0.86 (s, 9H), -0.01 (s, 3H), -0.02 (s, 3H); ^{13}C NMR (100.53 MHz, $CDCl_3$): δ 170.0, 166.0, 138.0, 133.4, 129.8, 129.8, 129.5, 128.5, 128.5, 128.2, 128.2, 127.5, 127.4, 127.4, 90.7, 79.1, 75.1, 73.8, 72.7, 72.2, 52.5, 25.8(3C), 18.0, -4.0, -5.2; HRMS (ESI-MS): m/z calcd for $C_{27}H_{36}O_8Si[M+Na]^+$: 539.2077, Found: 539.2080.

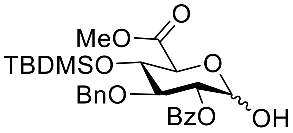

1-*O*-(((1-ethynylcyclohexyl)oxy)carbonyl)-2-*O*-benzoyl-3-*O*-benzyl-4-*O*-t-butyldimethyl silyl-5*S*-5-methoxycarbonyl-D-xylopyranoside (38c): (α:β ratio 4:1) Eluent for purification: 5% ethyl acetate in *n*-hexane; Thick syrup; yield 2.1 g (86%) from 1.9 g; $[\alpha]^{25}_{D}$ ($CHCl_3$, *c* 1.0): +72.9º; IR (cm^{-1}, $CHCl_3$): 3279, 2941, 2861, 2117, 1760, 1451, 1271, 1243, 1149, 1100, 1043, 1010, 899, 844, 754, 708; ^1H NMR (399.78 MHz, $CDCl_3$): δ 7.97 – 7.90 (m, 4H), 7.57 – 7.49 (m, 2H), 7.42 – 7.34 (m, 4H), 7.18 – 7.10 (m, 10H), 6.35 – 5.82 (m, 2H), 5.45 – 5.33 (m, 2H), 4.82 – 4.64 (m, 4H), 4.45 – 4.02 (m, 6H), 3.78 (s, 6H), 2.42 (s, 2H), 2.21 – 1.96 (m, 4H), 1.95 – 1.73 (m, 4H), 1.66 – 1.38 (m, 10H), 1.35 – 1.23 (m, 2H), 0.86 (s, 18H), -0.01 (d, 12H); ^{13}C NMR (100.53 MHz, $CDCl_3$): δ 168.8, 168.4, 165.3, 165.0, 151.1, 150.8, 137.9, 137.5, 133.4, 133.4, 130.0, 130.0, 129.9, 129.9, 129.4, 129.2, 128.4(4C), 128.2(4C), 127.8, 127.8, 127.6, 127.5, 127.4, 127.4, 95.0, 93.1, 82.5, 82.3,

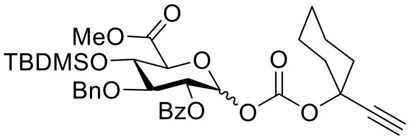

81.7, 79.5, 78.8, 78.5, 77.5, 75.5, 75.3, 75.1, 74.7, 74.6, 72.5, 72.3, 72.0, 71.7, 52.6, 52.6, 36.7, 36.7, 36.6, 36.5, 25.8(6C), 25.0, 24.9, 22.6, 22.6, 22.4, 22.3, 18.0, 18.0, -3.9, -4.1, -5.1, -5.2; HRMS (ESI-MS): m/z calcd for $C_{36}H_{46}O_{10}Si[M+Na]^+$: 689.2758, Found: 689.2758.

39 (a-i): To a solution of glycosyl donor (1.0 eq) and acceptor (0.8 eq) in anhydrous CH_2Cl_2 (5 mL/mmol) was added freshly activated 4Å MS powder (0.400 g/mmol) at 25 ºC under argon atmosphere. After 15 min of vigorous stirring at 25 °C, chloro[tris(2,4-di-tertbutylphenyl)phosphite]gold(I) **25** and AgOTf [8mol% each for reactions at 25 °C, 12mol% each for reactions at -20 °C and 15mol% each for reactions -40 °C] were added simultaneously to the reaction mixture at given temperature (Ref. Table 1) and stirred for 15 min to 1 h. After completion, the reaction was quenched by adding Et_3N, filtered through a bed of Celite®,the filtrate was concentrated *in vacuo* and crude residue was purified by silica gel column chromatography using ethyl acetate and hexane as mobile phase to afford the desired glycoside product.

Allyl 2-*O*-levulinoyl-3-*O*-benzyl-4-*O*-[2-*deoxy*-2-azido-3-*O*-benzyl-4-*O*-naphthyl-6-*O*-^{t}-butyldiphenylsilyl-D-glucopyranosyl]-5*S*-5-methoxycarbonyl-α-D-xylopyranoside (39a):

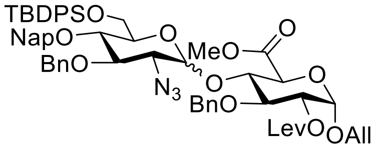

α:β ratio (4:1): Eluent for purification: 20% ethyl acetate in *n*-hexane; Thick syrup; yield 58 mg (77%) from 30 mg; ^1H NMR (400.31 MHz, $CDCl_3$): δ 7.90 – 7.85 (m, 1H), 7.83 – 7.66 (m, 7H), 7.55 – 7.51 (m, 2H), 7.46 – 7.33 (m, 14H), 7.32 – 7.28 (m, 2H), 7.26 – 7.16 (m, 1H), 5.94 (dddd, *J* = 16.8, 10.4, 6.3, 5.2 Hz, 1H), 5.52 (d, *J* = 3.6 Hz, 1H), 5.40 – 5.32 (m, 1H), 5.31 – 5.25 (m, 1H), 5.14 – 5.04 (m, 2H), 5.04 – 4.80 (m, 6H), 4.46 – 3.97 (m, 8H), 3.94 – 3.89 (m, 1H), 3.88 (s, 1H), 3.57 (s, 2H), 3.54 – 3.36 (m, 2H), 2.79 – 2.32 (m, 4H), 2.18 (s, 3H), 1.08 (s, 9H); ^{13}C NMR (100.67 MHz, $CDCl_3$): δ 206.2, 206.1, 172.1, 172.0, 169.9, 169.4, 139.1, 138.5, 137.9, 137.9, 136.0, 136.0, 135.9, 135.9, 135.8,

135.8, 135.7, 135.7, 135.6, 135.6, 133.7, 133.4, 133.4, 133.3(3C), 133.1, 133.0, 133.0, 132.9, 129.9, 129.8(3C), 128.6(4C), 128.3(4C), 128.2, 128.2, 128.1(3C), 128.0(3C), 127.9(4C), 127.8(4C), 127.7(6C), 127.4, 127.3, 127.3, 127.2, 126.4, 126.2, 126.2, 126.1, 126.0, 126.0, 125.7, 125.6, 118.4, 118.2, 101.3, 98.1, 95.5, 95.3, 83.5, 80.1, 79.8, 78.1, 77.9, 77.4, 76.2, 75.8, 75.8, 75.2, 75.1, 75.1, 74.9, 74.6, 73.4, 72.5, 72.4, 70.6, 70.2, 69.2, 69.2, 69.1, 67.2, 63.7, 62.3, 62.0, 52.9, 52.7, 37.9, 37.9, 29.9, 29.9, 27.9, 27.9, 27.0(6C), 19.5, 19.3;

Allyl 2-*O*-acetyl-3-*O*-benzyl-4-*O*-[2-*deoxy*-2-azido-3-*O*-benzyl-4-*O*-naphthyl-6-O-^{t}-butyldiphenylsilyl-D-glucopyranosyl]-5*S*-5-methoxycarbonyl-α-D-xylopyranoside (39b):

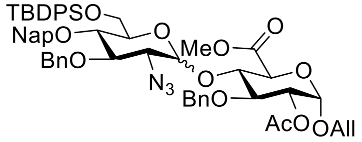

Eluent for purification: 12% ethyl acetate in *n*-hexane; thick syrup; yield 75 mg (79%) from 35 mg; $[\alpha]^{25}_{D}$ ($CHCl_3$, *c*1.0): +32.3º; IR (cm^{-1}, $CHCl_3$): 3002, 2929, 2860, 2108, 1750, 1460, 1367, 1268, 1039, 754, 703; ^{1}H NMR (399.78 MHz, $CDCl_3$): δ 7.86 – 7.82 (m, 1H), 7.79 – 7.74 (m, 2H), 7.69 – 7.62 (m, 5H), 7.52 – 7.47 (m, 2H), 7.35 (dddd, *J* = 17.4, 6.9, 5.3, 3.3 Hz, 15H), 7.28 – 7.24 (m, 2H), 5.91 – 5.81 (m, 1H), 5.47 (d, *J* = 3.6 Hz, 1H), 5.30 (dq, *J* = 17.2, 1.5 Hz, 1H), 5.23 (dd, *J* = 10.3, 1.3 Hz, 1H), 5.10 (d, *J* = 3.6 Hz, 1H), 5.05 (d, *J* = 11.3 Hz, 1H), 4.96 (d, *J* = 11.2 Hz, 1H), 4.95 – 4.88 (m, 4H), 4.84 (d, *J* = 11.2 Hz, 1H), 4.29 (d, *J* = 9.5 Hz, 1H), 4.21 (ddt, *J* = 13.2, 5.1, 1.4 Hz, 1H), 4.18 – 4.12 (m, 1H), 4.09 – 4.04 (m, 1H), 4.04 – 3.98 (m, 2H), 3.96 (dd, *J* = 7.4, 2.4 Hz, 2H), 3.85 (dd, *J* = 11.2, 1.5 Hz, 1H), 3.52 (s, 3H), 3.51 – 3.44 (m, 2H), 3.38 – 3.32 (m, 1H), 2.01 (s, 3H), 1.06 (s, 9H); ^{13}C NMR (100.53 MHz, $CDCl_3$): δ 170.3, 169.4, 138.4, 137.9, 136.0, 136.0, 135.9, 135.7, 135.7, 133.7, 133.4, 133.2, 133.0, 133.0, 129.8, 129.8, 128.7(4C), 128.3, 128.3, 128.2, 128.1, 128.0, 127.8(4C), 127.7, 127.7, 127.3, 127.3, 126.3, 126.2, 126.0, 125.6, 118.4, 98.2, 95.3, 80.2, 79.8, 77.9, 75.8, 75.3, 75.1, 75.0, 73.2, 72.5, 70.7, 69.0, 63.7, 62.0, 52.7, 27.0(3C), 20.9, 19.5; HRMS (ESI-MS): m/z calcd for $C_{59}H_{65}N_3O_{12}Si[M+Na]^+$: 1058.4235, Found: 1058.4208.

Allyl 2-*O*-acetyl-3-*O*-benzyl-4-*O*-[2-*deoxy*-2-azido-3-*O*-benzyl-4-*O*-(4-methoxybenzyl)-6-O-t-butyldiphenylsilyl-D-glucopyranosyl]-5*S*-5-methoxycarbonyl-α-D-xylopyranoside (39c):

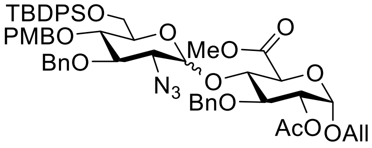

Eluent for purification: 20% ethyl acetate in *n*-hexane; Thick syrup; yield 60 mg (83%) from 27 mg; $[\alpha]^{25}_{D}$ ($CHCl_3$, *c*1.0): +42.5º; IR (cm^{-1}, $CHCl_3$): 3036, 2926, 2859, 2108, 1748, 1613, 1513, 1460, 1362, 1241, 1147, 1108, 1034, 820, 744, 700; ^{1}H NMR (399.78 MHz, $CDCl_3$): δ 7.69 – 7.64 (m, 4H), 7.42 – 7.30 (m, 16H), 7.13 – 7.09 (m, 2H), 6.84 – 6.80 (m, 2H), 5.91 – 5.81 (m, 1H), 5.44 (d, *J* = 3.7 Hz, 1H), 5.30 (dq, *J* = 17.0, 1.4 Hz, 1H), 5.23 (dd, *J* = 10.3, 1.3 Hz, 1H), 5.09 (d, *J* = 3.6 Hz, 1H), 4.97 – 4.89 (m, 3H), 4.87 (d, *J* = 10.5 Hz, 1H), 4.82 (dd, *J* = 10.9, 3.7 Hz, 2H), 4.68 (d, *J* = 10.5 Hz, 1H), 4.26 (d, *J* = 9.4 Hz, 1H), 4.20 (ddt, *J* = 13.3, 5.0, 1.4 Hz, 1H), 4.16 – 4.10 (m, 1H), 4.07 – 4.03 (m, 1H), 4.02 – 3.94 (m, 2H), 3.93 – 3.85 (m, 2H), 3.84 – 3.79 (m, 4H), 3.49 (s, 3H), 3.41 (d, *J* = 9.2 Hz, 1H), 3.31 (dd, *J* = 9.9, 3.7 Hz, 1H), 2.00 (s, 3H), 1.05 (s, 9H); ^{13}C NMR (100.53 MHz, $CDCl_3$): δ 170.4, 169.3, 159.3, 138.4, 138.0, 136.0, 136.0, 135.8, 135.8, 133.7, 133.2, 133.1, 130.6, 129.8, 129.8, 129.3, 129.3, 128.7, 128.7, 128.6, 128.6, 128.3, 128.3, 128.1, 127.8(3C), 127.7, 127.7, 127.3, 127.3, 118.4, 113.9, 113.9, 98.1, 95.3, 80.1, 79.7, 77.7, 75.7, 75.2, 75.0, 74.8, 73.2, 72.5, 70.7, 68.9, 63.7, 61.9, 55.4, 52.7, 27.0(3C), 20.9, 19.5; HRMS (ESI-MS): m/z calcd for $C_{56}H_{65}N_3O_{13}Si[M+Na]^+$: 1038.4184, Found: 1038.4170.

Allyl 2-*O*-acetyl-3-*O*-benzyl-4-*O*-[2-*deoxy*-2-azido-3-*O*-benzyl-4-*O*-benzyl-6-O-t-butyldiphenylsilyl-D-glucopyranosyl]-5*S*-5-methoxycarbonyl-α-D-xylopyranoside (39d):

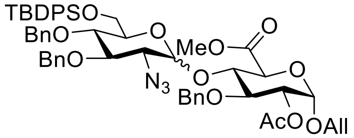

Eluent for purification: 15% ethyl acetate in *n*-hexane; Thick syrup; yield 63 mg (87%) from 28 mg; $[\alpha]^{25}_{D}$ ($CHCl_3$, *c*1.0): +48.1º; IR (cm^{-1}, $CHCl_3$): 3034, 2926, 2859, 2108, 1748, 1459, 1363, 1235, 1147, 1107, 1034, 742, 699; ^{1}H NMR (399.78 MHz, $CDCl_3$): δ 7.67 (dd, *J* = 8.1, 1.4 Hz, 2H), 7.63 (dd, *J* = 8.1, 1.3 Hz, 2H), 7.40 – 7.29 (m, 19H), 7.22 – 7.19 (m, 2H), 5.86 (dddd, *J* = 16.9, 10.6, 6.3, 5.1 Hz, 1H), 5.45 (d, *J* =

3.7 Hz, 1H), 5.30 (dq, J = 17.1, 1.5 Hz, 1H), 5.23 (dd, J = 10.3, 1.3 Hz, 1H), 5.09 (d, J = 3.6 Hz, 1H), 4.95 (d, J = 11.2 Hz, 1H), 4.92 (d, J = 3.6 Hz, 1H), 4.91 – 4.85 (m, 3H), 4.83 (d, J = 11.2 Hz, 1H), 4.77 (d, J = 11.1 Hz, 1H), 4.27 (d, J = 9.5 Hz, 1H), 4.20 (ddt, J = 13.3, 5.0, 1.3 Hz, 1H), 4.16 – 4.11 (m, 1H), 4.08 – 4.03 (m, 1H), 4.02 – 3.95 (m, 2H), 3.94 – 3.87 (m, 2H), 3.82 (dd, J = 11.5, 1.2 Hz, 1H), 3.50 (s, 3H), 3.43 (d, J = 8.9 Hz, 1H), 3.32 (dd, J = 10.0, 3.6 Hz, 1H), 2.00 (s, 3H), 1.04 (s, 9H); ^{13}C NMR (100.53 MHz, $CDCl_3$): δ 170.3, 169.4, 138.4, 138.4, 137.9, 136.0, 136.0, 135.7, 135.7, 133.7, 133.2, 133.0, 129.8, 129.8, 128.7, 128.7, 128.6, 128.6, 128.5, 128.5, 128.4, 128.4, 128.1, 127.8(4C), 127.7, 127.7, 127.5, 127.5, 127.3, 127.3, 118.5, 98.1, 95.3, 80.1, 79.8, 77.9, 75.8, 75.2, 75.0, 75.0, 73.2, 72.5, 70.7, 69.0, 63.7, 61.9, 52.7, 27.0(3C), 20.9, 19.5; HRMS (ESI-MS): m/z calcd for $C_{55}H_{63}N_3O_{12}Si[M+Na]^+$: 1008.4079, Found: 1008.4063.

Allyl 2-*O*-acetyl-3-*O*-benzyl-4-*O*-[2-*deoxy*-2-azido-3-*O*-benzyl-4-*O*-naphthyl-6-*O*-acetyl-D-glucopyranosyl]-5*S*-5-methoxycarbonyl-α-D-xylopyranoside

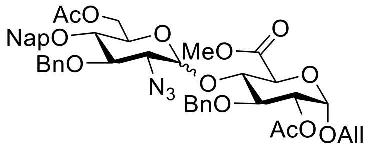

(39e): Eluent for purification: 25% ethyl acetate in *n*-hexane; thick syrup; yield 45 mg (76%) from 27 mg; $[\alpha]^{25}_D$ ($CHCl_3$, *c*1.0): +71.8º; IR (cm^{-1}, $CHCl_3$): 3037, 2924, 2858, 2107, 1744, 1687, 1454, 1367, 1235, 1128, 1030, 746, 699; ^{1}H NMR (399.78 MHz, $CDCl_3$): δ 7.84 – 7.77 (m, 3H), 7.68 – 7.65 (m, 1H), 7.51 – 7.46 (m, 2H), 7.41 – 7.27 (m, 11H), 5.88 (dddd, J = 16.8, 10.3, 6.2, 5.1 Hz, 1H), 5.51 (d, J = 3.7 Hz, 1H), 5.32 (dq, J = 17.1, 1.4 Hz, 1H), 5.25 (dq, J = 10.4, 1.3 Hz, 1H), 5.11 (d, J = 3.6 Hz, 1H), 4.97 (d, J = 11.0 Hz, 1H), 4.94 – 4.87 (m, 4H), 4.82 (d, J = 11.1 Hz, 1H), 4.74 (d, J = 11.2 Hz, 1H), 4.31 – 4.28 (m, 1H), 4.27 – 4.19 (m, 3H), 4.18 – 4.10 (m, 2H), 4.03 (ddt, J = 12.9, 5.9, 1.2 Hz, 1H), 3.95 (dd, J = 10.4, 8.4 Hz, 1H), 3.78 (s, 3H), 3.63 (dt, J = 10.1, 2.6 Hz, 1H), 3.57 (dd, J = 10.0, 8.4 Hz, 1H), 3.34 (dd, J = 10.3, 3.7 Hz, 1H), 1.99 (s, 3H), 1.87 (s, 3H); ^{13}C NMR (100.53 MHz, $CDCl_3$): δ 170.8, 170.3, 169.4, 138.3, 137.8, 135.0, 133.3, 133.2, 133.2, 128.7, 128.7, 128.6, 128.6, 128.5, 128.2(3C), 128.1, 127.9, 127.8, 127.2, 127.2, 127.0, 126.4, 126.3, 126.0, 118.5,

98.0, 95.4, 80.4, 79.7, 77.2, 75.7, 75.6, 75.2, 75.1, 73.3, 70.4, 69.8, 69.0, 63.5, 62.3, 52.9, 20.9, 20.7; HRMS (ESI-MS): m/z calcd for $C_{45}H_{49}N_3O_{13}$ [M+Na]$^+$: 862.3163, Found: 862.3164.

Allyl 2-*O*-acetyl-3-*O*-benzyl-4-*O*-[2-*deoxy*-2-azido-3-*O*-benzyl-4-*O*-naphthyl-6-O-levulinoyl-D-glucopyranosyl]-5*S*-5-methoxycarbonyl-α-D-xylopyranoside (39f): Eluent for purification: 30% ethyl acetate in *n*-hexane; thick syrup; yield 35 mg (68%) from 22 mg; $[\alpha]^{25}_D$ ($CHCl_3$, *c*1.0): +53.8º; IR (cm^{-1}, $CHCl_3$): 2922, 2860, 2108, 1743, 1645, 1457, 1363, 1237, 1154, 1047, 747, 699; ^{1}H NMR (399.78 MHz, $CDCl_3$): δ 7.84 – 7.76 (m, 3H), 7.69 – 7.66 (m, 1H), 7.50 – 7.45 (m, 2H), 7.40 – 7.27 (m, 11H), 5.88 (dddd, *J* = 17.0, 10.6, 6.2, 5.3 Hz, 1H), 5.50 (d, *J* = 3.7 Hz, 1H), 5.32 (dq, *J* = 17.3, 1.6 Hz, 1H), 5.24 (dd, *J* = 10.3, 1.3 Hz, 1H), 5.11 (d, *J* = 3.5 Hz, 1H), 4.99 – 4.93 (m, 2H), 4.92 – 4.87 (m, 3H), 4.81 (d, *J* = 11.1 Hz, 1H), 4.76 (d, *J* = 11.2 Hz, 1H), 4.30 – 4.20 (m, 4H), 4.16 – 4.10 (m, 2H), 4.05 – 3.99 (m, 1H), 3.94 (dd, *J* = 10.3, 8.1 Hz, 1H), 3.77 (s, 3H), 3.66 – 3.56 (m, 2H), 3.35 (dd, *J* = 10.3, 3.7 Hz, 1H), 2.68 – 2.59 (m, 2H), 2.52 – 2.38 (m, 2H), 2.13 (s, 3H), 1.99 (s, 3H); ^{13}C NMR (100.53 MHz, $CDCl_3$): δ 206.5, 172.5, 170.3, 169.5, 138.3, 137.8, 135.2, 133.3, 133.2, 133.1, 128.7, 128.7, 128.6, 128.6, 128.4, 128.2, 128.2, 128.1, 128.1, 127.8, 127.8, 127.3, 127.3, 126.9, 126.3, 126.2, 126.0, 118.5, 98.0, 95.4, 80.3, 79.7, 77.4, 75.7, 75.6, 75.2, 75.1, 73.3, 70.4, 69.9, 69.0, 63.5, 62.4, 53.0, 37.9, 29.9, 27.8, 20.9; HRMS (ESI-MS): m/z calcd for $C_{48}H_{53}N_3O_{14}$ [M+Na]$^+$: 918.3425, Found: 918.3400.

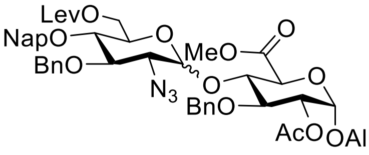

Allyl 2-*O*-benzoyl-3-*O*-benzyl-4-*O*-[2-*deoxy*-2-azido-3-*O*-benzyl-4-*O*-naphthyl-6-O-$^{t-}$butyldiphenylsilyl-D-glucopyranosyl]-5*S*-5-methoxycarbonyl-α-D-xylopyranoside (39g): Eluent for purification: 8% ethyl acetate in *n*-hexane; Thick syrup; yield 70 mg (81%) from 35 mg; $[\alpha]^{25}_D$ ($CHCl_3$, *c*1.0): +29.5º; IR (cm^{-1}, $CHCl_3$): 3036, 2926, 2859, 2108, 1731, 1459, 1267, 1103, 1038, 816, 745, 702; ^{1}H NMR

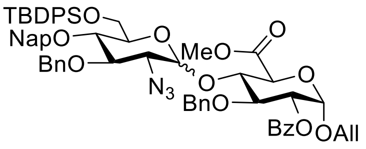

(400.31 MHz, $CDCl_3$): δ 8.09 (dd, *J* = 8.3, 1.2 Hz, 2H), 7.88 – 7.84 (m, 1H), 7.81 – 7.77 (m, 2H), 7.72 – 7.64 (m, 6H), 7.60 – 7.56 (m, 1H), 7.53 – 7.50 (m, 2H), 7.48 – 7.41 (m, 3H), 7.41 – 7.32 (m, 11H), 7.31 – 7.29 (m, 1H), 7.28 – 7.27 (m, 2H), 7.25 – 7.23 (m, 1H), 5.83 (dddd, *J* = 16.7, 10.3, 6.1, 5.0 Hz, 1H), 5.51 (d, *J* = 3.6 Hz, 1H), 5.29 (dq, *J* = 17.3, 1.6 Hz, 1H), 5.25 (d, *J* = 3.6 Hz, 1H), 5.22 (dd, *J* = 9.3, 3.6 Hz, 1H), 5.16 (dd, *J* = 10.4, 1.4 Hz, 1H), 5.06 (d, *J* = 11.2 Hz, 1H), 4.96 (d, *J* = 10.4 Hz, 2H), 4.90 (dd, *J* = 10.4, 3.7 Hz, 3H), 4.41 (d, *J* = 9.4 Hz, 1H), 4.37 – 4.32 (m, 1H), 4.24 (ddt, *J* = 13.1, 5.0, 1.5 Hz, 1H), 4.16 (dd, *J* = 9.3, 8.5 Hz, 1H), 4.06 – 4.00 (m, 2H), 3.99 – 3.95 (m, 2H), 3.89 (dd, *J* = 11.5, 1.4 Hz, 1H), 3.56 (s, 3H), 3.54 (d, *J* = 7.9 Hz, 1H), 3.39 – 3.33 (m, 1H), 1.08 (s, 9H); ^{13}C NMR (100.67 MHz, $CDCl_3$): δ 169.5, 165.9, 138.0, 137.9, 136.0, 136.0, 135.9, 135.7, 135.7, 133.7, 133.5, 133.4, 133.3, 133.1, 133.0, 129.9, 129.9, 129.8, 129.8, 129.6, 128.7, 128.7, 128.6, 128.6, 128.5, 128.5, 128.3, 128.3, 128.2, 128.1, 128.0, 127.8(4C), 127.7(4C), 126.3, 126.2, 126.0, 125.7, 118.1, 98.2, 95.5, 80.2, 79.6, 78.0, 75.7, 75.3, 75.1, 75.0, 73.4, 72.5, 70.9, 69.1, 63.7, 62.1, 52.7, 27.1(3C), 19.5; HRMS (ESI-MS): m/z calcd for $C_{64}H_{67}N_3O_{12}Si[M+Na]^+$: 1120.4392, Found: 1120.4384.

Allyl 2-*O*-benzoyl-3-*O*-benzyl-4-*O*-[2-*deoxy*-2-azido-3-*O*-benzyl-4-*O*-(4-methoxylbenzyl)-6-O-$^{t-}$butyldiphenylsilyl-D-glucopyranosyl]-5*S*-5-methoxycarbonyl-α-D-xylopyranoside

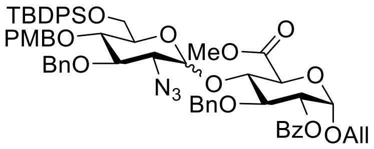

(39h): (α:β ratio 4:1):Eluent for purification: 15% ethyl acetate in *n*-hexane; Thick syrup; yield 55 mg (75%) from 30 mg; $[\alpha]^{25}_D$ ($CHCl_3$, *c*1.5): +43.4; IR (cm^{-1}, $CHCl_3$): 3034, 2928, 2860, 2106, 1728, 1611, 1513, 1457, 1259, 1102, 1035, 819, 742, 701; ^1H NMR (400.31 MHz, $CDCl_3$): δ 8.08 – 8.03 (m, 2H), 7.69 – 7.60 (m, 4H), 7.59 – 7.54 (m, 1H), 7.46 – 7.30 (m, 14H), 7.22 (d, *J* = 9.2 Hz, 4H), 7.17 – 7.09 (m, 2H), 6.87 – 6.79 (m, 2H), 5.80 (dddd, *J* = 17.0, 10.8, 6.1, 5.0 Hz, 1H), 5.46 (d, *J* = 3.7 Hz, 1H), 5.30 – 5.21 (m, 2H), 5.20 – 5.12 (m, 2H), 4.92 (d, *J* = 10.7 Hz, 1H), 4.89 – 4.84 (m, 2H), 4.84 – 4.78 (m, 2H), 4.68 (d, *J* = 10.4 Hz, 1H), 4.36 (d, *J* = 9.4 Hz, 1H), 4.31 (dd, *J* = 9.3, 8.4 Hz, 1H), 4.24 –

4.18 (m, 1H), 4.12 (dd, *J* = 9.4, 8.3 Hz, 1H), 4.04 – 3.93 (m, 2H), 3.89 (dd, *J* = 16.7, 8.5 Hz, 2H), 3.84 – 3.77 (m, 4H), 3.58 (s, 3H), 3.45 (d, *J* = 9.4 Hz, 1H), 3.30 (dd, *J* = 9.9, 3.6 Hz, 1H), 1.05 (s, 9H); ^{13}C NMR (100.67 MHz, $CDCl_3$): δ 169.4, 165.9, 159.3, 138.1, 137.9, 136.1, 136.1, 135.8, 135.8, 133.8, 133.5, 133.3, 133.1, 130.6, 129.9, 129.9, 129.8, 129.8, 129.6, 129.3, 129.3, 128.7(3C), 128.6, 128.6, 128.5, 128.5, 128.3, 128.3, 127.8(3C), 127.7(4C), 118.1, 113.9, 113.9, 98.2, 95.5, 80.1, 79.6, 77.8, 75.7, 75.3, 75.0, 74.8, 73.4, 72.6, 70.9, 69.1, 63.7, 62.0, 55.4, 52.7, 27.1(3C), 19.5; HRMS (ESI-MS): m/z calcd for $C_{61}H_{67}N_3O_{13}Si[M+Na]^+$: 1100.4341, Found: 1100.4325.

Allyl 2-*O*-benzoyl-3-*O*-benzyl-4-*O*-[2-*deoxy*-2-azido-3-*O*-benzyl-4-*O*-benzyl-6-O-^{t}-butyldiphenylsilyl-α-D-glucopyranosyl]-5*S*-5-methoxycarbonyl-α-D-xylopyranoside (39i):

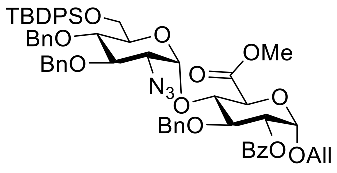

Eluent for purification: 7% ethyl acetate in *n*-hexane; yield 686 mg (85%) from 340 mg; α isomer: thick syrup; $[\alpha]^{25}_{D}$ ($CHCl_3$, *c*1.0): +6.9º; IR (cm^{-1}, $CHCl_3$): 3036, 2933, 2862, 2108, 1741, 1462, 1356, 1265, 1107, 1051, 871, 744, 699; ^{1}H NMR (400.31 MHz, $CDCl_3$): δ 8.10 (dd, *J* = 8.3, 1.3 Hz, 2H), 7.72 – 7.65 (m, 4H), 7.59 (tt, *J* = 7.0, 1.3 Hz, 1H), 7.48 – 7.30 (m, 17H), 7.28 – 7.26 (m, 2H), 7.26 – 7.20 (m, 4H), 5.83 (dddd, *J* = 16.6, 10.5, 6.1, 5.1 Hz, 1H), 5.50 (d, *J* = 3.7 Hz, 1H), 5.29 (dq, *J* = 17.3, 1.6 Hz, 1H), 5.25 (d, *J* = 3.5 Hz, 1H), 5.22 (dd, *J*= 9.3, 3.6 Hz, 1H), 5.16 (dq, *J* = 10.4, 1.3 Hz, 1H), 4.98 – 4.84 (m, 5H), 4.80 (d, *J* = 11.1 Hz, 1H), 4.41 (d, *J* = 9.4 Hz, 1H), 4.38 – 4.32 (m, 1H), 4.25 (ddt, *J* = 13.2, 5.0, 1.5 Hz, 1H), 4.16 (dd, *J* = 9.3, 8.5 Hz, 1H), 4.06 – 3.99 (m, 2H), 3.98 – 3.89 (m, 2H), 3.87 (dd, *J* = 11.5, 1.4 Hz, 1H), 3.56 (s, 3H), 3.51 (d, *J* = 9.1 Hz, 1H), 3.34 (dd, *J* = 10.0, 3.7 Hz, 1H), 1.08 (s, 9H); ^{13}C NMR (100.67 MHz, $CDCl_3$): δ 169.4, 165.8, 138.4, 138.0, 137.9, 136.0, 136.0, 135.7, 135.7, 133.7, 133.5, 133.3, 133.0, 129.9, 129.9, 129.8, 129.8, 129.6, 128.7, 128.7, 128.6, 128.6, 128.5(4C), 128.3, 128.3, 128.1, 127.8(4C), 127.7(4C), 127.5, 127.5, 118.1, 98.2, 95.5, 80.1, 79.6, 77.9, 75.7, 75.3, 75.0, 75.0, 73.4, 72.5, 70.9, 69.1, 63.7,

62.0, 52.7, 27.0(3C), 19.5; HRMS (ESI-MS): m/z calcd for $C_{60}H_{65}N_3O_{12}Si[M+Na]^+$: 1070.4235, Found: 1070.4225.

Allyl 2-*O*-benzoyl-3-*O*-benzyl-4-*O*-[2-*deoxy*-2-azido-3-*O*-benzyl-4-*O*-benzyl-6-O-$^{t-}$butyldiphenylsilyl-β-D-glucopyranosyl]-5*S*-5-methoxycarbonyl-α-D-xylopyranoside, β isomer: thick syrup; $[\alpha]^{25}_D$ ($CHCl_3$, *c*1.0): +48.9º; IR (cm^{-1}, $CHCl_3$): 3033, 2926, 2860, 2111, 1731, 1456, 1268, 1105, 1057, 748, 701; ^1H NMR (400.31 MHz, $CDCl_3$): δ 7.93 (dd, *J* = 8.3, 1.3 Hz, 2H),

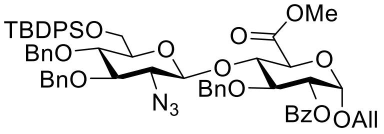

39iβ

7.74 – 7.70 (m, 2H), 7.64 (dd, *J* = 8.0, 1.3 Hz, 2H), 7.57 (tt, *J* = 7.1, 1.3 Hz, 2H), 7.44 – 7.27 (m, 16H), 7.15 – 7.02 (m, 7H), 5.87 – 5.76 (m, 1H), 5.27 (dq, *J* = 17.2, 1.6 Hz, 1H), 5.23 (d, *J* = 3.7 Hz, 1H), 5.18 (dd, *J* = 9.5, 3.8 Hz, 1H), 5.14 (dq, *J* = 10.4, 1.3 Hz, 1H), 4.90 – 4.81 (m, 3H), 4.77 – 4.66 (m, 3H), 4.46 – 4.39 (m, 2H), 4.33 (dd, *J* = 9.6, 8.5 Hz, 1H), 4.26 (ddt, *J* = 13.2, 5.0, 1.5 Hz, 1H), 4.16 (dd, *J* = 9.4, 8.5 Hz, 1H), 4.03 (ddt, *J* = 13.3, 6.1, 1.3 Hz, 1H), 3.89 – 3.77 (m, 6H), 3.47 – 3.35 (m, 2H), 3.17 (dq, *J* = 9.4, 1.7 Hz, 1H), 1.01 (s, 9H); ^{13}C NMR (100.67 MHz, $CDCl_3$): δ 170.0, 165.8, 138.6, 138.1, 138.0, 136.0, 136.0, 135.8, 135.8, 133.3(3C), 132.9, 130.0, 130.0, 129.9, 129.8, 129.7, 128.7, 128.7, 128.6, 128.6, 128.5, 128.5, 128.3, 128.3, 128.1(3C), 128.0, 128.0, 127.9(3C), 127.8, 127.8, 127.5, 127.5, 127.2, 118.0, 101.3, 95.7, 83.5, 78.2, 77.4, 77.0, 76.2, 75.9, 75.1, 74.3, 72.8, 70.3, 69.0, 67.2, 62.2, 52.9, 27.0(3C), 19.3; HRMS (ESI-MS): m/z calcd for $C_{60}H_{65}N_3O_{12}Si[M+Na]^+$: 1070.4235, Found: 1070.4233.

2-*O*-benzoyl-3-*O*-benzyl-4-*O*-[2-*deoxy*-2-azido-3,4-di-*O*-benzyl-6-O-$^{t-}$butyldiphenylsilyl-α-D-glucopyranosyl]-5*S*-5-methoxycarbonyl-D-xylopyranoside (S13): This compound was prepared from compound **39i** following the above described procedure for the preparation of compounds **S9** (**a**-**e**). Eluent for purification: 25% ethyl acetate in *n*-hexane; Thick syrup; yield 210

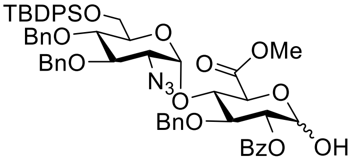

mg (95%) from 230 mg; $[\alpha]^{25}_{D}$ ($CHCl_3$, *c*1.0): +47.3º; IR (cm^{-1}, $CHCl_3$): 3357, 3032, 2932, 2862, 2108, 1732, 1455, 1267, 1146, 1103, 1035, 746, 701; ^1H NMR (400.31 MHz, $CDCl_3$): δ 8.10 – 8.04 (m, 2H), 7.70 – 7.63 (m, 4H), 7.52 – 7.47 (m, 1H), 7.44 – 7.28 (m, 17H), 7.28 – 7.27 (m, 2H), 7.26 – 7.18 (m, 4H), 5.66 – 5.59 (m, 1H), 5.33 (d, *J* = 3.6 Hz, 1H), 5.14 (dd, *J* = 7.6, 3.1 Hz, 1H), 4.90 – 4.84 (m, 3H), 4.78 – 4.69 (m, 2H), 4.62 (d, *J* = 10.6 Hz, 1H), 4.54 (d, *J* = 10.6 Hz, 1H), 4.35 (dd, *J* = 7.6, 6.7 Hz, 1H), 4.18 (t, *J* = 6.9 Hz, 1H), 4.00 (dd, *J* = 11.7, 2.8 Hz, 1H), 3.88 – 3.82 (m, 2H), 3.82 – 3.76 (m, 1H), 3.66 (dt, *J* = 9.5, 2.2 Hz, 1H), 3.54 (s, 3H), 3.33 – 3.23 (m, 2H), 1.05 (s, 9H); ^{13}C NMR (100.67 MHz, $CDCl_3$): δ 169.5, 165.9, 138.4, 138.0, 137.7, 136.0, 136.0, 135.7, 135.7, 133.6, 133.5, 133.1, 130.0, 130.0, 129.8, 129.8, 129.5, 128.8, 128.8, 128.6, 128.6, 128.5(4C), 128.2, 128.2, 128.0, 128.0, 127.8(5C), 127.7(4C), 99.1, 89.9, 80.0, 78.0, 77.6, 75.5, 75.4, 75.2, 74.4, 72.6, 72.2, 72.0, 63.6, 62.1, 52.5, 27.0(3C), 19.5; HRMS (ESI-MS): m/z calcd for $C_{57}H_{61}N_3O_{12}Si[M+Na]^+$: 1030.3922, Found: 1030.3907.

1-*O*-(((1-ethynylcyclohexyl)oxy)carbonyl)-2-*O*-benzoyl-3-*O*-benzyl-4-*O*-[2-*deoxy*-2-azido-3,4-di-*O*-benzyl-6-O-^{t}butyldiphenylsilyl-α-D-glucopyranosyl]-5*S*-5-methoxycarbonyl-D-xylopyranoside 40: This compound was prepared from compound **S13** by following the above described procedure for compounds **33** (**a**-**e**). Eluent for purification: 10% ethyl acetate in *n*-hexane; Thick syrup; yield 205 mg (85%) from 210 mg; $[\alpha]^{25}_{D}$ ($CHCl_3$, *c*1.0): +34.4º; IR (cm^{-1}, $CHCl_3$): 3286, 3031, 2934, 2861, 2107, 1759, 1454, 1270, 1240, 1143, 1099, 1032, 906, 747, 701; ^1H NMR (400.31 MHz, $CDCl_3$): δ 8.04 (td, *J* = 8.5, 1.4 Hz, 4H), 7.69 – 7.62 (m, 8H), 7.58 – 7.51 (m, 2H), 7.44 – 7.36 (m, 12H), 7.36 – 7.27 (m, 24H), 7.25 – 7.19 (m, 10H), 6.34 (d, *J* = 3.5 Hz, 1H), 5.87 (d, *J* = 6.2 Hz, 1H), 5.47 – 5.41 (m, 2H), 5.38 (dd, *J* = 9.4, 3.6 Hz, 1H), 5.34 (d, *J* = 3.7 Hz, 1H), 4.94 (d, *J* = 10.6 Hz, 1H), 4.90 (d, *J* = 6.7 Hz, 1H), 4.88 – 4.74 (m, 9H), 4.69 (d, *J* = 10.5 Hz, 1H), 4.50 (d, *J* = 9.4 Hz, 1H), 4.36 – 4.30 (m, 3H), 4.17 (dd, *J* = 9.5, 8.5

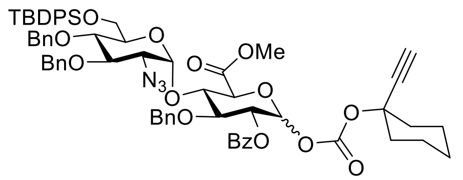

Hz, 1H), 4.06 (ddd, *J* = 7.3, 4.9, 2.4 Hz, 1H), 3.98 (ddd, *J* = 11.6, 4.3, 2.5 Hz, 2H), 3.91 – 3.80 (m, 6H), 3.58 – 3.55 (m, 1H), 3.54 (s, 3H), 3.53 (s, 3H), 3.45 (d, *J* = 7.8 Hz, 1H), 3.36 – 3.28 (m, 2H), 2.50 (s, 1H), 2.42 (s, 1H), 2.20 – 1.97 (m, 4H), 1.90 – 1.70 (m, 5H), 1.69 – 1.50 (m, 14H), 1.35 – 1.30 (m, 2H), 1.05 (s, 18H); ^{13}C NMR (100.53 MHz, $CDCl_3$): δ 168.6, 168.3, 165.4, 165.1, 151.1, 150.7, 138.4, 138.4, 137.9, 137.9, 137.6, 137.4, 136.0(4C), 135.7(4C), 133.7, 133.7, 133.6, 133.5, 133.1, 133.0, 130.1(4C), 129.9, 129.8(3C), 129.3, 129.3, 128.7(4C), 128.6(6C), 128.5(6C), 128.4, 128.4, 128.3, 128.3, 128.1, 128.1, 128.0(4C), 127.8(6C), 127.7(8C), 127.5, 127.5, 98.5, 98.2, 94.7, 92.6, 82.5, 82.4, 80.2, 80.0, 80.0, 79.3, 78.9, 78.6, 77.9, 77.9, 75.7, 75.6, 75.4, 75.3, 75.2, 75.1, 75.1, 74.9, 74.6, 74.5, 74.0, 72.8, 72.7, 72.6, 71.9, 71.3, 63.7, 63.6, 62.0, 61.9, 52.8, 52.8, 36.8, 36.8, 36.7, 36.6, 27.0(6C), 25.0, 25.0, 22.6, 22.6, 22.5, 22.4, 19.5, 19.5; HRMS (ESI-MS): m/z calcd for $C_{66}H_{71}N_3O_{14}Si[M+Na]^+$: 1180.4603, Found: 1180.4590.

2-benzoyloxy-3-*O*-benzyl-4-*O*-(2-*deoxy*-2-azido-3,4-di-O-benzyl-6-*O*-$^{t-}$butyldiphenylsilyl-α-D-glucopyranosyl)-5*S*-5-methoxycarbonyl-D-xylal (41): To a solution of glycosyl donor (1.0 eq) and acceptor (0.8 eq) in anhydrous CH_2Cl_2 (5 ml/mmol) was added freshly activated 4Å MS powder (0.400 g/mmol) at 25 °C under argon atmosphere. After 15 min of vigorous stirring at 25 °C, chloro[tris(2,4-di-t-butyl phenyl)phosphite]gold(I) (8mol%) **25** and AgOTf (8mol%) were added simultaneously to the reaction mixture and stirred for 15 min. After completion, the reaction mixture was quenched by adding excess of Et_3N and filtered through a bed of Celite®,the filtrate was concentrated *in vacuo* and the crude residue by silica gel column chromatography using 10% ethyl acetate and hexane as a mobile phase. Thick syrup; $[\alpha]^{25}_D$ (CH_2Cl_2, *c*1.1): +31.2º; IR (cm^{-1}, $CHCl_3$): 3032, 2925, 2858, 2106, 1738, 1688, 1457, 1261, 1447, 1038, 816, 742, 700; ^{1}H NMR (400.31 MHz, $CDCl_3$): δ 8.07 – 8.03 (m, 2H), 7.69 (ddt, *J* = 11.6, 6.8, 1.5 Hz, 4H), 7.59 (tt, *J* = 7.0, 1.3 Hz, 1H), 7.47 – 7.39 (m, 6H), 7.39 – 7.30 (m, 8H), 7.29 – 7.26 (m, 4H), 7.23 – 7.15

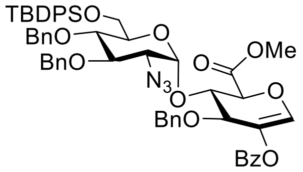

(m, 5H), 6.95 (s, 1H), 5.20 (d, *J* = 3.6 Hz, 1H), 4.93 – 4.91 (m, 3H), 4.87 (d, *J* = 10.7 Hz, 1H), 4.66 (d, *J* = 10.7 Hz, 1H), 4.59 – 4.52 (m, 3H), 4.29 (dd, *J* = 2.4, 1.2 Hz, 1H), 4.10 (dd, *J* = 10.3, 9.0 Hz, 1H), 3.96 – 3.86 (m, 3H), 3.74 (t, *J* = 9.3 Hz, 1H), 3.55 (s, 3H), 3.33 (dd, *J* = 10.3, 3.6 Hz, 1H), 1.06 (s, 9H); ^{13}C NMR (100.67 MHz, $CDCl_3$): δ 167.7, 164.8, 138.6, 138.1, 137.9, 137.5, 136.0, 136.0, 135.8, 135.8, 133.5, 133.5, 133.2, 130.2, 130.2, 129.9, 129.8, 129.8, 129.6, 128.7, 128.7, 128.6(4C), 128.5, 128.5, 128.4, 128.4, 128.1(5C), 128.0, 128.0, 127.9, 127.9, 127.7, 127.7, 99.0, 79.8, 78.4, 75.7, 75.5, 74.0, 73.4, 73.1, 71.2, 69.7, 63.5, 62.6, 52.3, 27.0(3C), 19.4; HRMS (ESI-MS): m/z calcd for $C_{57}H_{59}N_3O_{11}Si[M+Na]^+$: 1012.3817, Found: 1012.3810.

Compound 42: Eluent for purification: 20% ethyl acetate in *n*-hexane; Thick syrup; yield 45 mg (71%) from 35 mg; ^{1}H NMR (399.78 MHz, $CDCl_3$): δ 7.71 (td, *J* = 6.8, 6.3, 1.7 Hz, 4H), 7.43 – 7.38 (m, 5H), 7.37 – 7.31 (m, 4H), 7.30 – 7.28 (m, 2H), 6.03 (d, *J* = 3.3 Hz, 1H), 5.97 – 5.86 (m, 1H), 5.43 – 5.35 (m, 1H), 5.34 – 5.27 (m, 2H), 5.22 (dd, *J* = 10.3, 1.3 Hz, 1H), 4.98 (d, *J* = 3.6

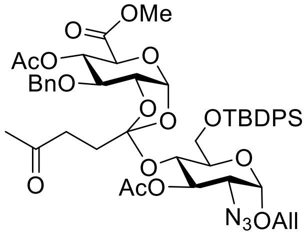

Hz, 1H), 4.67 (d, *J* = 11.8 Hz, 1H), 4.55 – 4.50 (m, 2H), 4.24 (ddt, *J* = 12.8, 5.0, 1.2 Hz, 1H), 4.15 (t, *J* = 2.6 Hz, 1H), 4.06 – 4.00 (m, 1H), 3.92 (dd, *J* = 11.2, 1.9 Hz, 1H), 3.88 (t, *J* = 2.7 Hz, 1H), 3.80 (dd, *J* = 11.3, 5.8 Hz, 1H), 3.75 – 3.71 (m, 1H), 3.64 – 3.58 (m, 1H), 3.53 (s, 3H), 3.21 (dd, *J* = 10.8, 3.5 Hz, 1H), 2.56 – 2.36 (m, 2H), 2.07 (s, 3H), 2.06 (s, 3H), 1.77 (s, 3H), 1.06 (s, 9H); ^{13}C NMR (100.53 MHz, $CDCl_3$): δ 207.8, 170.2, 169.8, 169.1, 136.5, 135.9, 135.9, 135.7, 135.7, 133.8, 133.4, 133.2, 129.8, 129.8, 128.7, 128.7, 128.5, 128.2, 128.2, 127.8, 127.8, 127.7, 127.7, 124.3, 118.3, 96.1, 94.1, 76.1, 72.7, 72.4, 71.6, 71.1, 70.6, 70.1, 68.4, 66.2, 62.8, 61.1, 52.4, 38.6, 33.7, 30.0, 27.0, 20.9(3C), 20.9, 20.9, 19.5; HRMS (ESI-MS): m/z calcd for $C_{48}H_{59}N_3O_{15}Si[M+Na]^+$: 968.3613, Found: 968.3600.

Allyl 2-*deoxy*-2-azido-3-*O*-(4-methoxybenzyl)-6-*O*-^{t}-butyldiphenylsilyl-4-*O*-(2-*O*-benzoyl-3-*O*-benzyl-4-*O*-levulinoyl-5*S*-5-(methoxycarbonyl)-β-D-xylopyranosyl)-α-D-glucopyranoside (43a): This compound was prepared by the glycosylation reaction of the donor **38b** and acceptor **31c** following the above described procedure for the preparation of compound **41**. Eluent

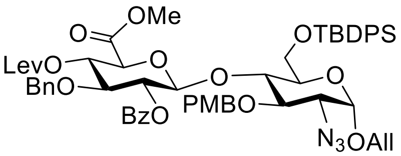

for purification: 25% ethyl acetate in *n*-hexane; Thick syrup; yield 22 mg (35%) from 35 mg; $[\alpha]^{25}_{D}$ ($CHCl_3$, *c*1.0): +68.8º; IR (cm^{-1}, $CHCl_3$): 3032, 2928, 2859, 2109, 1739, 1644, 1455, 1365, 1256, 1150, 1079, 1034, 752, 704; ^{1}H NMR (400.31 MHz, $CDCl_3$): δ 7.78 (t, *J* = 7.9 Hz, 4H), 7.70 – 7.67 (m, 2H), 7.52 – 7.43 (m, 7H), 7.38 (t, *J* = 7.2 Hz, 2H), 7.31 (t, *J* = 7.7 Hz, 2H), 7.16 – 7.12 (m, 5H), 6.91 (d, *J* = 8.6 Hz, 2H), 5.76 (ddt, *J* = 16.8, 11.3, 5.7 Hz, 1H), 5.39 (dd, *J* = 9.4, 8.3 Hz, 1H), 5.34 (t, *J* = 9.6 Hz, 1H), 5.21 – 5.18 (m, 1H), 5.16 (s, 1H), 5.12 – 5.08 (m, 1H), 4.98 (d, *J* = 8.1 Hz, 1H), 4.87 (d, *J* = 3.7 Hz, 1H), 4.65 (d, *J* = 11.2 Hz, 1H), 4.60 (d, *J* = 7.1 Hz, 1H), 4.55 (d, *J* = 11.6 Hz, 1H), 4.23 – 4.18 (m, 1H), 3.96 (dd, *J* = 13.0, 5.2 Hz, 1H), 3.93 – 3.84 (m, 4H), 3.81 (s, 3H), 3.71 – 3.66 (m, 5H), 3.33 (dd, *J* = 10.3, 3.7 Hz, 2H), 2.72 (t, *J* = 6.3 Hz, 2H), 2.63 – 2.48 (m, 2H), 2.18 (s, 3H), 1.10 (s, 9H); ^{13}C NMR (100.67 MHz, $CDCl_3$): δ 206.1, 171.4, 167.3, 164.6, 159.3, 137.6, 136.0, 136.0, 135.6, 135.6, 134.0, 133.3, 133.3, 132.5, 130.7, 130.5, 130.5, 130.2, 130.0, 129.7, 129.2, 128.6, 128.5, 128.3, 128.3, 128.1, 128.1, 128.0, 127.8(4C), 118.1, 113.8, 113.8, 100.3, 96.5, 79.8, 77.7, 76.7, 75.6, 74.2, 73.2, 73.1, 71.9, 71.2, 68.5, 62.9, 61.1, 55.4, 52.9, 37.8, 29.9, 27.9, 26.9(3C), 19.5; HRMS (ESI-MS): m/z calcd for $C_{59}H_{67}N_3O_{15}Si[M+Na]^+$: 1108.4239, Found: 1108.4225.

Allyl 2-*deoxy*-2-azido-3-*O*-(4-methoxybenzyl)-6-*O*-benzoyl-4-*O*-(2-*O*-benzoyl-3-*O*-benzyl-4-*O*-^{t}-butyldimethylsilyl-5*S*-5-(methoxycarbonyl)-β-D-xylopyranosyl)-α-D-glucopyranoside (44): This compound was prepared by the glycosylation reaction performed between the

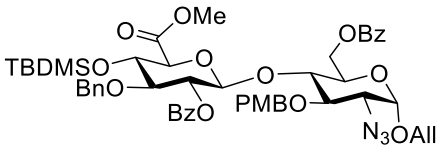

donor **38c** and acceptor **31a**following the above described procedure for the preparation of compound **41**. Eluent for purification: 8% ethyl acetate in *n*-hexane; white solid; yield 2.8 g (91%) from 1.5 g; $[\alpha]^{25}_D$ ($CHCl_3$, *c*1.0): +88.2º; IR (cm^{-1}, $CHCl_3$): 3028, 2948, 2862, 2109, 1733, 1610, 1514, 1456, 1261, 1141, 1100, 1068, 1036, 844, 754, 708; ^1H NMR (400.31 MHz, $CDCl_3$): δ 8.00 – 7.94 (m, 4H), 7.63 – 7.58 (m, 1H), 7.48 – 7.39 (m, 5H), 7.37 – 7.32 (m, 2H), 7.16 – 7.10 (m, 5H), 6.92 – 6.88 (m, 2H), 5.82 (dddd, *J* = 16.7, 10.4, 6.2, 5.3 Hz, 1H), 5.40 (t, *J* = 8.2 Hz, 1H), 5.21 (dq, *J* = 17.3, 1.6 Hz, 1H), 5.16 – 5.08 (m, 2H), 4.85 – 4.80 (m, 2H), 4.67 – 4.62 (m, 3H), 4.45 (dd, *J* = 12.2, 2.1 Hz, 1H), 4.39 (dd, *J* = 12.1, 4.1 Hz, 1H), 4.12 (t, *J* = 8.3 Hz, 1H), 4.07 (ddt, *J* = 12.9, 5.3, 1.5 Hz, 1H), 3.96 – 3.90 (m, 3H), 3.86 (d, *J* = 8.6 Hz, 1H), 3.81 (s, 3H), 3.76 – 3.71 (m, 1H), 3.67 – 3.62 (m, 4H), 3.34 – 3.27 (m, 1H), 0.82 (s, 9H), -0.04 (s, 6H); ^{13}C NMR (100.67 MHz, $CDCl_3$): δ 168.4, 166.1, 165.0, 159.3, 137.7, 133.5, 133.5, 133.1, 130.6, 130.3, 130.3, 129.8, 129.8, 129.7, 129.7, 129.2, 128.7(4C), 128.2, 128.2, 127.5(3C), 118.4, 113.9, 113.9, 101.2, 96.6, 82.6, 78.2, 77.8, 77.3, 75.5, 75.0, 74.2, 72.3, 69.1, 68.8, 63.0, 62.6, 55.4, 52.5, 25.8(3C), 18.0, -3.9, -5.1; HRMS (ESI-MS): m/z calcd for $C_{51}H_{61}N_3O_{14}Si[M+Na]^+$: 990.3820, Found: 990.3820.

Allyl 2-*deoxy*-2-azido-3-*O*-(4-methoxybenzyl)-6-*O*-benzoyl-4-*O*-(2-*O*-benzoyl-3-*O*-benzyl-5*S*-5-(methoxycarbonyl)-β-D-xylopyranosyl)-α-D-glucopyranoside (45): This compound was prepared from compound **44** following procedure described for the compound **4**. Eluent for purification: 35% ethyl acetate in *n*-hexane; white solid; yield 2.2 g (89%) from 2.8 g; mp 133.9 ºC;

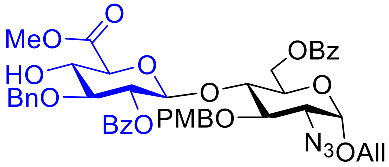

$[\alpha]^{25}_D$ ($CHCl_3$, *c*1.0): +97.4º; IR (cm^{-1}, $CHCl_3$): 3515, 3025, 2921, 2854, 2108, 1727, 1608, 1513, 1452, 1259, 1032, 826, 751, 705; ^1H NMR (400.31 MHz, $CDCl_3$): δ 8.05 – 8.01 (m, 2H), 7.95 – 7.91 (m, 2H), 7.58 (ddt, *J* = 8.7, 7.1, 1.3 Hz, 1H), 7.48 – 7.41 (m, 3H), 7.40 – 7.33 (m, 4H), 7.16

– 7.12 (m, 5H), 6.90 – 6.86 (m, 2H), 5.83 (dddd, *J* = 17.2, 10.4, 6.2, 5.3 Hz, 1H), 5.33 (dd, *J* = 9.5, 8.0 Hz, 1H), 5.23 (dq, *J* = 17.2, 1.6 Hz, 1H), 5.15 (dq, *J* = 10.4, 1.3 Hz, 1H), 5.06 (d, *J* = 10.5 Hz, 1H), 4.85 (d, *J* = 3.6 Hz, 1H), 4.78 – 4.66 (m, 4H), 4.46 – 4.38 (m, 2H), 4.12 – 4.01 (m, 2H), 3.99 – 3.91 (m, 3H), 3.81 – 3.77 (m, 4H), 3.73 (d, *J* = 9.8 Hz, 1H), 3.68 – 3.63 (m, 4H), 3.33 (ddd, *J* = 8.8, 3.7, 1.2 Hz, 1H), 2.99 (d, *J* = 2.8 Hz, 1H); ^{13}C NMR (100.67 MHz, $CDCl_3$): δ 169.2, 166.1, 165.0, 159.3, 137.8, 133.5, 133.5, 133.1, 130.7, 129.9, 129.9, 129.7(4C), 129.2, 128.7, 128.7, 128.6, 128.6, 128.5, 128.5, 128.1, 128.1, 127.9, 118.4, 113.8, 113.8, 101.4, 96.6, 81.1, 78.2, 77.9, 75.1, 74.8, 74.5, 73.3, 72.2, 69.0, 68.8, 63.0, 62.6, 55.4, 52.9; HRMS (ESI-MS): m/z calcd for $C_{45}H_{47}N_3O_{14}$ $[M+Na]^+$: 876.2956, Found: 876.2934.

Allyl 2-*deoxy*-2-azido-3-*O*-(4-methoxybenzyl)-6-*O*-benzoyl-4-*O*-(2-*O*-benzoyl-3-*O*-benzyl-4-*O*-(2-deoxy-2-azido-3,4-di-*O*-benzyl-6-O-^{t}butyldiphenylsilyl α-D-glucopyranosyl)-5*S*-5-(methoxycarbonyl)-β-D-xylopyranosyl)-α-D-glucopyranoside (46): To a solution of glycosyl donor **33c** (1.7 g, 2.2 mmol) and acceptor **45** (1.5 g, 0.8 eq) in anhydrous CH_2Cl_2 (10 mL) was added freshly activated 4Å MS powder (1.2 g/mmol) at 25 ºC under argon atmosphere,the reaction mixture was stirred vigorously at -45 ºC for 30 min. Then chloro[tris(2,4-di-tertbutylphenyl)phosphite]gold(I) **25** and AgOTf (15mol% each) were added simultaneously and the mixture stirred at -40 ºC for 1h. After completion, the reaction was quenched by adding Et_3N and filtered through a bed of Celite®,the filtrate was concentrated *in vacuo* and crude residue was purified by silica gel column chromatography using 15% ethyl acetate in hexane as a mobile phase to afford the glycosylated trisaccharide **46** (2.3 g, 90%) selectively as α-isomer. Thick syrup; $[\alpha]^{25}_{D}$ ($CHCl_3$, *c*1.0): +64.9º; IR (cm^{-1}, $CHCl_3$): 3031, 2931, 2863, 2107, 1731, 1609, 1512, 1456, 1261, 1069, 1033, 749, 703; ^{1}H NMR (400.31 MHz, $CDCl_3$): δ 8.09 (dd, *J* = 8.3, 1.3 Hz, 2H), 7.95 (dd, *J* = 8.4, 1.3 Hz, 2H), 7.69 – 7.63 (m, 4H), 7.58 – 7.53 (m, 1H), 7.51 – 7.46 (m,

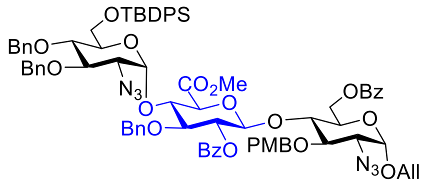

1H), 7.45 – 7.30 (m, 21H), 7.22 – 7.17 (m, 6H), 6.85 – 6.80 (m, 2H), 5.83 (dddd, *J* = 16.6, 10.4, 6.2, 5.3 Hz, 1H), 5.46 (dd, *J* = 8.8, 7.9 Hz, 1H), 5.40 (d, *J* = 3.7 Hz, 1H), 5.23 (dq, *J* = 17.2, 1.5 Hz, 1H), 5.15 (dq, *J* = 10.4, 1.2 Hz, 1H), 5.04 (d, *J* = 10.3 Hz, 1H), 4.91 – 4.80 (m, 6H), 4.77 (d, *J* = 11.1 Hz, 1H), 4.70 (d, *J* = 10.4 Hz, 1H), 4.62 (d, *J* = 10.3 Hz, 1H), 4.45 (qd, *J* = 12.2, 3.0 Hz, 2H), 4.26 – 4.21 (m, 1H), 4.08 (ddt, *J* = 12.9, 5.3, 1.4 Hz, 1H), 3.97 (dd, *J* = 3.4, 2.0 Hz, 1H), 3.96 – 3.89 (m, 5H), 3.89 – 3.83 (m, 3H), 3.78 – 3.74 (m, 1H), 3.73 (s, 3H), 3.42 – 3.36 (m, 4H), 3.30 (ddd, *J* = 10.0, 8.0, 3.7 Hz, 2H), 1.05 (s, 9H); ^{13}C NMR (100.67 MHz, $CDCl_3$): δ 167.9, 166.1, 165.0, 159.3, 138.4, 137.9, 137.4, 136.0, 136.0, 135.7, 135.7, 133.7, 133.7, 133.5, 133.1, 133.0, 130.4, 130.2, 130.2, 129.9(3C), 129.8, 129.7, 129.7, 129.6, 129.1, 128.8, 128.8, 128.7, 128.7, 128.6, 128.6, 128.5(4C), 128.3, 128.3, 128.1, 127.9, 127.8(5C), 127.7, 127.7, 127.5, 127.5, 118.4, 113.9, 113.9, 101.2, 97.7, 96.6, 82.4, 80.1, 78.3, 77.8, 77.8, 75.7, 75.4, 75.1, 75.1, 74.8, 74.6, 73.8, 72.6, 69.1, 68.8, 63.6, 63.0, 62.5, 61.9, 55.3, 52.6, 27.0(3C), 19.5; HRMS (ESI-MS): m/z calcd for $C_{81}H_{86}N_6O_{18}Si[M+Na]^+$: 1481.5666, Found: 1481.5657.

2-*deoxy*-2-Azido-3-*O*-(4-methoxybenzyl)-6-*O*-benzoyl-4-*O*-(2-*O*-benzoyl-3-*O*-benzyl-4-*O*-(2-*deoxy*-2-azido-3,4-di-*O*-benzyl-6-O-^{t}butyldiphenylsilyl α-D-glucopyranosyl)-5*S*-5-(methoxycarbonyl)-β-D-xylopyranosyl)-D-glucopyranoside (S14): This compound was prepared from compound **46** following the above described procedure for the preparation of **S9** (**a**-**e**).Eluent for purification: 25% ethyl acetate in *n*-hexane; Thick syrup; yield 1.7 g (87%) from 2.0 g; $[\alpha]^{25}_{D}$ (CH_2Cl_2, *c*0.5): +39.2º; IR (cm^{-1}, $CHCl_3$): 3030, 2926, 2860, 2108, 1734, 1609, 1512, 1457, 1261, 1069, 1033, 748, 702; ^{1}H NMR (400.31 MHz, $CDCl_3$): δ 8.11 – 8.04 (m, 4H), 7.99 – 7.92 (m, 4H), 7.65 (ddt, *J* = 13.1, 6.8, 1.5 Hz, 8H), 7.57 – 7.53 (m, 2H), 7.45 – 7.29 (m, 43H), 7.22 – 7.16 (m, 13H), 6.83 (dd, *J* = 8.6, 6.5 Hz, 4H), 5.49 – 5.38 (m, 4H), 5.18 (t, *J* = 3.5 Hz, 1H), 4.99 (dd,

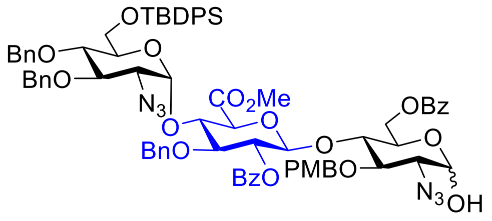

J = 36.4, 10.3 Hz, 2H), 4.89 – 4.74 (m, 12H), 4.71 – 4.61 (m, 4H), 4.52 – 4.34 (m, 5H), 4.26 – 4.18 (m, 2H), 3.98 – 3.91 (m, 8H), 3.90 – 3.81 (m, 9H), 3.73 (d, J = 4.2 Hz, 6H), 3.43 – 3.26 (m, 14H), 2.86 (s, 1H), 1.04 (s, 18H); ^{13}C NMR (100.67 MHz, $CDCl_3$): δ 167.8, 166.2, 165.1, 159.3, 138.3, 137.8, 137.4, 136.0, 136.0, 135.7, 135.7, 133.7, 133.7, 133.5, 133.0, 130.3, 130.3, 130.2, 129.9, 129.8 (3C), 129.7, 129.7, 129.6, 129.0, 128.8, 128.7, 128.6 (3C), 128.5 (5C), 128.3 (3C), 128.1, 127.9, 127.8 (4C), 127.7, 127.7, 127.5, 127.5, 113.9, 113.9, 101.1, 97.7, 91.8, 82.4, 80.1, 77.9, 77.8, 77.4, 75.7, 75.5, 75.0, 75.0, 74.8, 74.6, 73.8, 72.5, 69.0, 63.6, 63.5, 62.3, 61.8, 55.3, 52.6, 27.0 (3C), 19.5; HRMS (ESI-MS): m/z calcd for $C_{78}H_{82}N_6O_{18}Si[M+Na]^+$: 1441.5353, Found: 1441.5366.

1-*O*-(((1-ethynylcyclohexyl)oxy)carbonyl)-2-*deoxy*-2-Azido-3-*O*-(4-methoxybenzyl)-6-*O*-benzoyl-4-*O*-(2-*O*-benzoyl-3-*O*-benzyl-4-*O*-(2-deoxy-2-azido-3,4-di-*O*-benzyl-6-O-^{t}-butyldiphenylsilyl a-D-glucopyranosyl)-5*S*-5-(methoxycarbonyl)-β-D-xylopyranosyl)-D-glucopyranoside (47): This compound was prepared from compound **S14** following the above described procedure for the preparation of compound **33** (**a**-**e**). Eluent for purification:

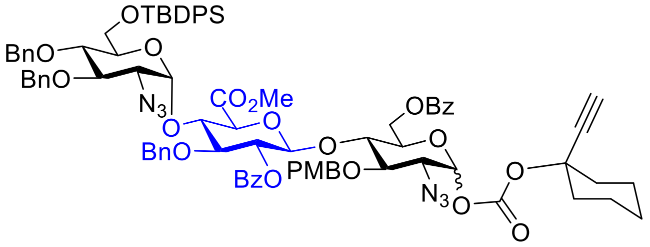

18% ethyl acetate in *n*-hexane; White solid; yield 1.65 g (88%) from 1.7 g; mp 78.7 ºC; $[\alpha]^{25}_D$ (CH_2Cl_2, c1.0): +30.9º; IR (cm^{-1}, $CHCl_3$): 3284, 2934, 2862, 2110, 1742, 1730, 1608, 1513, 1454, 1261, 1076, 1030, 911, 744, 702; ^{1}H NMR (400.31 MHz, $CDCl_3$): δ 8.10 – 8.05 (m, 2H), 7.99 – 7.95 (m, 2H), 7.65 (ddt, J = 12.9, 6.7, 1.5 Hz, 4H), 7.57 – 7.53 (m, 1H), 7.52 – 7.48 (m, 1H), 7.45 – 7.36 (m, 7H), 7.36 – 7.27 (m, 14H), 7.21 – 7.16 (m, 6H), 6.86 – 6.81 (m, 2H), 5.43 (dd, J = 9.1, 7.9 Hz, 1H), 5.39 (d, J = 3.7 Hz, 1H), 5.21 – 5.17 (m, 1H), 4.98 (d, J = 10.3 Hz, 1H), 4.90 – 4.81 (m, 4H), 4.75 (d, J = 11.1 Hz, 1H), 4.72 (d, J = 7.8 Hz, 1H), 4.69 (d, J = 10.5 Hz, 1H), 4.65 (d, J = 10.4 Hz, 1H), 4.48 – 4.38 (m, 2H), 4.20 (dd, J = 9.3, 8.5 Hz, 1H), 3.98 – 3.89 (m, 3H),

3.88 – 3.80 (m, 4H), 3.74 (s, 3H), 3.54 – 3.48 (m, 2H), 3.46 – 3.42 (m, 1H), 3.40 (s, 3H), 3.37 – 3.33 (m, 1H), 3.29 (dd, *J* = 9.9, 3.7 Hz, 1H), 2.57 (s, 1H), 2.16 – 2.06 (m, 2H), 1.89 – 1.77 (m, 2H), 1.67 – 1.63 (m, 1H), 1.58 – 1.46 (m, 4H), 1.33 – 1.27 (m, 1H), 1.04 (s, 9H); ^{13}C NMR (100.67 MHz, $CDCl_3$): δ 167.8, 166.0, 165.0, 159.5, 150.8, 138.4, 137.9, 137.4, 136.0, 136.0, 135.7, 135.7, 133.8, 133.7, 133.5, 133.0, 130.5, 130.5, 129.9(7C), 129.8, 129.5, 128.9, 128.9, 128.7(4C), 128.6, 128.6, 128.5, 128.5, 128.4, 128.4, 128.1, 127.9, 127.8(7C), 127.5, 127.5, 113.9, 113.9, 101.0, 97.7, 95.5, 82.4, 82.3, 80.7, 80.1, 79.1, 77.8, 77.1, 75.7, 75.7, 75.6, 75.1, 75.0, 74.7, 74.7, 73.8, 73.7, 72.6, 64.3, 63.6, 62.2, 61.9, 55.3, 52.7, 36.8, 36.7, 27.0(3C), 25.0, 22.6, 22.6, 19.5; HRMS (ESI-MS): m/z calcd for $C_{87}H_{92}N_6O_{20}Si[M+Na]^+$: 1591.6033, Found: 1591.6047.

Allyl 2-*deoxy*-2-azido-3-*O*-benzyl-6-*O*-acetyl-4-*O*-(2-*O*-acetyl-3-*O*-benzyl-4-*O*-(2-*deoxy*-2-azido-3-*O*-(4-methoxybenzyl)-6-*O*-benzoyl-4-*O*-(2-*O*-benzoyl-3-*O*-benzyl-5*S*-5-methoxycarbonyl-4-*O*-(2-*deoxy*-2-azido-3,4-di-*O*-benzyl-6-O-^{t}butyldiphenylsilyl-α-D-glucopyranosyl)-β-D-xylopyranosyl)-α-D-glucopyranosyl)-5*R*-5-(methoxycarbonyl)-β-D-xylopyranosyl)-α-D-glucopyranoside (50a): To a solution of the glycosyl donor **47** (25 mg, 0.016 mmol) and acceptor **4** (7.0 mg, 0.01 mmol) in anhydrous CH_2Cl_2 (0.5 mL) was added freshly activated 4Å MS powder at 25 °C under argon atmosphere. The reaction mixture was stirred for 30 min,

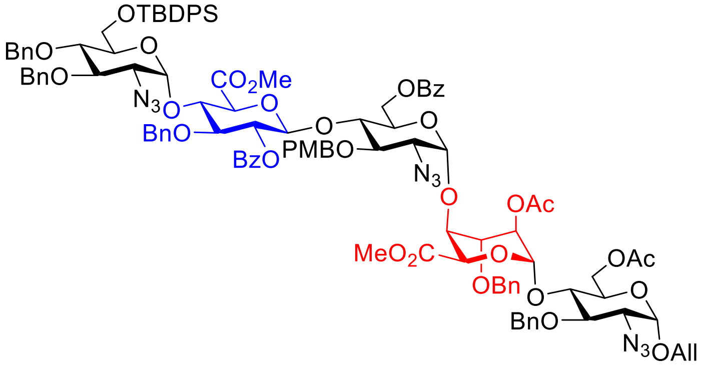

chloro[tris(2,4-di-tertbutylphenyl)phosphite]gold(I) **25** and AgOTf (10mol% each) were added simultaneously at -10 ºC and the mixture allowed to warm to 25 ºC over 30 min. After completion, the reaction was quenched with Et_3N and filtered through a bed of Celite®,the filtrate was concentrated in vacuo and crude residue was purified by silica gel column chromatography

using 20% ethyl acetate in hexane as mobile phase to afford the glycosylated pentasaccharide **50a** (18 mg, 86%) selectively as α-isomer. Thick syrup; $[\alpha]^{25}_{D}$ ($CHCl_3$, *c*0.9): +102.9º; IR (cm^{-1}, $CHCl_3$): 2956, 2926, 2862, 2108, 1737, 1456, 1369, 1256, 1146, 1105, 1071, 1035, 745, 706; ^1H NMR (600.40 MHz, $CDCl_3$): δ 8.08 (d, *J* = 7.7 Hz, 2H), 7.99 (d, *J* = 7.7 Hz, 2H), 7.65 (dd, *J* = 19.8, 7.0 Hz, 5H), 7.53 (t, *J* = 7.4 Hz, 2H), 7.46 – 7.39 (m, 6H), 7.38 – 7.35 (m, 4H), 7.34 – 7.30 (m, 11H), 7.29 – 7.27 (m, 4H), 7.25 – 7.24 (m, 1H), 7.22 – 7.17 (m, 8H), 7.15 (dd, *J* = 9.3, 7.2 Hz, 2H), 6.81 (d, *J* = 8.6 Hz, 2H), 5.93 (ddt, *J* = 16.6, 11.0, 5.7 Hz, 1H), 5.43 (t, *J* = 8.5 Hz, 1H), 5.39 (d, *J* = 3.7 Hz, 1H), 5.37 – 5.33 (m, 1H), 5.27 (d, *J* = 10.3 Hz, 1H), 5.19 (d, *J* = 4.0 Hz, 1H), 5.03 (d, *J* = 10.3 Hz, 1H), 4.93 (d, *J* = 3.7 Hz, 1H), 4.90 – 4.85 (m, 4H), 4.84 – 4.80 (m, 2H), 4.78 – 4.73 (m, 3H), 4.69 – 4.64 (m, 4H), 4.53 (d, *J* = 10.4 Hz, 1H), 4.49 (d, *J* = 4.2 Hz, 1H), 4.46 (d, *J* = 12.2 Hz, 1H), 4.40 – 4.34 (m, 2H), 4.24 – 4.15 (m, 3H), 4.04 (dd, *J* = 12.9, 6.3 Hz, 1H), 3.96 – 3.86 (m, 6H), 3.86 – 3.81 (m, 6H), 3.73 – 3.69 (m, 4H), 3.66 (dd, *J* = 10.3, 8.6 Hz, 1H), 3.41 – 3.34 (m, 5H), 3.32 (dd, *J* = 10.0, 3.7 Hz, 1H), 3.25 (s, 3H), 3.21 (dd, *J* = 10.3, 3.6 Hz, 1H), 2.12 (s, 3H), 2.04 (s, 3H), 1.03 (s, 9H); ^{13}C NMR (150.99 MHz, $CDCl_3$): δ 170.9, 170.2, 169.3, 167.7, 166.0, 164.8, 159.3, 138.3, 137.9, 137.7, 137.4, 137.3, 136.0, 136.0, 135.7, 135.7, 133.9, 133.6, 133.6, 133.1, 132.9, 130.2, 130.2, 130.1, 129.9, 129.9, 129.8, 129.8, 129.7, 129.7, 129.5, 129.0, 129.0, 128.9, 128.8, 128.8, 128.7, 128.7, 128.6 (4C), 128.5, 128.5, 128.4, 128.4, 128.3, 128.3, 128.2, 128.1, 127.9 (3C), 127.8 (7C), 127.7, 127.7, 127.6, 127.5, 127.5, 118.5, 113.8, 113.8, 100.8, 97.9, 97.8, 97.1, 96.6, 82.5, 80.2, 78.4, 77.7, 77.4, 77.2, 75.9, 75.8, 75.5, 75.0, 75.0, 74.9, 74.9, 74.8, 73.8, 73.6, 73.6, 72.6, 72.5, 69.6, 69.5, 69.3, 69.2, 68.8, 63.6, 63.3, 62.6, 62.2, 62.0, 61.8, 55.3, 52.7, 51.8, 27.0 (3C), 21.0, 20.9, 19.5; HRMS (ESI-MS): m/z calcd for $C_{112}H_{121}N_9O_{30}Si[M+Na]^+$: 2122.7886, Found: 2122.7530.

1,2-*O*-isopropylidene-3-*O*-benzyl-4-*O*-(2-*deoxy*-2-azido-3-*O*-(4-methoxybenzyl)-6-*O*-benzoyl-4-*O*-(2-*O*-benzoyl-3-*O*-benzyl-5*S*-5-methoxycarbonyl-4-*O*-(2-*deoxy*-2-azido-3,4-di-*O*-benzyl-6-*O*-$^{t-}$butyldiphenylsilyl-α-D-glucopyranosyl)-β-D-xylopyranosyl)-α-D-glucopyranosyl)-5*R*-5-(methoxycarbonyl)-α-D-xylopyranose (48): To a solution of glycosyl donor **47** (340 mg, 0.217 mmol) and acceptor **5** (61 mg, 0.18 mmol) in anhydrous CH_2Cl_2 (2.0 mL) was added freshly activated 4Å MS powder at 25 ºC under argon atmosphere. After vigorous stirring for 30 min,

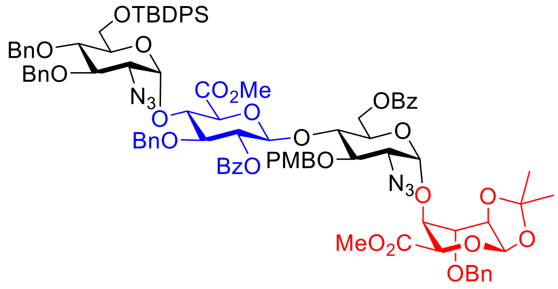

chloro[tris(2,4-di-tertbutylphenyl)phosphite]gold(I) **25** and AgOTf (8mol% each) were added simultaneously and the reaction mixture was stirred for 15 min, the reaction was quenched with Et_3N and filtered through a bed of Celite®,the filtrate was concentrated *in vacuo* and the crude residue was purified by silica gel column chromatography using 18% ethyl acetate in hexane as mobile phase to afford the glycosylated tetrasaccharide **48** (286 mg, 91%) selectively as α-isomer. White solid; mp 88.1 ºC; $[\alpha]^{25}_D$ ($CHCl_3$, *c*1.0): +49.0º; IR (cm^{-1}, $CHCl_3$): 3025, 2932, 2109, 1732, 1513, 1456, 1369, 1265, 1216, 1138, 1071, 1036, 750, 707;^{1}H NMR (400.31 MHz, $CDCl_3$): δ 8.11 – 8.06 (m, 2H), 8.03 – 7.99 (m, 2H), 7.69 – 7.63 (m, 4H), 7.57 – 7.50 (m, 2H), 7.46 – 7.41 (m, 4H), 7.39 – 7.36 (m, 5H), 7.35 – 7.30 (m, 13H), 7.29 – 7.28 (m, 2H), 7.23 – 7.15 (m, 8H), 6.82 (d, *J* = 8.6 Hz, 2H), 5.47 – 5.41 (m, 1H), 5.40 (d, *J* = 3.6 Hz, 1H), 5.26 (d, *J* = 2.3 Hz, 1H), 5.02 (d, *J* = 10.3 Hz, 1H), 4.90 (d, *J* = 10.9 Hz, 1H), 4.86 – 4.81 (m, 3H), 4.80 – 4.75 (m, 3H), 4.67 (d, *J* = 10.4 Hz, 1H), 4.62 – 4.55 (m, 4H), 4.39 (dd, *J* = 12.4, 3.1 Hz, 1H), 4.34 (d, *J* = 1.4 Hz, 1H), 4.22 (t, *J* = 8.9 Hz, 1H), 4.13 – 4.09 (m, 1H), 3.99 – 3.94 (m, 3H), 3.94 – 3.89 (m, 3H), 3.88 – 3.83 (m, 3H), 3.82 – 3.76 (m, 2H), 3.72 (s, 3H), 3.54 (s, 3H), 3.39 (s, 3H), 3.38 – 3.29 (m, 3H), 1.43 (s, 3H), 1.36 (s, 3H), 1.05 (s, 9H); ^{13}C NMR (100.67 MHz, $CDCl_3$): δ 169.0, 167.7, 166.0, 164.8, 159.3, 138.4, 137.8, 137.4, 137.1, 136.0, 136.0, 135.7, 135.7, 133.7, 133.7,

133.5, 133.0, 130.4, 130.4, 130.3, 129.9 (3C), 129.8, 129.7 (3C), 129.1, 128.9, 128.9, 128.7 (4C), 128.6, 128.6, 128.5 (4C), 128.5, 128.3 (3C), 128.1, 127.9, 127.9, 127.8 (6C), 127.7, 127.7, 127.5, 127.5, 113.8, 113.8, 112.3, 100.8, 97.7, 97.6, 96.9, 82.6, 80.2, 77.8, 77.3, 77.2, 75.7, 75.4, 75.4, 75.1, 75.0, 74.9, 74.7, 73.6, 73.1, 72.8, 72.6, 72.5, 71.3, 69.9, 63.6, 63.3, 62.2, 61.8, 55.3, 52.6, 52.4, 27.9, 27.0 (3C), 26.3, 19.5; HRMS (ESI-MS): m/z calcd for $C_{95}H_{102}N_6O_{24}Si[M+Na]^+$: 1761.6612, Found: 1761.6615.

S15: Acetonide and PMB Deprotection: To the compound **48** (275 mg, 0.158 mmol) in a 25 mL round bottom flask was added 75% aq. dichloroacetic acid (5 mL) at 0 ºC and the solution was stirred for 1 h. The reaction mixture was diluted with ice-cold water (25 mL), neutralized by portion-wise addition of the solid $NaHCO_3$ and extracted with CH_2Cl_2 (3 x 15 mL). The combined organic phases were washed with brine solution, dried over anhydrous Na_2SO_4 and concentrated *in vacuo*, the crude residue was further purified by column chromatography using ethyl acetate and *n*-hexane as mobile phase to afford 175 mg (70%) of the compound **S15**.

1-*O*-(((1-ethynylcyclohexyl)oxy)carbonyl)-2-O-acetyl-3-*O*-benzyl-4-*O*-(2-*deoxy*-2-azido-3-*O*-(4-methoxybenzyl)-6-*O*-benzoyl-4-*O*-(2-*O*-benzoyl-3-*O*-benzyl-5*S*-5-methoxycarbonyl-4-*O*-(2-*deoxy*-2-azido-3,4-di-*O*-benzyl-6-*O*-ᵗbutyldiphenylsilyl-α-D-glucopyranosyl)-β-D-xylopyranosyl)-α-D-glucopyranosyl)-5*R*-5-(methoxycarbonyl)-D-xylopyranose (49): To a solution of compound **S15** (148 mg, 0.094 mmol) in anhydrous CH_2Cl_2 (2 mL) was added 3.0 eq of DMAP followed by the portion-wise addition of the ethynyl cyclohexyl (4-nitrophenyl) carbonate **24**

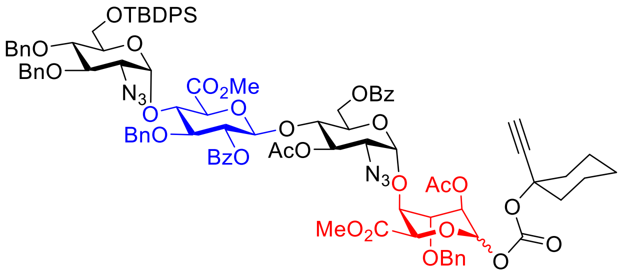

(1.2 eq) and the reaction mixture was stirred at 25 ºC for 3 h. After complete consumption of the starting hemiacetal, acetic anhydride (2.0 eq) was added and the stirring continued for

another 2 h. The reaction mixture was concentrated in vacuo and purified by silica gel column chromatography using 20% ethyl acetate in *n*-hexane as a mobile phase to obtain compound **49** (120 mg, 71%) of the desired carbonate donor. Thick syrup; $[\alpha]^{25}_{D}$ ($CHCl_3$, *c*0.8): +77.1º; IR (cm^{-1}, $CHCl_3$): 2954, 2924, 2829, 2109, 1747, 1457, 1371, 1269, 1222, 1142, 1074, 1037, 910, 750, 705;^{1}H NMR (400.31 MHz, $CDCl_3$): δ 8.10 – 8.02 (m, 2H), 8.00 – 7.94 (m, 2H), 7.68 – 7.61 (m, 4H), 7.57 (t, *J* = 7.4 Hz, 1H), 7.48 – 7.27 (m, 24H), 7.22 – 7.14 (m, 7H), 5.91 (d, *J* = 1.7 Hz, 1H), 5.38 (d, *J* = 3.6 Hz, 1H), 5.35 – 5.26 (m, 2H), 5.07 (dd, *J* = 3.4, 1.7 Hz, 1H), 4.90 – 4.80 (m, 3H), 4.79 – 4.68 (m, 4H), 4.65 (d, *J* = 10.8 Hz, 2H), 4.60 (dd, *J* = 12.6, 2.6 Hz, 2H), 4.42 – 4.30 (m, 2H), 4.16 – 4.06 (m, 2H), 3.94 (dq, *J* = 6.1, 3.7 Hz, 2H), 3.90 – 3.82 (m, 5H), 3.77 (dd, *J* = 19.0, 9.5 Hz, 2H), 3.59 (s, 3H), 3.51 (s, 3H), 3.36 – 3.24 (m, 3H), 2.65 (s, 1H), 2.23 – 2.09 (m, 5H), 2.01 (s, 3H), 1.93 – 1.83 (m, 2H), 1.75 – 1.63 (m, 3H), 1.58 – 1.49 (m, 2H), 1.39 – 1.31 (m, 1H), 1.04 (s, 9H); ^{13}C NMR (100.67 MHz, $CDCl_3$): δ 171.1, 169.6, 168.1, 168.0, 165.9, 164.6, 151.0, 138.4, 137.8, 137.4, 136.9, 136.0, 136.0, 135.7, 135.7, 133.7, 133.6, 133.5, 132.9, 129.9 (3C), 129.8, 129.7 (3C), 129.0, 128.8, 128.8, 128.7, 128.7, 128.6 (4C), 128.5 (4C), 128.3 (3C), 128.1 (3C), 127.9, 127.8 (7C), 127.5, 127.5, 101.2, 98.0, 97.7, 93.3, 82.6, 82.5, 80.1, 78.8, 77.8, 75.8, 75.7, 75.4, 75.1, 75.0, 74.6, 74.4, 74.3, 74.0, 73.2, 73.2, 73.1, 72.6, 70.1, 69.6, 66.4, 63.6, 62.0, 61.9, 61.7, 52.6, 52.6, 36.8, 36.8, 27.0 (3C), 25.0, 22.7, 22.7, 20.9, 20.7, 19.5; HRMS (ESI-MS): m/z calcd for $C_{97}H_{104}N_6O_{27}Si[M+Na]^+$: 1835.6616, Found: 1835.6498.

Allyl 2-*deoxy*-2-azido-3-*O*-benzyl-6-*O*-acetyl-4-*O*-(2-*O*-acetyl-3-*O*-benzyl-4-*O*-(2-*deoxy*-2-azido-3-*O*-acetyl-6-*O*-benzoyl-4-*O*-(2-*O*-benzoyl-3-*O*-benzyl-5*S*-5-methoxycarbonyl-4-*O*-(2-*deoxy*-2-azido-3,4-di-*O*-benzyl-6-O-^{t}butyldiphenylsilyl-α-D-glucopyranosyl)-β-D-xylopyranosyl)-α-D-glucopyranosyl)-5*R*-5-(methoxycarbonyl)-β-D-xylopyranosyl)-α-D-

glucopyranoside (50b): This pentasaccharide was prepared by the glycosylation of donor **49** and acceptor **6** following the above mentioned procedure for the compound **S5**. Eluent for purification: 20% ethyl acetate in *n*-hexane; Thick syrup; yield 20 mg (75%) from 5 mg; $[\alpha]^{25}_{D}$ ($CHCl_3$, *c*0.3): +206.7º; IR (cm^{-1}, $CHCl_3$): 2957, 2925, 2863, 2109, 1742, 1457, 1371, 1264, 1225, 1145, 1073, 1036, 744, 705; ^{1}H NMR (399.78 MHz, $CDCl_3$) δ 8.05 (d, *J* = 7.3 Hz, 2H), 7.95 (d, *J* = 7.3 Hz, 2H), 7.67 – 7.61 (m, 4H), 7.55 (t, *J* = 7.4 Hz, 1H), 7.49 – 7.42 (m, 3H), 7.41 – 7.37 (m, 4H), 7.37 – 7.26 (m, 18H), 7.25 – 7.24 (m, 1H), 7.21 – 7.12 (m, 10H), 5.93 (ddt, *J* = 16.5, 10.8, 5.7 Hz, 1H), 5.39 – 5.24 (m, 6H), 4.95 (dd, *J* = 18.9, 3.4 Hz, 2H), 4.90 – 4.82 (m, 4H), 4.81 – 4.74 (m, 3H), 4.71 (d, *J* = 16.0 Hz, 1H), 4.68 – 4.61 (m, 4H), 4.44 (d, *J* = 4.4 Hz, 1H), 4.41 – 4.30 (m, 3H), 4.23 – 4.16 (m, 2H), 4.11 (t, *J* = 9.0 Hz, 1H), 4.04 (dd, *J* = 12.9, 6.1 Hz, 1H), 3.97 – 3.82 (m, 12H), 3.78 (d, *J* = 9.4 Hz, 1H), 3.54 (s, 3H), 3.50 (s, 3H), 3.39 – 3.31 (m, 2H), 3.28 (dd, *J* = 9.8, 3.6 Hz, 1H), 3.13 (dd, *J* = 10.8, 3.2 Hz, 1H), 2.12 (s, 3H), 2.06 (s, 3H), 2.02 (s, 3H), 1.04 (s, 9H); ^{13}C NMR (150.97 MHz, $CDCl_3$): δ 171.0, 170.3, 169.8, 169.6, 168.0, 165.9, 164.6, 138.3, 138.0, 137.8, 137.5, 137.3, 136.0, 136.0, 135.7, 135.7, 133.8, 133.6, 133.6, 133.2, 132.9, 129.9, 129.8 (3C), 129.7, 129.7, 129.6, 128.9 (3C), 128.7 (4C), 128.6 (4C), 128.5, 128.5, 128.4, 128.4, 128.3, 128.3, 128.2, 128.1, 127.9 (5C), 127.8 (7C), 127.6 (3C), 118.5, 101.2, 98.0, 97.8, 97.7, 96.6, 82.5, 80.1, 78.4, 77.7, 76.2, 75.8, 75.8, 75.2, 75.1, 75.0, 74.9, 74.5, 74.3, 73.8, 73.3, 73.2, 72.6, 70.0, 69.8, 69.5, 69.5, 69.3, 68.8, 63.5, 63.3, 62.1, 61.8, 61.8, 61.1, 52.7, 52.4, 27.0 (3C), 21.0, 20.9, 20.8, 19.5; HRMS (ESI-MS): m/z calcd for $C_{106}H_{115}N_9O_{30}Si[M+Na]^+$: 2044.7417, Found: 2044.7214.

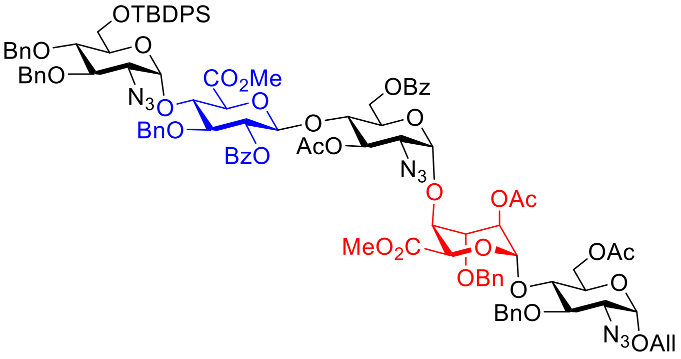

Allyl 2-*deoxy*-2-azido-3-*O*-benzyl-6-*O*-acetyl-4-*O*-(2-*O*-acetyl-3-*O*-benzyl-4-*O*-(2-*deoxy*-2-azido-6-*O*-benzoyl-4-*O*-(2-*O*-benzoyl-3-*O*-benzyl-5*S*-5-methoxycarbonyl-4-*O*-(2-*deoxy*-2-azido-3,4-di-*O*-benzyl-6-O-^{t}-butyldiphenylsilyl-α-D-glucopyranosyl)-β-D-xylopyranosyl)-α-D-glucopyranosyl)-5*R*-5-(methoxycarbonyl)-β-D-xylopyranosyl)-α-D-glucopyranoside (51): To a biphasic solution of the pentasaccharide **50a** (15 mg, 0.007 mmol) in 10:1 CH_2Cl_2:H_2O (1 mL) was added DDQ (2.5 eq) at 25 ºC and stirred for 30 min. After complete consumption of the strating material, the reaction mixture was filtered through a bed of Celite®, washed with CH_2Cl_2. The filtrate was sequentially washed with saturated aq. $Na_2S_2O_3$ and brine solution. Organic layer was dried over anhydrous Na_2SO_4 and concentrated *in vacuo*. The crude residue was purified by silica gel column chromatography using 25% ethyl acetate in *n*-hexane as mobile phase to obtain 12 mg (85%) of the compound **51**. Thick syrup; $[\alpha]^{25}_{D}$ ($CHCl_3$, *c*0.3): +69.0º; IR (cm^{-1}, $CHCl_3$): 2919, 2851, 2110, 1736, 1458, 1376, 1216, 1109, 1031, 757, 707;^{1}H NMR (600.40 MHz, $CDCl_3$): δ 8.03 (d, *J* = 7.3 Hz, 2H), 7.95 (d, *J* = 7.2 Hz, 2H), 7.63 (dd, *J* = 15.0, 6.8 Hz, 4H), 7.56 (t, *J* = 7.4 Hz, 1H), 7.52 (t, *J* = 7.4 Hz, 1H), 7.43 (d, *J* = 7.8 Hz, 2H), 7.42 – 7.38 (m, 5H), 7.36 – 7.28 (m, 17H), 7.24 (s, 1H), 7.22 – 7.15 (m, 9H), 7.09 (t, *J* = 6.8 Hz, 1H), 5.93 (ddt, *J* = 16.6, 11.3, 5.9 Hz, 1H), 5.39 – 5.33 (m, 3H), 5.28 – 5.25 (m, 1H), 5.20 (d, *J* = 3.6 Hz, 1H), 4.93 (d, *J* = 3.6 Hz, 1H), 4.88 – 4.84 (m, 4H), 4.79 (dd, *J* = 10.4, 4.1 Hz, 2H), 4.75 – 4.72 (m, 3H), 4.67 (d, *J* = 10.4 Hz, 4H), 4.55 (d, *J* = 3.7 Hz, 1H), 4.38 (d, *J* = 11.7 Hz, 1H), 4.24 (dd, *J* = 12.3, 2.9 Hz, 1H), 4.22 – 4.17 (m, 4H), 4.10 (d, *J* = 8.9 Hz, 1H), 4.05 – 4.02 (m, 2H), 3.97 (t, *J* = 8.4 Hz, 1H), 3.95 – 3.92 (m, 1H), 3.92 – 3.89 (m, 2H), 3.88 – 3.85 (m, 5H), 3.83 (d, *J* = 7.9 Hz, 1H), 3.80 – 3.76 (m, 2H), 3.55 (s, 3H), 3.54 –

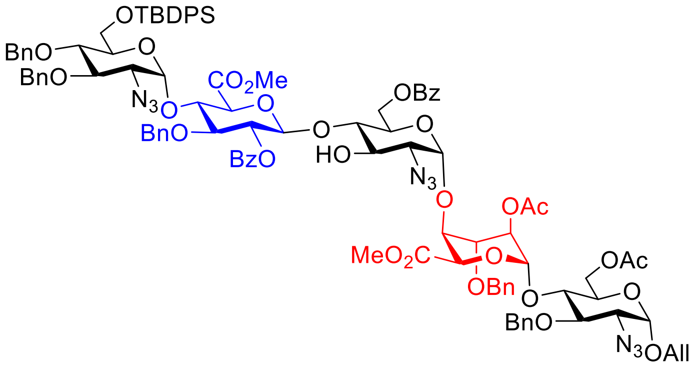

3.50 (m, 1H), 3.43 – 3.38 (m, 5H), 3.32 (dt, J = 6.4, 3.5 Hz, 1H), 3.10 (dd, J = 10.4, 3.4 Hz, 1H), 2.12 (s, 3H), 2.06 (s, 3H), 1.03 (s, 9H); ^{13}C NMR (150.97 MHz, $CDCl_3$): δ 171.0, 170.3, 169.3, 167.8, 165.9, 164.9, 138.1, 137.8, 137.6, 137.4, 137.1, 136.0, 136.0, 135.7, 135.7, 134.0, 133.5, 133.4, 133.1, 132.8, 129.9(4C), 129.7, 129.7, 129.6, 129.5, 128.9, 128.9, 128.7(4C), 128.6(6C), 128.4(2C), 128.2(3C), 128.1, 128.0(5C), 127.9(3C), 127.8(4C), 127.6(2C), 127.5, 118.6, 101.2, 98.0, 97.6, 97.3, 96.5, 81.8, 81.1, 80.0, 78.4, 77.7, 75.8, 75.8, 75.1, 74.8, 74.6, 74.4, 73.8, 73.5, 73.5, 72.9, 72.8, 72.7, 69.6, 69.4, 69.2, 68.9, 68.8, 68.5, 63.4, 63.3, 62.2, 62.0, 61.8, 61.8, 53.2, 52.2, 27.0(3C), 21.0, 20.8, 19.4; HRMS (ESI-MS): m/z calcd for $C_{104}H_{113}N_9O_{29}Si[M+Na]^+$: 2002.7311, Found: 2002.7004.

Allyl 2-*deoxy*-2-azido-3-*O*-benzyl-6-*O*-acetyl-4-*O*-(2-*O*-acetyl-3-*O*-benzyl-4-*O*-(2-*deoxy*-2-azido-3-*O*-(4-methoxybenzyl)-6-*O*-benzoyl-4-*O*-(2-*O*-benzoyl-3-*O*-benzyl-5*S*-5-methoxycarbonyl-4-*O*-(2-*deoxy*-2-azido-3,4-di-*O*-benzyl-α-D-glucopyranosyl)-β-D-xylopyranosyl)-α-D-glucopyranosyl)-5*R*-5-(methoxycarbonyl)-β-D-xylopyranosyl)-α-D-glucopyranoside (52): This compound was prepared from compound **50a** following the above procedure described for the preparation of compound **S8**. Eluent for purification: 40% ethyl acetate in *n*-hexane; Thick syrup; yield 11 mg (85%) from 15 mg;

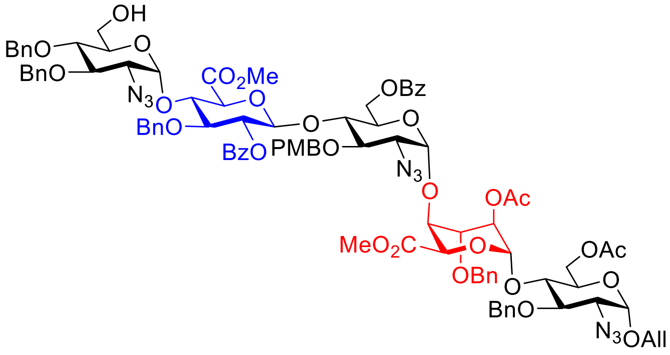

$[\alpha]^{25}_D$ ($CHCl_3$, c0.3): +63.3º; IR (cm^{-1}, $CHCl_3$): 3020, 2922, 2109, 1735, 1470, 1412, 1380, 1214, 1100, 1028, 747, 668; ^{1}H NMR (400.31 MHz, $CDCl_3$): δ 8.08 – 8.04 (m, 2H), 8.03 – 7.98 (m, 2H), 7.58 (t, J = 7.4 Hz, 1H), 7.52 (t, J = 7.4 Hz, 1H), 7.47 – 7.40 (m, 4H), 7.38 – 7.31 (m, 9H), 7.31 – 7.26 (m, 8H), 7.25 (s, 2H), 7.22 – 7.13 (m, 6H), 7.10 (dd, J = 7.1, 2.4 Hz, 2H), 6.88 (d, J = 8.7 Hz, 2H), 5.98 – 5.88 (m, 1H), 5.43 – 5.38 (m, 2H), 5.35 (dd, J = 17.2, 1.5 Hz, 1H), 5.28 – 5.24

(m, 1H), 5.18 (d, $J = 3.9$ Hz, 1H), 5.03 (d, $J = 10.2$ Hz, 1H), 4.92 (d, $J = 3.6$ Hz, 1H), 4.88 – 4.74 (m, 8H), 4.69 – 4.62 (m, 5H), 4.56 (d, $J = 10.3$ Hz, 1H), 4.50 (d, $J = 4.1$ Hz, 1H), 4.48 – 4.43 (m, 1H), 4.41 – 4.34 (m, 2H), 4.29 – 4.24 (m, 1H), 4.22 – 4.16 (m, 2H), 4.04 (dd, $J = 12.9, 6.2$ Hz, 1H), 3.98 – 3.93 (m, 1H), 3.93 – 3.89 (m, 2H), 3.87 (d, $J = 9.5$ Hz, 1H), 3.85 – 3.81 (m, 4H), 3.79 (s, 3H), 3.77 – 3.70 (m, 2H), 3.70 – 3.64 (m, 2H), 3.63 (s, 3H), 3.52 (t, $J = 9.4$ Hz, 1H), 3.41 – 3.34 (m, 2H), 3.31 – 3.24 (m, 4H), 3.22 (dd, $J = 10.3, 3.6$ Hz, 1H), 2.11 (s, 3H), 2.04 (s, 3H); ^{13}C NMR (150.99 MHz, $CDCl_3$): δ 170.8, 170.1, 169.4, 168.1, 166.1, 164.8, 159.4, 138.0, 137.9, 137.8, 137.5, 137.3, 133.9, 133.6, 133.2, 130.2(3C), 129.8, 129.8, 129.7(3C), 129.6, 129.0, 129.0, 128.8, 128.8, 128.7, 128.7, 128.6(4C), 128.5, 128.5, 128.3, 128.3, 128.2, 128.2, 128.1(3C), 128.0(4C), 127.9, 127.8(4C), 127.6, 118.5, 113.9, 113.9, 100.9, 98.0, 97.7, 97.2, 96.7, 82.5, 80.1, 78.4, 77.8, 77.3, 77.3, 76.0, 75.6, 75.5, 75.3, 75.2, 75.1, 74.9, 74.9, 73.9, 73.8, 73.6, 72.7, 72.3, 69.6, 69.6, 69.3, 69.3, 68.9, 63.5, 63.4, 62.8, 62.3, 62.1, 61.4, 55.4, 53.0, 51.9, 21.0, 20.9; HRMS (ESI-MS): m/z calcd for $C_{96}H_{103}N_9O_{30}$ $[M+Na]^+$: 1884.6709, Found: 1884.6536.

Allyl 2-*deoxy*-2-azido-3-*O*-benzyl-6-*O*-acetyl-4-*O*-(2-*O*-acetyl-3-*O*-benzyl-4-*O*-(2-*deoxy*-2-azido-6-*O*-benzoyl-4-*O*-(2-*O*-benzoyl-3-*O*-benzyl-5*S*-5-methoxycarbonyl-4-*O*-(2-*deoxy*-2-azido-3,4-di-*O*-benzyl-α-D-glucopyranosyl)-β-D-xylopyranosyl)-α-D-glucopyranosyl)-5*R*-5-(methoxycarbonyl)-β-D-xylopyranosyl)-α-D-glucopyranoside (53): This compound was prepared from compound **52** by following the above described procedure for the preparation of compound **51** [yield 5 mg (76%) from 7 mg] or alternately, this compound was prepared from compound **51** following the above procedure for compound **S8** [yield 5 mg (81%) from 7 mg]. Eluent for purification: 45% ethyl acetate in *n*-hexane; Thick syrup; $[\alpha]^{25}_D$ ($CHCl_3$, *c*0.2): +48.0º;

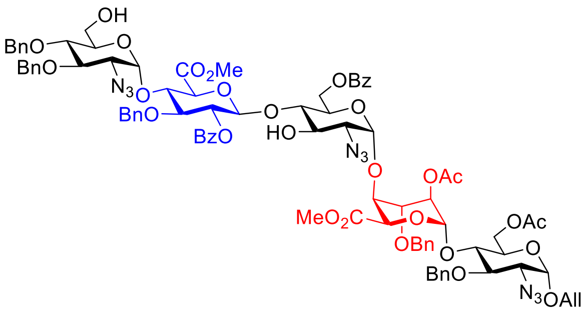

IR (cm^{-1}, $CHCl_3$): 2922, 2109, 1733, 1456, 1376, 1216, 1027, 756; ^{1}H NMR (600.40 MHz, $CDCl_3$): δ 8.04 (d, *J* = 7.8 Hz, 2H), 7.99 (d, *J* = 7.5 Hz, 2H), 7.59 (t, *J* = 7.3 Hz, 1H), 7.55 – 7.53 (m, 1H), 7.45 (t, *J* = 7.6 Hz, 2H), 7.41 (t, *J* = 7.5 Hz, 3H), 7.37 – 7.32 (m, 12H), 7.24 – 7.13 (m, 10H), 5.96 (ddt, *J* = 16.0, 10.6, 5.4 Hz, 1H), 5.45 (d, *J* = 3.2 Hz, 1H), 5.41 – 5.36 (m, 2H), 5.28 (d, *J* = 10.1 Hz, 1H), 5.24 – 5.19 (m, 1H), 4.96 – 4.94 (m, 1H), 4.92 – 4.88 (m, 2H), 4.87 – 4.82 (m, 4H), 4.77 (d, *J* = 9.4 Hz, 2H), 4.72 (d, *J* = 12.3 Hz, 2H), 4.71 – 4.69 (m, 2H), 4.67 (d, *J* = 5.8 Hz, 1H), 4.63 (d, *J* = 3.1 Hz, 1H), 4.40 (d, *J* = 12.1 Hz, 1H), 4.35 – 4.29 (m, 2H), 4.28 – 4.19 (m, 5H), 4.16 (s, 1H), 4.07 (dd, *J* = 12.6, 5.9 Hz, 1H), 4.01 – 3.96 (m, 3H), 3.92 – 3.90 (m, 1H), 3.89 – 3.86 (m, 4H), 3.83 (s, 3H), 3.82 – 3.80 (m, 1H), 3.79 (d, *J* = 9.2 Hz, 1H), 3.69 – 3.66 (m, 1H), 3.57 (t, *J* = 9.2 Hz, 2H), 3.47 (d, *J* = 10.3 Hz, 1H), 3.43 (s, 3H), 3.41 – 3.39 (m, 1H), 3.32 (dd, *J* = 10.1, 3.4 Hz, 1H), 3.15 (dd, *J* = 10.3, 2.9 Hz, 1H), 2.13 (s, 3H), 2.10 (s, 3H); ^{13}C NMR (150.99 MHz, $CDCl_3$): δ 170.9, 170.3, 169.3, 168.5, 166.0, 164.9, 138.0, 137.9, 137.8, 137.4, 137.0, 133.9, 133.5, 133.2, 129.9(3C), 129.7, 129.7, 128.9, 128.9, 128.7(4C), 128.6(6C), 128.5, 128.2(6C), 128.1(6C), 128.0, 128.0, 127.8, 127.8, 127.5, 118.5, 101.3, 98.1, 97.5, 97.2, 96.7, 82.1, 81.5, 80.0, 78.5, 77.7, 75.8, 75.6, 75.2, 74.8, 74.7, 74.2, 74.0, 73.4, 73.4, 73.1, 72.7, 72.4, 69.8, 69.4, 69.4, 68.9, 68.9, 68.6, 63.5, 63.4, 62.3, 62.1, 62.0, 61.3, 53.5, 52.2, 21.0, 20.8; HRMS (ESI-MS): m/z calcd for $C_{88}H_{95}N_9O_{29}$ $[M+Na]^+$: 1764.6133, Found: 1764.6050.

*****

Supplementary Figure S1a. ^{1}H NMR Spectrum (400.31 MHz, $CDCl_3$) of Compound S1

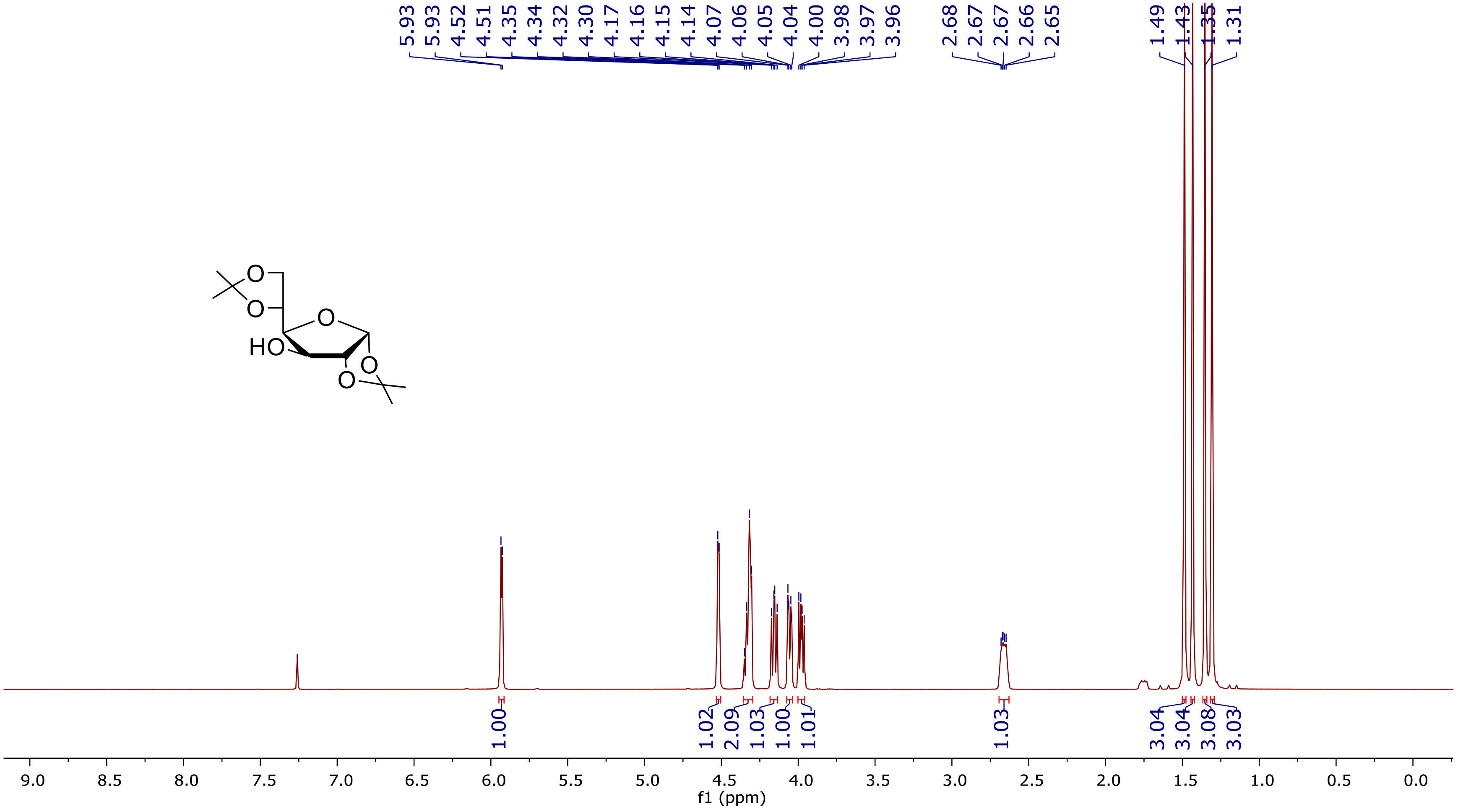

Supplementary Figure S1b.^{13}C NMR Spectrum (100.67 MHz, $CDCl_3$) of Compound S1

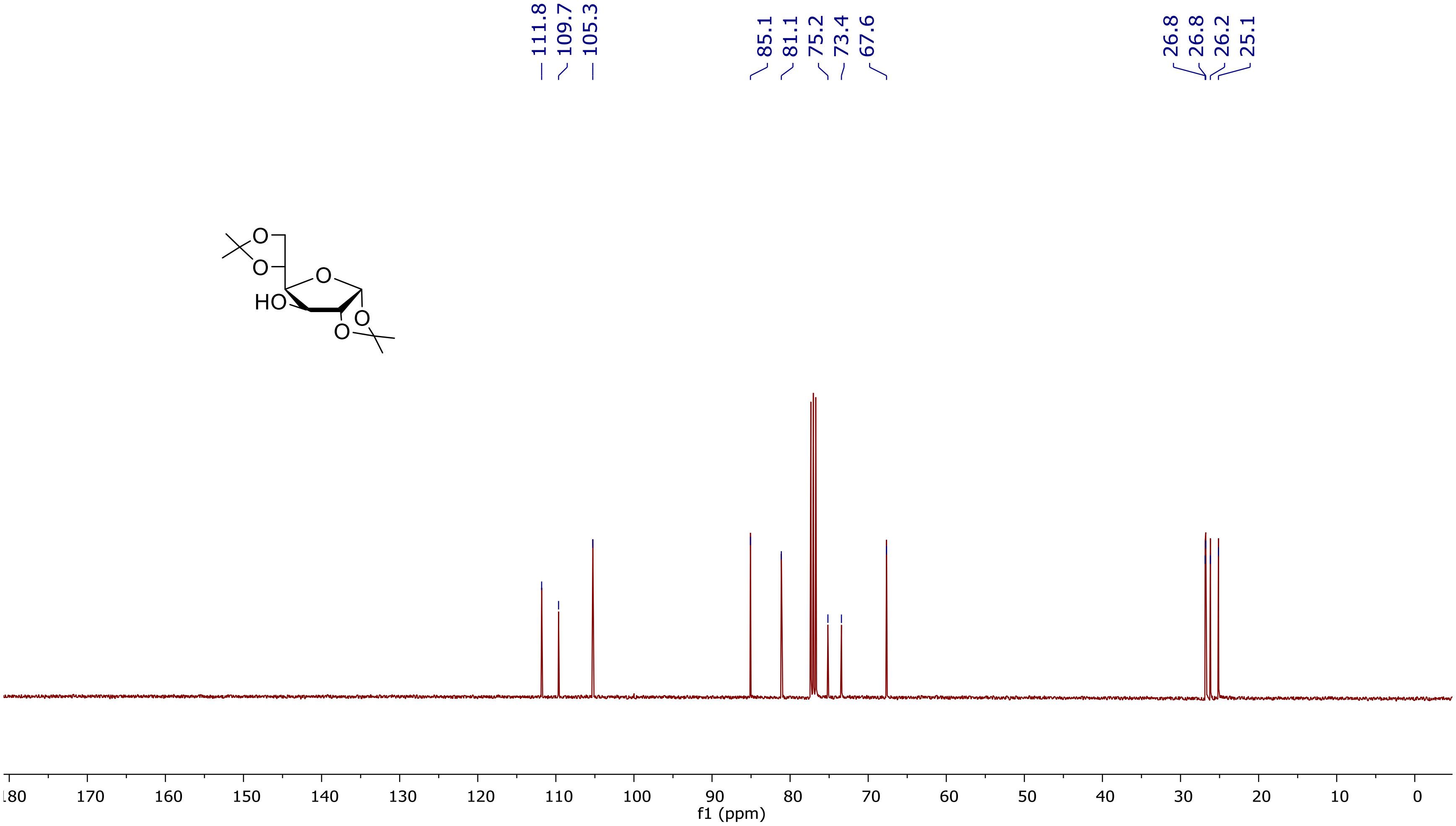

Supplementary Figure S1c. DEPT NMR Spectrum (100.67 MHz, $CDCl_3$) of Compound S1

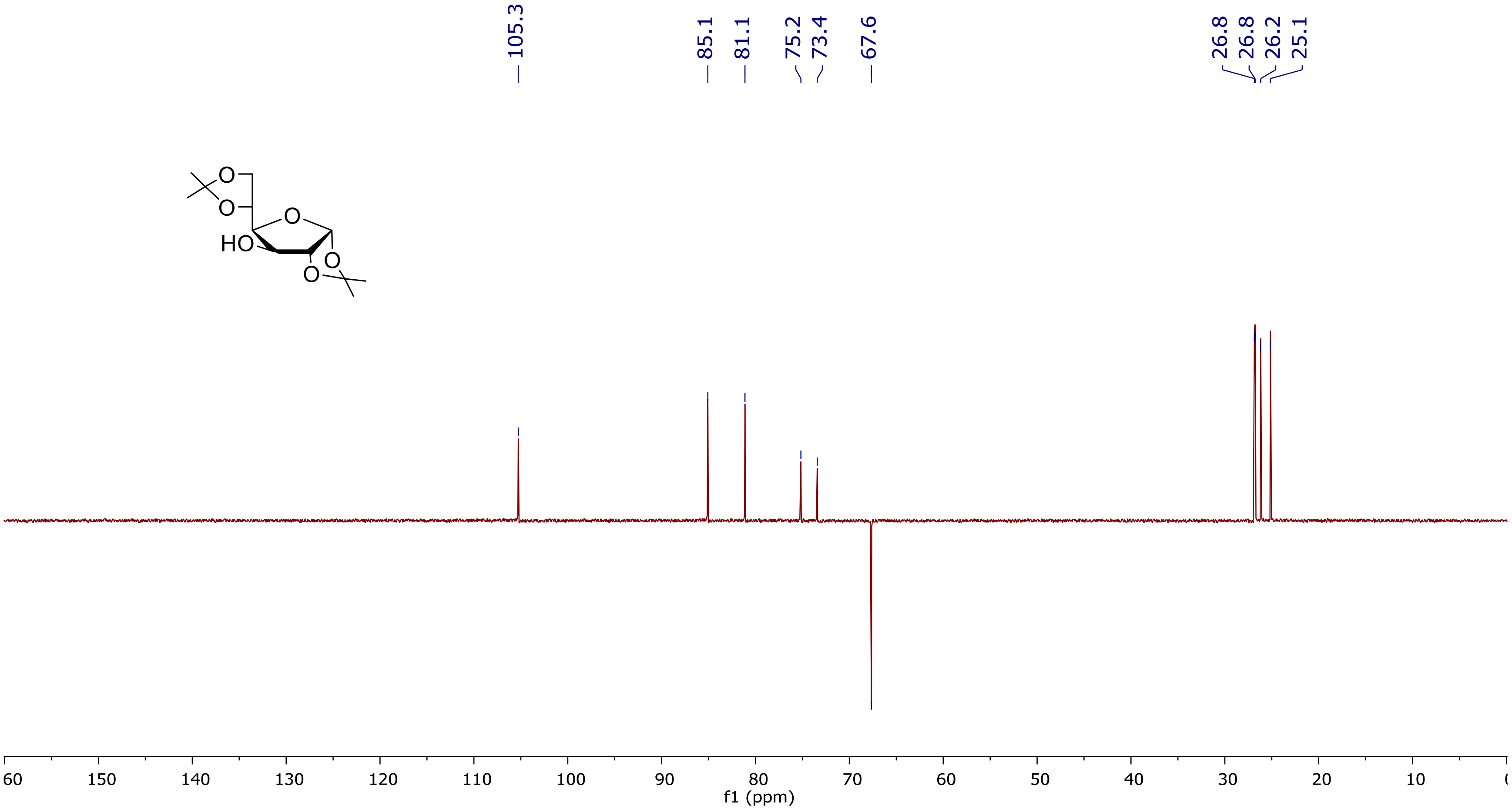

Supplementary Figure S2a.^{1}H NMR Spectrum (400.31 MHz, $CDCl_3$) of Compound 34

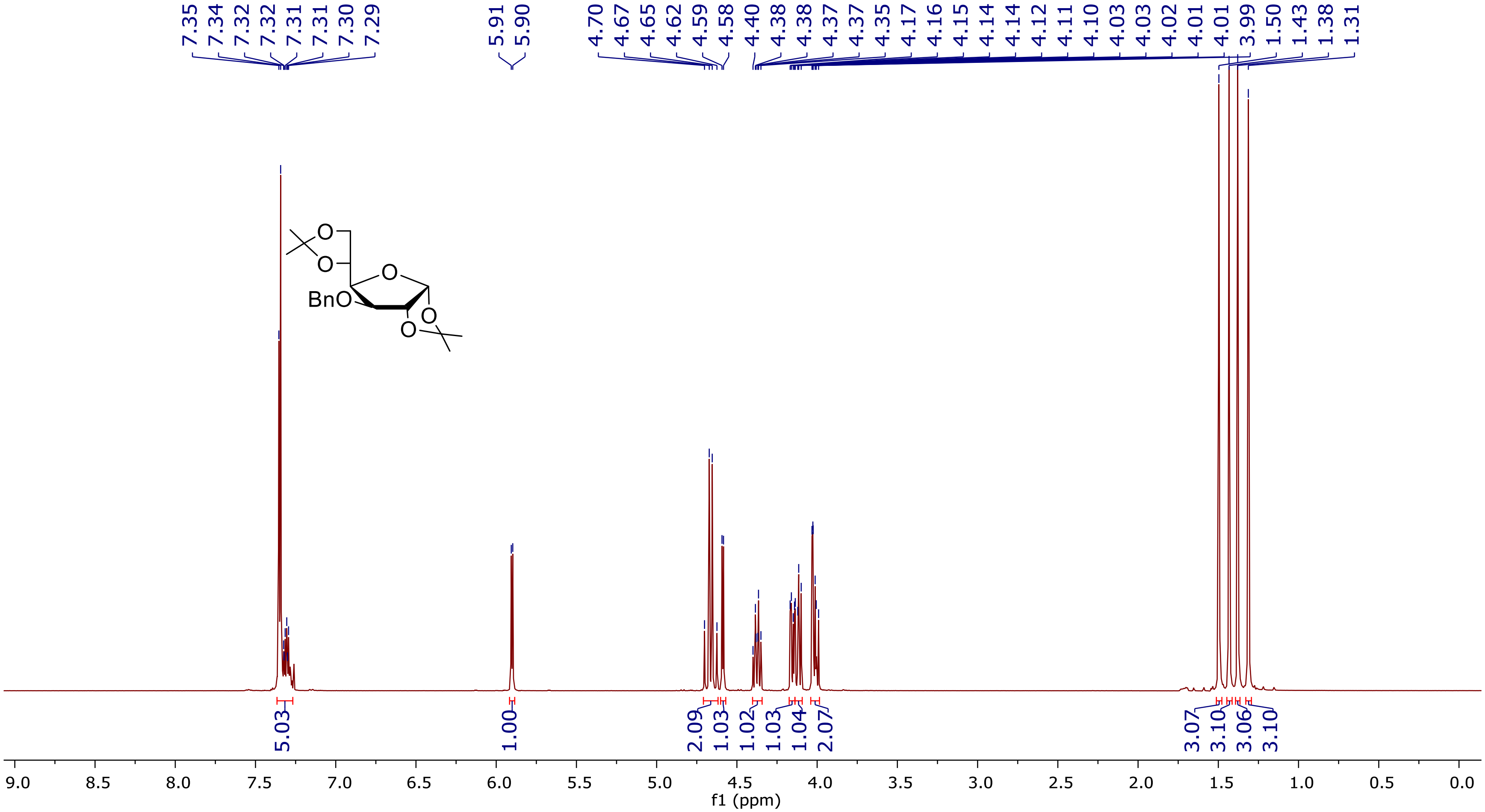

Supplementary Figure S2b.^{13}C NMR Spectrum (100.67 MHz, $CDCl_3$) of Compound 34

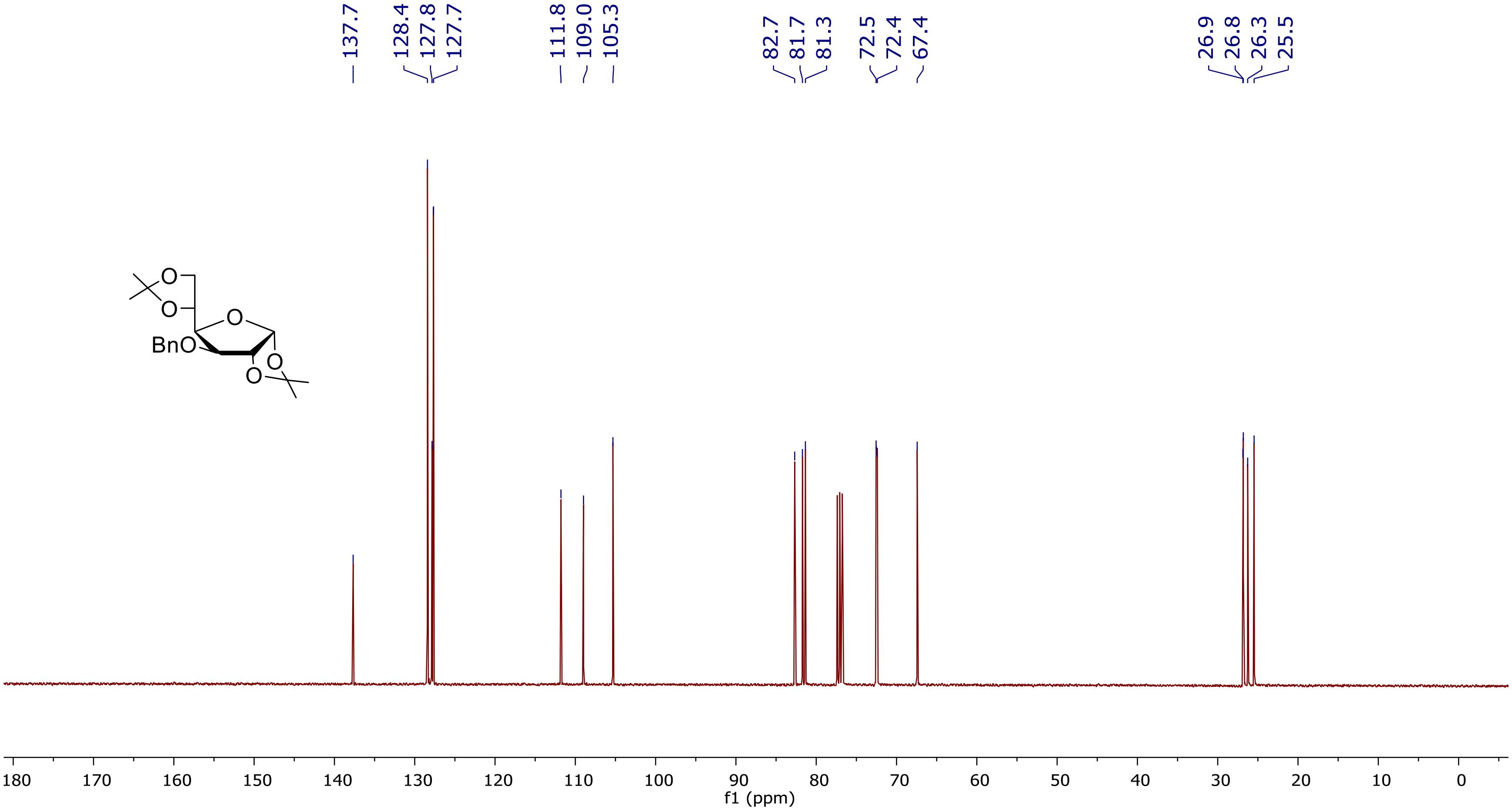

Supplementary Figure S2c. DEPT NMR Spectrum (100.67 MHz, $CDCl_3$) of Compound 34

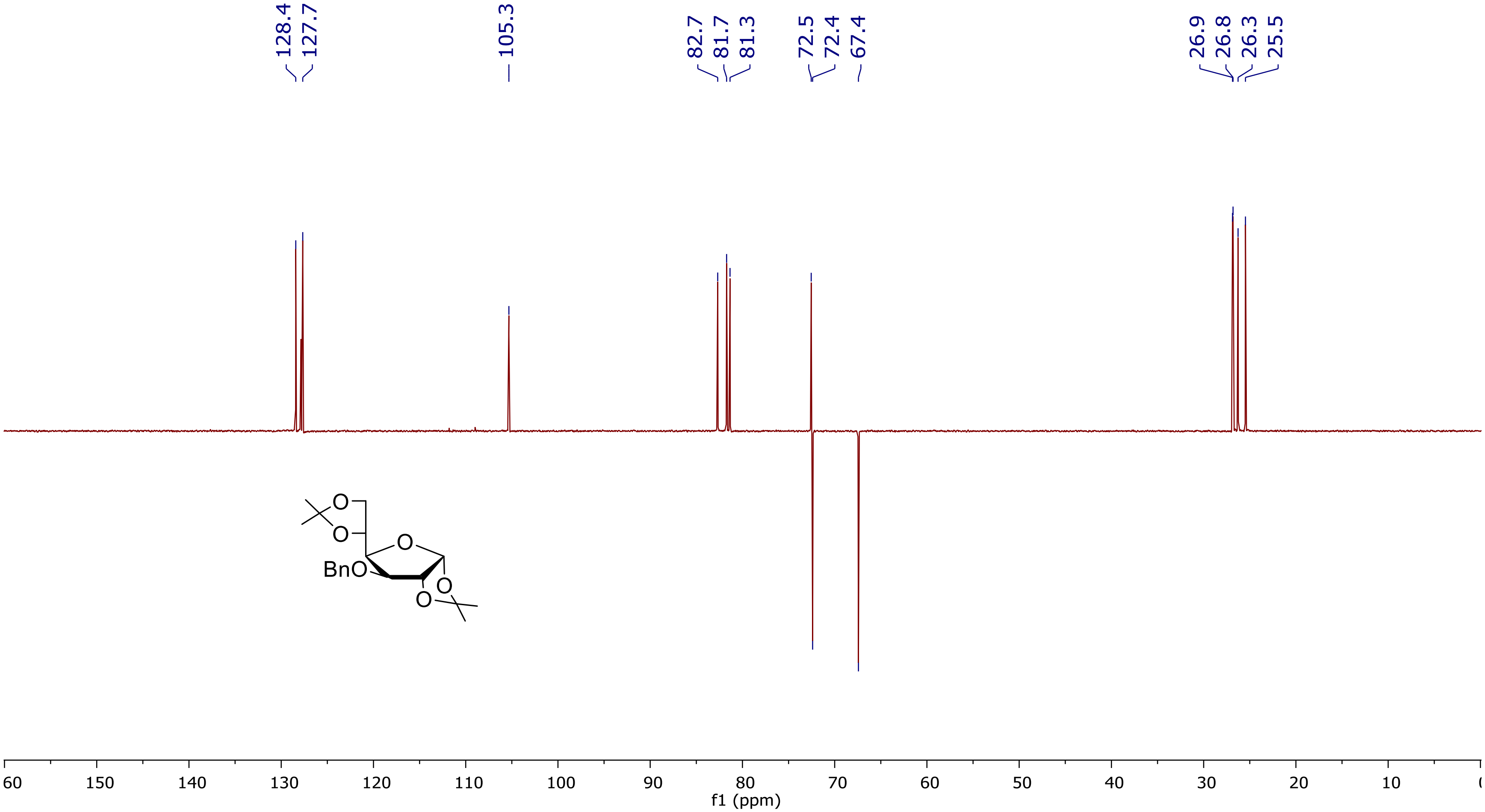

Supplementary Figure S3a. ^{1}H NMR Spectrum (399.78 MHz, CD_3OD) of Compound S2a

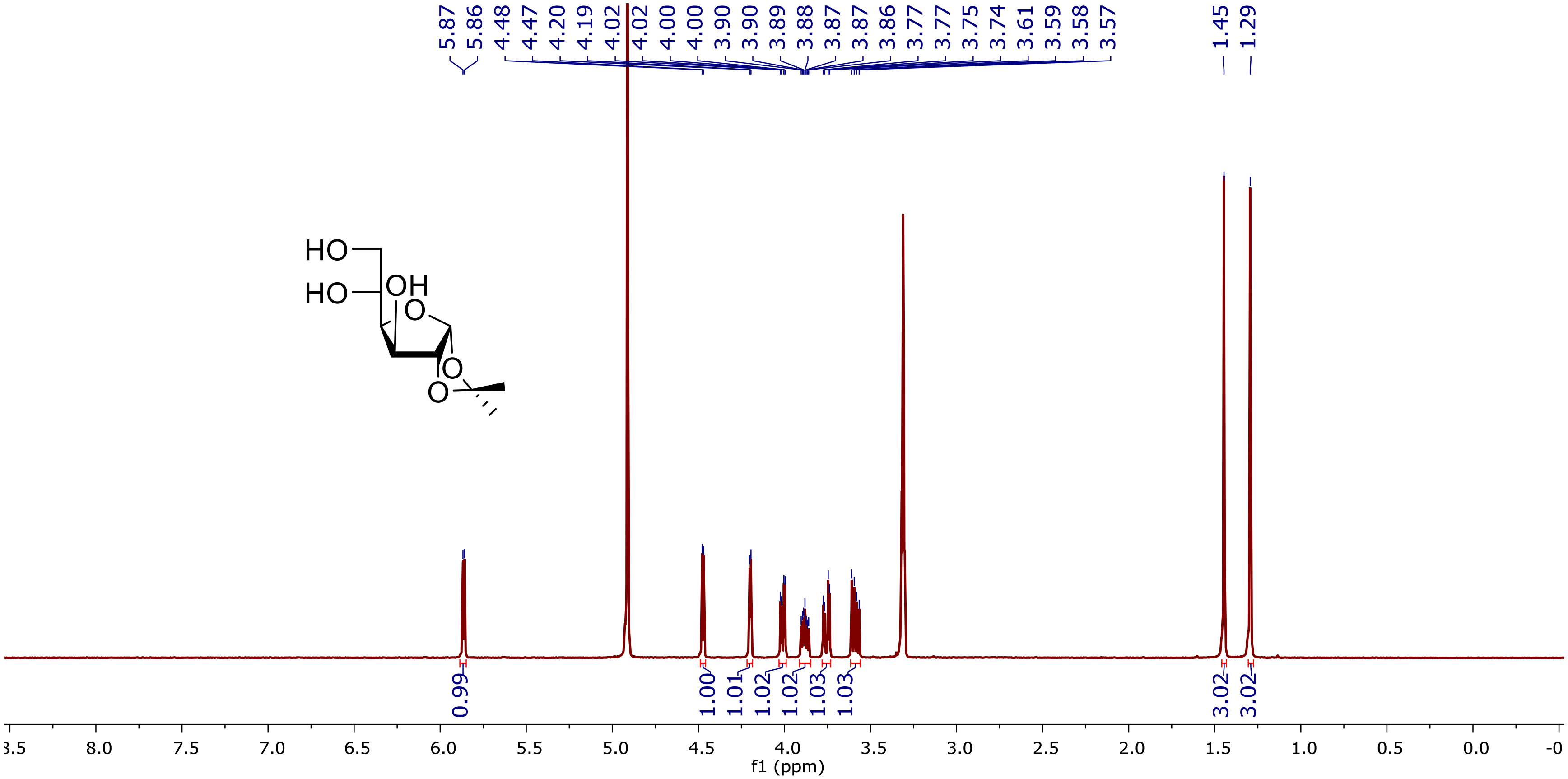

Supplementary Figure S3b. ^{13}C **NMR Spectrum (100.53 MHz, CD_3OD) of Compound S2a**

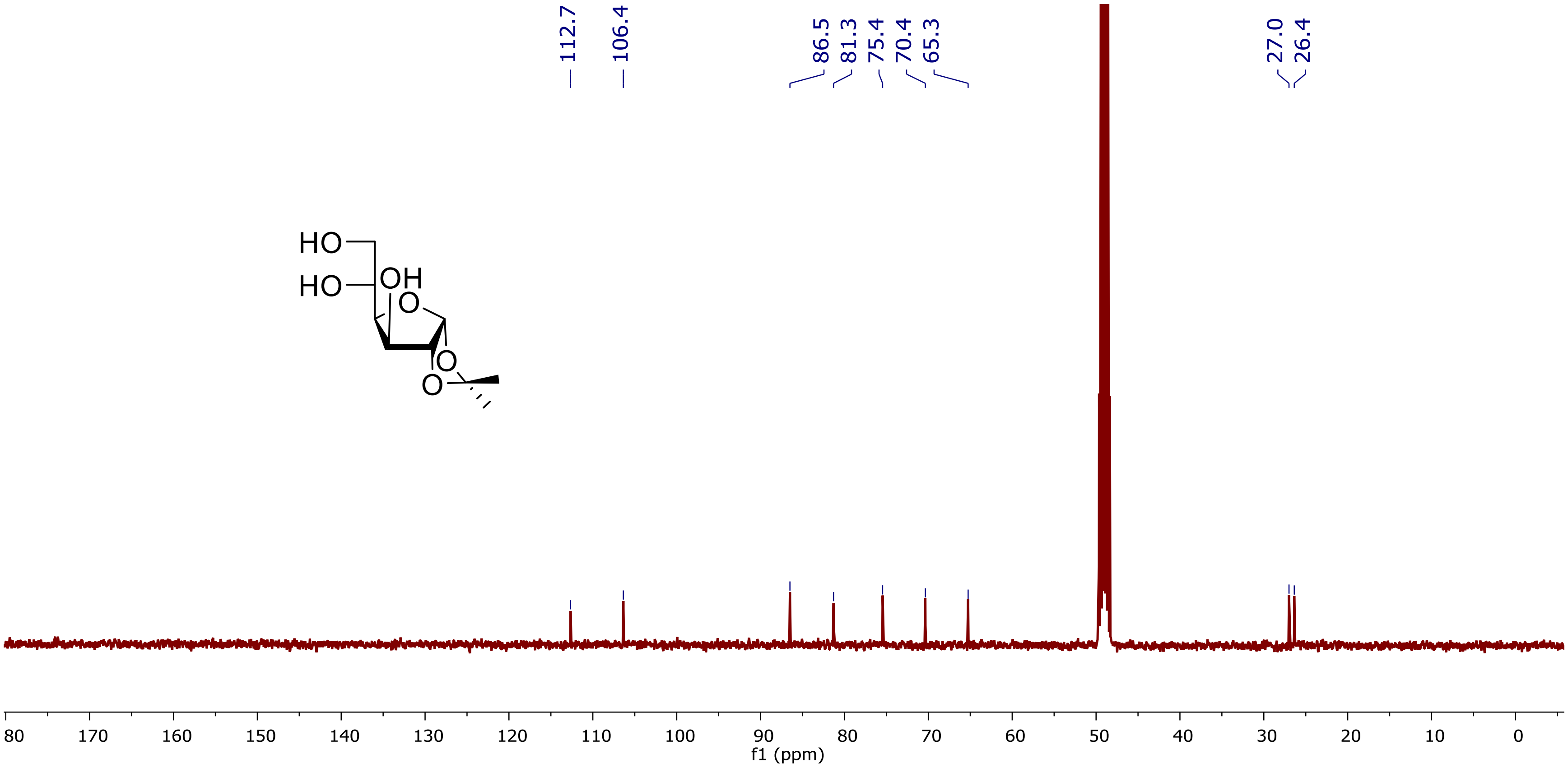

Supplementary Figure S3c. DEPT NMR Spectrum (100.53 MHz, CD_3OD) of Compound S2a

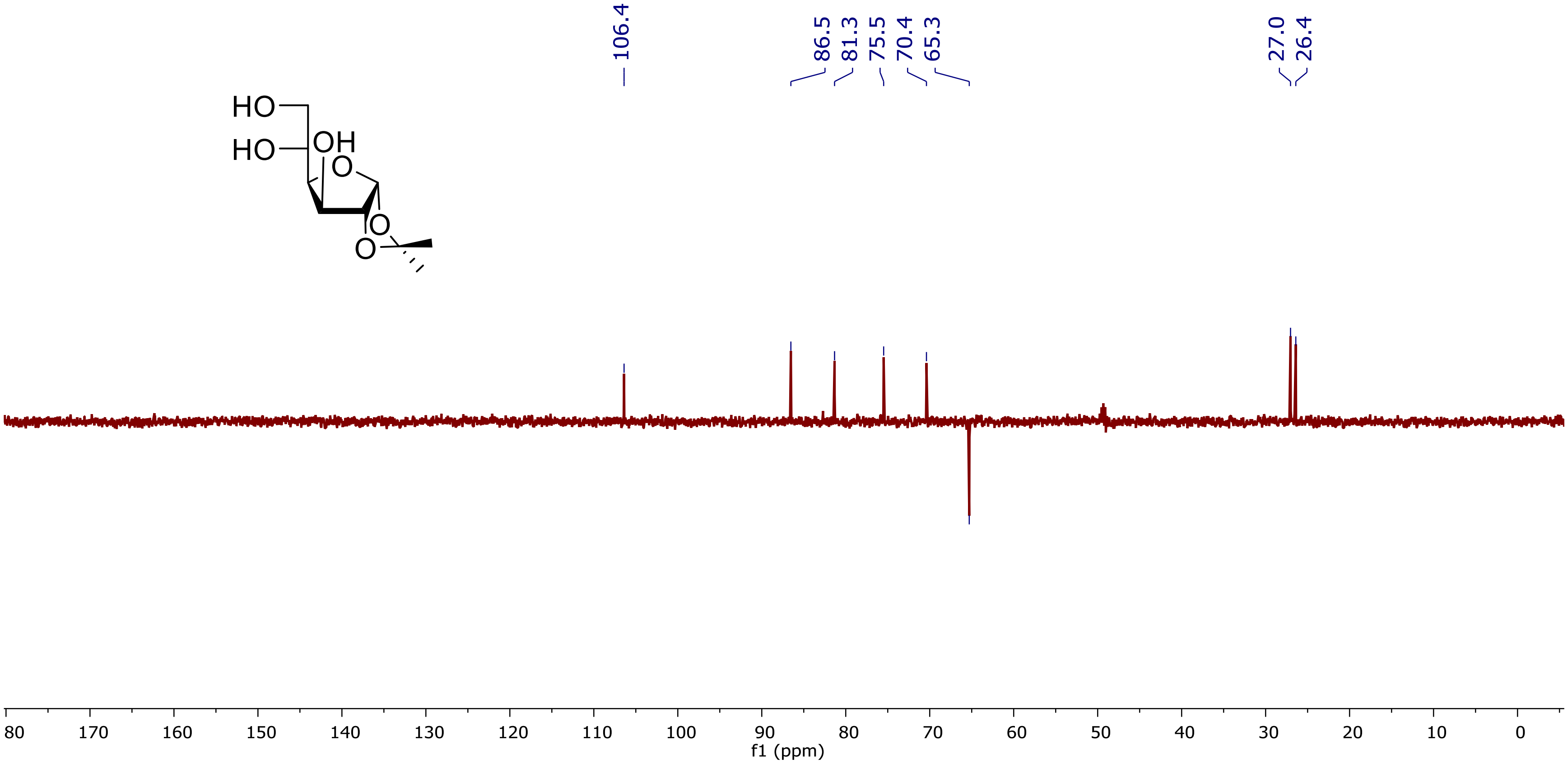

Supplementary Figure S4a.^{1}H NMR Spectrum (399.78 MHz, $CDCl_3$) of Compound S2b

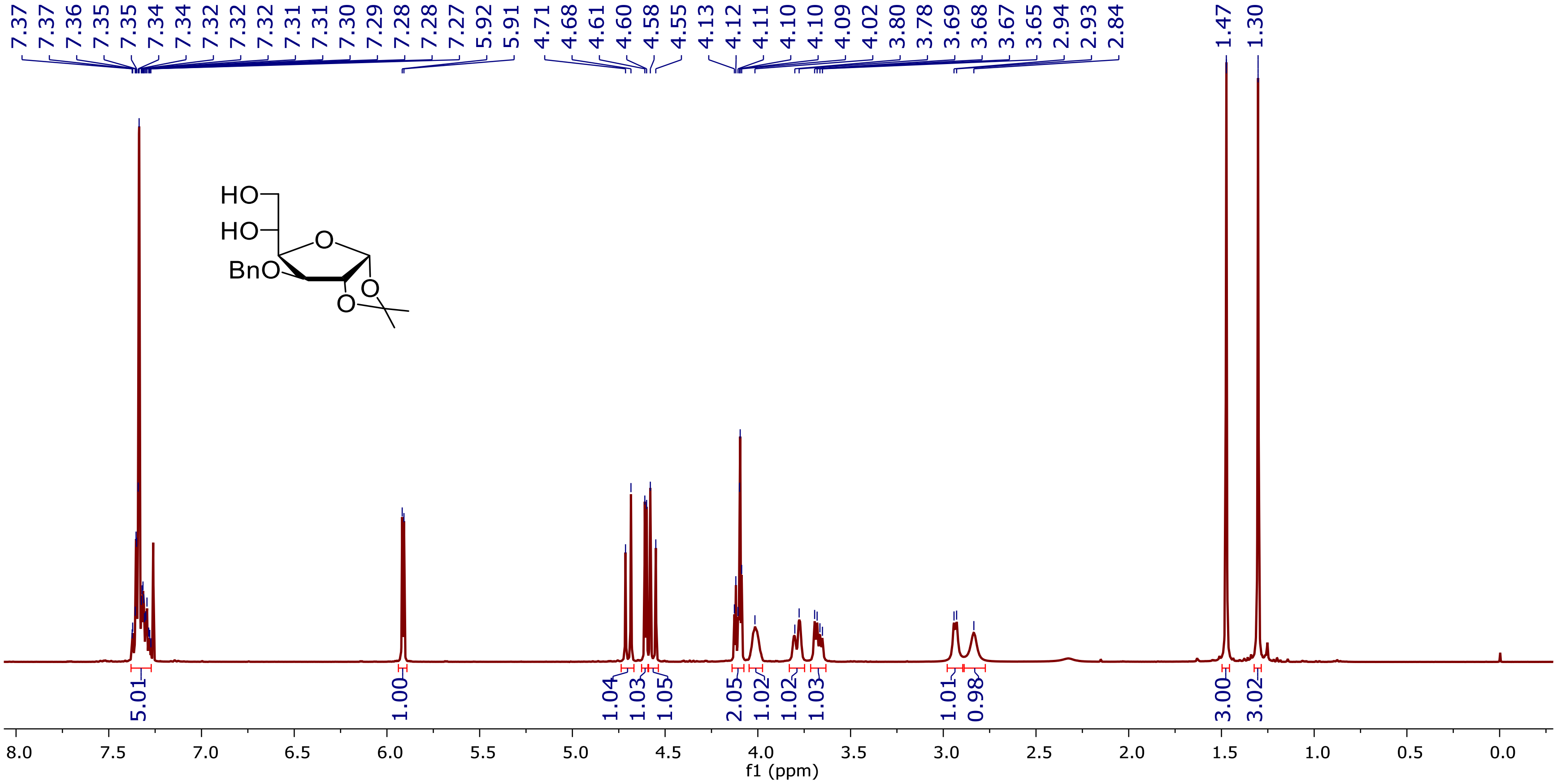

Supplementary Figure S4b.^{13}C NMR Spectrum (100.53 MHz, $CDCl_3$) of Compound S2b

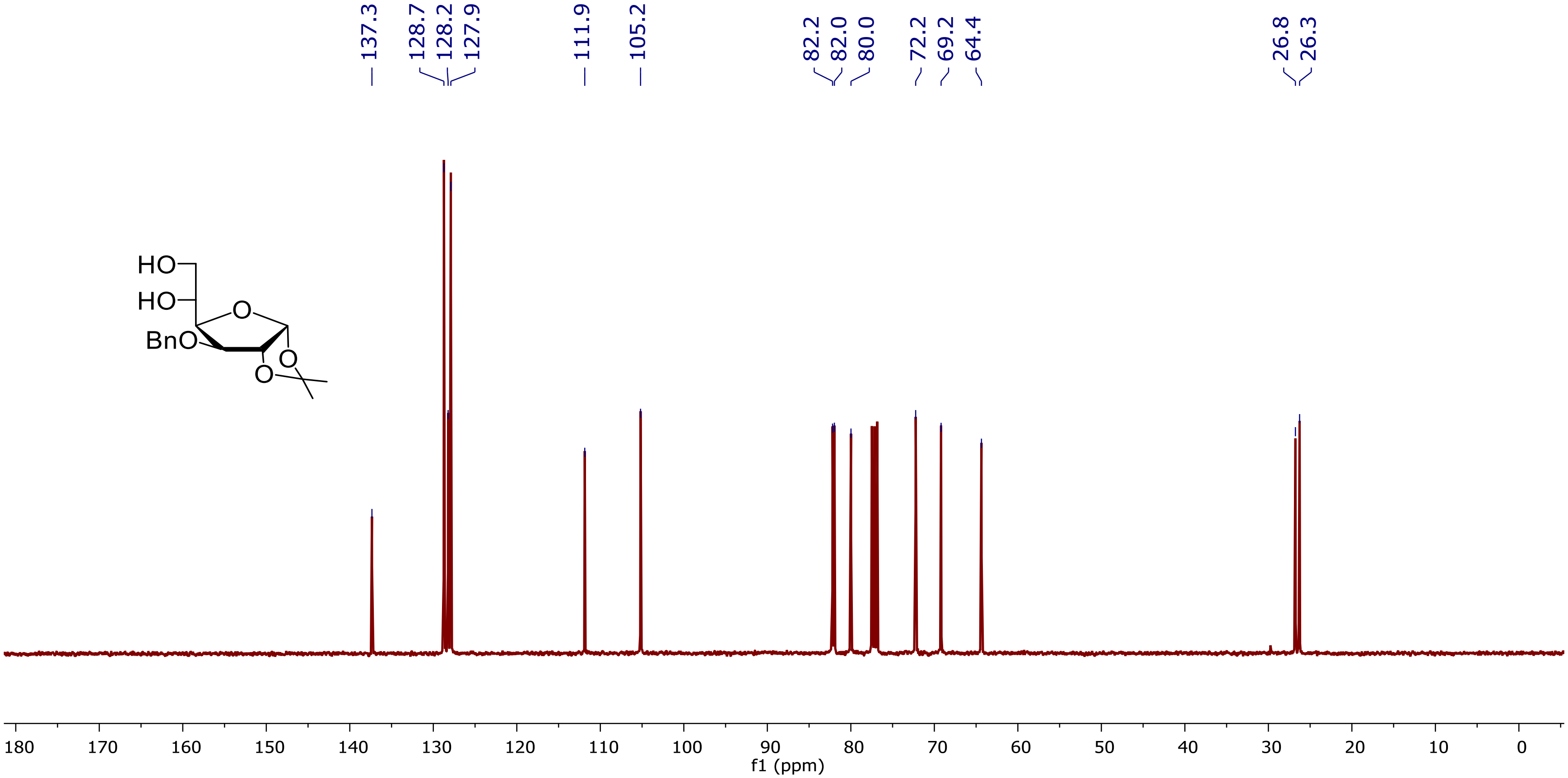

Supplementary Figure S4c.DEPT NMR Spectrum (100.53 MHz, $CDCl_3$) of Compound S2b

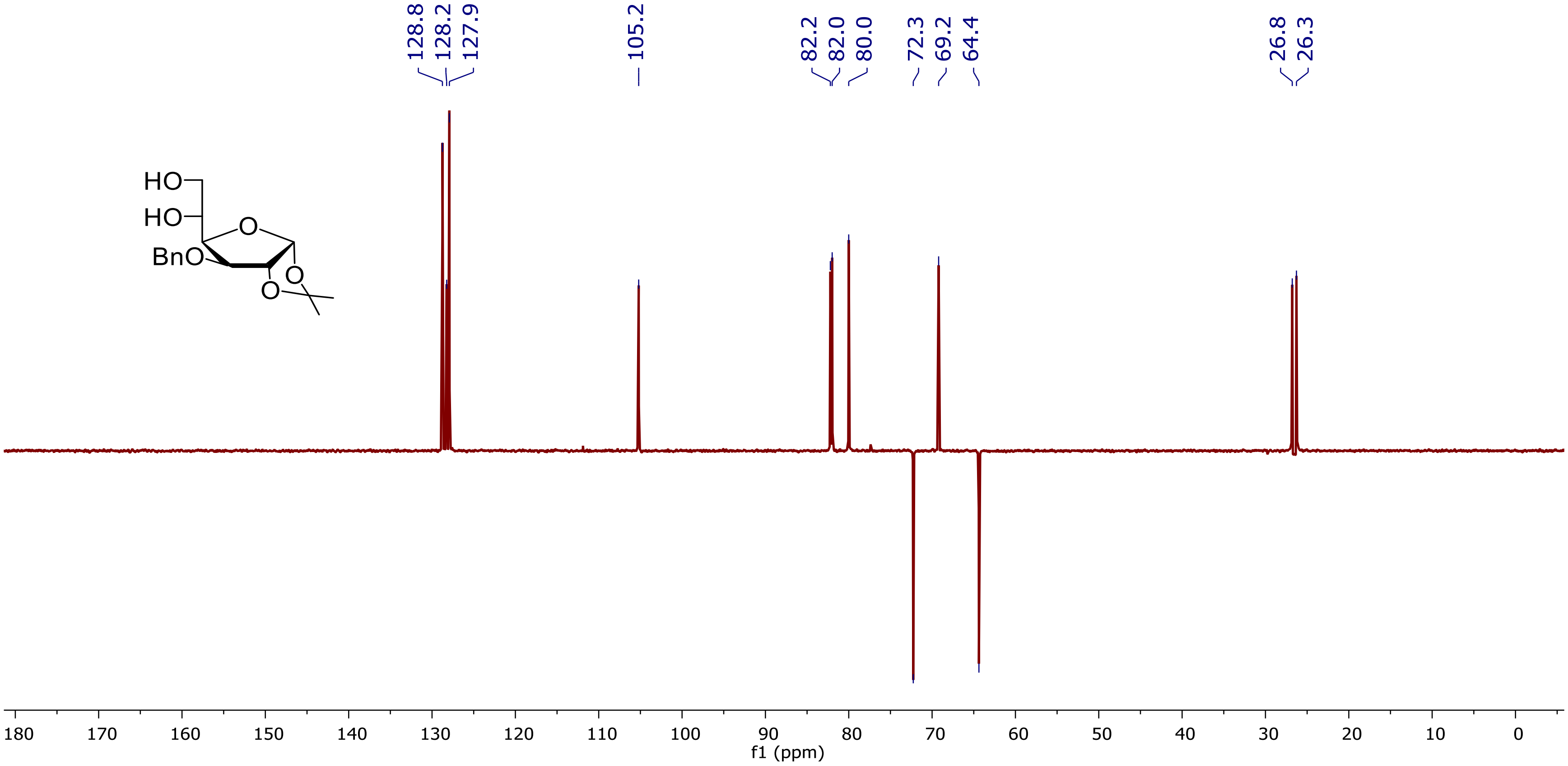

Supplementary Figure S5a. ^{1}H NMR Spectrum (400.31 MHz, $CDCl_3$) of Compound 14b

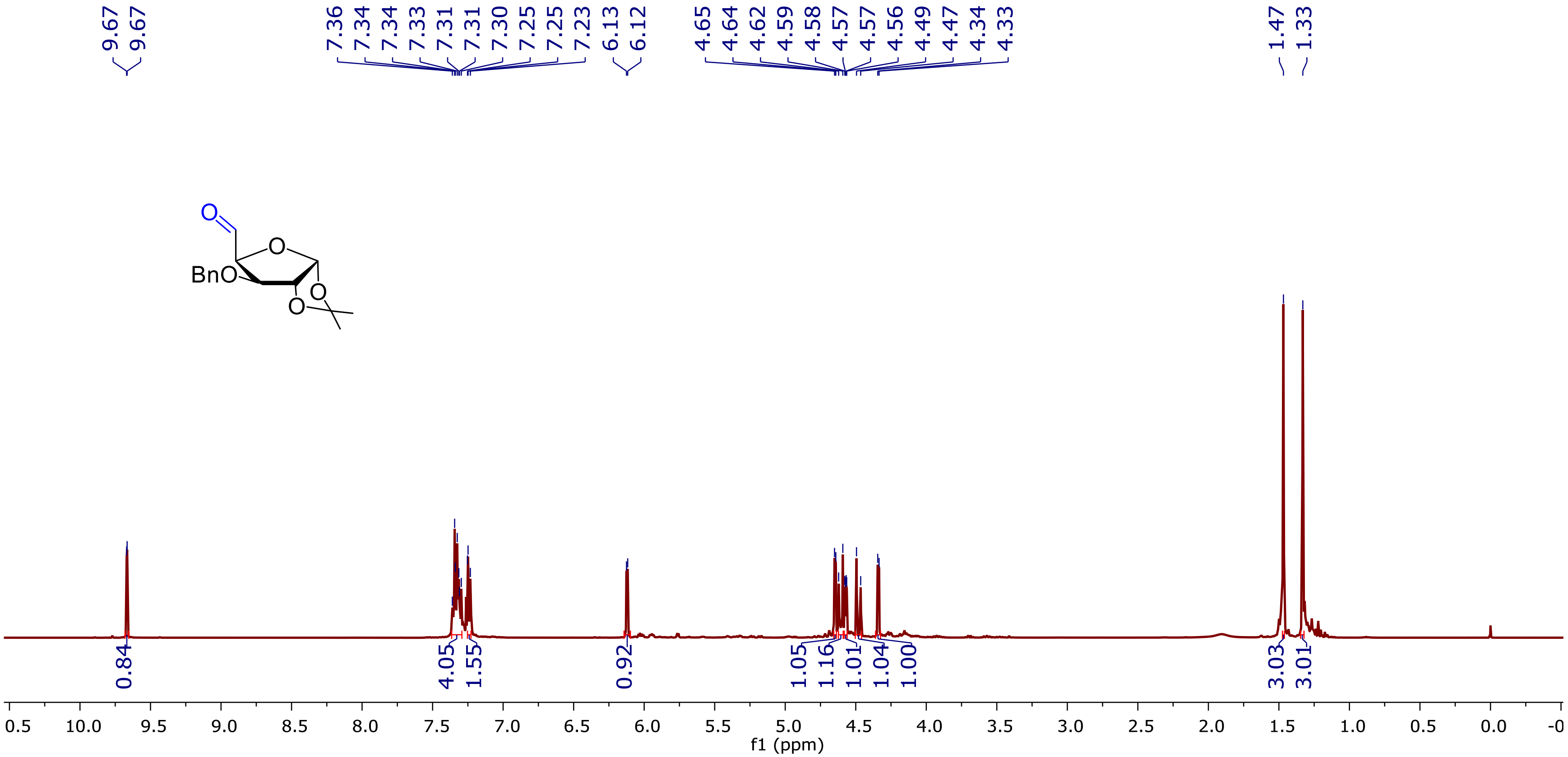

Supplementary Figure S5b. ^{13}C NMR Spectrum (100.67 MHz, $CDCl_3$) of Compound 14b

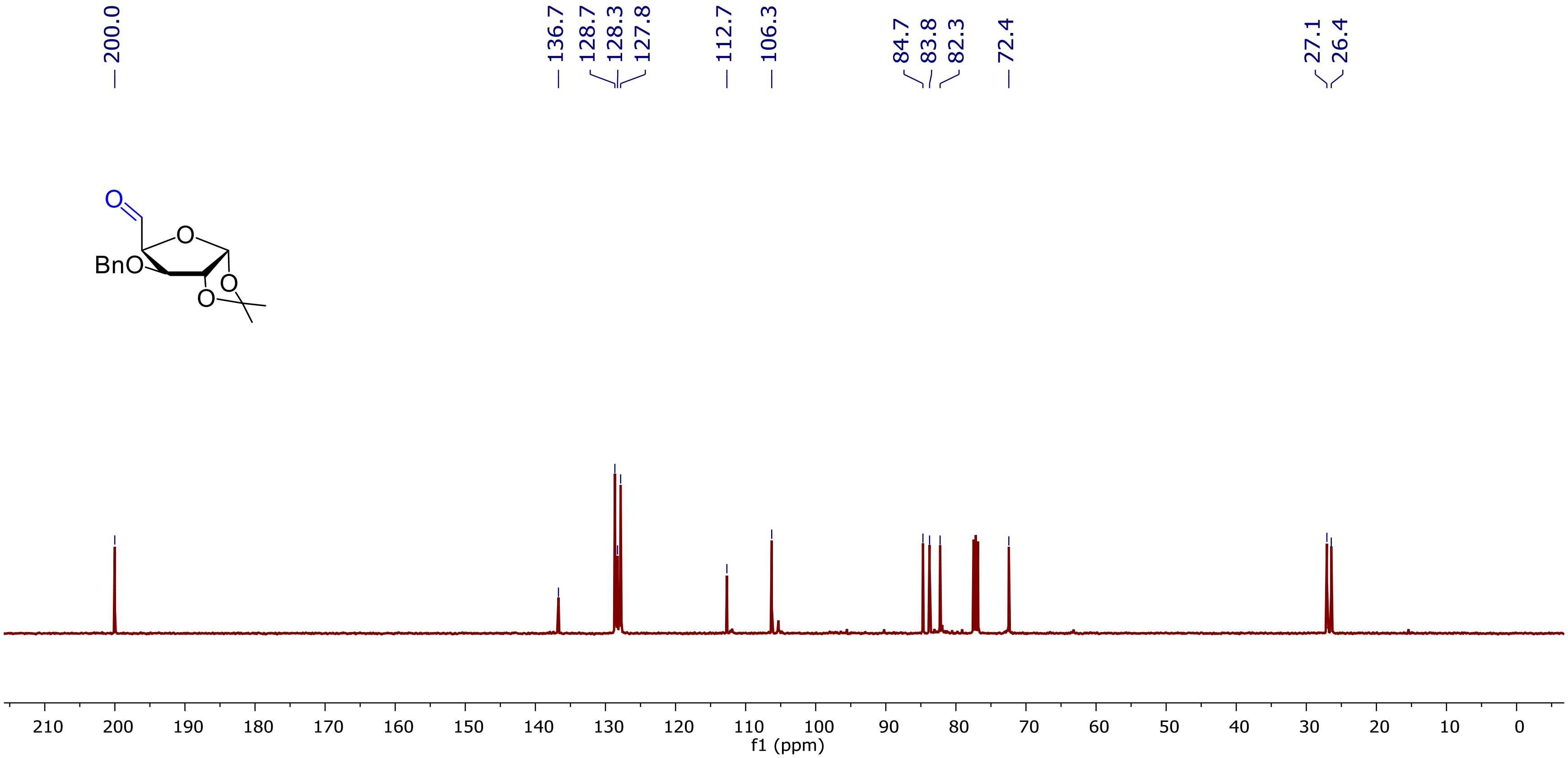

Supplementary Figure S5c. DEPT NMR Spectrum (100.67 MHz, $CDCl_3$) of Compound 14b

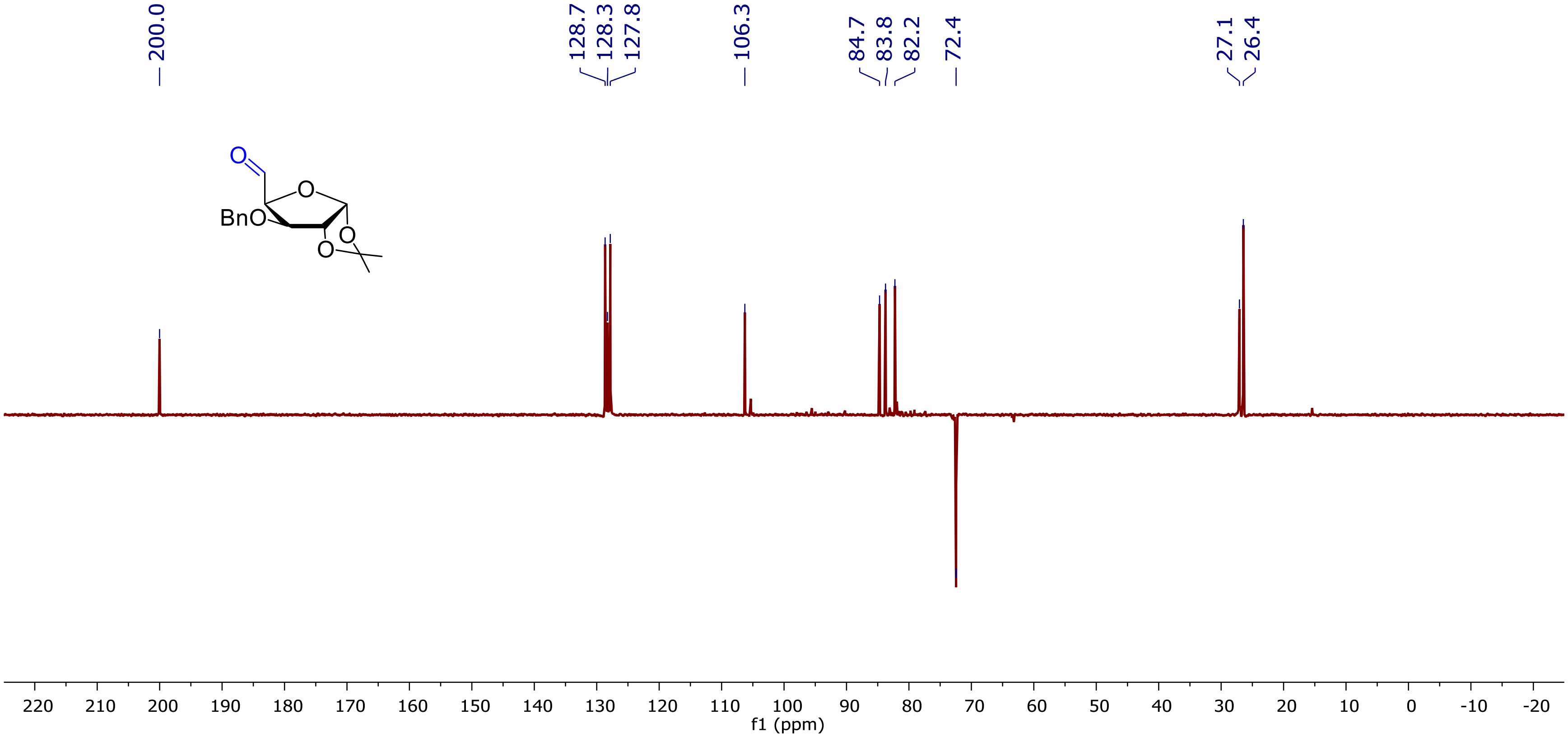

Supplementary Figure S6a. ^{1}H NMR Spectrum (400.31 MHz,$CDCl_3$) of Compound 15a

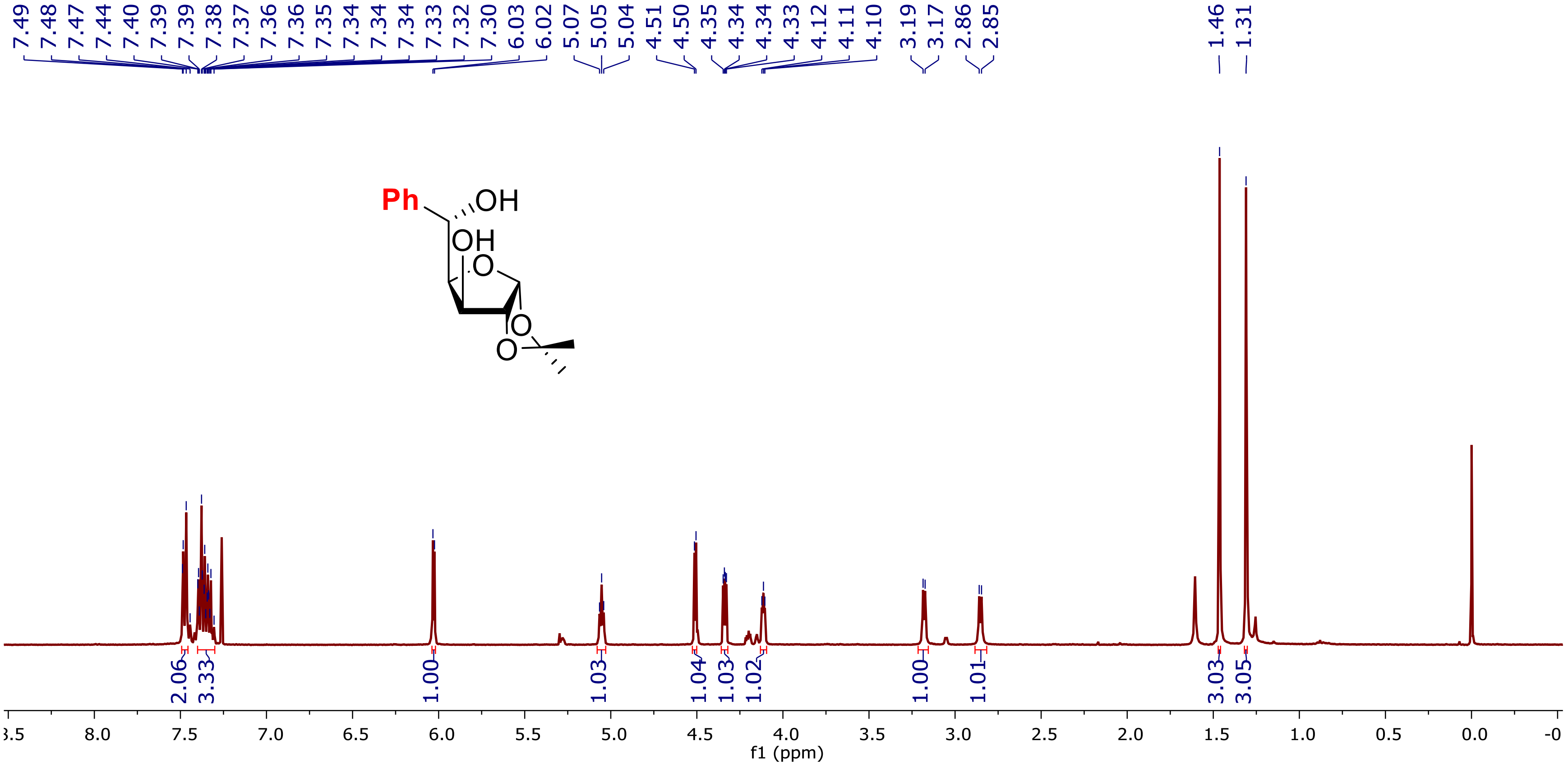

Supplementary Figure S6b. ^{13}C NMR Spectrum (100.67 MHz, $CDCl_3$) of Compound 15a

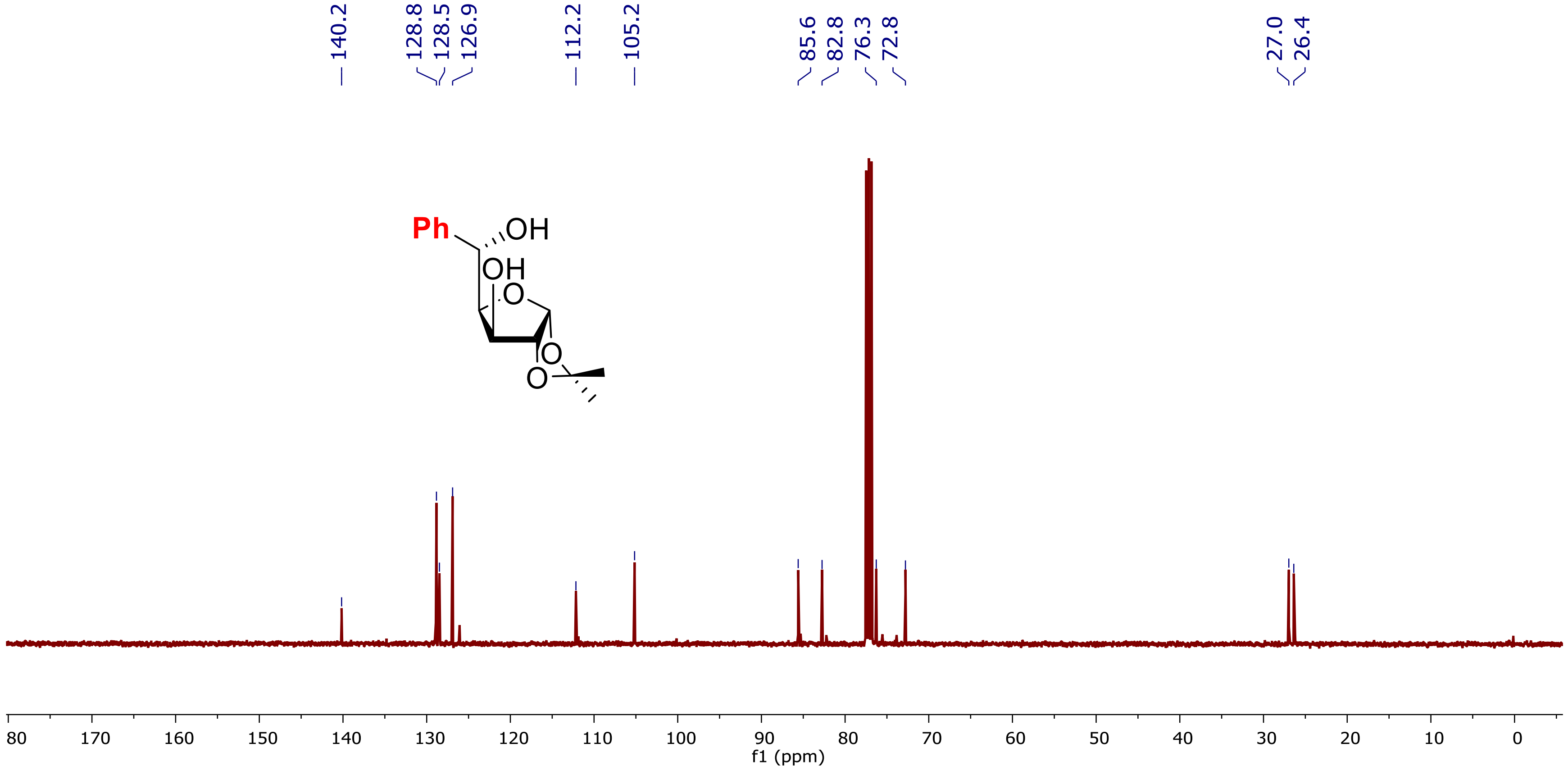

Supplementary Figure S6c. DEPT NMR Spectrum (100.67 MHz, $CDCl_3$) of Compound 15a

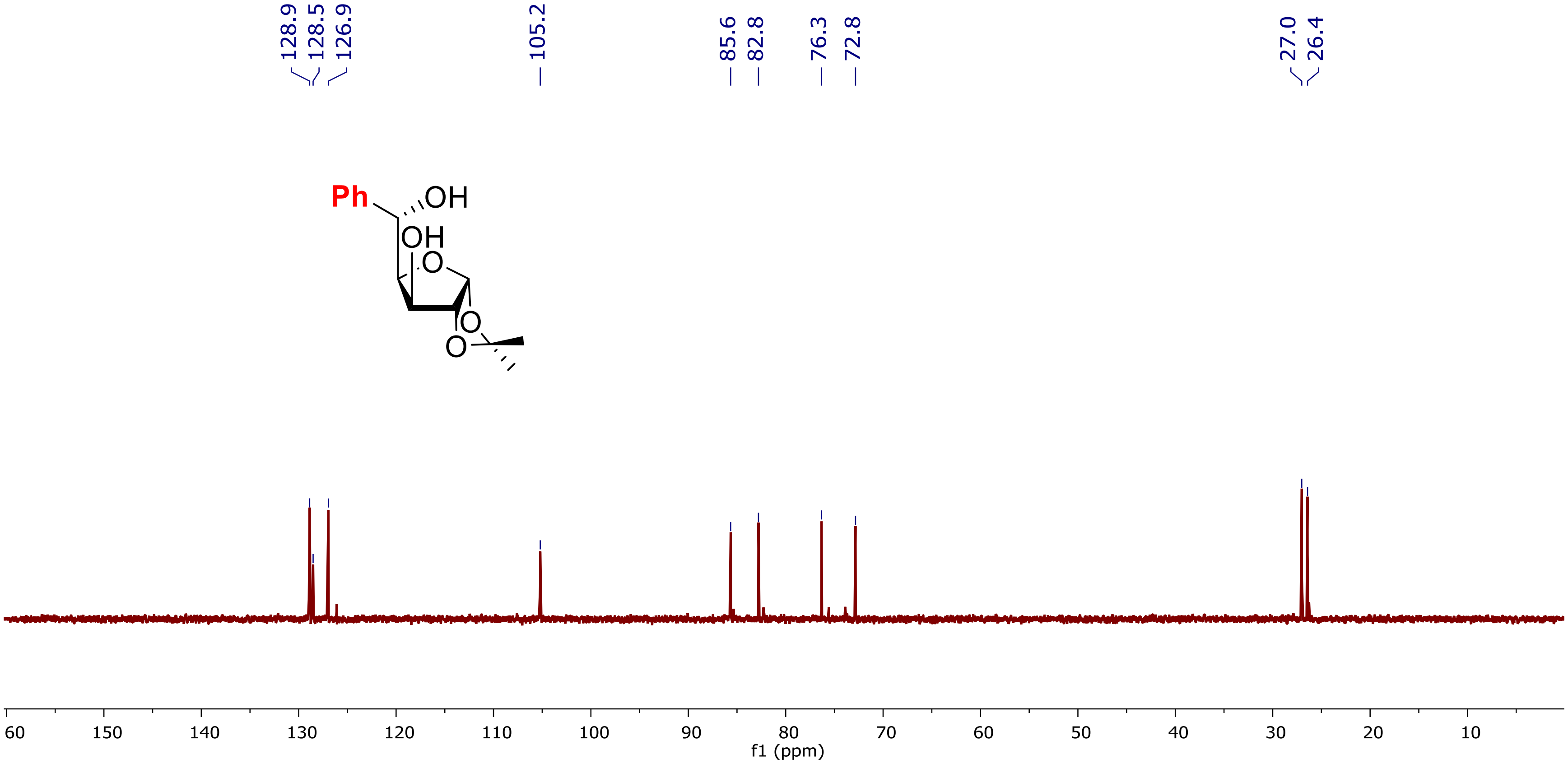

Supplementary Figure S7a. ^{1}H NMR Spectrum (400.31 MHz, $CDCl_3$) of Compound 16a

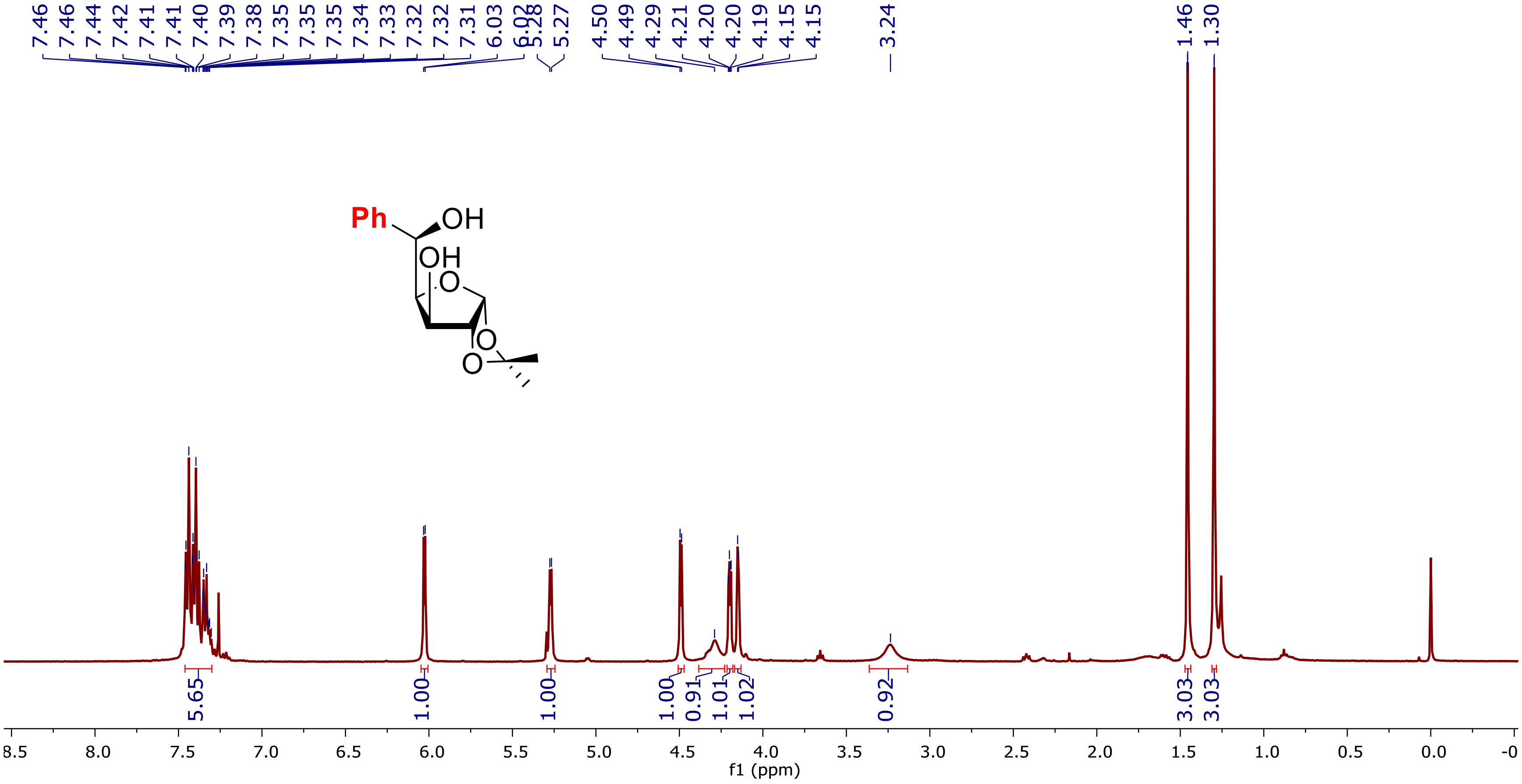

Supplementary Figure S7b. ^{13}C NMR Spectrum (100.67 MHz, $CDCl_3$) of Compound 16a

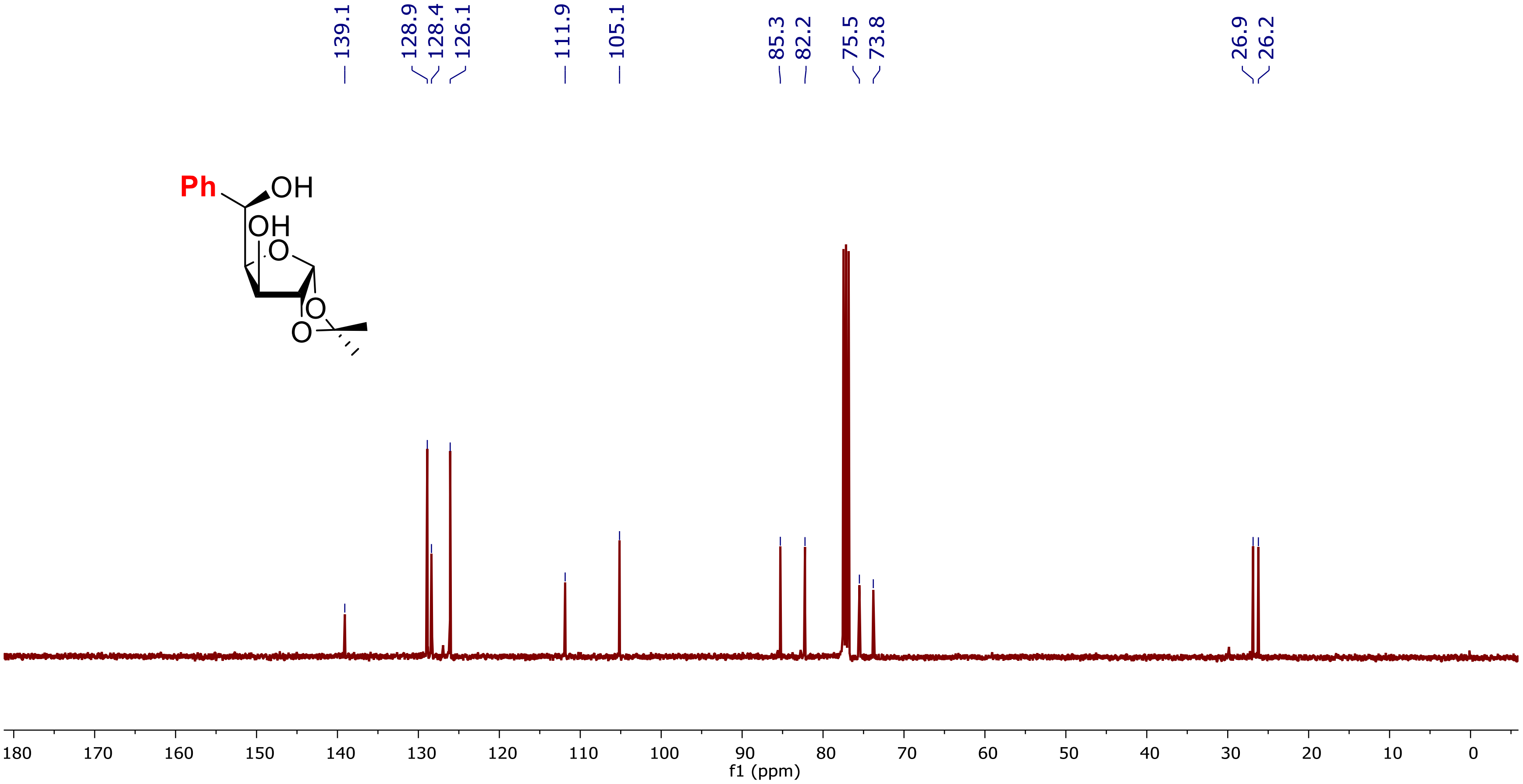

Supplementary Figure S7c. DEPT NMR Spectrum (100.67 MHz, $CDCl_3$) of Compound 16a

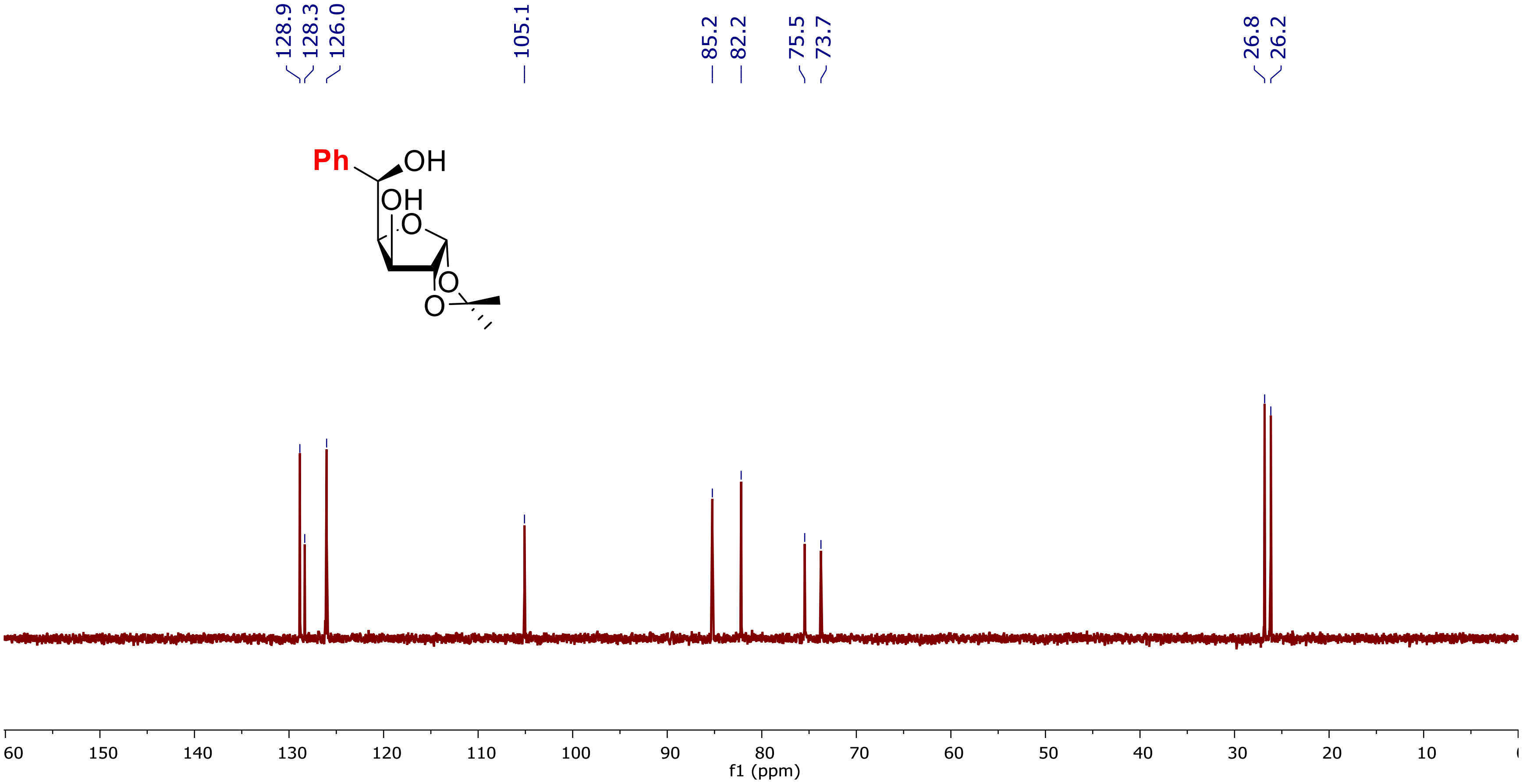

Supplementary Figure S8a. ^{1}H NMR Spectrum (399.78 MHz, $CDCl_3$) of Compound 15b

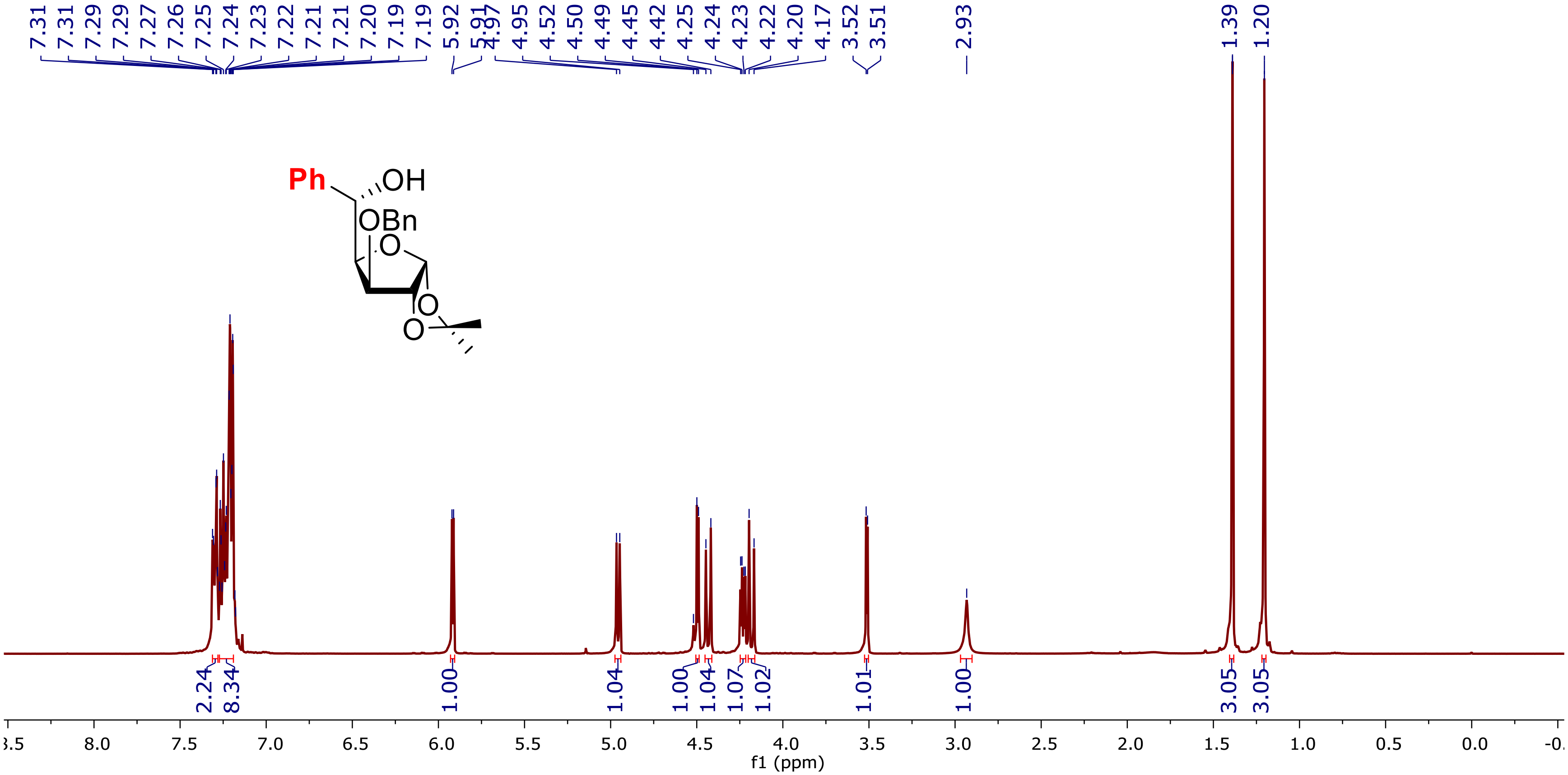

Supplementary Figure S8b. ^{13}C NMR Spectrum (100.53 MHz, $CDCl_3$) of Compound 15b

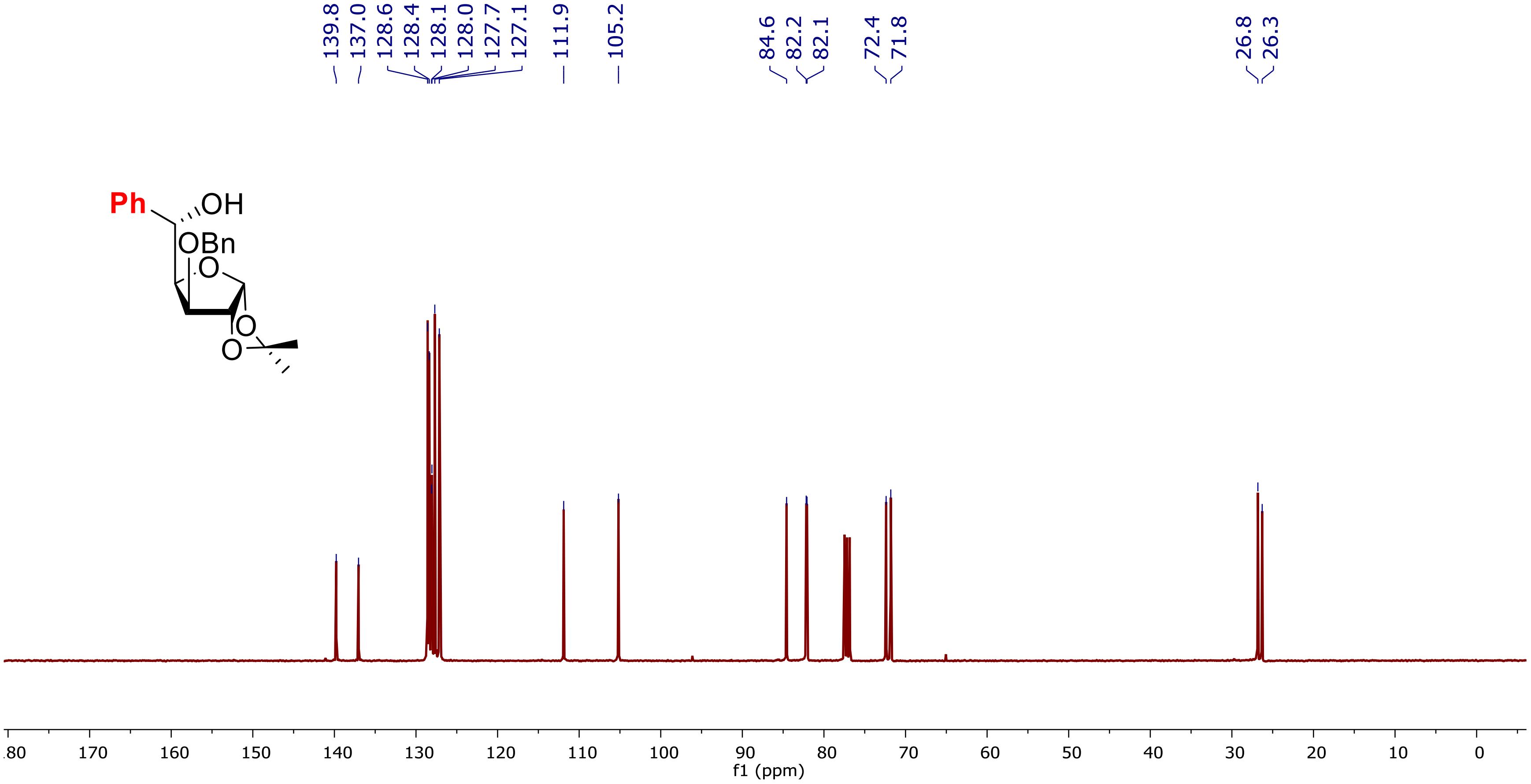

Supplementary Figure S8c. DEPT NMR Spectrum (100.53 MHz, $CDCl_3$) of Compound 15b

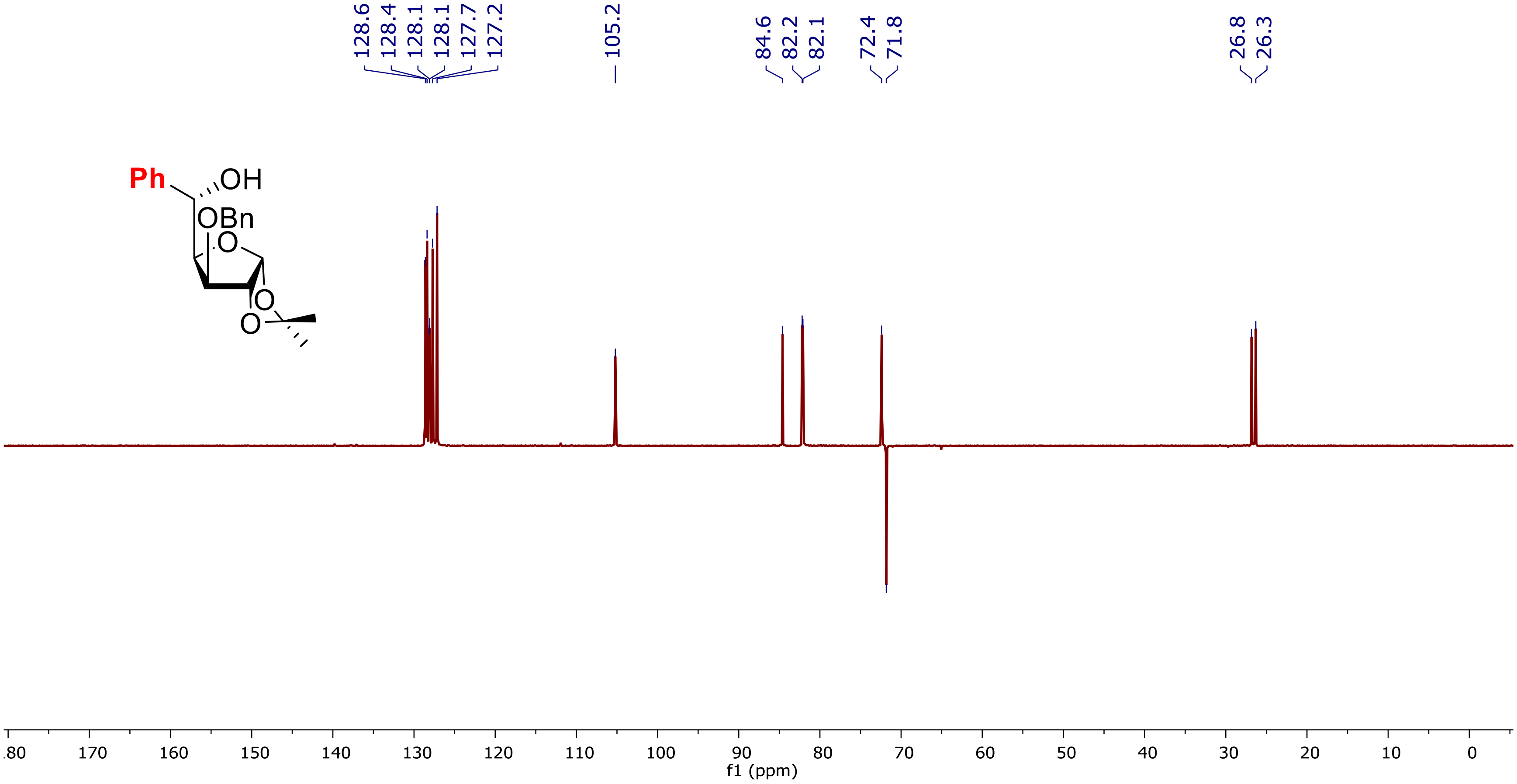

Supplementary Figure S9a. ¹H NMR Spectrum (399.78 MHz, $CDCl_3$) of Compound 16b

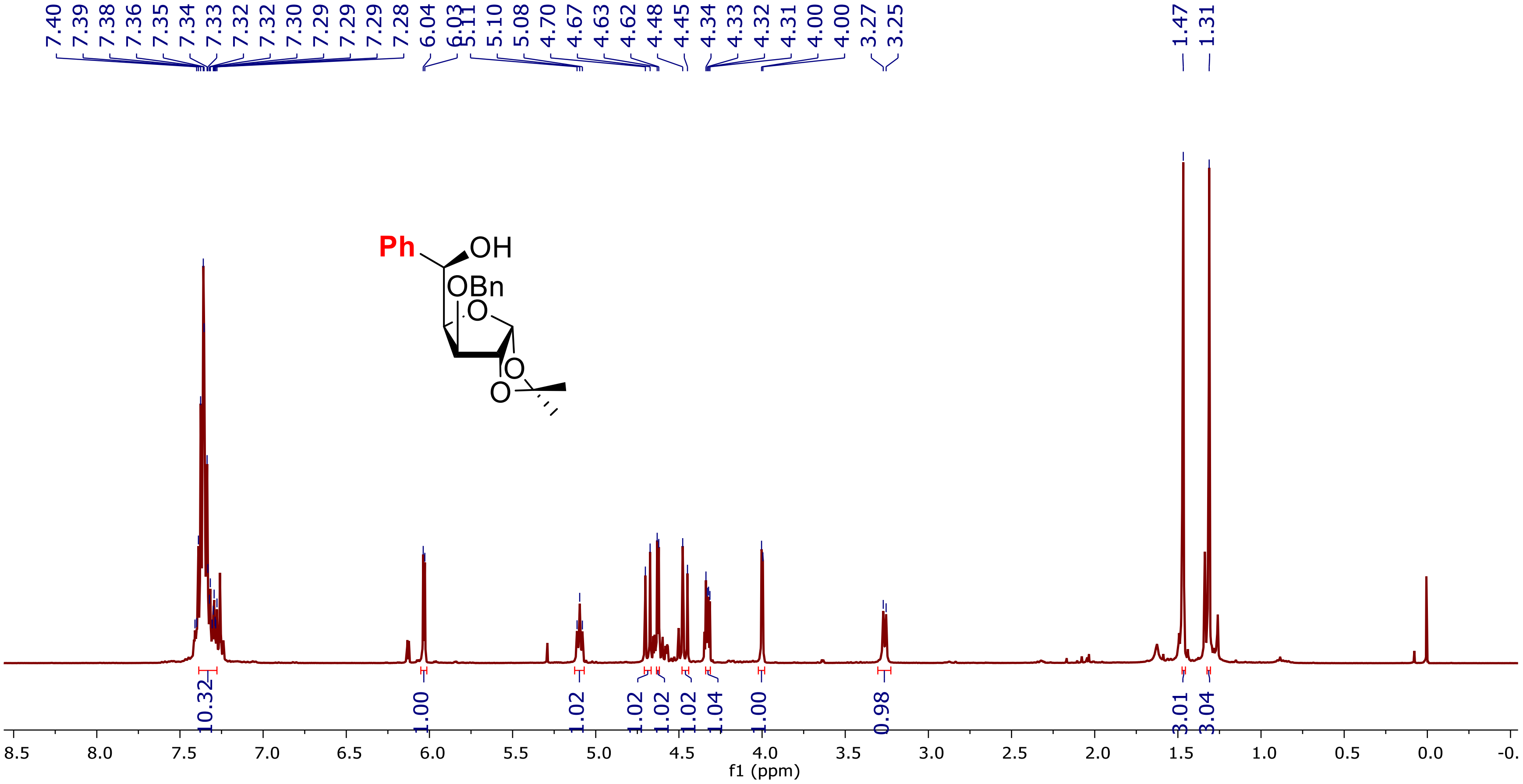

Supplementary Figure S9b. ^{13}C NMR Spectrum (100.53 MHz, $CDCl_3$) of Compound 16b

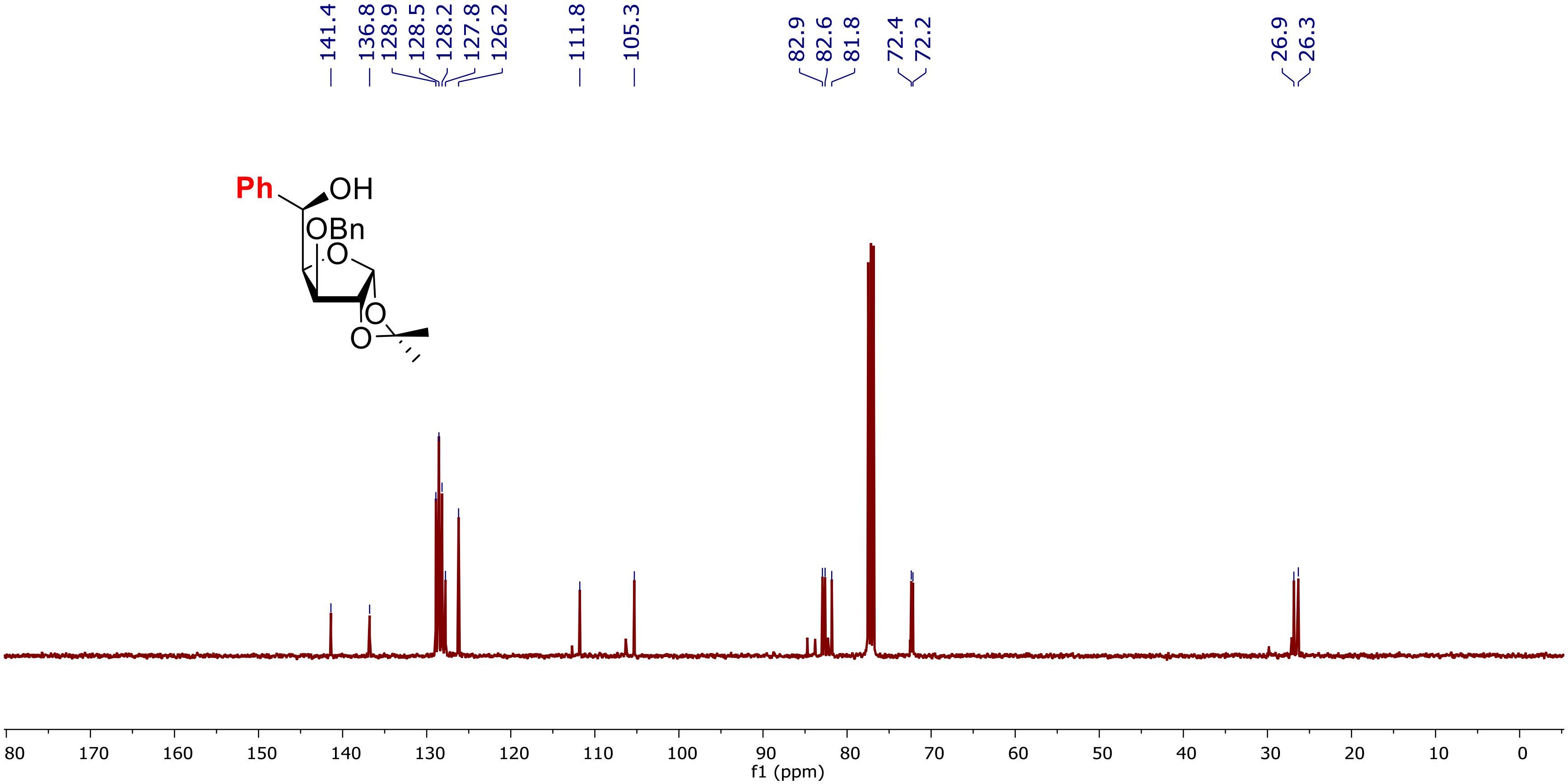

Supplementary Figure S9c. DEPT NMR Spectrum (100.53 MHz, $CDCl_3$) of Compound 16b

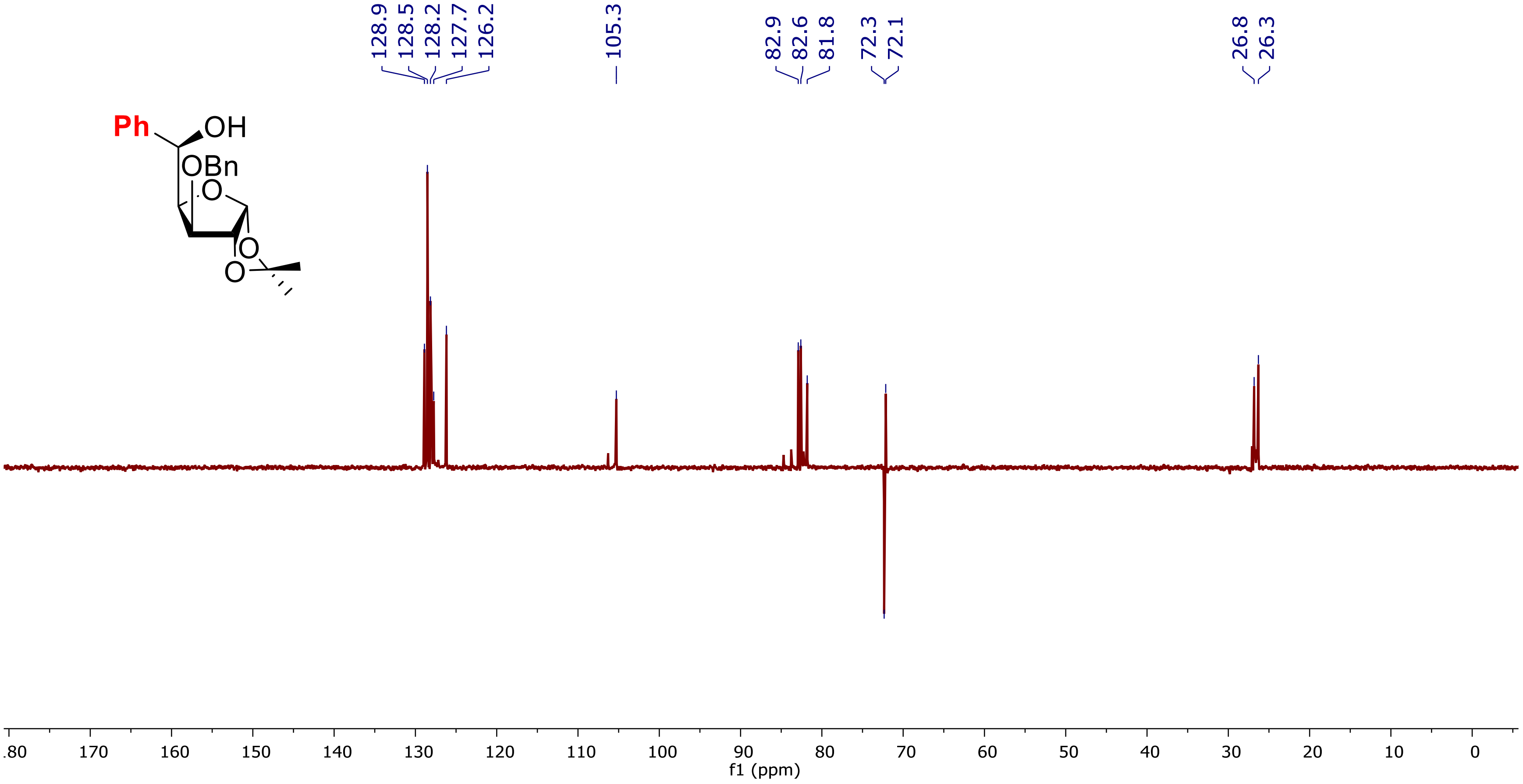

Supplementary Figure S10a. ^{1}H NMR Spectrum (400.31 MHz, $CDCl_3$) of Compound 17a

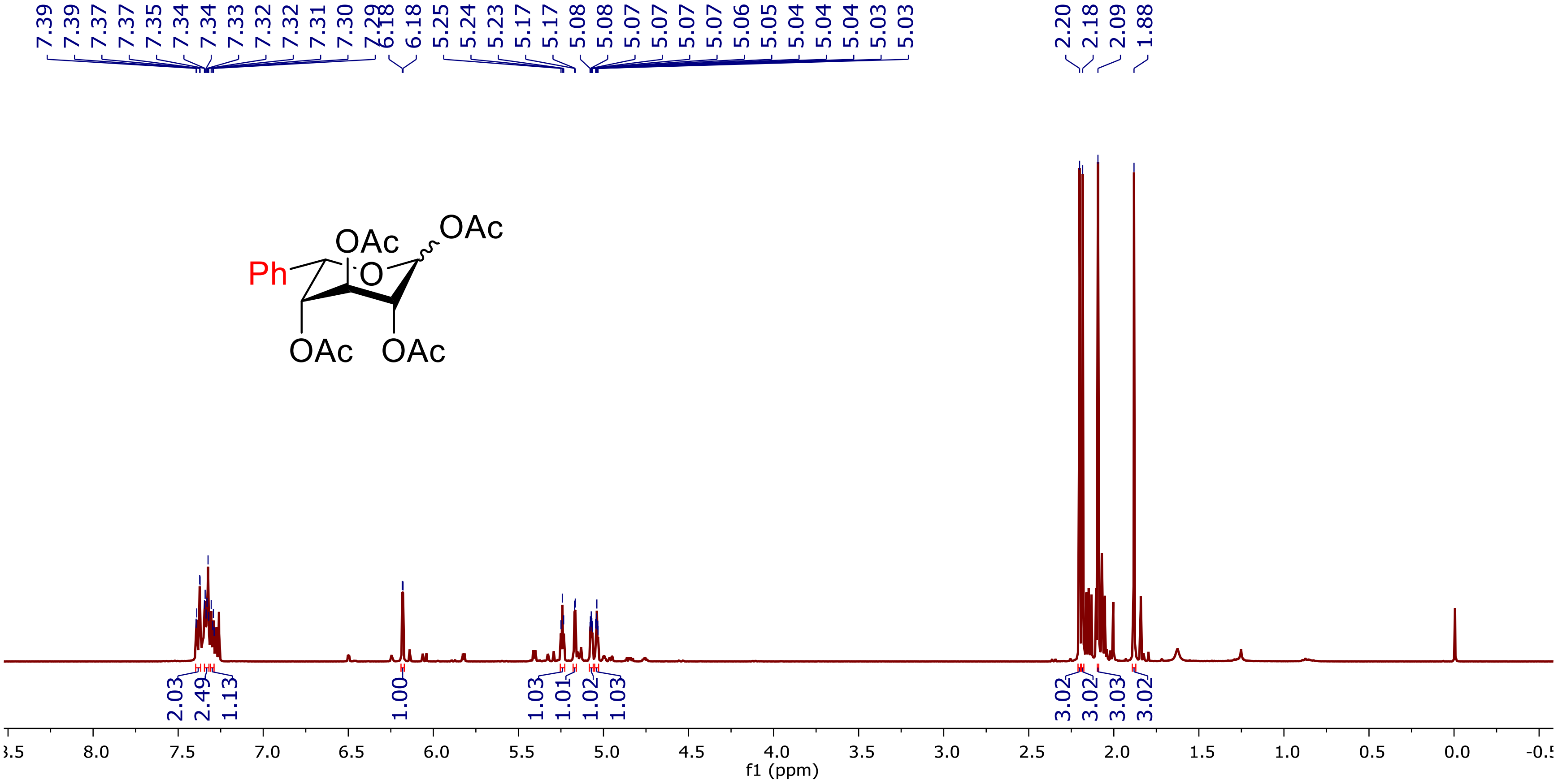

Supplementary Figure S10b. ¹³C NMR Spectrum (100.53 MHz, $CDCl_3$) of Compound 17a

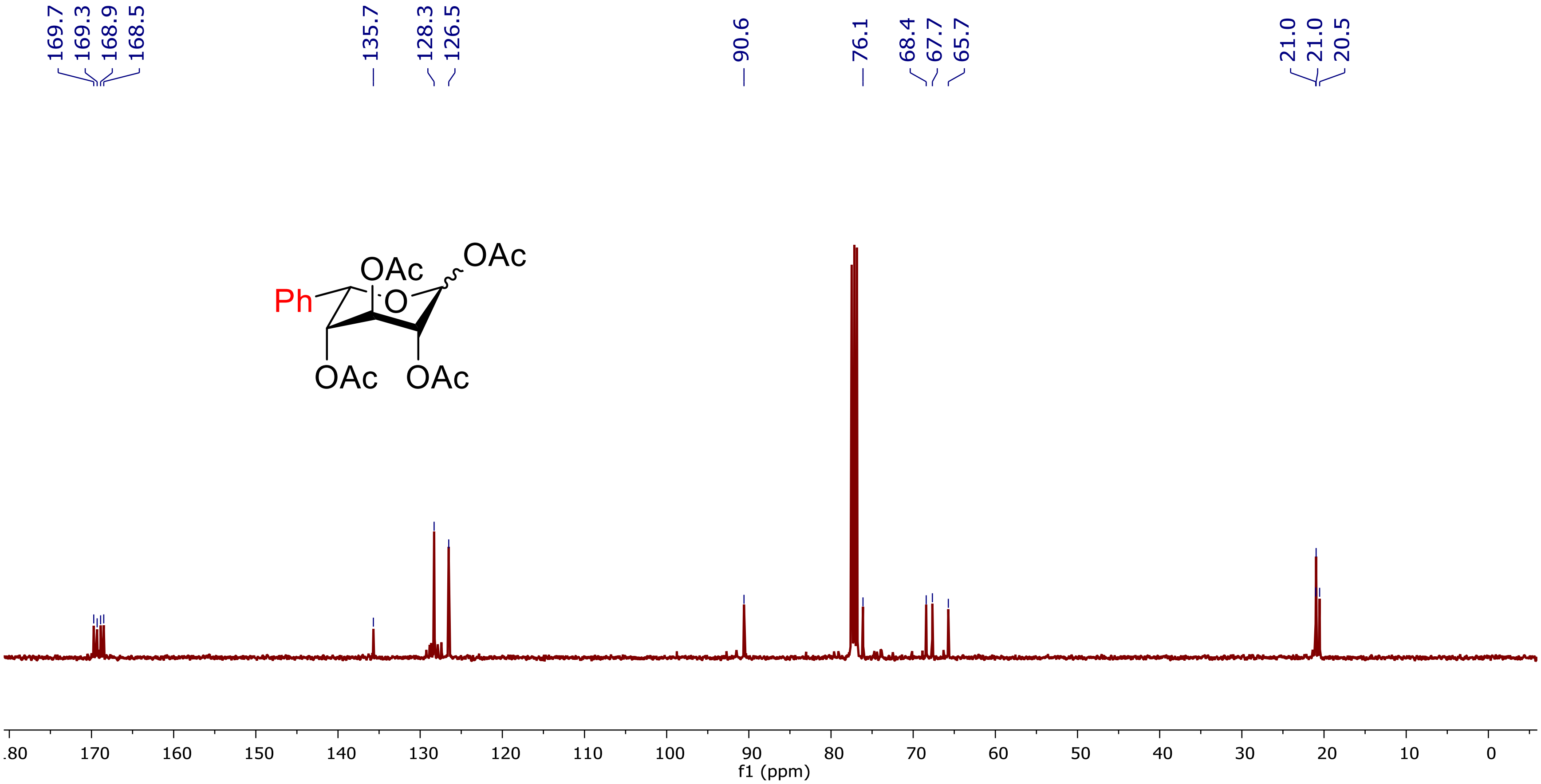

Supplementary Figure S10c. DEPT NMR Spectrum (100.53 MHz, $CDCl_3$) of Compound 17a

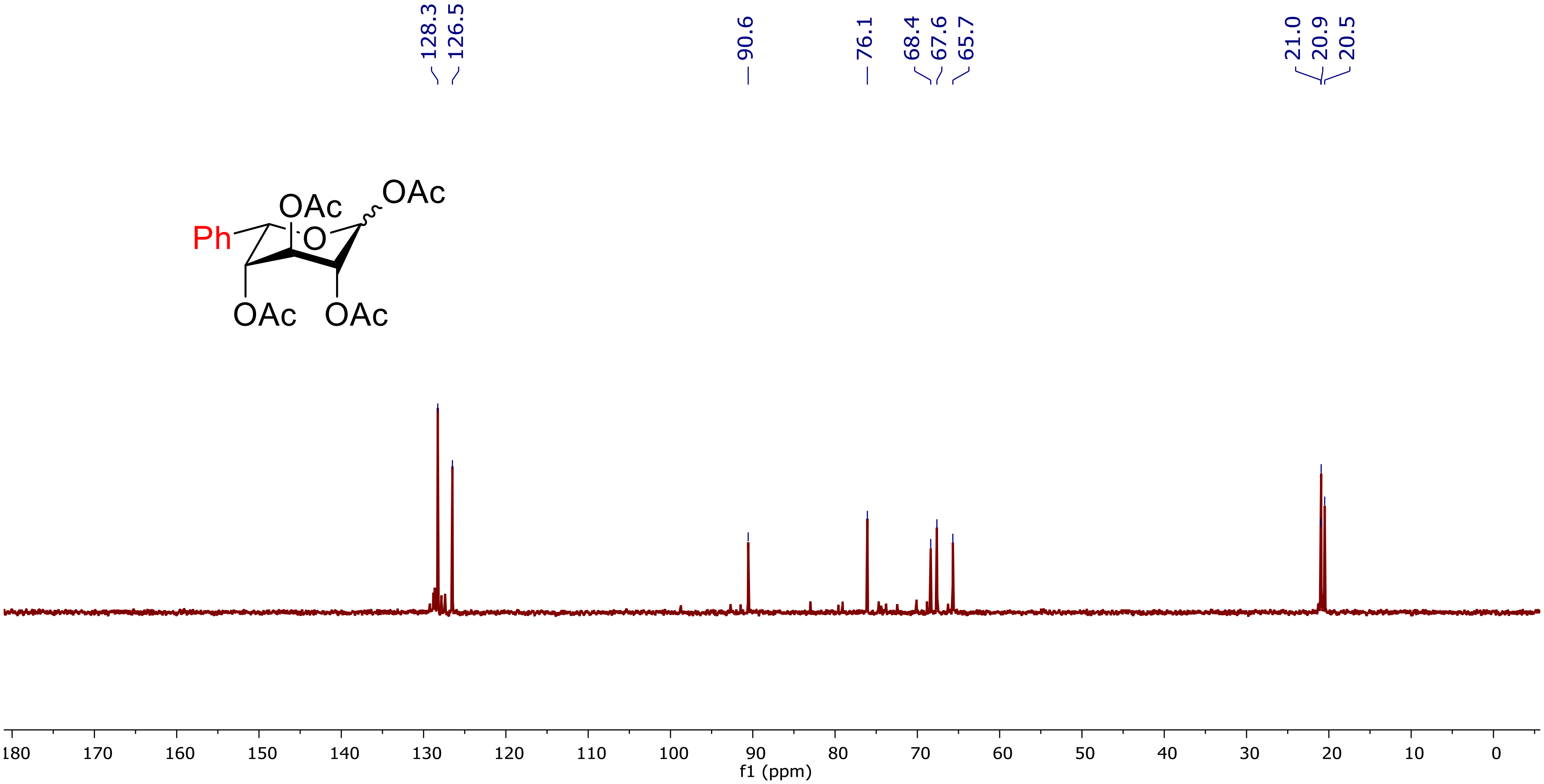

Supplementary Figure S11a. ¹H NMR Spectrum (400.31 MHz, $CDCl_3$) of Compound 17b

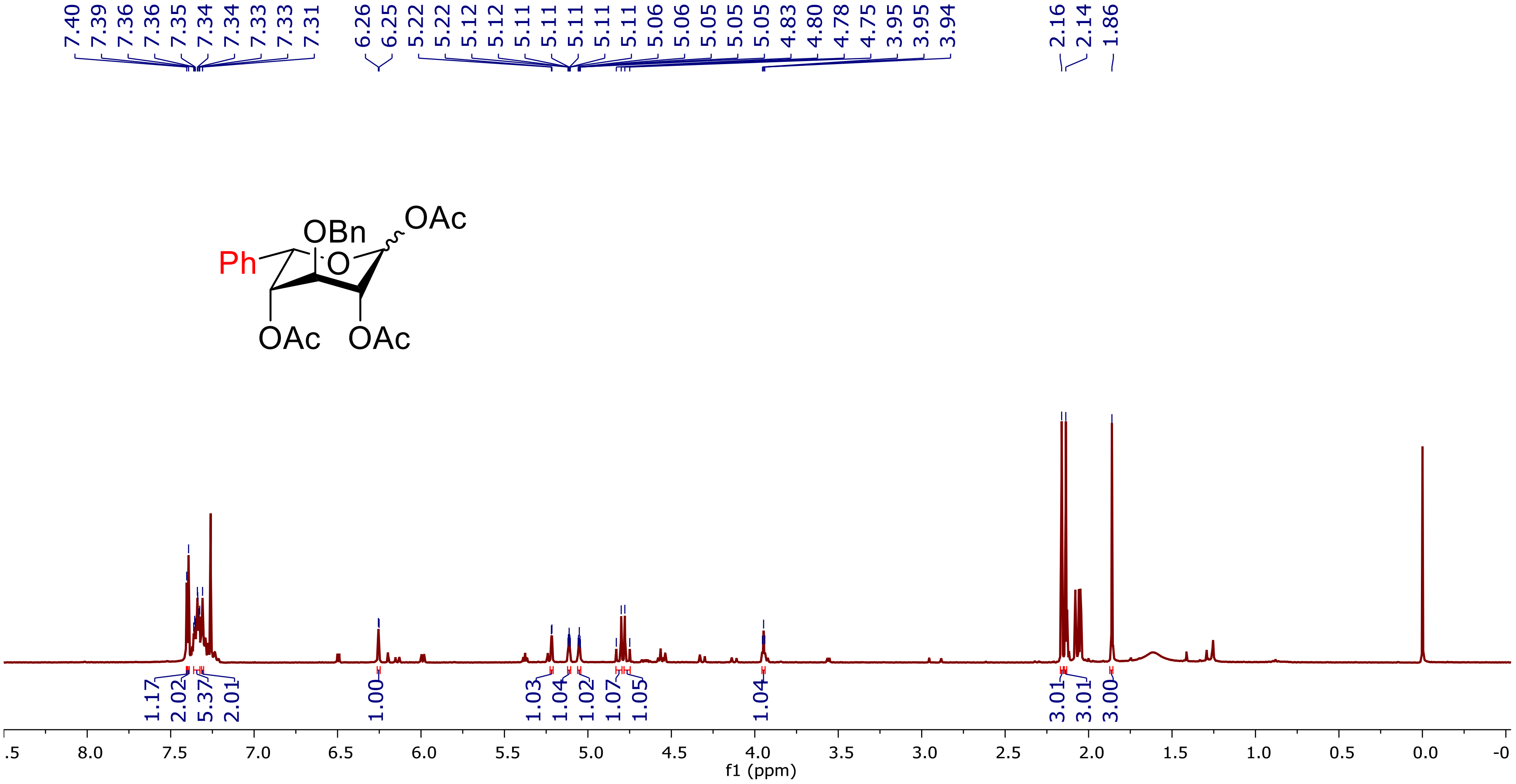

Supplementary Figure S11b. ^{13}**C NMR Spectrum (100.67 MHz, $CDCl_3$) of Compound 17b**

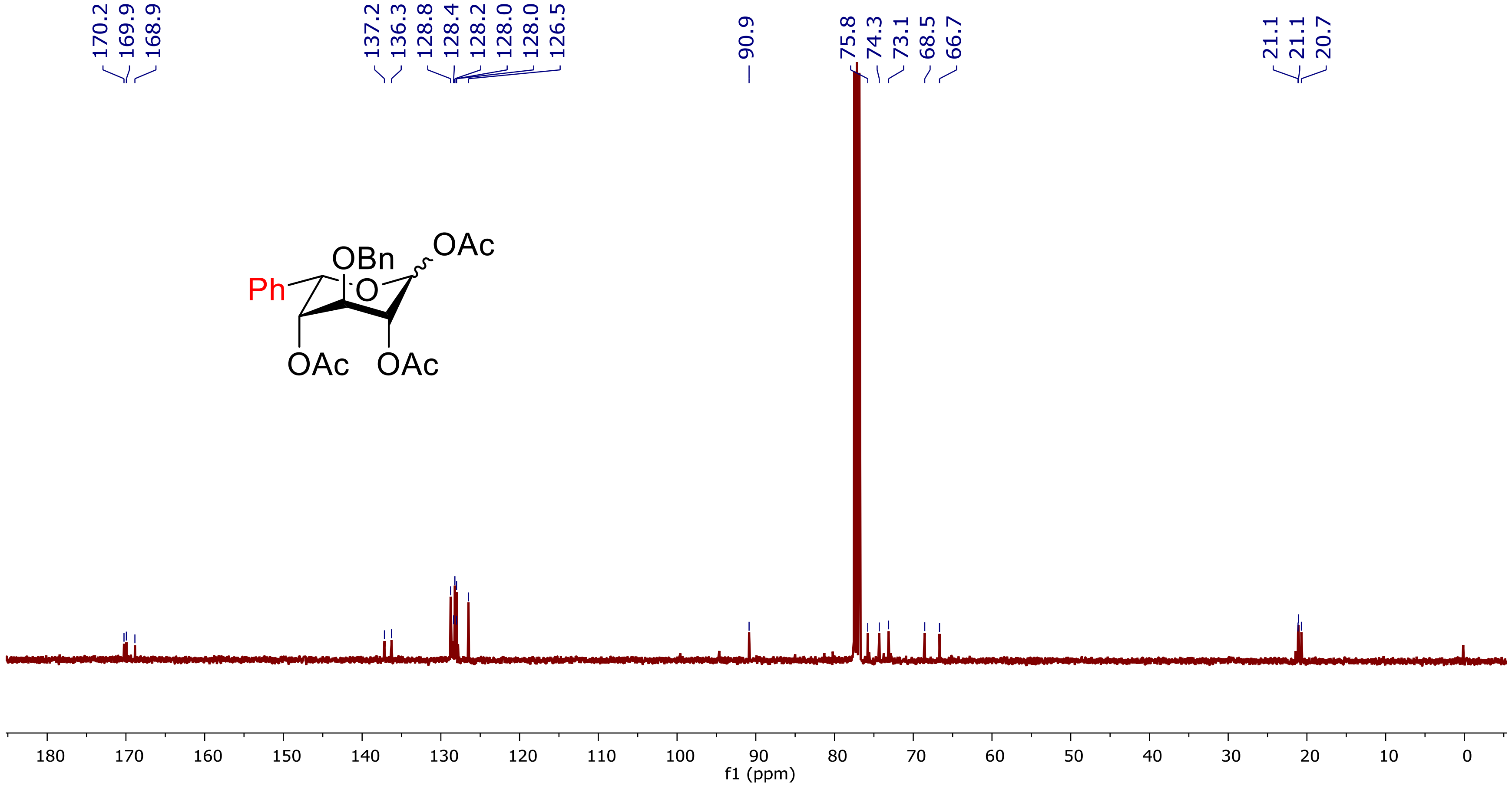

Supplementary Figure S11c. DEPT NMR Spectrum (100.53 MHz, $CDCl_3$) of Compound 17b

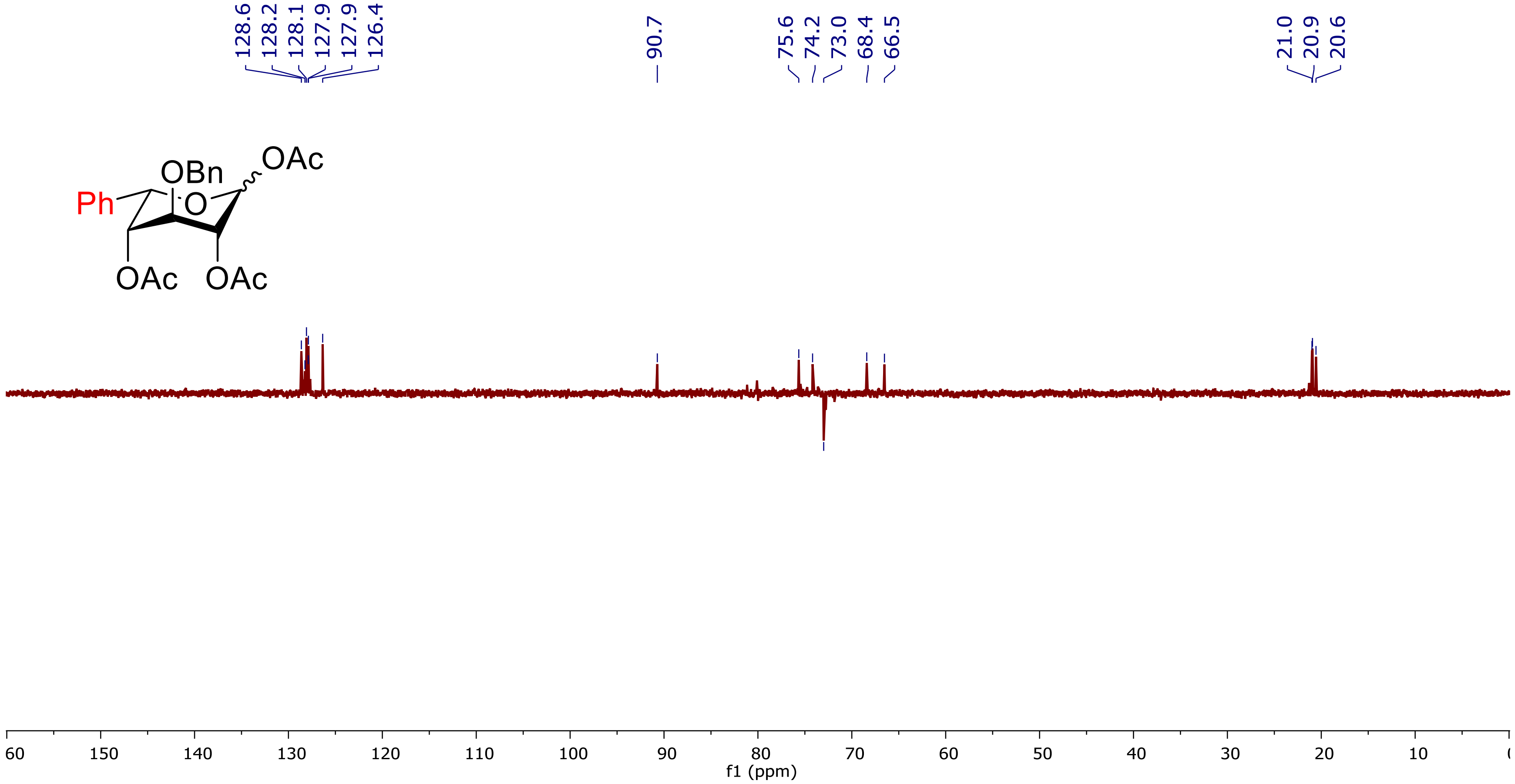

Supplementary Figure S12a. ^{1}H NMR Spectrum (400.31 MHz, $CDCl_3$) of Compound 18a

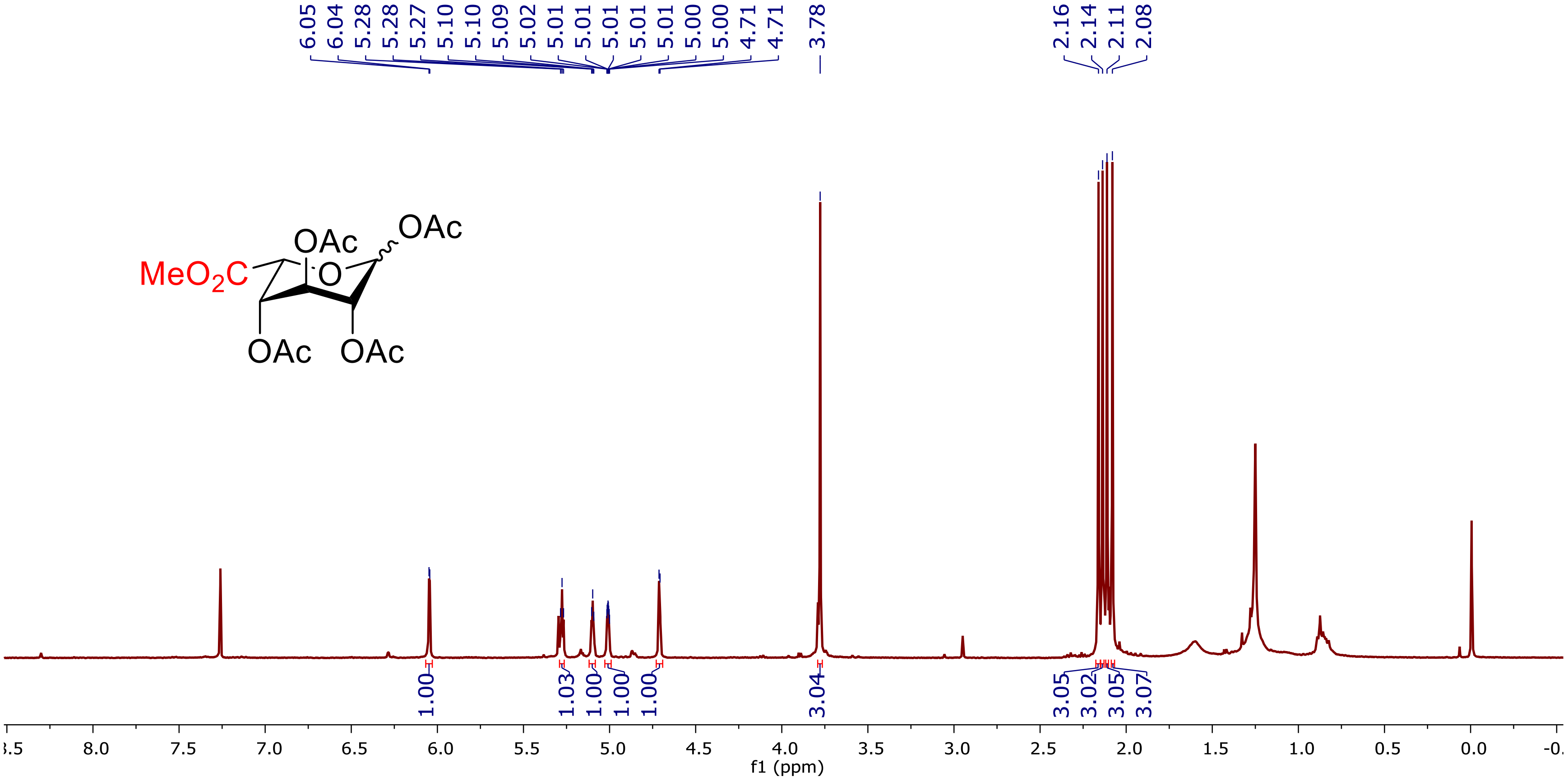

Supplementary Figure S12b. ^{13}C NMR Spectrum (100.53 MHz, $CDCl_3$) of Compound 18a

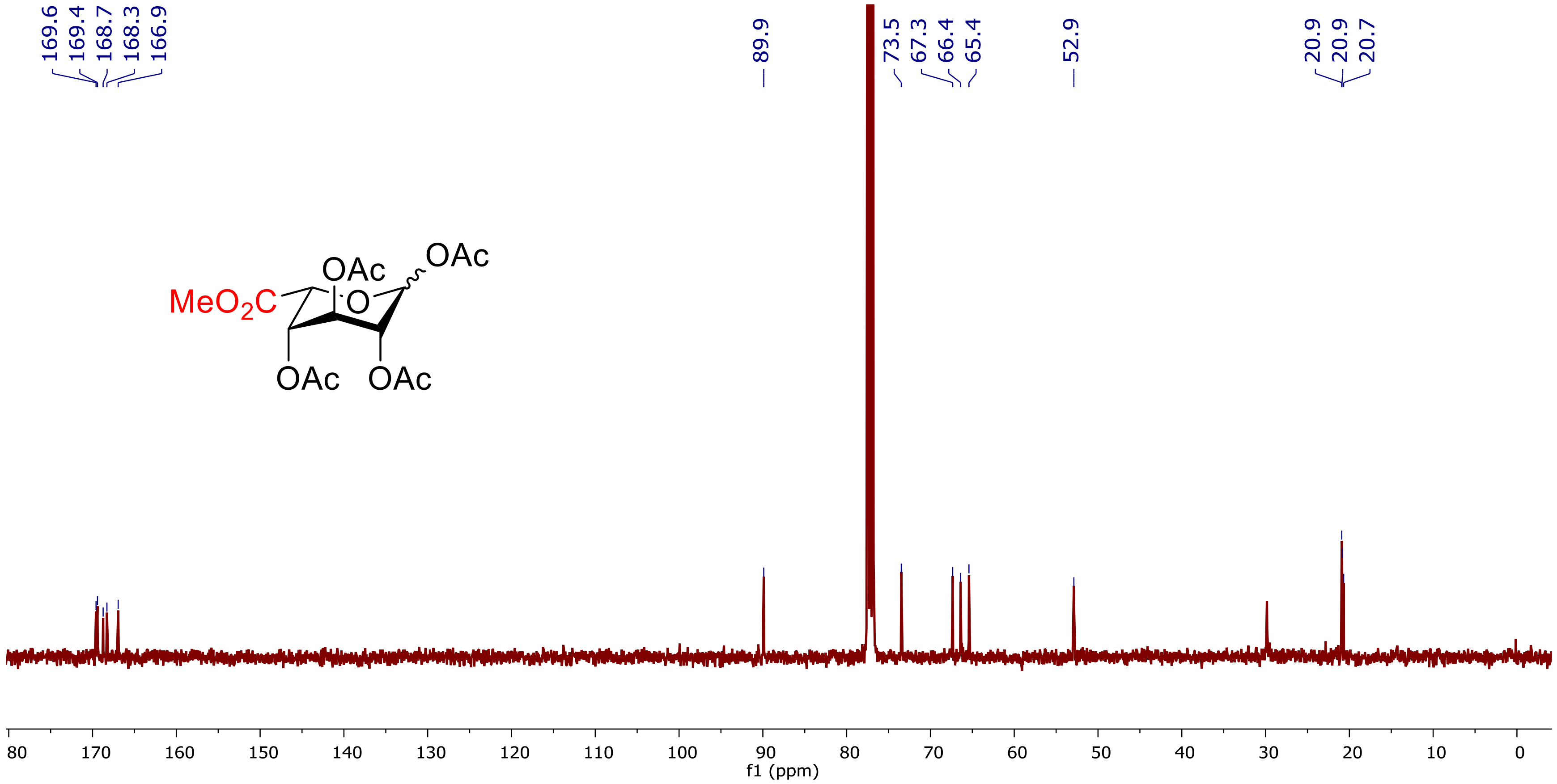

Supplementary Figure S12c. DEPT NMR Spectrum (100.53 MHz, CDCl$_3$) of Compound 18a

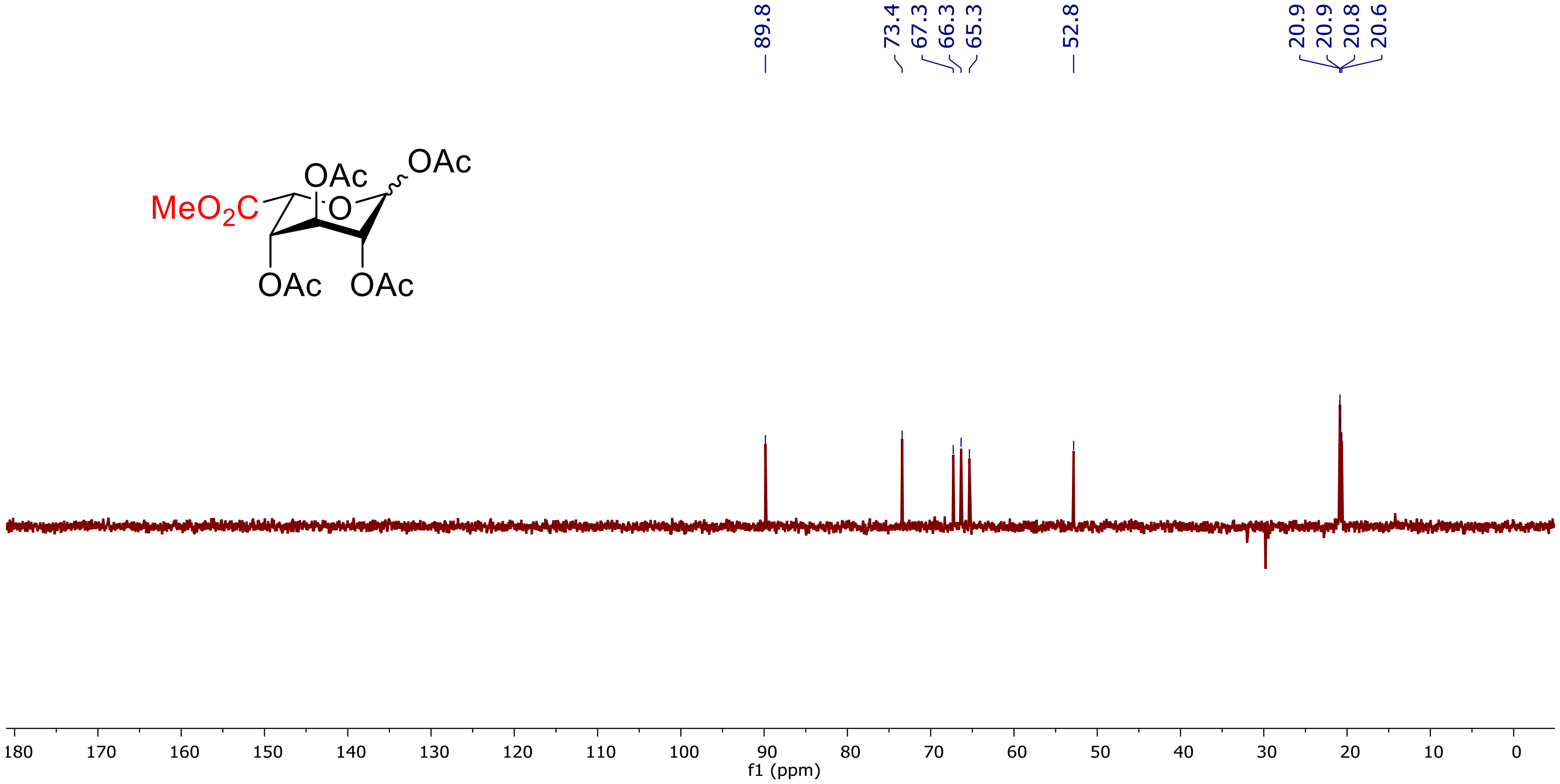

Supplementary Figure S13a. ^1H NMR Spectrum (400.31 MHz, $CDCl_3$) of Compound 19 (Ido isomer)

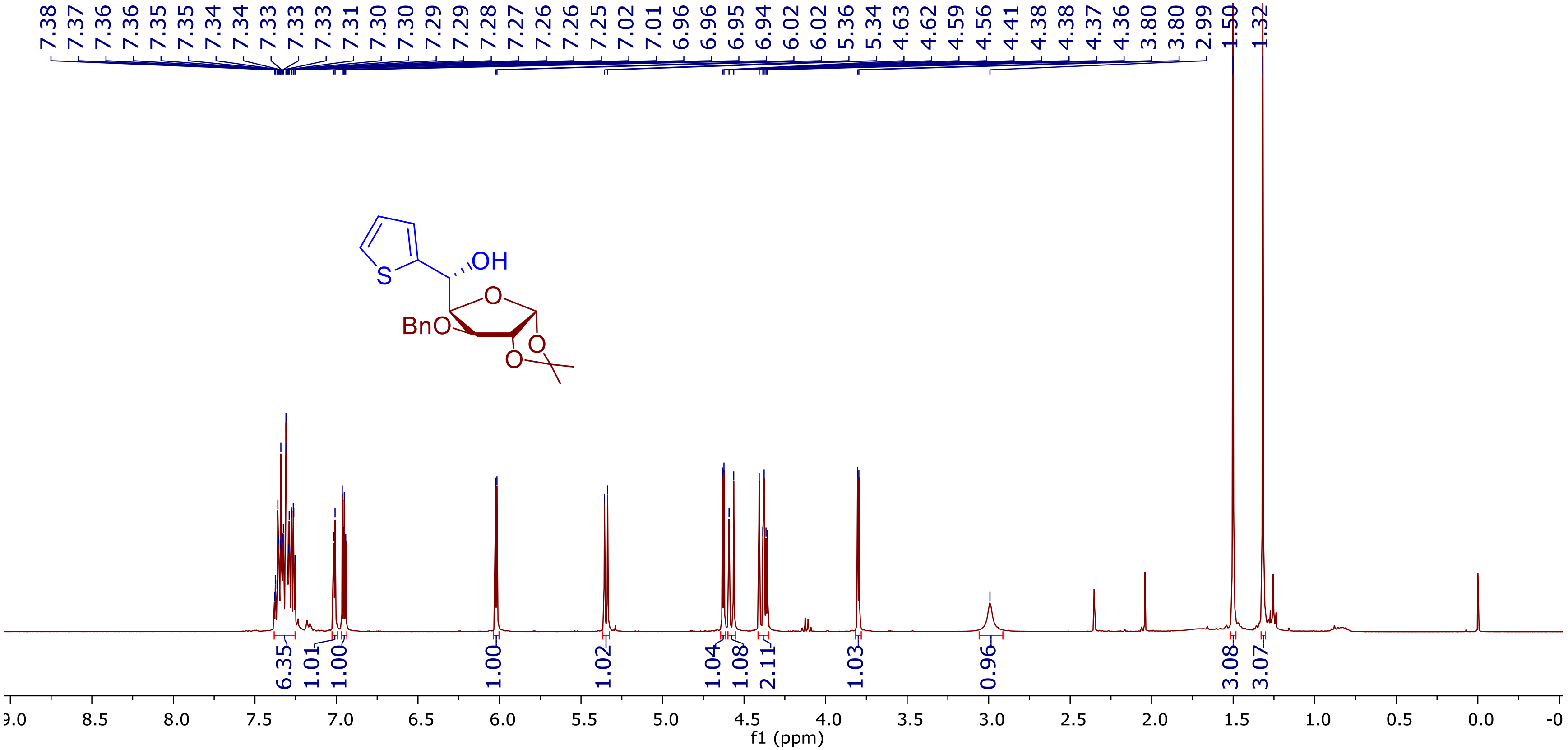

Supplementary Figure S13b. ^{13}C NMR Spectrum (100.67 MHz, $CDCl_3$) of Compound 19 (Ido isomer)

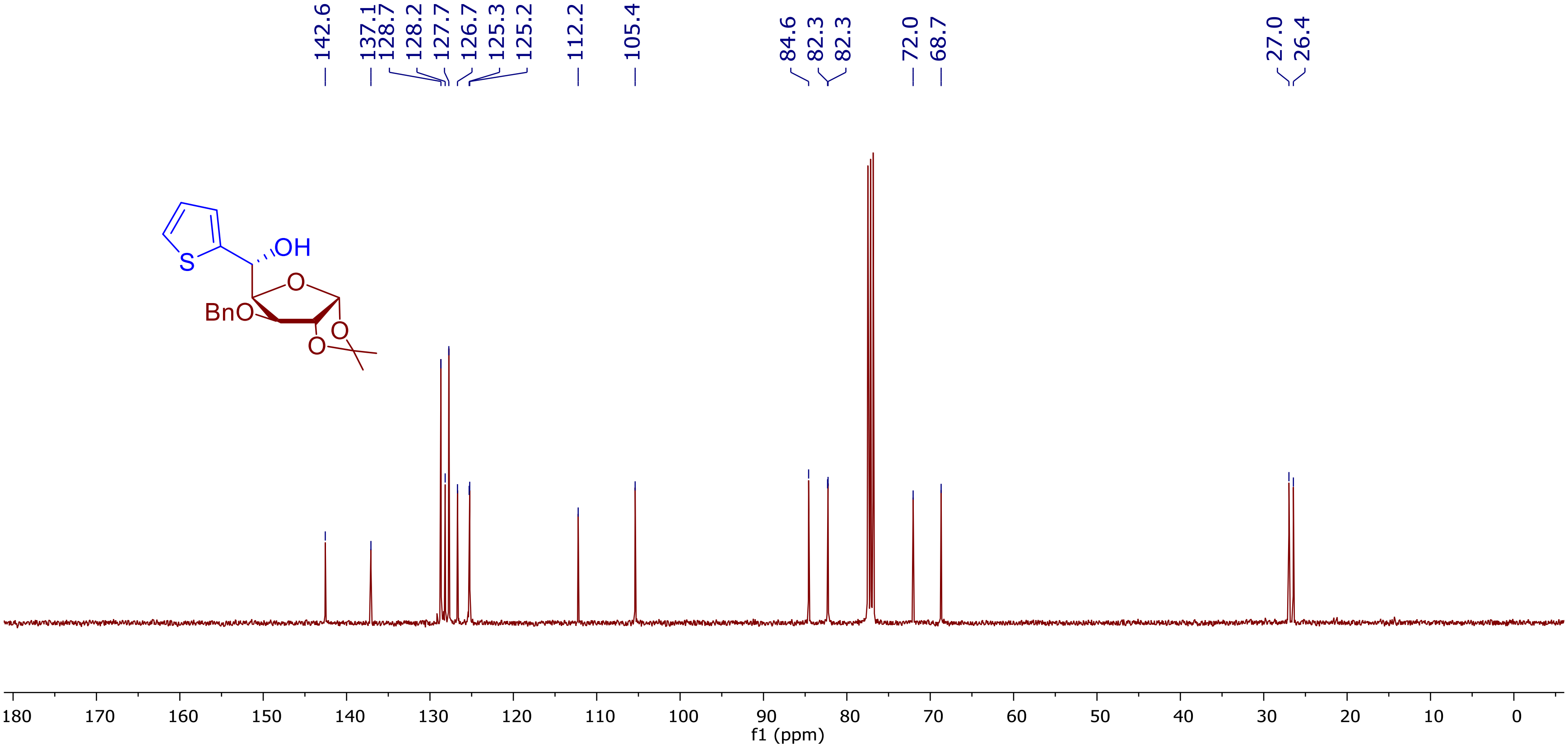

Supplementary Figure S13c. DEPT NMR Spectrum (100.67 MHz, $CDCl_3$) of Compound 19 (Ido isomer)

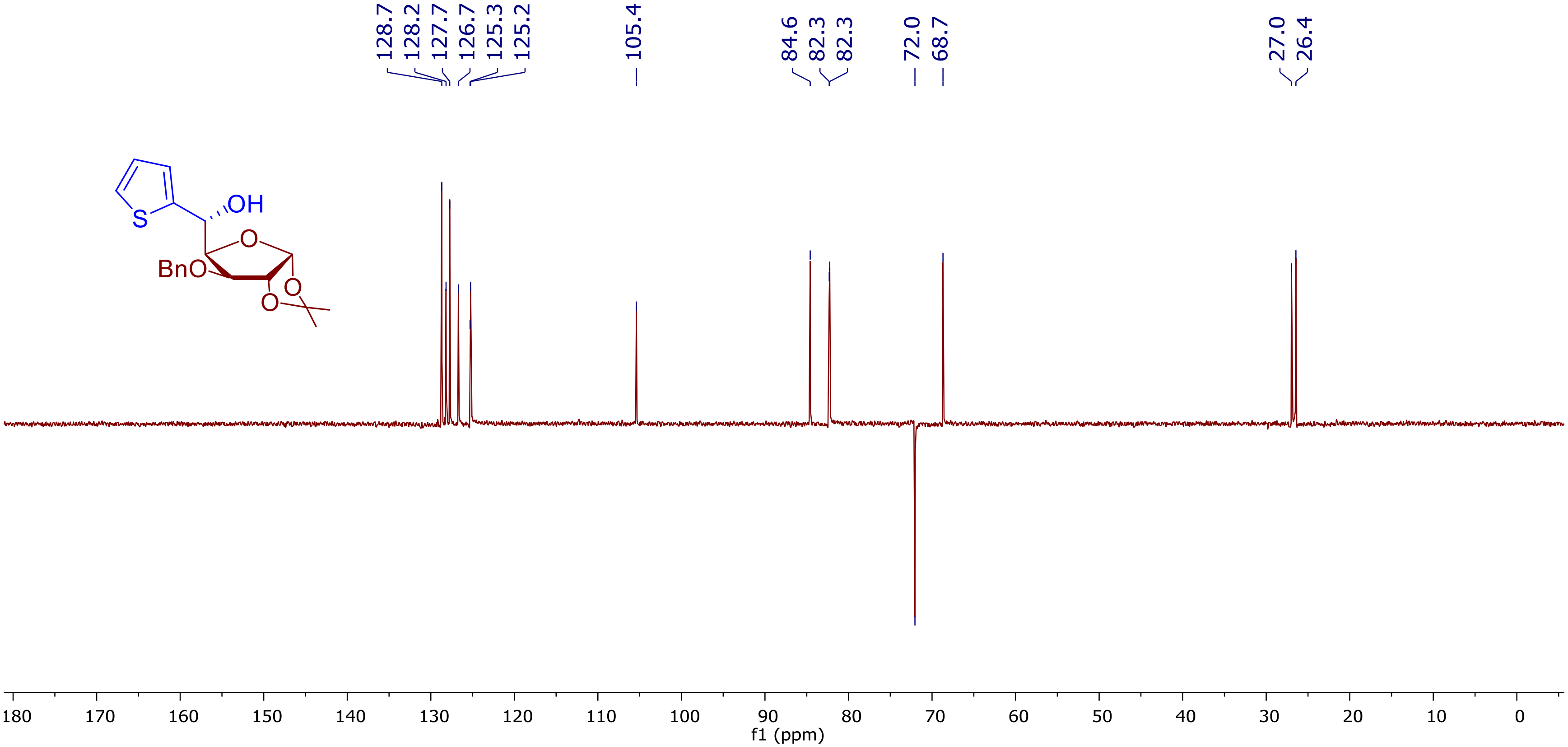

Supplementary Figure S14a. ^{1}H NMR Spectrum (400.31 MHz, $CDCl_3$) of Compound 19 (Glc isomer)

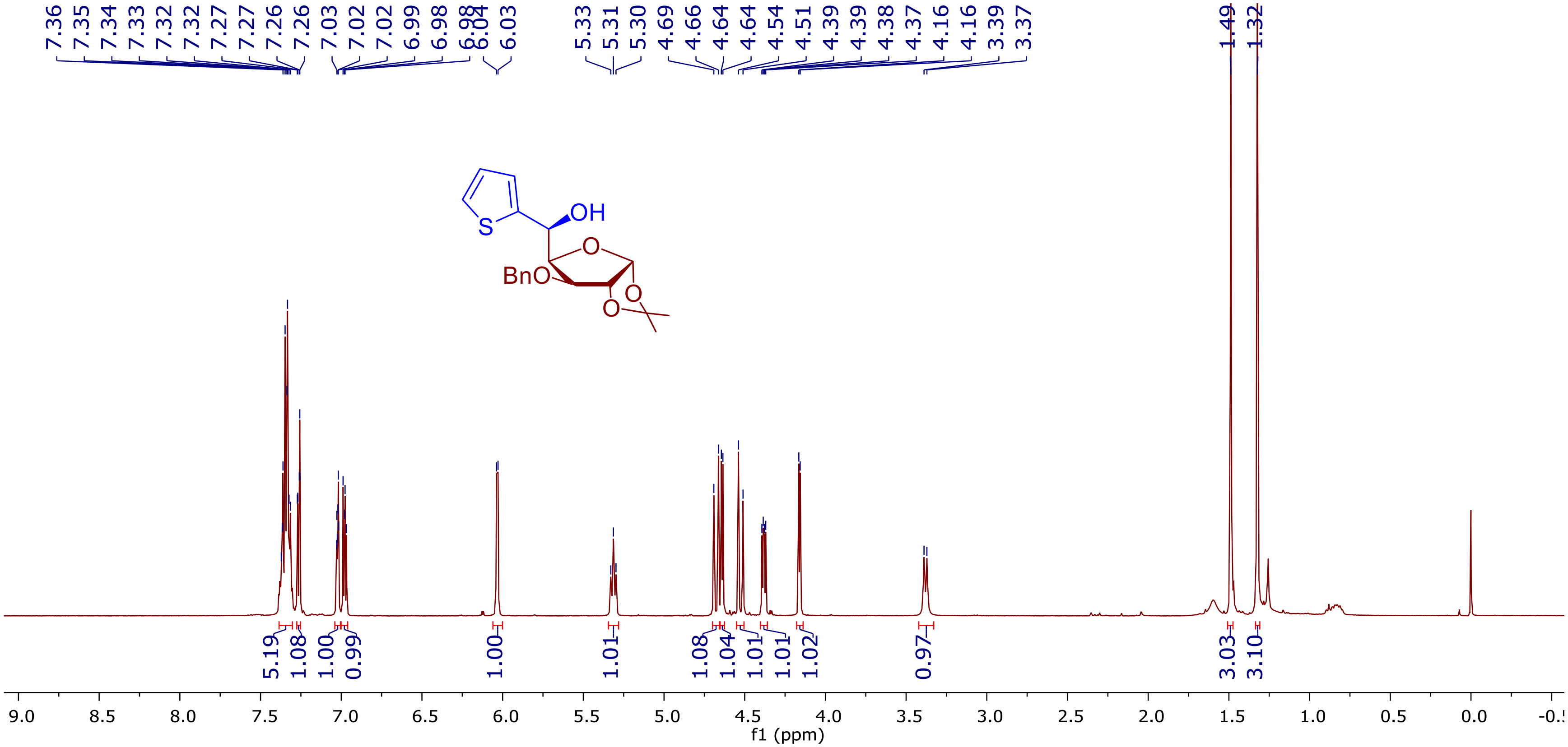

Supplementary Figure S14b. ^{13}C NMR Spectrum (100.67 MHz, $CDCl_3$) of Compound 19 (Glc isomer)

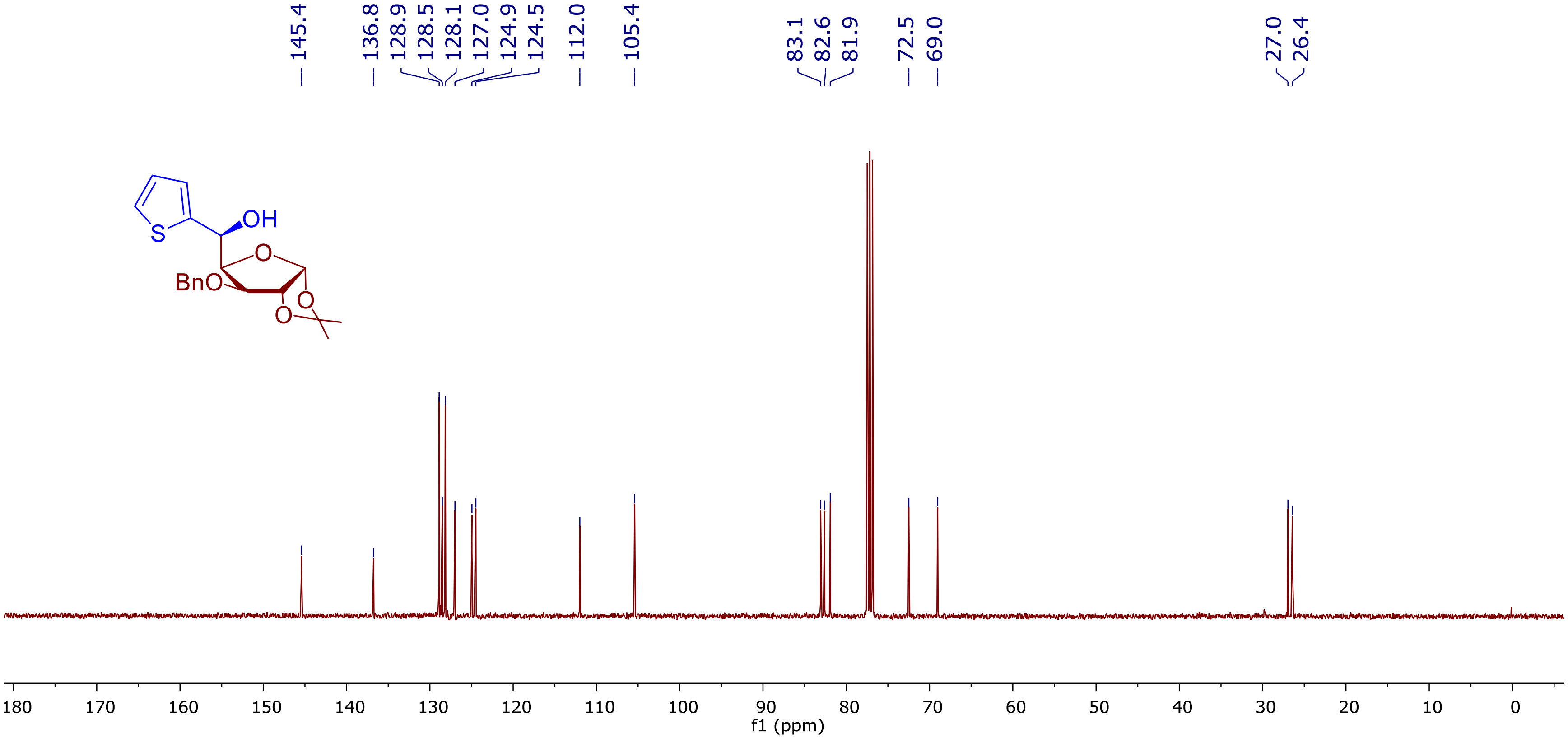

Supplementary Figure S14c. DEPT NMR Spectrum (100.67 MHz, $CDCl_3$) of Compound 19 (Glc isomer)

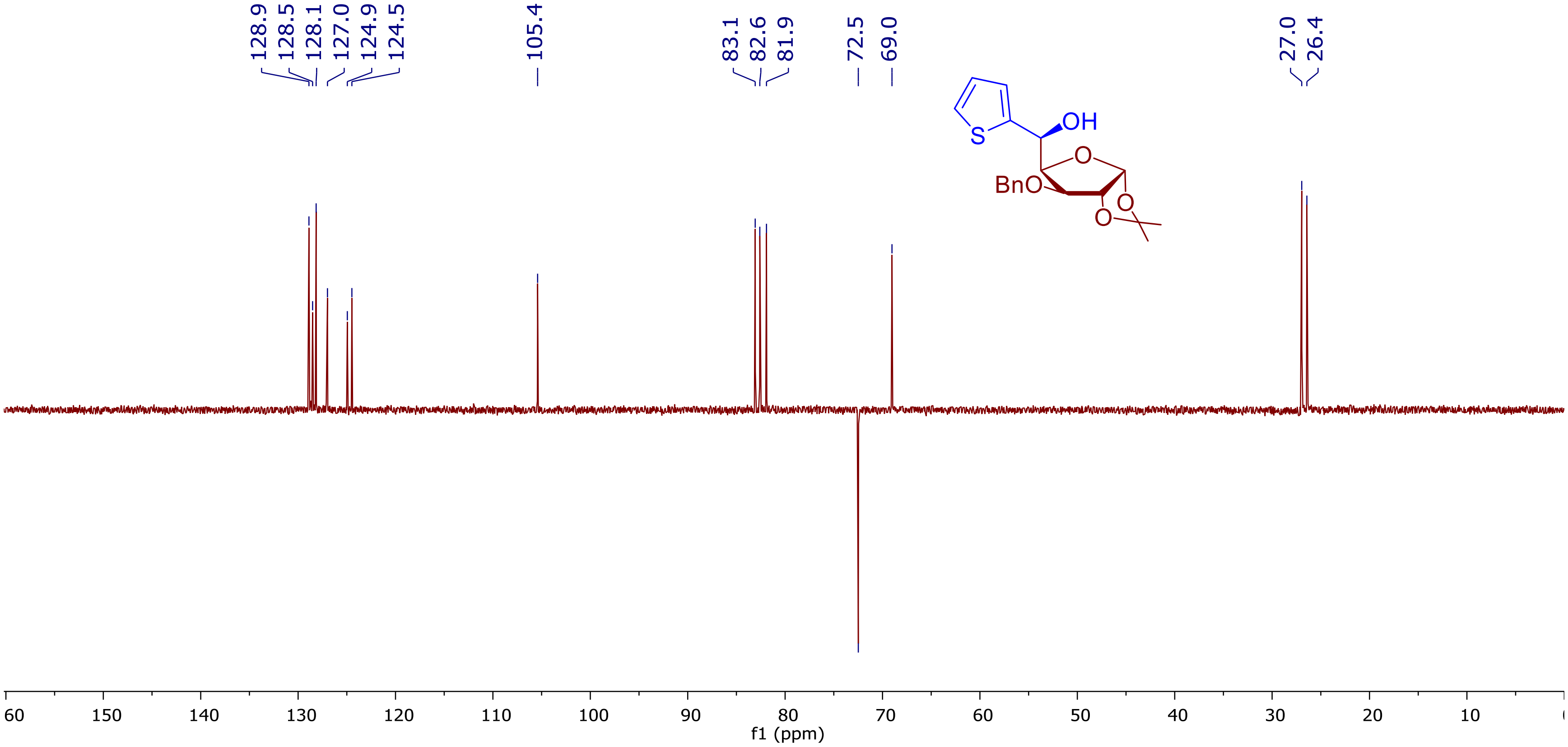

Supplementary Figure S15a.^{1}H NMR Spectrum (400.31 MHz, $CDCl_3$) of Compound 20

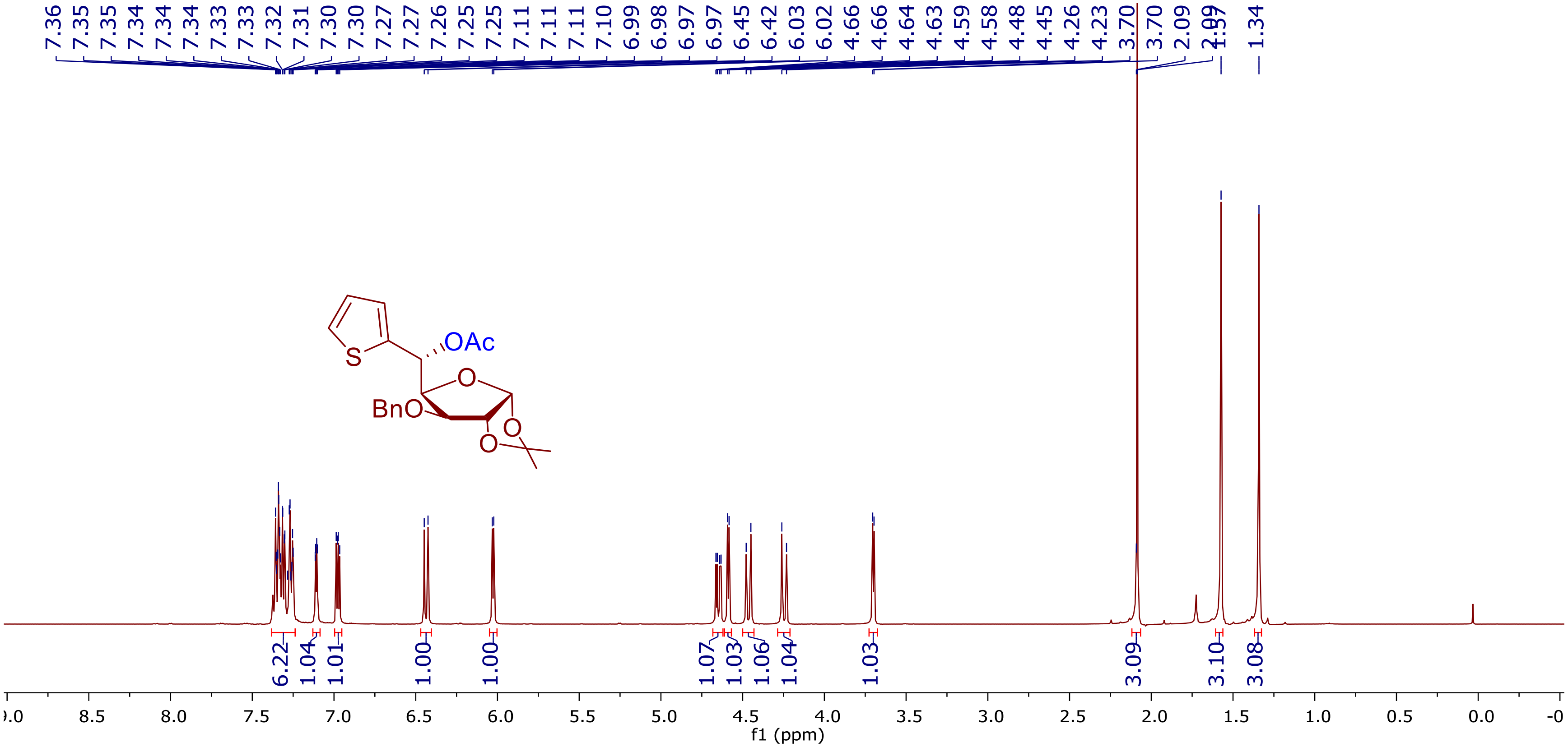

Supplementary Figure S15b. ^{13}C NMR Spectrum (100.67 MHz, $CDCl_3$) of Compound 20

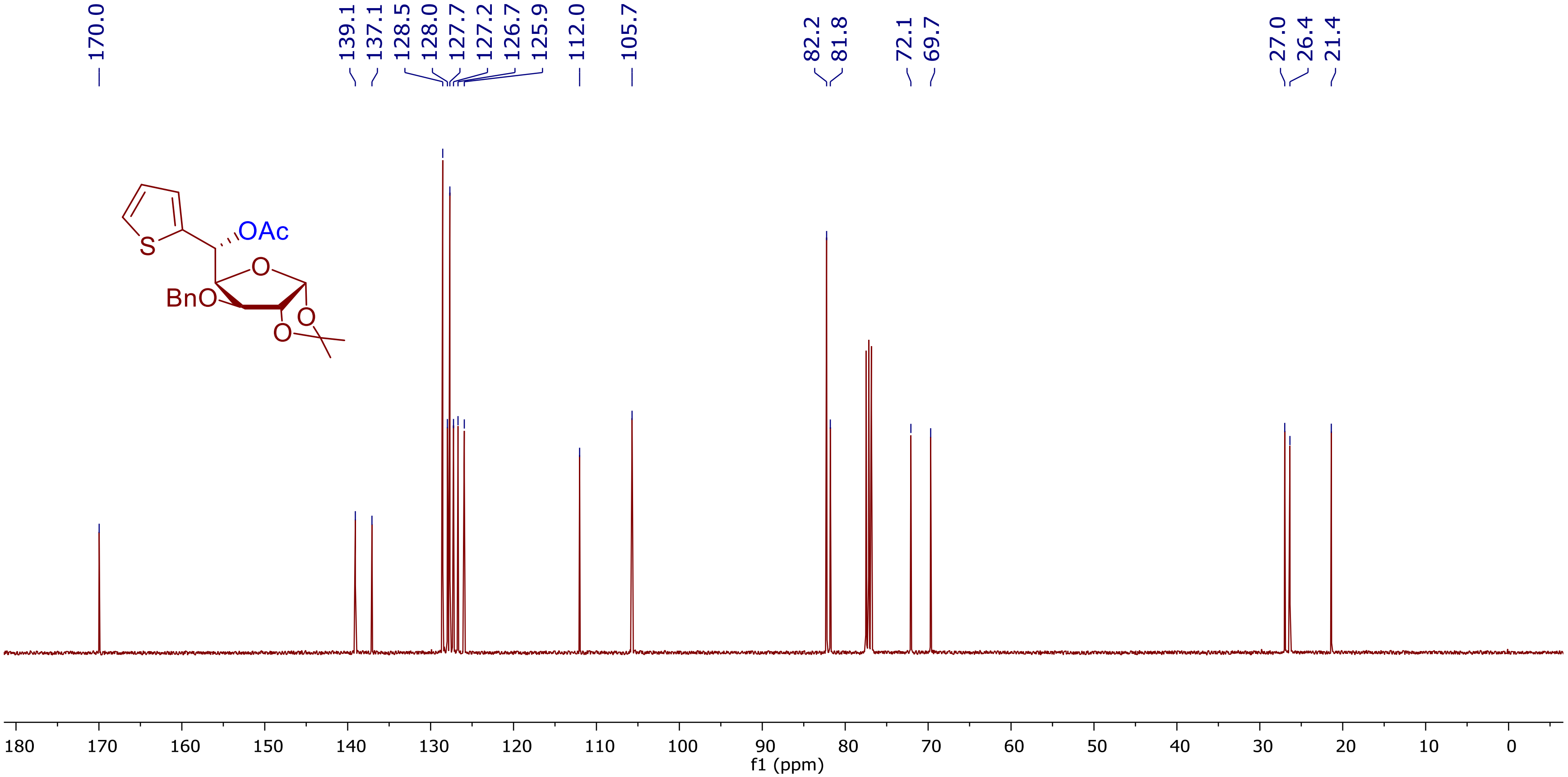

Supplementary Figure S15c. DEPT NMR Spectrum (100.67 MHz, $CDCl_3$) of Compound 20

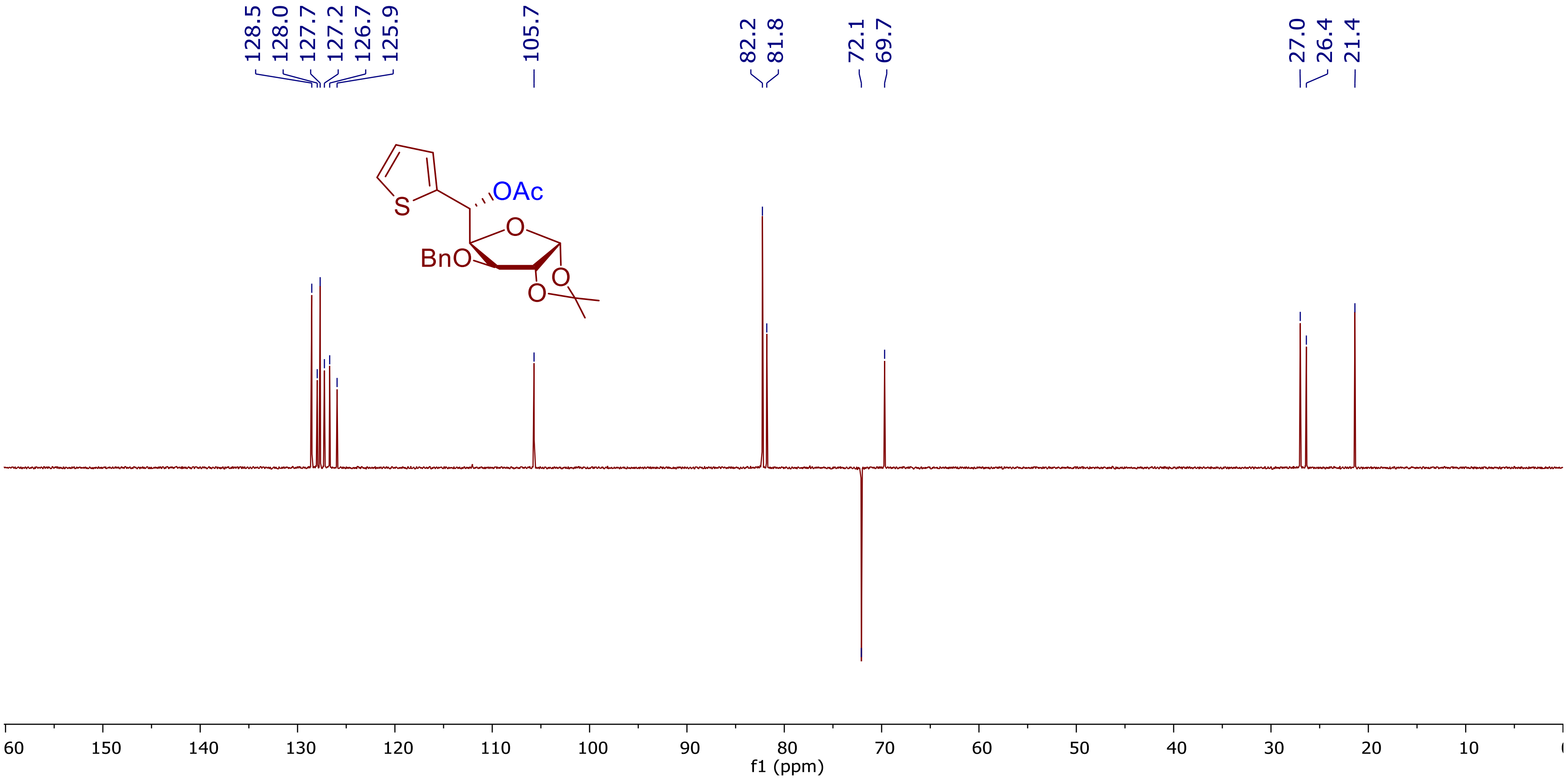

Supplementary Figure S16a. ^{1}H NMR Spectrum (400.31 MHz, $CDCl_3$) of Compound S3

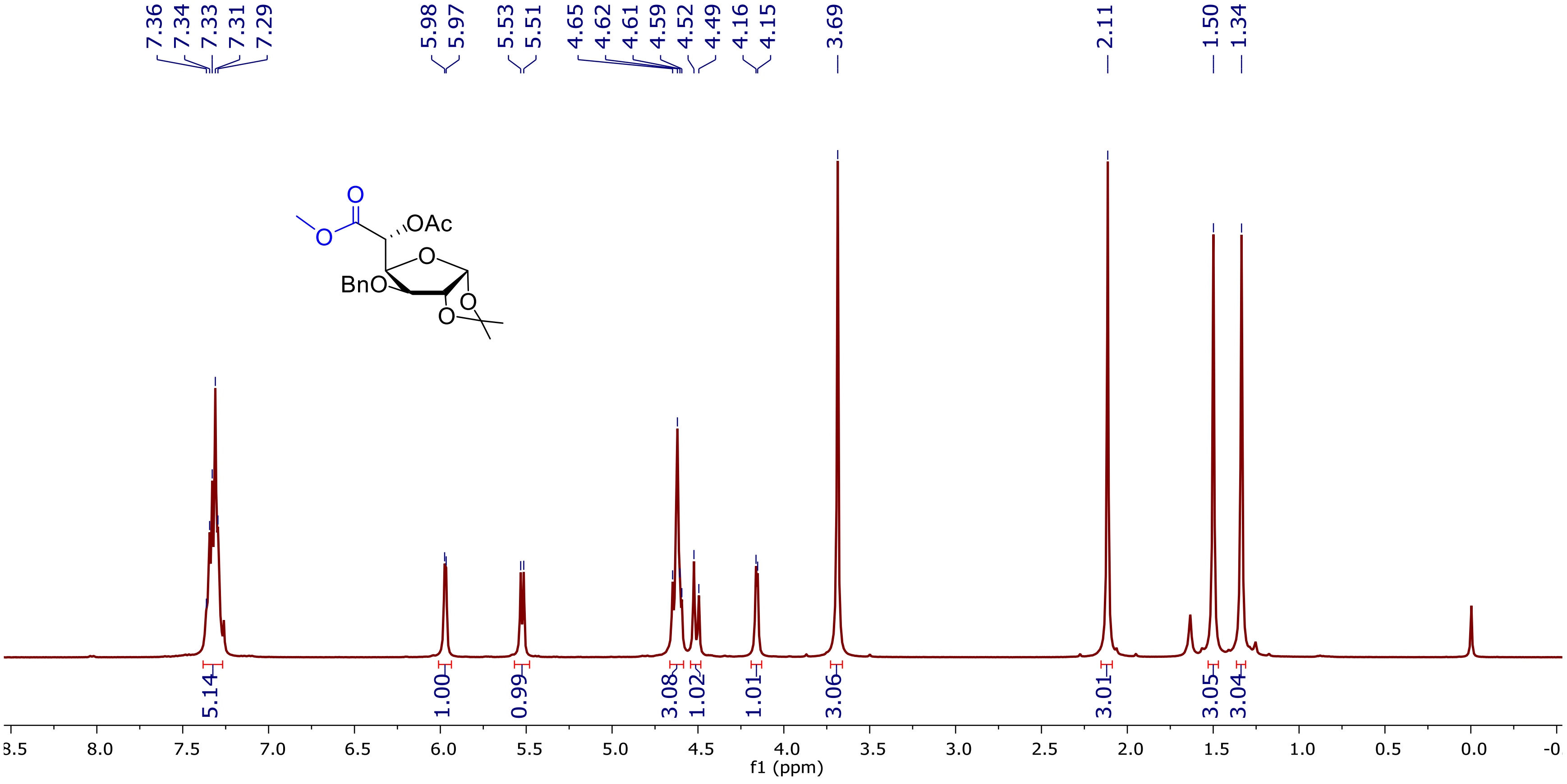

Supplementary Figure S16b. ^{13}C NMR Spectrum (100.67 MHz, $CDCl_3$) of Compound S3

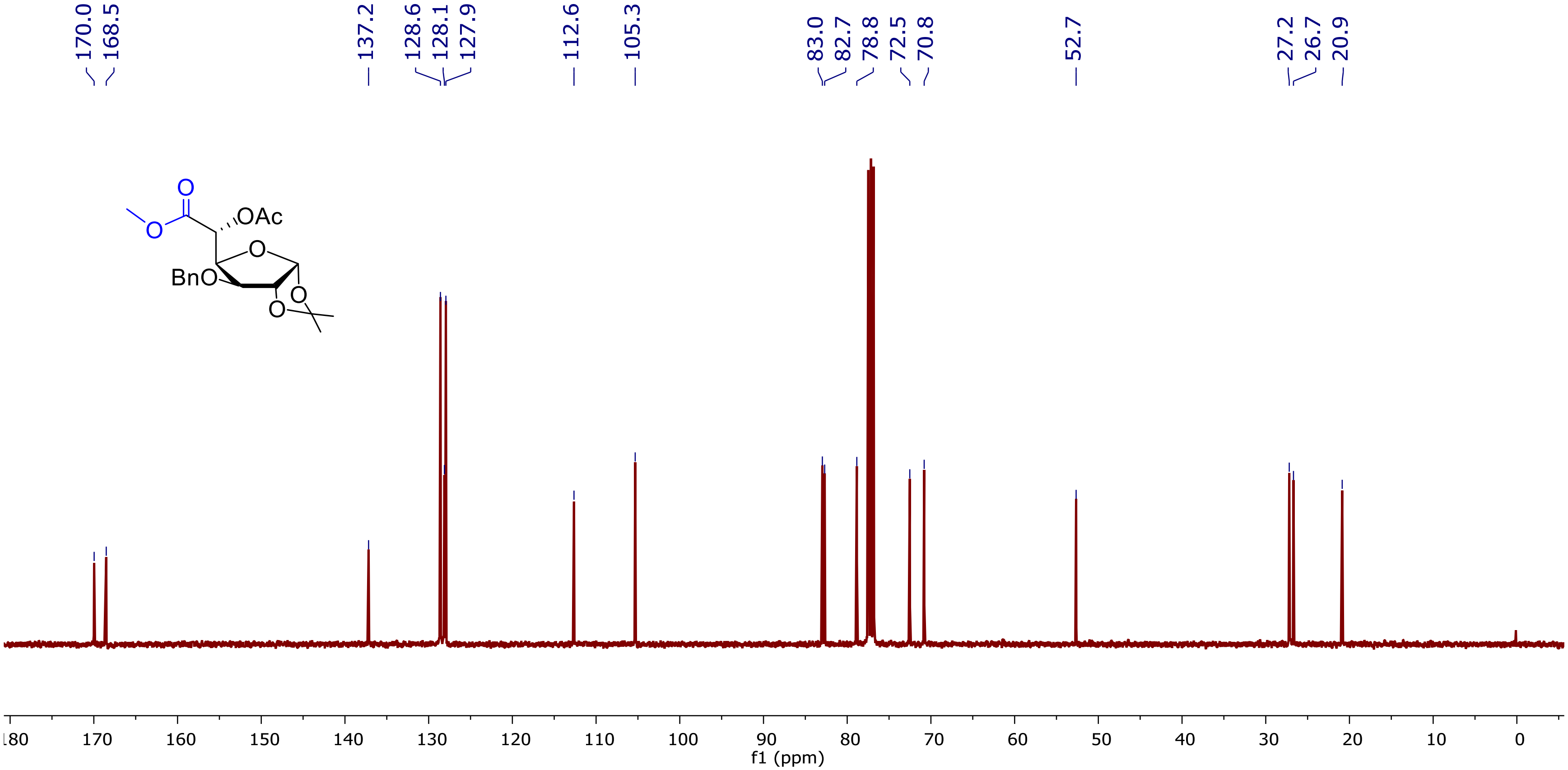

Supplementary Figure S16c. DEPT NMR Spectrum (100.67 MHz, $CDCl_3$) of Compound S3

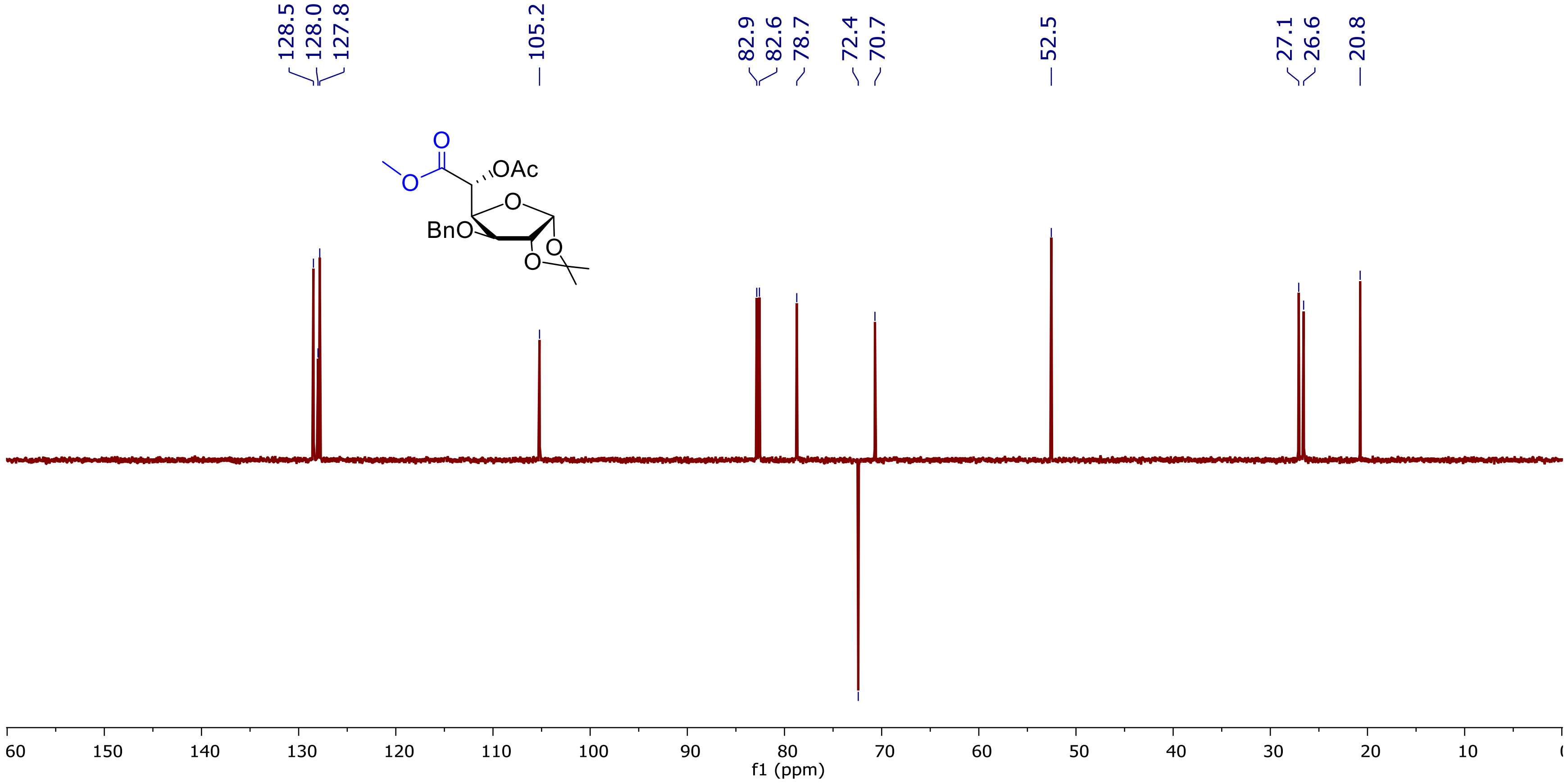

Supplementary Figure S17a. ^{1}H NMR Spectrum (399.78 MHz, $CDCl_3$) of Compound 21

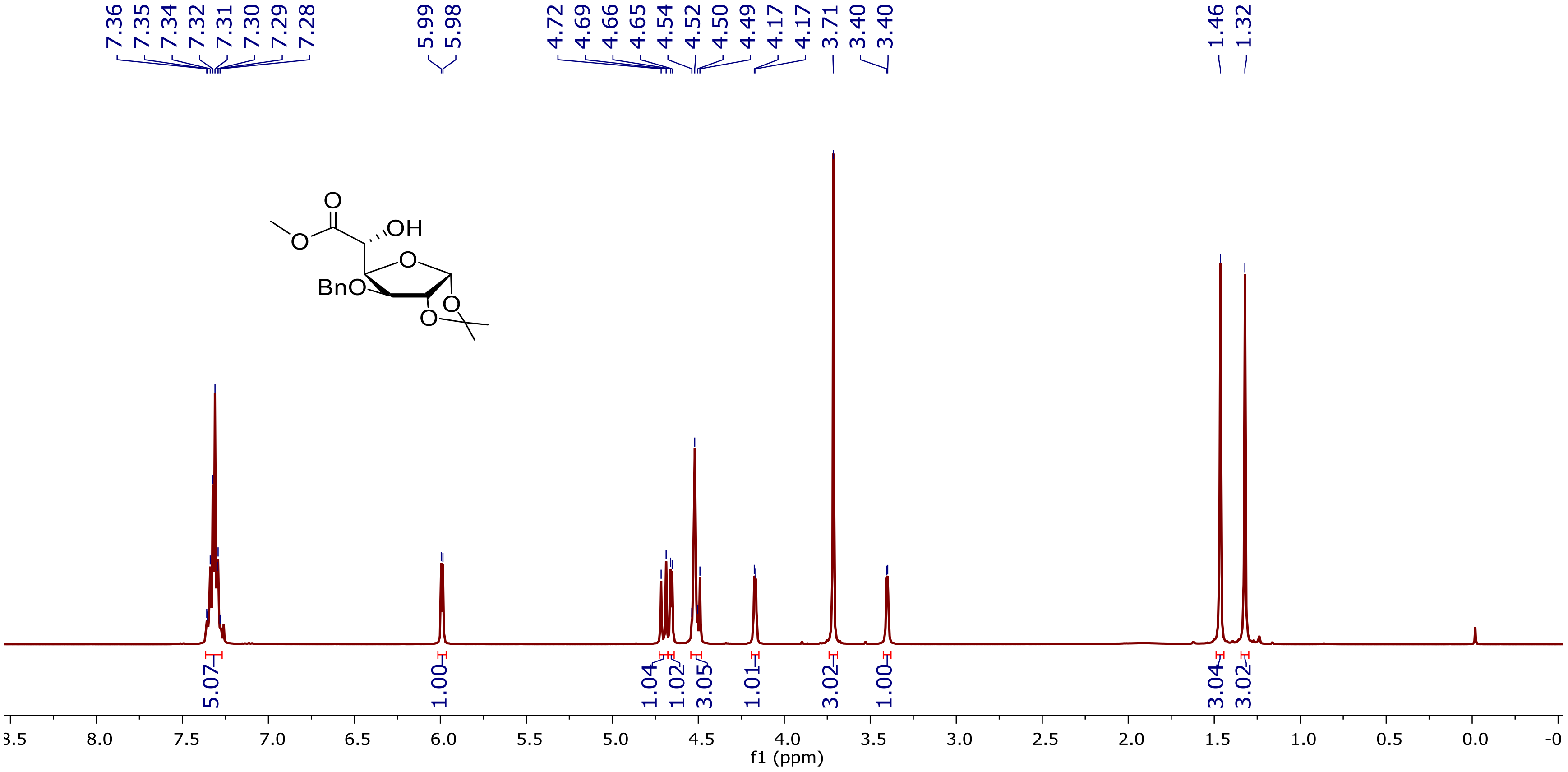

Supplementary Figure S17b. ^{13}C NMR Spectrum (100.53 MHz, $CDCl_3$) of Compound 21

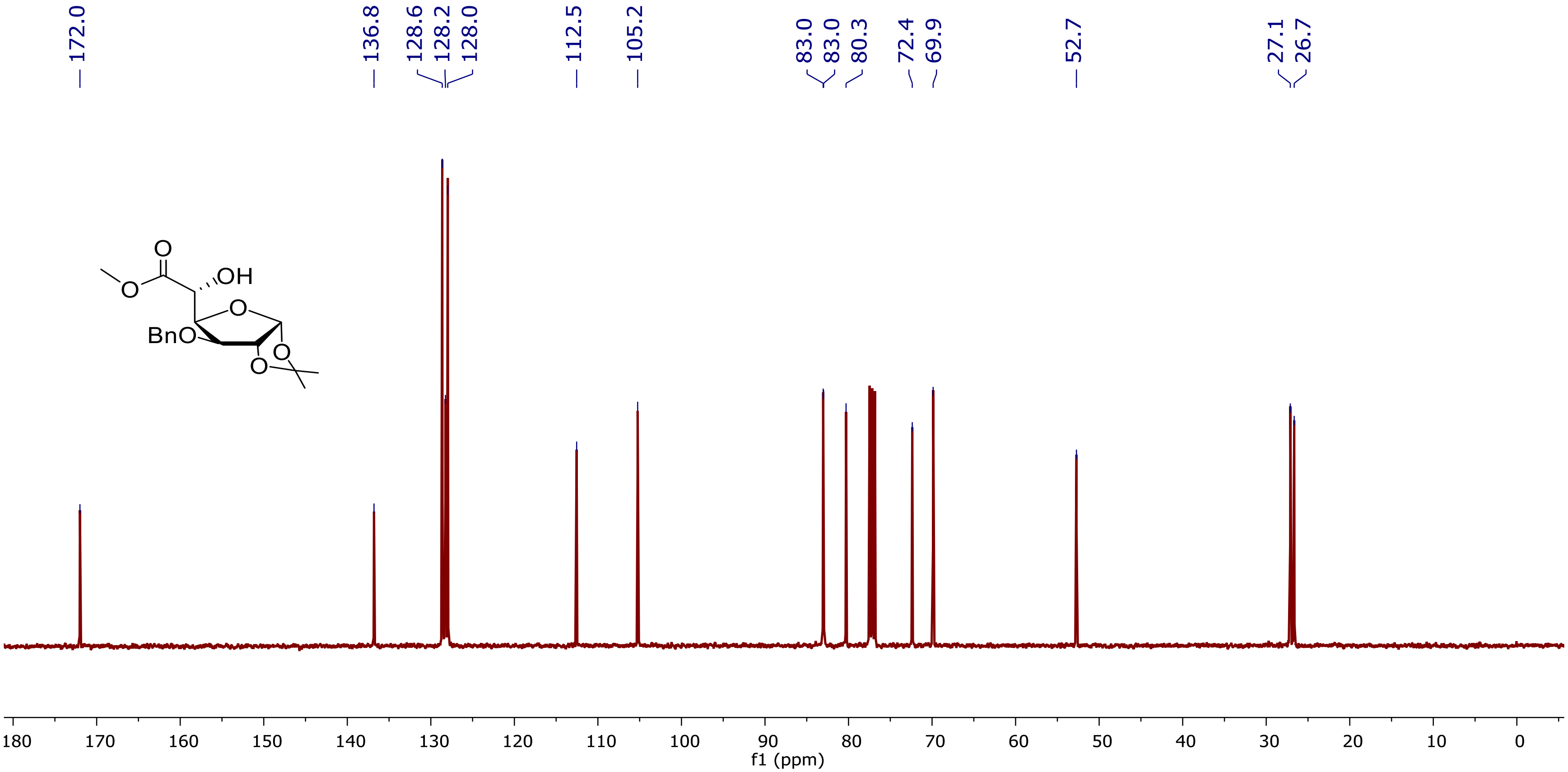

Supplementary Figure S17c. DEPT NMR Spectrum (100.53 MHz, CDCl$_3$) of Compound 21

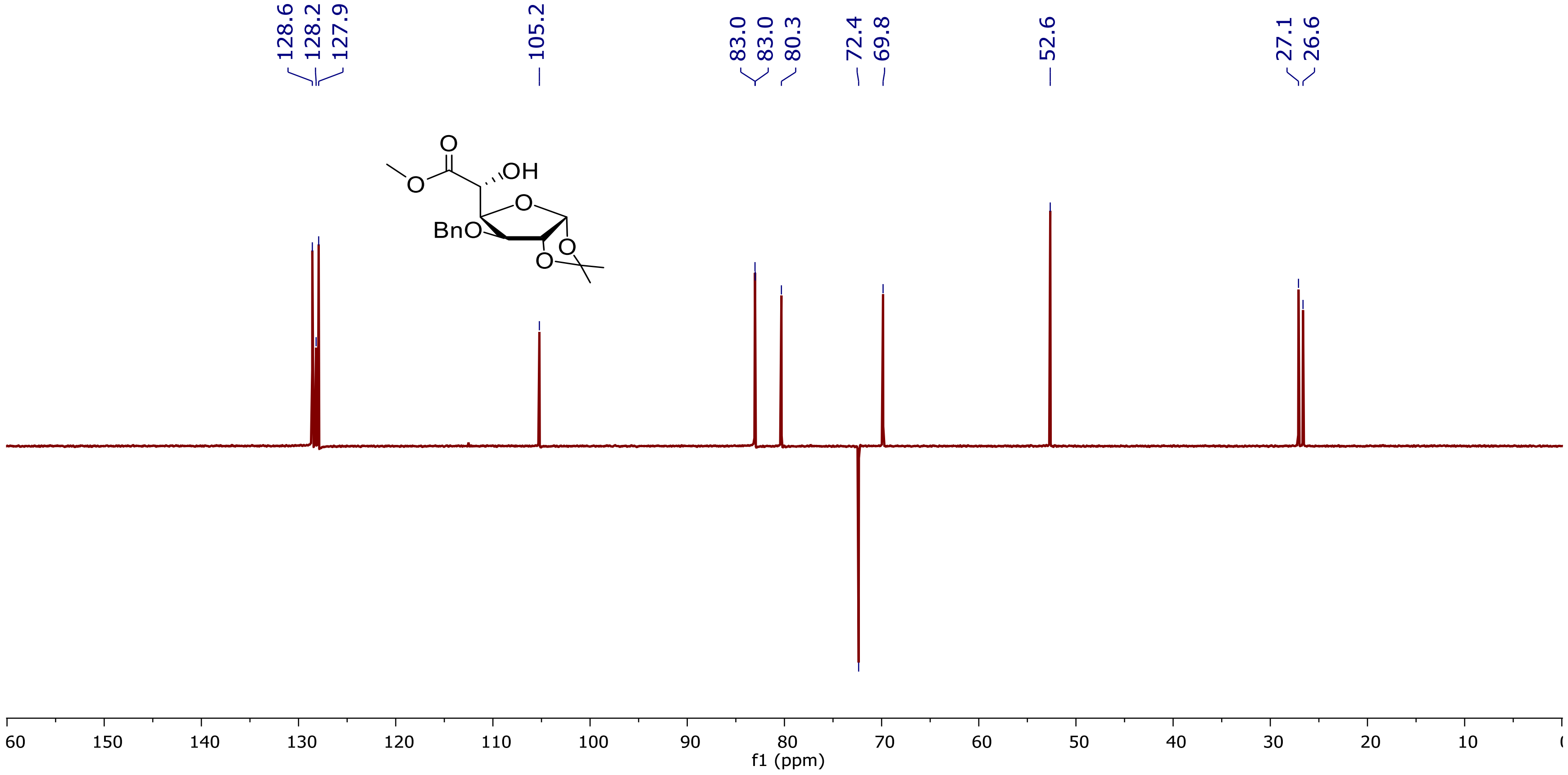

Supplementary Figure S18a. ^{1}H NMR Spectrum (400.31 MHz, $CDCl_3$) of Compound 5

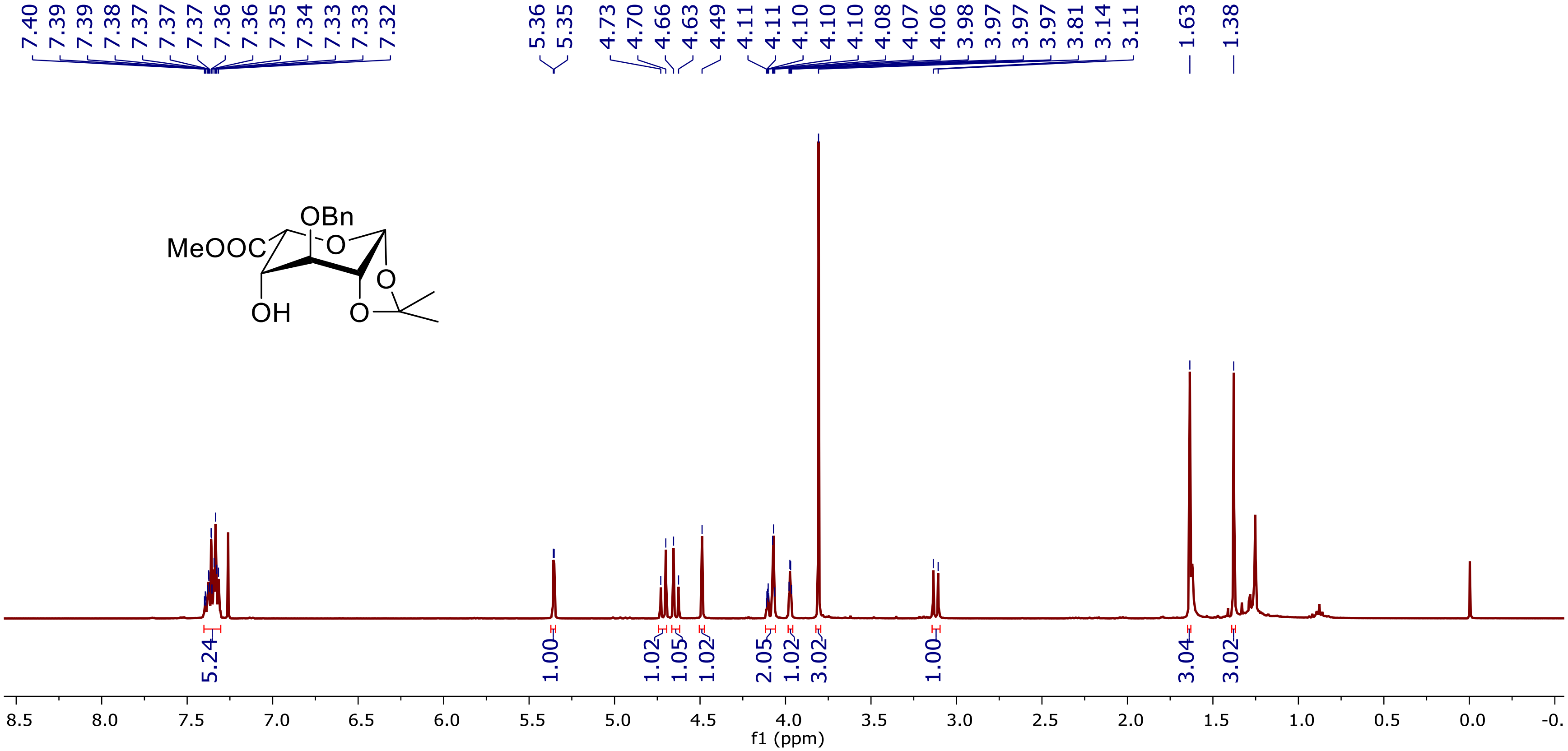

Supplementary Figure S18b. ^{13}C NMR Spectrum (100.67 MHz, $CDCl_3$) of Compound 5

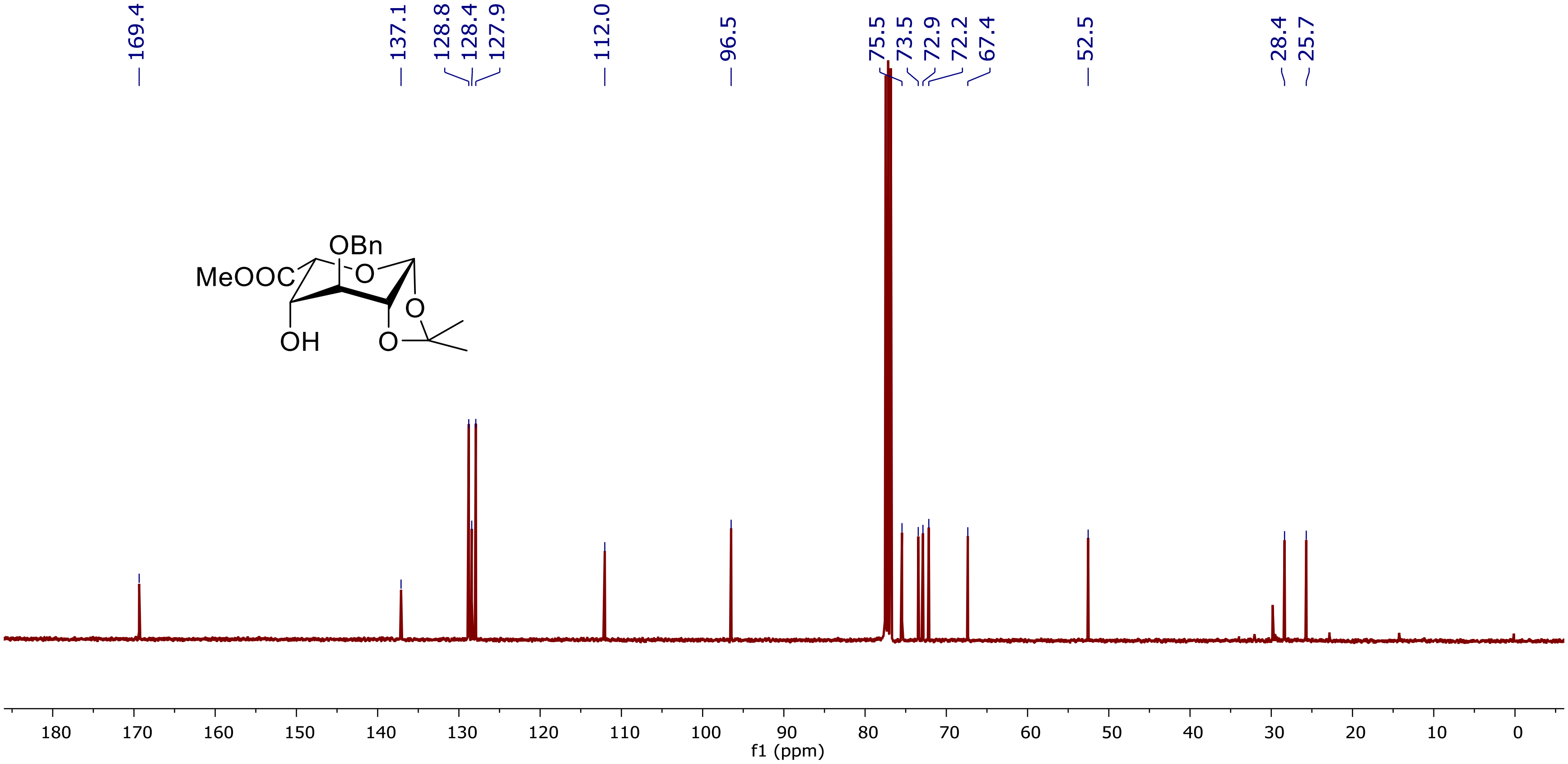

Supplementary Figure S18c. DEPT NMR Spectrum (100.67 MHz, $CDCl_3$) of Compound 5

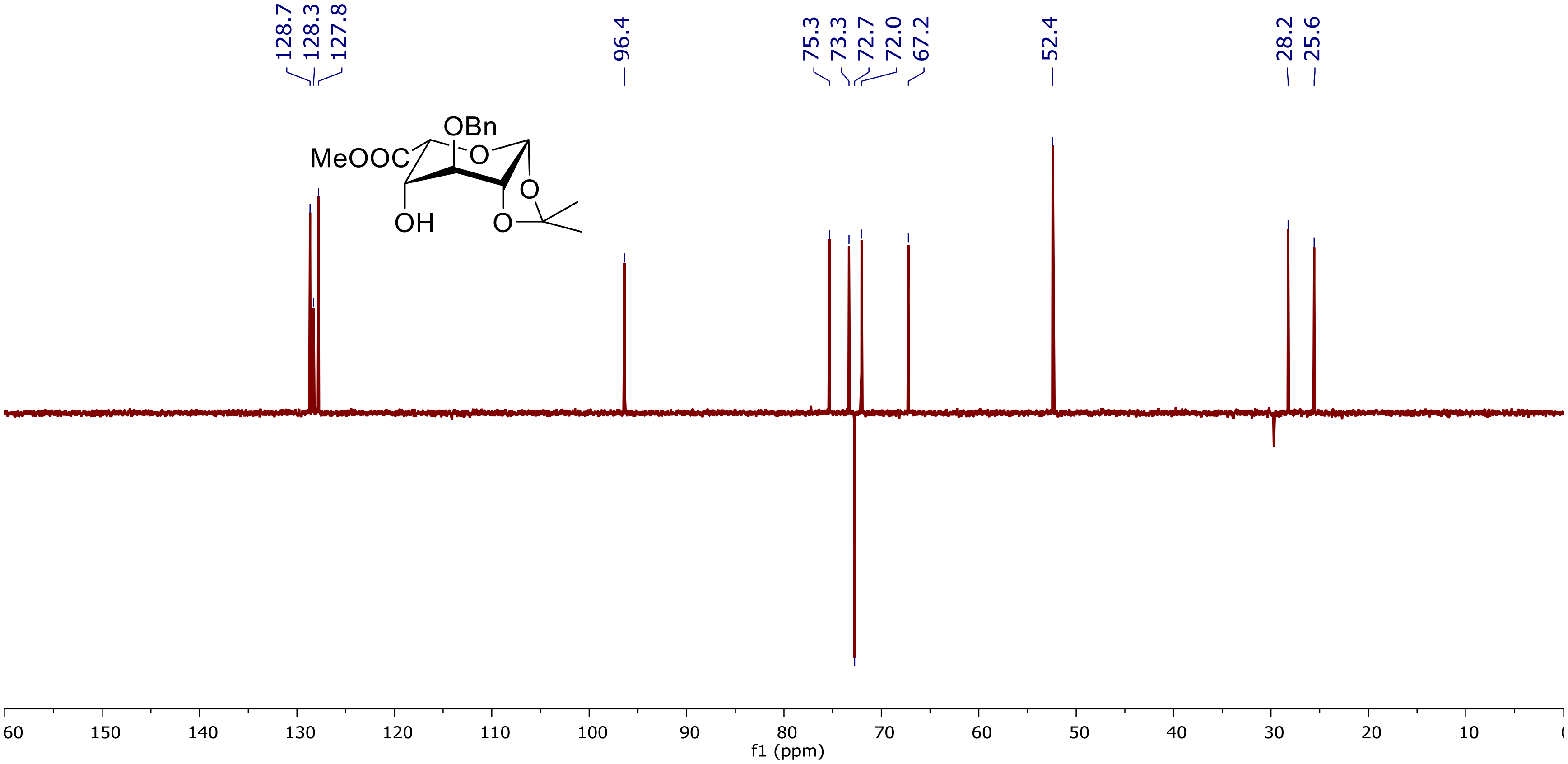

Supplementary Figure S19a. ^{1}H NMR Spectrum (400.31 MHz, $CDCl_3$) of Compound 23

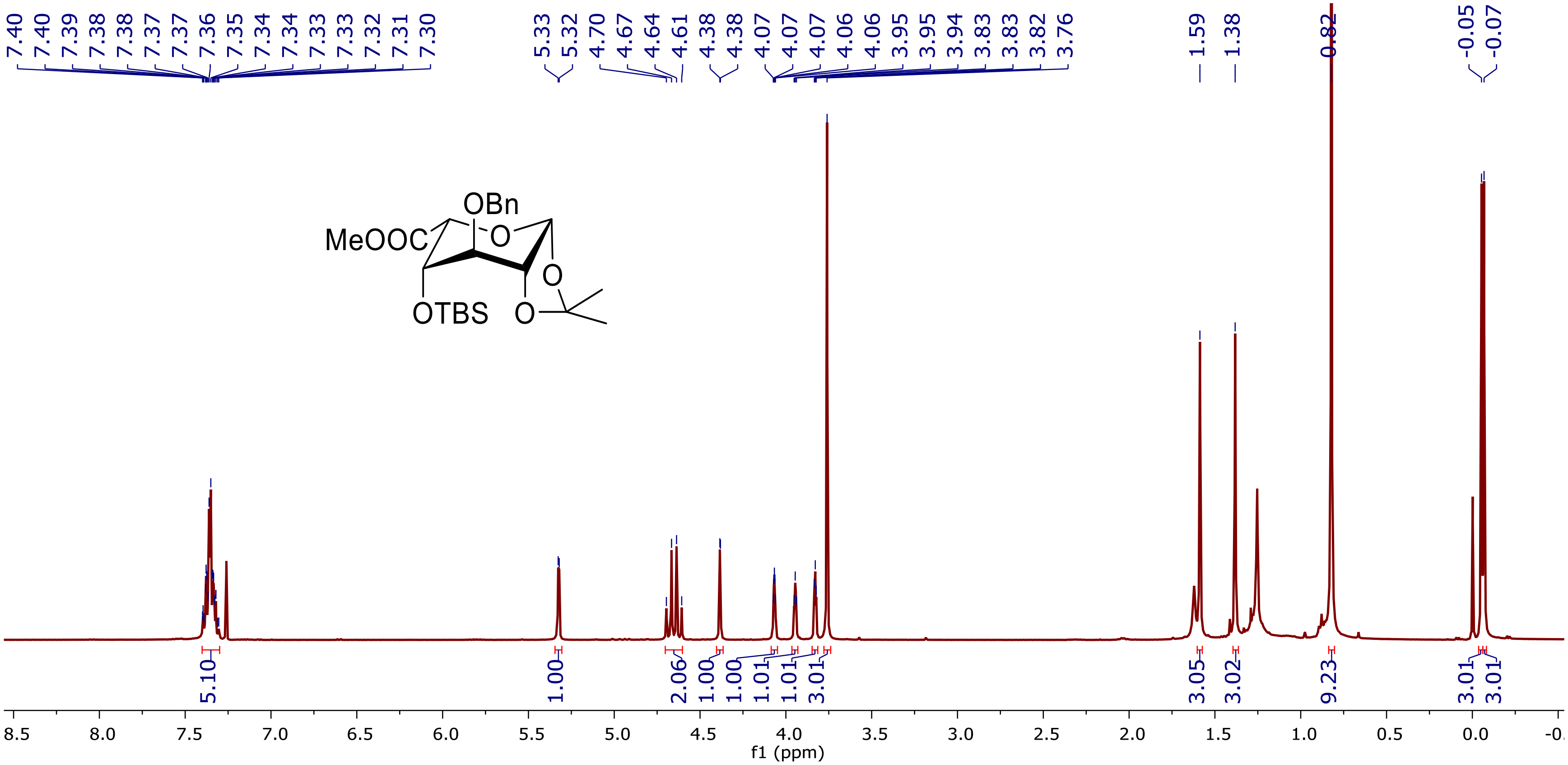

Supplementary Figure S19b. ^{13}C NMR Spectrum (100.67 MHz, $CDCl_3$) of Compound 23

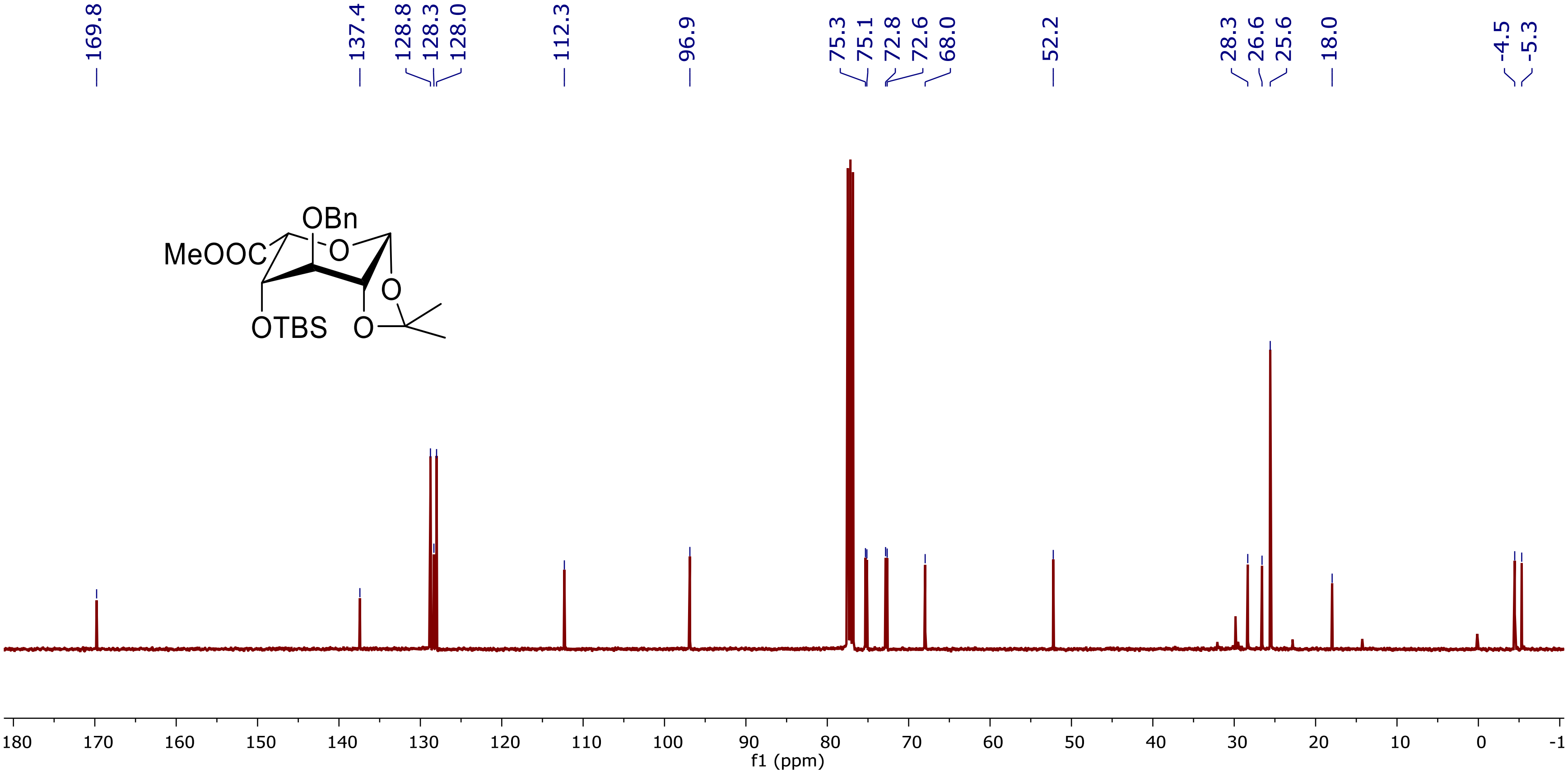

Supplementary Figure S19c. DEPT NMR Spectrum (100.67 MHz, $CDCl_3$) of Compound 23

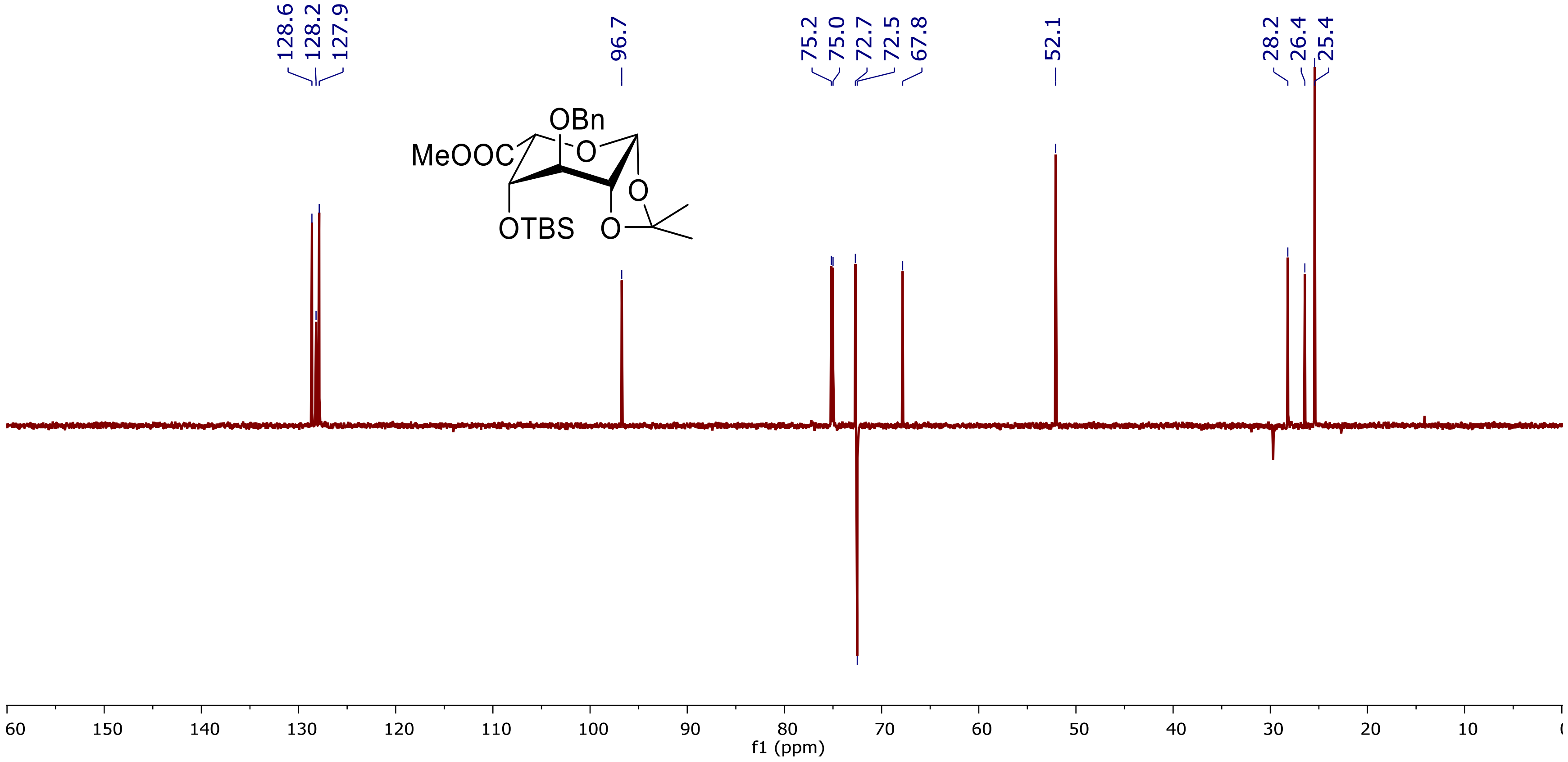

Supplementary Figure S20a. ^{1}H NMR Spectrum (400.31 MHz, $CDCl_3$) of Compound S4

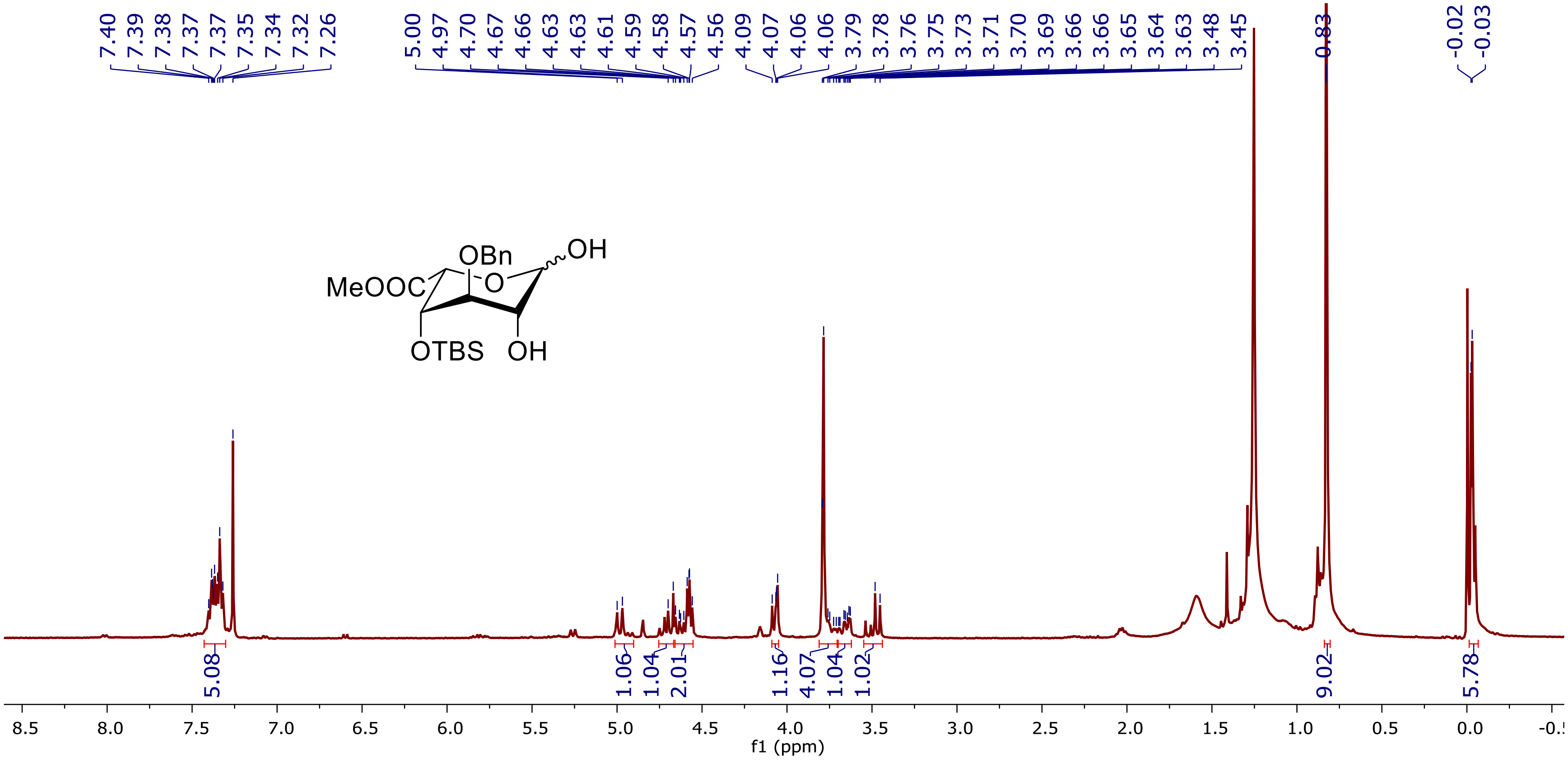

Supplementary Figure S20b. ^{13}C NMR Spectrum (100.67 MHz, $CDCl_3$) of Compound S4

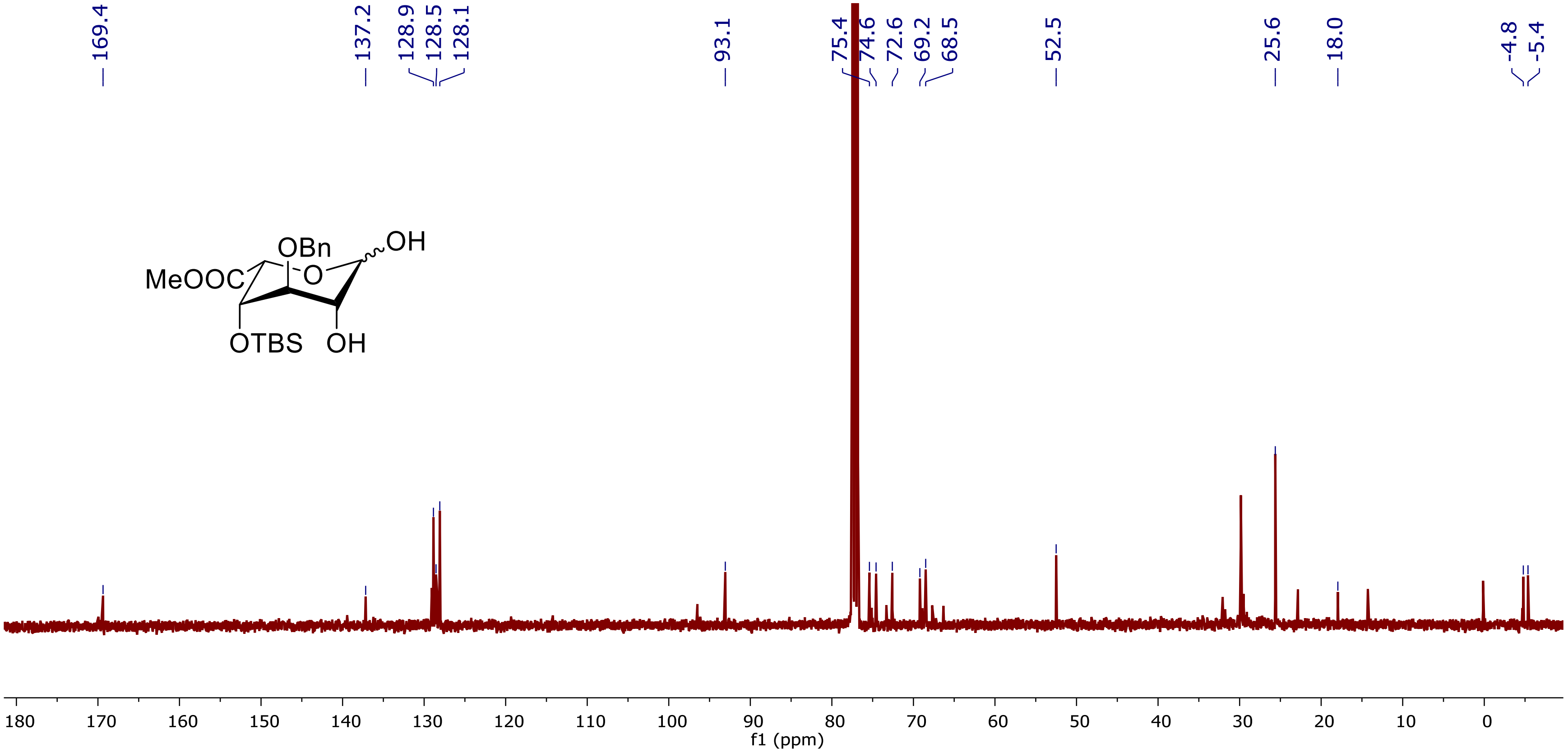

Supplementary Figure S20c. DEPT NMR Spectrum (100.67 MHz, $CDCl_3$) of Compound S4

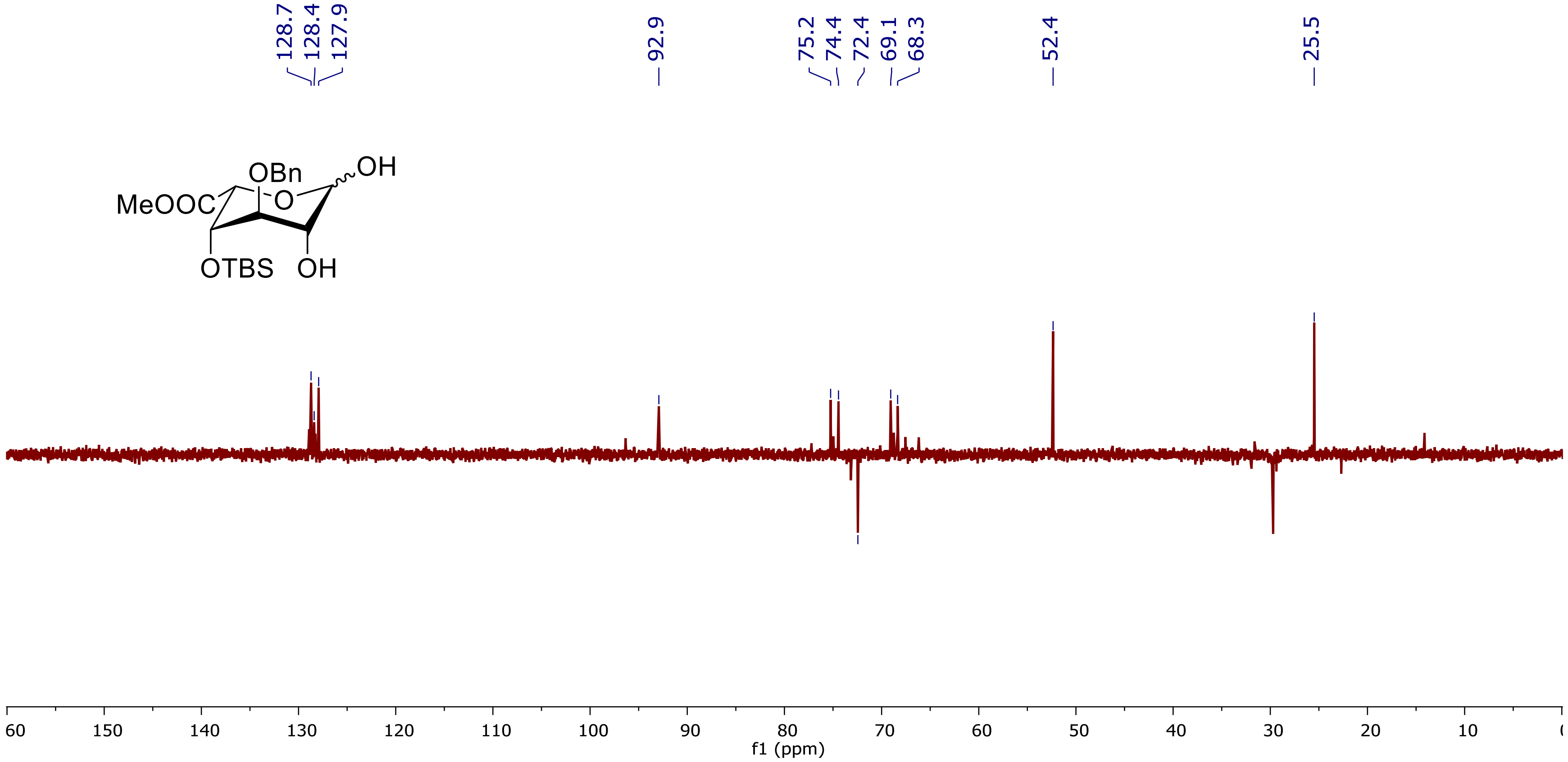

Supplementary Figure S21a. ^{1}H NMR Spectrum (399.78 MHz, $CDCl_3$) of Compound 10

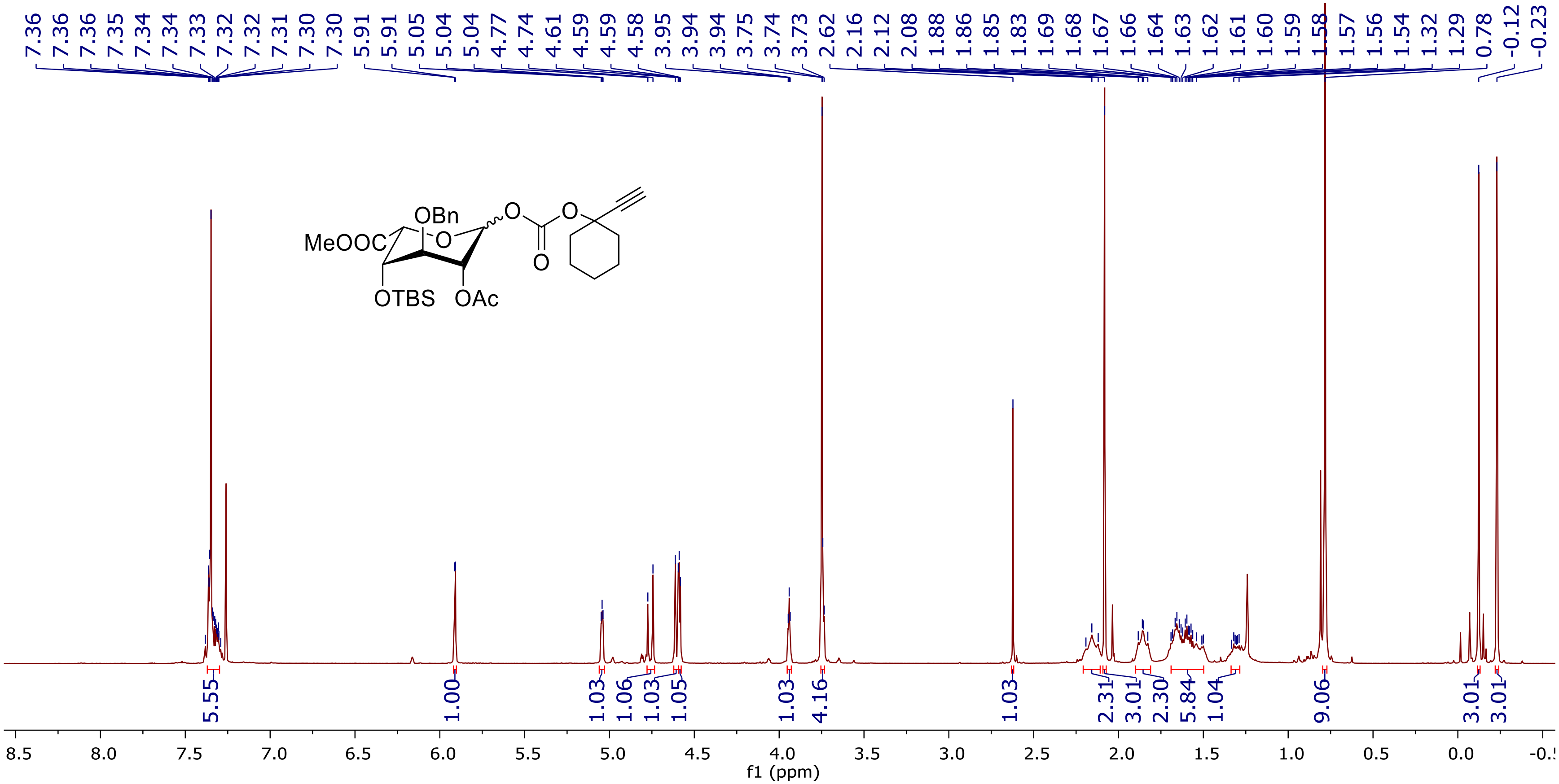

Supplementary Figure S21b. ^{13}C NMR Spectrum (100.67 MHz, $CDCl_3$) of Compound 10

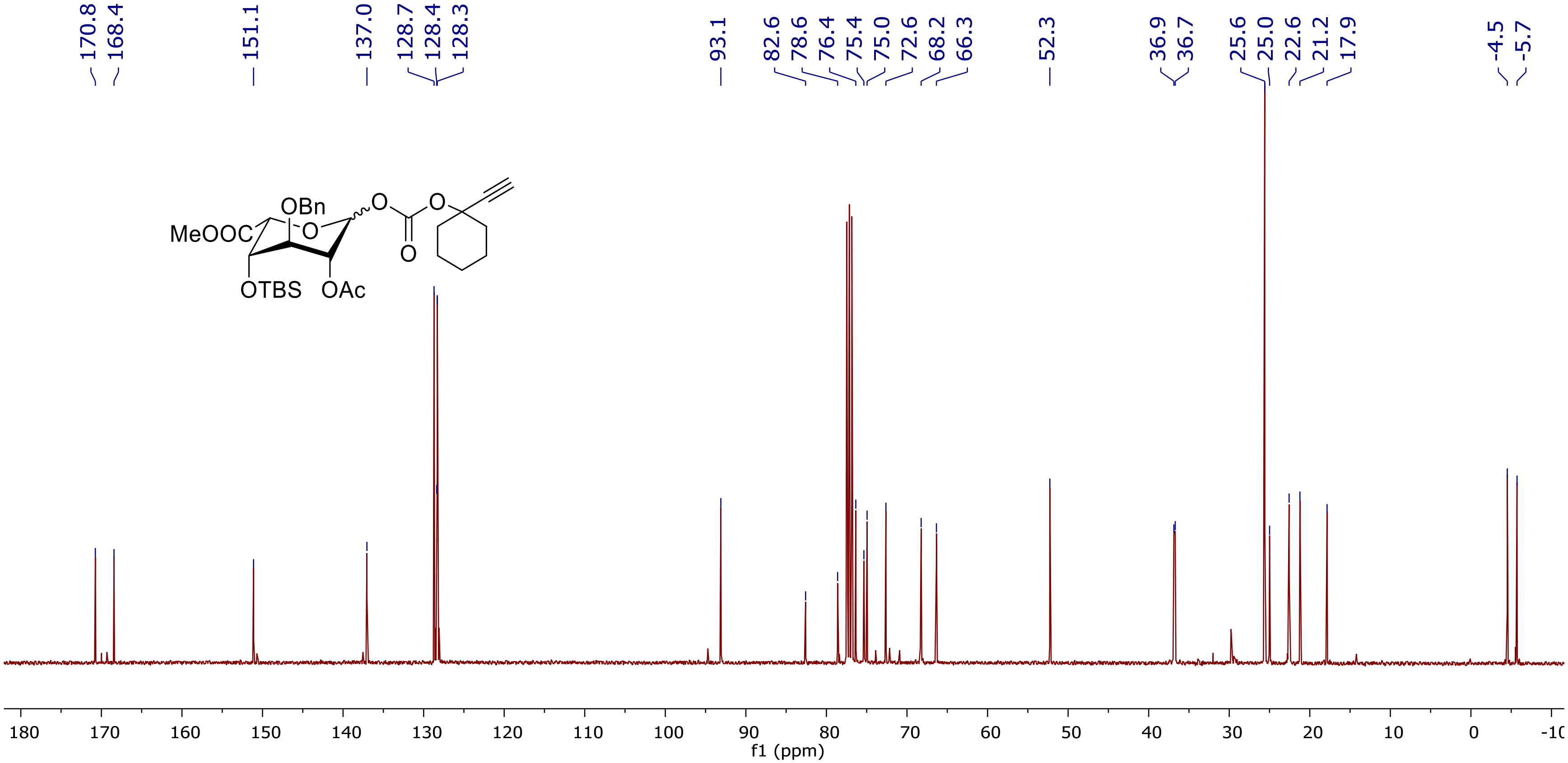

Supplementary Figure S21c. DEPT NMR Spectrum (100.67 MHz, $CDCl_3$) of Compound 10

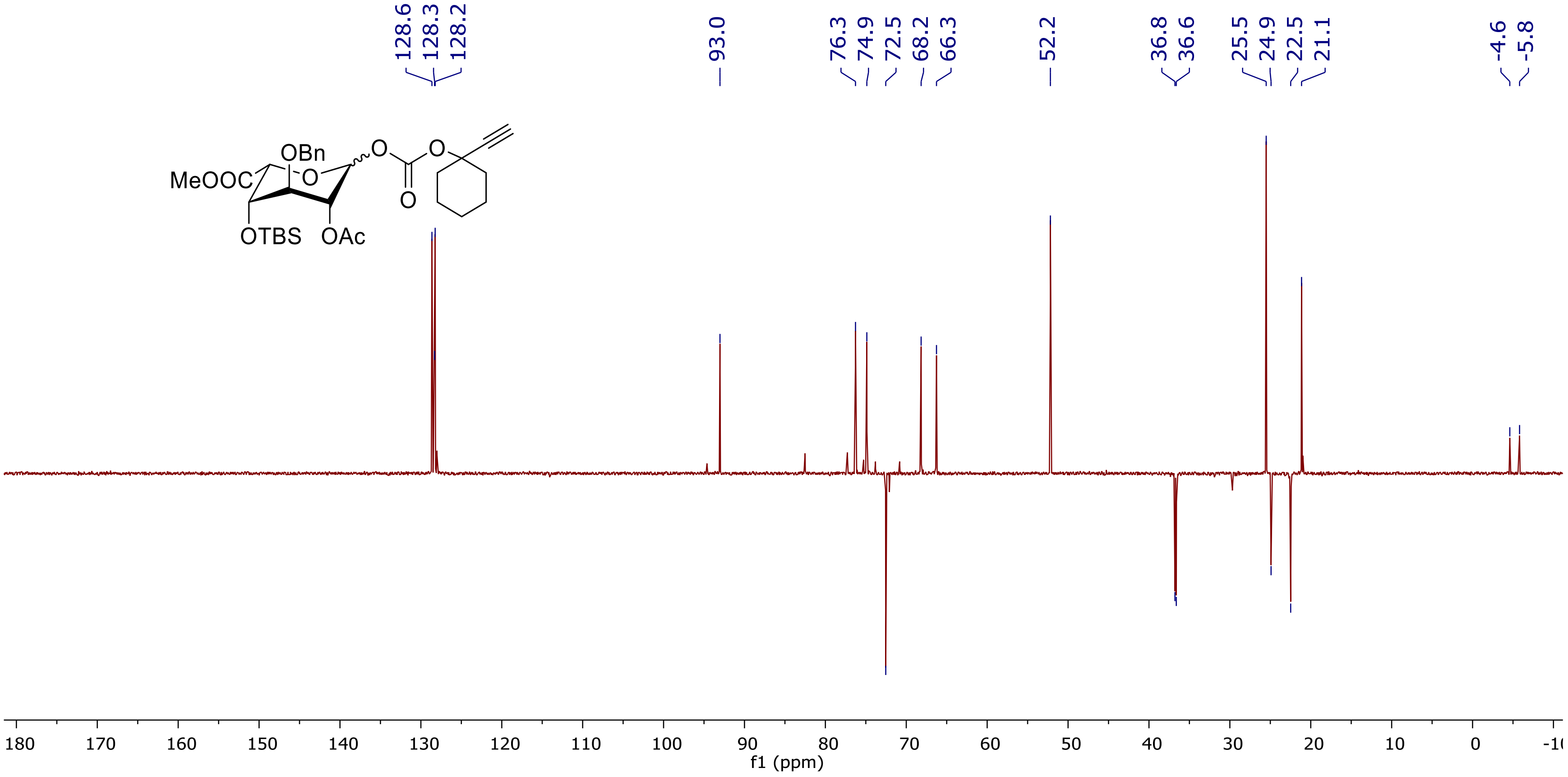

Supplementary Figure S22a. ^{1}H NMR Spectrum (400.31 MHz, $CDCl_3$) of Compound S5

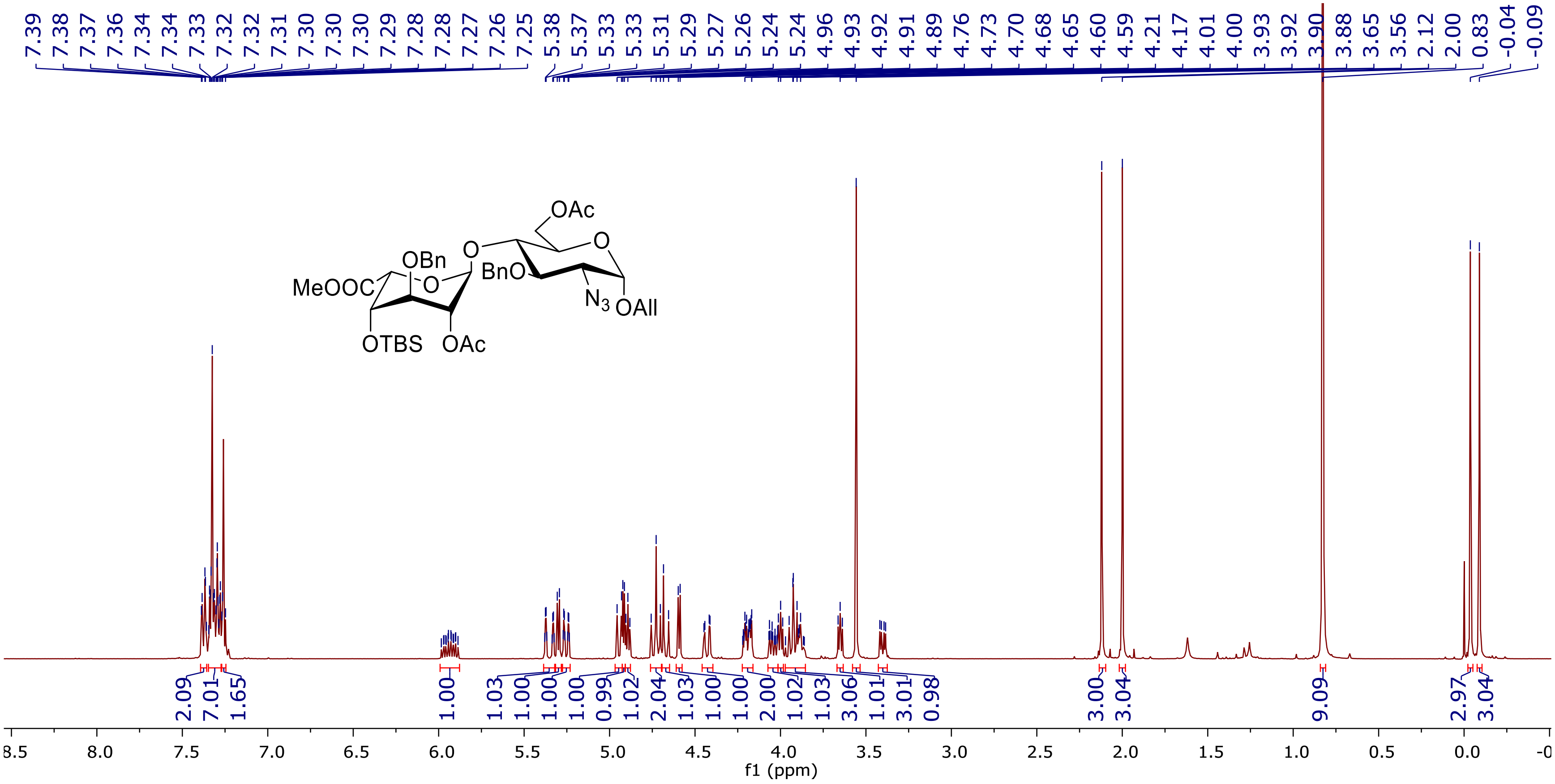

Supplementary Figure S22b. ^{13}C NMR Spectrum (100.67 MHz, $CDCl_3$) of Compound S5

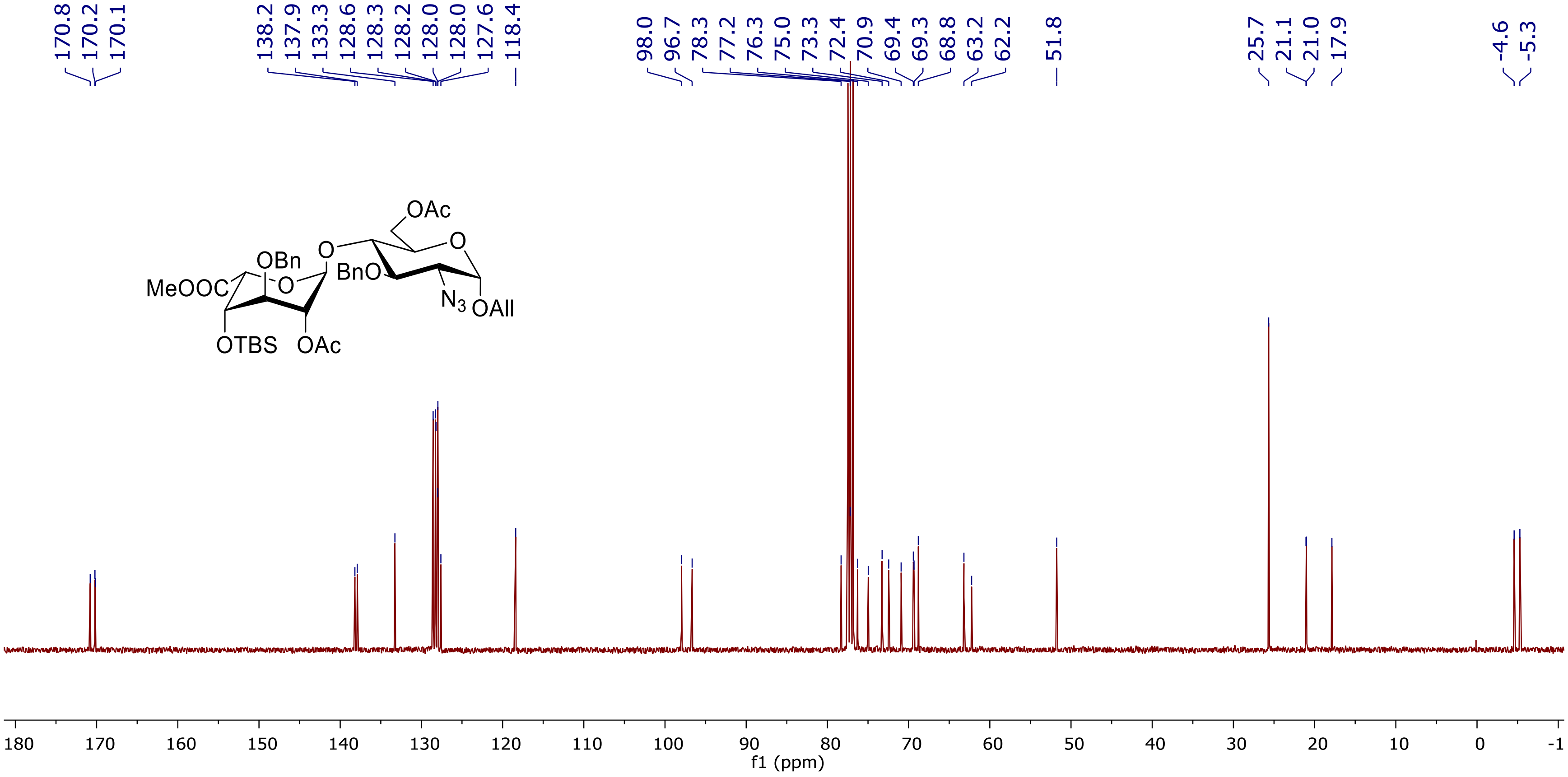

Supplementary Figure S22c. DEPT NMR Spectrum (100.67 MHz, $CDCl_3$) of Compound S5

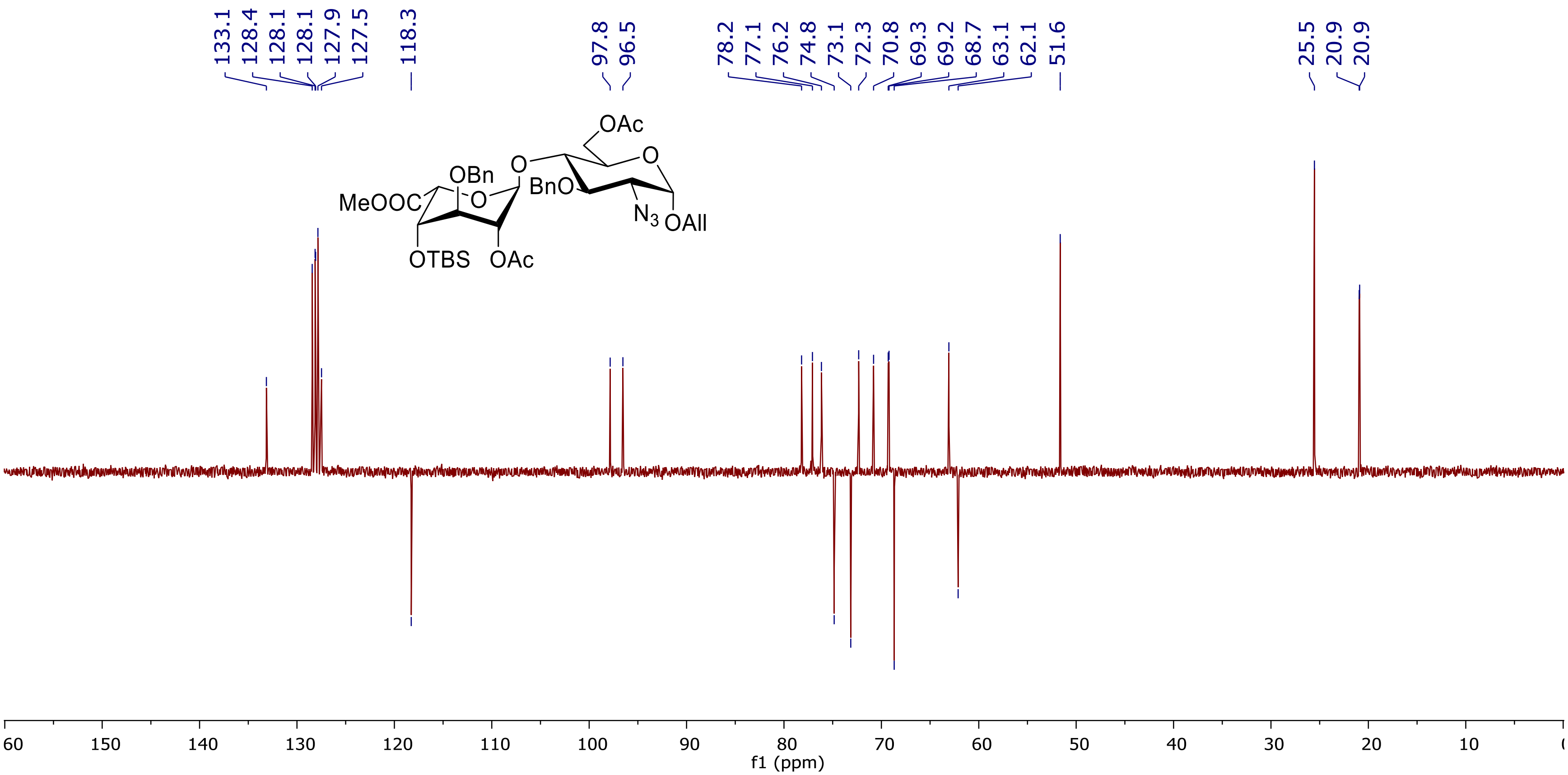

Supplementary Figure S22d. COSY NMR Spectrum (600.40, 600.40 MHz, $CDCl_3$) of Compound S5

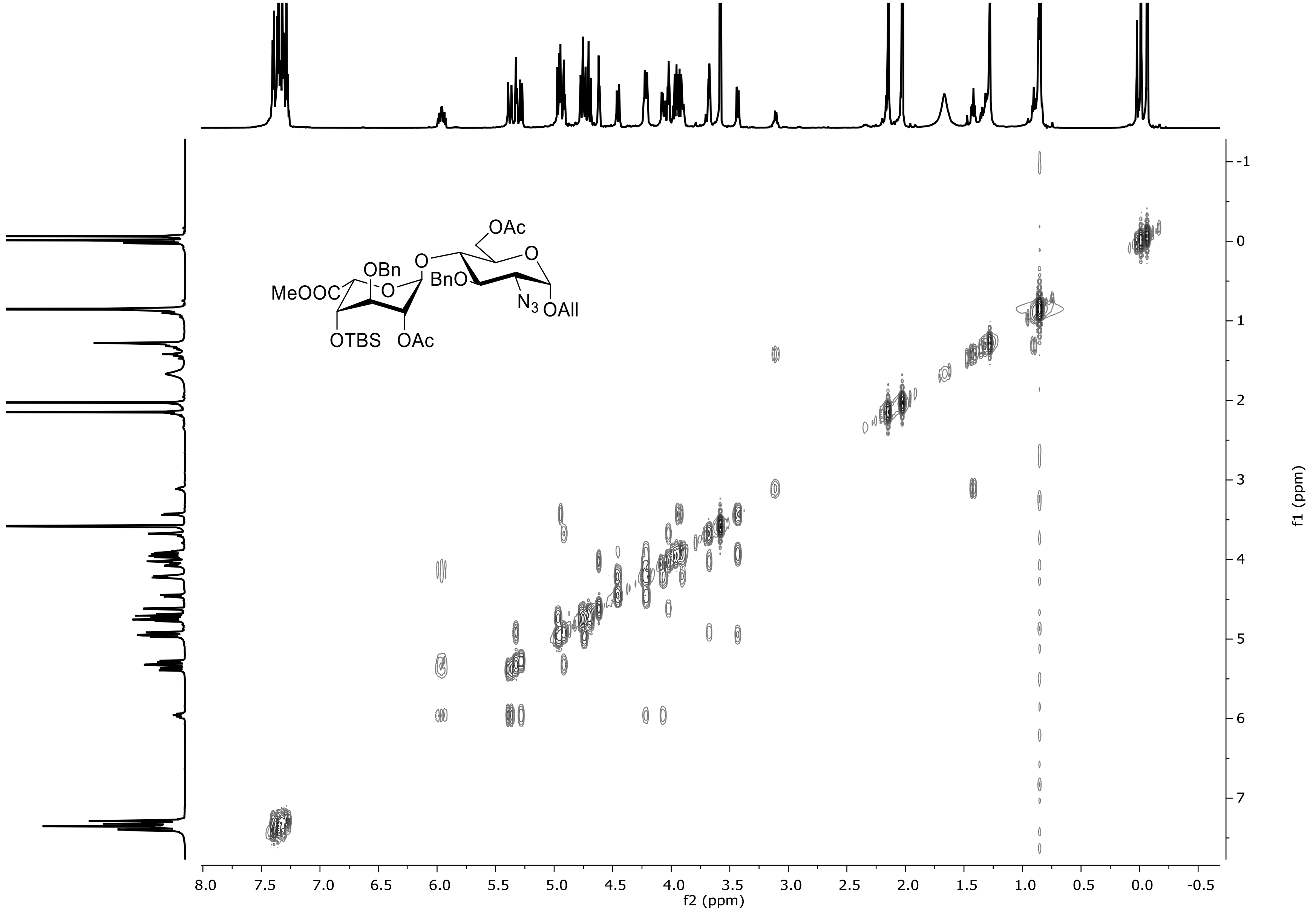

Supplementary Figure S22e. COSY NMR Spectrum (600.40, 600.40 MHz, $CDCl_3$) of Compound S5 (Sugar region expanded)

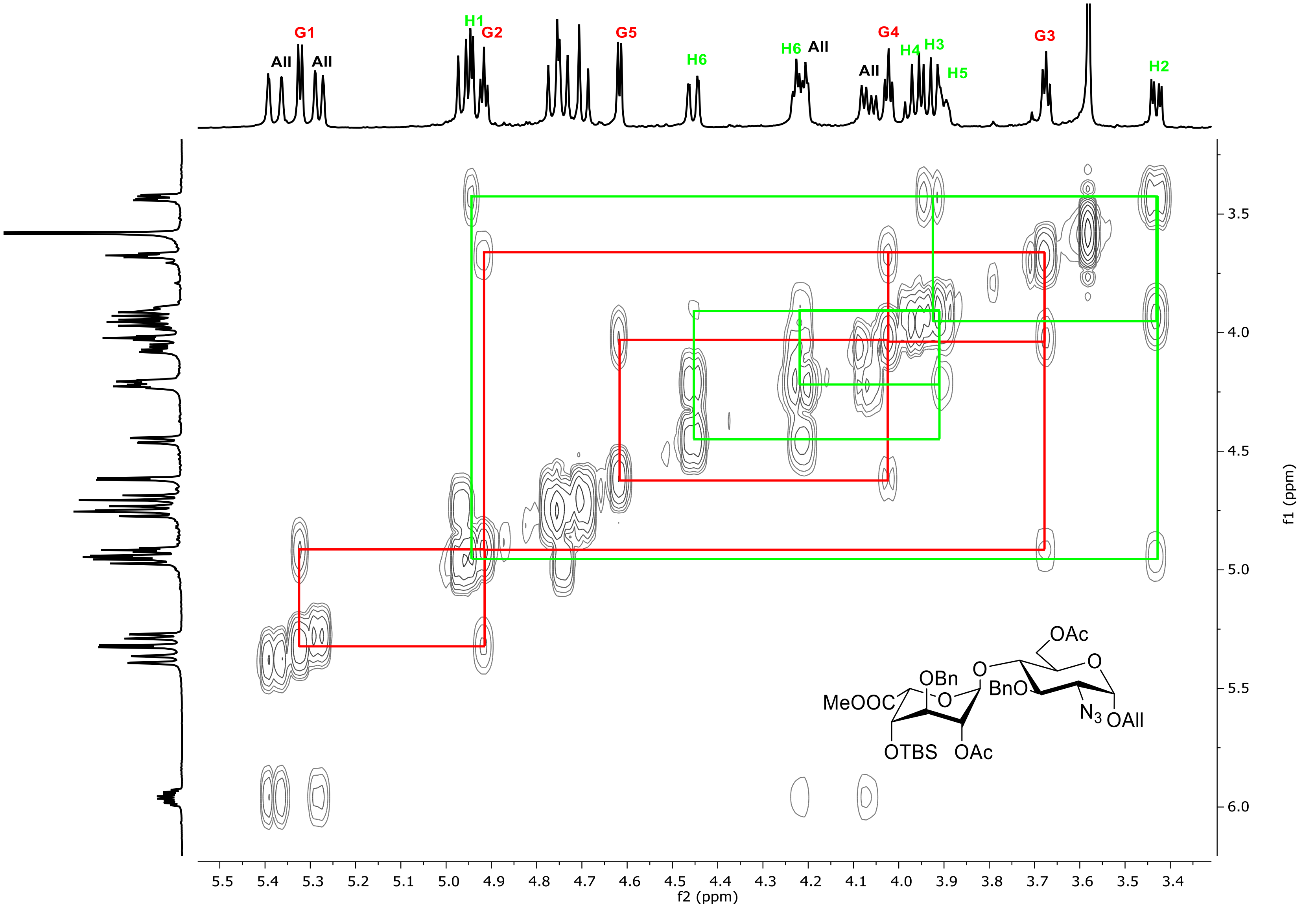

Supplementary Figure S22f. HSQC NMR Spectrum (600.40, 150.99MHz, $CDCl_3$) of Compound S5

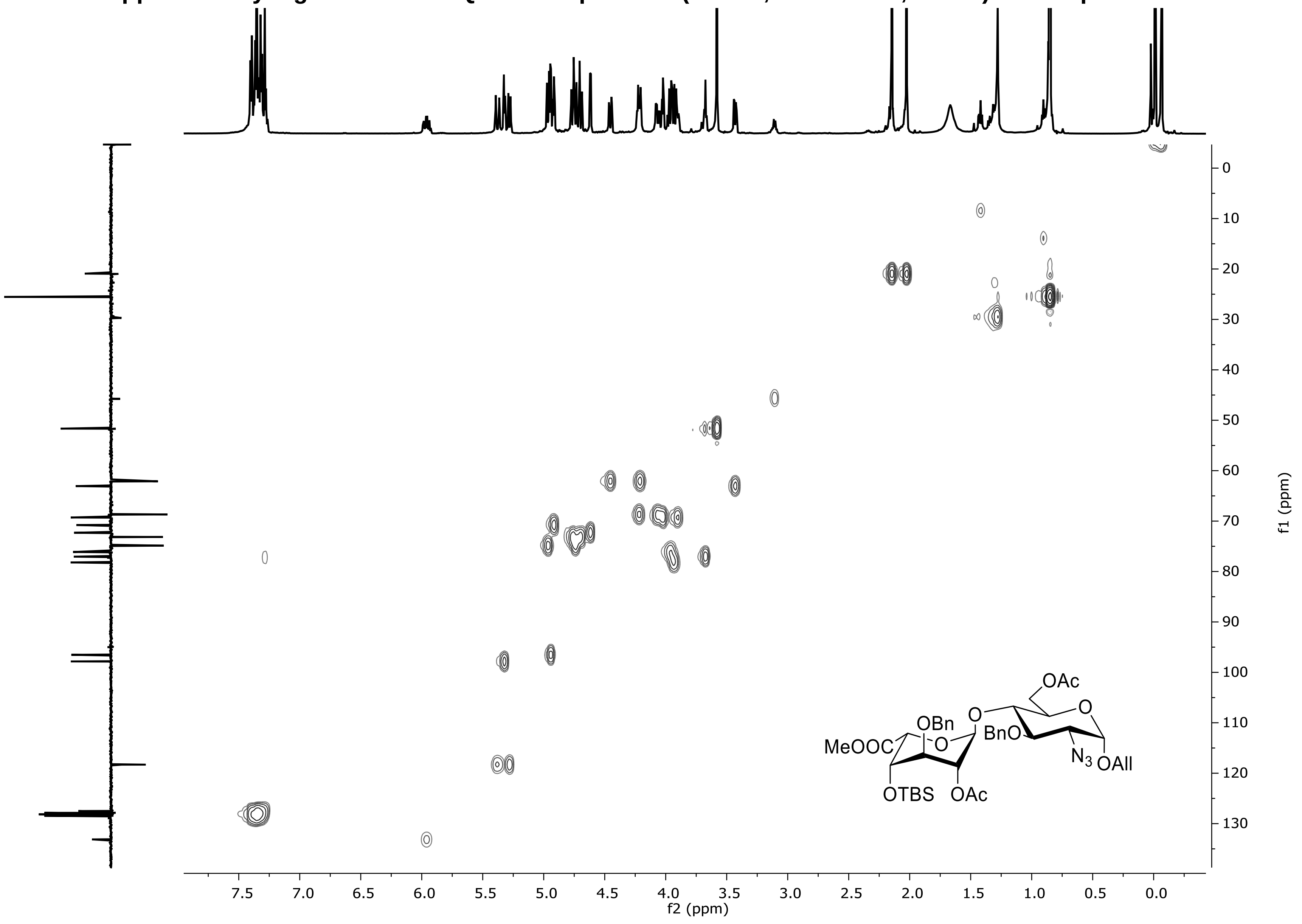

Supplementary Figure S22g. HSQC NMR Spectrum (600.40, 150.99 MHz, $CDCl_3$) of Compound S5 (Sugar region expanded)

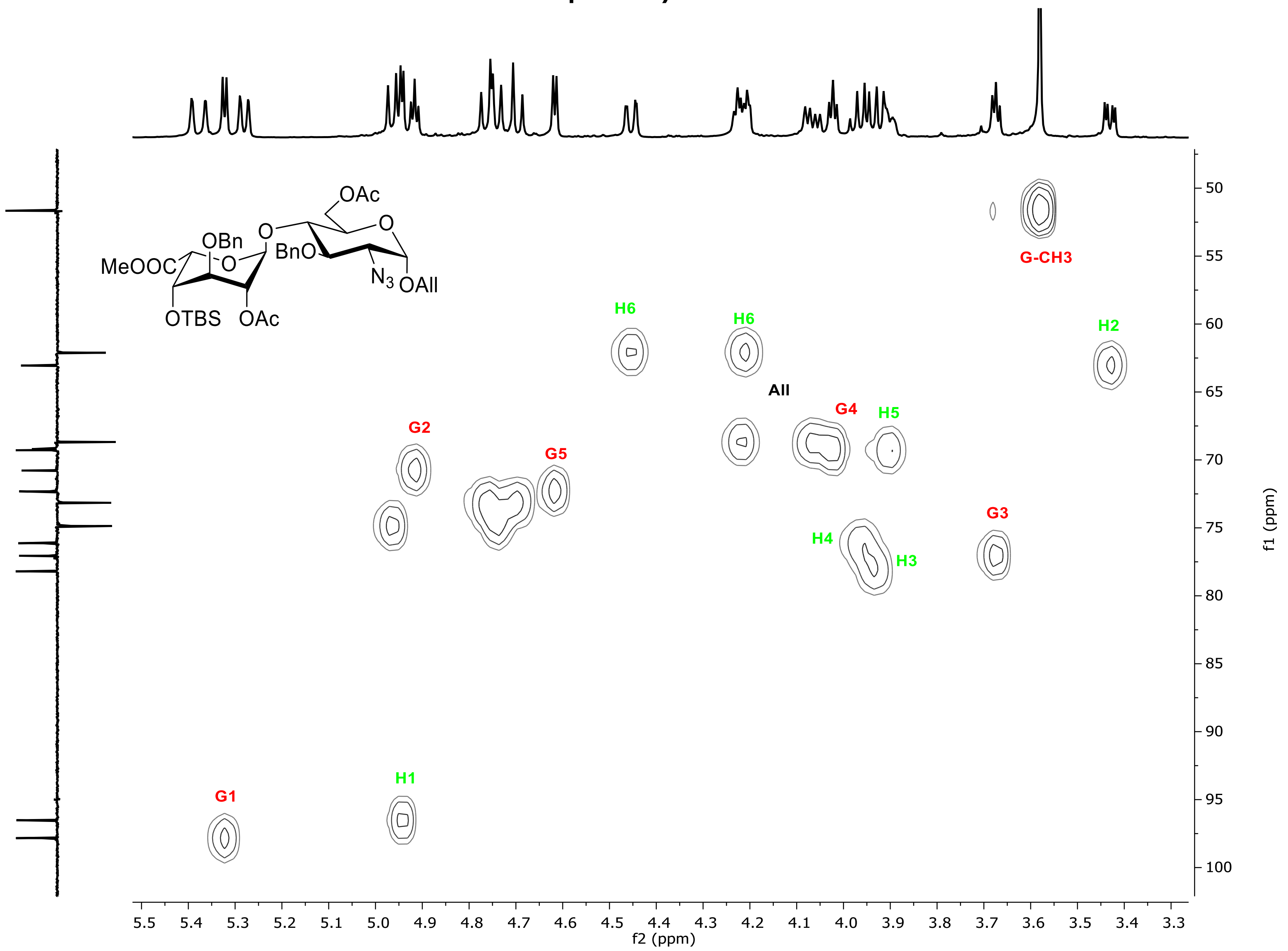

Supplementary Figure S22h. Coupled HSQC NMR Spectrum (600.40, 150.99 MHz, $CDCl_3$) of Compound S5

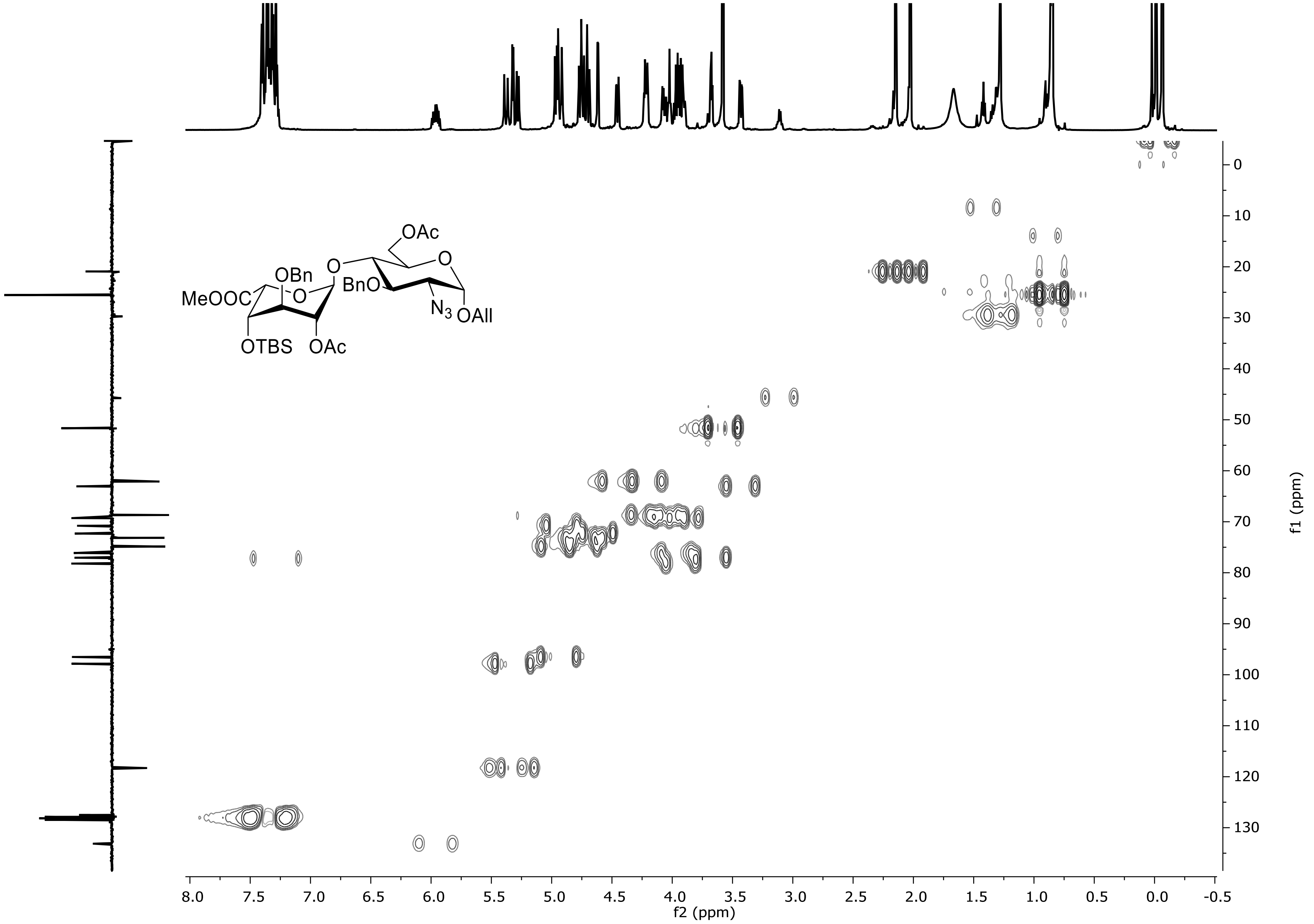

Supplementary Figure S22i. Coupled HSQC NMR Spectrum (600.40, 150.99 MHz, $CDCl_3$) of Compound GW-260-2D (Sugar region expanded)

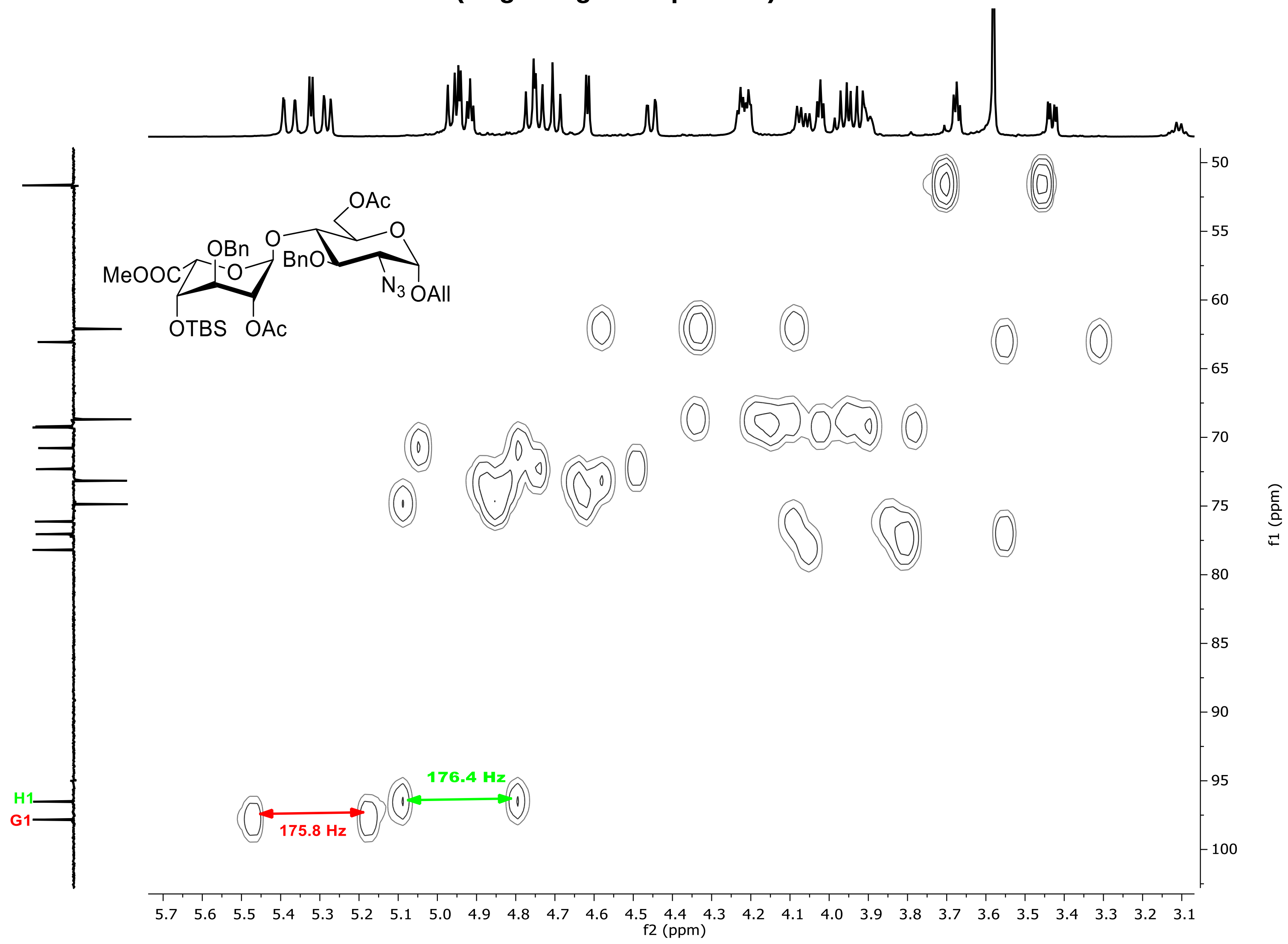

Supplementary Figure S22j. HMBC NMR Spectrum (600.40, 150.99 MHz, CDCl₃) of Compound S5

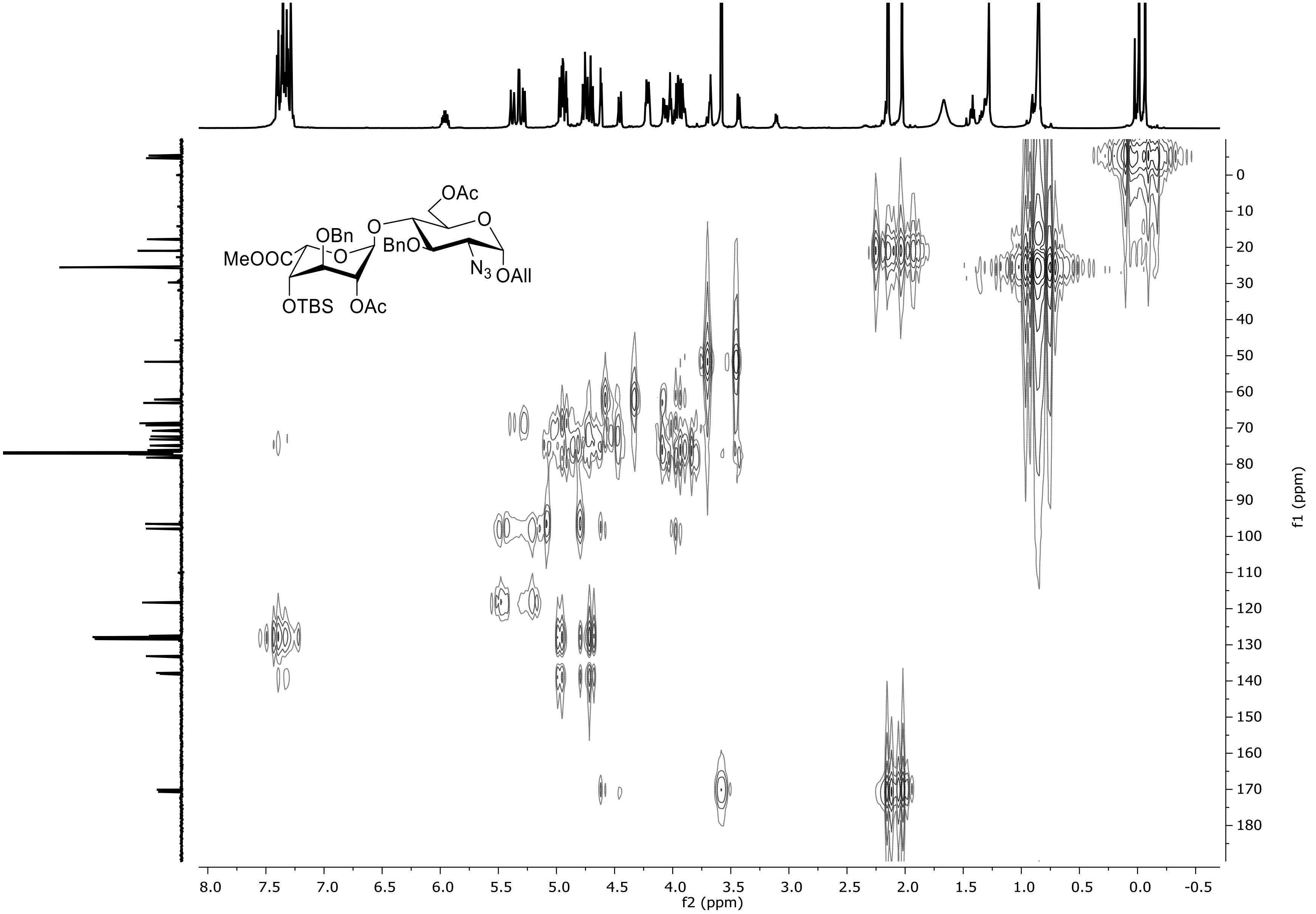

Supplementary Figure S22k. HMBC Spectrum (600.40, 150.99 MHz, $CDCl_3$) of Compound S5 (Sugar region expanded)

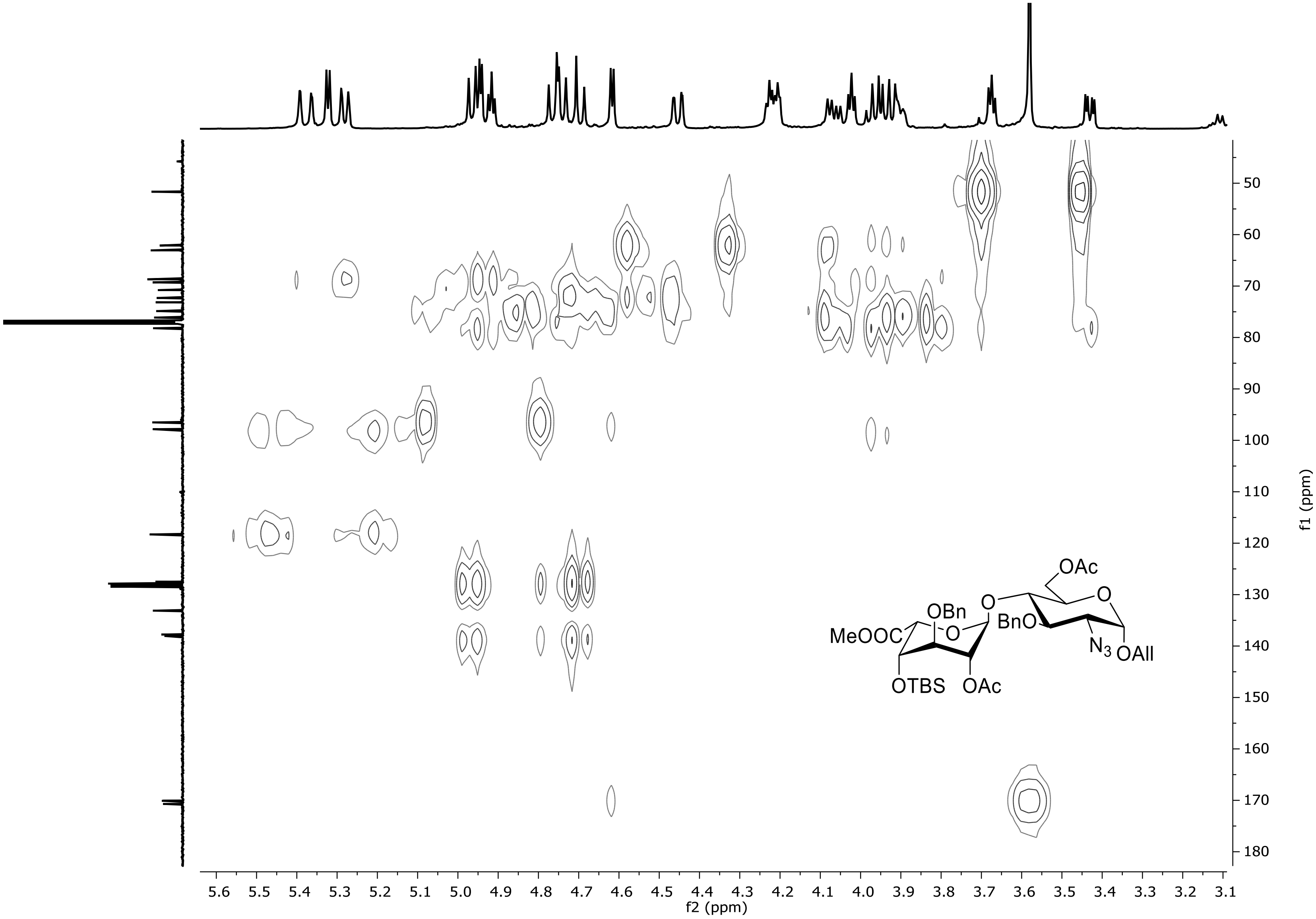

Supplementary Figure S22l. HMBC NMR Spectrum (600.40, 150.99 MHz, $CDCl_3$) of Compound S5 (Carbonyl region expanded)

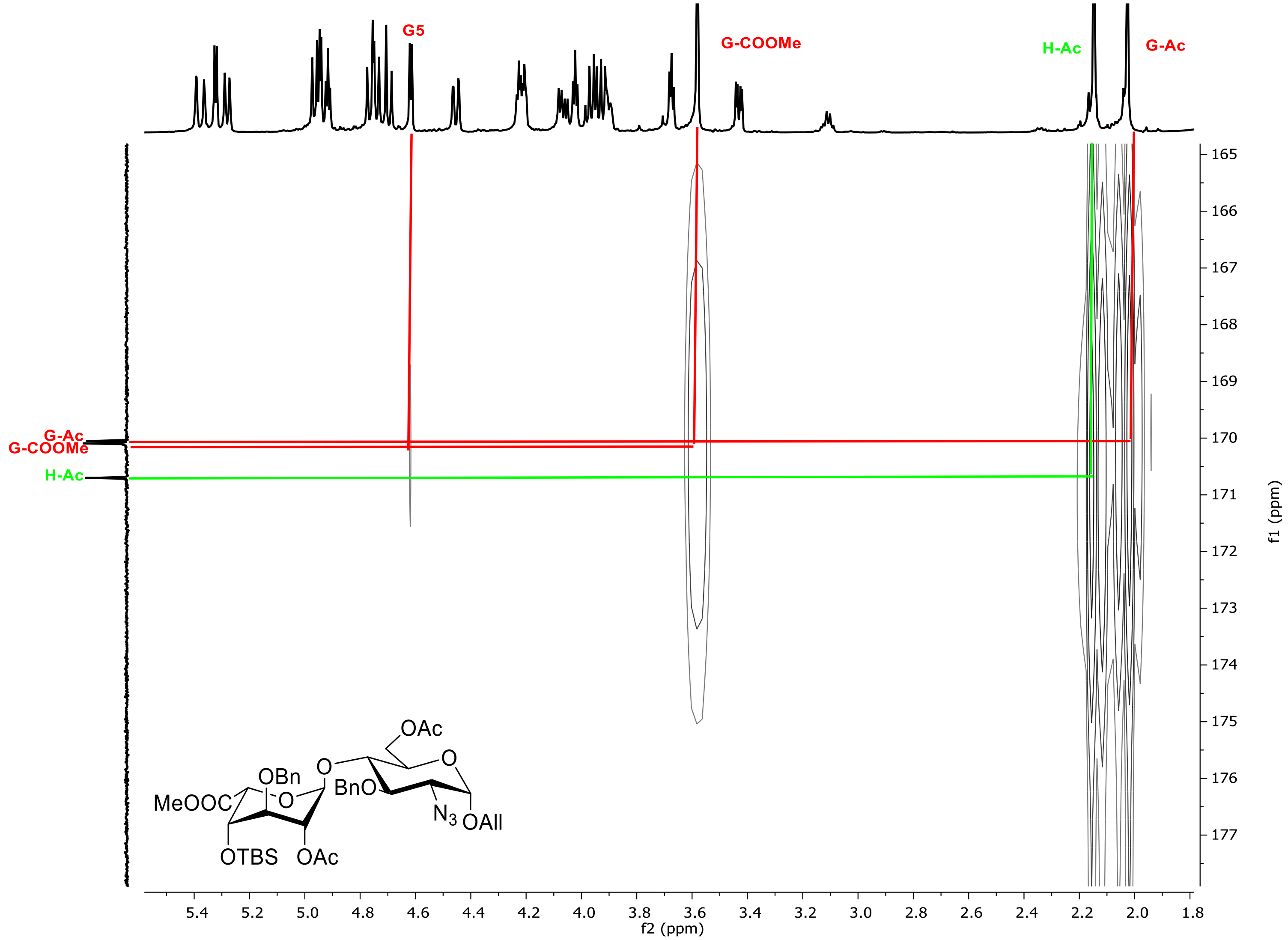

Supplementary Figure S22m. TOCSY NMR Spectrum (600.40, 600.40 MHz, $CDCl_3$) of Compound S5

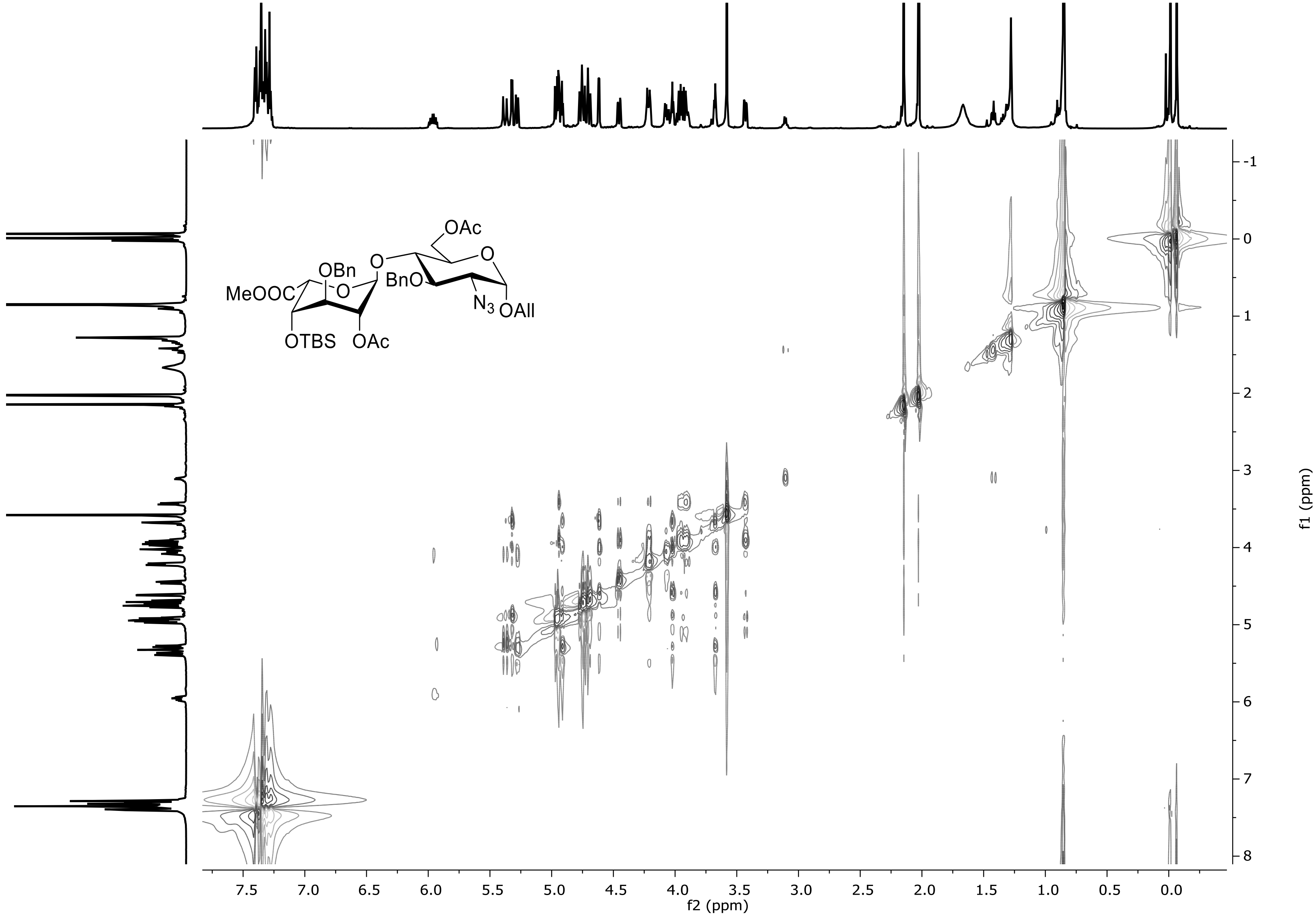

Supplementary Figure S22n. TOCSY NMR Spectrum (600.40, 600.40 MHz, $CDCl_3$) of Compound S5 (Sugar region expanded)

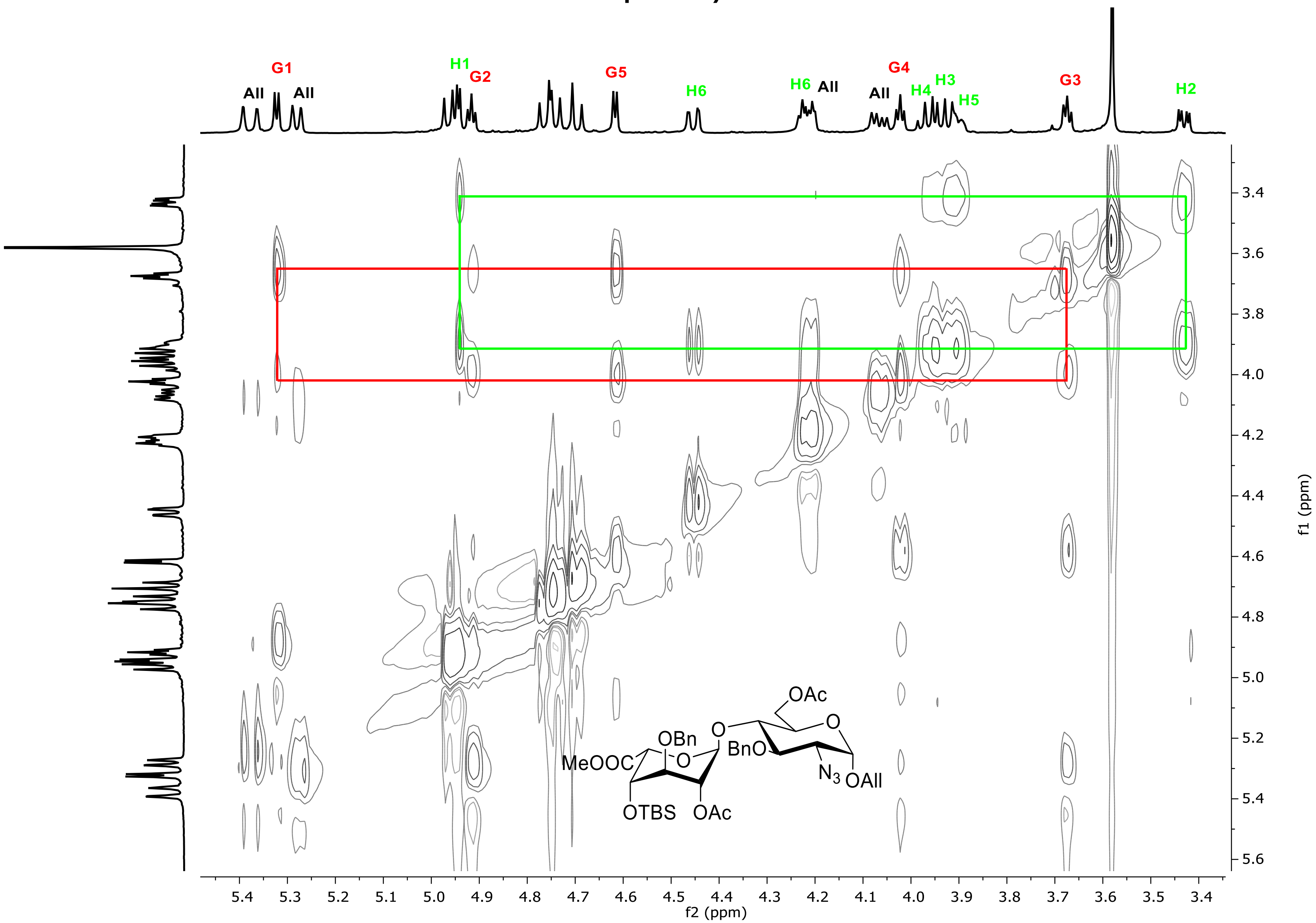

Supplementary Figure S22o. HSQC-TOCSY NMR Spectrum (600.40, 150.99 MHz, $CDCl_3$) of Compound S5

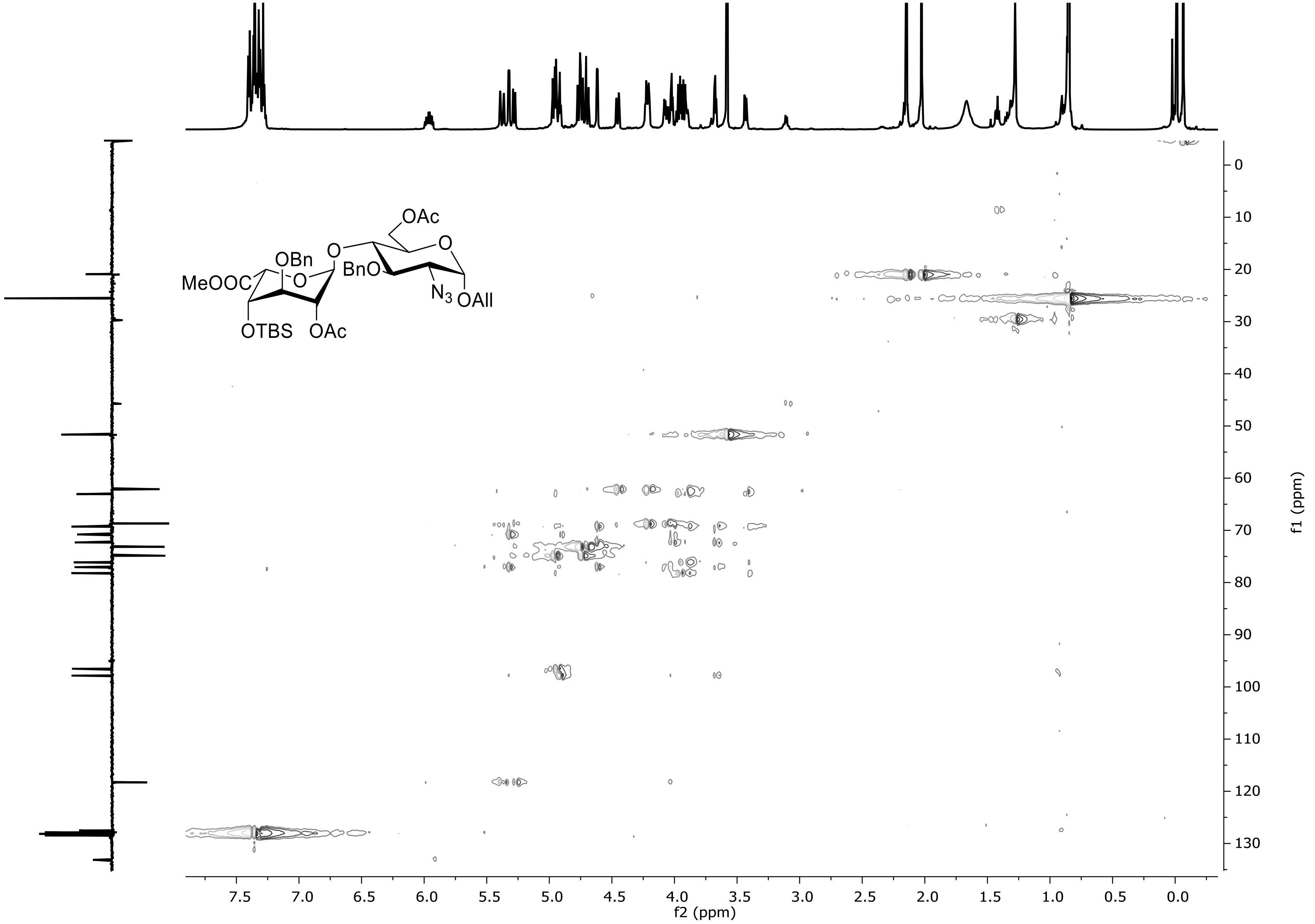

Supplementary Figure S22p. HSQC-TOCSY NMR Spectrum (600.40, 150.99 MHz, $CDCl_3$) of Compound S5 (Sugar region expanded)

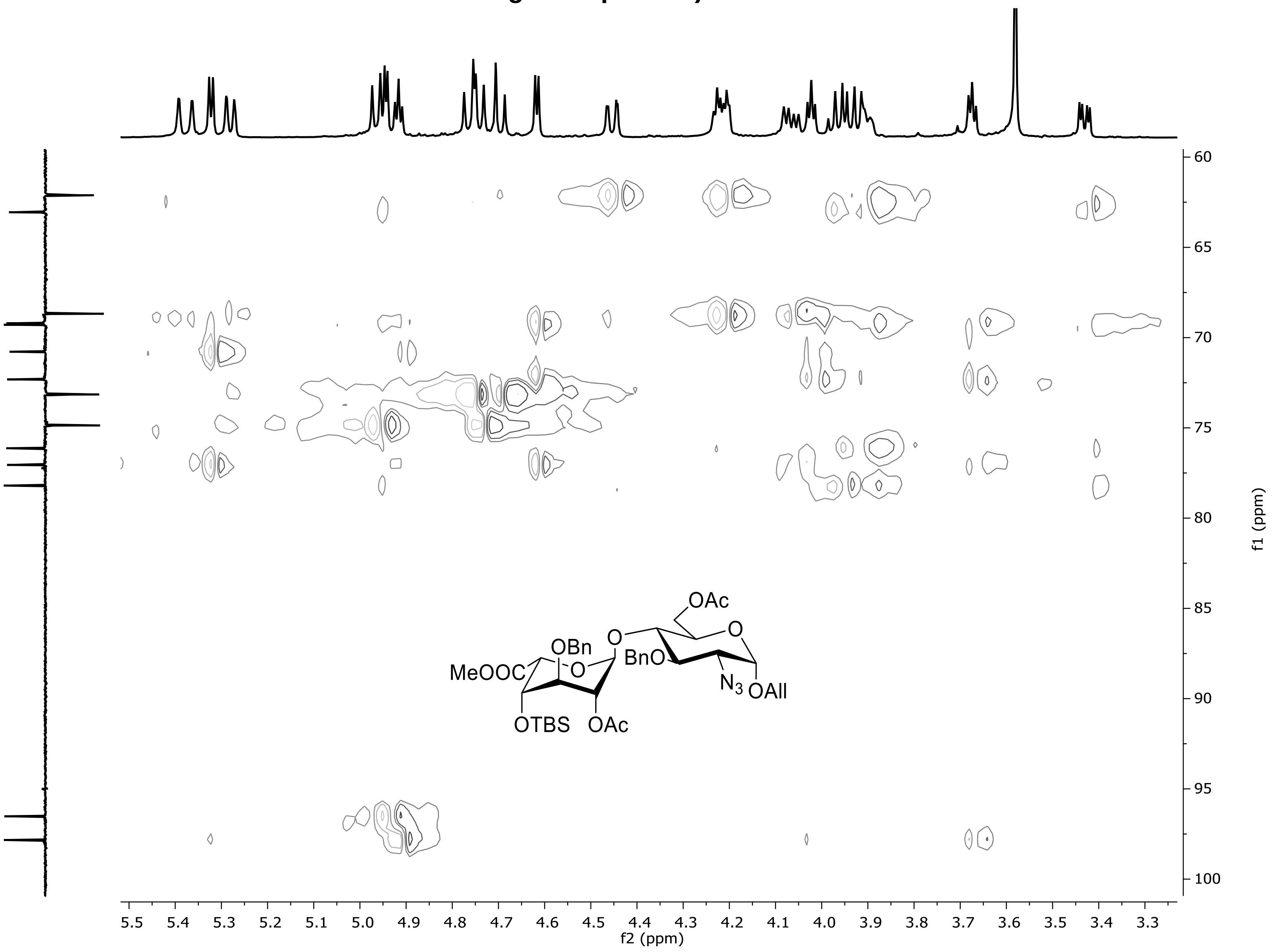

Supplementary Figure S23a. ^{1}H NMR Spectrum (400.31 MHz, $CDCl_3$) of Compound 4

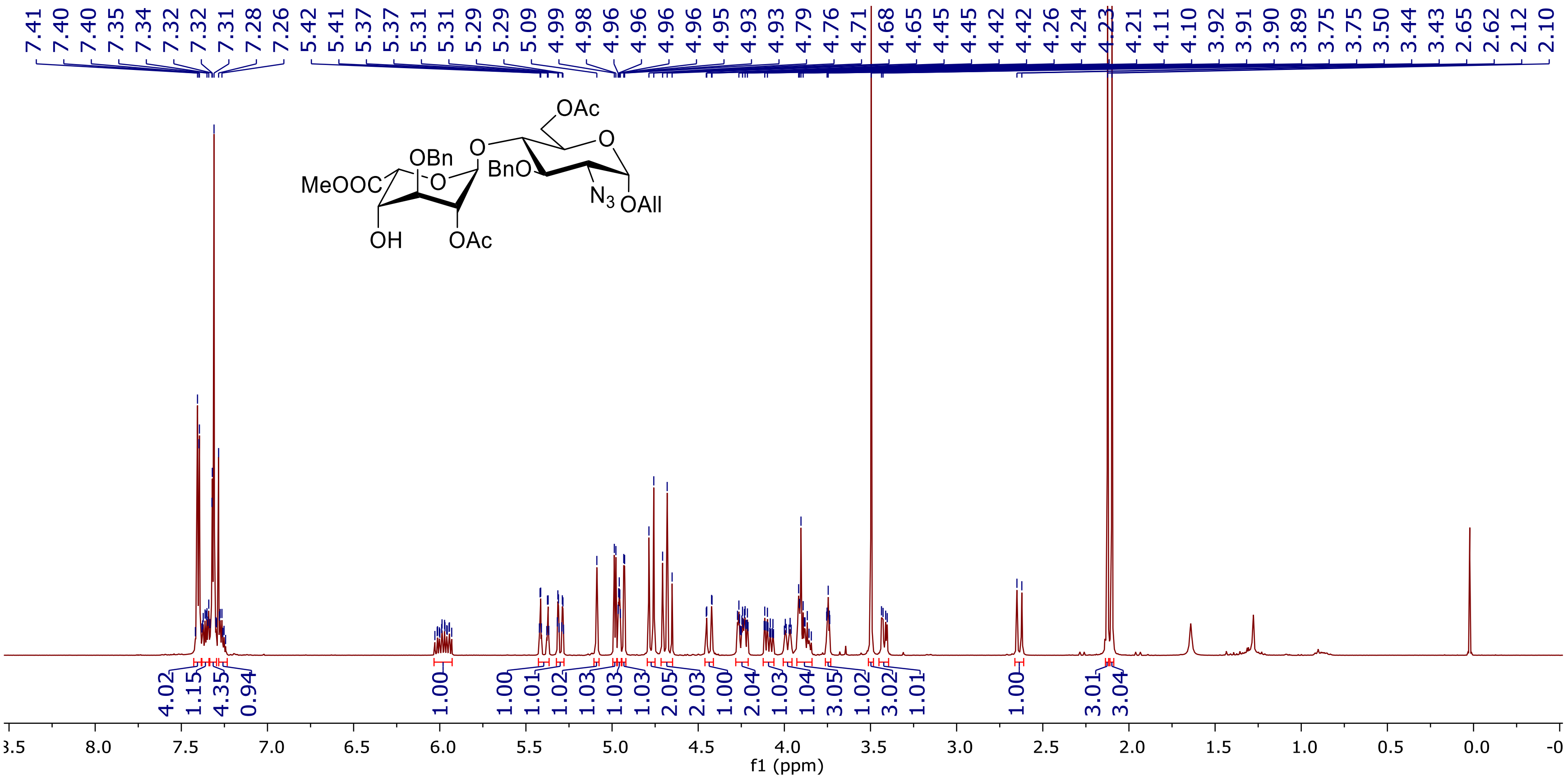

Supplementary Figure S23b. ^{13}C NMR Spectrum (100.67 MHz, $CDCl_3$) of Compound 4

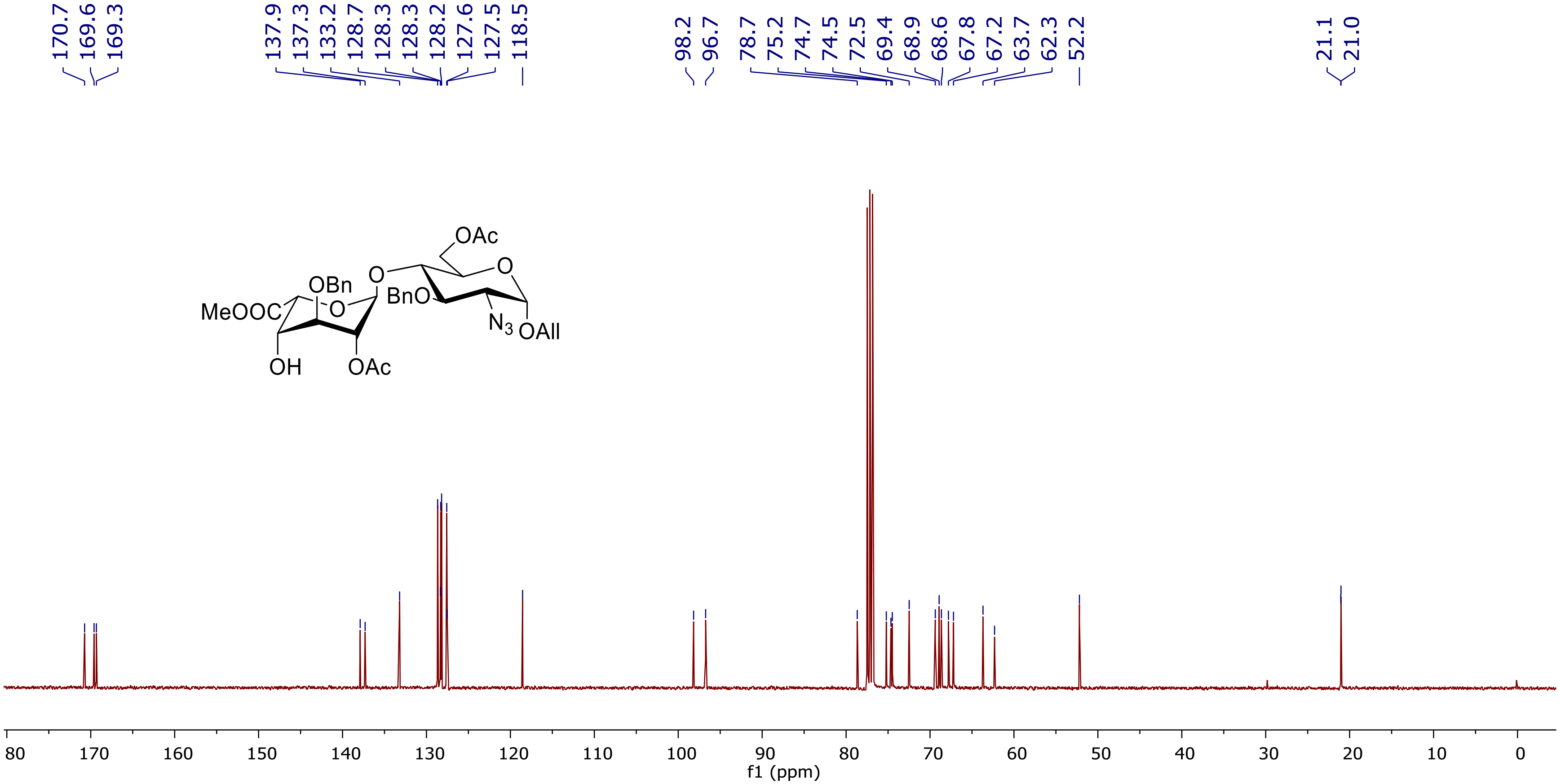

Supplementary Figure S23c. DEPT NMR Spectrum (100.67 MHz, $CDCl_3$) of Compound 4

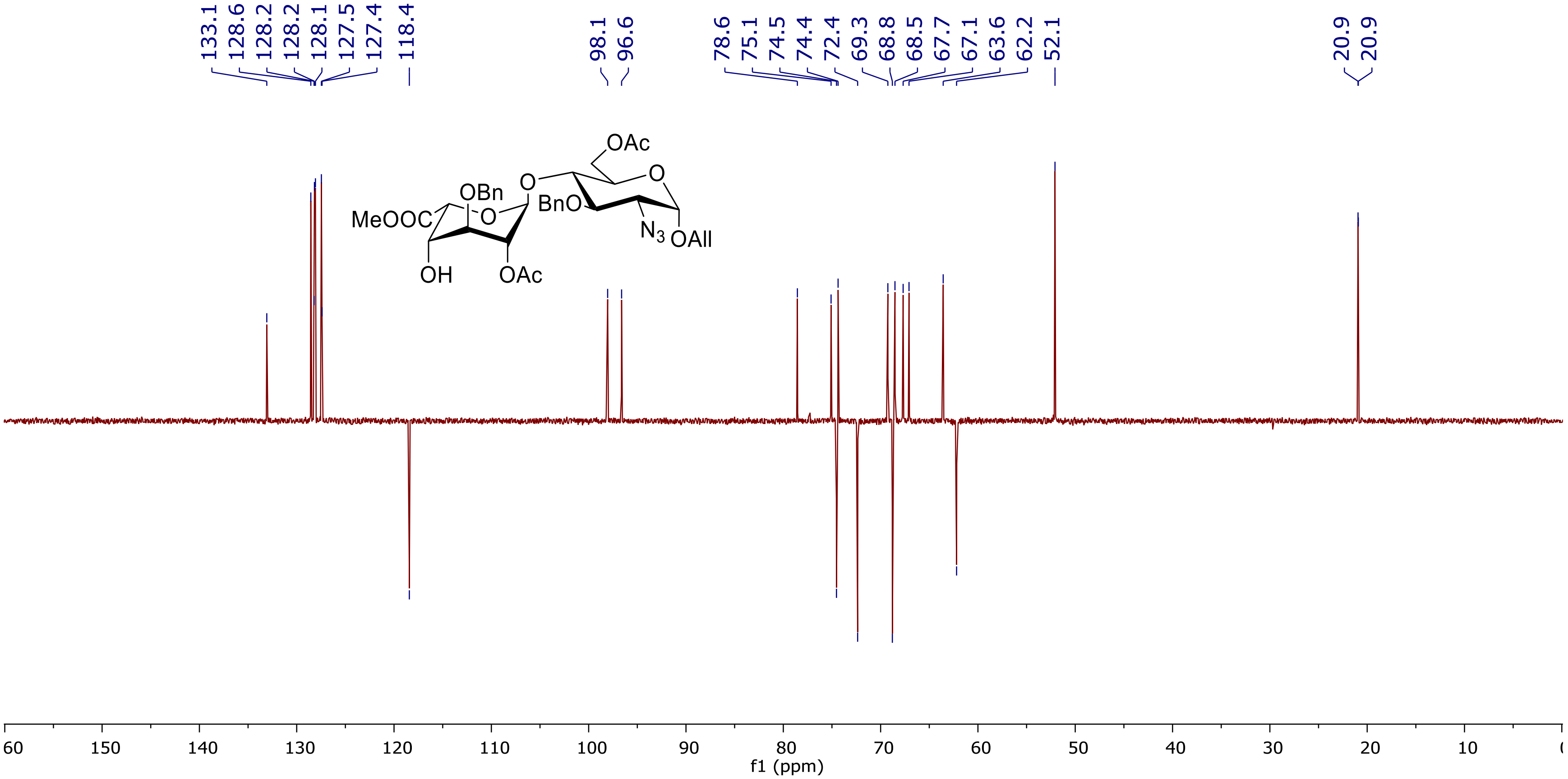

Supplementary Figure S23d. COSY NMR Spectrum (600.40, 600.40 MHz, $CDCl_3$) of Compound 4

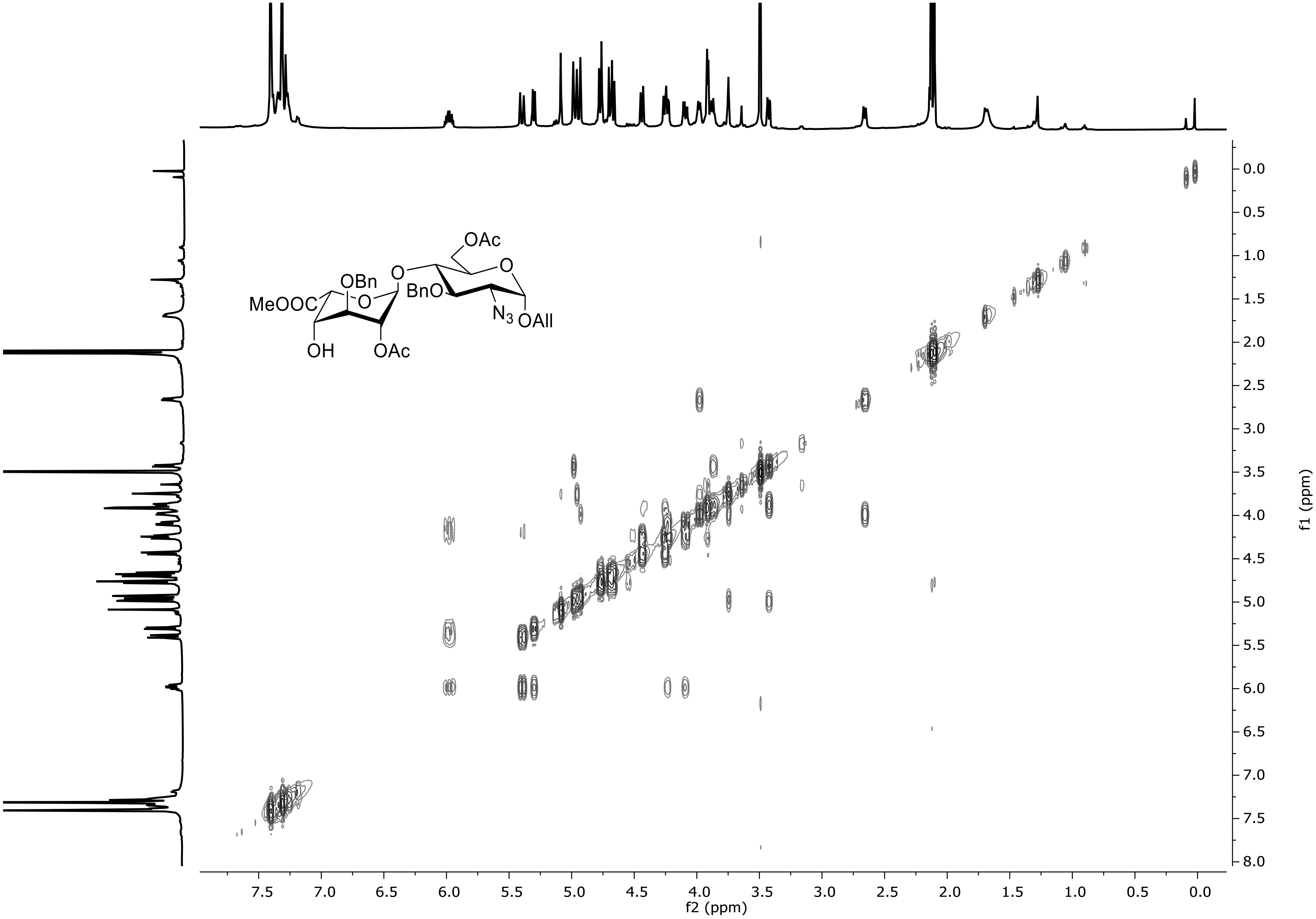

Supplementary Figure S23e. COSY NMR Spectrum (600.40, 600.40 MHz, $CDCl_3$) of Compound 4 (Sugar region expanded)

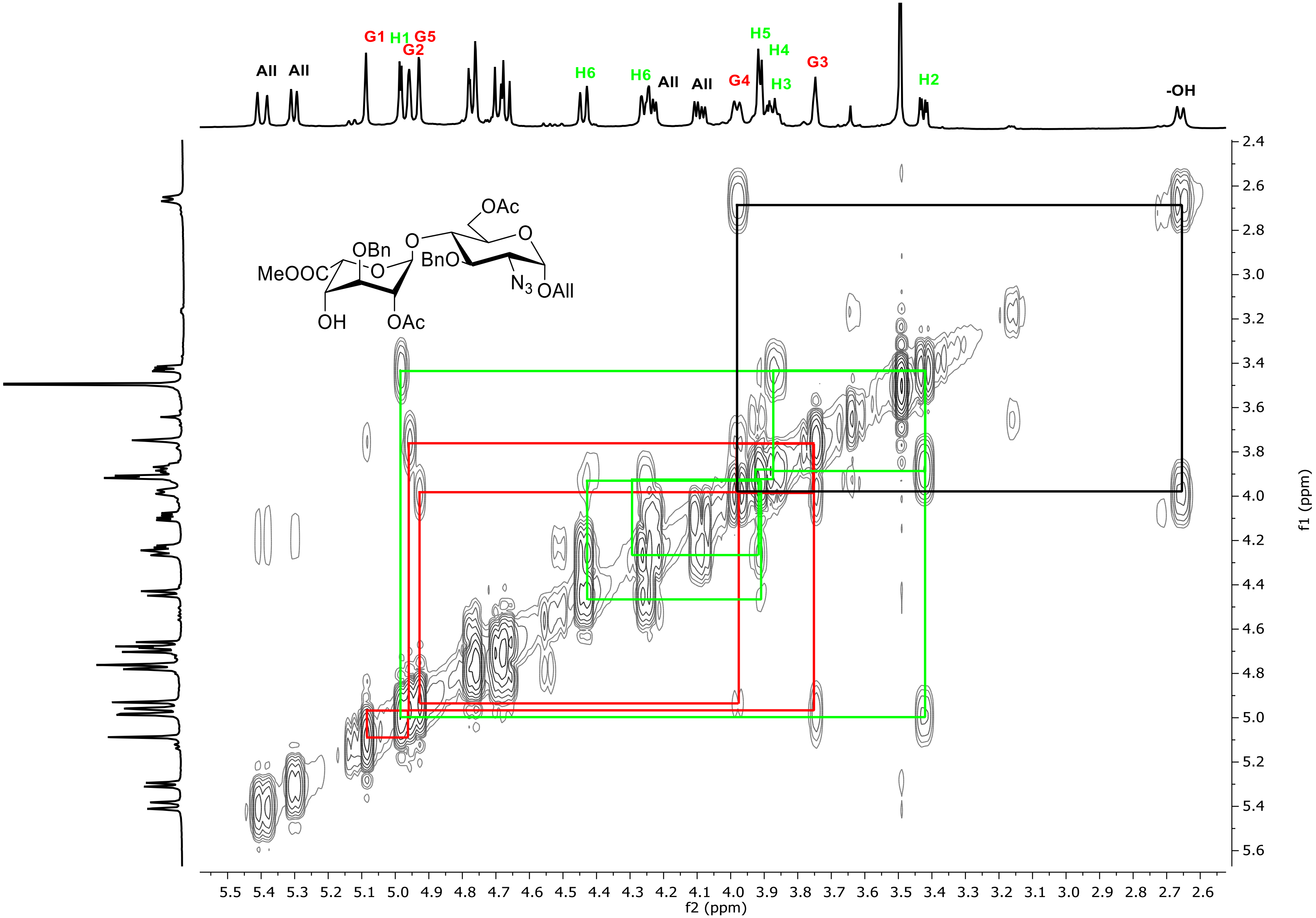

Supplementary Figure S23f. HSQC NMR Spectrum (600.40, 150.99MHz, $CDCl_3$) of Compound 4

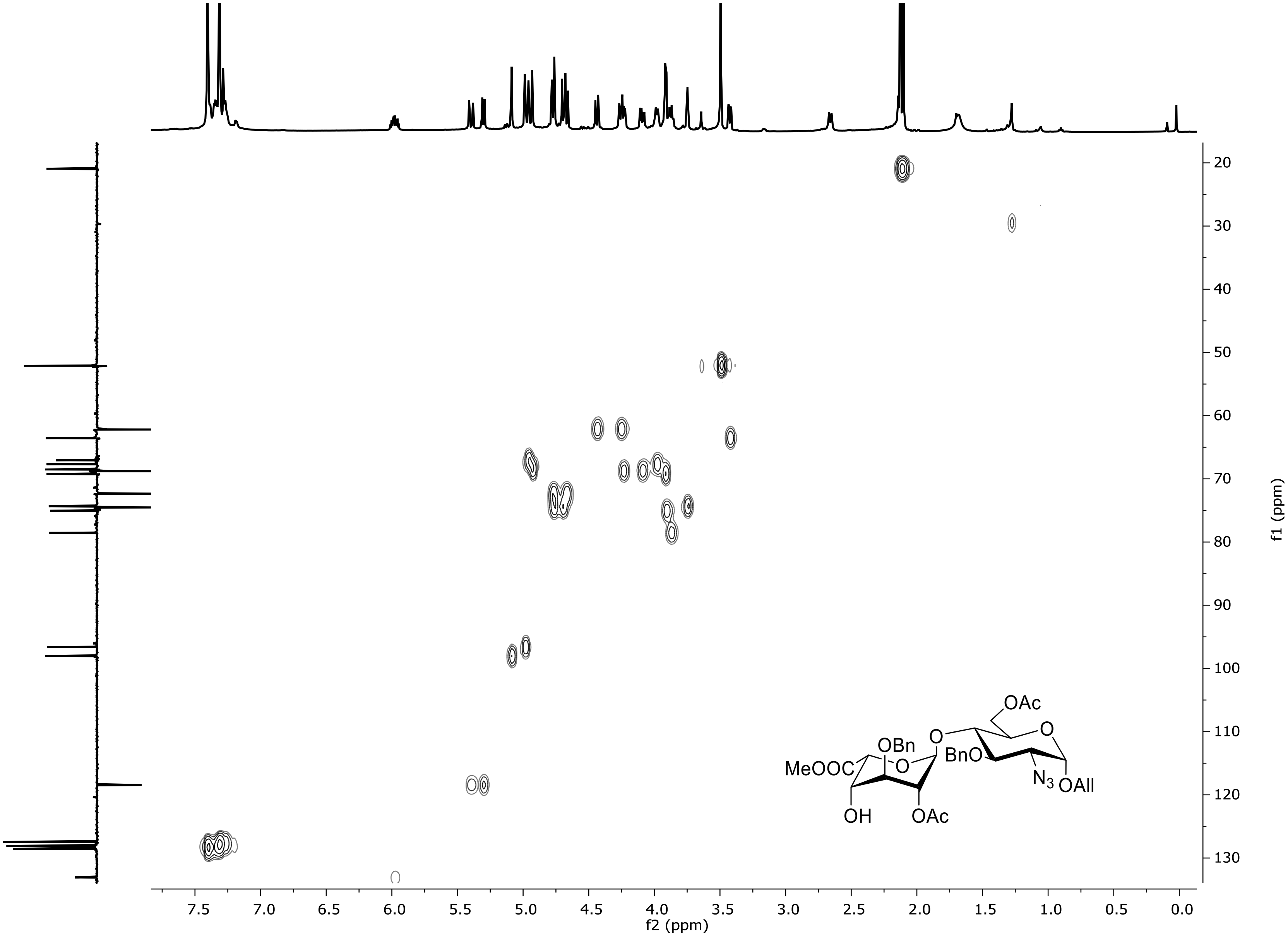

Supplementary Figure S23g. HSQC NMR Spectrum (600.40, 150.99 MHz, $CDCl_3$) of Compound 4 (Sugar region expanded)

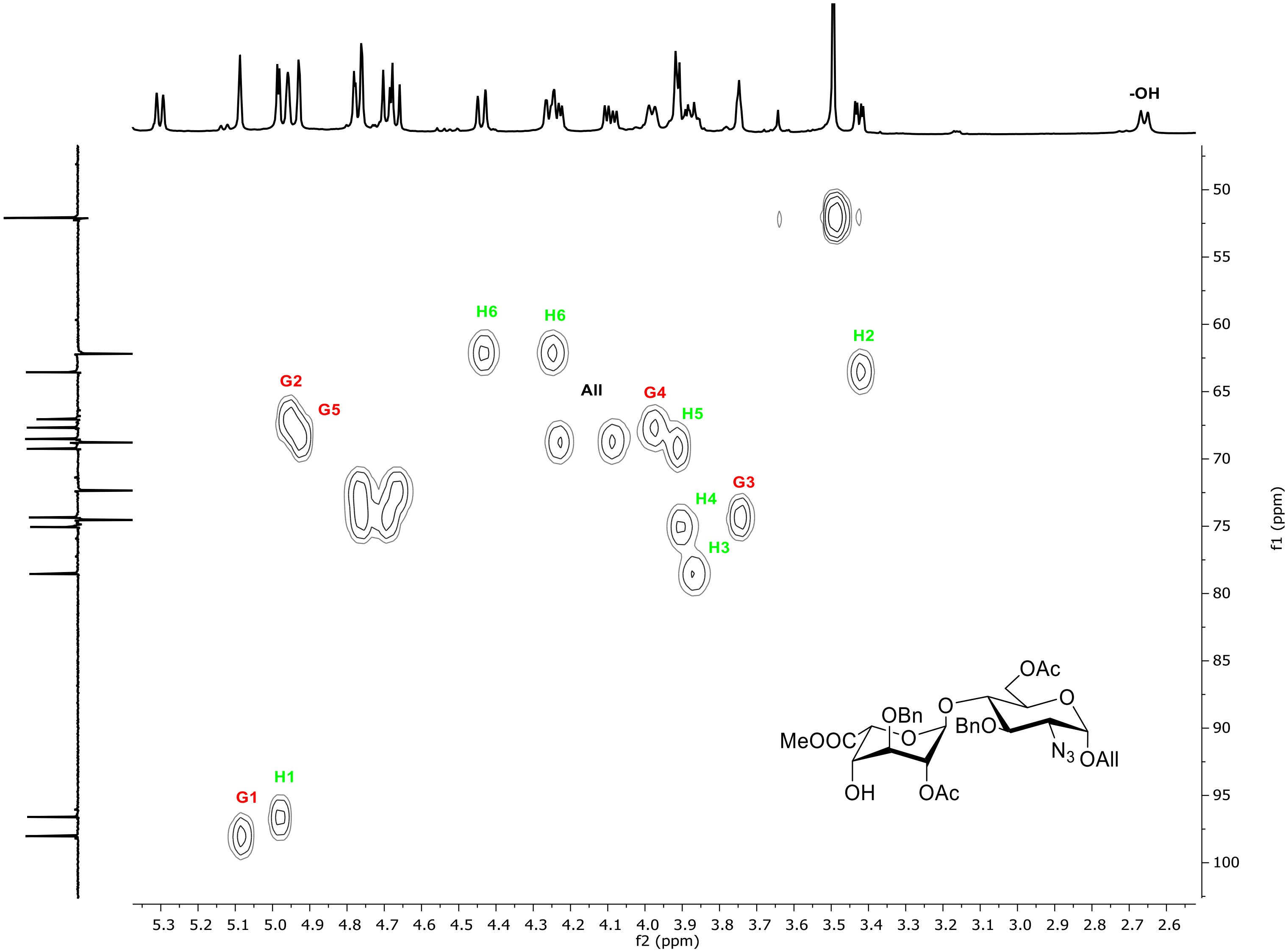

Supplementary Figure S23h. Coupled HSQC NMR Spectrum (600.40, 150.99 MHz, $CDCl_3$) of Compound 4

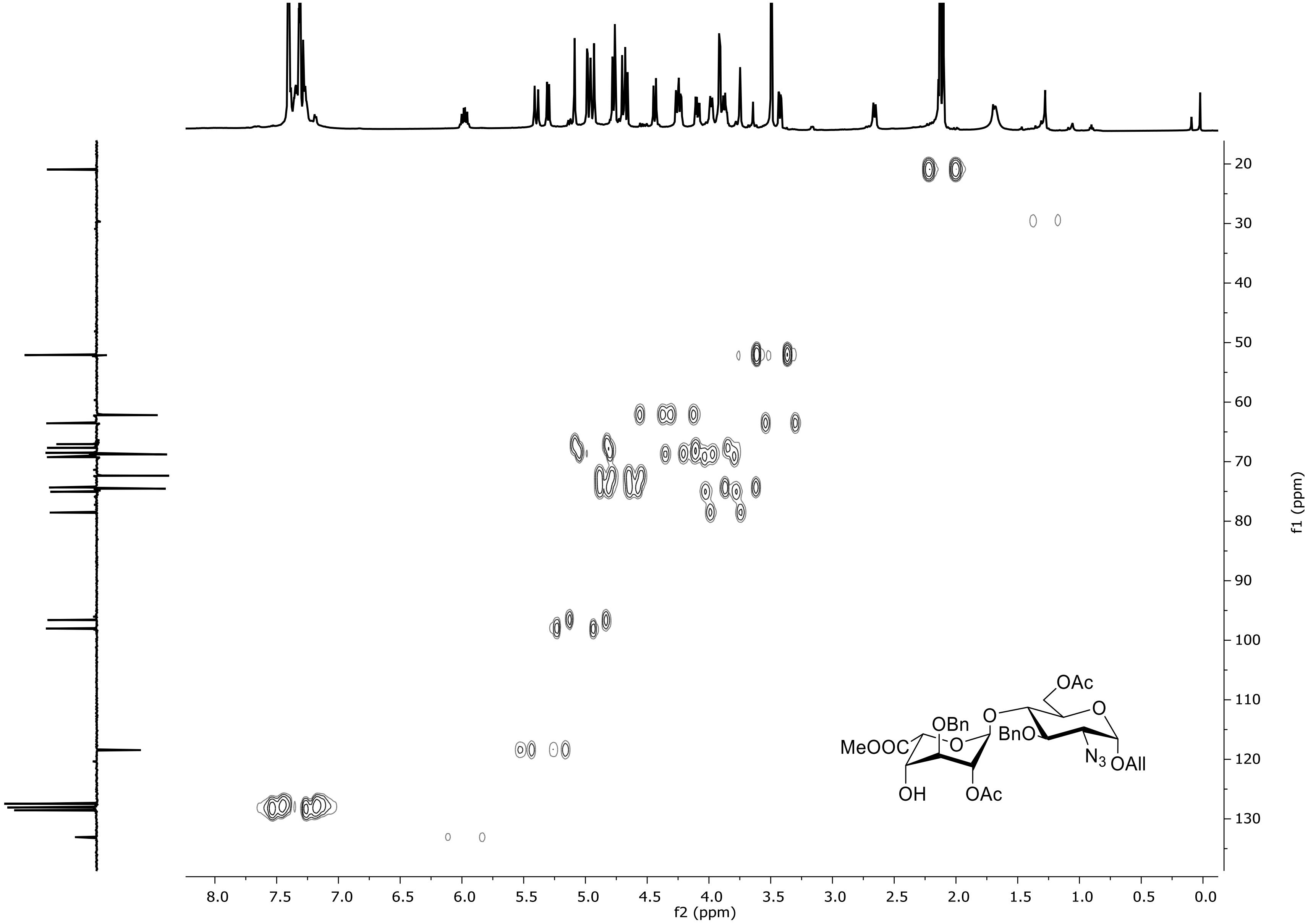

Supplementary Figure S23i. Coupled HSQC NMR Spectrum (600.40, 150.99 MHz, CDCl$_3$) of Compound 4 (Sugar region expanded)

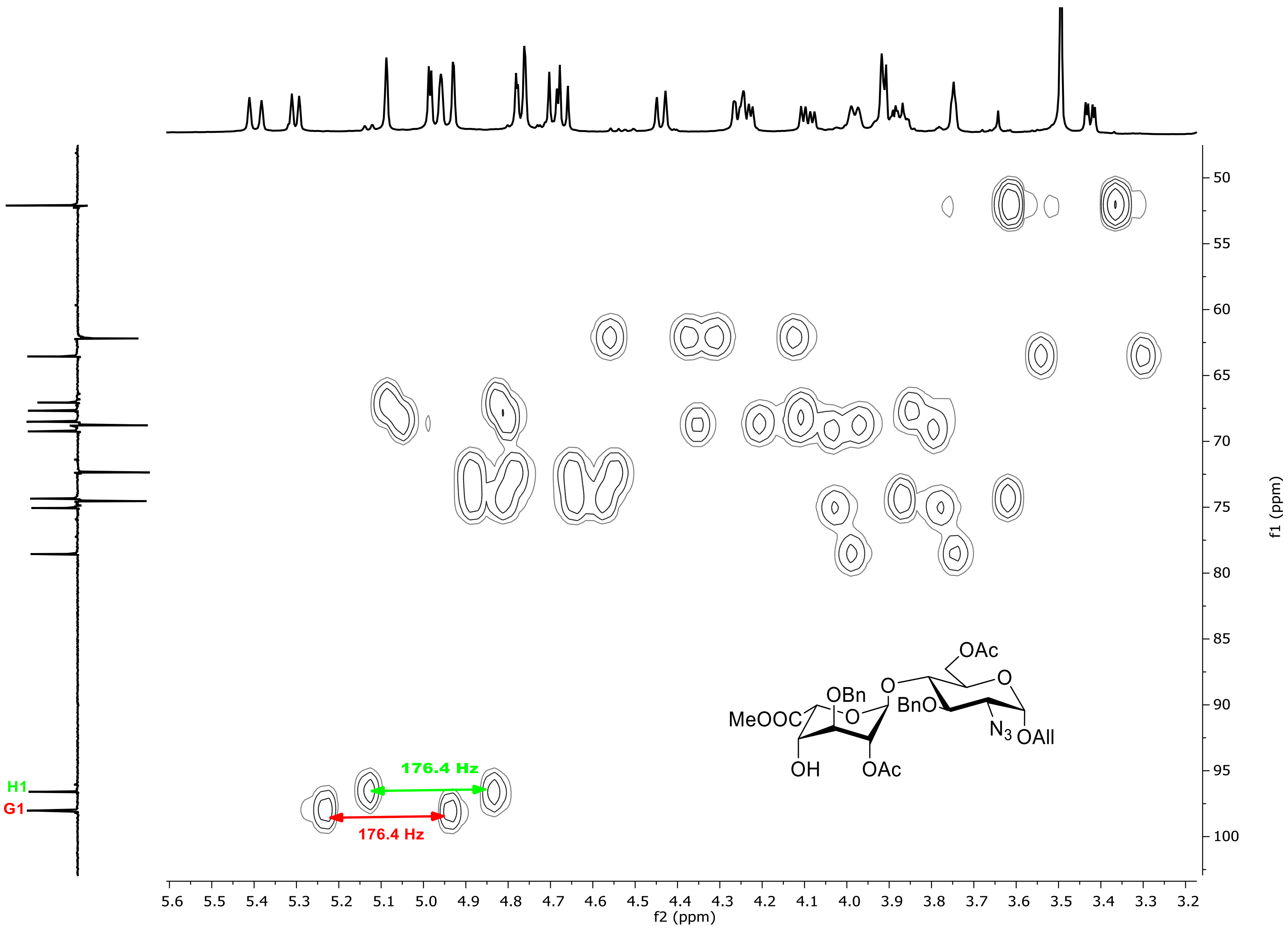

Supplementary Figure S23j. HMBC NMR Spectrum (600.40, 150.99 MHz, $CDCl_3$) of Compound 4

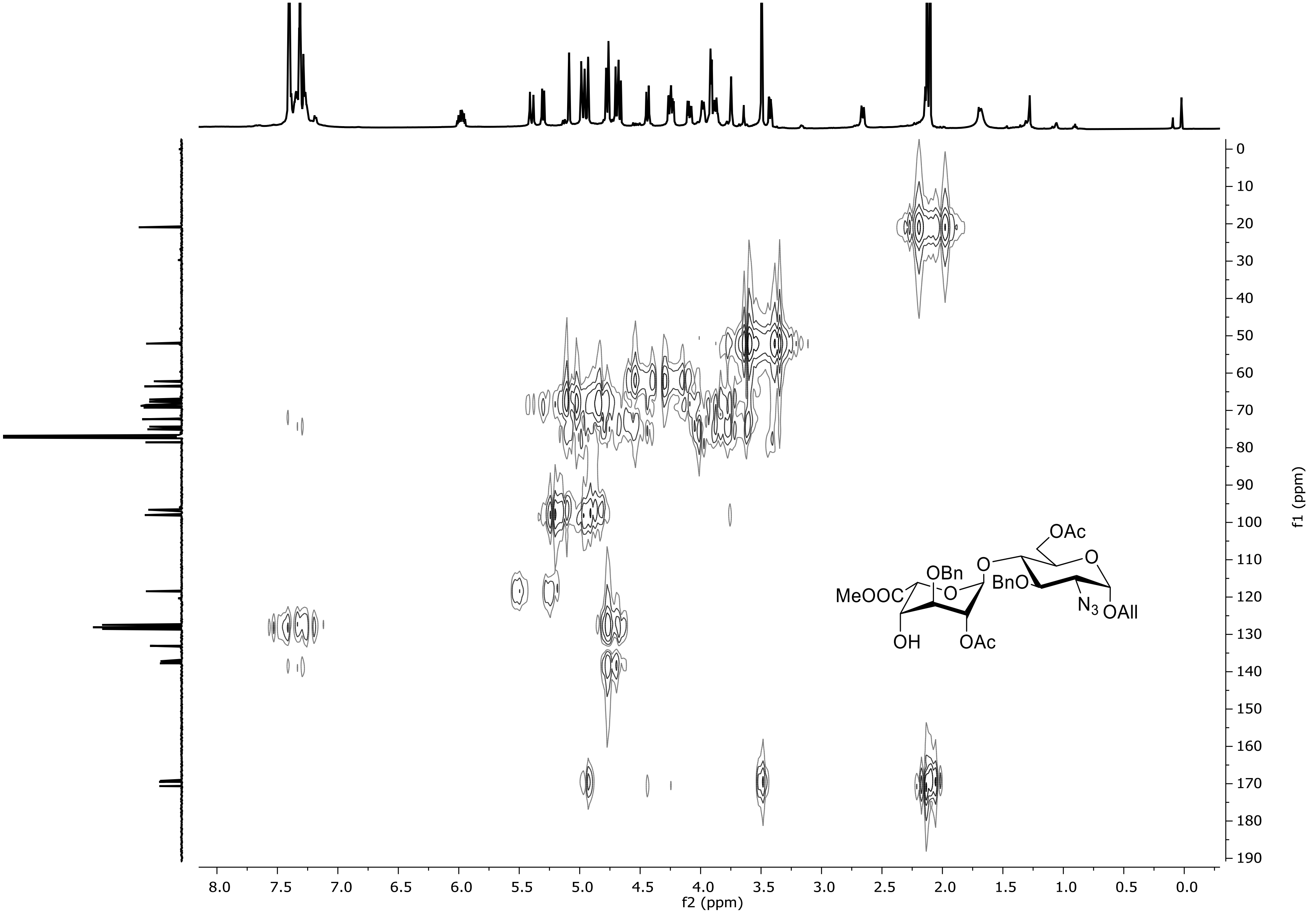

Supplementary Figure S23k. HMBC Spectrum (600.40, 150.99 MHz, $CDCl_3$) of Compound 4 (Sugar region expanded)

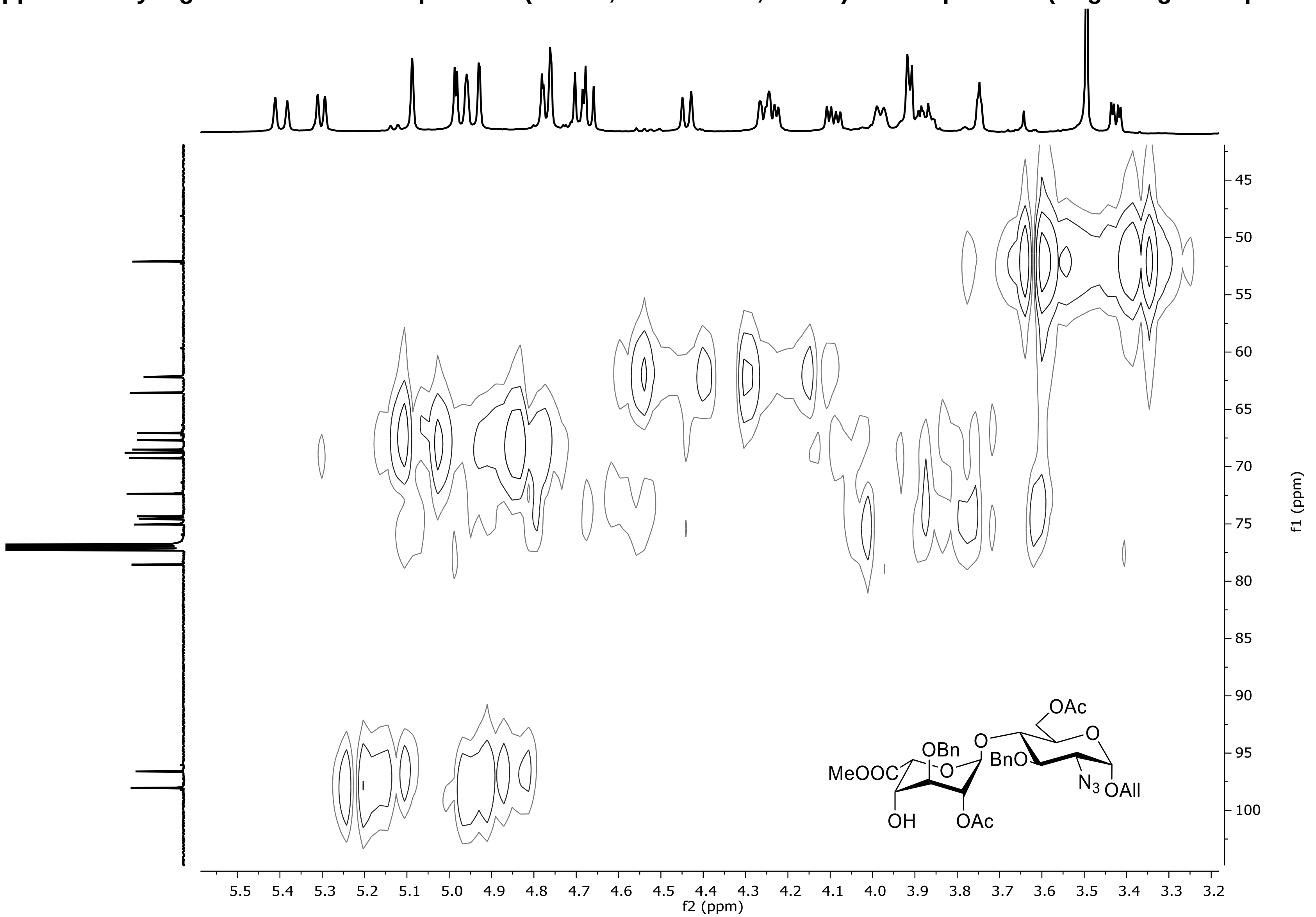

Supplementary Figure S23l. HMBC NMR Spectrum (600.40, 150.99 MHz, $CDCl_3$) of Compound 4 (Carbonyl Region expanded)

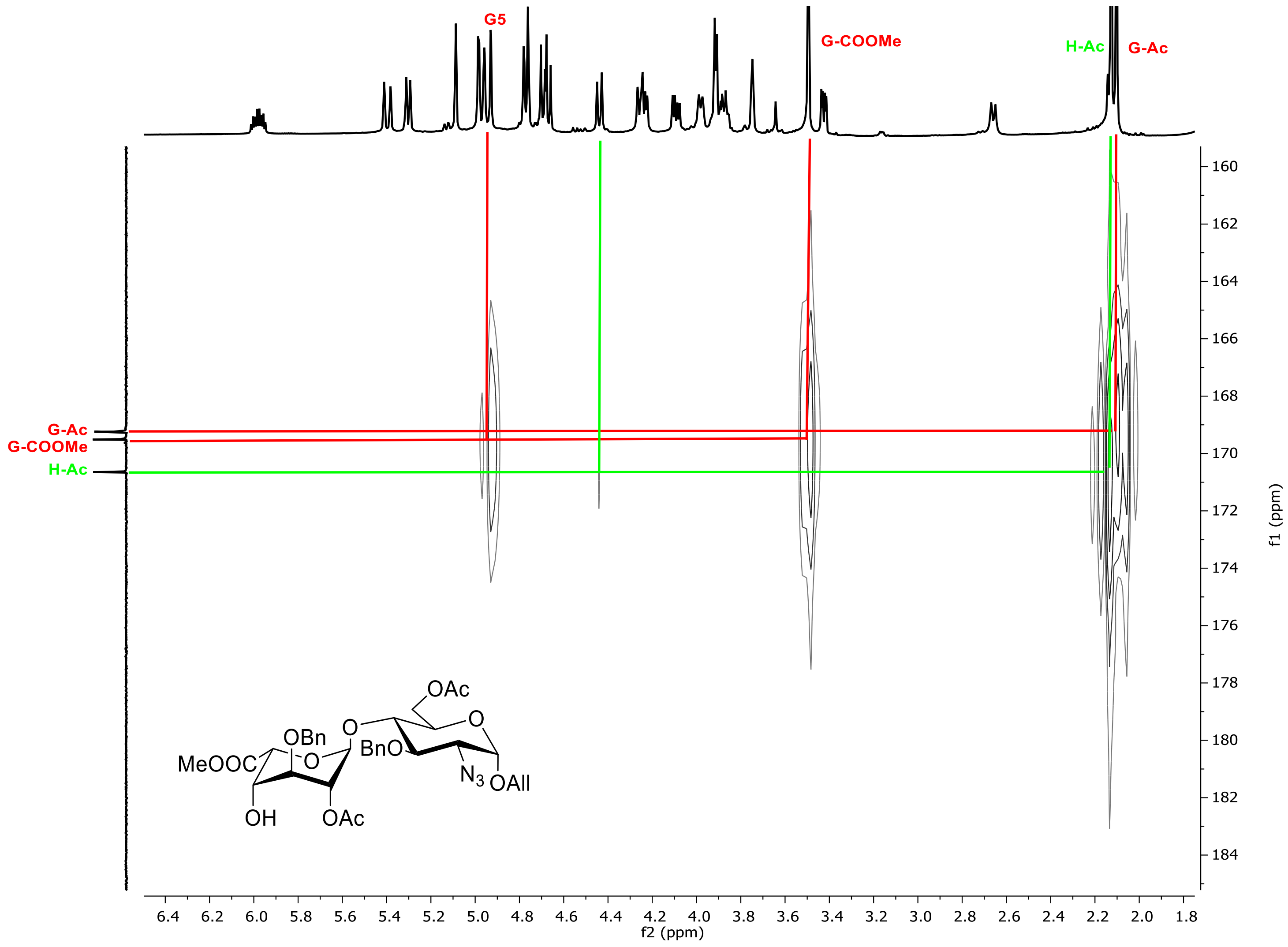

Supplementary Figure S23m. TOCSY NMR Spectrum (600.40, 600.40 MHz, $CDCl_3$) of Compound 4

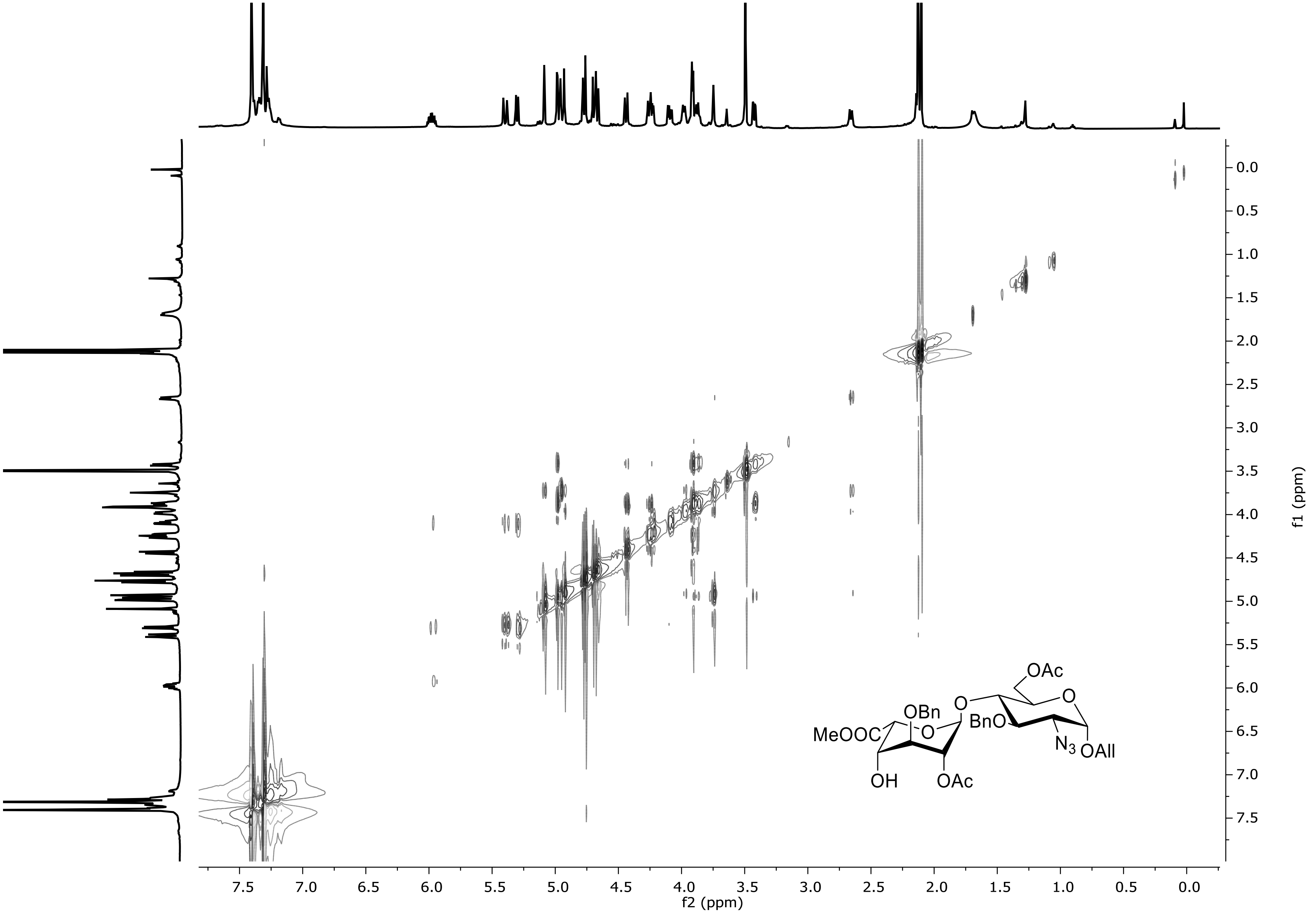

Supplementary Figure S23n. TOCSY NMR Spectrum (600.40, 600.40 MHz, $CDCl_3$) of Compound 4 (Sugar region expanded)

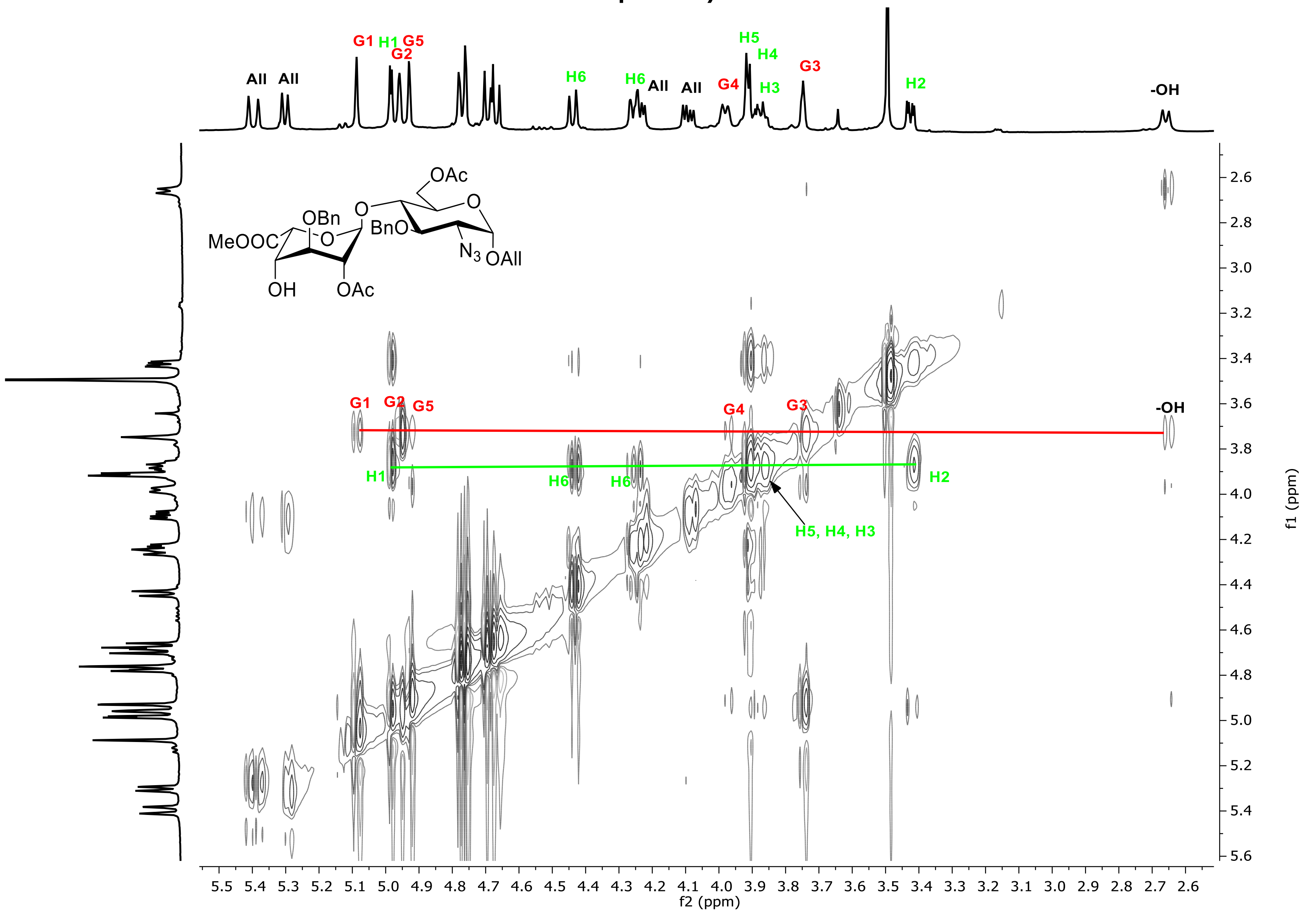

Supplementary Figure S23o. HSQC-TOCSY NMR Spectrum (600.40, 150.99 MHz, $CDCl_3$) of Compound 4

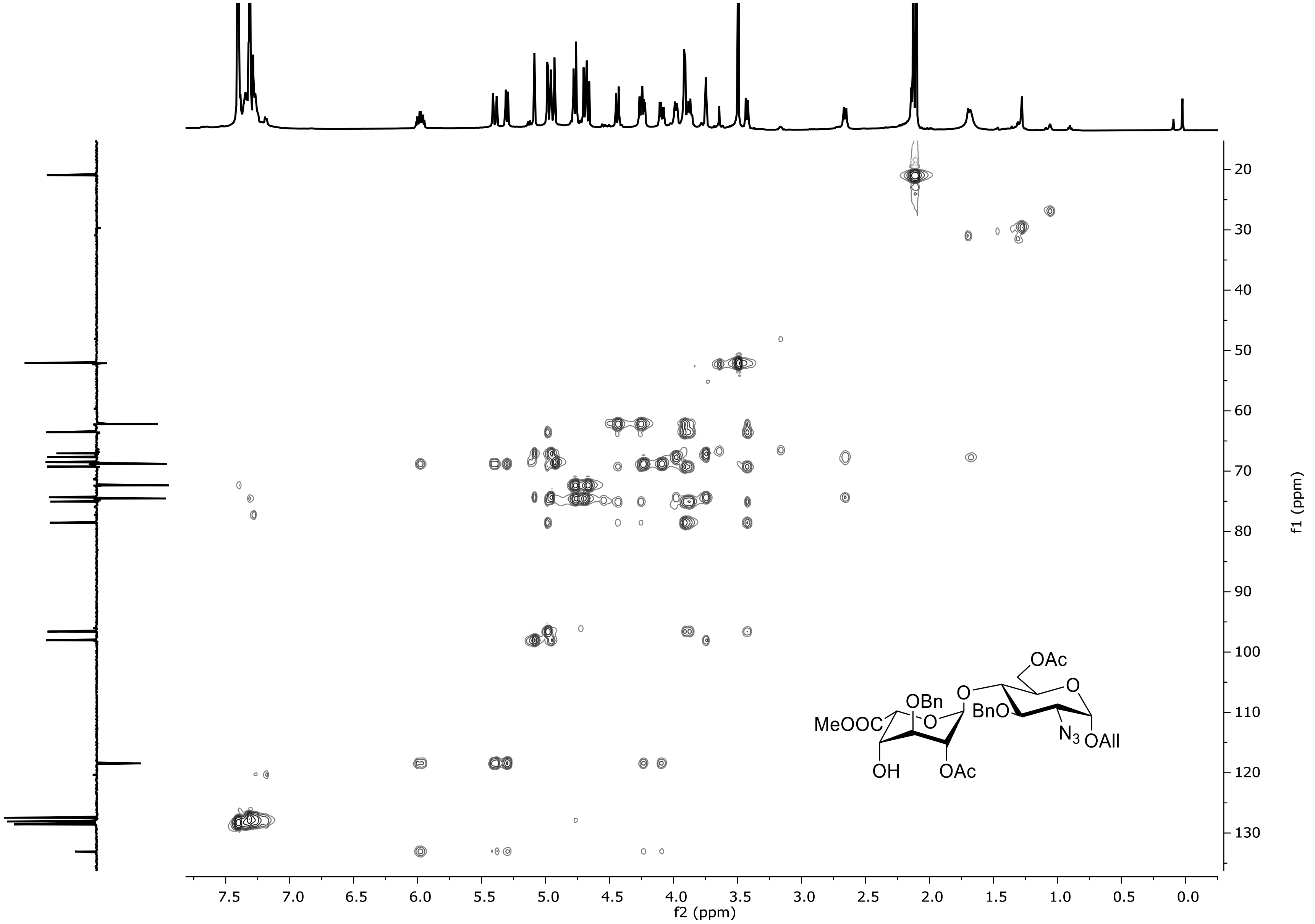

Supplementary Figure S23p. HSQC-TOCSY NMR Spectrum (600.40, 150.99 MHz, $CDCl_3$) of Compound 4 (Sugar region expanded)

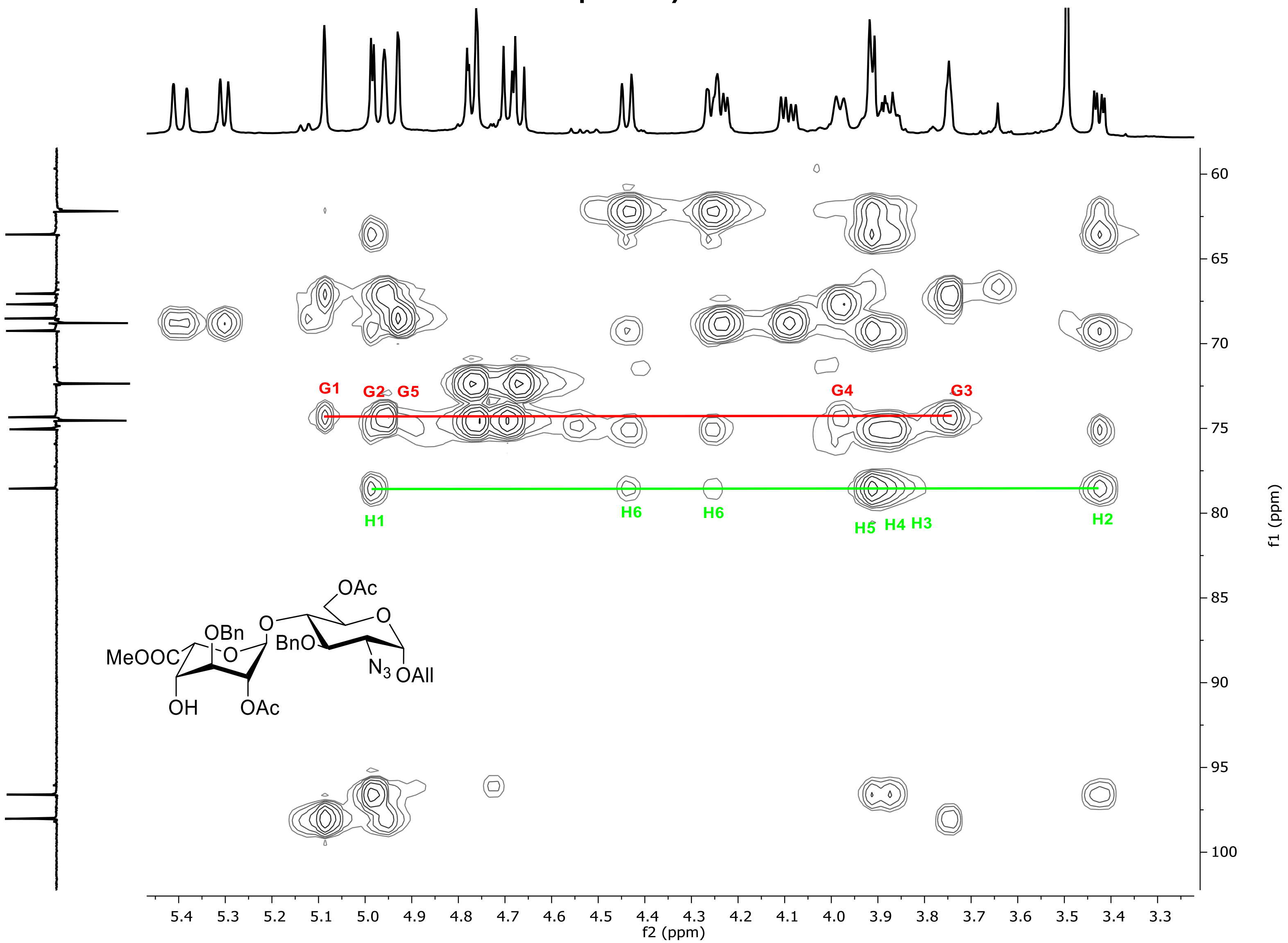

Supplementary Figure S24a. ^{1}H NMR Spectrum (400.31 MHz, $CDCl_3$) of Compound S6

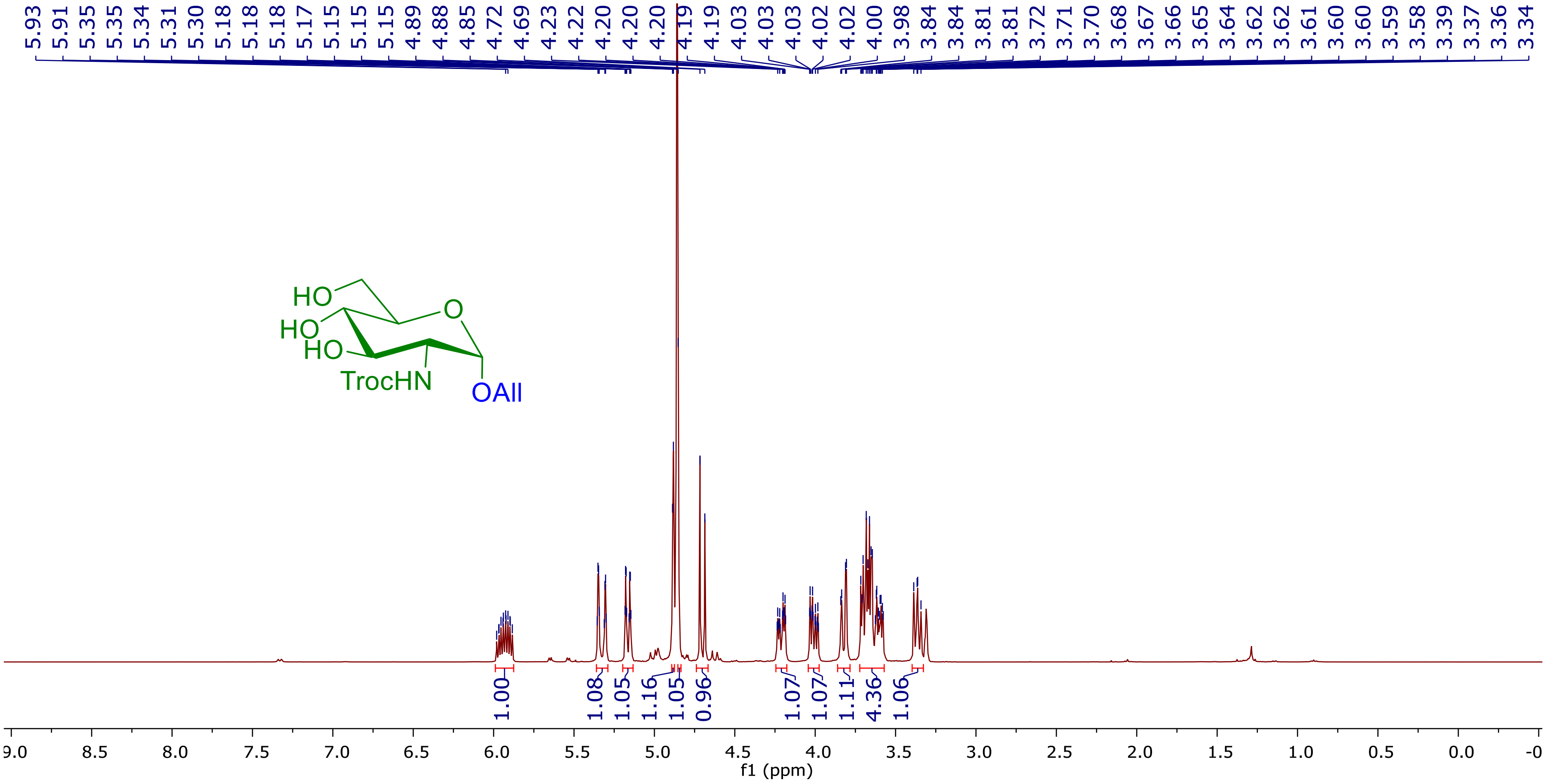

Supplementary Figure S24b. ^{13}C NMR Spectrum (100.67 MHz, $CDCl_3$) of Compound S6

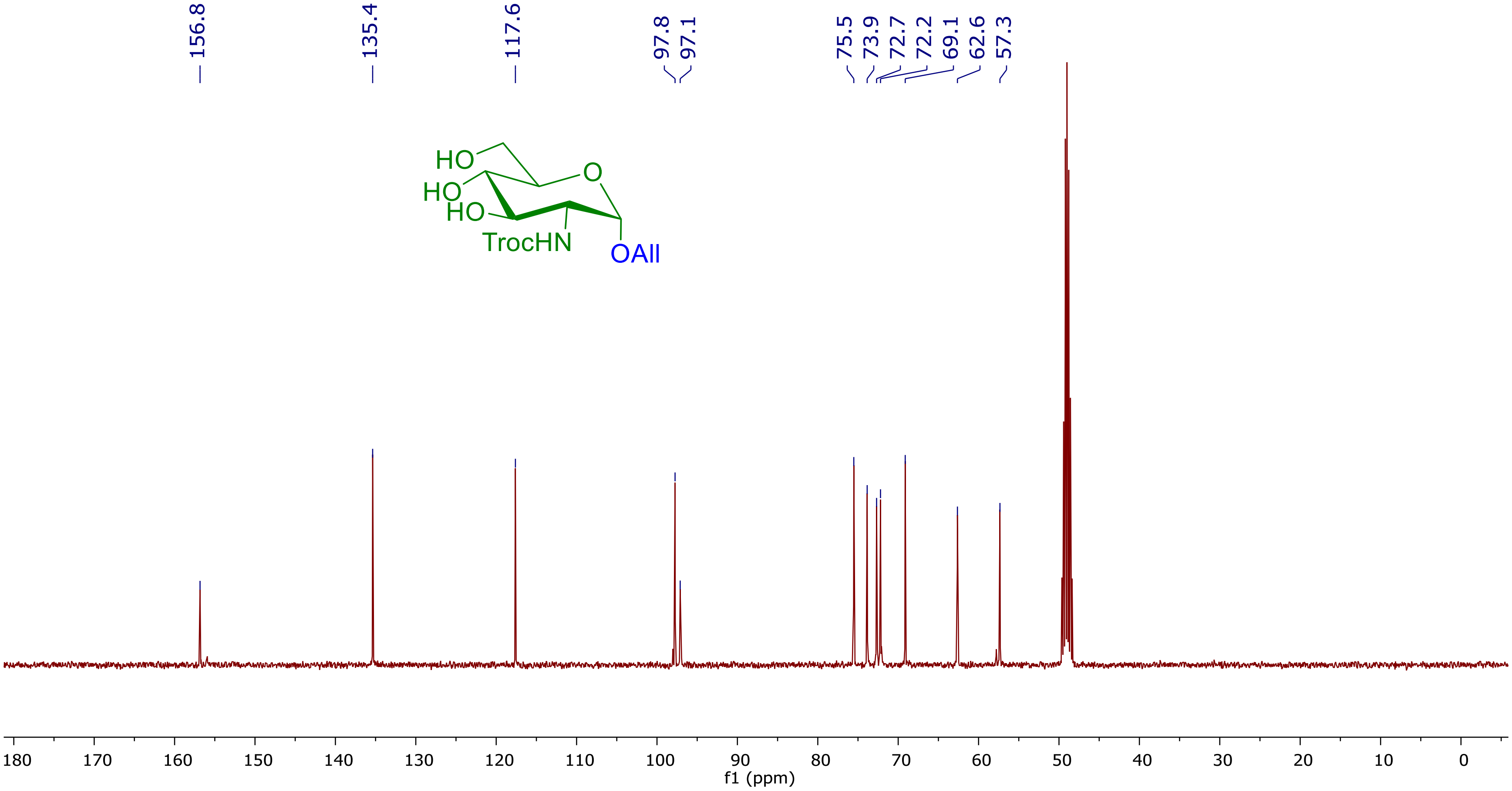

Supplementary Figure S24c. DEPT NMR Spectrum (100.67 MHz, $CDCl_3$) of Compound S6

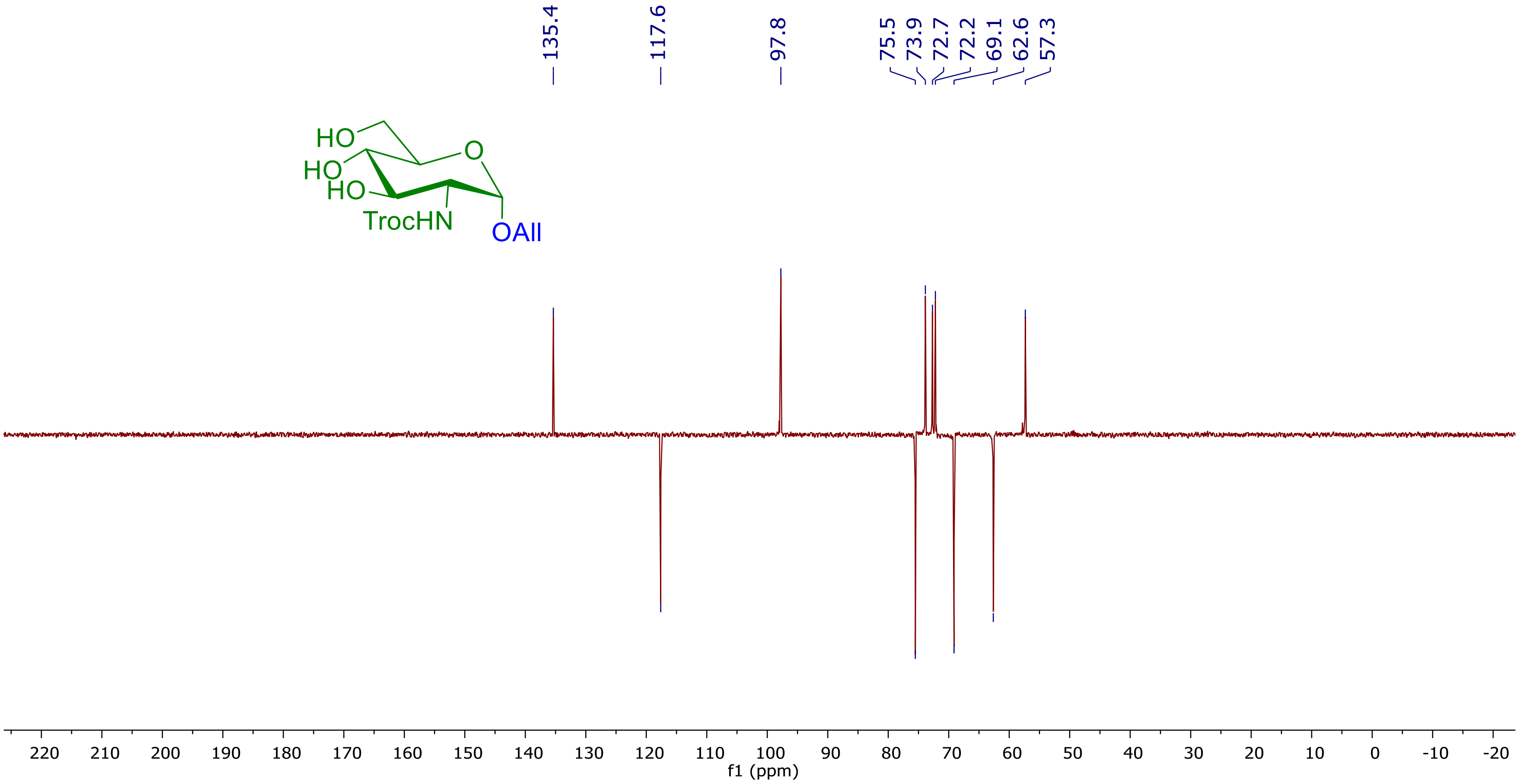

Supplementary Figure S25a. ¹H NMR Spectrum (400.31 MHz, $CDCl_3$) of Compound 27

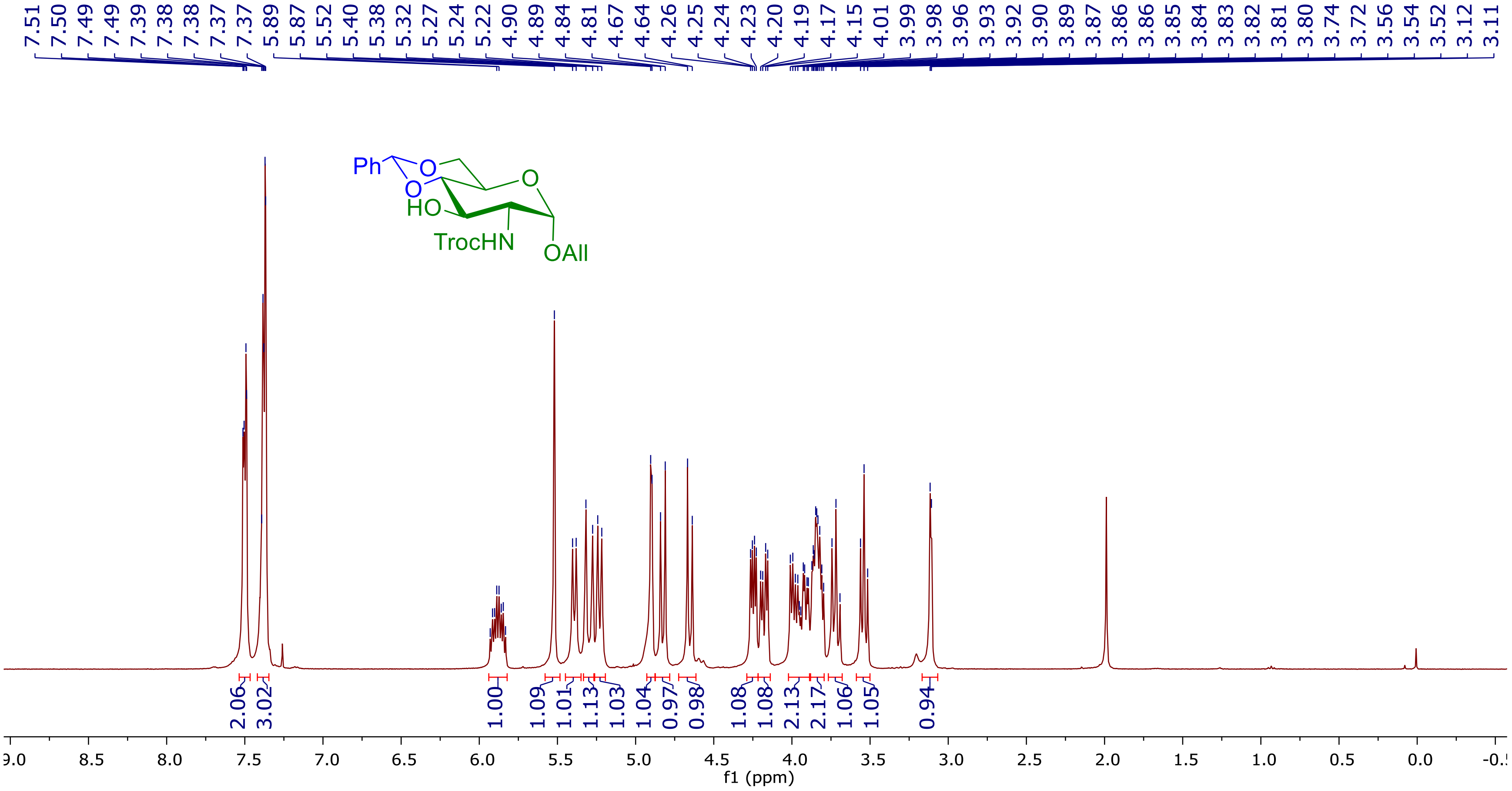

Supplementary Figure S25b. ^{13}C NMR Spectrum (100.67 MHz, $CDCl_3$) of Compound 27

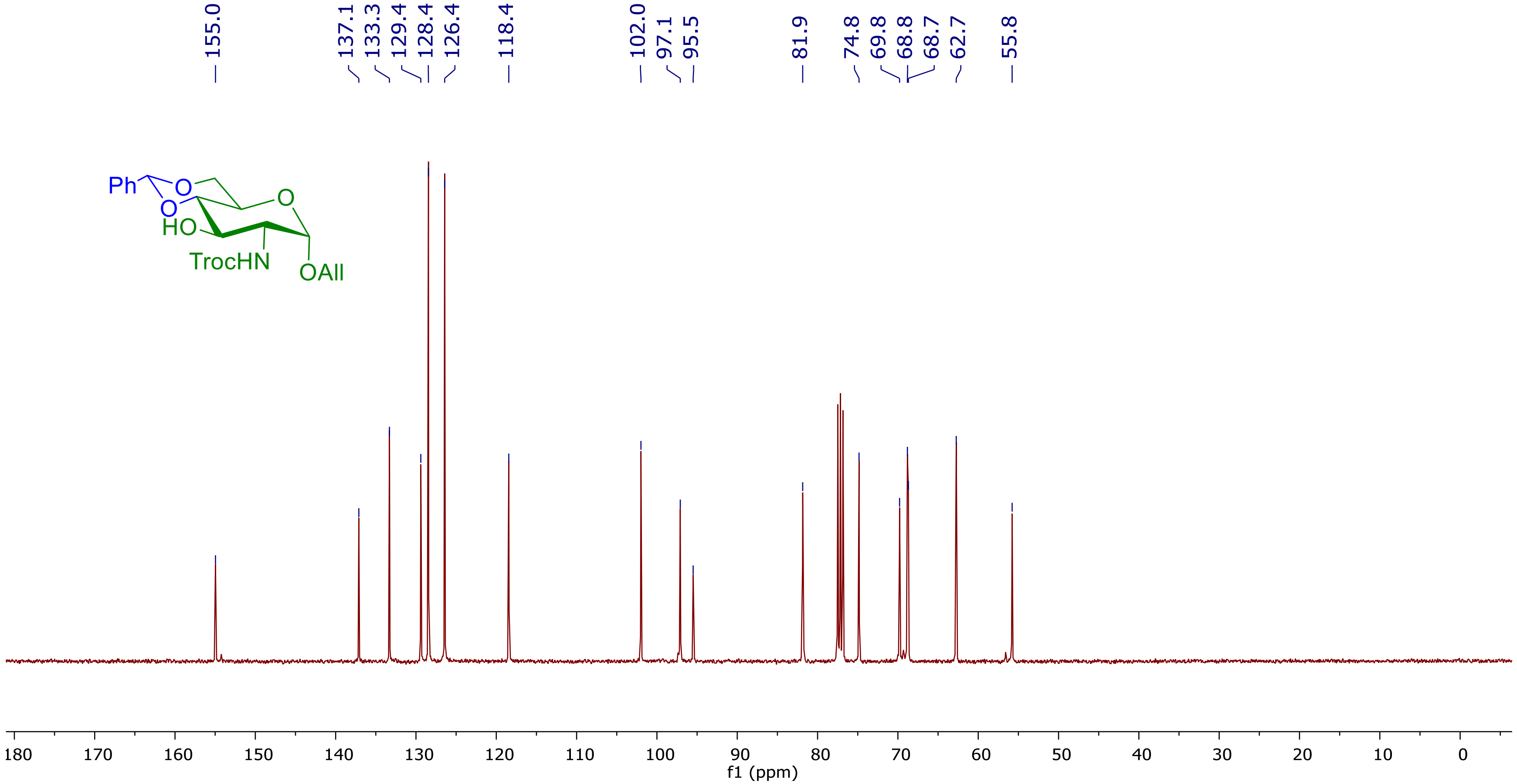

Supplementary Figure S25c. DEPT NMR Spectrum (100.67 MHz, $CDCl_3$) of Compound 27

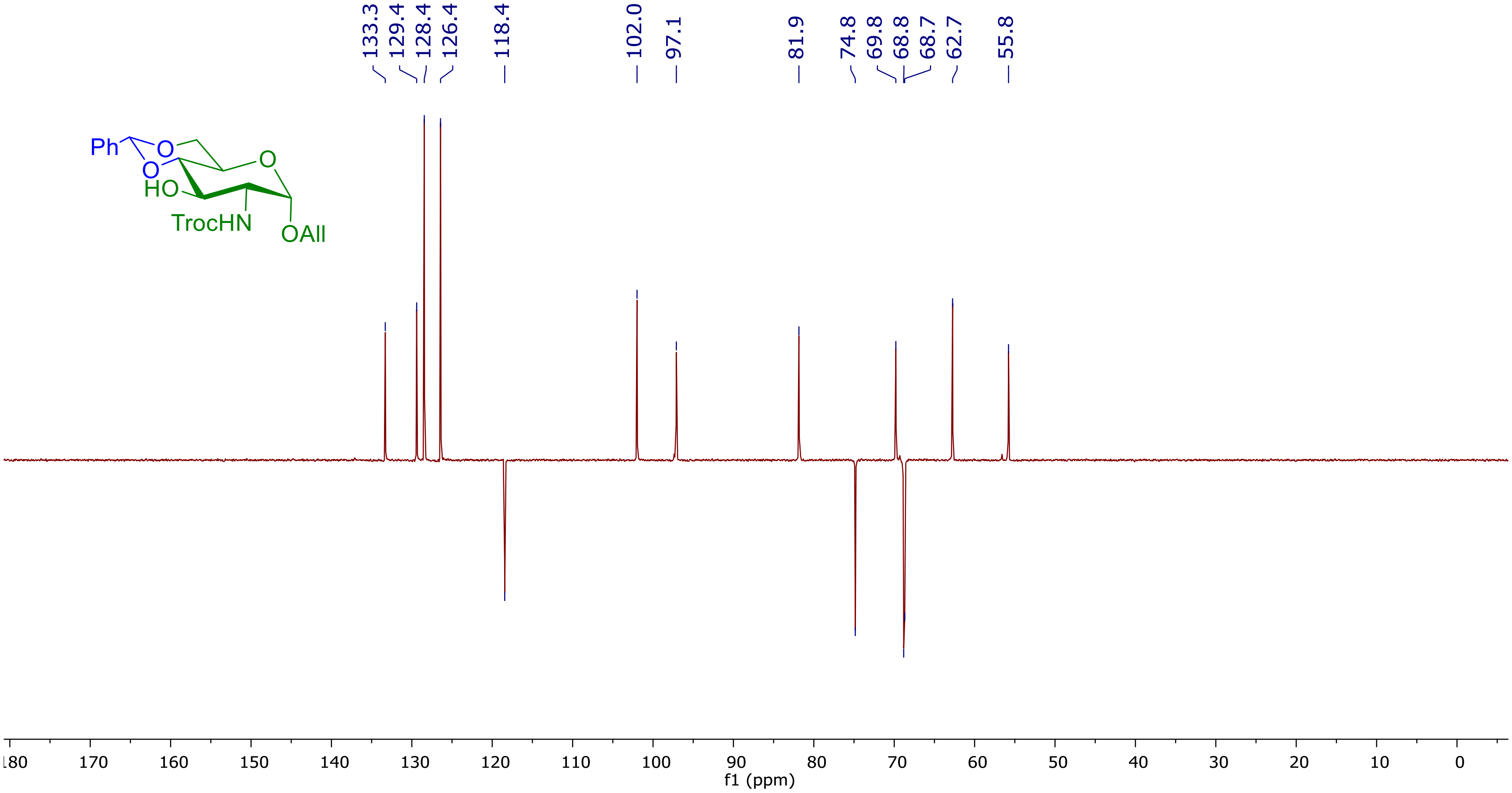

Supplementary Figure S26a. ^{1}H NMR Spectrum (400.31 MHz, $CDCl_3$) of Compound 28

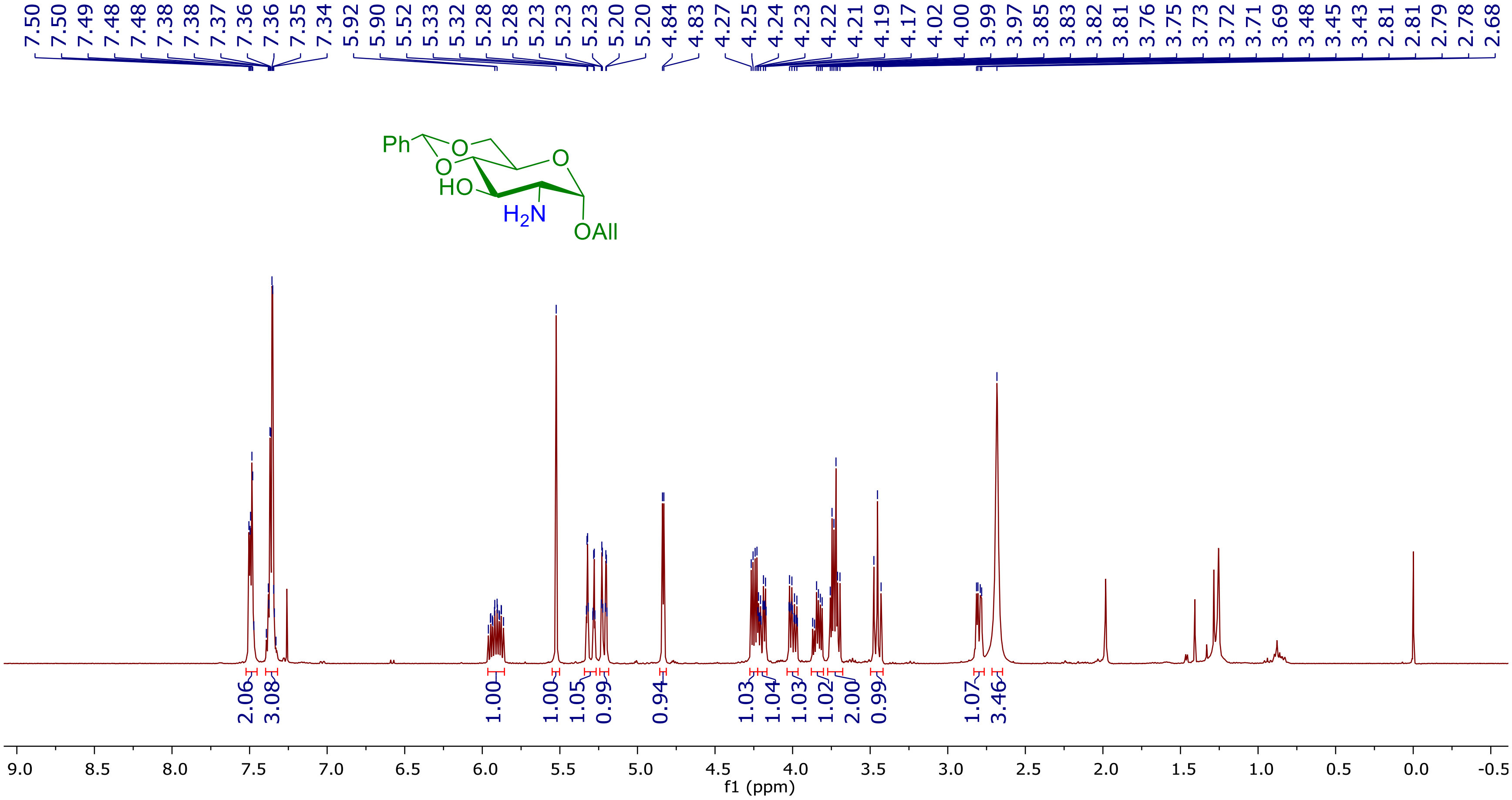

Supplementary Figure S26b. ^{13}C NMR Spectrum (100.67 MHz, $CDCl_3$) of Compound 28

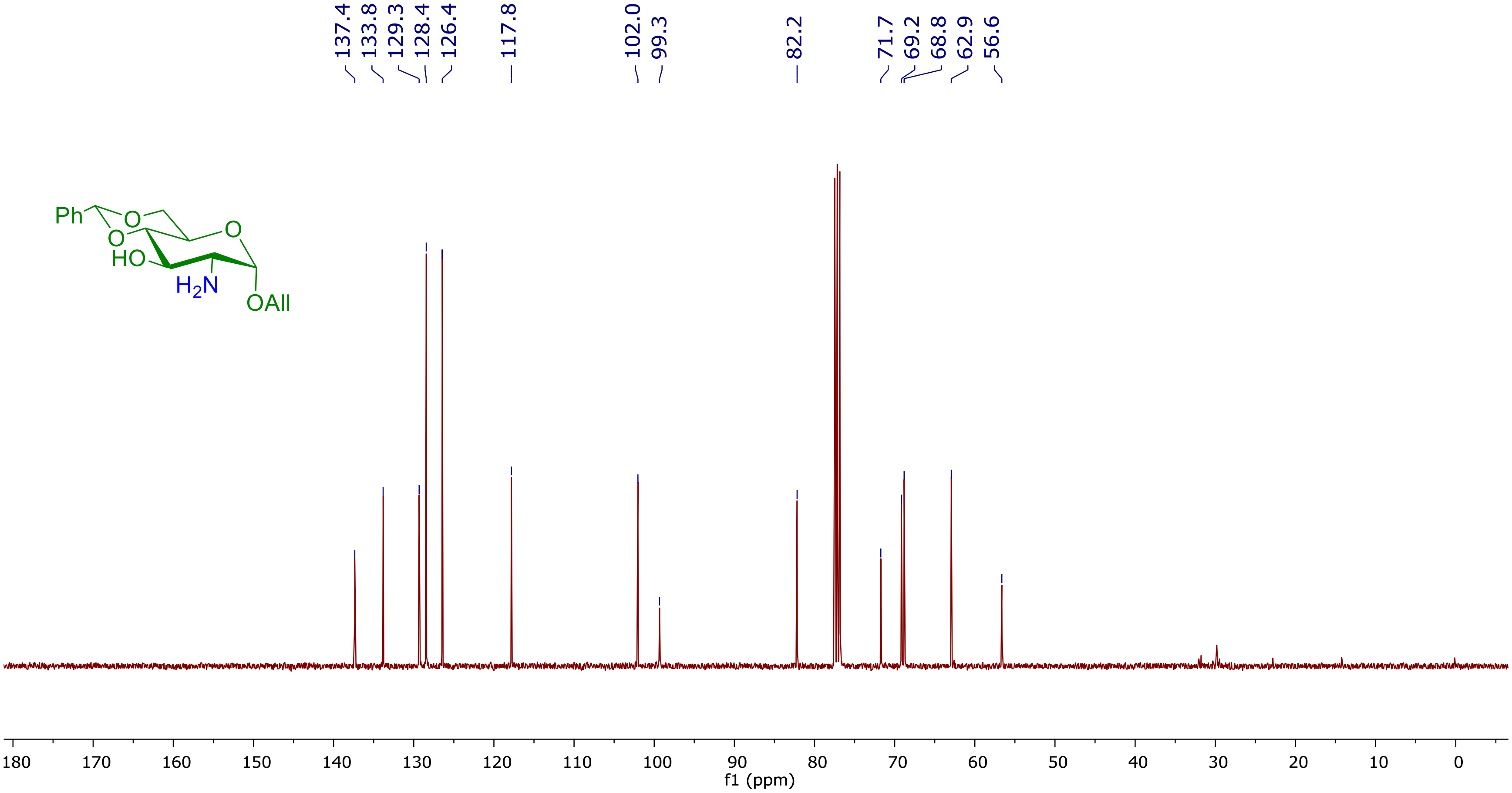

Supplementary Figure S26c. DEPT NMR Spectrum (100.67 MHz, $CDCl_3$) of Compound 28

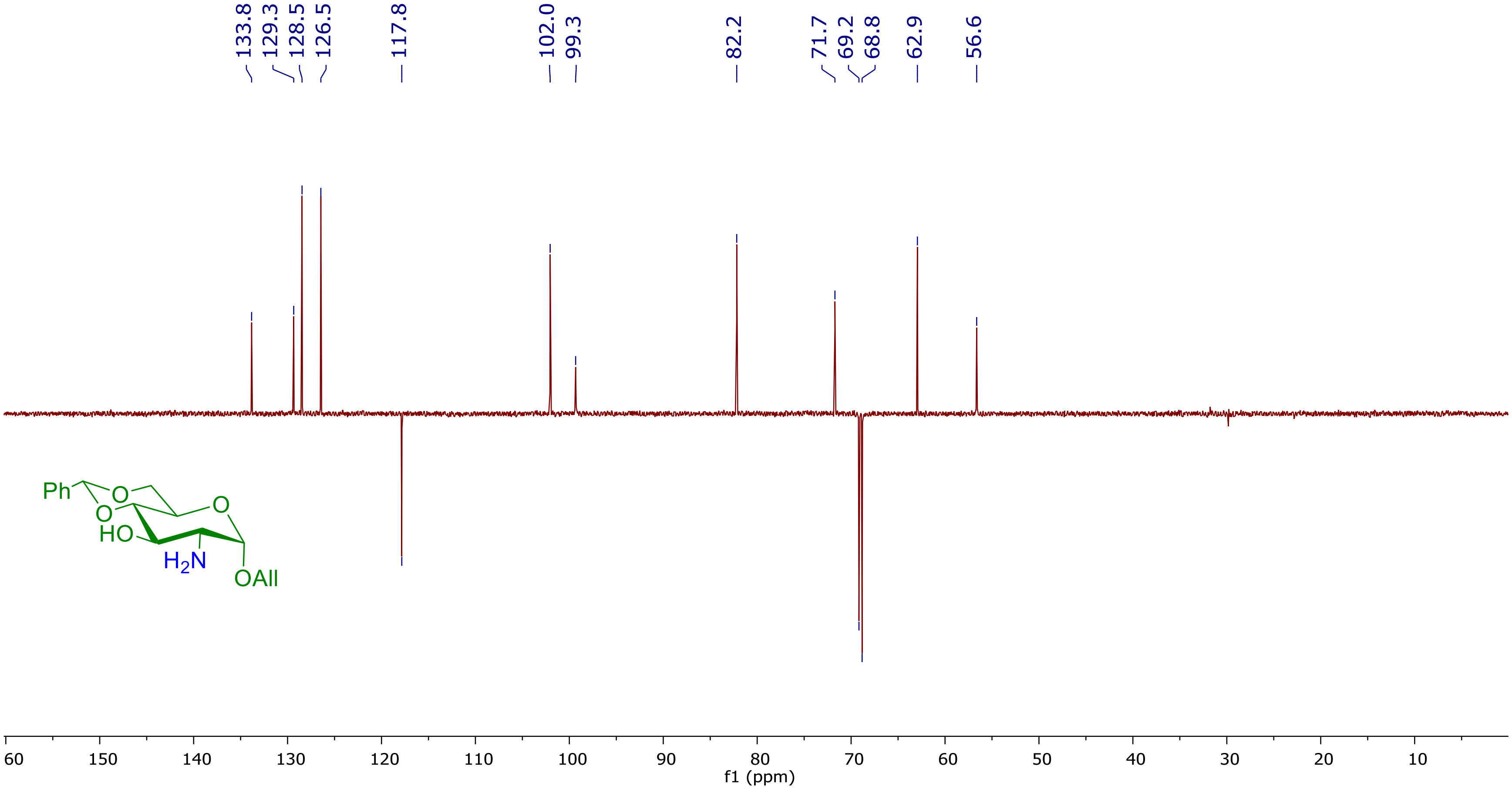

Supplementary Figure S27a. ^{1}H NMR Spectrum (400.31 MHz, $CDCl_3$) of Compound 29

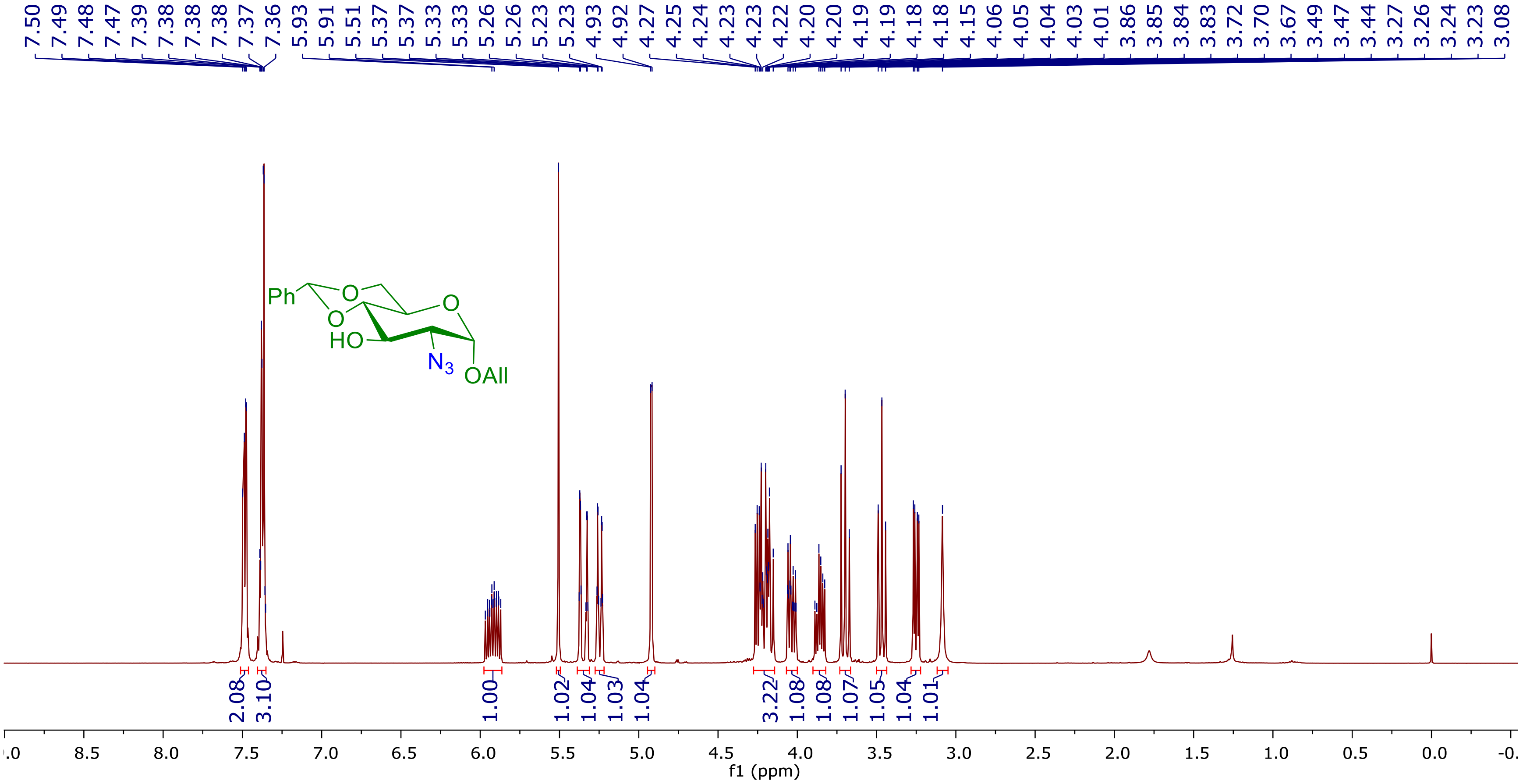

Supplementary Figure S27b. ^{13}C NMR Spectrum (100.67 MHz, $CDCl_3$) of Compound 29

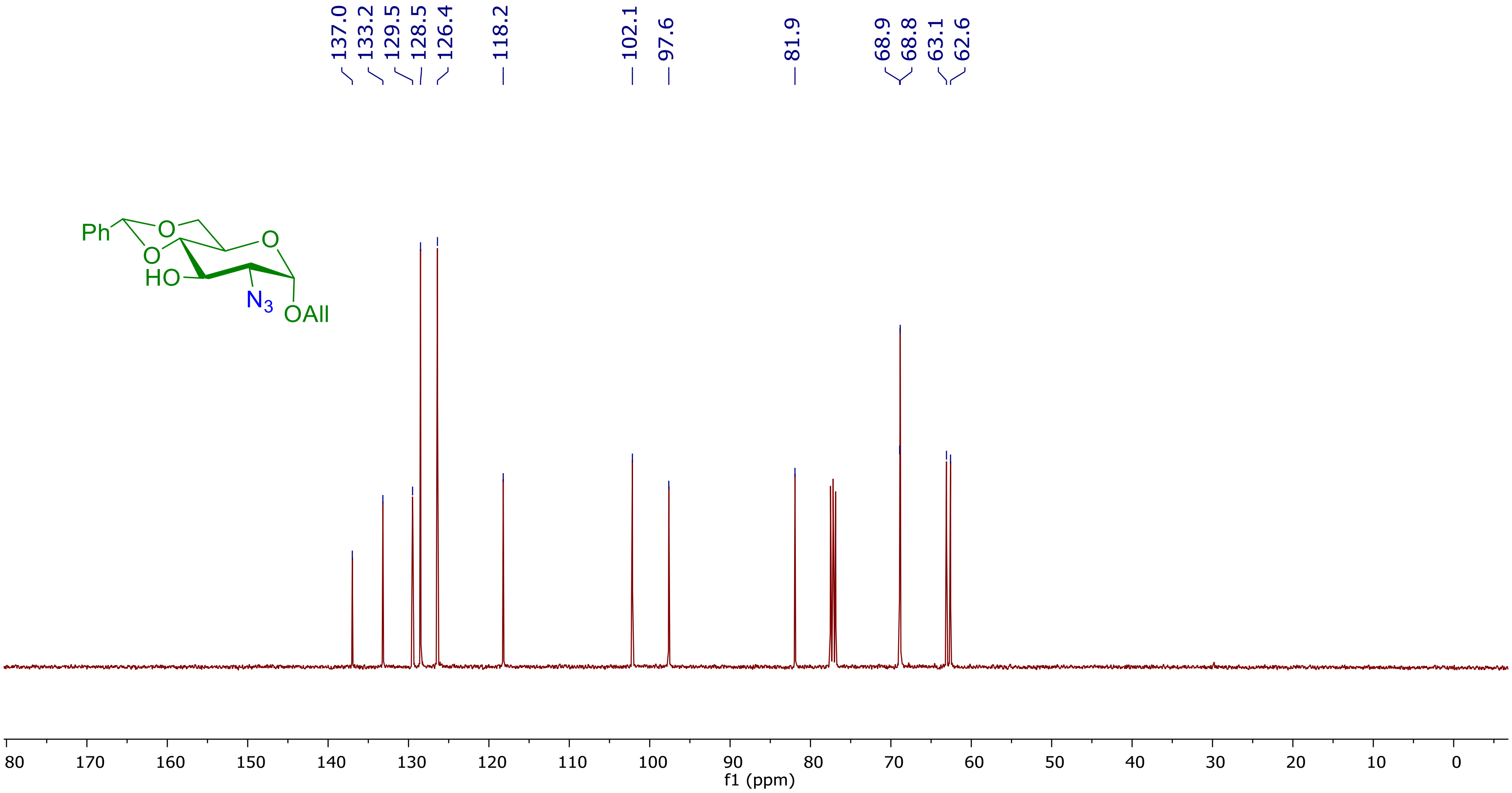

Supplementary Figure S27c. DEPT NMR Spectrum (100.67 MHz, $CDCl_3$) of Compound 29

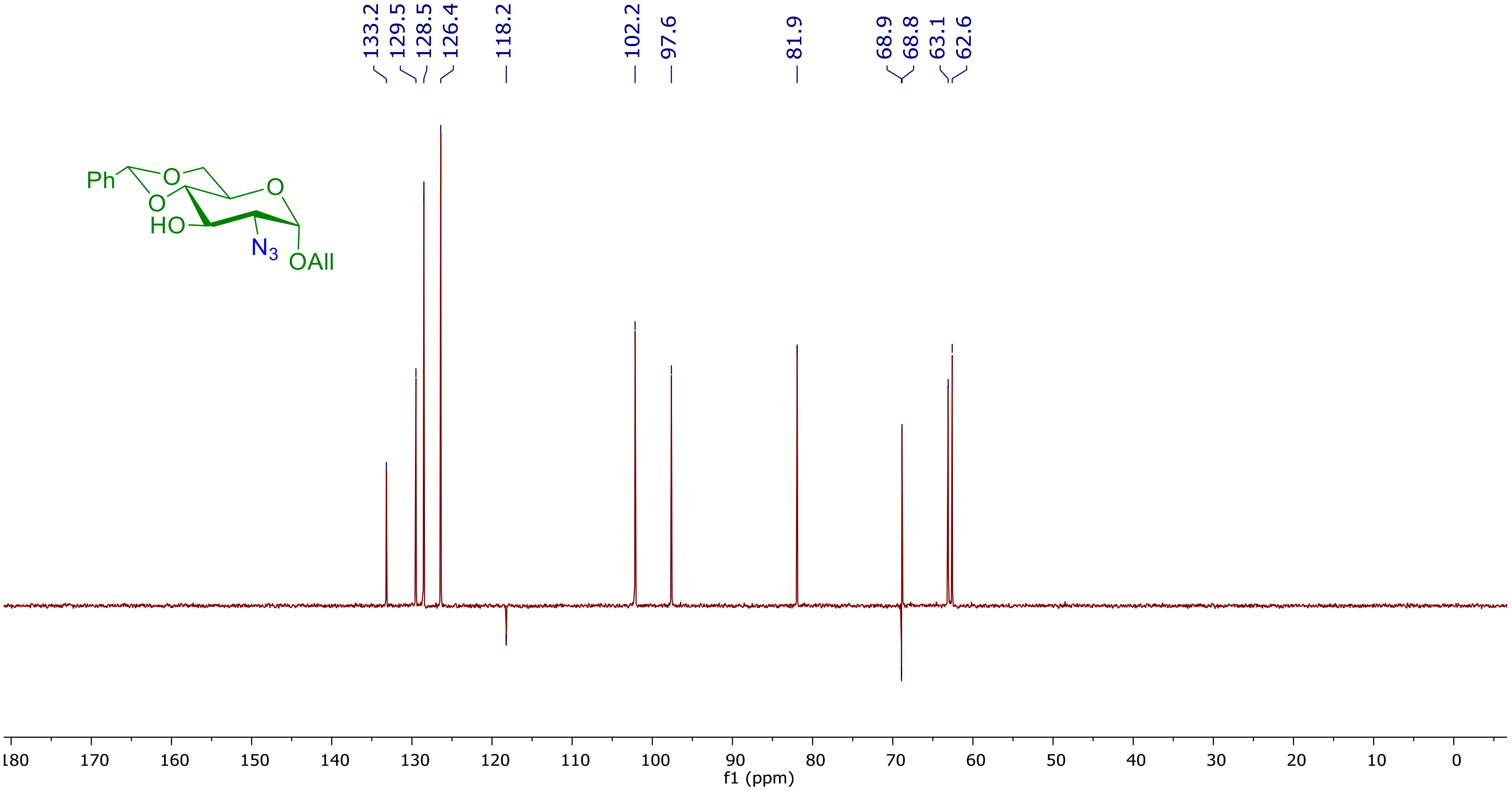

Supplementary Figure S28a. ^{1}H NMR Spectrum (399.78 MHz, $CDCl_3$) of Compound 30a

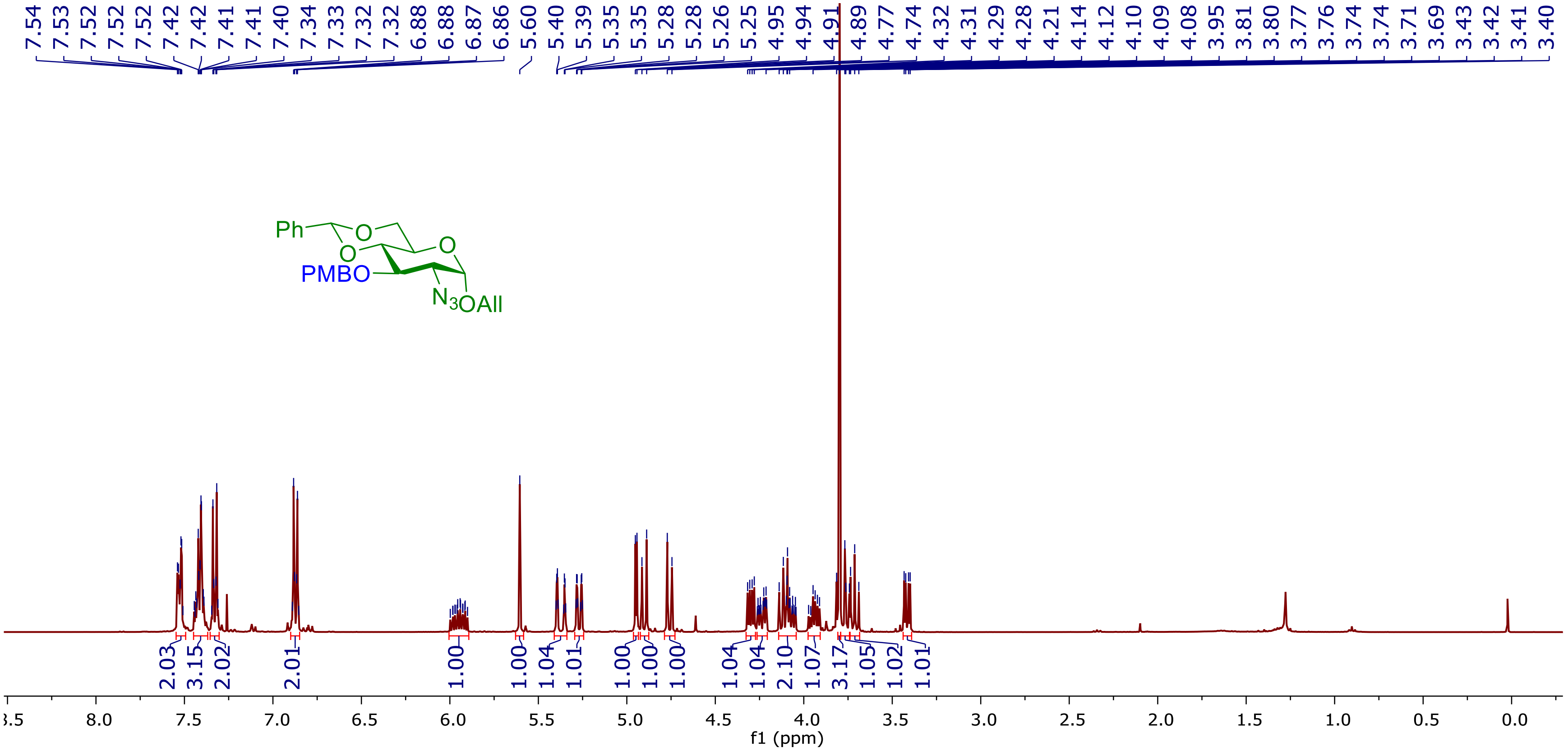

Supplementary Figure S28b. ^{13}C NMR Spectrum (100.53 MHz, $CDCl_3$) of Compound 30a

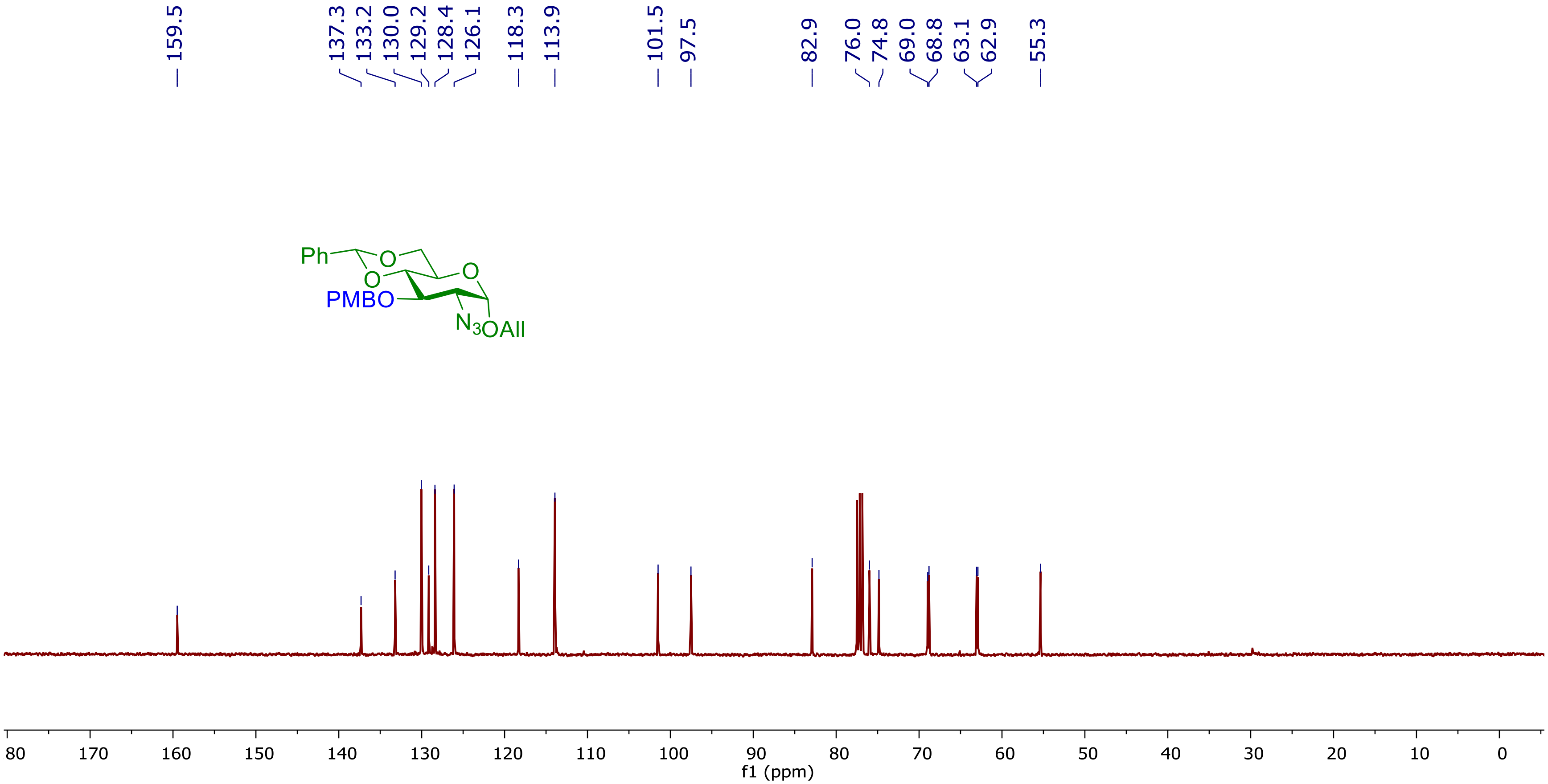

Supplementary Figure S28c. DEPT NMR Spectrum (100.53 MHz, $CDCl_3$) of Compound 30a

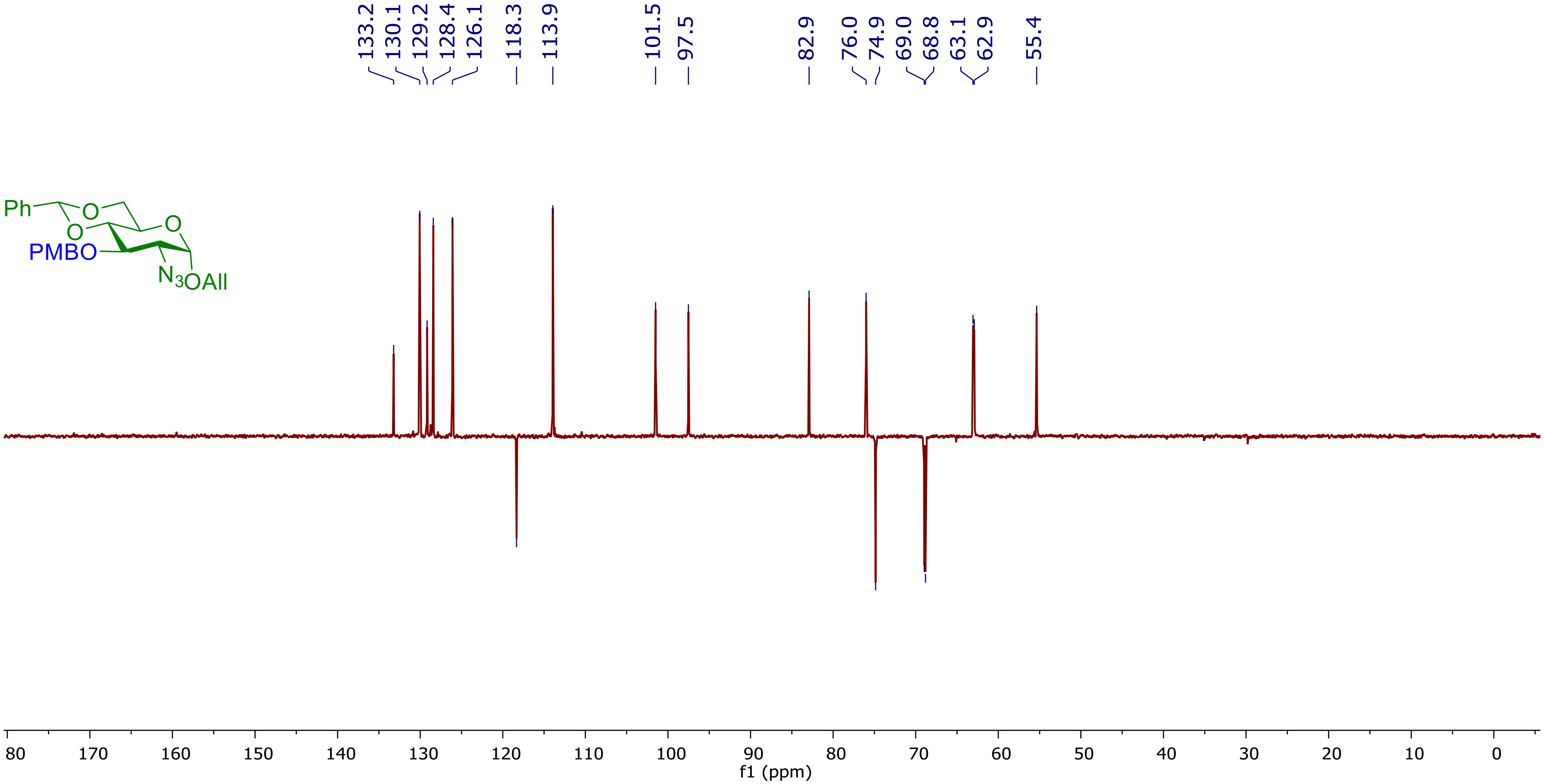

Supplementary Figure S29a. ^{1}H NMR Spectrum (400.31 MHz, $CDCl_3$) of Compound 30b

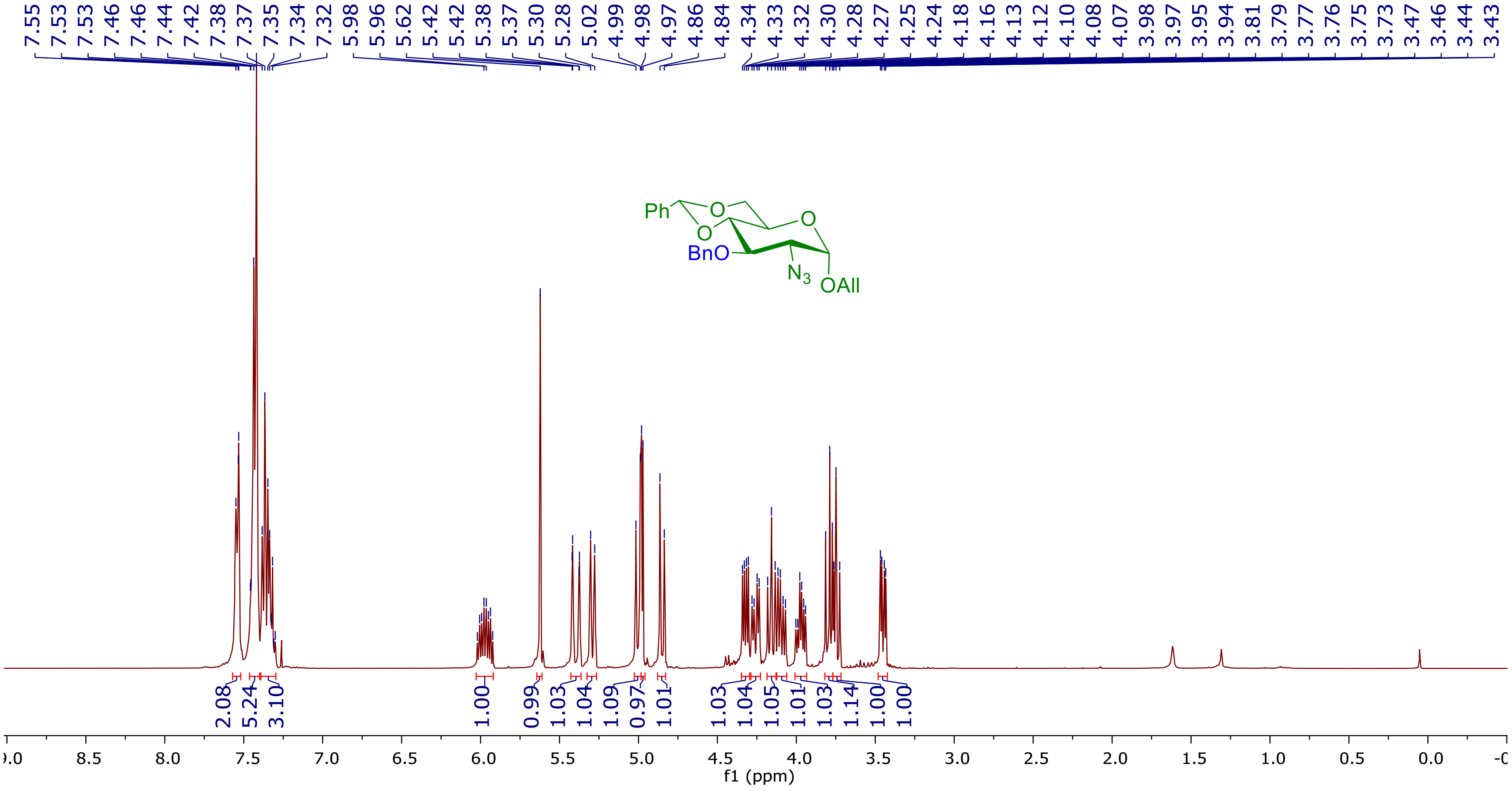

Supplementary Figure S29b. ^{13}C NMR Spectrum (100.67 MHz, $CDCl_3$) of Compound 30b

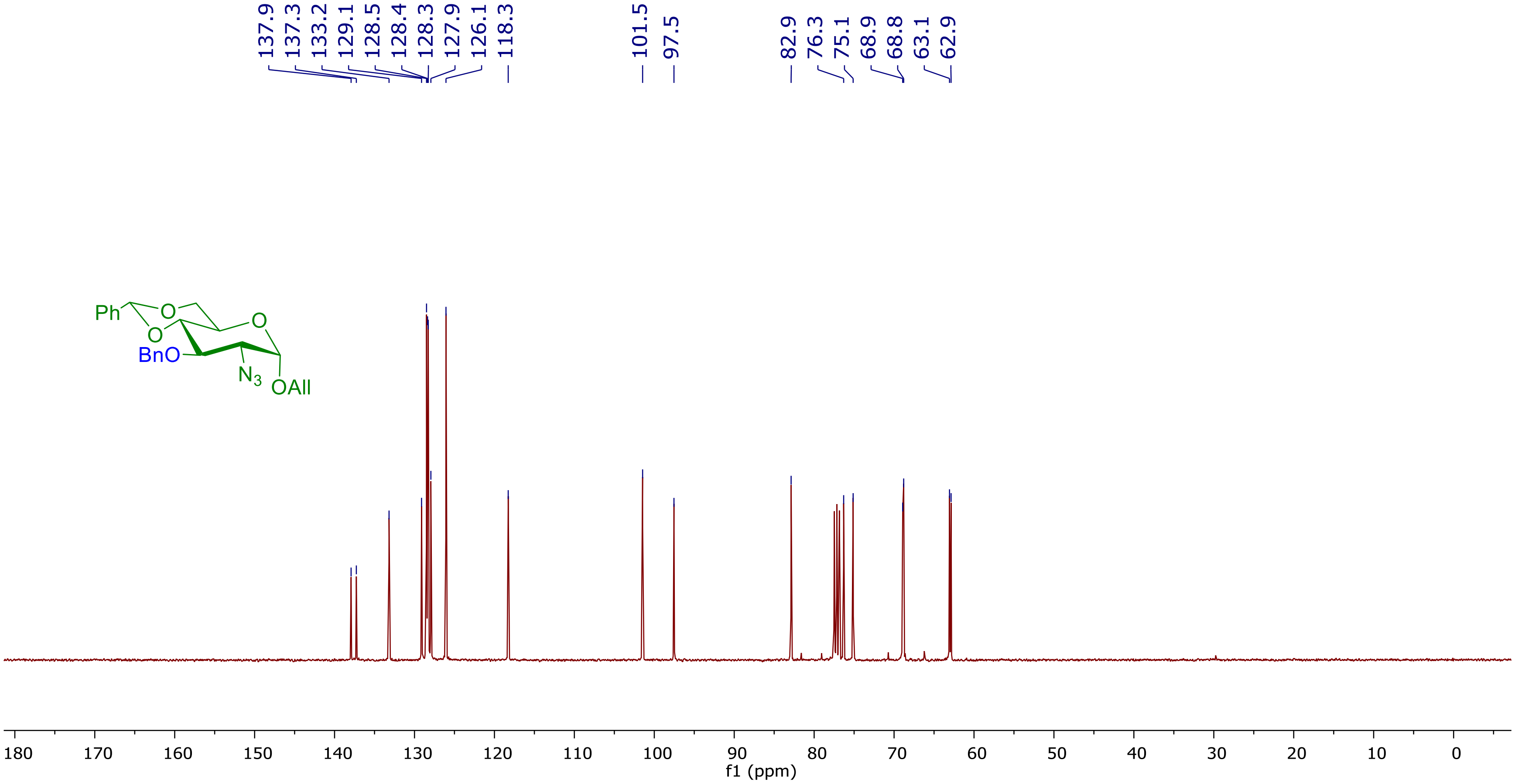

Supplementary Figure S29c. DEPT NMR Spectrum (100.67 MHz, $CDCl_3$) of Compound 30b

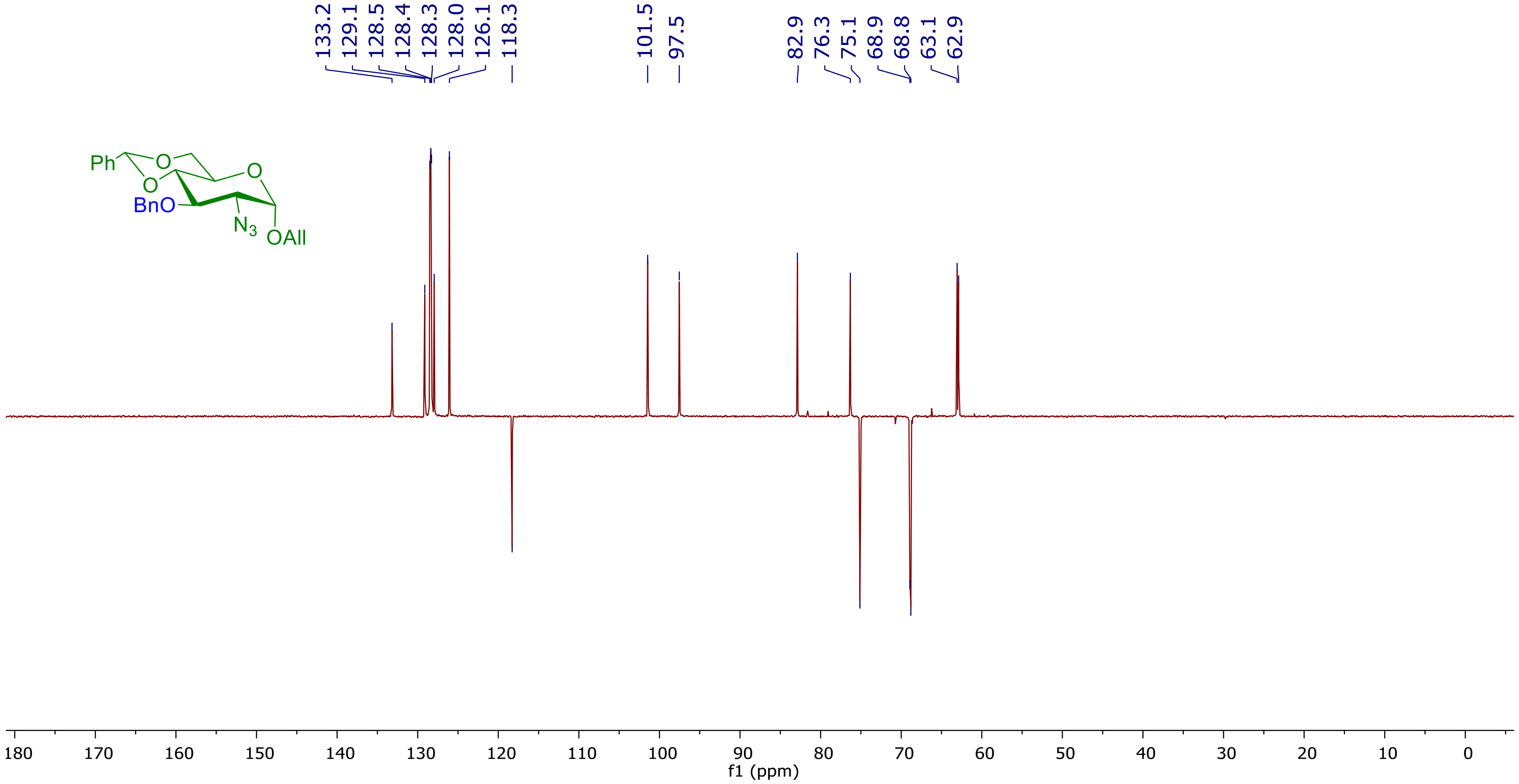

Supplementary Figure S30a. ^{1}H NMR Spectrum (400.31 MHz, $CDCl_3$) of Compound 30d

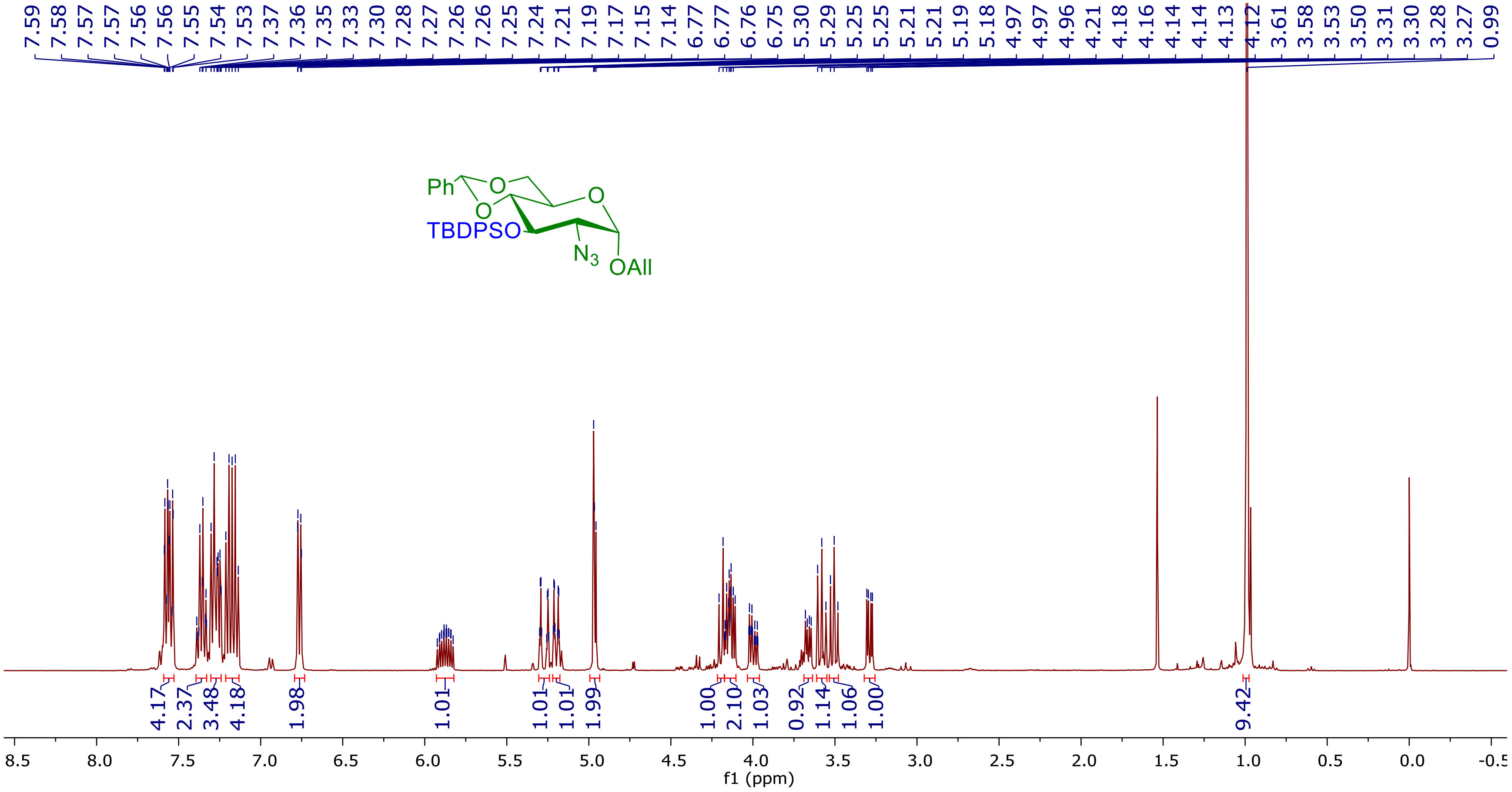

Supplementary Figure S30b. ^{13}C NMR Spectrum (100.67 MHz, $CDCl_3$) of Compound 30d

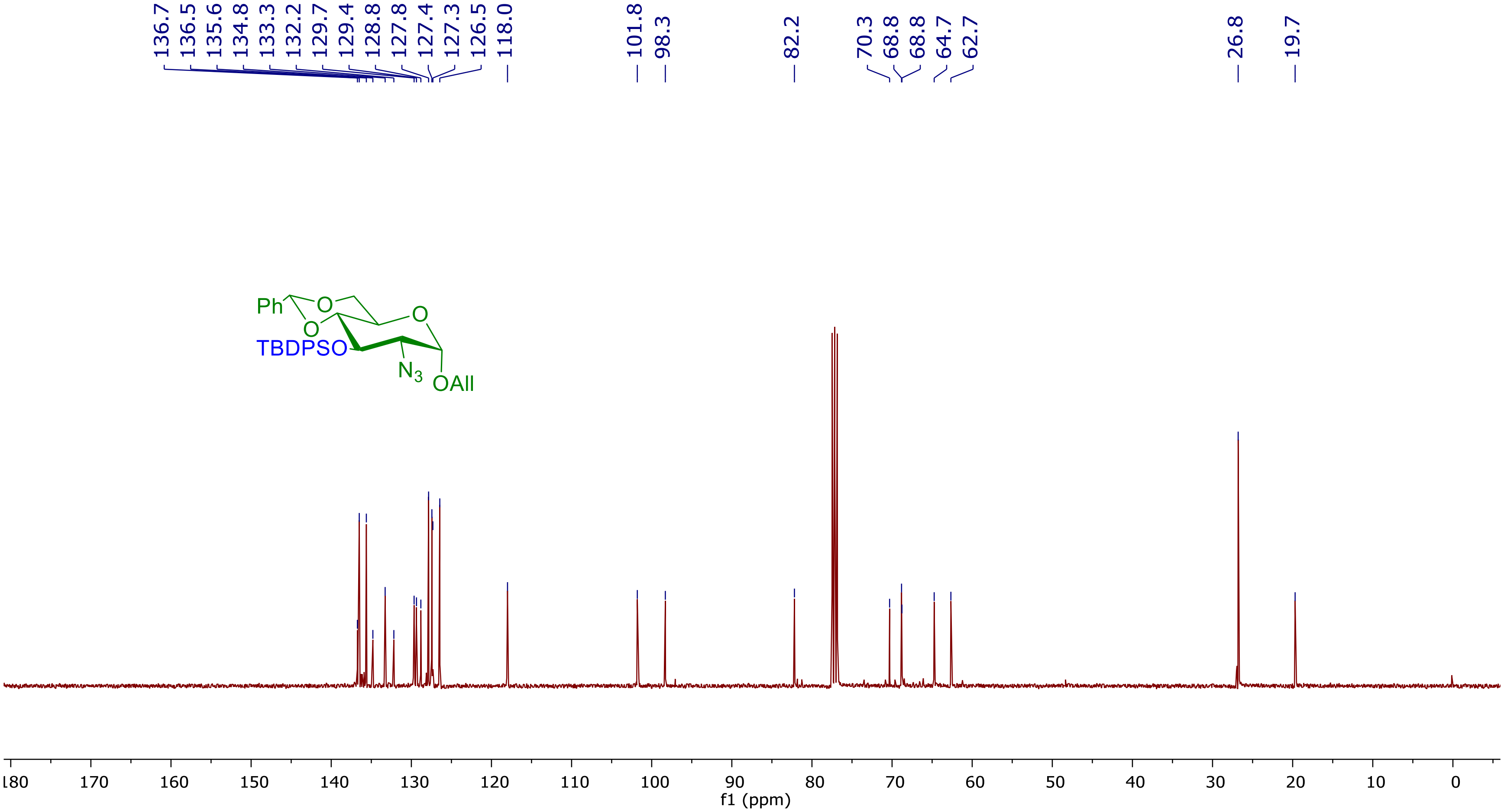

Supplementary Figure S30c. DEPT NMR Spectrum (100.67 MHz, $CDCl_3$) of Compound 30d

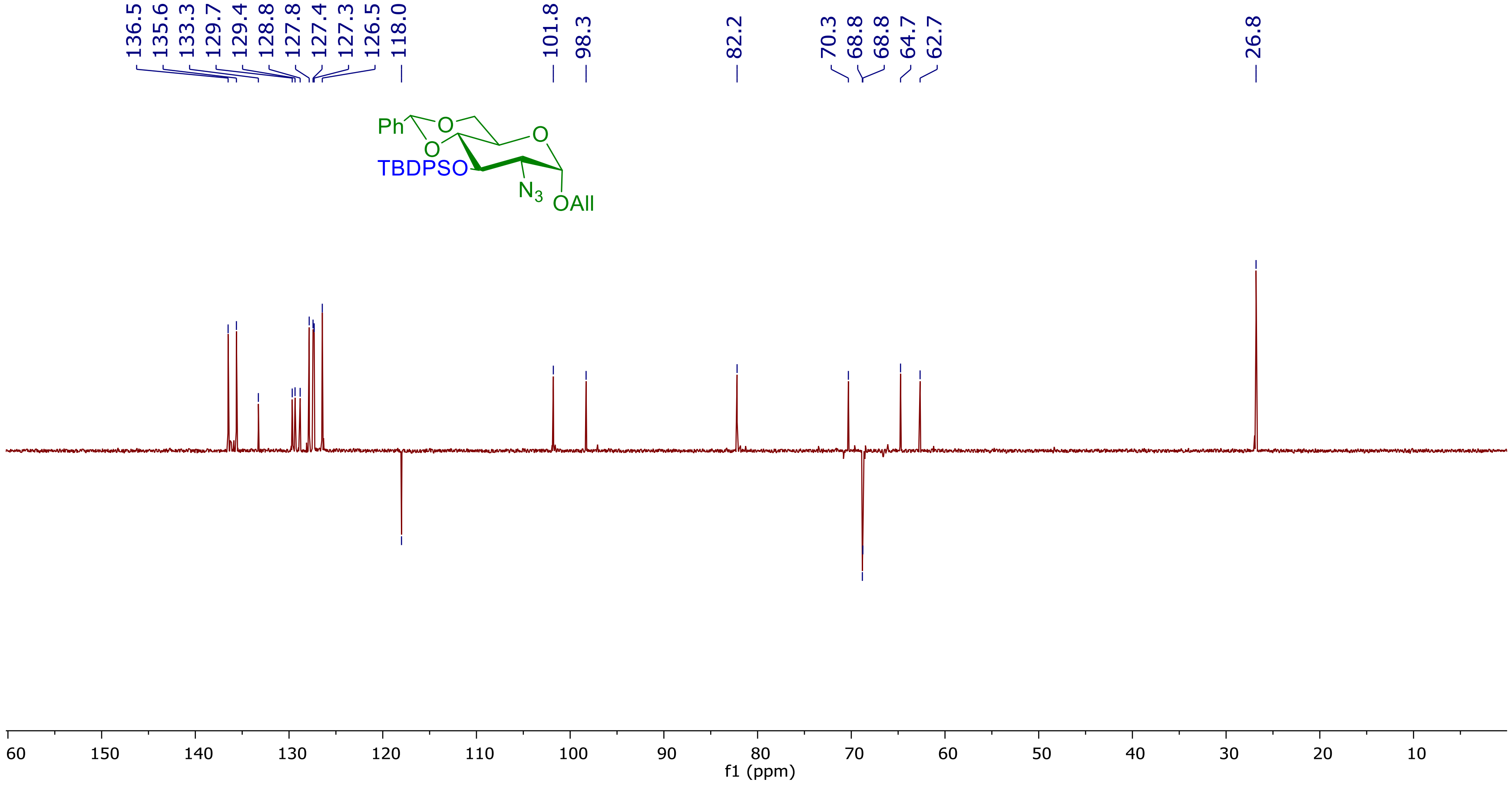

Supplementary Figure S31a. ^{1}H NMR Spectrum (400.31 MHz, $CDCl_3$) of Compound 30e

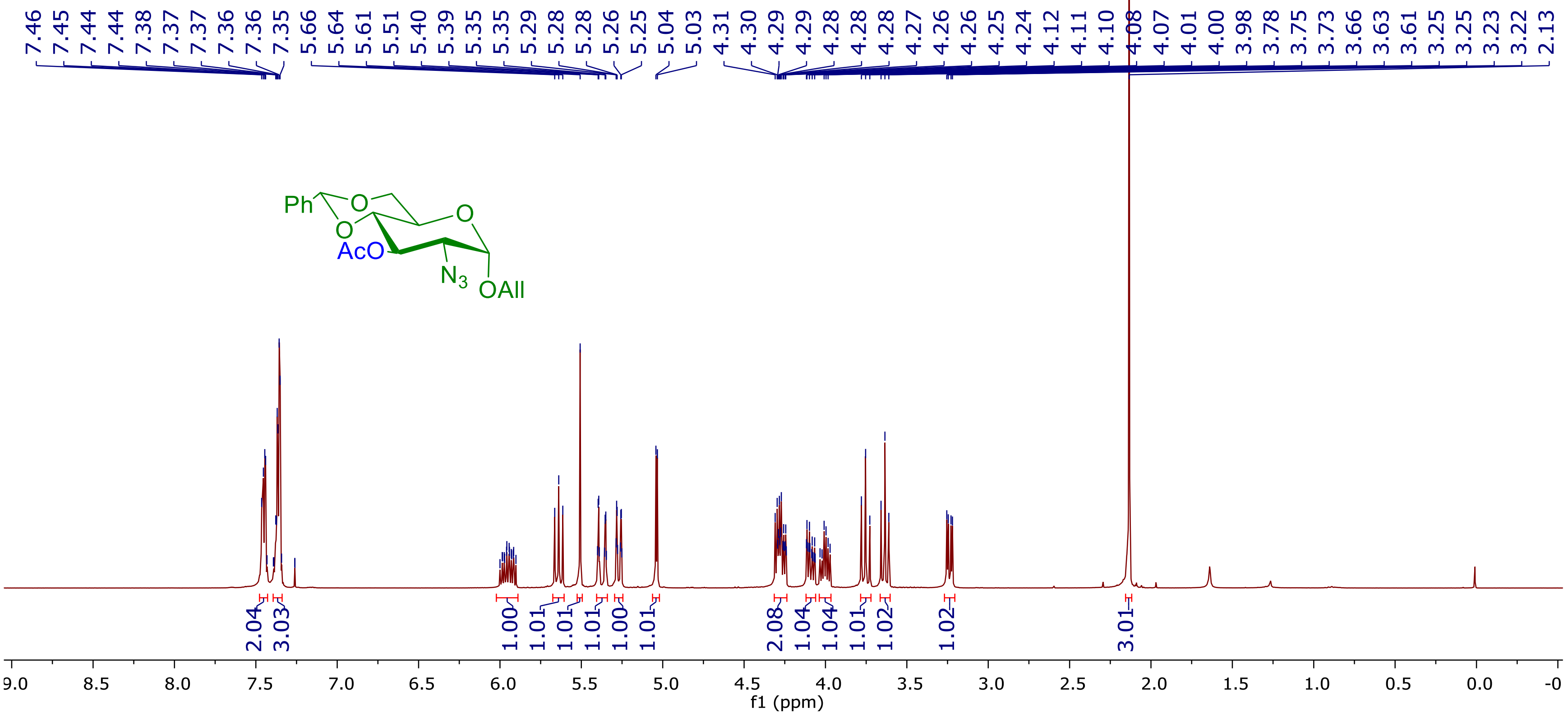

Supplementary Figure S31b. ^{13}C NMR Spectrum (100.67 MHz, $CDCl_3$) of Compound 30e

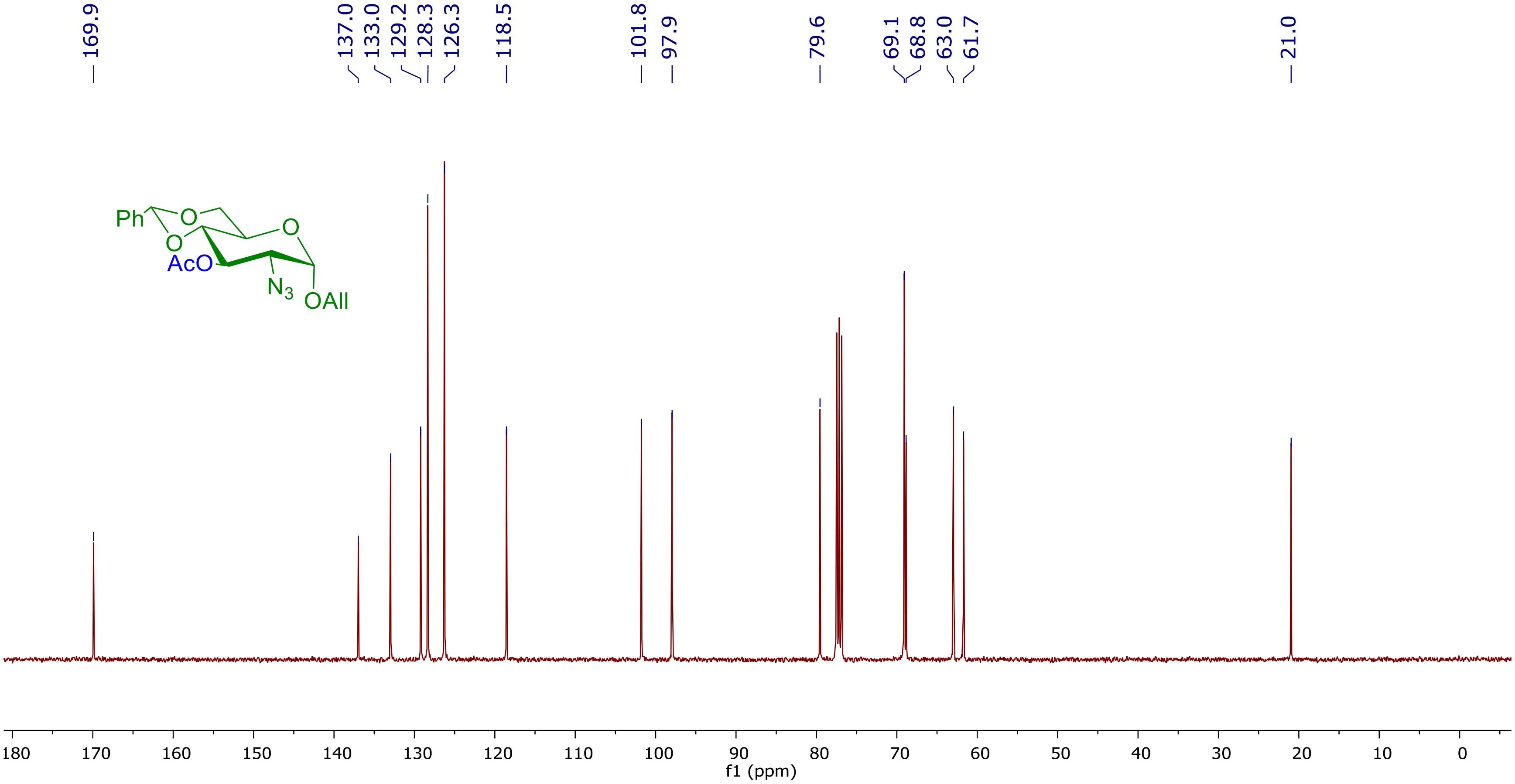

Supplementary Figure S31c. DEPT NMR Spectrum (100.67 MHz, $CDCl_3$) of Compound 30e

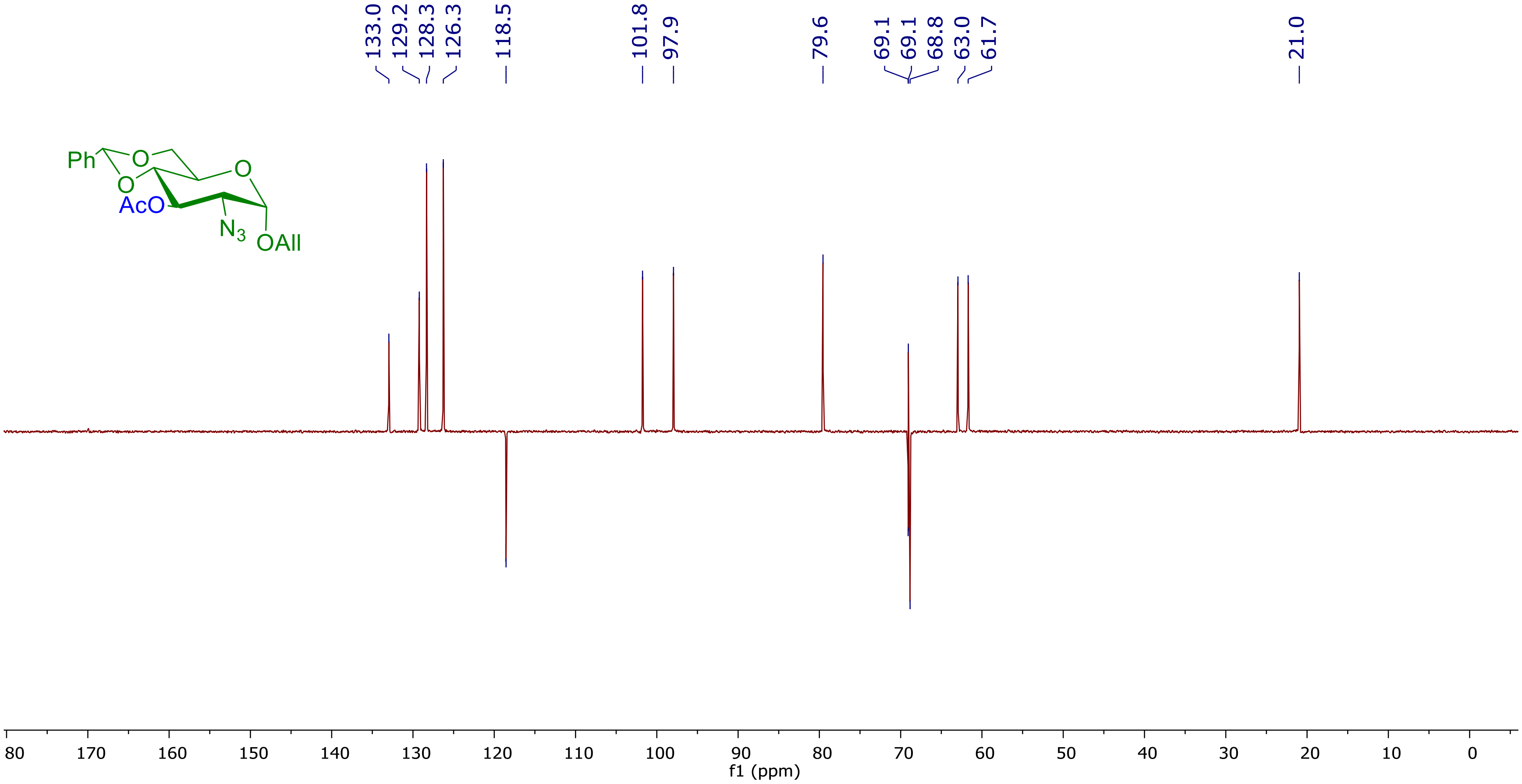

Supplementary Figure S32a. ^{1}H NMR Spectrum (400.31 MHz, $CDCl_3$) of Compound S7a

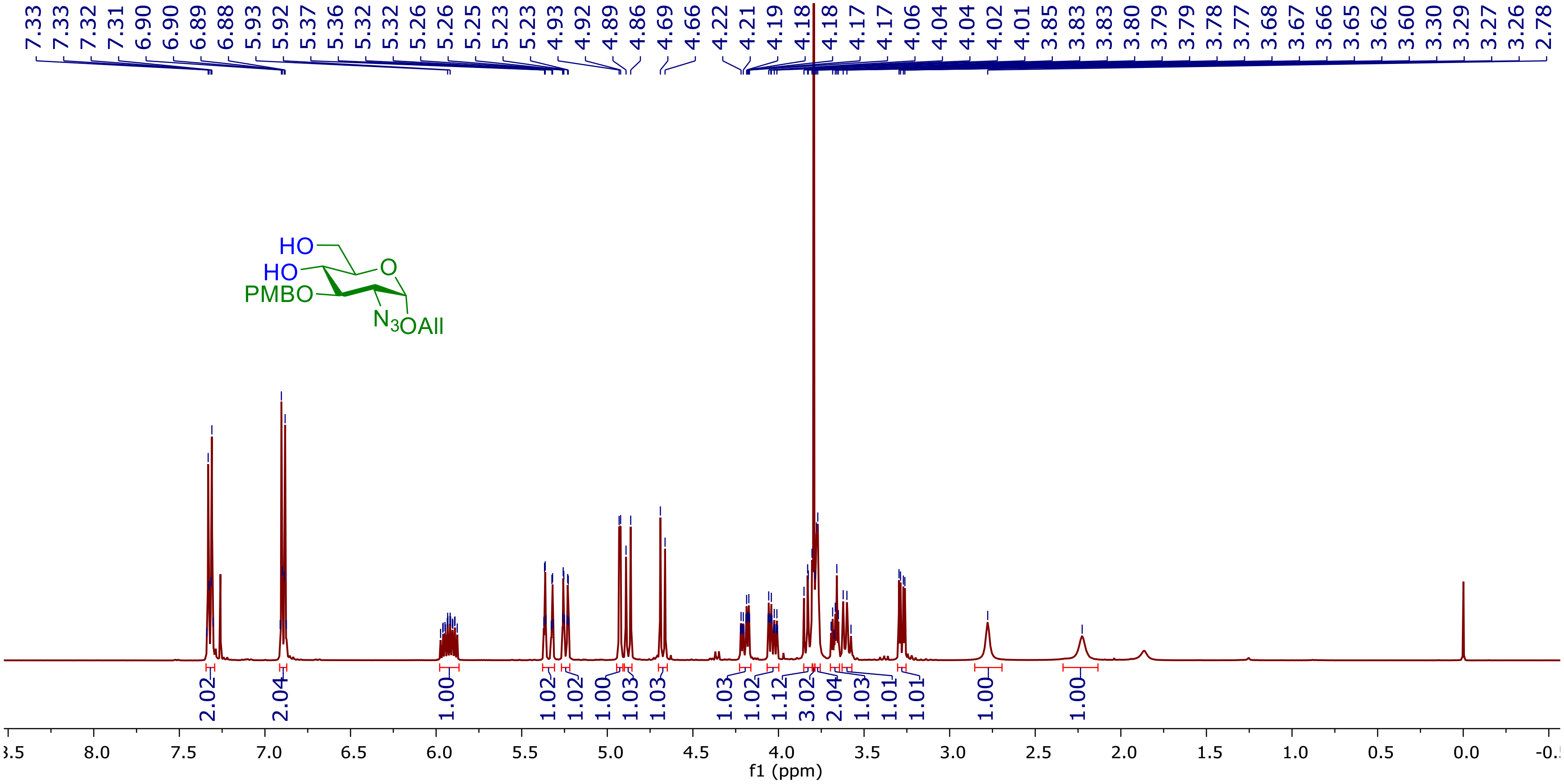

Supplementary Figure S32b. ^{13}C NMR Spectrum (100.67 MHz, $CDCl_3$) of Compound S7a

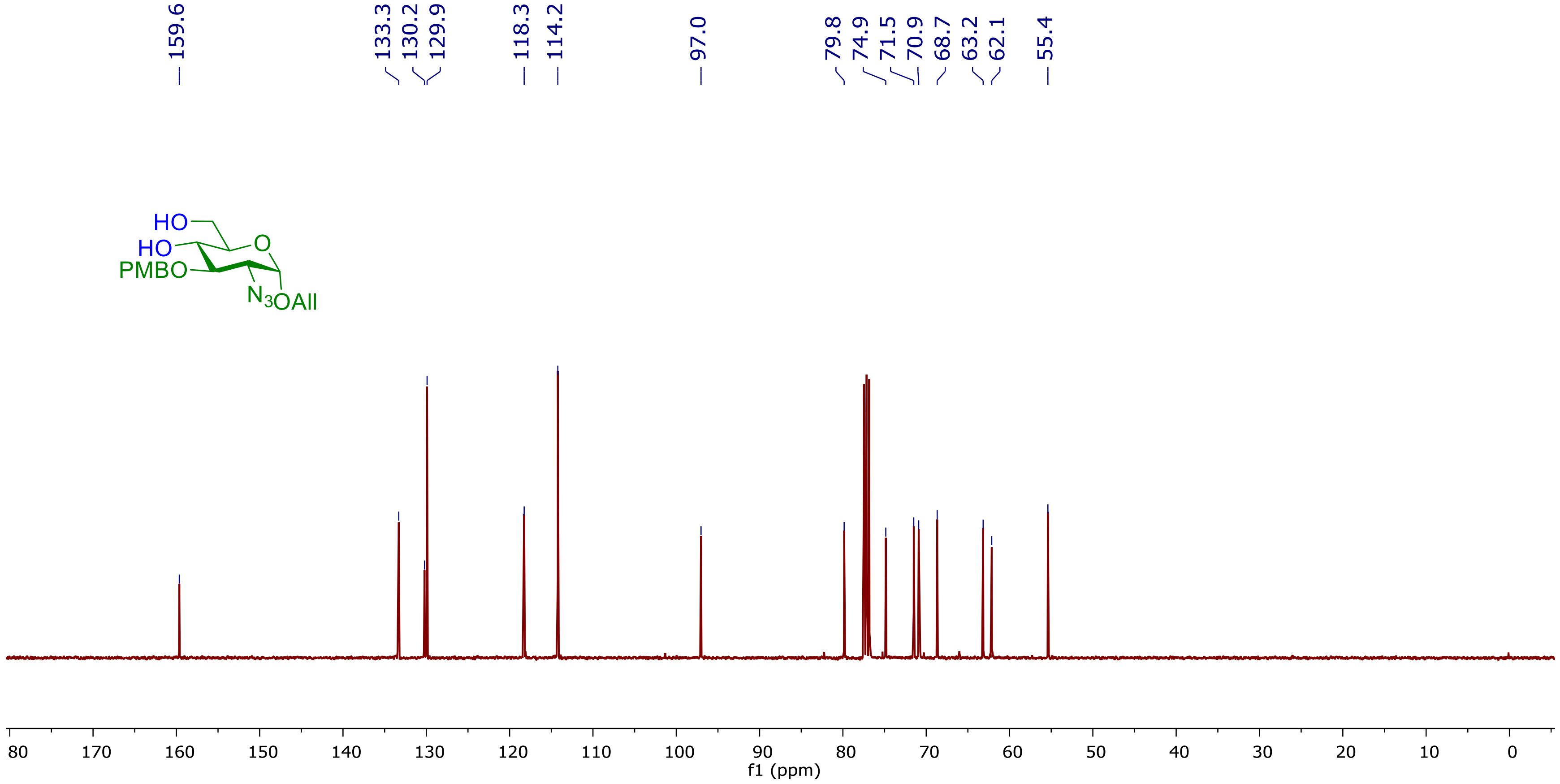

Supplementary Figure S32c. DEPT NMR Spectrum (100.67 MHz, $CDCl_3$) of Compound S7a

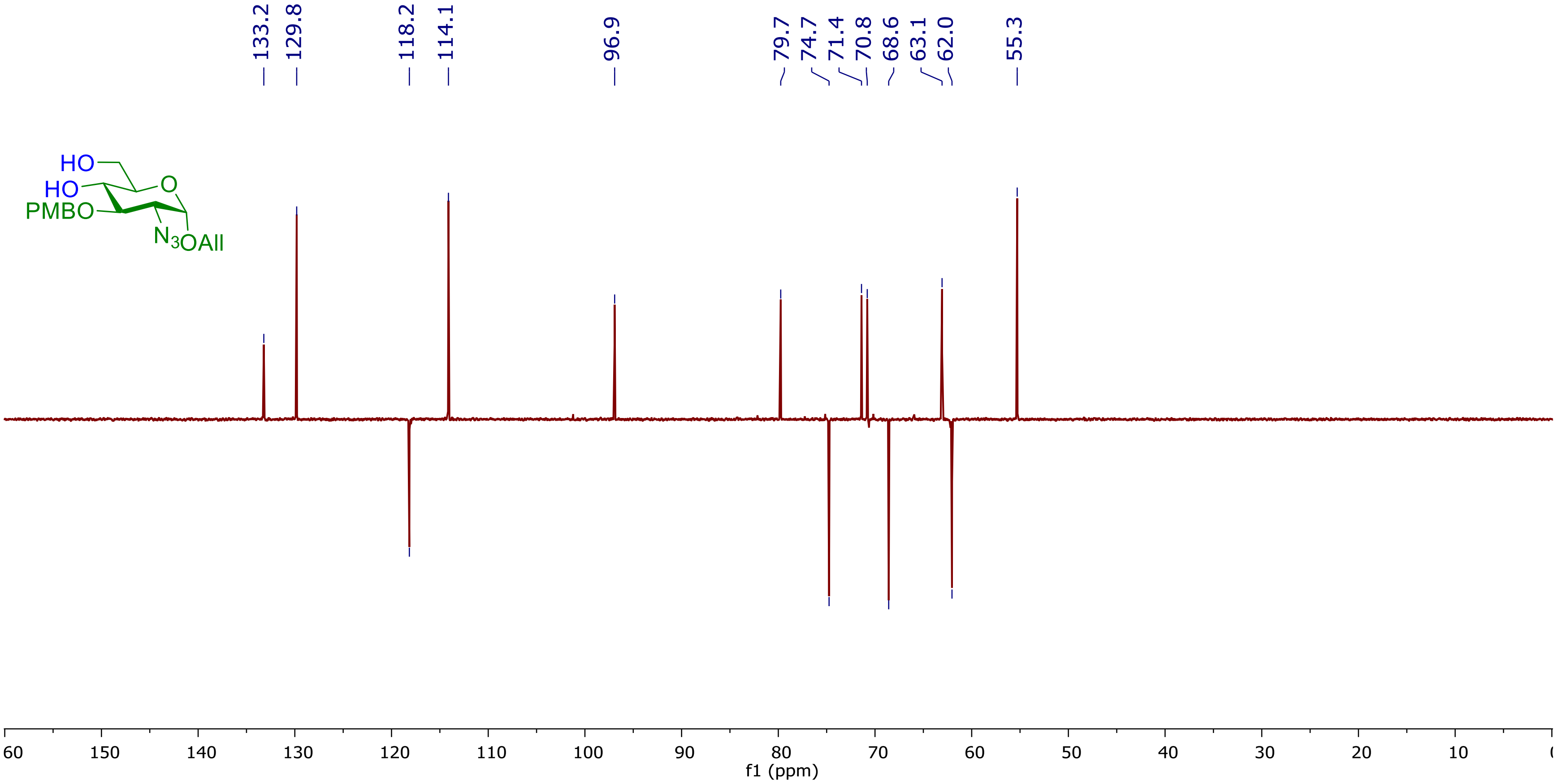

Supplementary Figure S33a. ¹H NMR Spectrum (400.31 MHz, $CDCl_3$) of Compound S7b

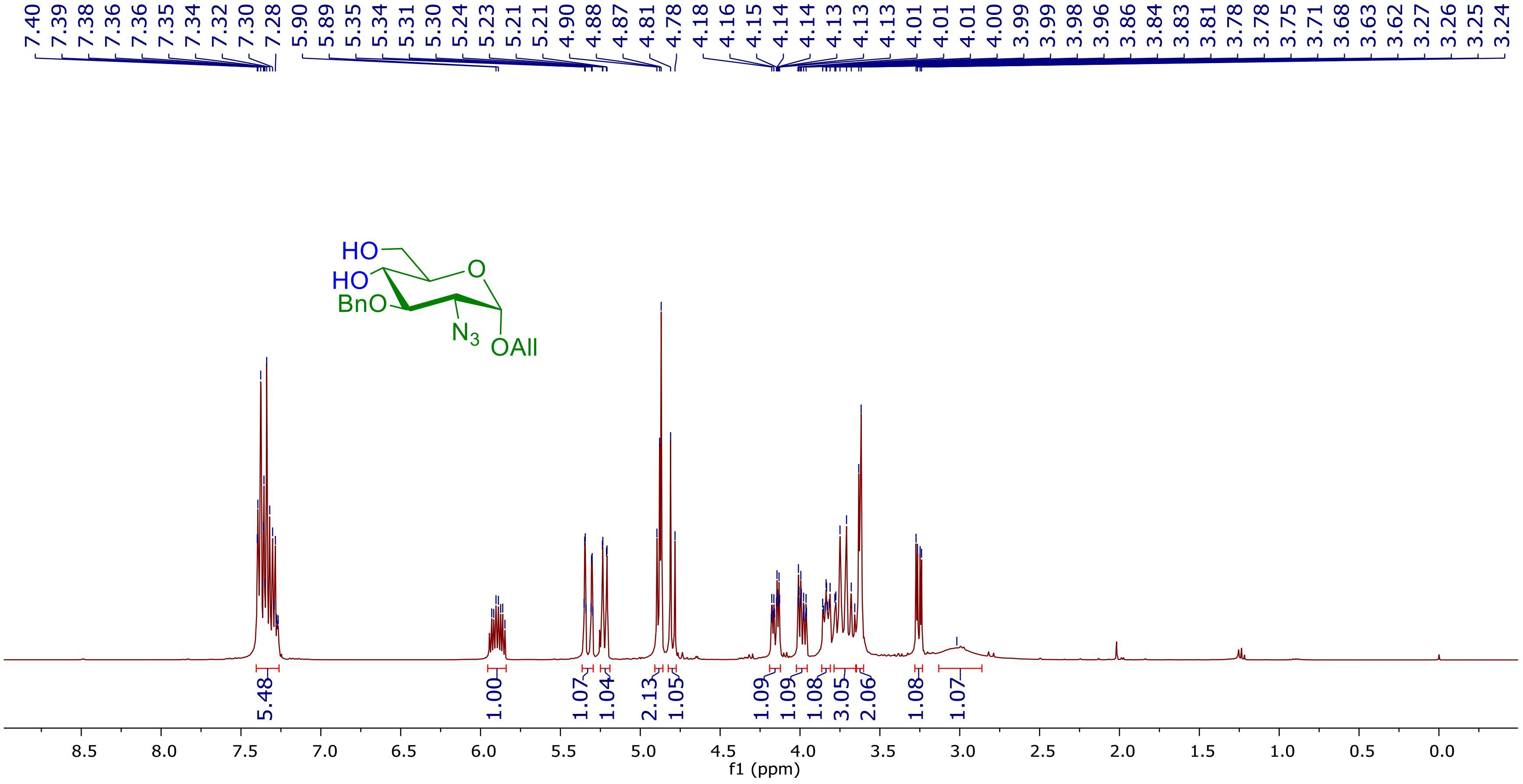

Supplementary Figure S33b. ^{13}C NMR Spectrum (100.67 MHz, $CDCl_3$) of Compound S7b

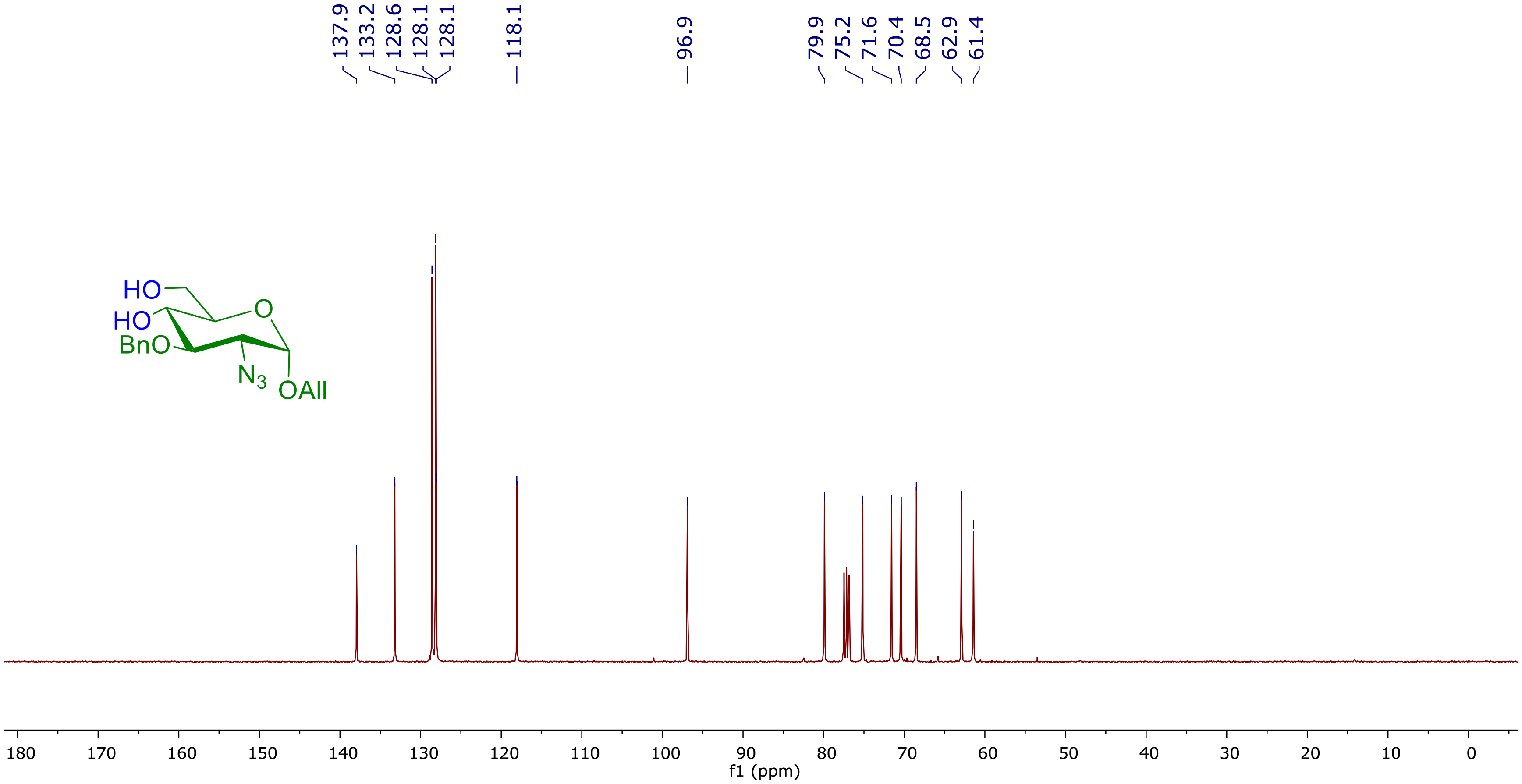

Supplementary Figure S33c. DEPT NMR Spectrum (100.67 MHz, $CDCl_3$) of Compound S7b

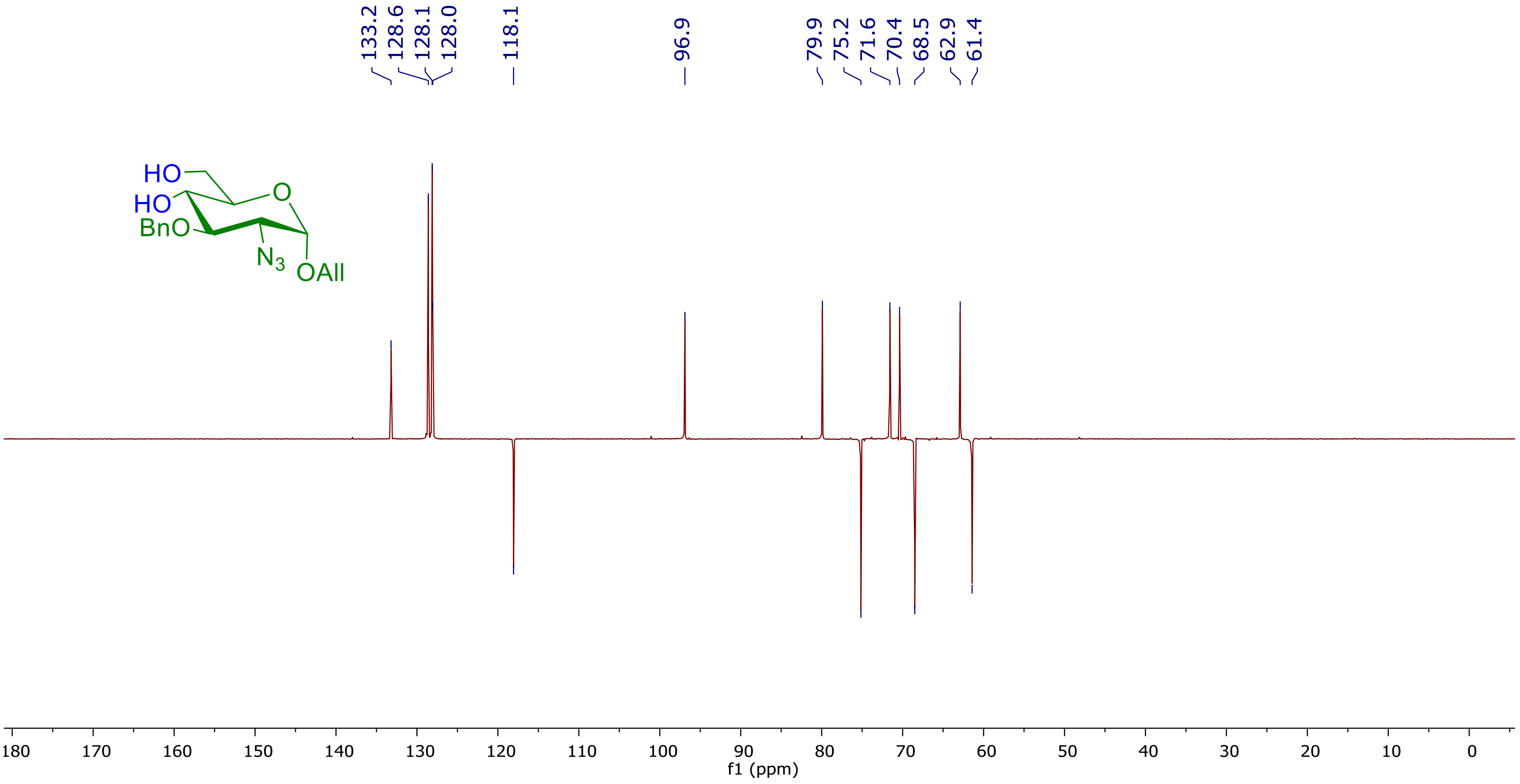

Supplementary Figure S34a. ^{1}H NMR Spectrum (400.31 MHz, $CDCl_3$) of Compound S7c

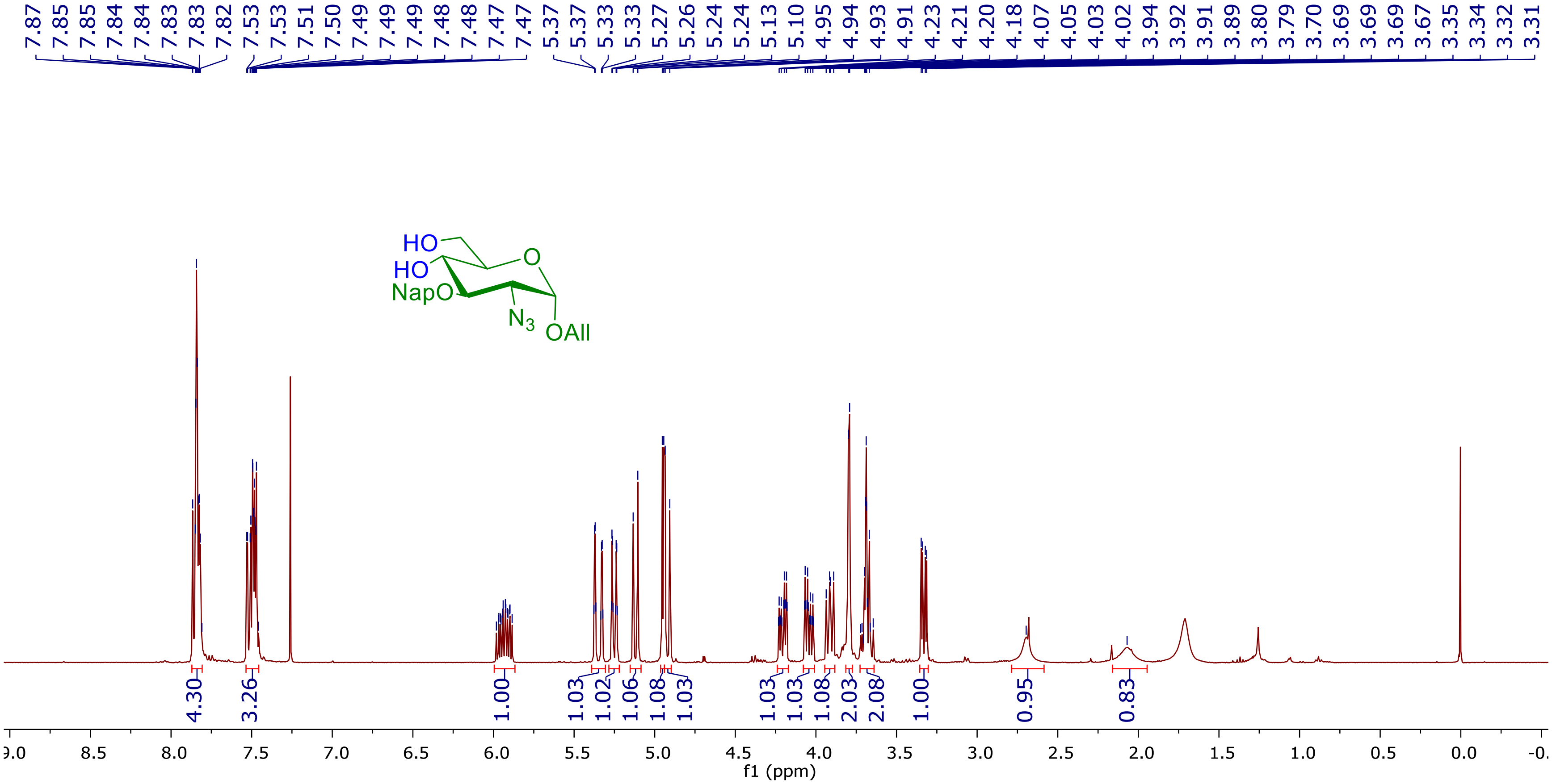

Supplementary Figure S34b. ^{13}C NMR Spectrum (100.67 MHz, $CDCl_3$) of Compound S7c

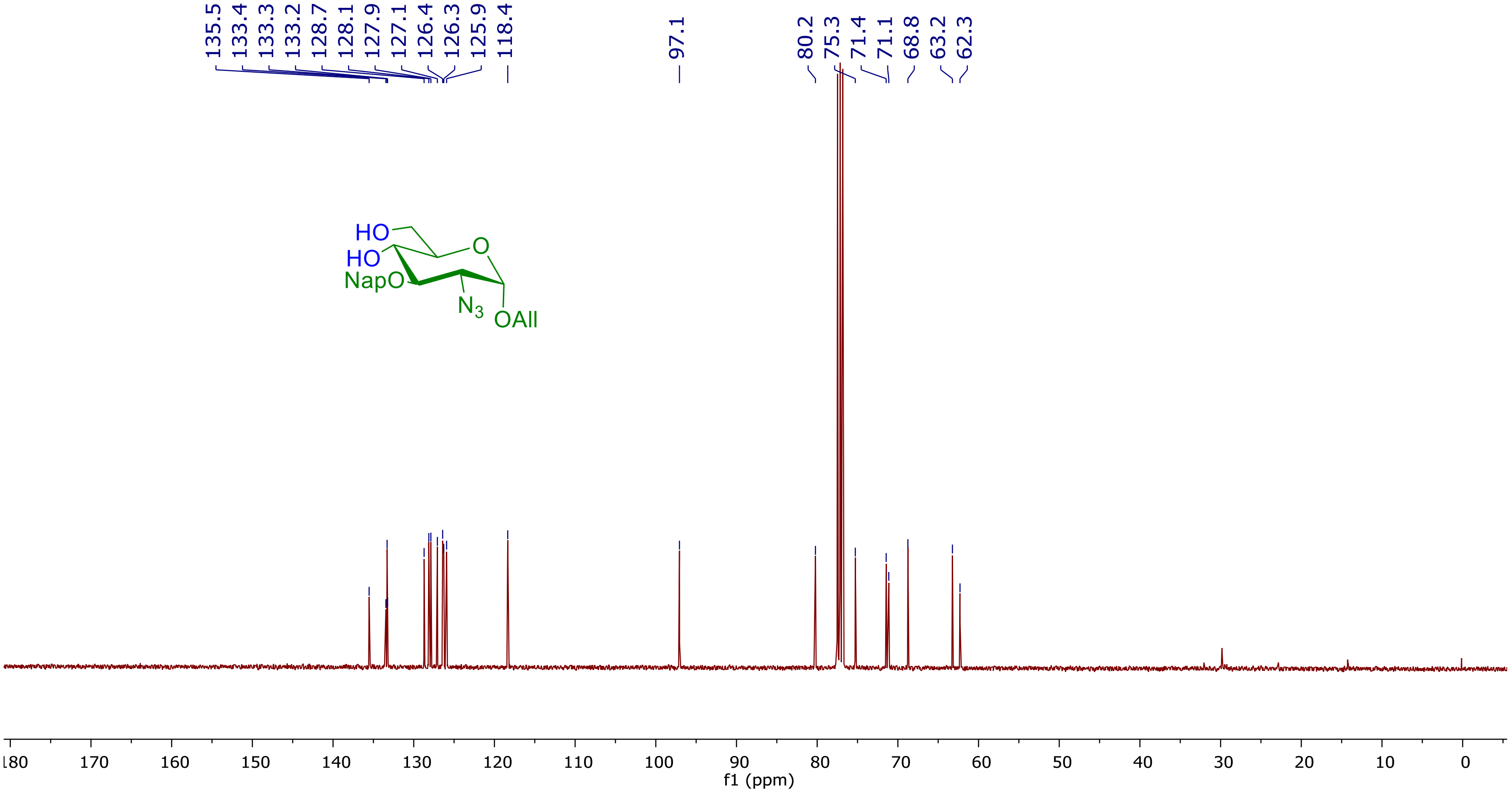

Supplementary Figure S34c. DEPT NMR Spectrum (100.67 MHz, $CDCl_3$) of Compound S7c

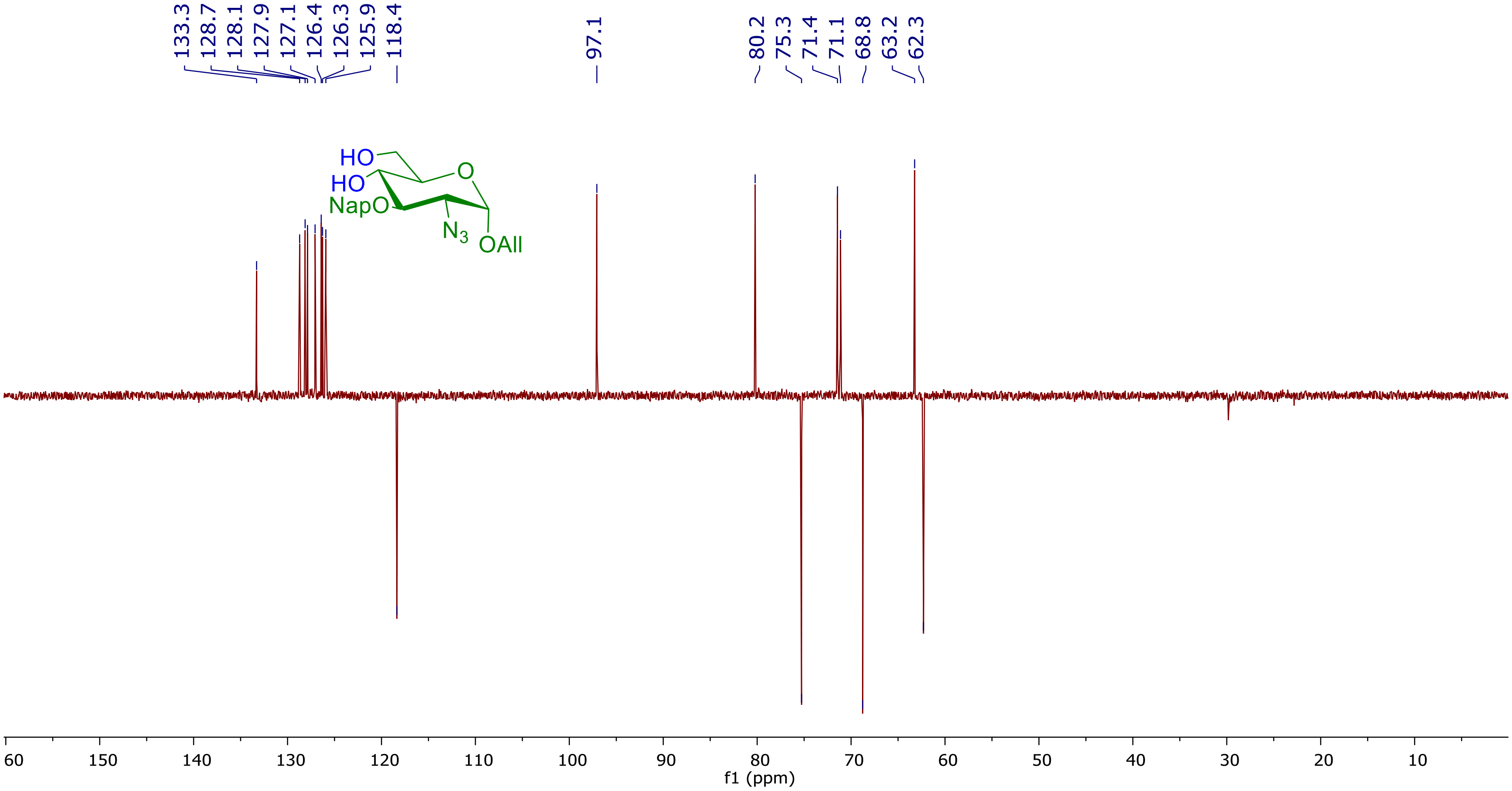

Supplementary Figure S35a. ^{1}H NMR Spectrum (400.31 MHz, $CDCl_3$) of Compound S7d

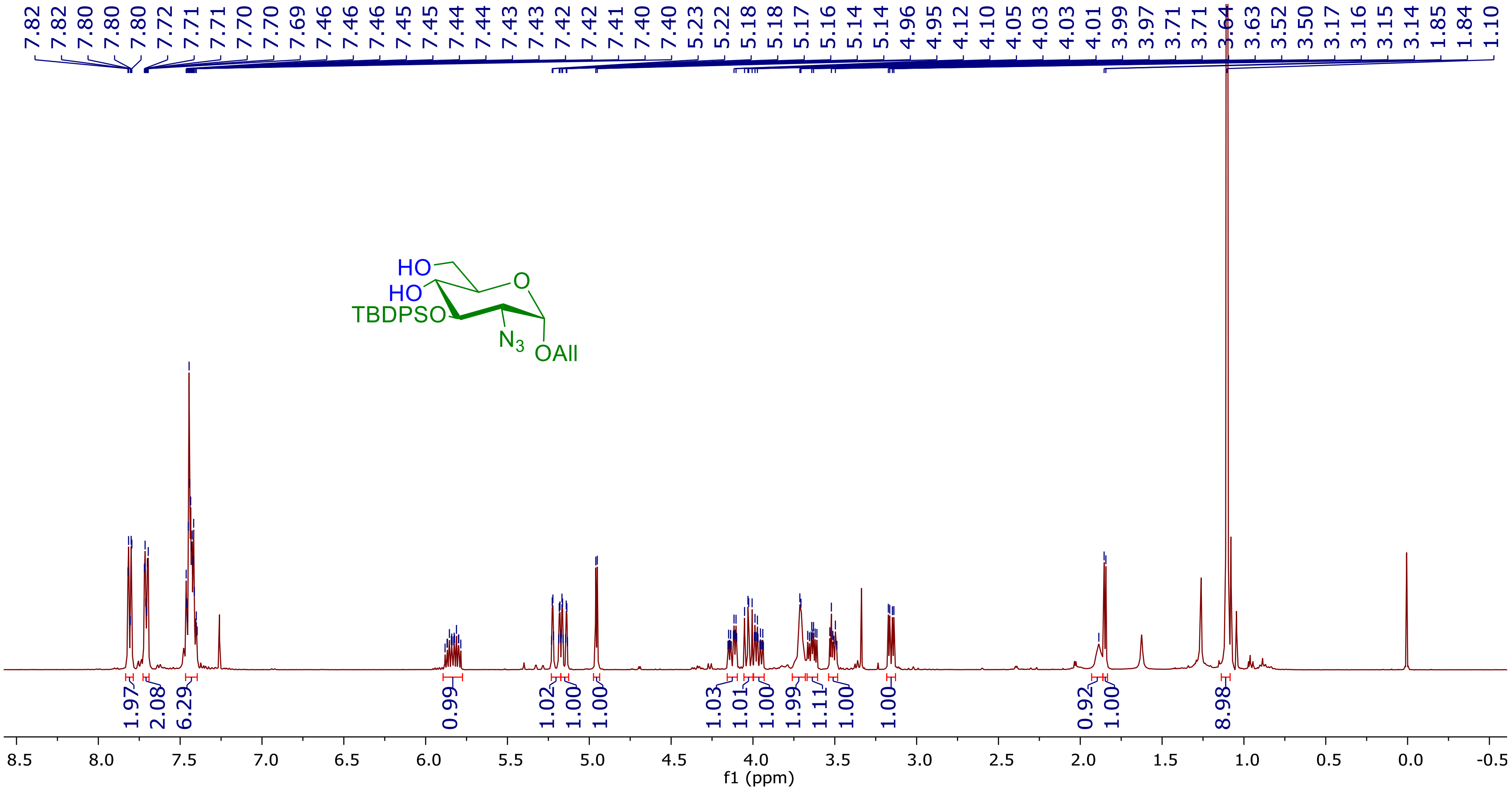

Supplementary Figure S35b. ^{13}C NMR Spectrum (100.67 MHz, $CDCl_3$) of Compound S7d

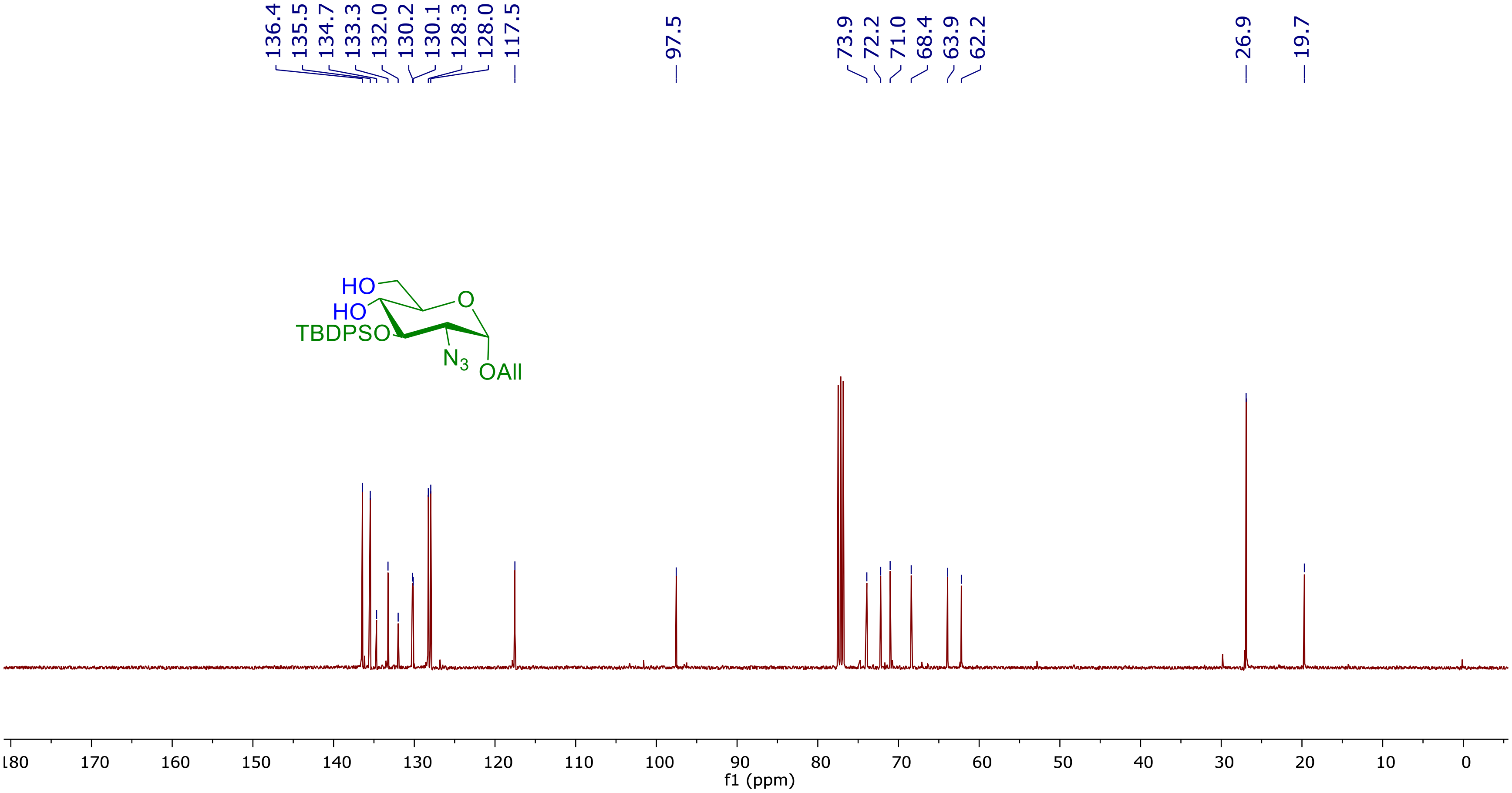

Supplementary Figure S35c. DEPT NMR Spectrum (100.67 MHz, CDCl$_3$) of Compound S7d

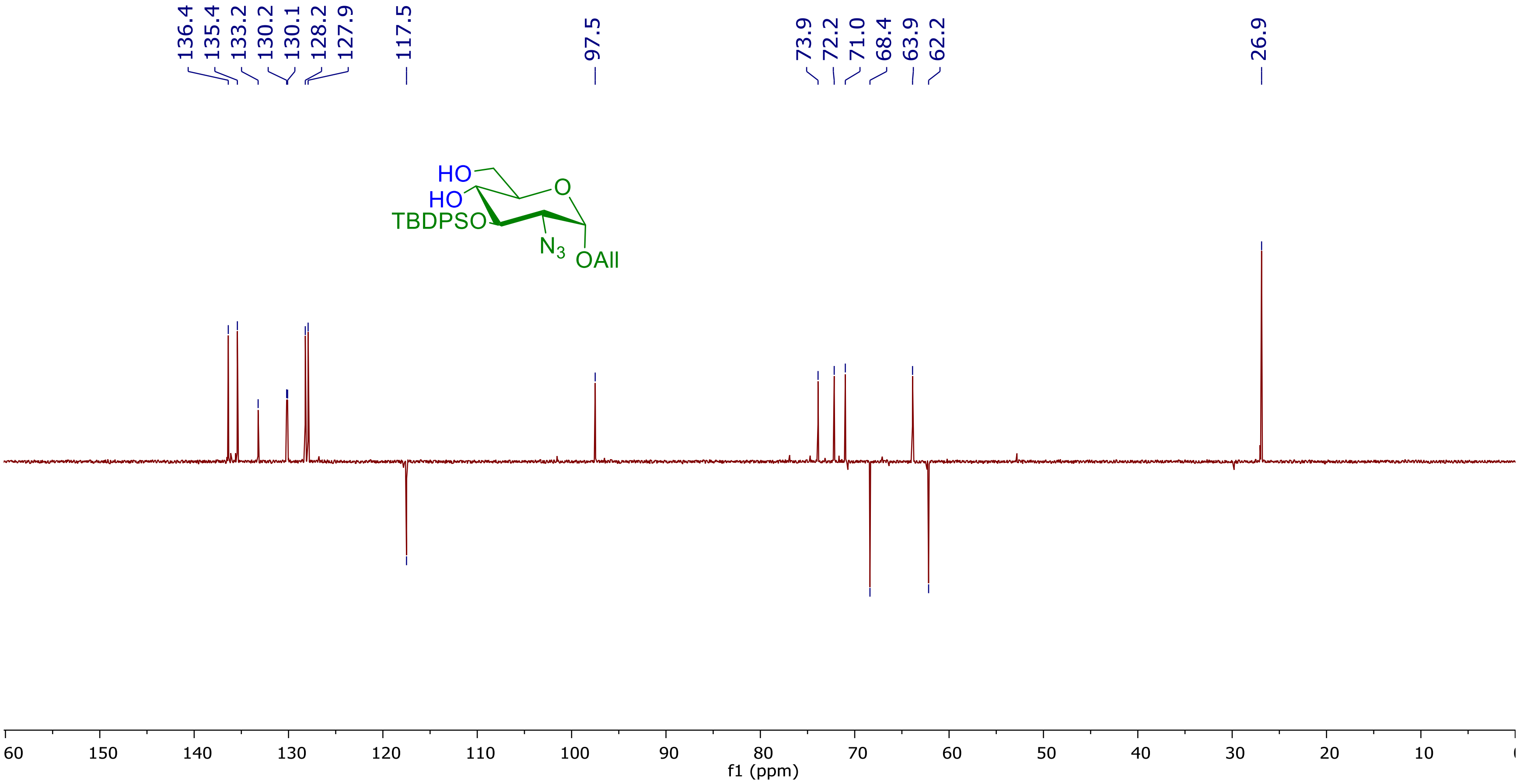

Supplementary Figure S36a. ^{1}H NMR Spectrum (400.31 MHz, $CDCl_3$) of Compound S7e

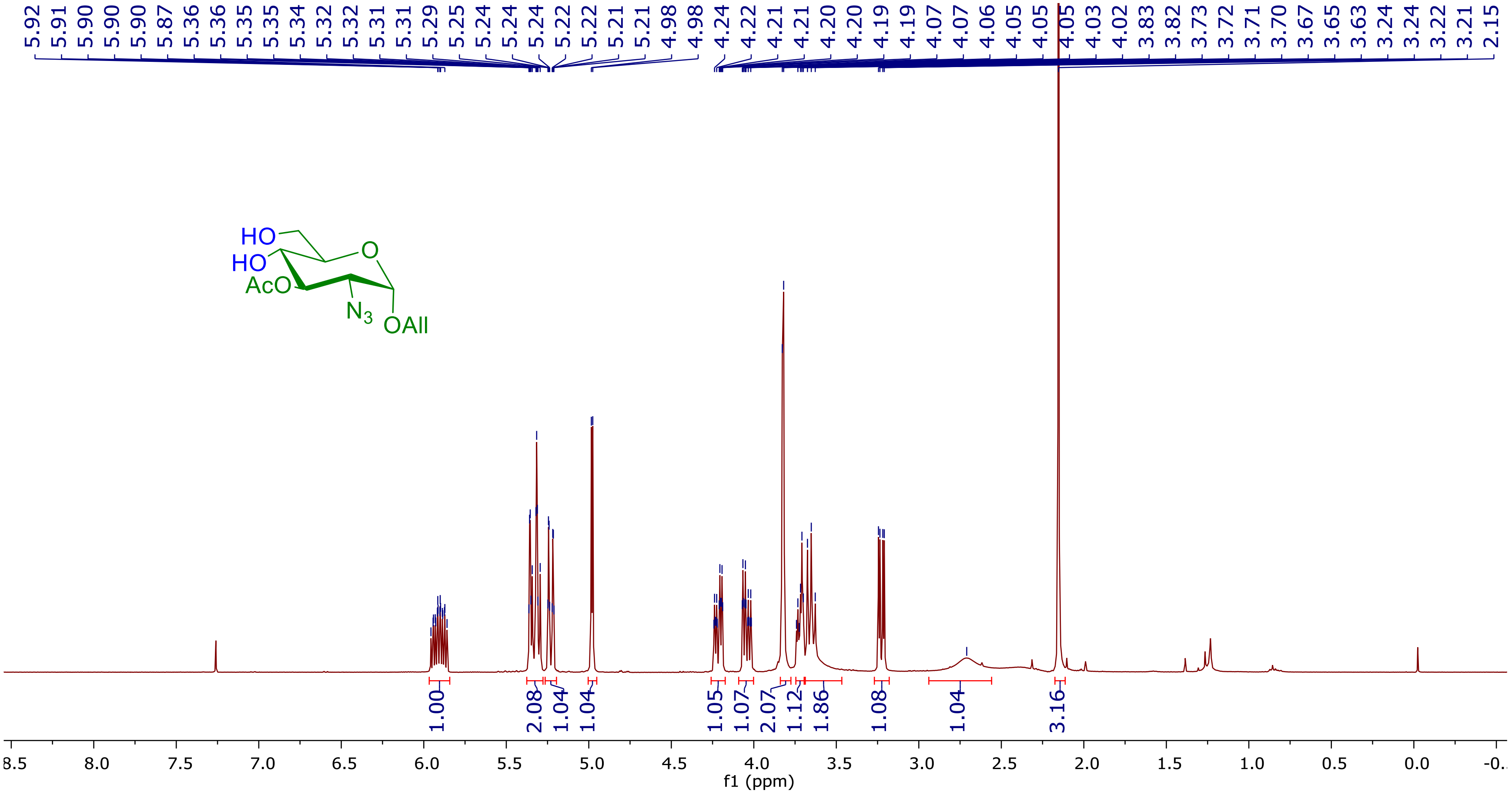

Supplementary Figure S36b. ^{13}C NMR Spectrum (100.67 MHz, $CDCl_3$) of Compound S7e

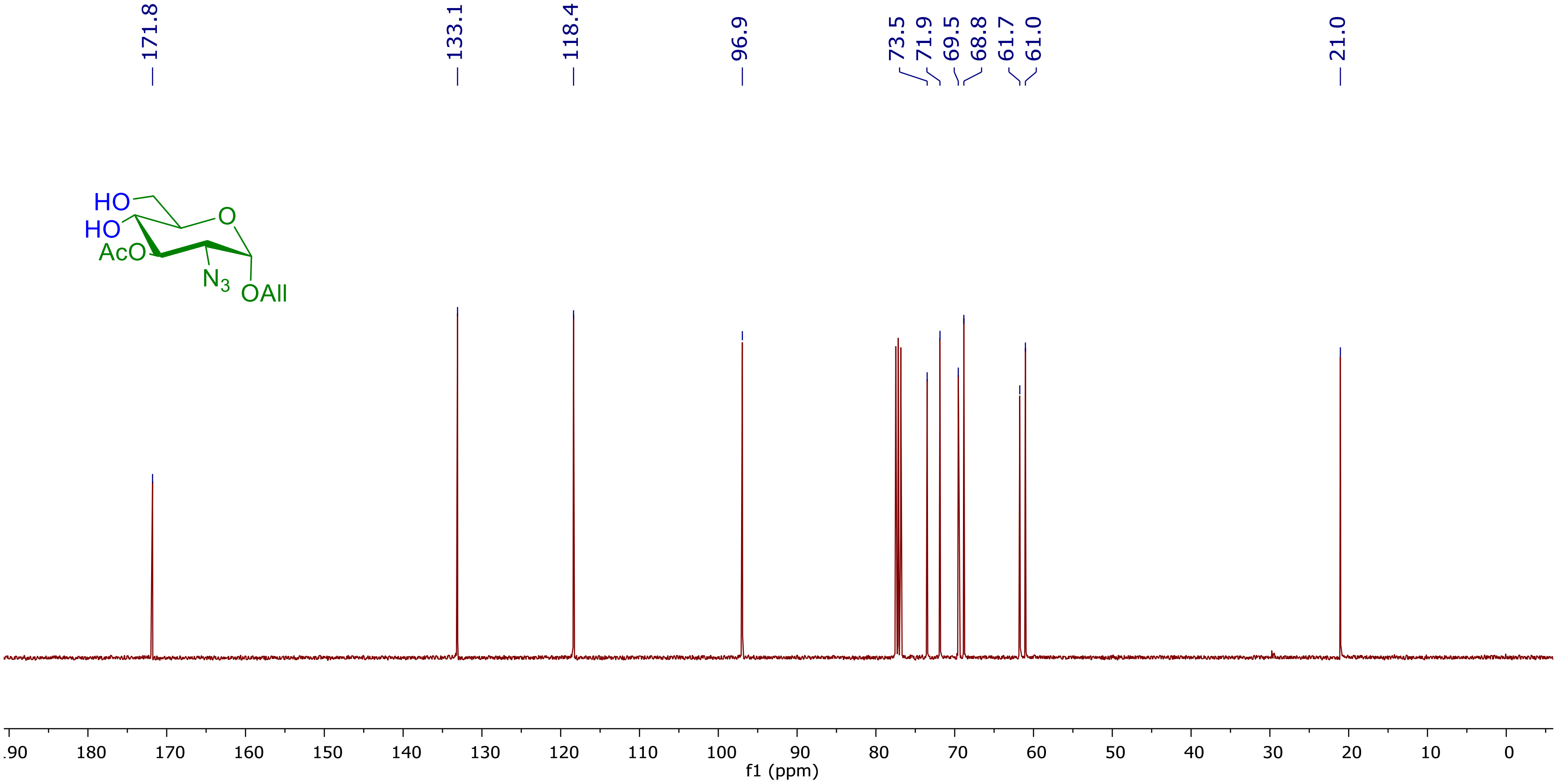

Supplementary Figure S36c. DEPT NMR Spectrum (100.67 MHz, $CDCl_3$) of Compound S7e

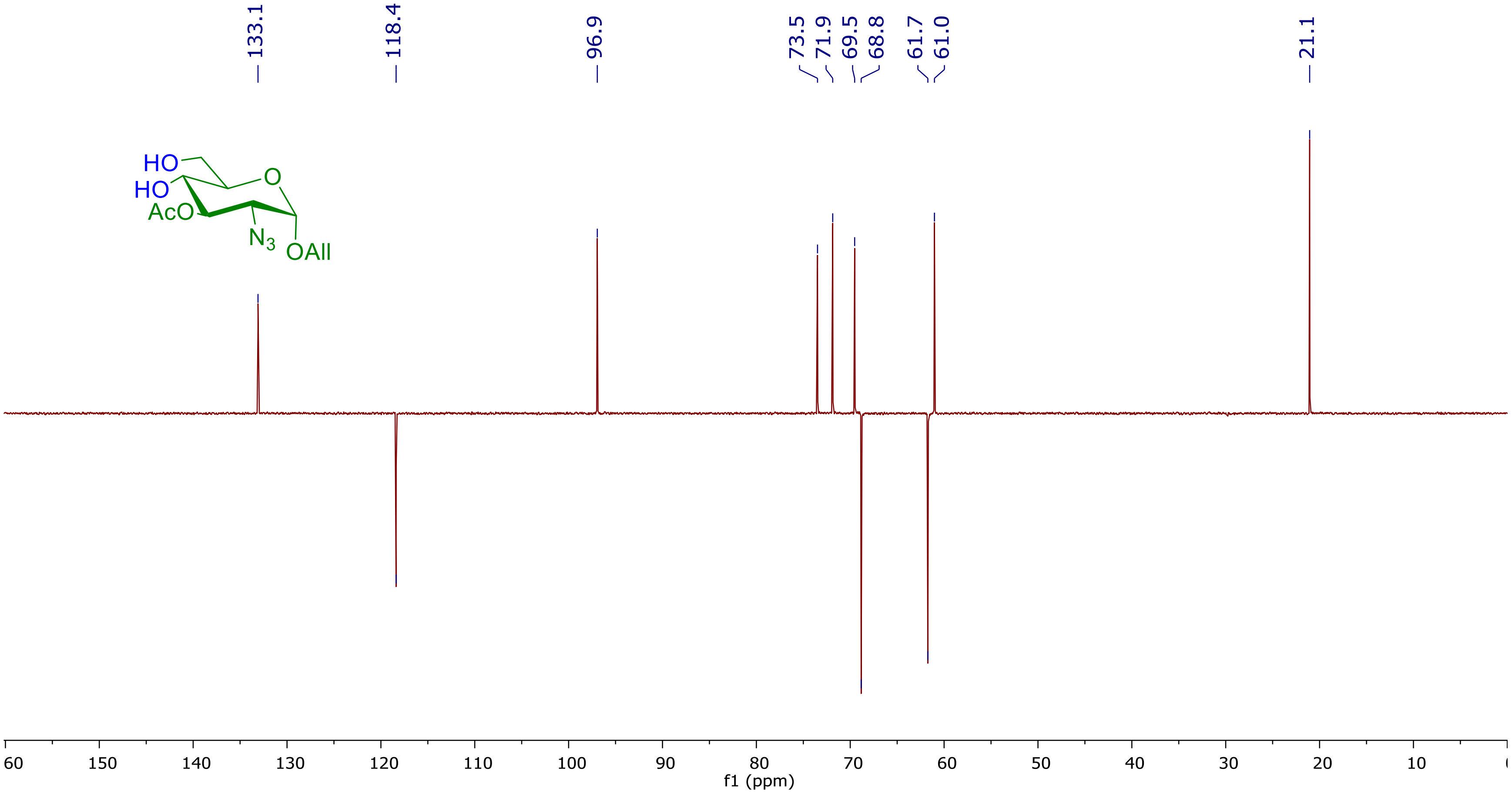

Supplementary Figure S37a. ^{1}H NMR Spectrum (400.31 MHz, $CDCl_3$) of Compound 31a

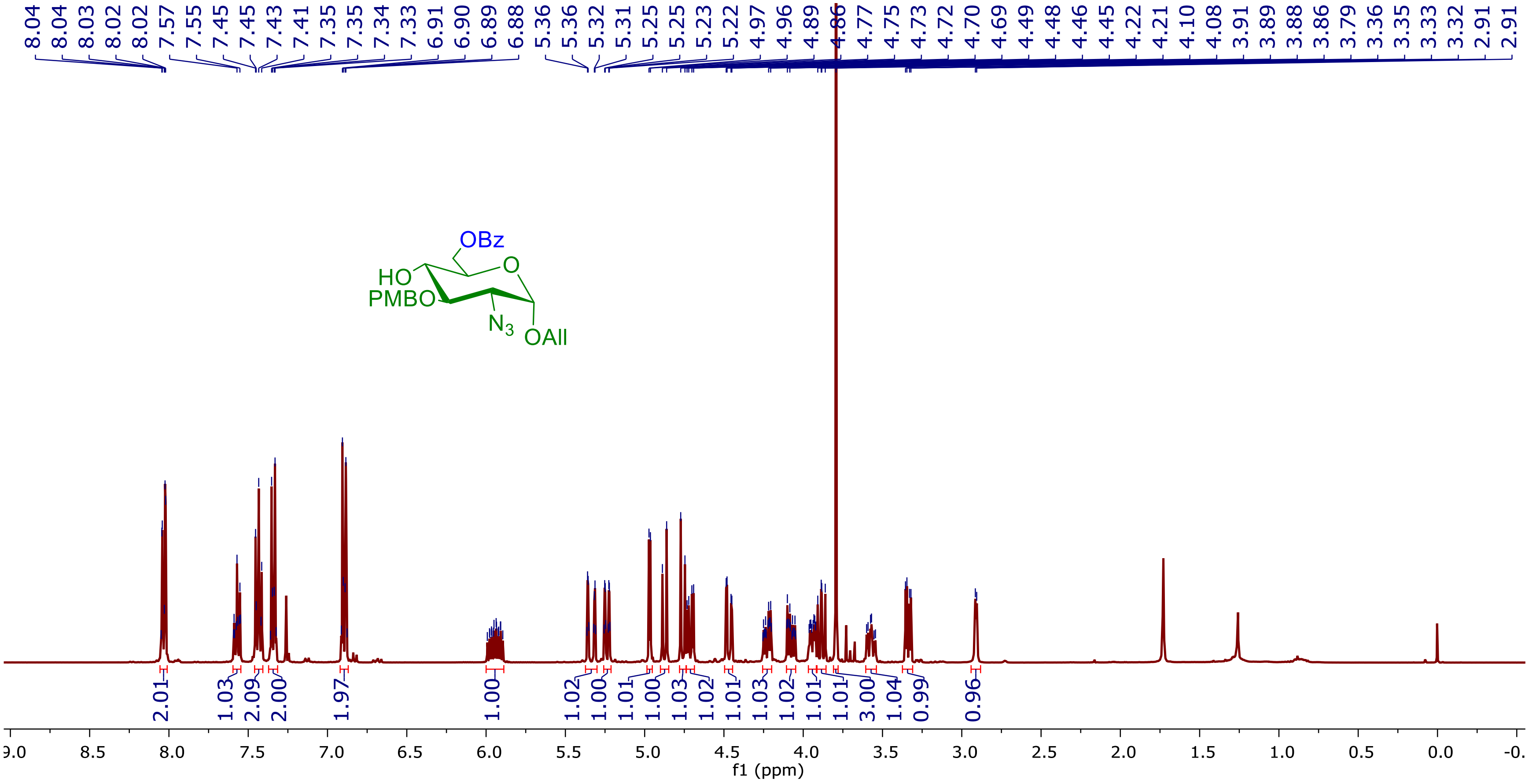

Supplementary Figure S37b. ^{13}C NMR Spectrum (100.67 MHz, $CDCl_3$) of Compound 31a

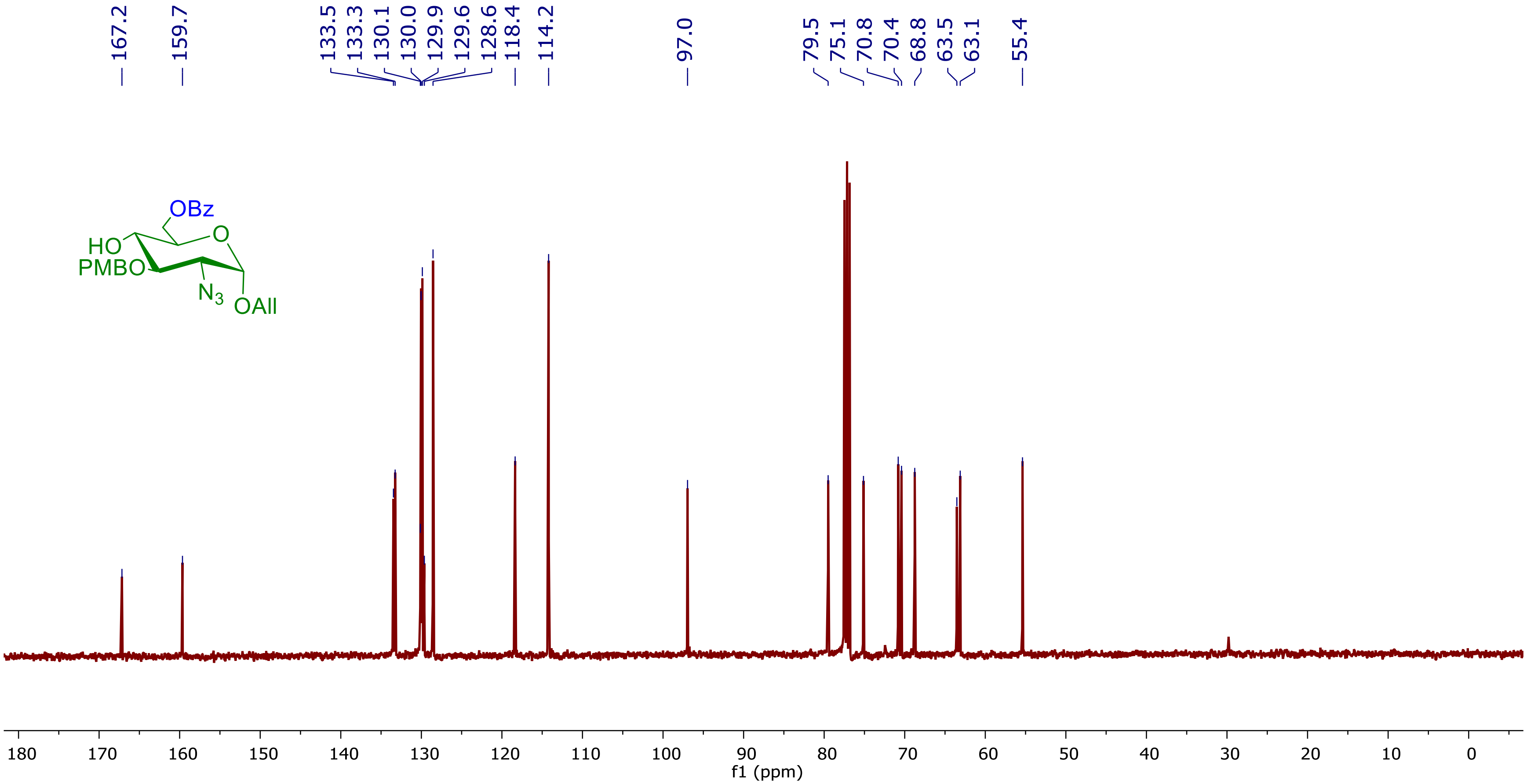

Supplementary Figure S37c. DEPT NMR Spectrum (100.67 MHz, $CDCl_3$) of Compound 31a

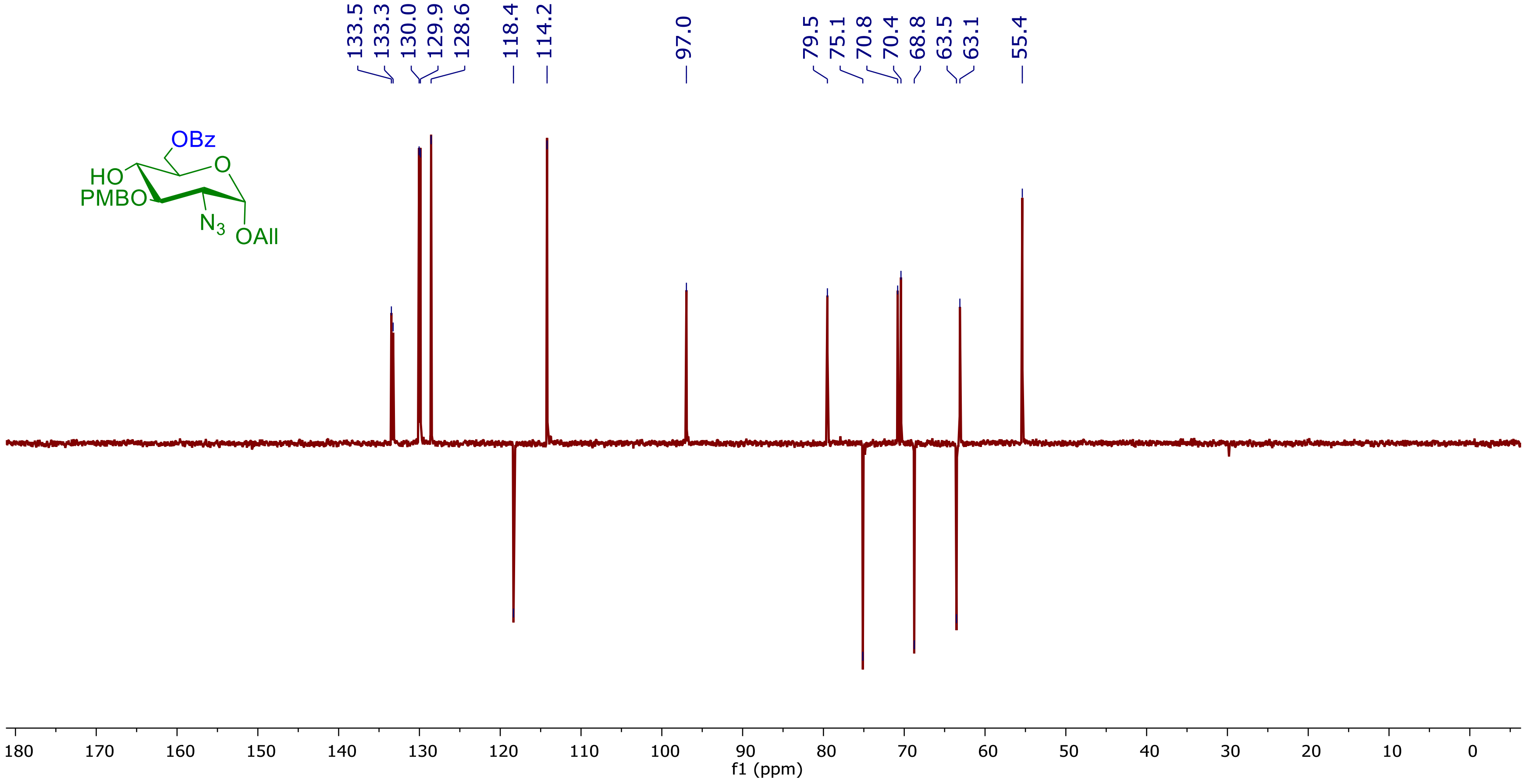

Supplementary Figure S38a. ^{1}H NMR Spectrum (400.31 MHz, $CDCl_3$) of Compound 6

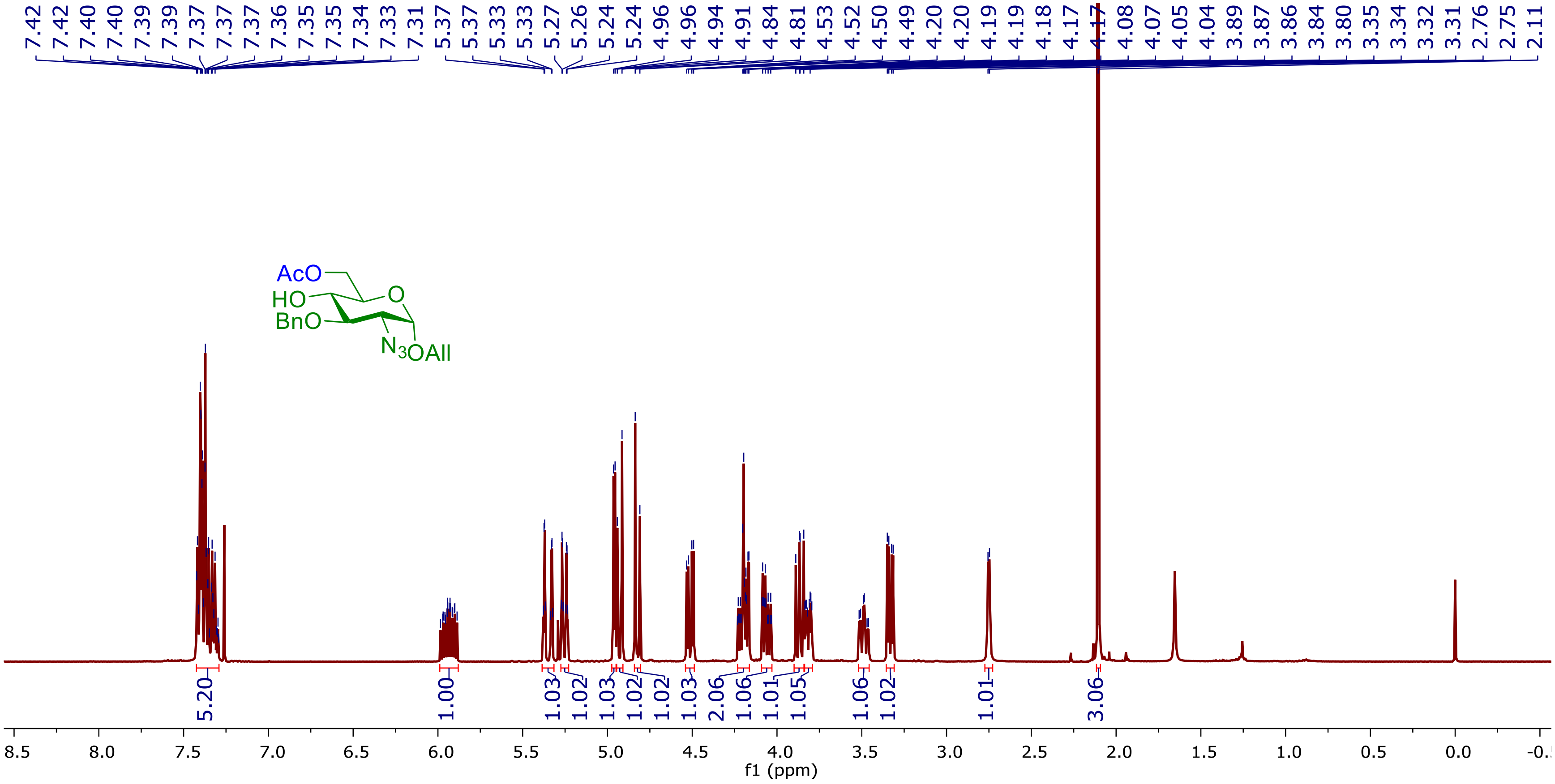

Supplementary Figure S38b ^{13}C NMR Spectrum (100.53 MHz, $CDCl_3$) of Compound 6

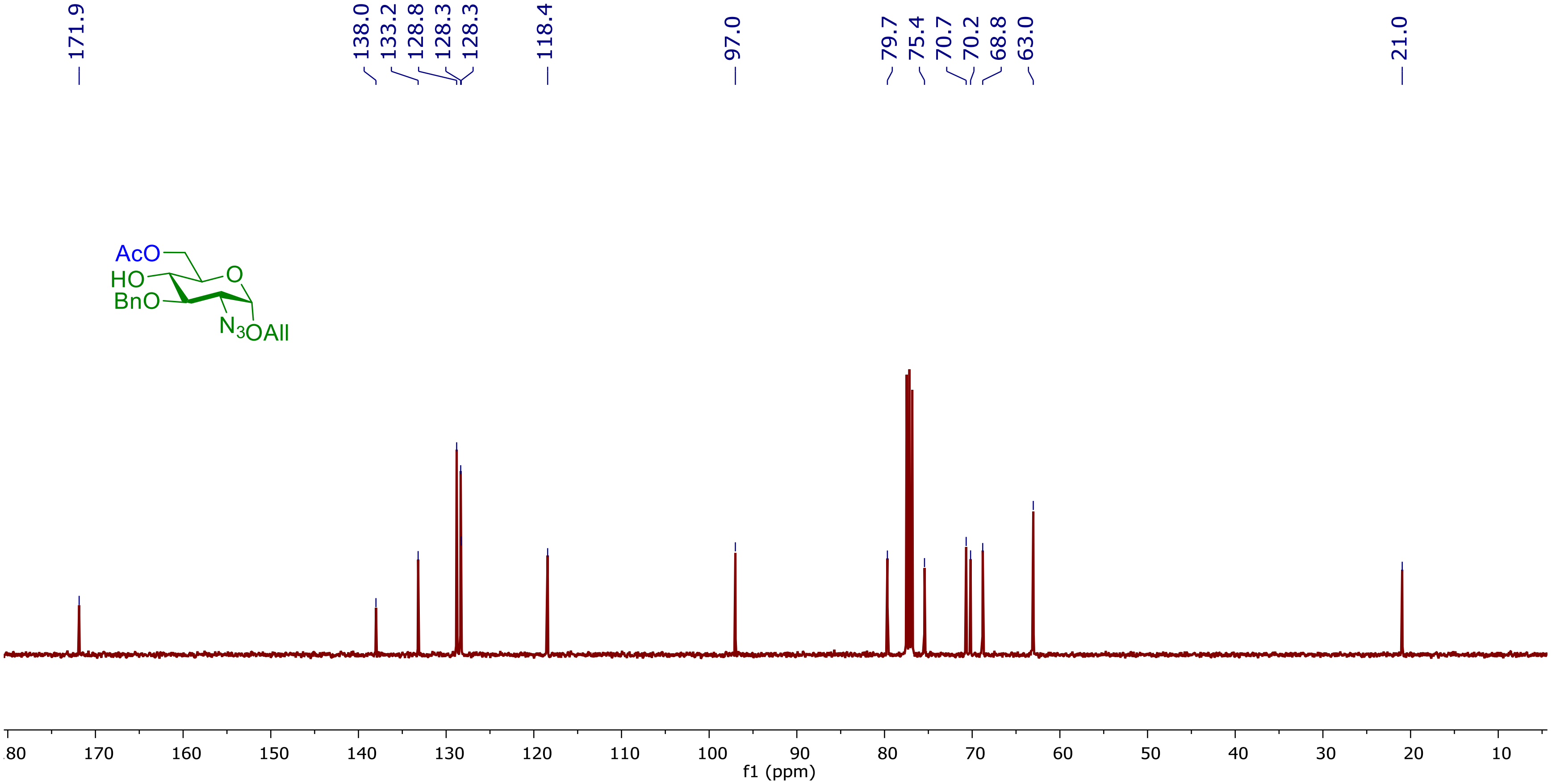

Supplementary Figure S38c. DEPT NMR Spectrum (100.53 MHz, $CDCl_3$) of Compound 6

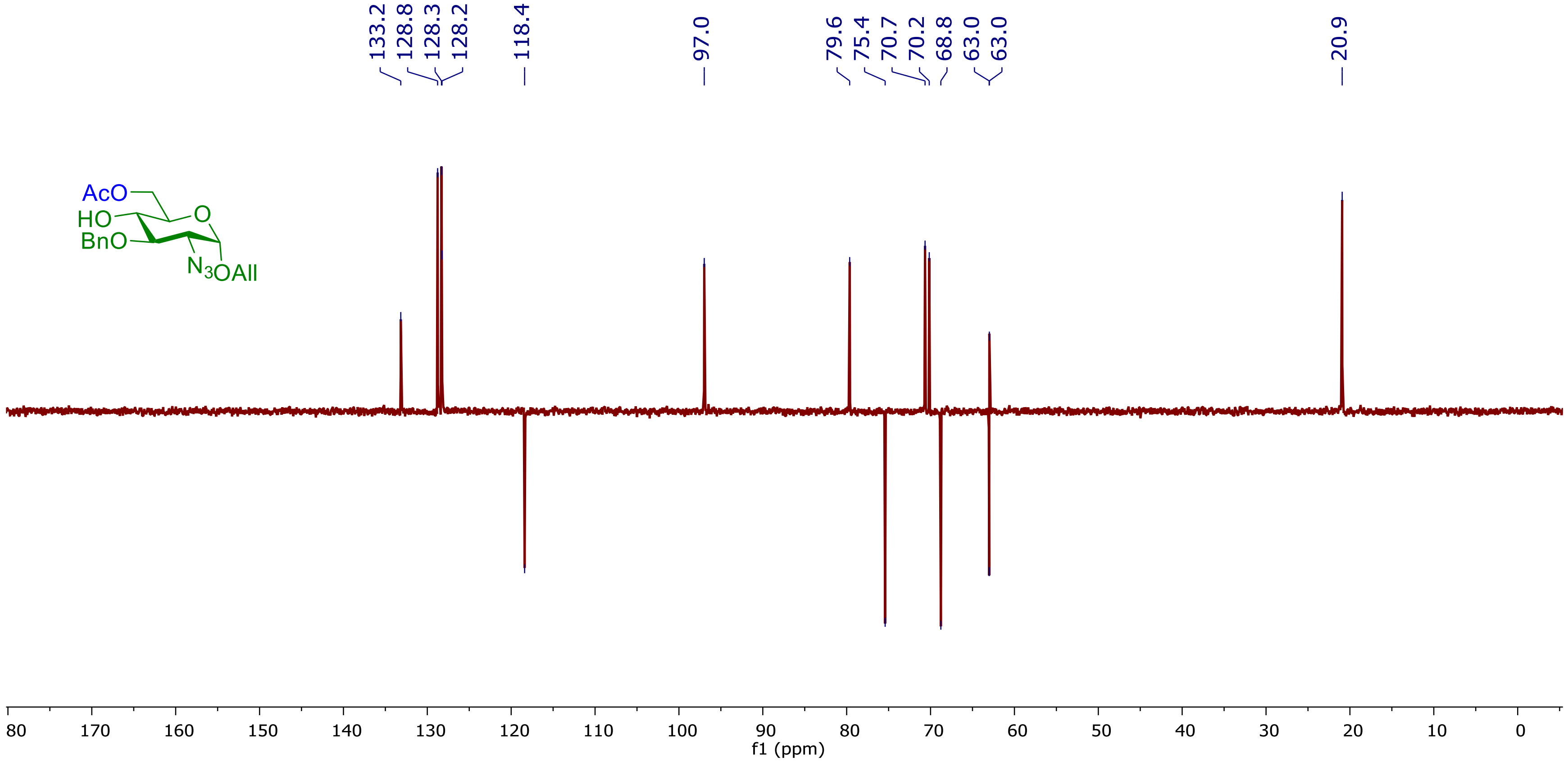

Supplementary Figure S40a. ^1H NMR Spectrum (400.31 MHz, $CDCl_3$) of Compound 31b

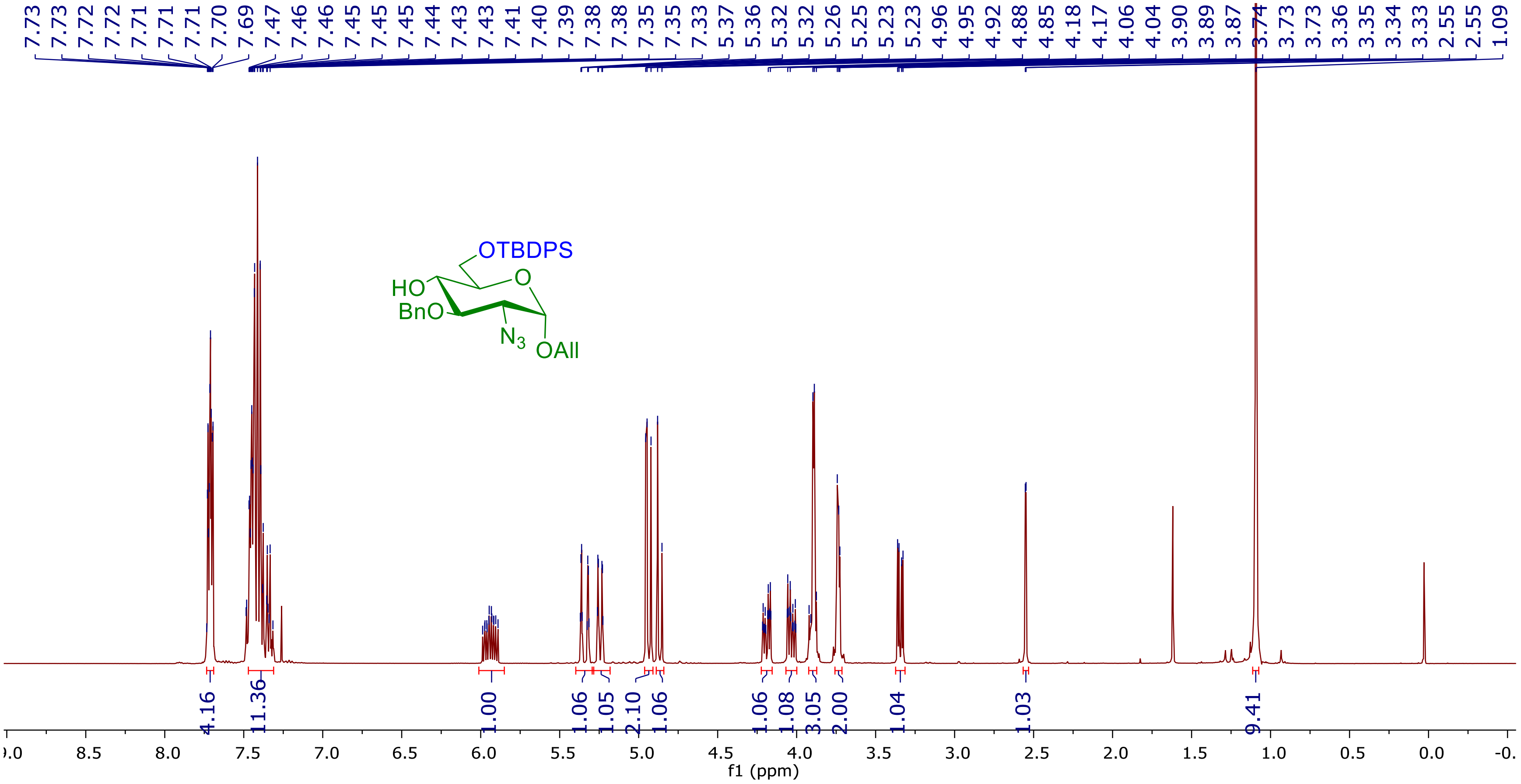

Supplementary Figure S40b. ^{13}C NMR Spectrum (100.67 MHz, $CDCl_3$) of Compound 31b

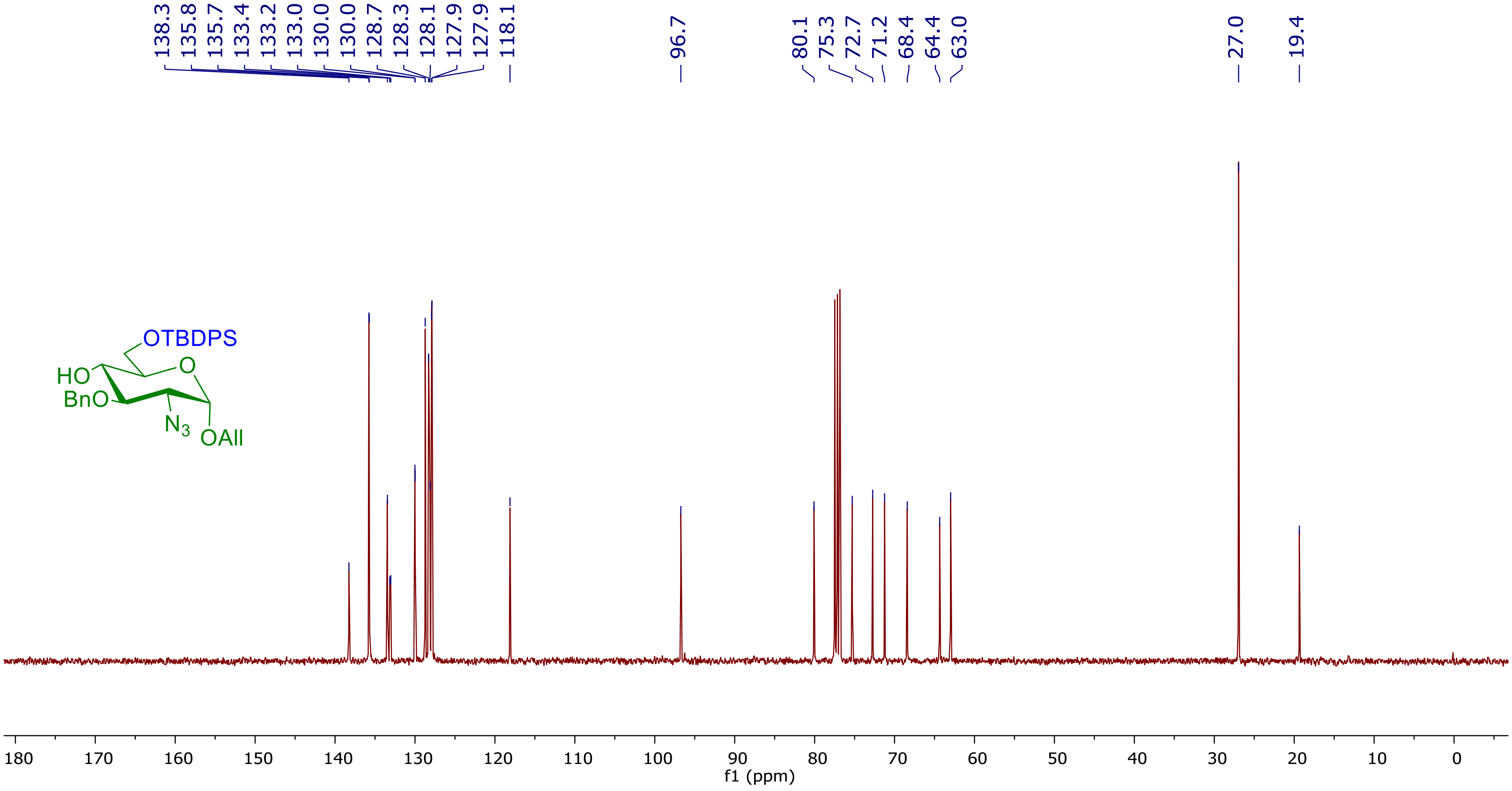

Supplementary Figure S40c. DEPT NMR Spectrum (100.67 MHz, $CDCl_3$) of Compound 31b

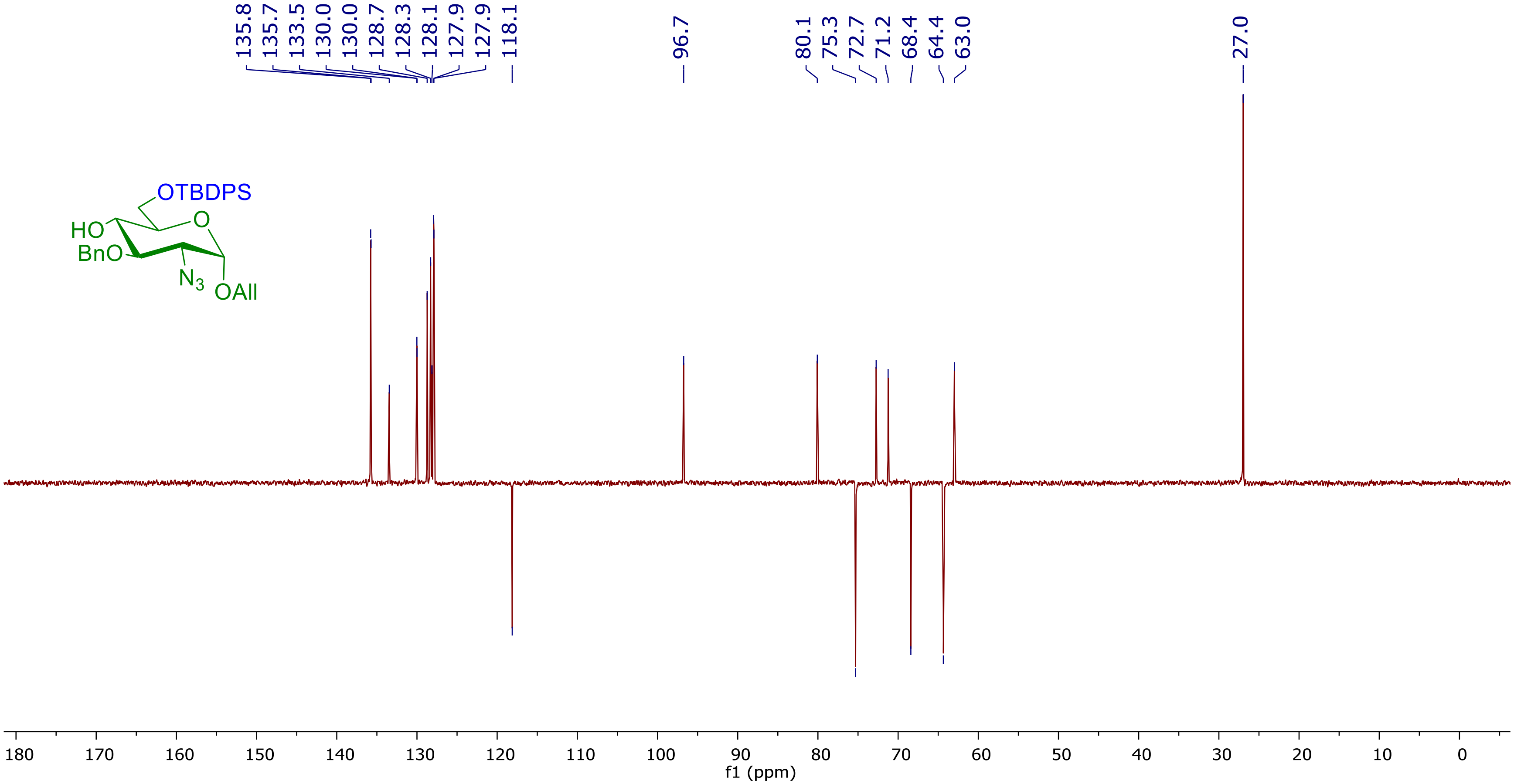

Supplementary Figure S41a. ^{1}H NMR Spectrum (400.31 MHz, $CDCl_3$) of Compound 31c

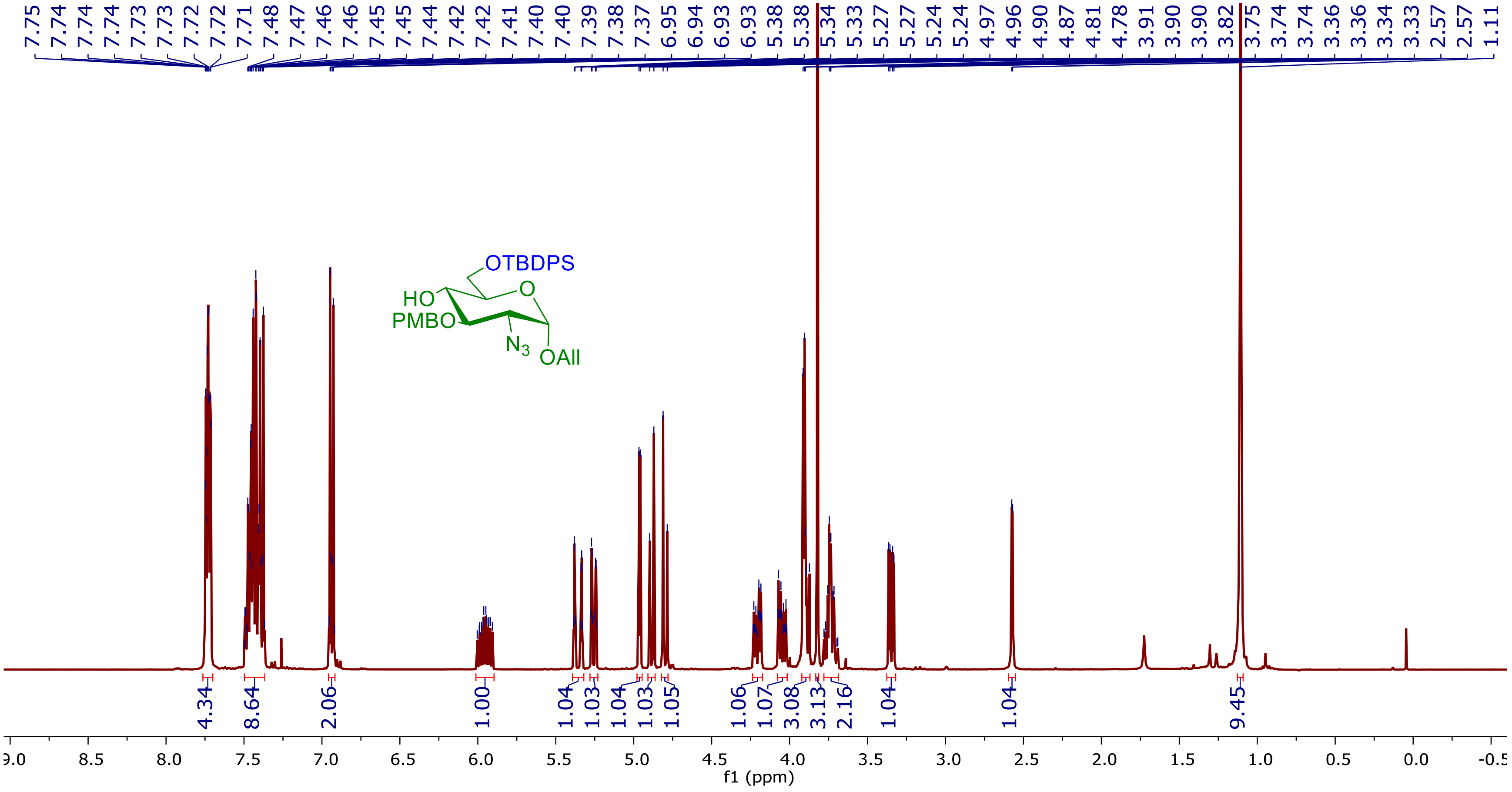

Supplementary Figure S41b. ^{13}C NMR Spectrum (100.67 MHz, $CDCl_3$) of Compound 31c

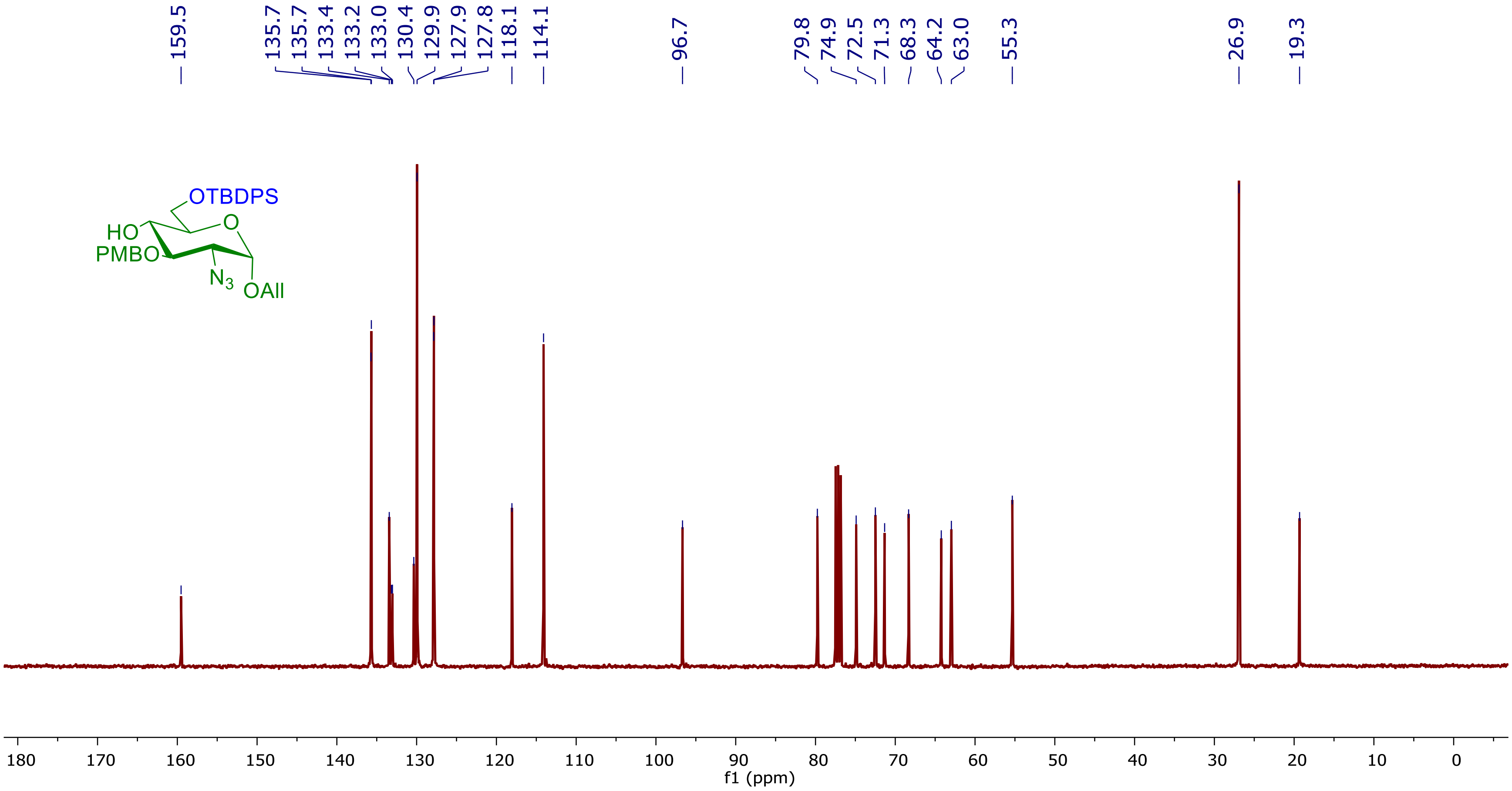

Supplementary Figure S41c. DEPT NMR Spectrum (100.67 MHz, CDCl$_3$) of Compound 31c

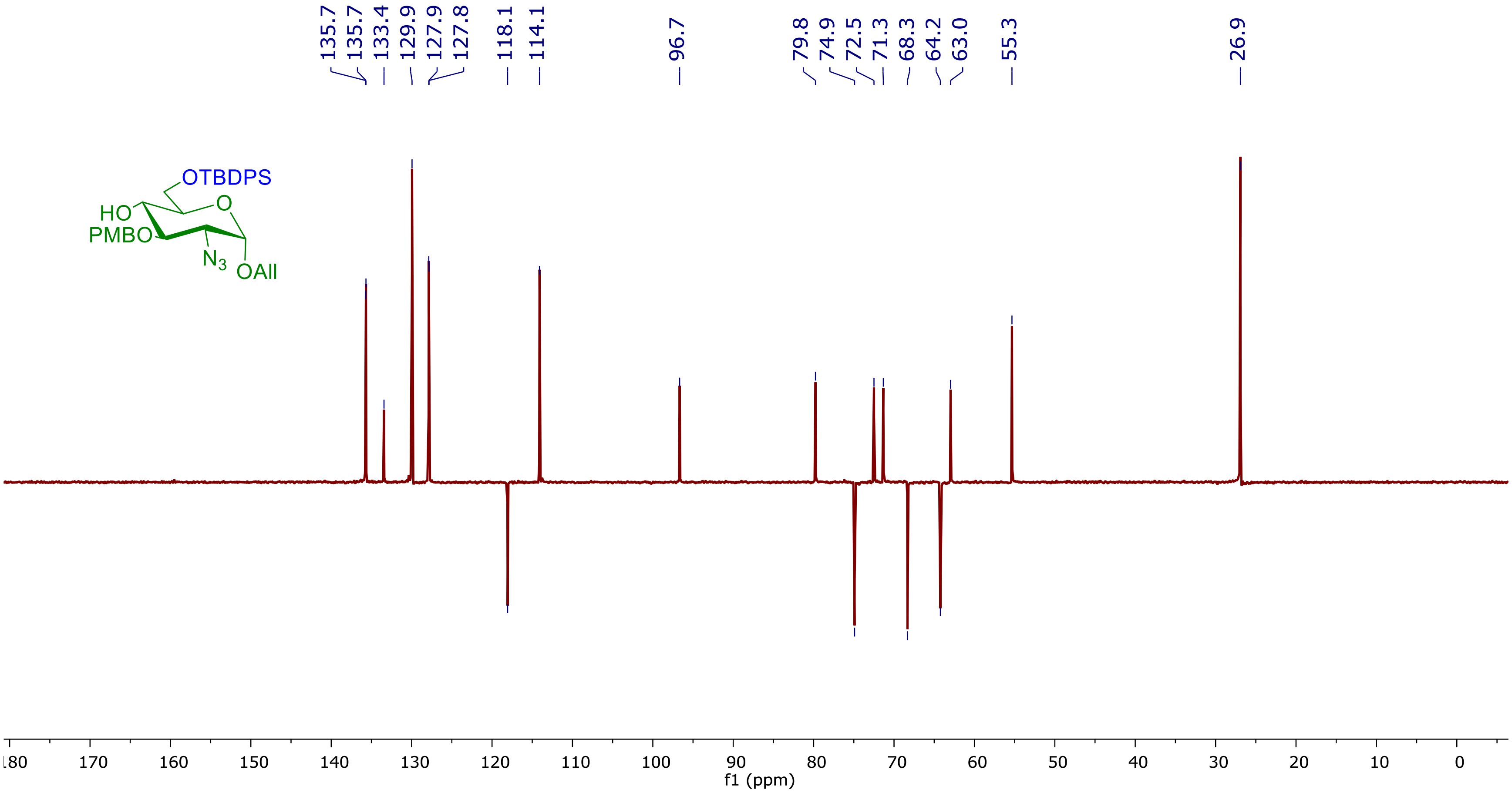

Supplementary Figure S42a. ¹H NMR Spectrum (400.31 MHz, CDCl₃) of Compound 31d

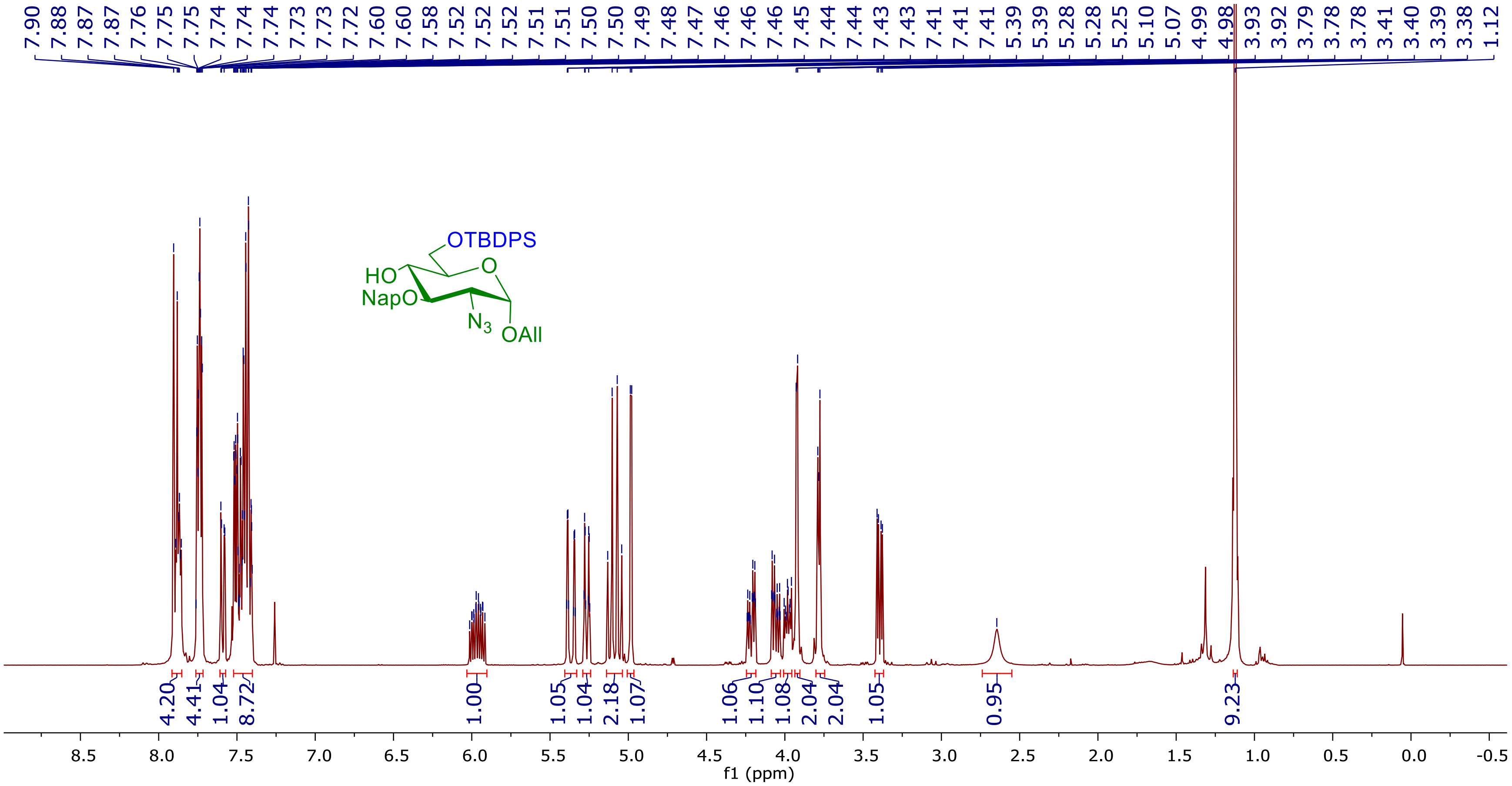

Supplementary Figure S42b. ^{13}C NMR Spectrum (100.67 MHz, $CDCl_3$) of Compound 31d

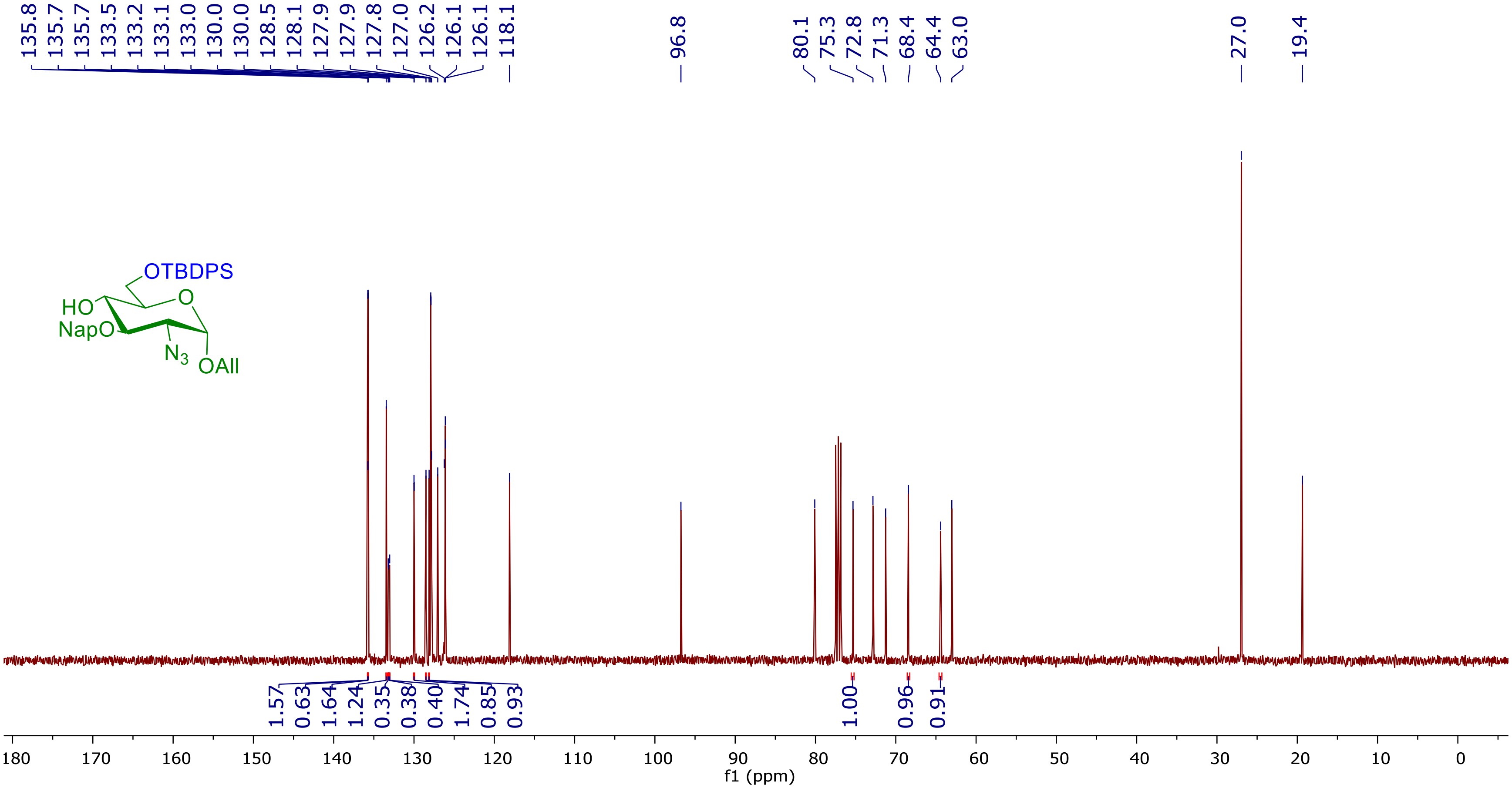

Supplementary Figure S42c. DEPT NMR Spectrum (100.67 MHz, CDCl₃) of Compound 31d

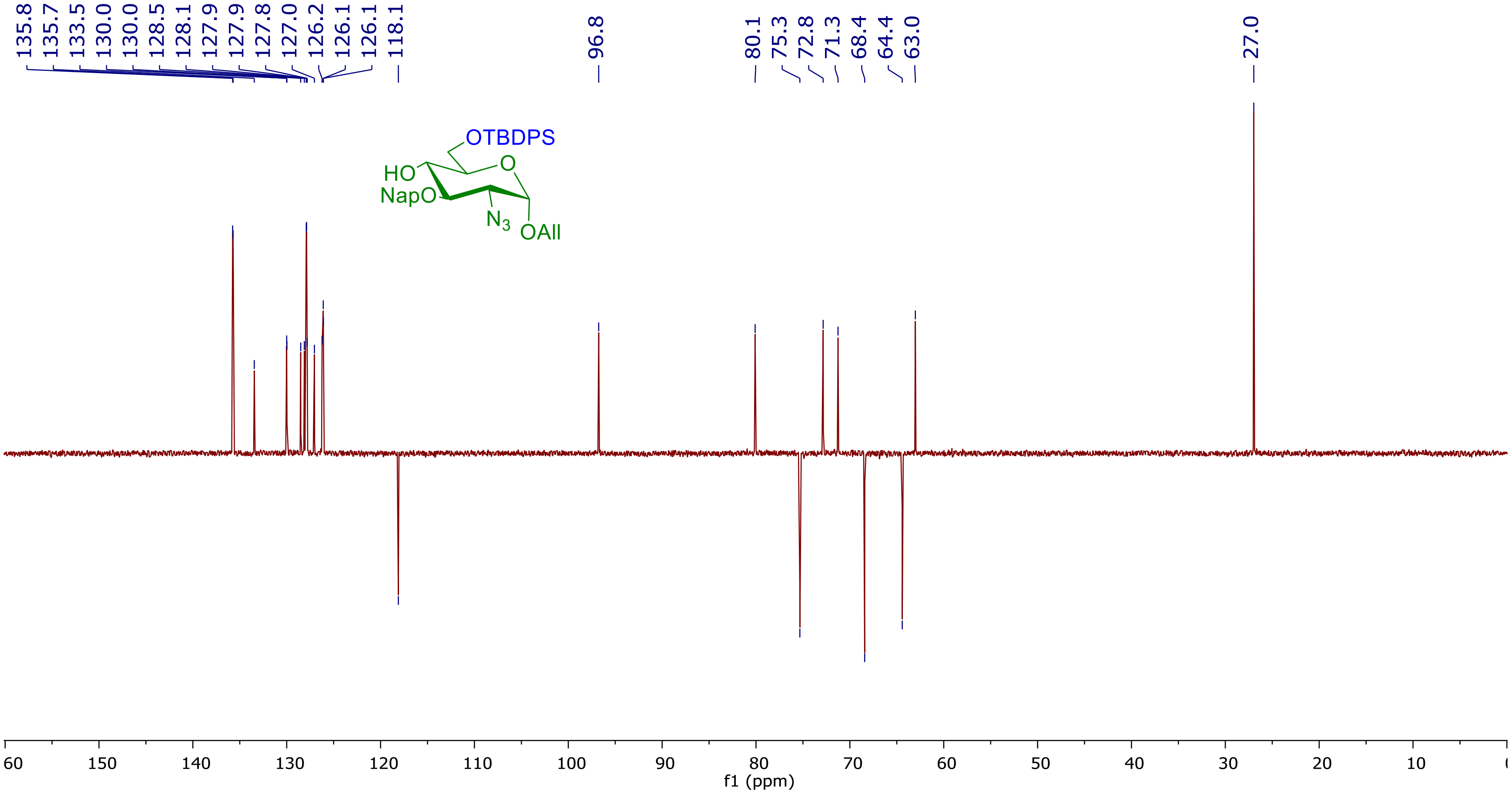

Supplementary Figure S43a. ^{1}H NMR Spectrum (400.31 MHz, $CDCl_3$) of Compound 31e

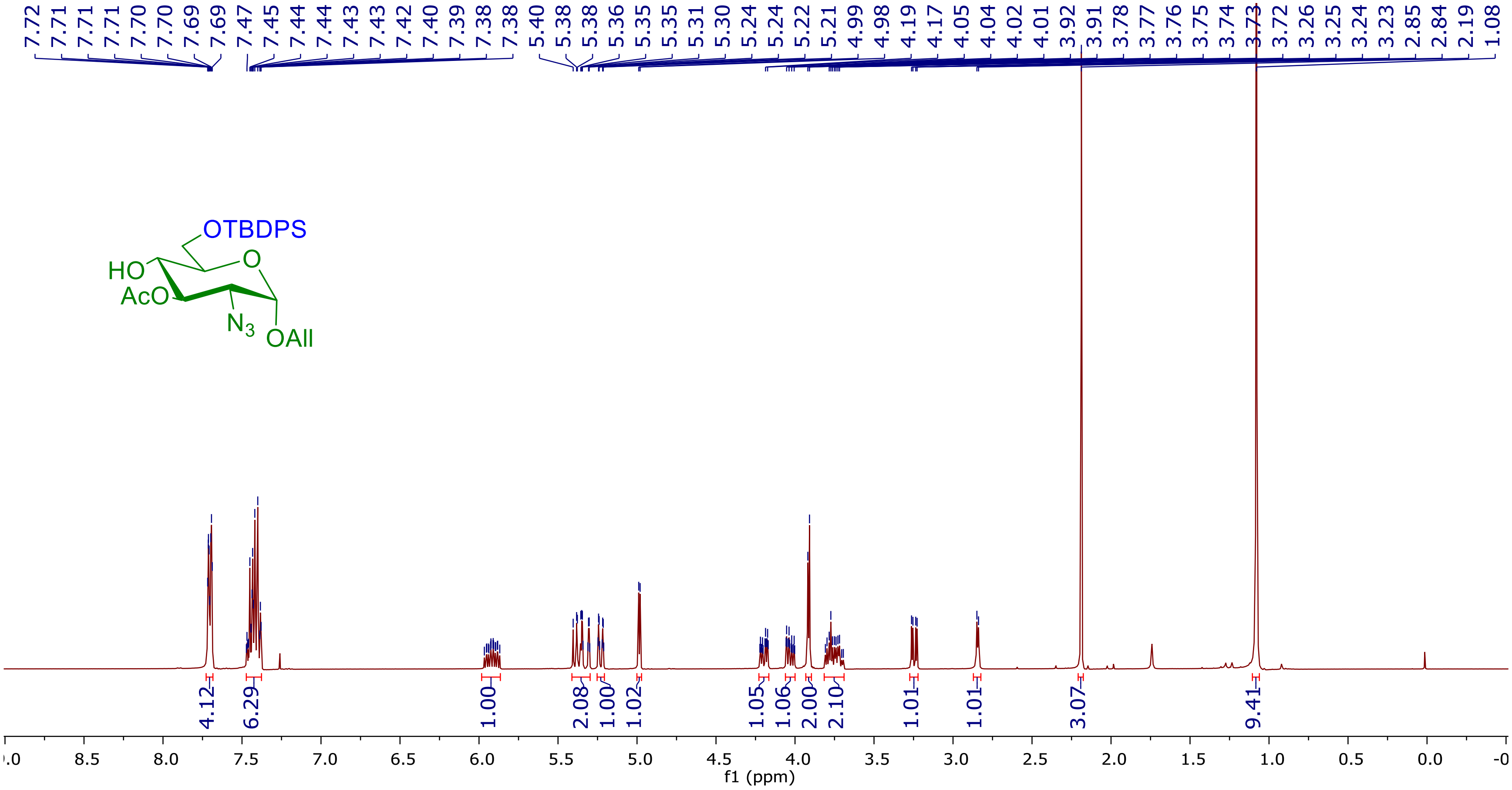

Supplementary Figure S43b. ^{13}C NMR Spectrum (100.67 MHz, $CDCl_3$) of Compound 31e

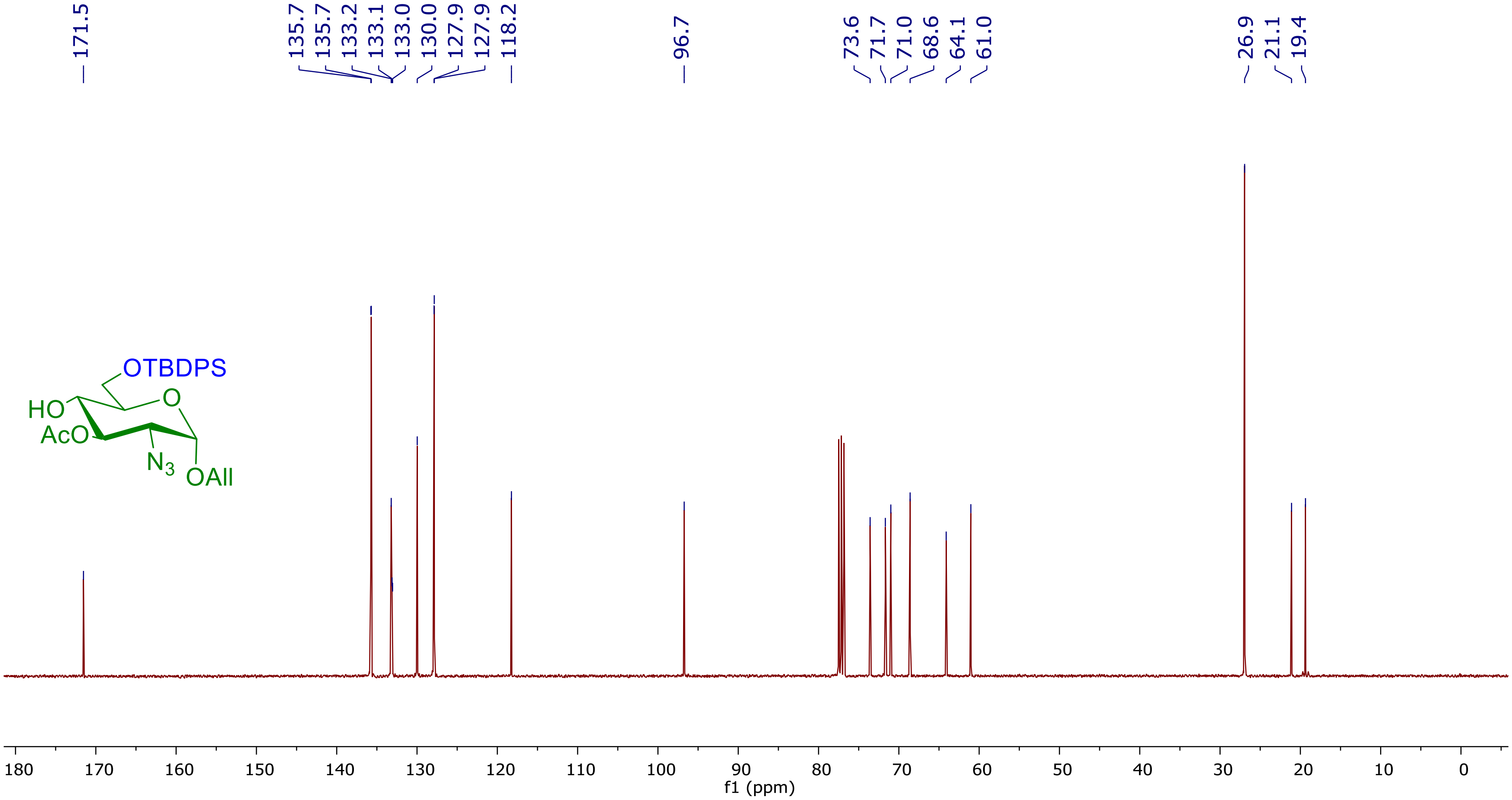

Supplementary Figure S43c. DEPT NMR Spectrum (100.67 MHz, $CDCl_3$) of Compound 31e

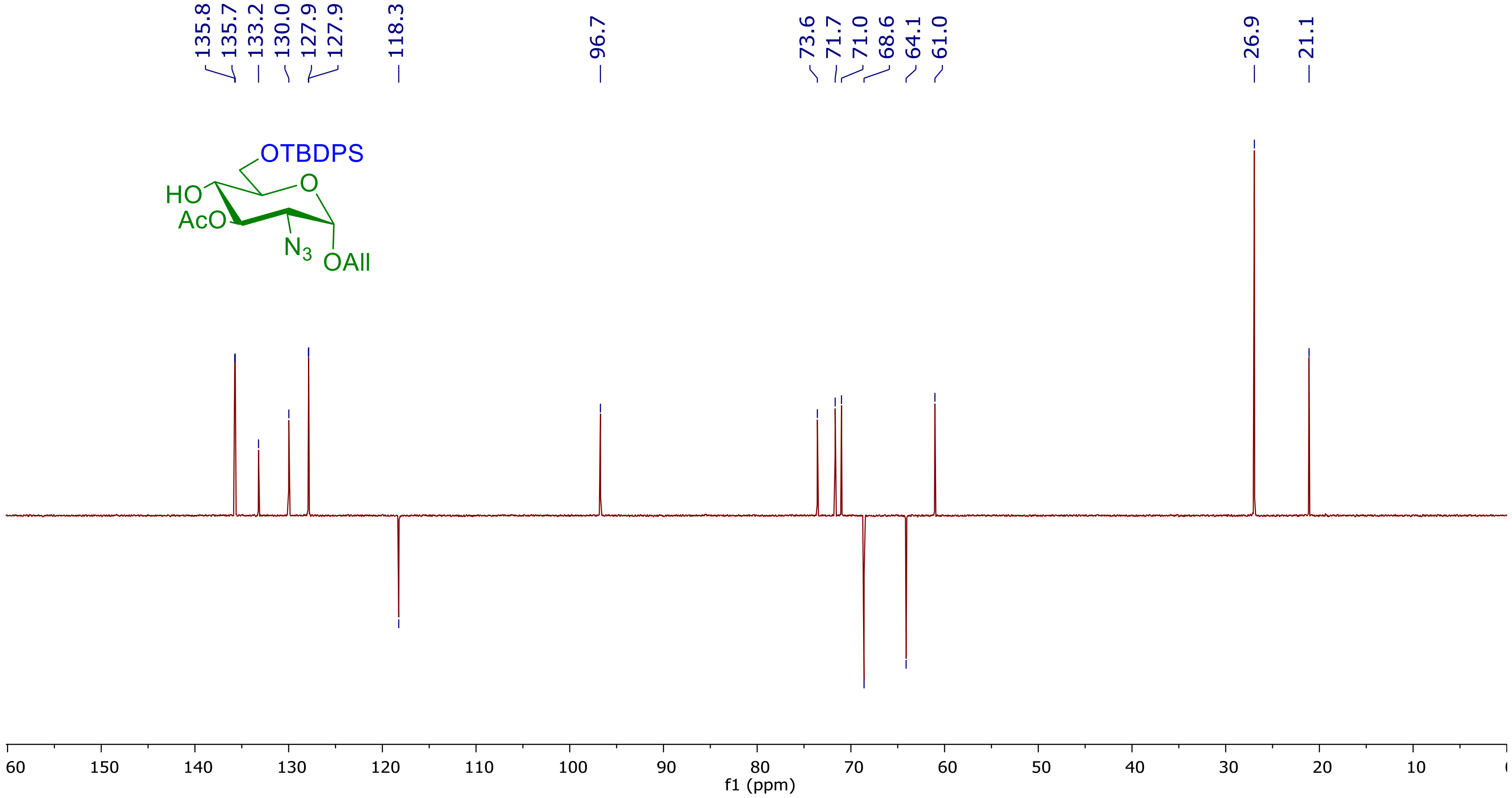

Supplementary Figure S44a. ^{1}H NMR Spectrum (400.31 MHz, $CDCl_3$) of Compound 31f

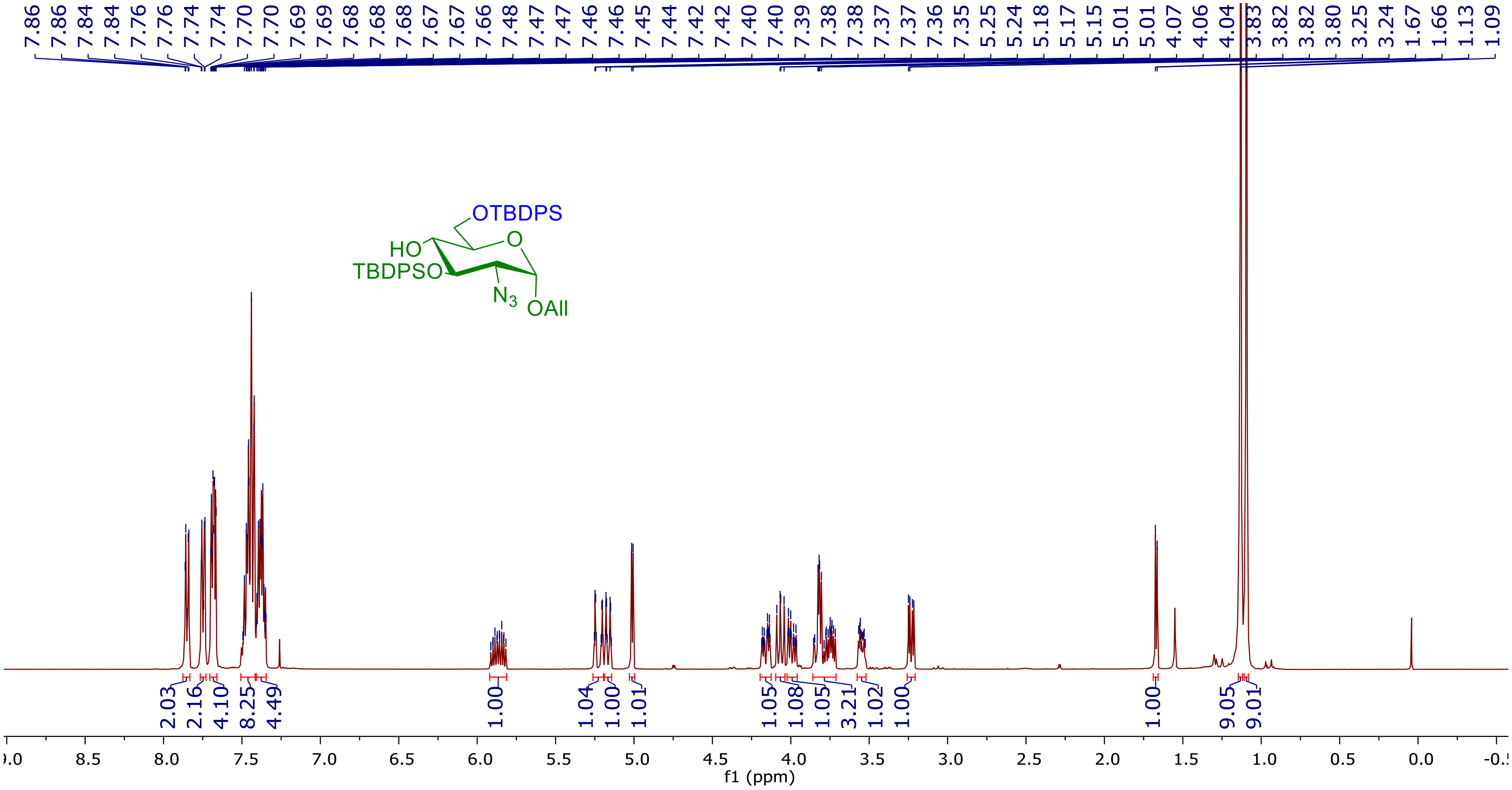

Supplementary Figure S44b. ^{13}C NMR Spectrum (100.67 MHz, $CDCl_3$) of Compound 31f

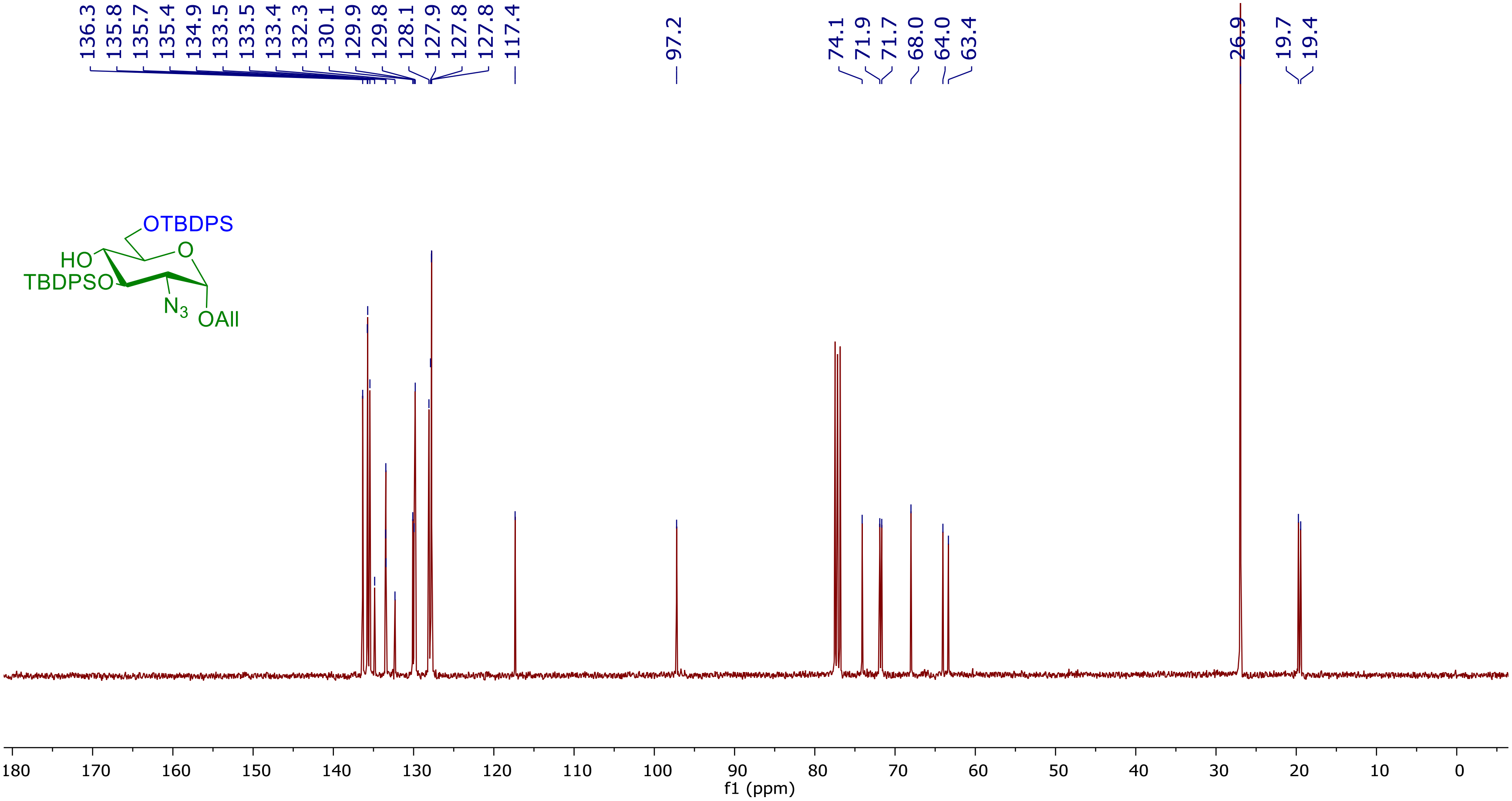

Supplementary Figure S44c. DEPT NMR Spectrum (100.67 MHz, $CDCl_3$) of Compound 31f

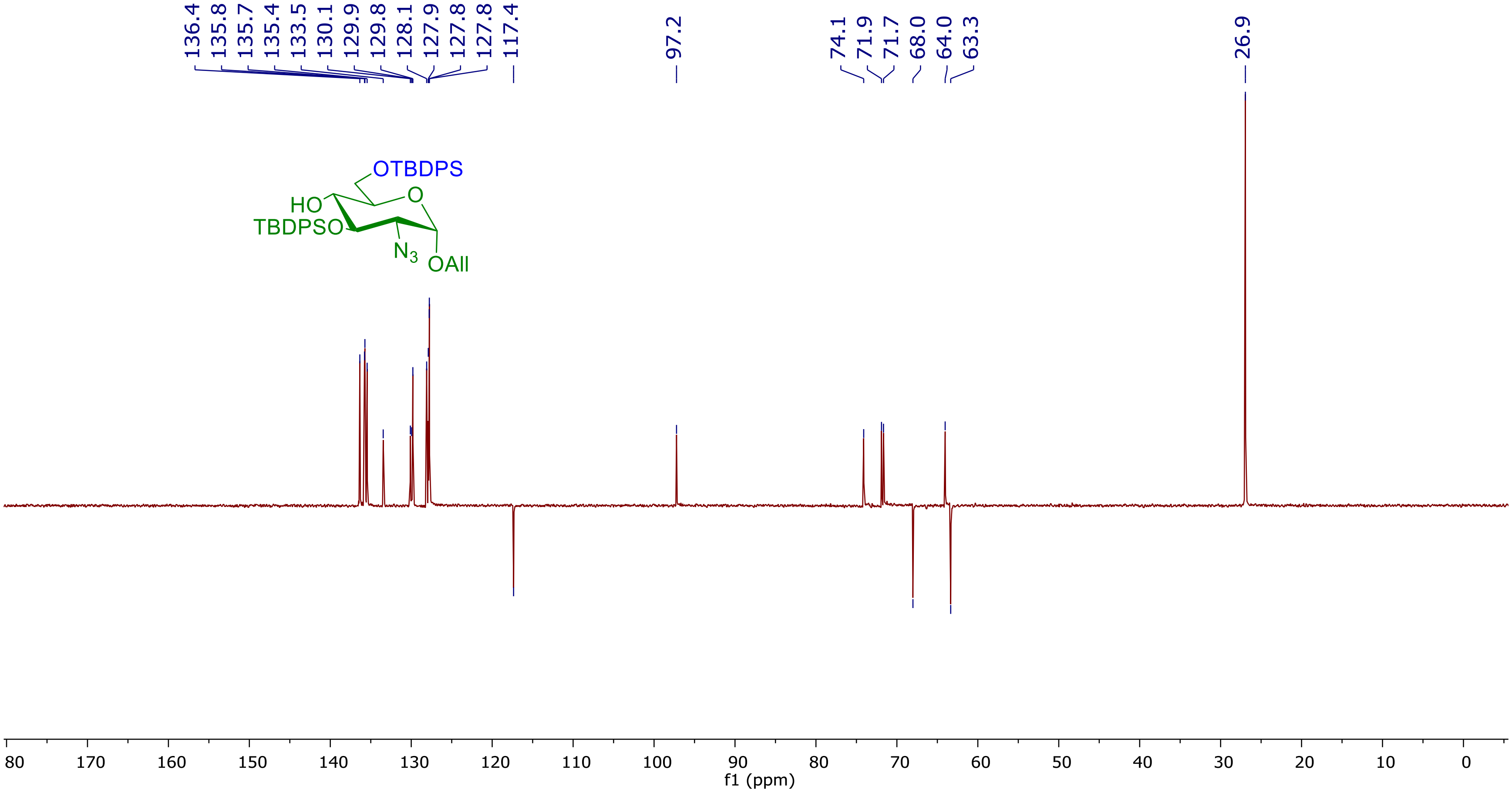

Supplementary Figure S45a. ^{1}H NMR Spectrum (400.31 MHz, $CDCl_3$) of Compound 32a

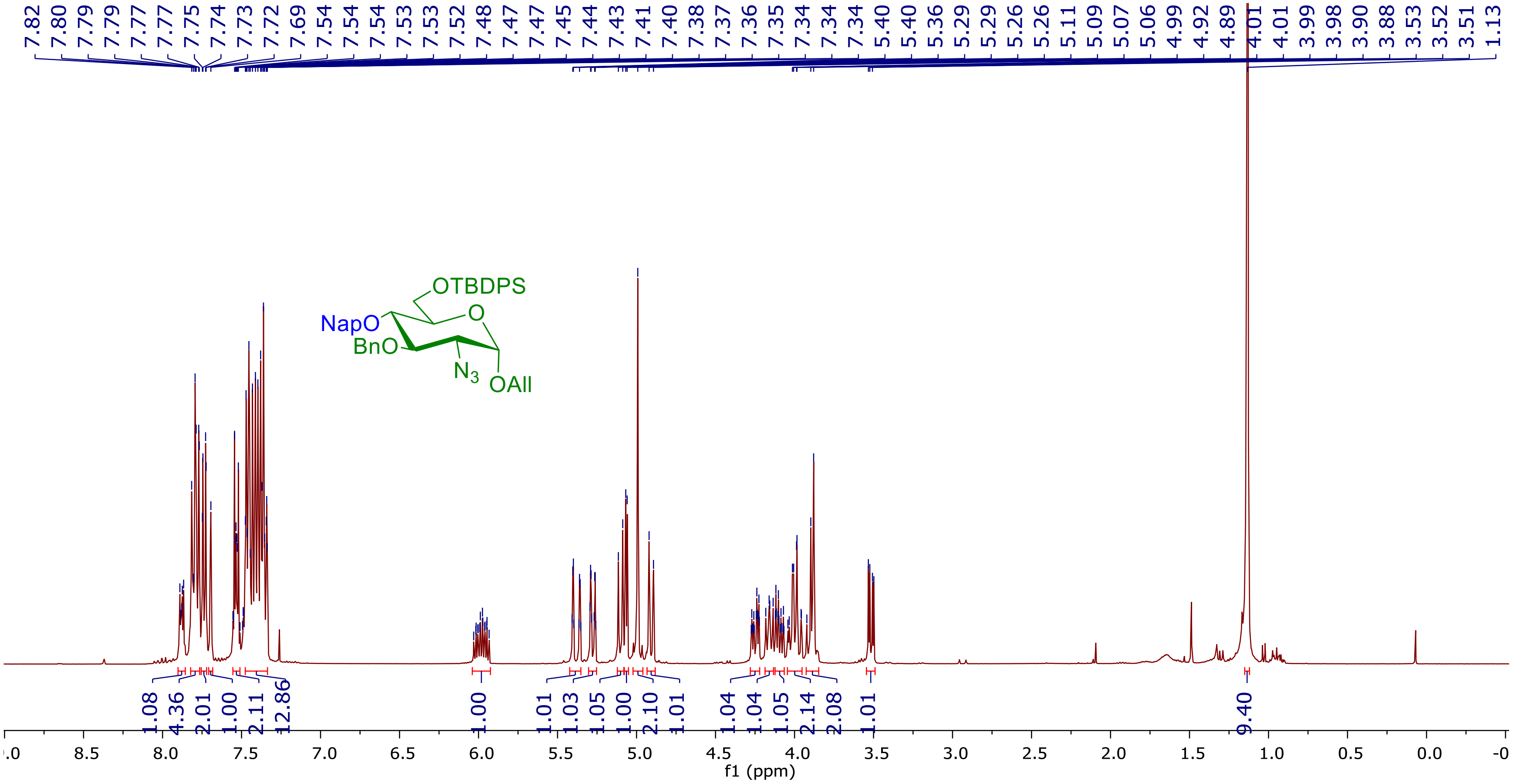

Supplementary Figure S45b. ^{13}C NMR Spectrum (100.67 MHz, $CDCl_3$) of Compound 32a

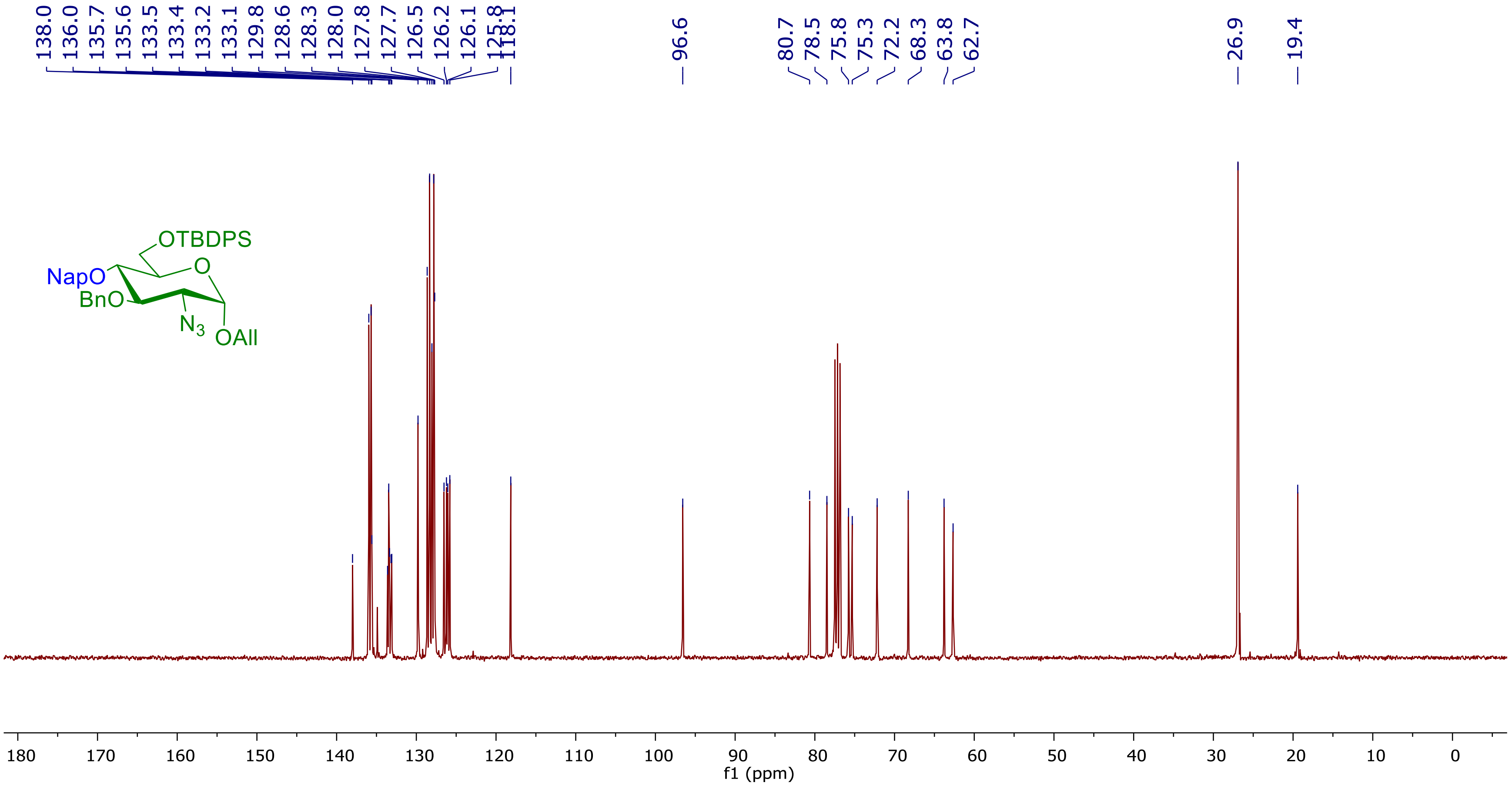

Supplementary Figure S45c. DEPT NMR Spectrum (100.67 MHz, $CDCl_3$) of Compound 32a

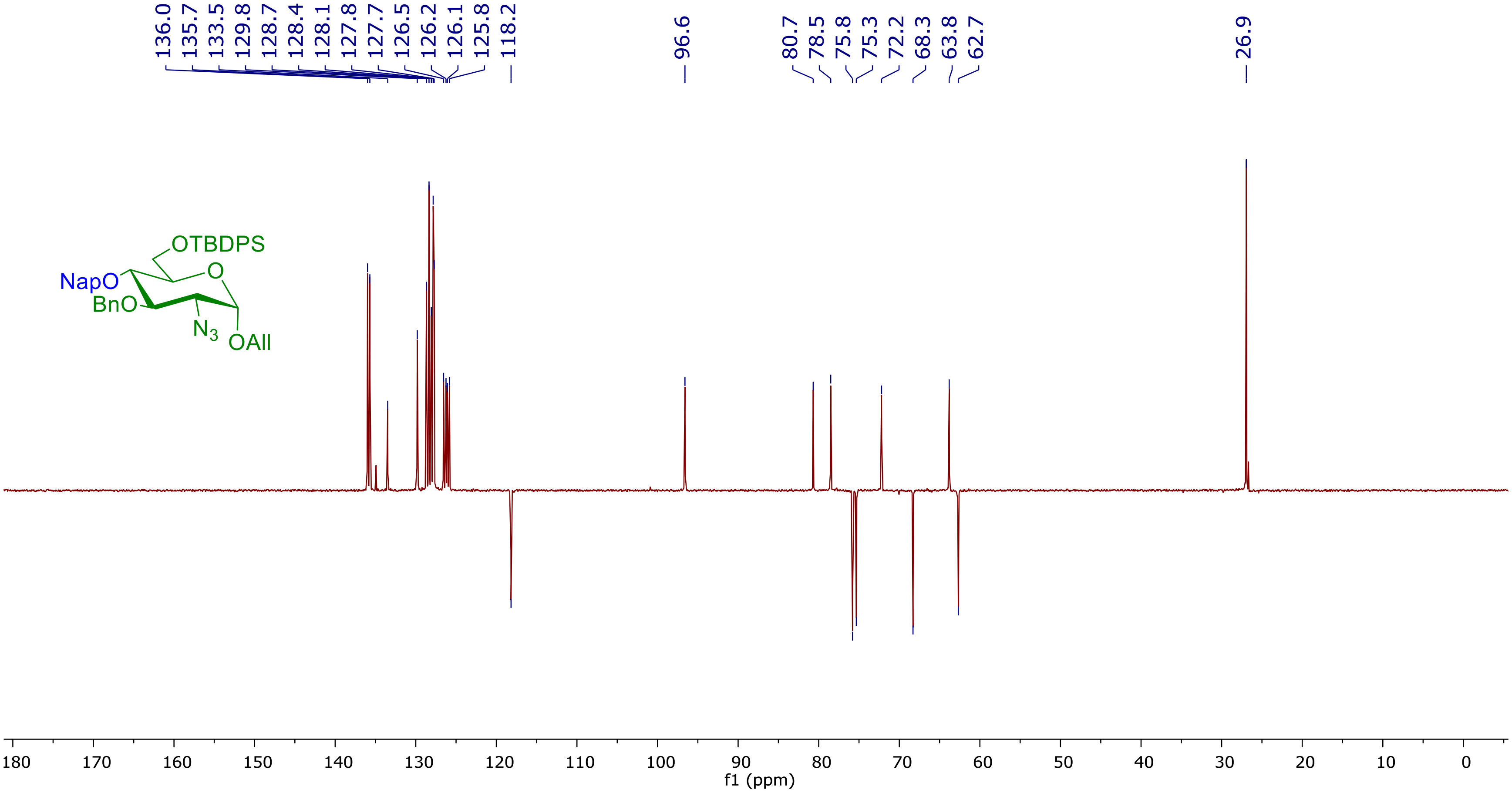

Supplementary Figure S46a. ^{1}H NMR Spectrum (400.31 MHz, $CDCl_3$) of Compound 32b

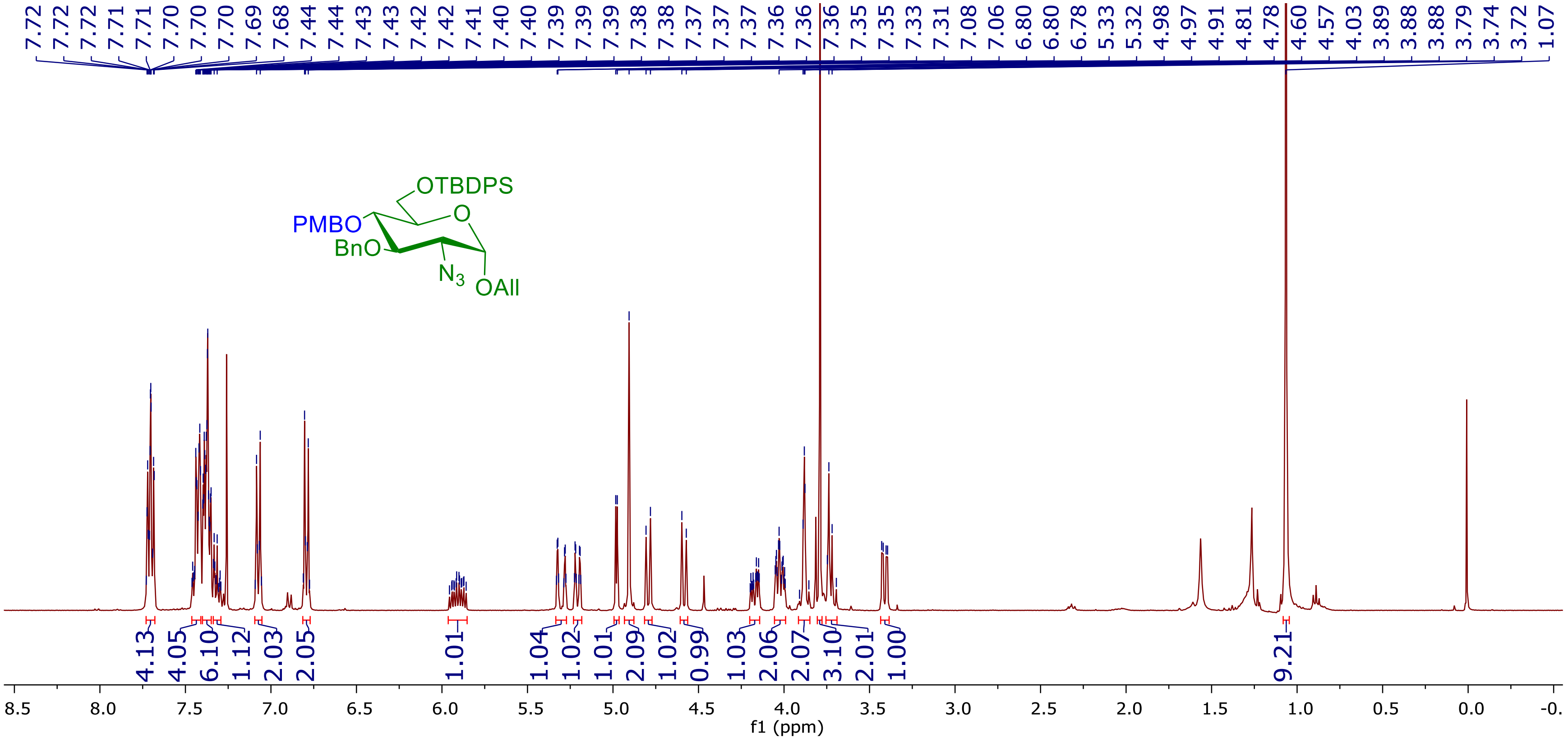

Supplementary Figure S46b. ^{13}C NMR Spectrum (100.67 MHz, $CDCl_3$) of Compound 32b

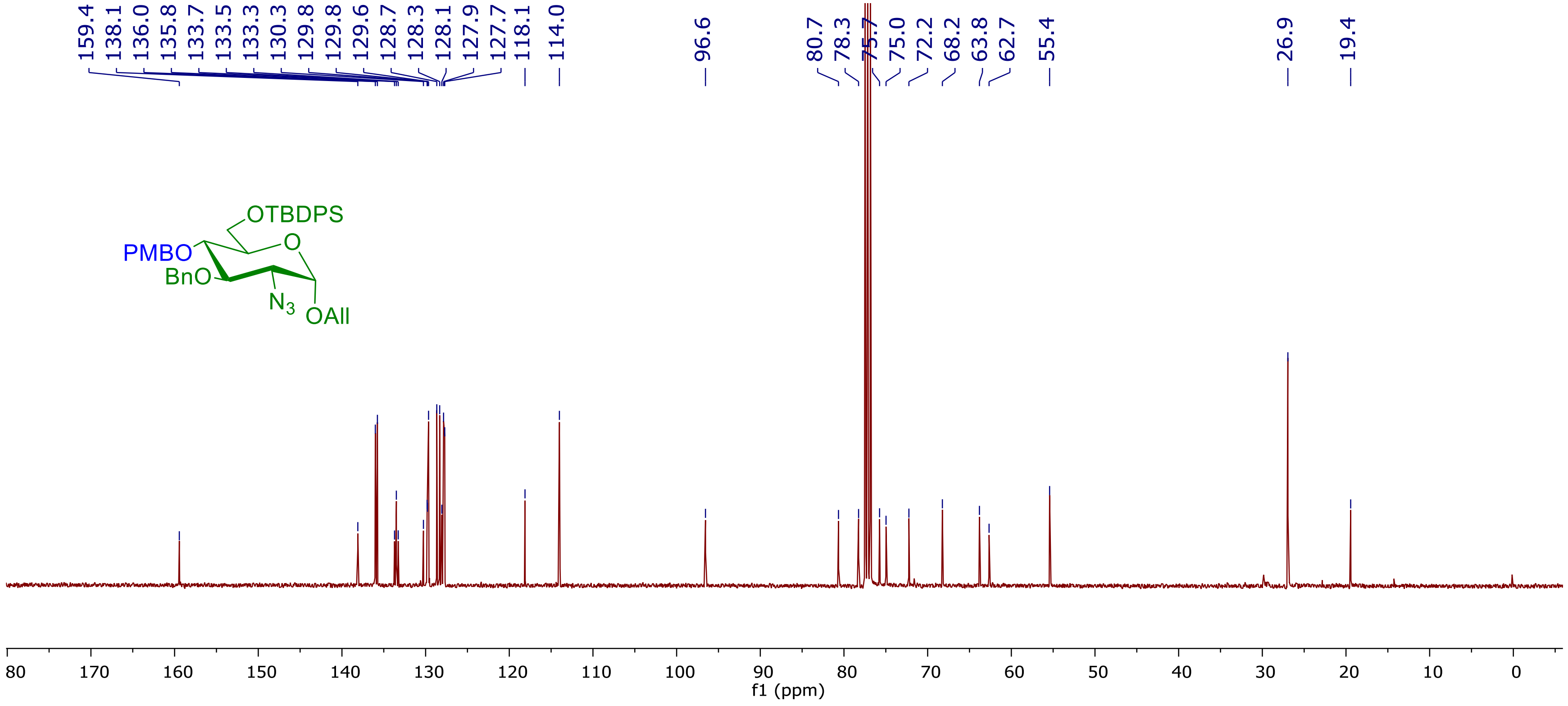

Supplementary Figure S46c. DEPT NMR Spectrum (100.67 MHz, CDCl₃) of Compound 32b

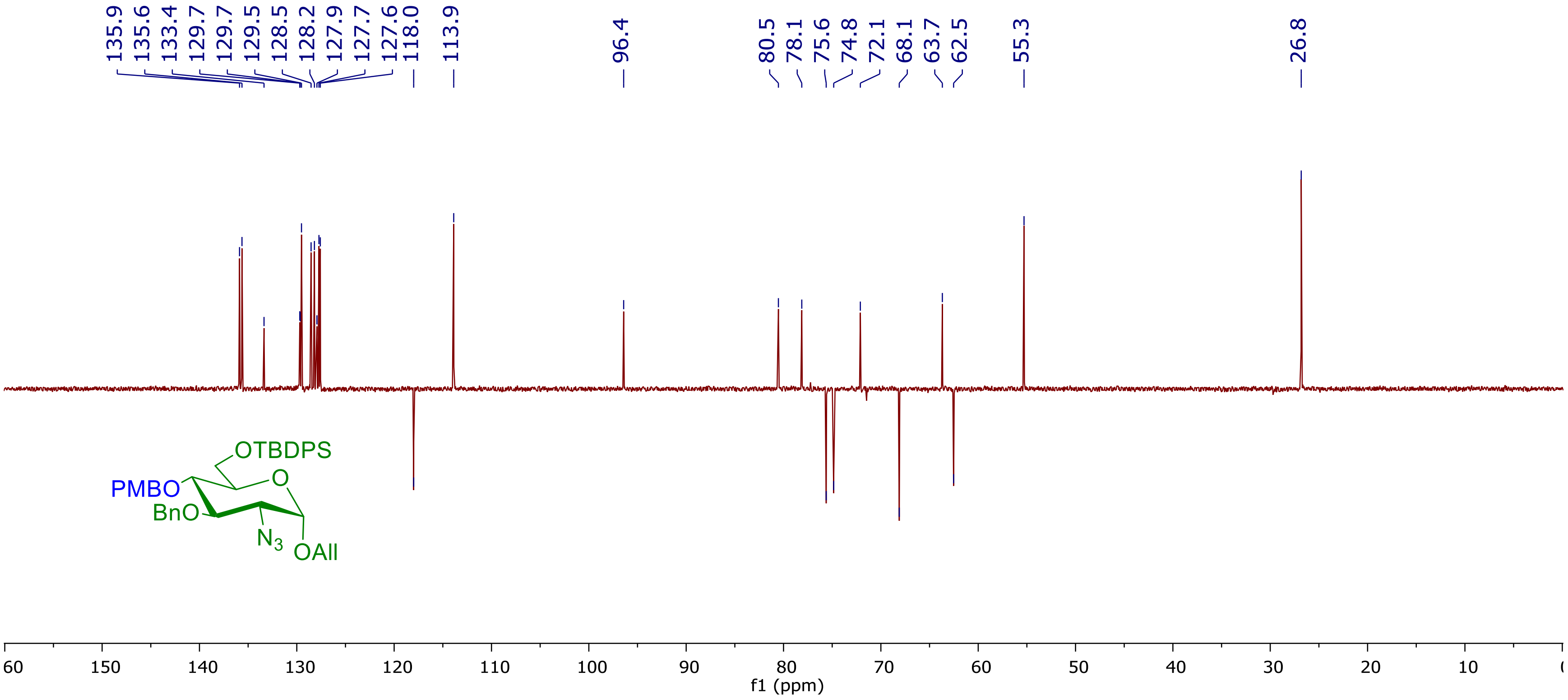

Supplementary Figure S47a. ¹H NMR Spectrum (400.31 MHz, $CDCl_3$) of Compound 32c

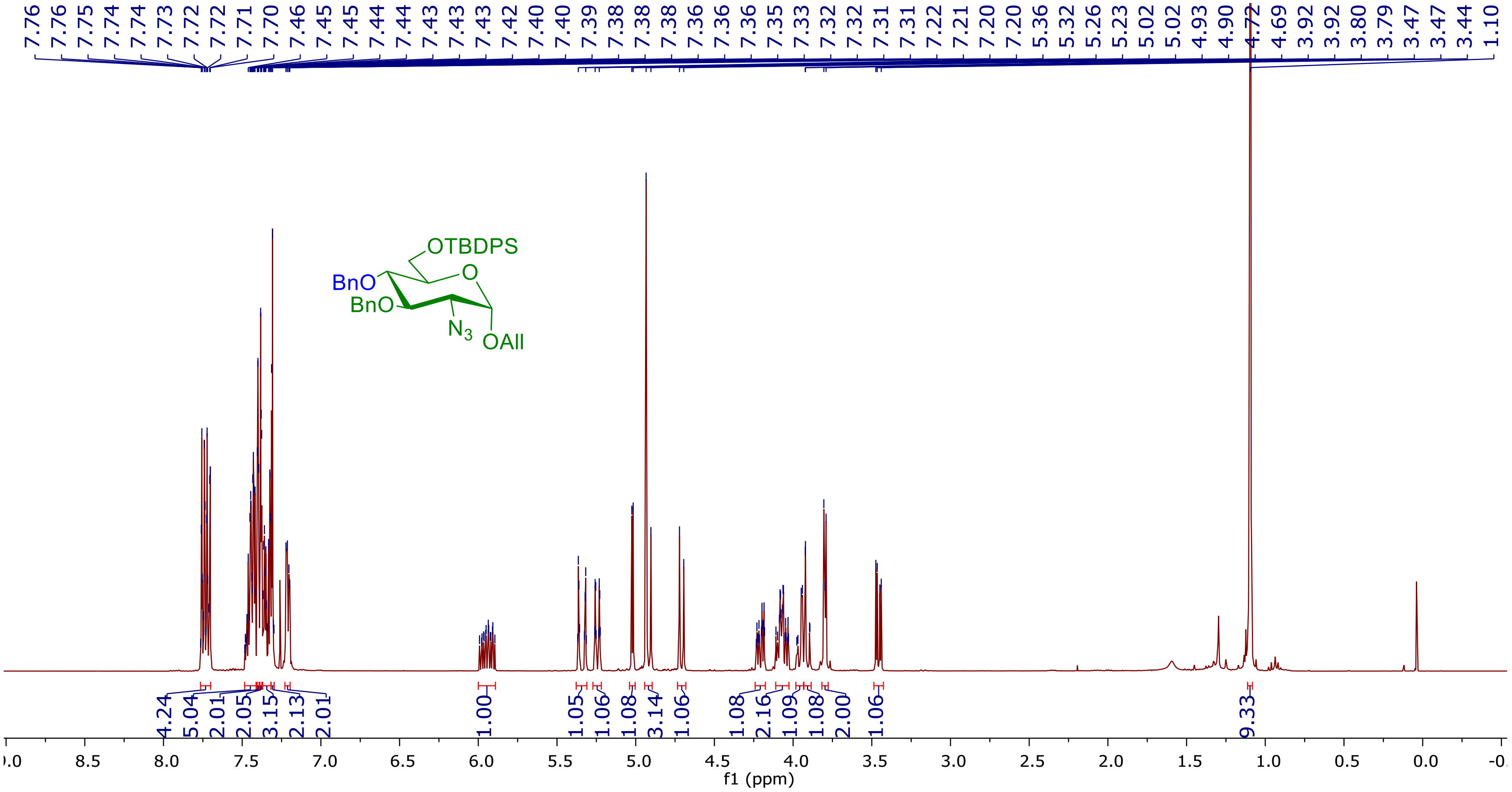

Supplementary Figure S47b. ^{13}C NMR Spectrum (100.67 MHz, $CDCl_3$) of Compound 32c

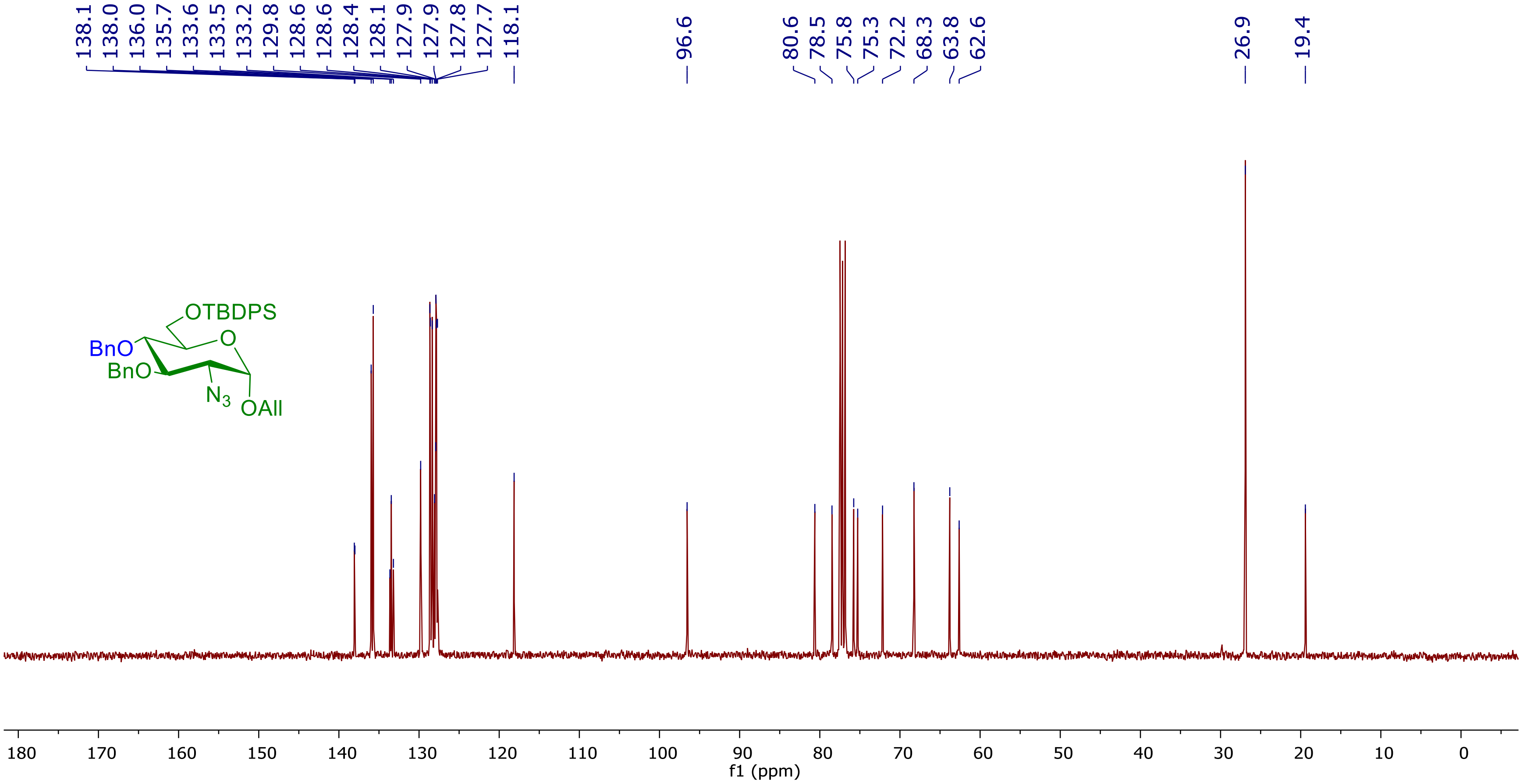

Supplementary Figure S47c. DEPT NMR Spectrum (100.67 MHz, $CDCl_3$) of Compound 32c

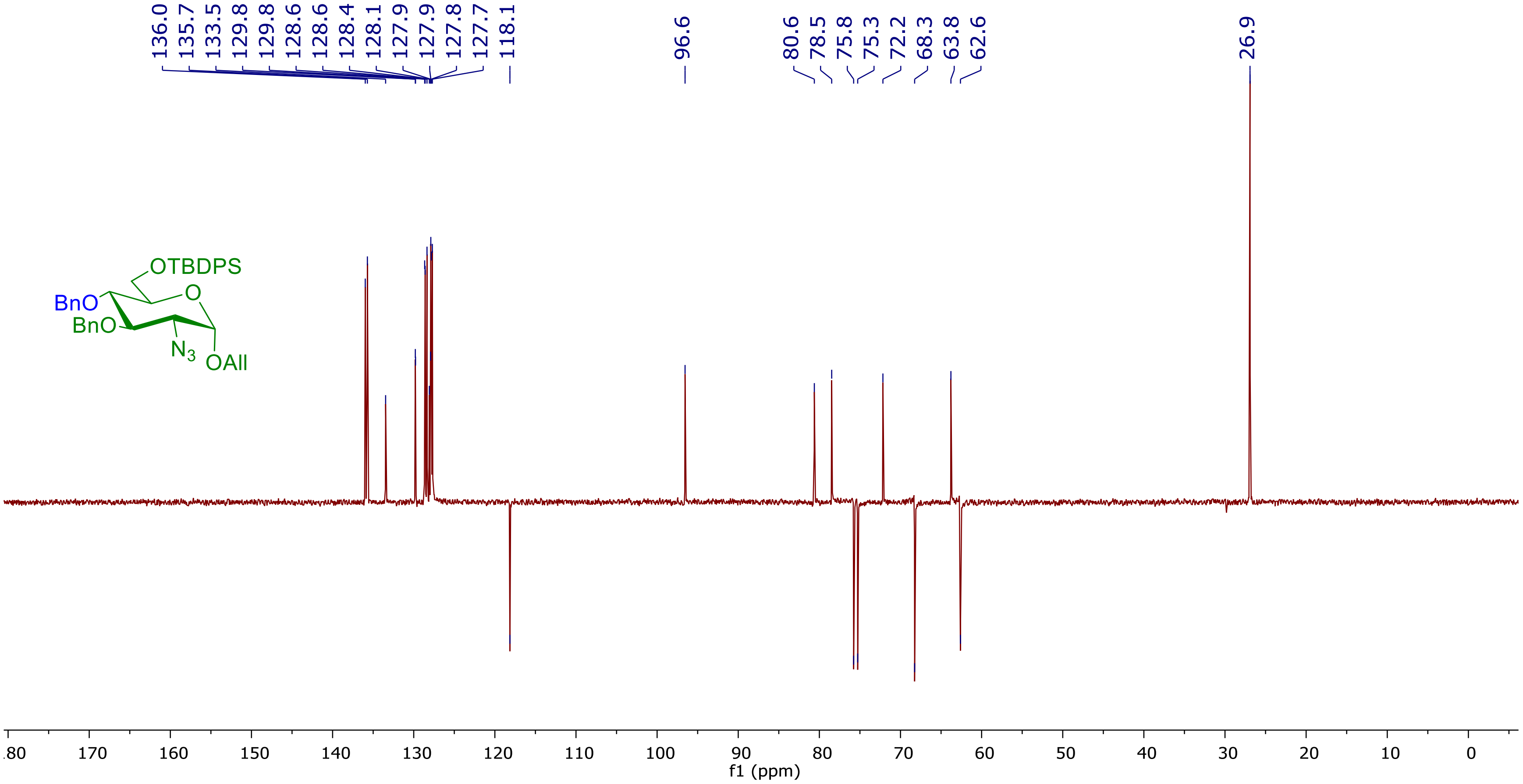

Supplementary Figure S48a. ^{1}H NMR Spectrum (400.31 MHz, $CDCl_3$) of Compound S8

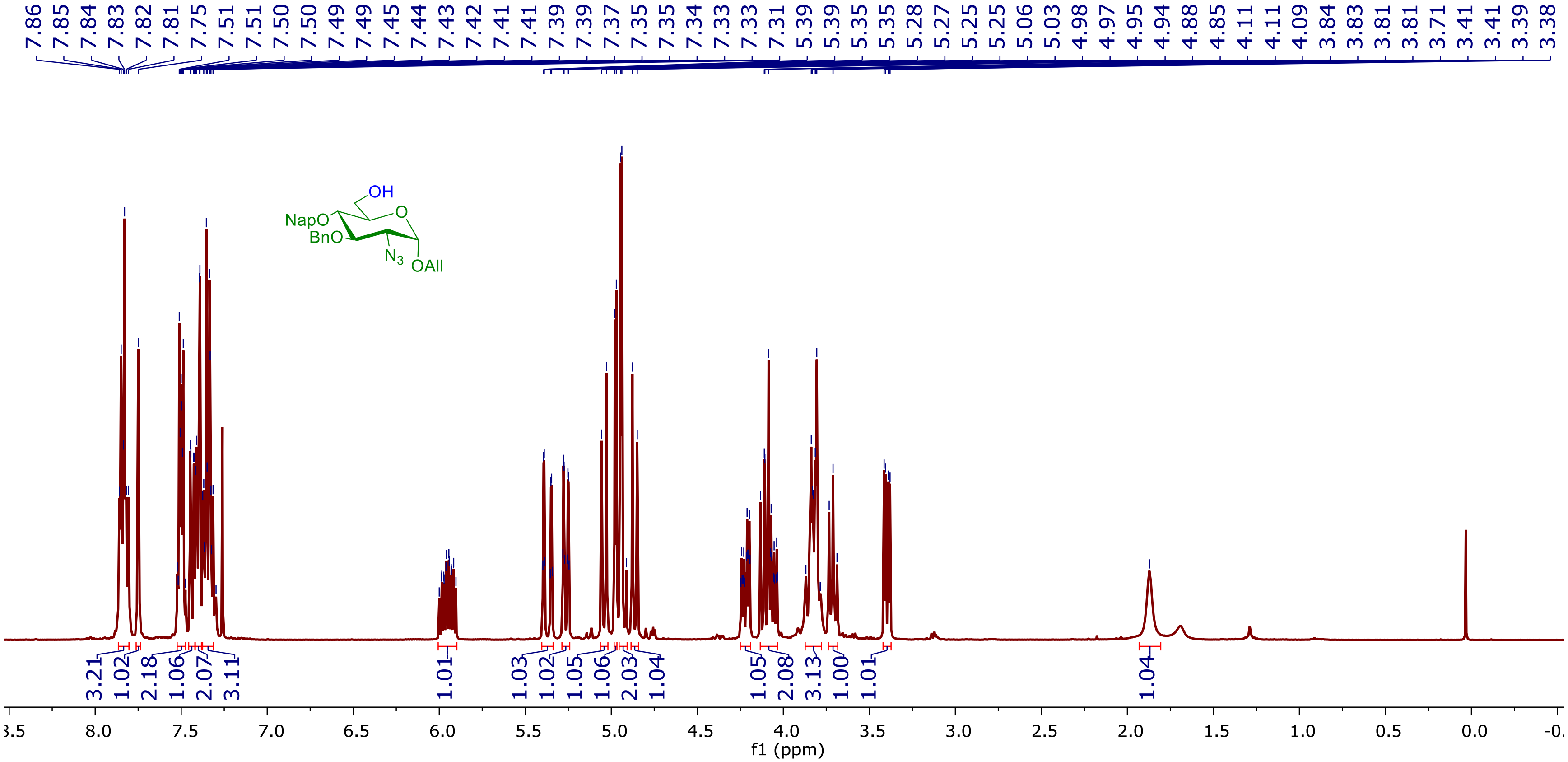

Supplementary Figure S48b. ^{13}C NMR Spectrum (100.66 MHz, $CDCl_3$) of Compound S8

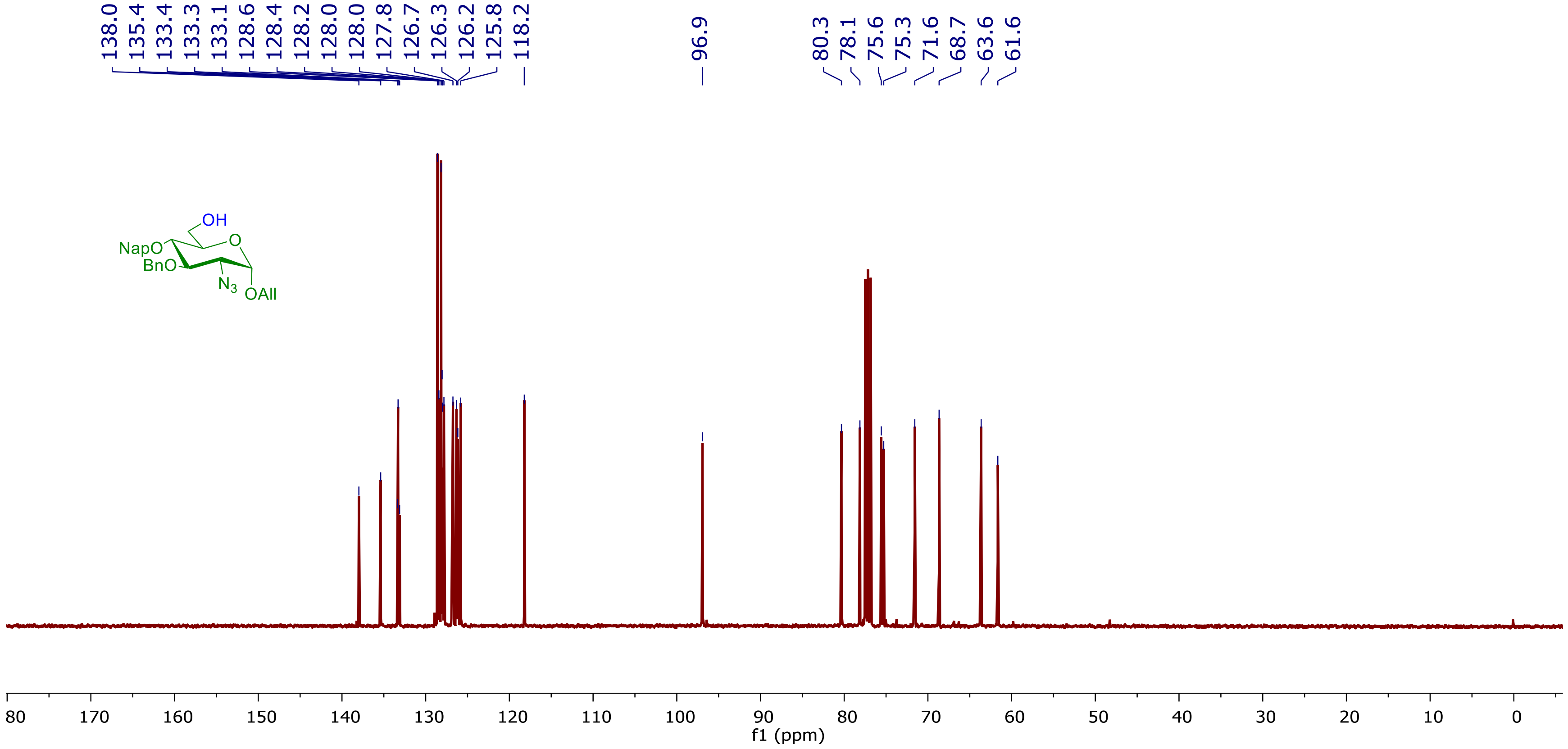

Supplementary Figure S48c. DEPT NMR Spectrum (100.66 MHz, CDCl₃) of Compound S8

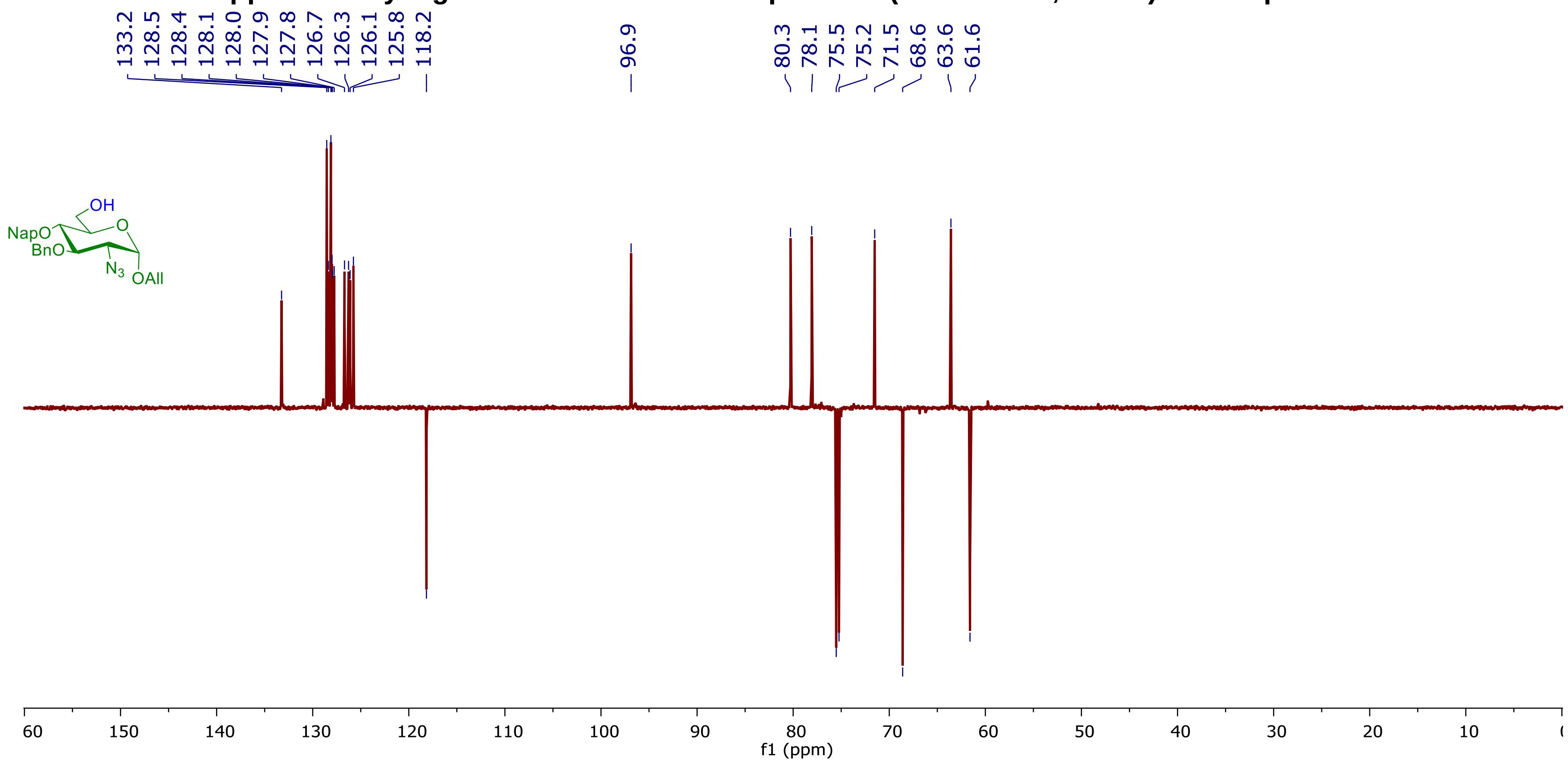

Supplementary Figure S49a. ^{1}H NMR Spectrum (400.31 MHz, $CDCl_3$) of Compound 32d

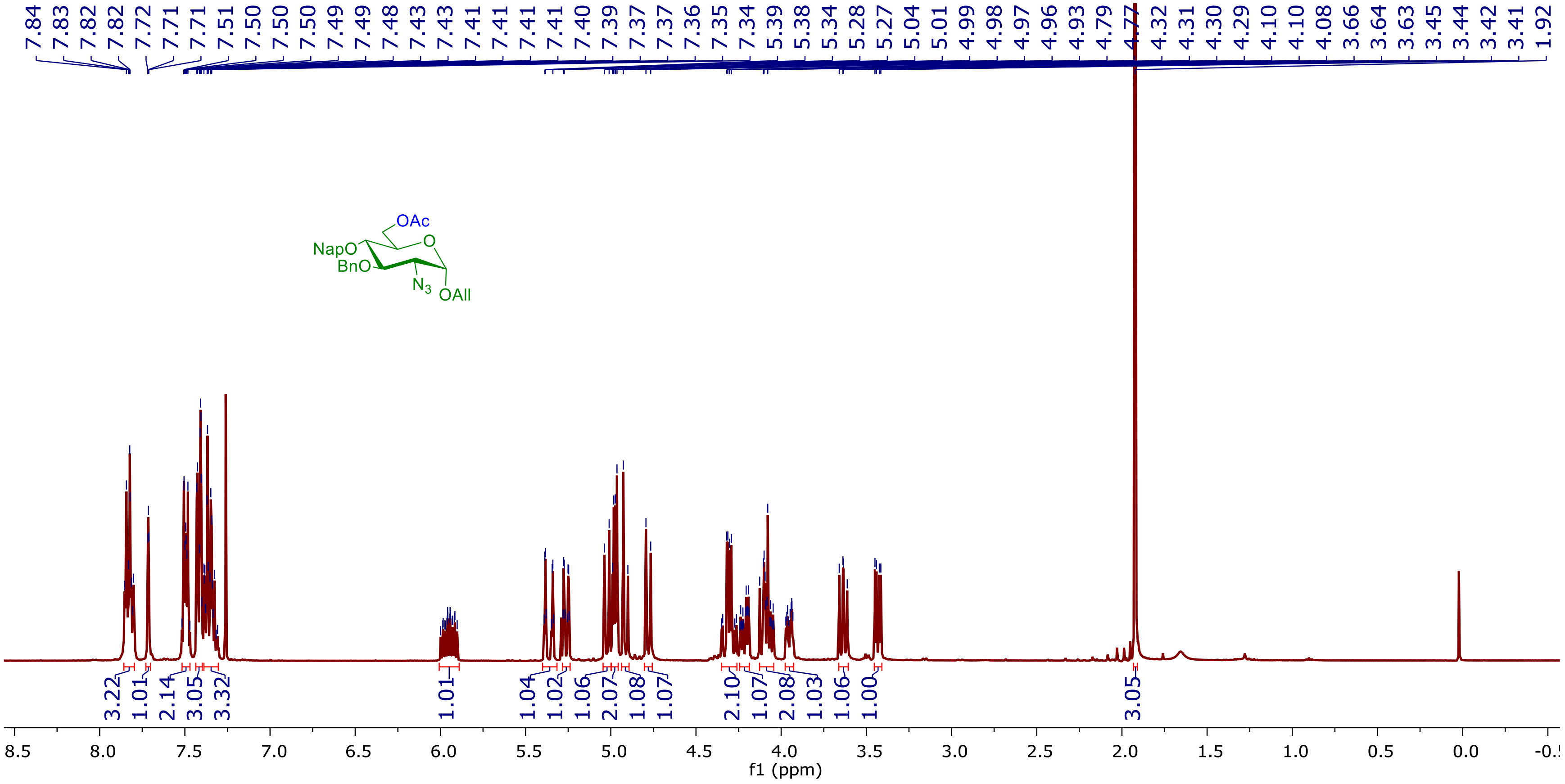

Supplementary Figure S49b. ^{13}C NMR Spectrum (100.66 MHz, $CDCl_3$) of Compound 32d

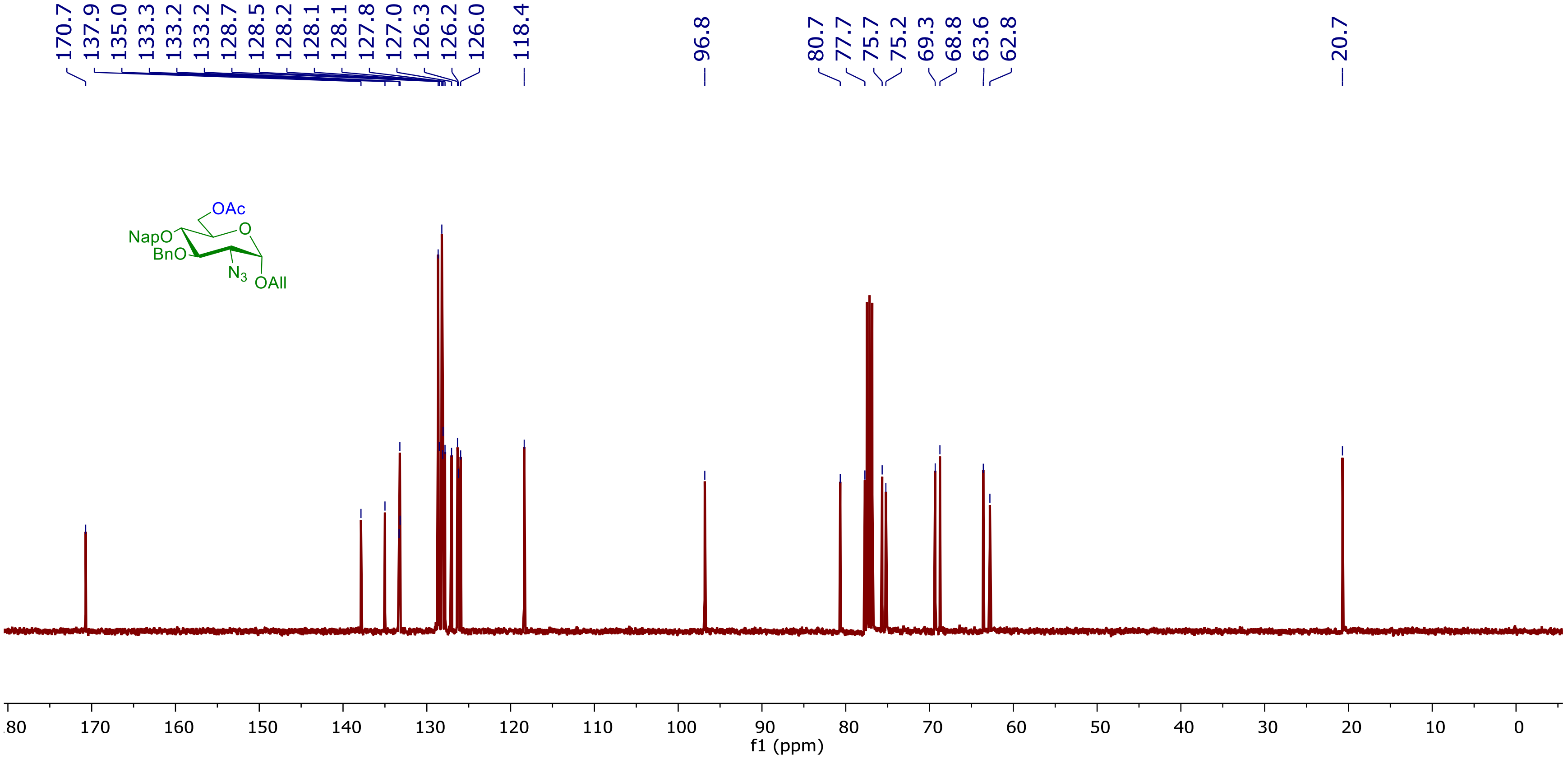

Supplementary Figure S49c. DEPT NMR Spectrum (100.66 MHz, $CDCl_3$) of Compound 32d

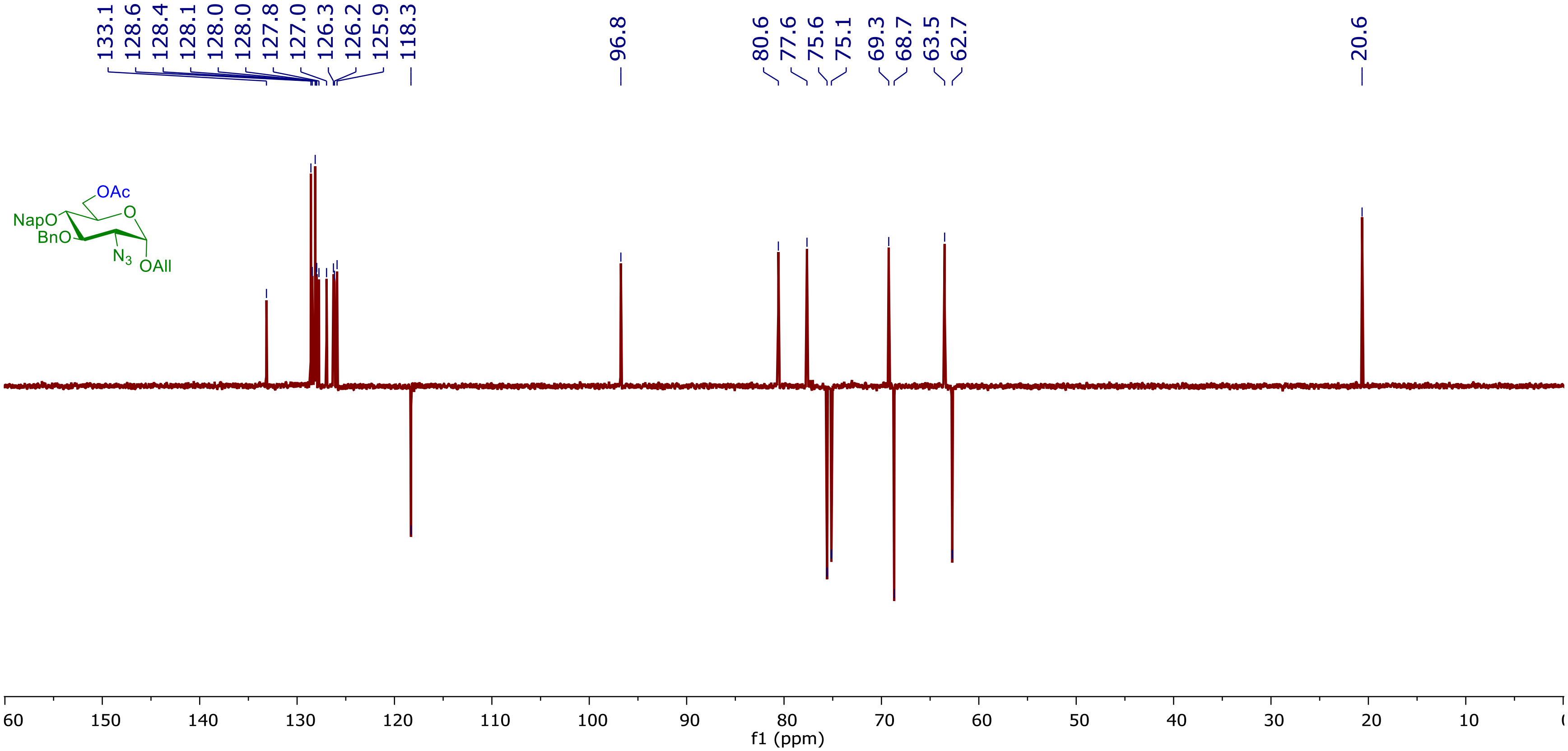

Supplementary Figure S50a. ¹H NMR Spectrum (400.31 MHz, $CDCl_3$) of Compound 32e

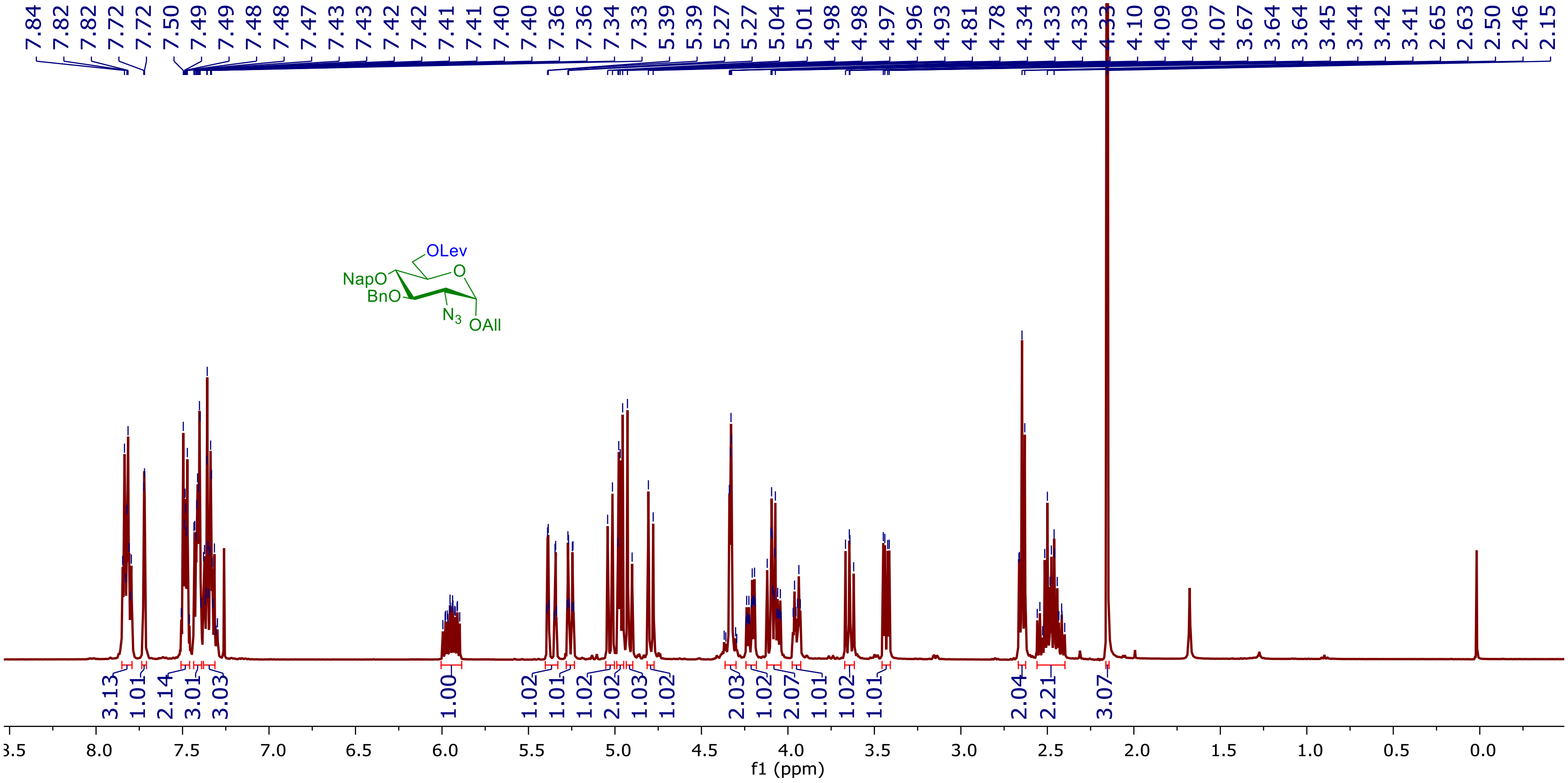

Supplementary Figure S50b. ^{13}C NMR Spectrum (100.66 MHz, $CDCl_3$) of Compound 32e

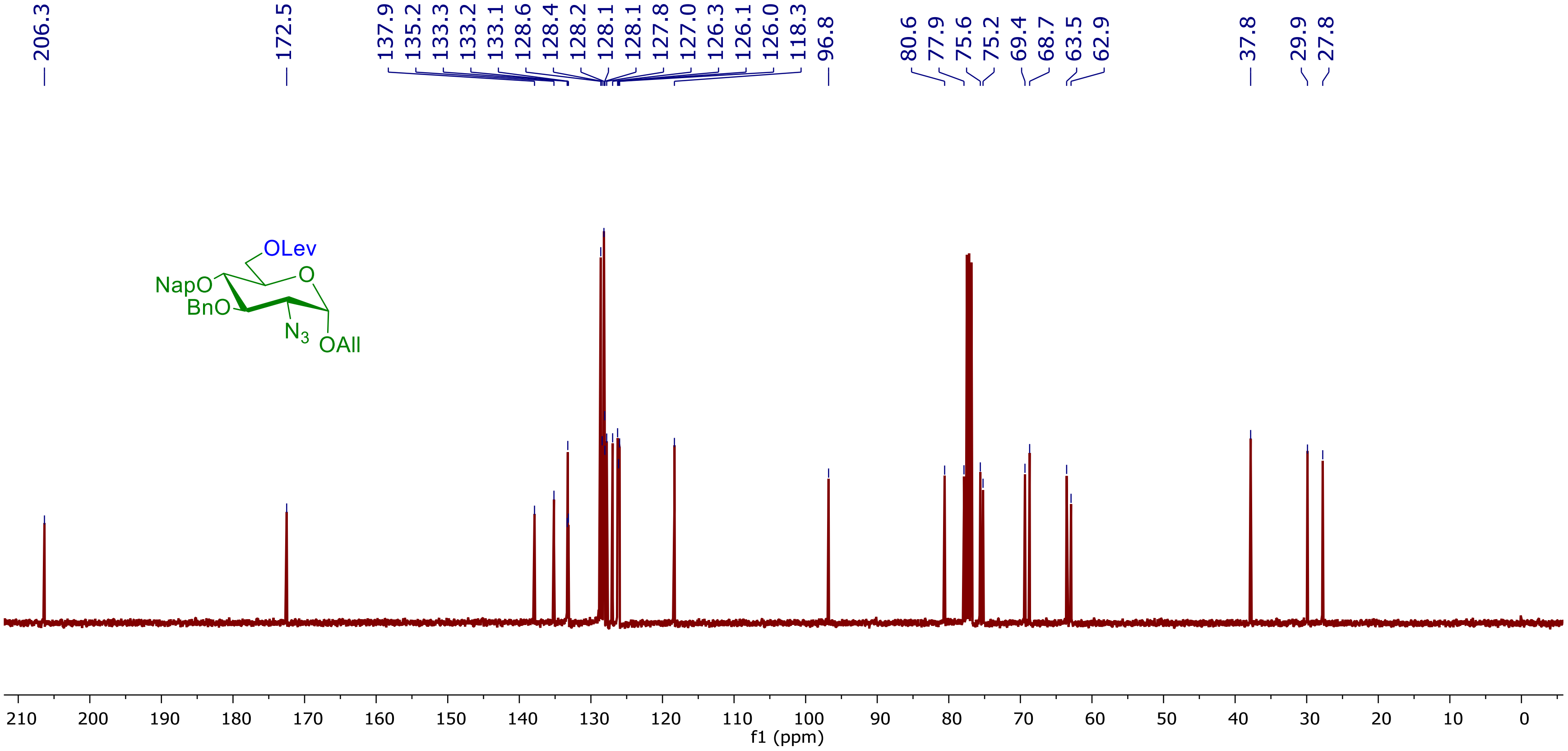

Supplementary Figure S50c. DEPT NMR Spectrum (100.66 MHz, $CDCl_3$) of Compound 32e

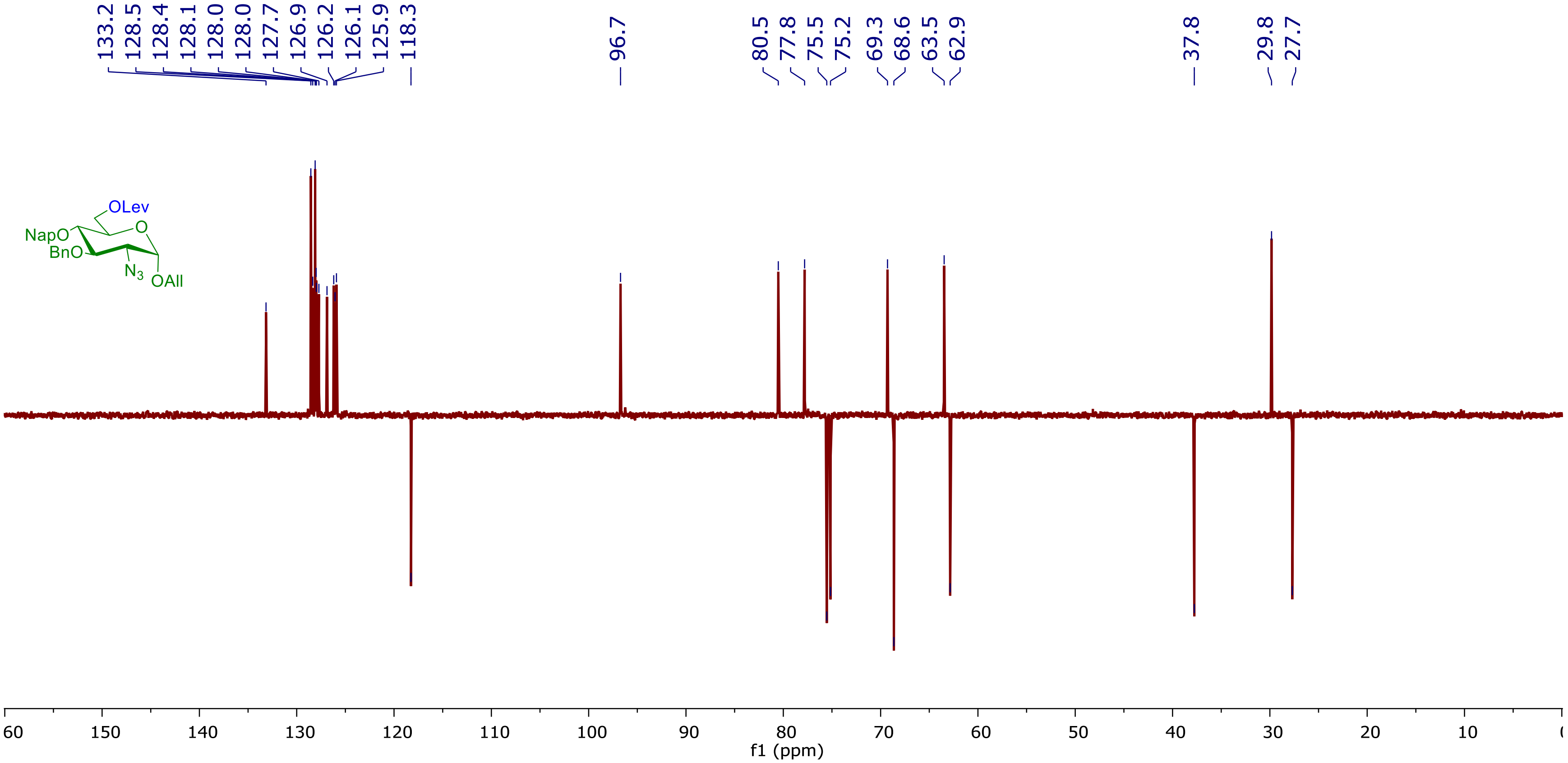

Supplementary Figure S51a. ^{1}H NMR Spectrum (400.31 MHz, $CDCl_3$) of Compound S9a

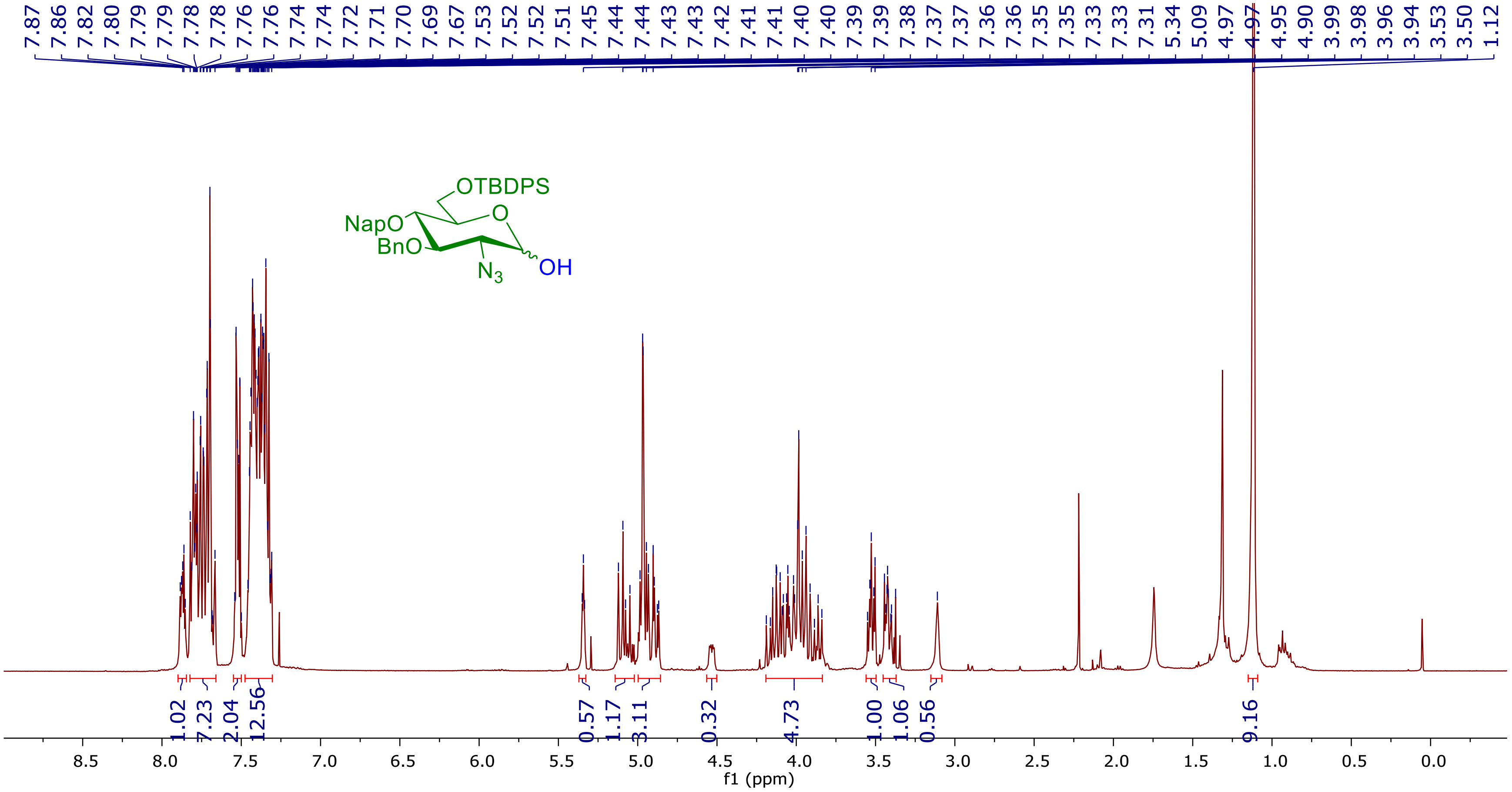

Supplementary Figure S51b. ^{13}C NMR Spectrum (100.67 MHz, $CDCl_3$) of Compound S9a

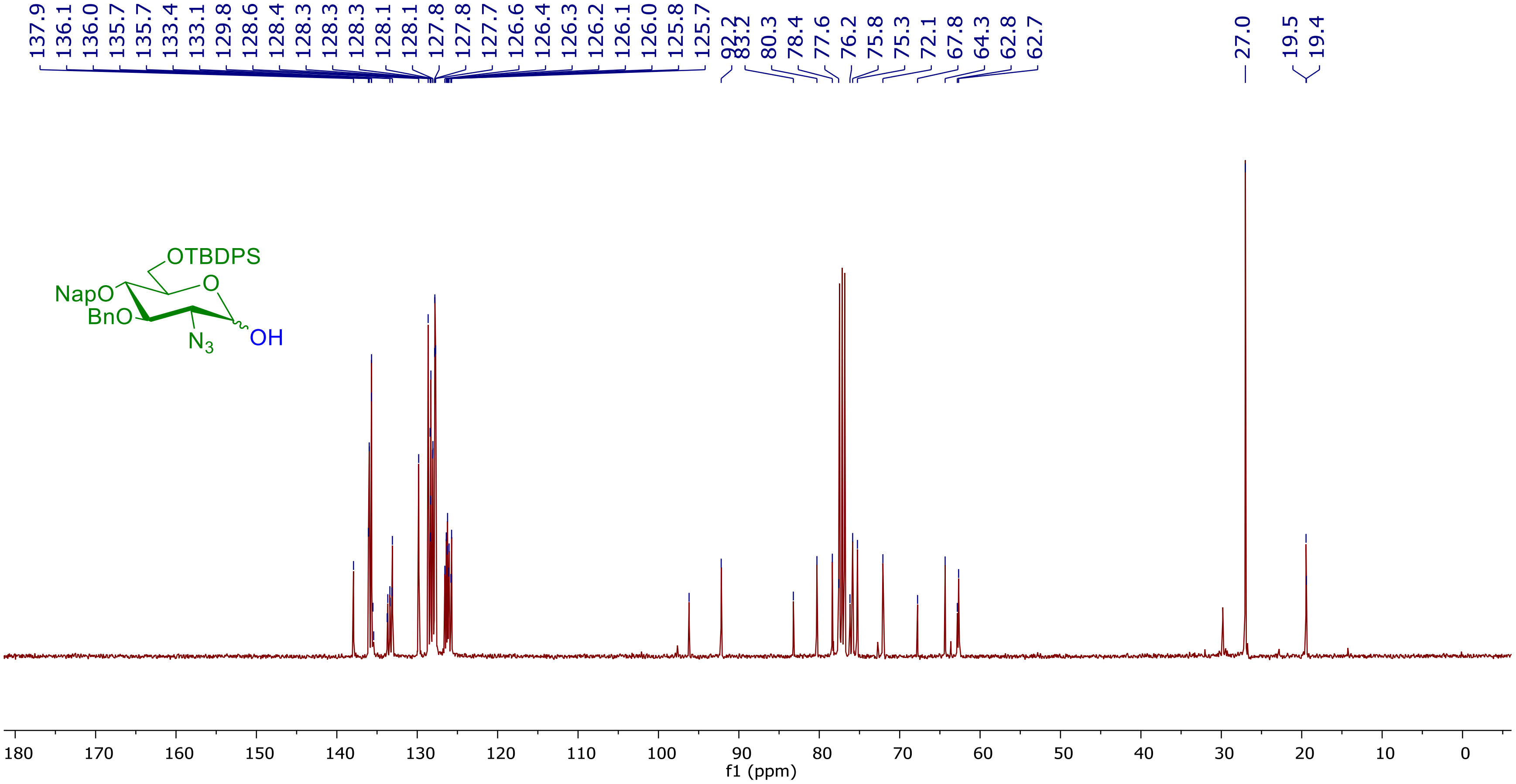

Supplementary Figure S51c. DEPT NMR Spectrum (100.67 MHz, $CDCl_3$) of Compound S9a

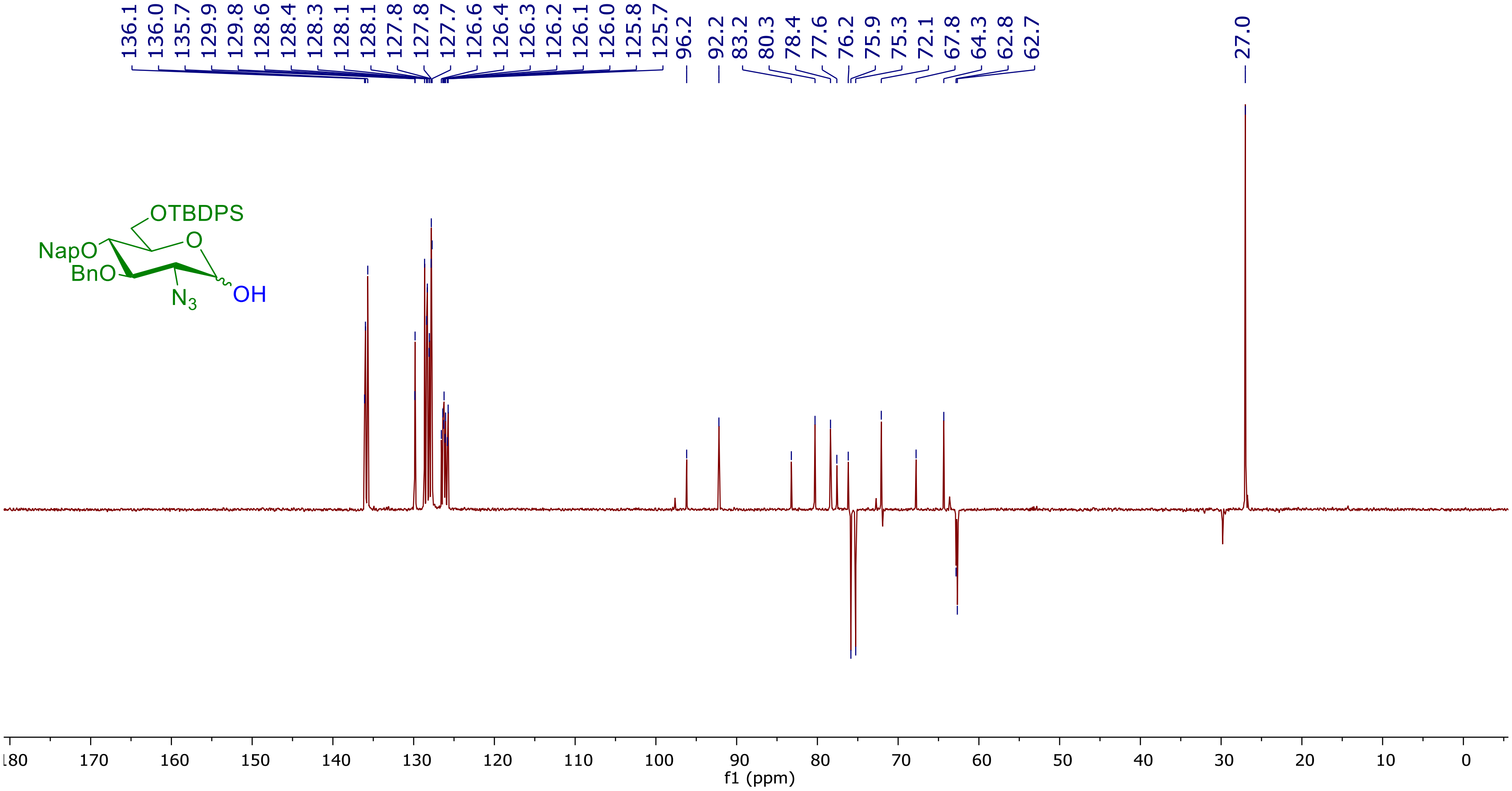

Supplementary Figure S52a. ^{1}H NMR Spectrum (400.31 MHz, $CDCl_3$) of Compound S9c

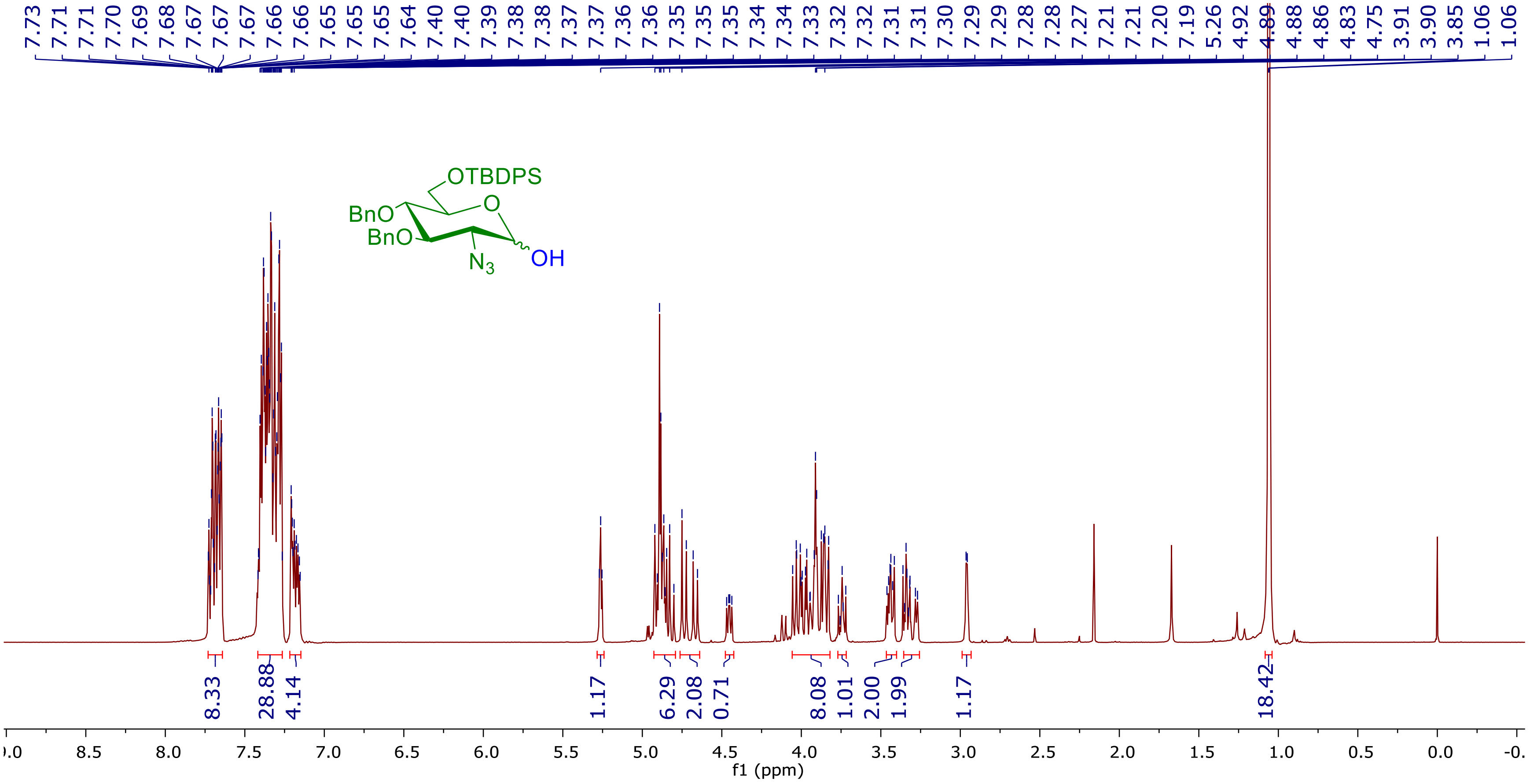

Supplementary Figure S52b. ^{13}C NMR Spectrum (100.67 MHz, $CDCl_3$) of Compound S9c

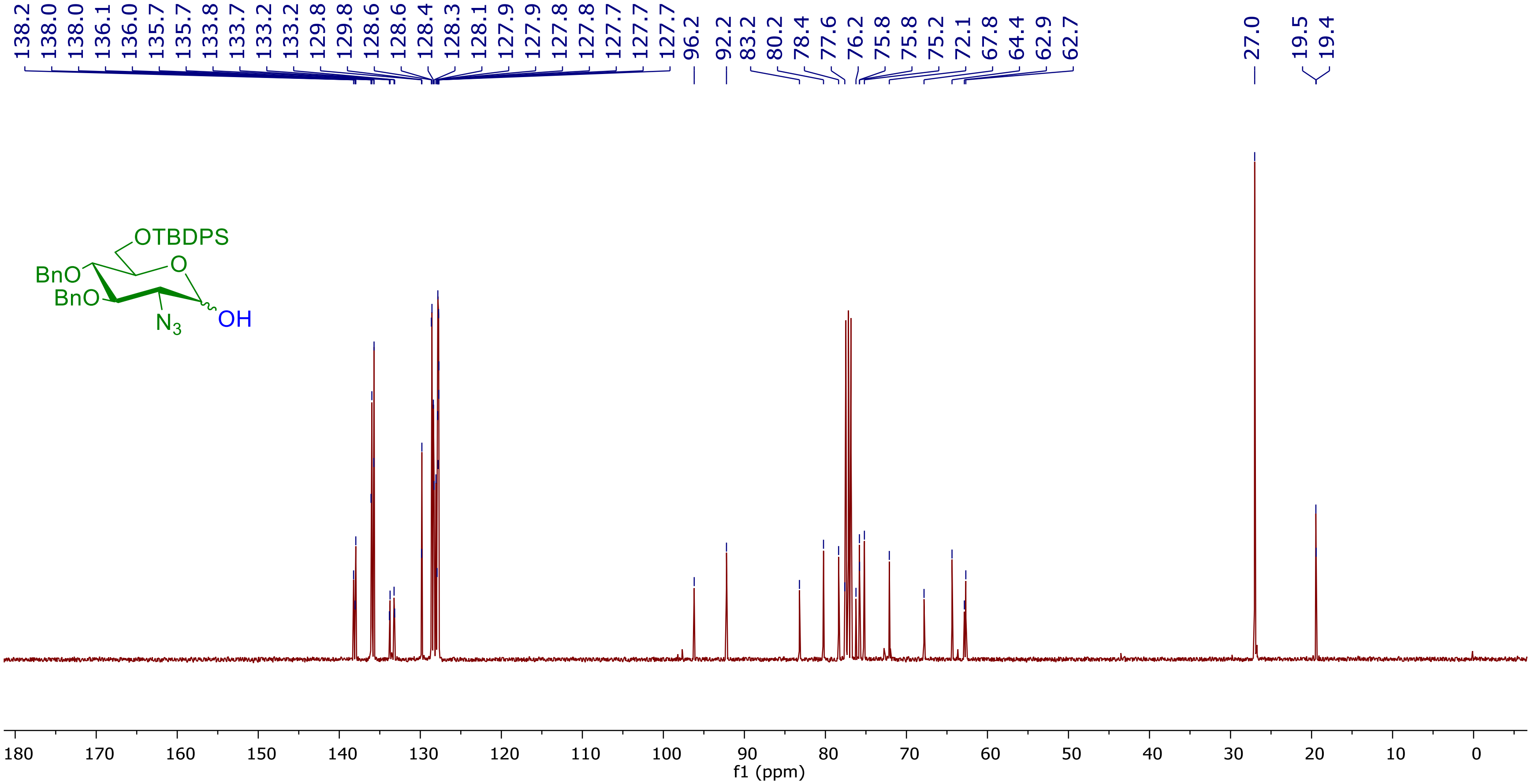

Supplementary Figure S52c. DEPT NMR Spectrum (100.67 MHz, $CDCl_3$) of Compound S9c

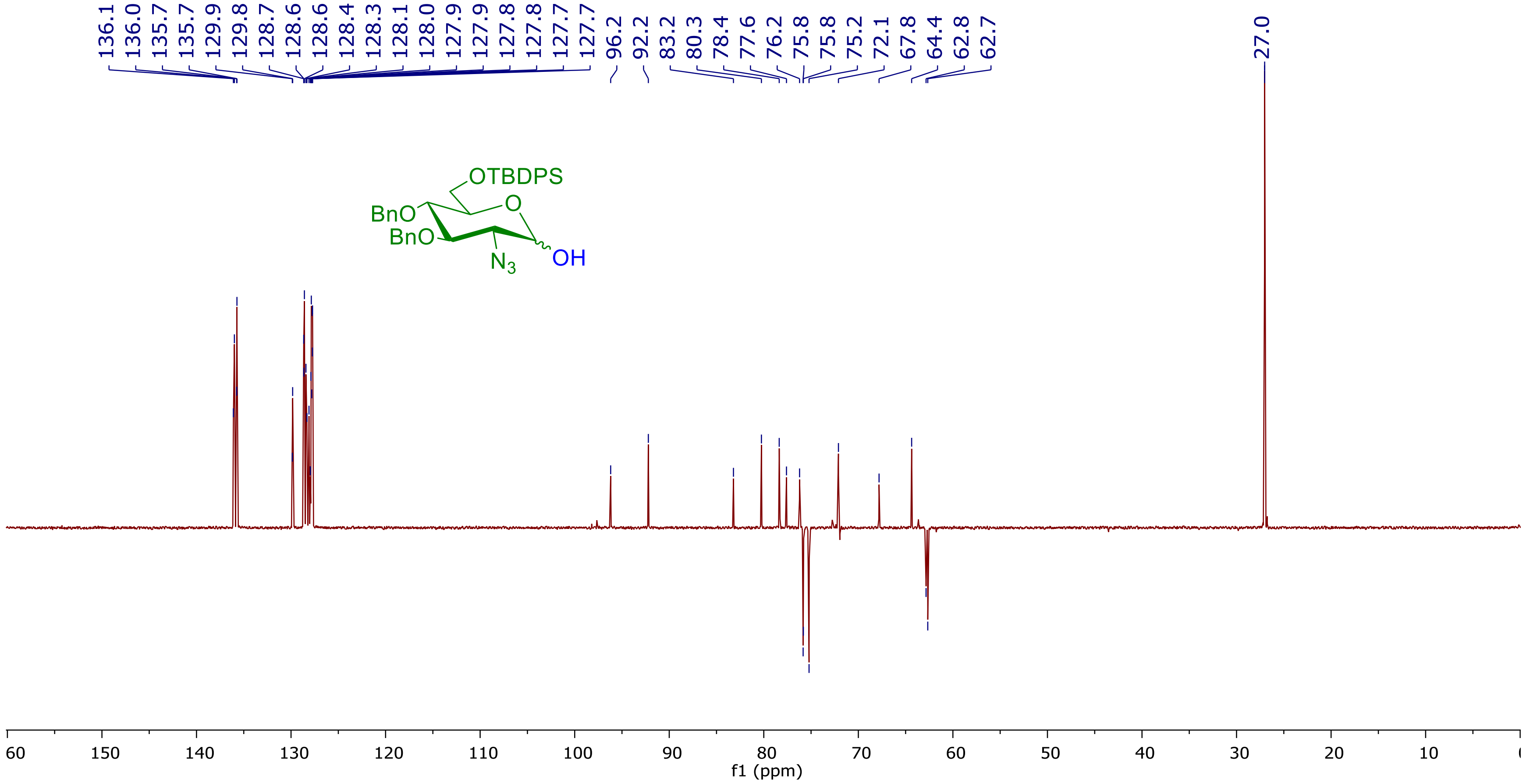

Supplementary Figure S53a. ^{1}H NMR Spectrum (400.31 MHz, $CDCl_3$) of Compound S9d

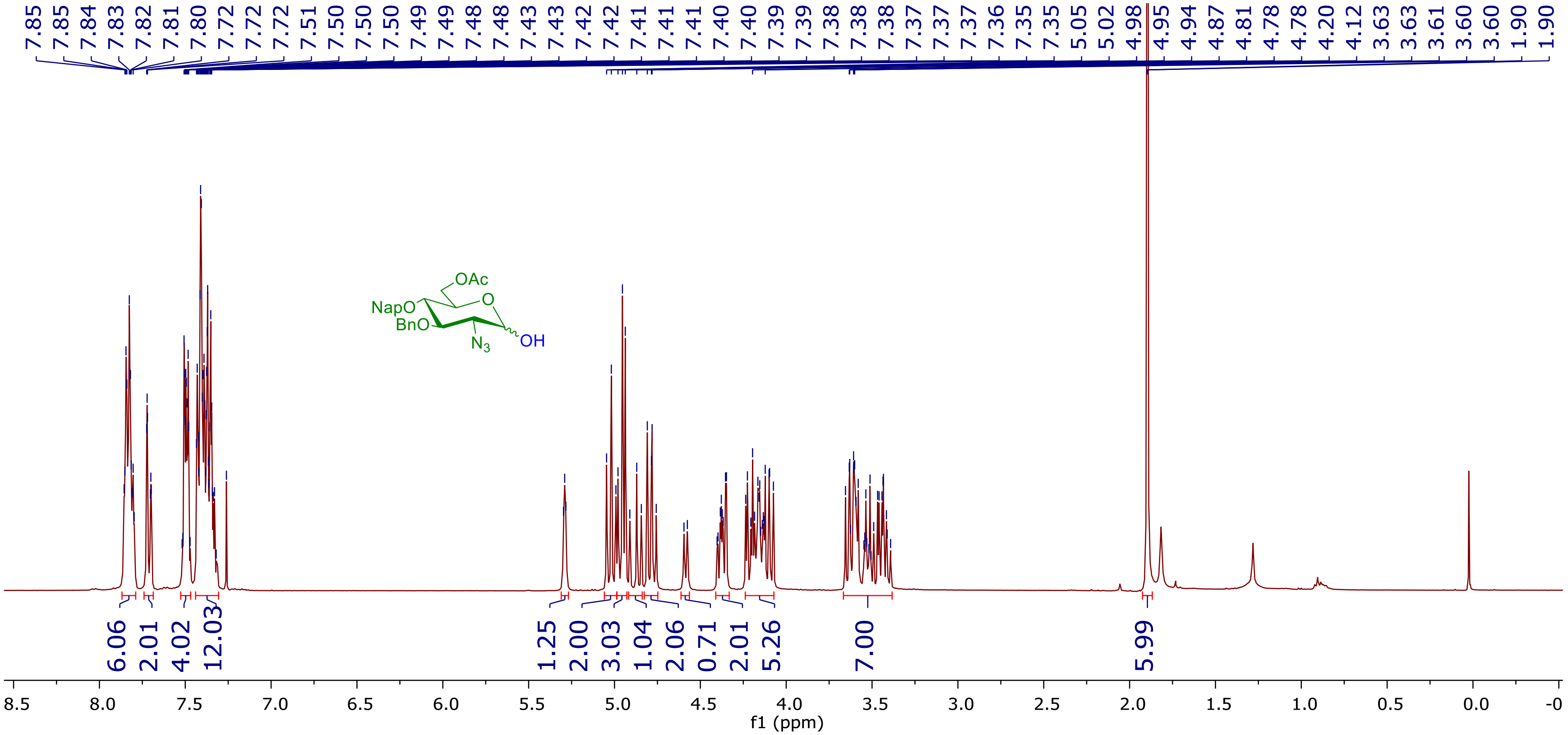

Supplementary Figure S53b. ^{13}C NMR Spectrum (100.67 MHz, $CDCl_3$) of Compound S9d

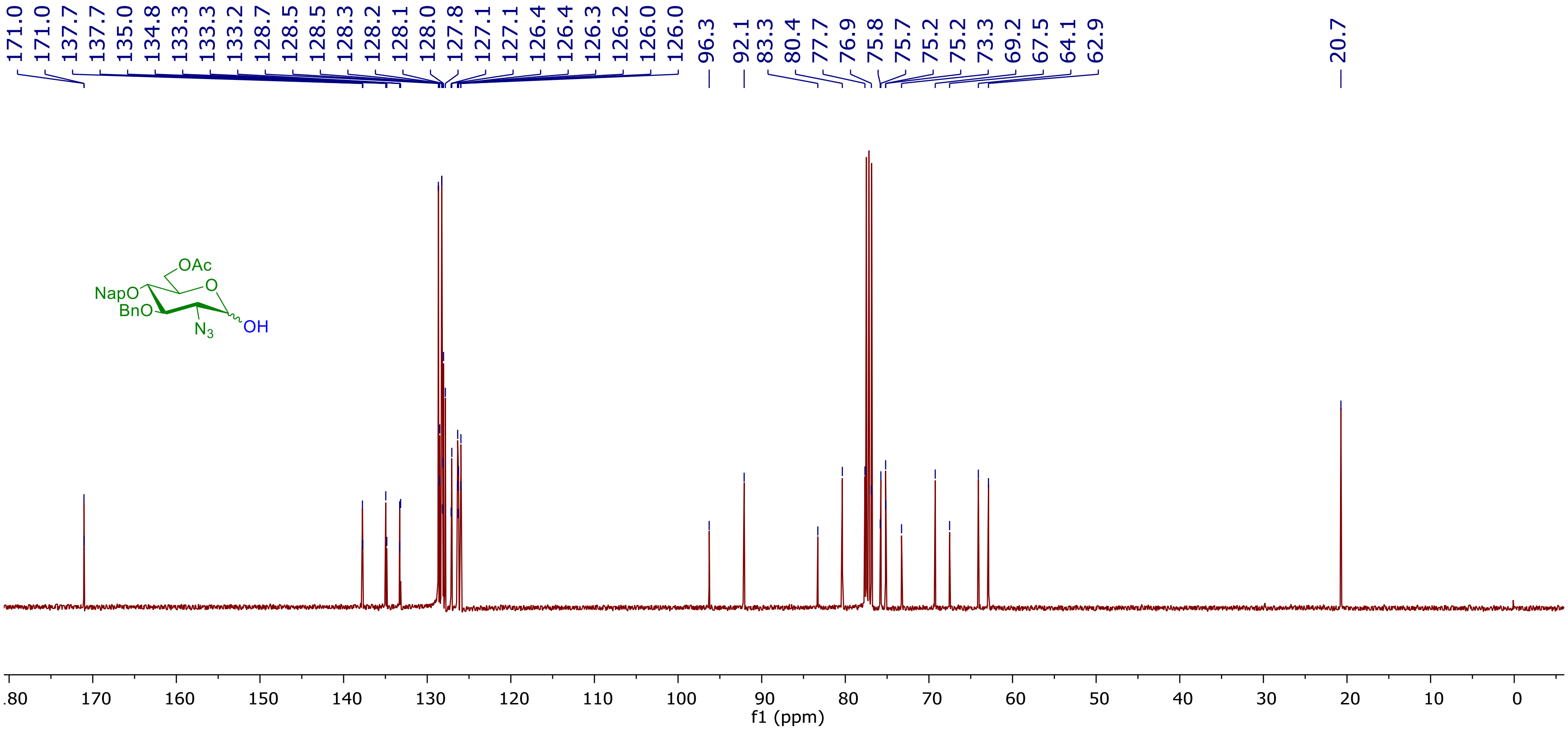

Supplementary Figure S53c. DEPT NMR Spectrum (100.67 MHz, $CDCl_3$) of Compound S9d

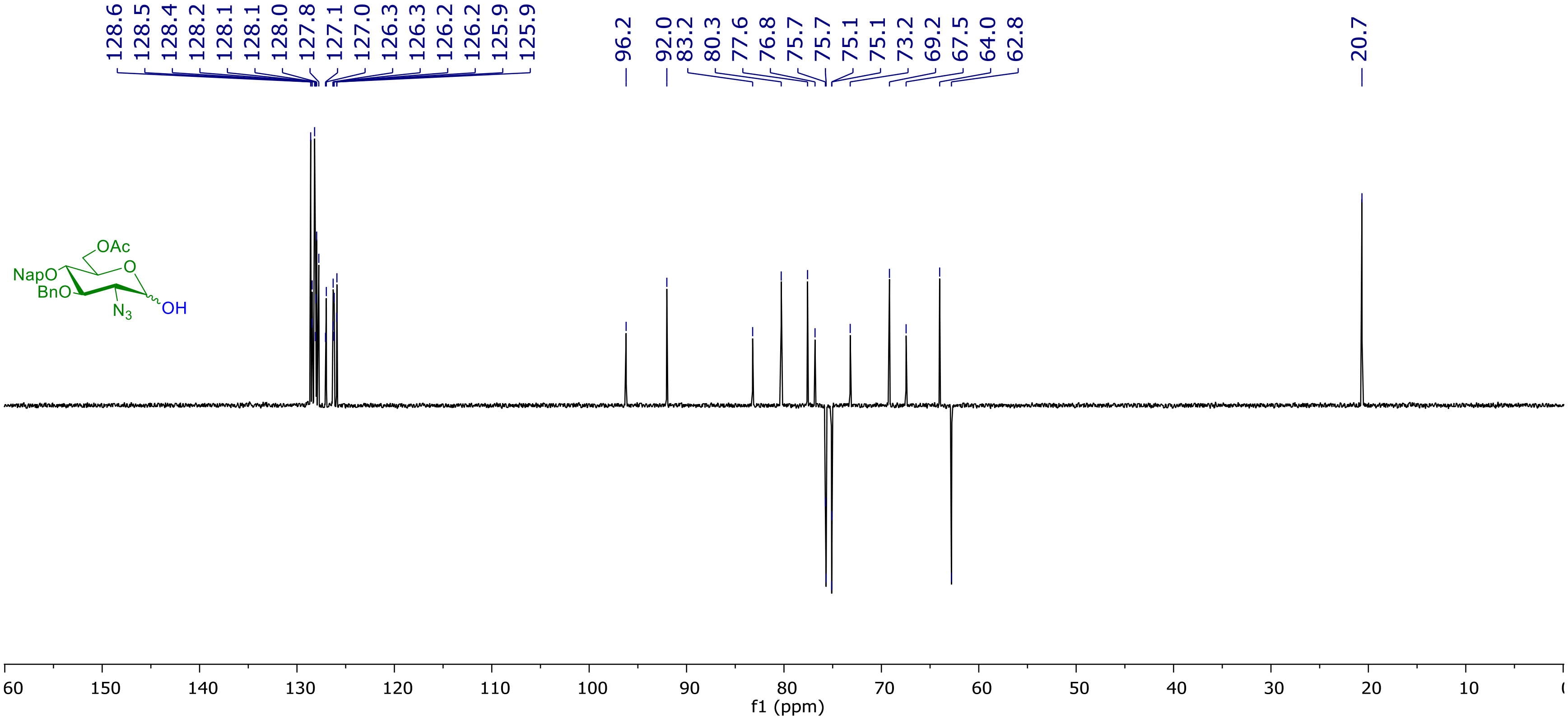

Supplementary Figure S54a. ¹H NMR Spectrum (400.31 MHz, $CDCl_3$) of Compound S9e

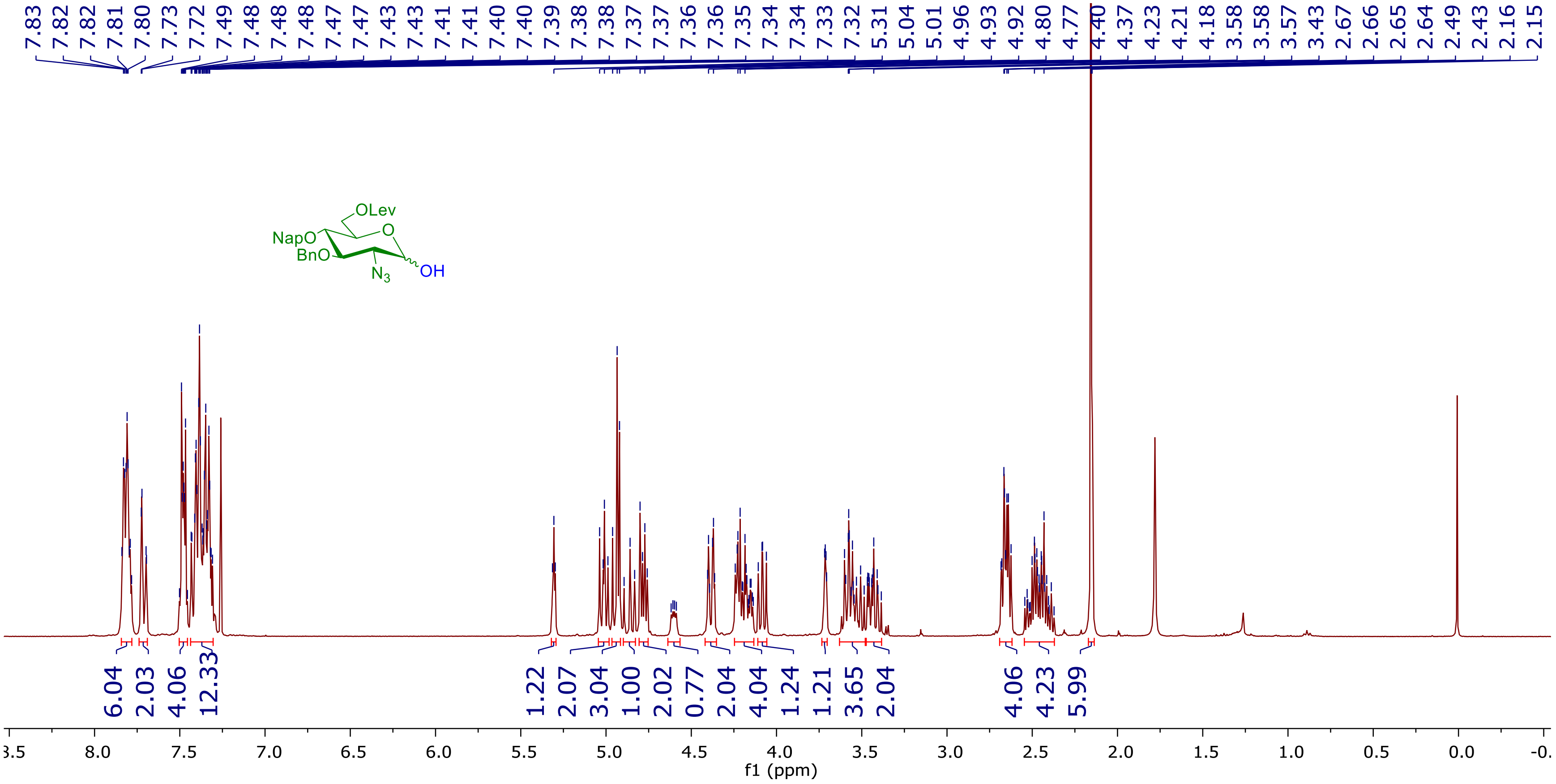

Supplementary Figure S54b. ^{13}C NMR Spectrum (100.66 MHz, $CDCl_3$) of Compound S9e

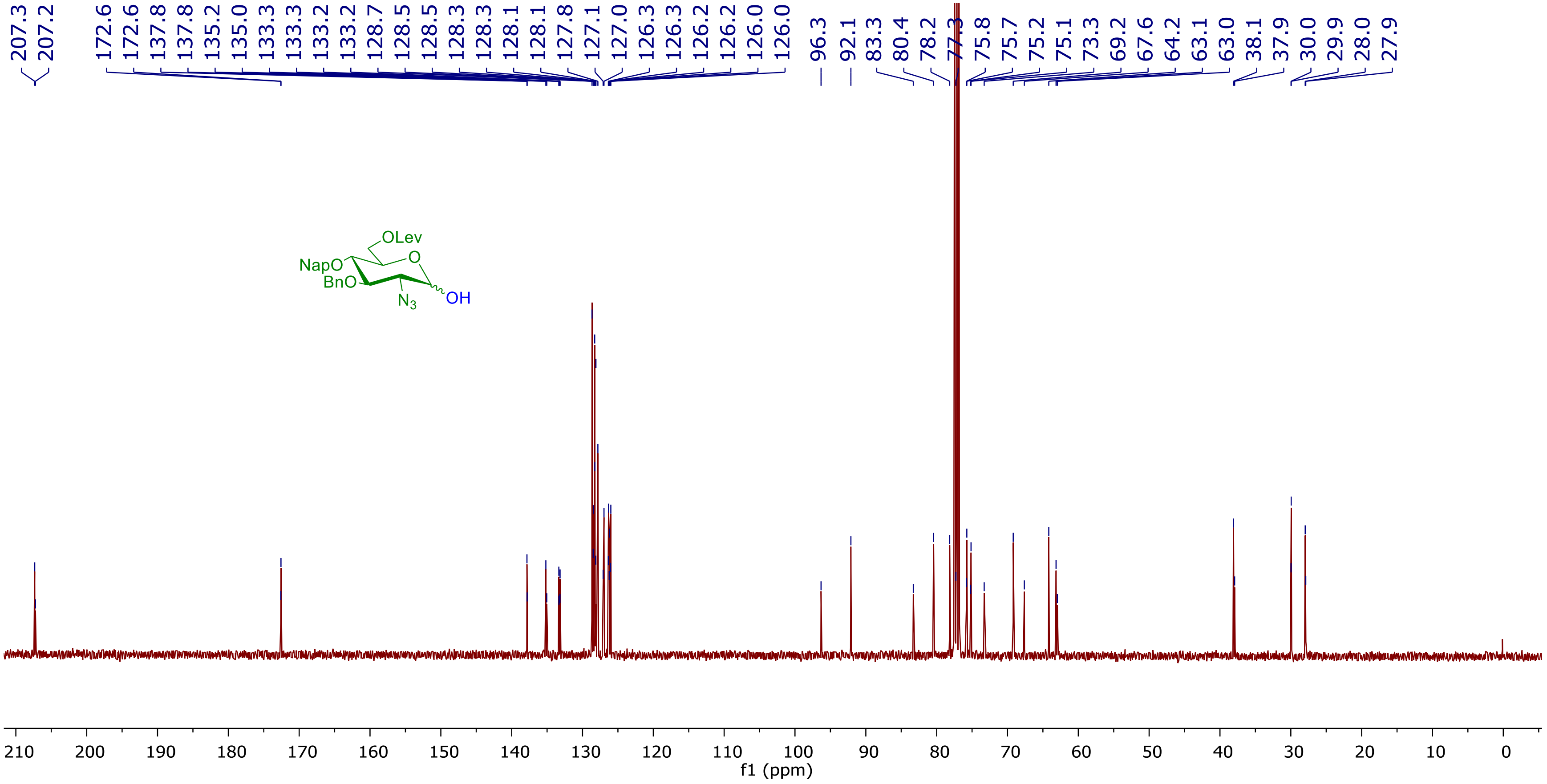

Supplementary Figure S54c. DEPT NMR Spectrum (100.66 MHz, $CDCl_3$) of Compound S9e

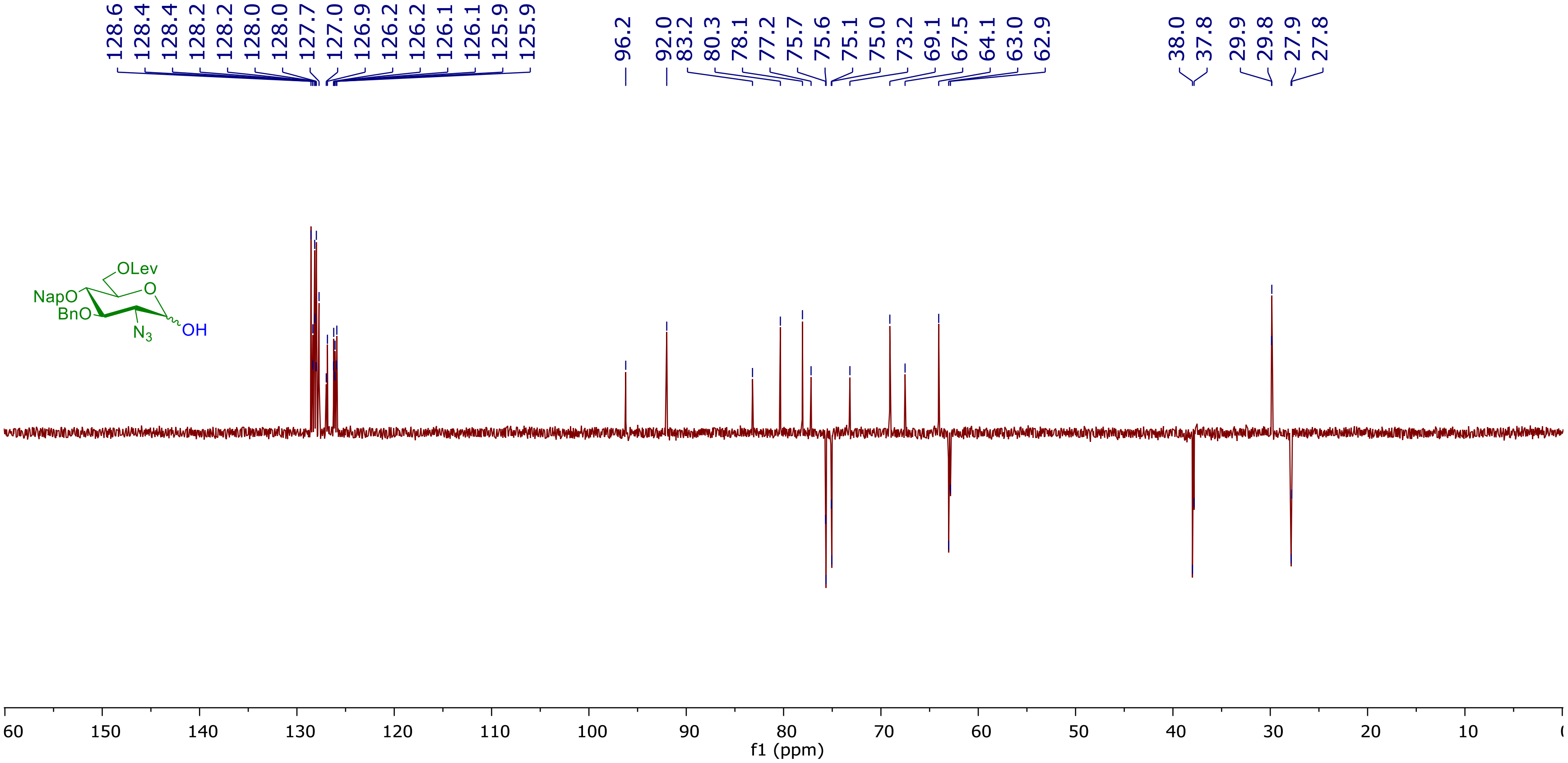

Supplementary Figure S55a. ^{1}H NMR Spectrum (400.31 MHz, $CDCl_3$) of Compound 33a (α isomer)

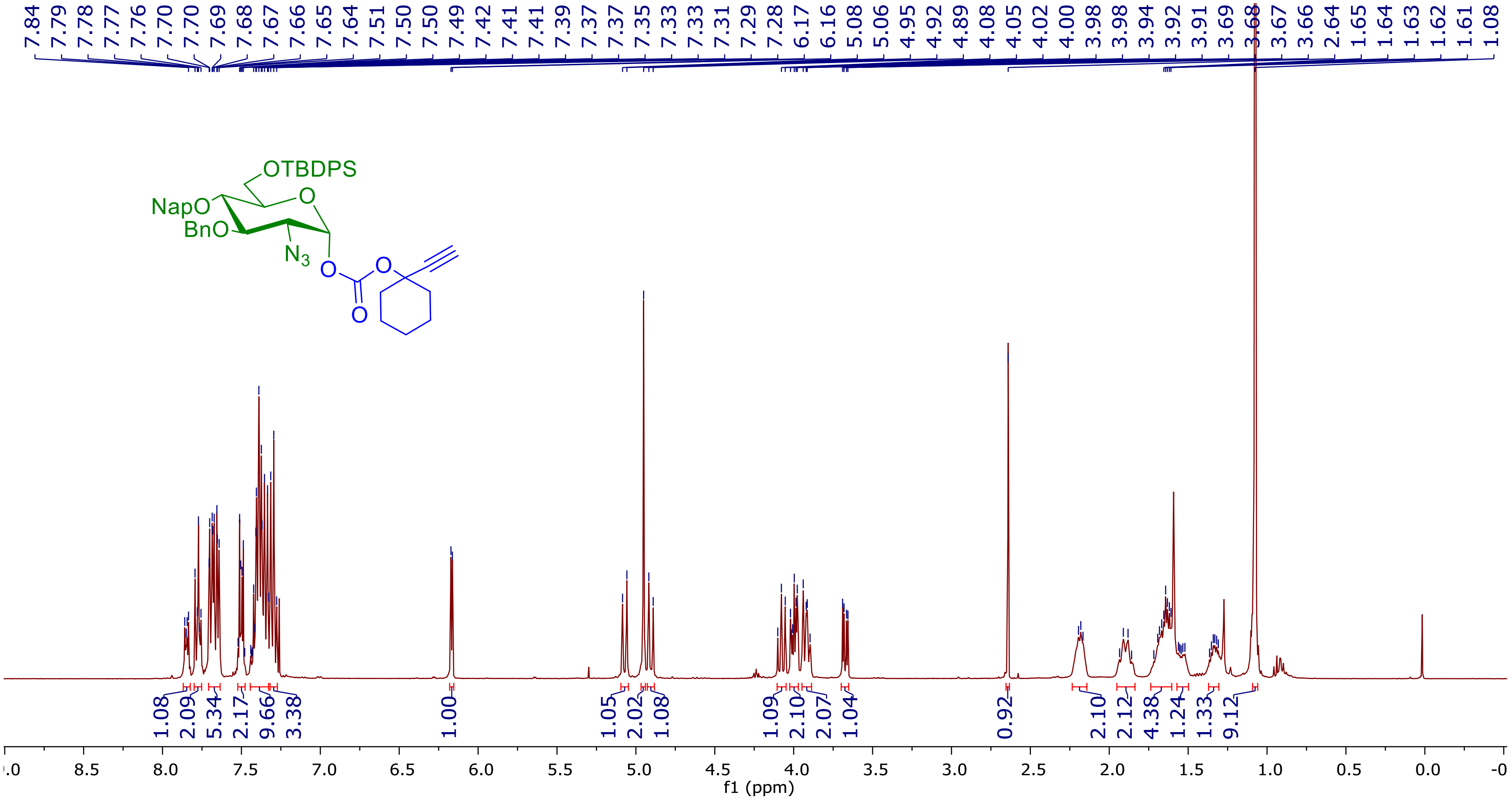

Supplementary Figure S55b. ^{13}C NMR Spectrum (100.67 MHz, $CDCl_3$) of Compound 33a (α isomer)

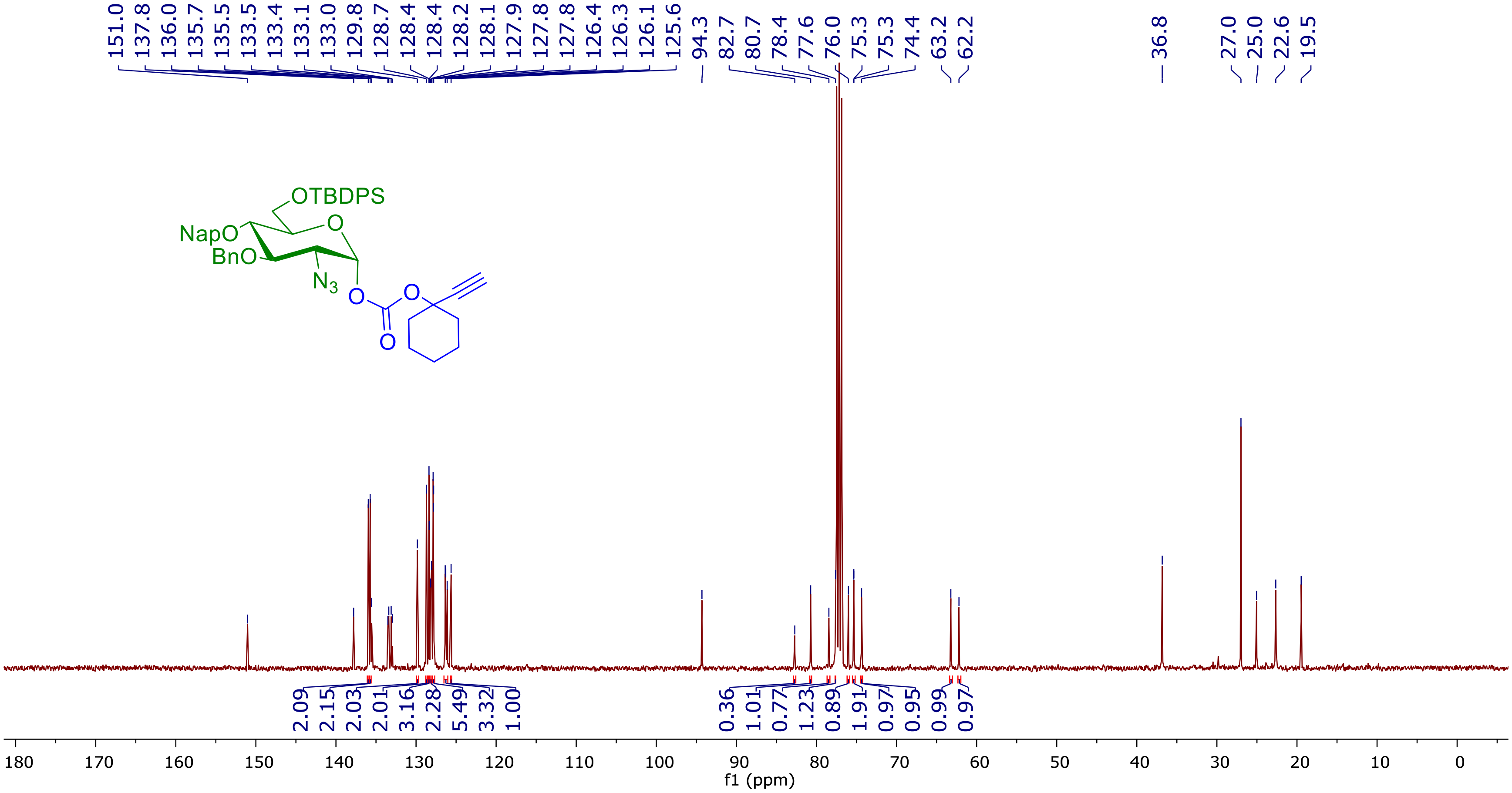

Supplementary Figure S55c. DEPT NMR Spectrum (100.67 MHz, $CDCl_3$) of Compound 33a (α isomer)

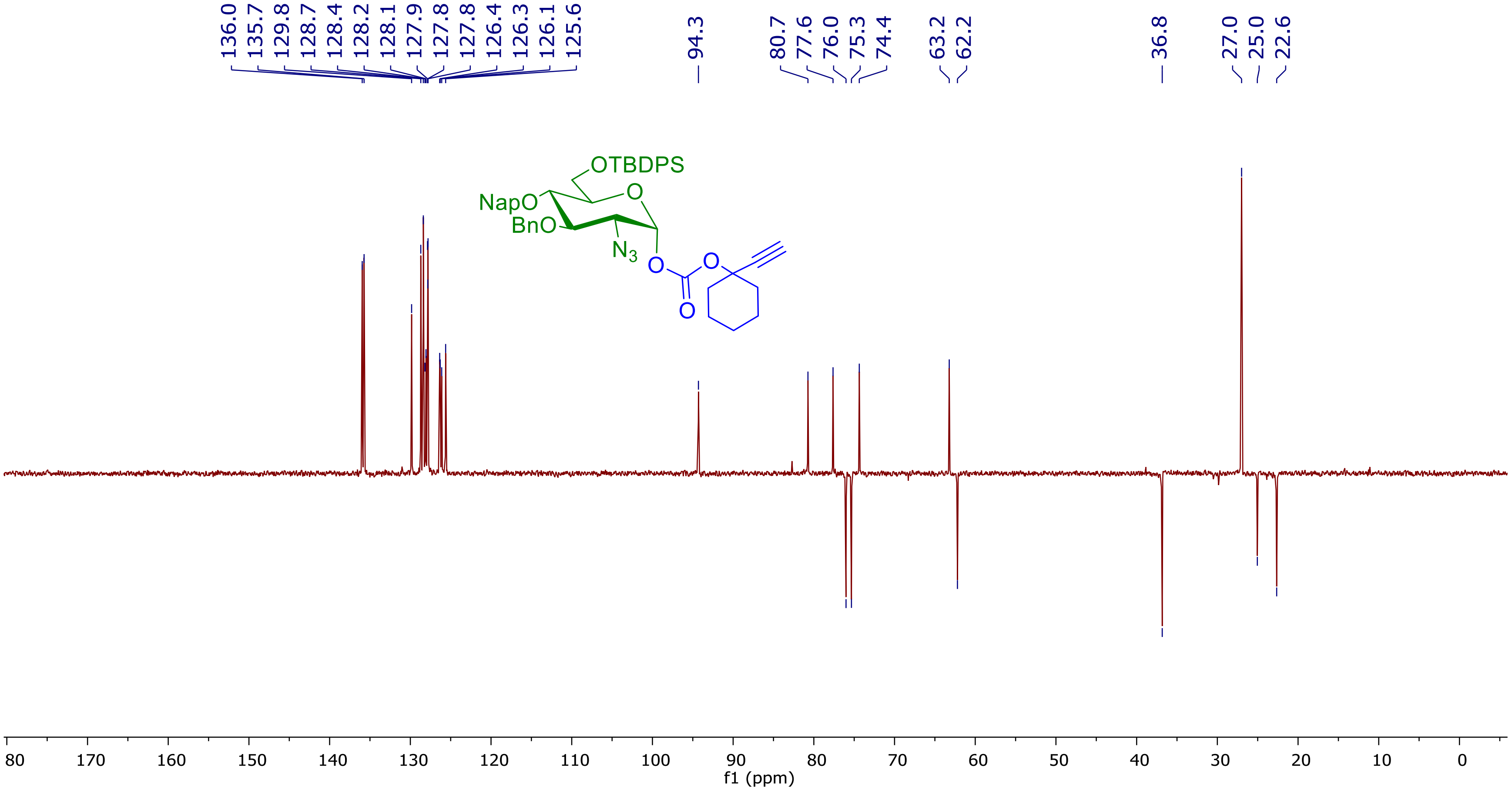

Supplementary Figure S56a. ¹H NMR Spectrum (400.31 MHz, CDCl₃) of Compound 33a (β isomer)

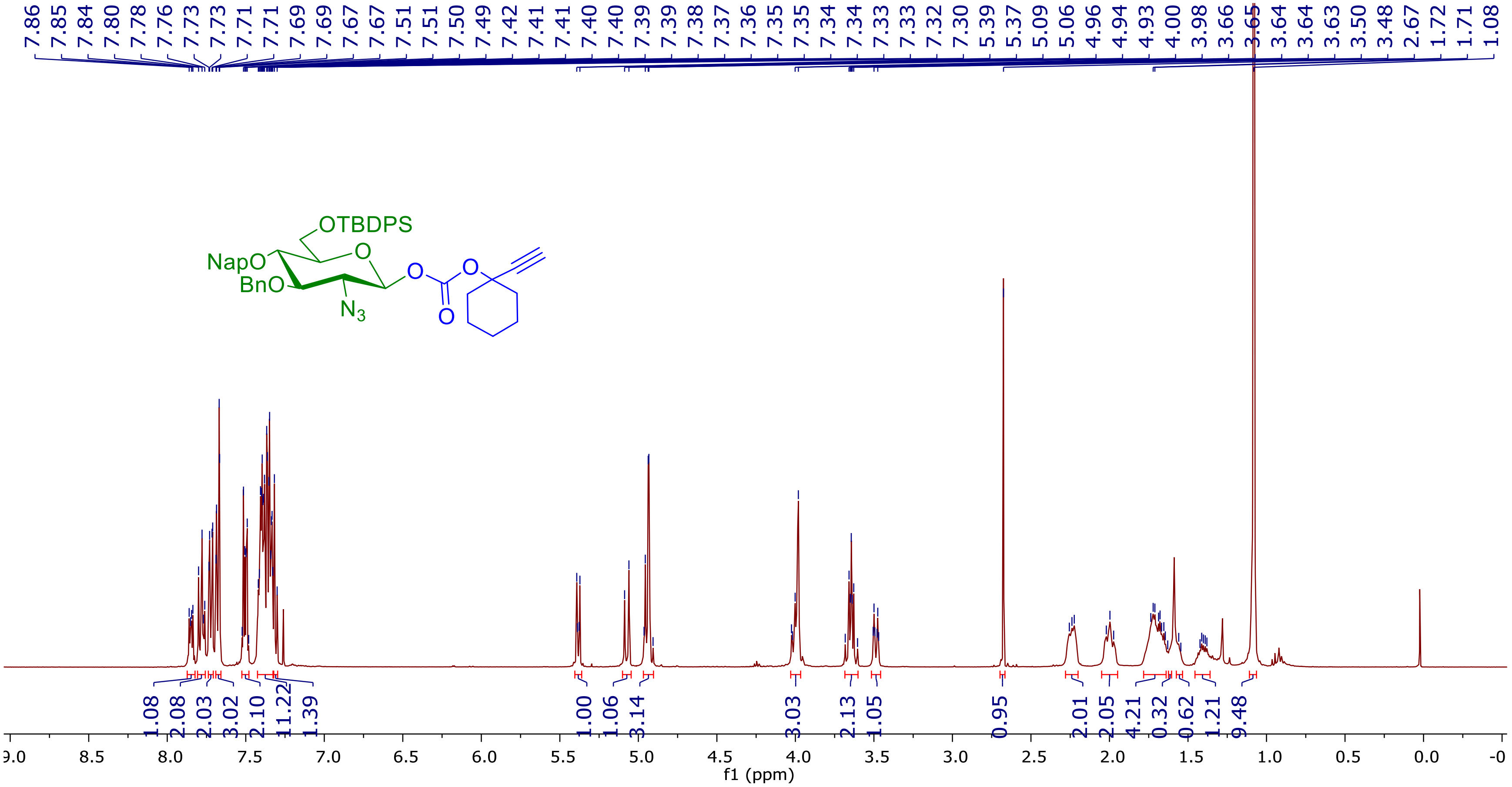

Supplementary Figure S56b. ^{13}C NMR Spectrum (100.67 MHz, $CDCl_3$) of Compound 33a (β isomer)

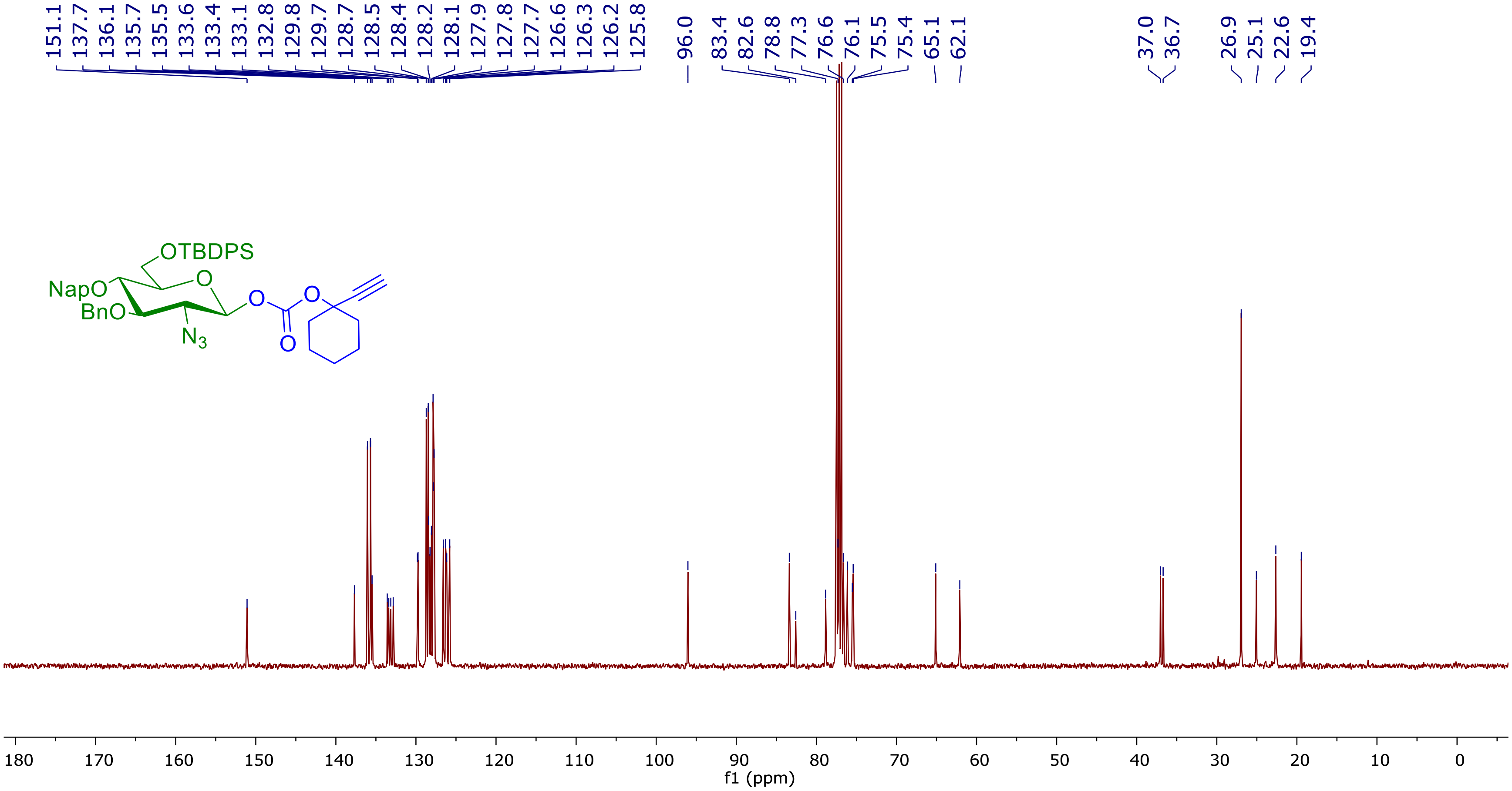

Supplementary Figure S56c. DEPT NMR Spectrum (100.67 MHz, $CDCl_3$) of Compound 33a (β isomer)

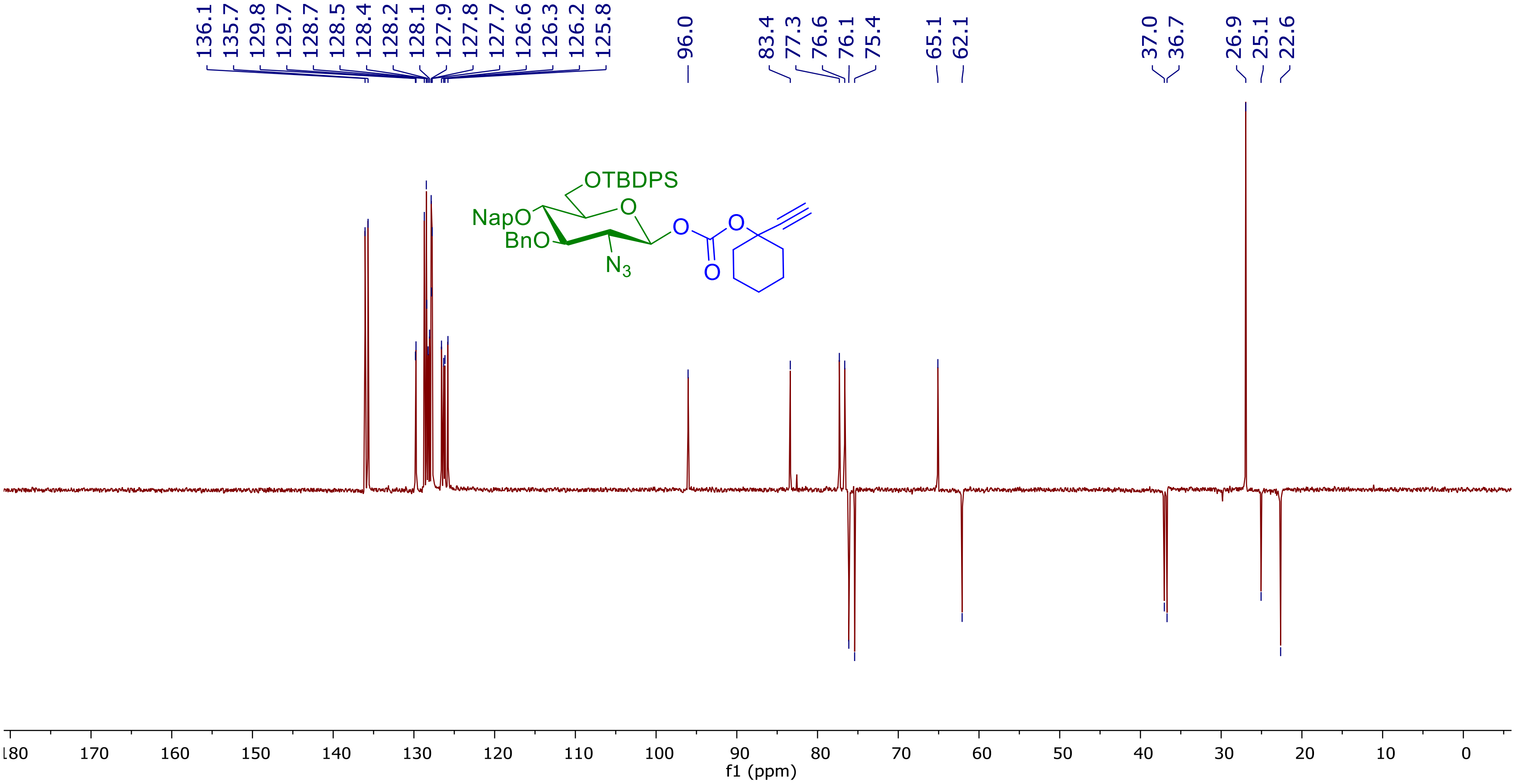

Supplementary Figure S57a. ^{1}H NMR Spectrum (400.31 MHz, $CDCl_3$) of Compound 33b

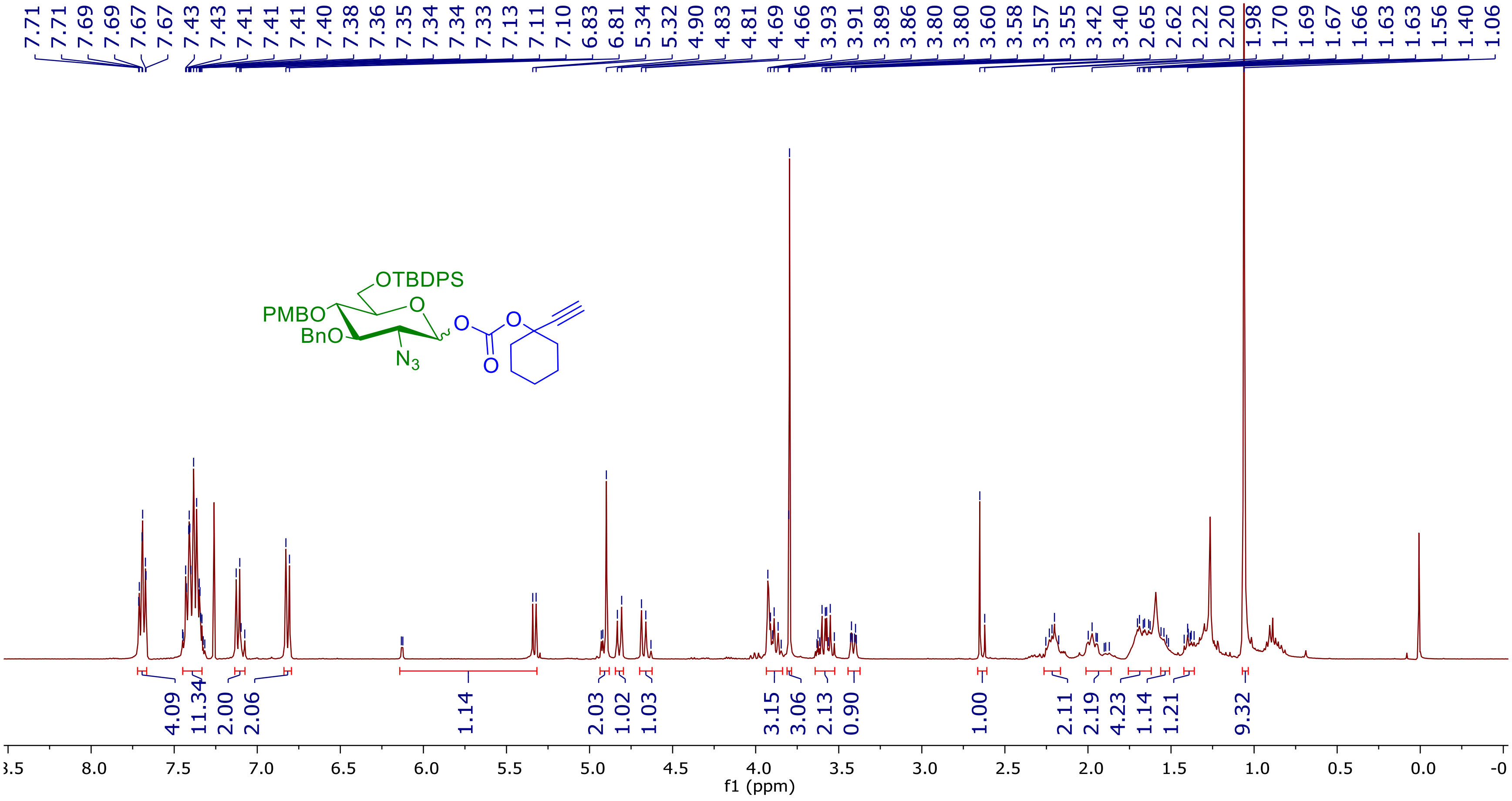

Supplementary Figure S57b. ^{13}C NMR Spectrum (100.67 MHz, $CDCl_3$) of Compound 33b

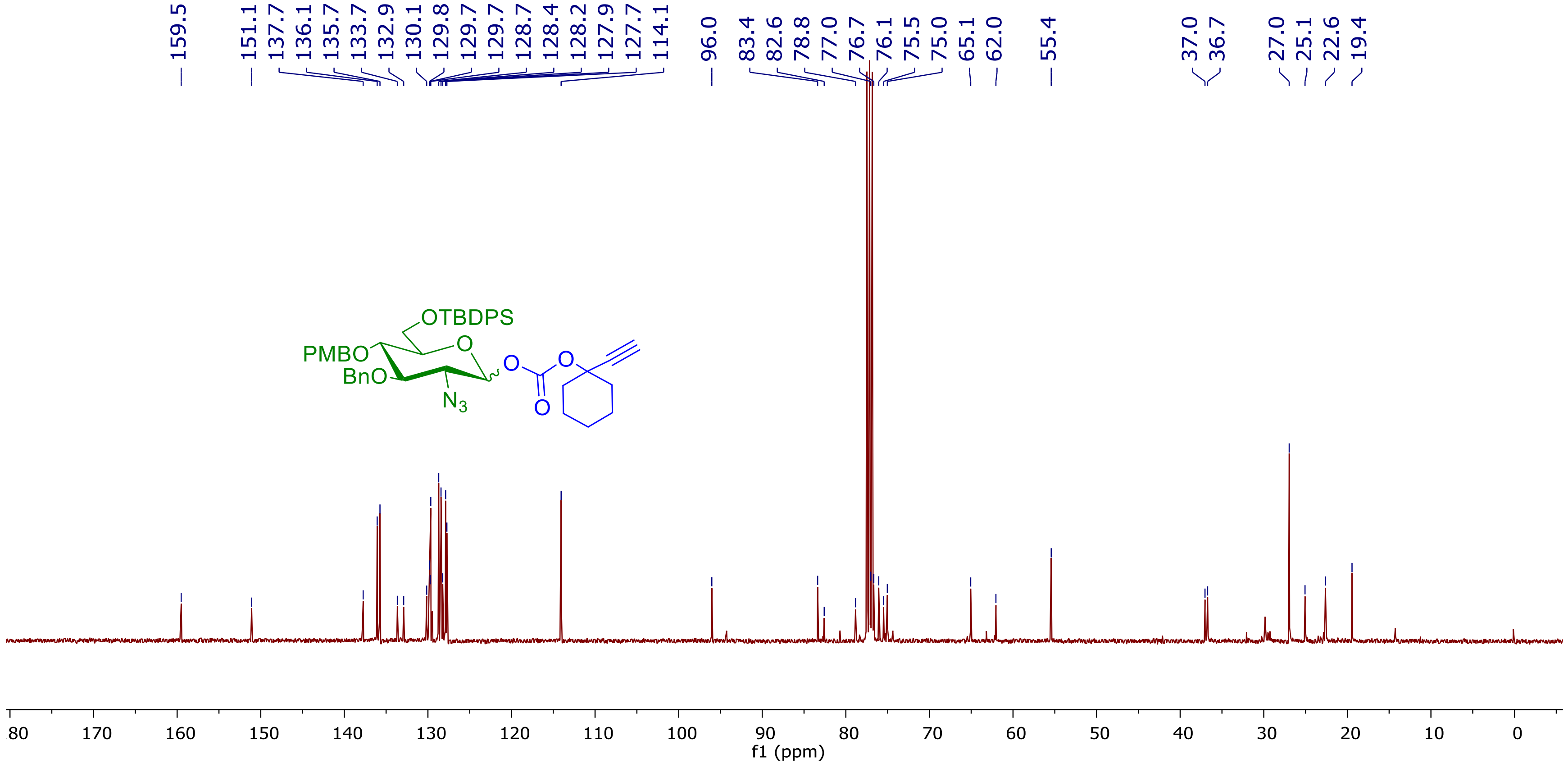

Supplementary Figure S57c. DEPT NMR Spectrum (100.67 MHz, $CDCl_3$) of Compound 33b

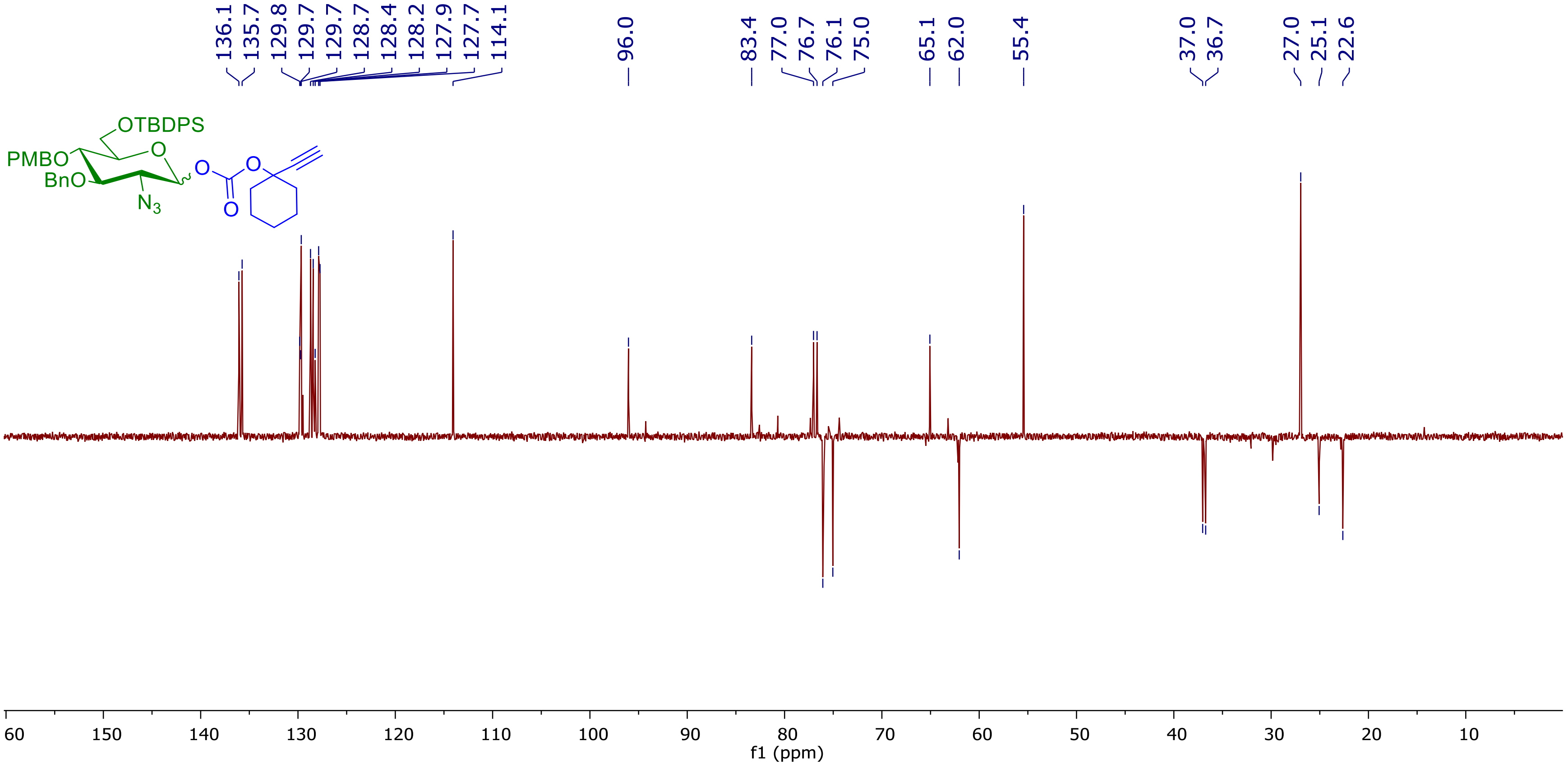

Supplementary Figure S58a. ^{1}H NMR Spectrum (400.31 MHz, $CDCl_3$) of Compound 33c

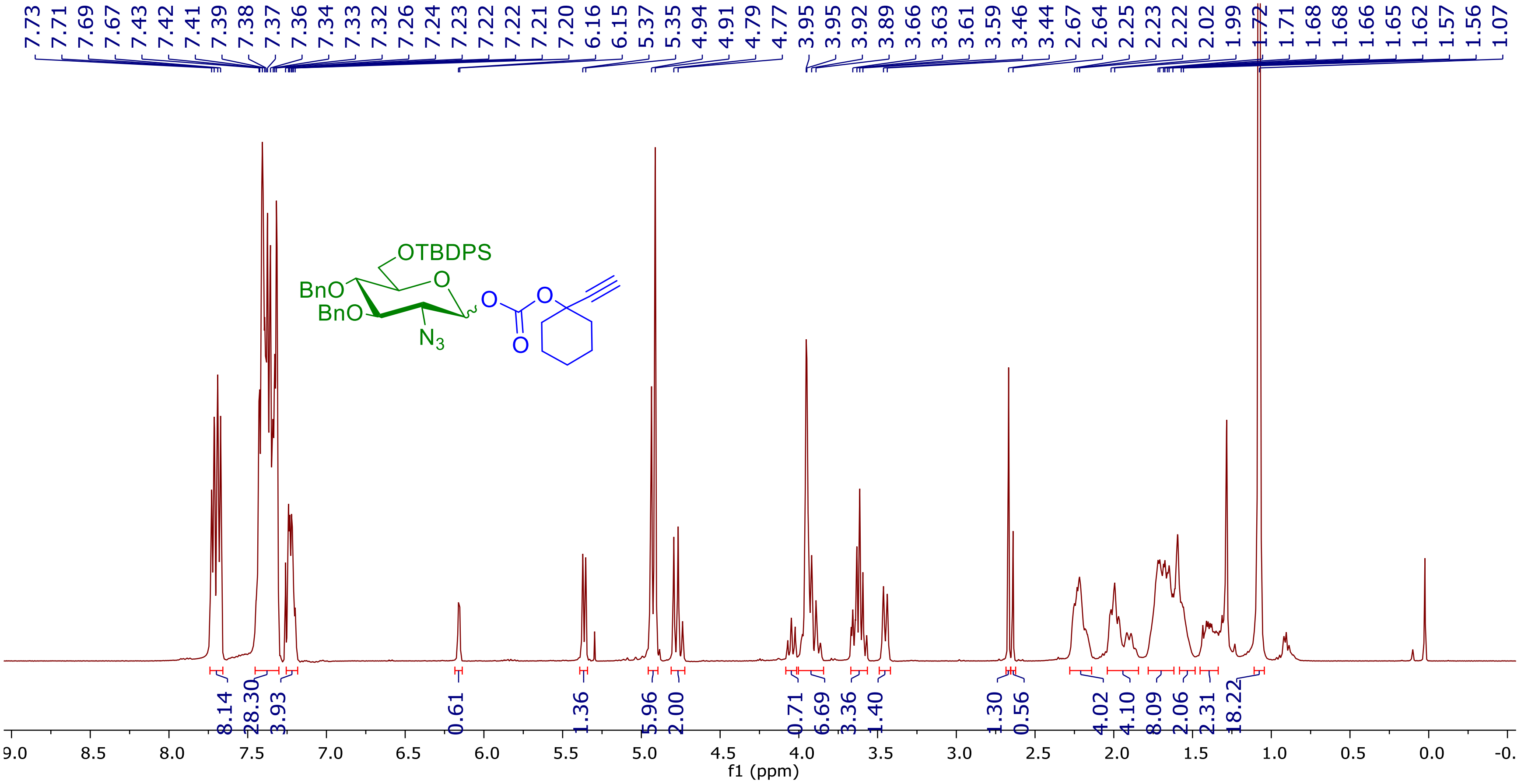

Supplementary Figure S58b. ^{13}C NMR Spectrum (100.67 MHz, $CDCl_3$) of Compound 33c

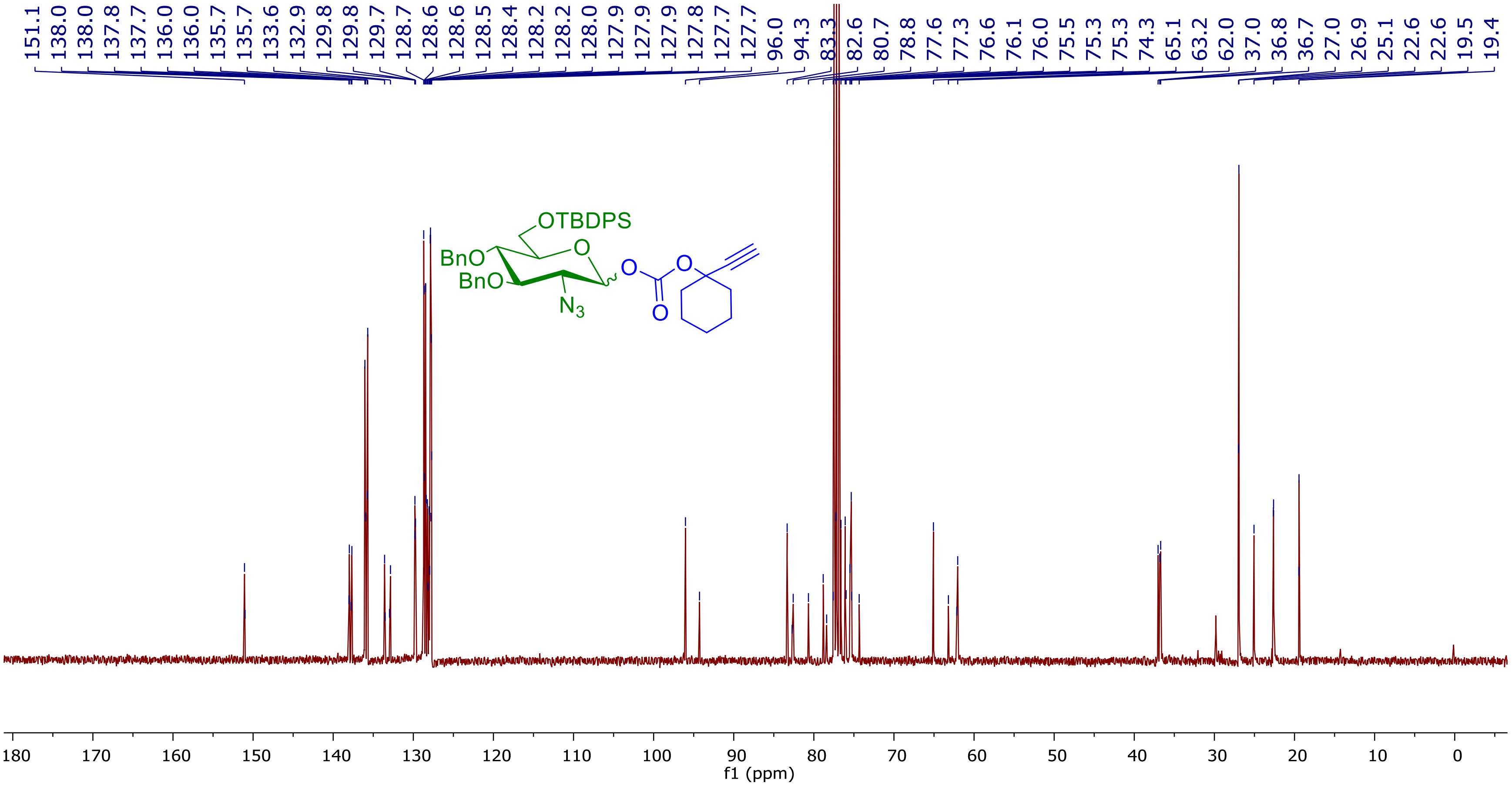

Supplementary Figure S58c. DEPT NMR Spectrum (100.67 MHz, $CDCl_3$) of Compound 33c

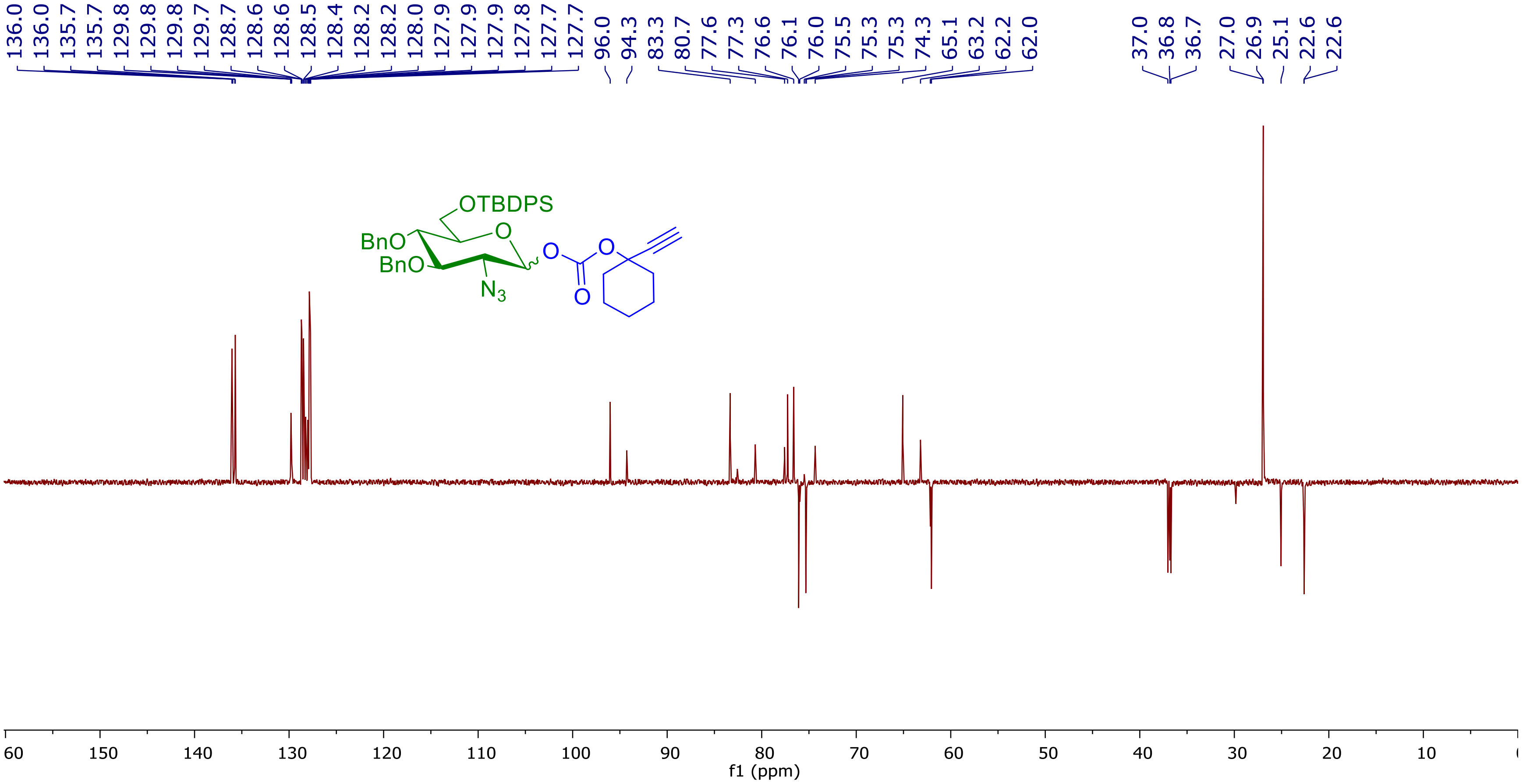

Supplementary Figure S59a. ¹H NMR Spectrum (399.78 MHz, $CDCl_3$) of Compound 33d

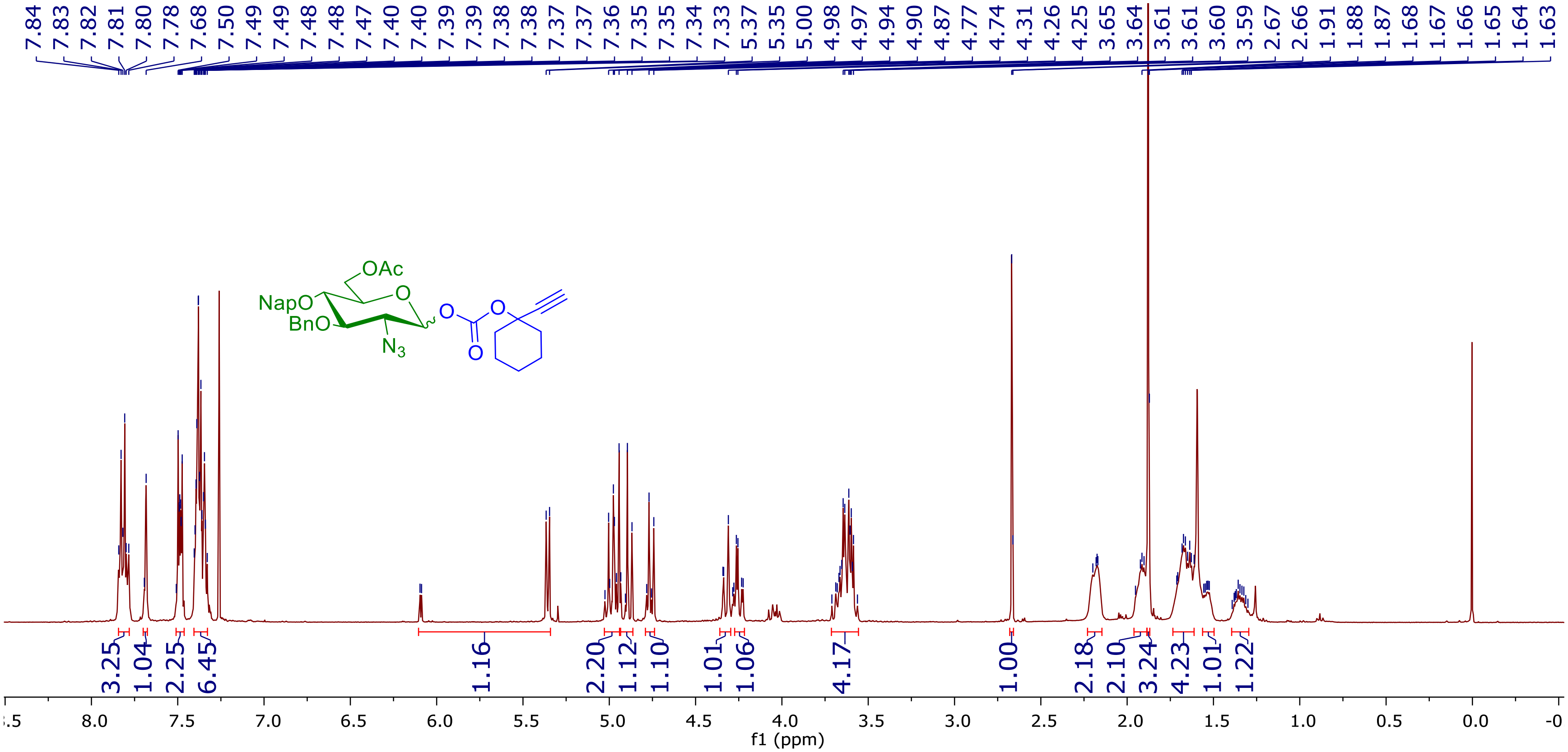

Supplementary Figure S59b. ^{13}C NMR Spectrum (100.53 MHz, $CDCl_3$) of Compound 33d

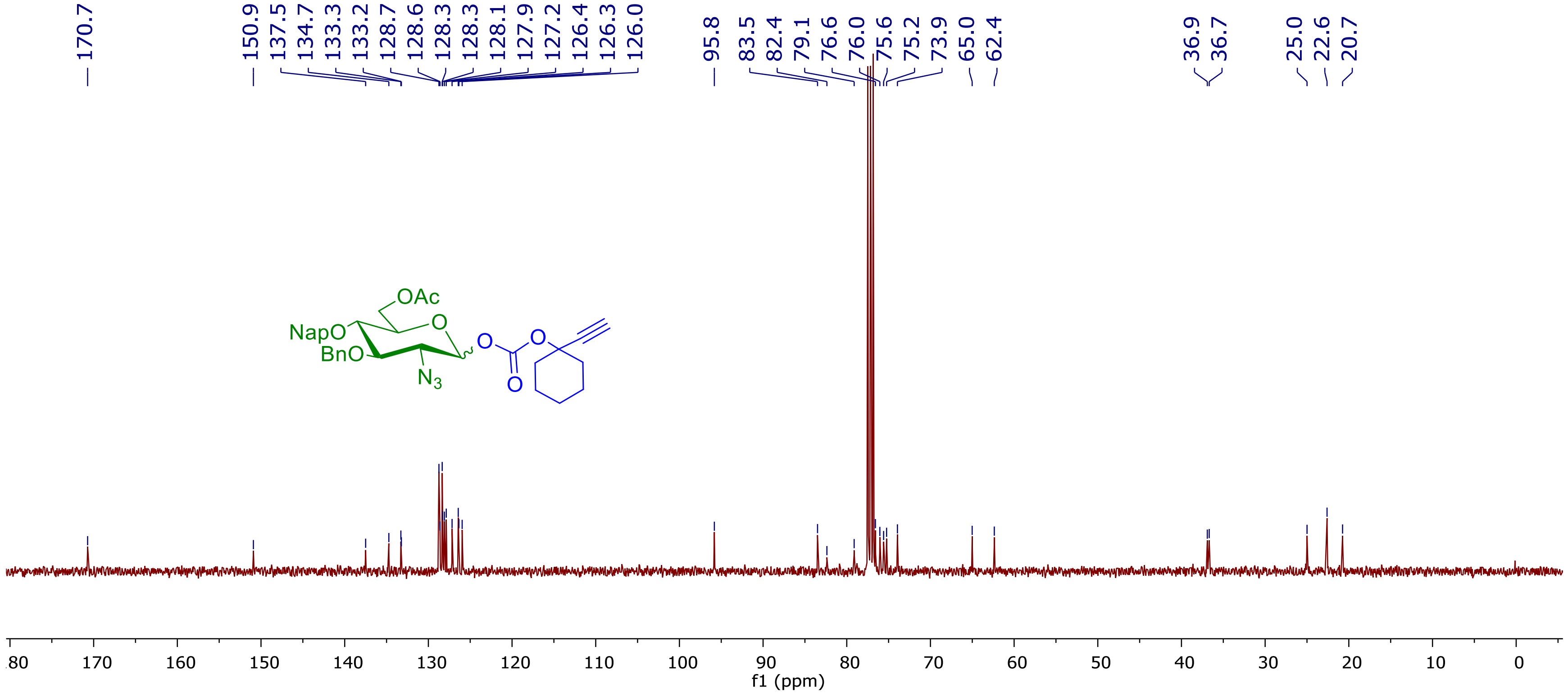

Supplementary Figure S59c. DEPT NMR Spectrum (100.53 MHz, $CDCl_3$) of Compound 33d

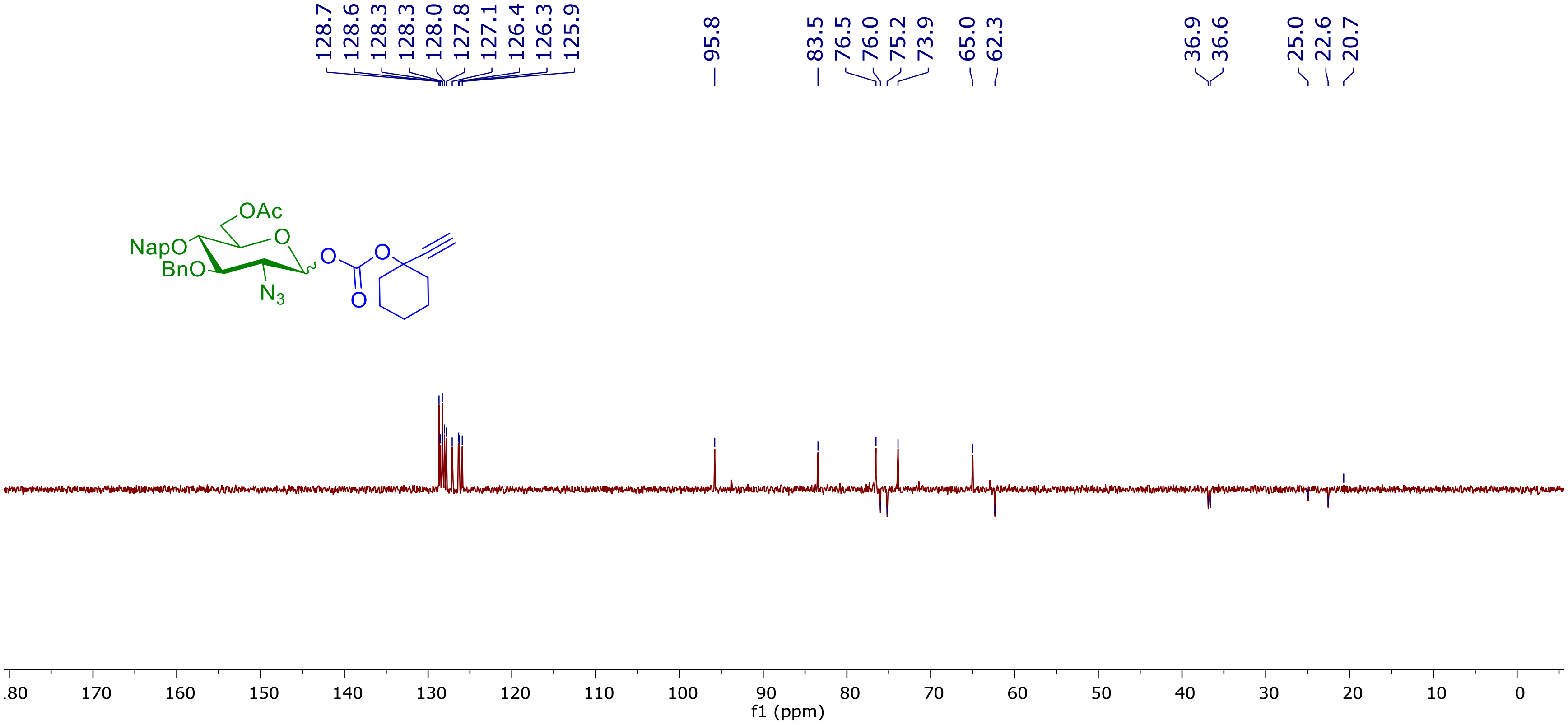

Supplementary Figure S60a. ^{1}H NMR Spectrum (399.78 MHz, $CDCl_3$) of Compound 33e

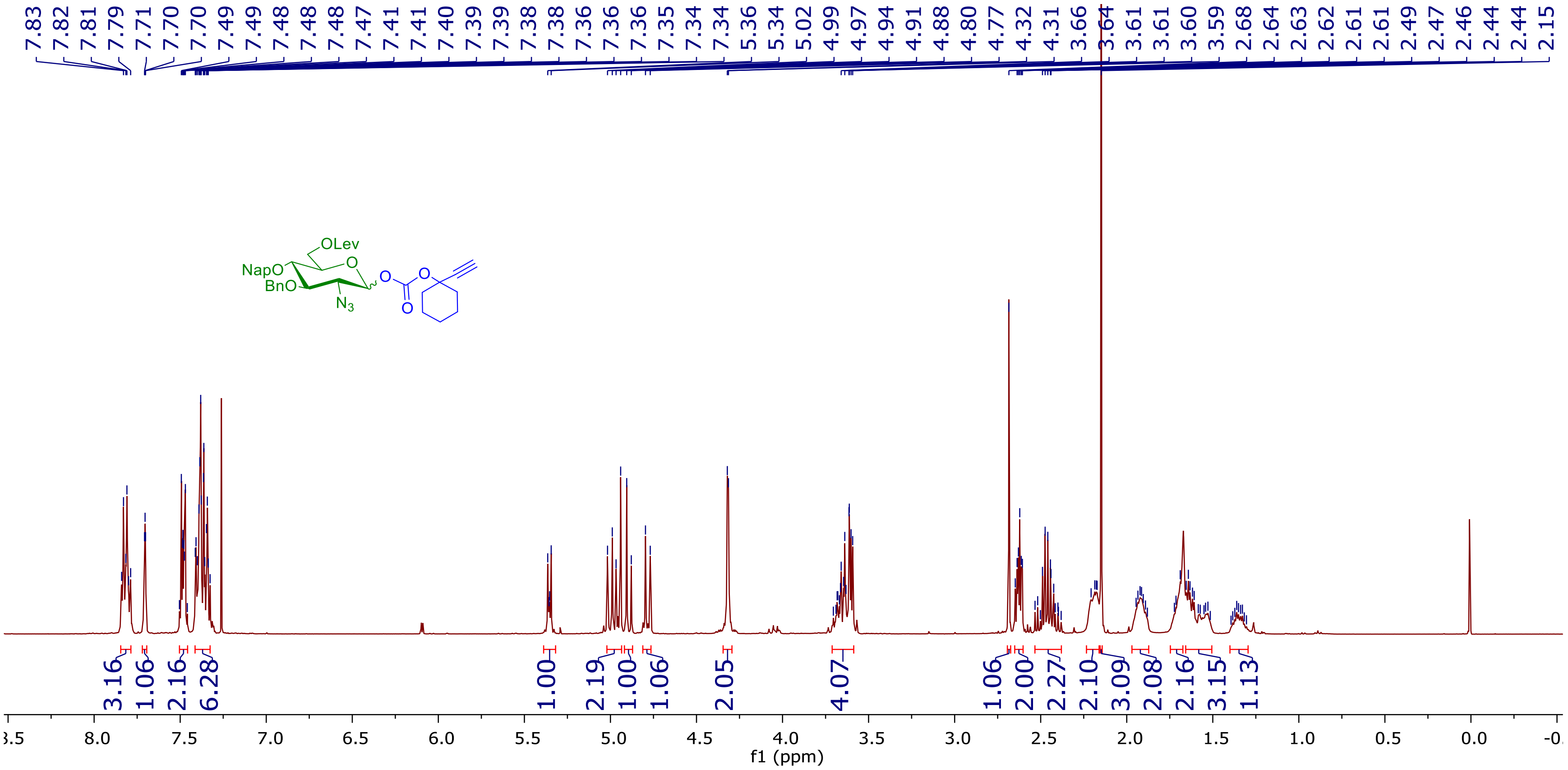

Supplementary Figure S60b. ^{13}C NMR Spectrum (100.53 MHz, $CDCl_3$) of Compound 33e

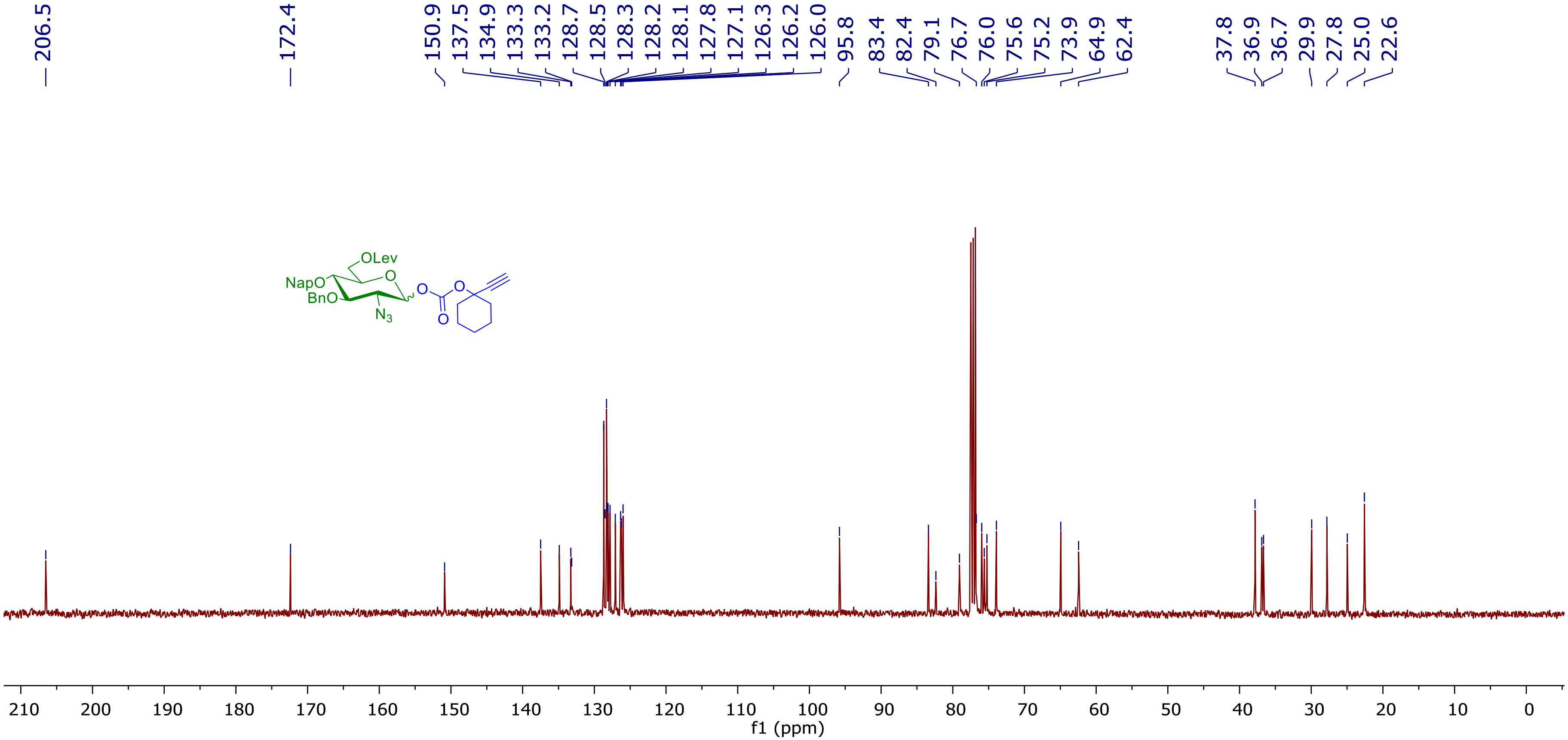

Supplementary Figure S60c. DEPT NMR Spectrum (100.67 MHz, $CDCl_3$) of Compound 33e

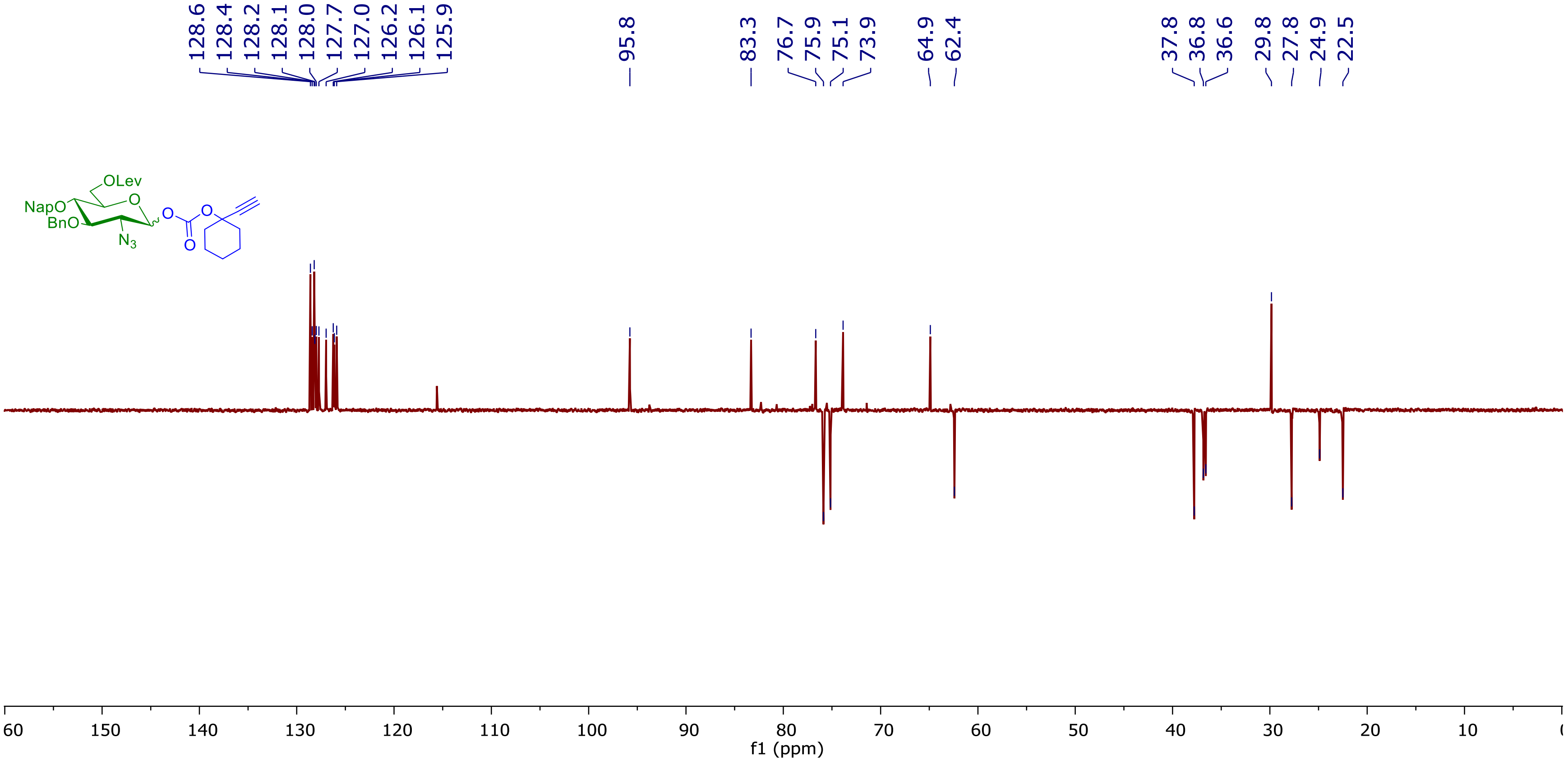

Supplementary Figure S61a. ^{1}H NMR Spectrum (400.31 MHz, $CDCl_3$) of Compound S10

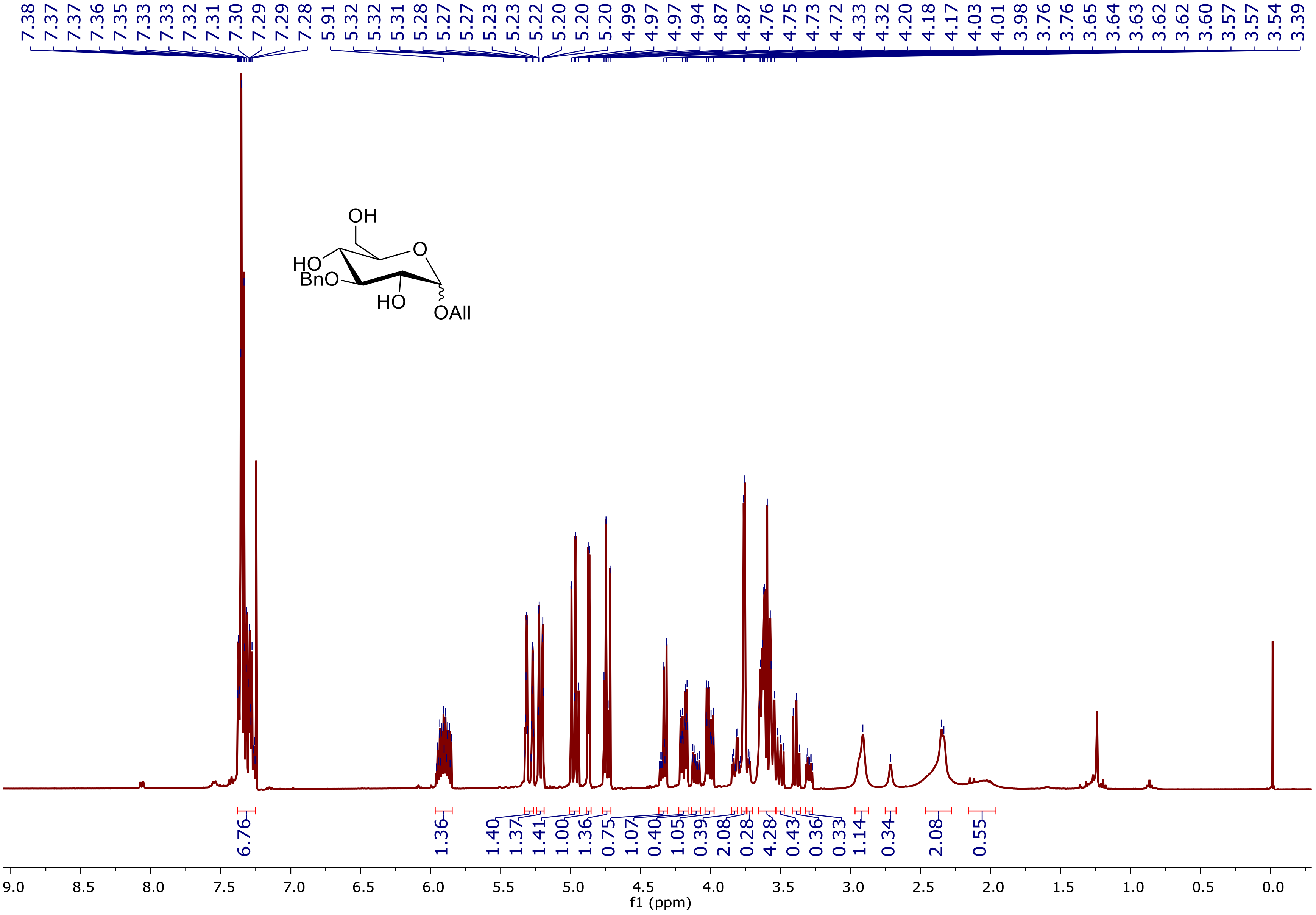

Supplementary Figure S61b. ^{13}C NMR Spectrum (100.67 MHz, $CDCl_3$) of Compound S10

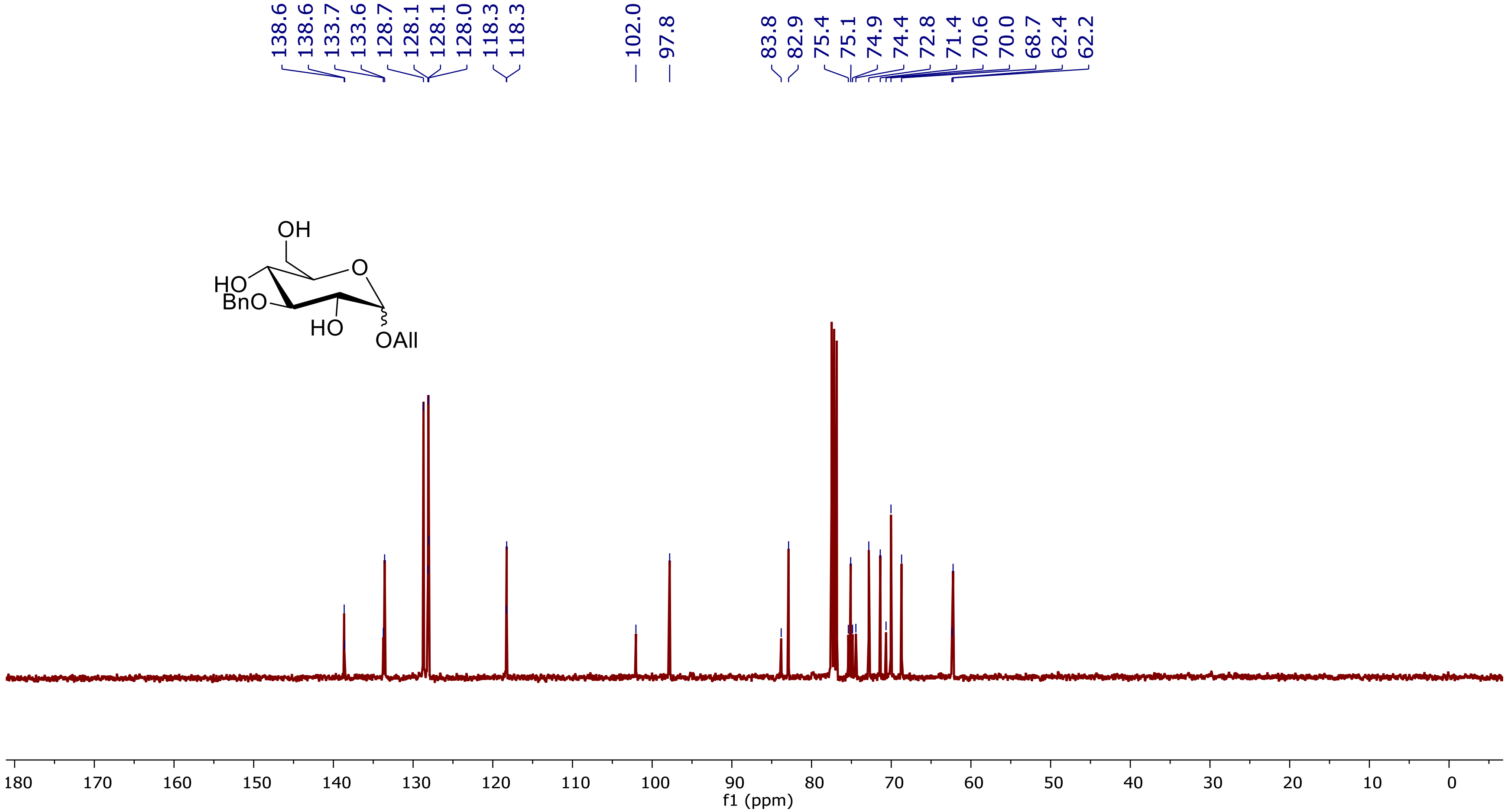

Supplementary Figure S61c. DEPT NMR Spectrum (100.67 MHz, $CDCl_3$) of Compound S10

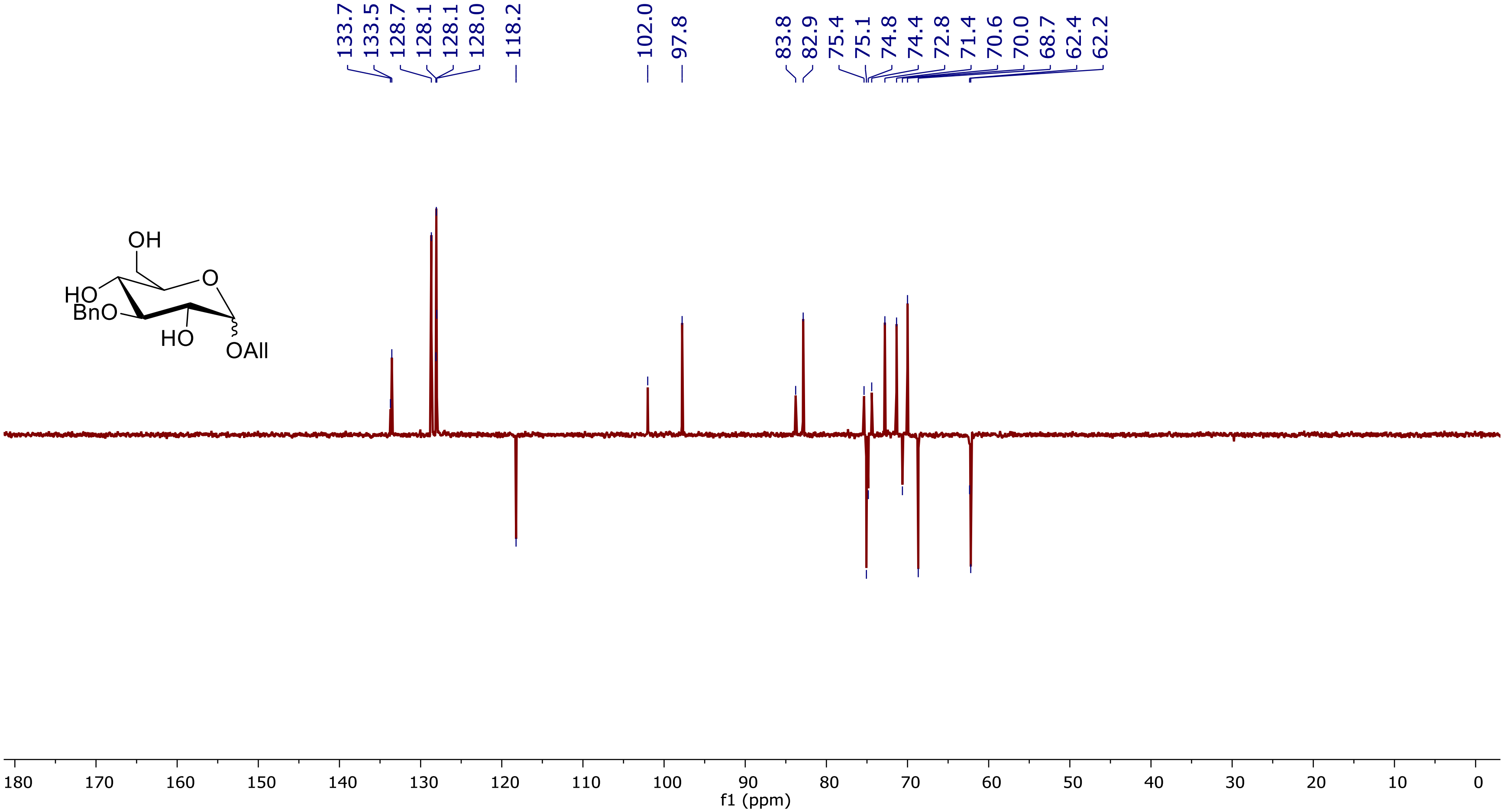

Supplementary Figure S62a. ^{1}H NMR Spectrum (400.31 MHz, $CDCl_3$) of Compound S11

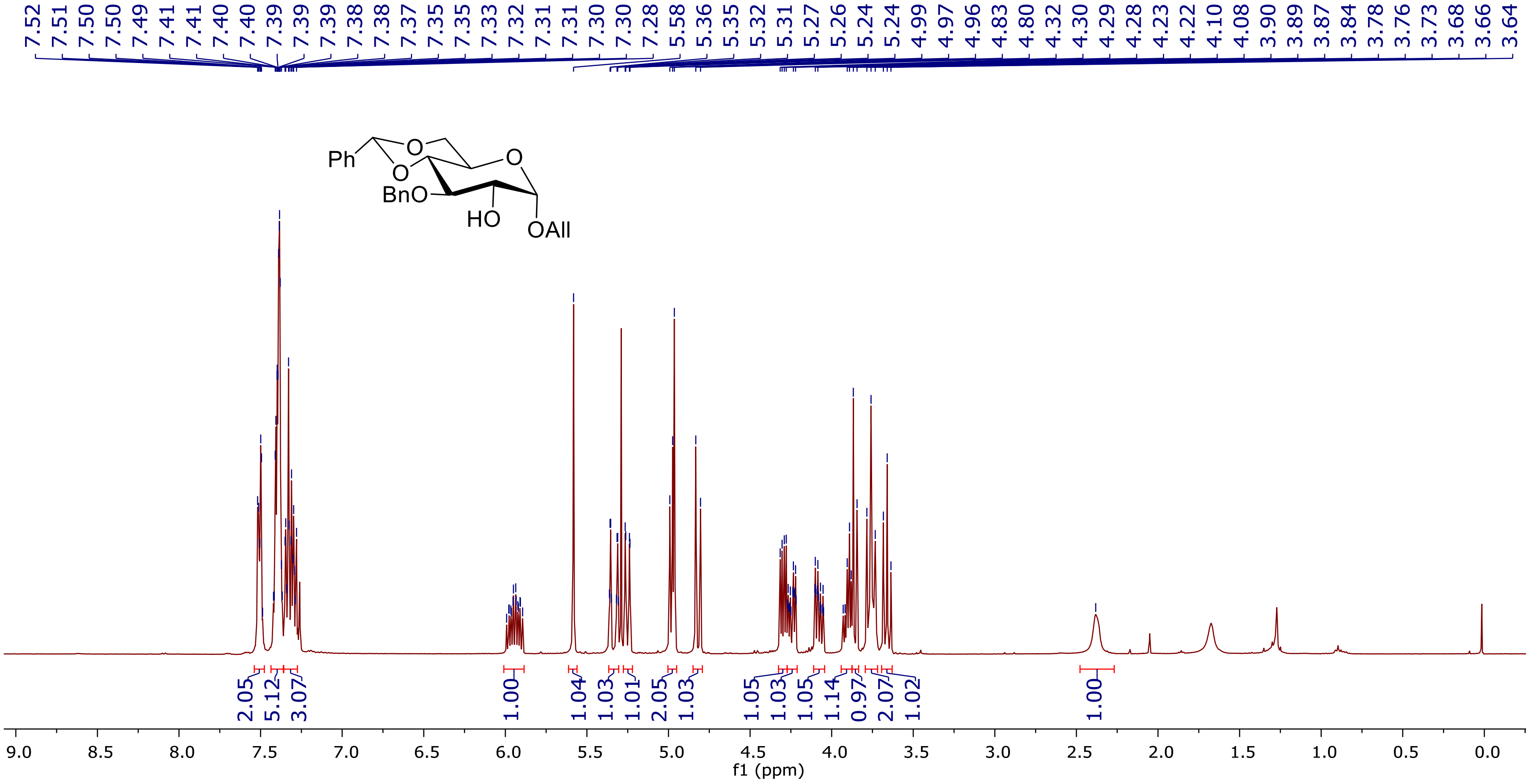

Supplementary Figure S62b. ^{13}C NMR Spectrum (100.67 MHz, $CDCl_3$) of Compound S11

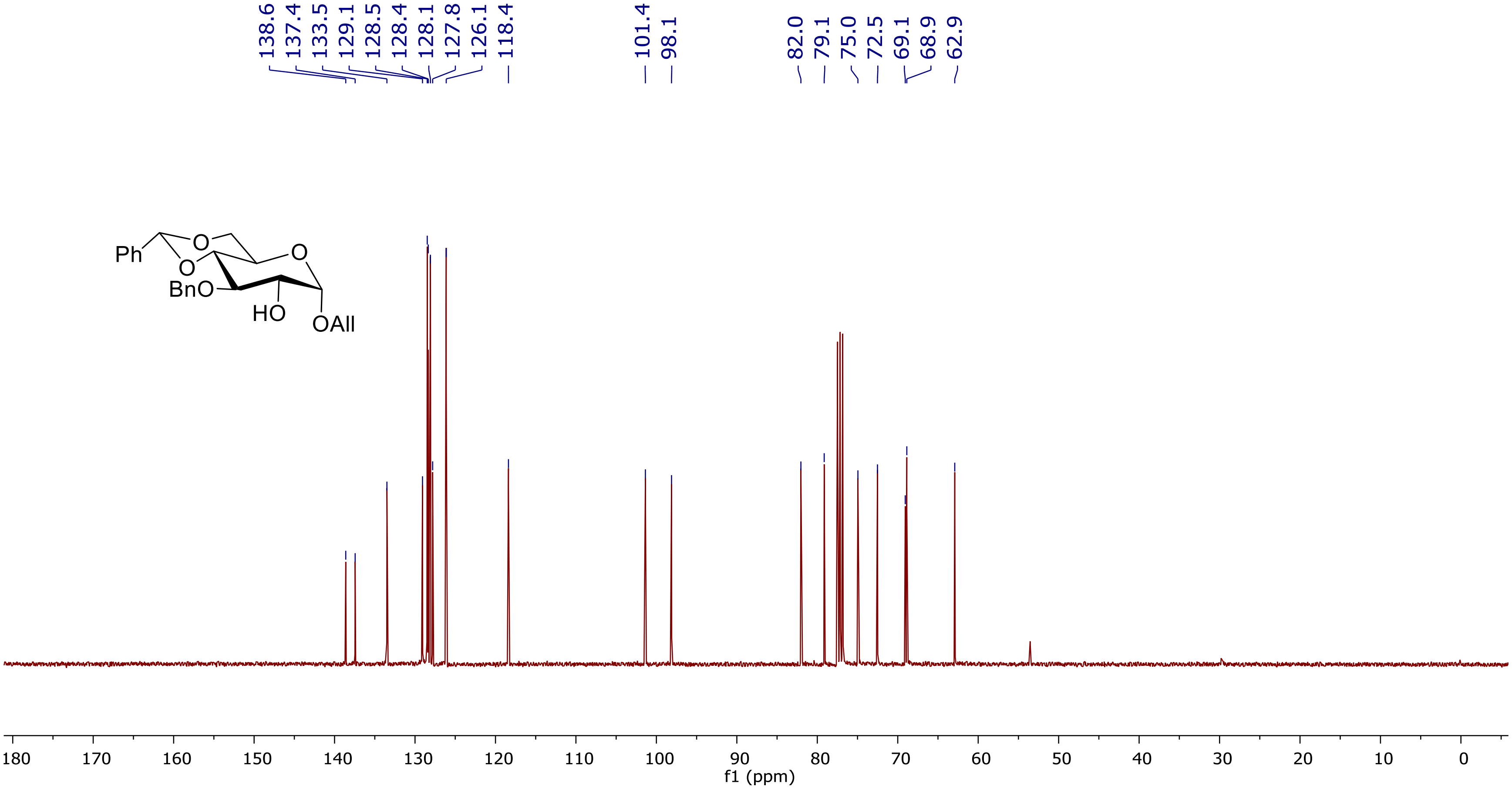

Supplementary Figure S62c. DEPT NMR Spectrum (100.67 MHz, $CDCl_3$) of Compound S11

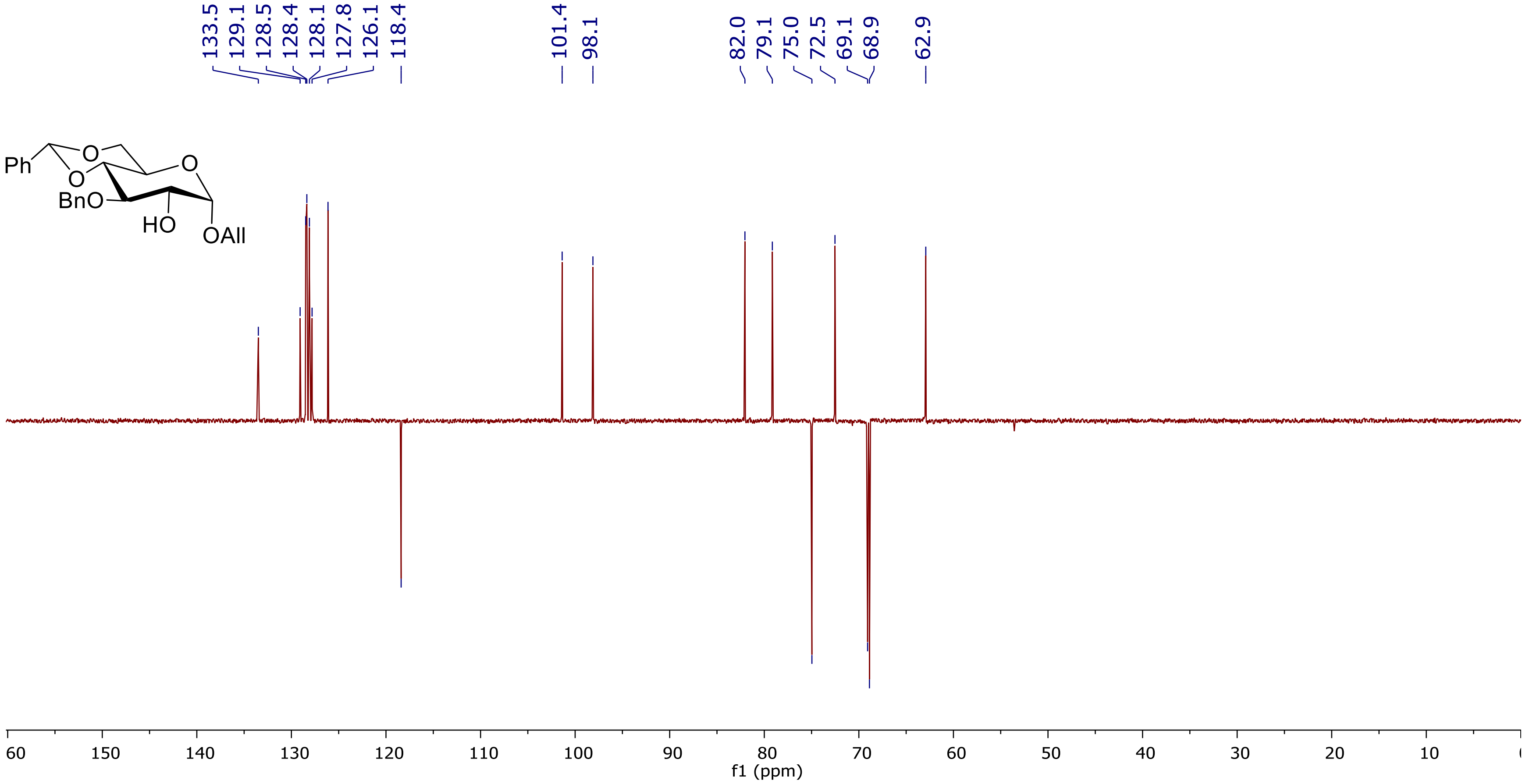

Supplementary Figure S63a. ^{1}H NMR Spectrum (400.31 MHz, $CDCl_3$) of Compound 35a

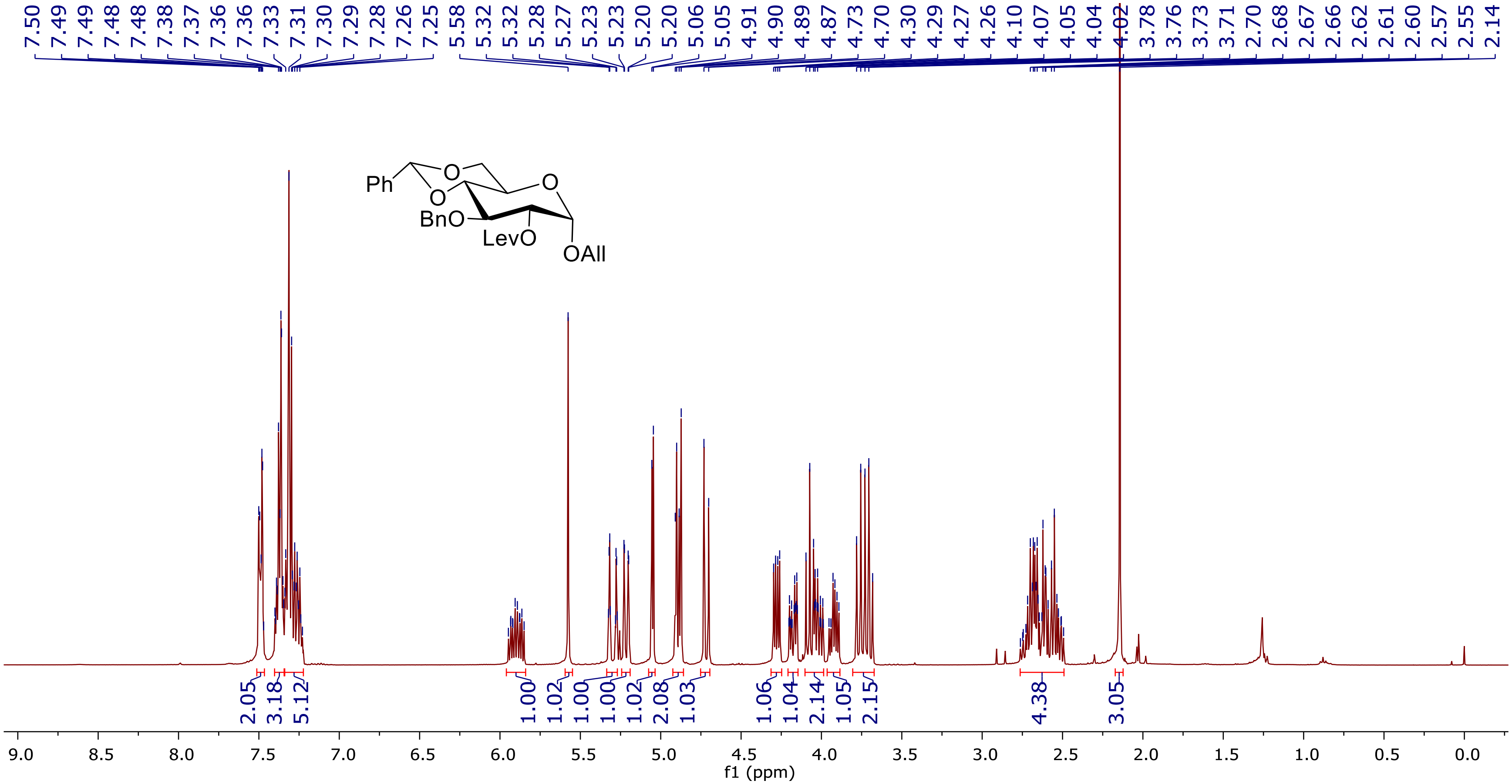

Supplementary Figure S63b. ^{13}C NMR Spectrum (100.67 MHz, $CDCl_3$) of Compound 35a

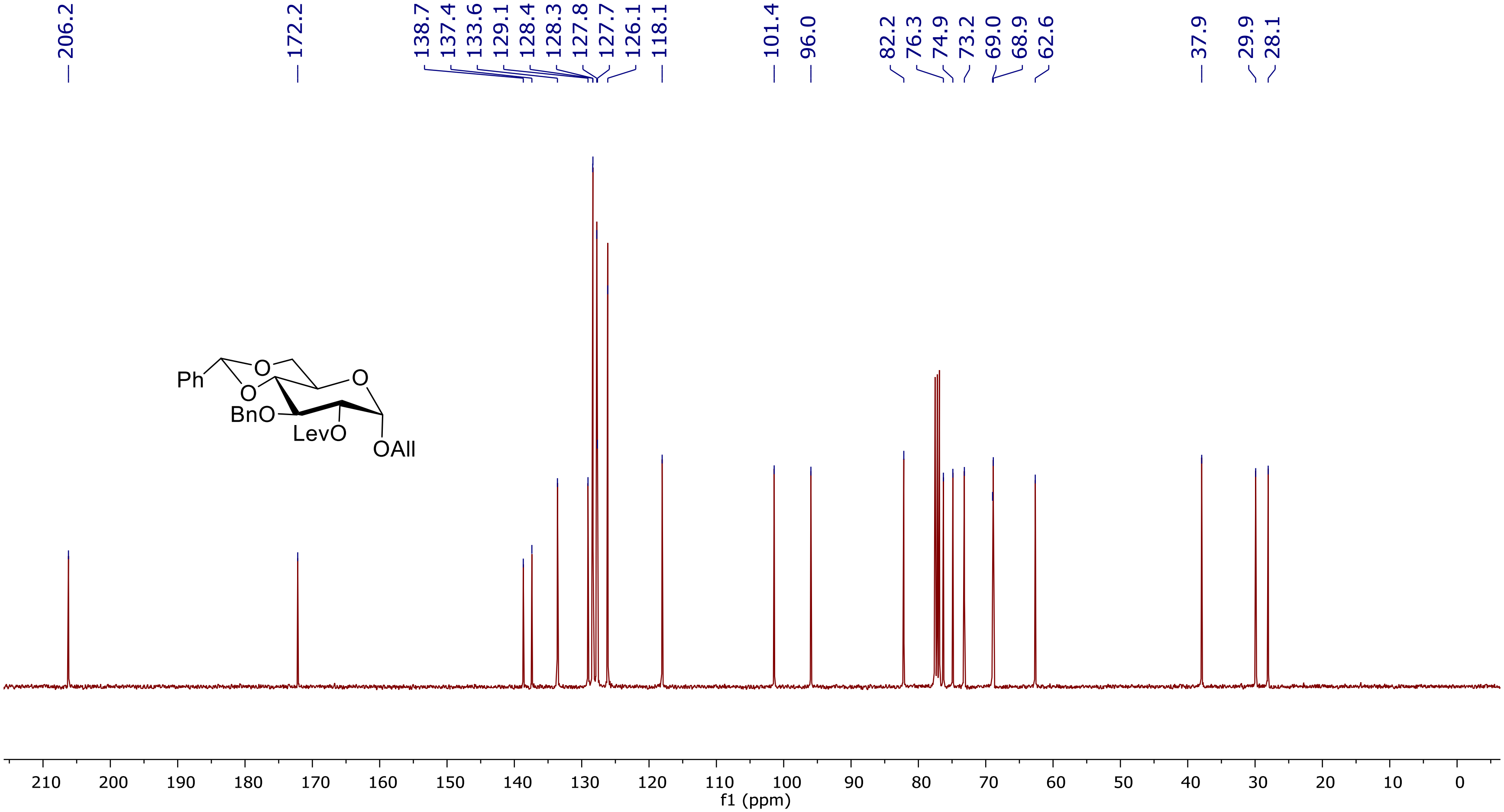

Supplementary Figure S63c. DEPT NMR Spectrum (100.67 MHz, $CDCl_3$) of Compound 35a

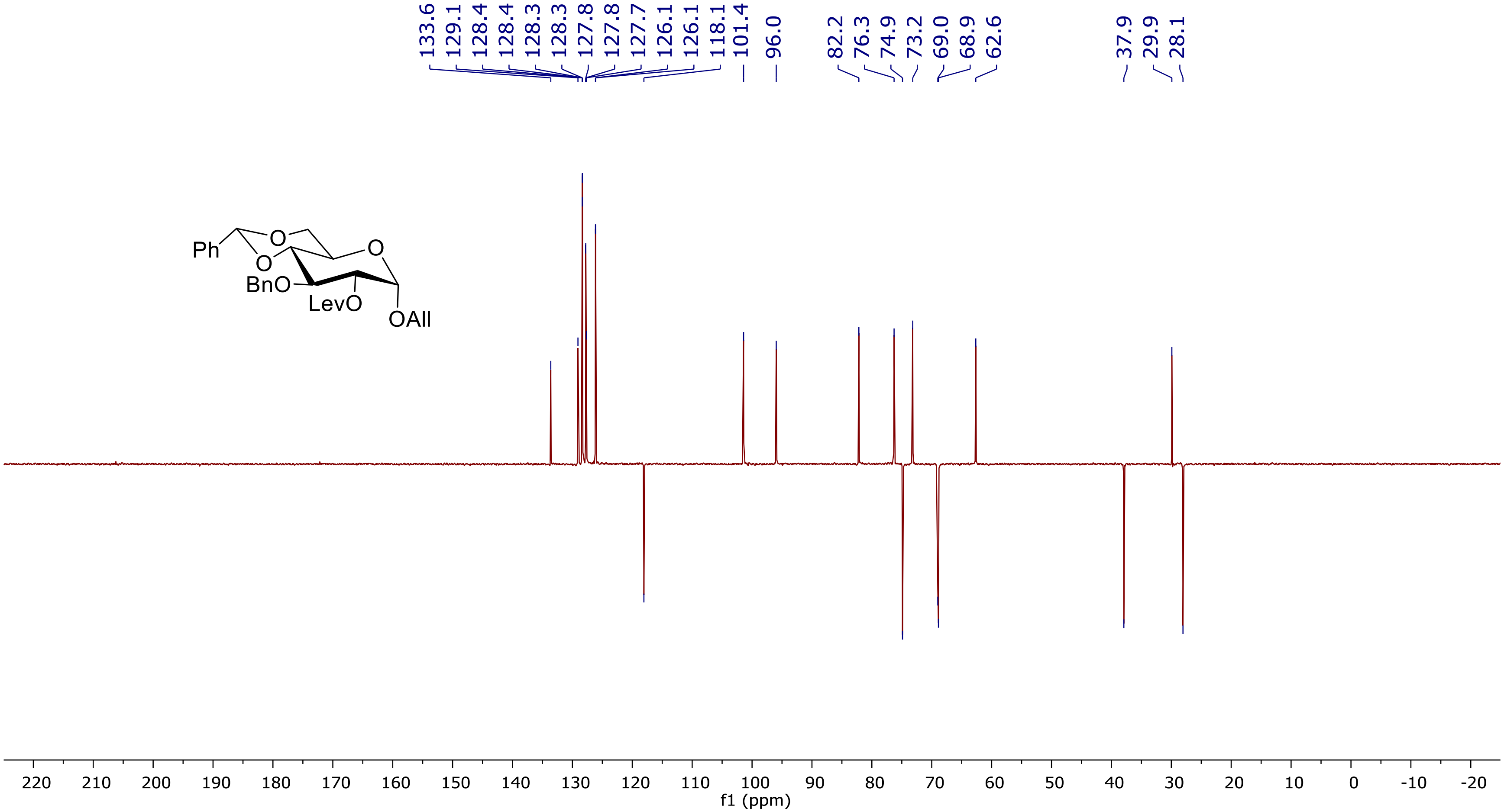

Supplementary Figure S64a. ^{1}H NMR Spectrum (400.31 MHz, $CDCl_3$) of Compound 35c

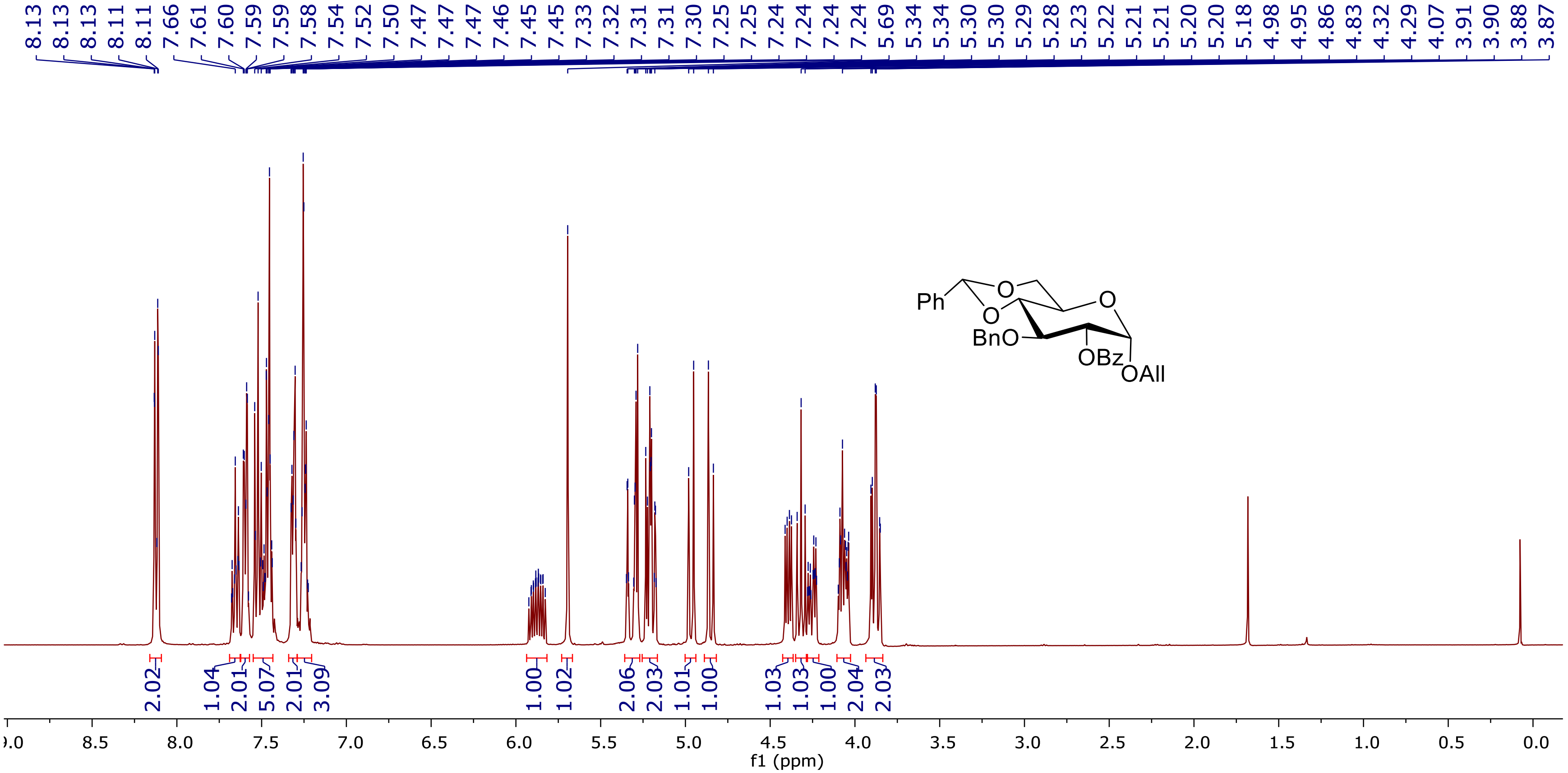

Supplementary Figure S64b. ^{13}C NMR Spectrum (100.67 MHz, $CDCl_3$) of Compound 35c

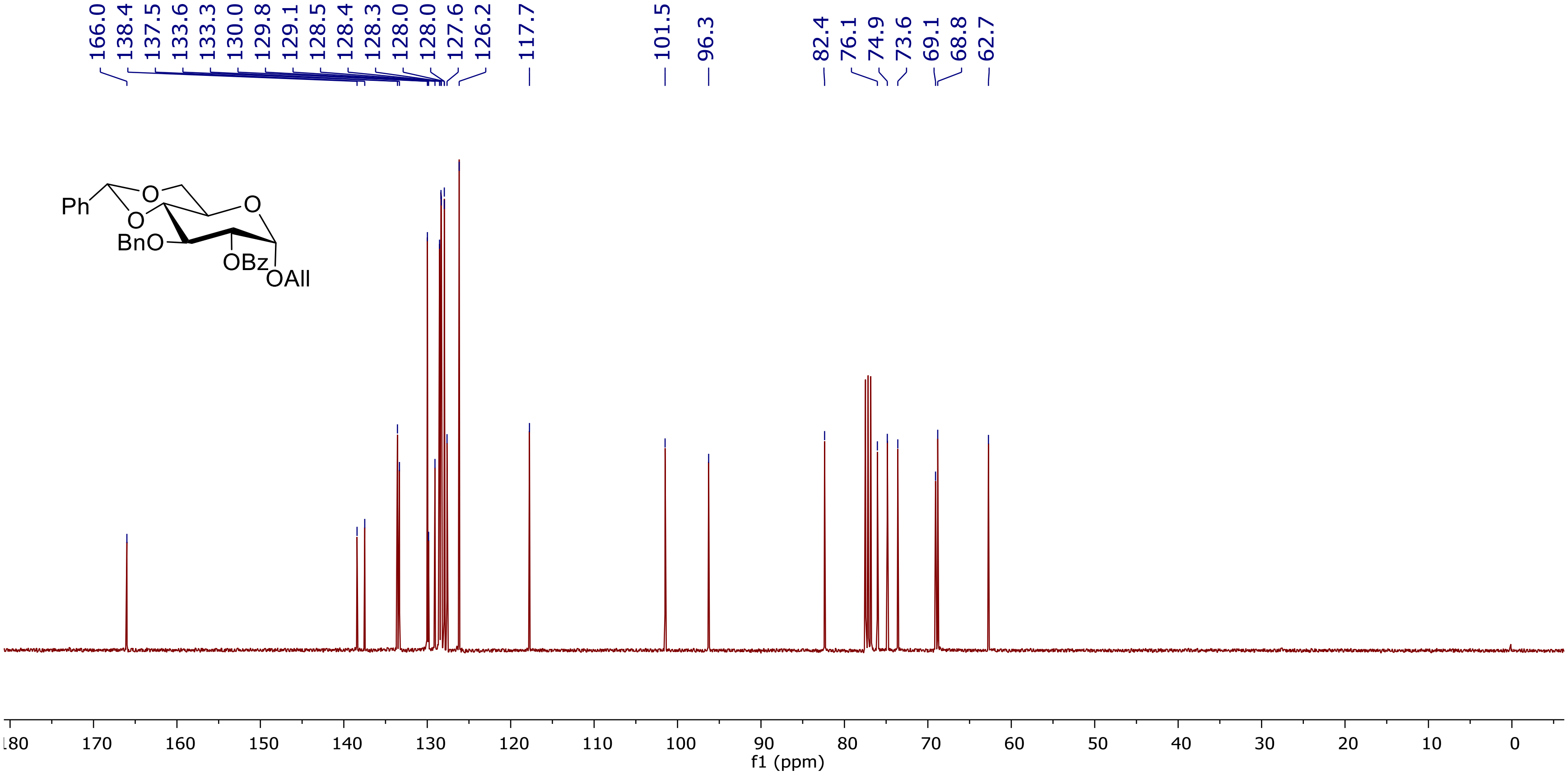

Supplementary Figure S64c. DEPT NMR Spectrum (100.67 MHz, $CDCl_3$) of Compound 35c

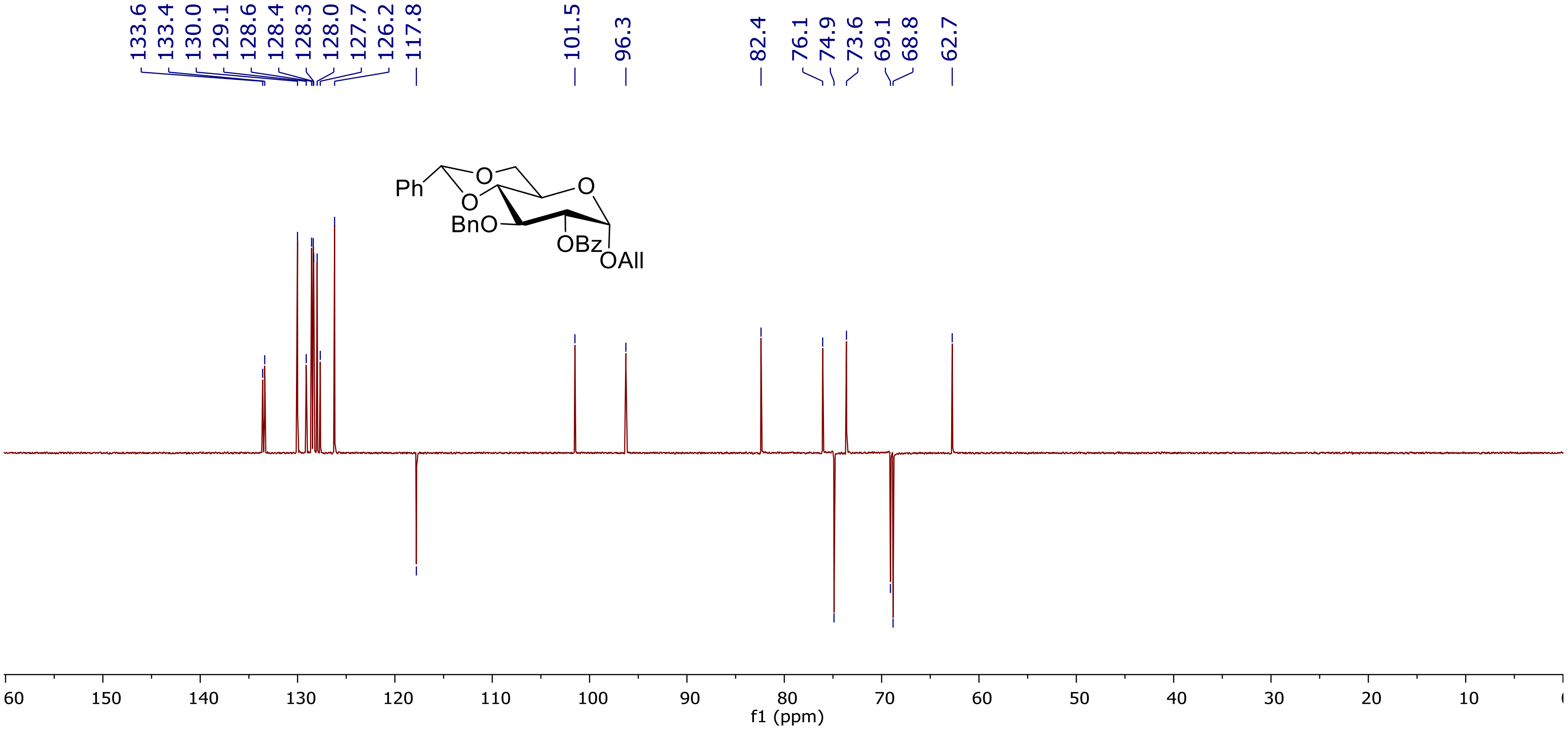

Supplementary Figure S65a. ^{1}H NMR Spectrum (400.31 MHz, $CDCl_3$) of Compound S12a

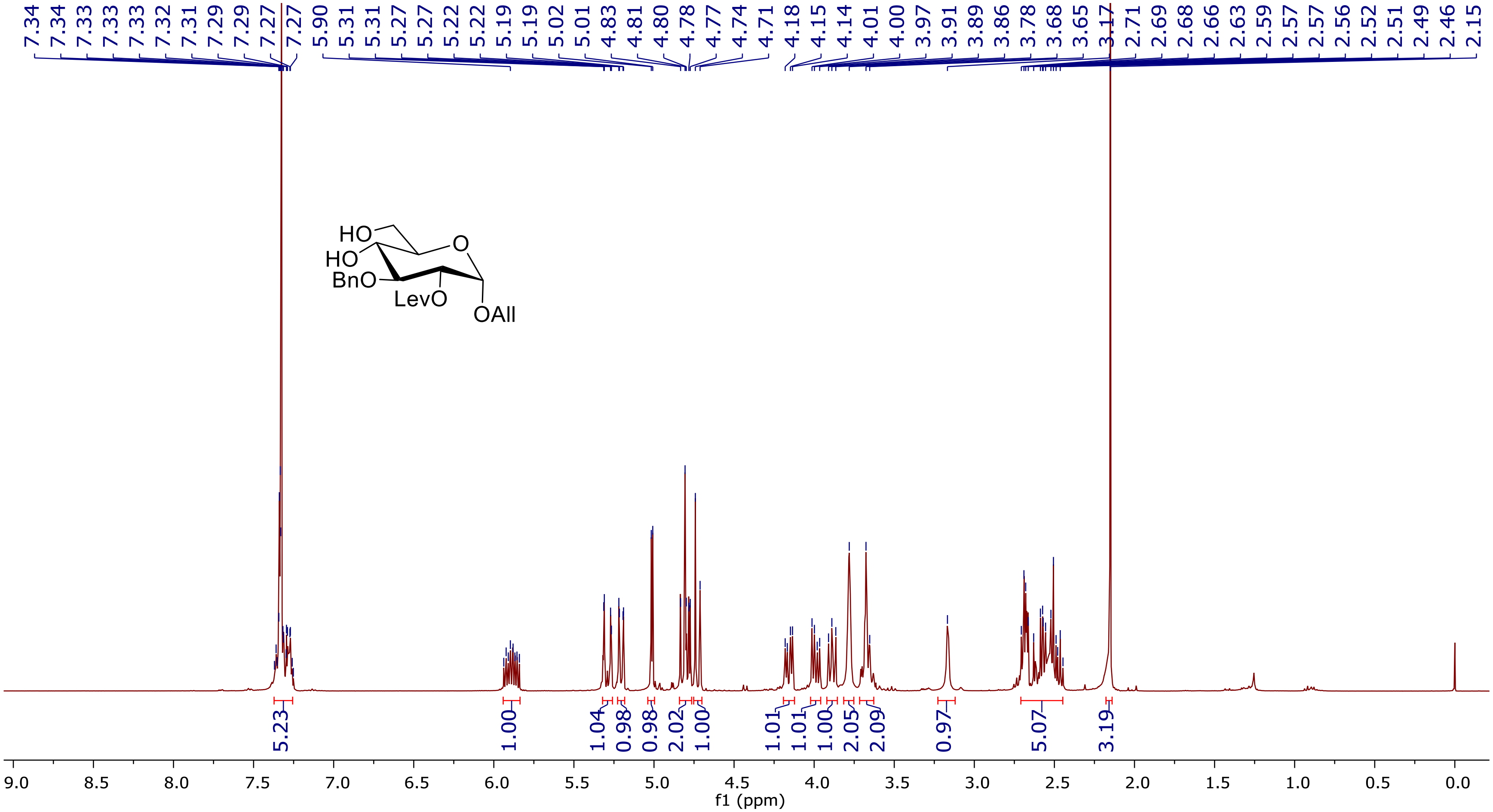

Supplementary Figure S65b. ^{13}C NMR Spectrum (100.67 MHz, $CDCl_3$) of Compound S12a

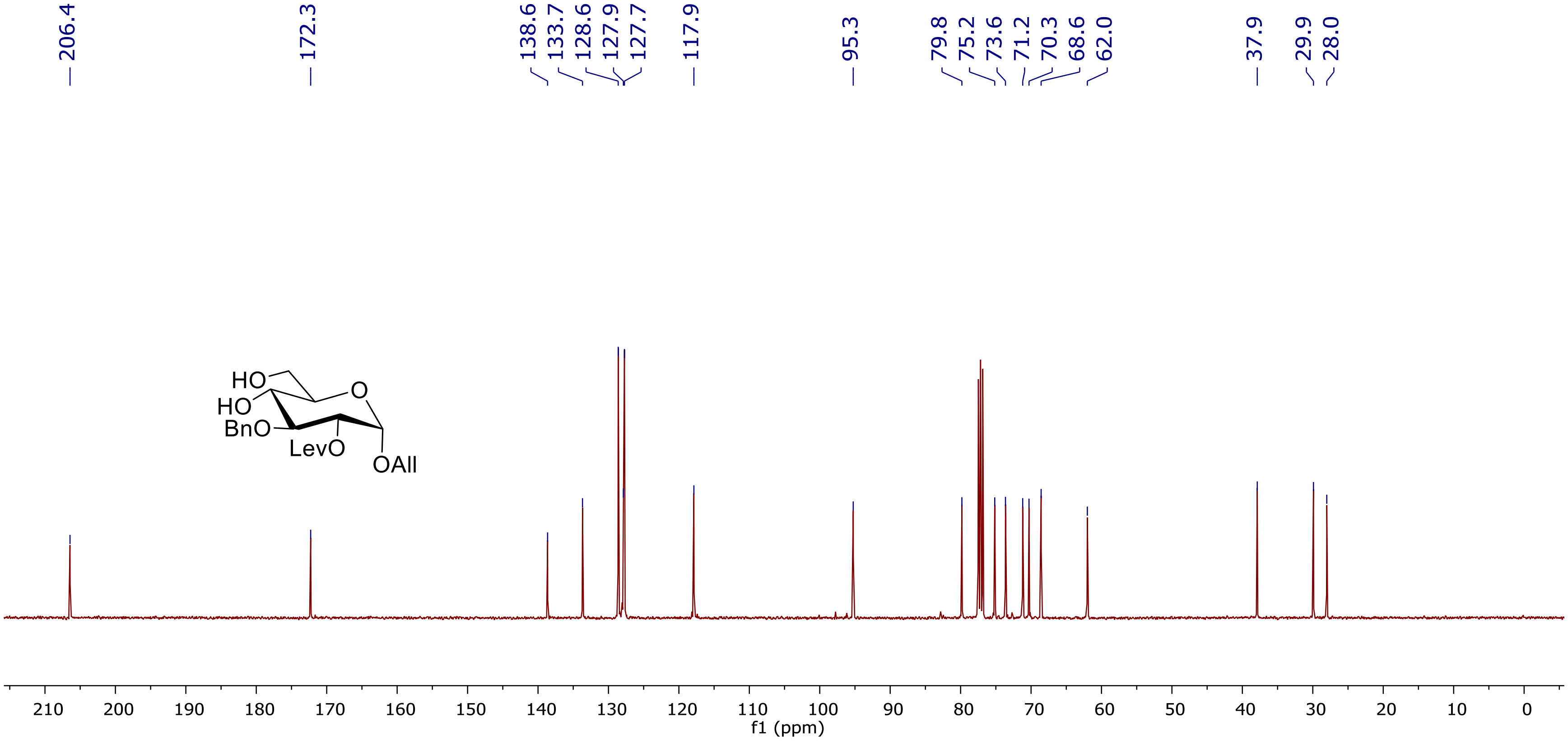

Supplementary Figure S65c. DEPT NMR Spectrum (100.67 MHz, $CDCl_3$) of Compound S12a

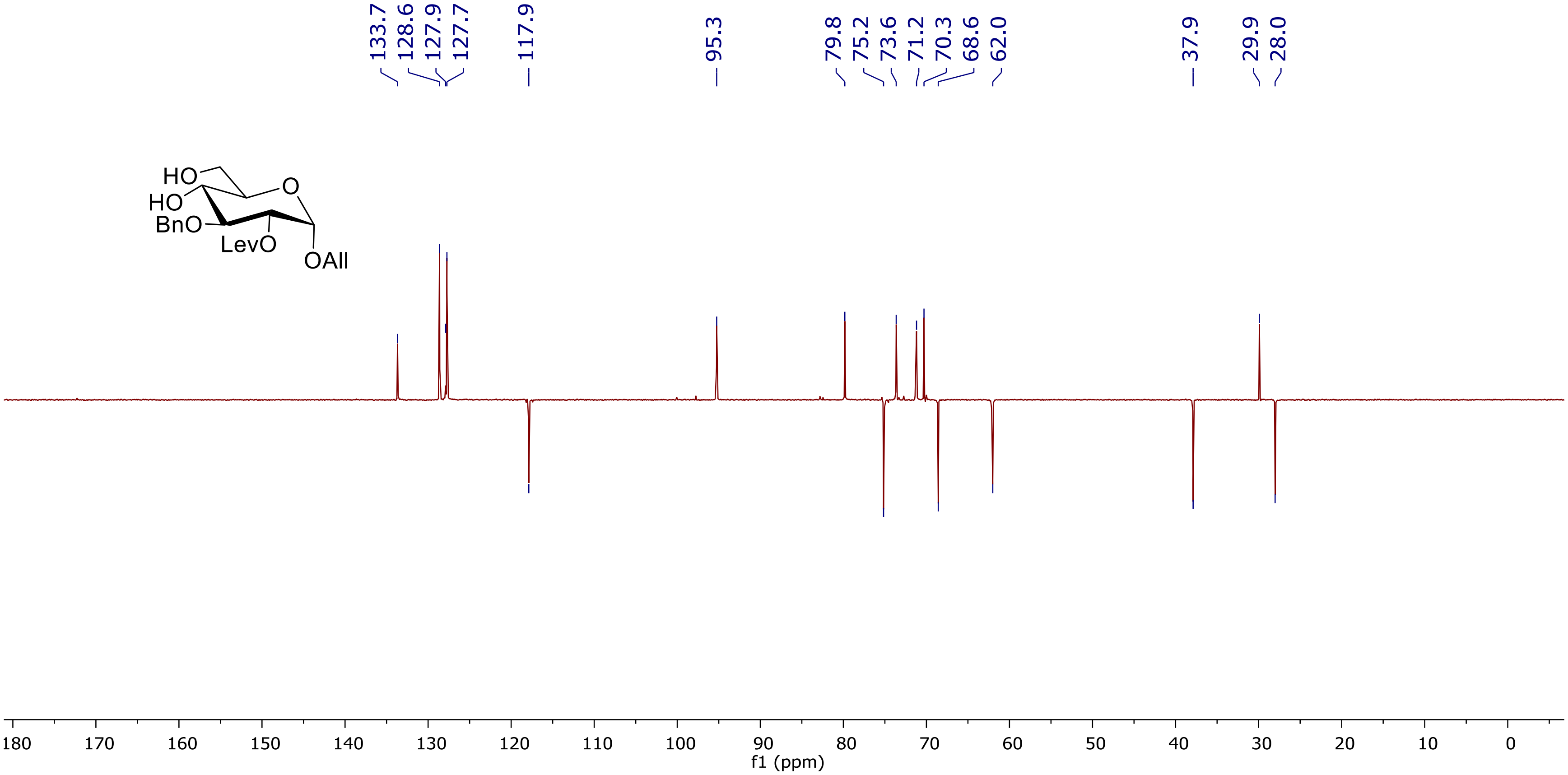

Supplementary Figure S66a. ¹H NMR Spectrum (400.31 MHz, $CDCl_3$) of Compound S12b

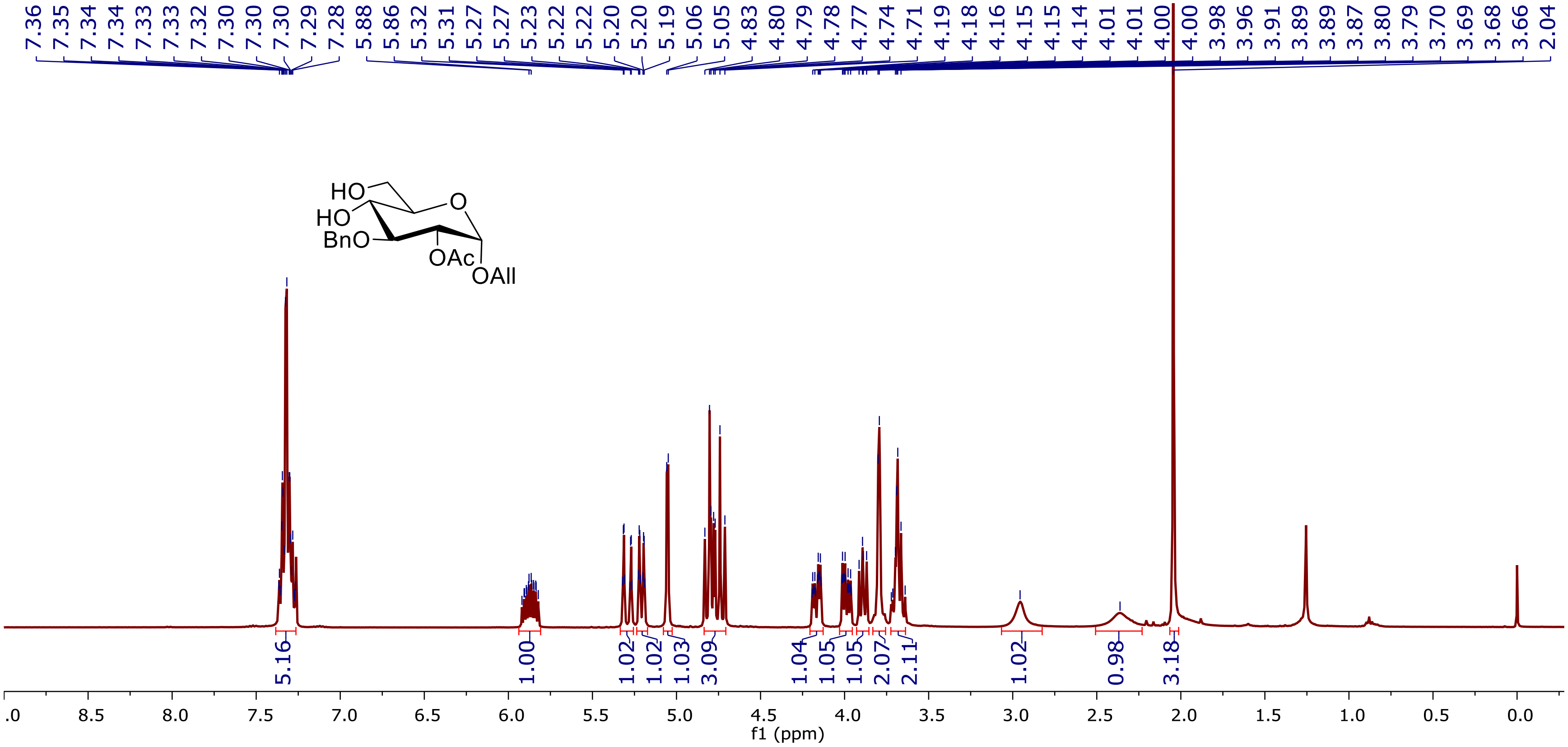

Supplementary Figure S66b. ^{13}C NMR Spectrum (100.67 MHz, $CDCl_3$) of Compound S12b

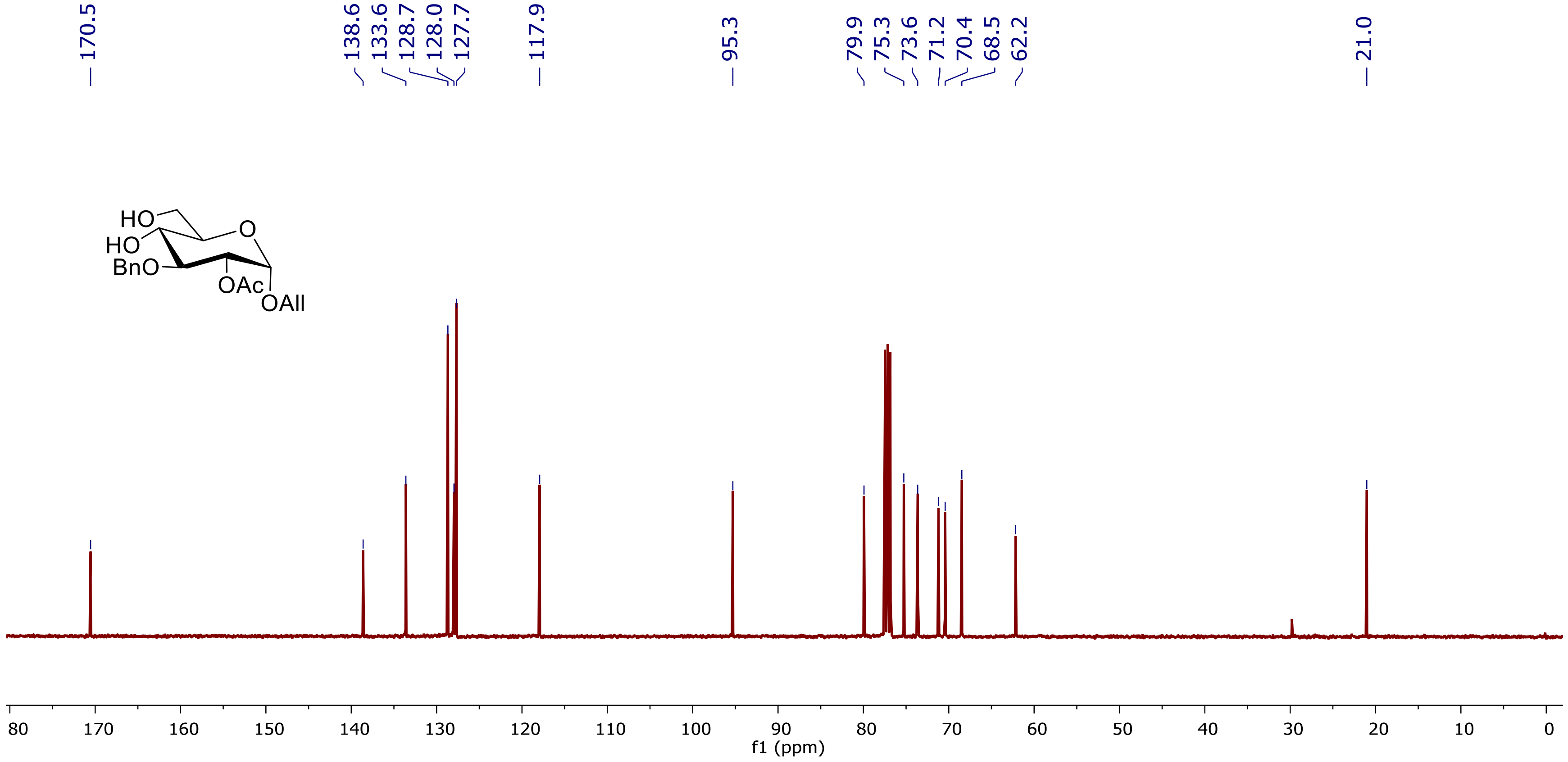

Supplementary Figure S66c. DEPT NMR Spectrum (100.67 MHz, $CDCl_3$) of Compound S12b

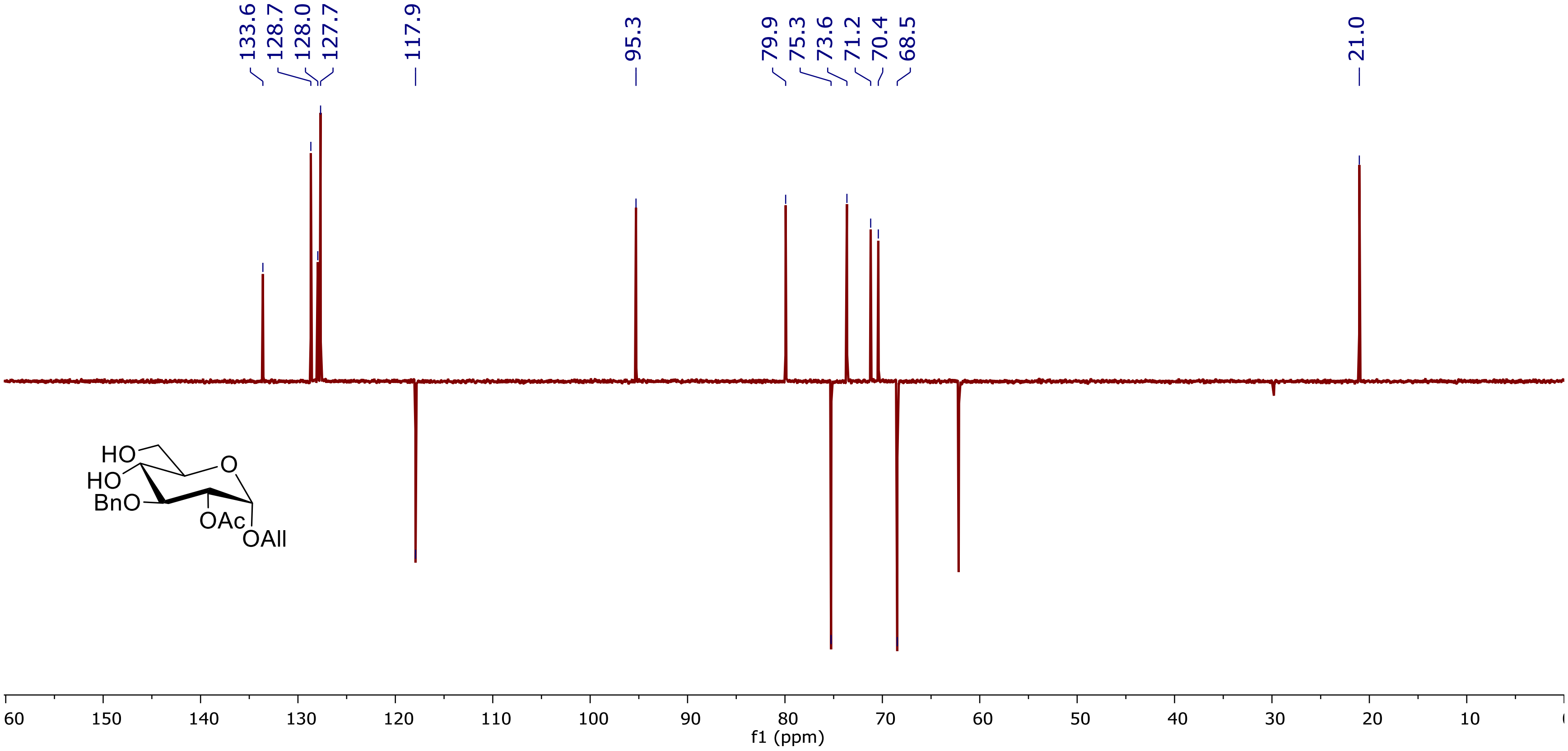

Supplementary Figure S67a. ^{1}H NMR Spectrum (400.31 MHz, $CDCl_3$) of Compound S12c

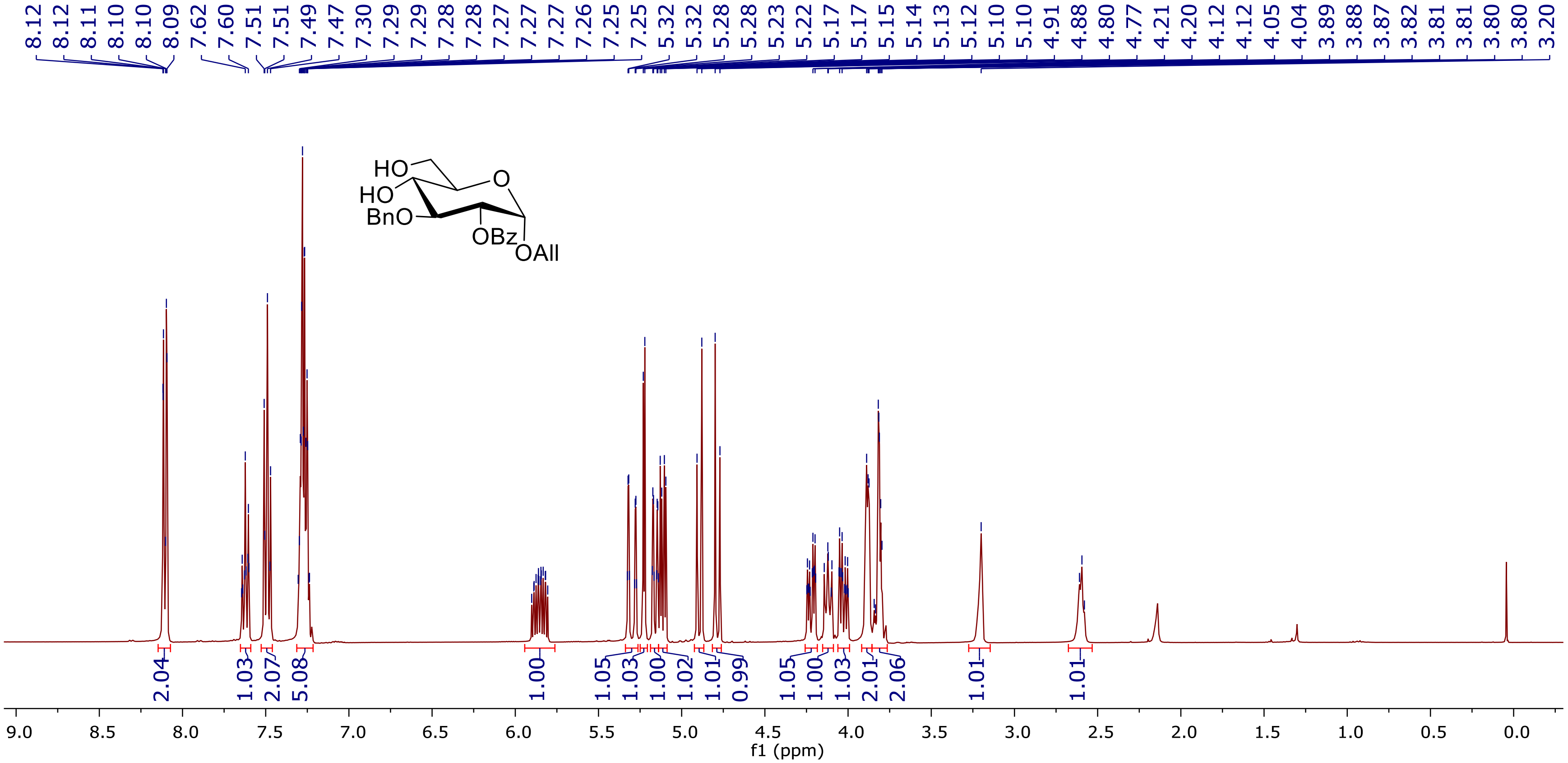

Supplementary Figure S67b. ^{13}C NMR Spectrum (100.67 MHz, $CDCl_3$) of Compound S12c

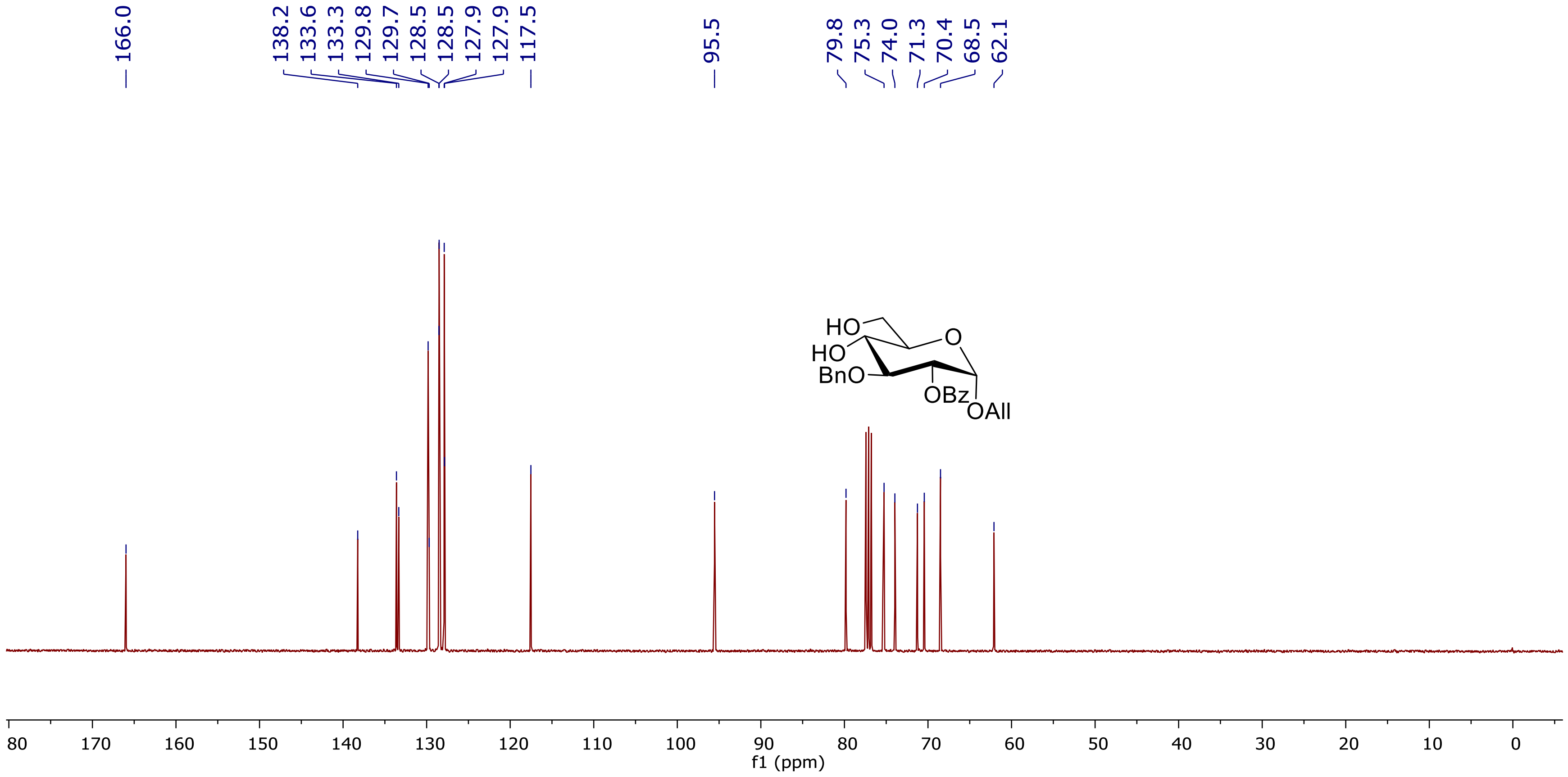

Supplementary Figure S67c. DEPT NMR Spectrum (100.67 MHz, $CDCl_3$) of Compound S12c

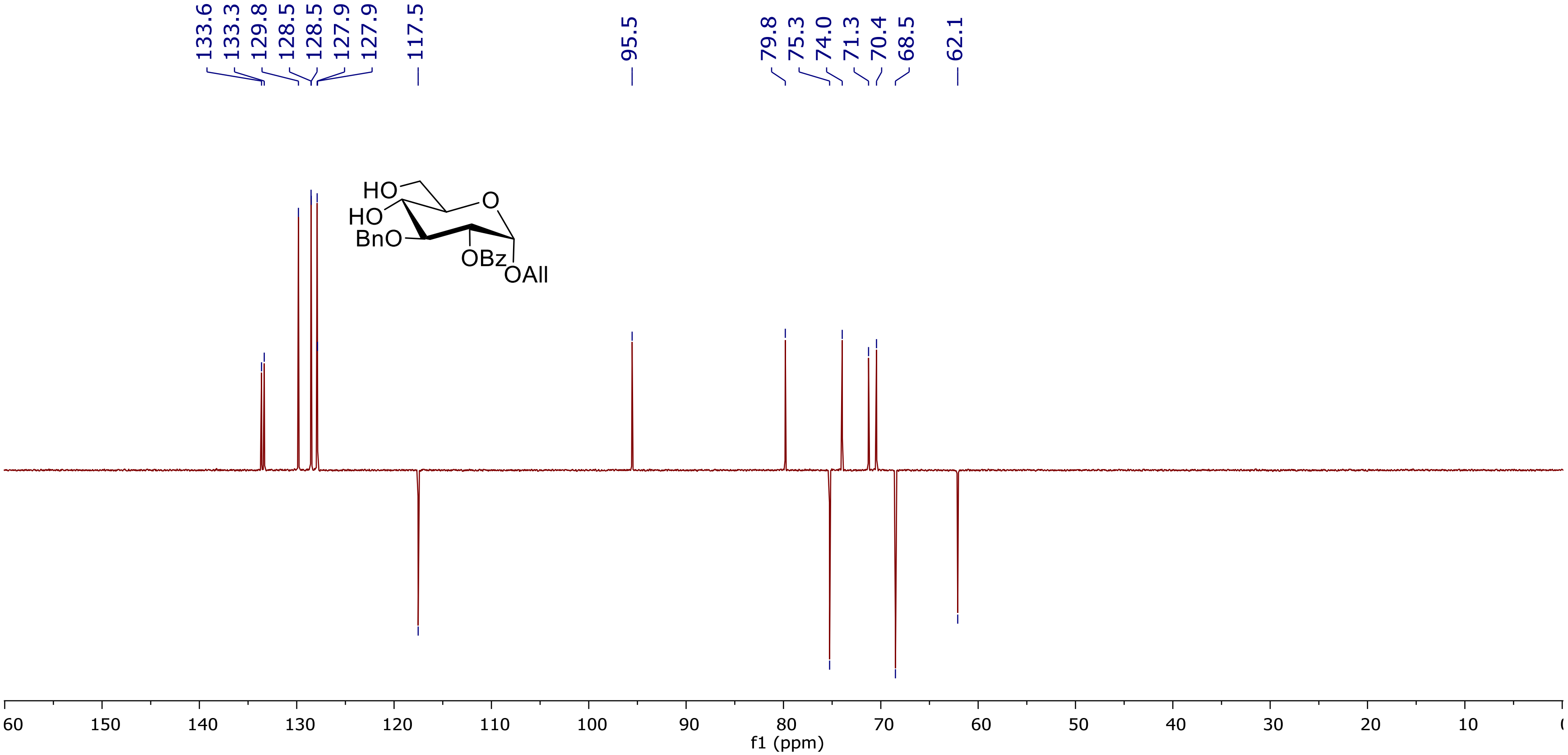

Supplementary Figure S68a. ^{1}H NMR Spectrum (400.31 MHz, $CDCl_3$) of Compound 36a

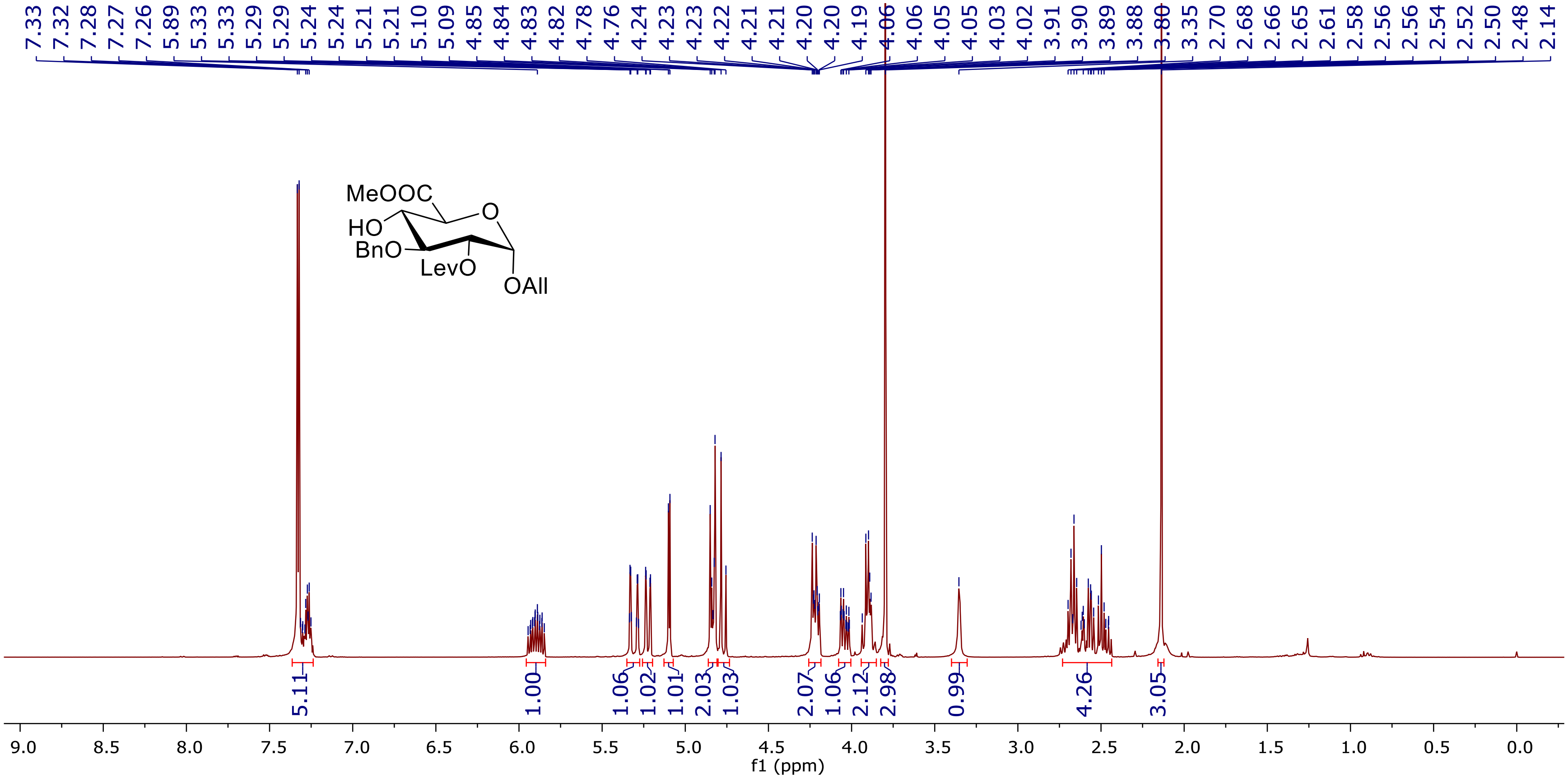

Supplementary Figure S68b. ^{13}C NMR Spectrum (100.67 MHz, $CDCl_3$) of Compound 36a

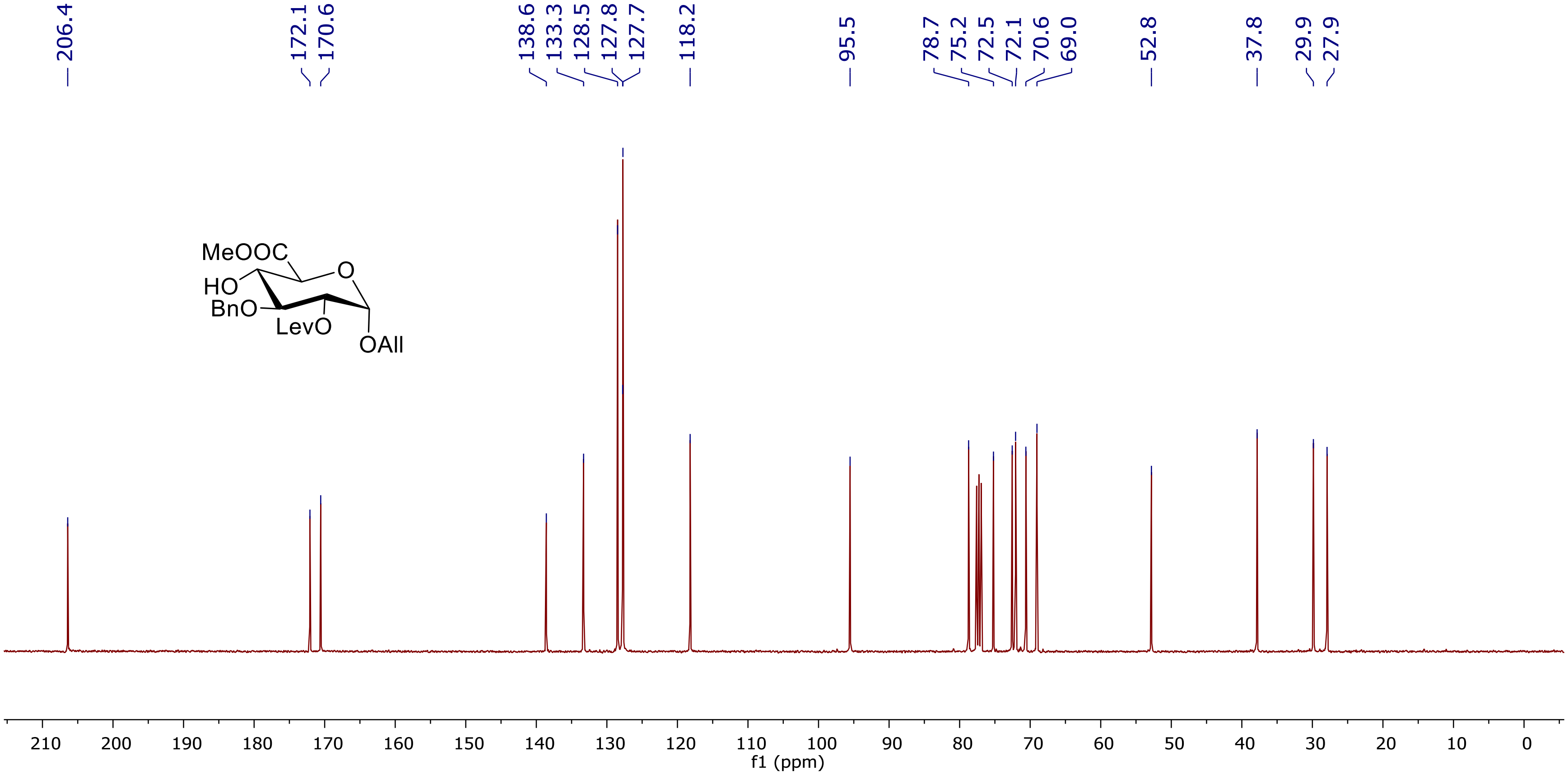

Supplementary Figure S68c. DEPT NMR Spectrum (100.67 MHz, $CDCl_3$) of Compound 36a

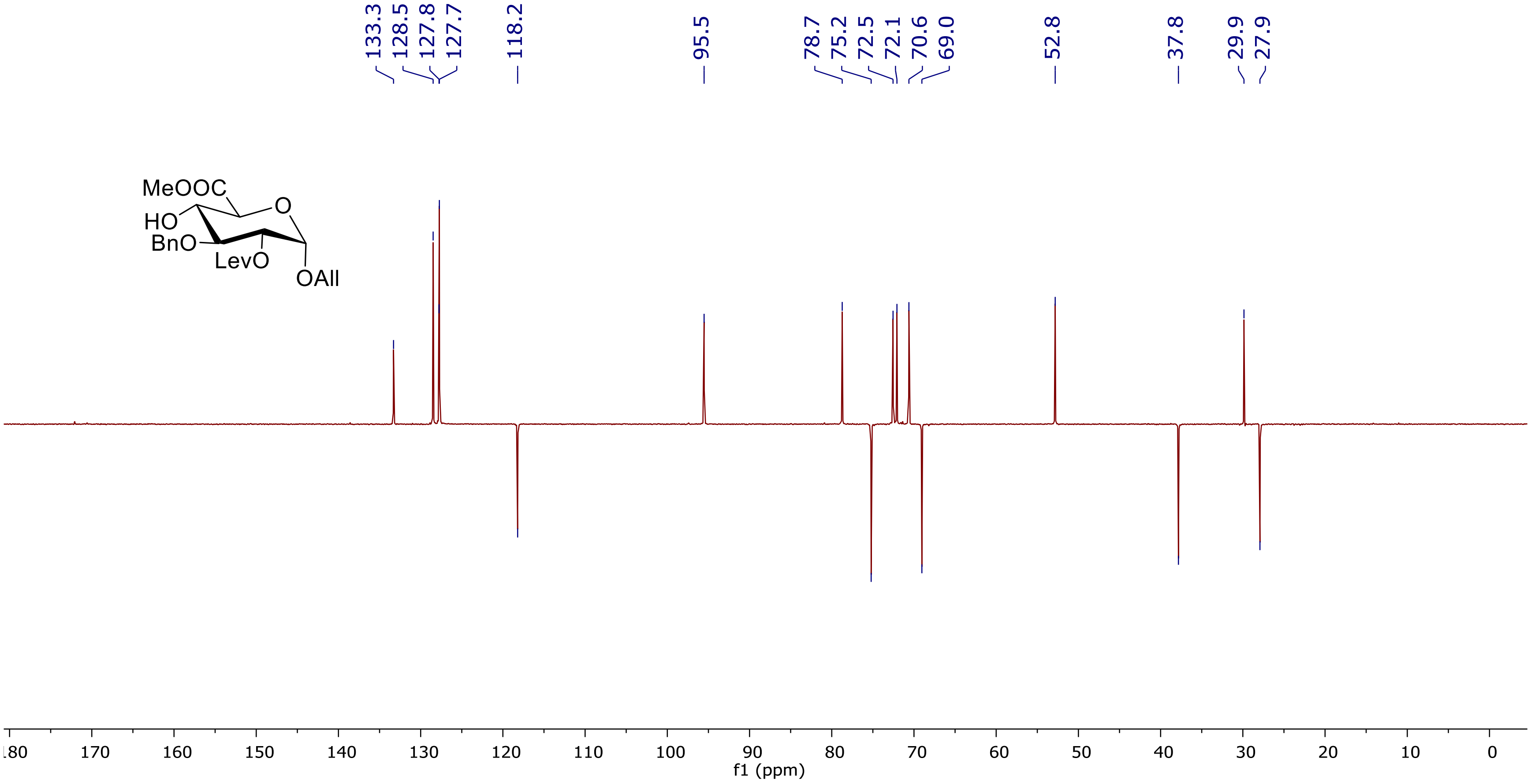

Supplementary Figure S69a. ^{1}H NMR Spectrum (400.31 MHz, $CDCl_3$) of Compound 36b

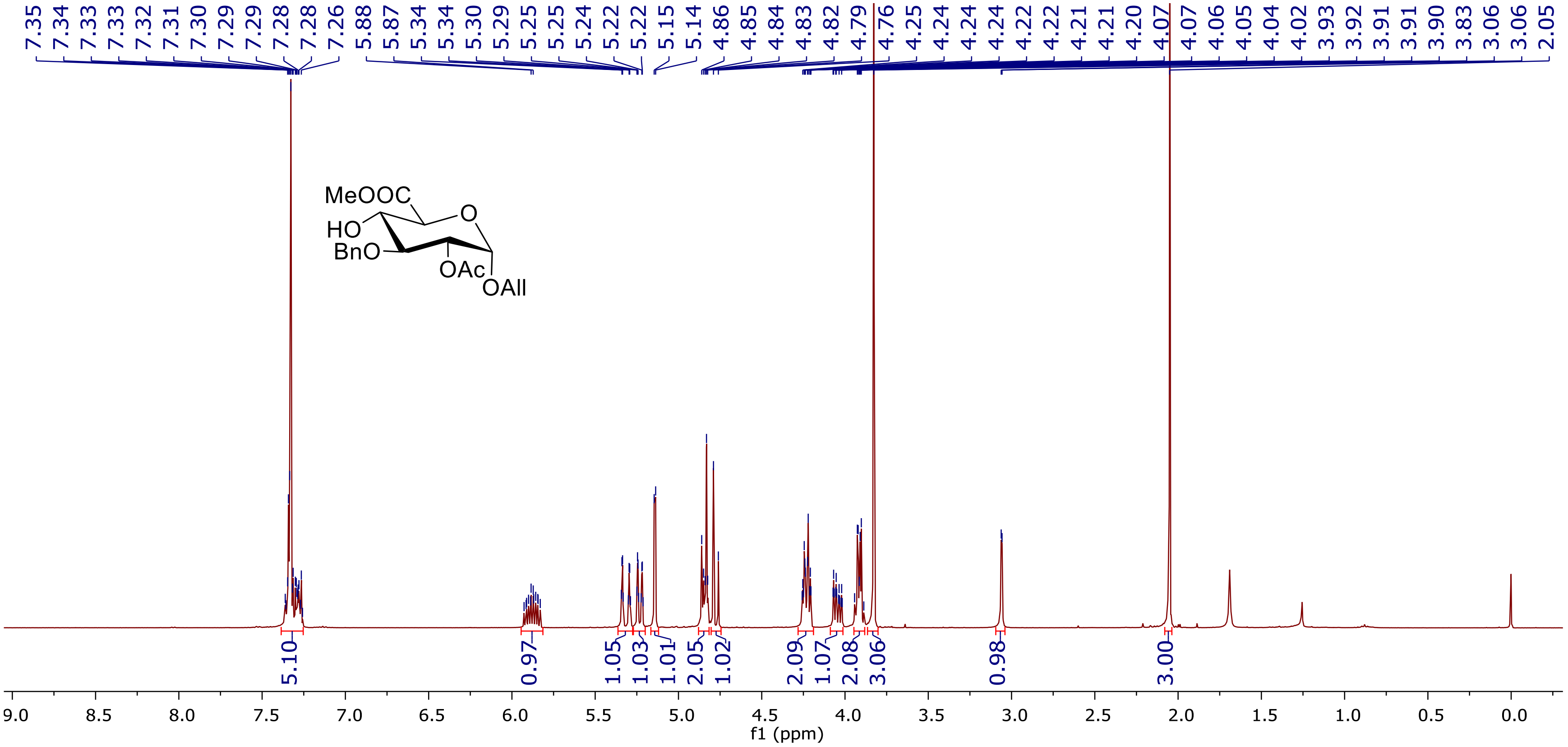

Supplementary Figure S69b. ^{13}C NMR Spectrum (100.67 MHz, $CDCl_3$) of Compound 36b

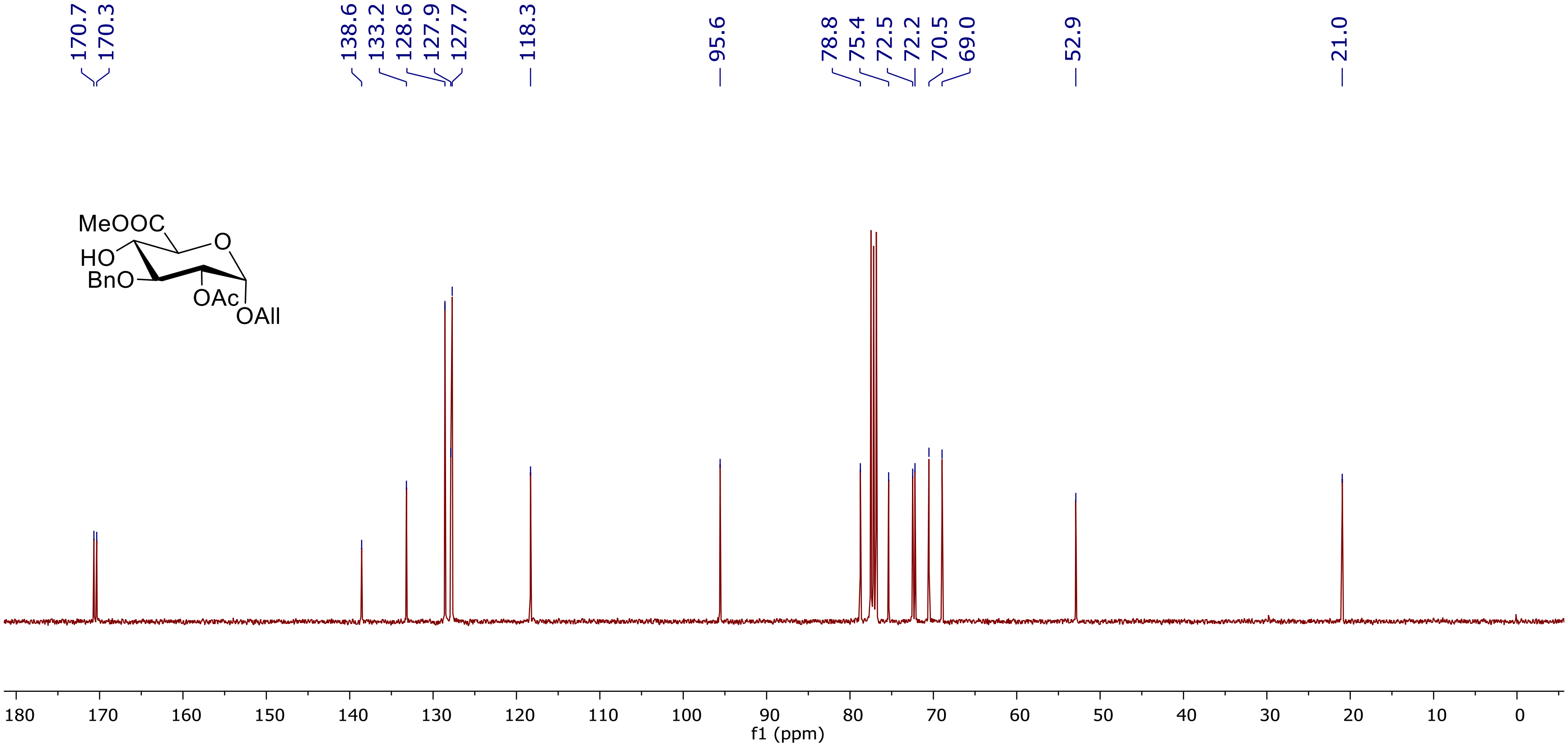

Supplementary Figure S69c. DEPT NMR Spectrum (100.67 MHz, $CDCl_3$) of Compound 36b

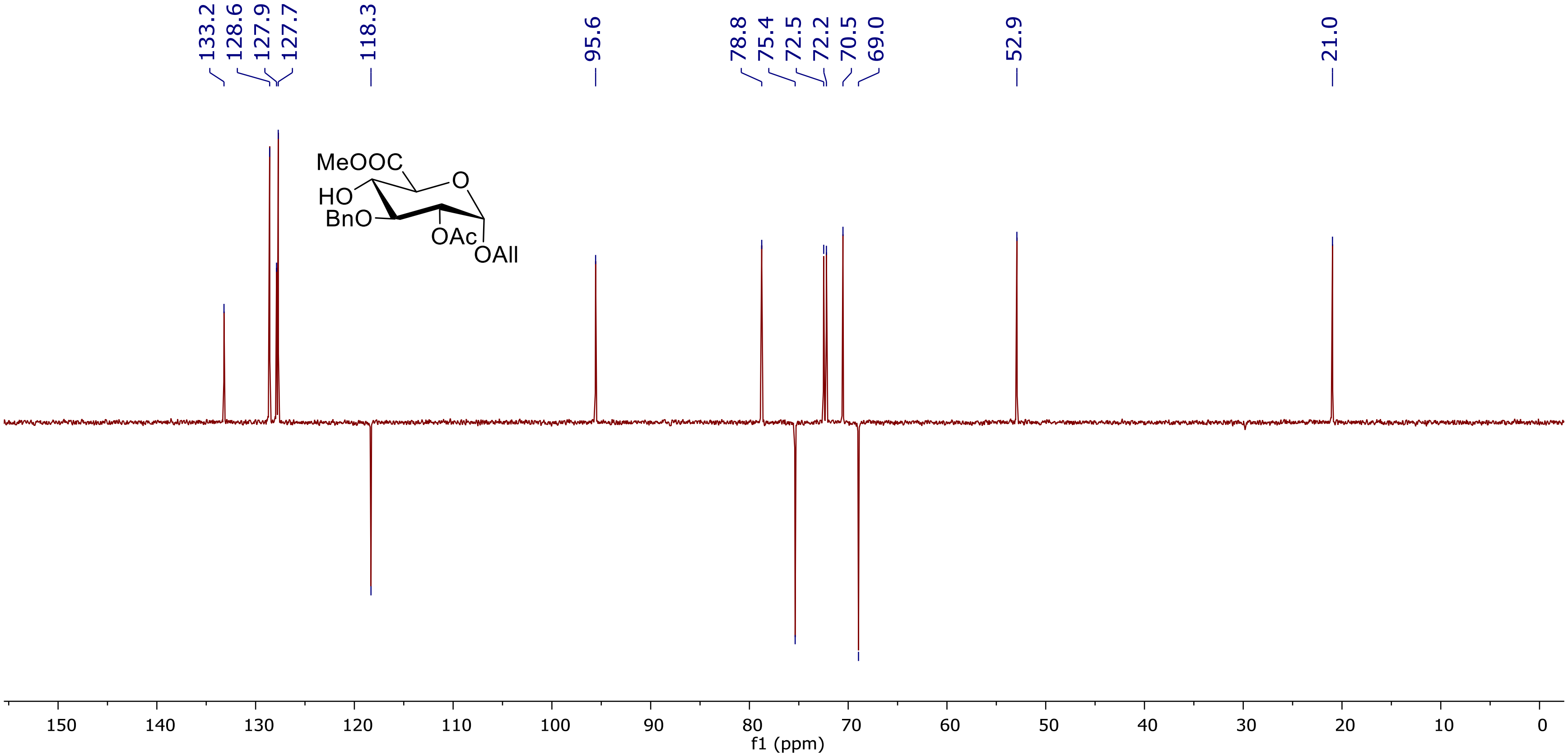

Supplementary Figure S70a. ^{1}H NMR Spectrum (400.31 MHz, $CDCl_3$) of Compound 36c

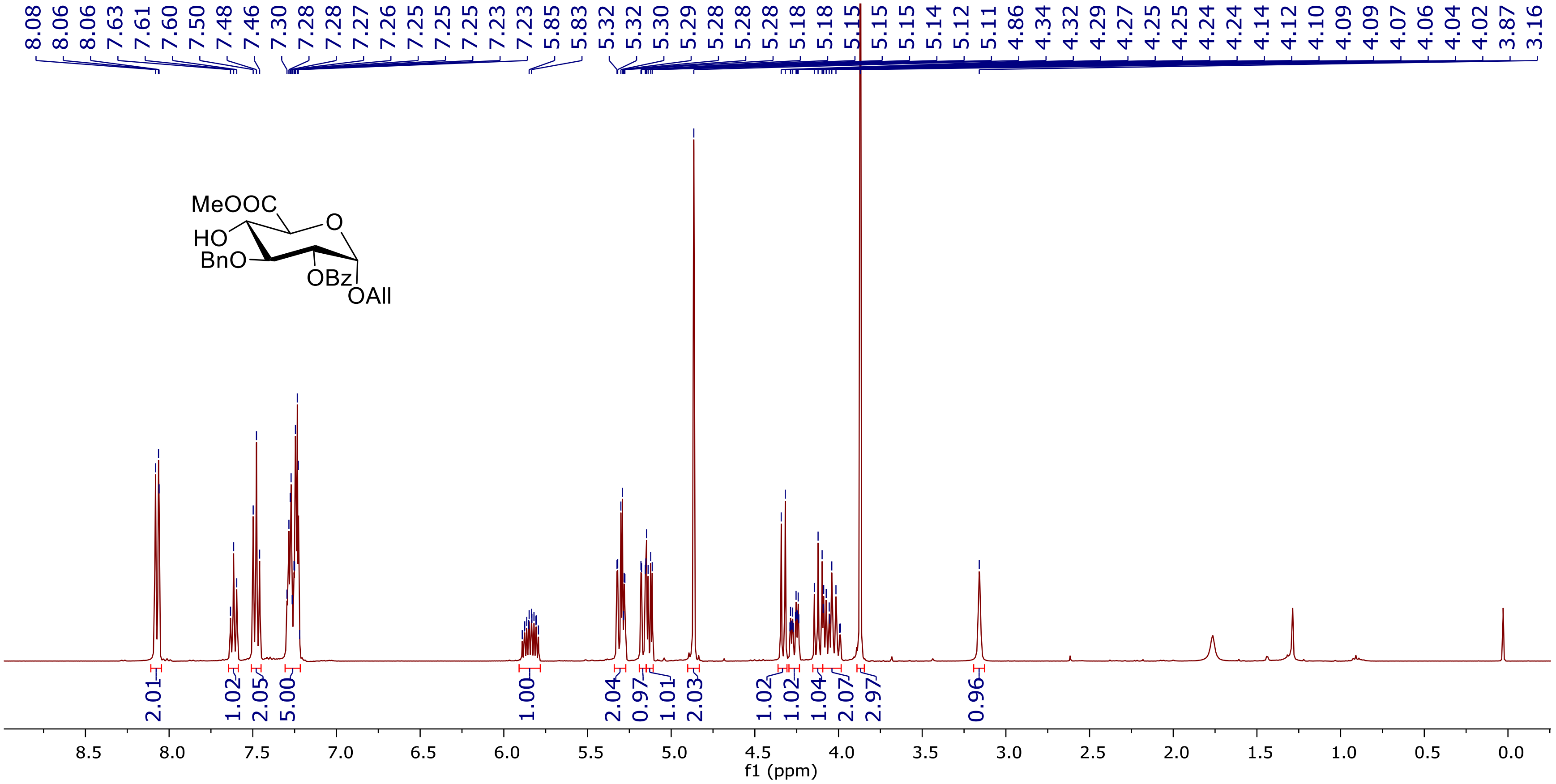

Supplementary Figure S70b. ^{13}C NMR Spectrum (100.67 MHz, $CDCl_3$) of Compound 36c

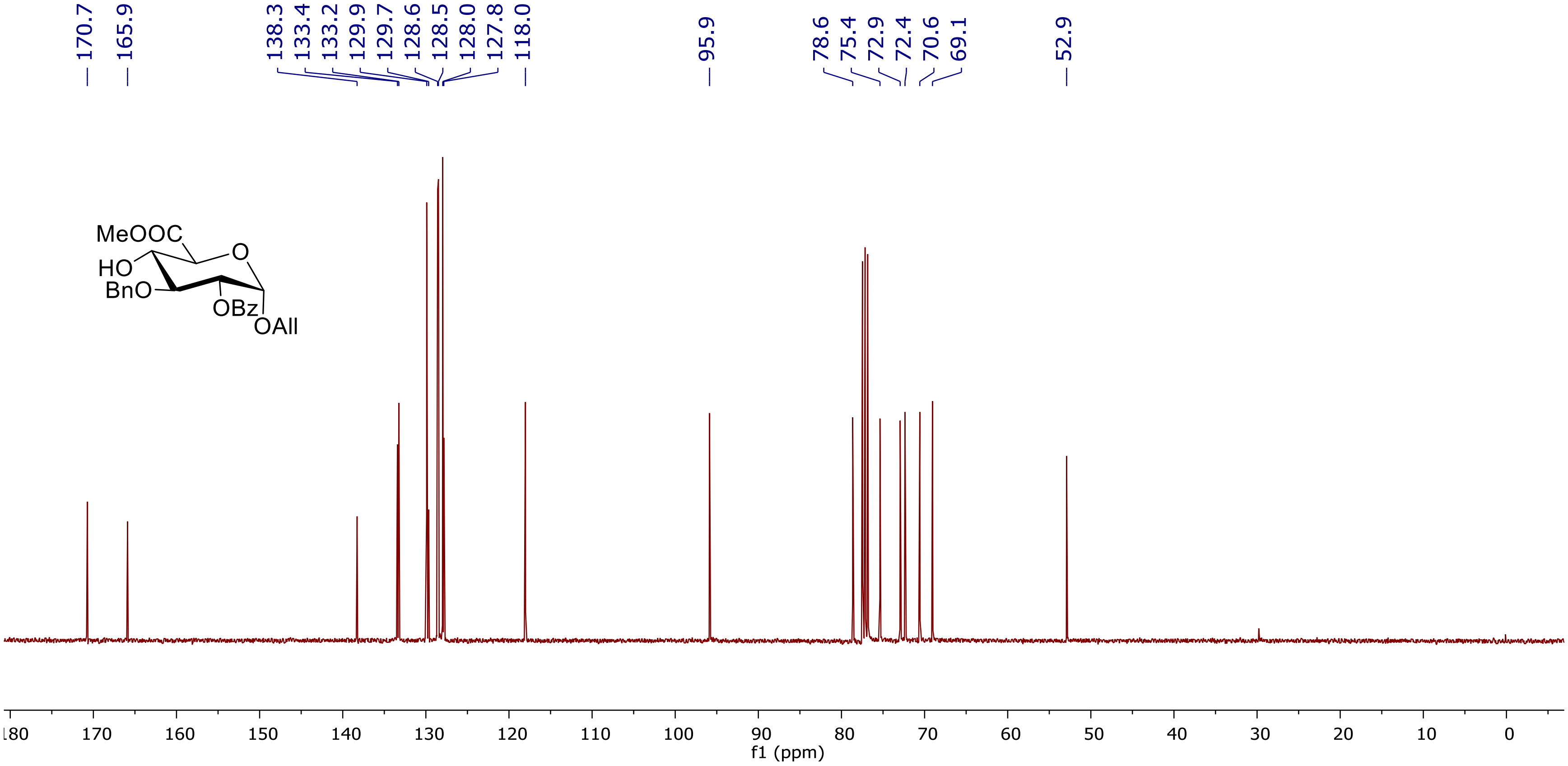

Supplementary Figure S70c. DEPT NMR Spectrum (100.67 MHz, $CDCl_3$) of Compound 36c

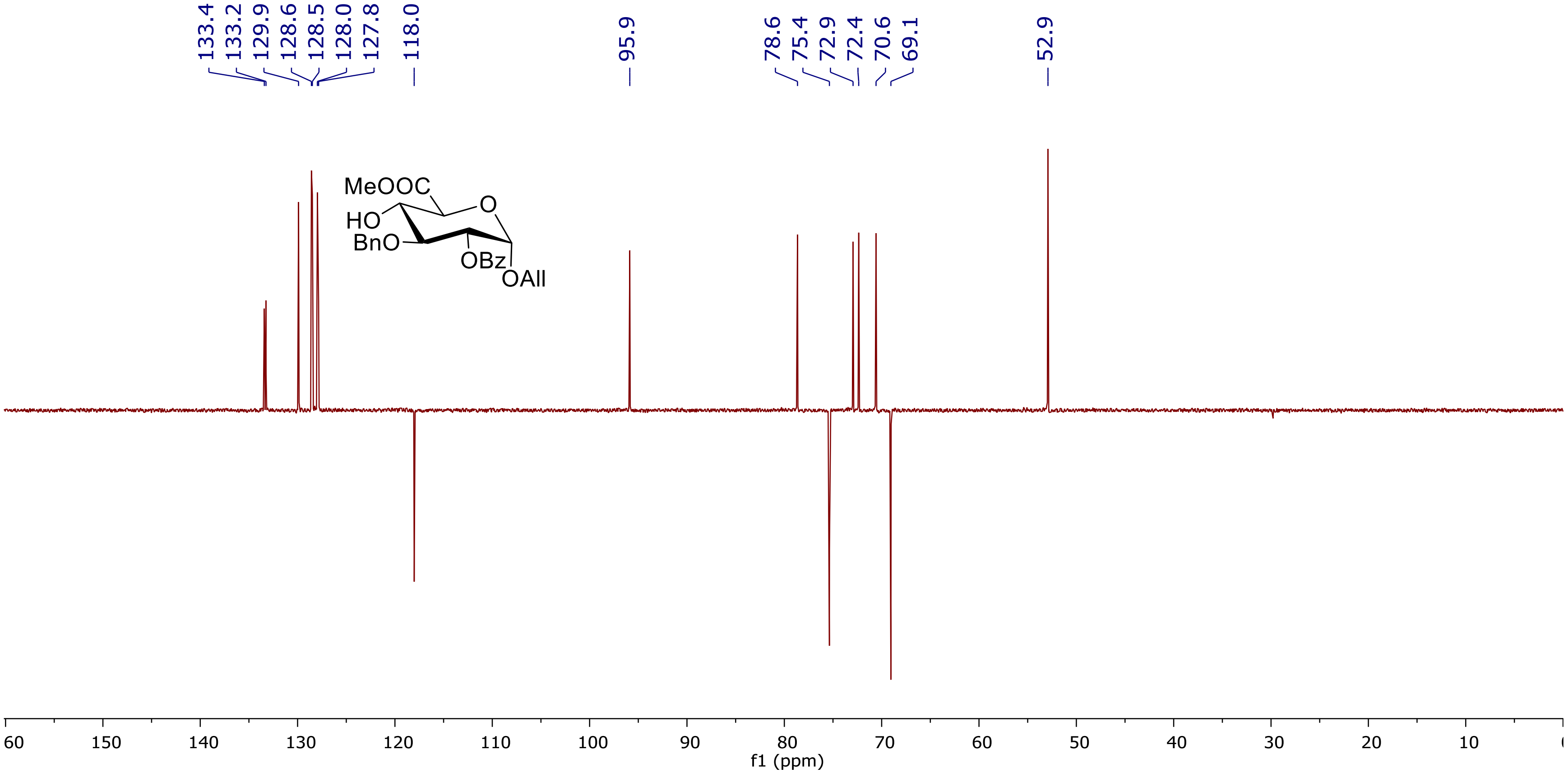

Supplementary Figure S71a. ^{1}H NMR Spectrum (400.31 MHz, $CDCl_3$) of Compound 37a

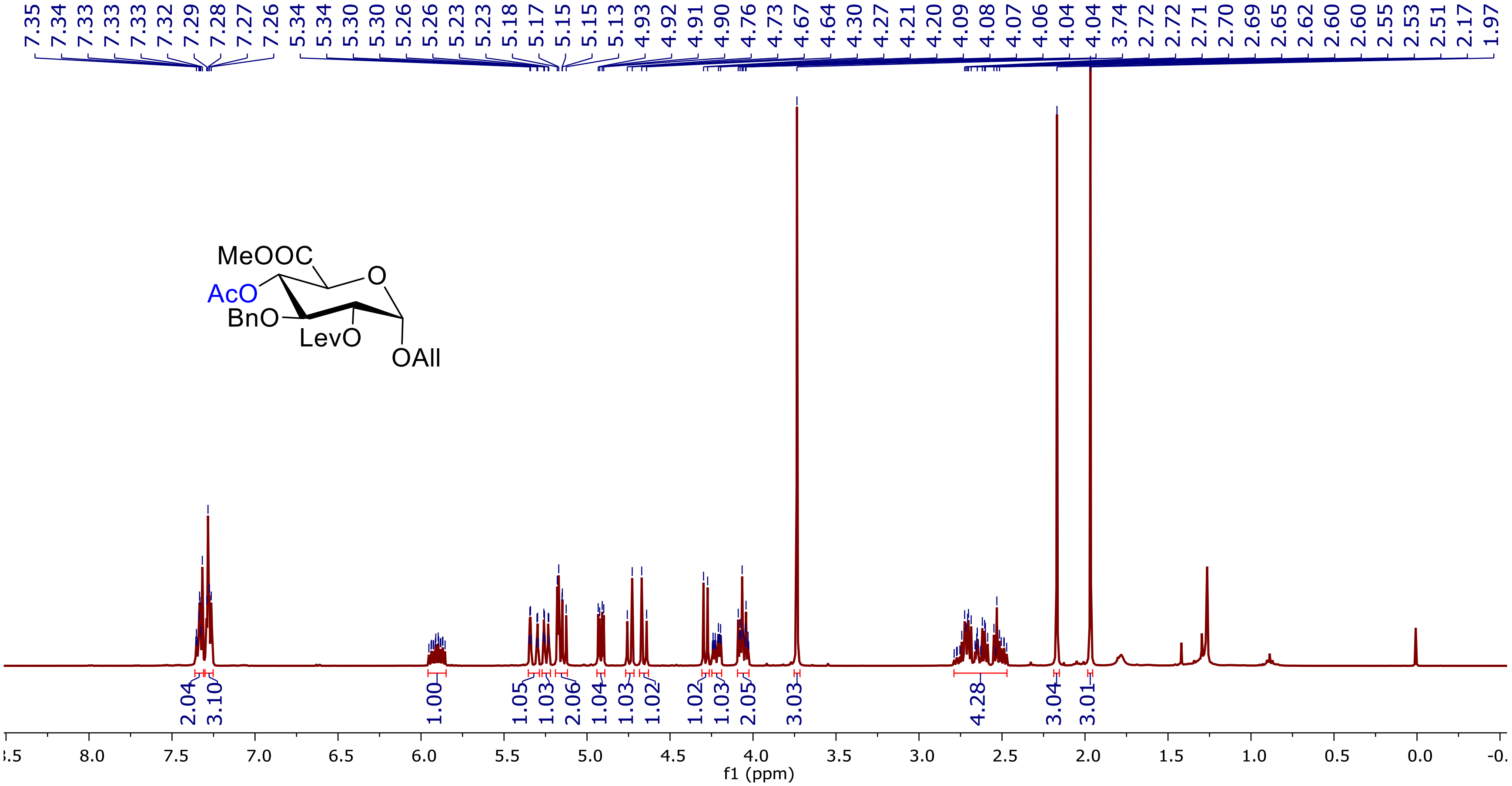

Supplementary Figure S71b. ^{13}C NMR Spectrum (100.67 MHz, $CDCl_3$) of Compound 37a

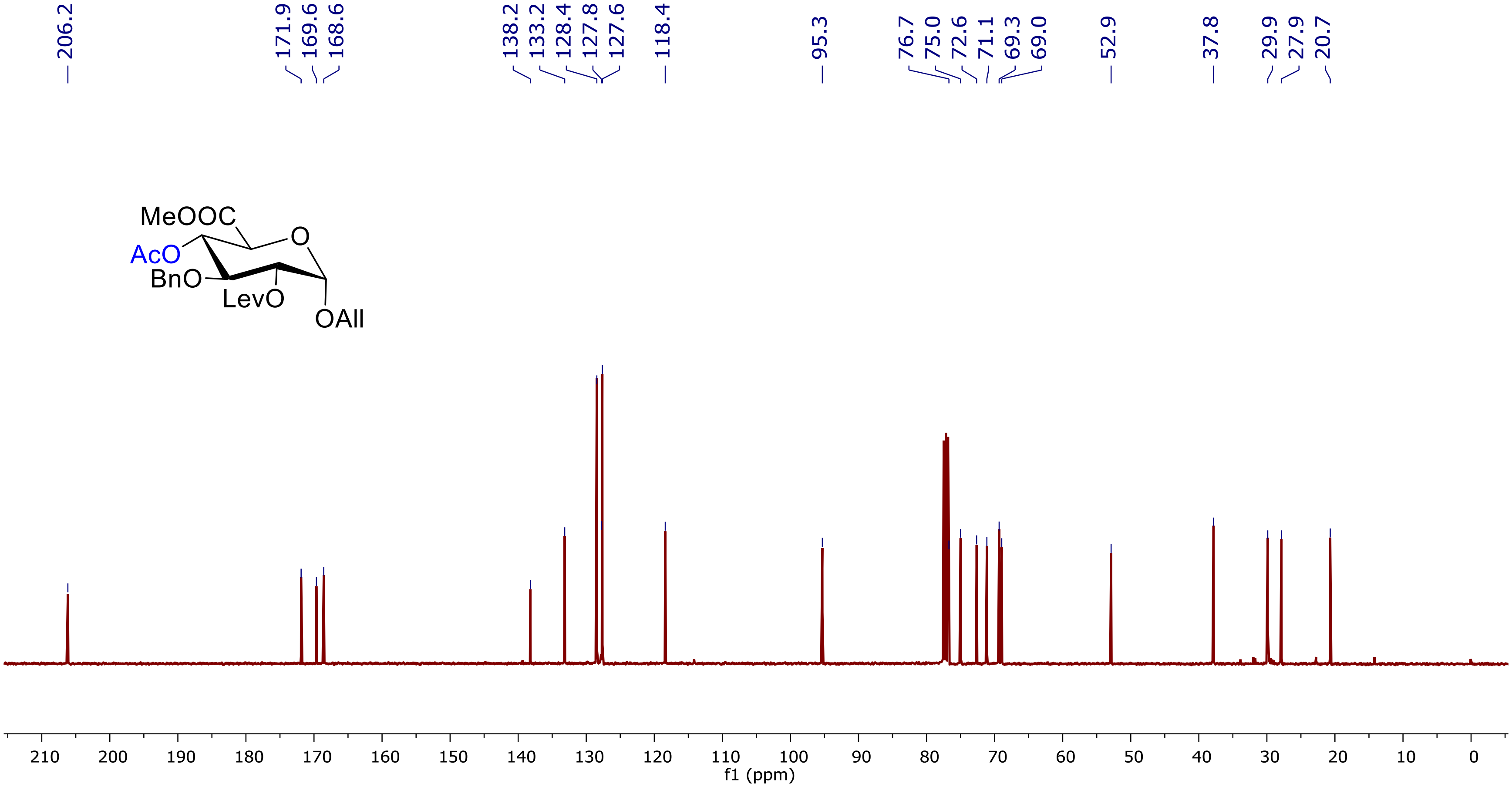

Supplementary Figure S71c. DEPT NMR Spectrum (100.67 MHz, $CDCl_3$) of Compound 37a

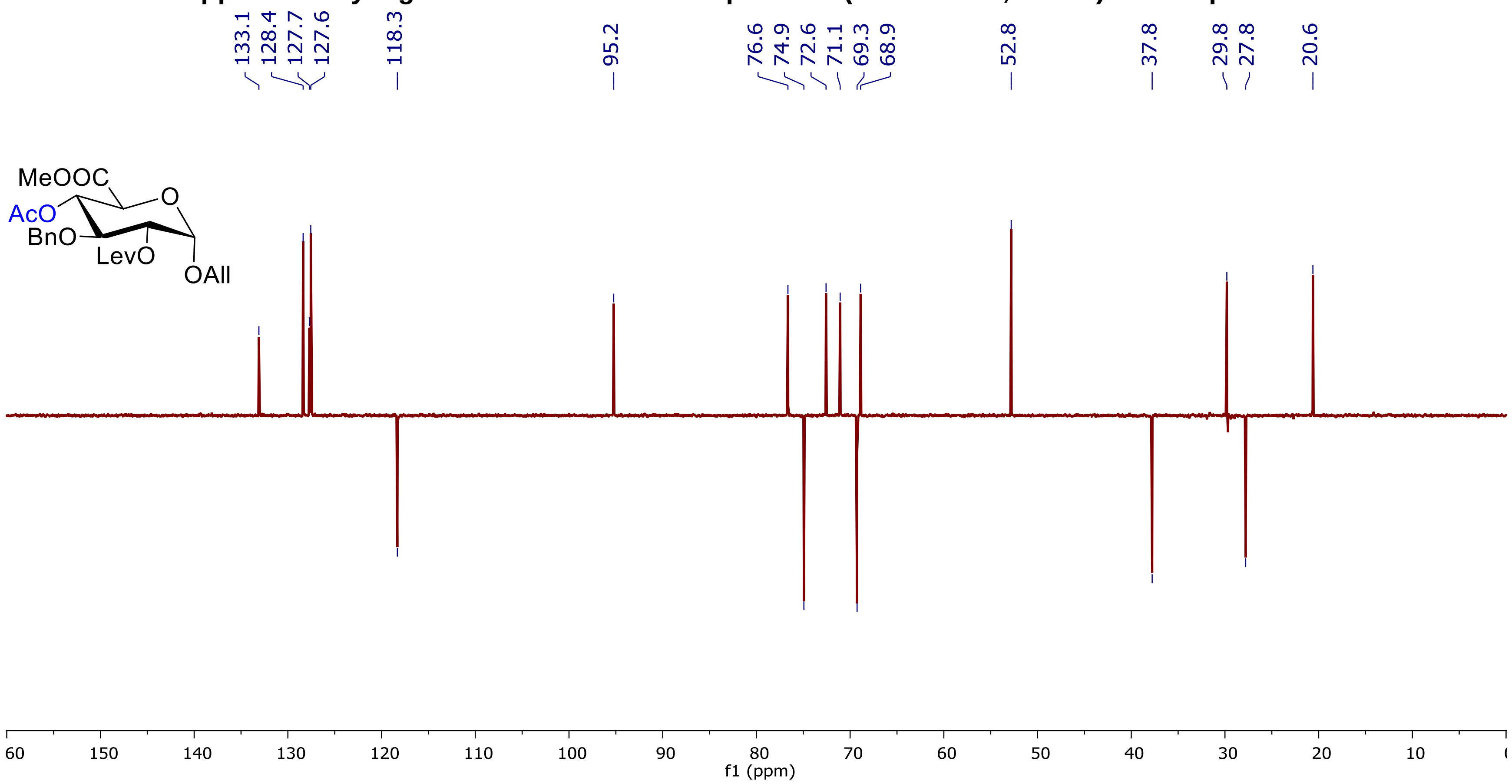

Supplementary Figure S72a. ¹H NMR Spectrum (400.31 MHz, $CDCl_3$) of Compound 37b

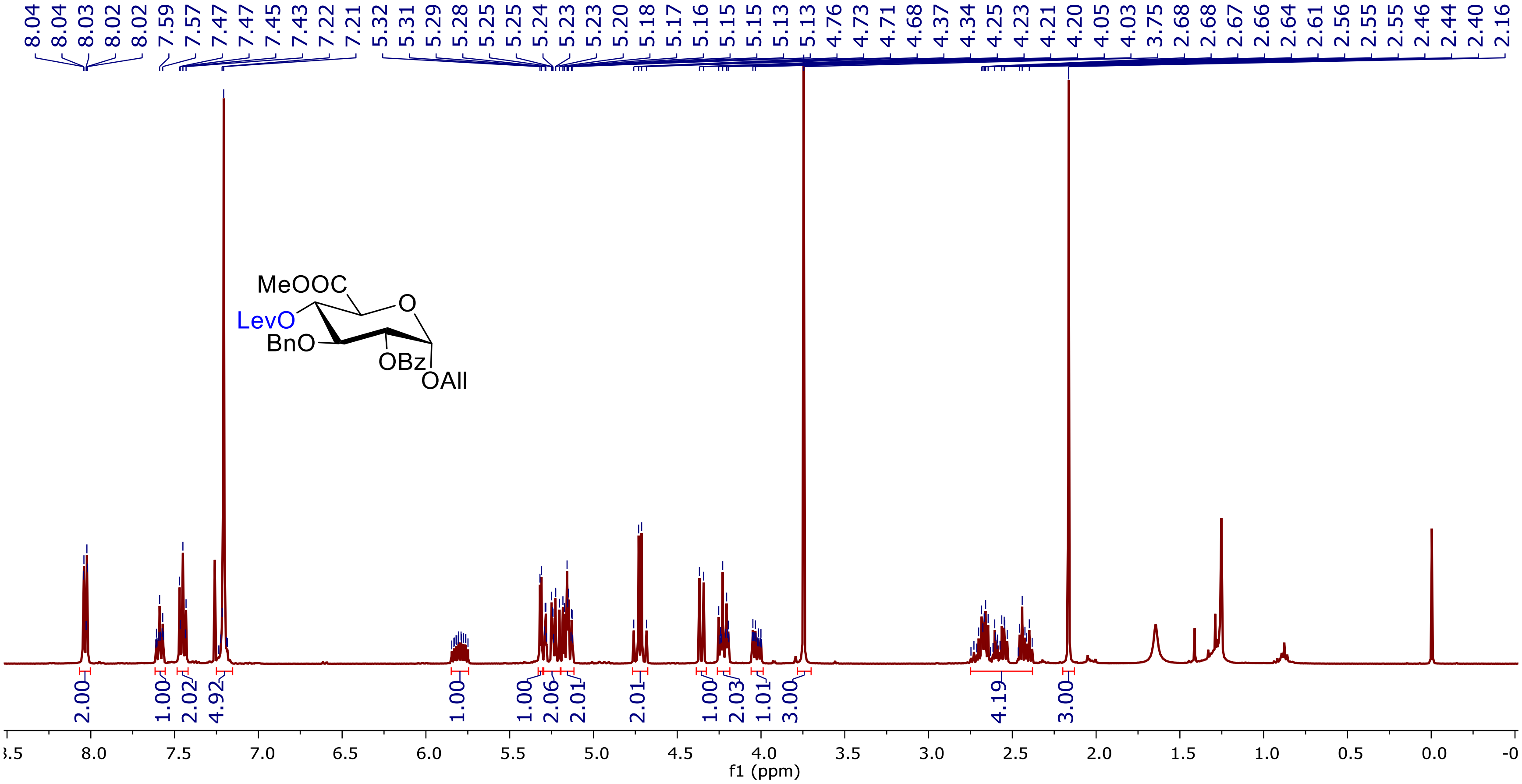

Supplementary Figure S72b. ^{13}C NMR Spectrum (100.67 MHz, $CDCl_3$) of Compound 37b

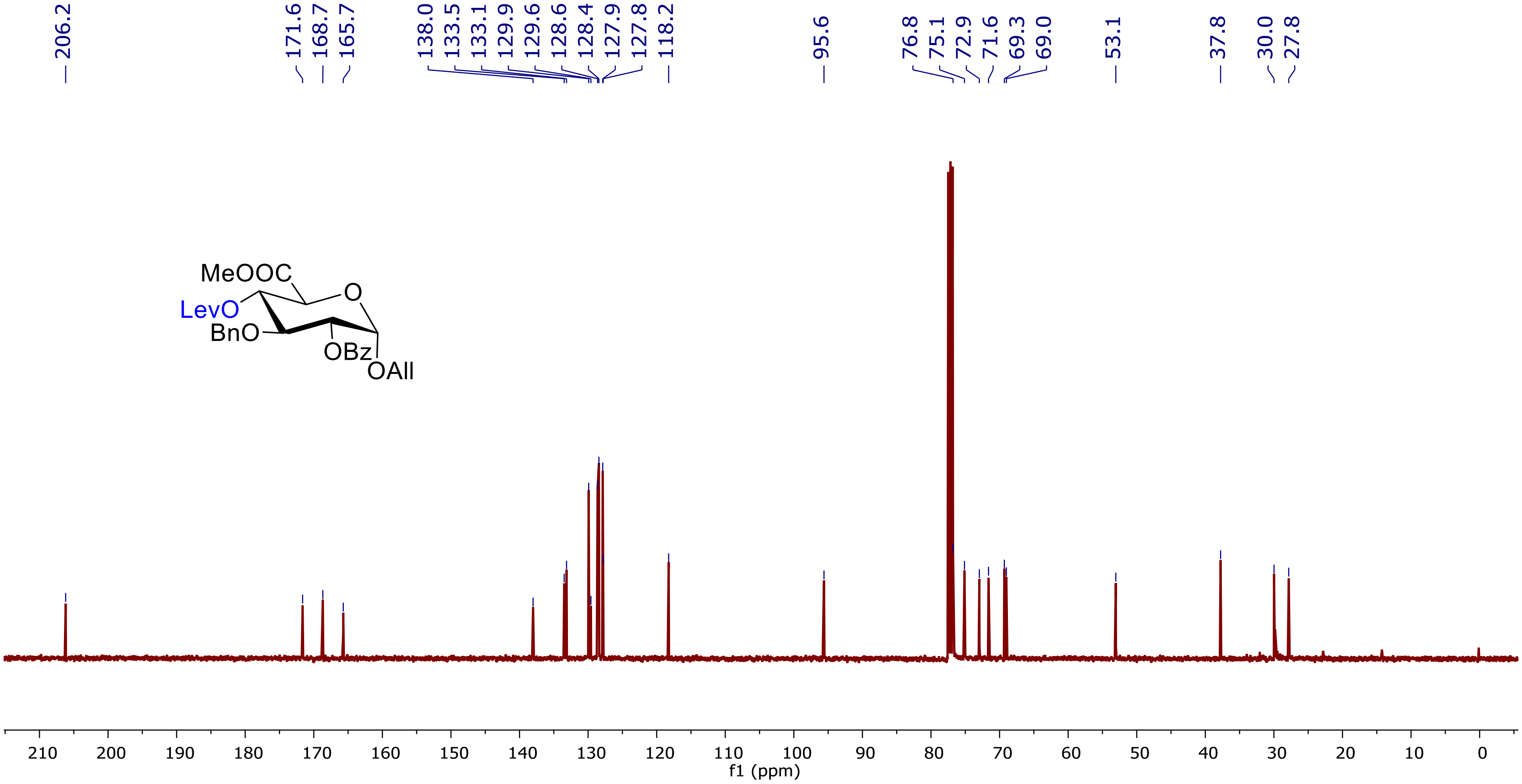

Supplementary Figure S72c. DEPT NMR Spectrum (100.67 MHz, $CDCl_3$) of Compound 37b

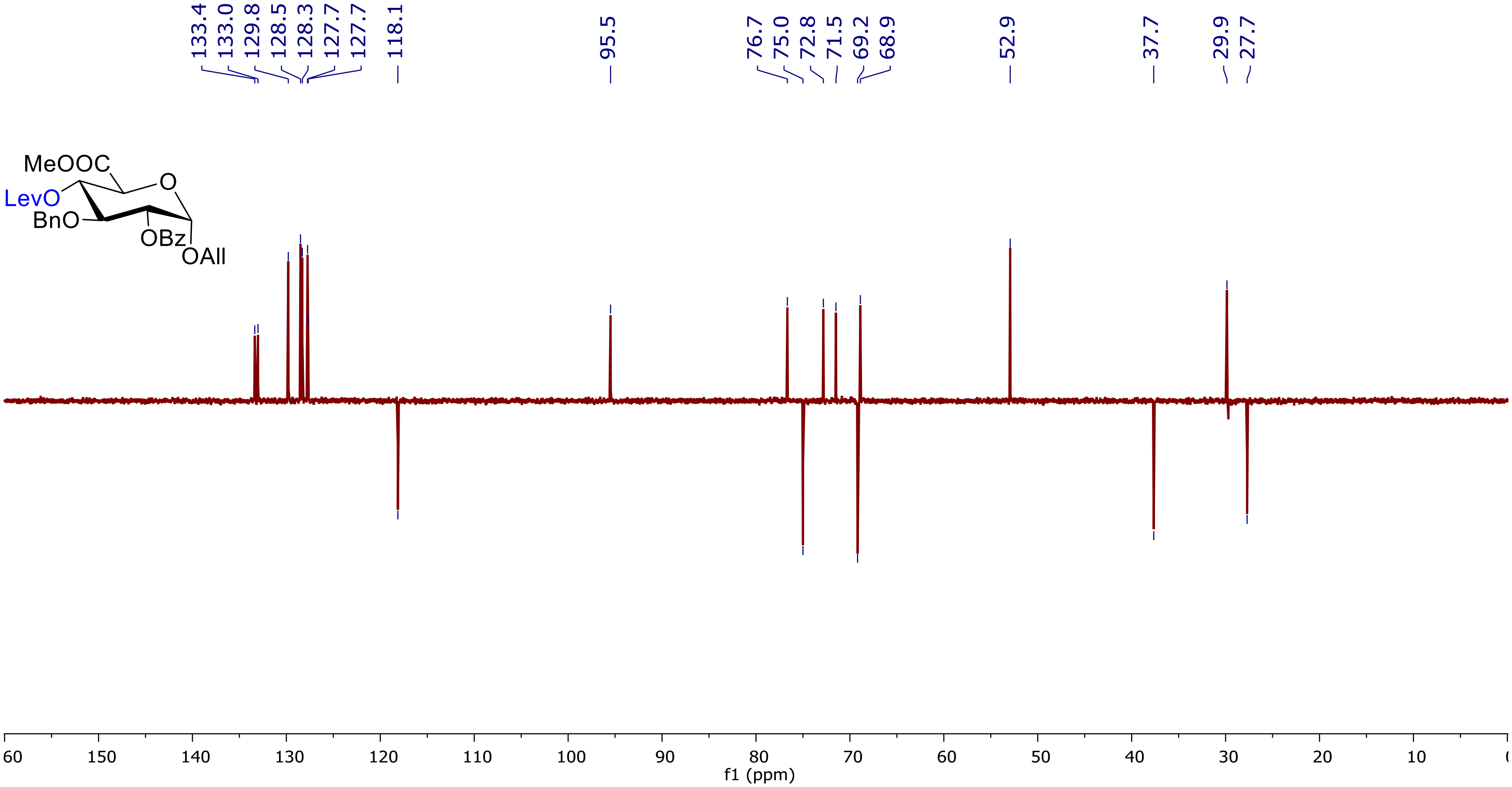

Supplementary Figure S73a. ¹H NMR Spectrum (400.31 MHz, $CDCl_3$) of Compound 37c

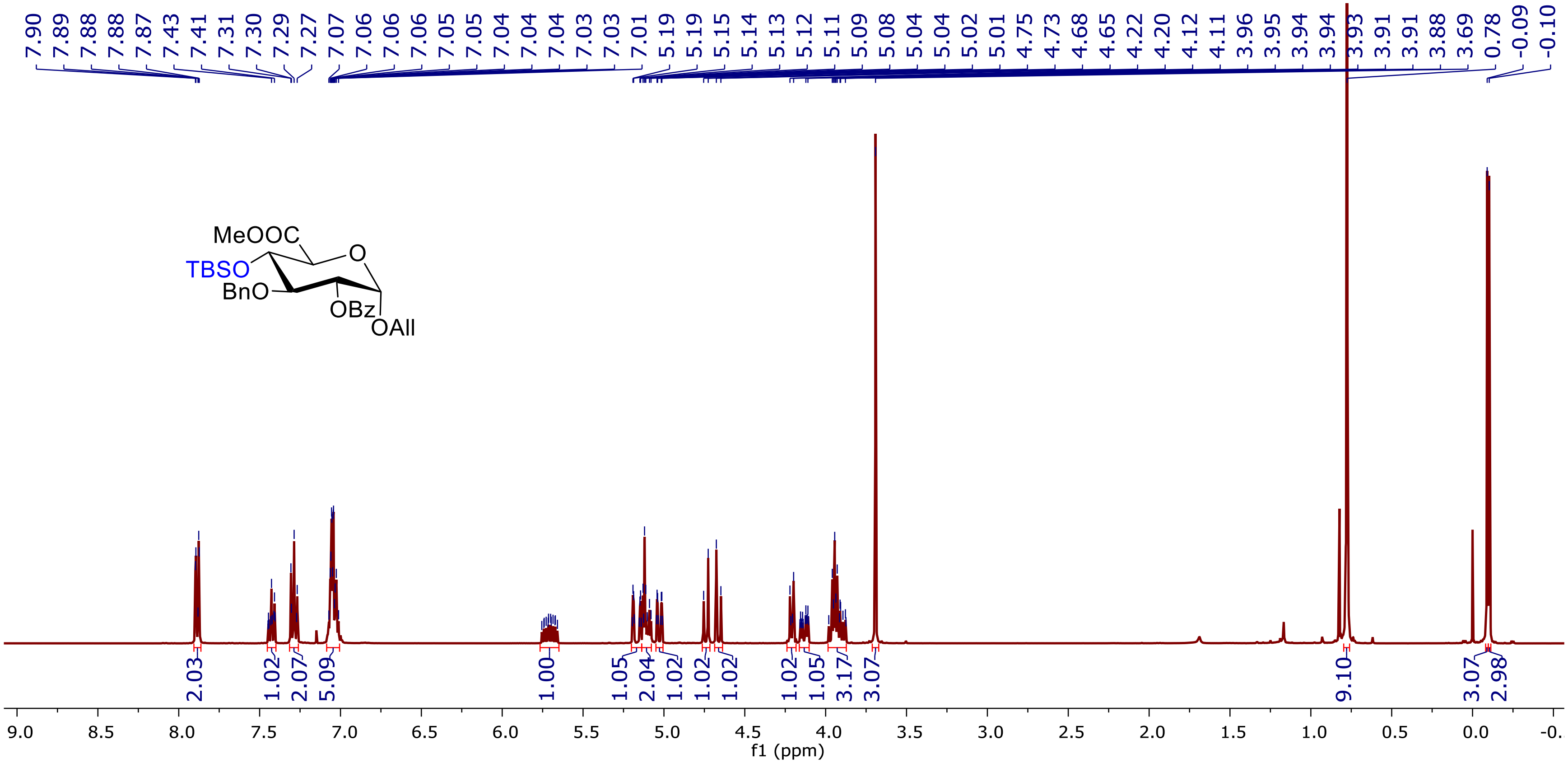

Supplementary Figure S73b. ^{13}C NMR Spectrum (100.67 MHz, $CDCl_3$) of Compound 37c

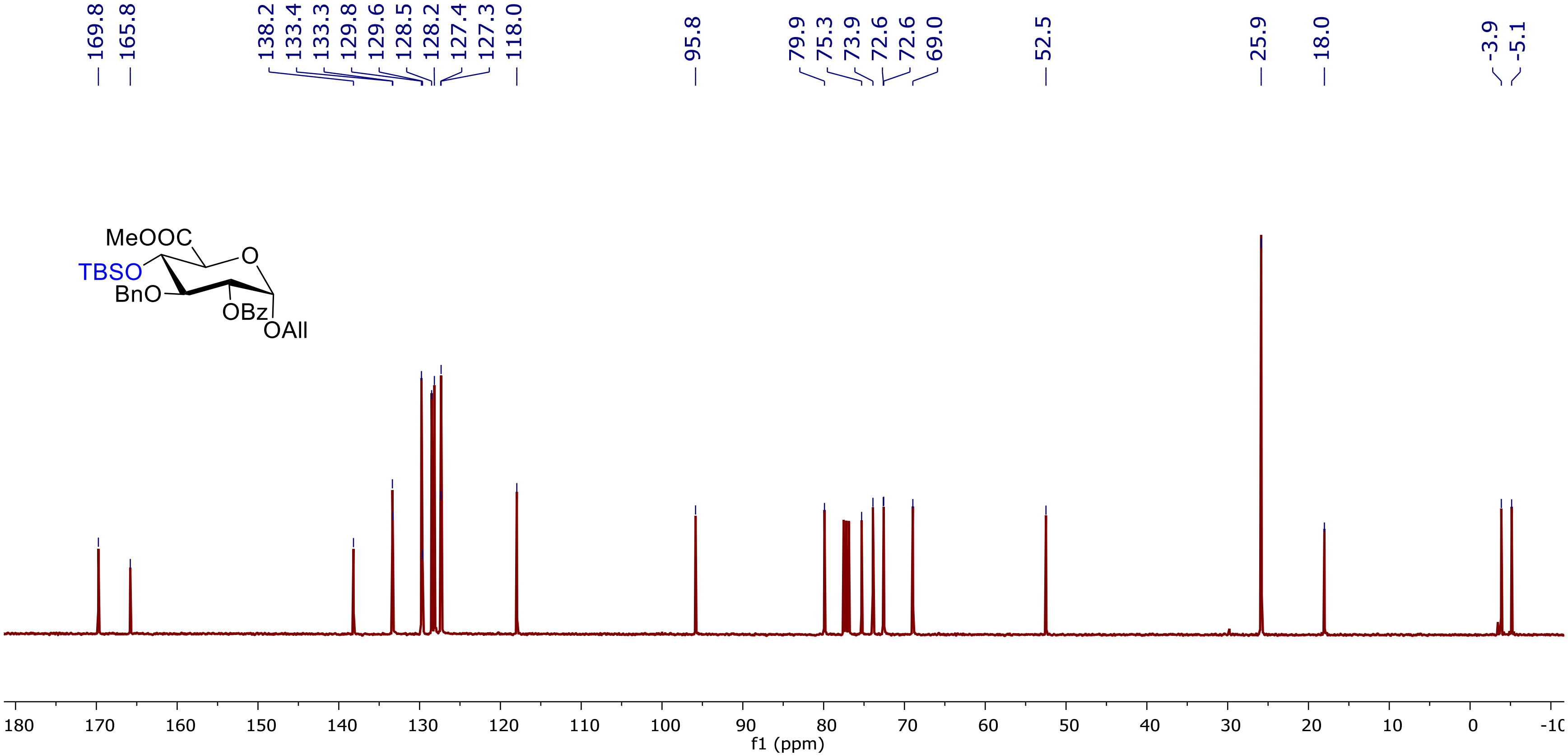

Supplementary Figure S73c. DEPT NMR Spectrum (100.67 MHz, $CDCl_3$) of Compound 37c

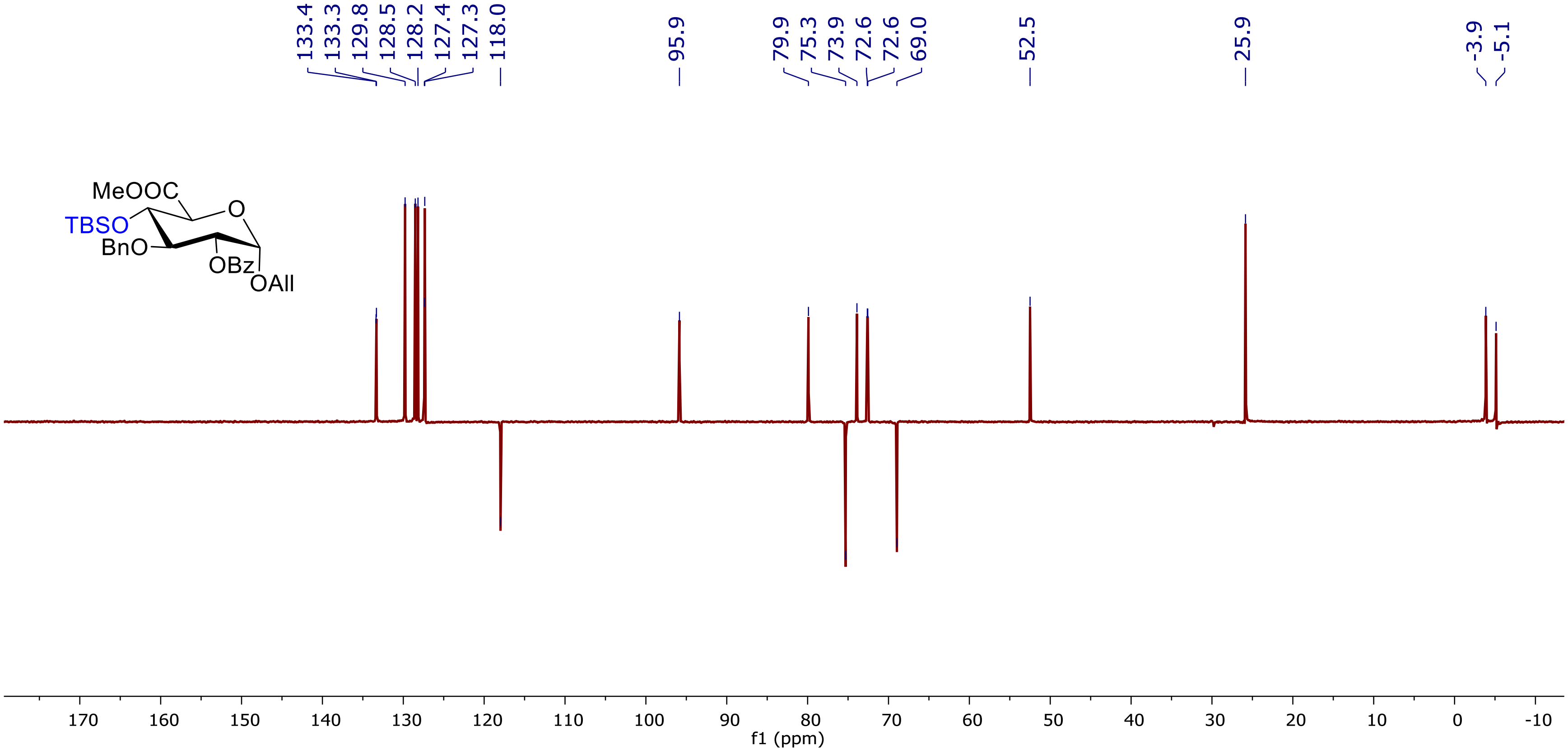

Supplementary Figure S74a. ^{1}H NMR Spectrum (400.31 MHz, $CDCl_3$) of Compound 38a

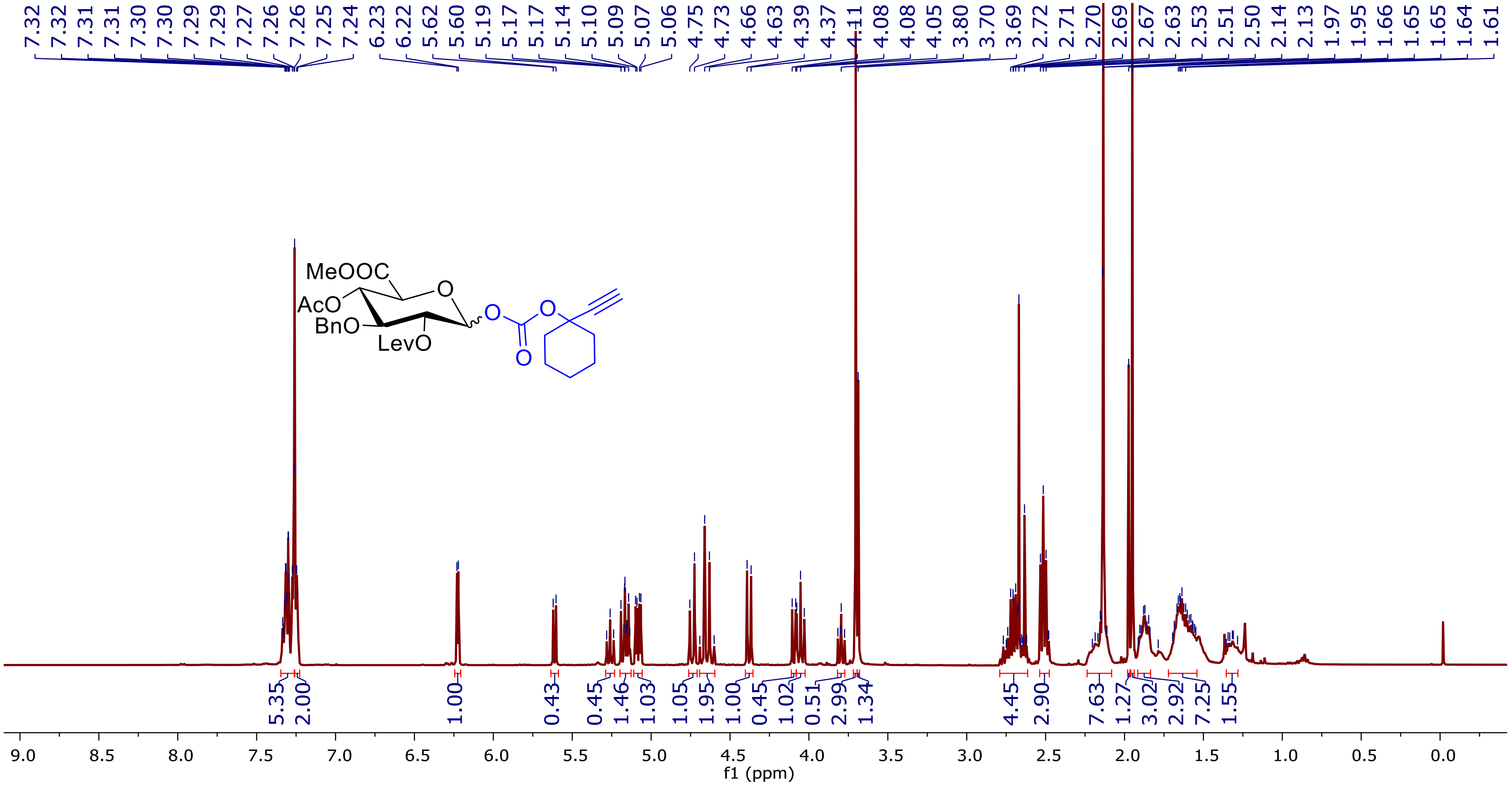

Supplementary Figure S74b. ^{13}C NMR Spectrum (100.67 MHz, $CDCl_3$) of Compound 38a

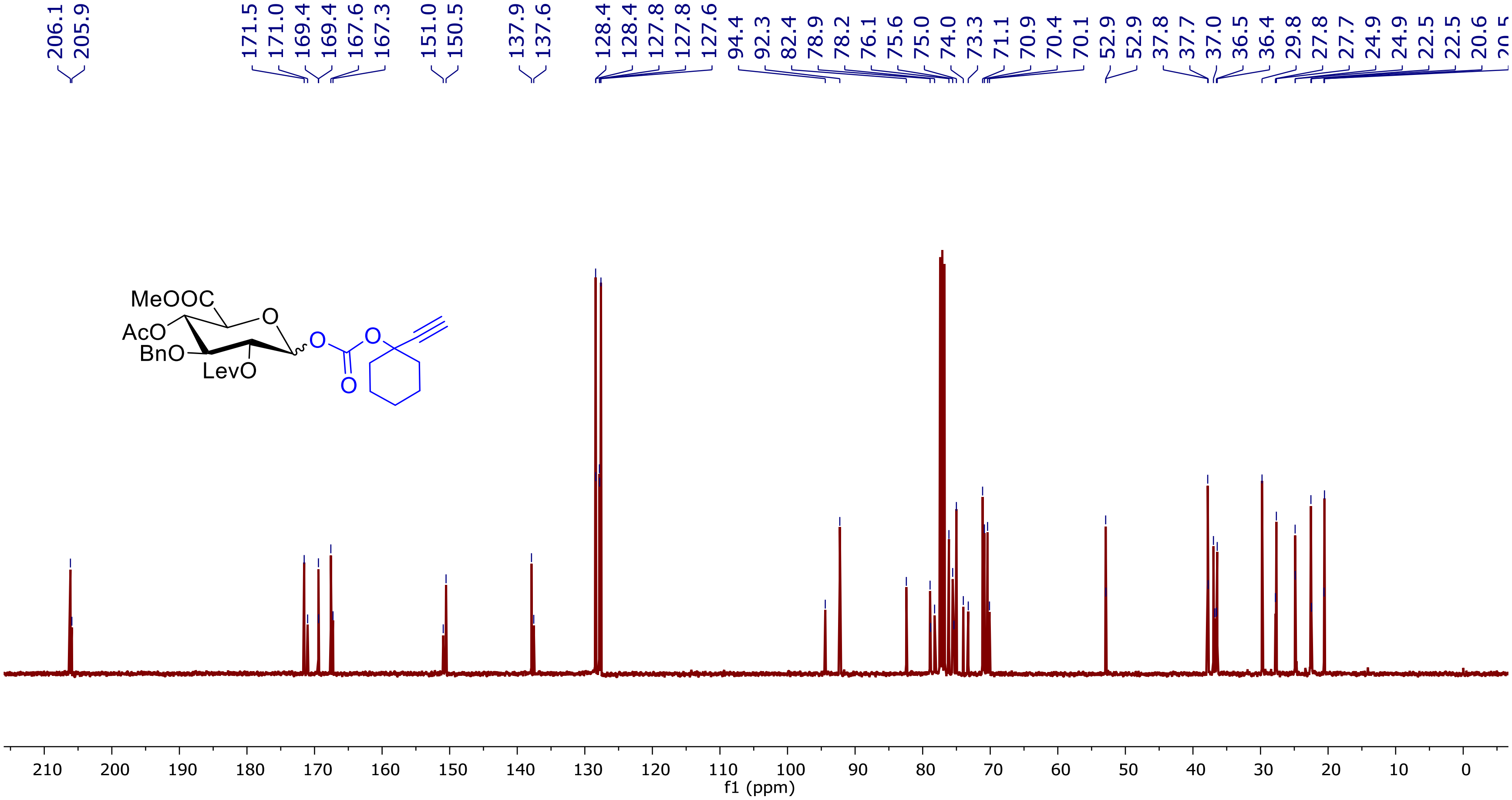

Supplementary Figure S74c. DEPT NMR Spectrum (100.67 MHz, CDCl₃) of Compound 38a

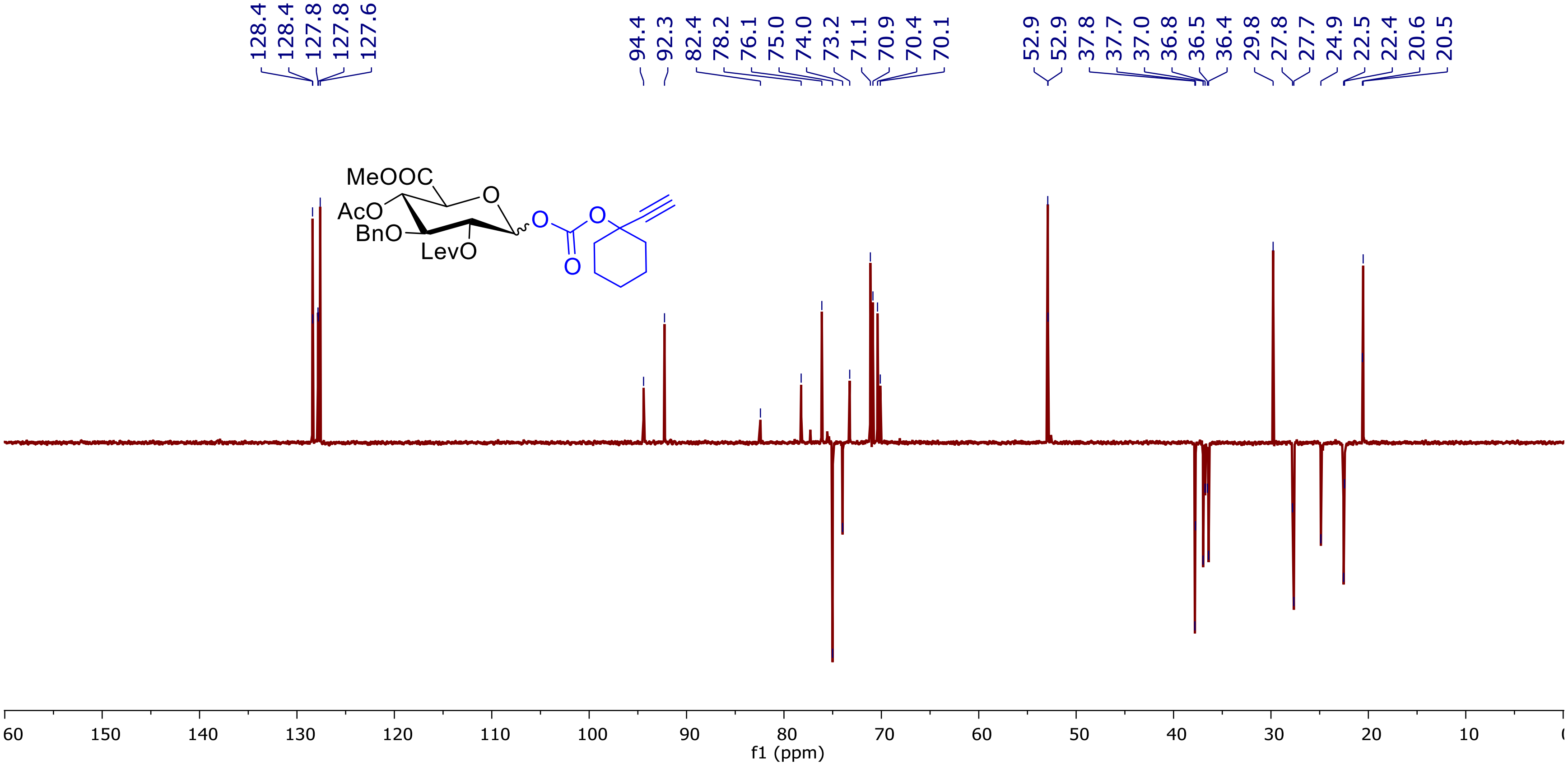

Supplementary Figure S75a. ^{1}H NMR Spectrum (400.31 MHz, $CDCl_3$) of Compound 38b

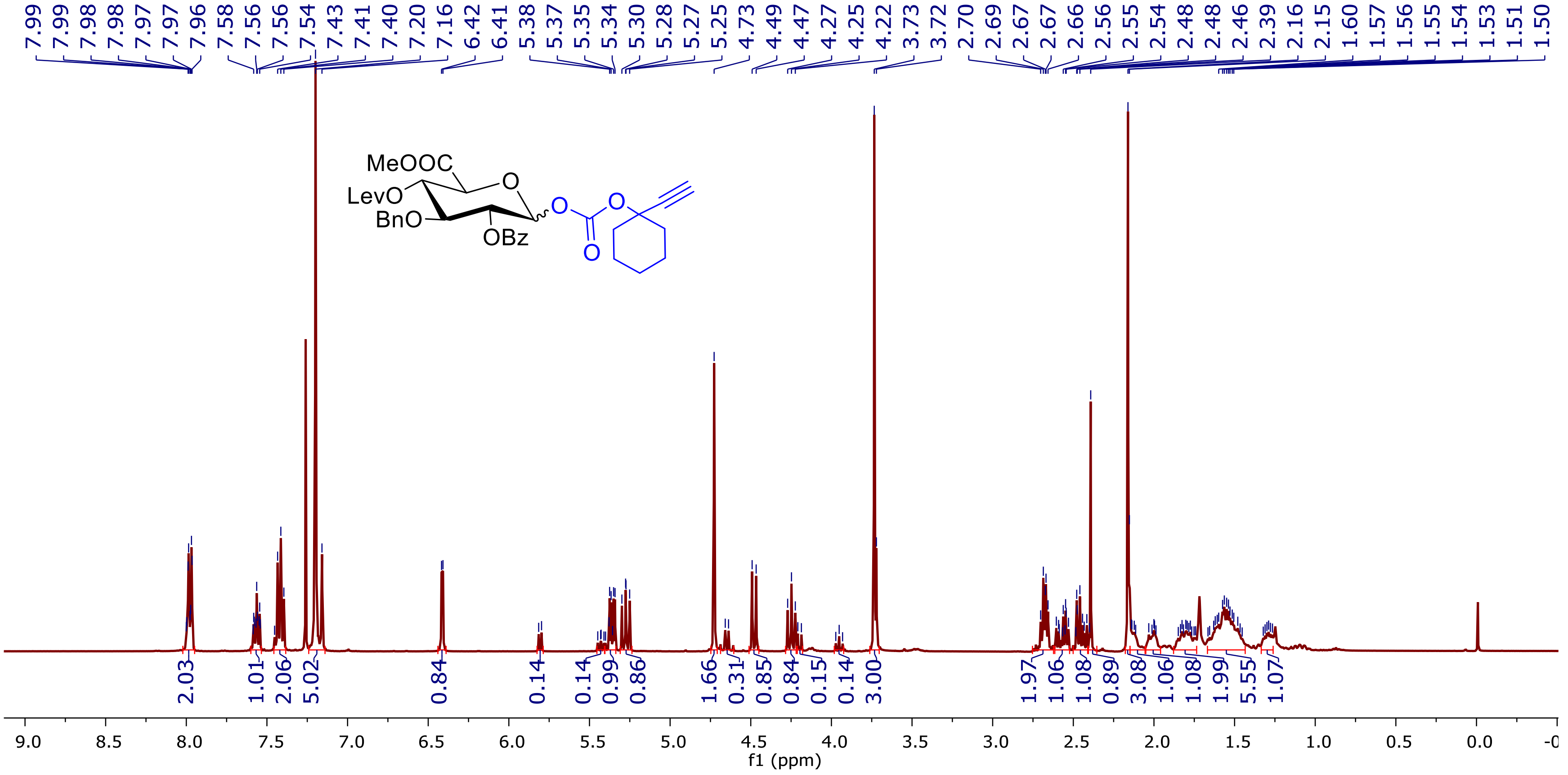

Supplementary Figure S75b. ^{13}C NMR Spectrum (100.67 MHz, $CDCl_3$) of Compound 38b

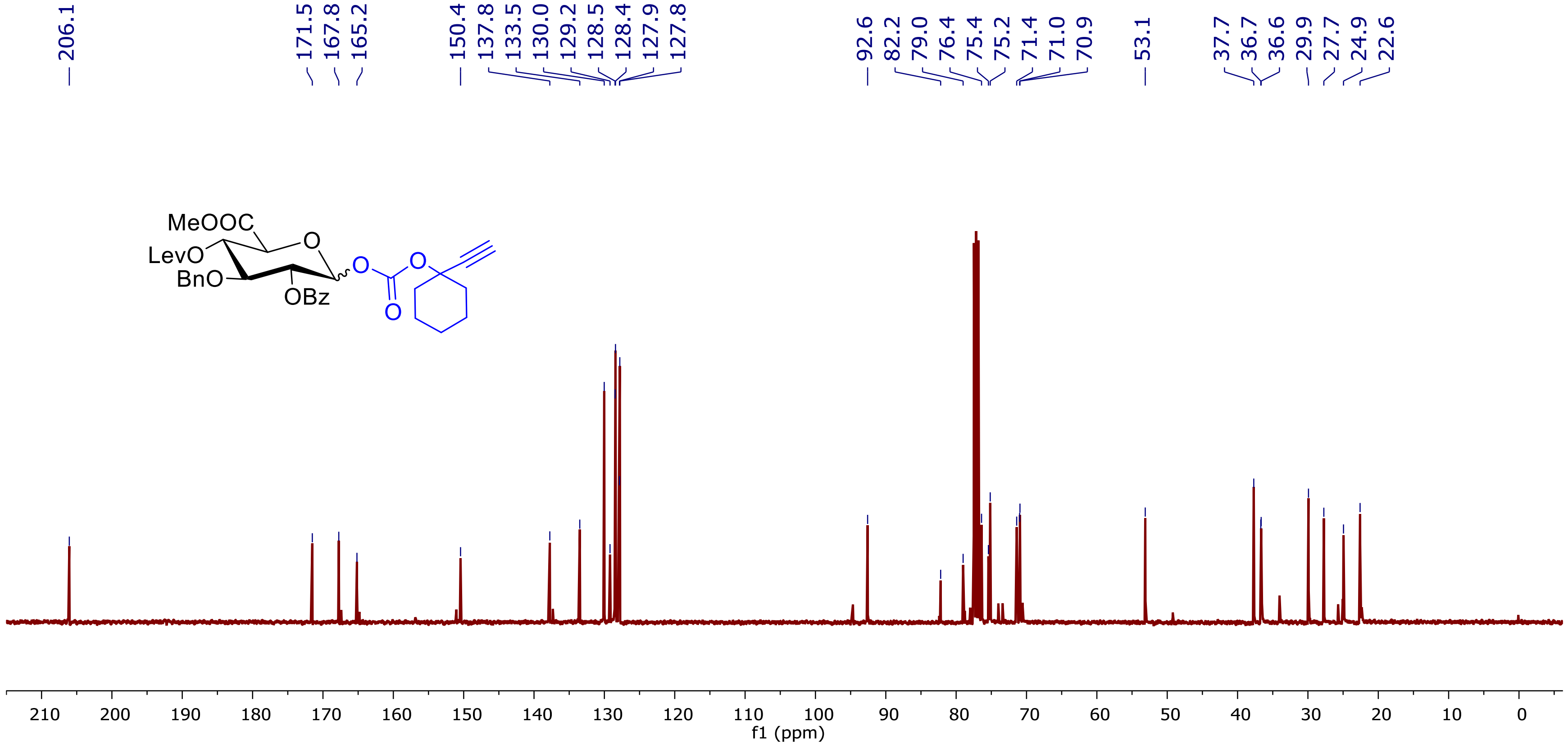

Supplementary Figure S75c. DEPT NMR Spectrum (100.67 MHz, $CDCl_3$) of Compound 38b

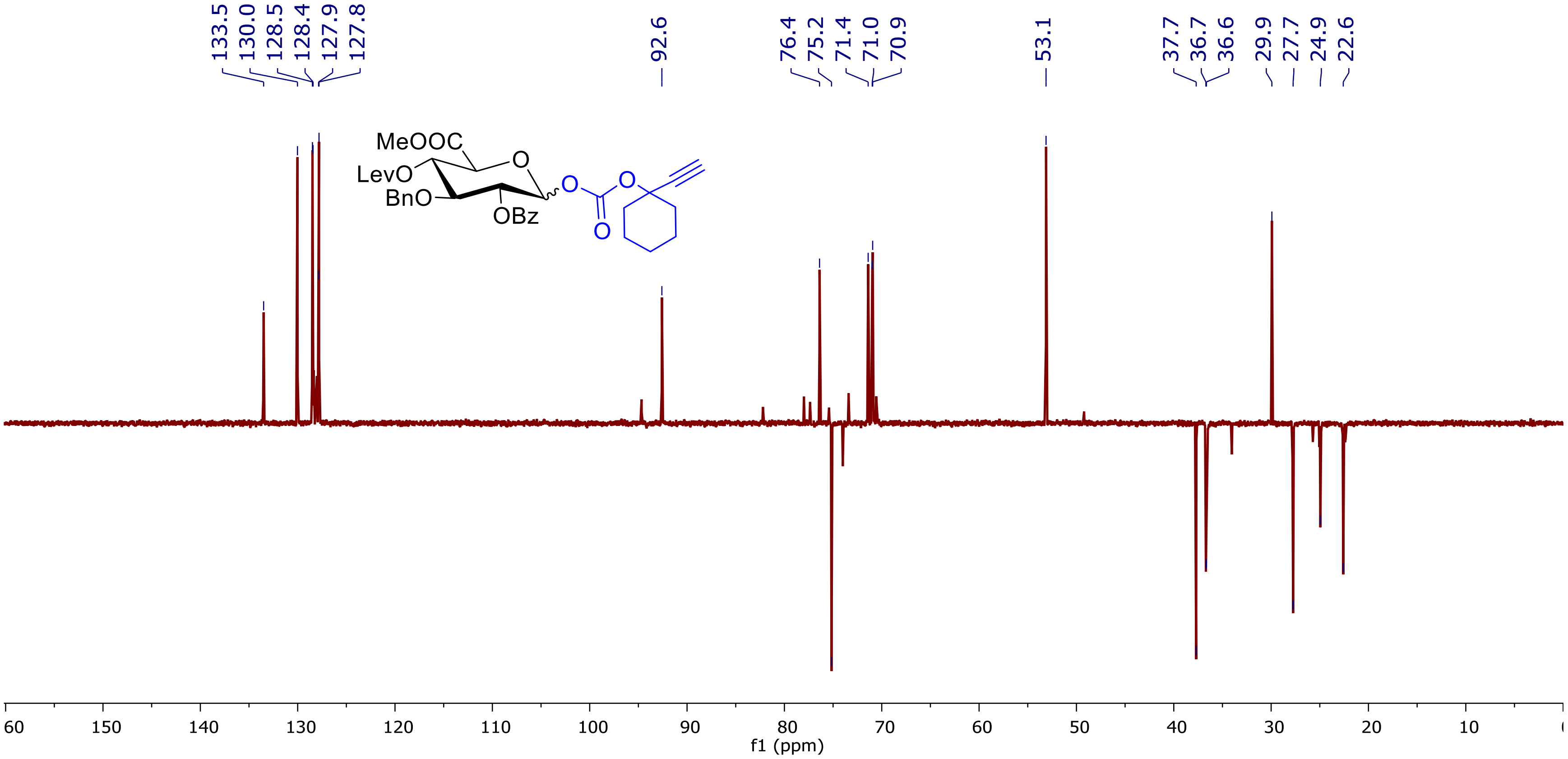

Supplementary Figure S76a. ^{1}H NMR Spectrum (400.31 MHz, $CDCl_3$) of Compound 38c Hemiacetal intermediate

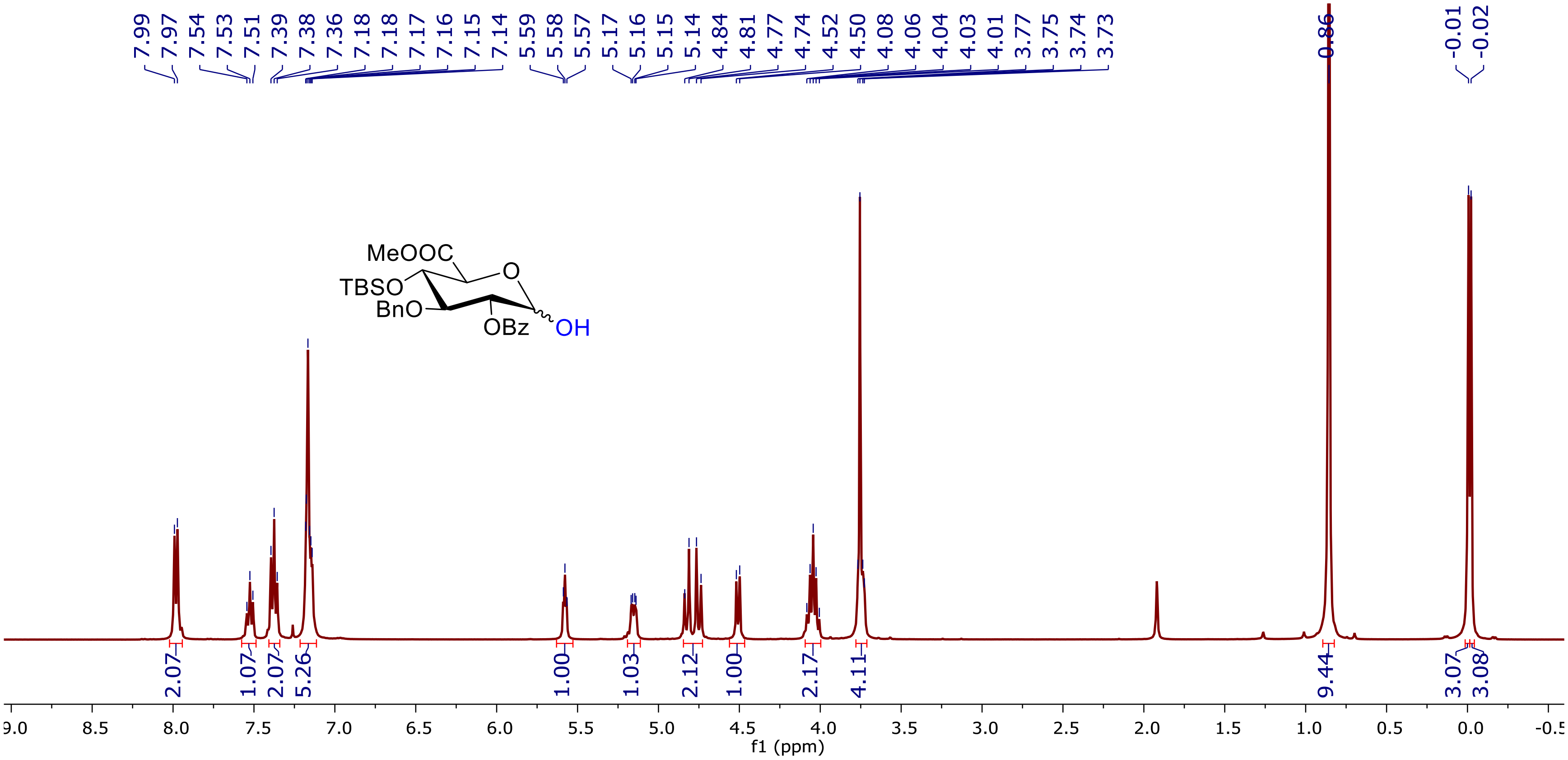

Supplementary Figure S76b. ^{13}C NMR Spectrum (100.67 MHz, $CDCl_3$) of Compound 38c Hemiacetal intermediate

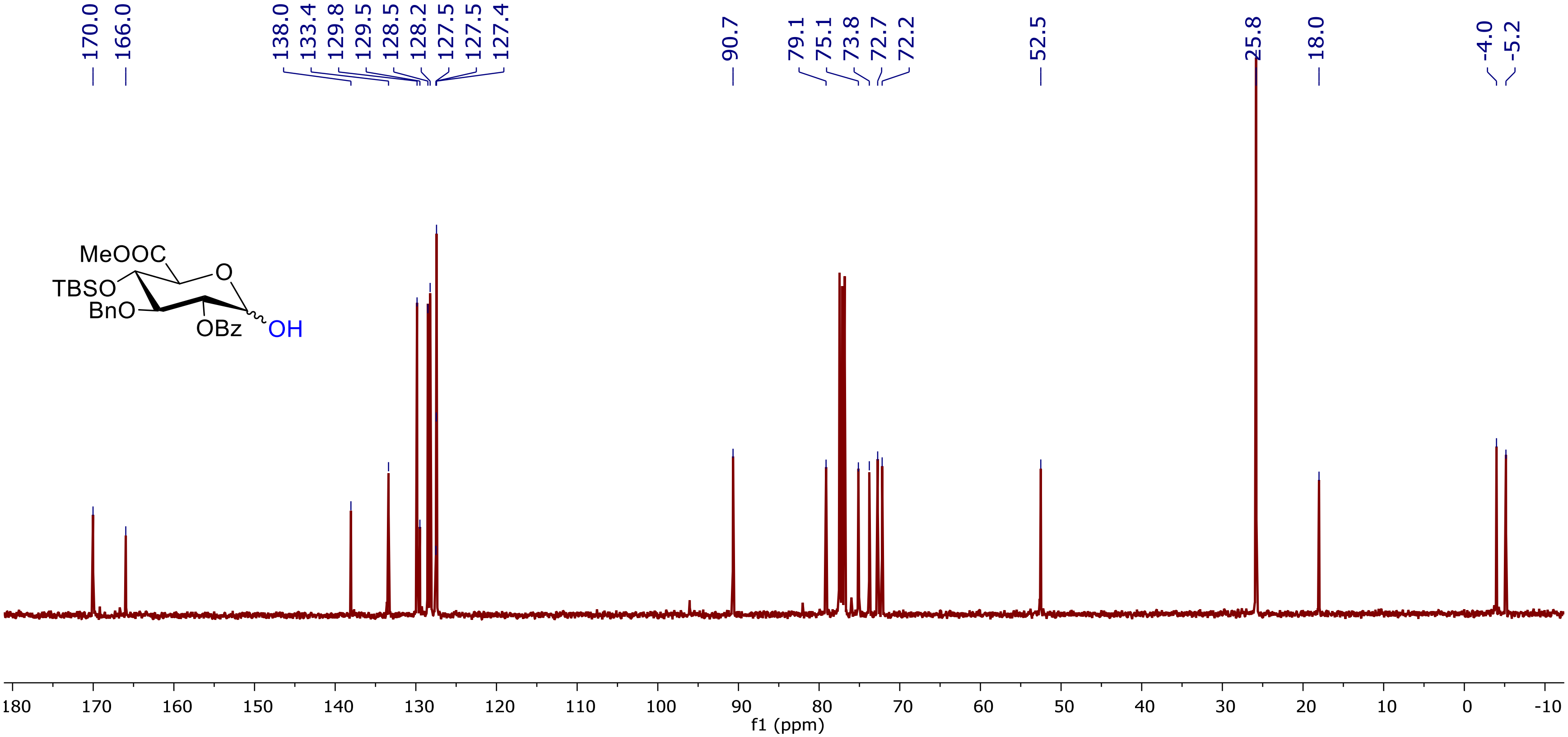

Supplementary Figure S76c. DEPT NMR Spectrum (100.67 MHz, $CDCl_3$) of Compound 38c Hemiacetal intermediate

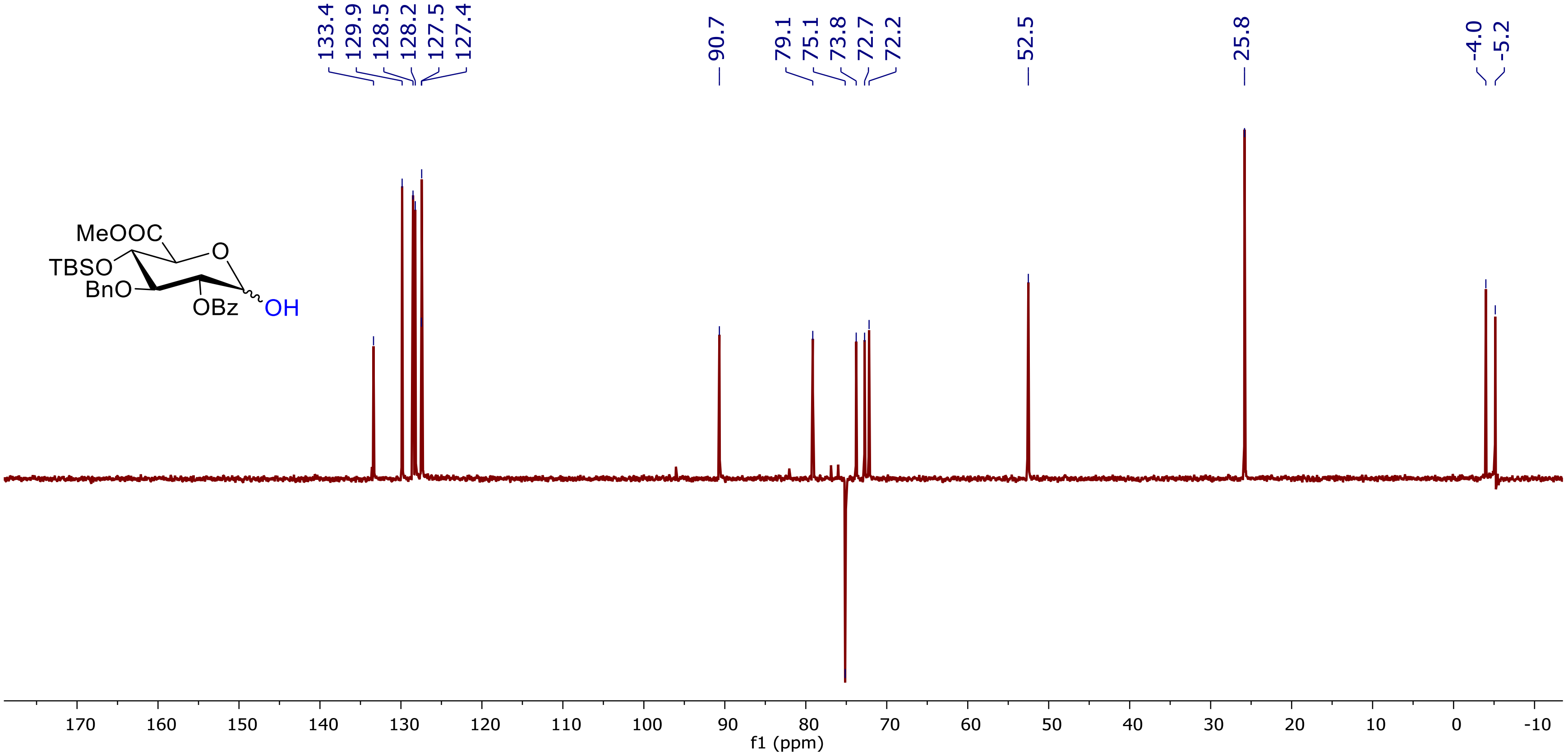

Supplementary Figure S77a. ¹H NMR Spectrum (400.31 MHz, $CDCl_3$) of Compound 38c

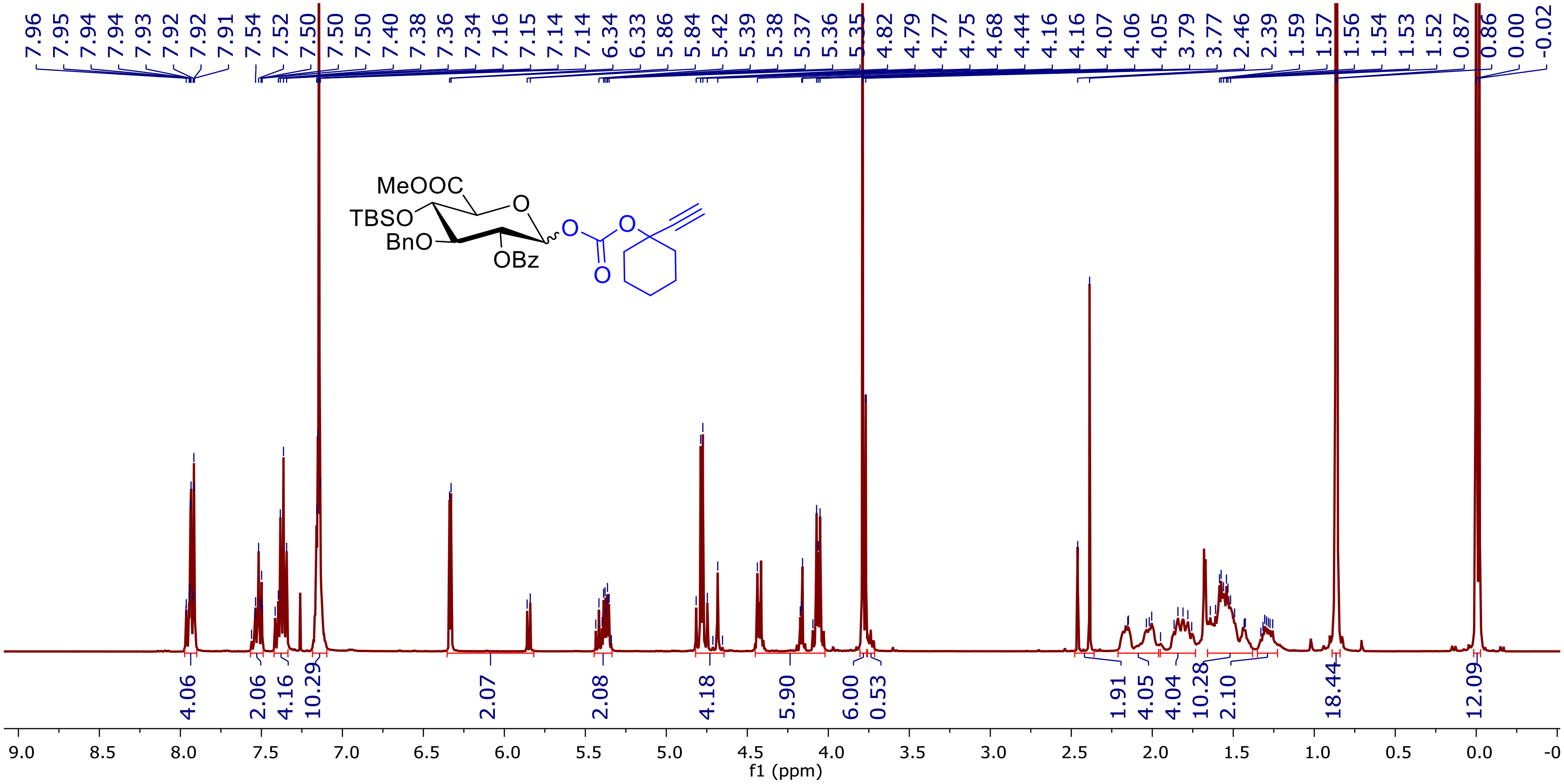

Supplementary Figure S77b. ^{13}C NMR Spectrum (100.67 MHz, $CDCl_3$) of Compound 38c

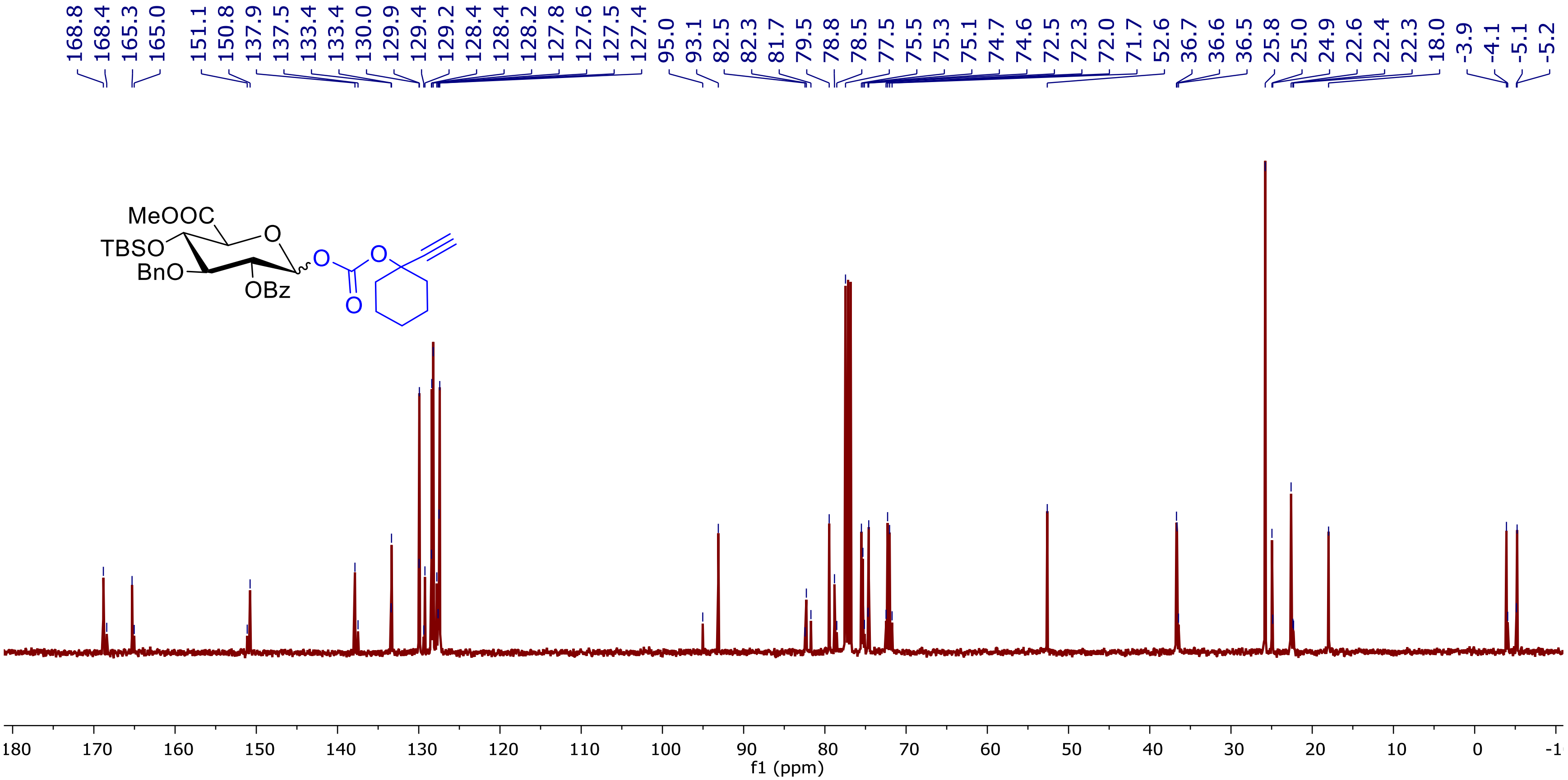

Supplementary Figure S77c. DEPT NMR Spectrum (100.67 MHz, $CDCl_3$) of Compound 38c

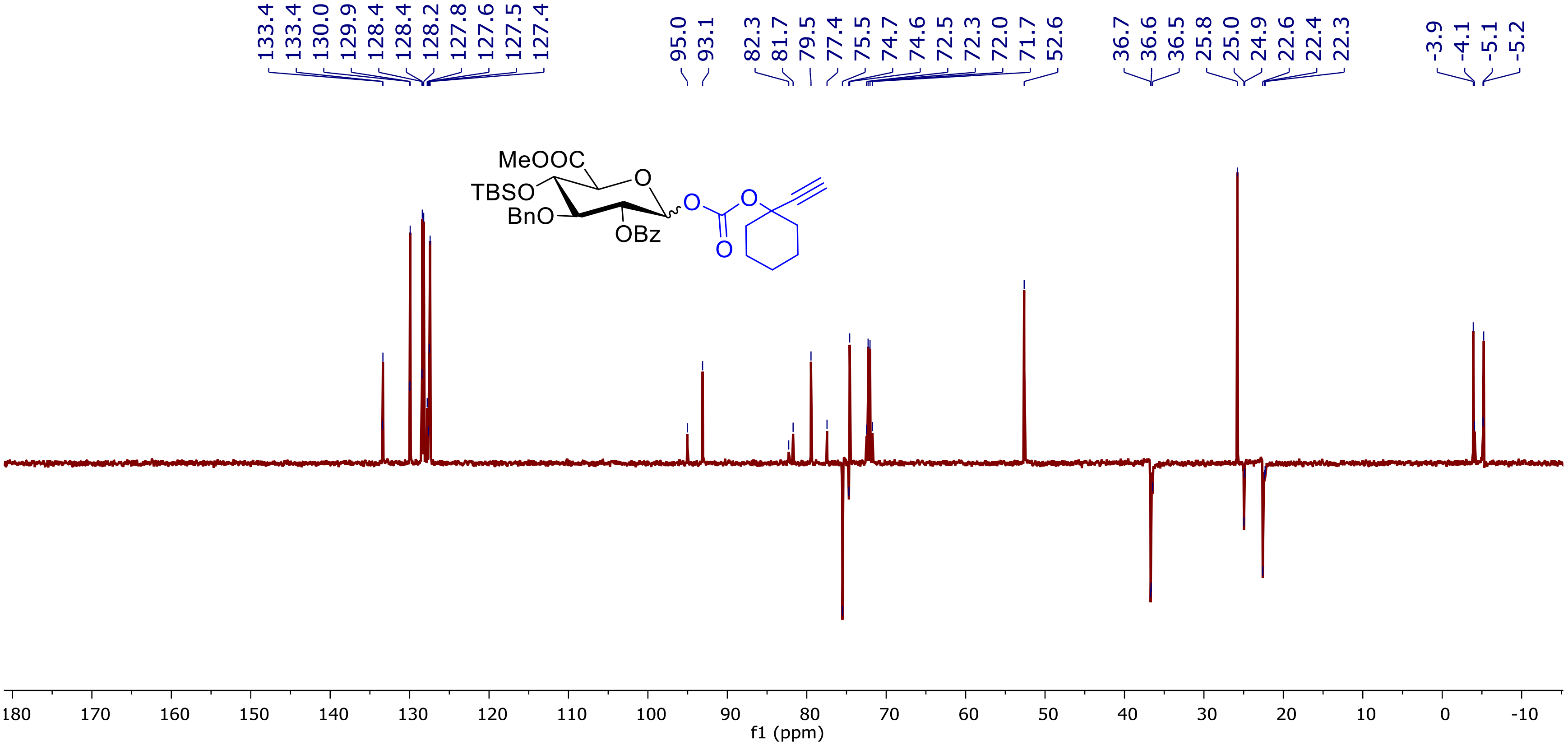

Supplementary Figure S78a. ¹H NMR Spectrum (400.31 MHz, CDCl₃) of Compound 39a

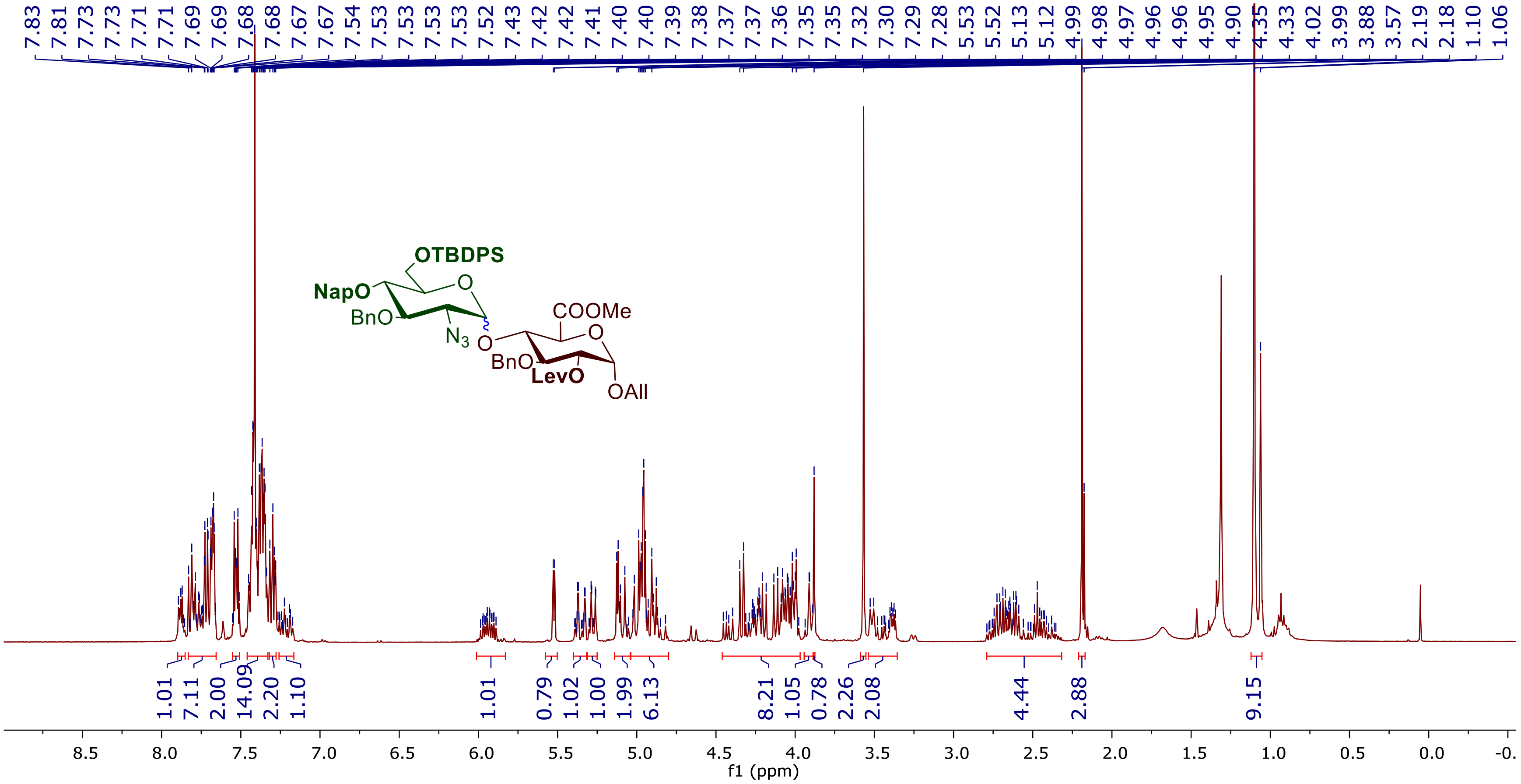

Supplementary Figure S78b. ^{13}C NMR Spectrum (100.67 MHz, $CDCl_3$) of Compound 39a

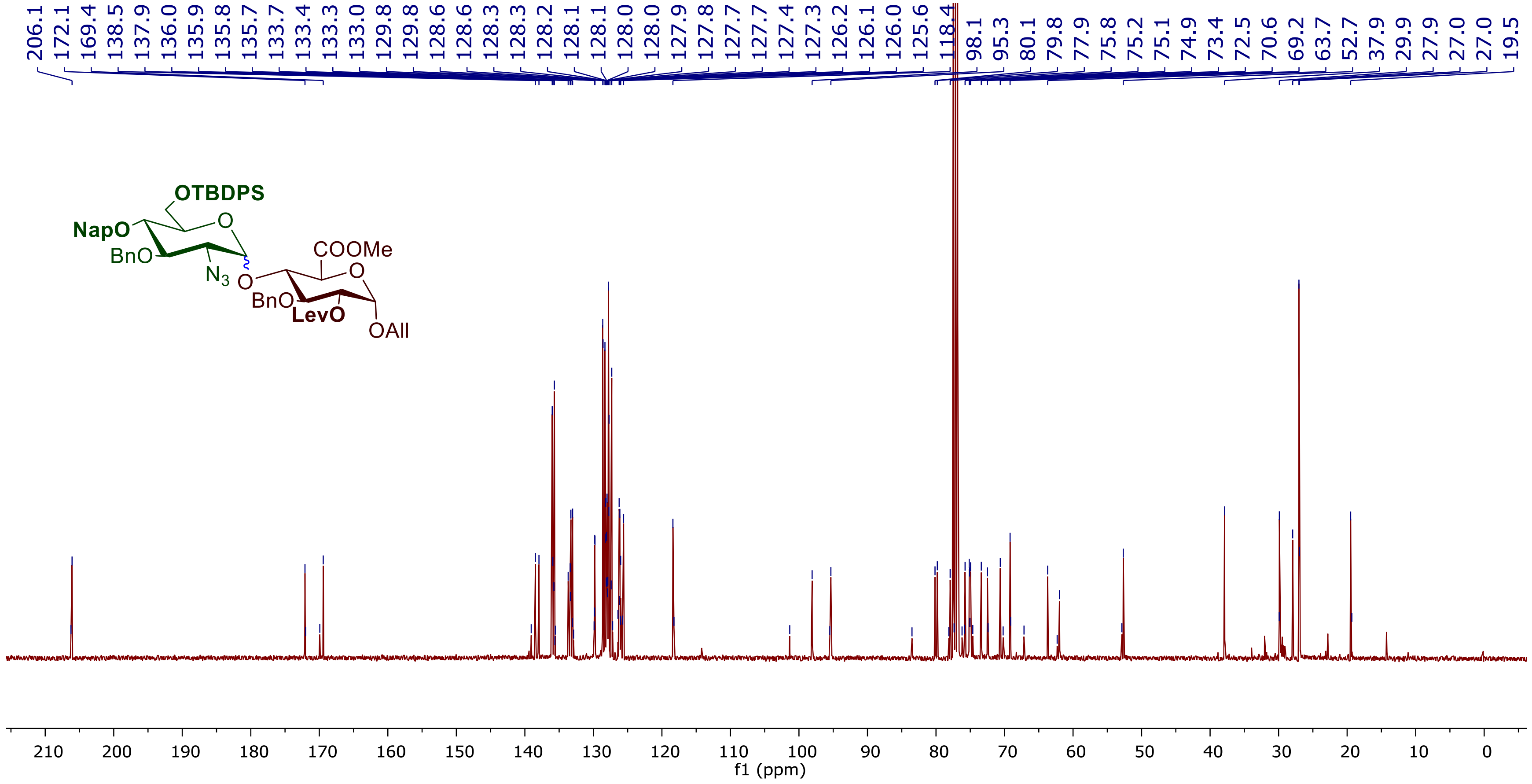

Supplementary Figure S78c. DEPT NMR Spectrum (100.67 MHz, $CDCl_3$) of Compound 39a

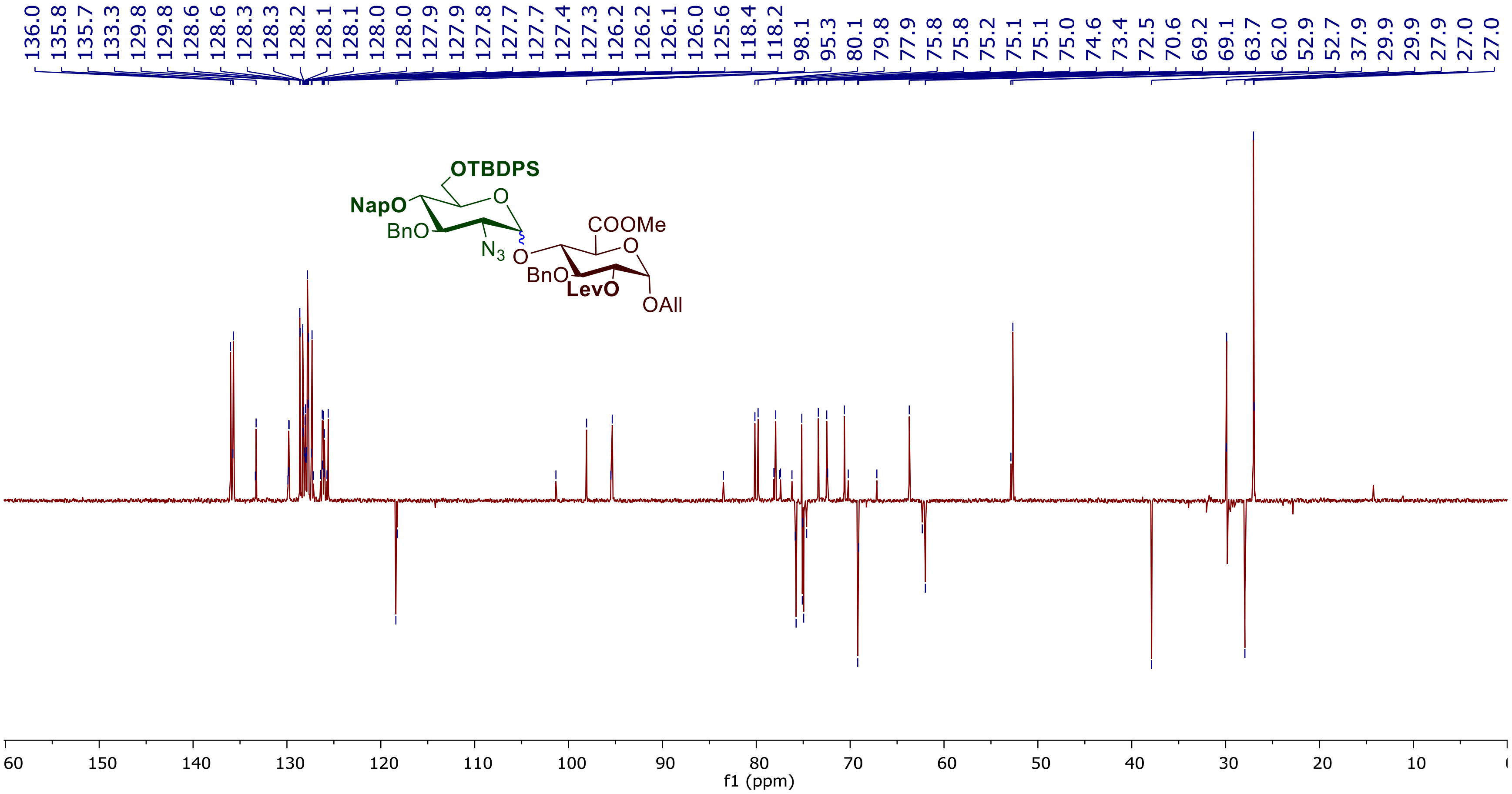

Supplementary Figure S79a. ^{1}H NMR Spectrum (400.31 MHz, $CDCl_3$) of Compound 39b

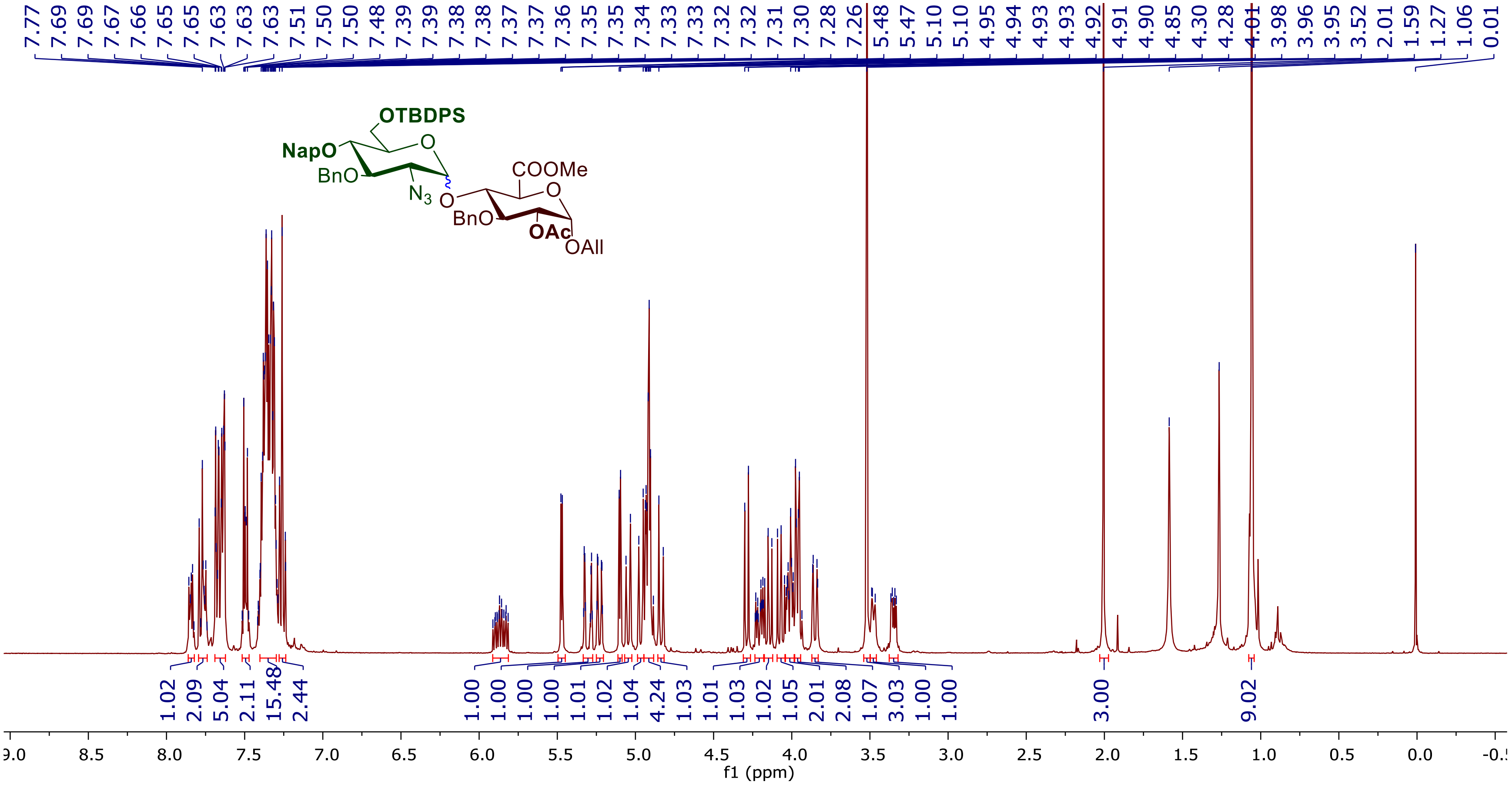

Supplementary Figure S79b. ^{13}C NMR Spectrum (100.67 MHz, $CDCl_3$) of Compound 39b

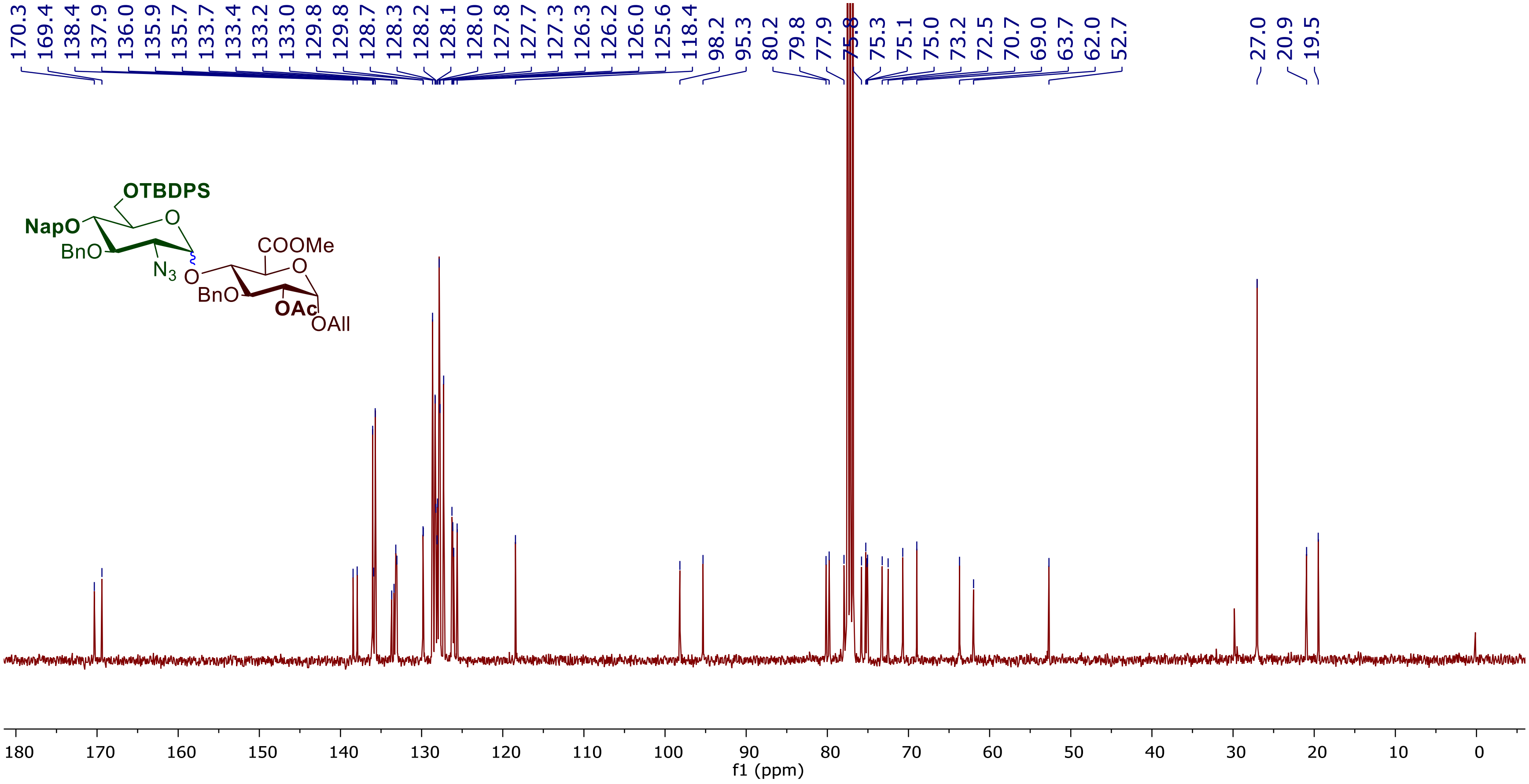

Supplementary Figure S79c. DEPT NMR Spectrum (100.67 MHz, $CDCl_3$) of Compound 39b

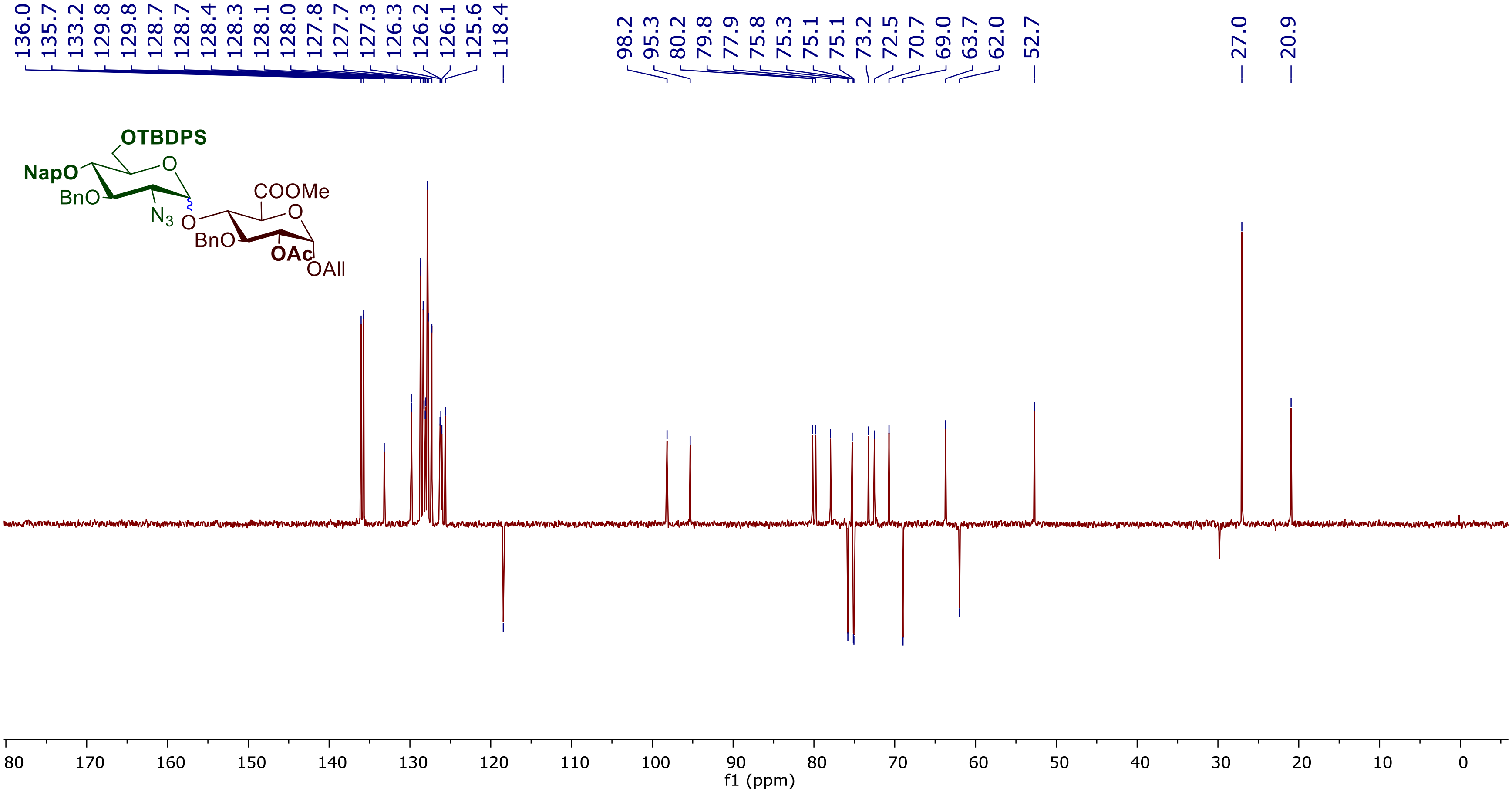

Supplementary Figure S80a. ¹H NMR Spectrum (400.31 MHz, CDCl₃) of Compound 39c

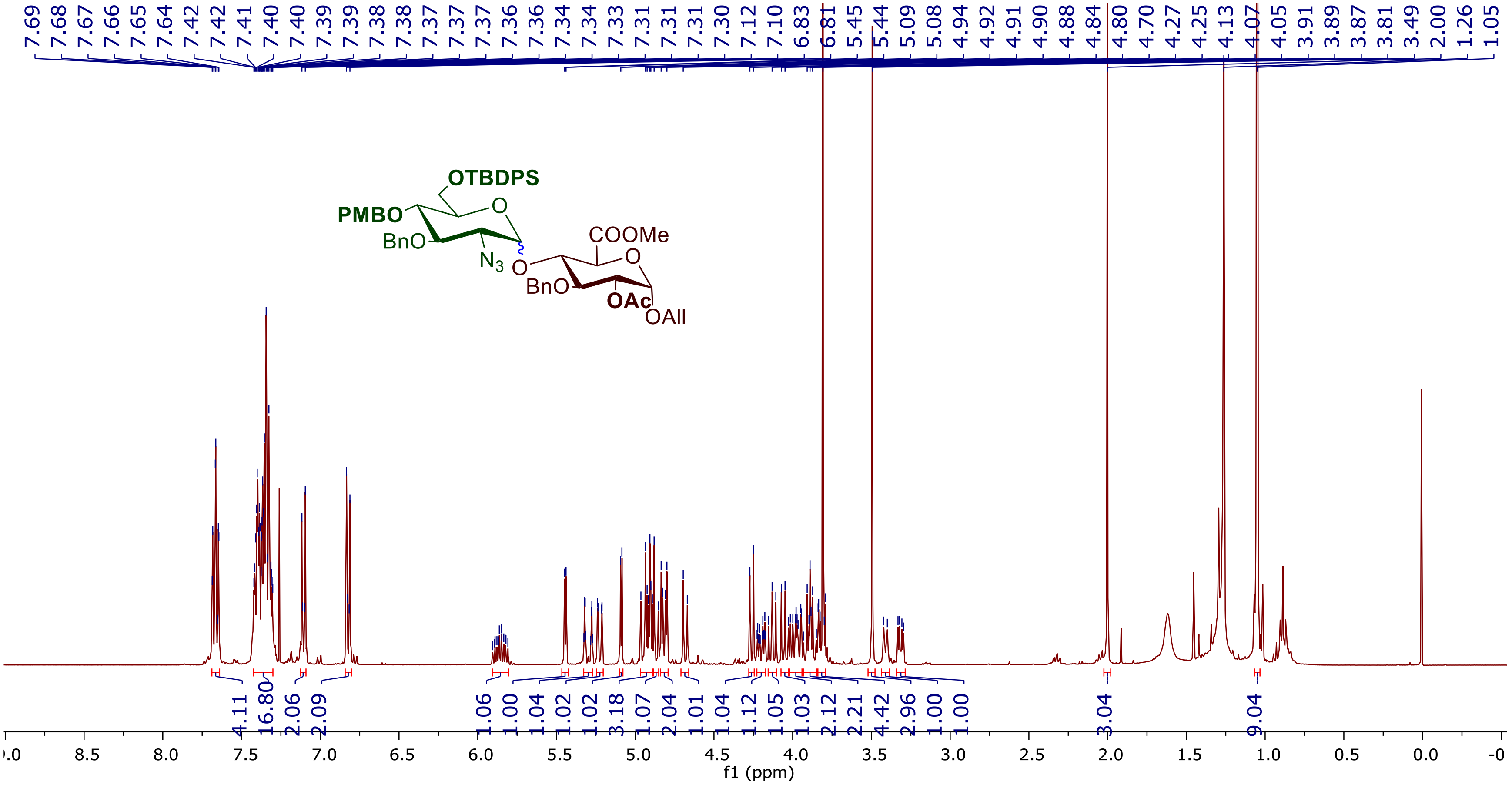

Supplementary Figure S80b. ^{13}C NMR Spectrum (100.67 MHz, $CDCl_3$) of Compound 39c

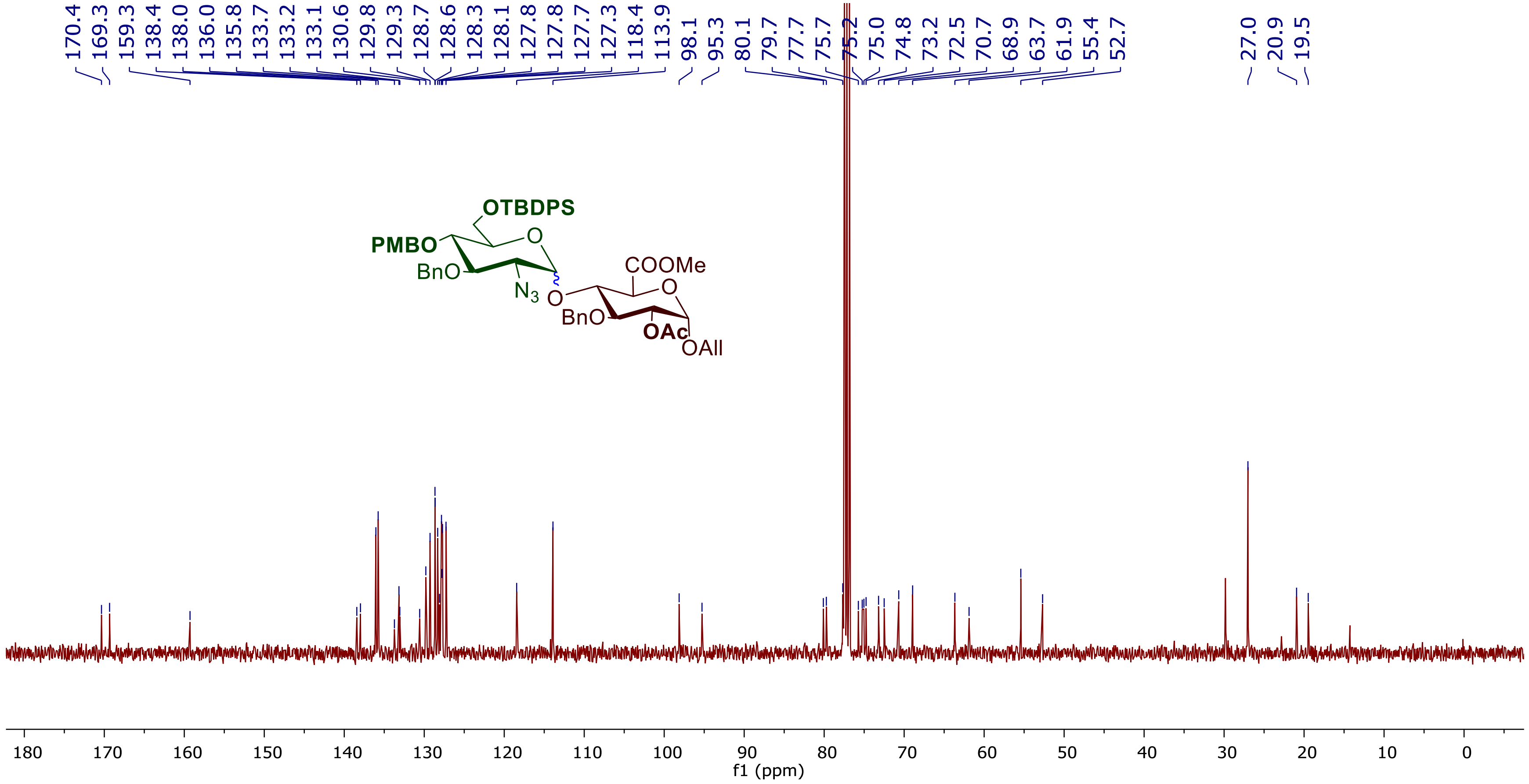

Supplementary Figure S80c. DEPT NMR Spectrum (100.67 MHz, $CDCl_3$) of Compound 39c

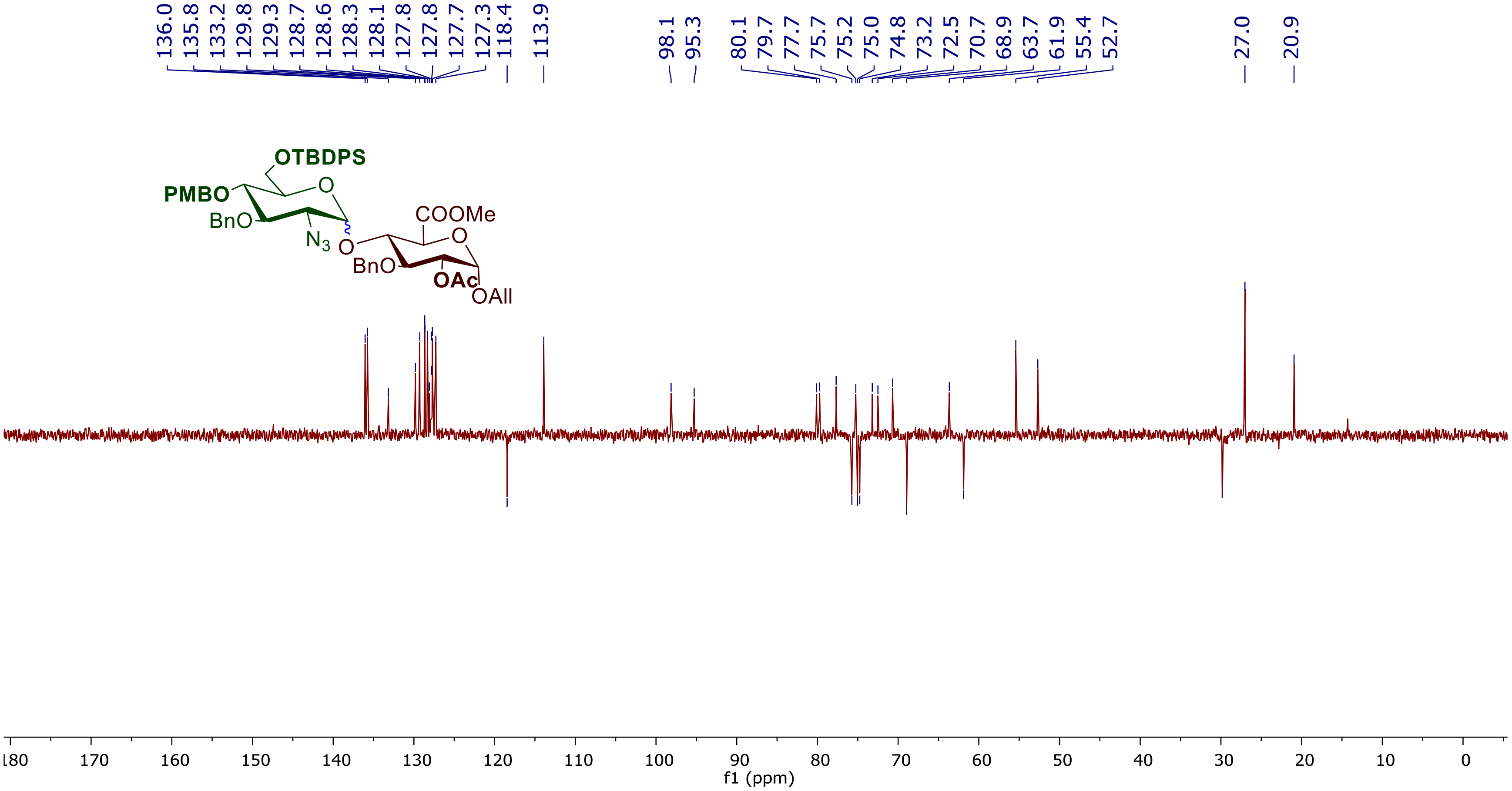

Supplementary Figure S81a. ^{1}H NMR Spectrum (400.31 MHz, $CDCl_3$) of Compound 39d

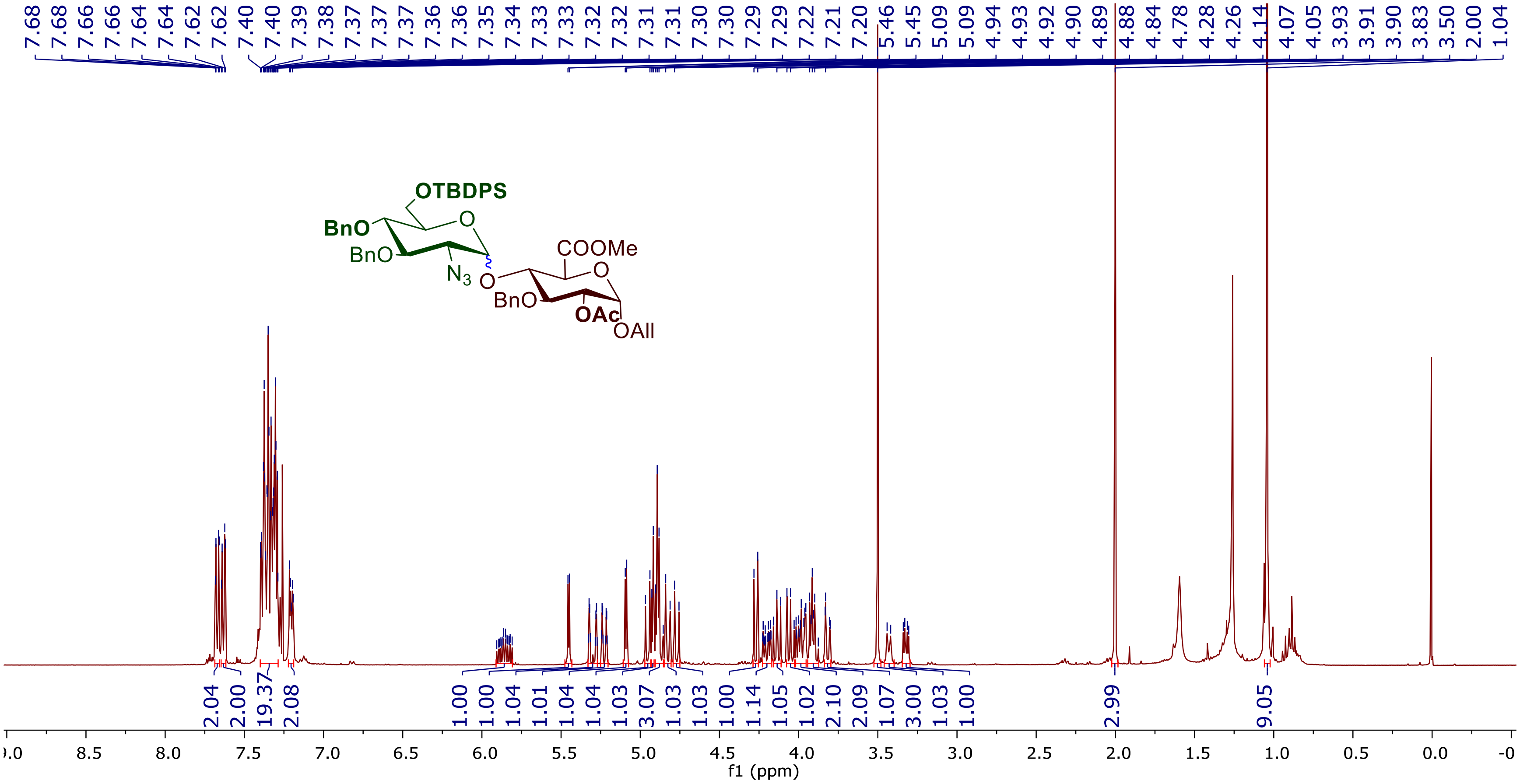

Supplementary Figure S81b. ^{13}C NMR Spectrum (100.67 MHz, $CDCl_3$) of Compound 39d

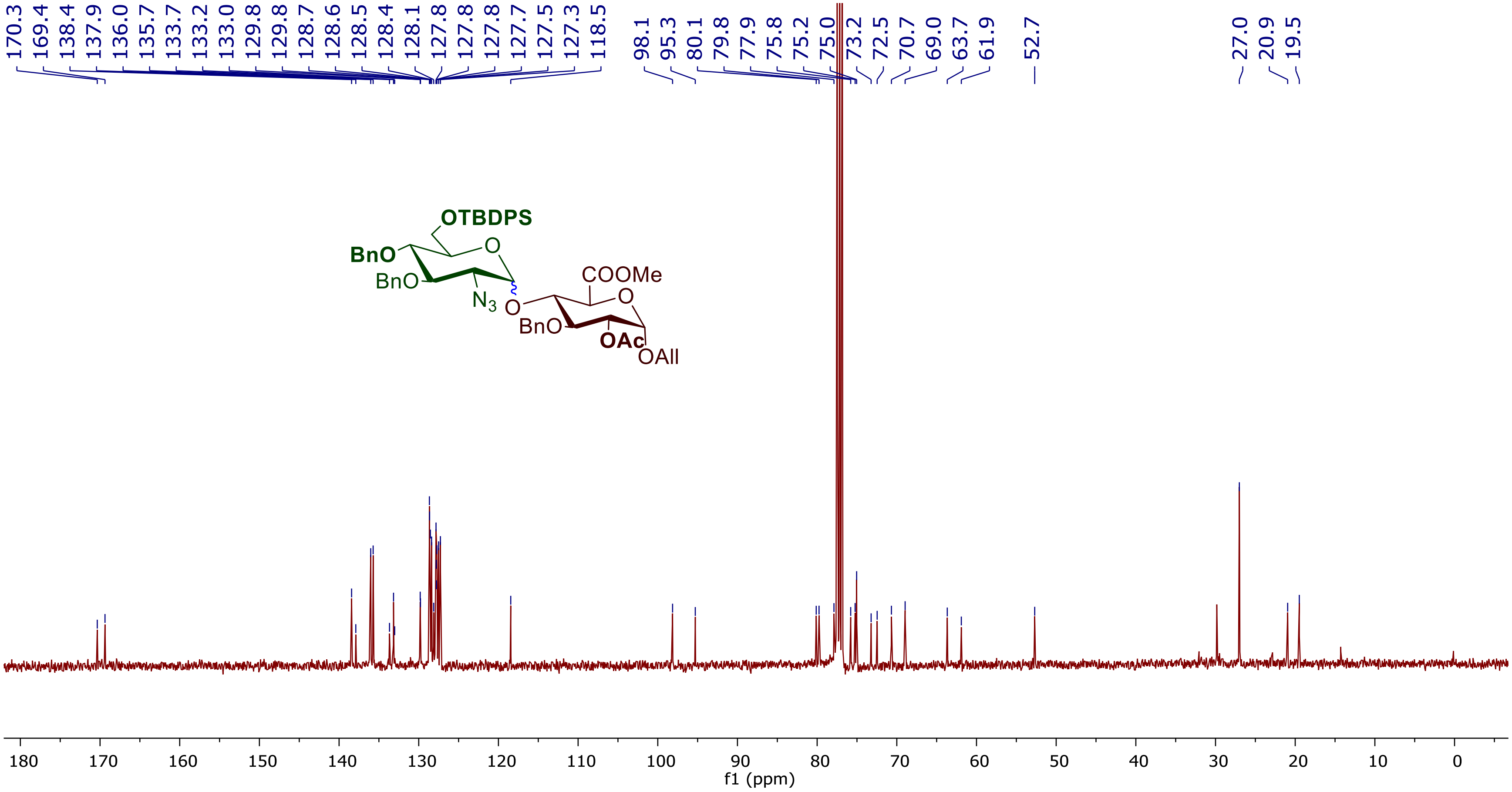

Supplementary Figure S81c. DEPT NMR Spectrum (100.67 MHz, $CDCl_3$) of Compound 39d

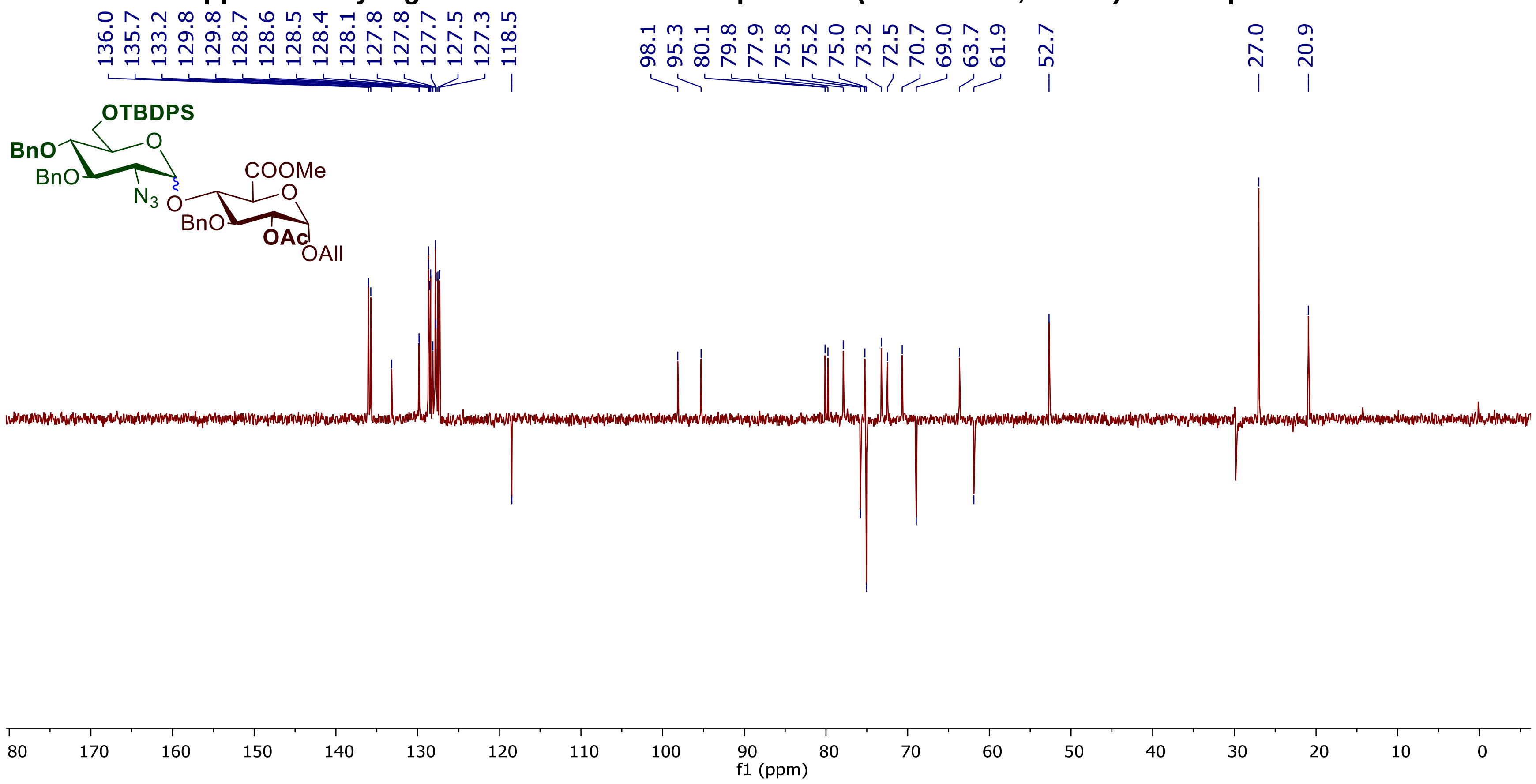

Supplementary Figure S82a. ^{1}H NMR Spectrum (400.31 MHz, $CDCl_3$) of Compound 39e

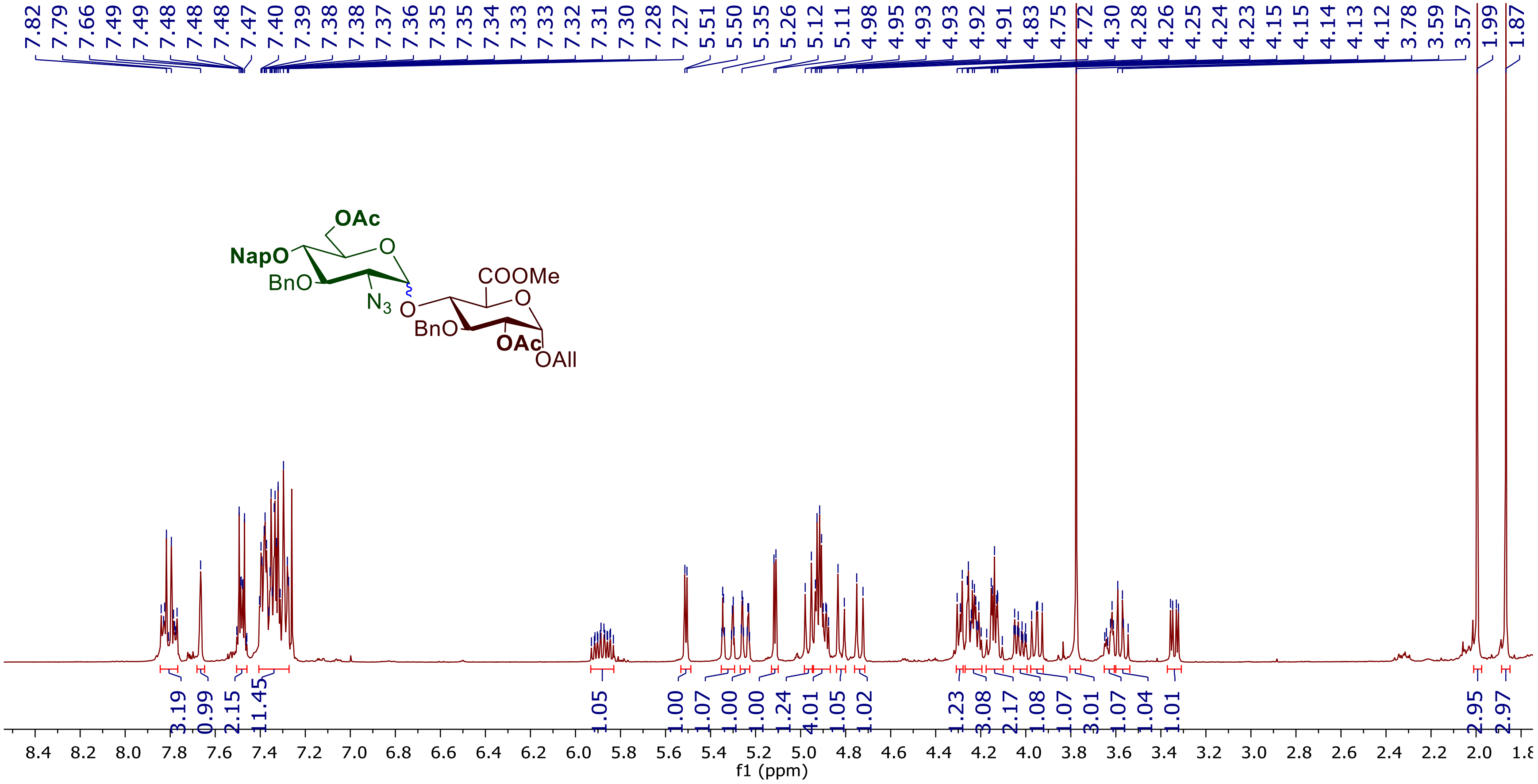

Supplementary Figure S82b. ^{13}C NMR Spectrum (100.67 MHz, $CDCl_3$) of Compound 39e

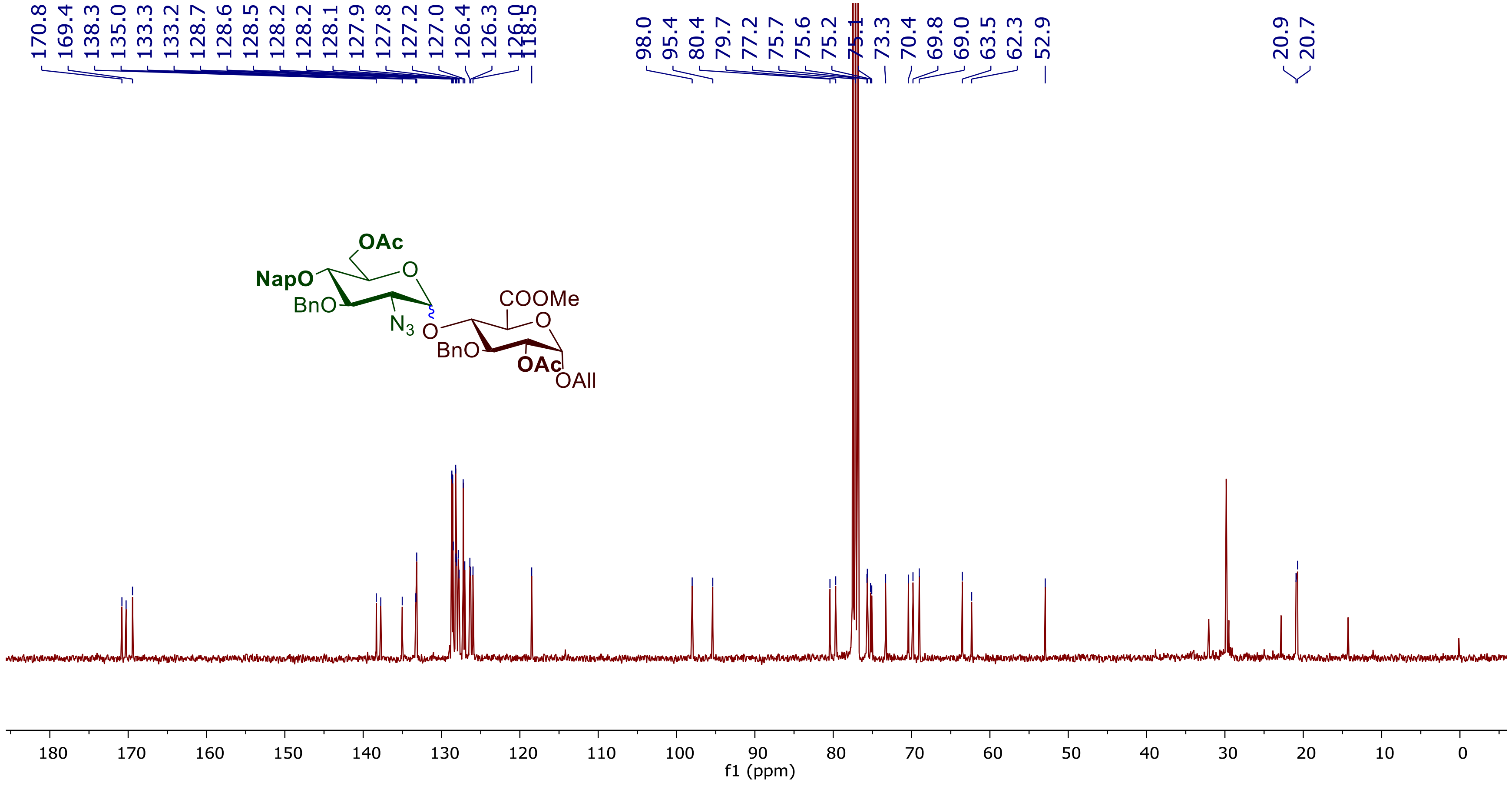

Supplementary Figure S82c. DEPT NMR Spectrum (100.67 MHz, $CDCl_3$) of Compound 39e

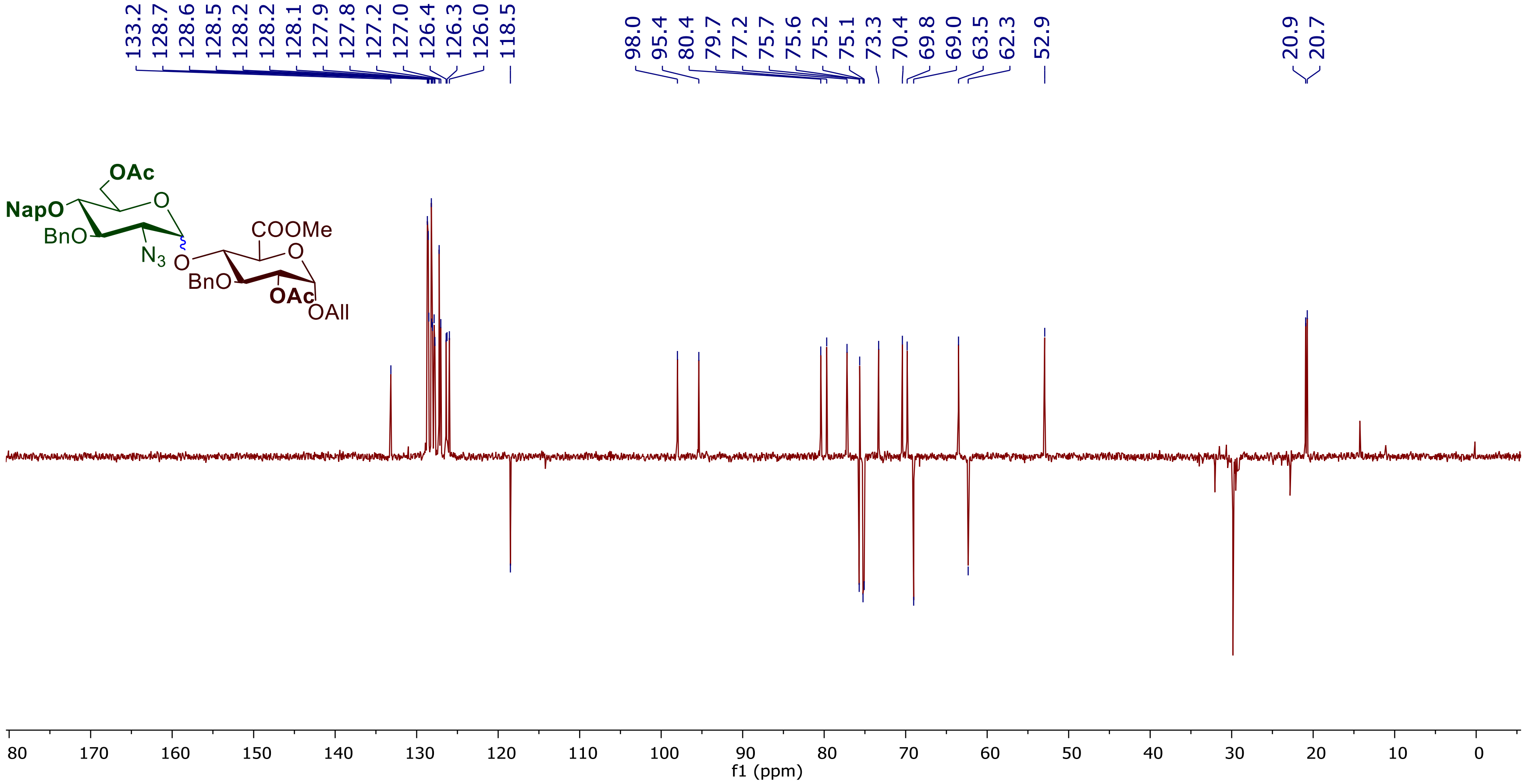

Supplementary Figure S83a. 1H NMR Spectrum (400.31 MHz, CDCl3) of Compound 39f

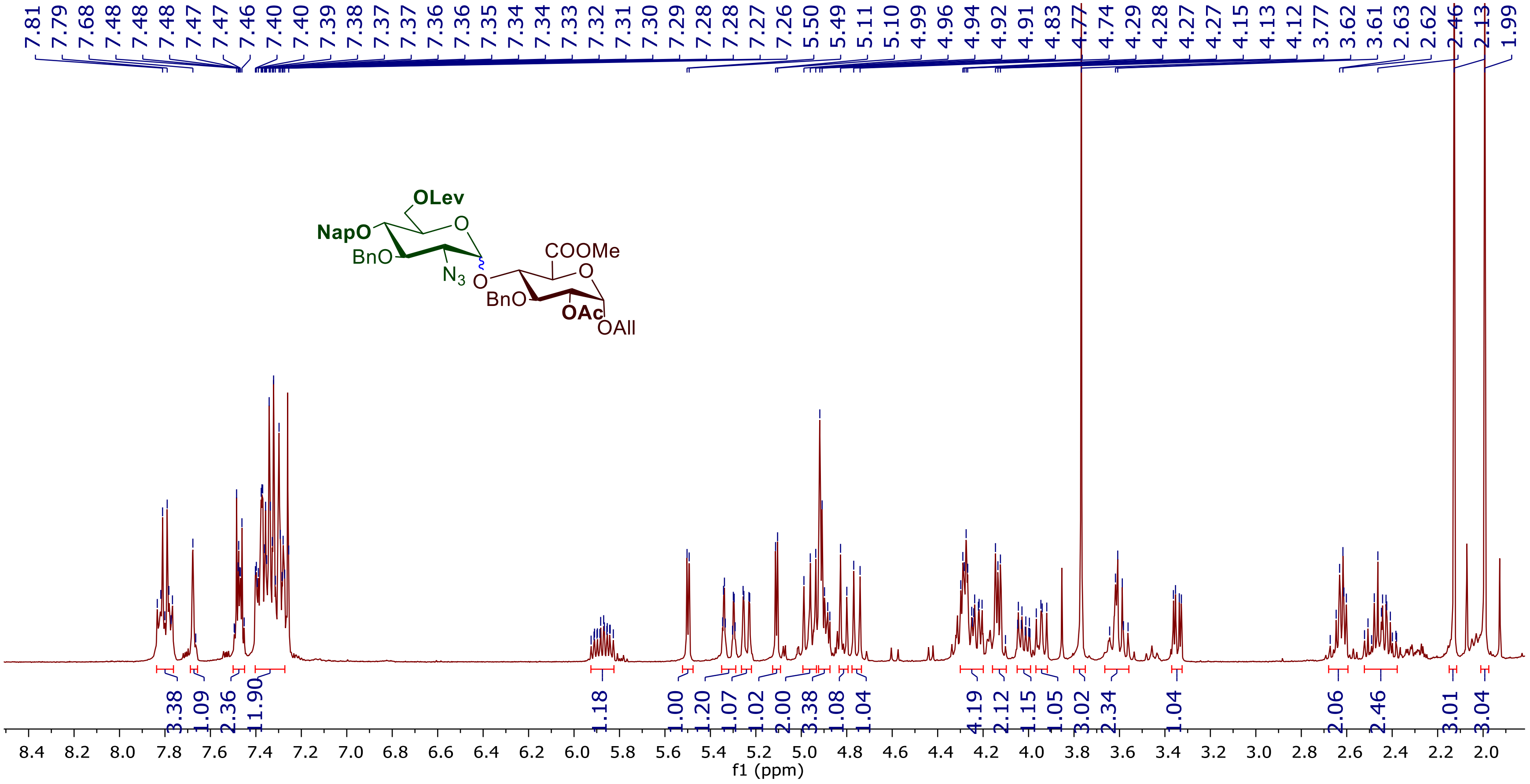

Supplementary Figure S83b. ^{13}C NMR Spectrum (100.67 MHz, $CDCl_3$) of Compound 39f

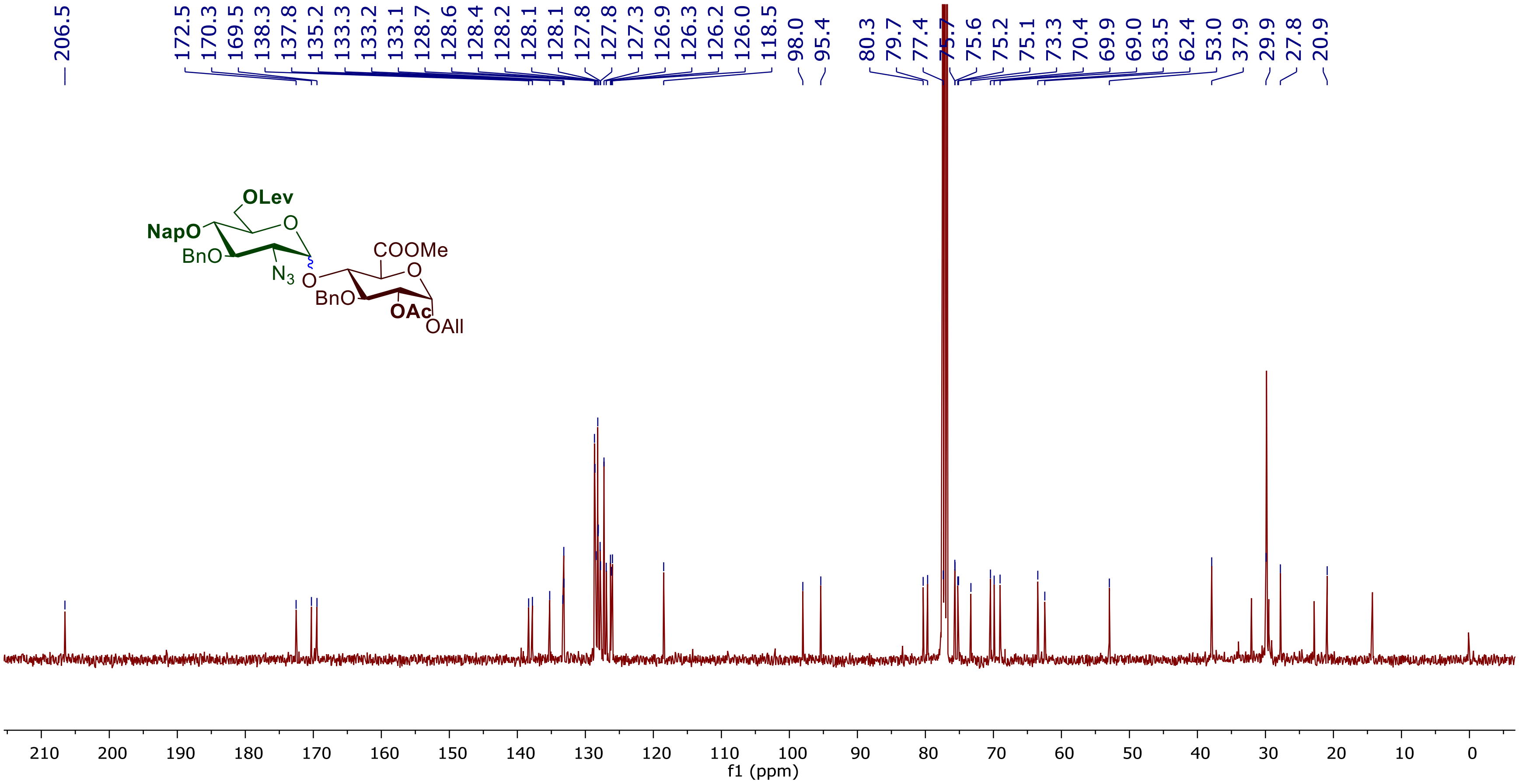

Supplementary Figure S83c. DEPT NMR Spectrum (100.67 MHz, $CDCl_3$) of Compound 39f

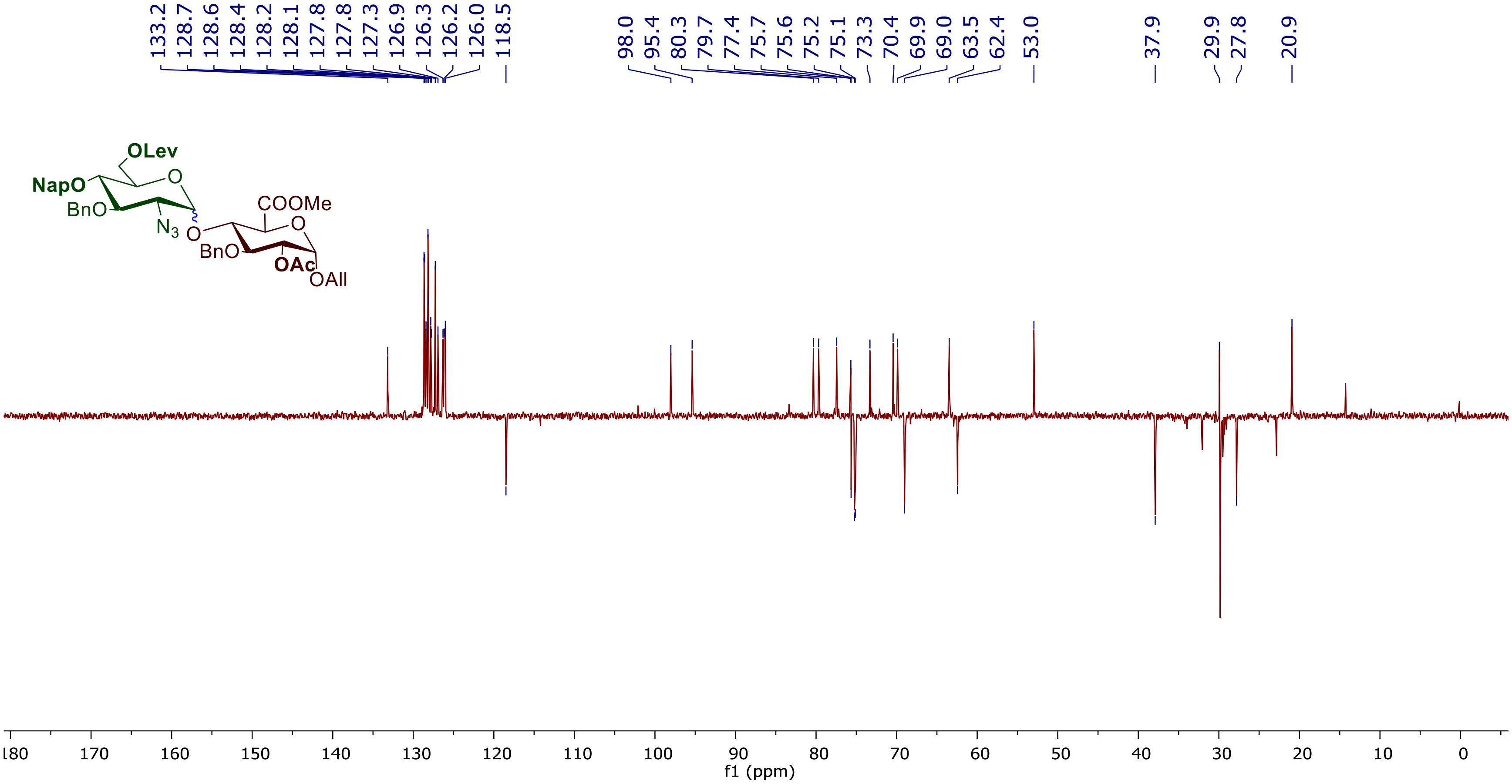

Supplementary Figure S84a. ^{1}H NMR Spectrum (400.31 MHz, $CDCl_3$) of Compound 39g

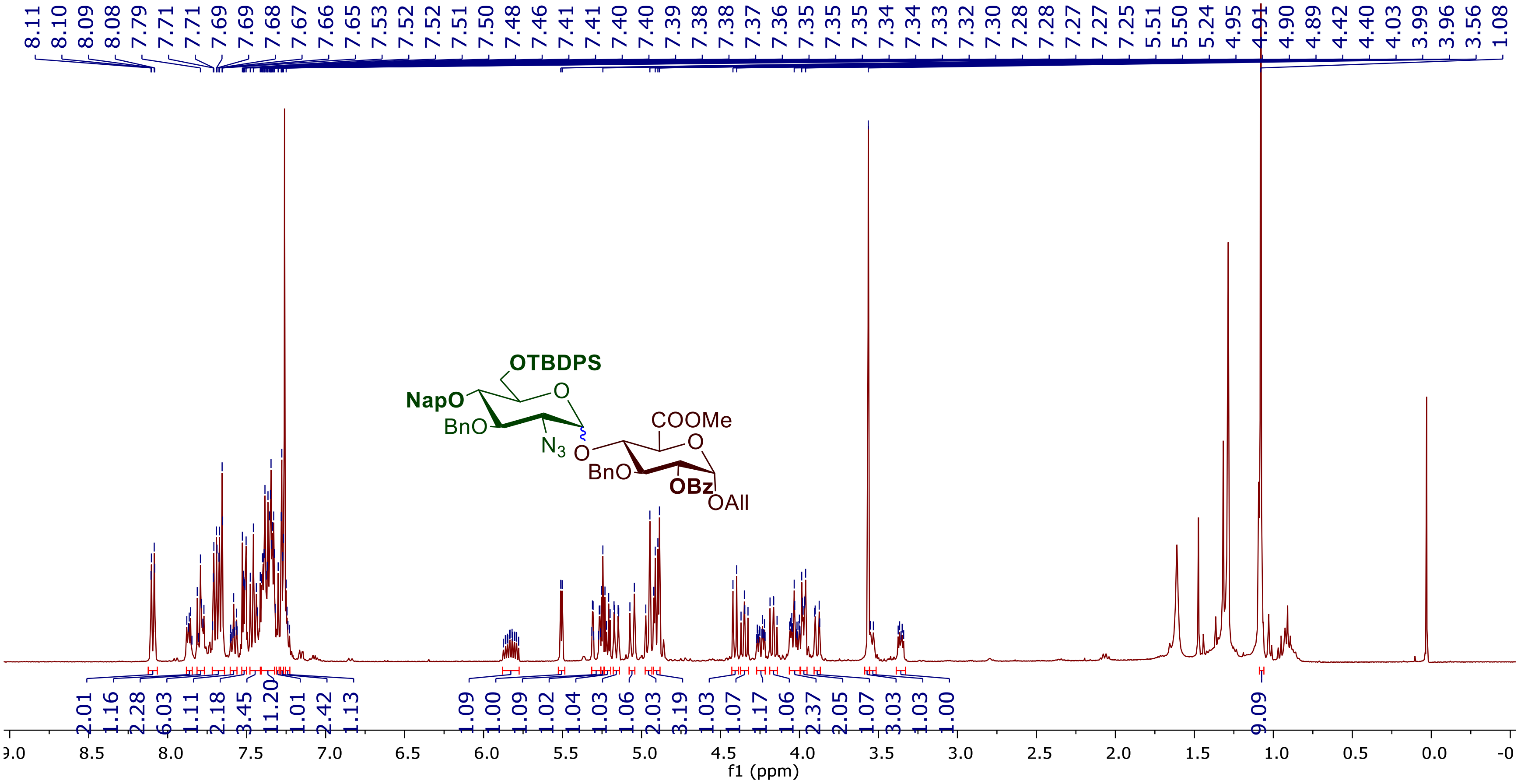

Supplementary Figure S84b. ^{13}C NMR Spectrum (100.67 MHz, $CDCl_3$) of Compound 39g

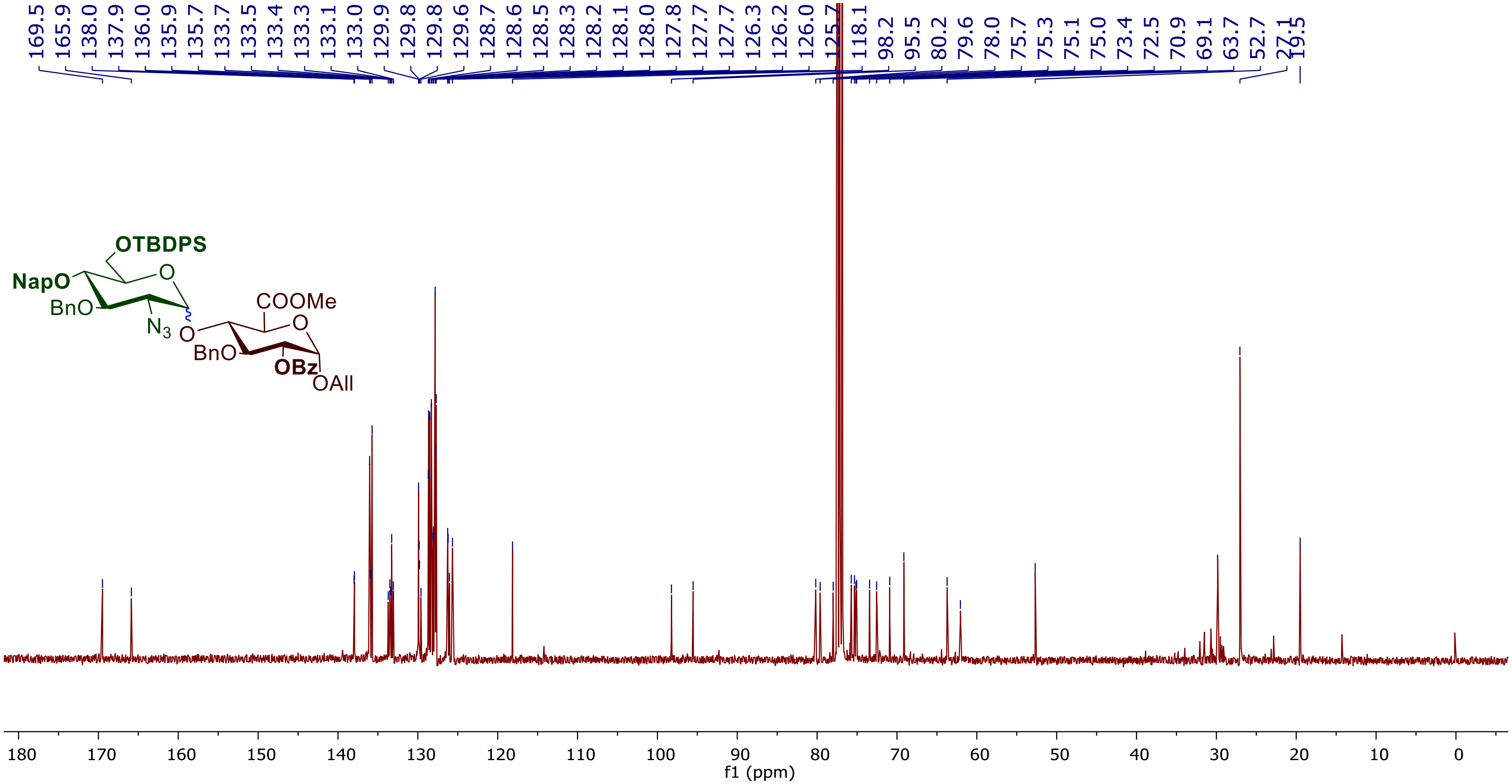

Supplementary Figure S84c. DEPT NMR Spectrum (100.67 MHz, $CDCl_3$) of Compound 39g

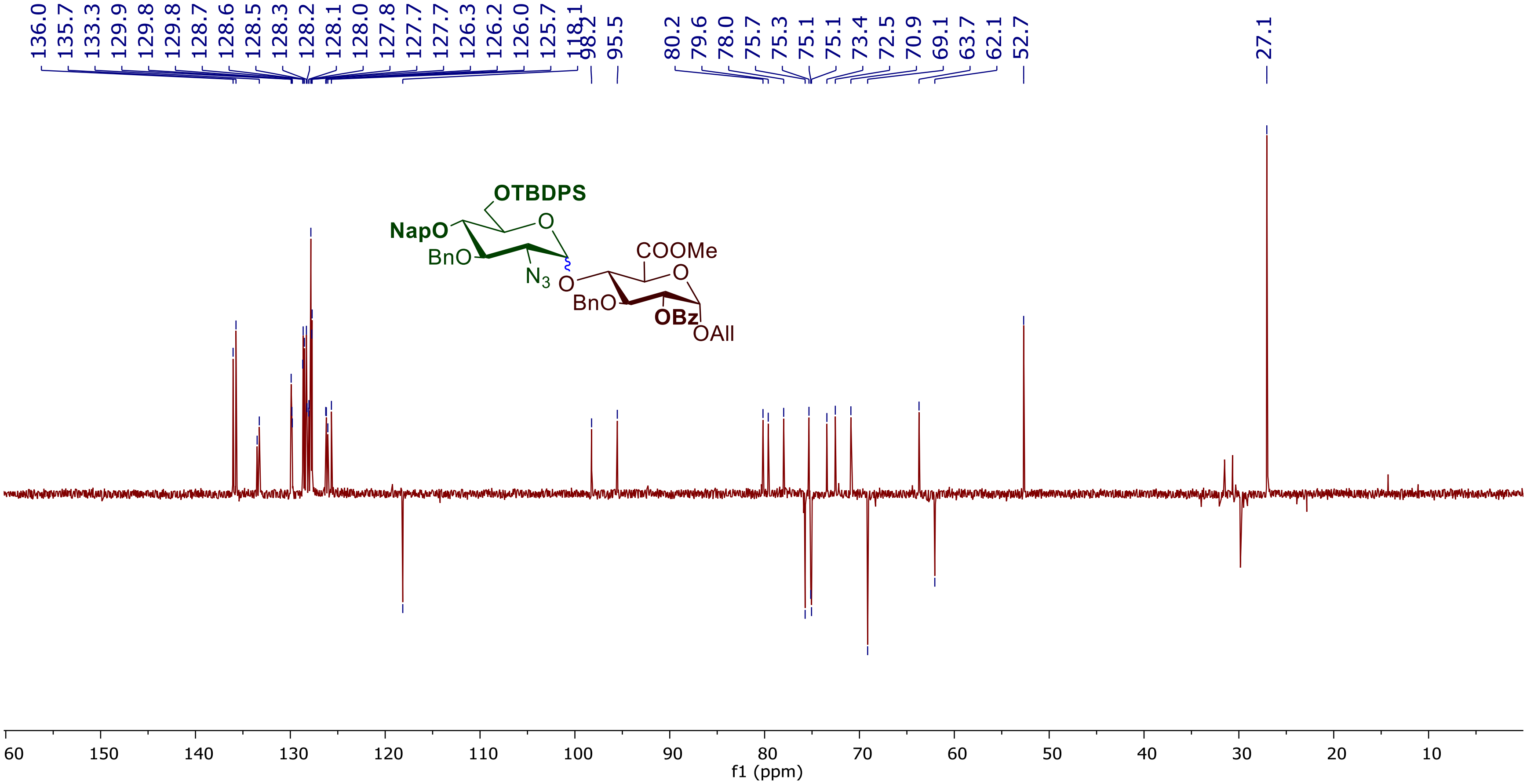

Supplementary Figure S85a. ^{1}H NMR Spectrum (400.31 MHz, $CDCl_3$) of Compound 39h

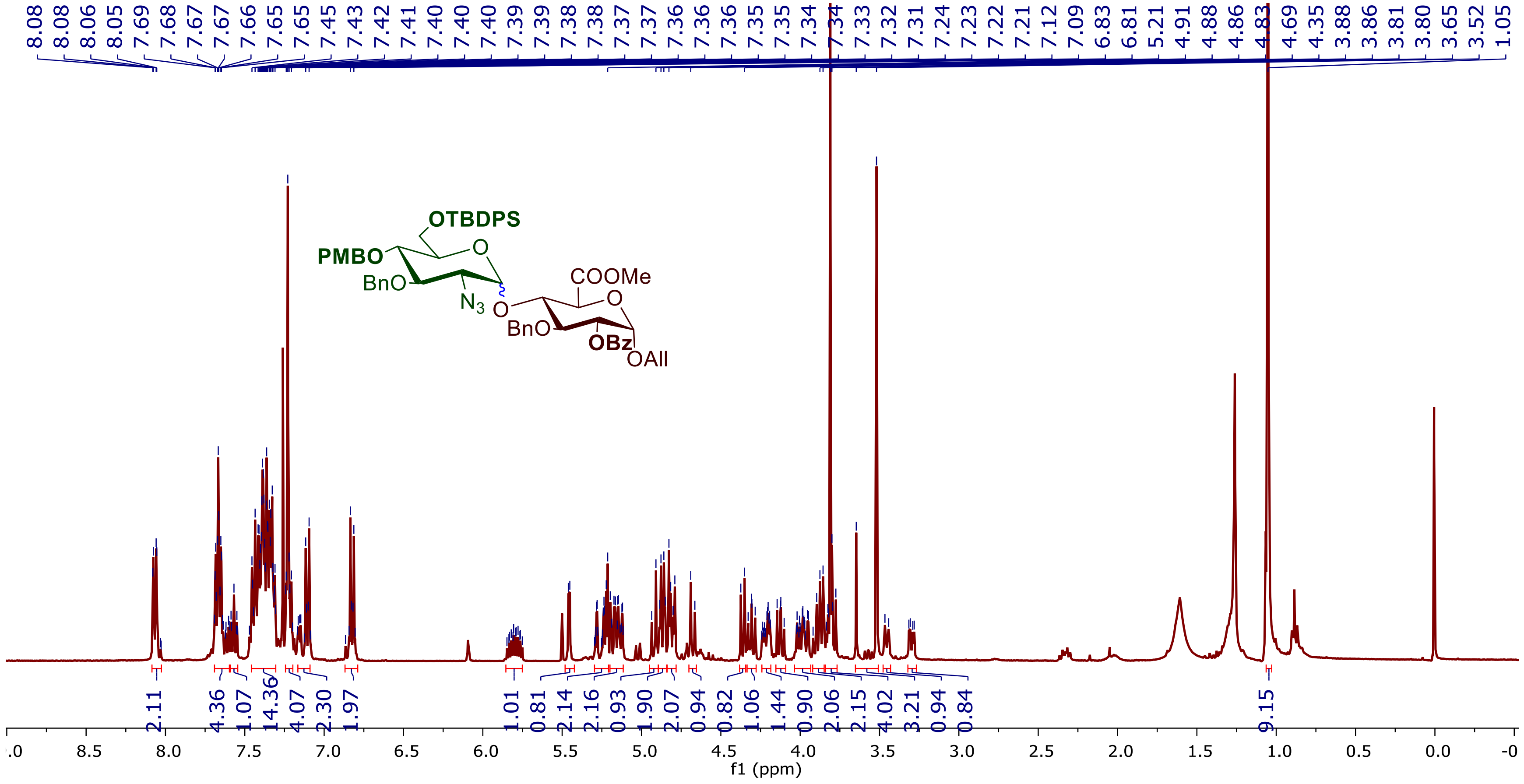

Supplementary Figure S85b. ^{13}C NMR Spectrum (100.67 MHz, $CDCl_3$) of Compound 39h

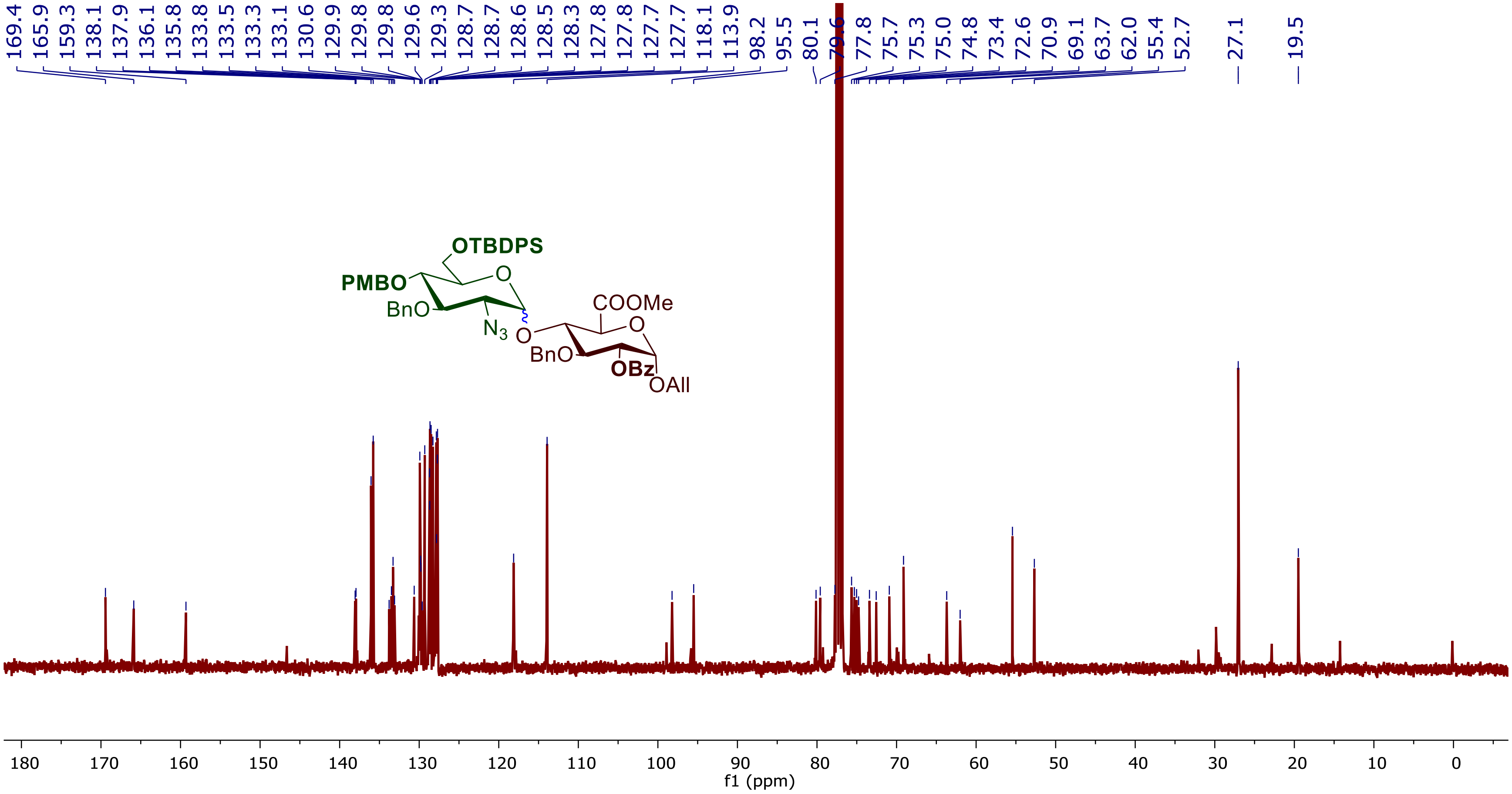

Supplementary Figure S85b. DEPT NMR Spectrum (100.67 MHz, $CDCl_3$) of Compound 39h

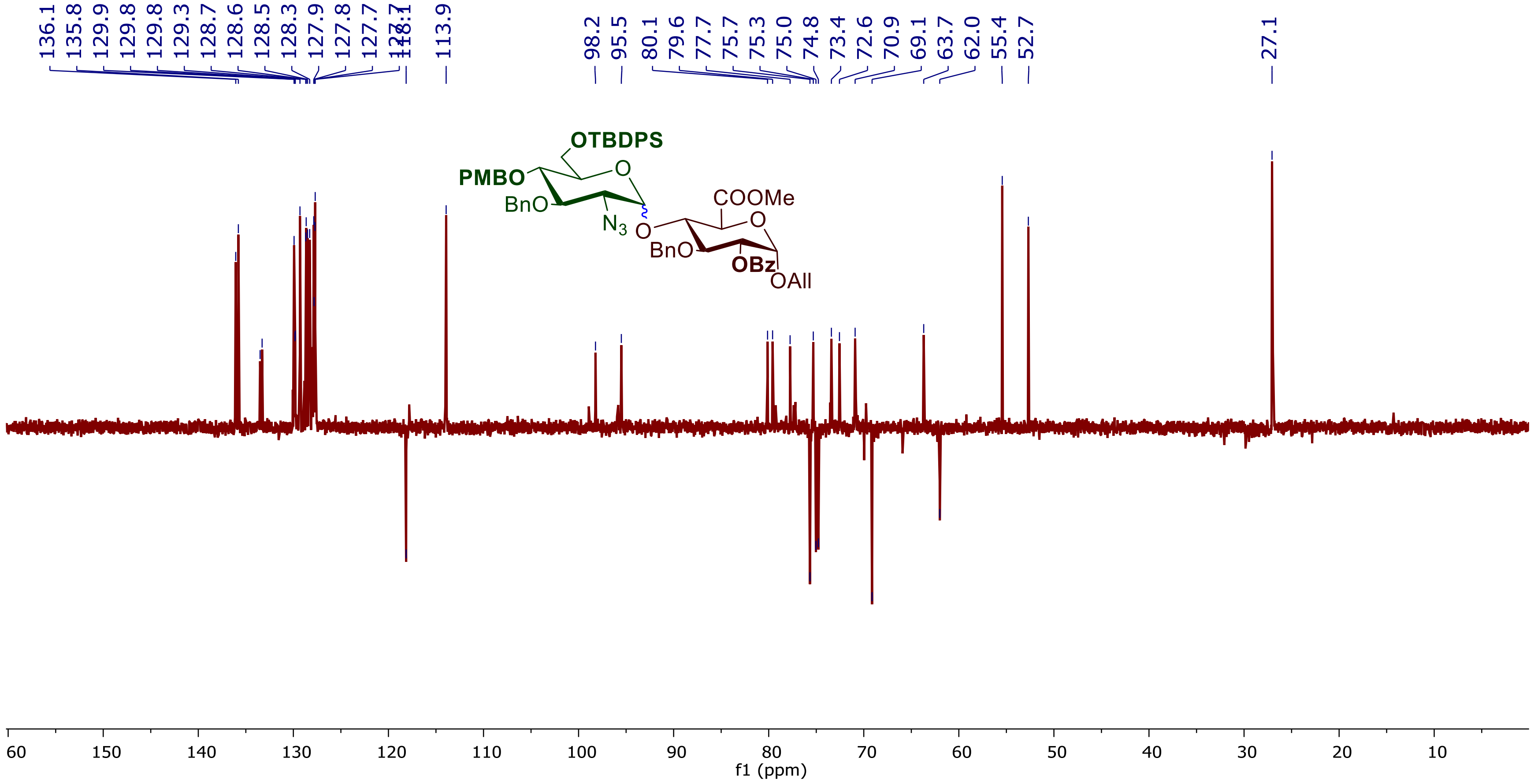

Supplementary Figure S86a. ^{1}H NMR Spectrum (400.31 MHz, $CDCl_3$) of Compound 39i (α isomer)

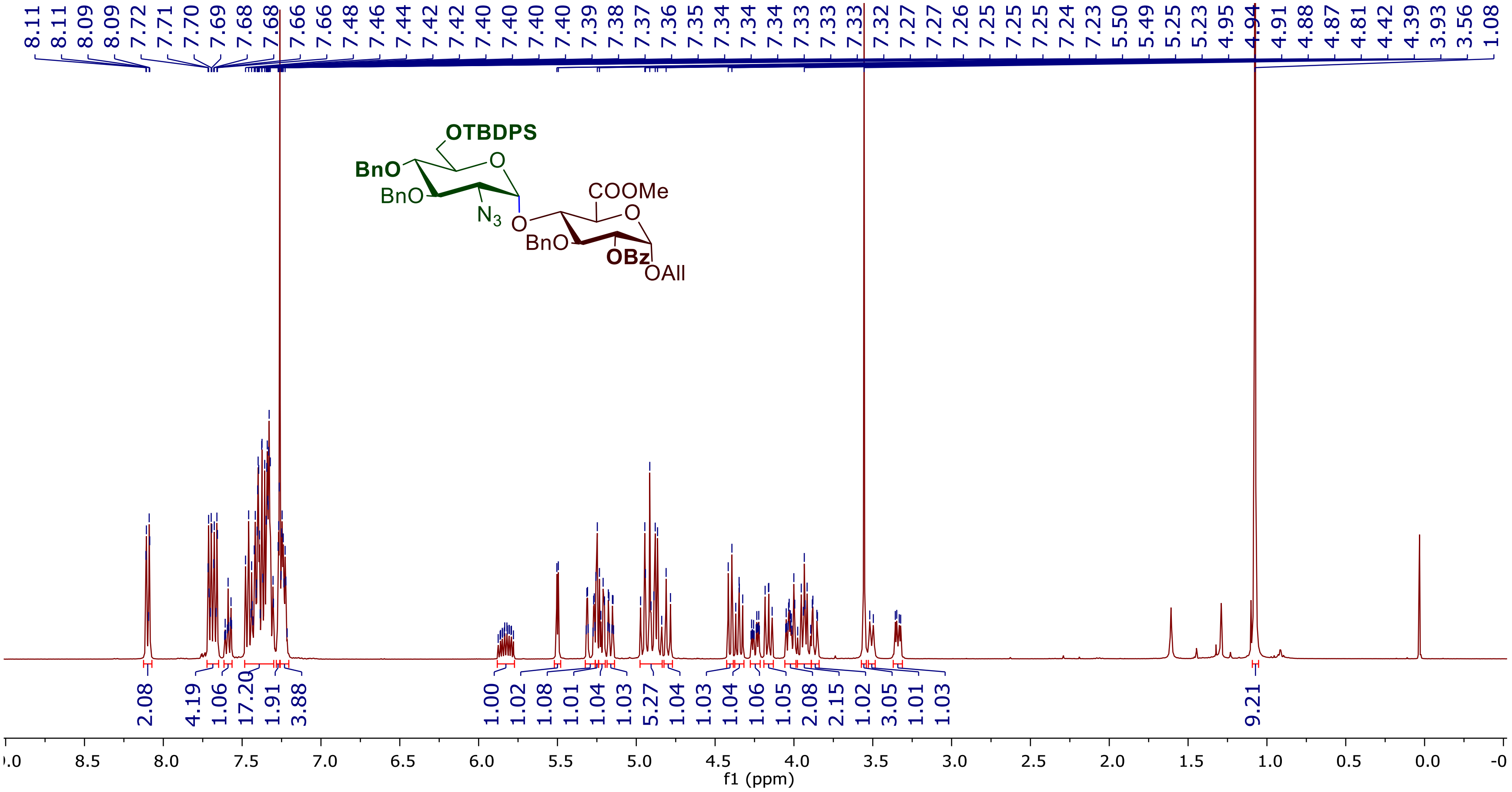

Supplementary Figure S86b. ^{13}C NMR Spectrum (100.67 MHz, $CDCl_3$) of Compound 39i (α isomer)

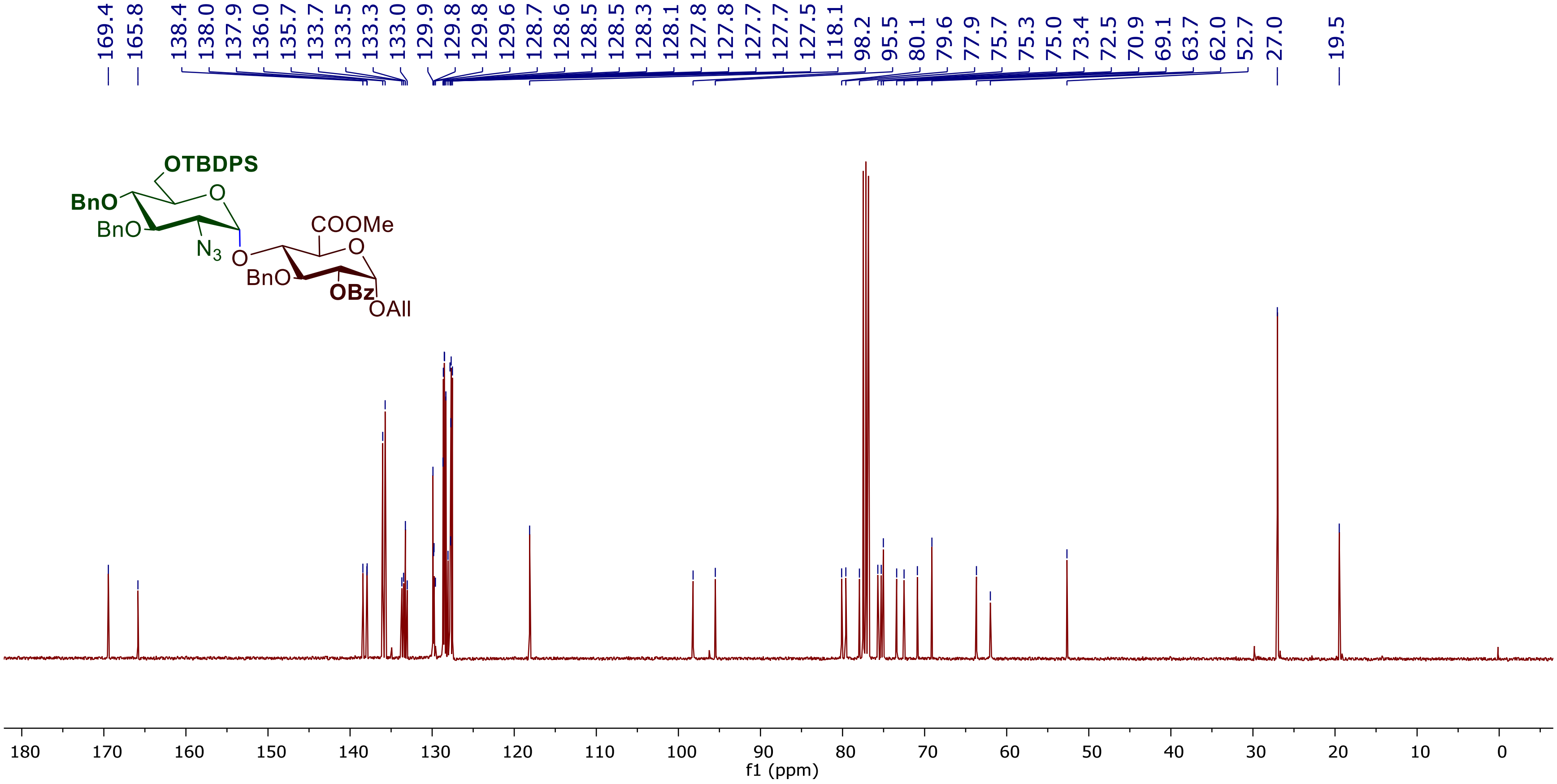

Supplementary Figure S86c. DEPT NMR Spectrum (100.67 MHz, $CDCl_3$) of Compound 39i (α isomer)

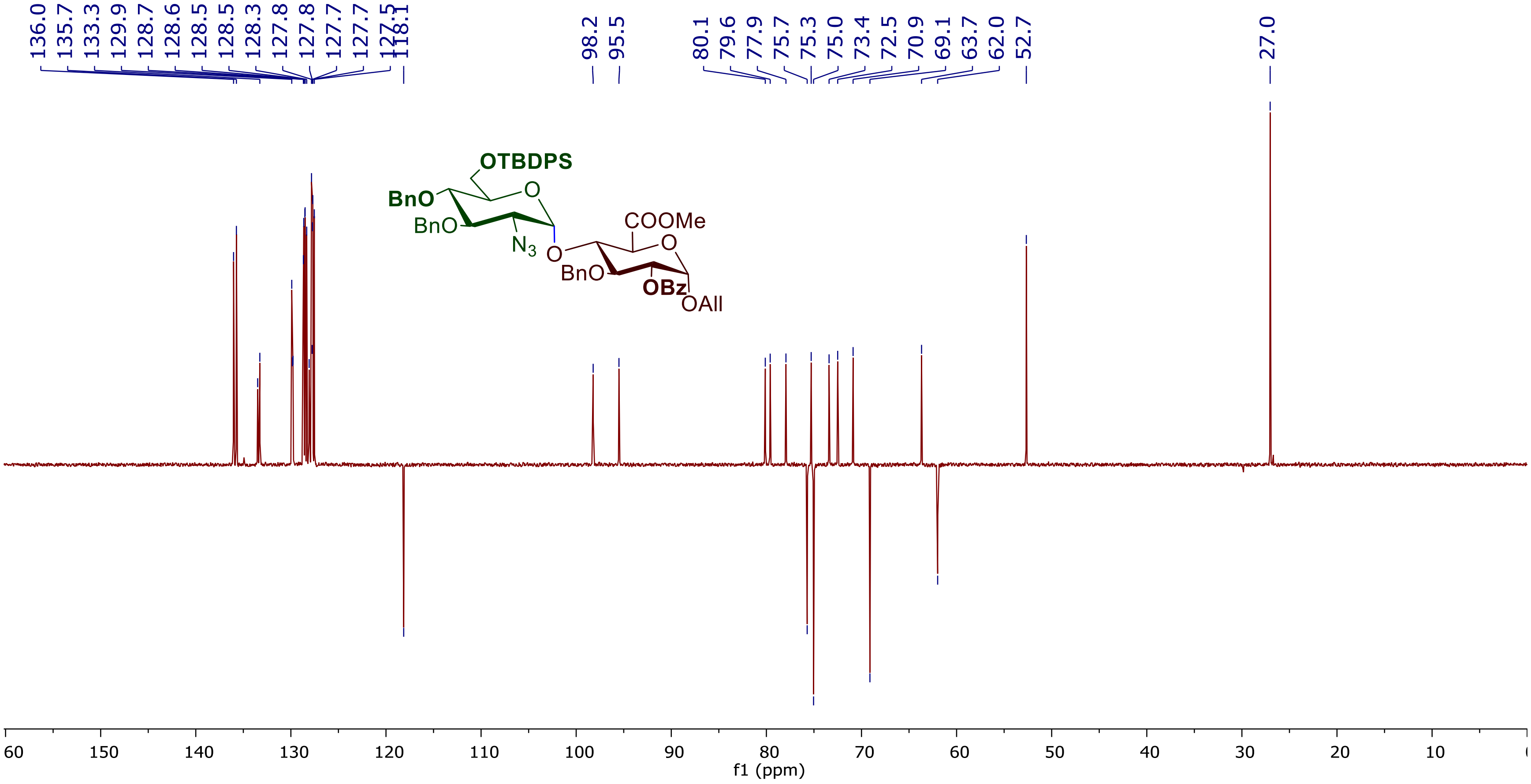

Supplementary Figure S87a. ^{1}H NMR Spectrum (400.31 MHz, $CDCl_3$) of Compound 39i (β isomer)

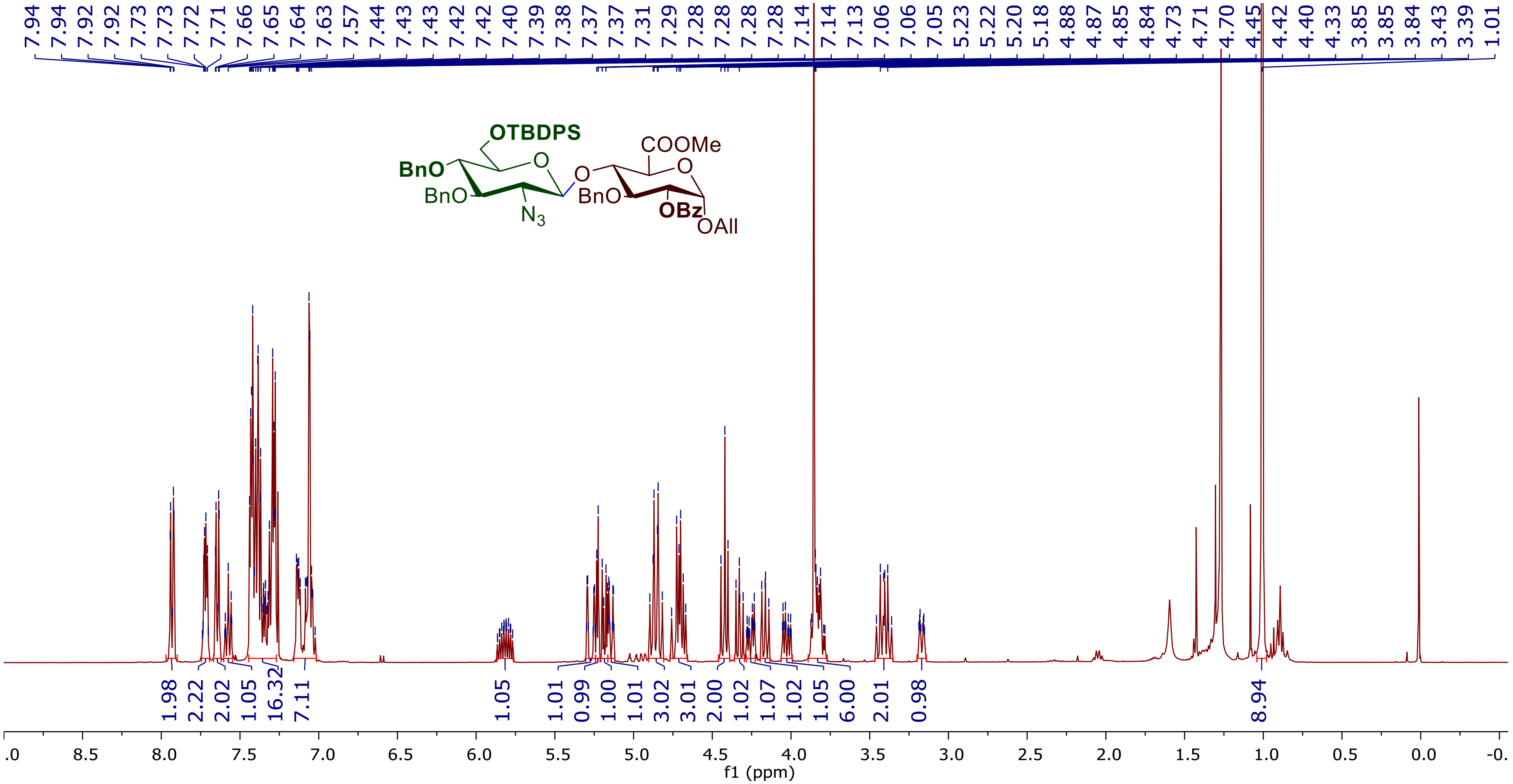

Supplementary Figure S87b. ^{13}C NMR Spectrum (100.67 MHz, $CDCl_3$) of Compound 39i (β isomer)

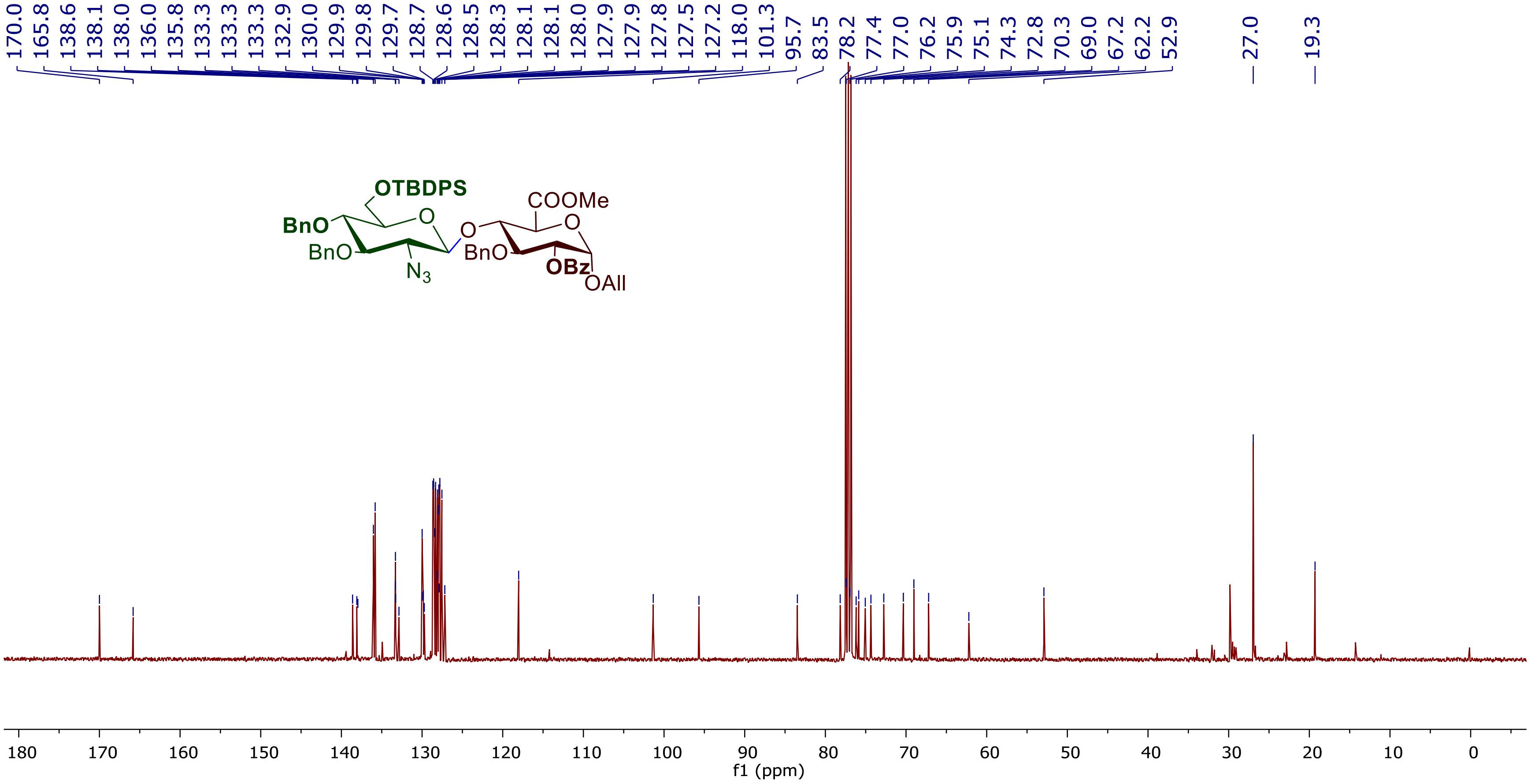

Supplementary Figure S87c. DEPT NMR Spectrum (100.67 MHz, $CDCl_3$) of Compound 39i (β isomer)

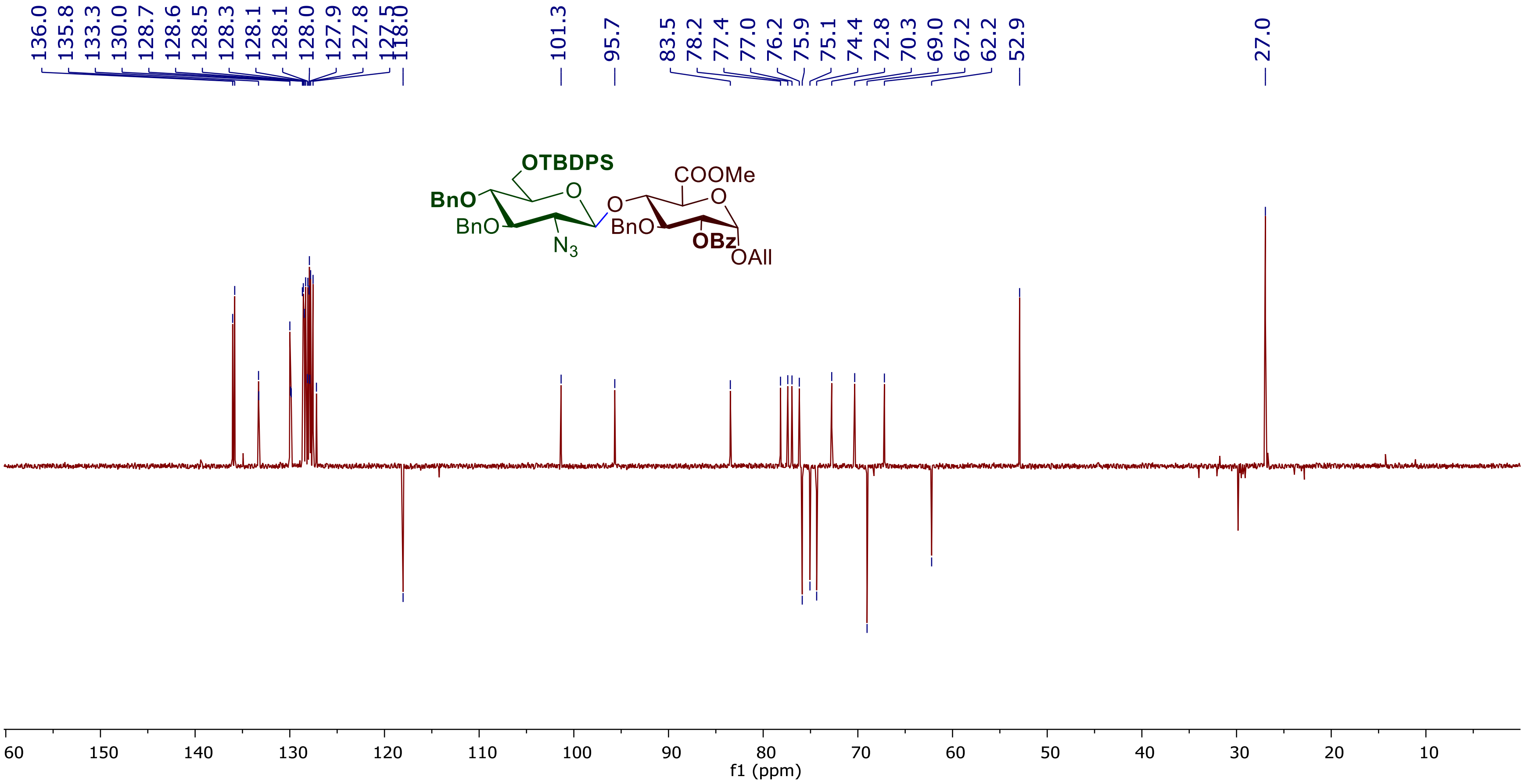

Supplementary Figure S88a. ¹H NMR Spectrum (400.31 MHz, $CDCl_3$) of Compound S13

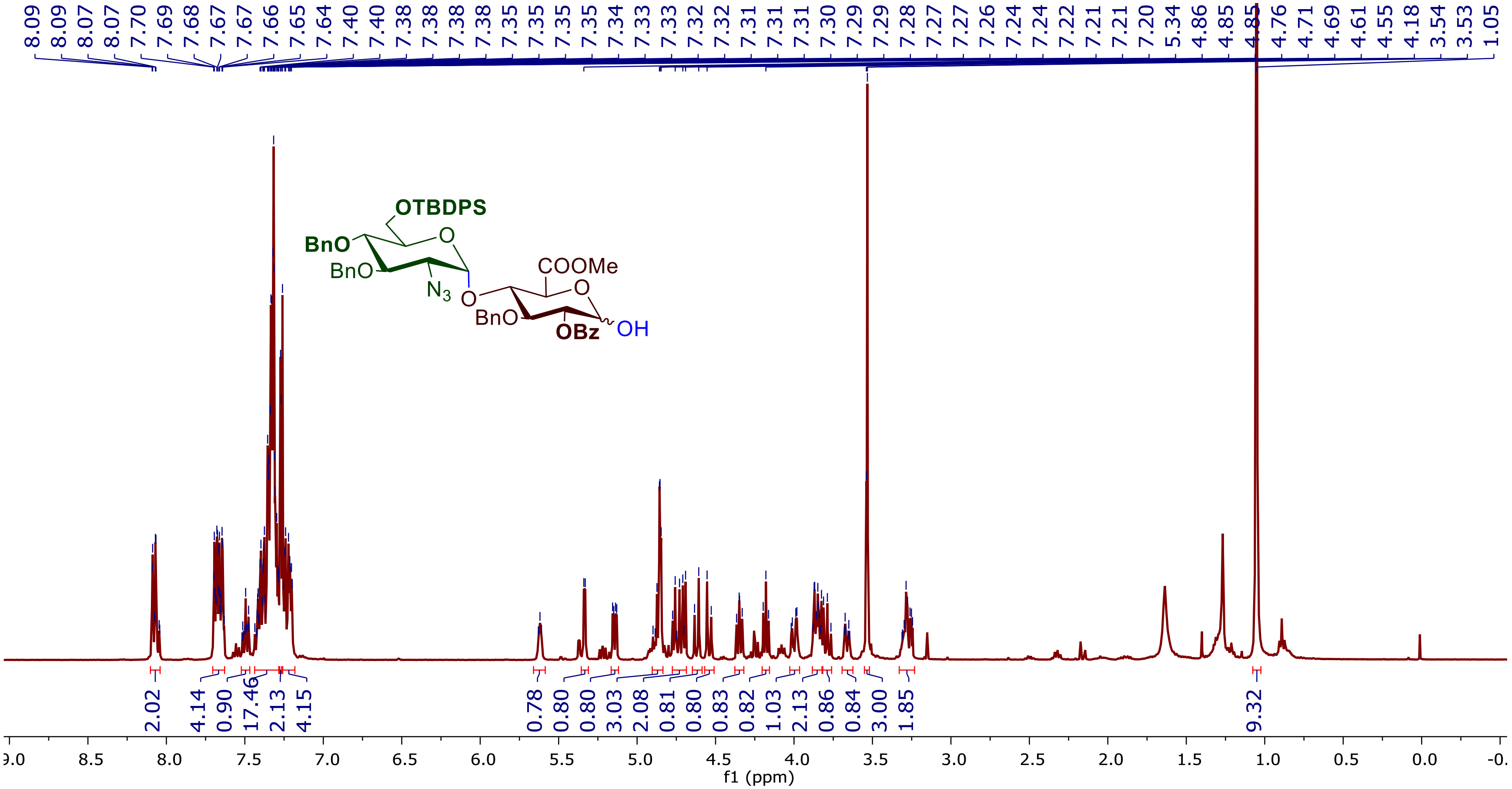

Supplementary Figure S88b. ^{13}C NMR Spectrum (100.67 MHz, $CDCl_3$) of Compound S13

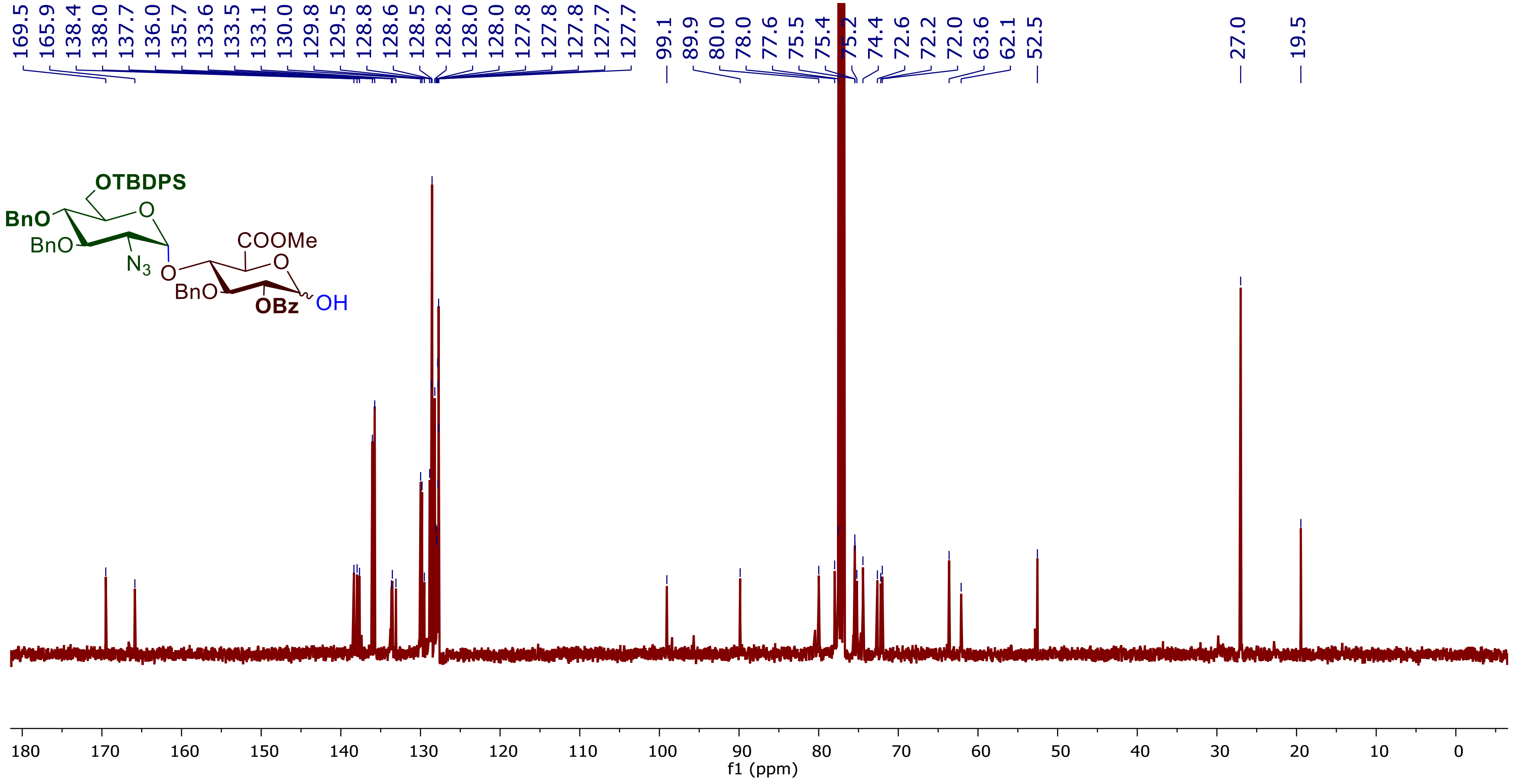

Supplementary Figure S88c. DEPT NMR Spectrum (100.67 MHz, $CDCl_3$) of Compound S13

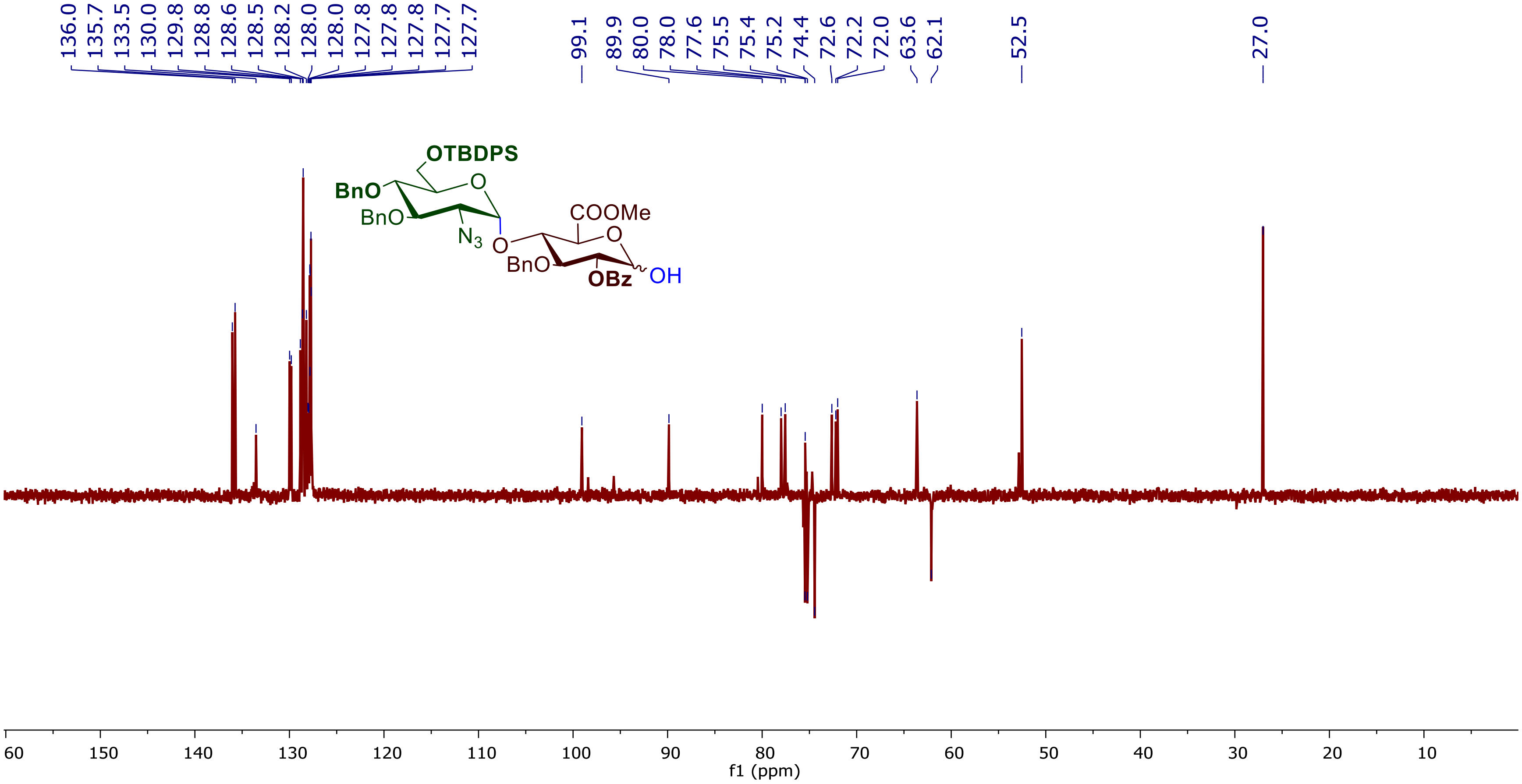

Supplementary Figure S89a. ^{1}H NMR Spectrum (400.31 MHz, CDCl₃) of Compound 40

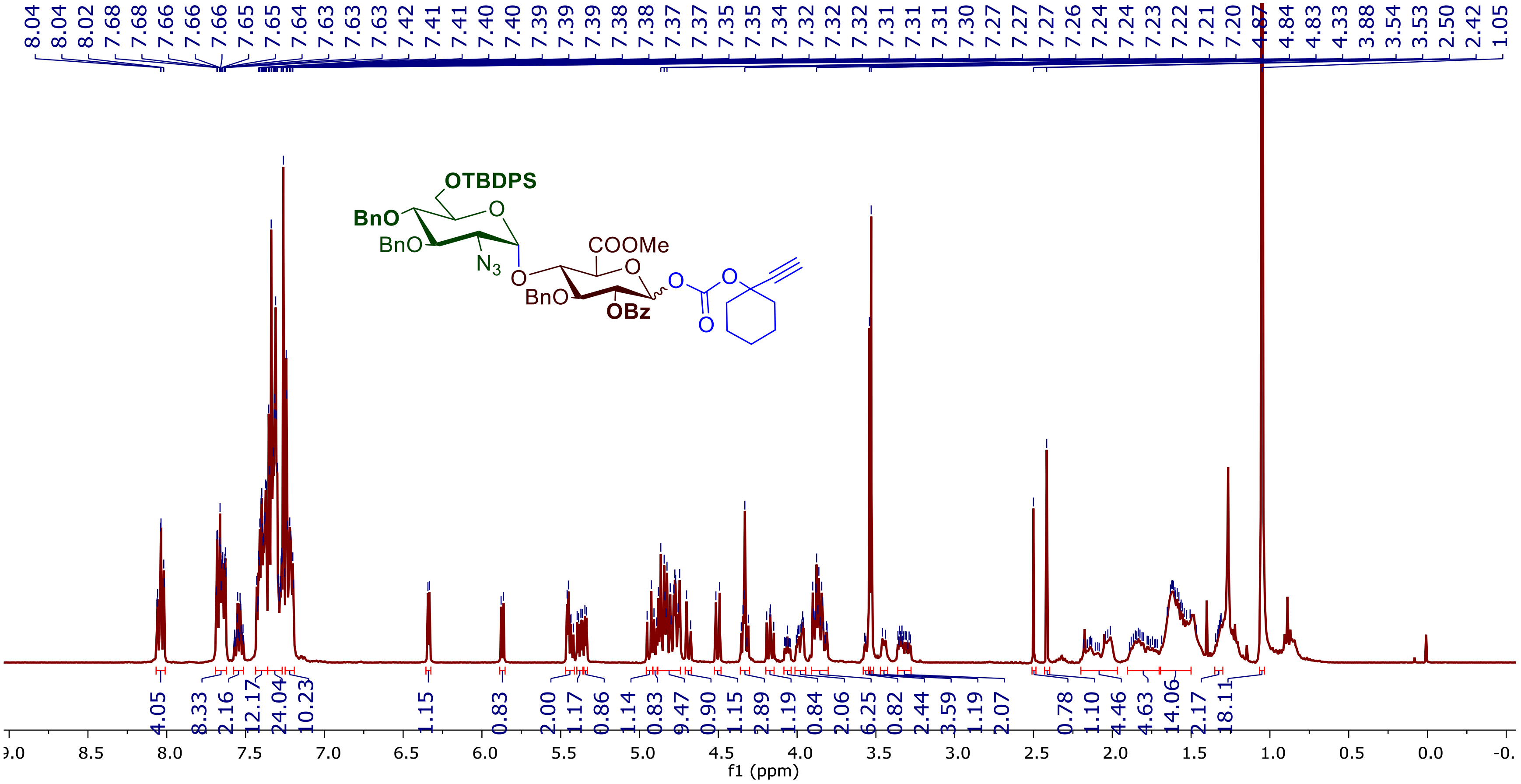

Supplementary Figure S89b. ^{13}C NMR Spectrum (100.67 MHz, $CDCl_3$) of Compound 40

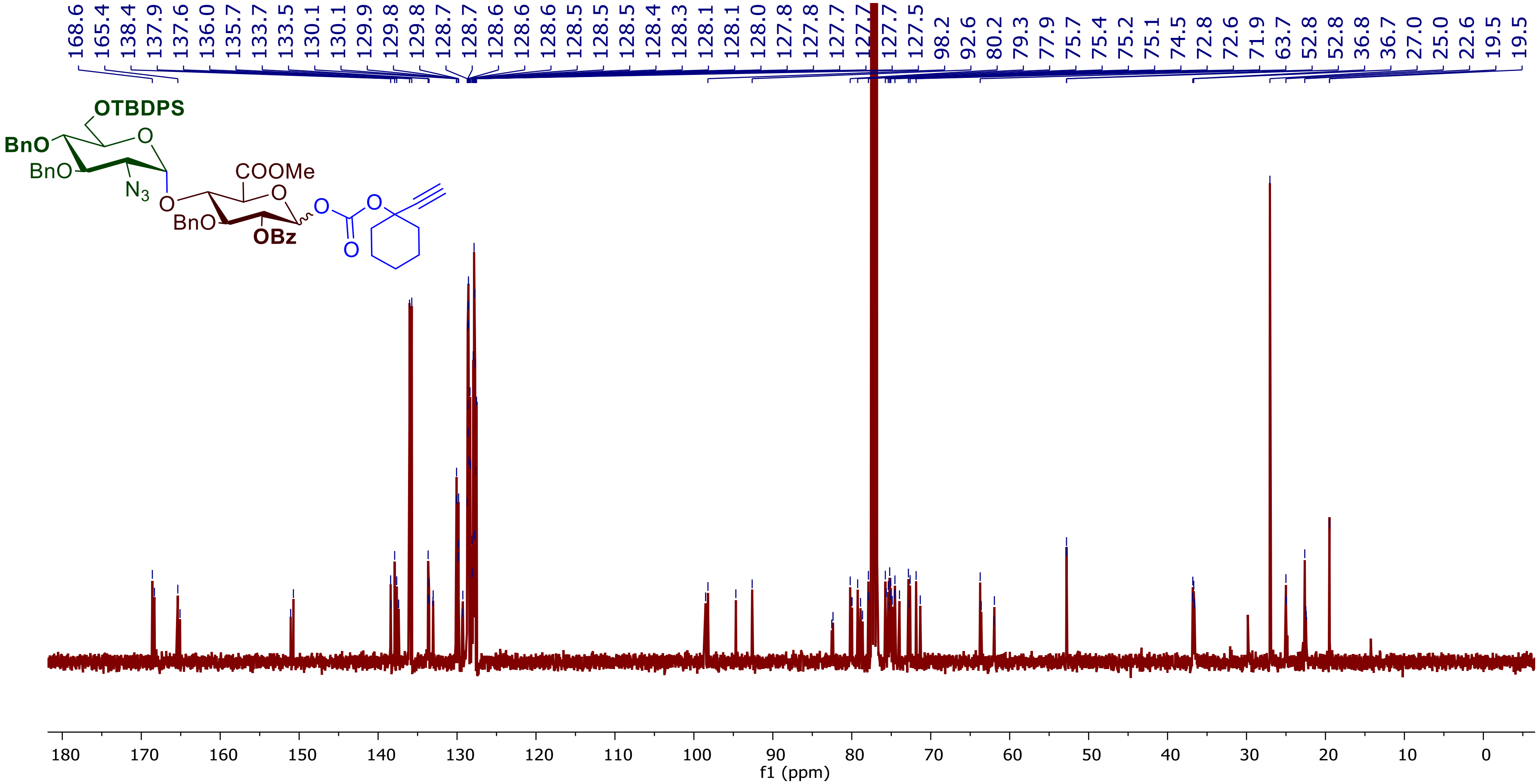

Supplementary Figure S89c. DEPT NMR Spectrum (100.67 MHz, $CDCl_3$) of Compound 40

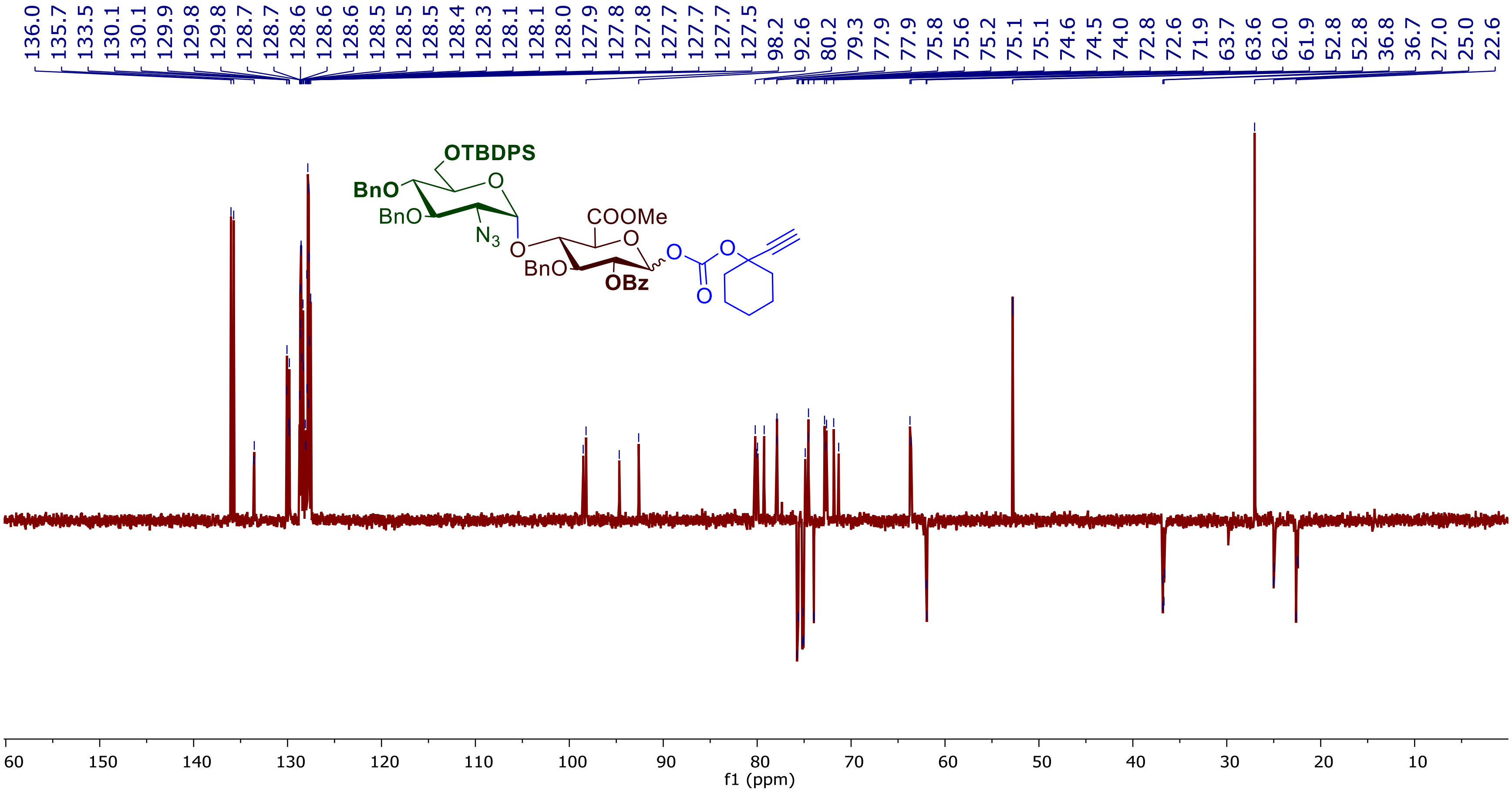

Supplementary Figure S90a. ^{1}H NMR Spectrum (400.31 MHz, $CDCl_3$) of Compound 41

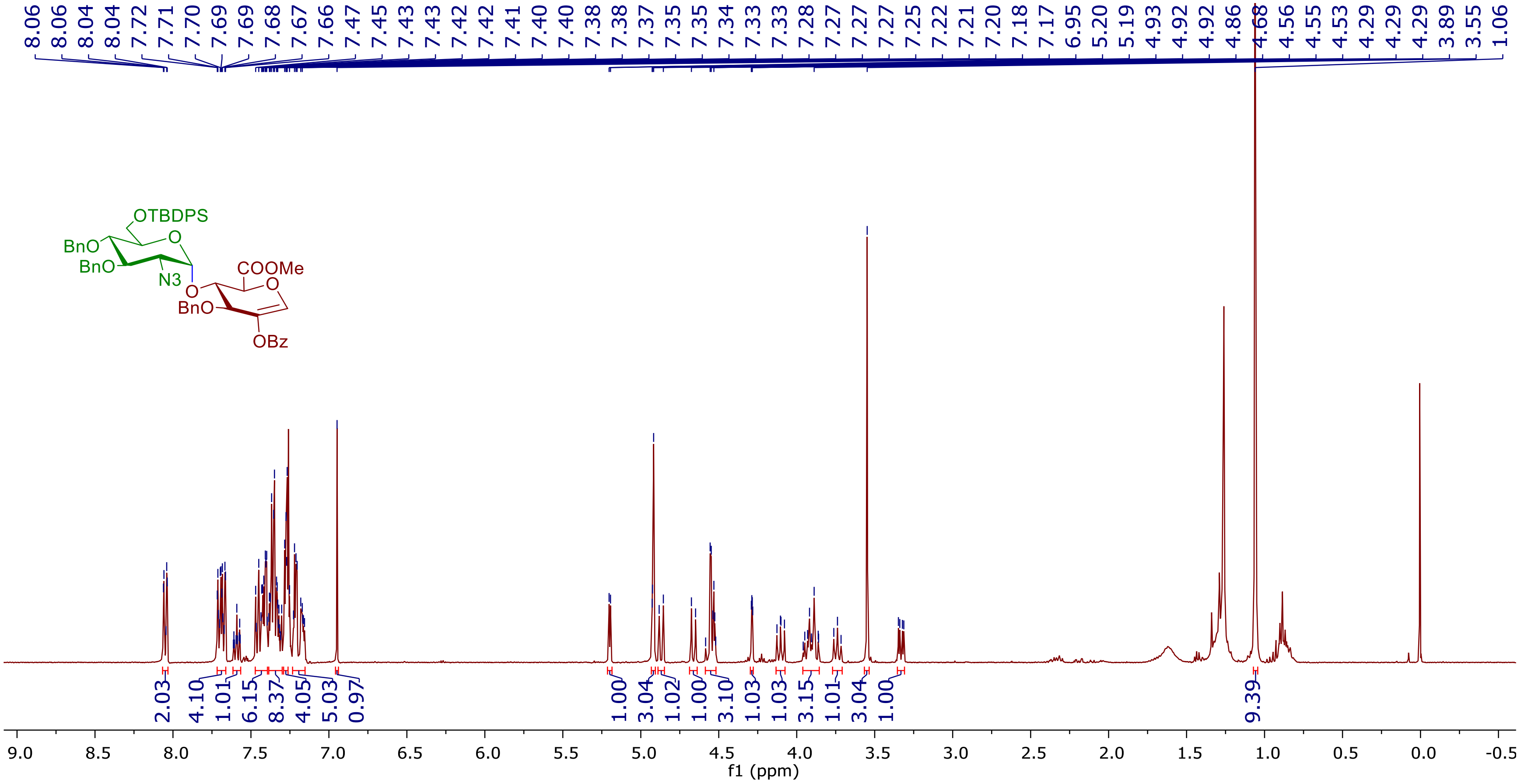

Supplementary Figure S90b. ^{13}C NMR Spectrum (100.67 MHz, $CDCl_3$) of Compound 41

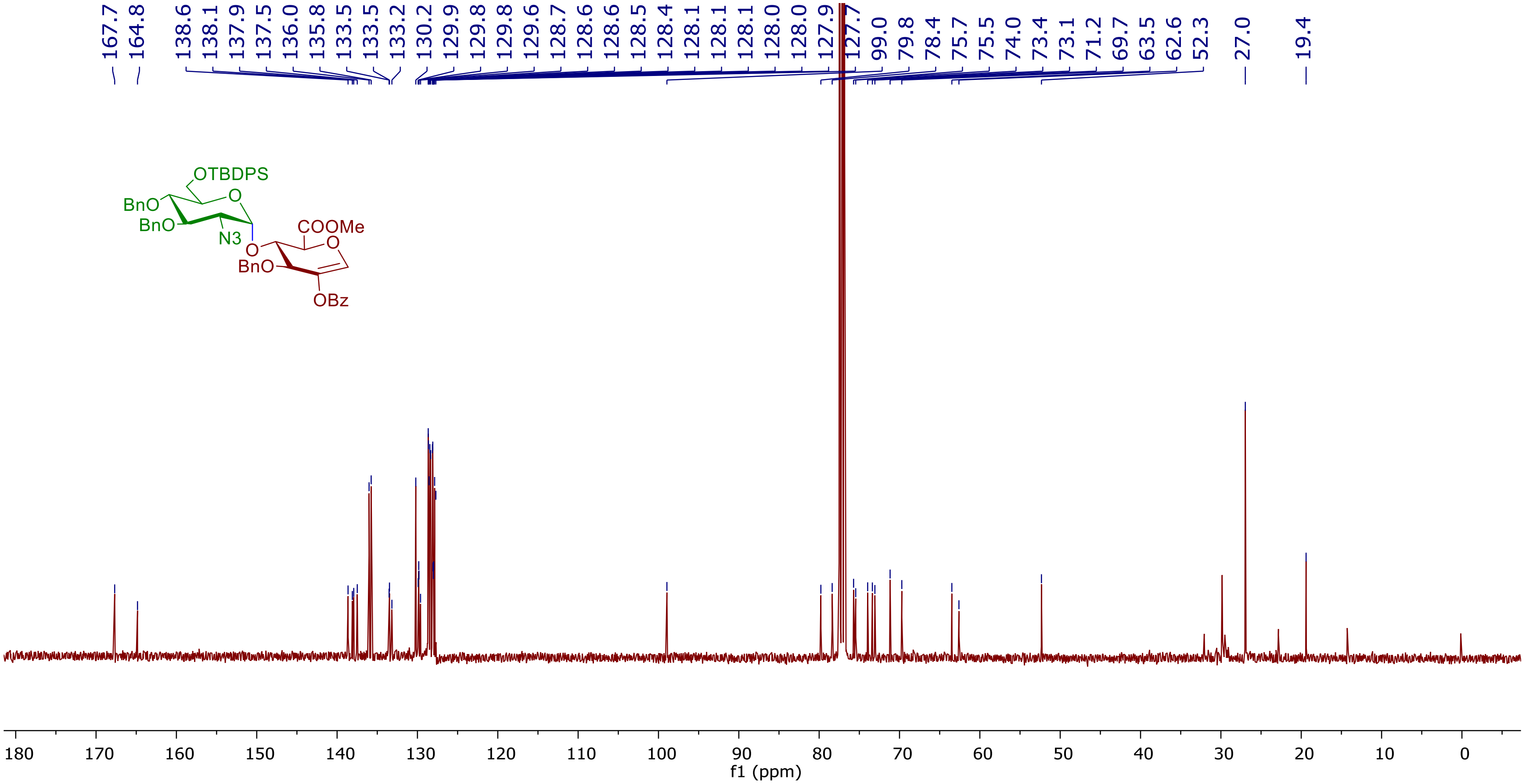

Supplementary Figure S90c. DEPT NMR Spectrum (100.67 MHz, $CDCl_3$) of Compound 41

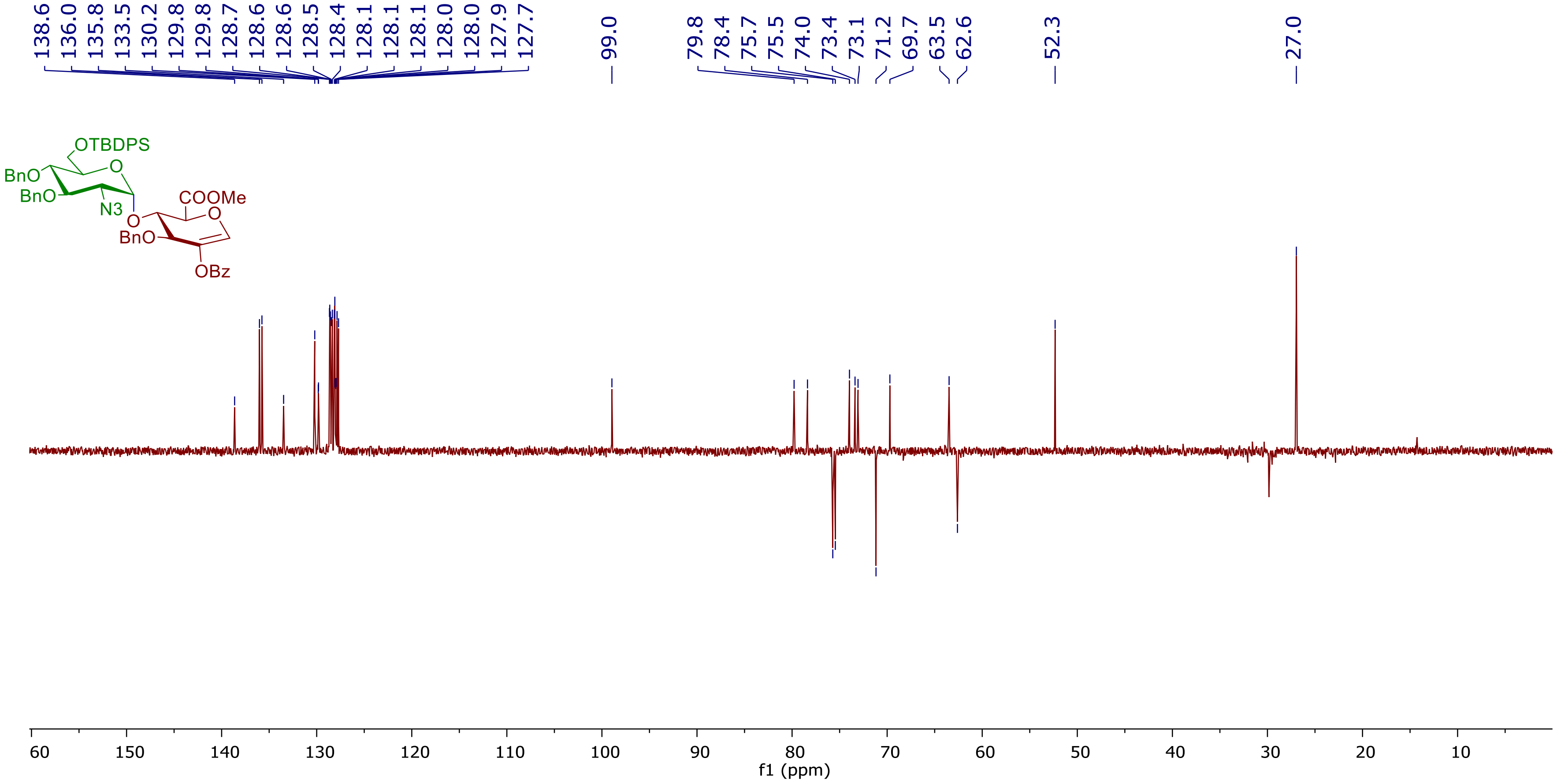

Supplementary Figure S91a. ^{1}H NMR Spectrum (400.31 MHz, $CDCl_3$) of Compound 42

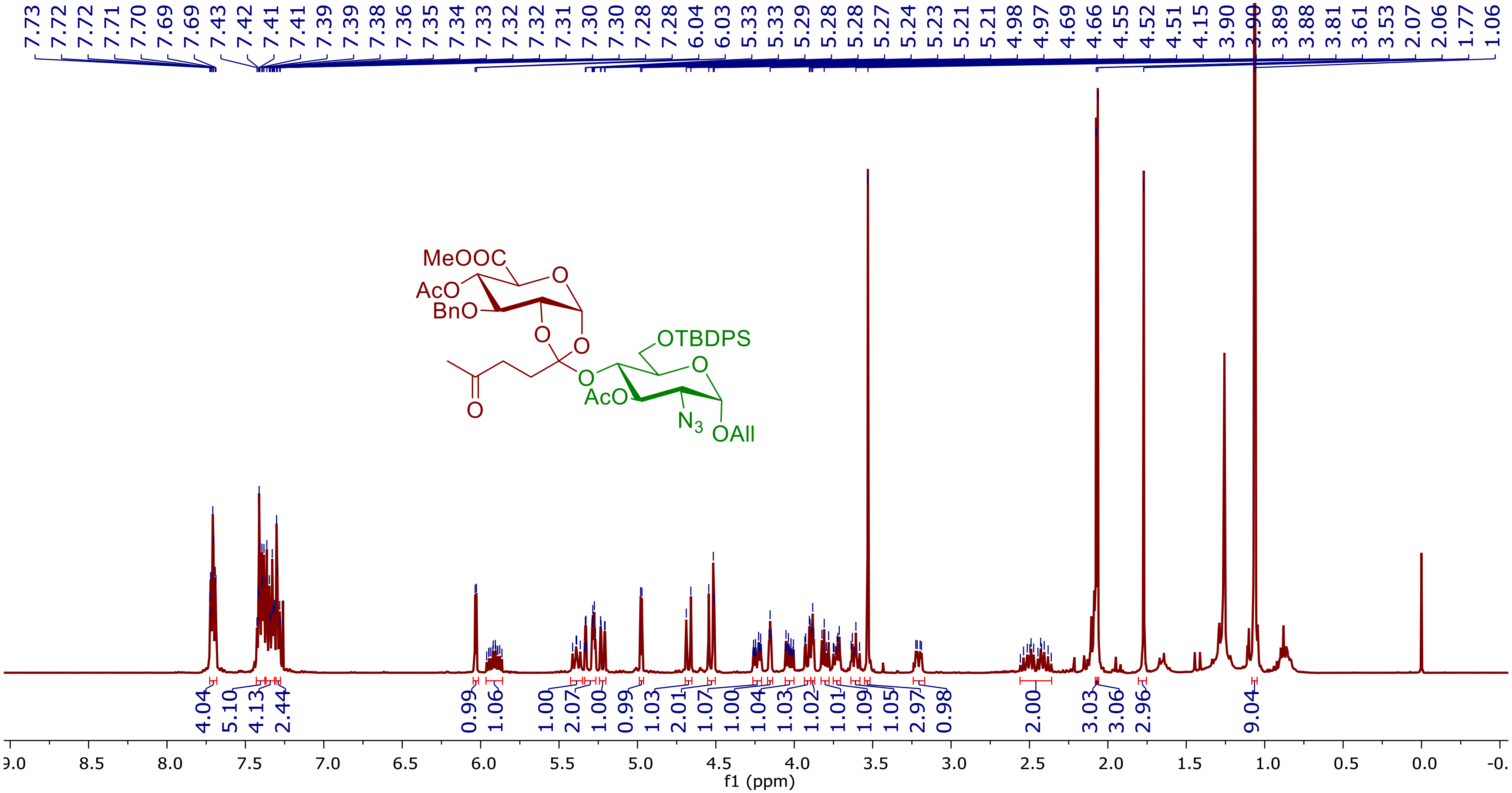

Supplementary Figure S91b. ^{13}C NMR Spectrum (100.67 MHz, $CDCl_3$) of Compound 42

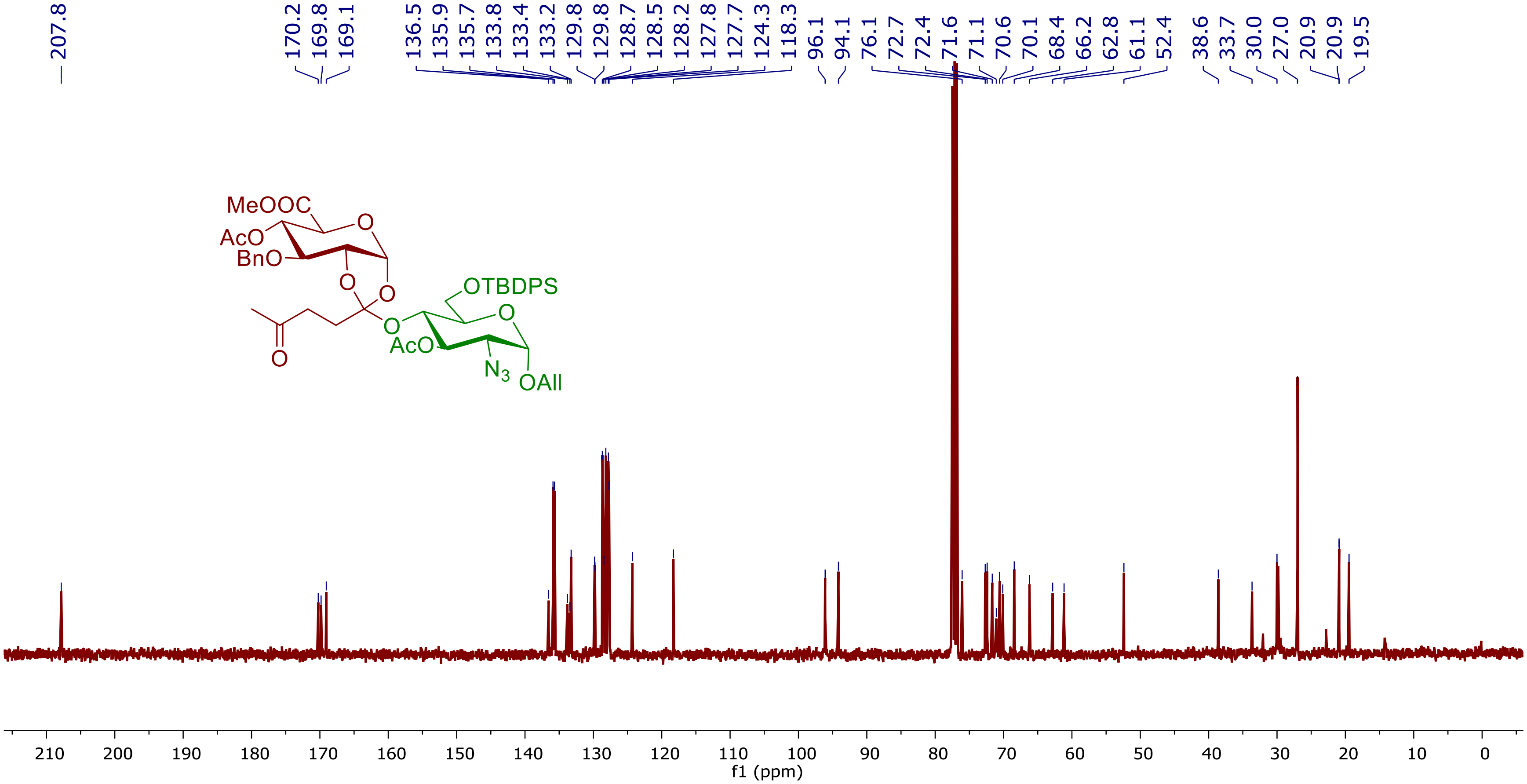

Supplementary Figure S91c. DEPT NMR Spectrum (100.67 MHz, $CDCl_3$) of Compound 42

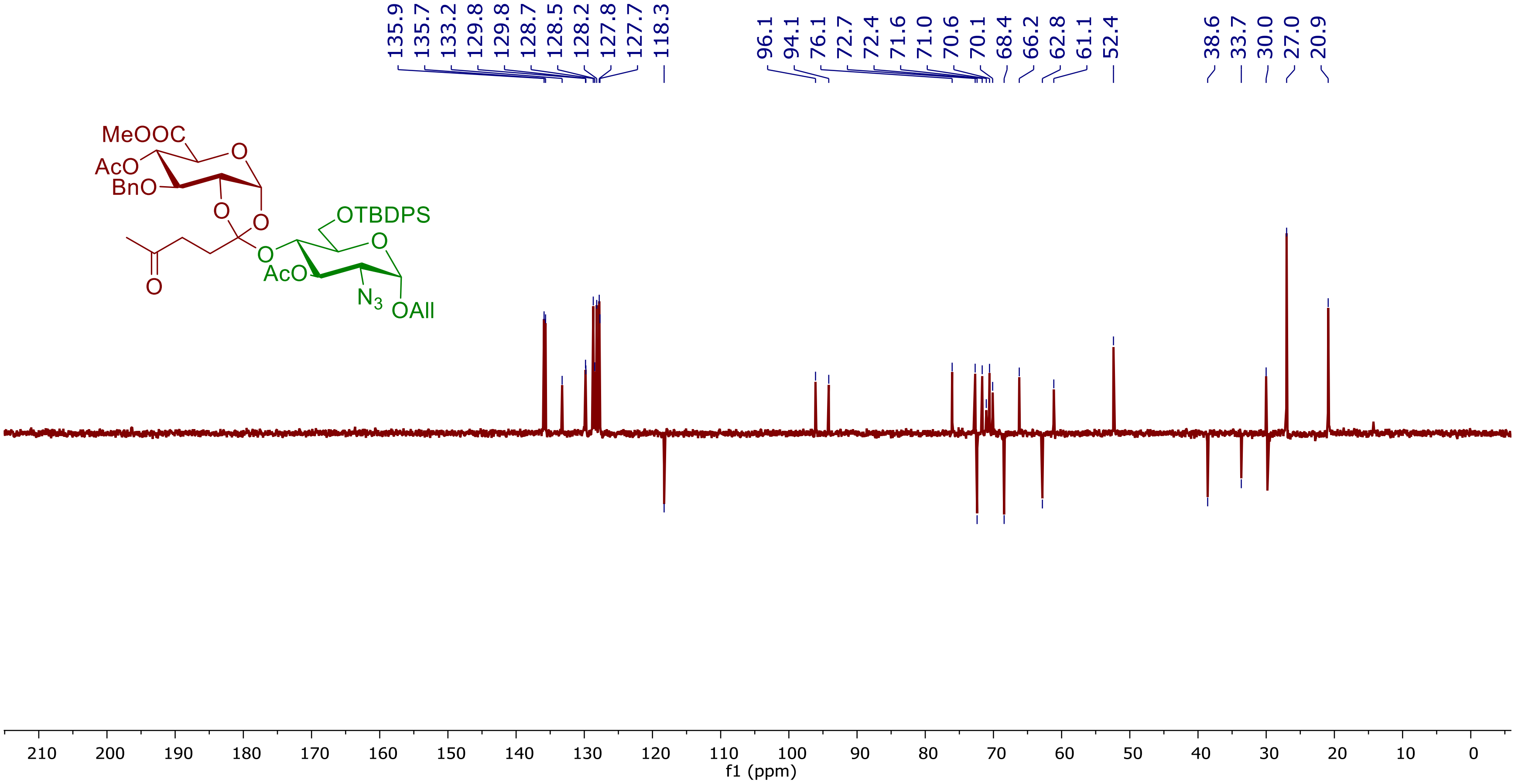

Supplementary Figure S92a. ¹H NMR Spectrum (400.31 MHz, $CDCl_3$) of Compound 43

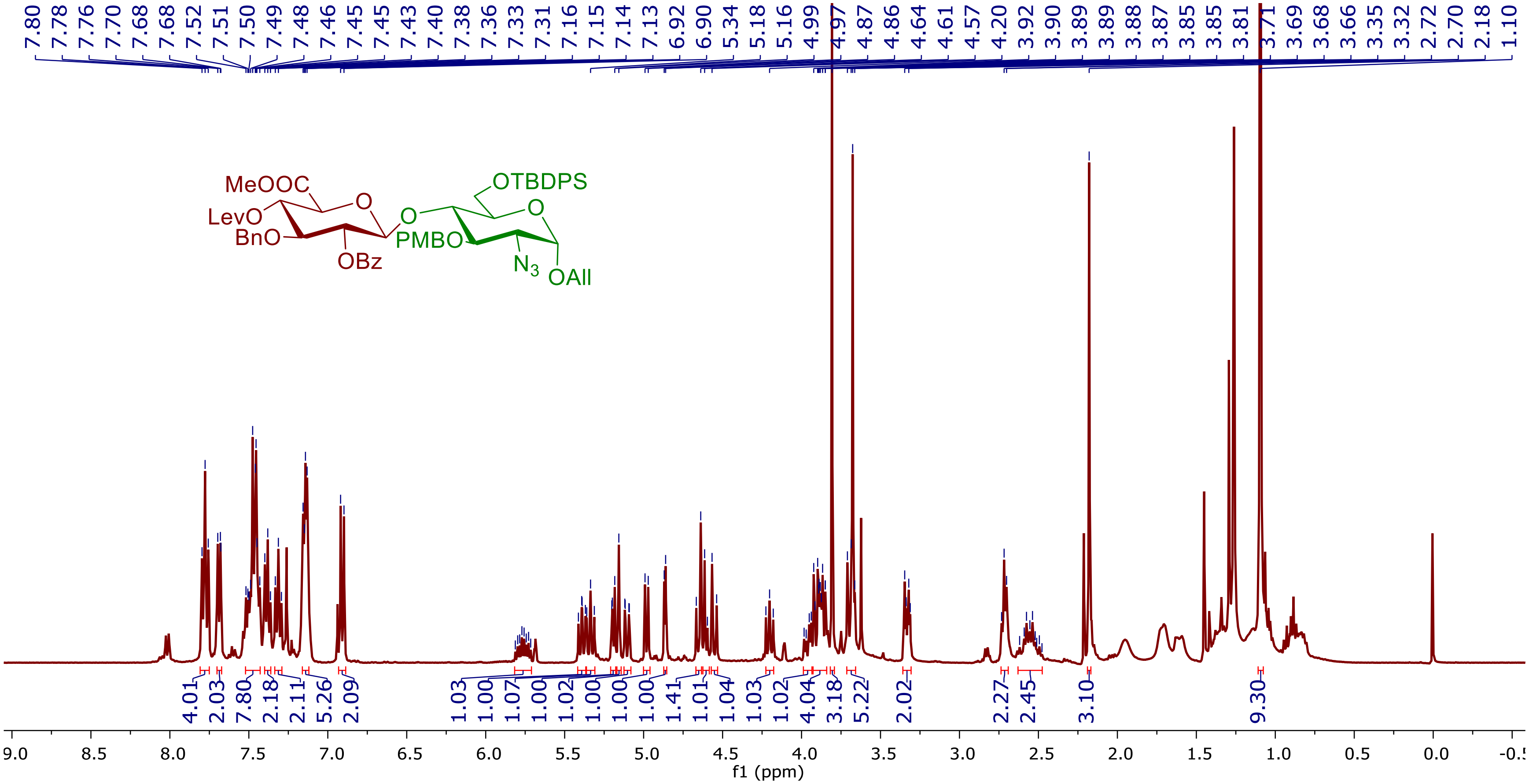

Supplementary Figure S92b. ^{13}C NMR Spectrum (100.67 MHz, $CDCl_3$) of Compound 43

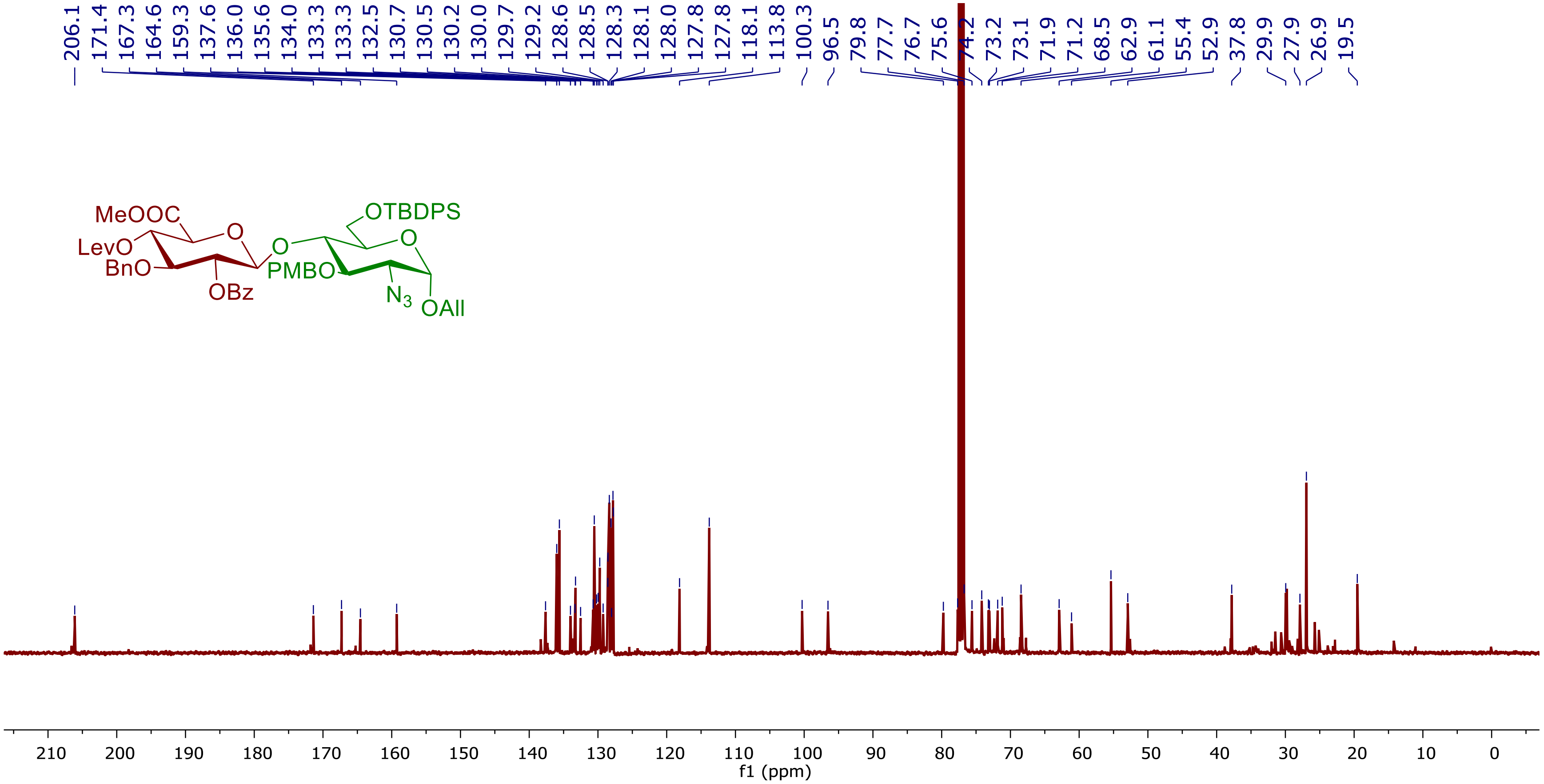

Supplementary Figure S92c. DEPT NMR Spectrum (100.67 MHz, $CDCl_3$) of Compound 43

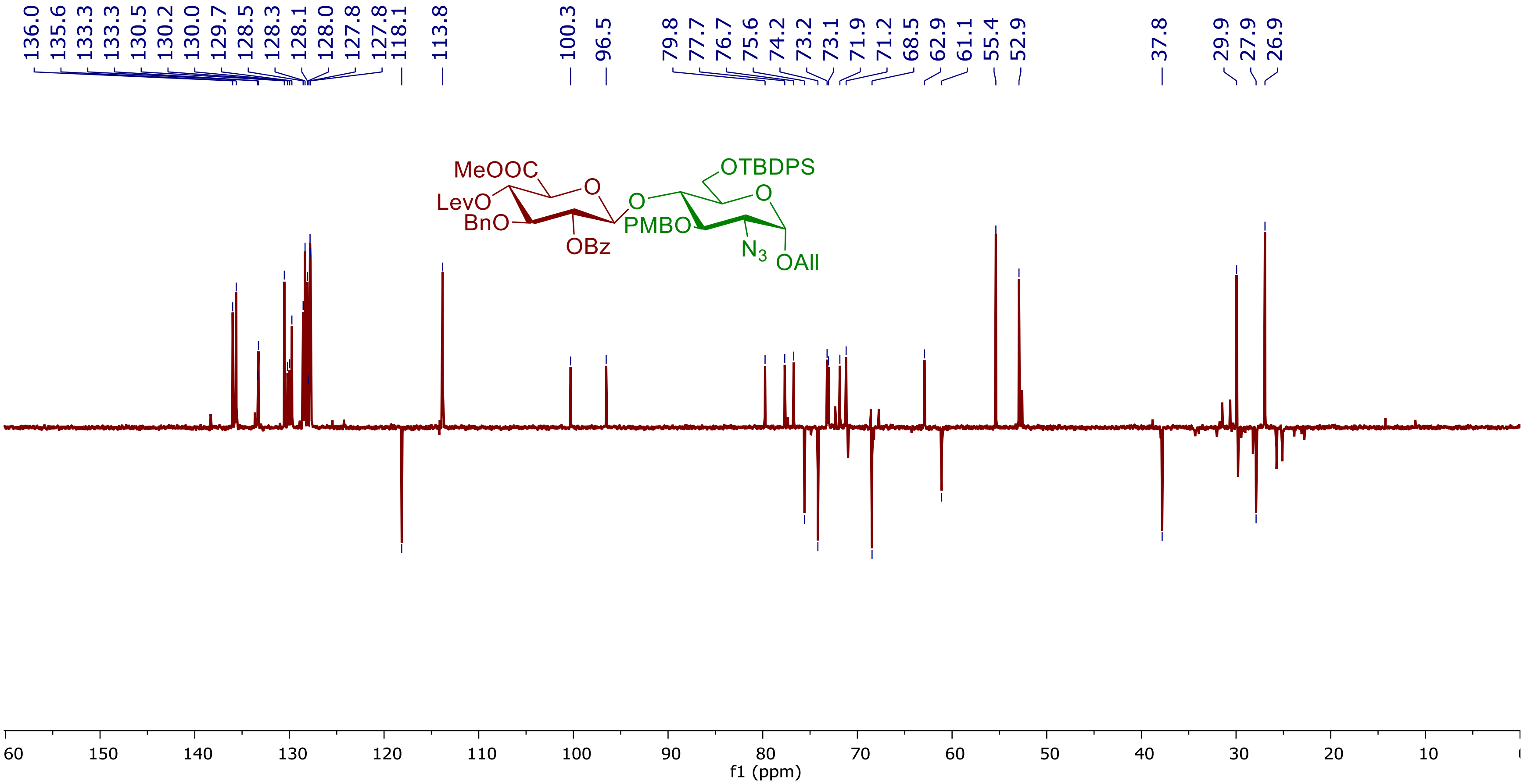

Supplementary Figure S93a. ^{1}H NMR Spectrum (400.31 MHz, $CDCl_3$) of Compound 44

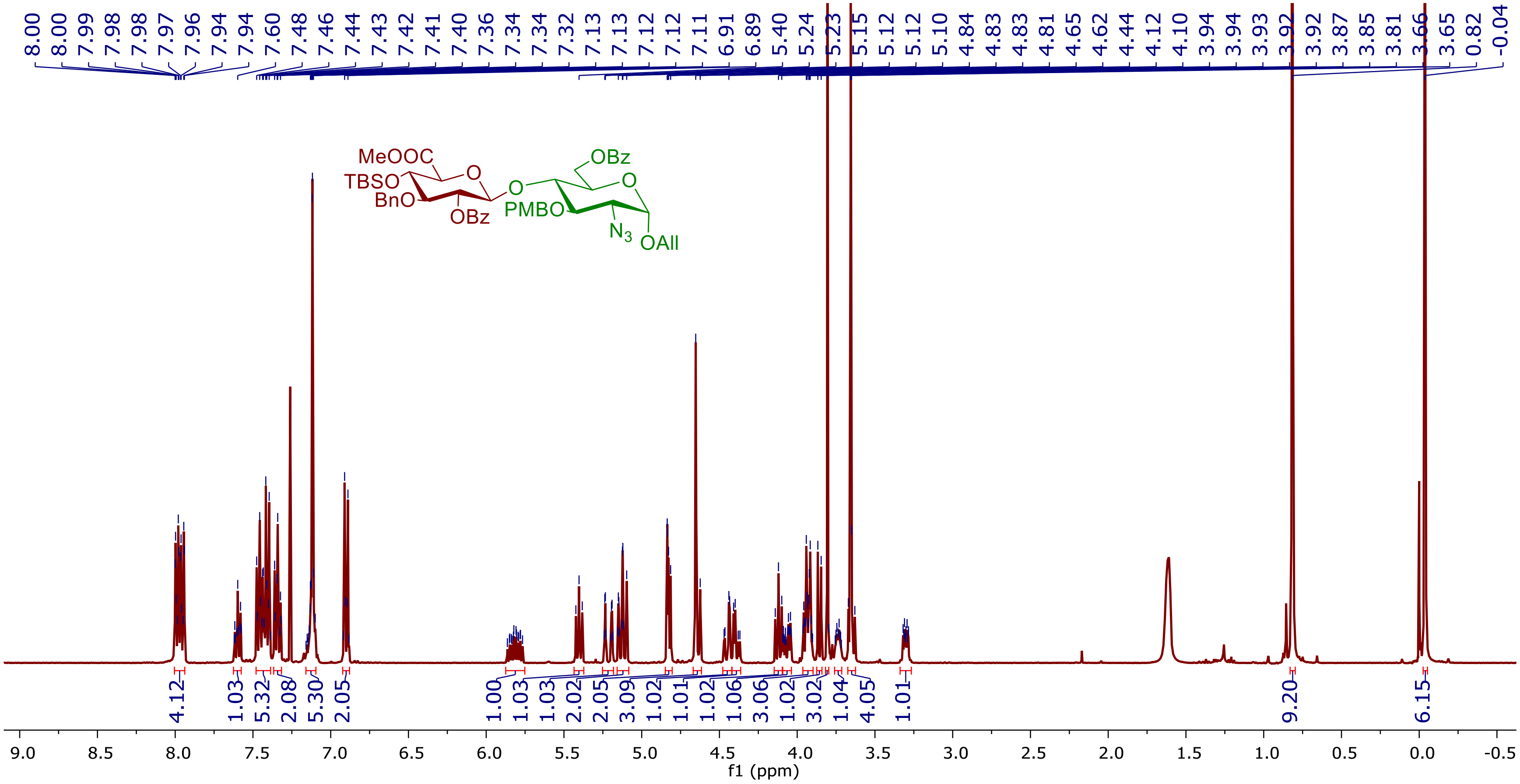

Supplementary Figure S93b. ^{13}C NMR Spectrum (100.67 MHz, $CDCl_3$) of Compound 44

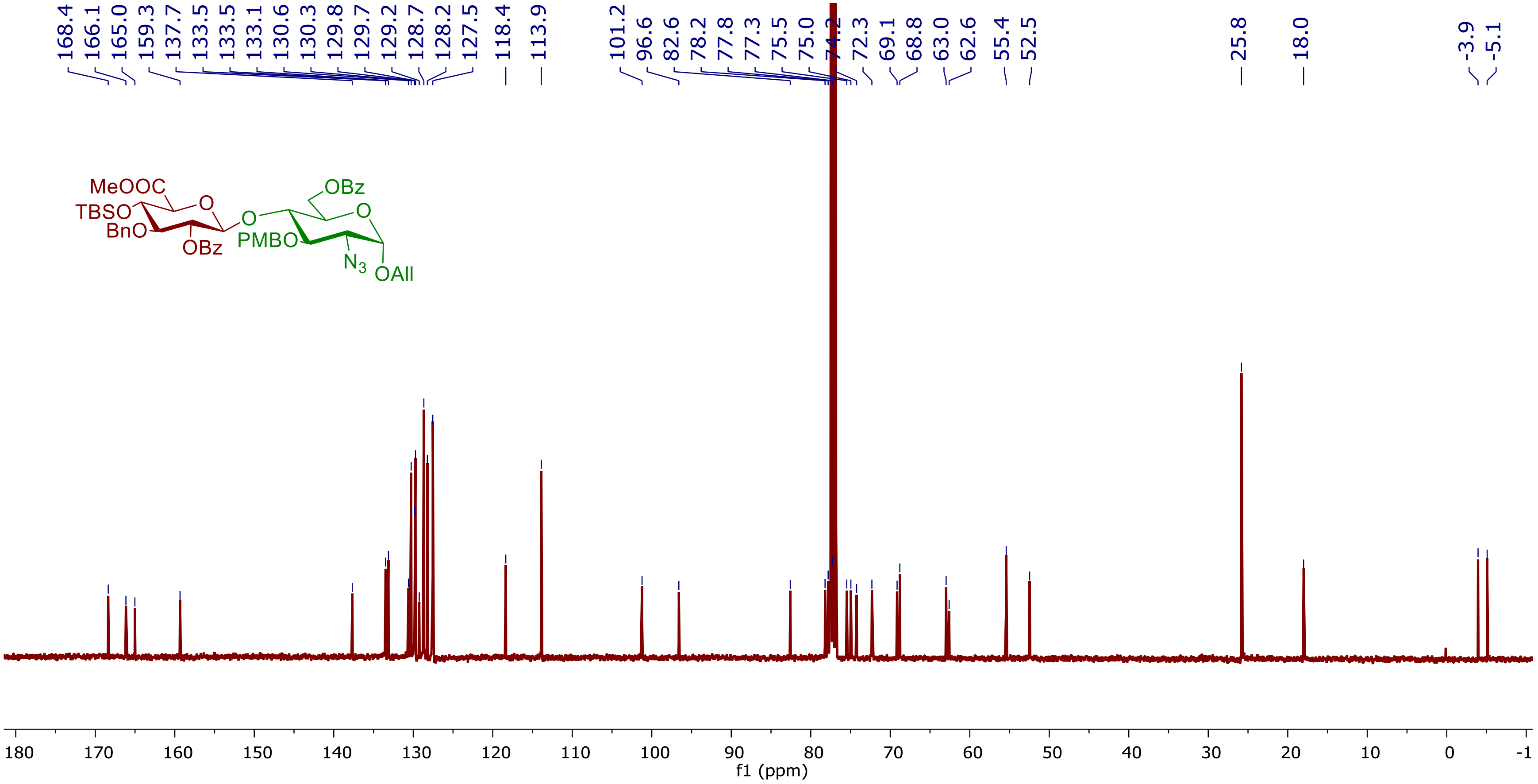

Supplementary Figure S93c. DEPT NMR Spectrum (100.67 MHz, $CDCl_3$) of Compound 44

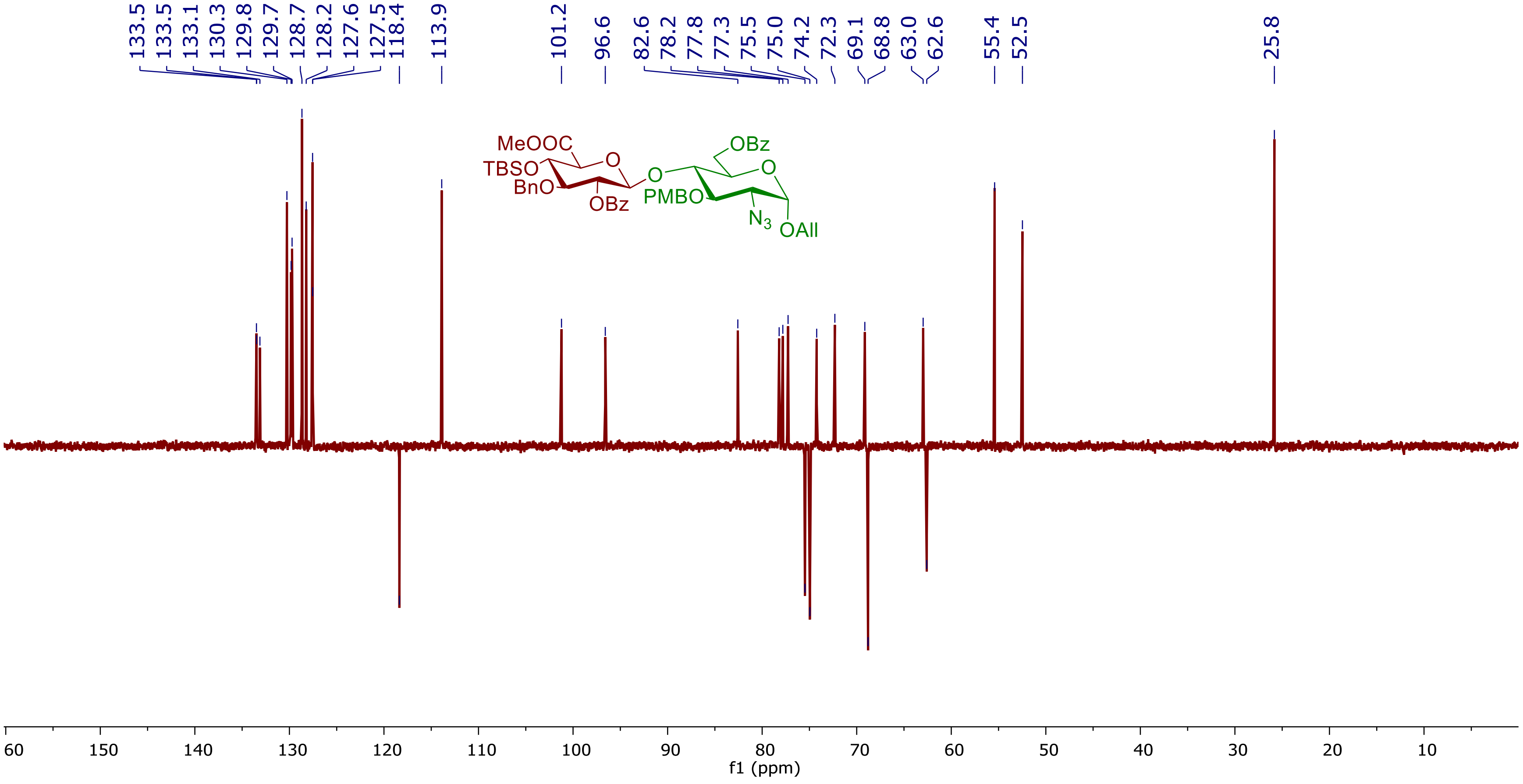

Supplementary Figure S94a. ^{1}H NMR Spectrum (400.31 MHz, $CDCl_3$) of Compound 45

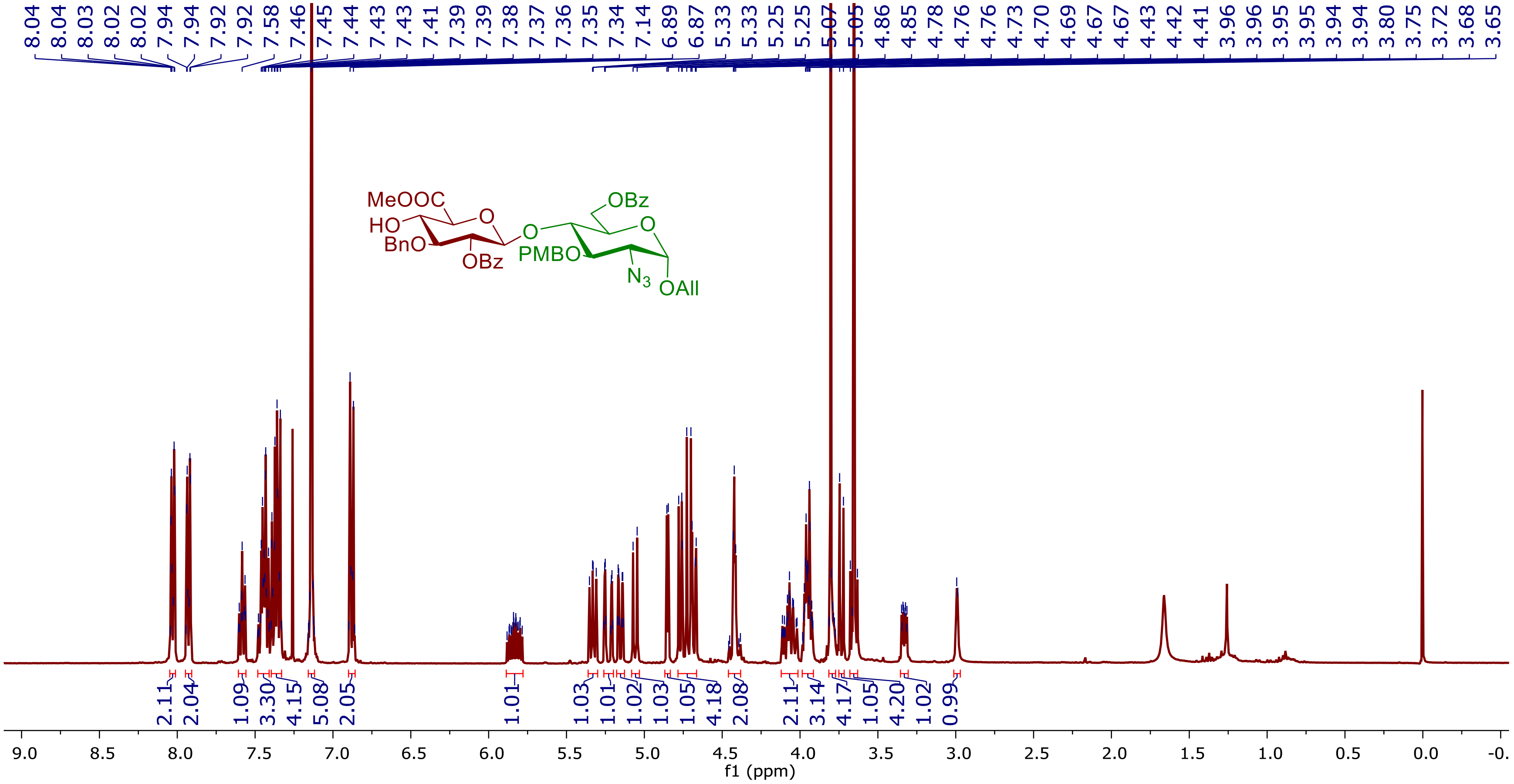

Supplementary Figure S94b. ^{13}C NMR Spectrum (100.67 MHz, $CDCl_3$) of Compound 45

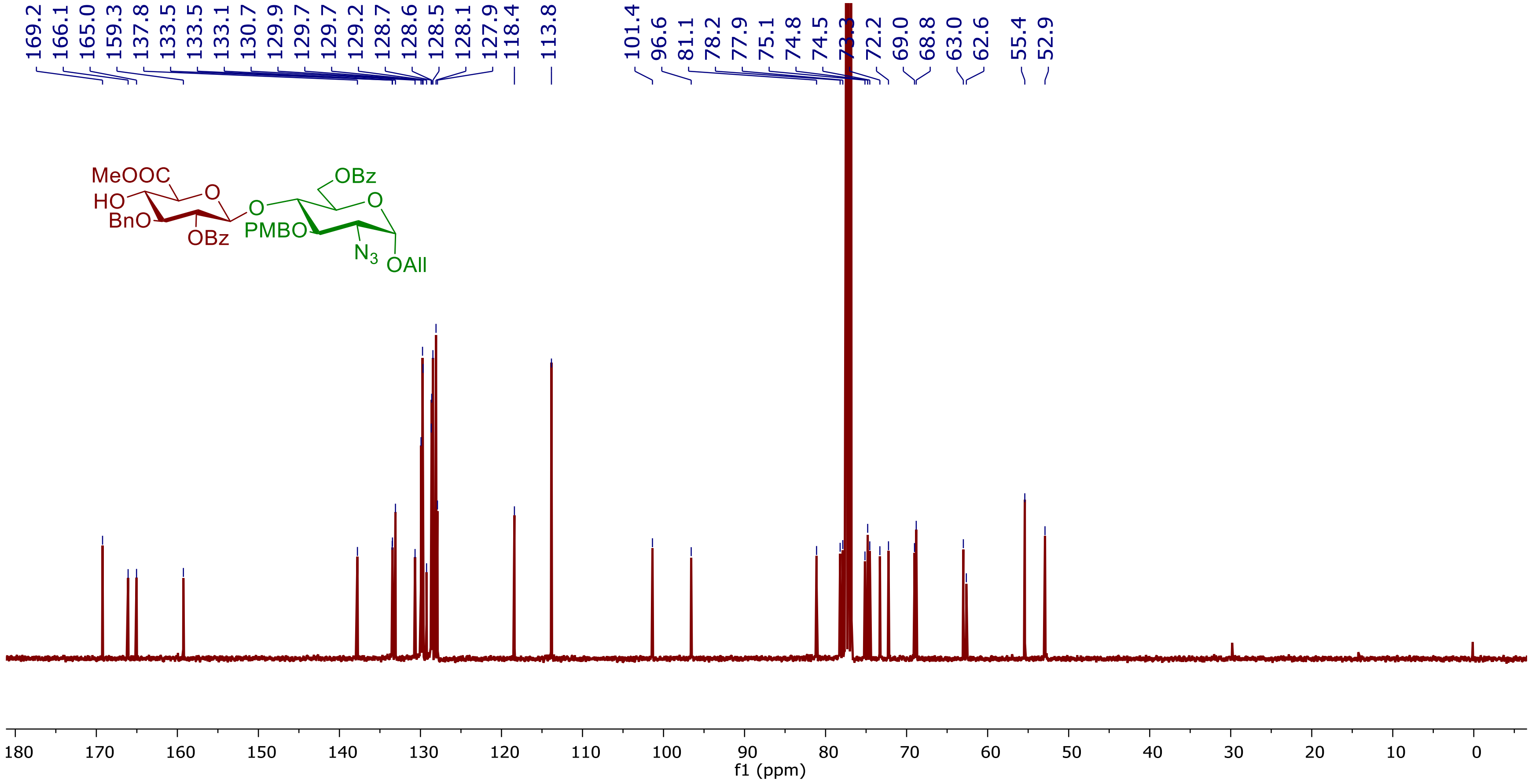

Supplementary Figure S94c. DEPT NMR Spectrum (100.67 MHz, $CDCl_3$) of Compound 45

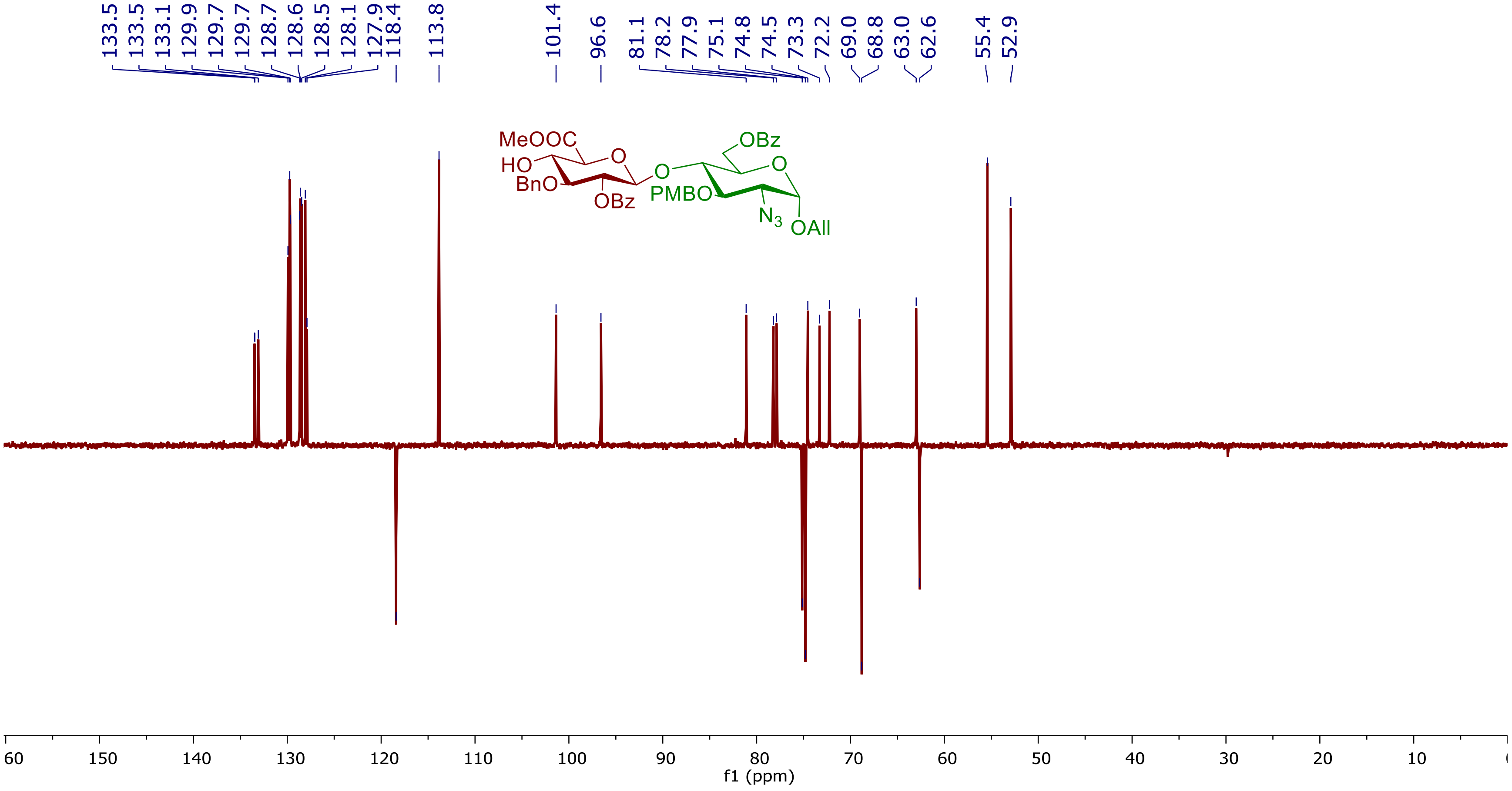

Supplementary Figure S95a. ^{1}H NMR Spectrum (400.31 MHz, $CDCl_3$) of Compound 46

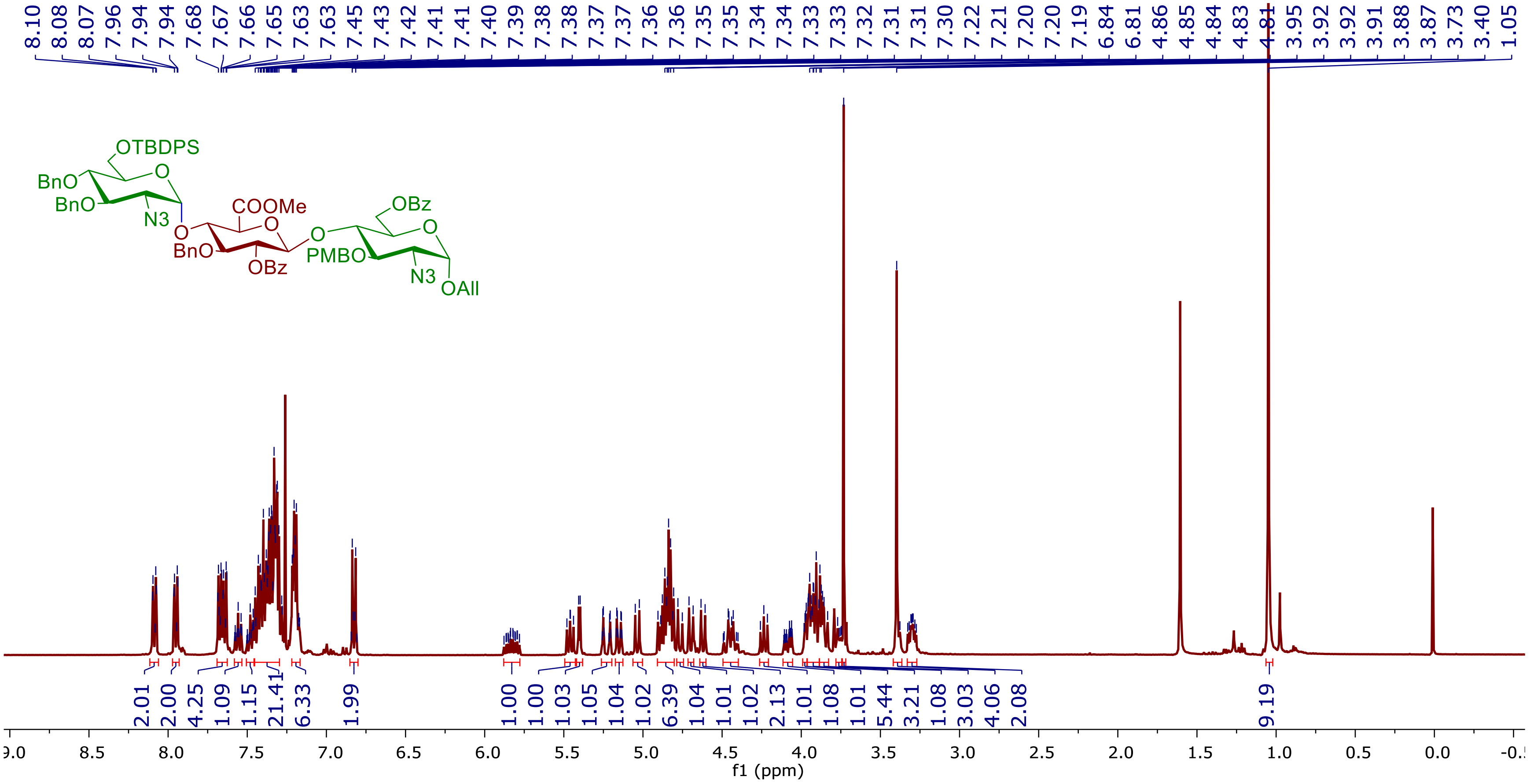

Supplementary Figure S95b. ^{13}C NMR Spectrum (100.67 MHz, $CDCl_3$) of Compound 46

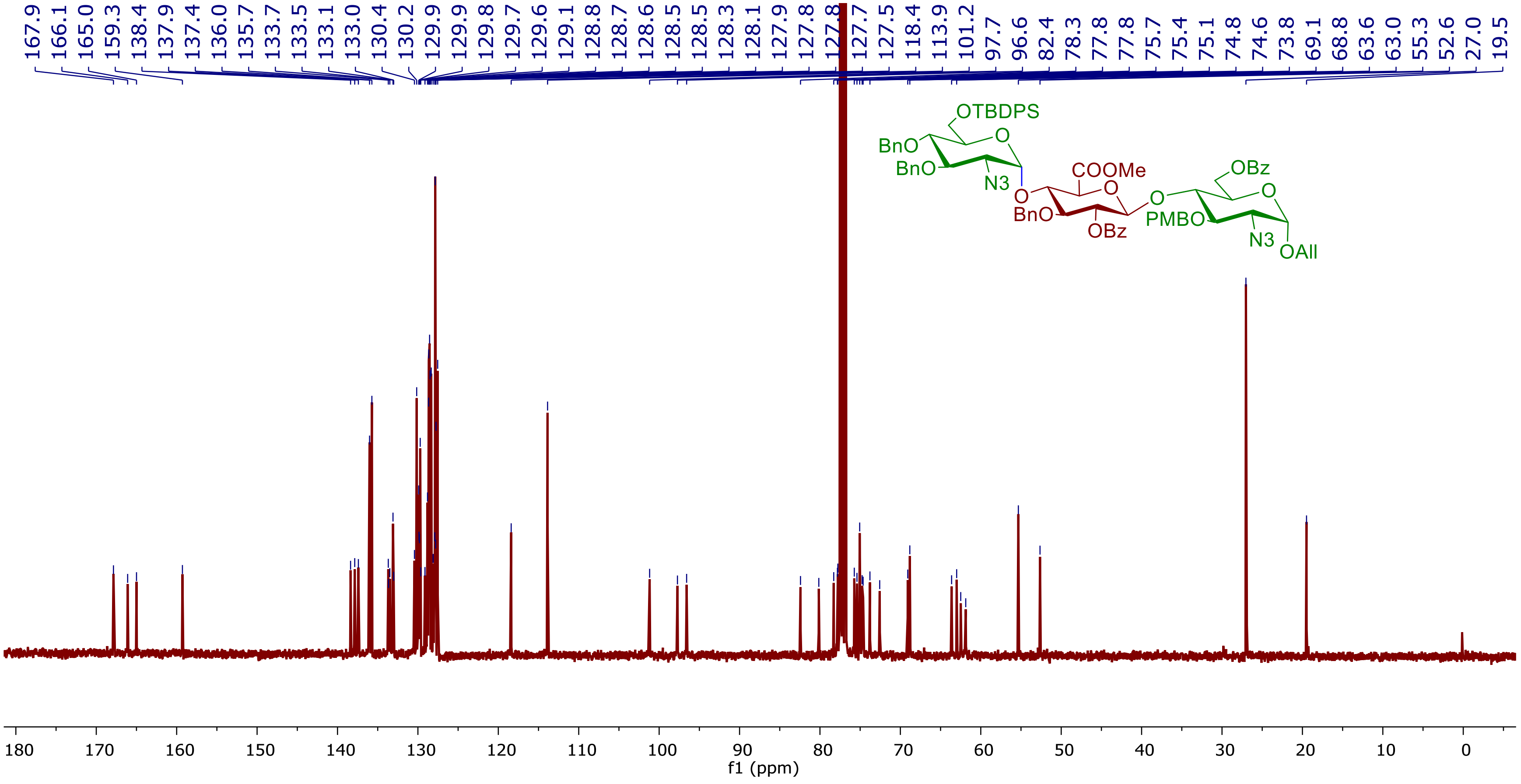

Supplementary Figure S95c. DEPT NMR Spectrum (100.67 MHz, $CDCl_3$) of Compound 46

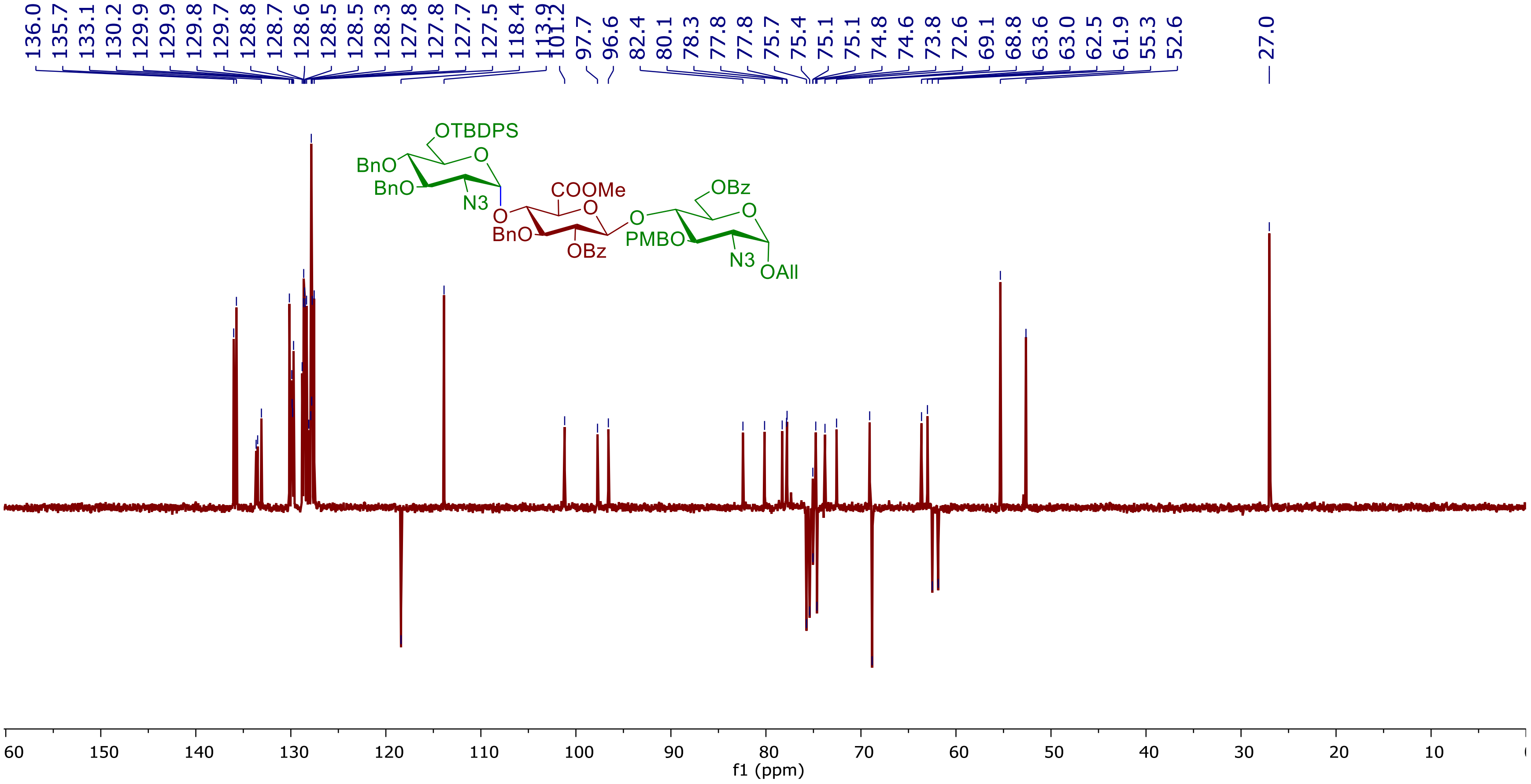

Supplementary Figure S95d. COSY NMR Spectrum (600.40, 600.40 MHz, $CDCl_3$) of Compound 46

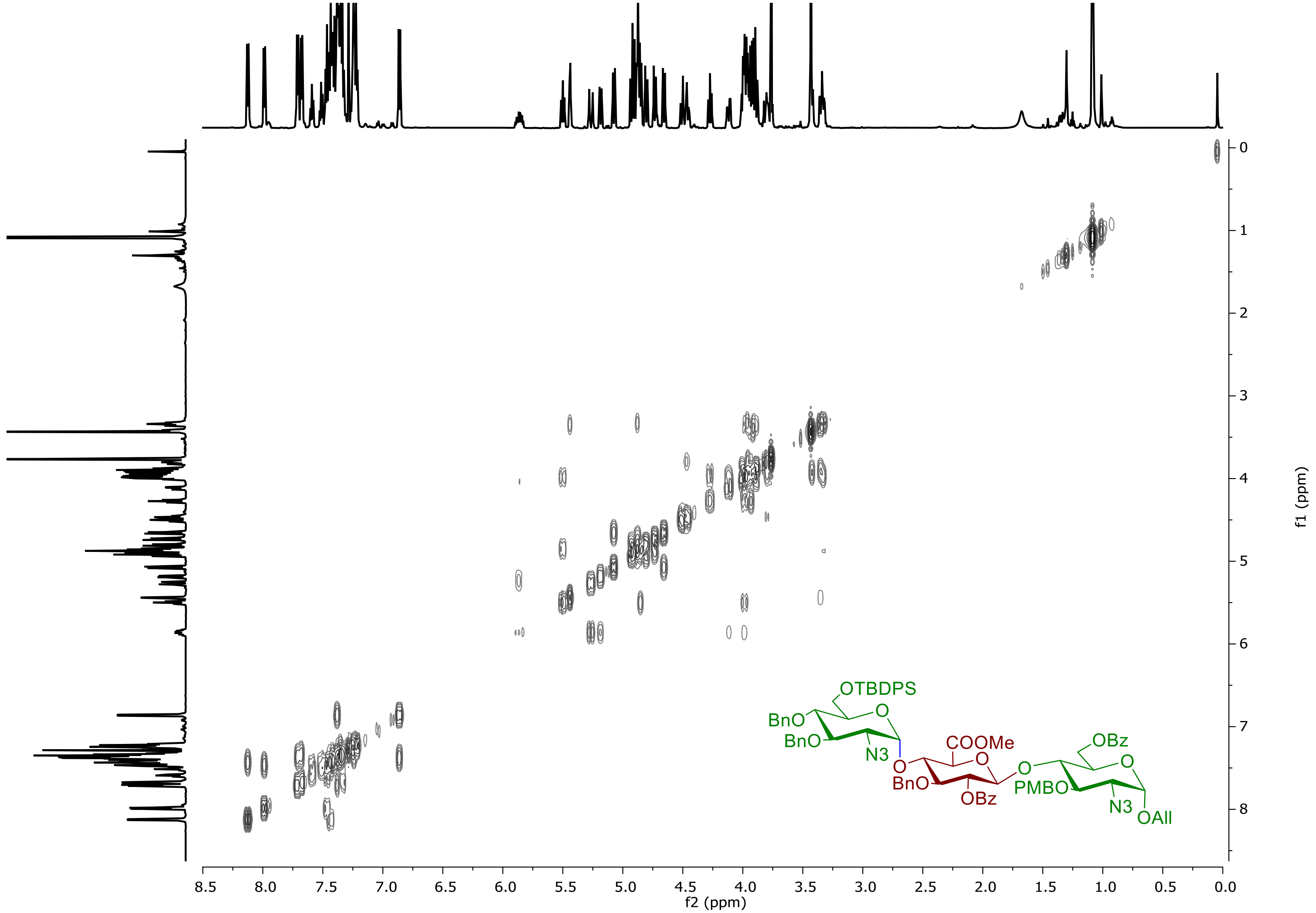

Supplementary Figure S95e. COSY NMR Spectrum (600.40, 600.40 MHz, $CDCl_3$) of Compound 46 (Sugar region expanded)

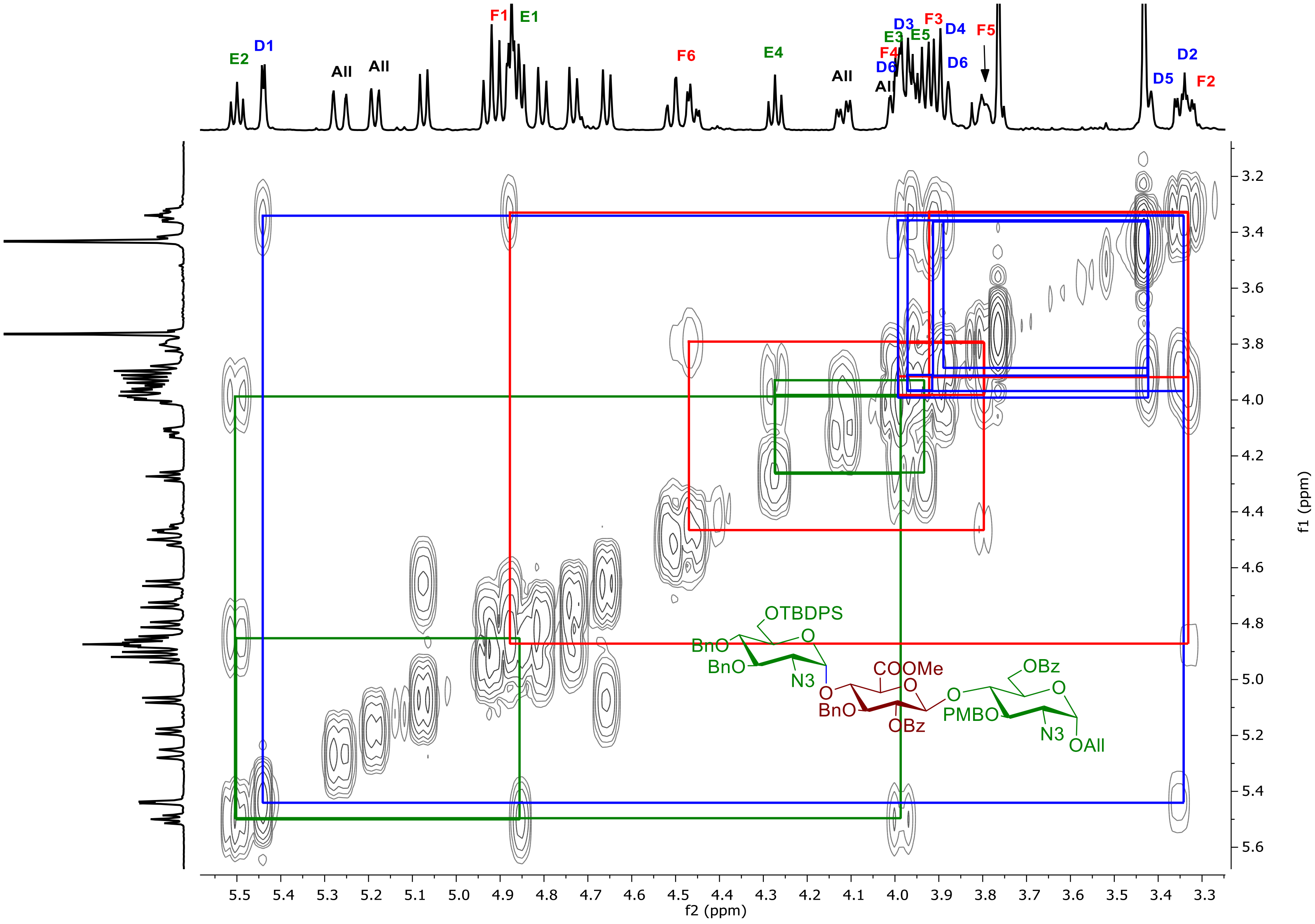

Supplementary Figure S95f. HSQC NMR Spectrum (600.40, 150.99 MHz, $CDCl_3$) of Compound 46

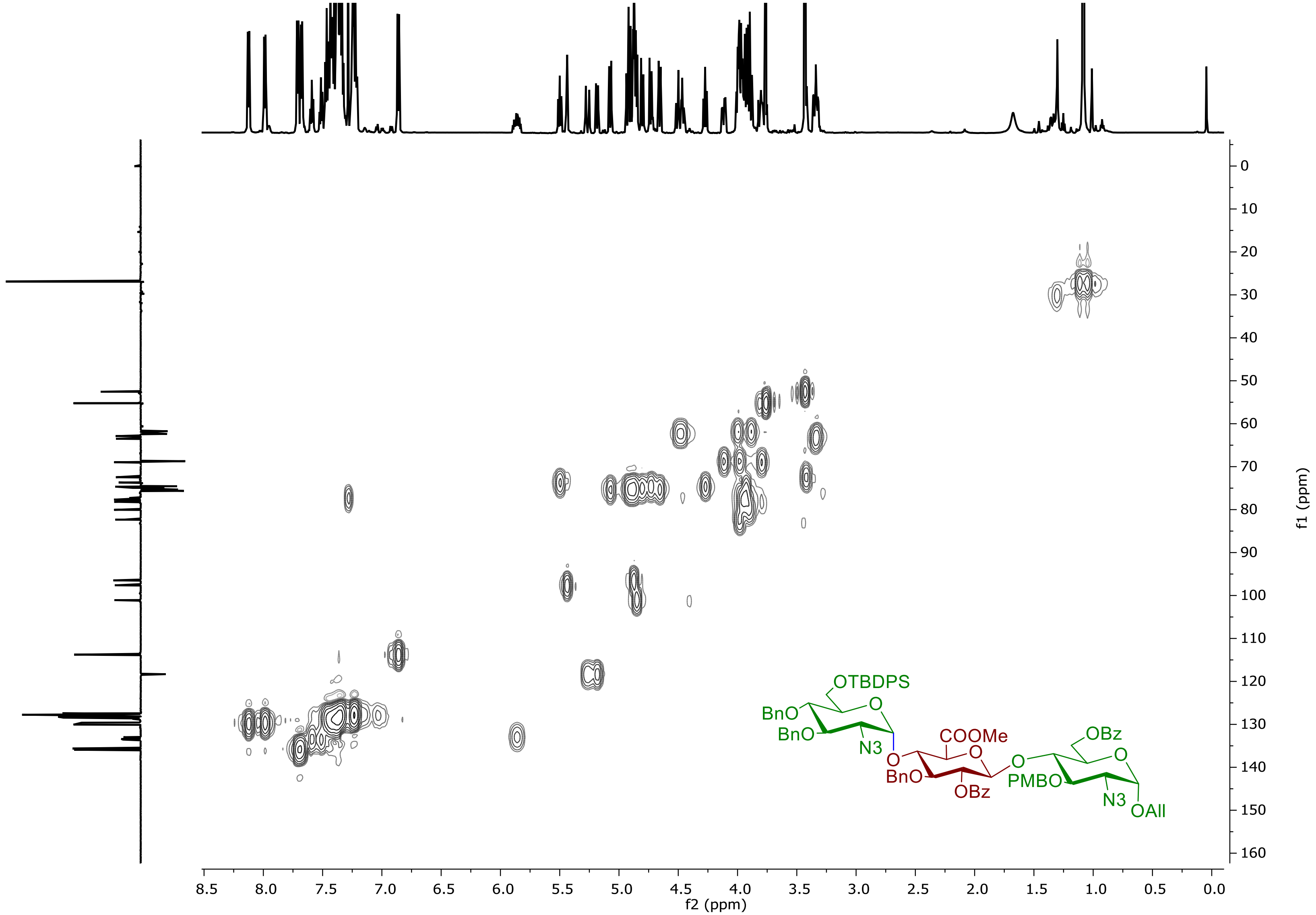

Supplementary Figure S95g. HSQC NMR Spectrum (600.40, 150.99 MHz, $CDCl_3$) of Compound 46 (Sugar region expanded)

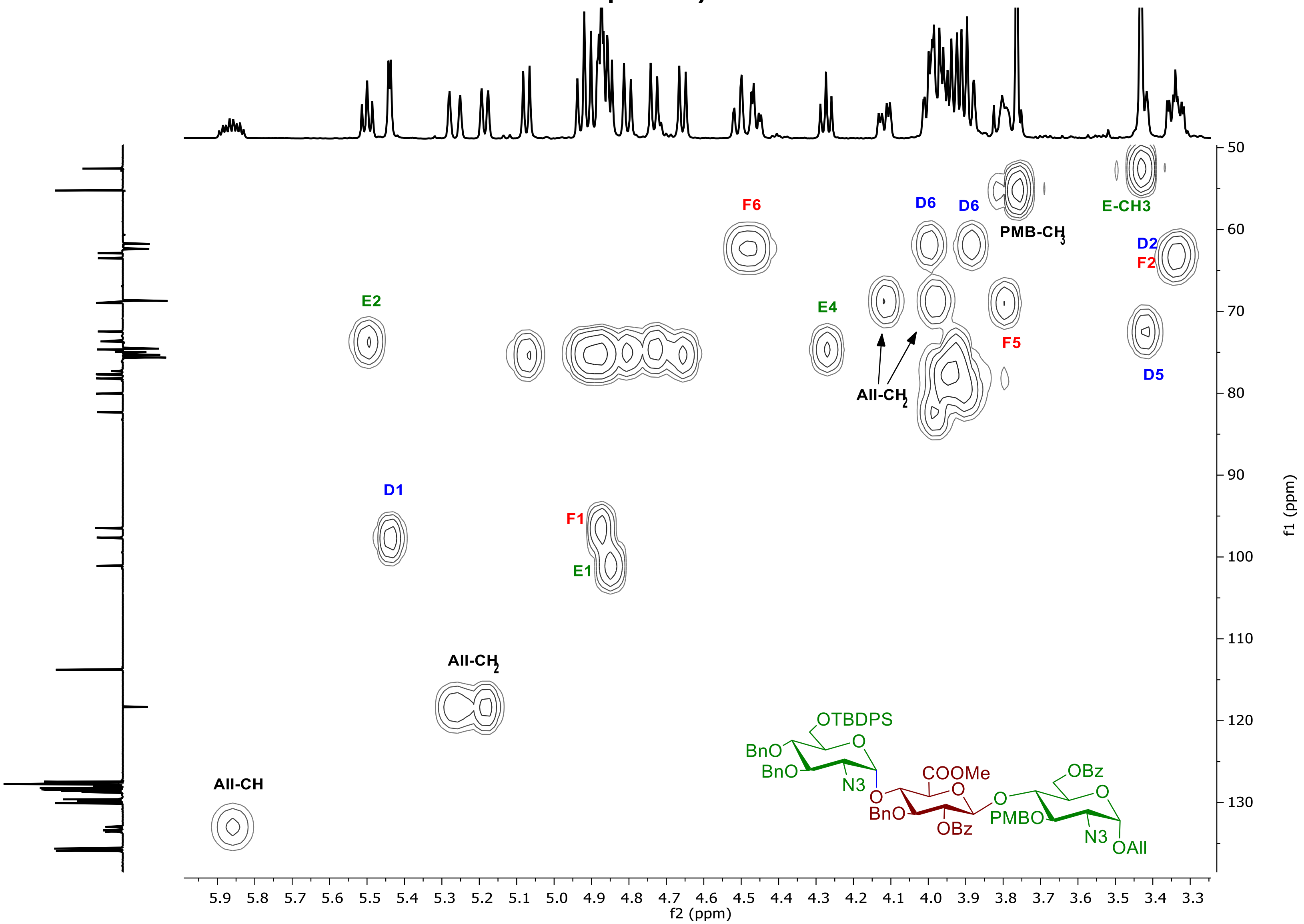

Supplementary Figure S95h. Coupled HSQC NMR Spectrum (600.40, 150.99 MHz, $CDCl_3$) of Compound 46

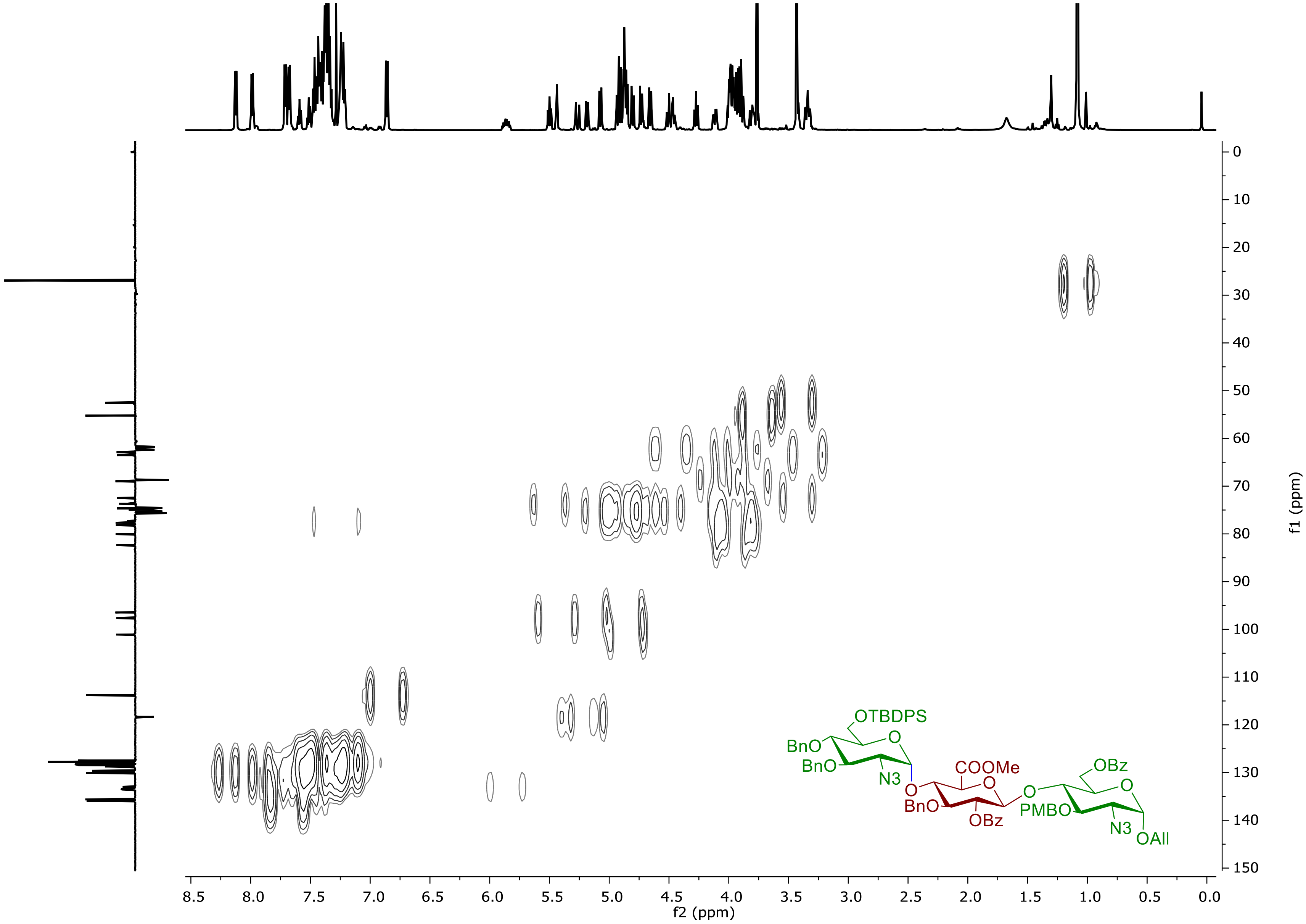

Supplementary Figure S95i. Coupled HSQC NMR Spectrum (600.40, 150.99 MHz, $CDCl_3$) of Compound 46 (Sugar region expanded)

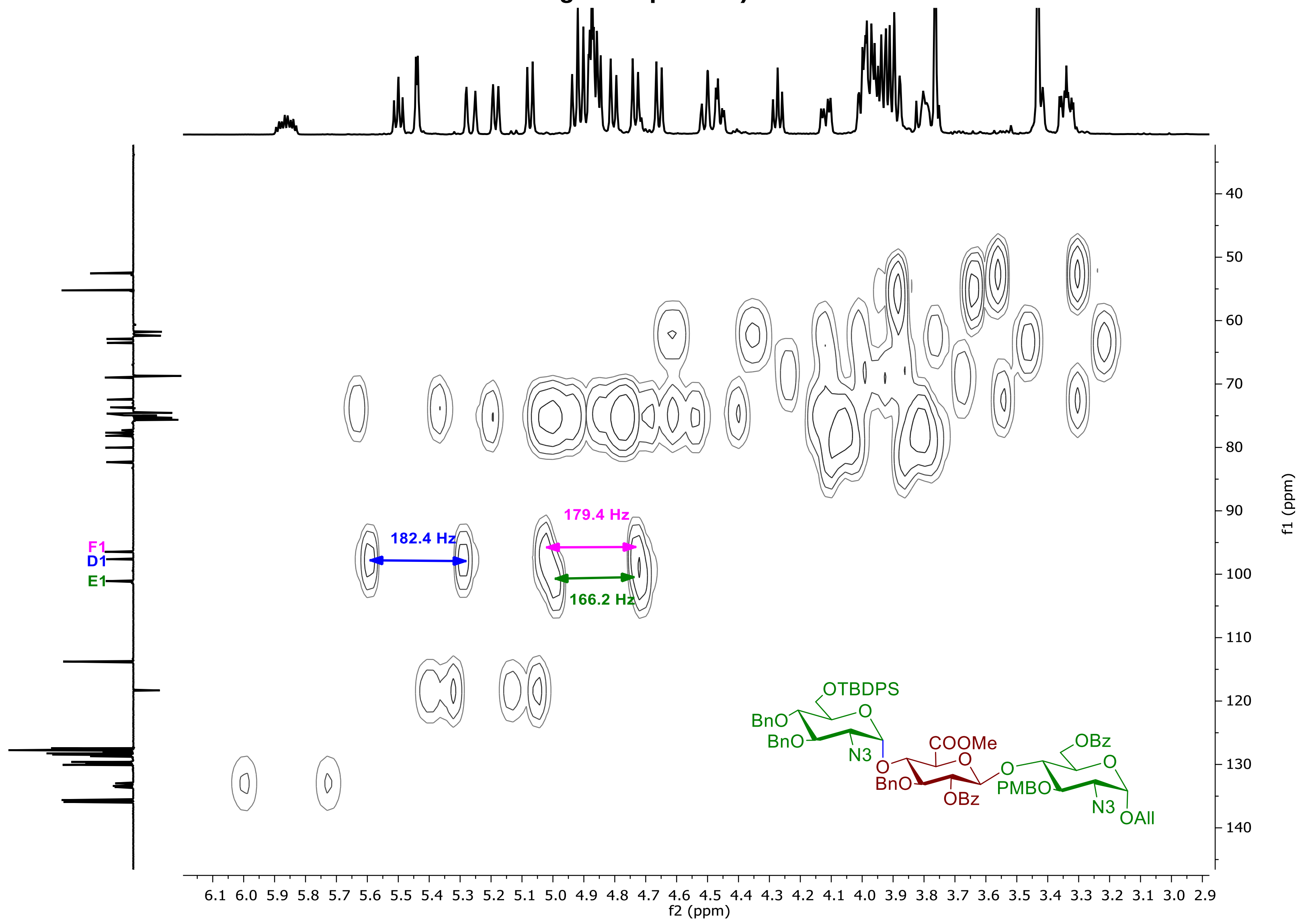

Supplementary Figure S95j. HMBC NMR Spectrum (600.40, 150.99 MHz, $CDCl_3$) of Compound 46

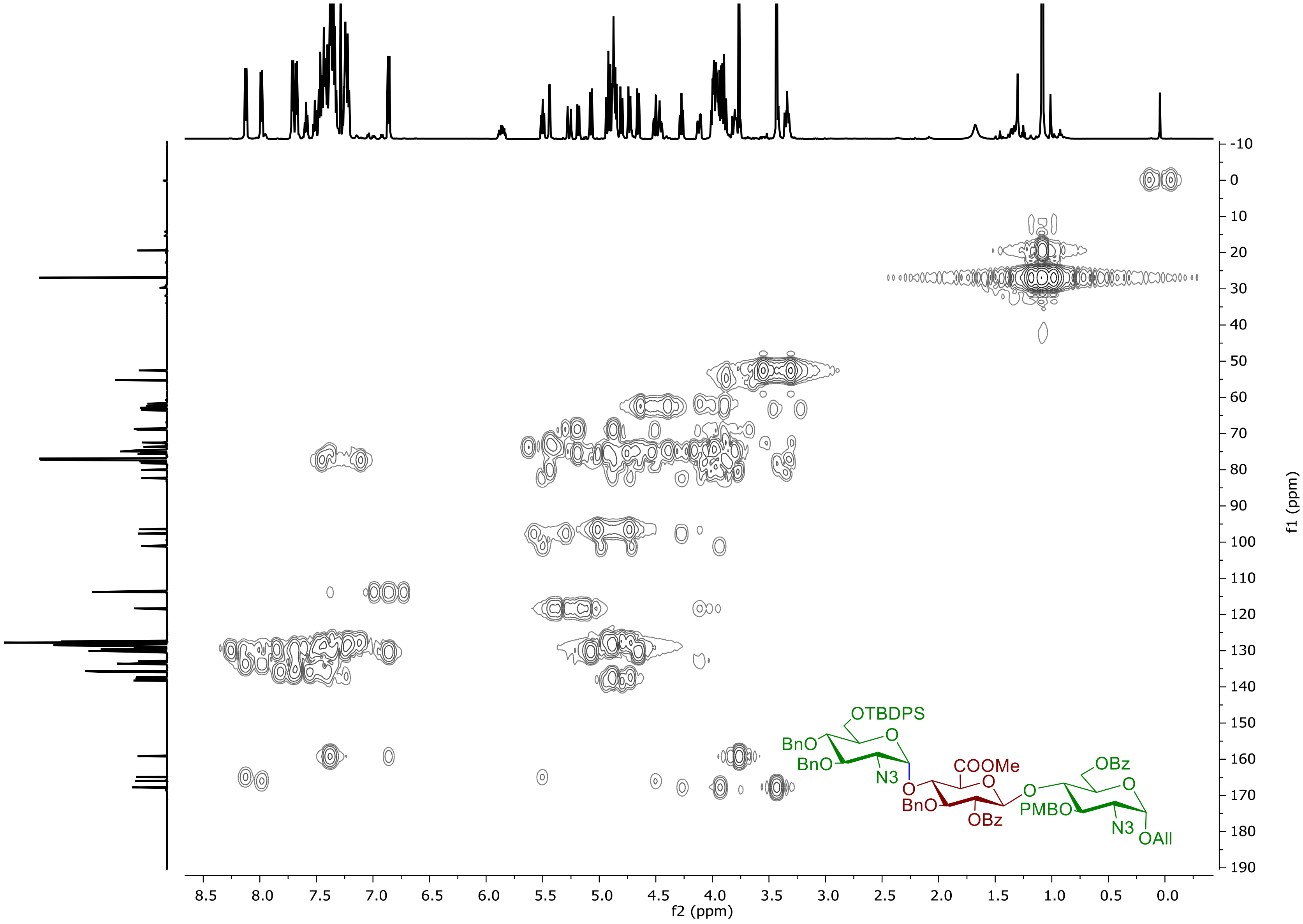

Supplementary Figure S95k. HMBC NMR Spectrum (600.40, 150.99 MHz, $CDCl_3$) of Compound 46 (Sugar region expanded)

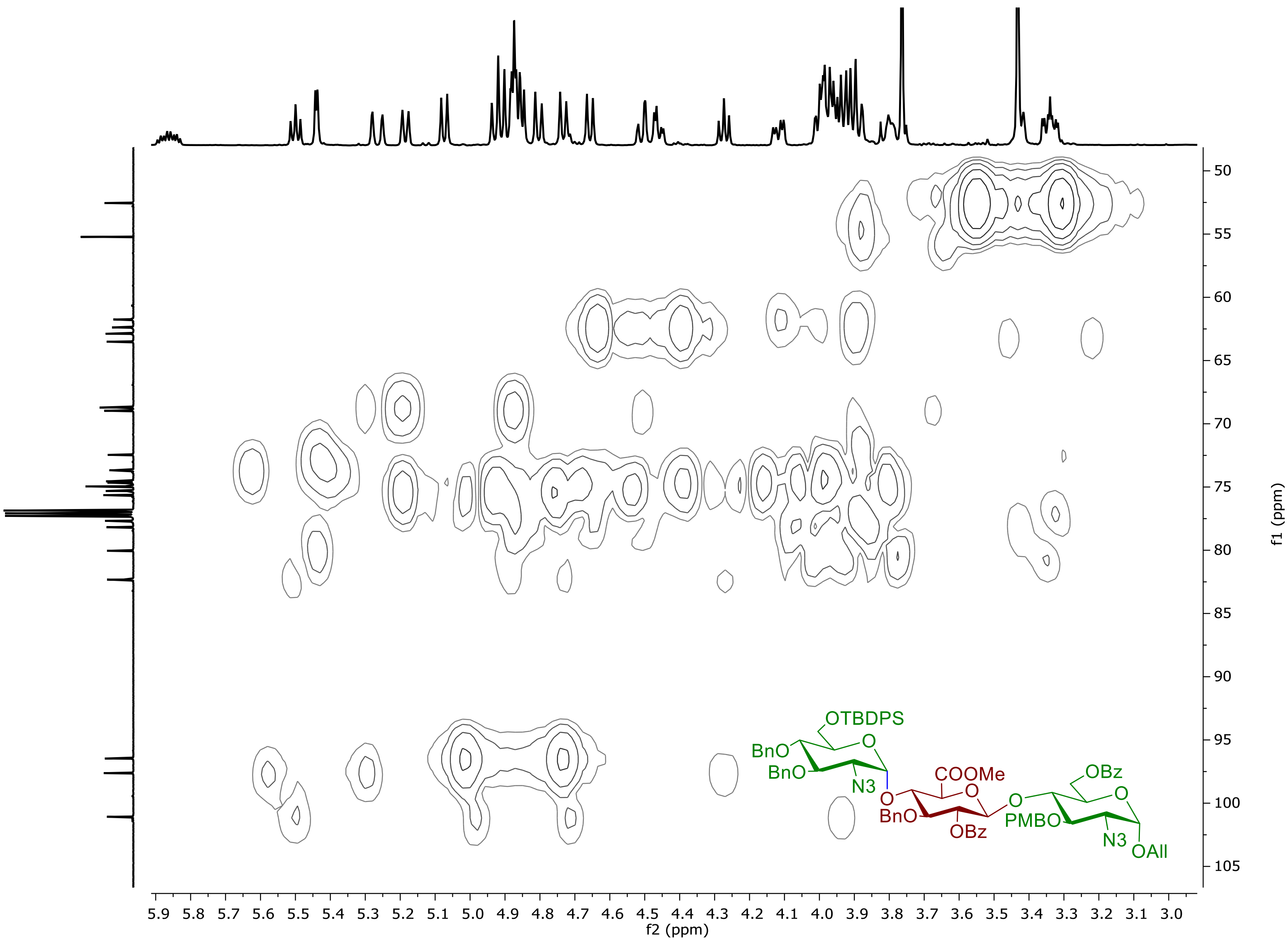

Supplementary Figure S95l. HMBC NMR Spectrum (600.40, 150.99 MHz, $CDCl_3$) of Compound 46 (Carbonyl Region expanded)

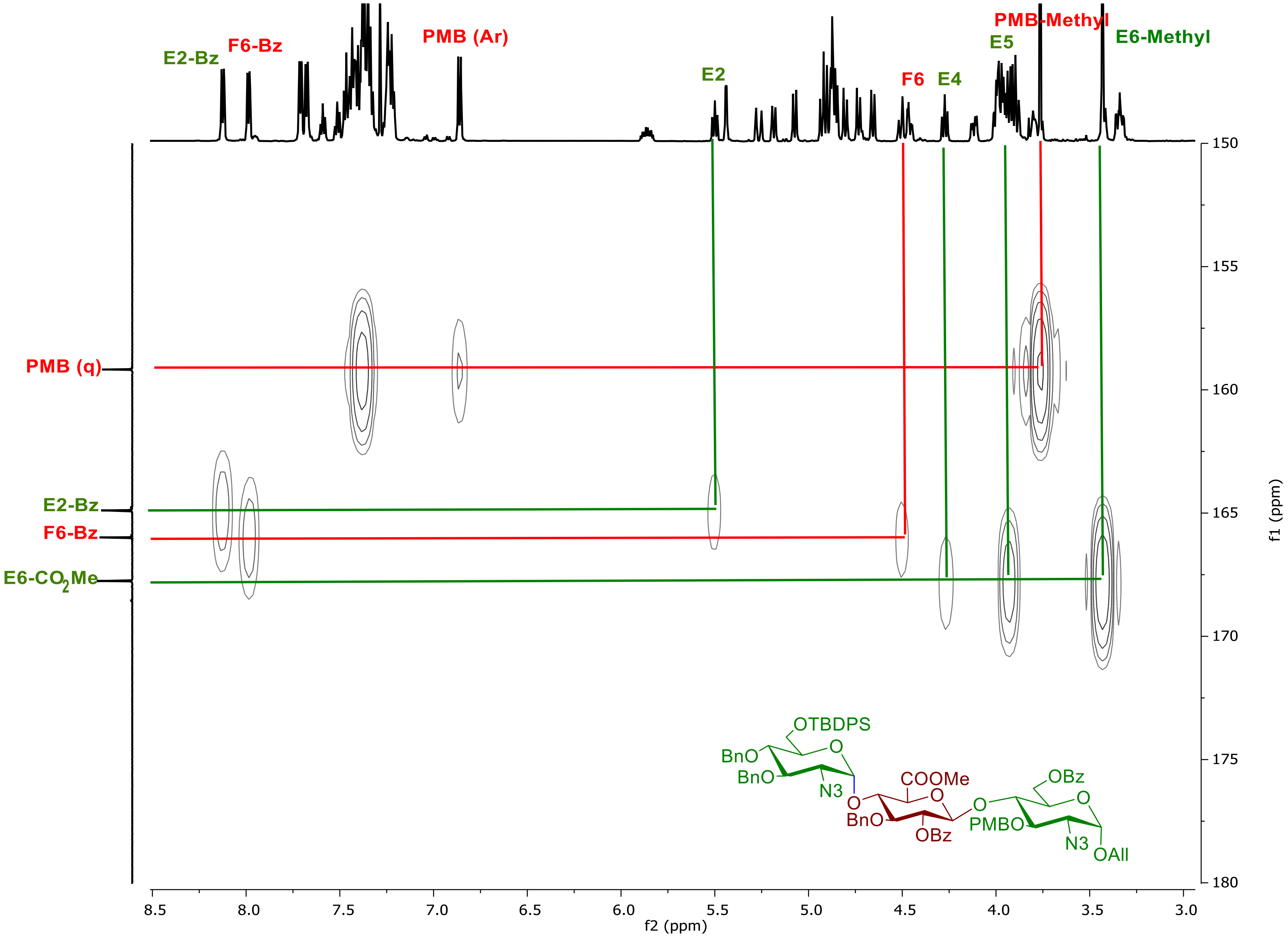

Supplementary Figure S95m. TOCSY NMR Spectrum (400.31, 400.31 MHz, $CDCl_3$) of Compound 46

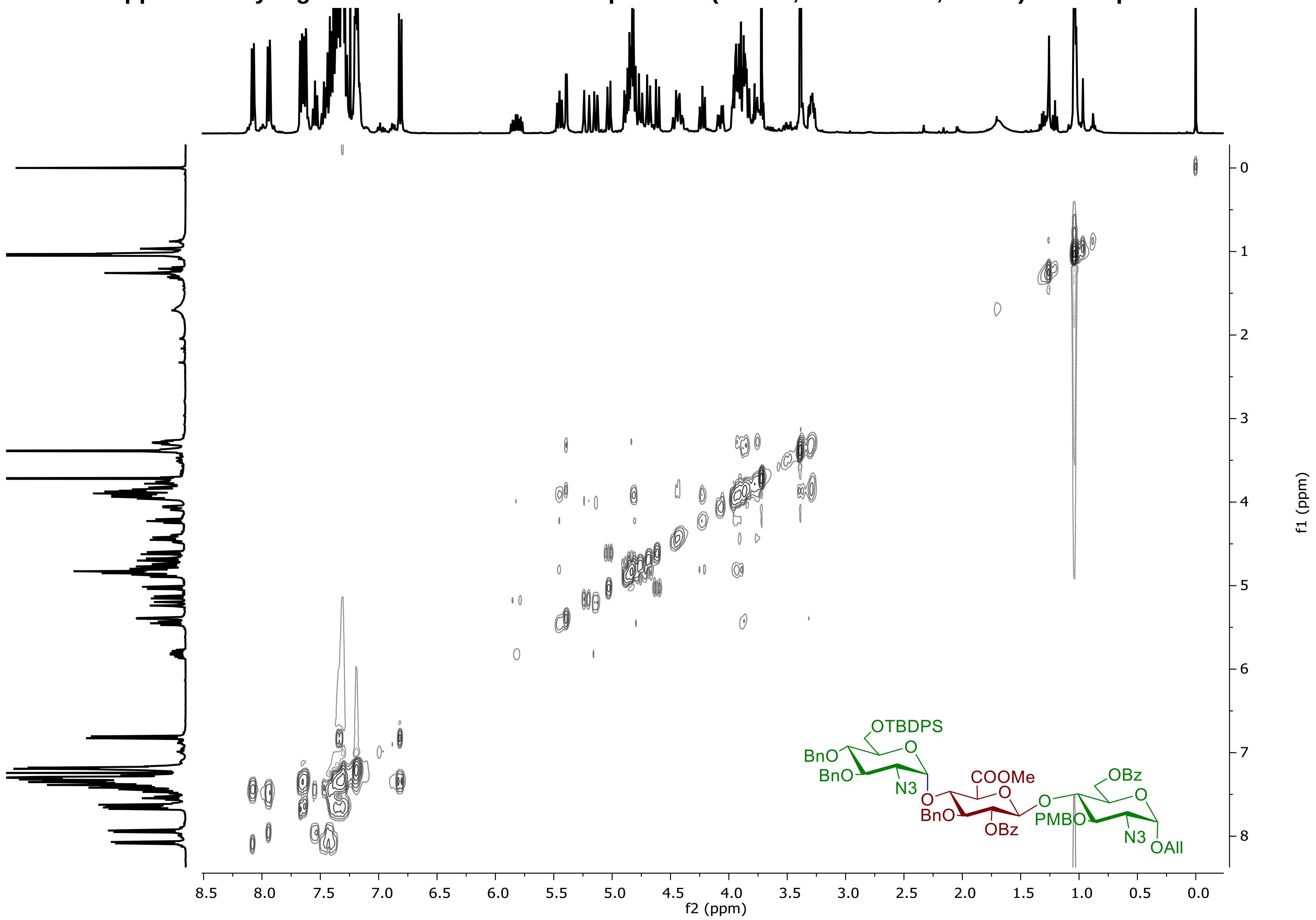

Supplementary Figure S95n. TOCSY NMR Spectrum (400.31, 400.31 MHz, $CDCl_3$) of Compound 46 (Sugar region expanded)

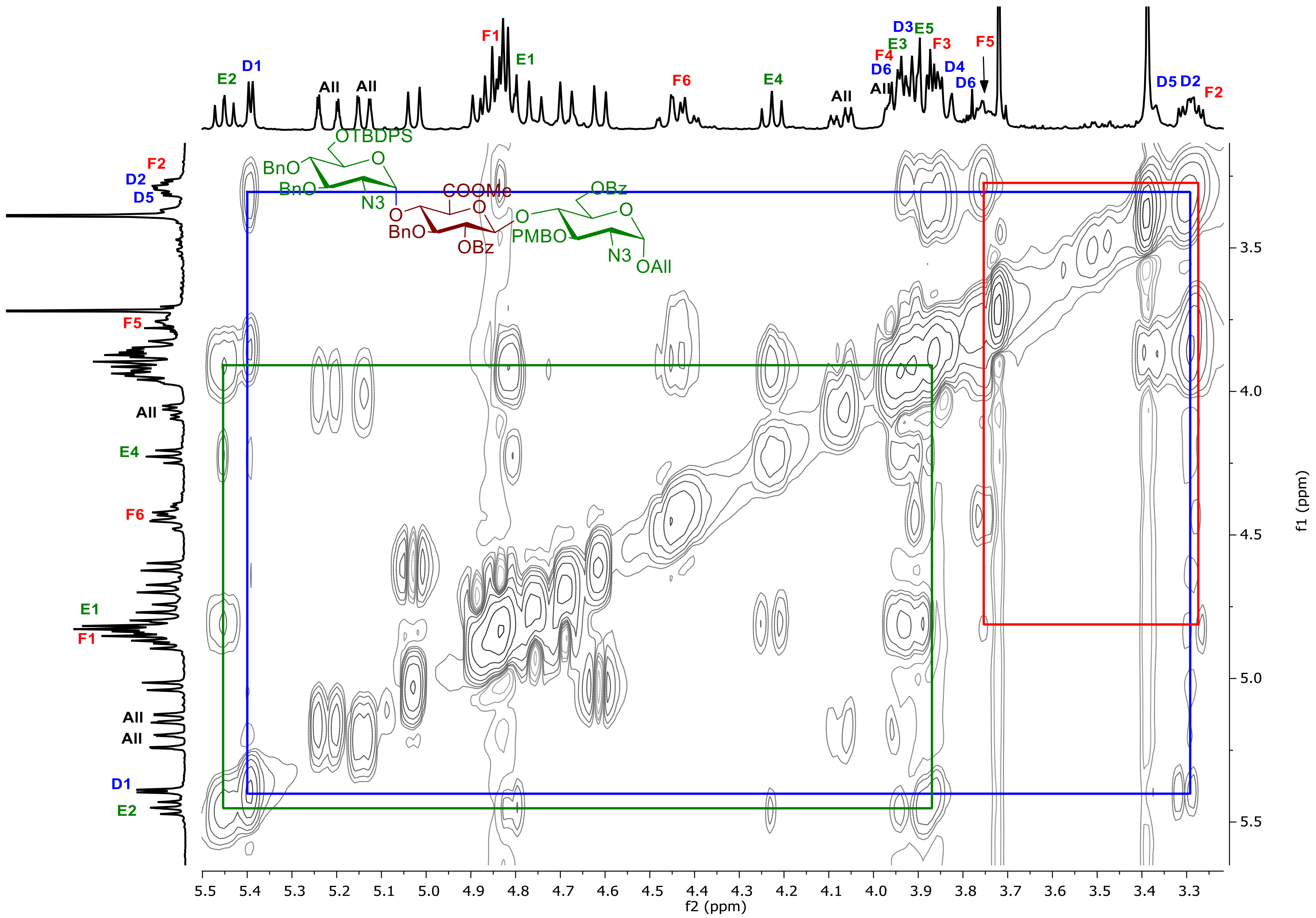

Supplementary Figure S95o. HSQC-TOCSY NMR Spectrum (400.31, 100.67 MHz, $CDCl_3$) of Compound 46

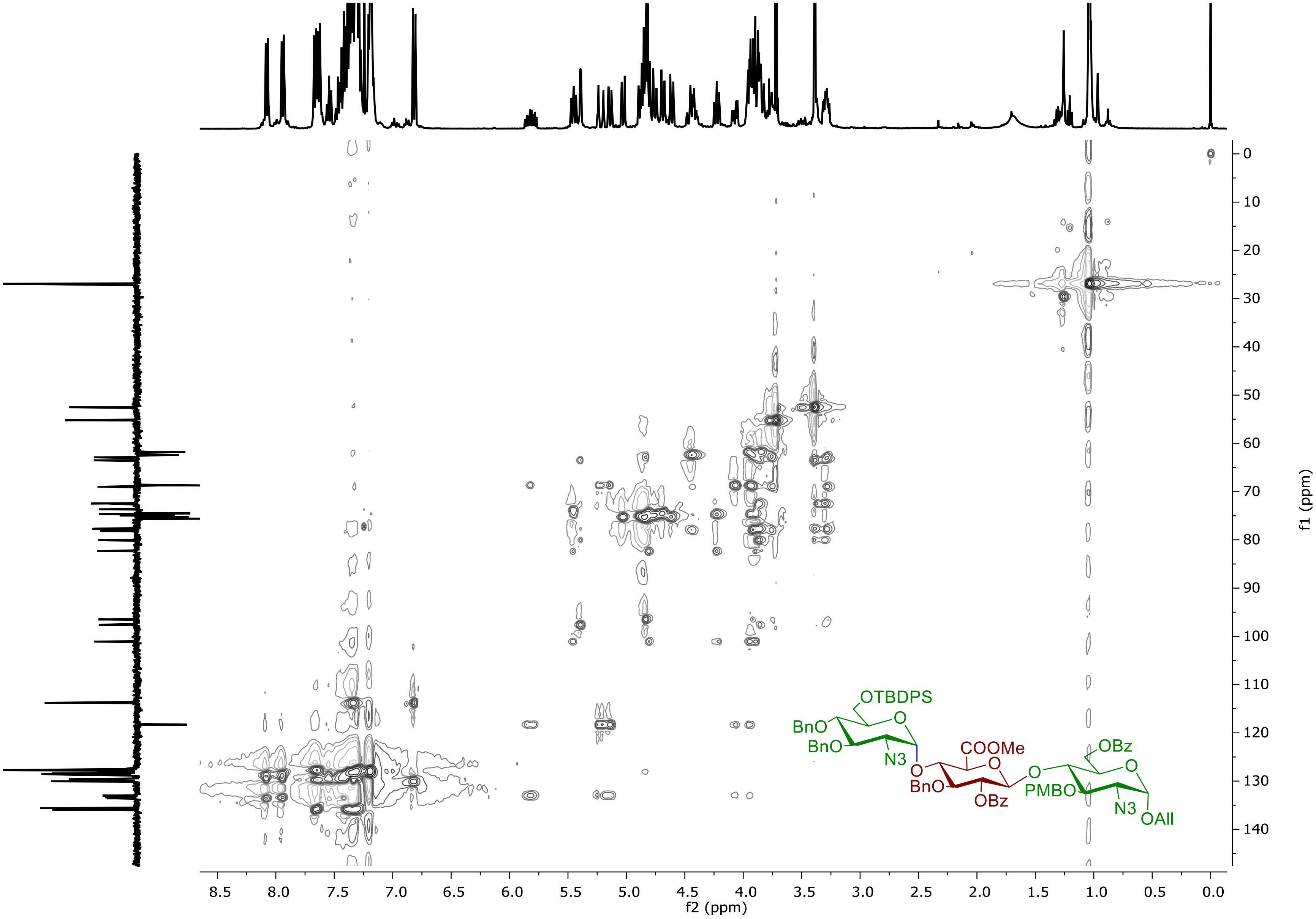

Supplementary Figure S95p. HSQC-TOCSY NMR Spectrum (400.31, 100.67 MHz, CDCl₃) of Compound 46 (Sugar region expanded)

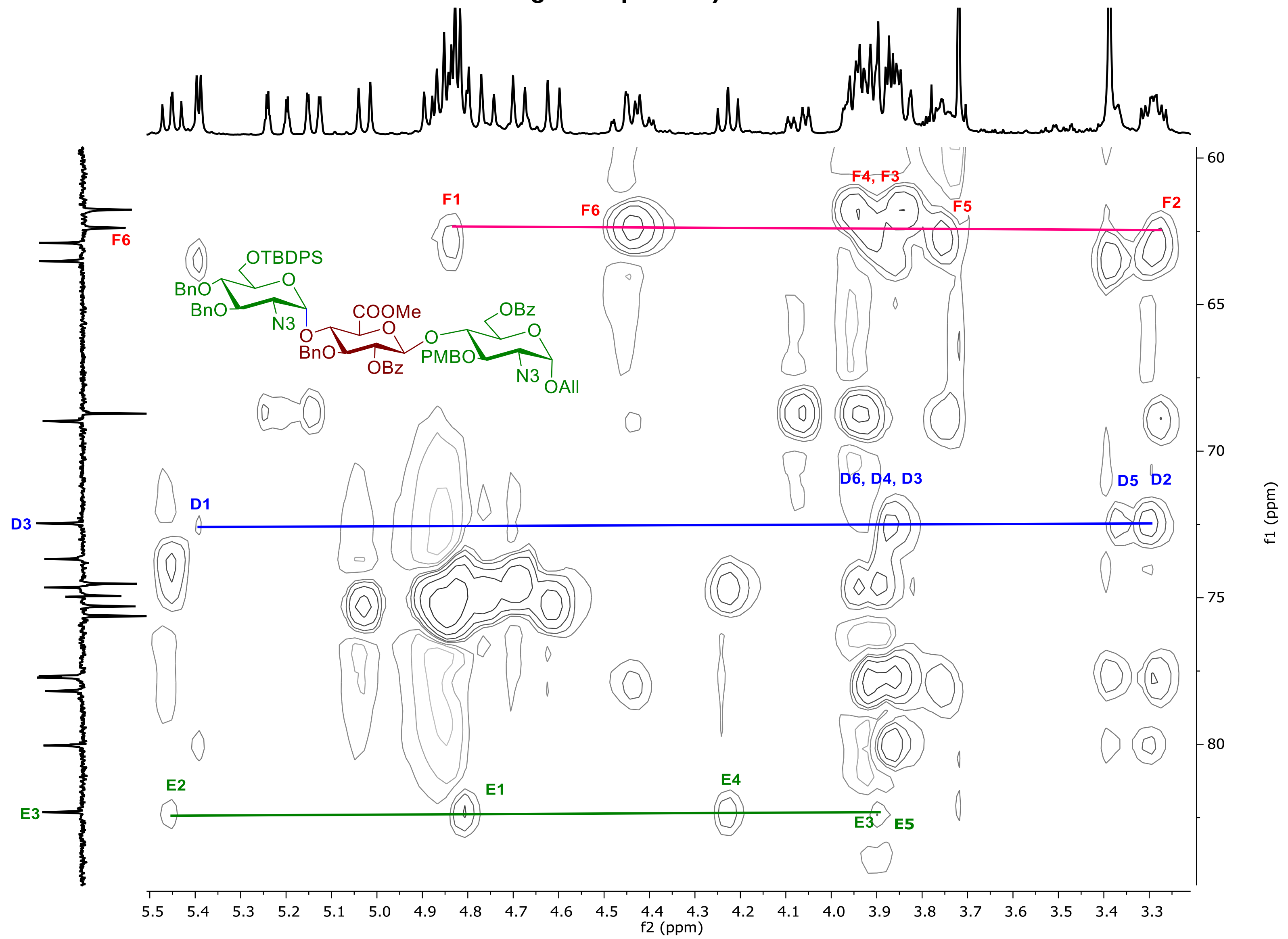

Supplementary Figure S96a. ^{1}H NMR Spectrum (400.31 MHz, $CDCl_3$) of Compound S14

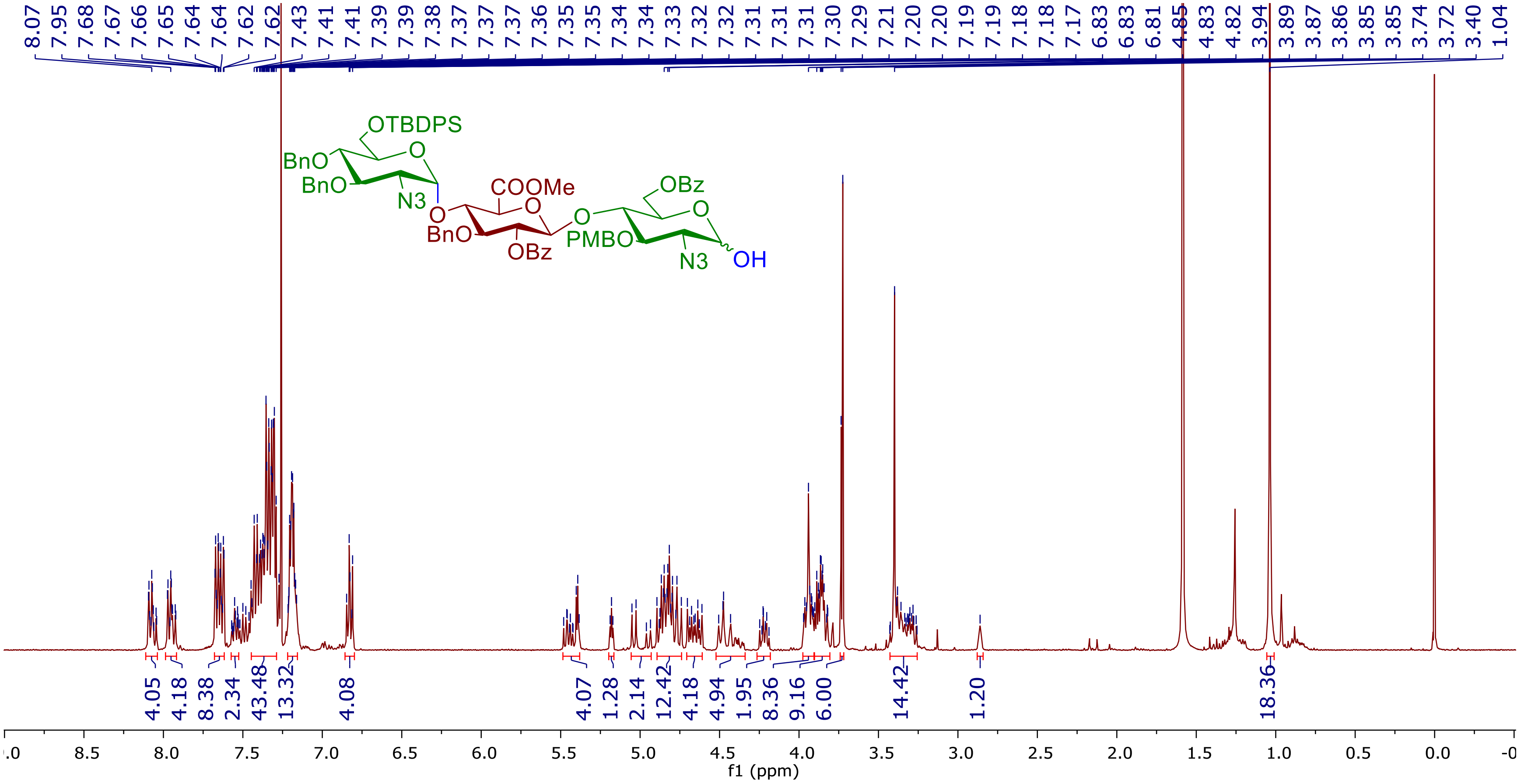

Supplementary Figure S96b. ^{13}C NMR Spectrum (100.67 MHz, $CDCl_3$) of Compound S14

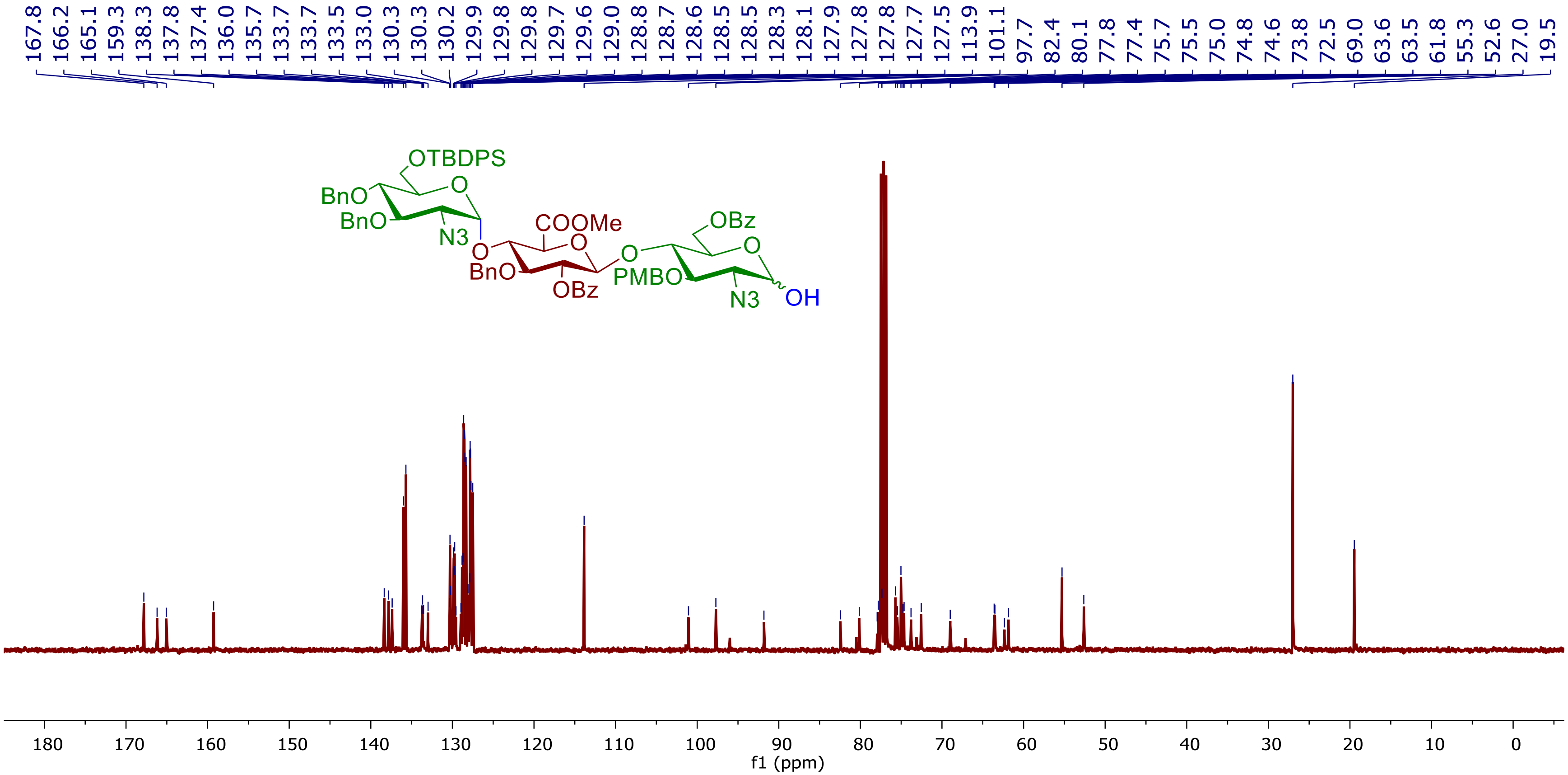

Supplementary Figure S96c. DEPT NMR Spectrum (100.67 MHz, $CDCl_3$) of Compound S14

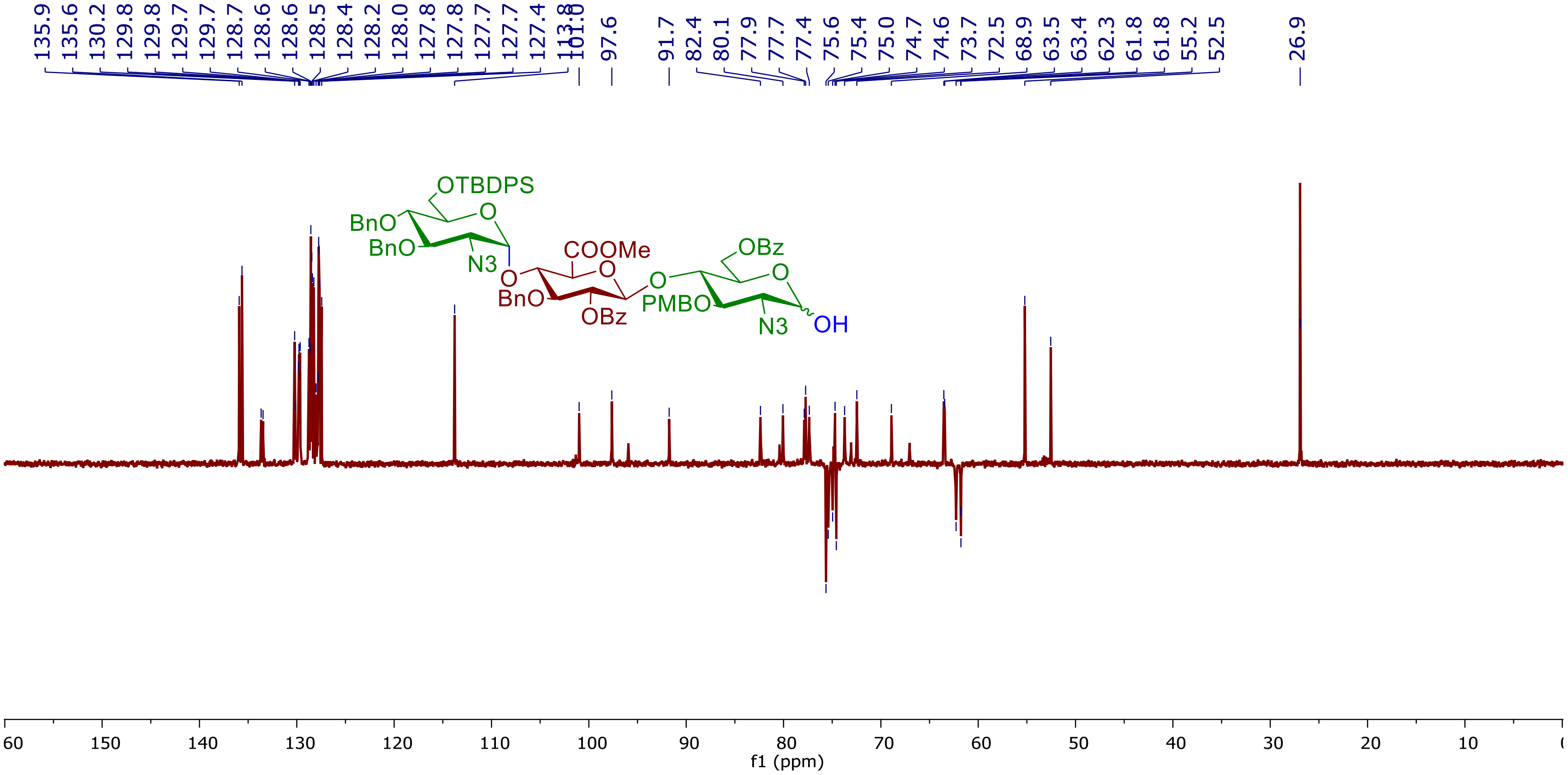

Supplementary Figure S97a. ¹H NMR Spectrum (400.31 MHz, $CDCl_3$) of Compound 47

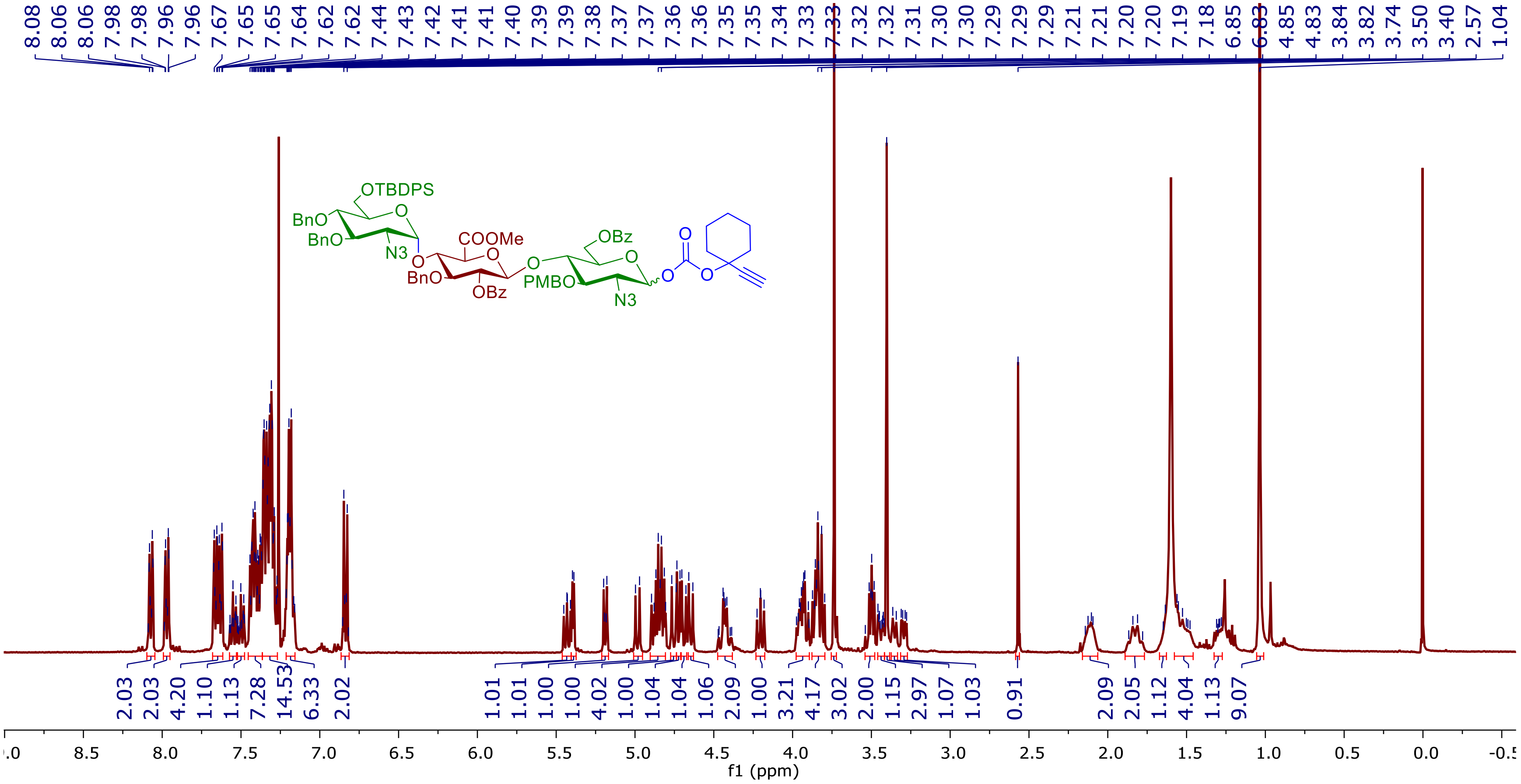

Supplementary Figure S97b. ^{13}C NMR Spectrum (100.67 MHz, $CDCl_3$) of Compound 47

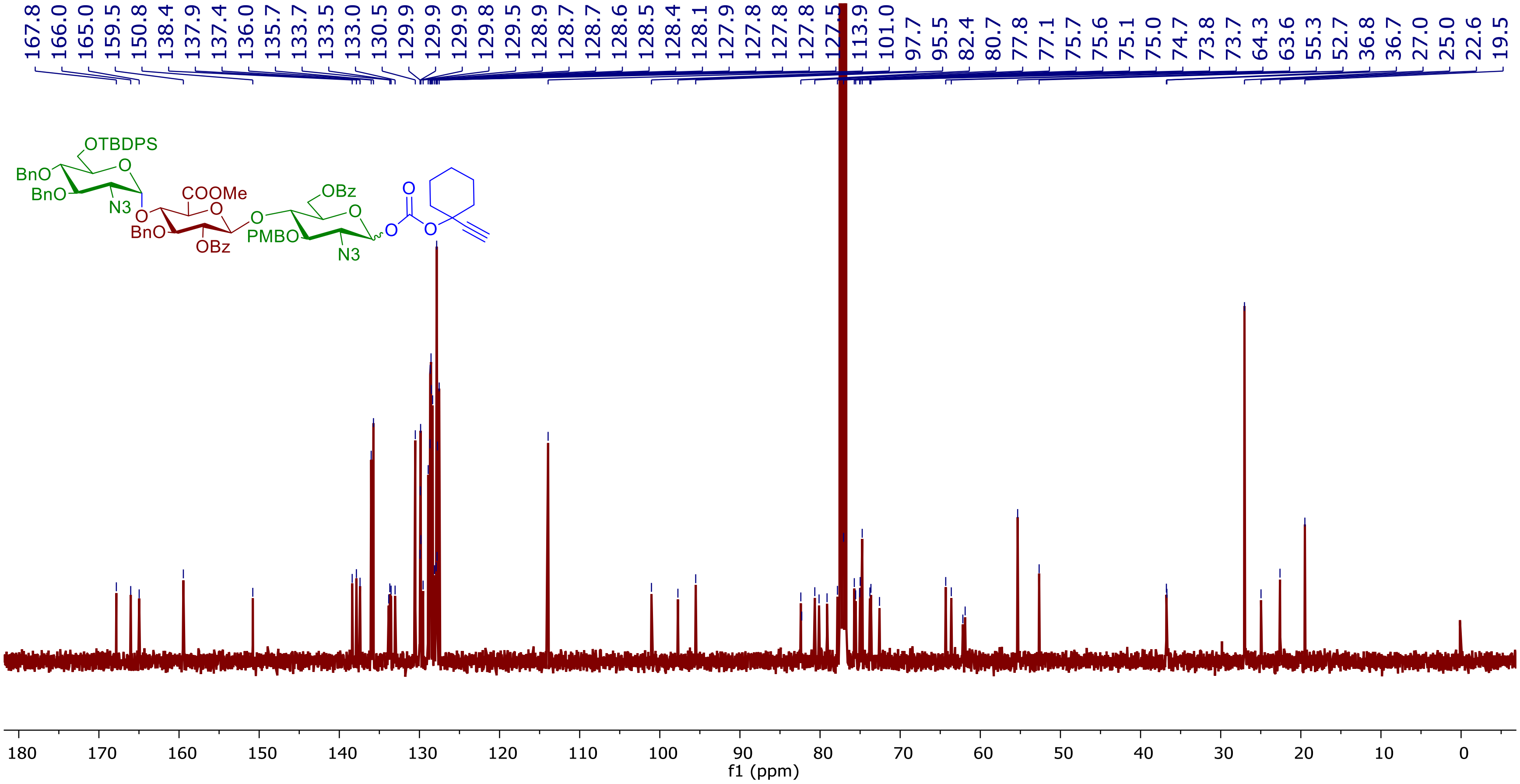

Supplementary Figure S97c. DEPT NMR Spectrum (100.67 MHz, $CDCl_3$) of Compound 47

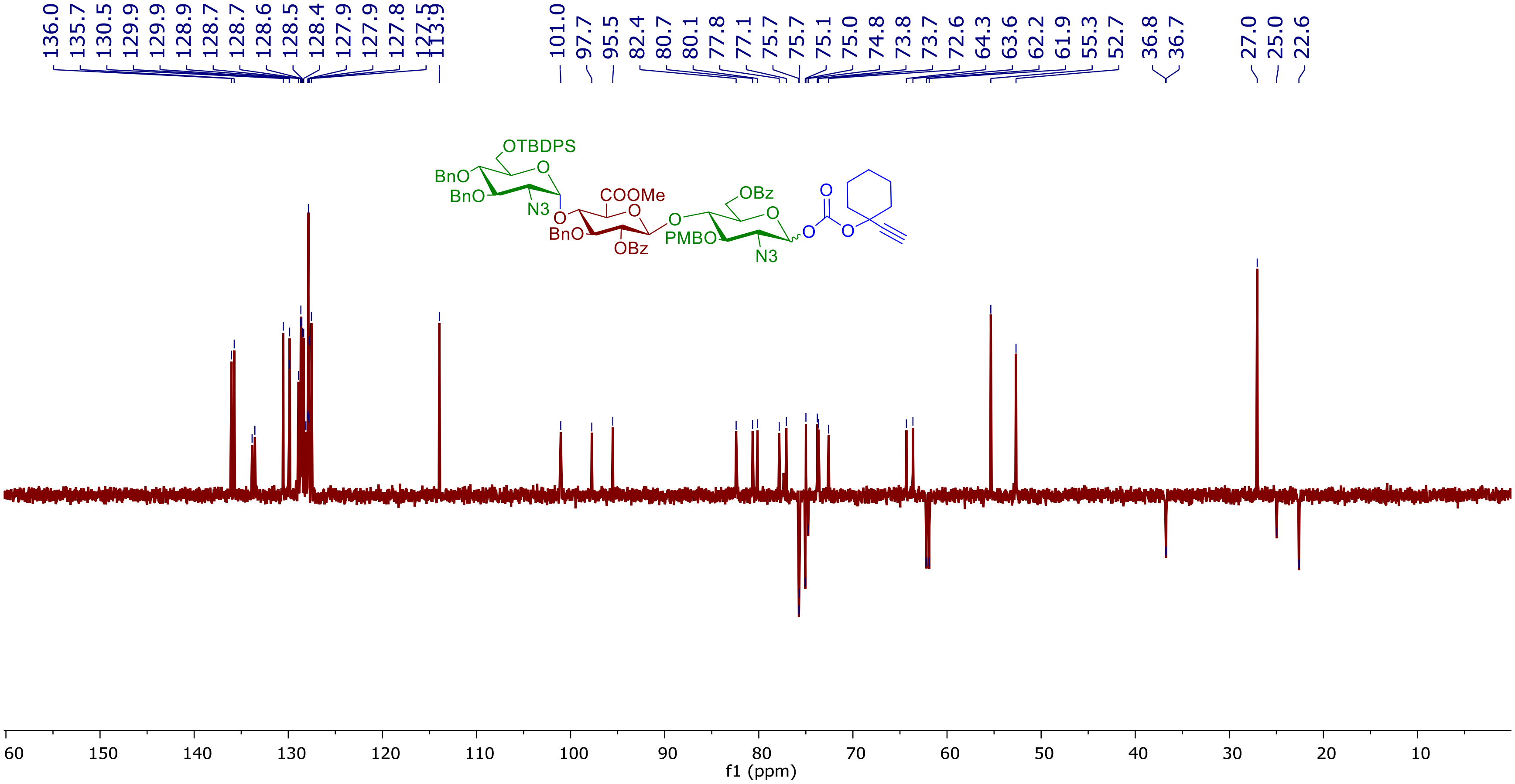

Supplementary Figure S97d. COSY NMR Spectrum (600.40, 600.40 MHz, $CDCl_3$) of Compound 47

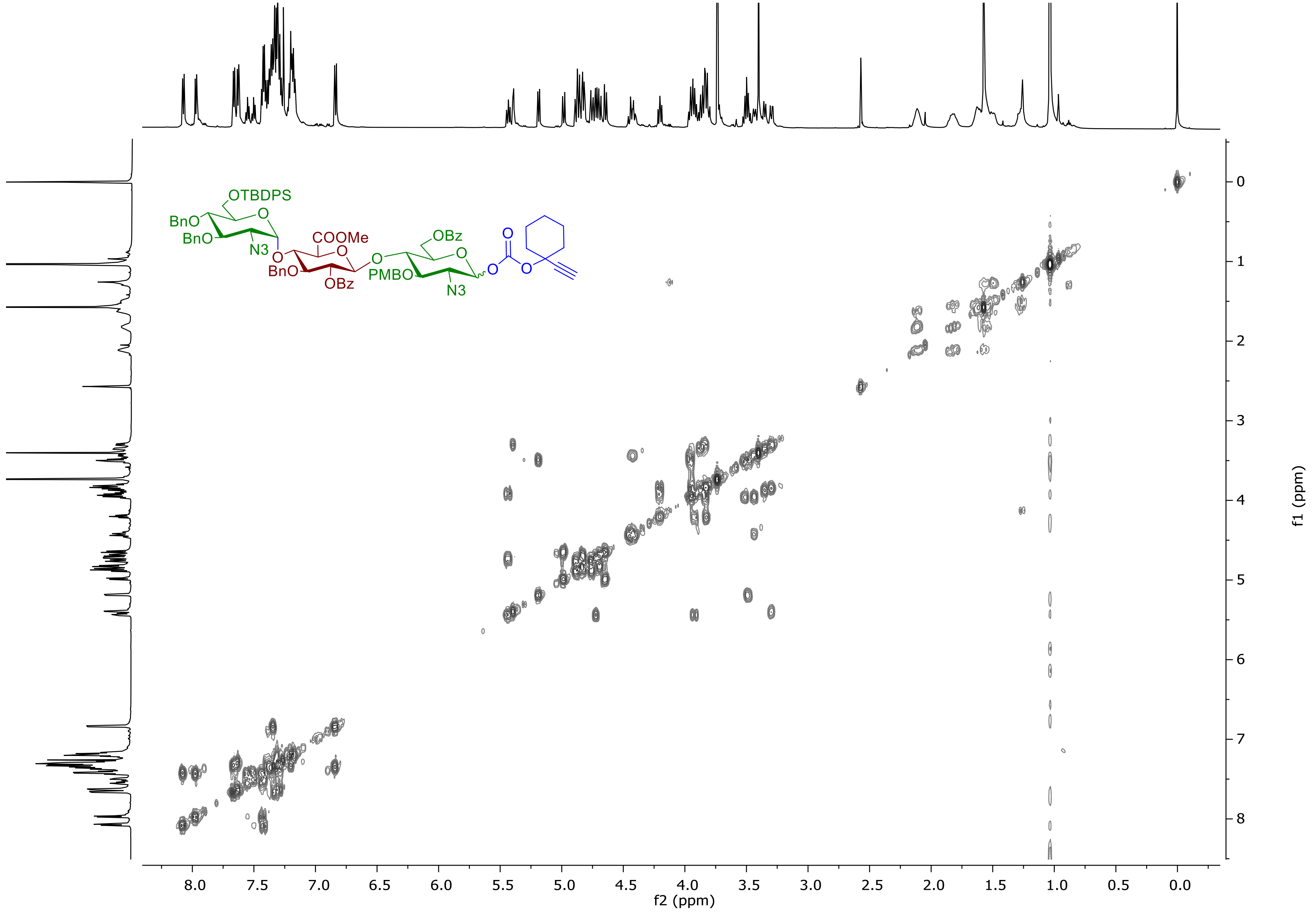

Supplementary Figure S97e. COSY NMR Spectrum (600.40, 600.40 MHz, $CDCl_3$) of Compound 47 (Sugar region expanded)

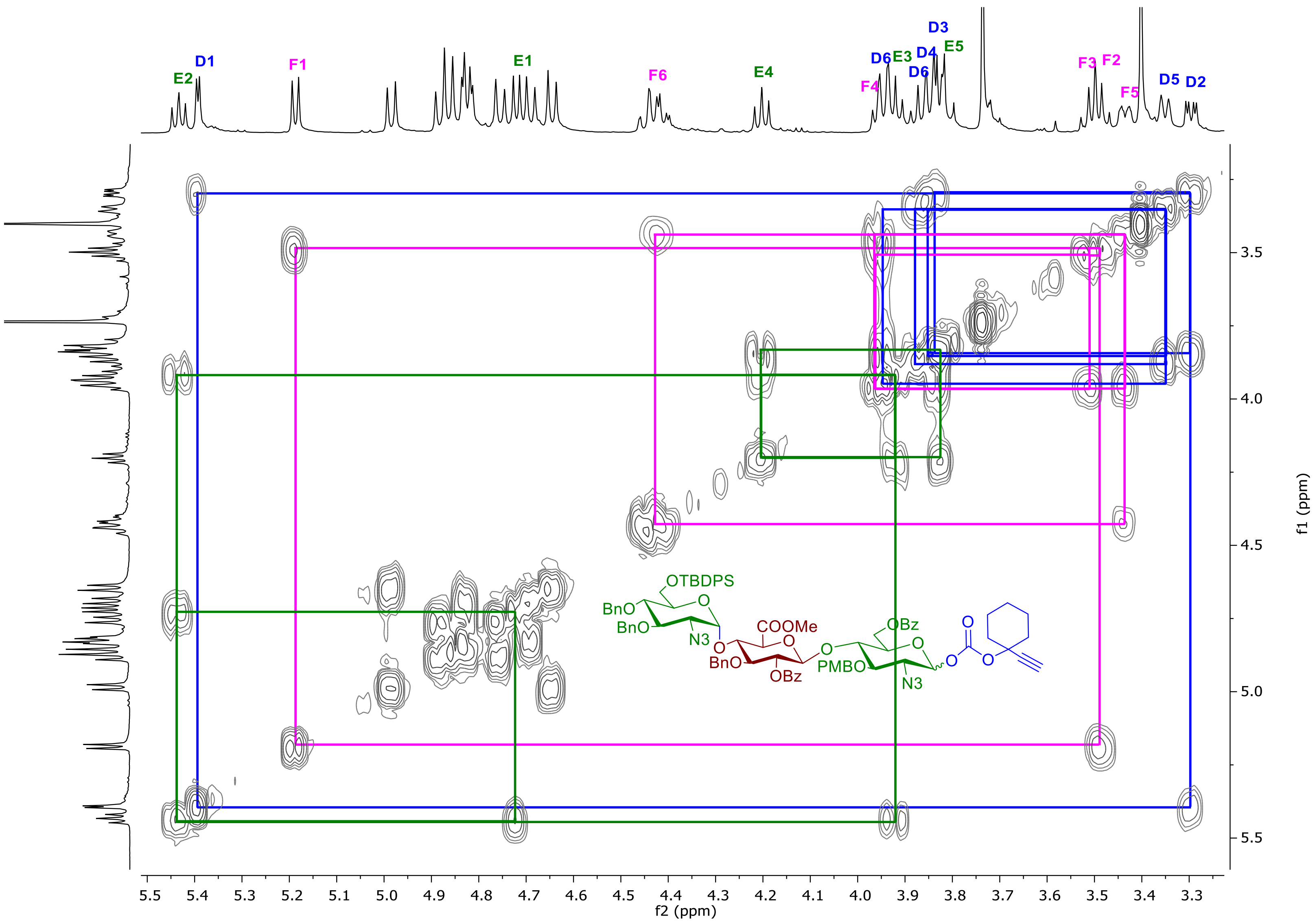

Supplementary Figure S97f. HSQC NMR Spectrum (600.40, 150.99MHz, $CDCl_3$) of Compound 47

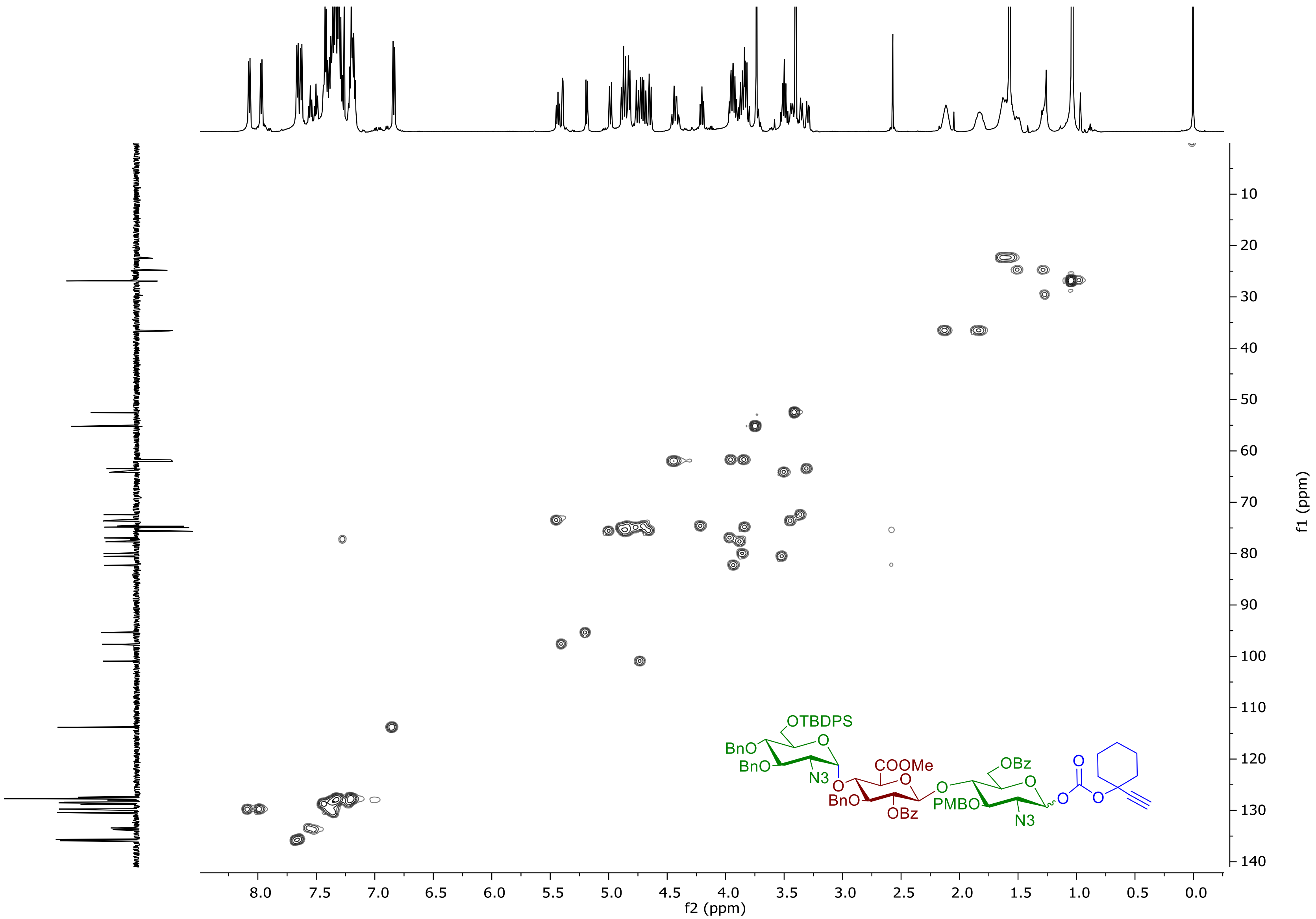

Supplementary Figure S97g. HSQC NMR Spectrum (600.40, 150.99 MHz, $CDCl_3$) of Compound 47 (Sugar region expanded)

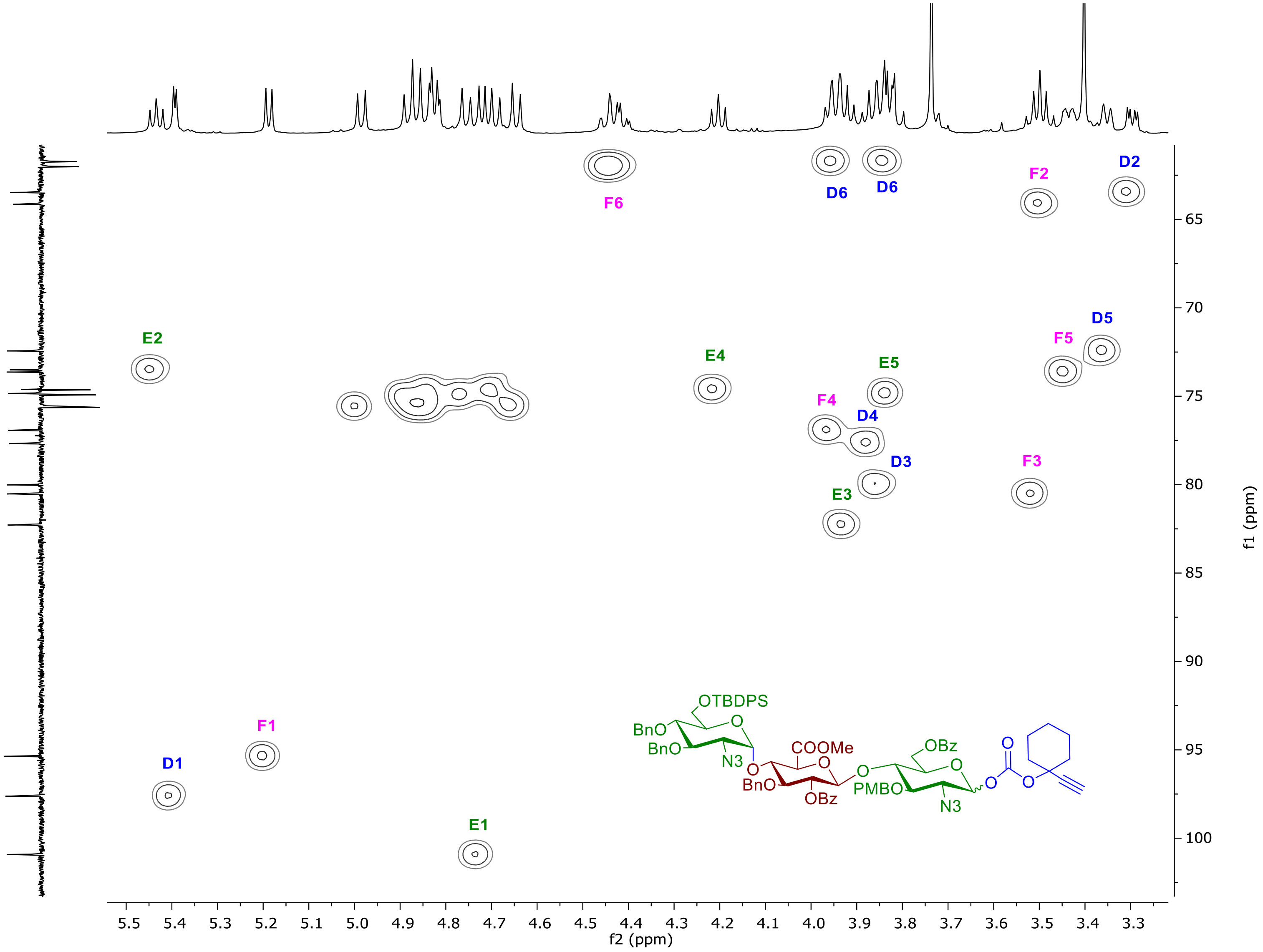

Supplementary Figure S97h. Coupled HSQC NMR Spectrum (600.40, 150.99 MHz, $CDCl_3$) of Compound 47

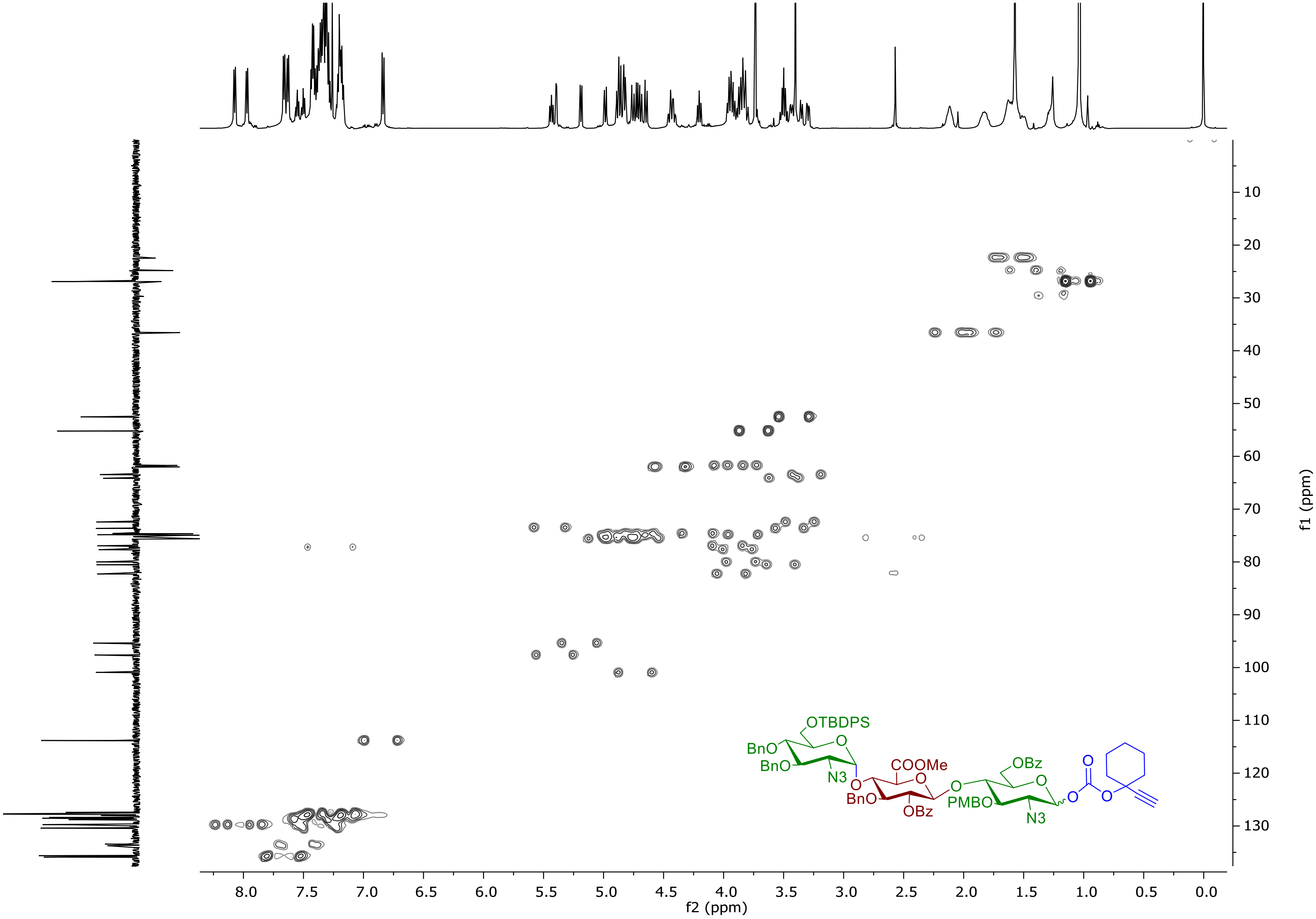

Supplementary Figure S97i. Coupled HSQC NMR Spectrum (600.40, 150.99 MHz, $CDCl_3$) of Compound 47 (Sugar region expanded)

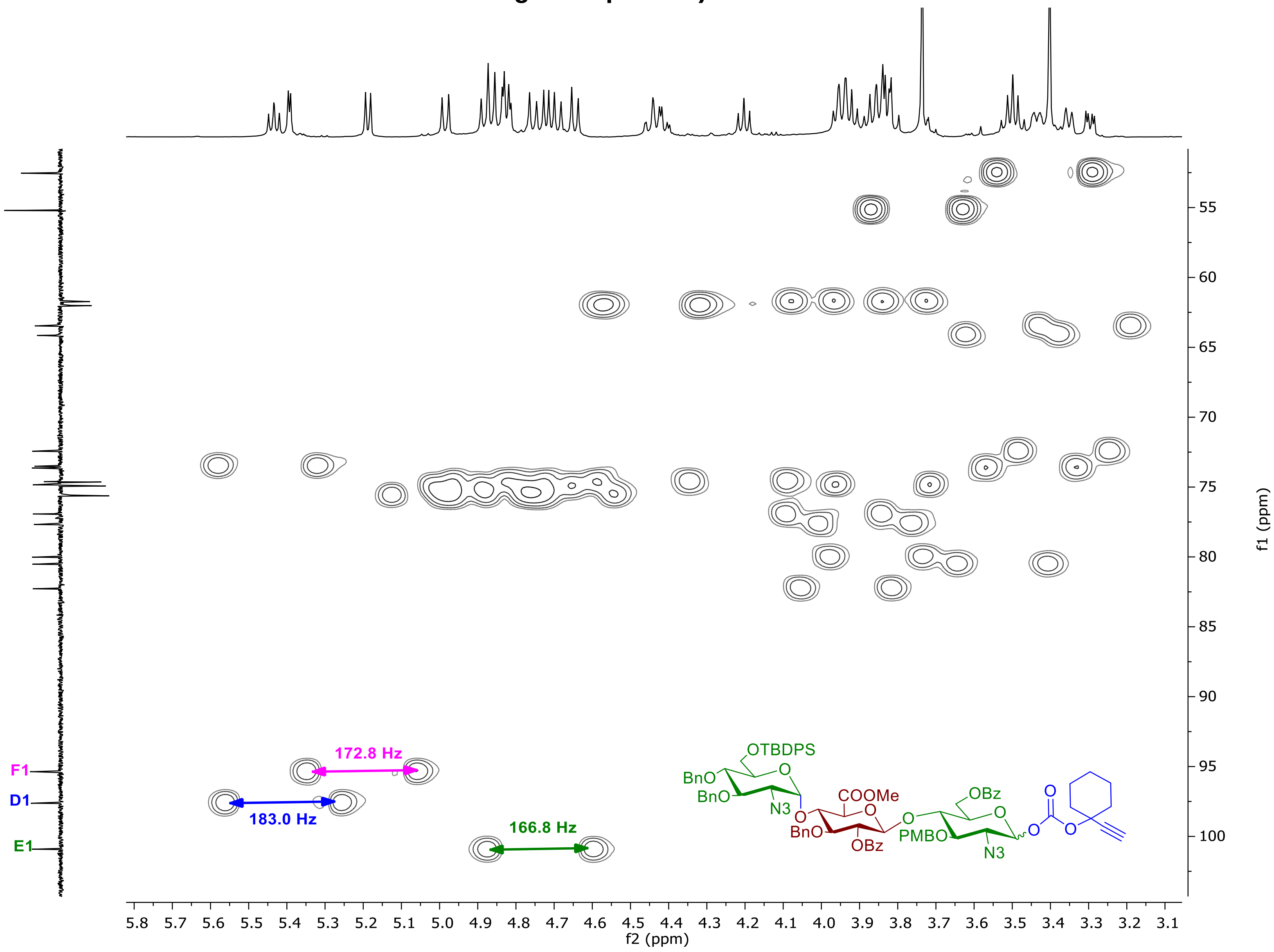

Supplementary Figure S97j. HMBC NMR Spectrum (600.40, 150.99 MHz, $CDCl_3$) of Compound 47

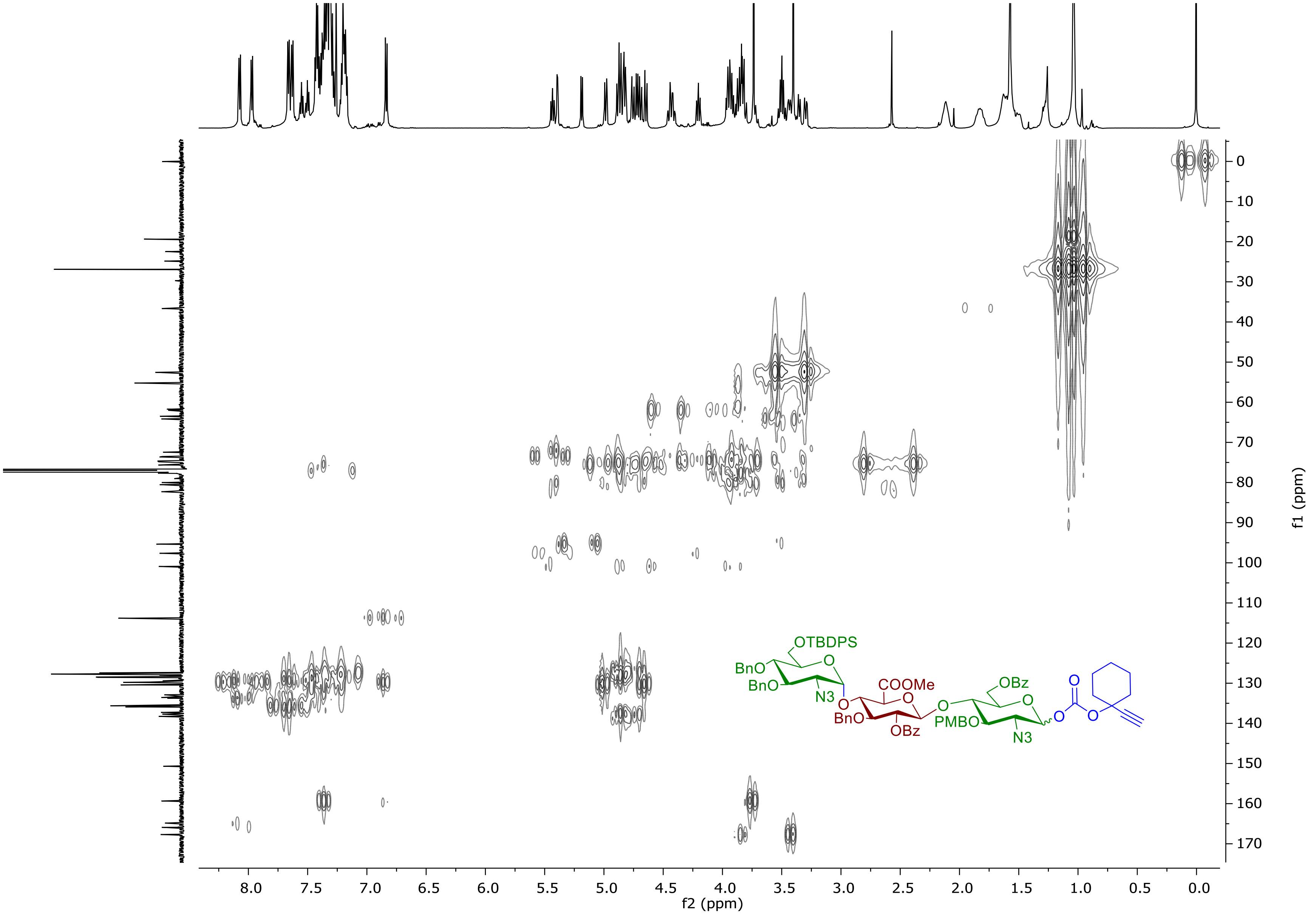

Supplementary Figure S97k. HMBC Spectrum (600.40, 150.99 MHz, $CDCl_3$) of Compound 47 (Sugar region expanded)

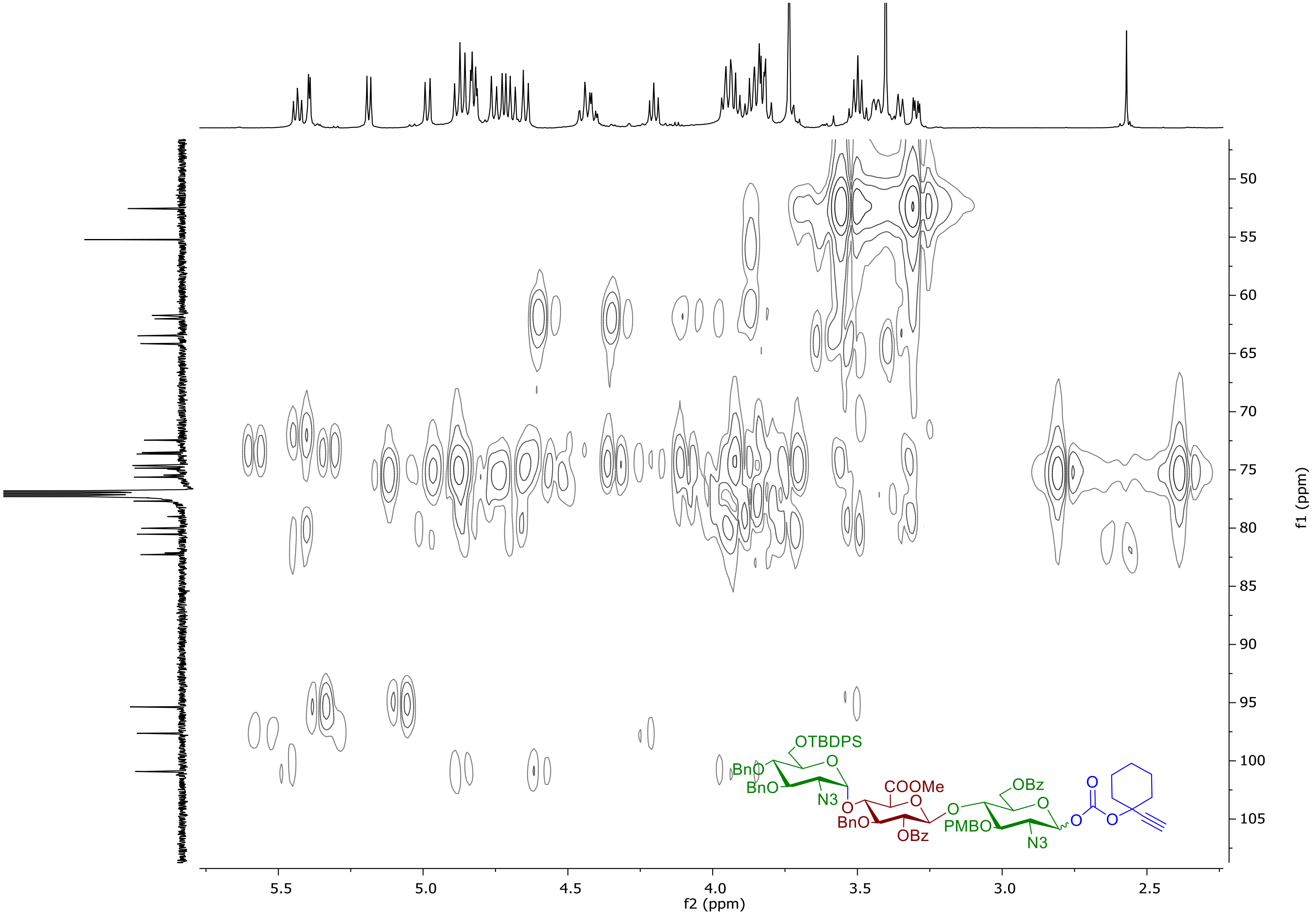

Supplementary Figure S97l. HMBC NMR Spectrum (600.40, 150.99 MHz, $CDCl_3$) of Compound 47 (Carbonyl Region expanded)

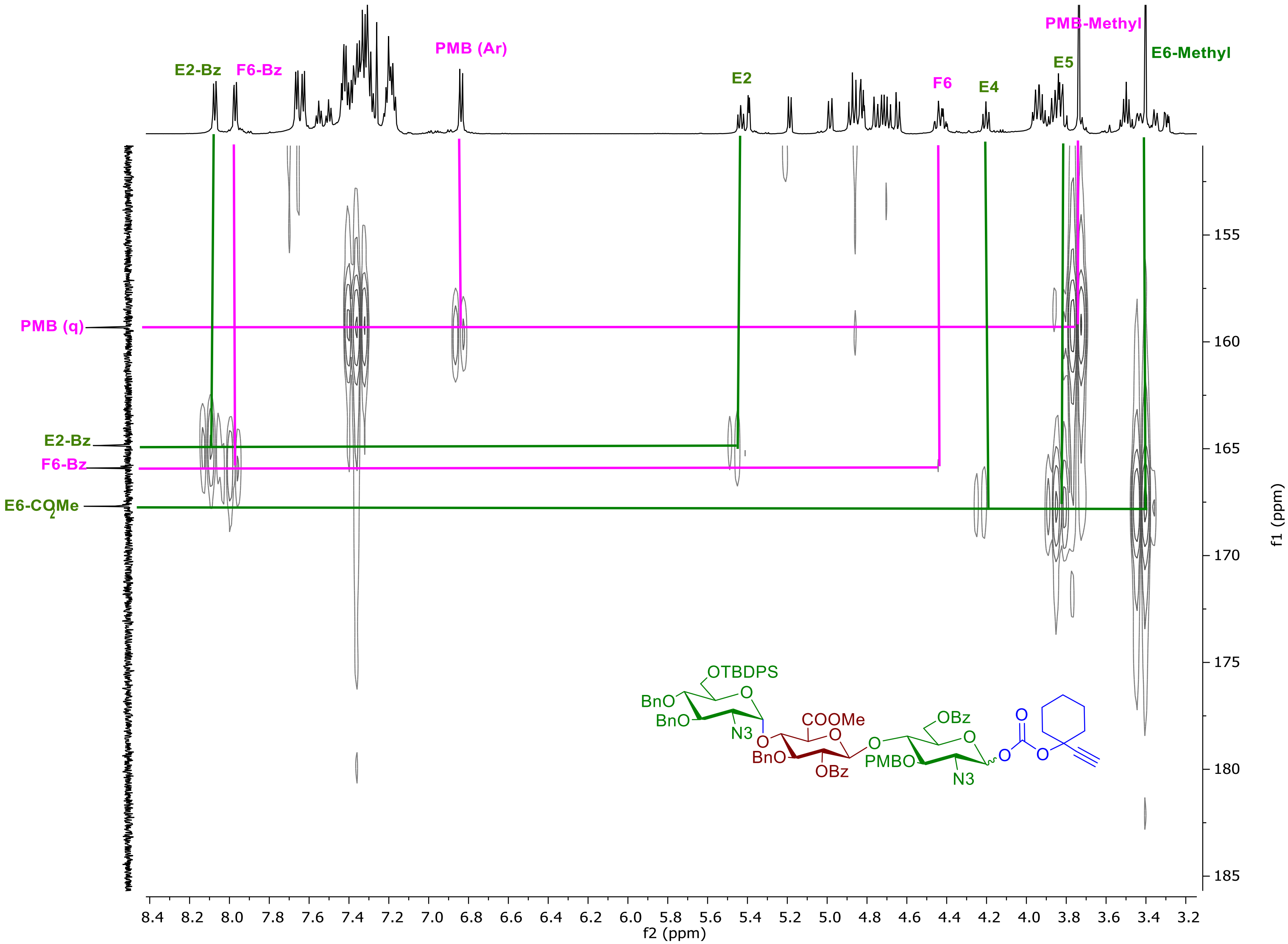

Supplementary Figure S97m. TOCSY NMR Spectrum (600.40, 600.40 MHz, $CDCl_3$) of Compound 47

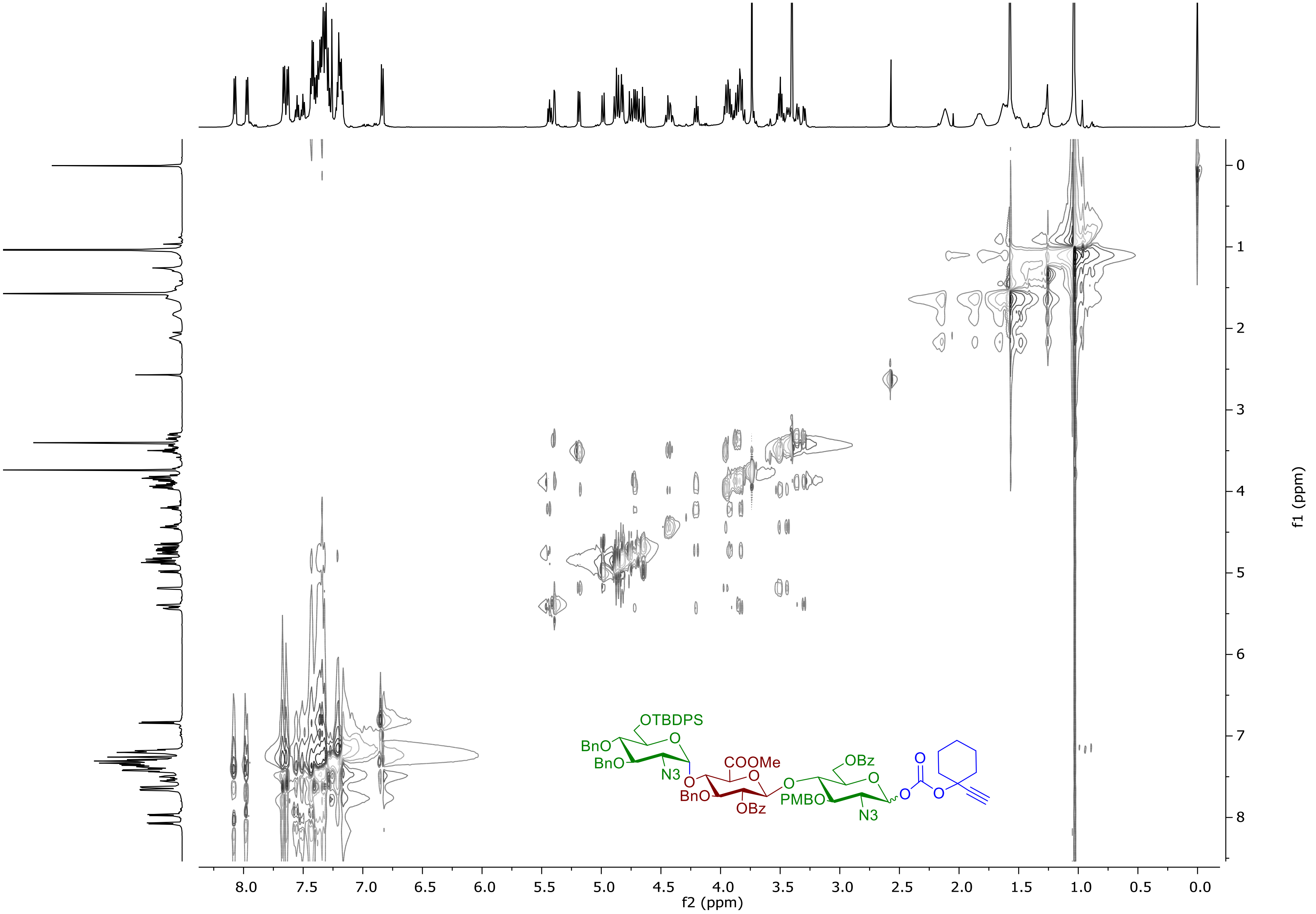

Supplementary Figure S97n. TOCSY NMR Spectrum (600.40, 600.40 MHz, $CDCl_3$) of Compound 47 (Sugar region expanded)

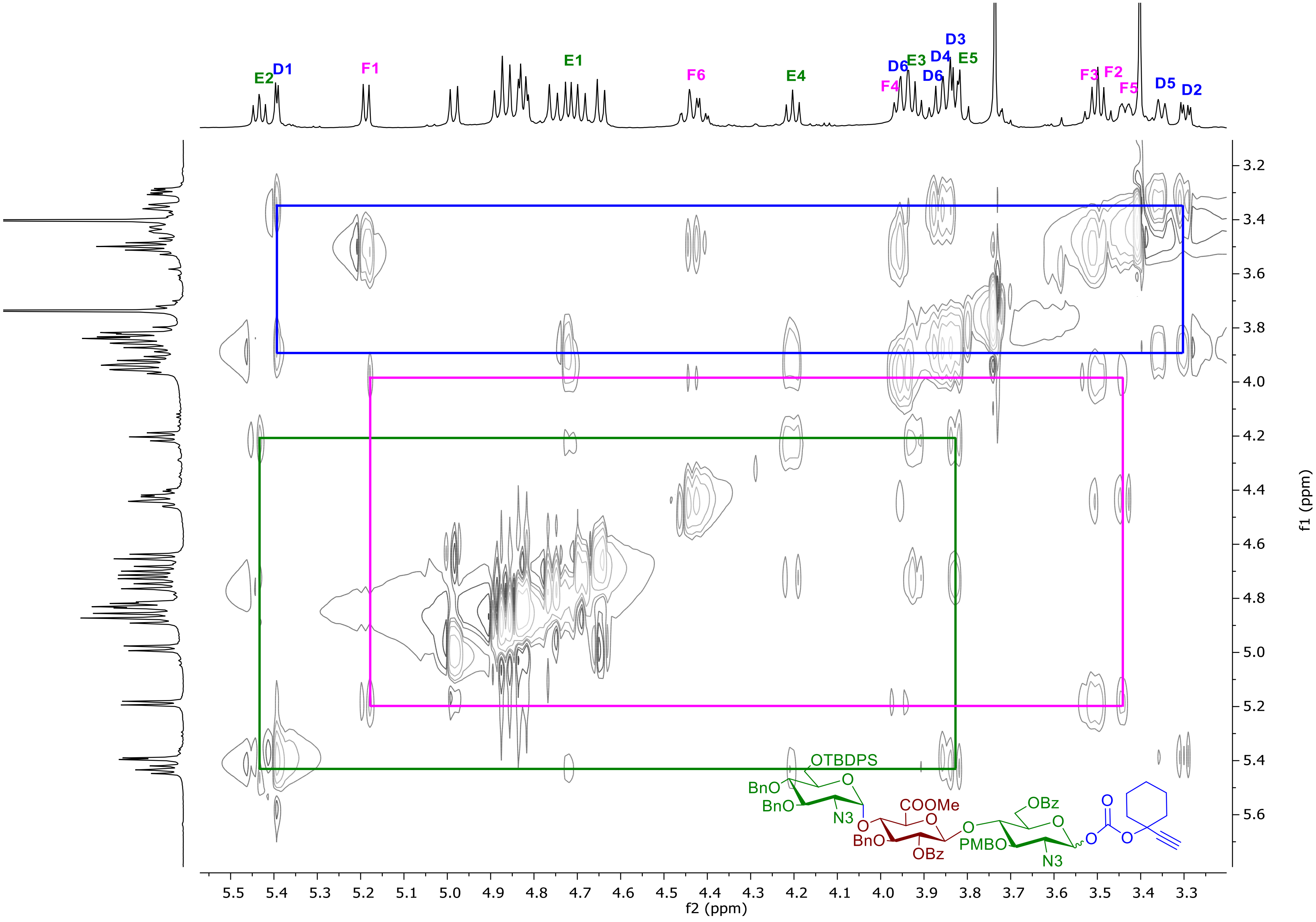

Supplementary Figure S97o. HSQC-TOCSY NMR Spectrum (600.40, 150.99 MHz, $CDCl_3$) of Compound 47

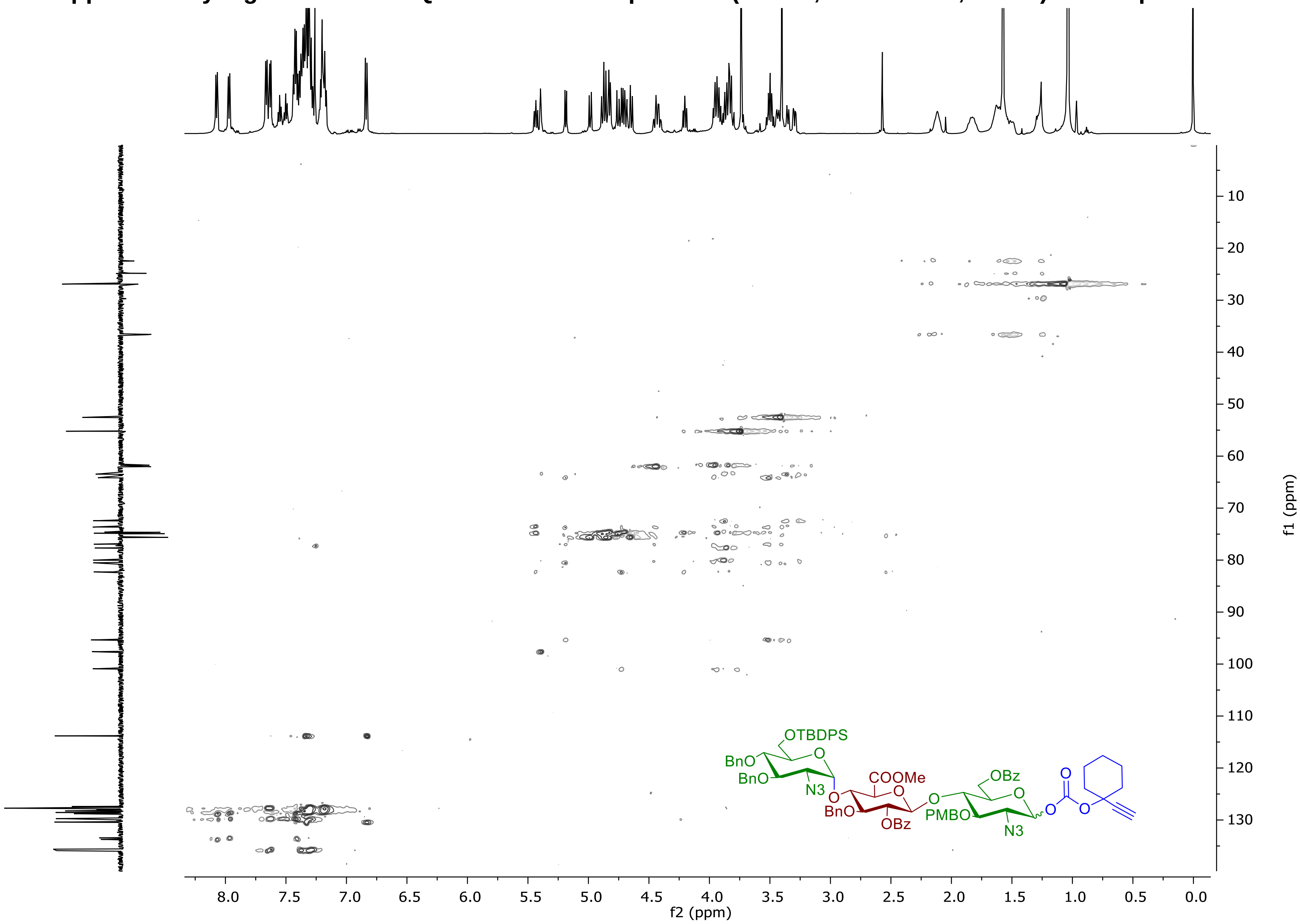

Supplementary Figure S97p. HSQC-TOCSY NMR Spectrum (600.40, 150.99 MHz, $CDCl_3$) of Compound 47 (Sugar region expanded)

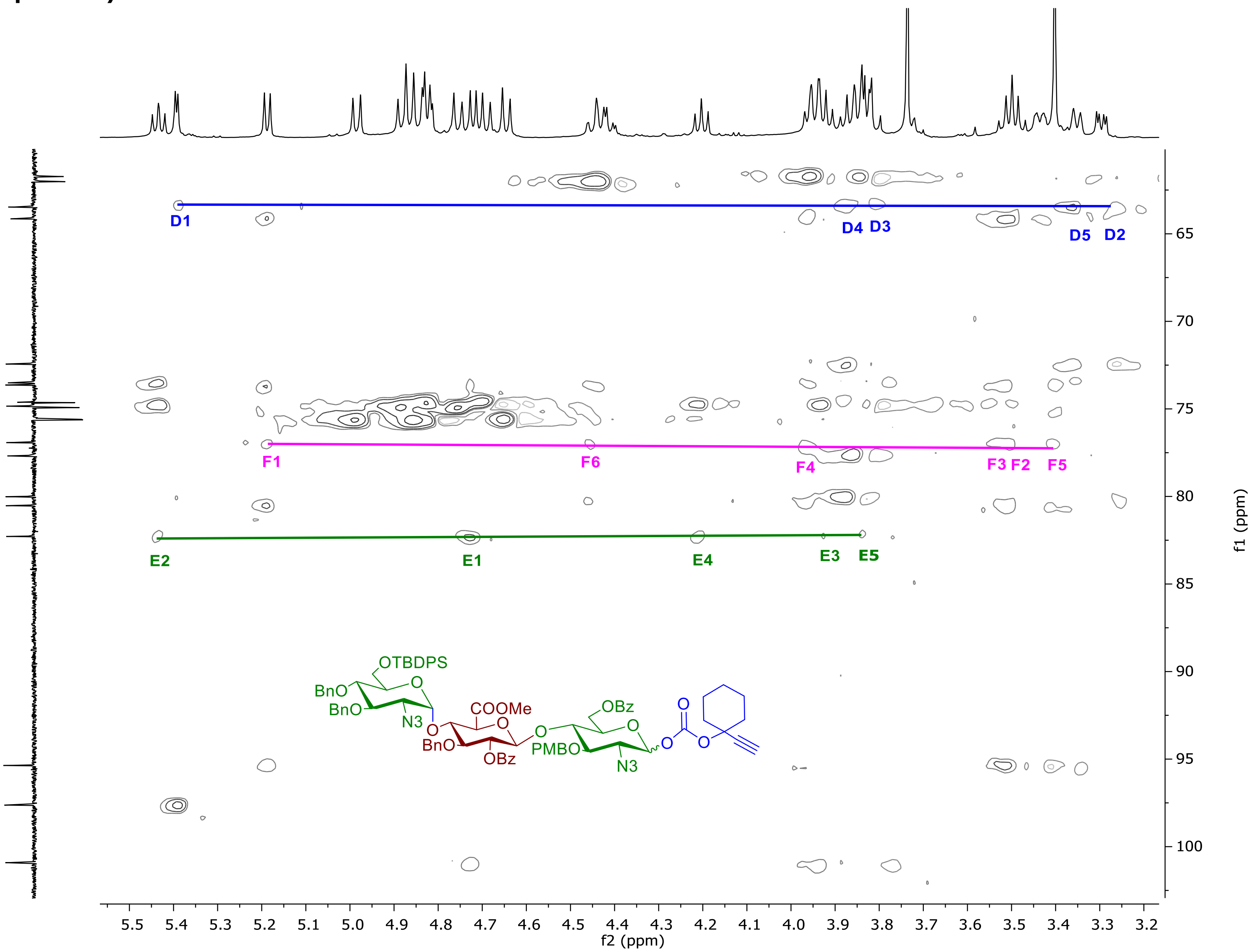

Supplementary Figure S98a. ^{1}H NMR Spectrum (600.40 MHz, $CDCl_3$) of Compound 50a

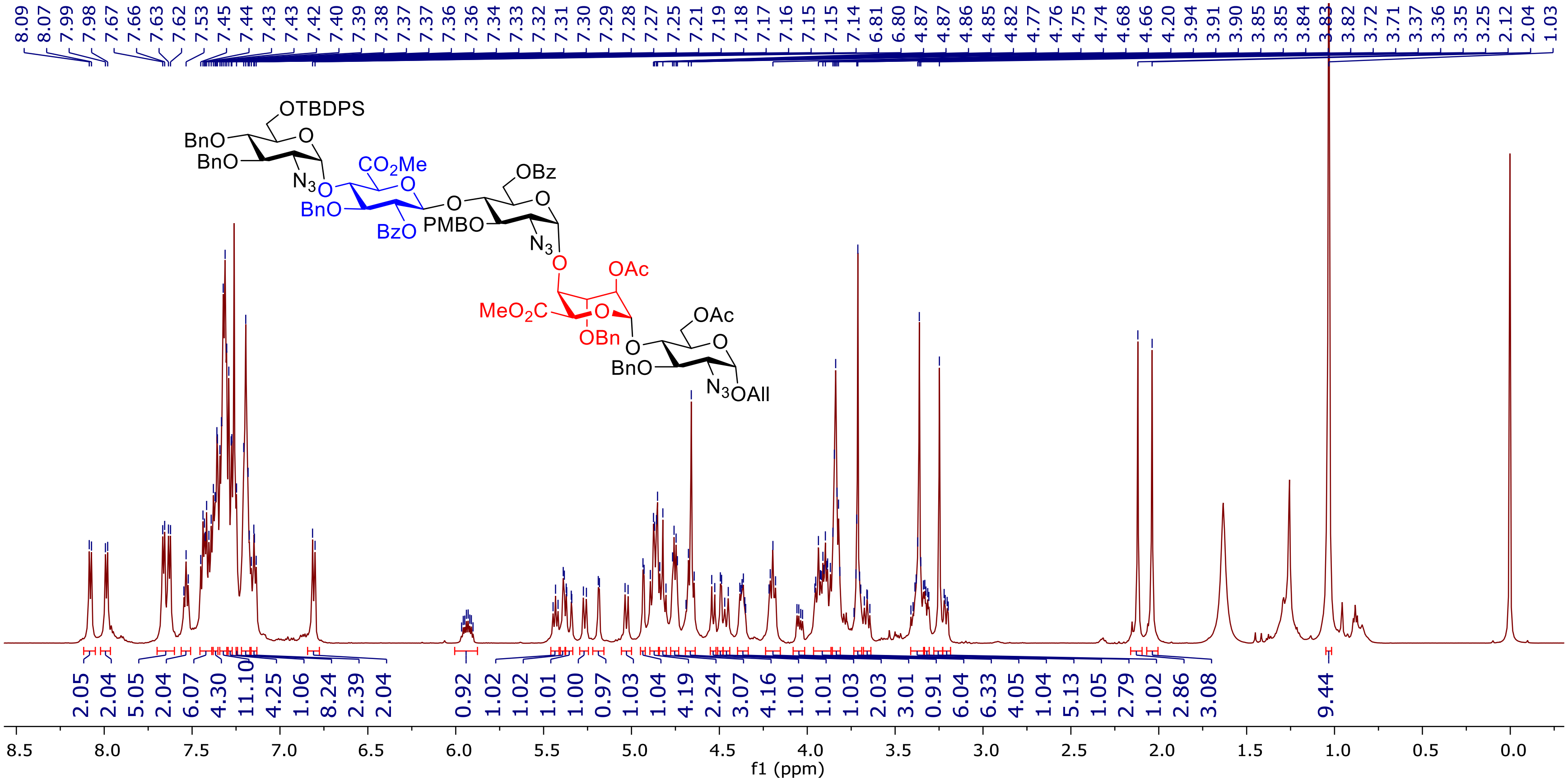

Supplementary Figure S98b. ^{13}C NMR Spectrum (150.99 MHz, $CDCl_3$) of Compound 50a

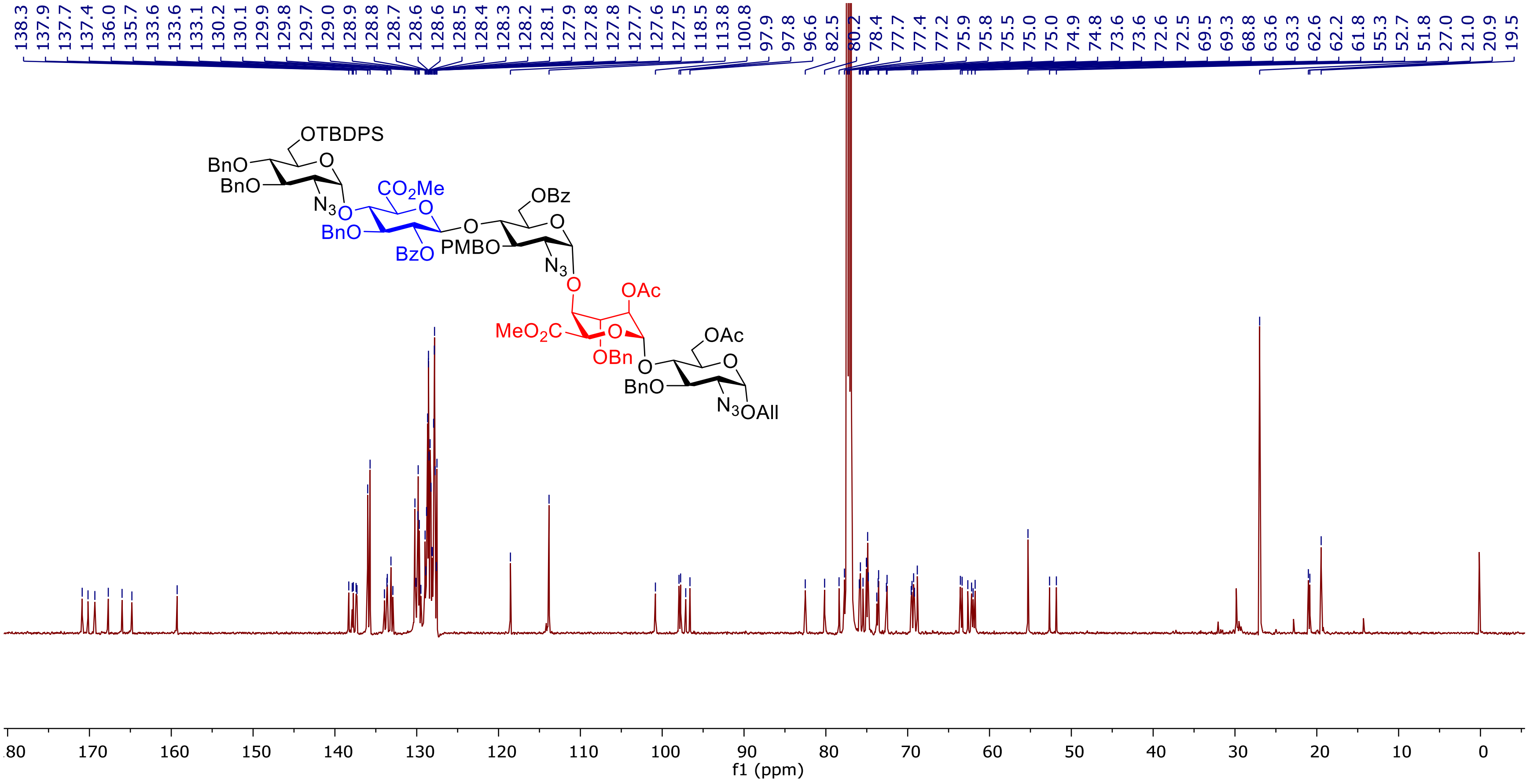

Supplementary Figure S98c. DEPT NMR Spectrum (150.99 MHz, $CDCl_3$) of Compound 50a

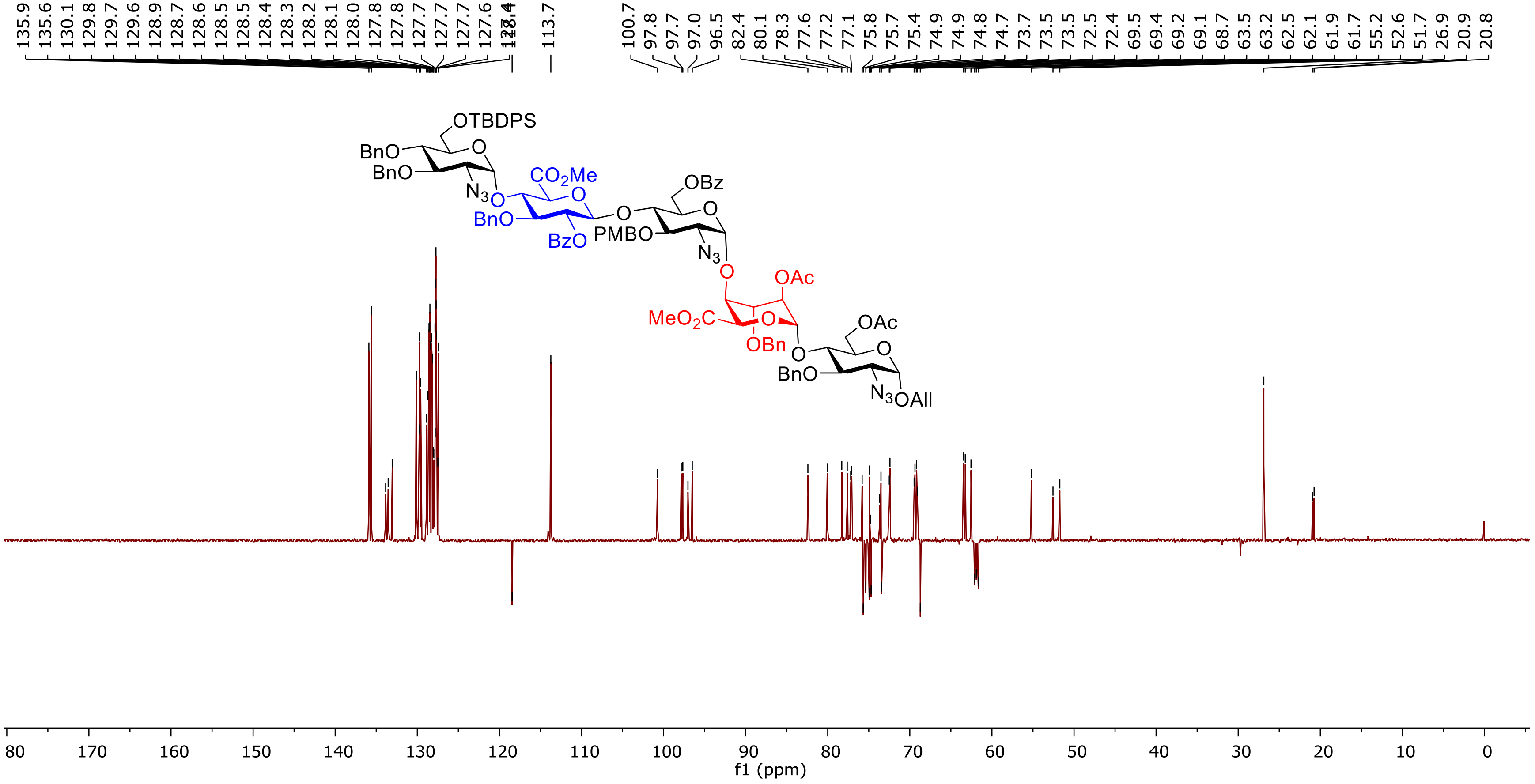

Supplementary Figure S98d. COSY NMR Spectrum (600.40, 600.40 MHz, $CDCl_3$) of Compound 50a

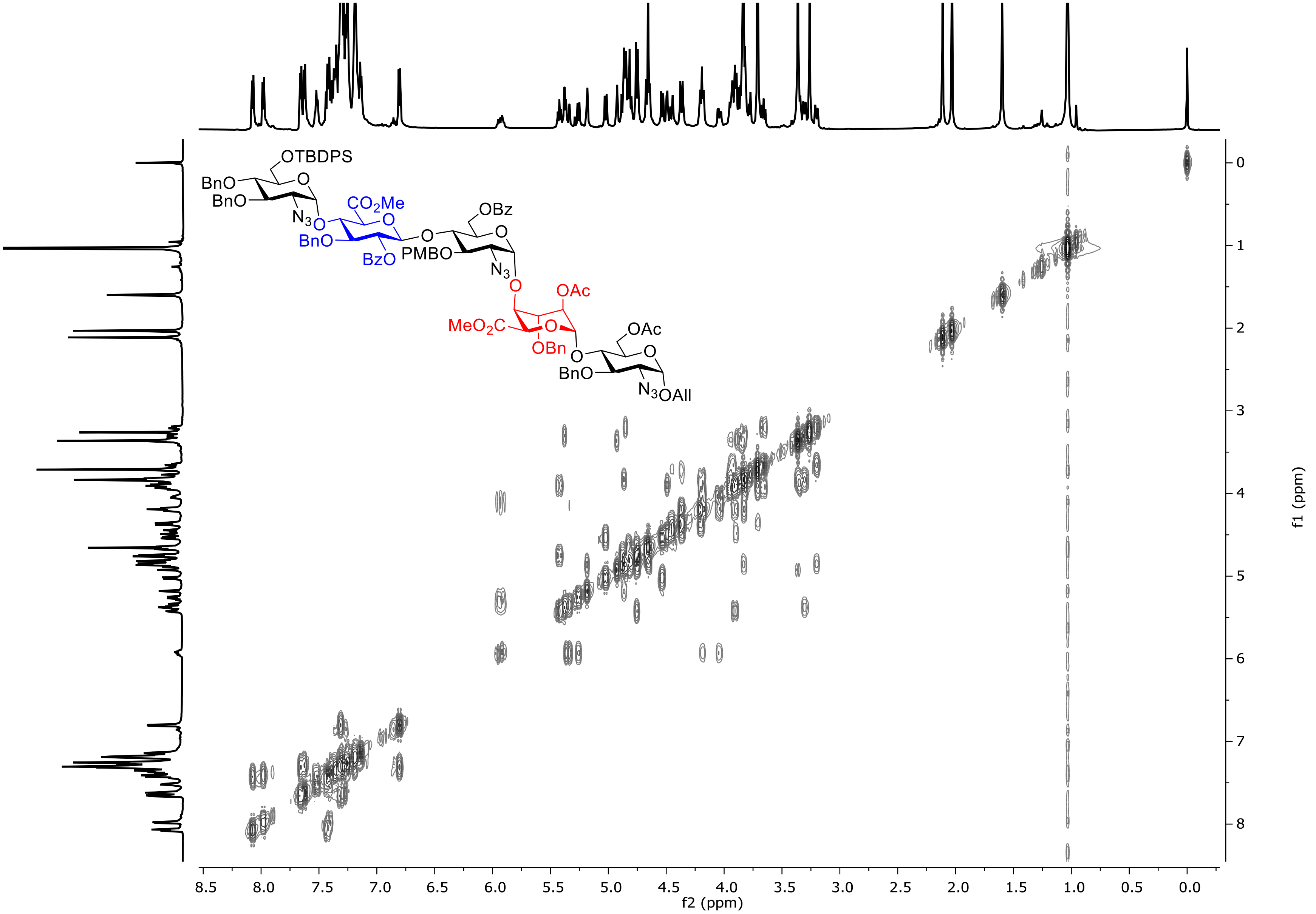

Supplementary Figure S98e. COSY NMR Spectrum (600.40, 600.40 MHz, $CDCl_3$) of Compound 50a (Sugar region expanded)

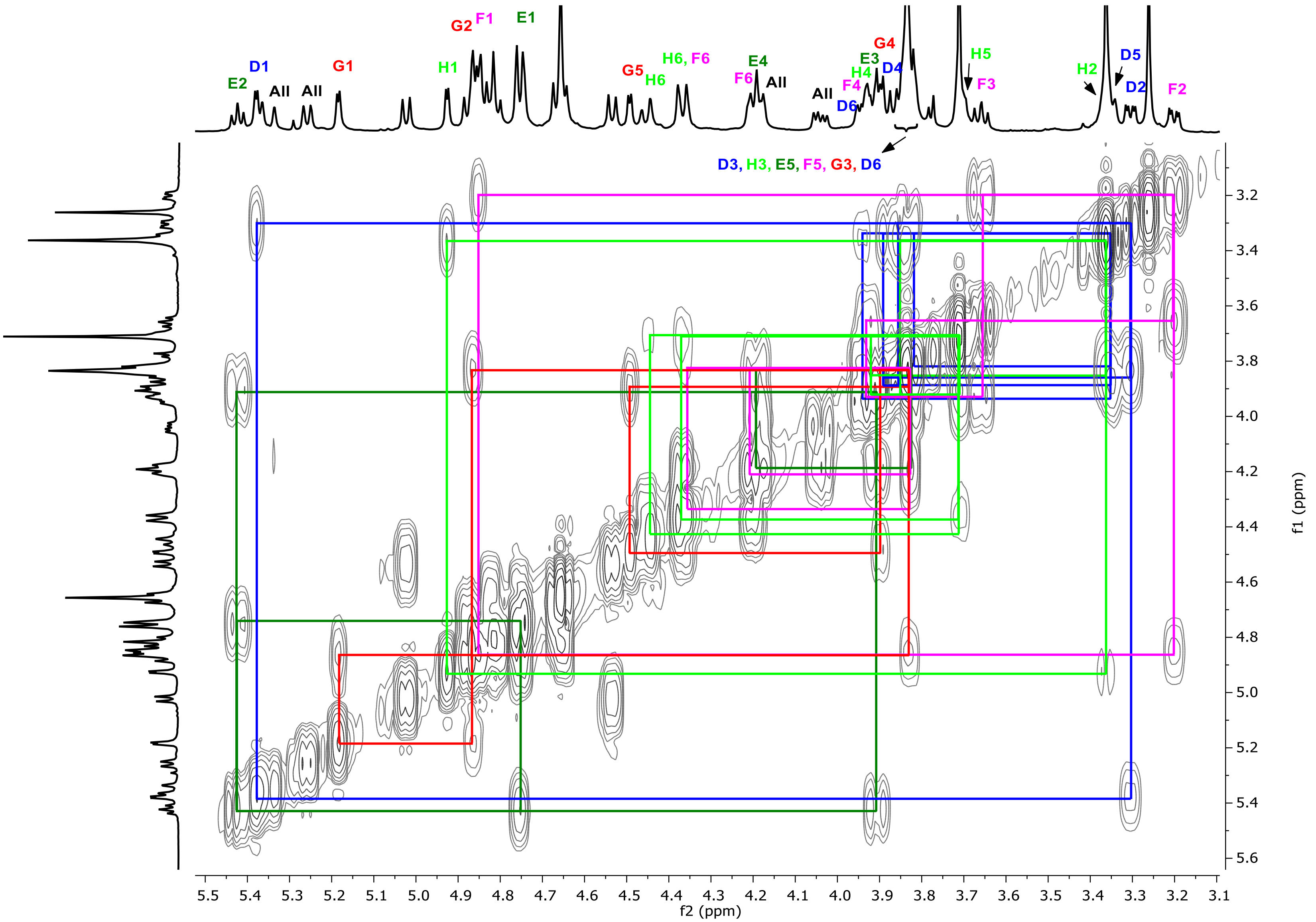

Supplementary Figure S98f. HSQC NMR Spectrum (600.40, 150.99MHz, $CDCl_3$) of Compound 50a

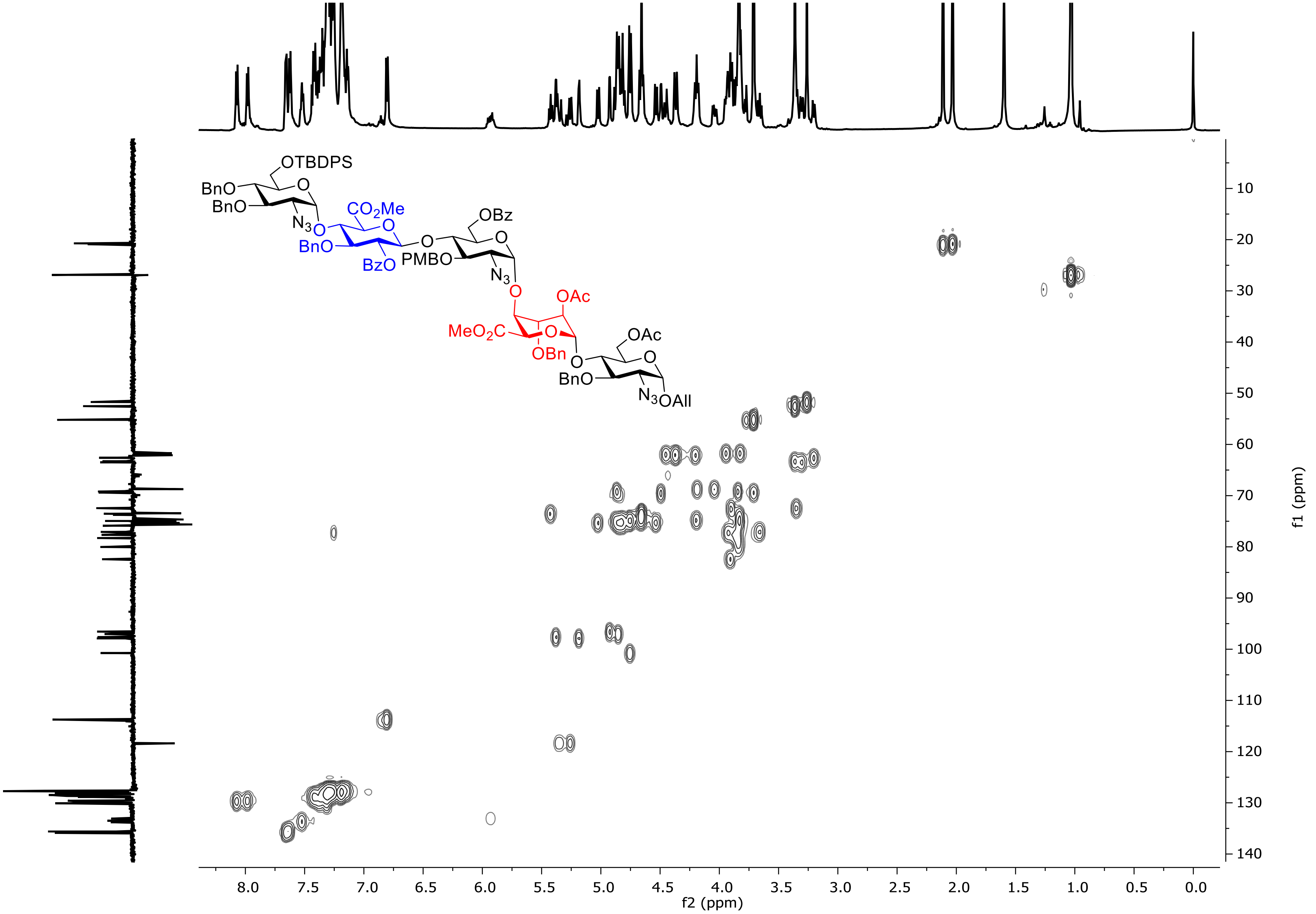

Supplementary Figure S98g. HSQC NMR Spectrum (600.40, 150.99 MHz, CDCl₃) of Compound 50a (Sugar region expanded)

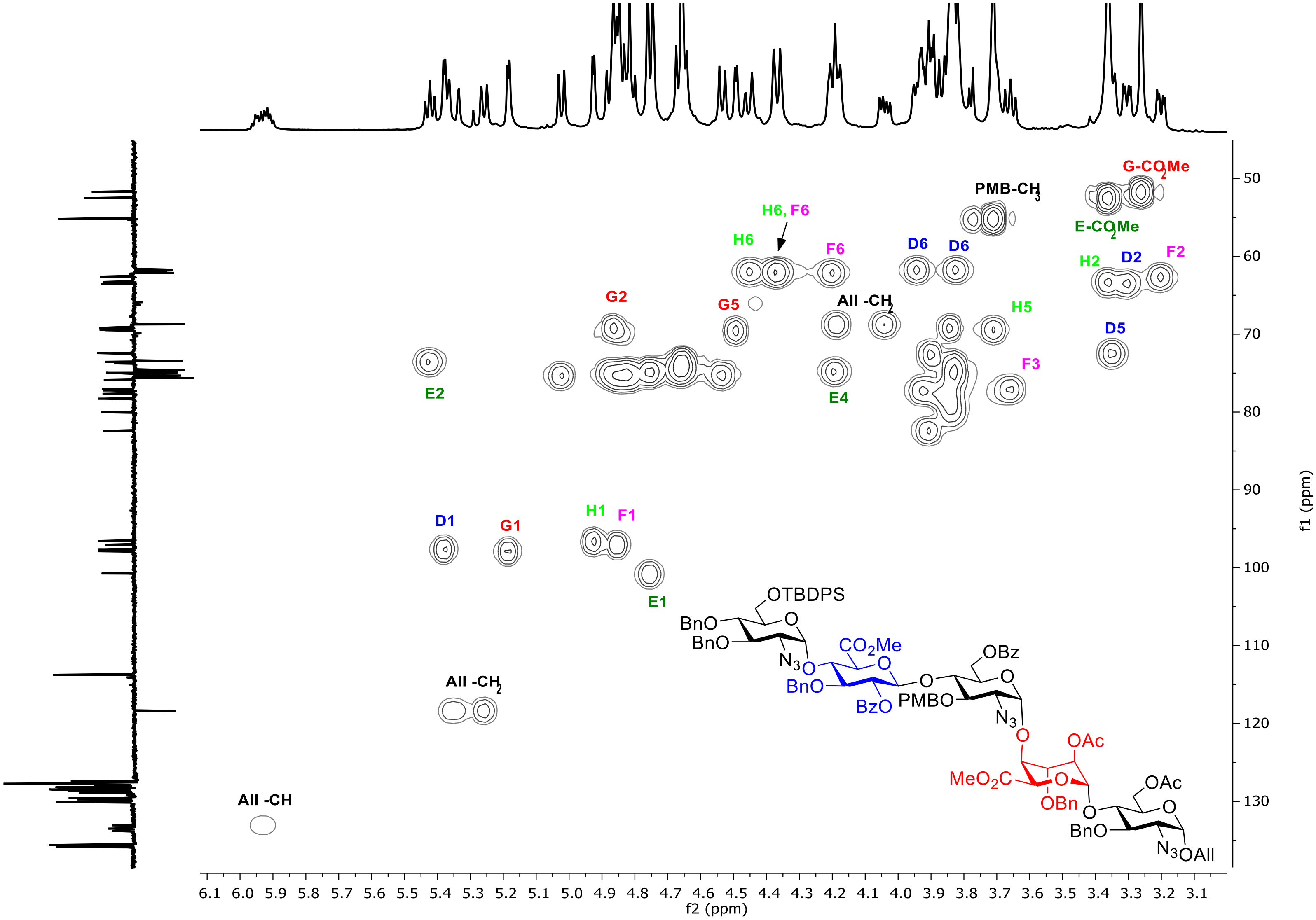

Supplementary Figure S98h. Coupled HSQC NMR Spectrum (600.40, 150.99 MHz, $CDCl_3$) of Compound 50a

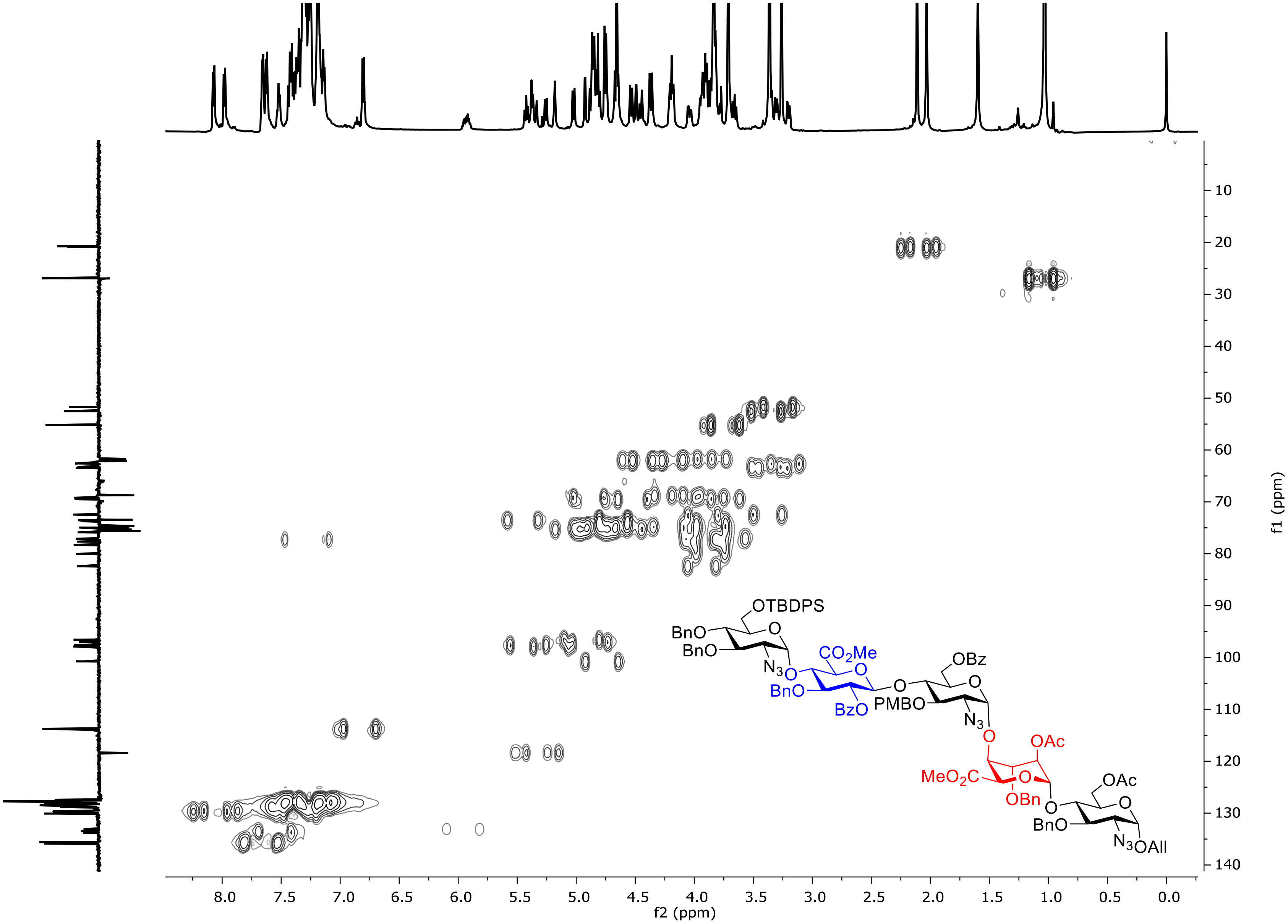

Supplementary Figure S98i. Coupled HSQC NMR Spectrum (600.40, 150.99 MHz, $CDCl_3$) of Compound 50A (Sugar region expanded)

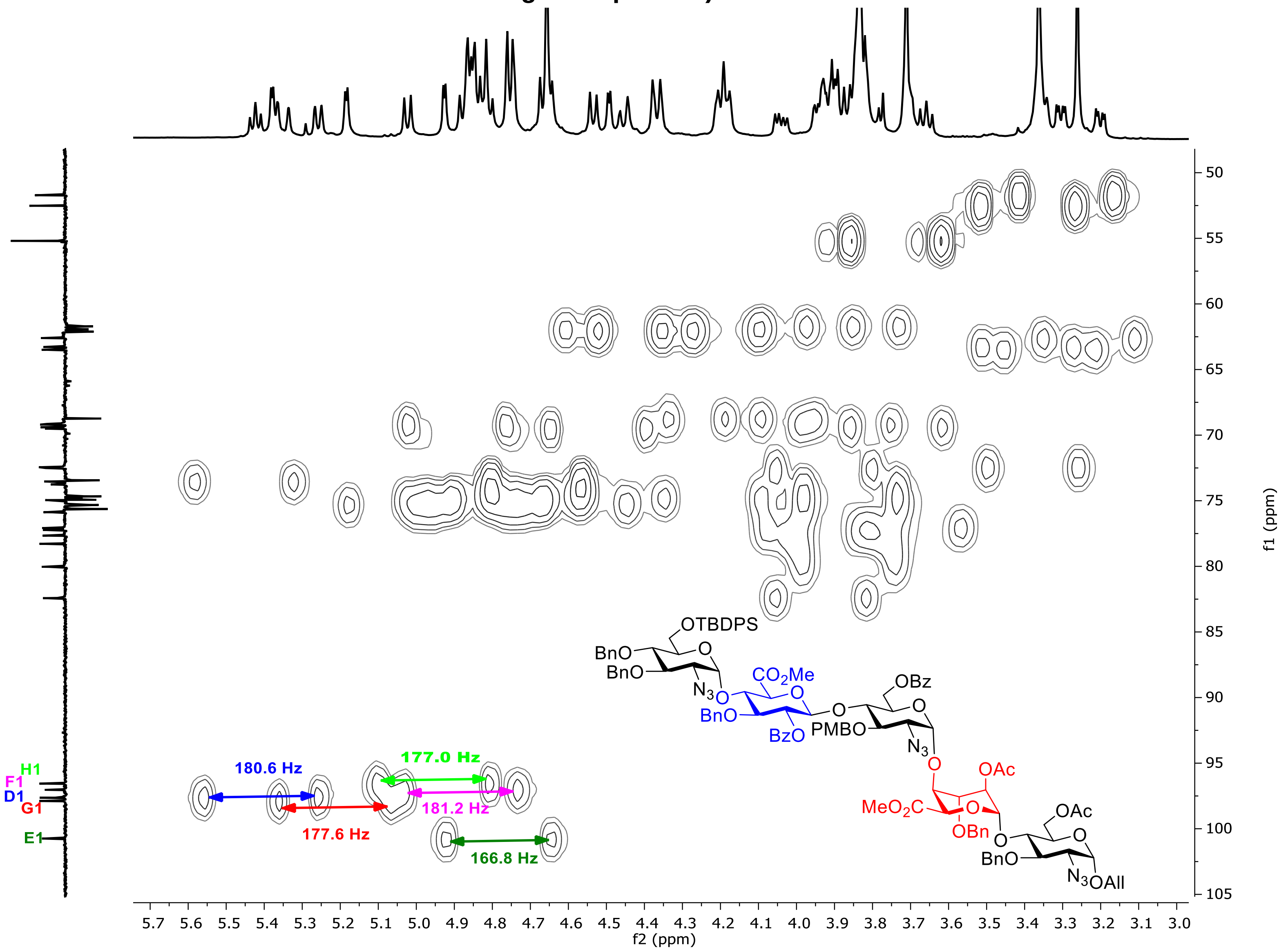

Supplementary Figure S98j. HMBC NMR Spectrum (600.40, 150.99 MHz, $CDCl_3$) of Compound 50a

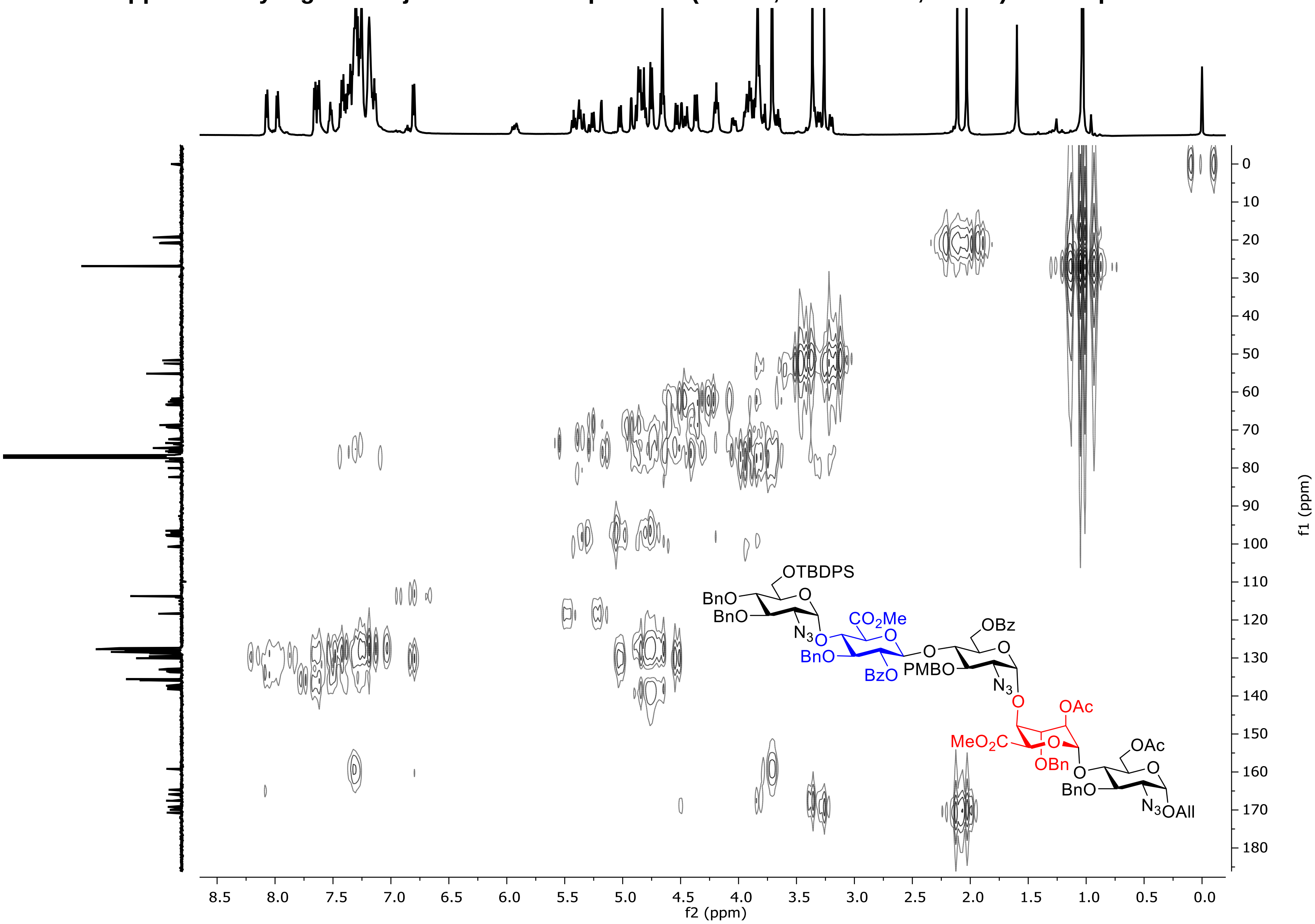

Supplementary Figure S98k. HMBC Spectrum (600.40, 150.99 MHz, $CDCl_3$) of Compound 50A (Sugar region expanded)

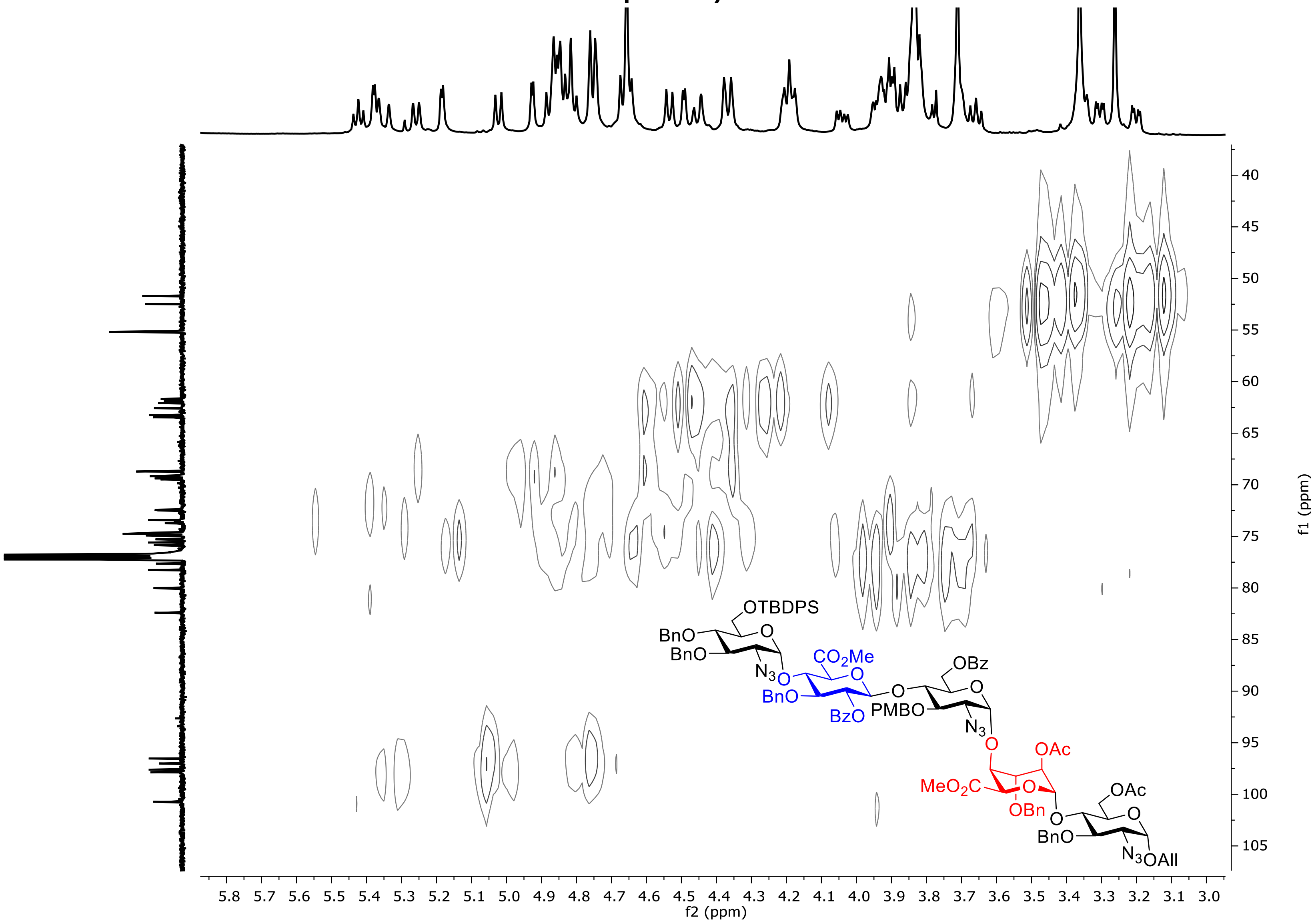

Supplementary Figure S98l. HMBC NMR Spectrum (600.40, 150.99 MHz, $CDCl_3$) of Compound 50a (Carbonyl Coupling)

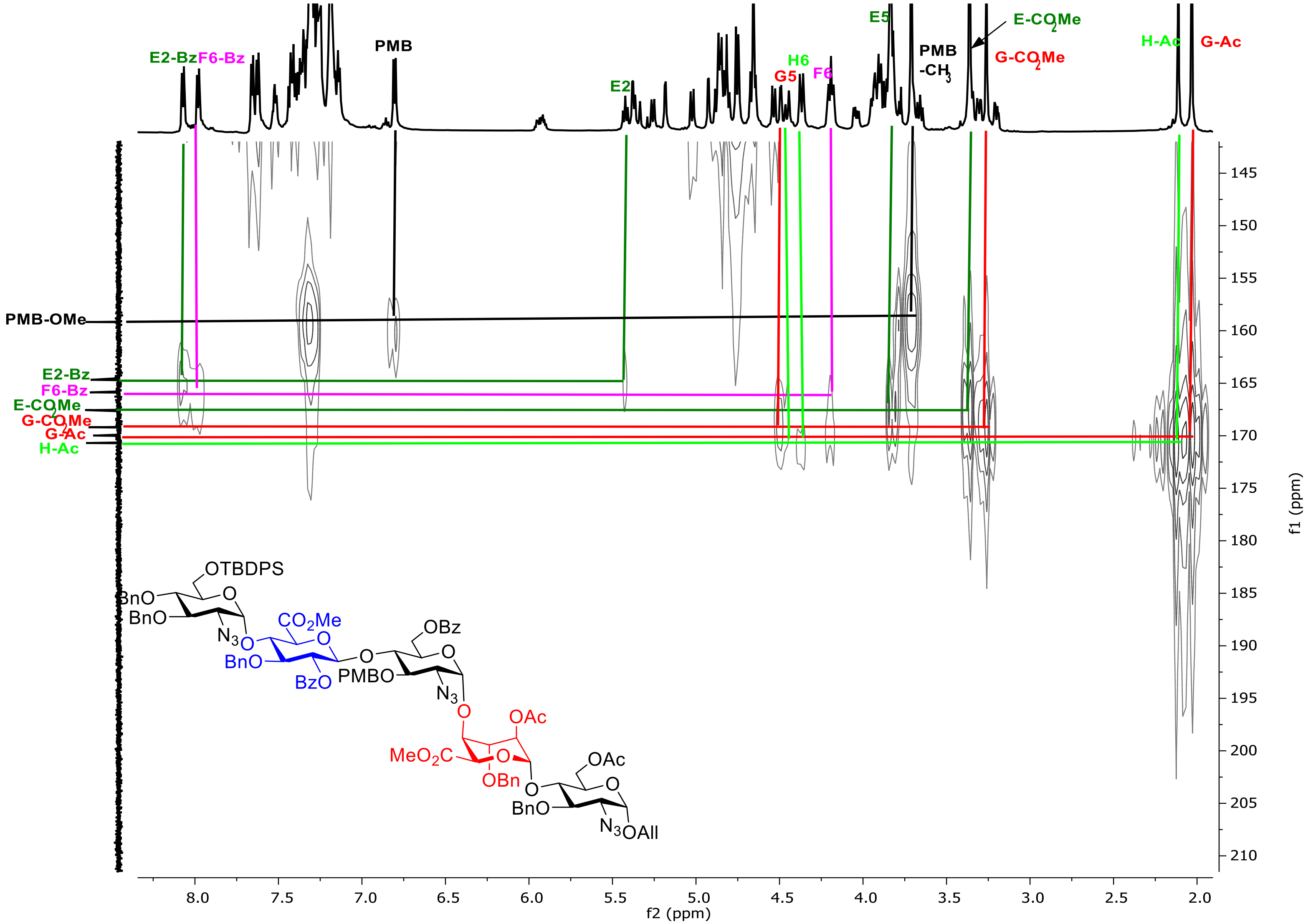

Supplementary Figure S98m. TOCSY NMR Spectrum (600.40, 600.40 MHz, $CDCl_3$) of Compound 50a

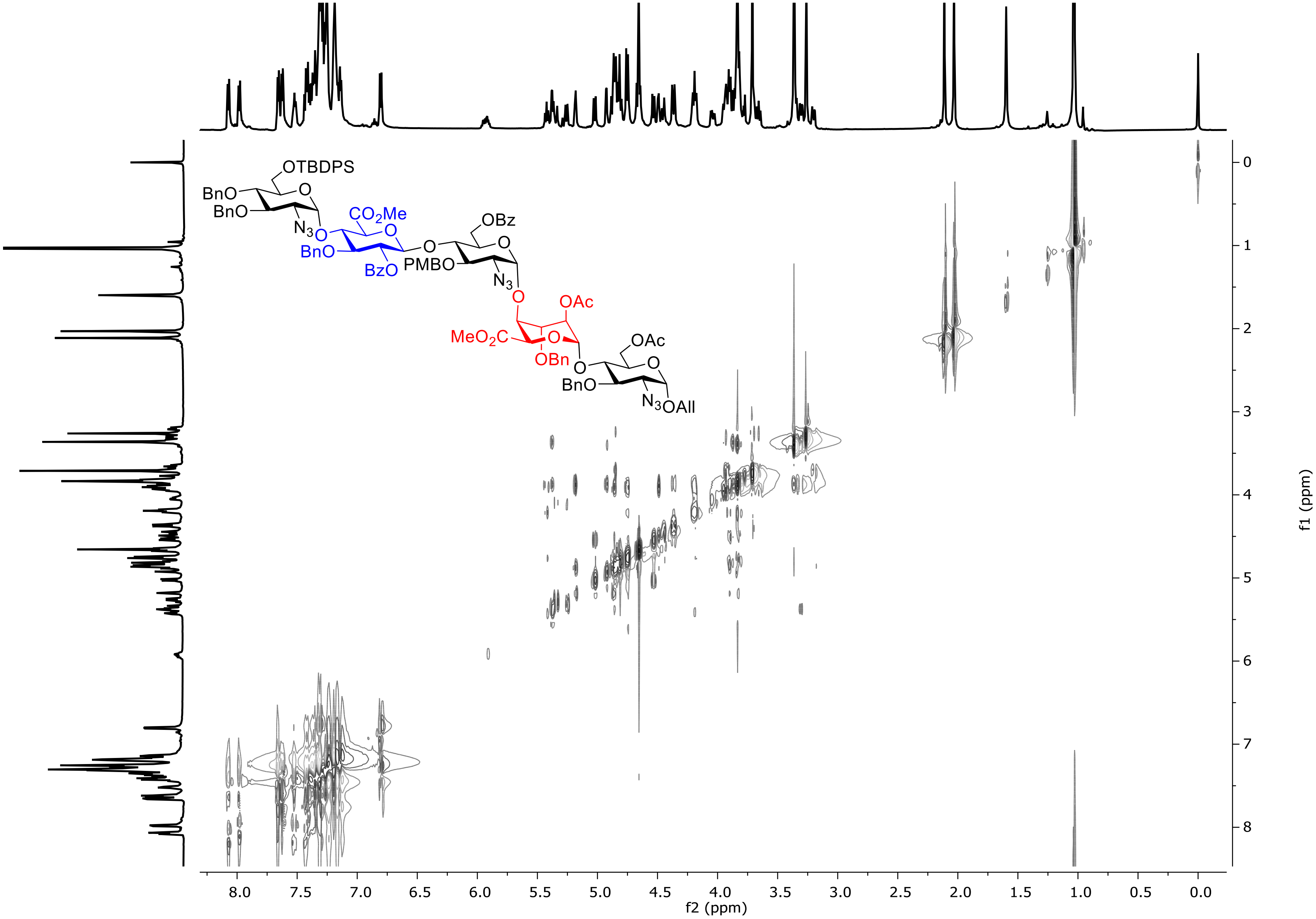

Supplementary Figure S98n. TOCSY NMR Spectrum (600.40, 600.40 MHz, $CDCl_3$) of Compound 50a (Sugar region expanded)

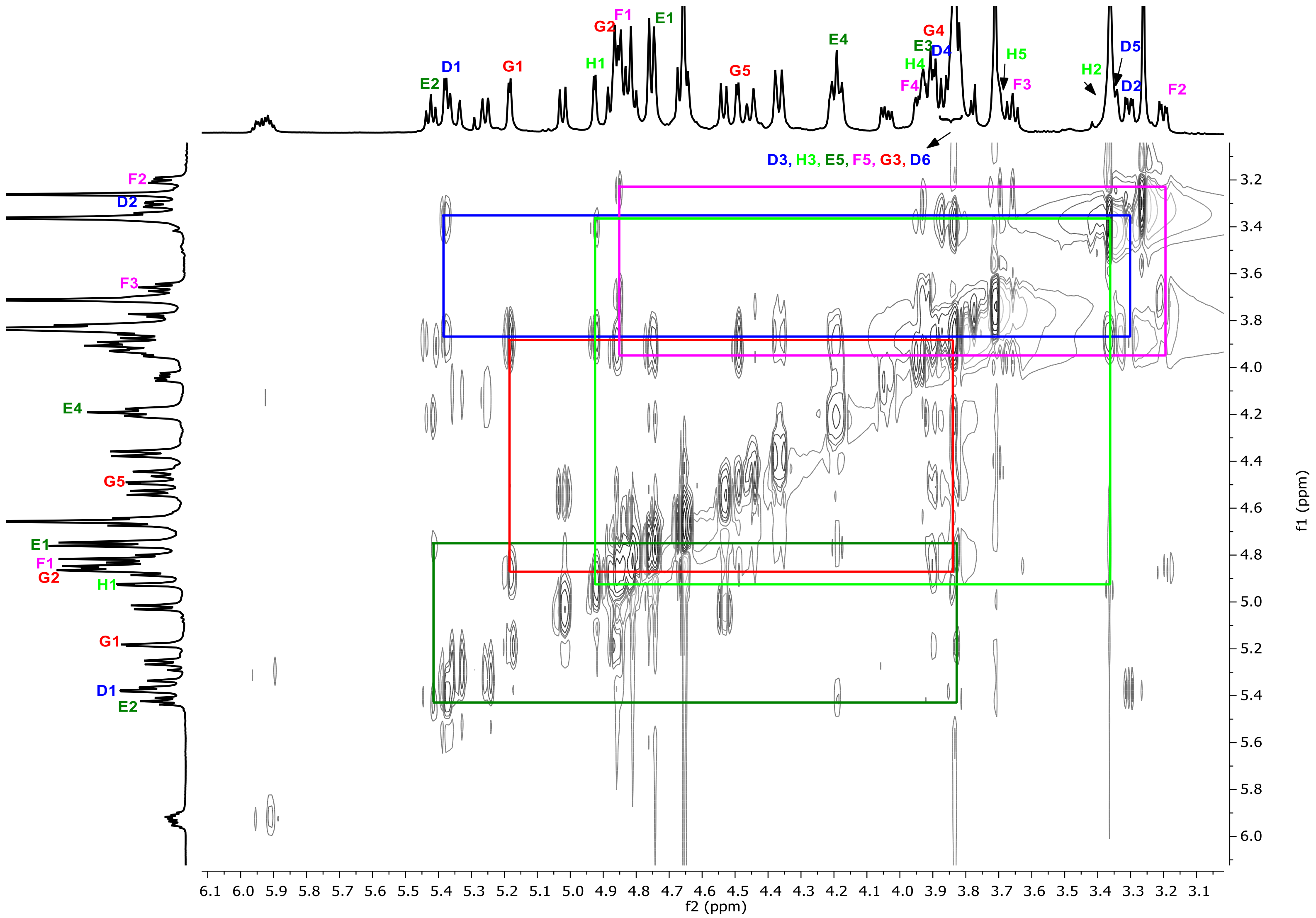

Supplementary Figure S98o. HSQC-TOCSY NMR Spectrum (600.40, 150.99 MHz, $CDCl_3$) of Compound 50a

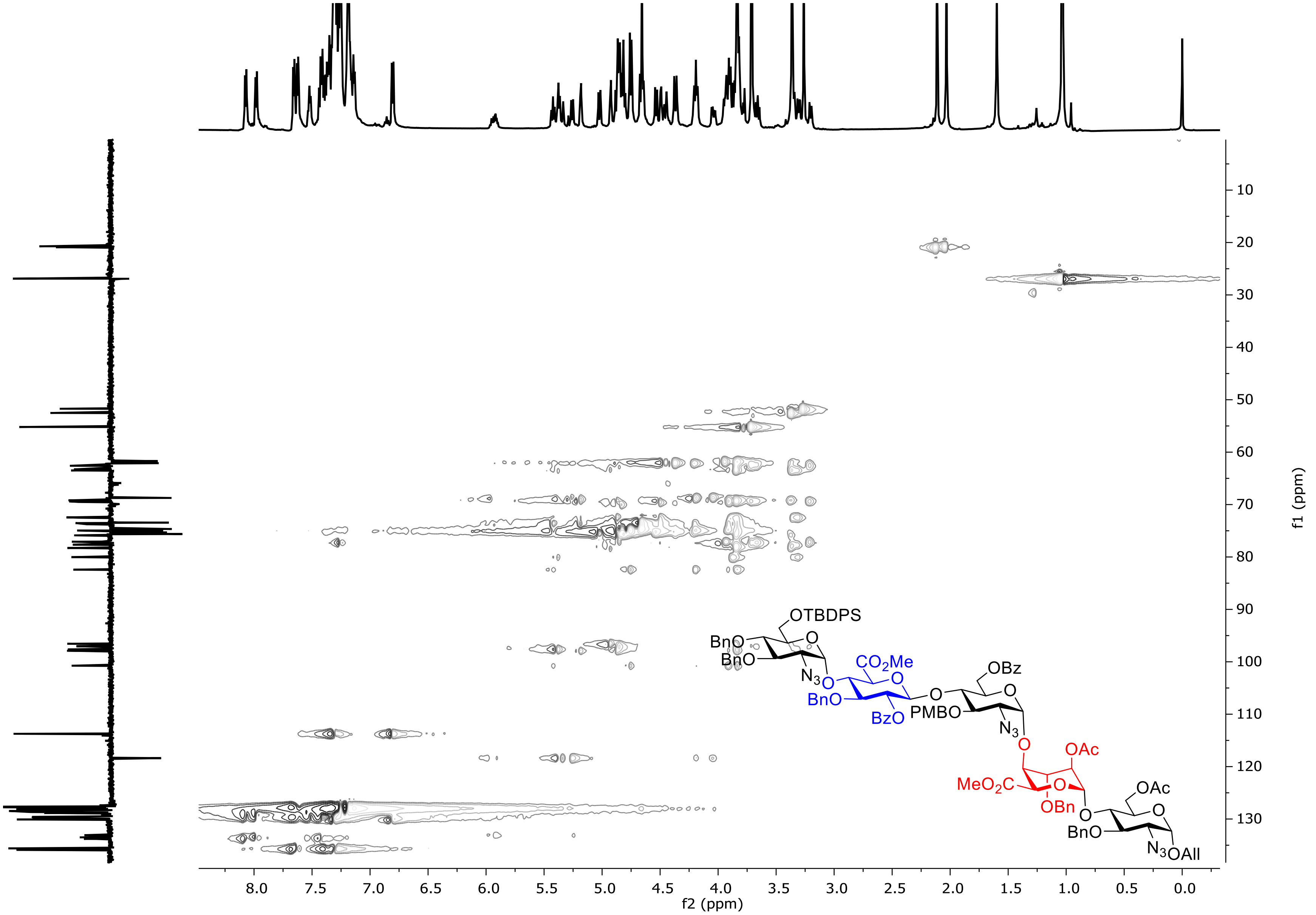

Supplementary Figure S98p. HSQC-TOCSY NMR Spectrum (600.40, 150.99 MHz, $CDCl_3$) of Compound 50a (Sugar region expanded)

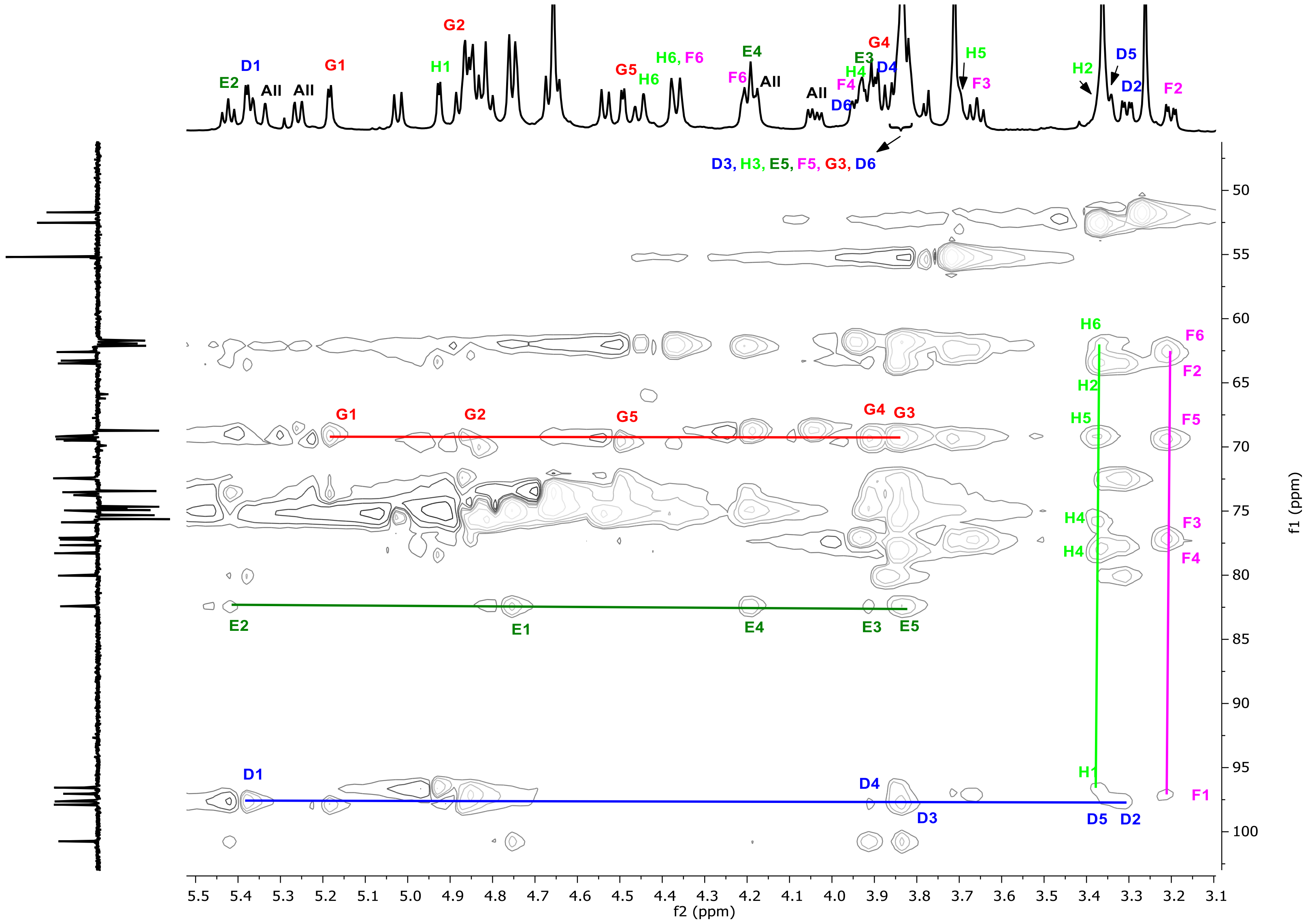

Supplementary Figure S99a. ¹H NMR Spectrum (400.31 MHz, $CDCl_3$) of Compound 48

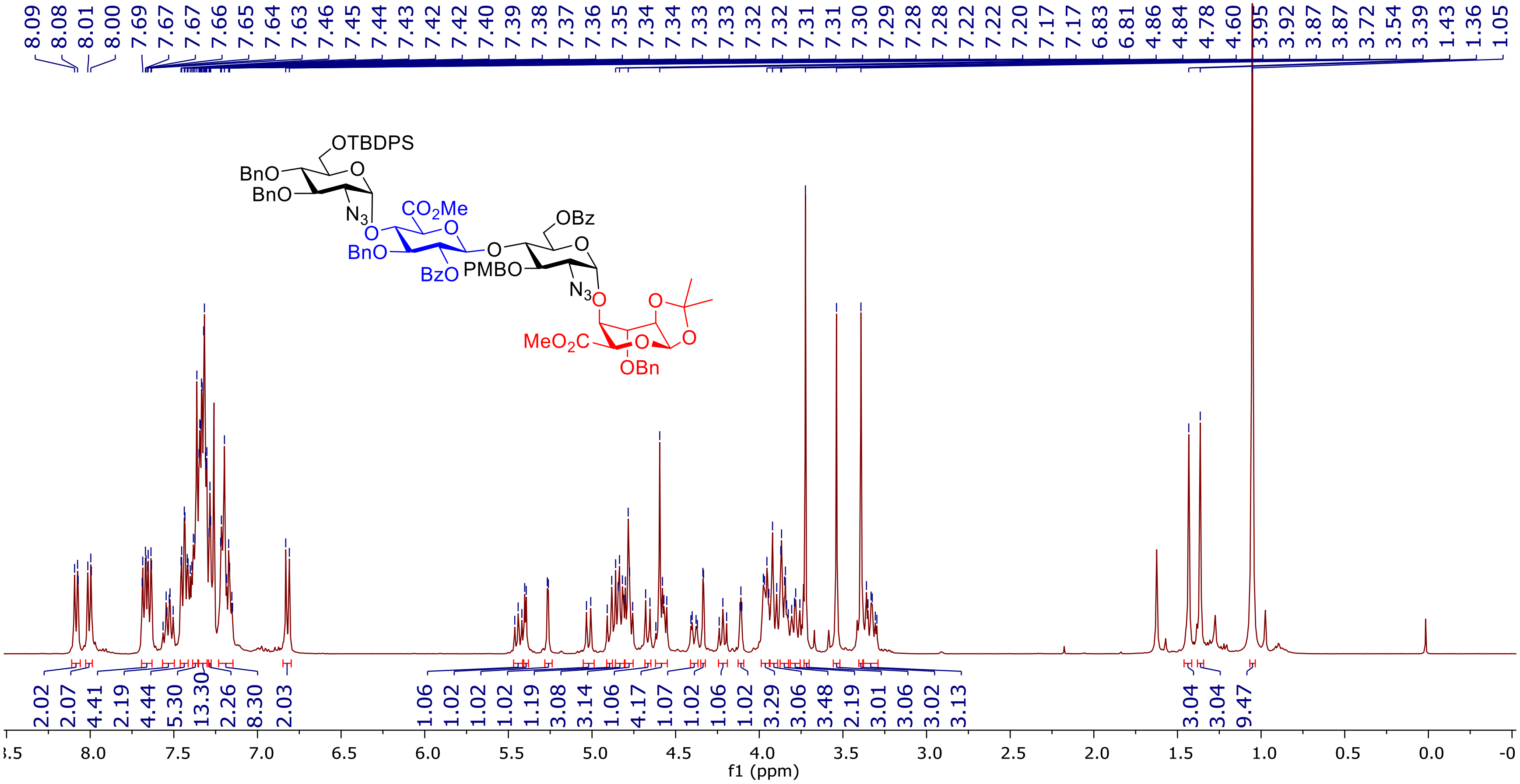

Supplementary Figure S99b. ^{13}C NMR Spectrum (100.67 MHz, $CDCl_3$) of Compound 48

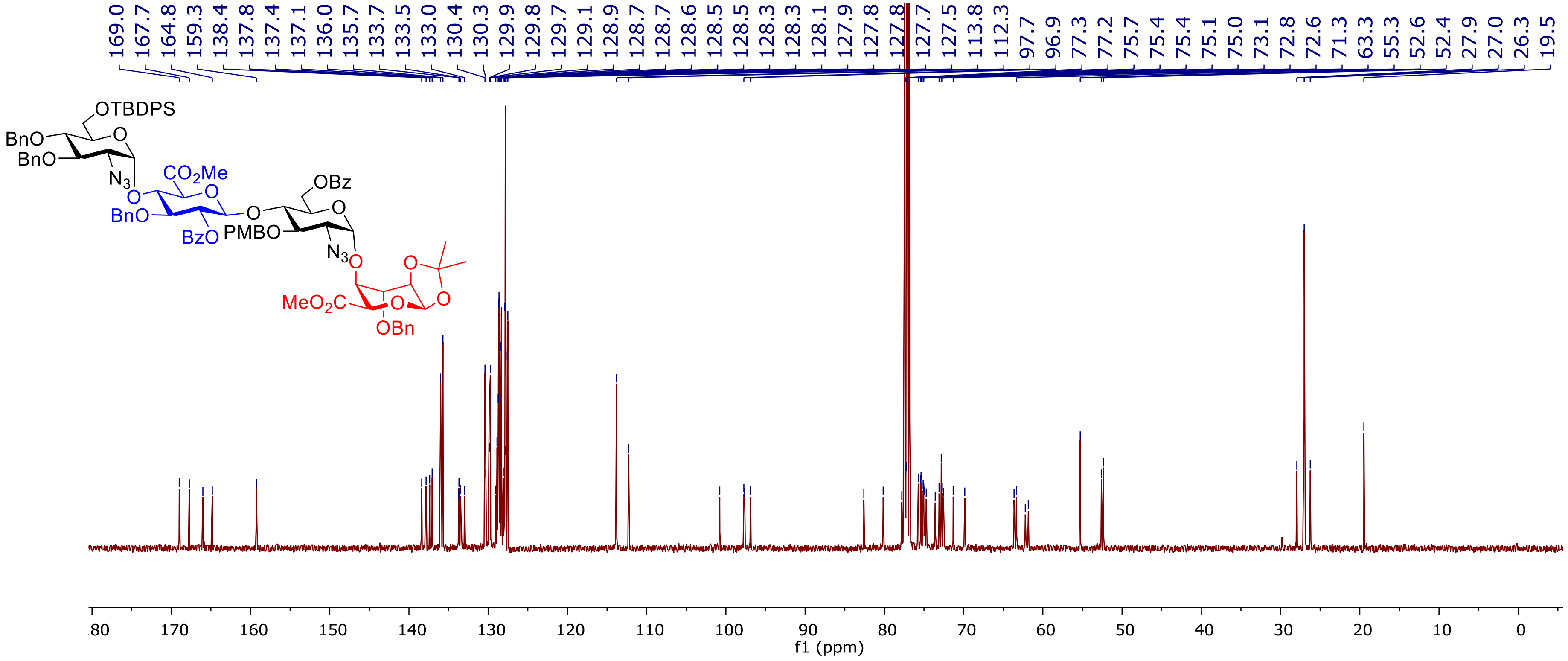

Supplementary Figure S99c. DEPT NMR Spectrum (100.67 MHz, $CDCl_3$) of Compound 48

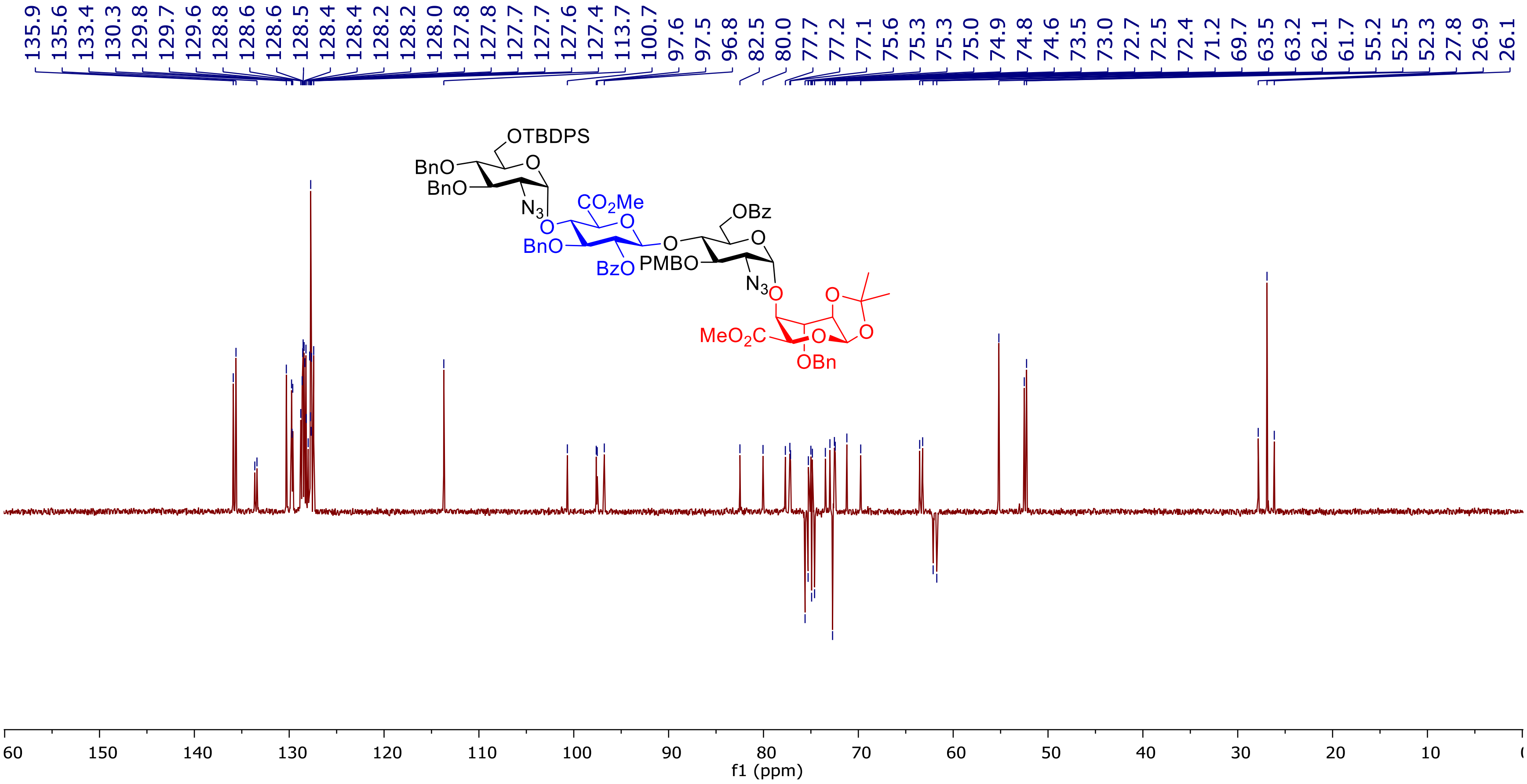

Supplementary Figure S99d. COSY NMR Spectrum (600.40, 600.40 MHz, $CDCl_3$) of Compound 48

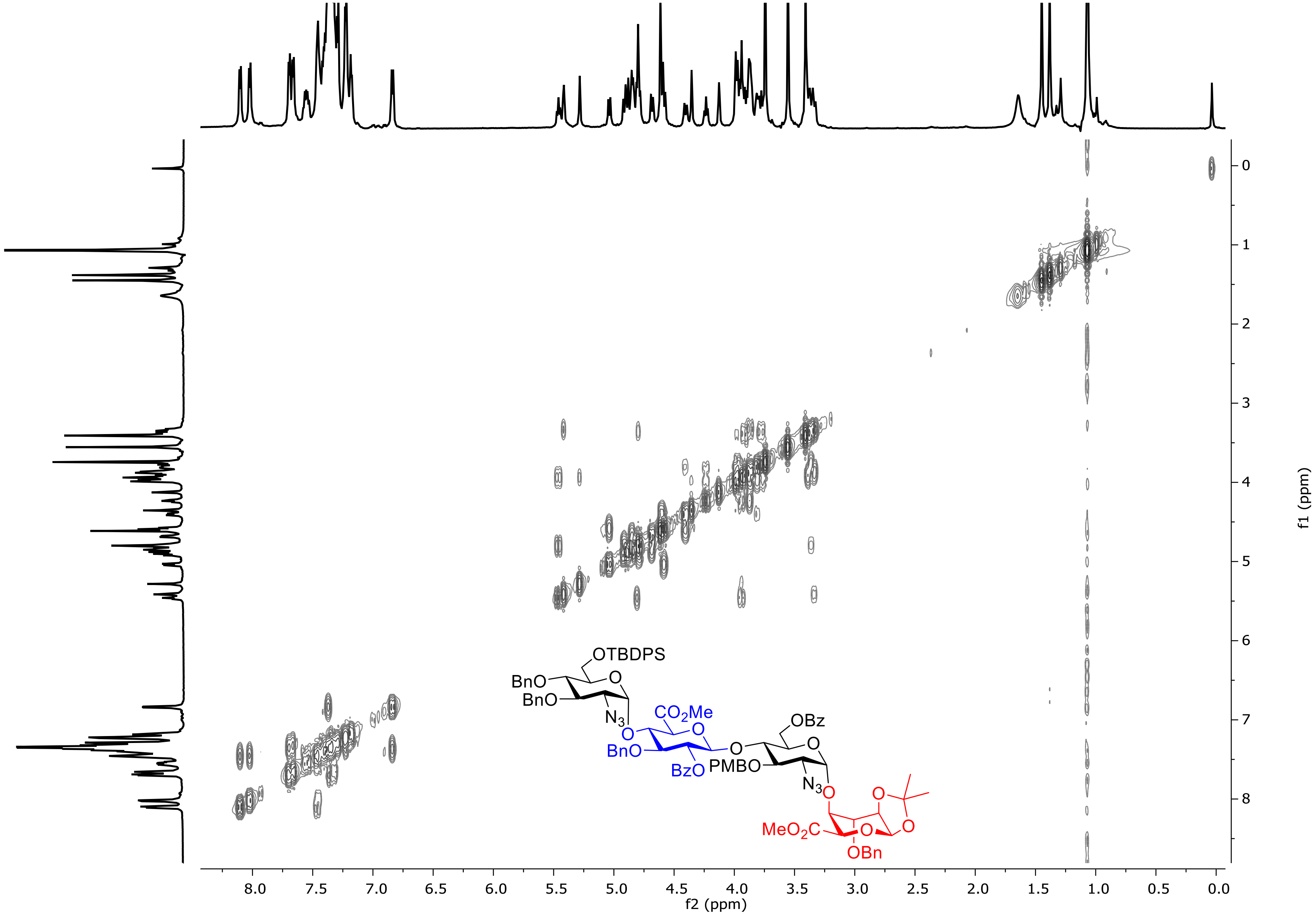

Supplementary Figure S99e. COSY NMR Spectrum (600.40, 600.40 MHz, $CDCl_3$) of Compound 48 (Sugar region expanded)

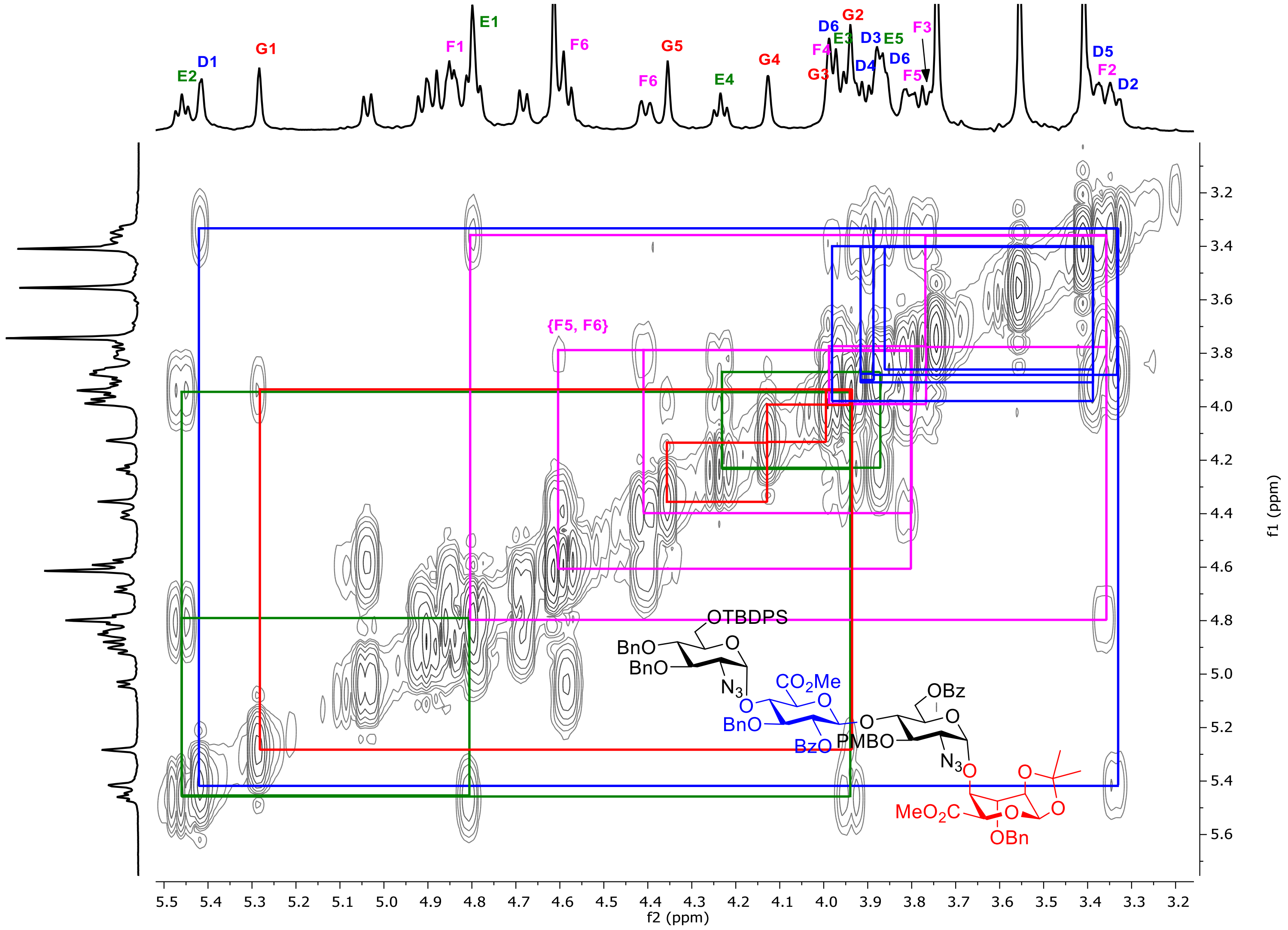

Supplementary Figure S99f. HSQC NMR Spectrum (600.40, 150.99 MHz, $CDCl_3$) of Compound 48

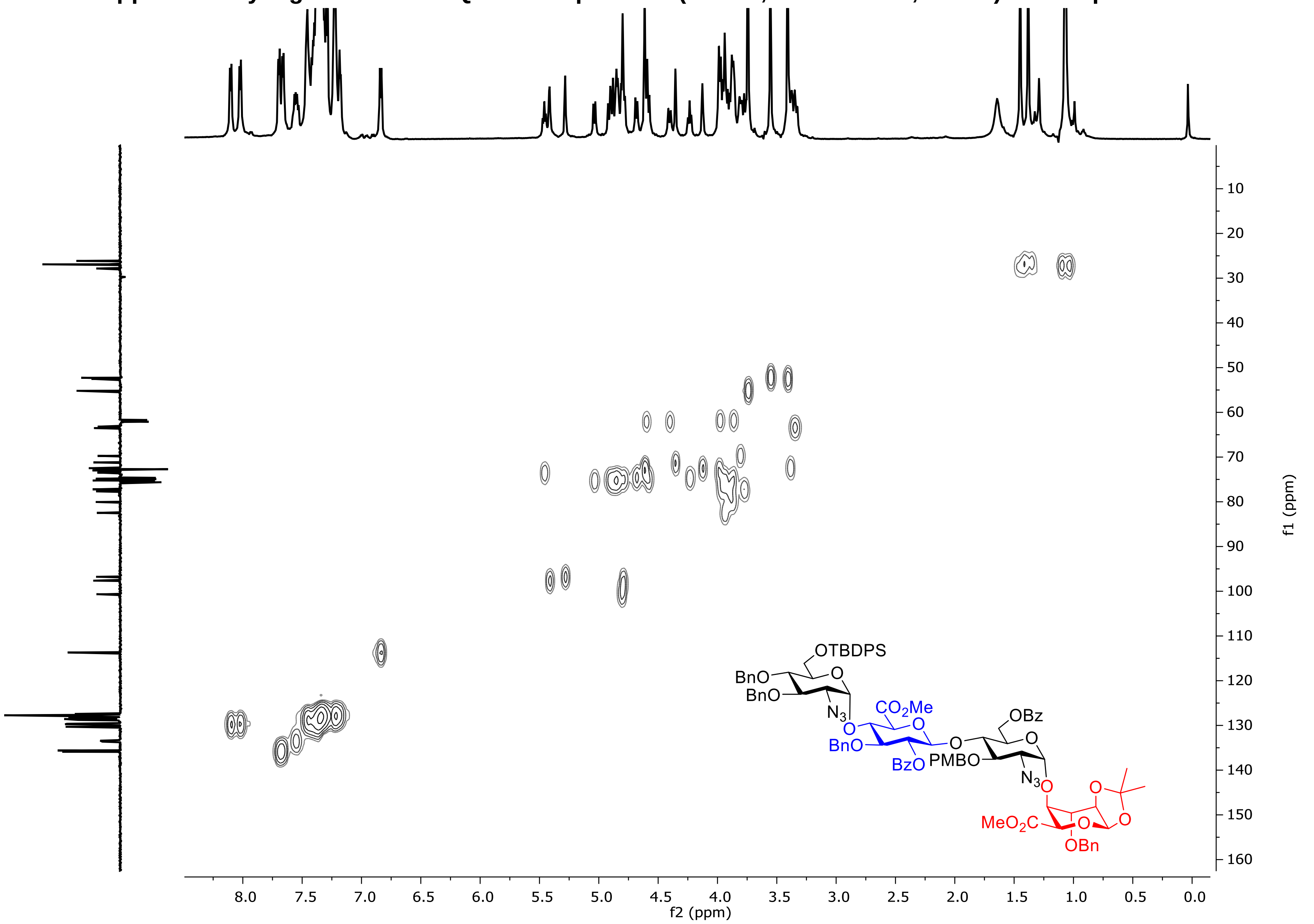

Supplementary Figure S99g. HSQC NMR Spectrum (600.40, 150.99 MHz, CDCl3) of Compound 48 (Sugar region expanded)

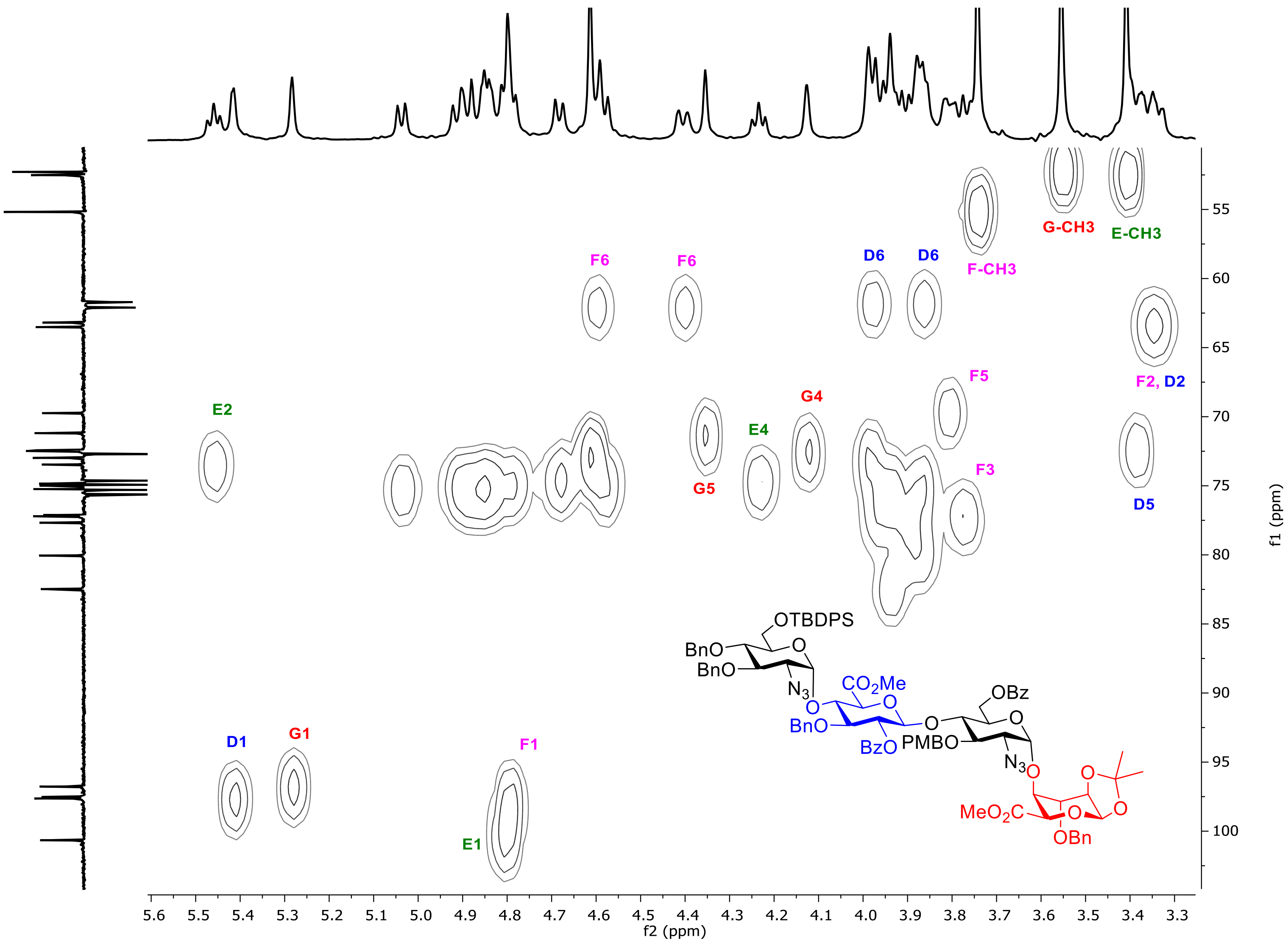

Supplementary Figure S99h. Coupled HSQC NMR Spectrum (600.40, 150.99 MHz, $CDCl_3$) of Compound 48

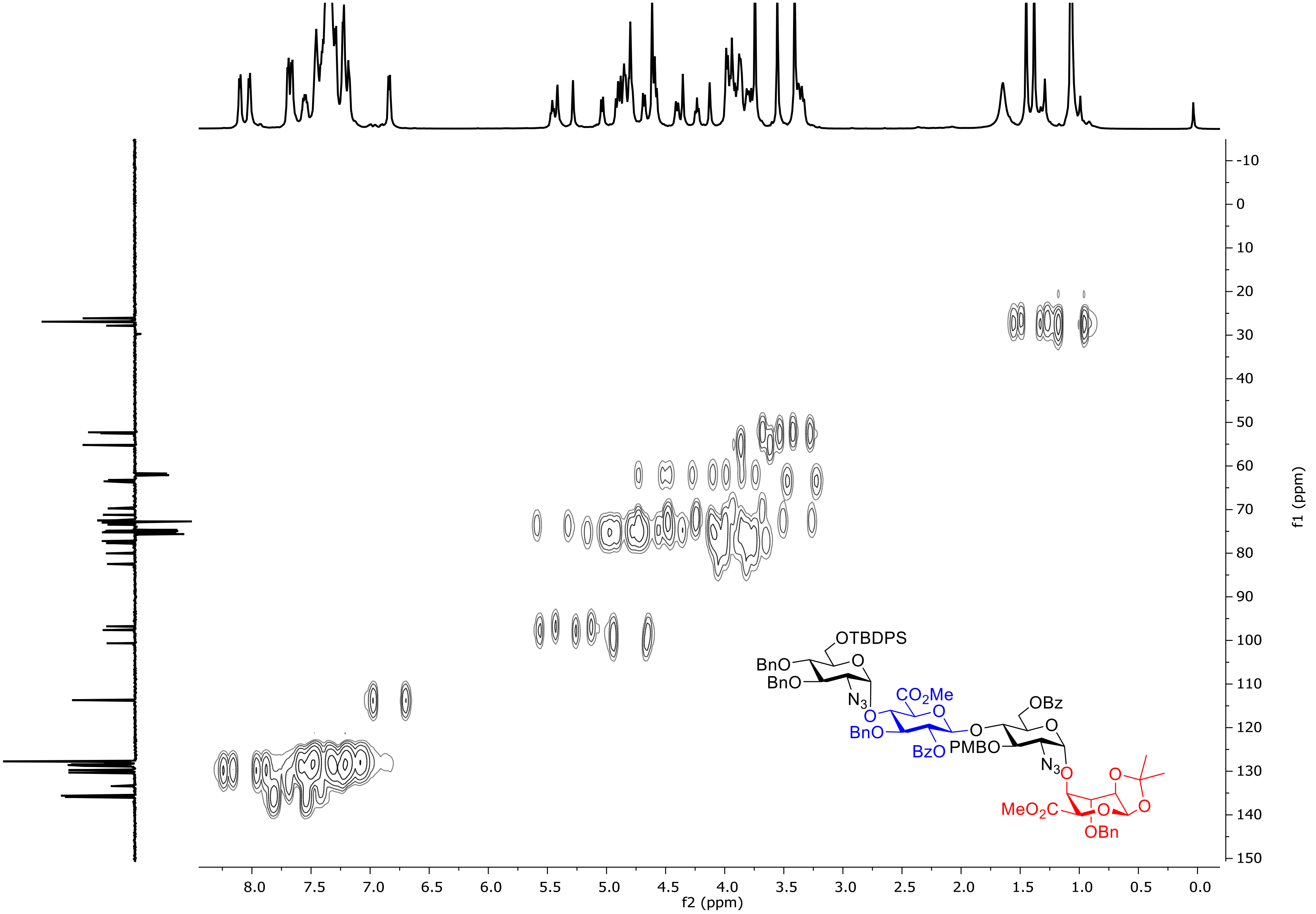

Supplementary Figure S99i. Coupled HSQC NMR Spectrum (600.40, 150.99 MHz, $CDCl_3$) of Compound 48 (Sugar region expanded)

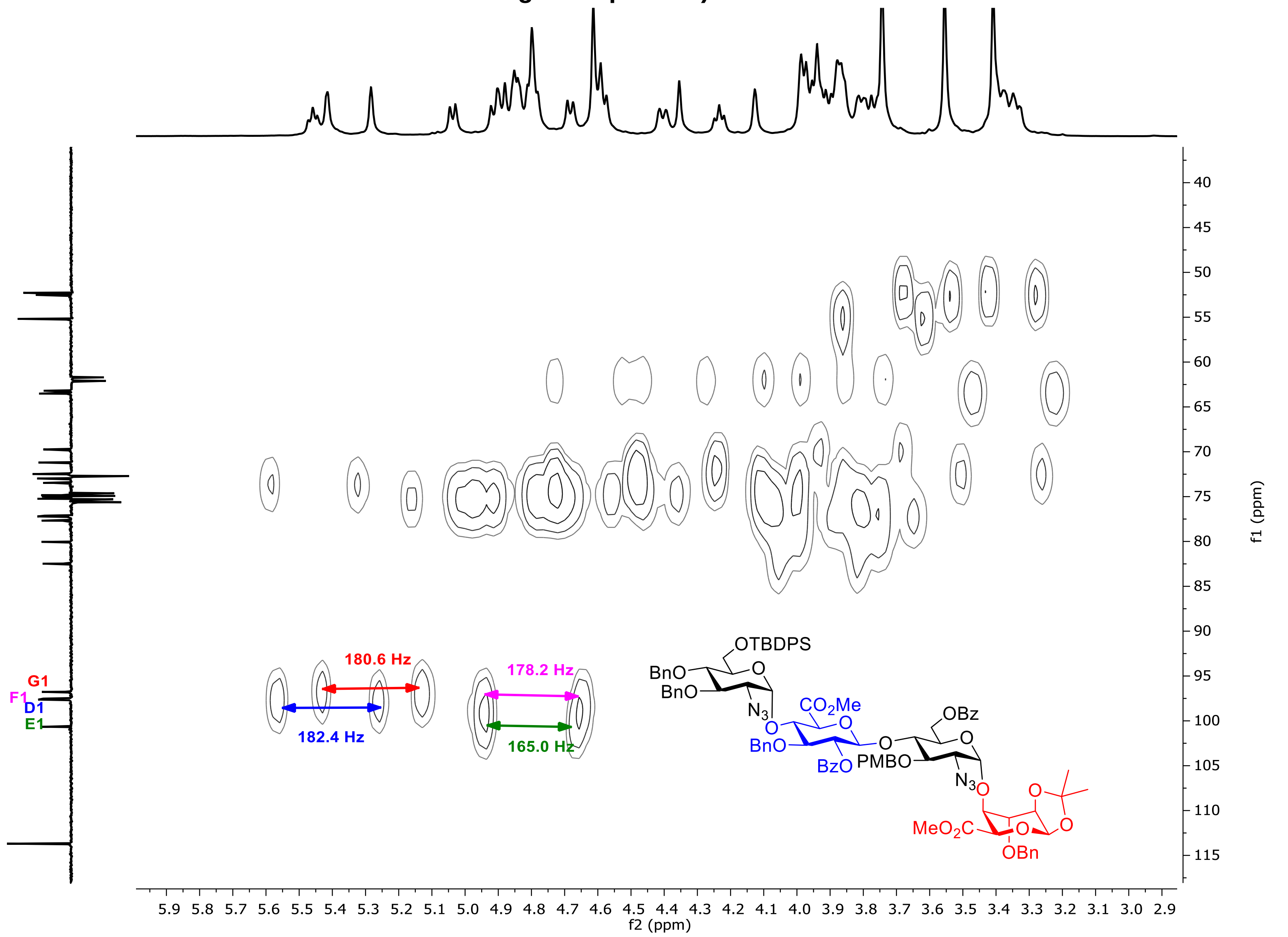

Supplementary Figure S99j. HMBC NMR Spectrum (600.40, 150.99 MHz, $CDCl_3$) of Compound 48

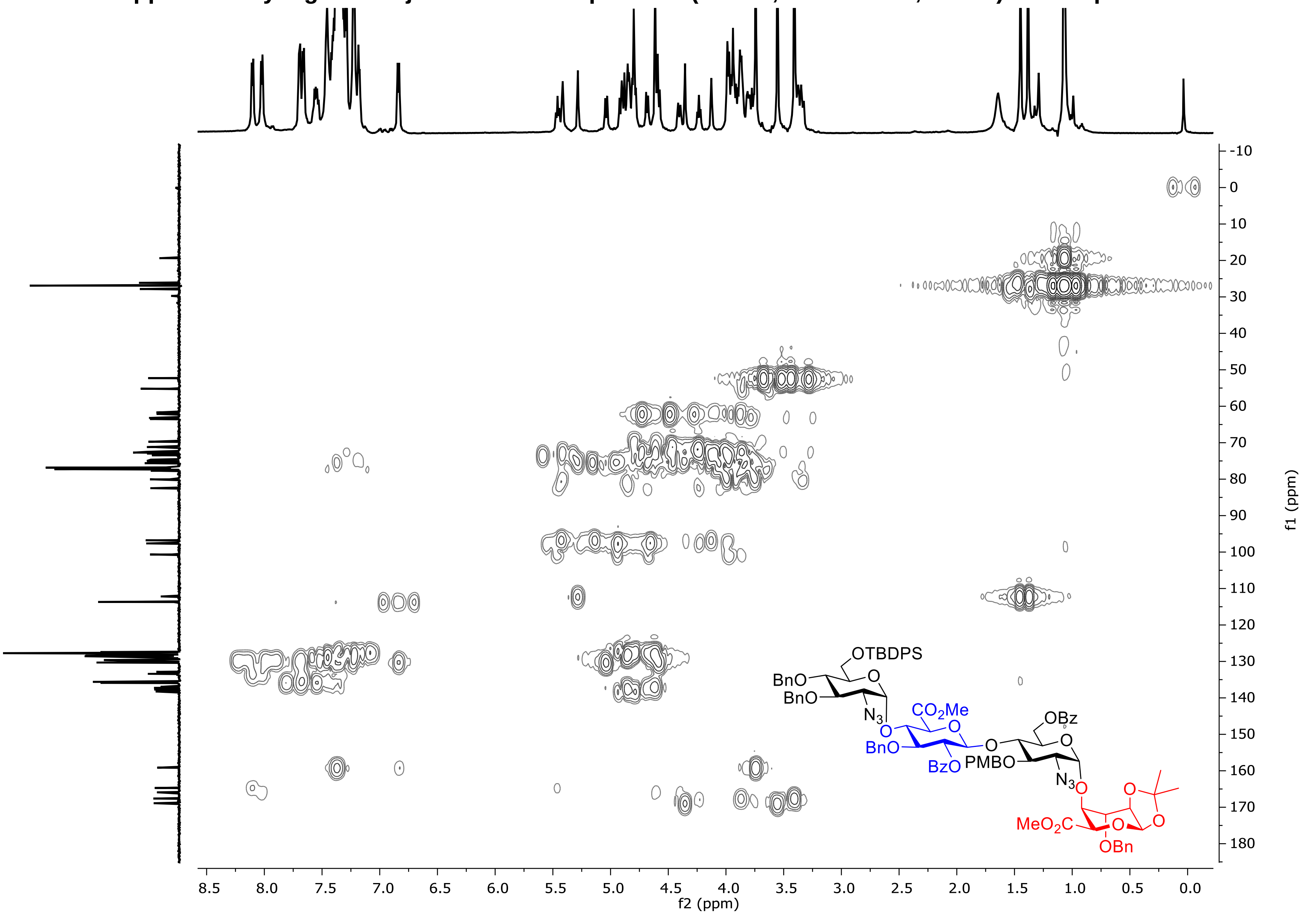

Supplementary Figure S99k. HMBC Spectrum (600.40, 150.99 MHz, $CDCl_3$) of Compound 48 (Sugar region expanded)

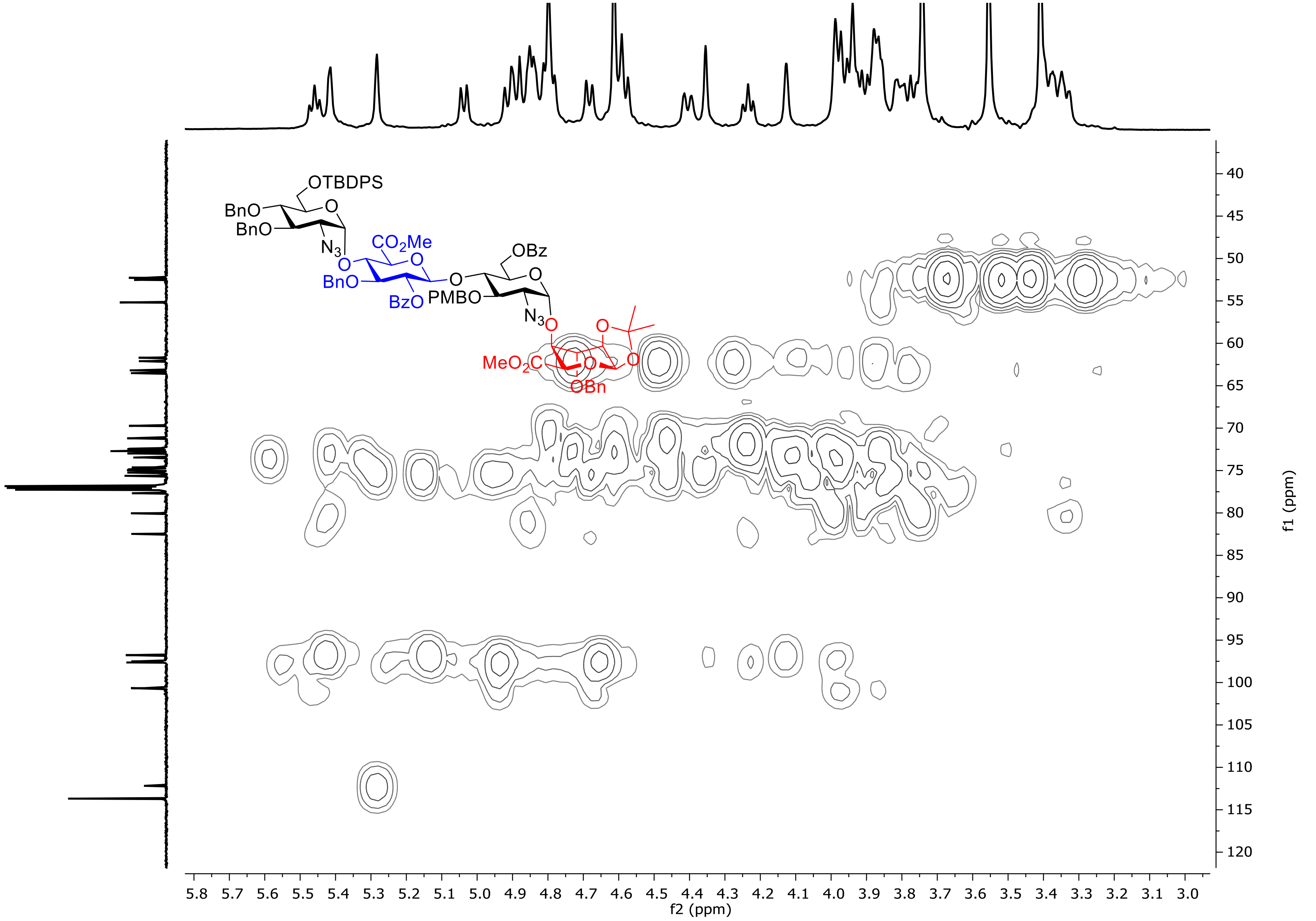

Supplementary Figure S99l. HMBC NMR Spectrum (600.40, 150.99 MHz, $CDCl_3$) of Compound 48 (Carbonyl Region expanded)

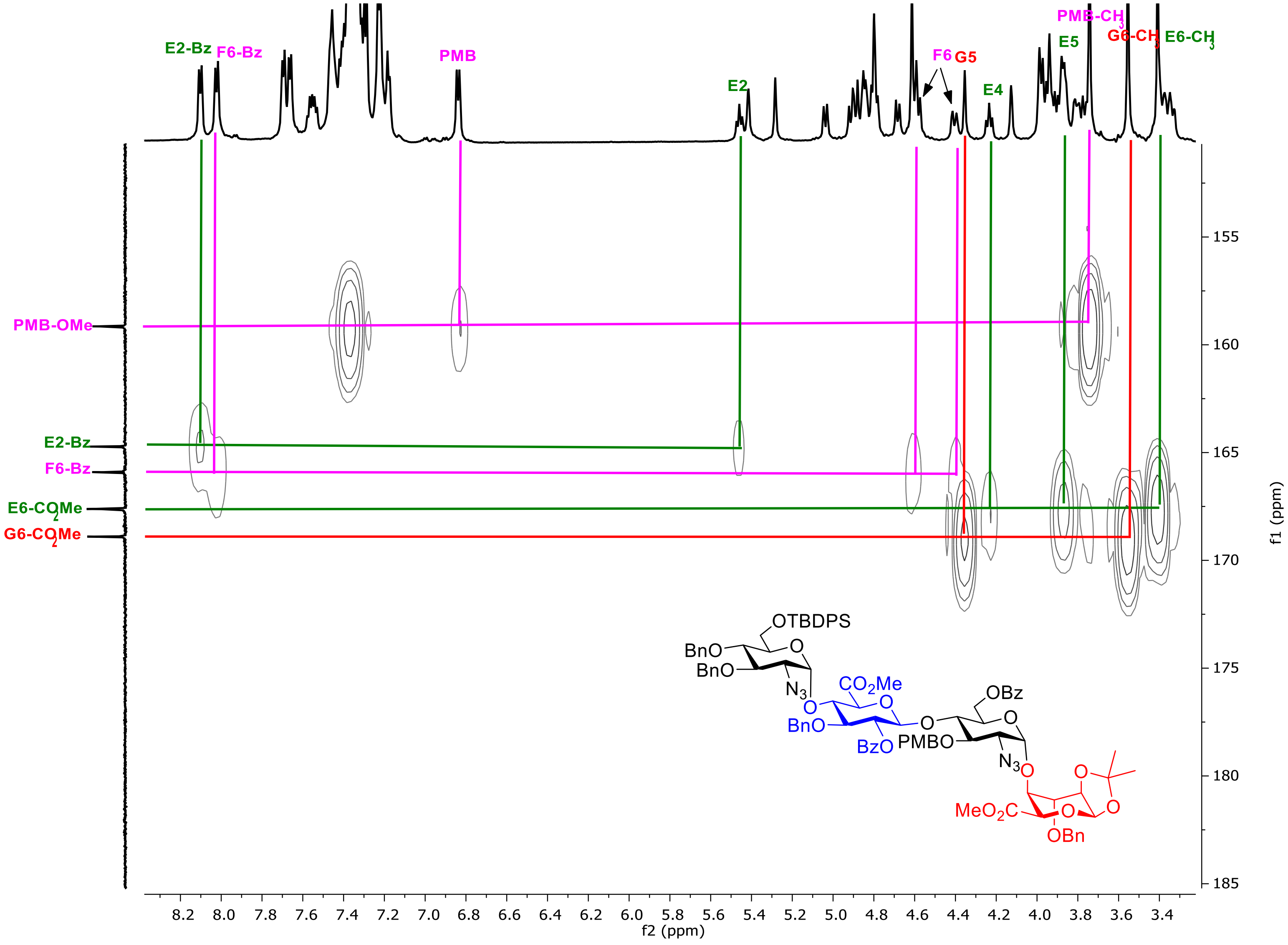

Supplementary Figure S99m. TOCSY NMR Spectrum (400.31, 400.31 MHz, $CDCl_3$) of Compound 48

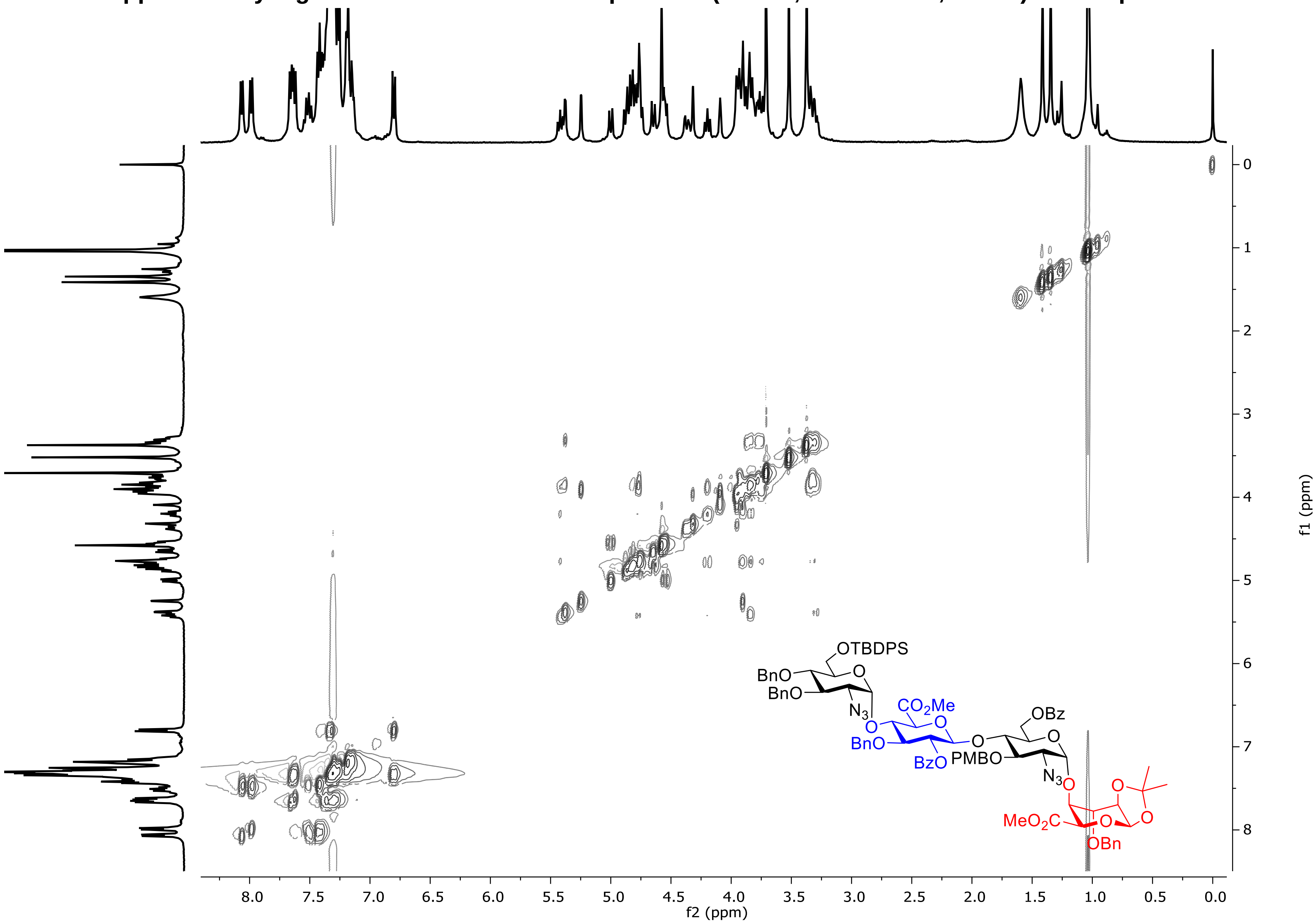

Supplementary Figure S99n. TOCSY NMR Spectrum (400.31, 400.31 MHz, $CDCl_3$) of Compound 48 (Sugar region expanded)

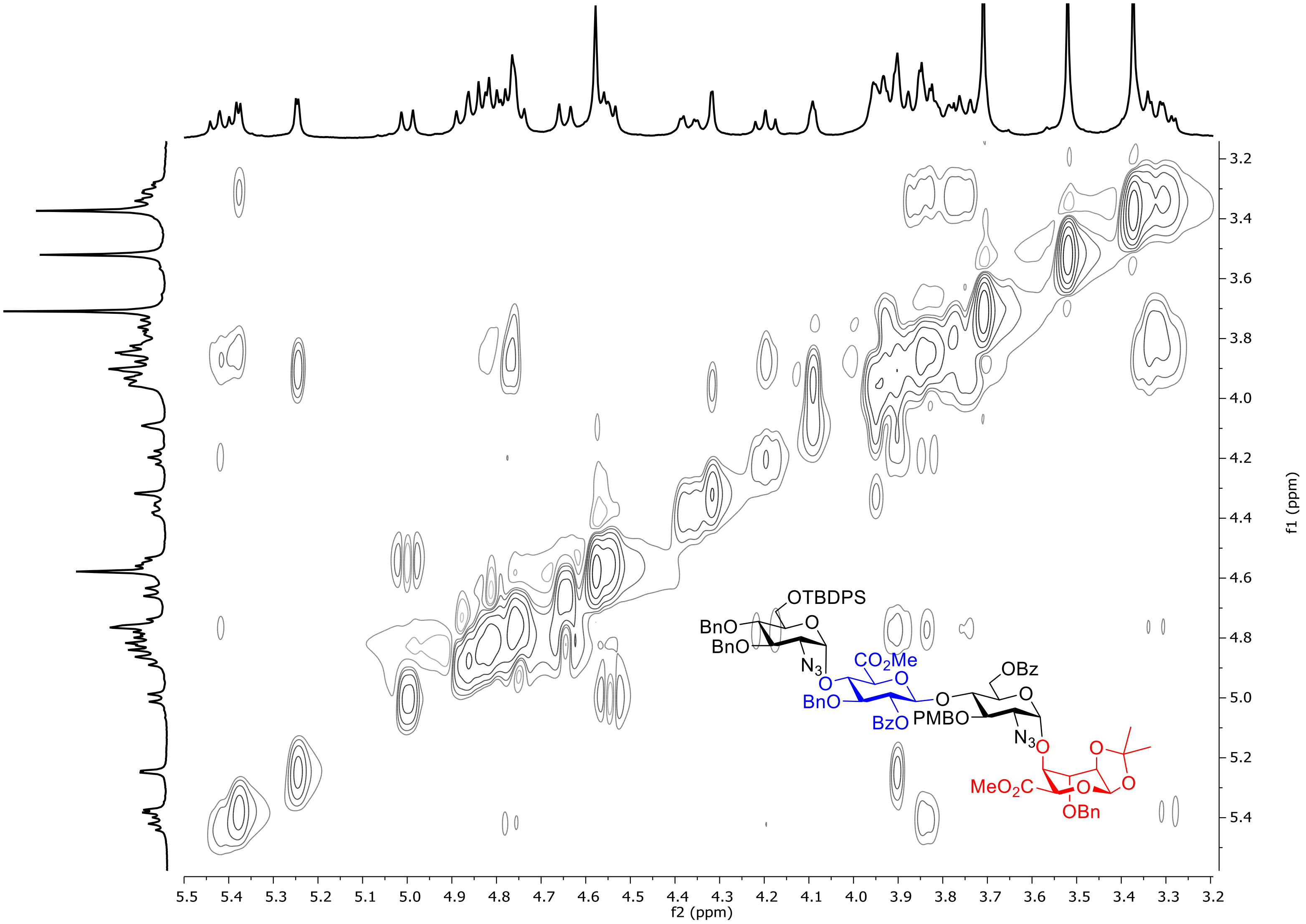

Supplementary Figure S99o. HSQC-TOCSY NMR Spectrum (400.31, 100.67 MHz, $CDCl_3$) of Compound 48

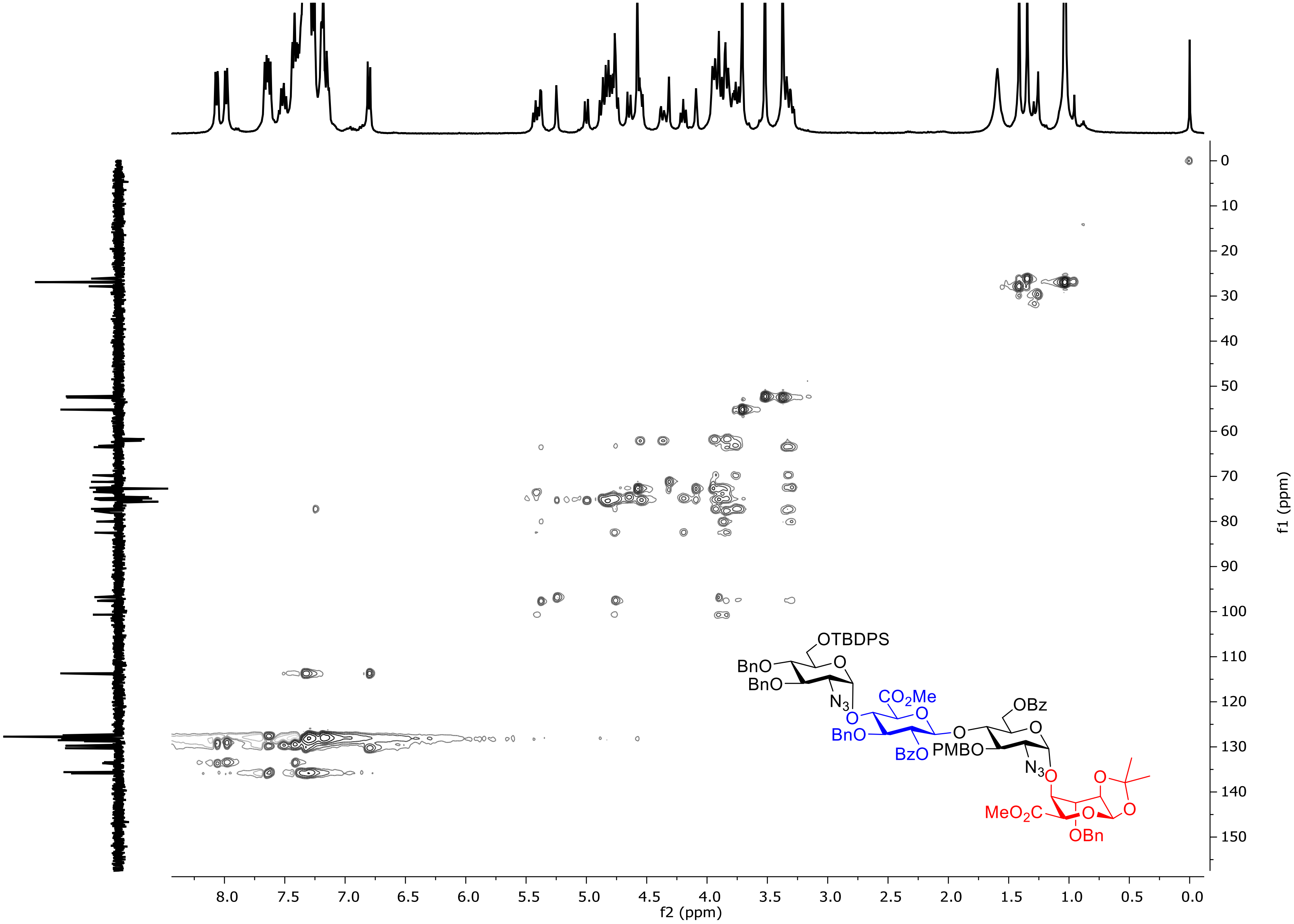

Supplementary Figure S99p. HSQC-TOCSY NMR Spectrum (400.31, 100.67 MHz, CDCl₃) of Compound 48 (Sugar region expanded)

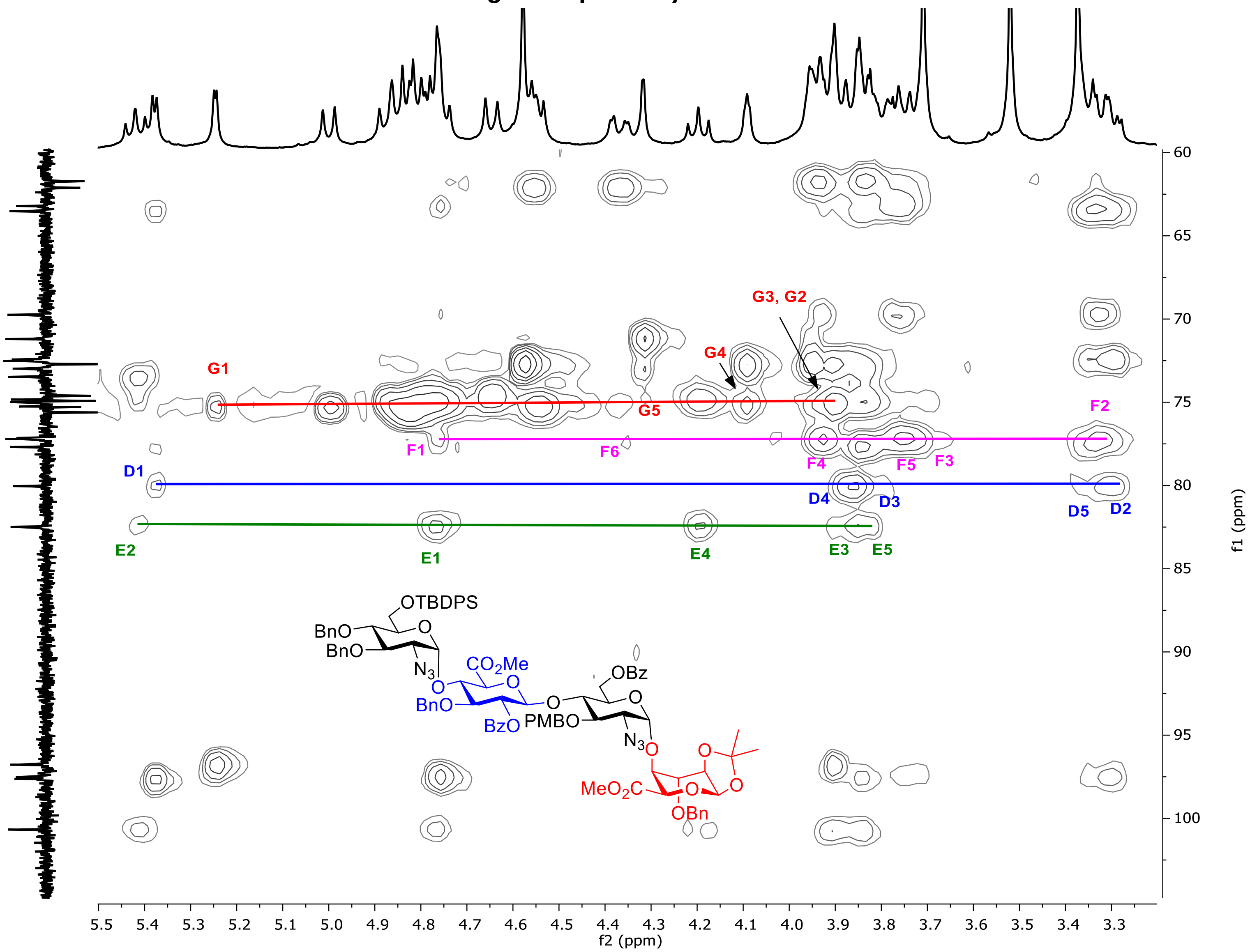

Supplementary Figure S100a. ^{1}H NMR Spectrum (400.31 MHz, CDCl₃) of Compound 49

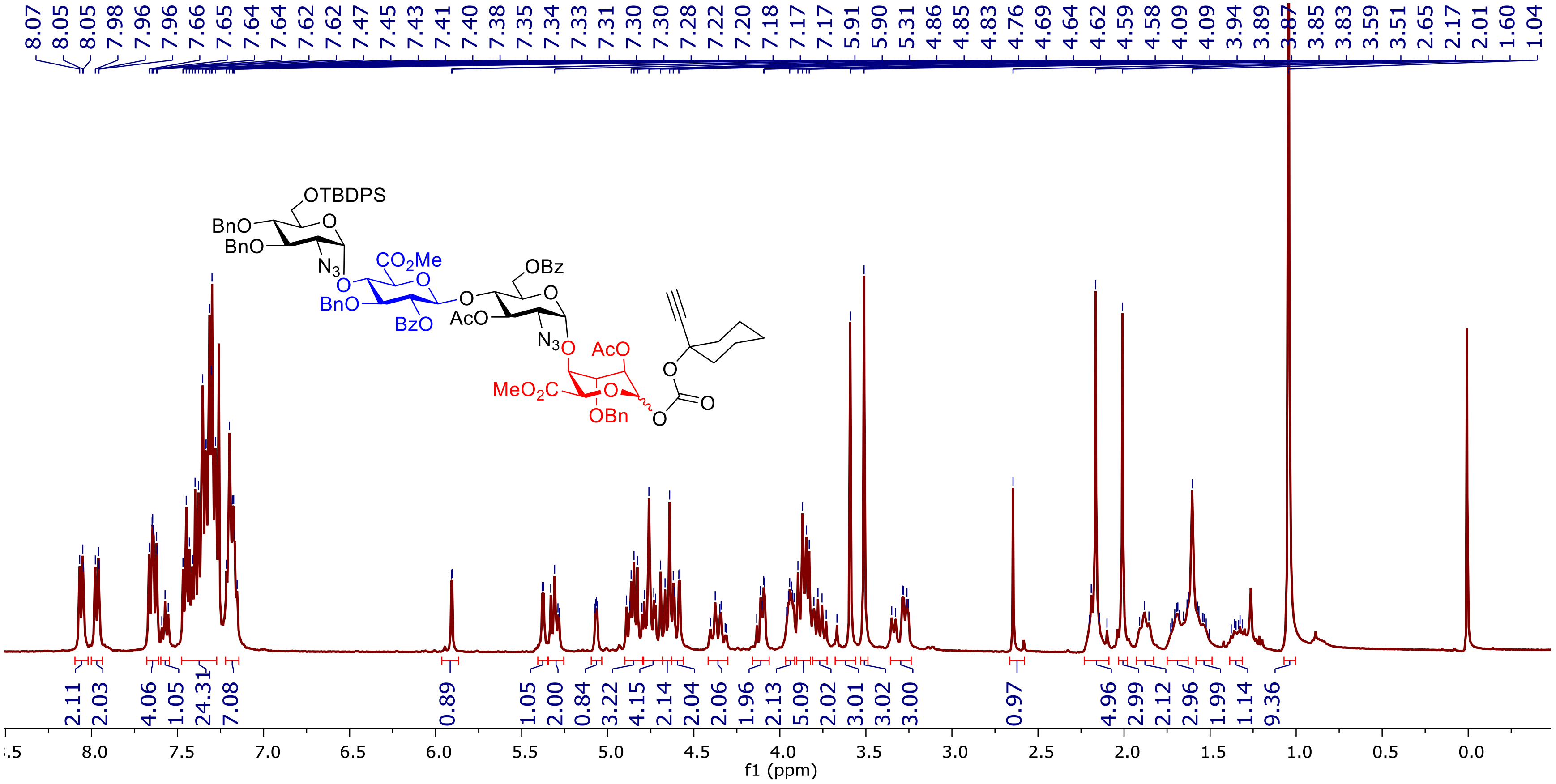

Supplementary Figure S100b. ^{13}C NMR Spectrum (100.67 MHz, CDCl$_3$) of Compound 49

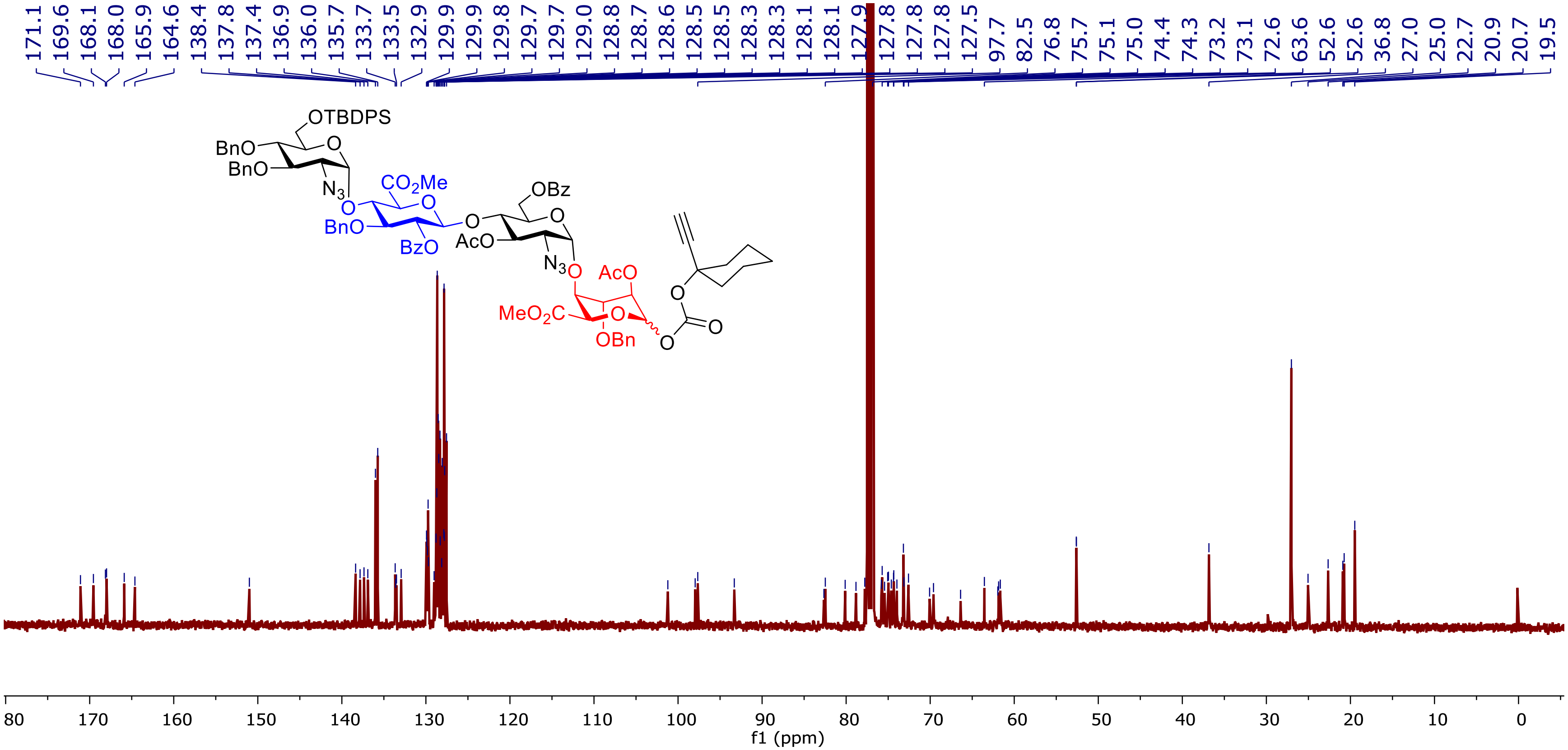

Supplementary Figure S100c. DEPT NMR Spectrum (100.67 MHz, CDCl₃) of Compound 49

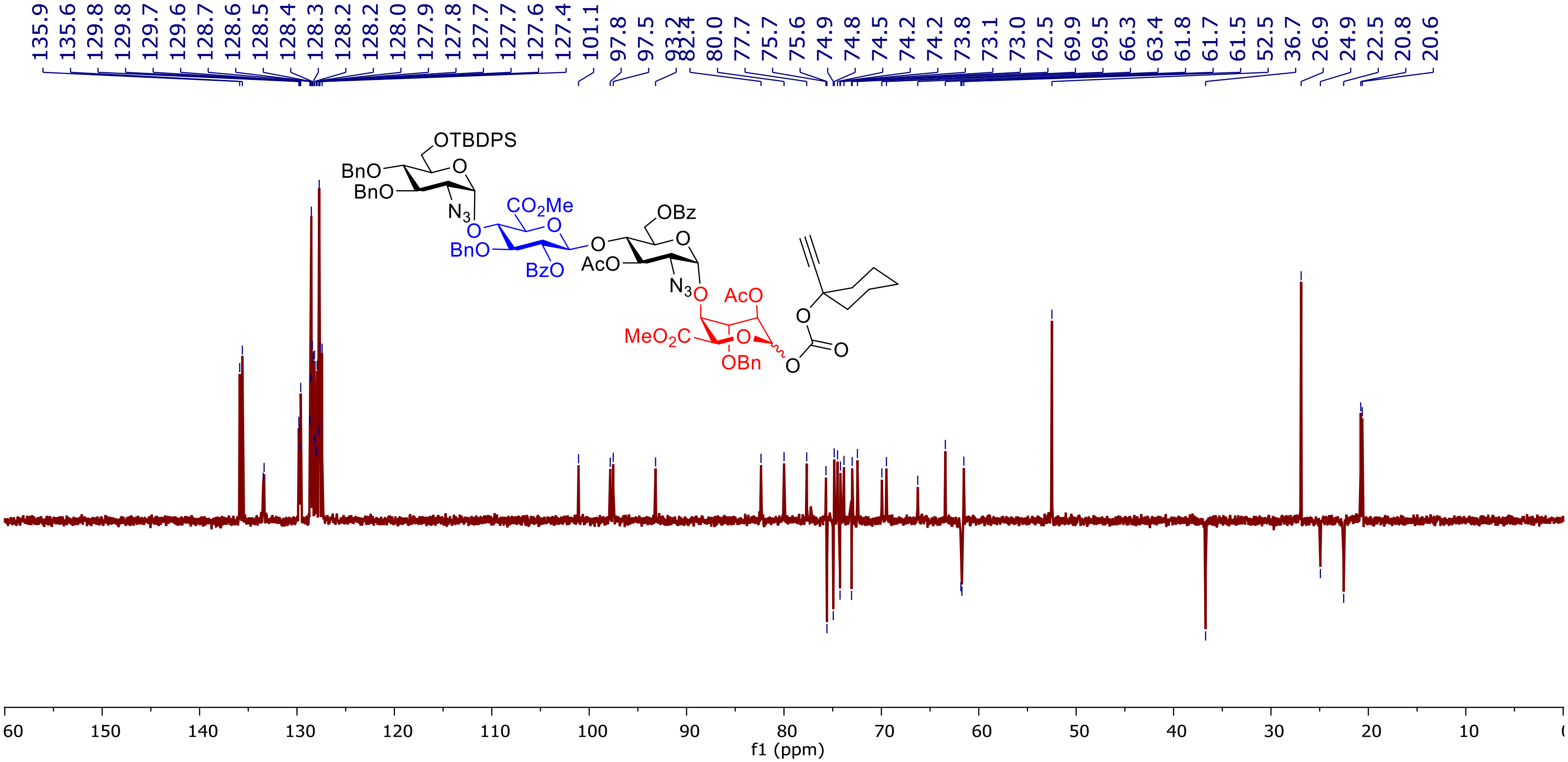

Supplementary Figure S101a. ¹H NMR Spectrum (399.78 MHz, CDCl₃) of Compound 50b

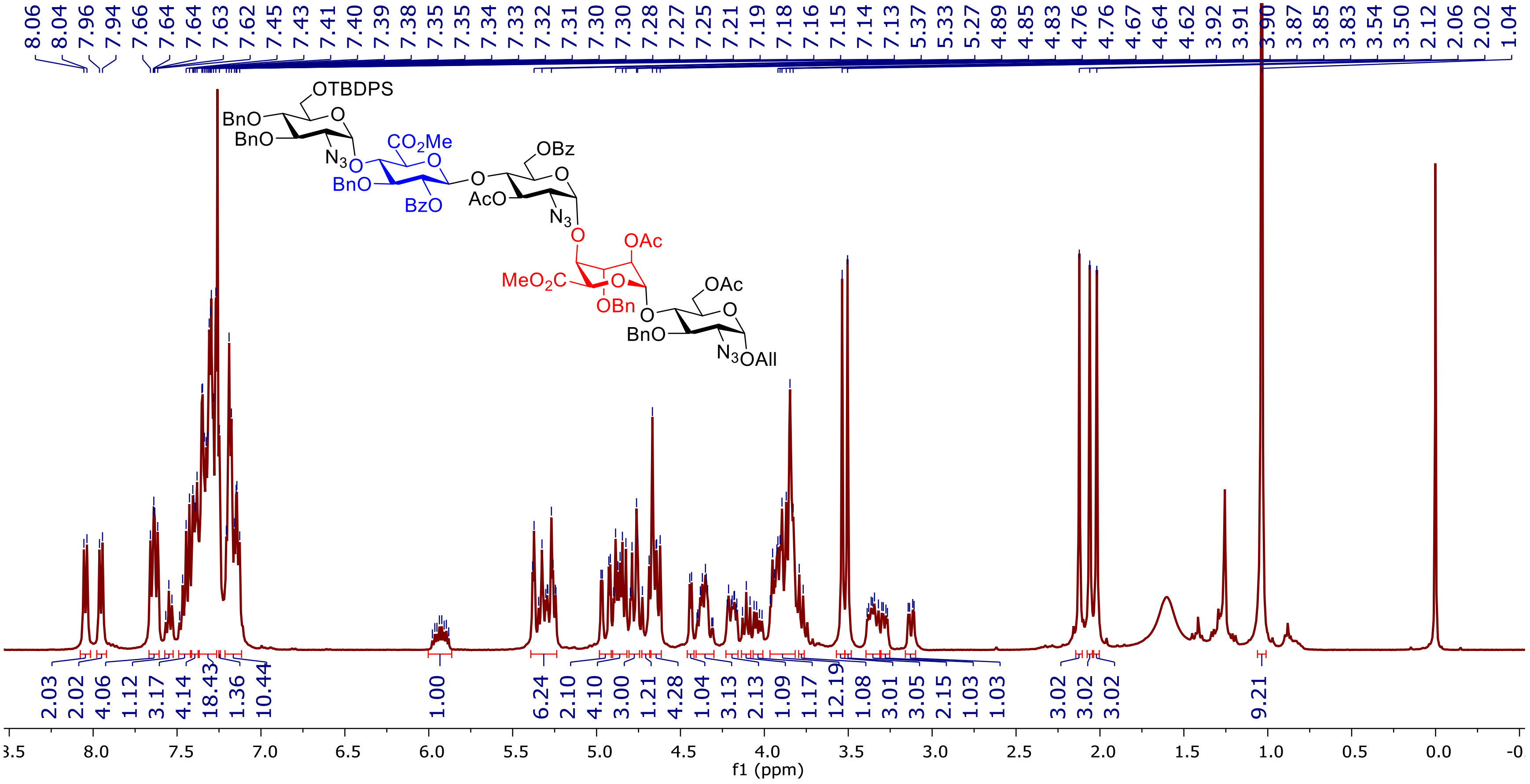

Supplementary Figure S101b. ^{13}C NMR Spectrum (150.97 MHz, $CDCl_3$) of Compound 50b

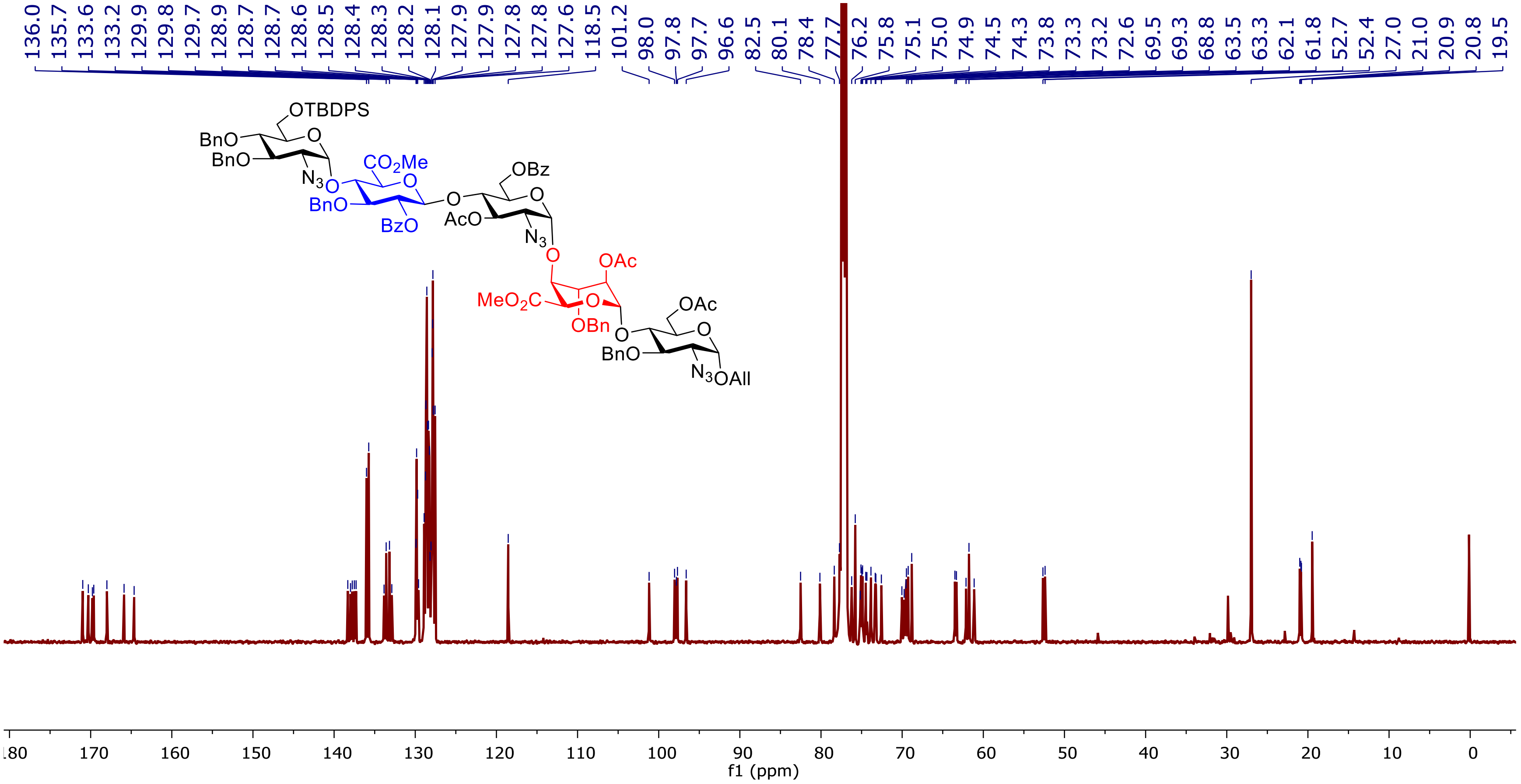

Supplementary Figure S101c. DEPT NMR Spectrum (150.97 MHz, $CDCl_3$) of Compound 50b

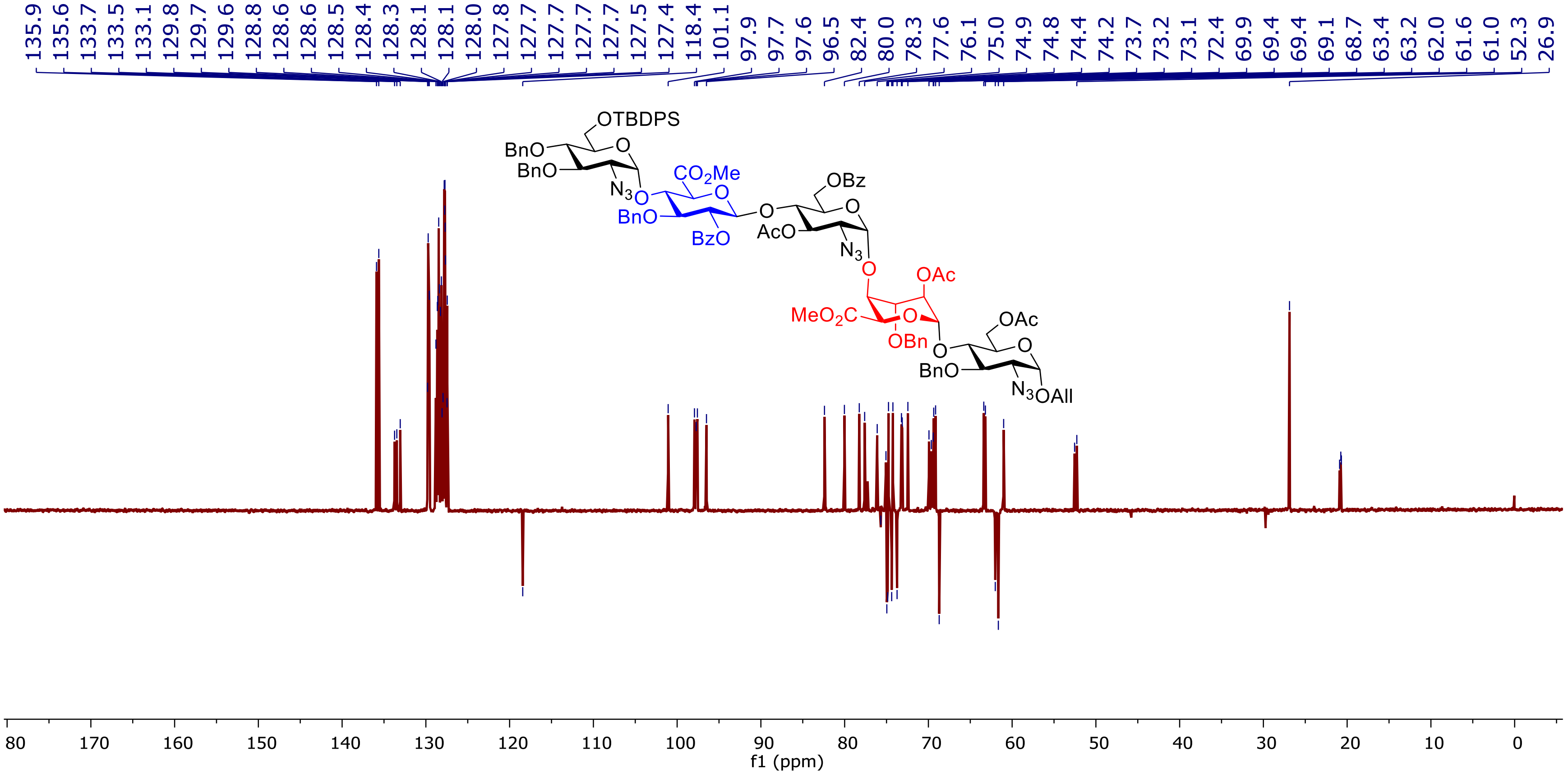

Supplementary Figure S101d. COSY NMR Spectrum (600.40, 600.40 MHz, CDCl3) of Compound 50b

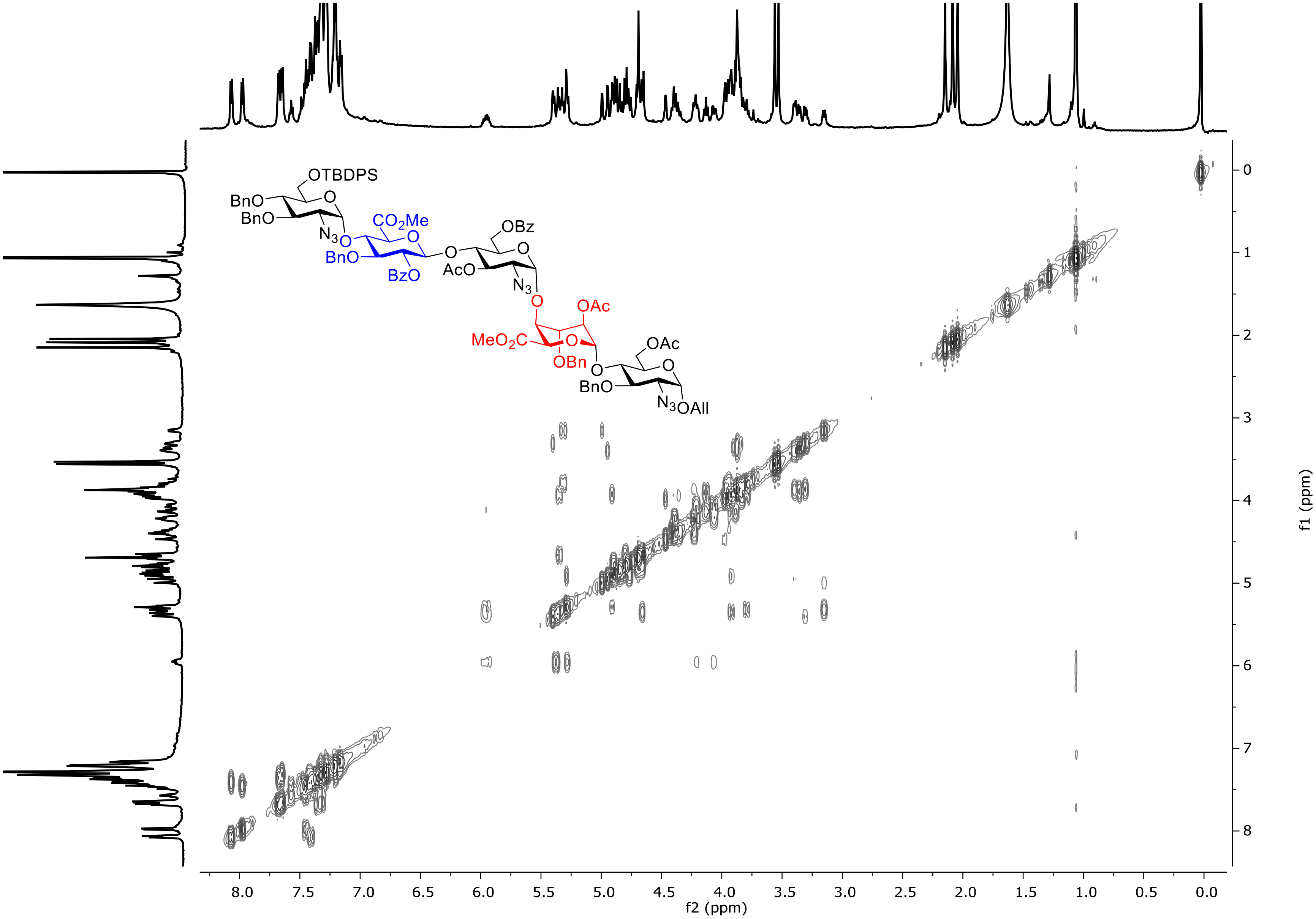

Supplementary Figure S101e. COSY NMR Spectrum (600.40, 600.40 MHz, $CDCl_3$) of Compound 50b (Sugar region expanded)

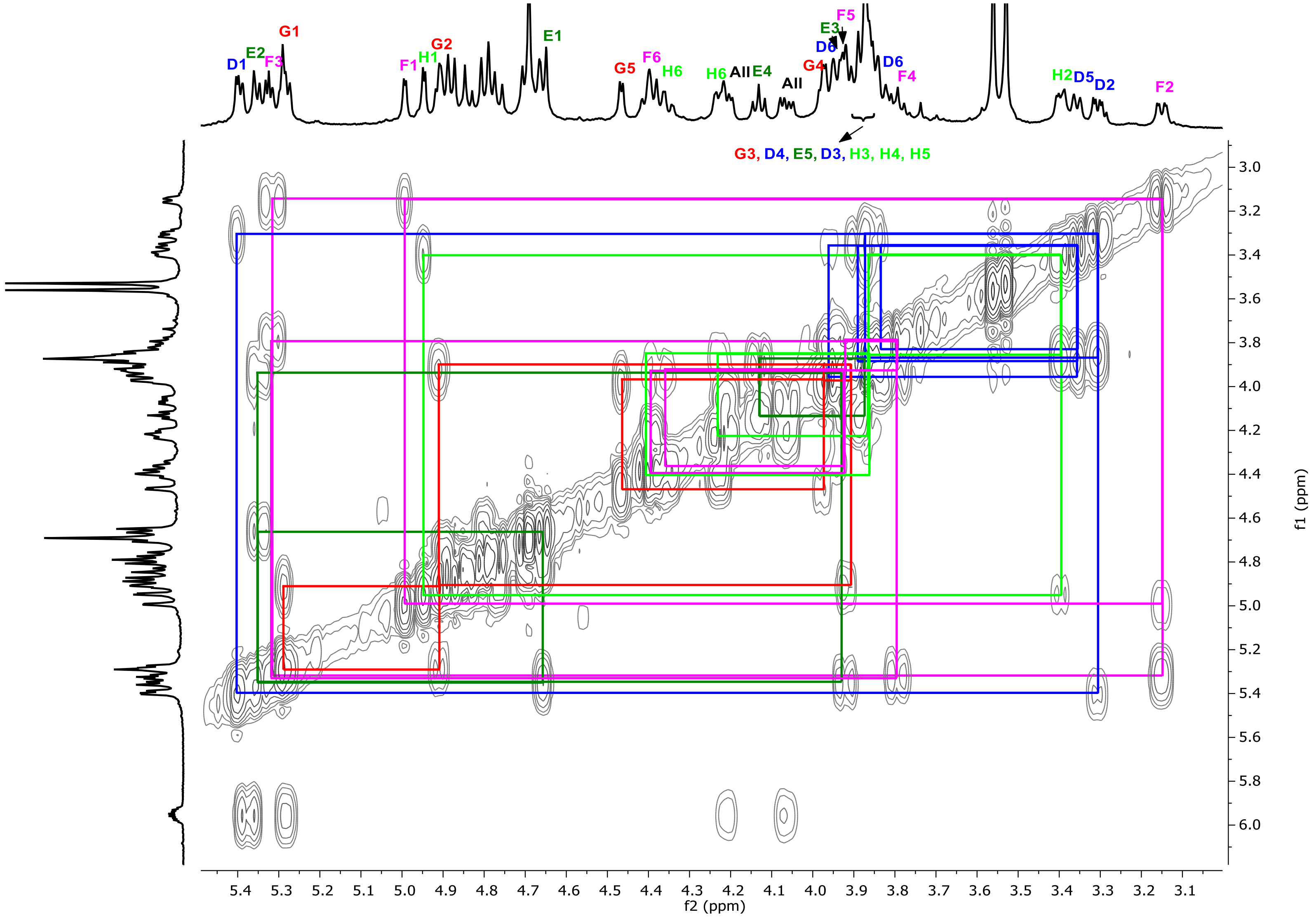

Supplementary Figure S101f. HSQC NMR Spectrum (600.40, 150.99MHz, $CDCl_3$) of Compound 50b

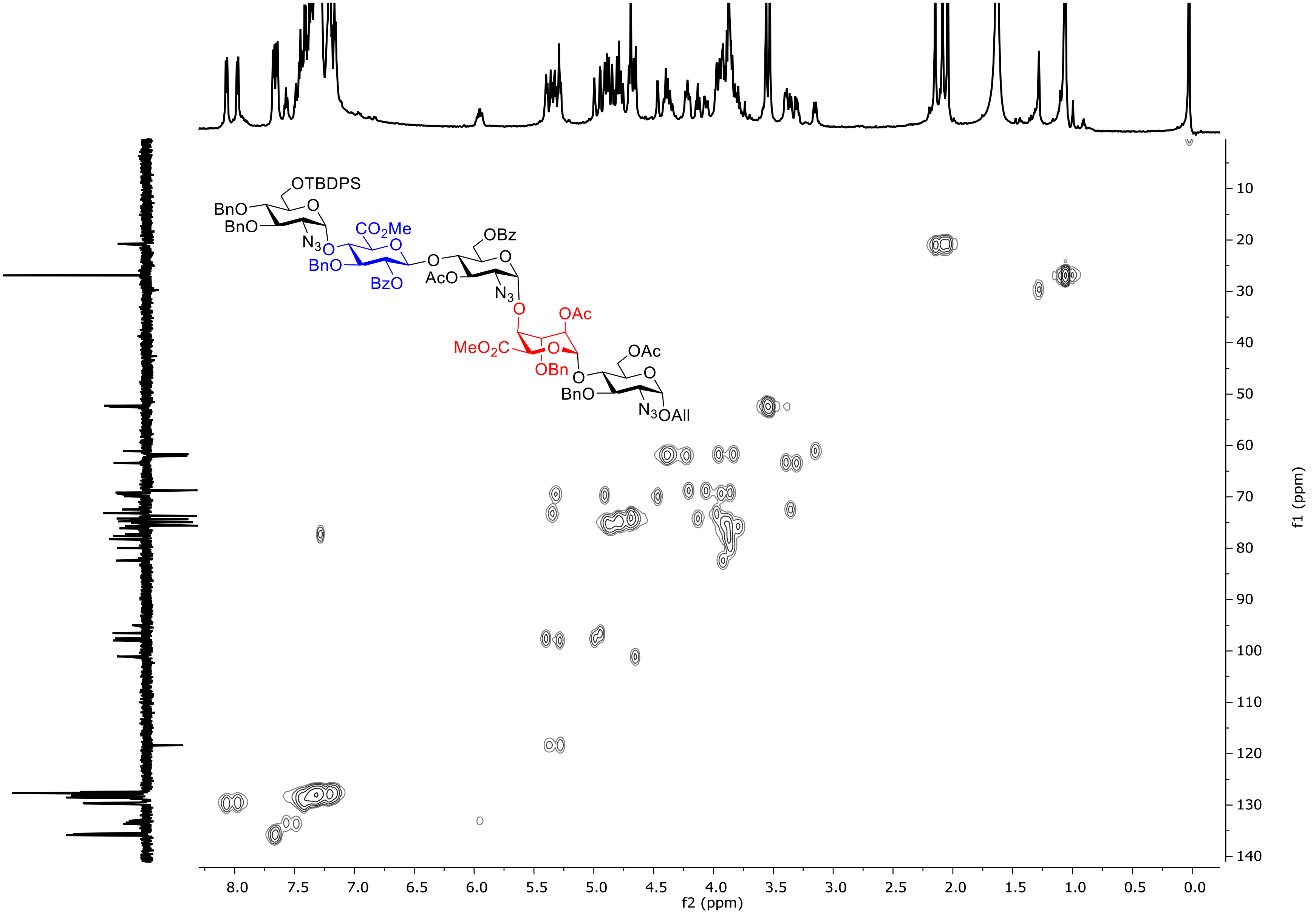

Supplementary Figure S101g. HSQC NMR Spectrum (600.40, 150.99 MHz, $CDCl_3$) of Compound 50b (Sugar region expanded)

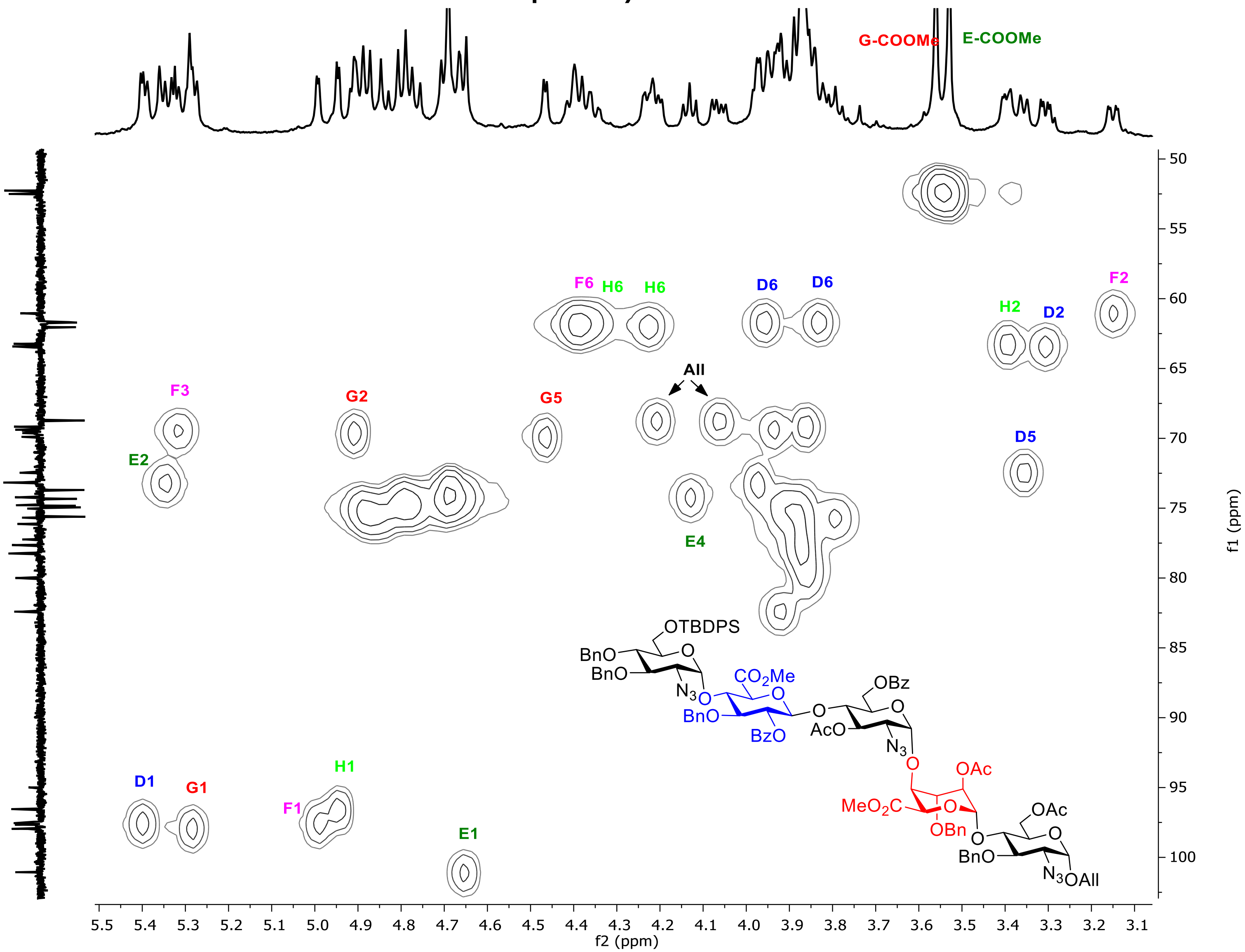

Supplementary Figure S101h. Coupled HSQC NMR Spectrum (600.40, 150.99 MHz, $CDCl_3$) of Compound 50b

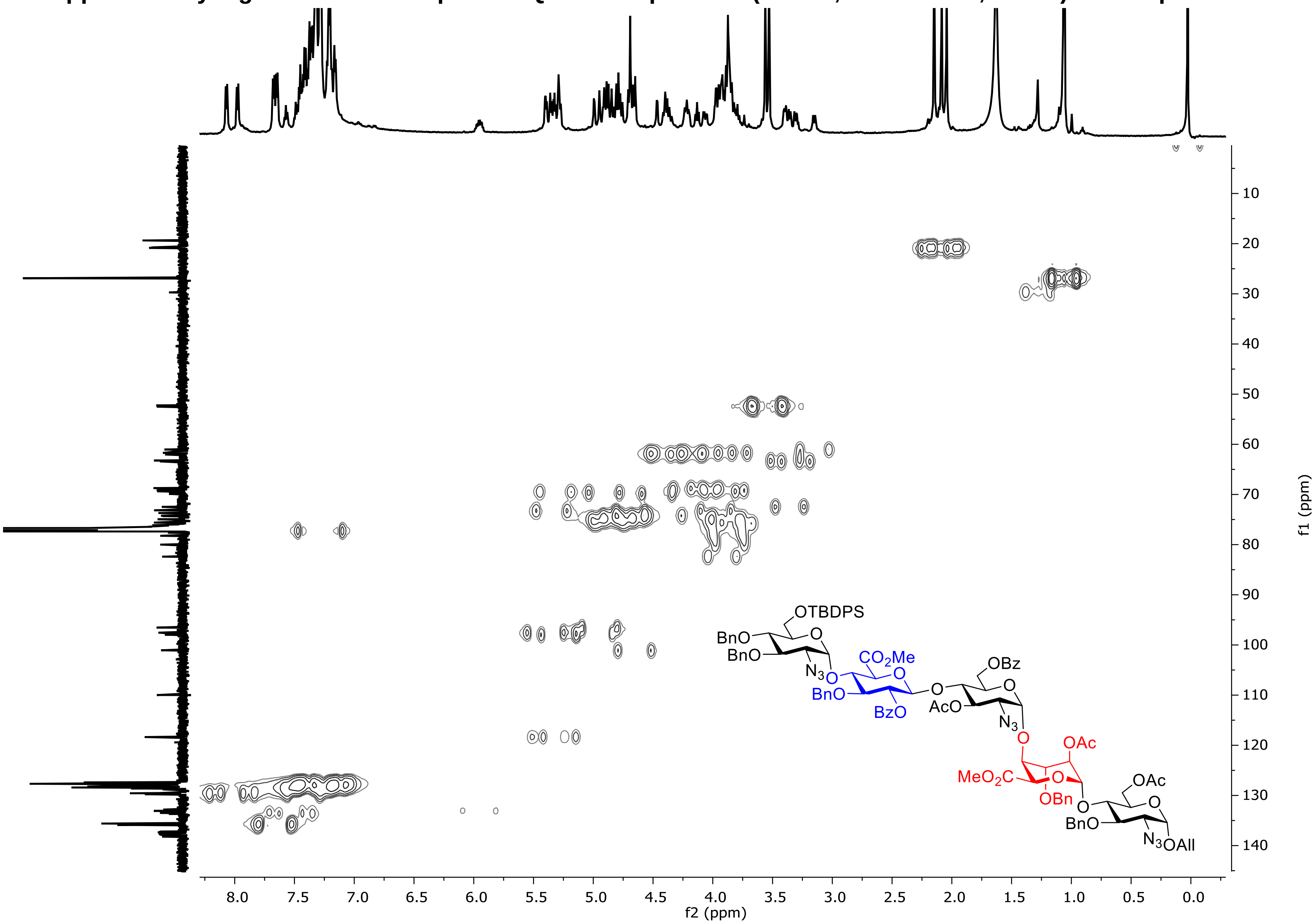

Supplementary Figure S101i. Coupled HSQC NMR Spectrum (600.40, 150.99 MHz, $CDCl_3$) of Compound 50b (Sugar region expanded)

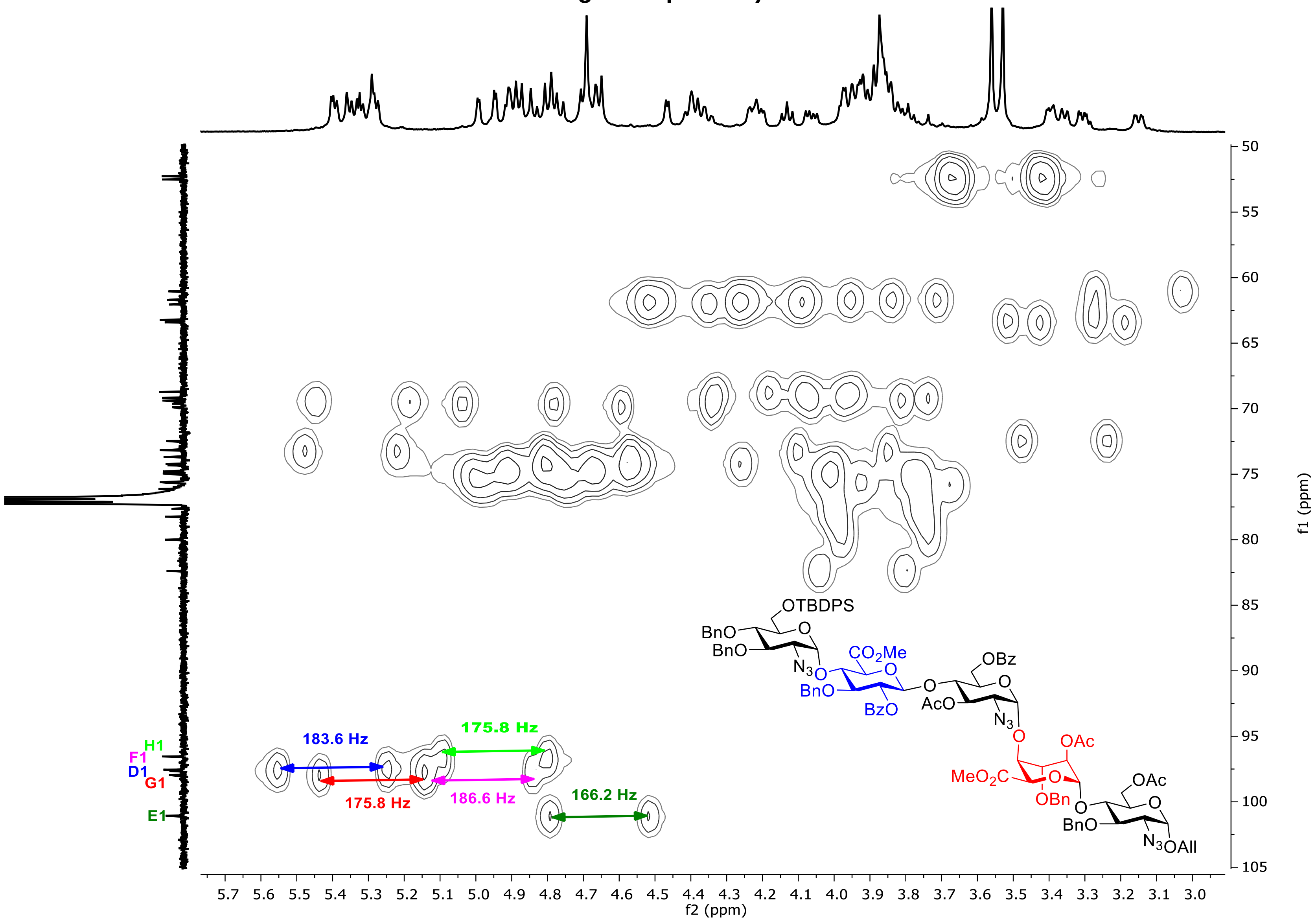

Supplementary Figure S101j. HMBC NMR Spectrum (600.40, 150.99 MHz, CDCl₃) of Compound 50b

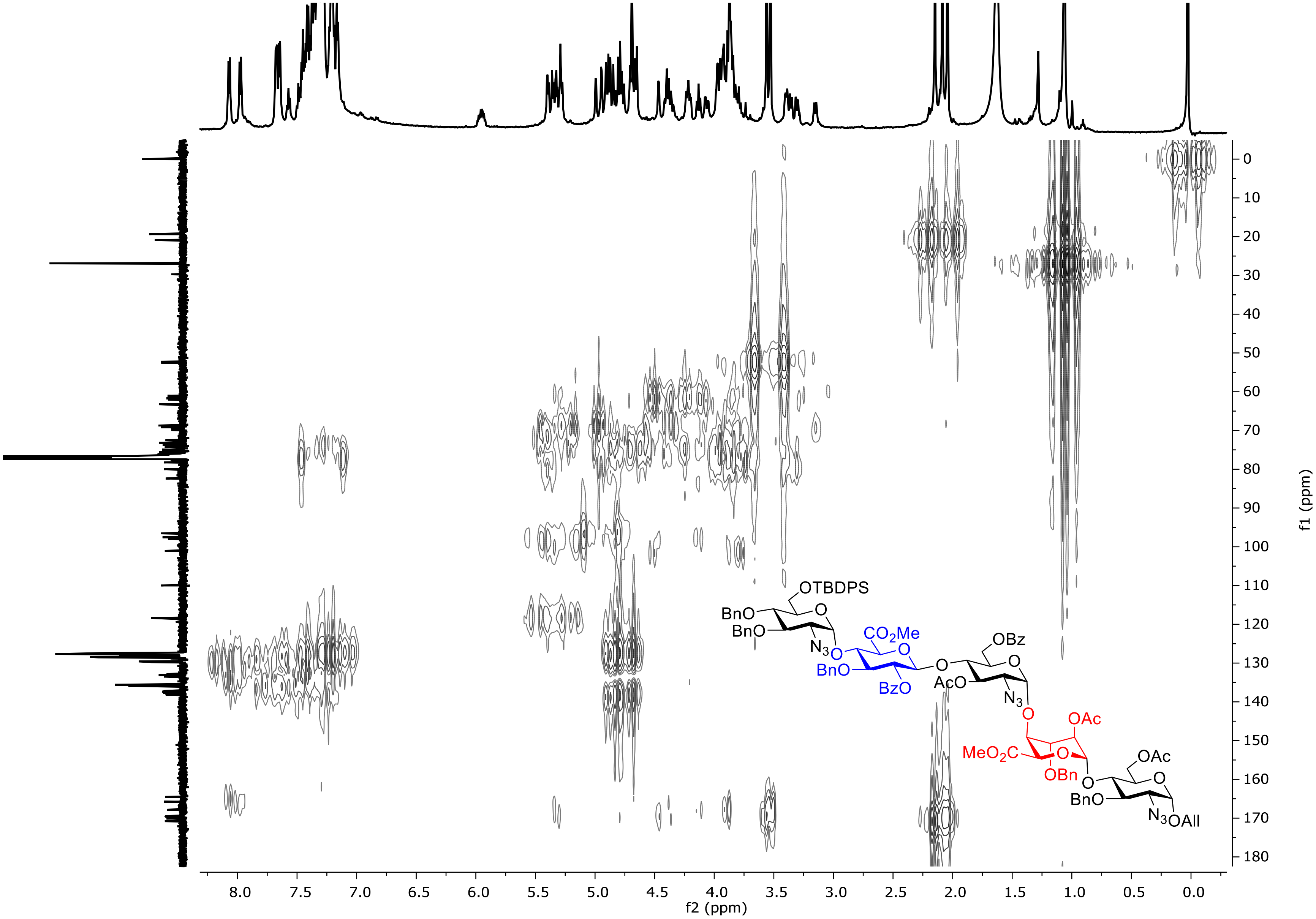

Supplementary Figure S101k. HMBC Spectrum (600.40, 150.99 MHz, $CDCl_3$) of Compound 50b (Sugar region expanded)

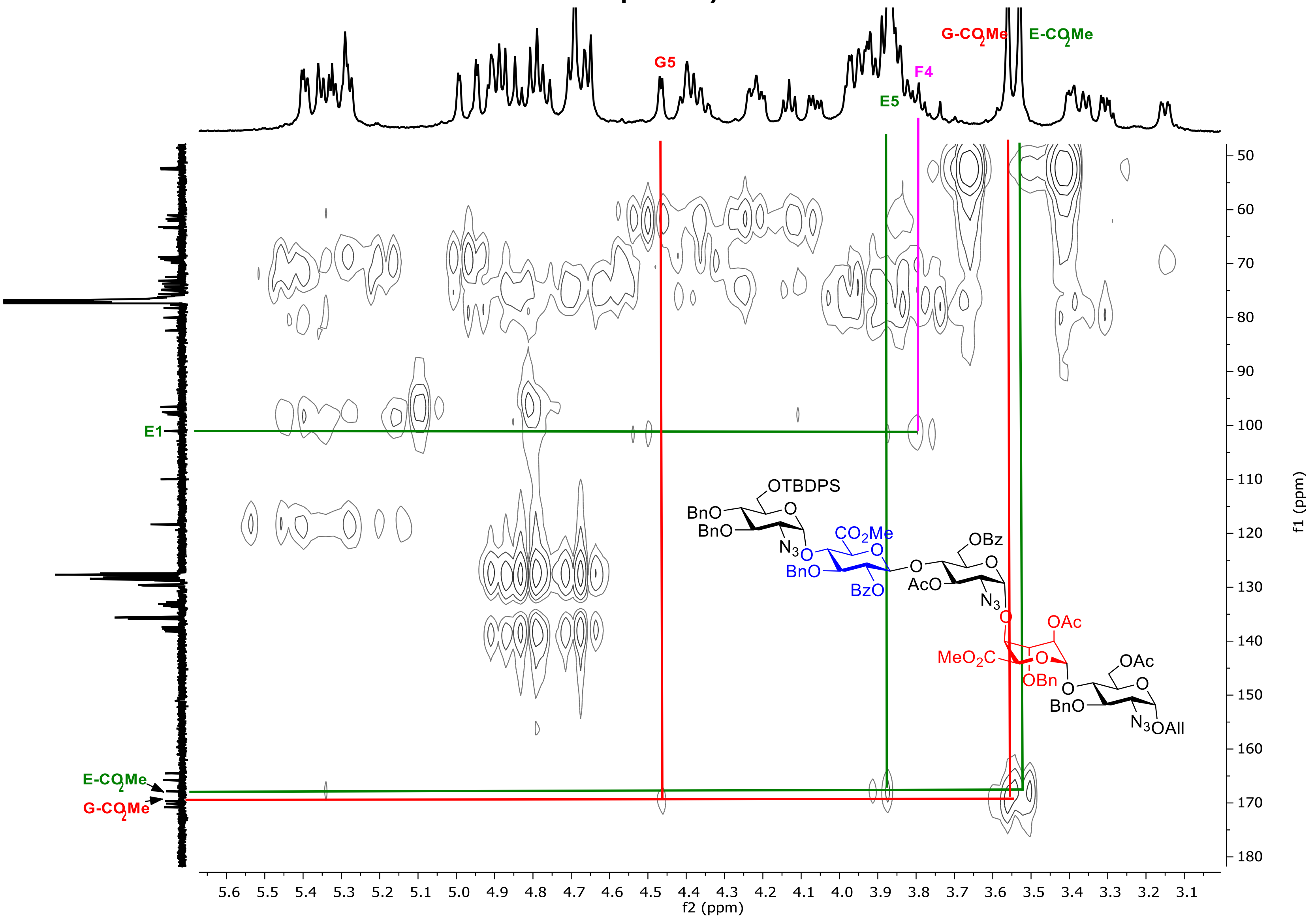

Supplementary Figure S101l. TOCSY NMR Spectrum (600.40, 600.40 MHz, $CDCl_3$) of Compound 50b

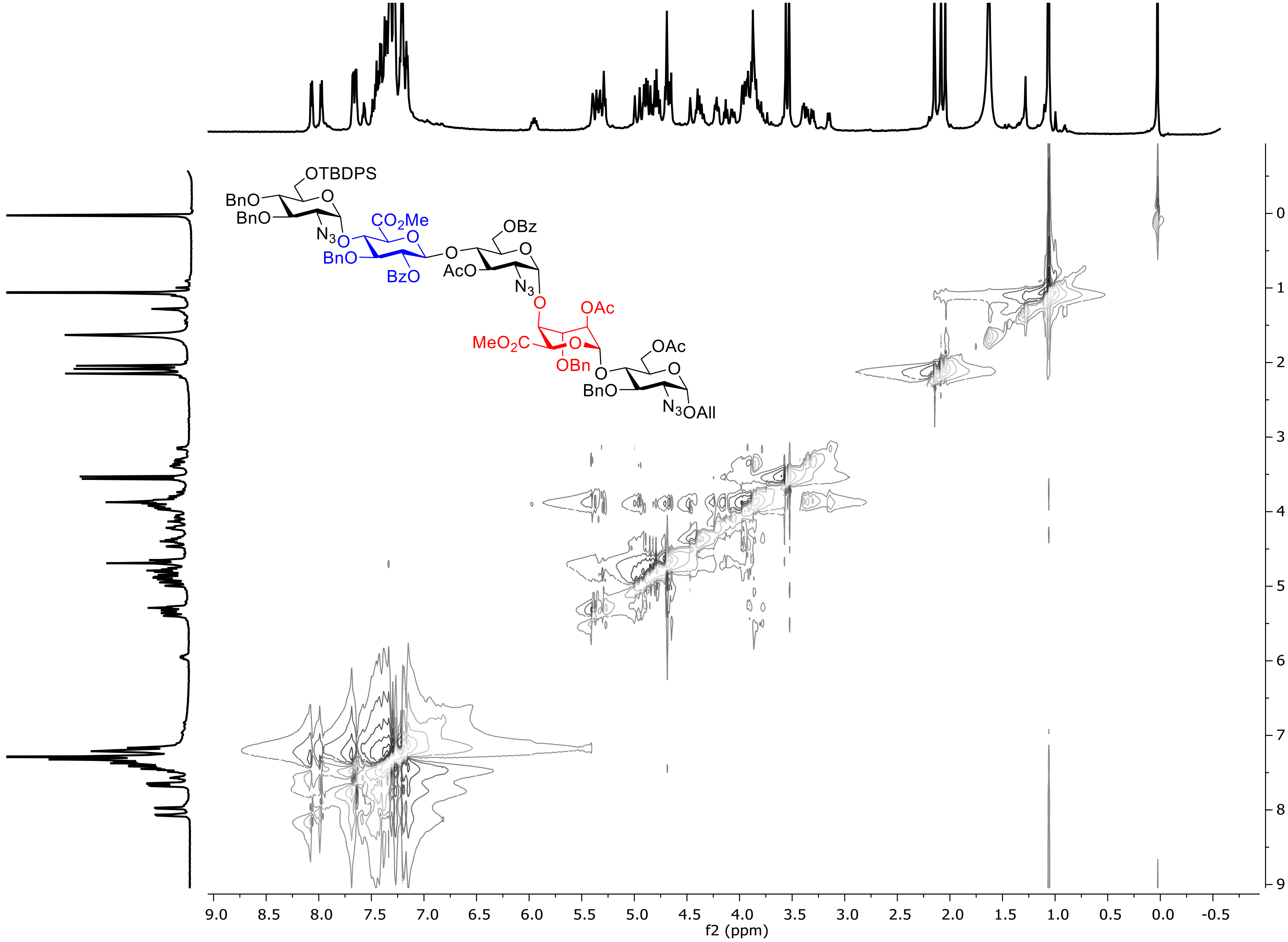

Supplementary Figure S101m. TOCSY NMR Spectrum (600.40, 600.40 MHz, $CDCl_3$) of Compound 50b (Sugar region expanded)

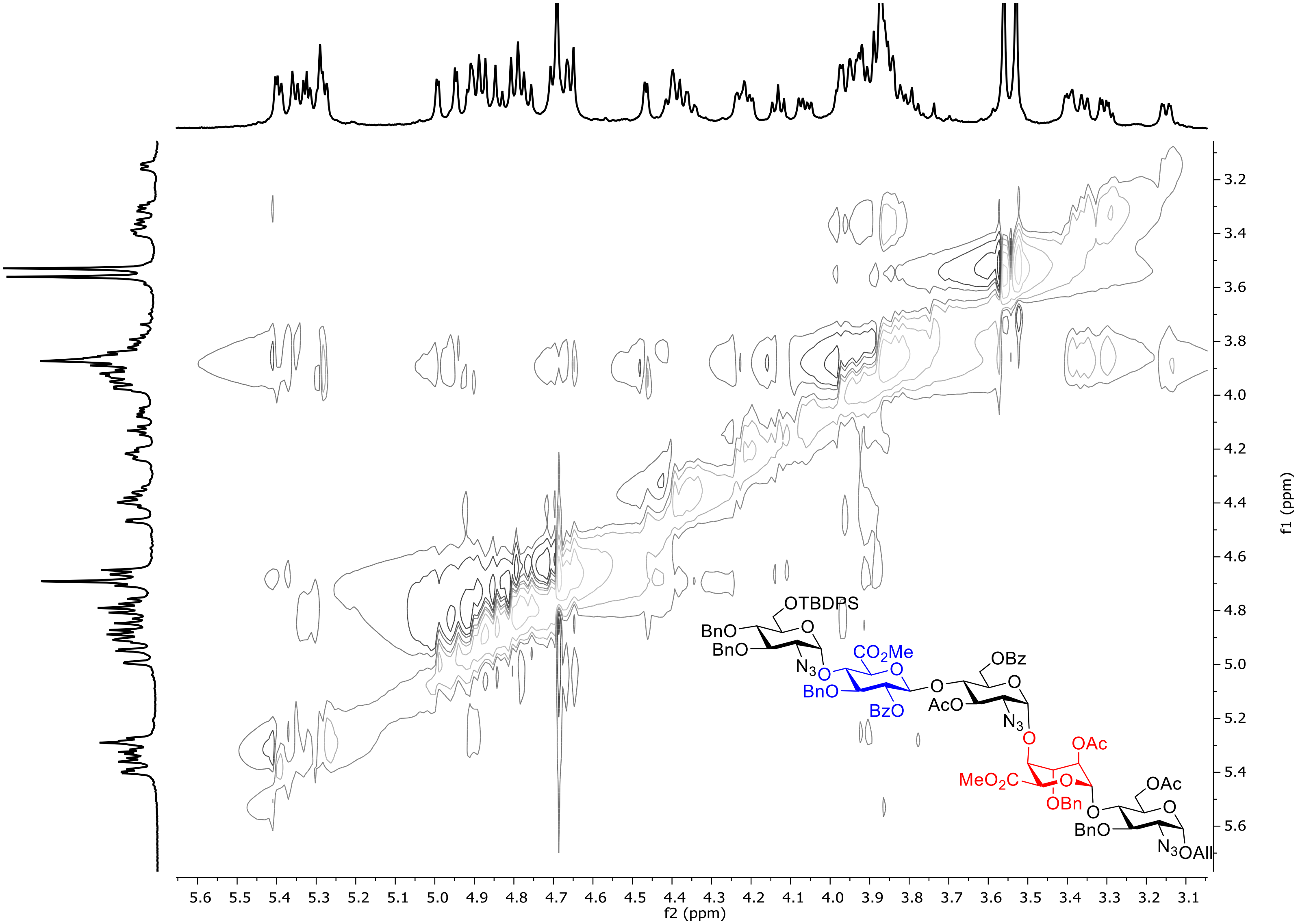

Supplementary Figure S101n. HSQC-TOCSY NMR Spectrum (600.40, 150.99 MHz, $CDCl_3$) of Compound 50b

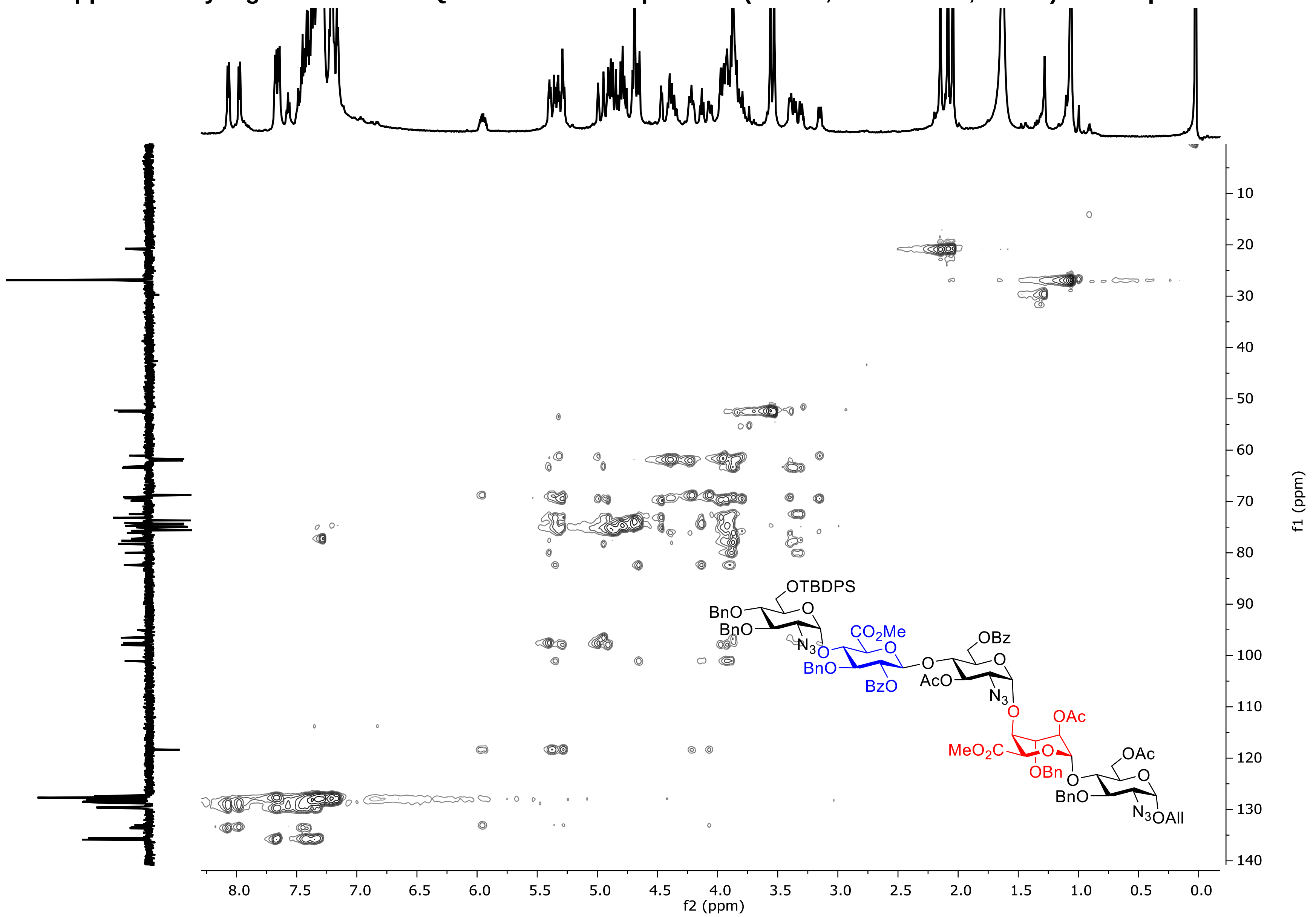

Supplementary Figure S101o. HSQC-TOCSY NMR Spectrum (600.40, 150.99 MHz, $CDCl_3$) of Compound 50b (Sugar region expanded)

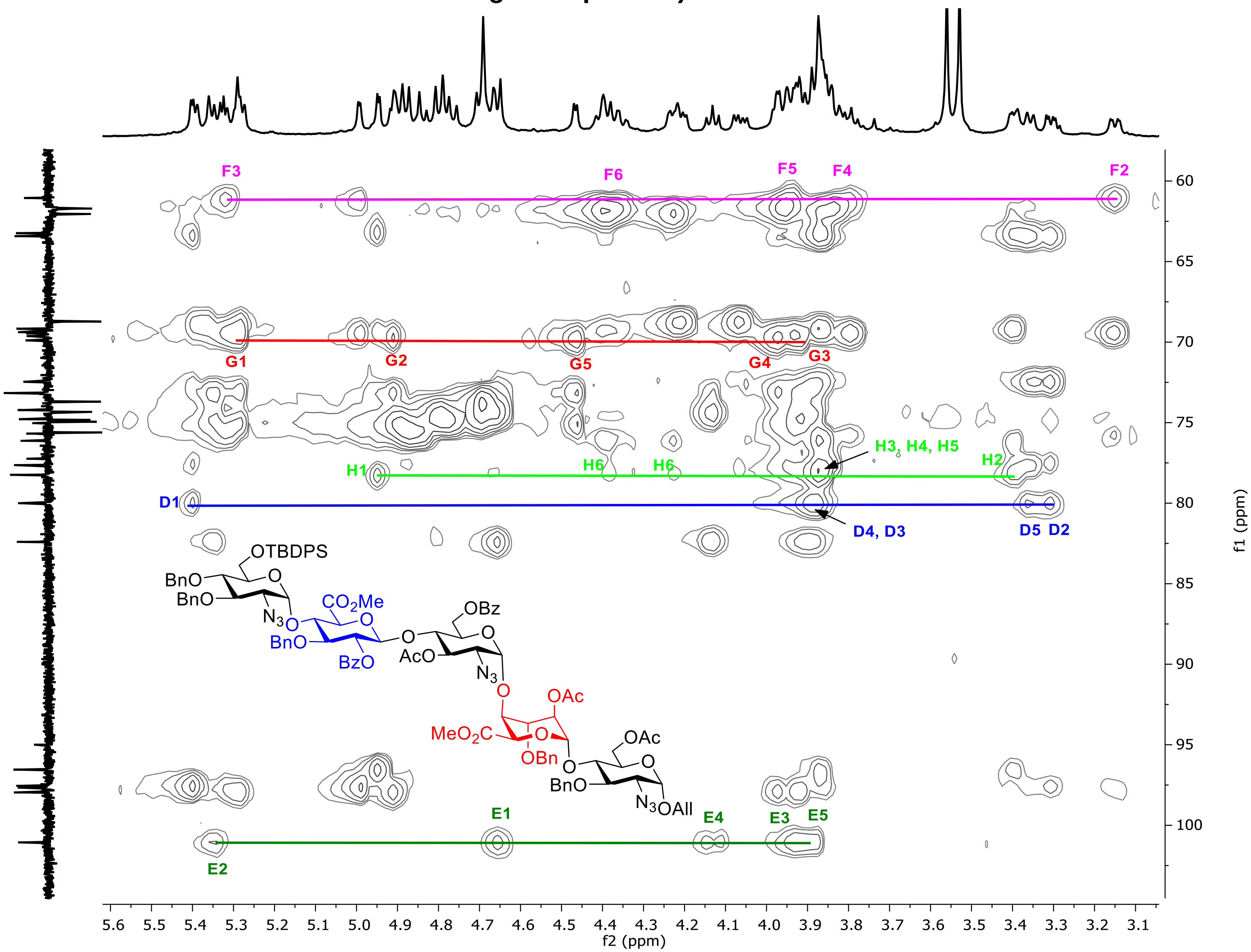

Supplementary Figure S102a. ^{1}H NMR Spectrum (600.40 MHz, $CDCl_3$) of Compound 51

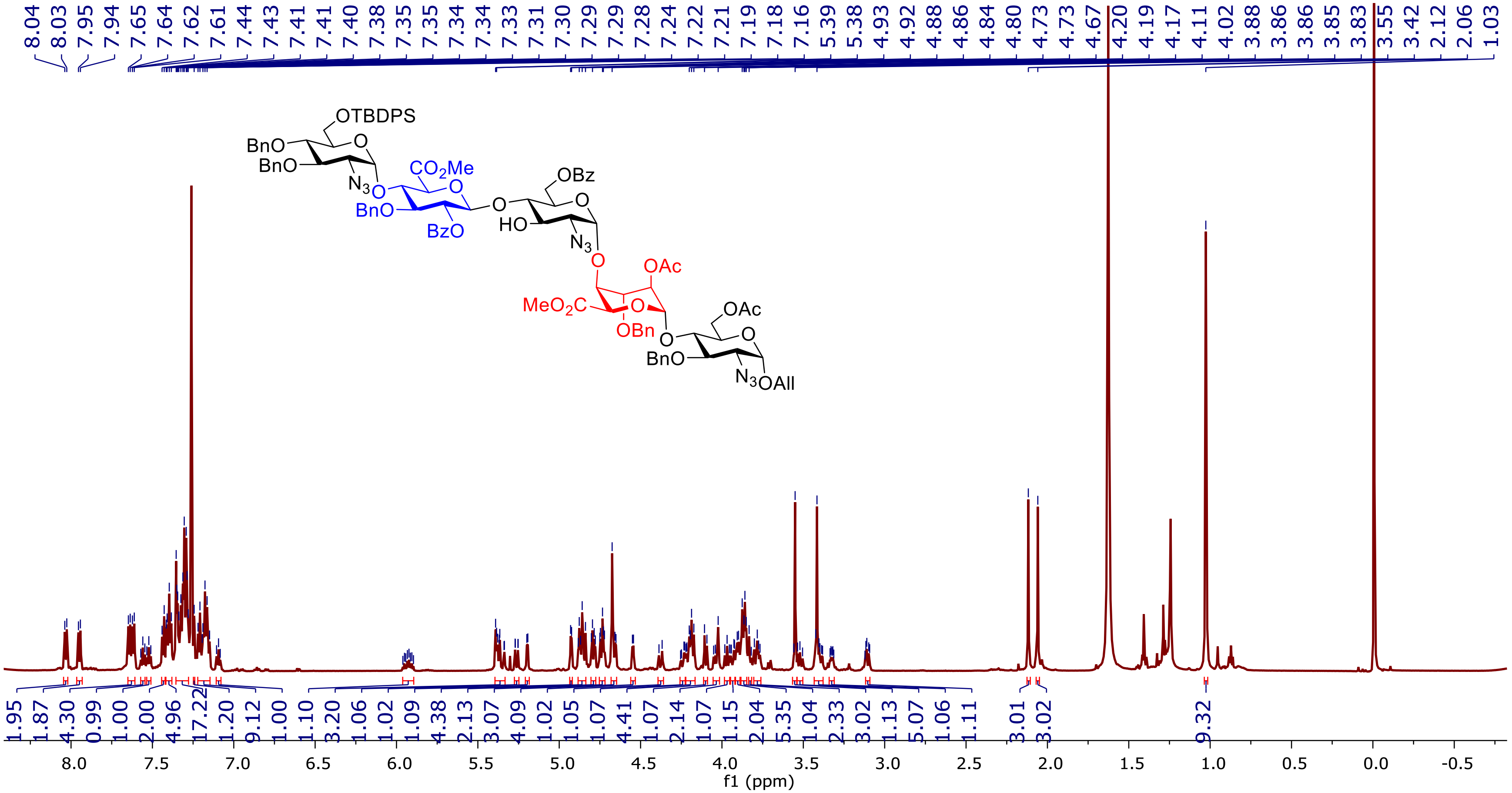

Supplementary Figure S102b. ^{13}C NMR Spectrum (150.97 MHz, $CDCl_3$) of Compound 51

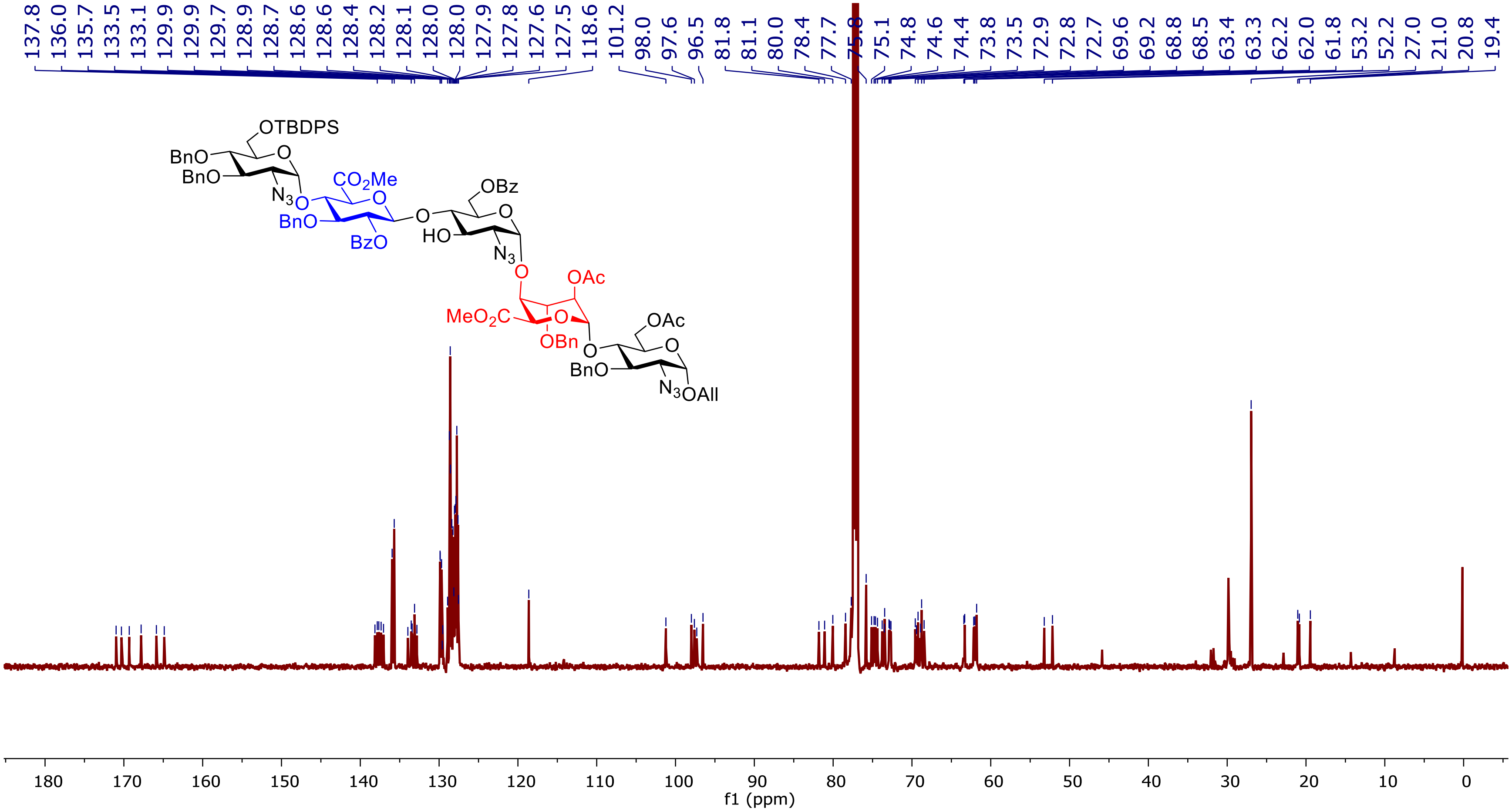

Supplementary Figure S102c. DEPT NMR Spectrum (150.97 MHz, $CDCl_3$) of Compound 51

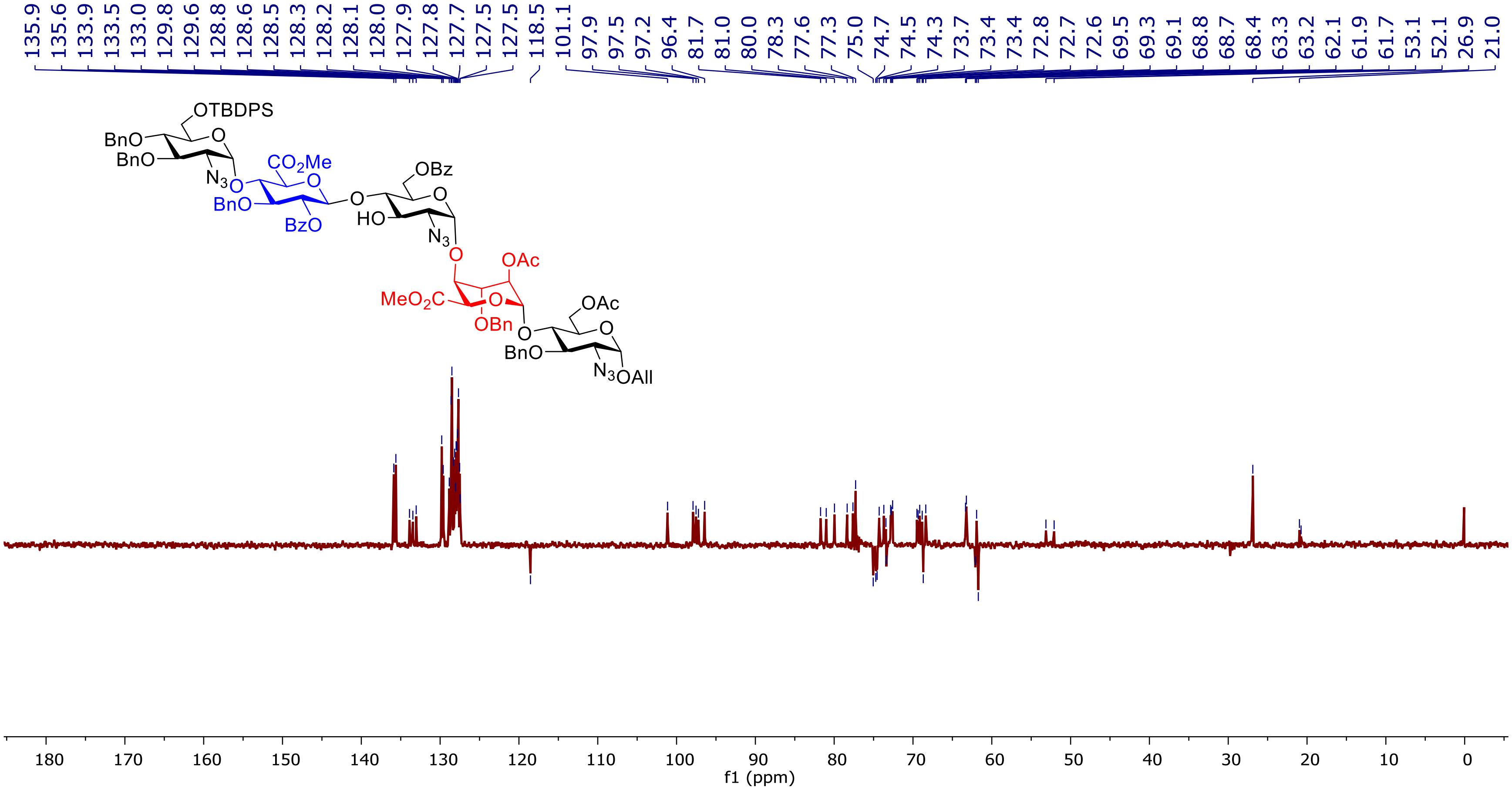

Supplementary Figure S102d. COSY NMR Spectrum (600.40, 600.40 MHz, $CDCl_3$) of Compound 51

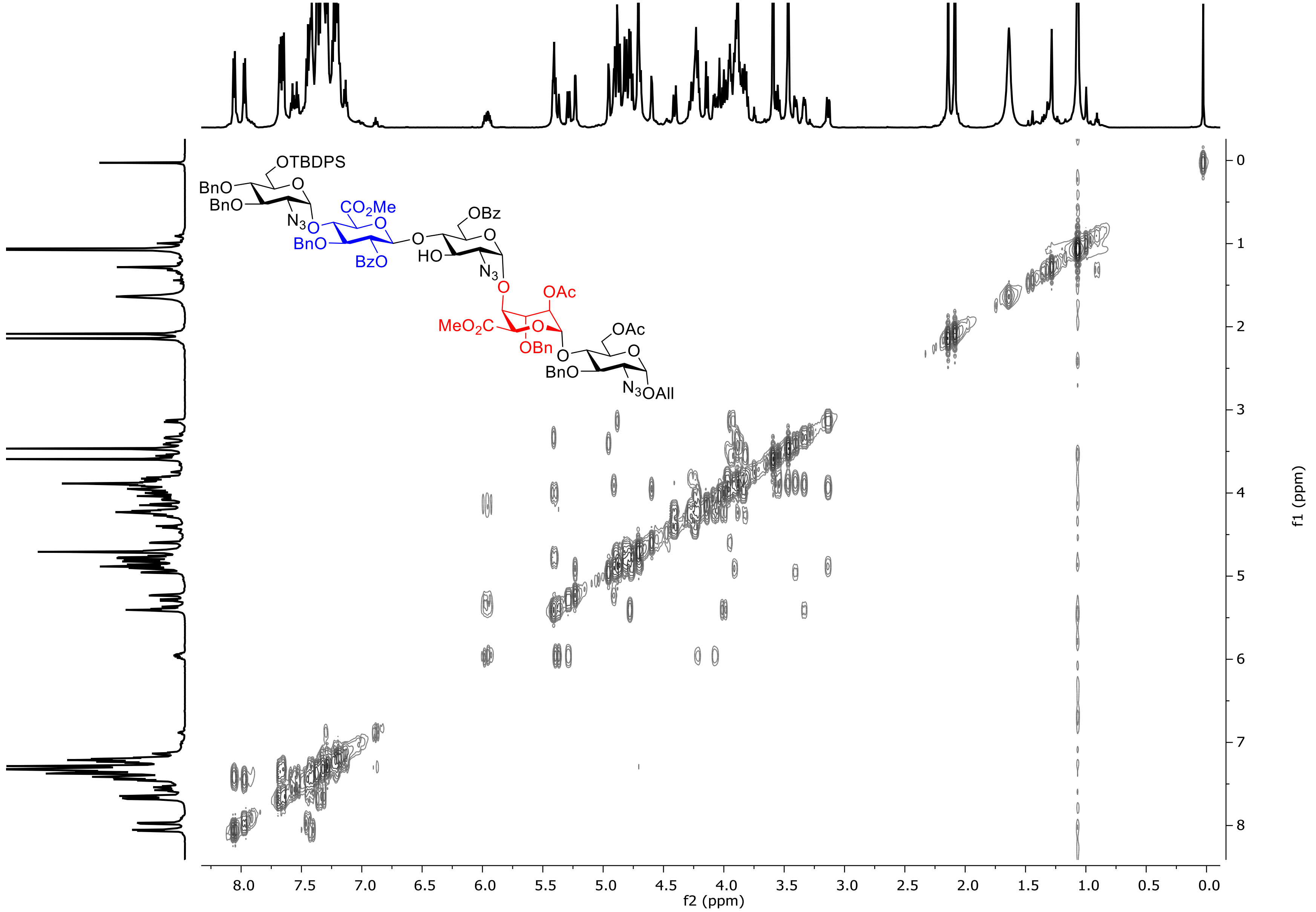

Supplementary Figure S102e. COSY NMR Spectrum (600.40, 600.40 MHz, $CDCl_3$) of Compound 51 (Sugar region expanded)

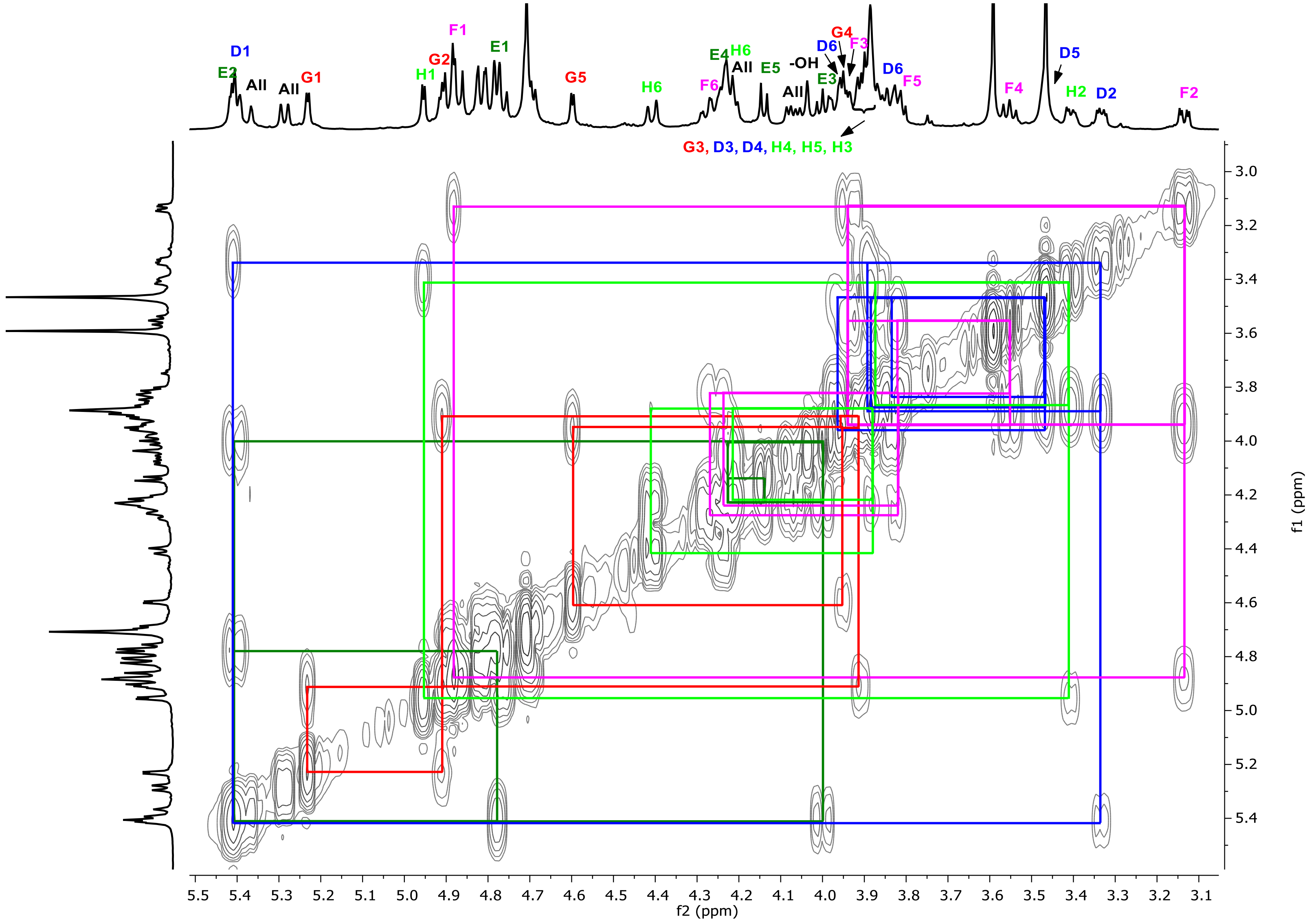

Supplementary Figure S102f. HSQC NMR Spectrum (600.40, 150.99MHz, $CDCl_3$) of Compound 51

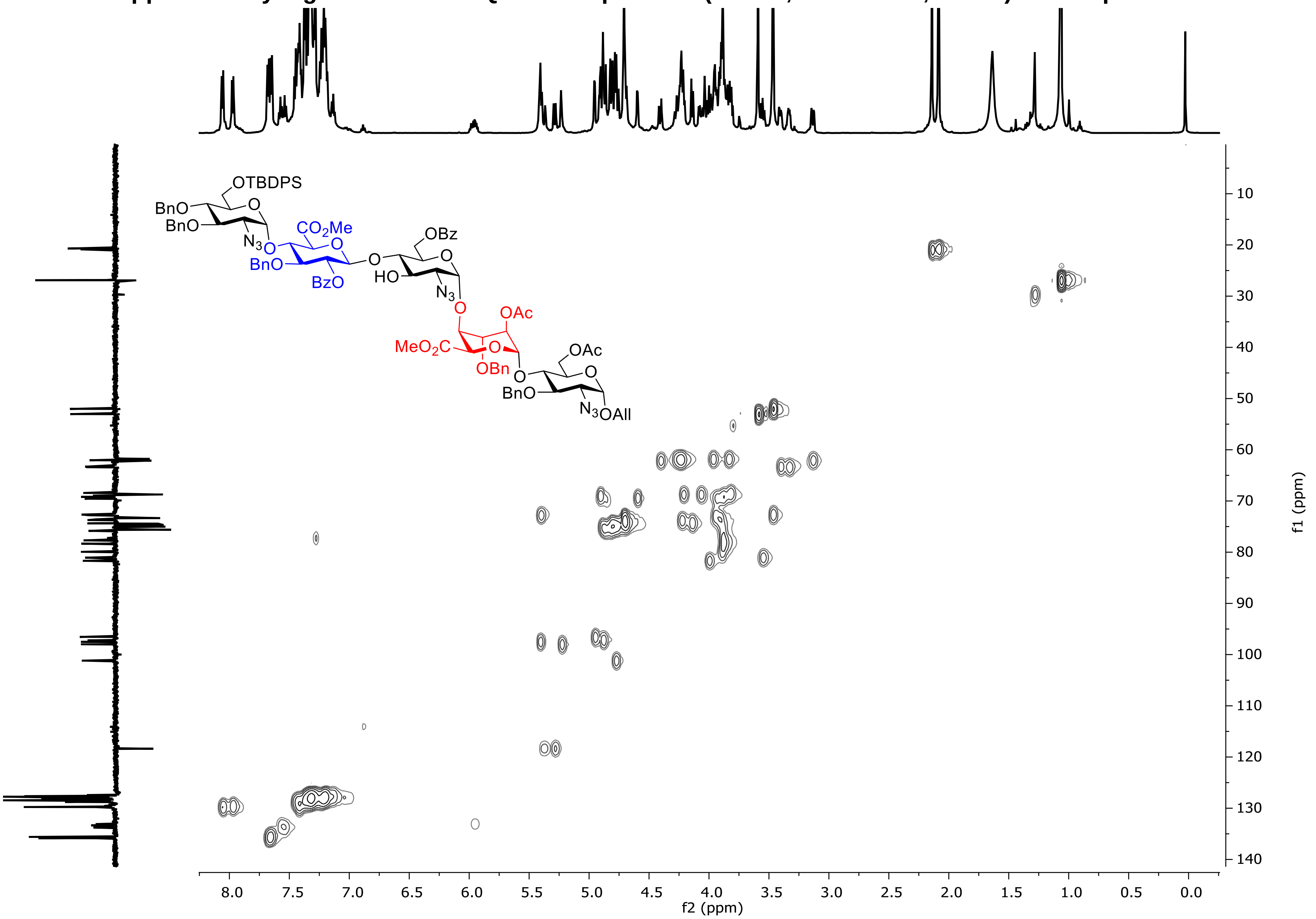

Supplementary Figure S102g. HSQC NMR Spectrum (600.40, 150.99 MHz, CDCl3) of Compound 51 (Sugar region expanded)

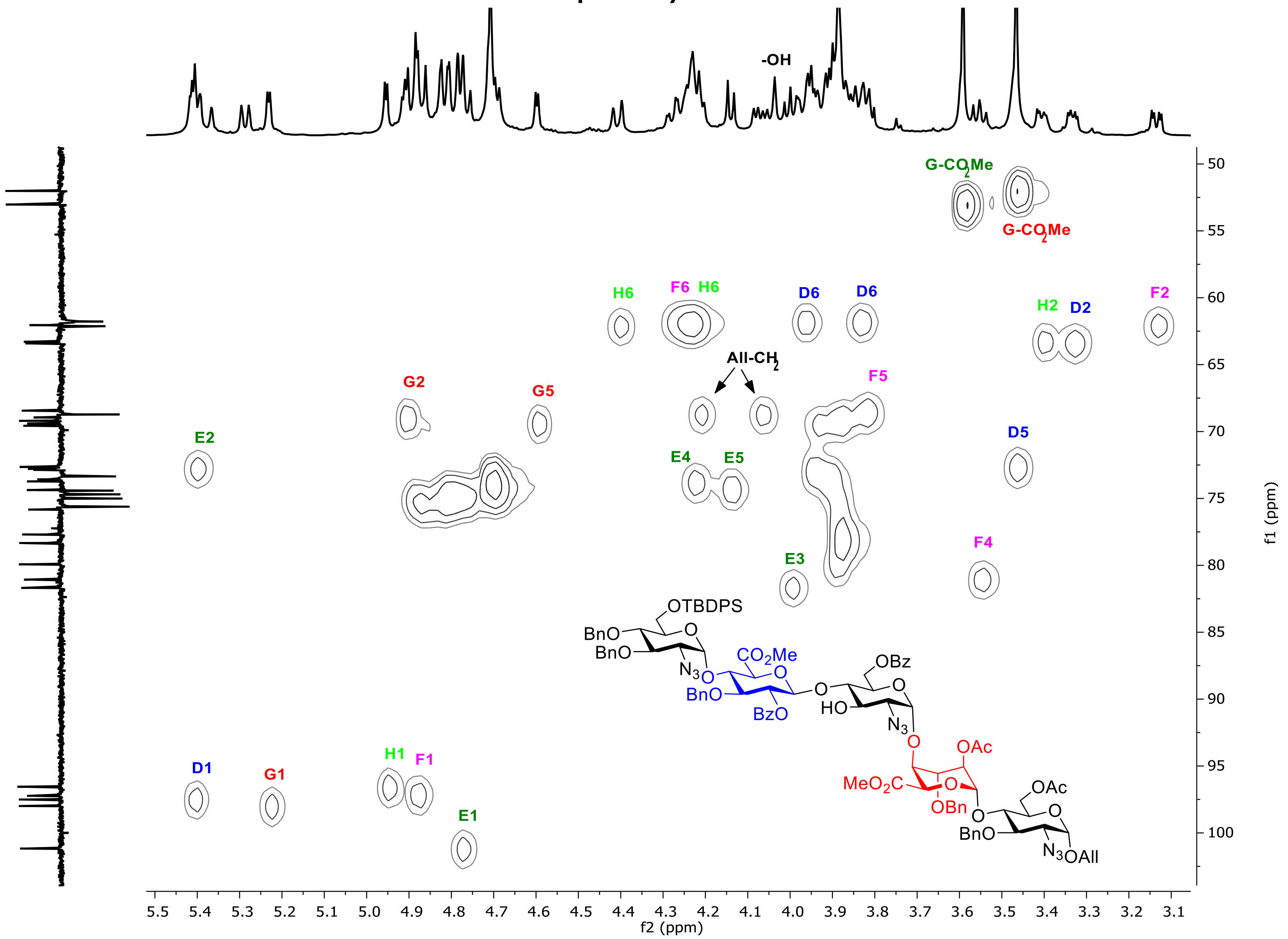

Supplementary Figure S102h. Coupled HSQC NMR Spectrum (600.40, 150.99 MHz, $CDCl_3$) of Compound 51

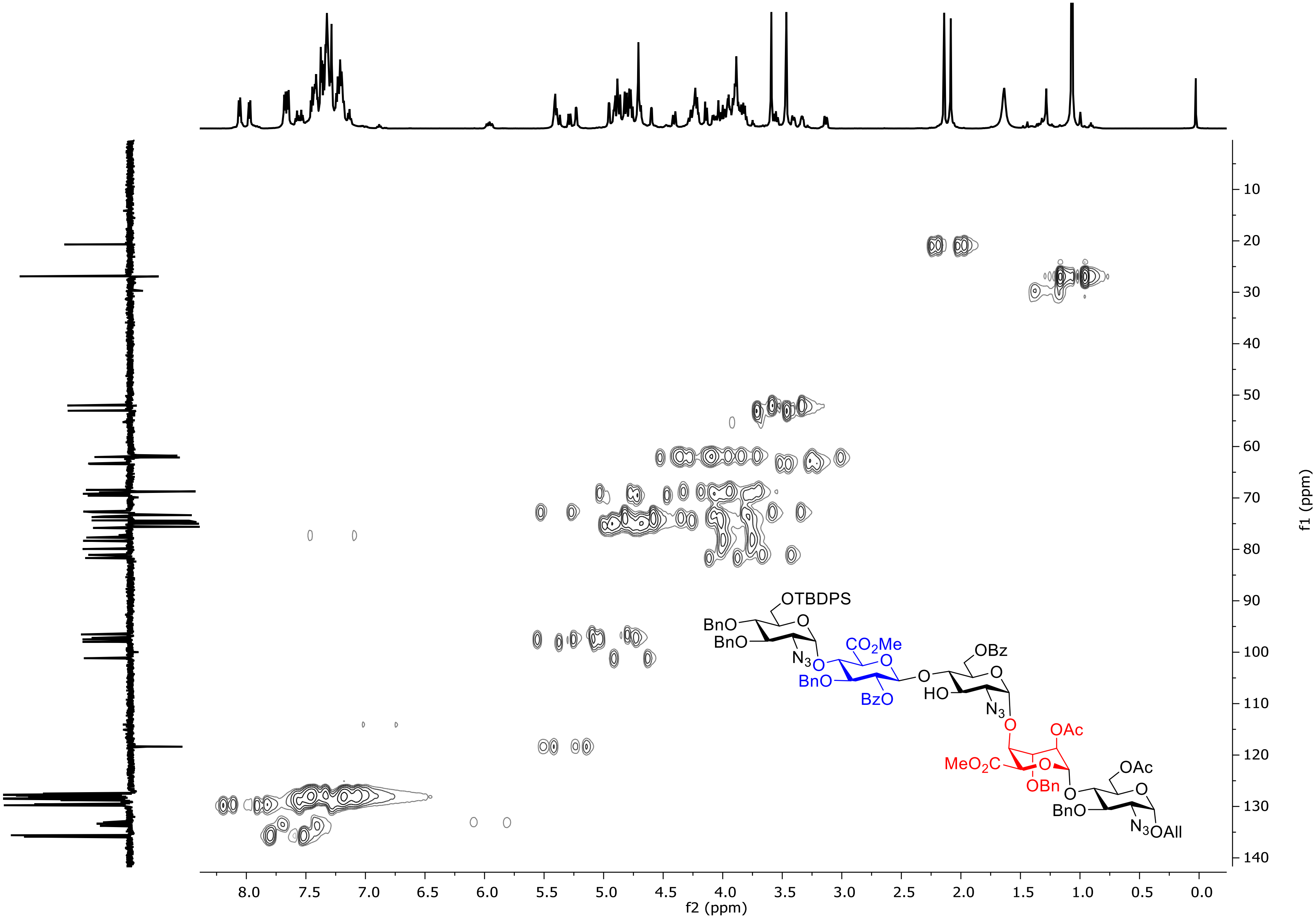

Supplementary Figure S102i. Coupled HSQC NMR Spectrum (600.40, 150.99 MHz, $CDCl_3$) of Compound 51 (Sugar region expanded)

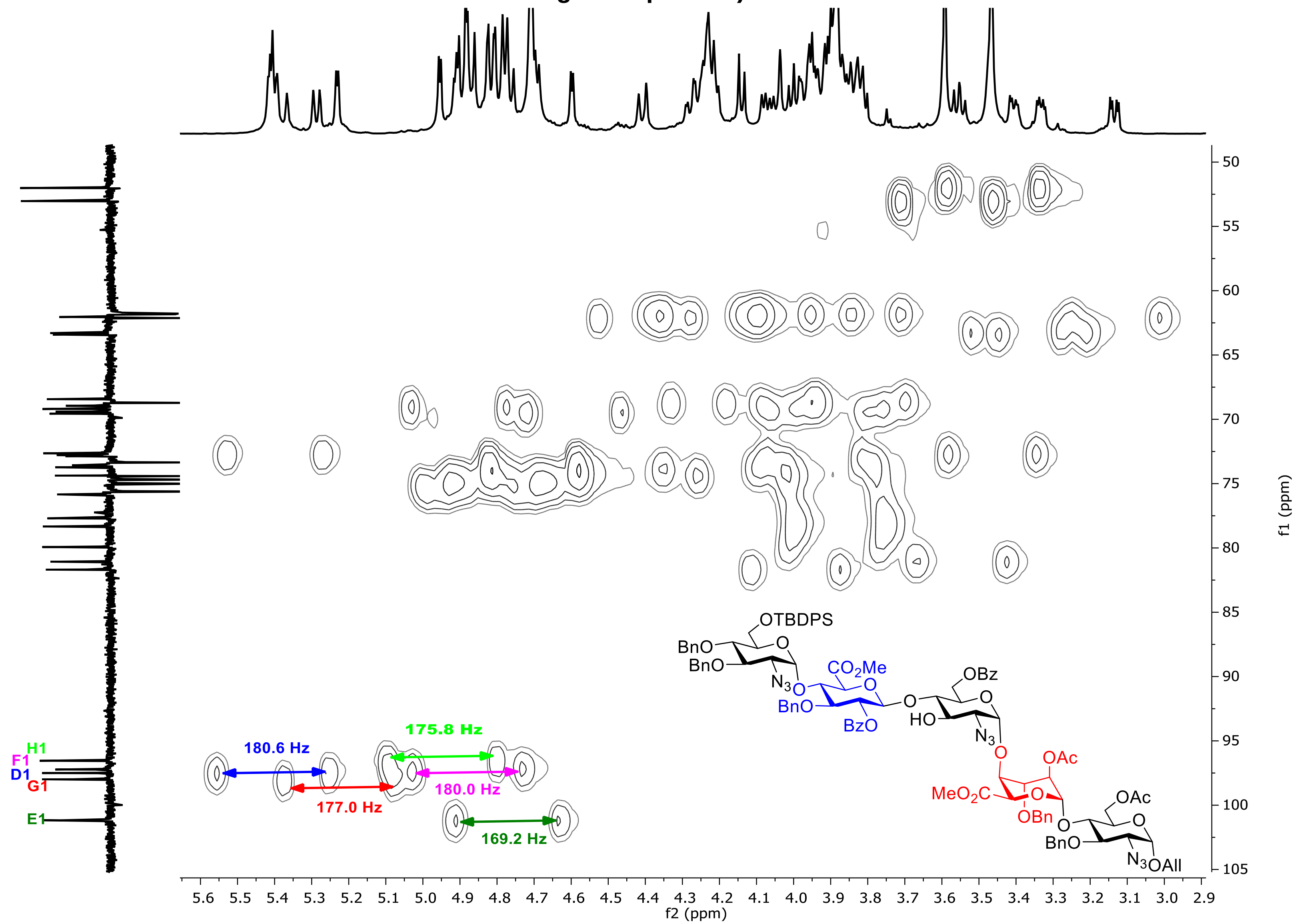

Supplementary Figure S102j. HMBC NMR Spectrum (600.40, 150.99 MHz, CDCl₃) of Compound 51

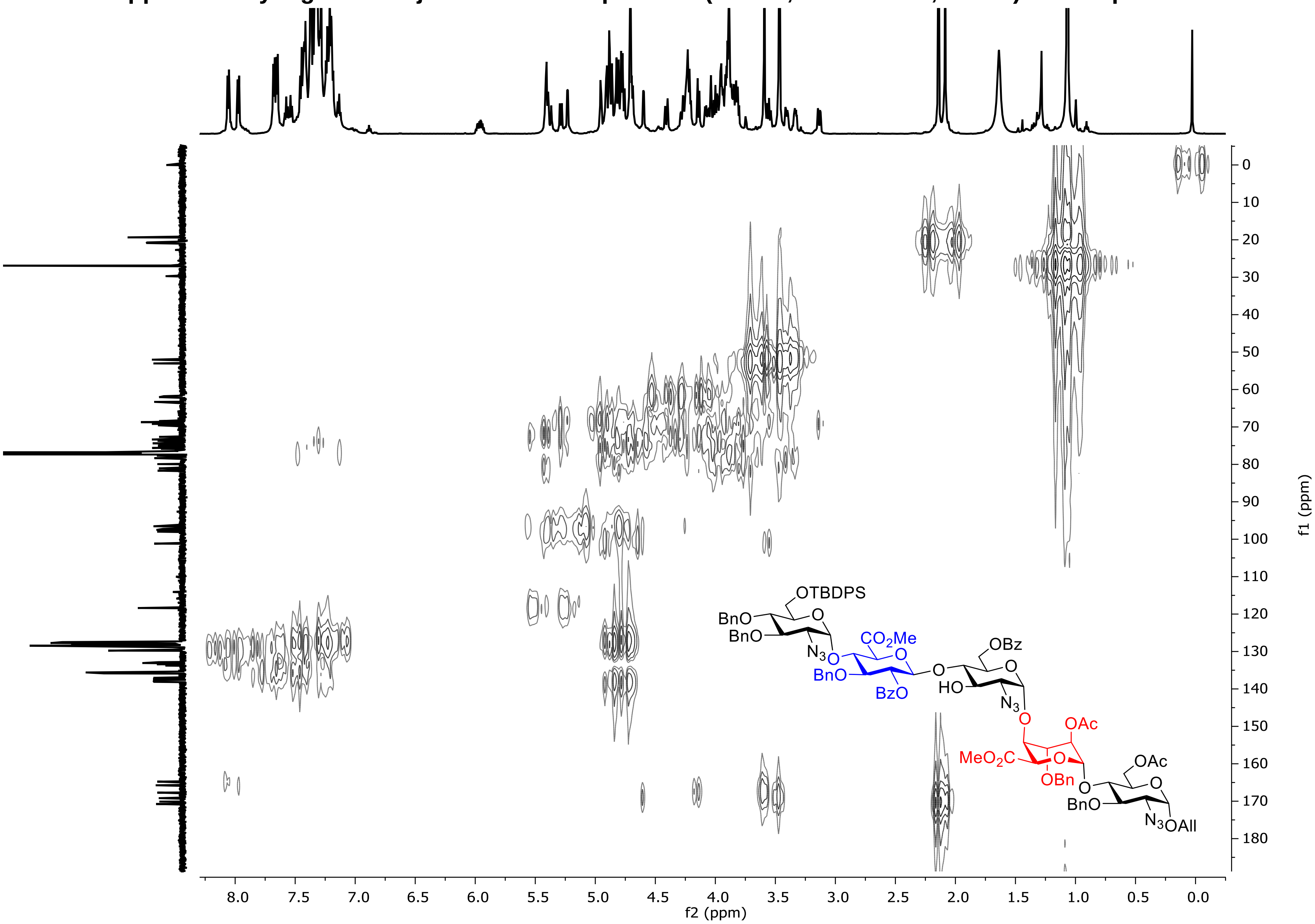

Supplementary Figure S102k. HMBC Spectrum (600.40, 150.99 MHz, $CDCl_3$) of Compound 51 (Sugar region expanded)

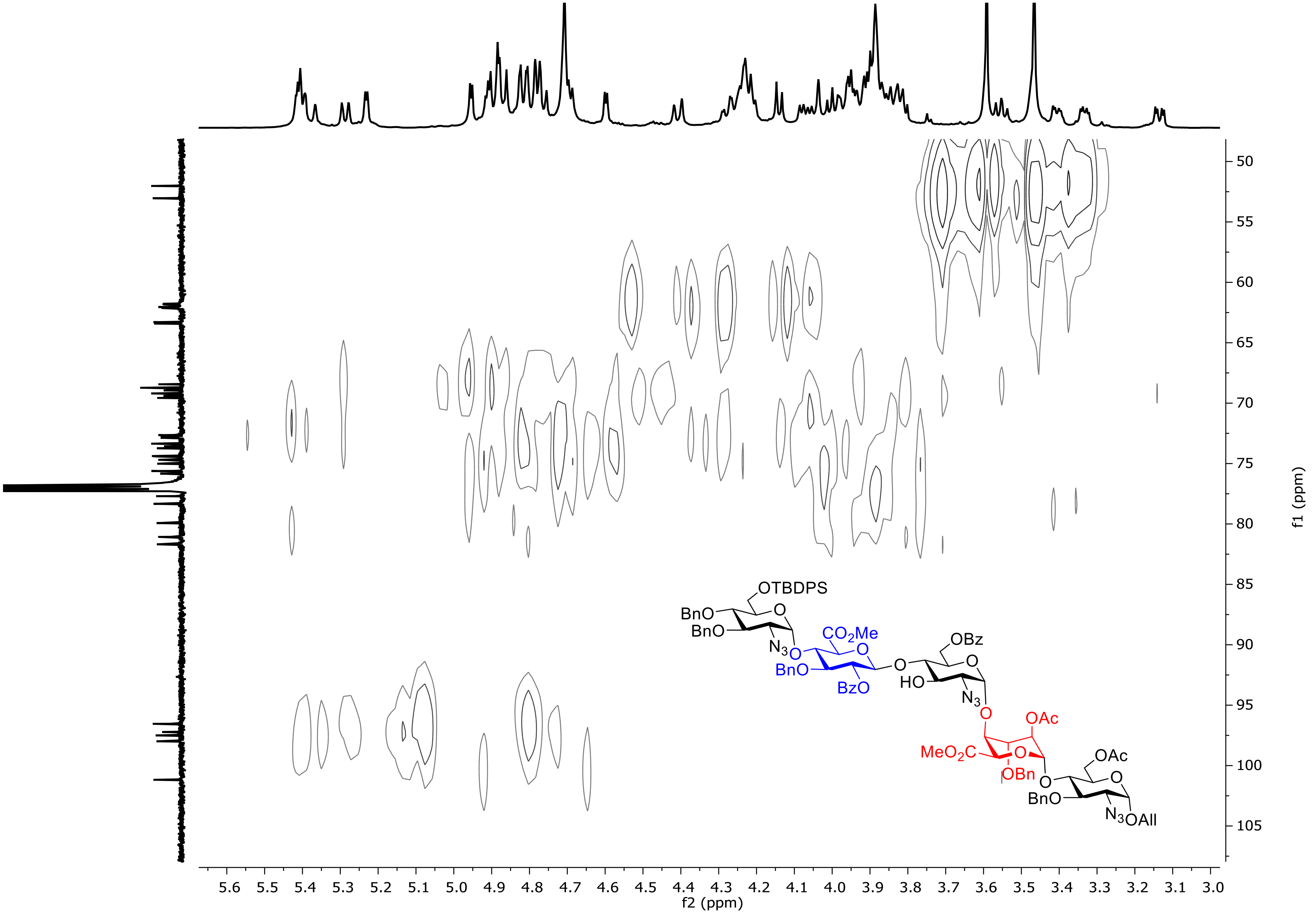

Supplementary Figure S102l. HMBC NMR Spectrum (600.40, 150.99 MHz, $CDCl_3$) of Compound 51 (Carbonyl region expanded)

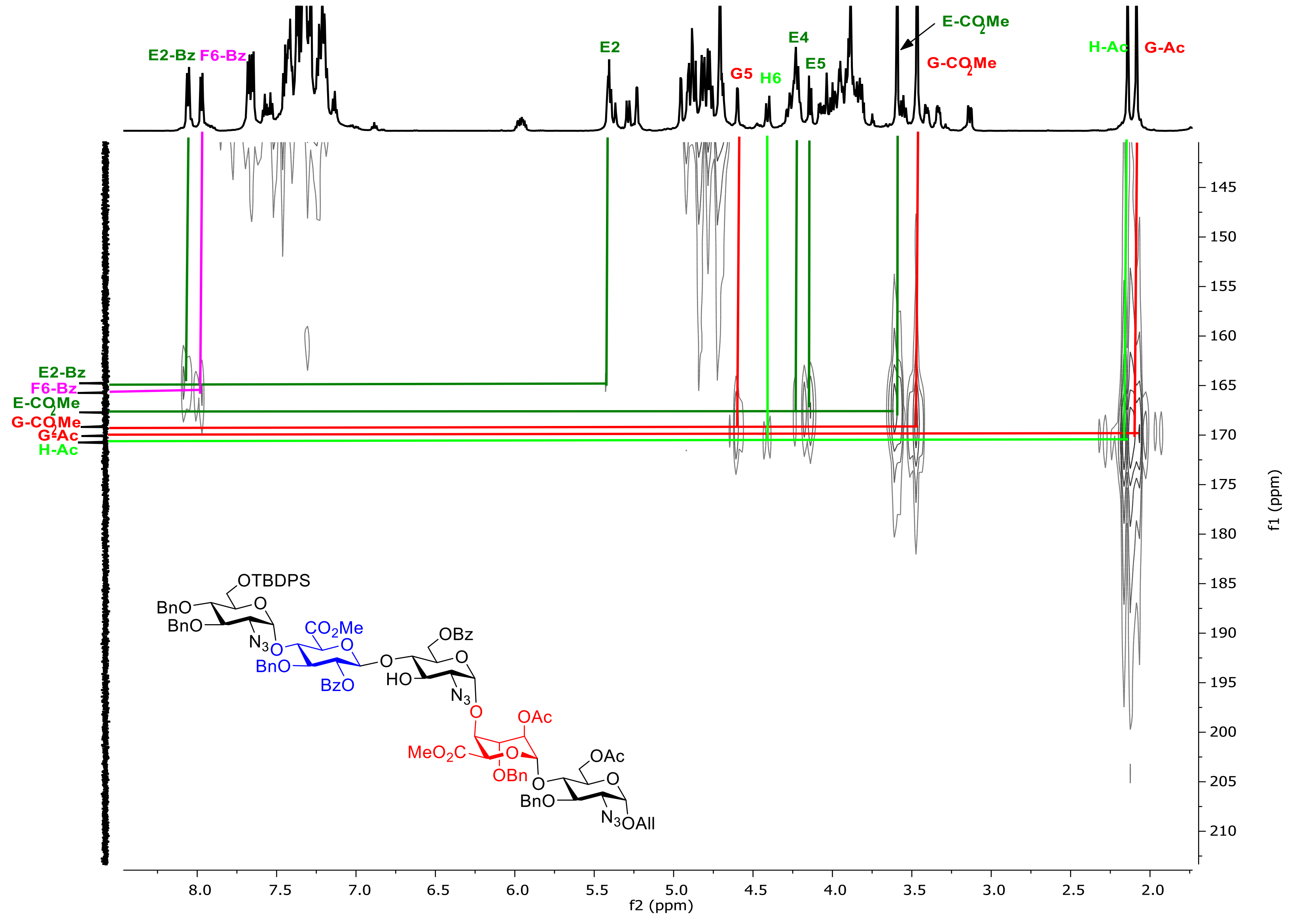

Supplementary Figure S102m. TOCSY NMR Spectrum (600.40, 600.40 MHz, CDCl3) of Compound 51

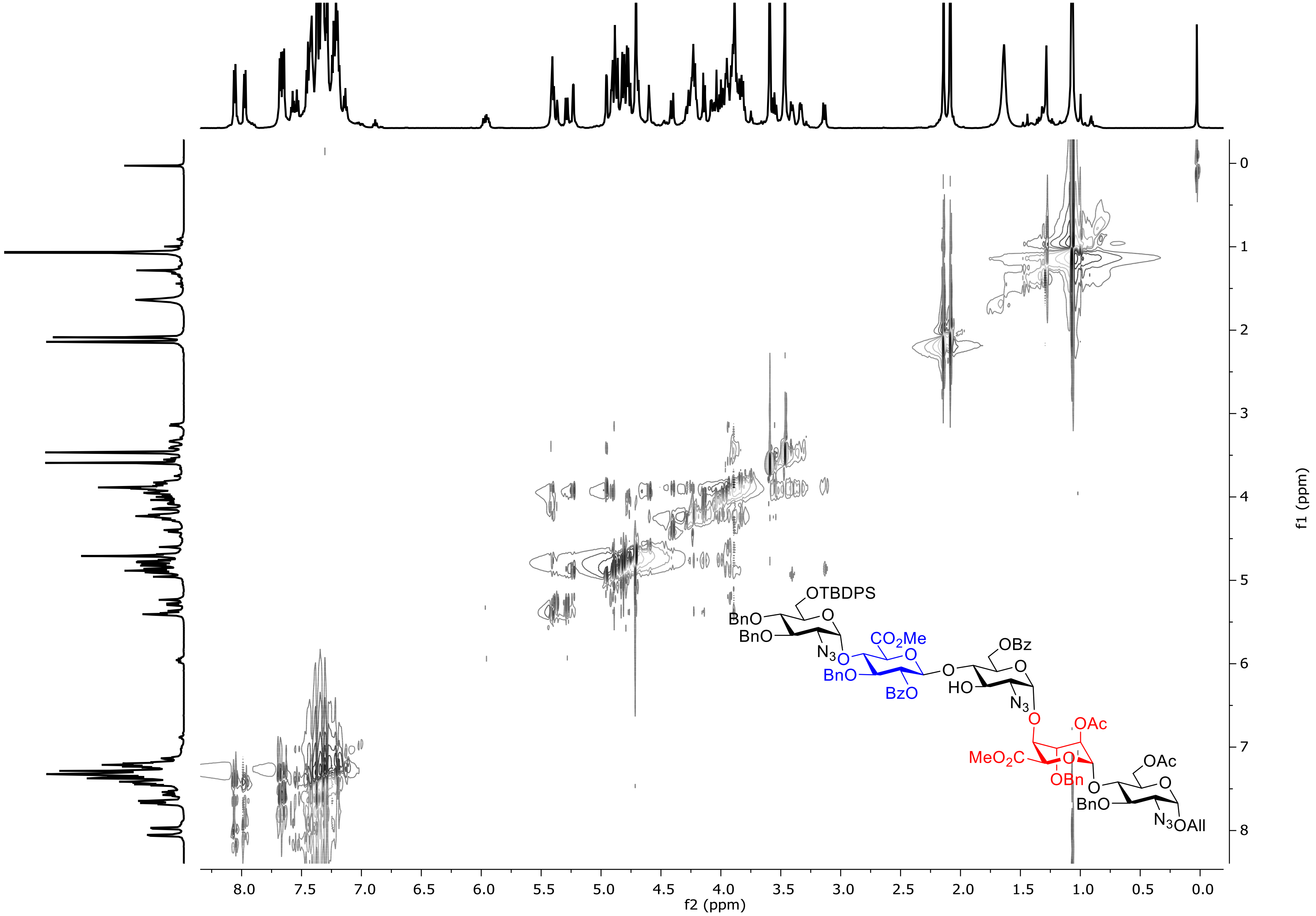

Supplementary Figure S102n. TOCSY NMR Spectrum (600.40, 600.40 MHz, $CDCl_3$) of Compound 51 (Sugar region expanded)

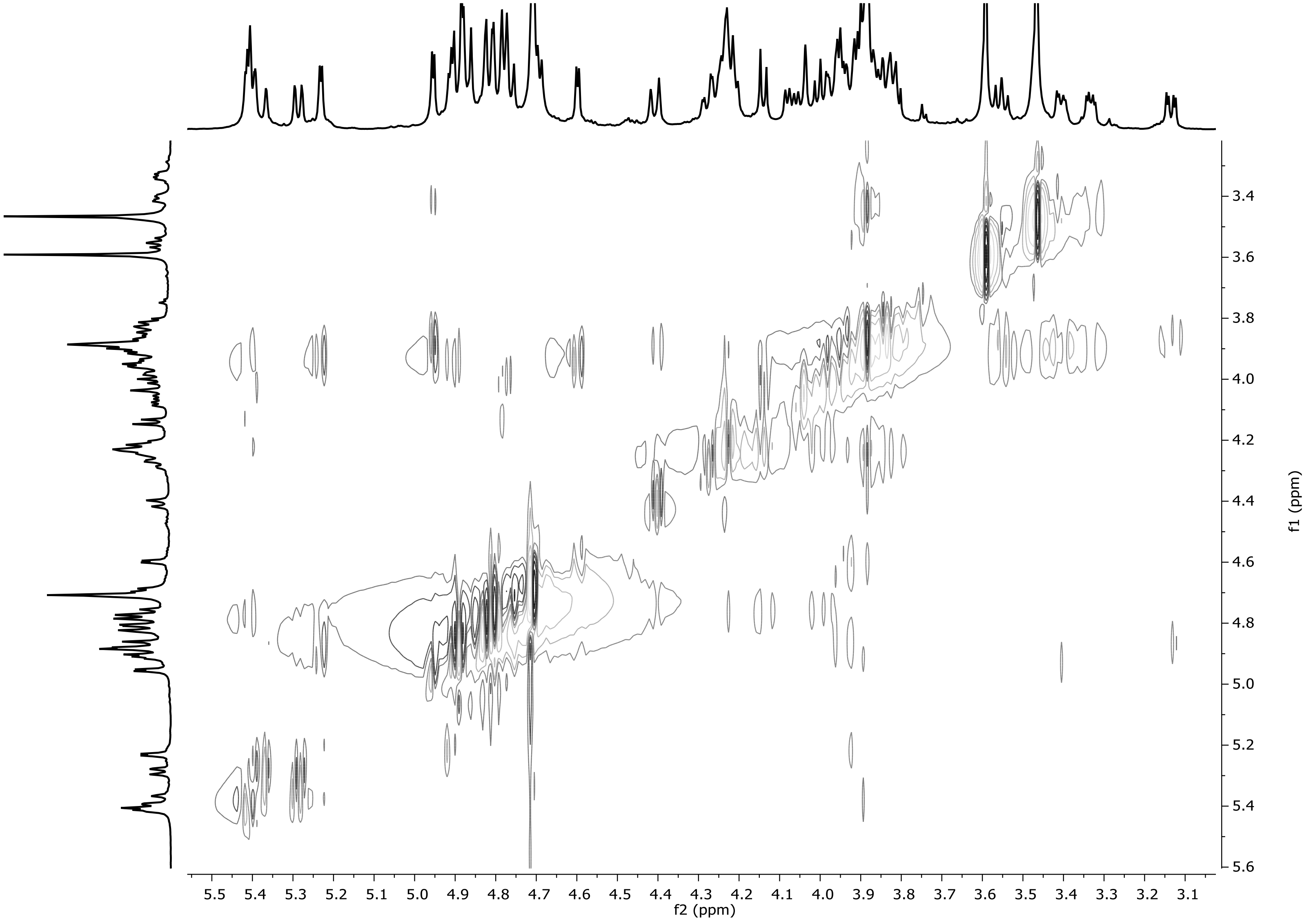

Supplementary Figure S102o. HSQC-TOCSY NMR Spectrum (600.40, 150.99 MHz, $CDCl_3$) of Compound 51

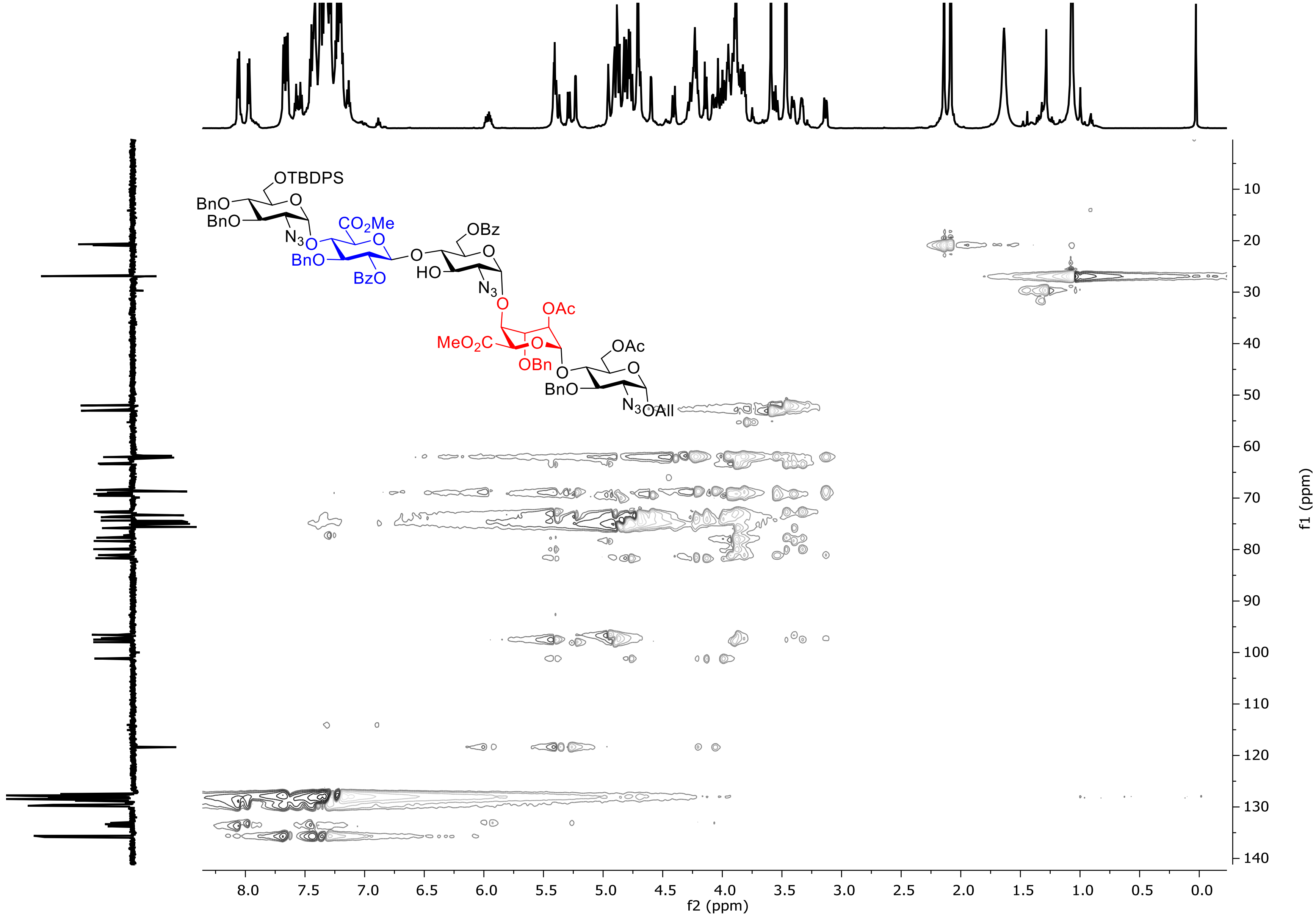

Supplementary Figure S102p. HSQC-TOCSY NMR Spectrum (600.40, 150.99 MHz, $CDCl_3$) of Compound 51 (Sugar region expanded)

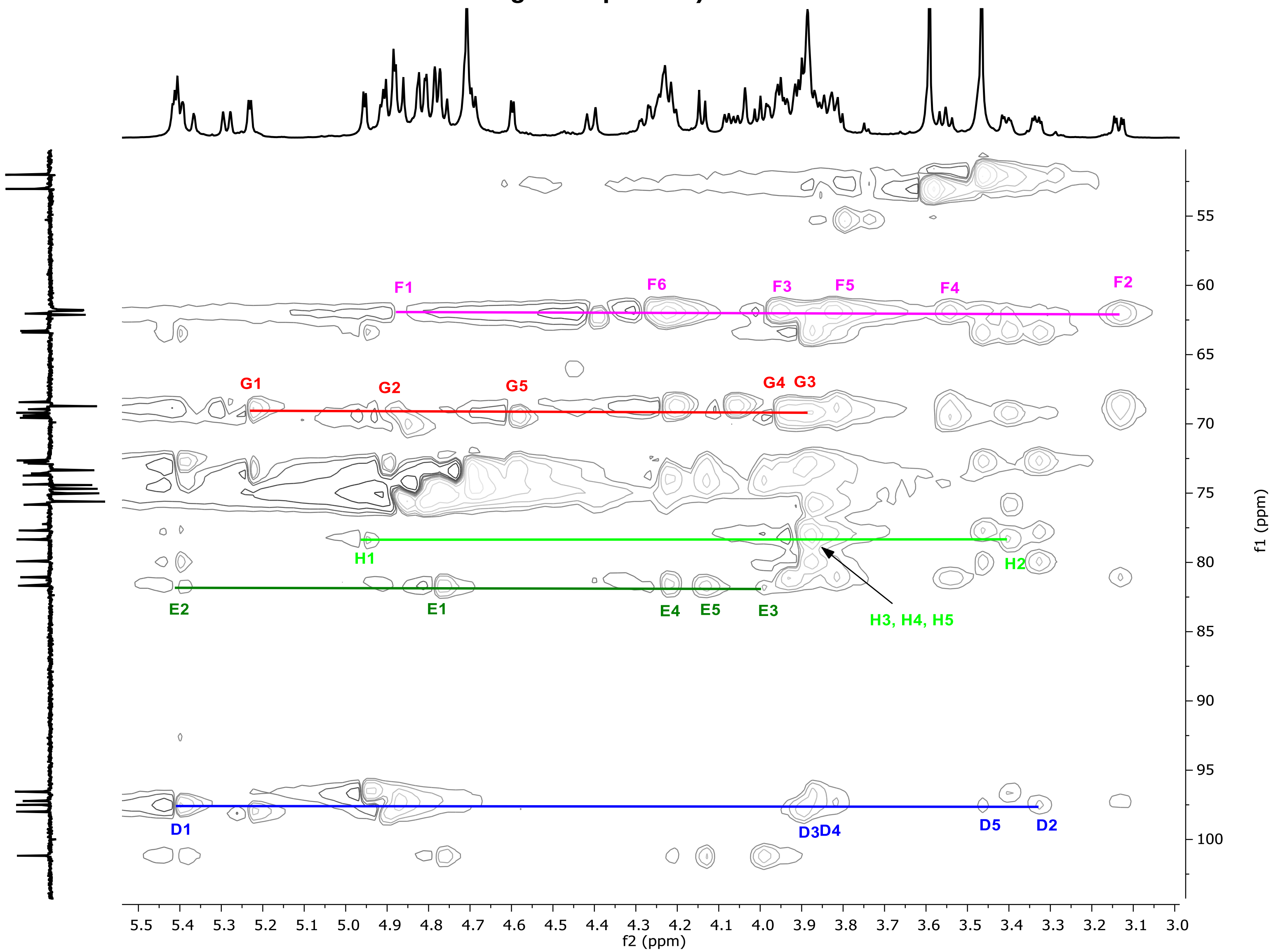

Supplementary Figure S103a. ^{1}H NMR Spectrum (400.31 MHz, $CDCl_3$) of Compound 52

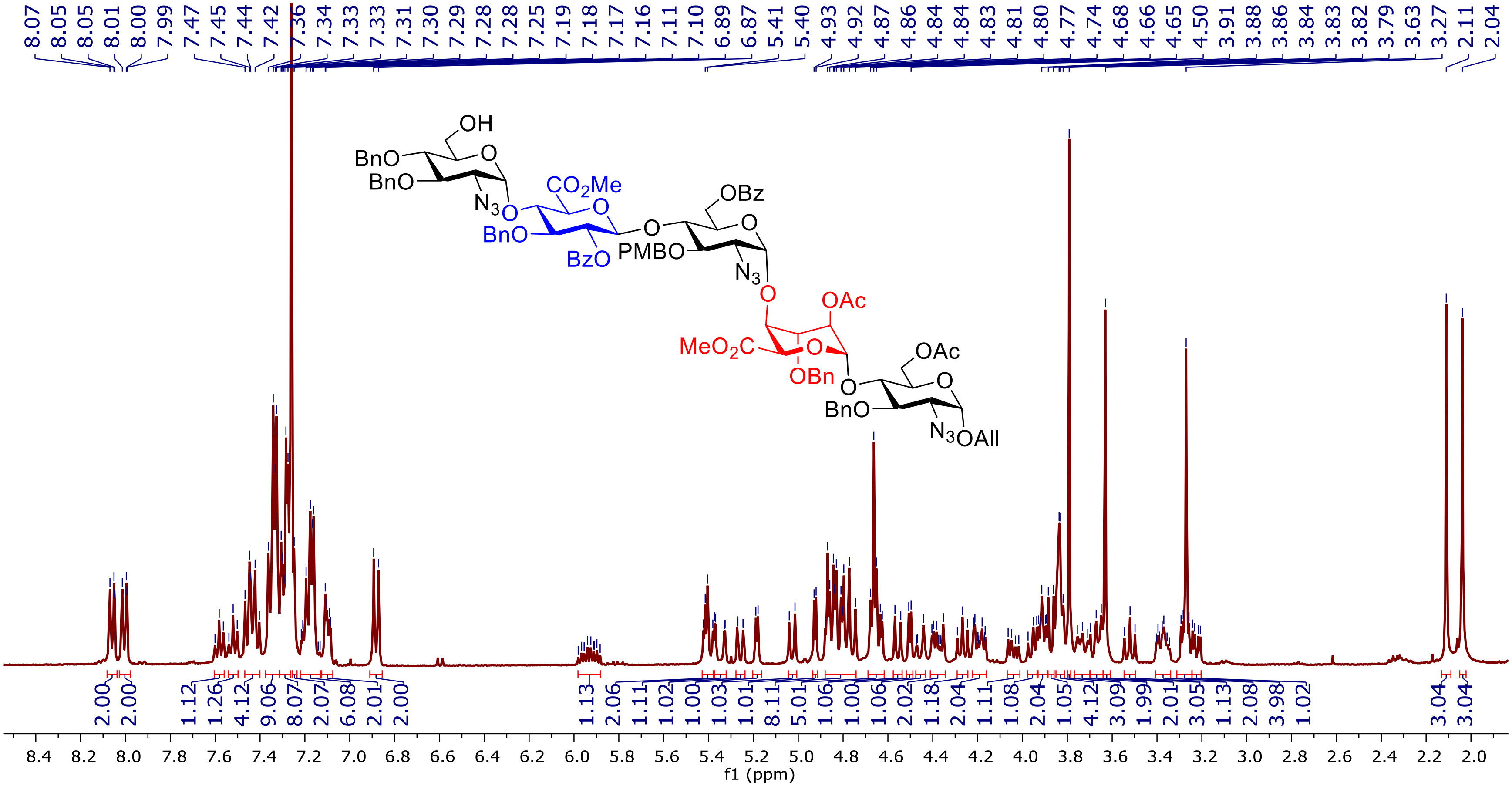

Supplementary Figure S103b. ^{13}C NMR Spectrum (150.99 MHz, $CDCl_3$) of Compound 52

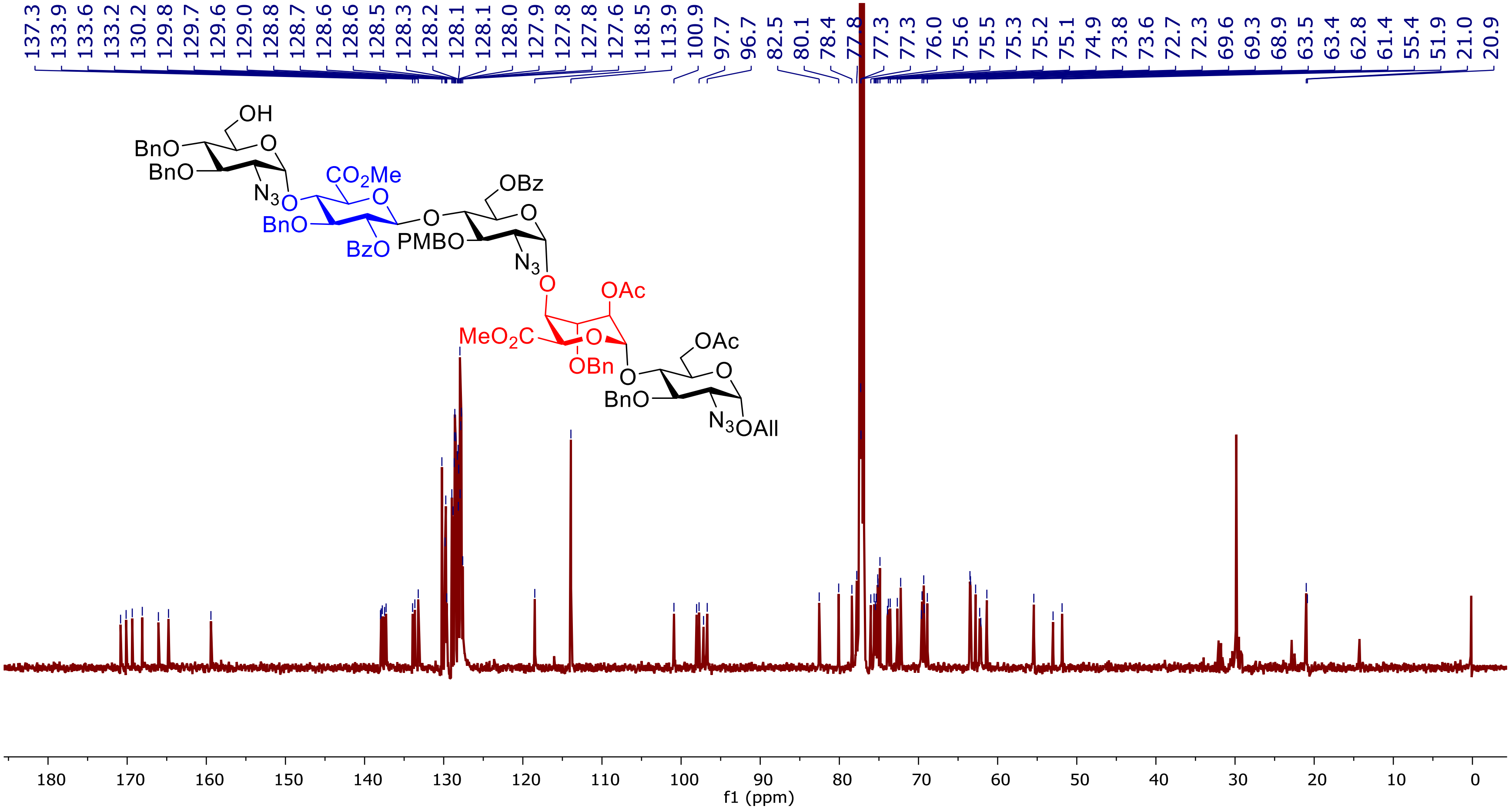

Supplementary Figure S103c. DEPT NMR Spectrum (150.97 MHz, $CDCl_3$) of Compound 52

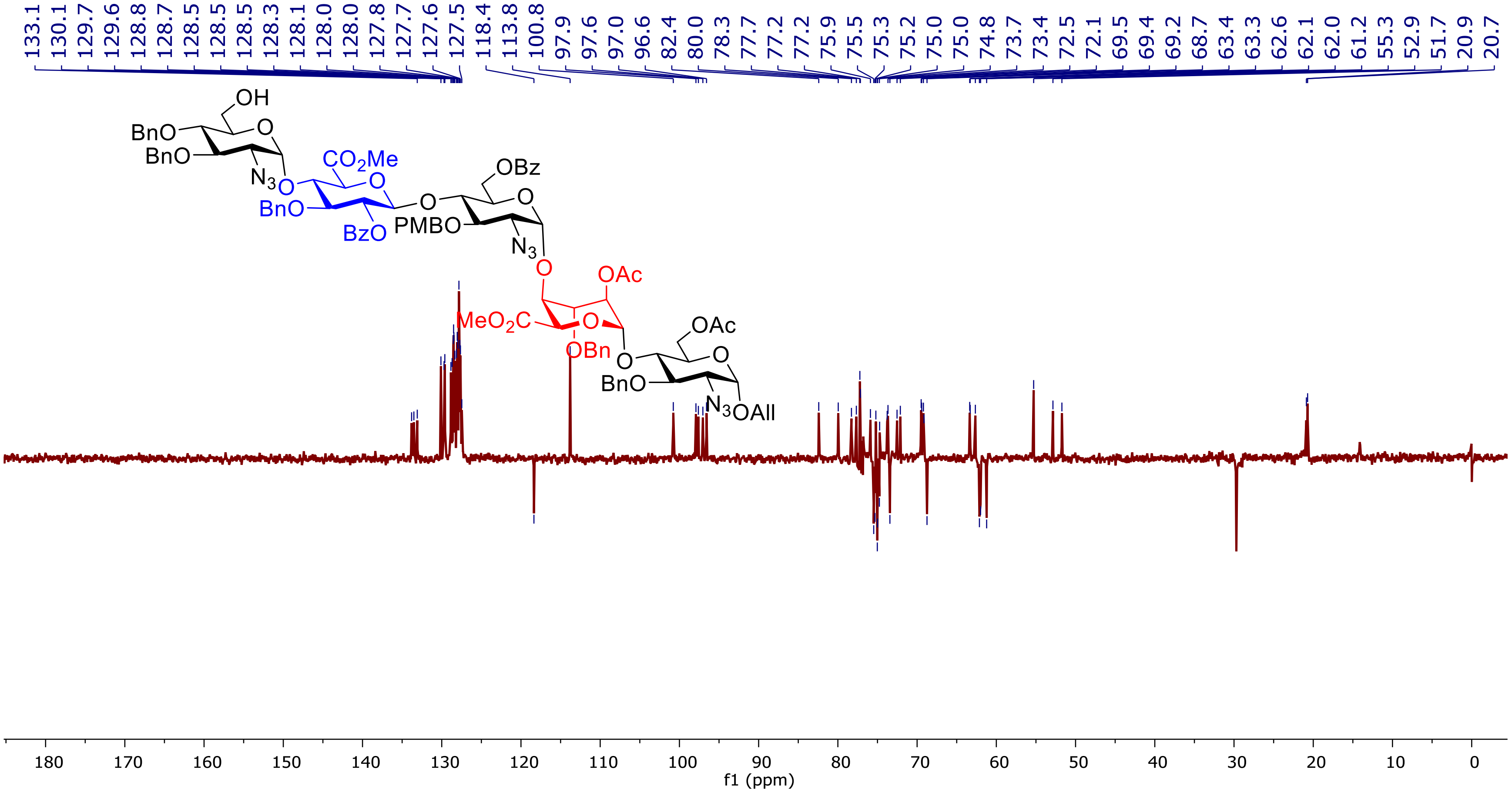

Supplementary Figure S103d. COSY NMR Spectrum (600.40, 600.40 MHz, $CDCl_3$) of Compound 52

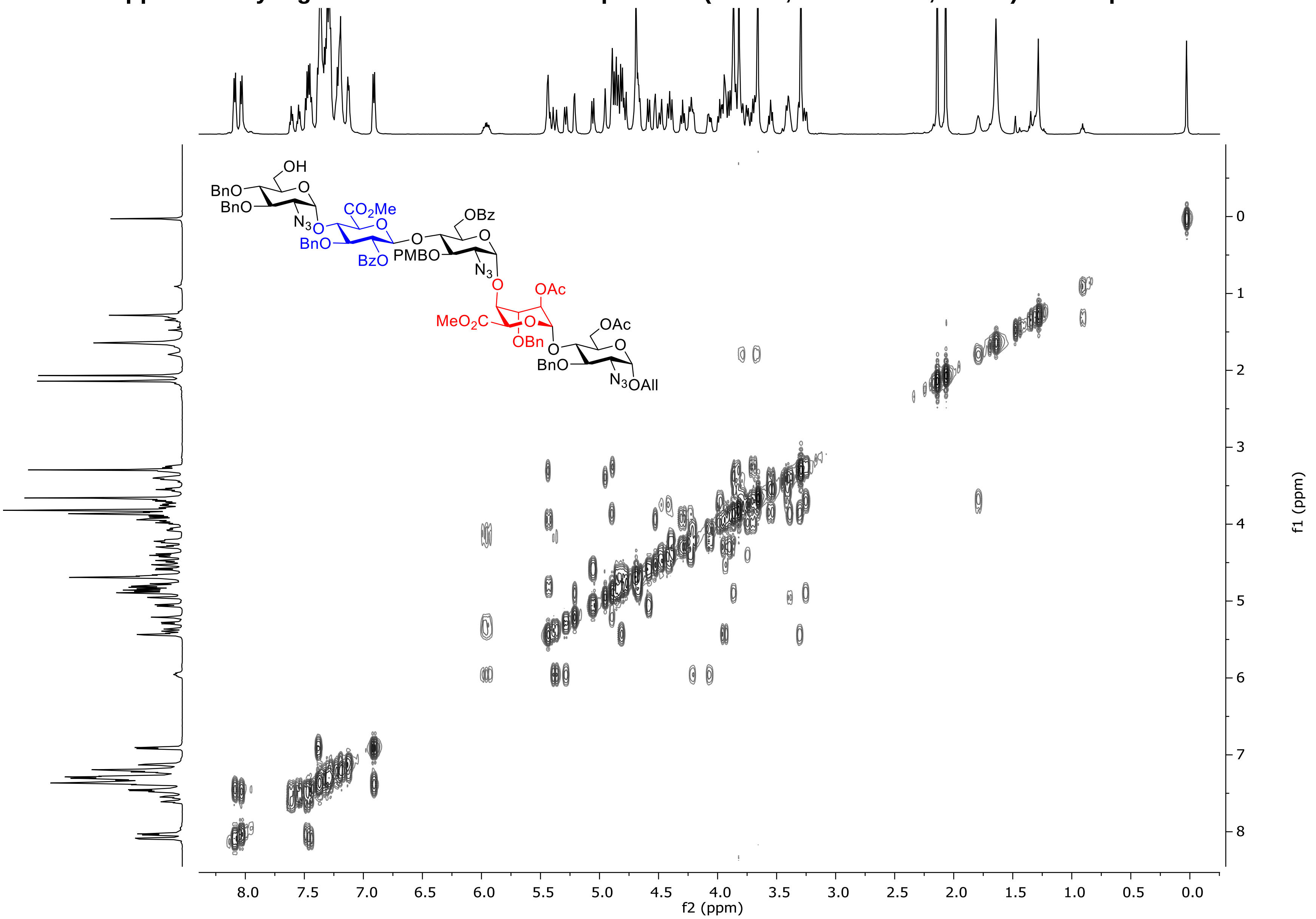

Supplementary Figure S103e. COSY NMR Spectrum (600.40, 600.40 MHz, $CDCl_3$) of Compound 52 (Sugar region expanded)

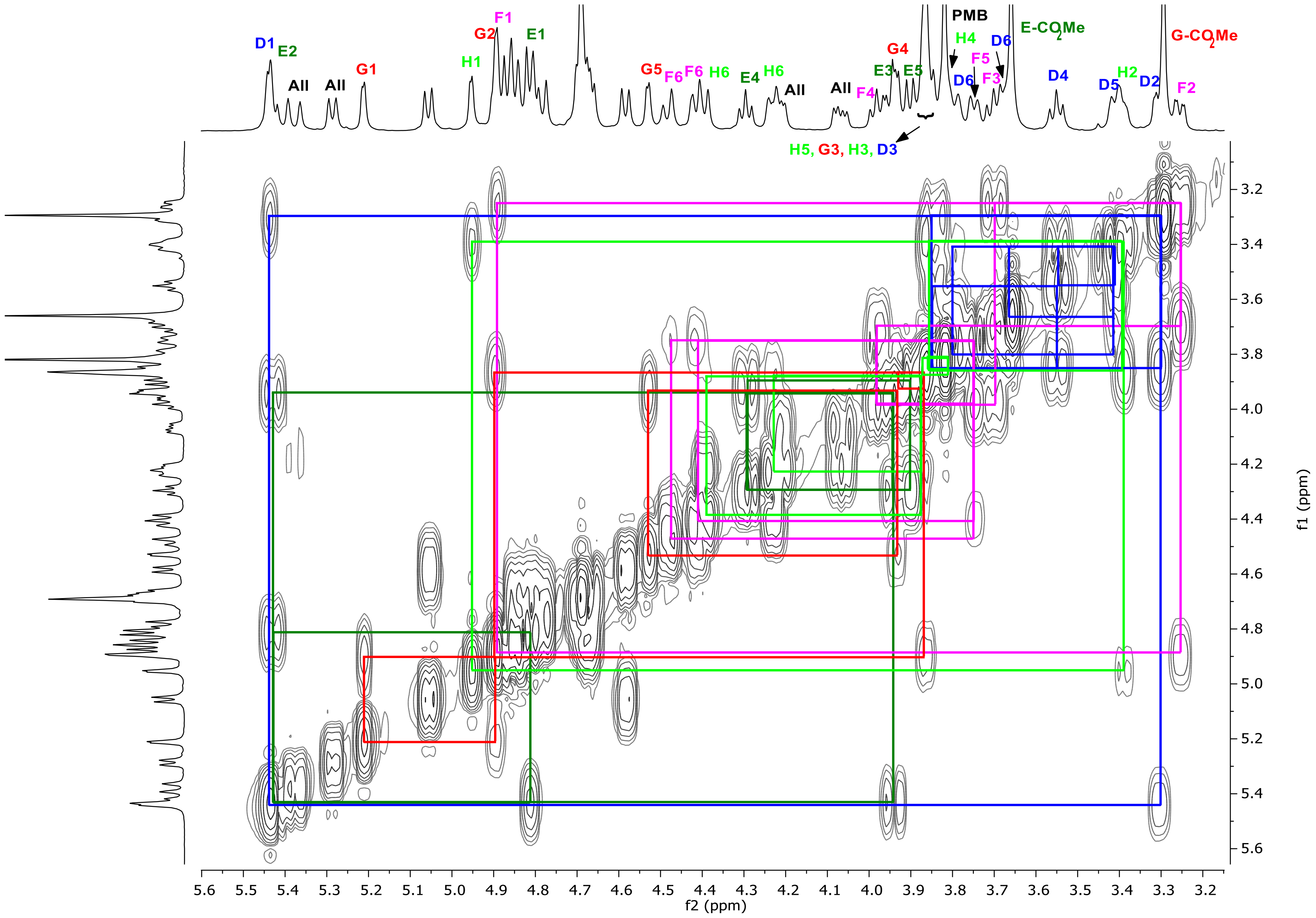

Supplementary Figure S103f. HSQC NMR Spectrum (600.40, 150.99MHz, CDCl₃) of Compound 52

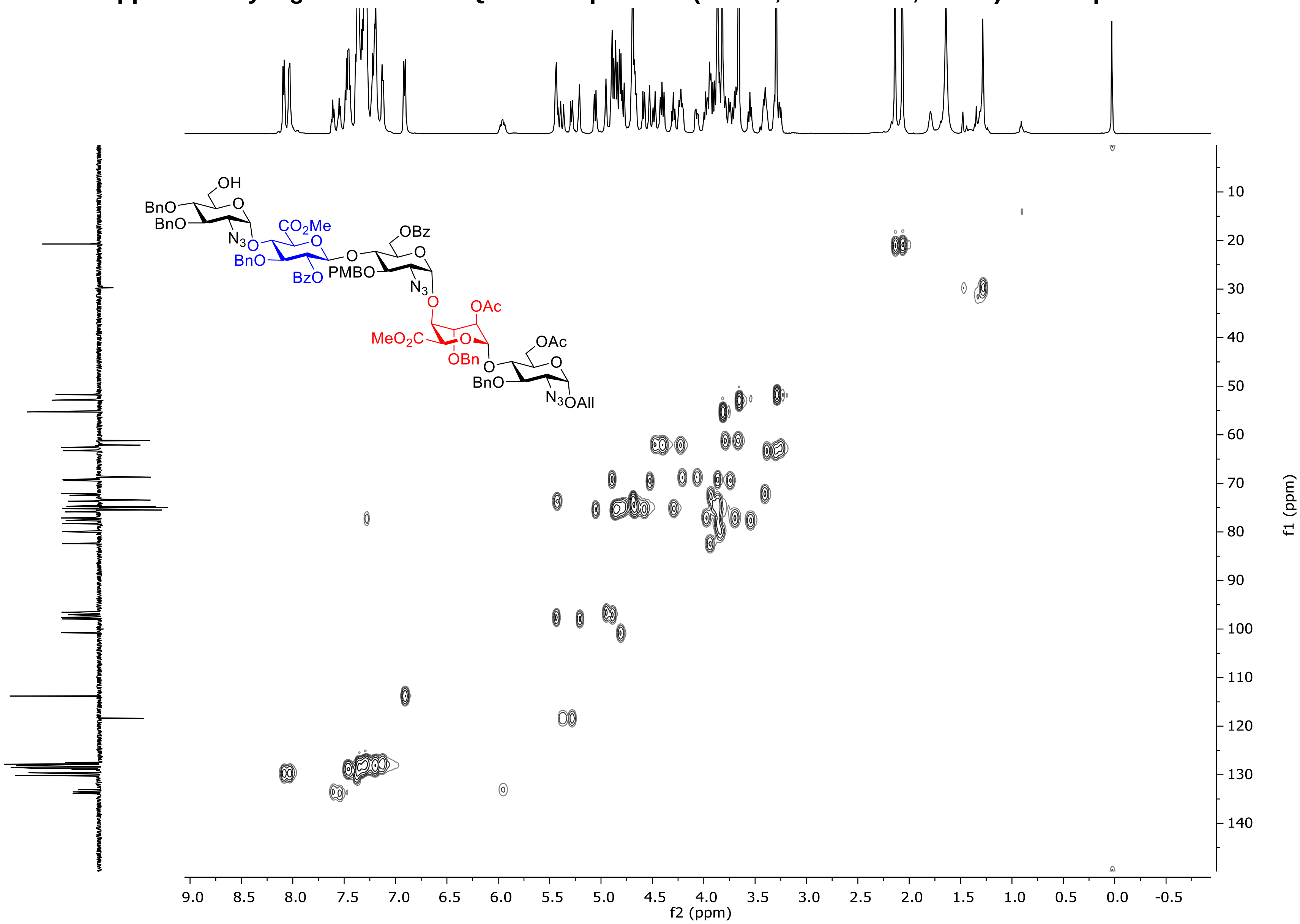

Supplementary Figure S103g. HSQC NMR Spectrum (600.40, 150.99 MHz, CDCl₃) of Compound 52 (Sugar region expanded)

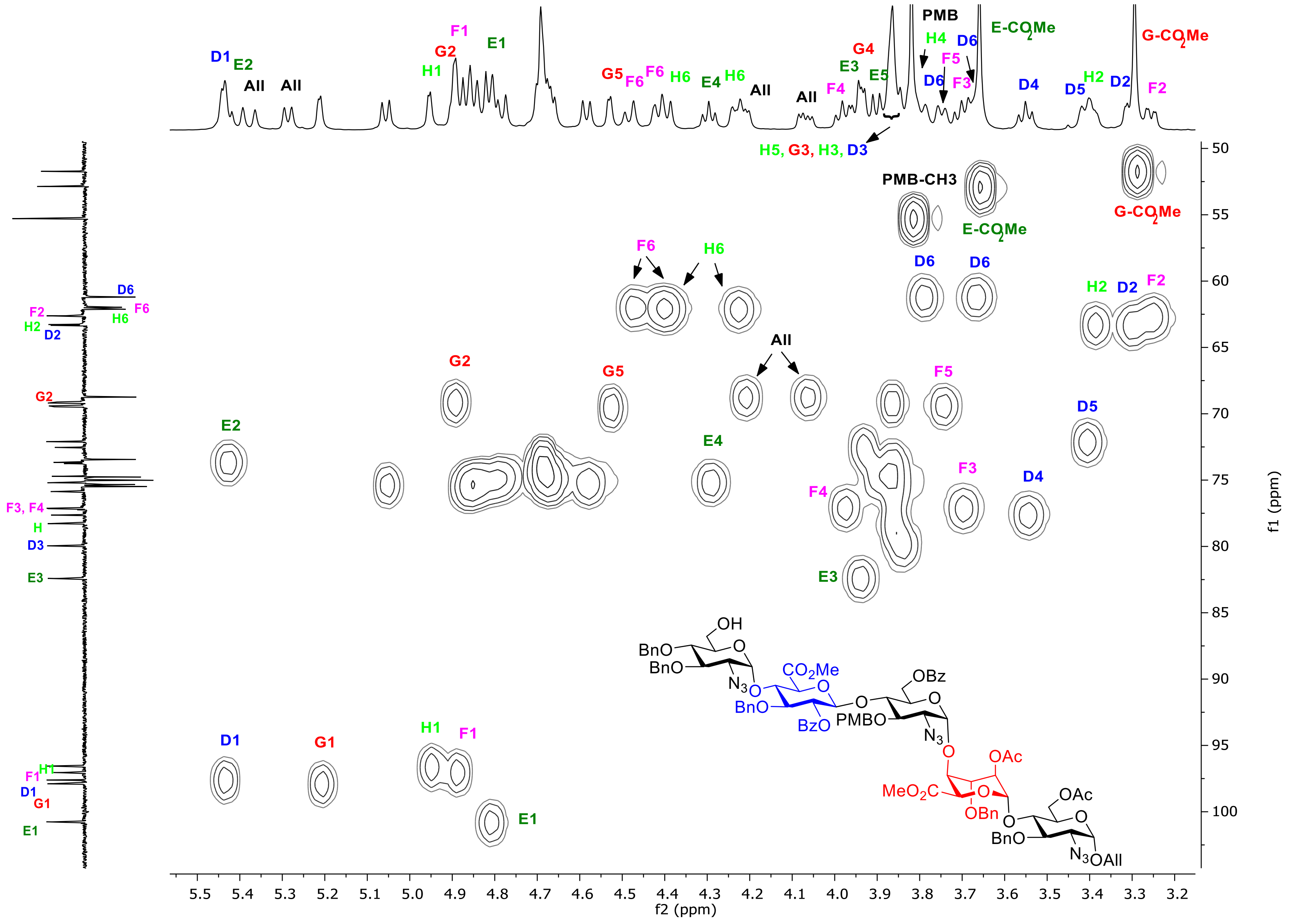

Supplementary Figure S103h. Coupled HSQC NMR Spectrum (600.40, 150.99 MHz, $CDCl_3$) of Compound 52

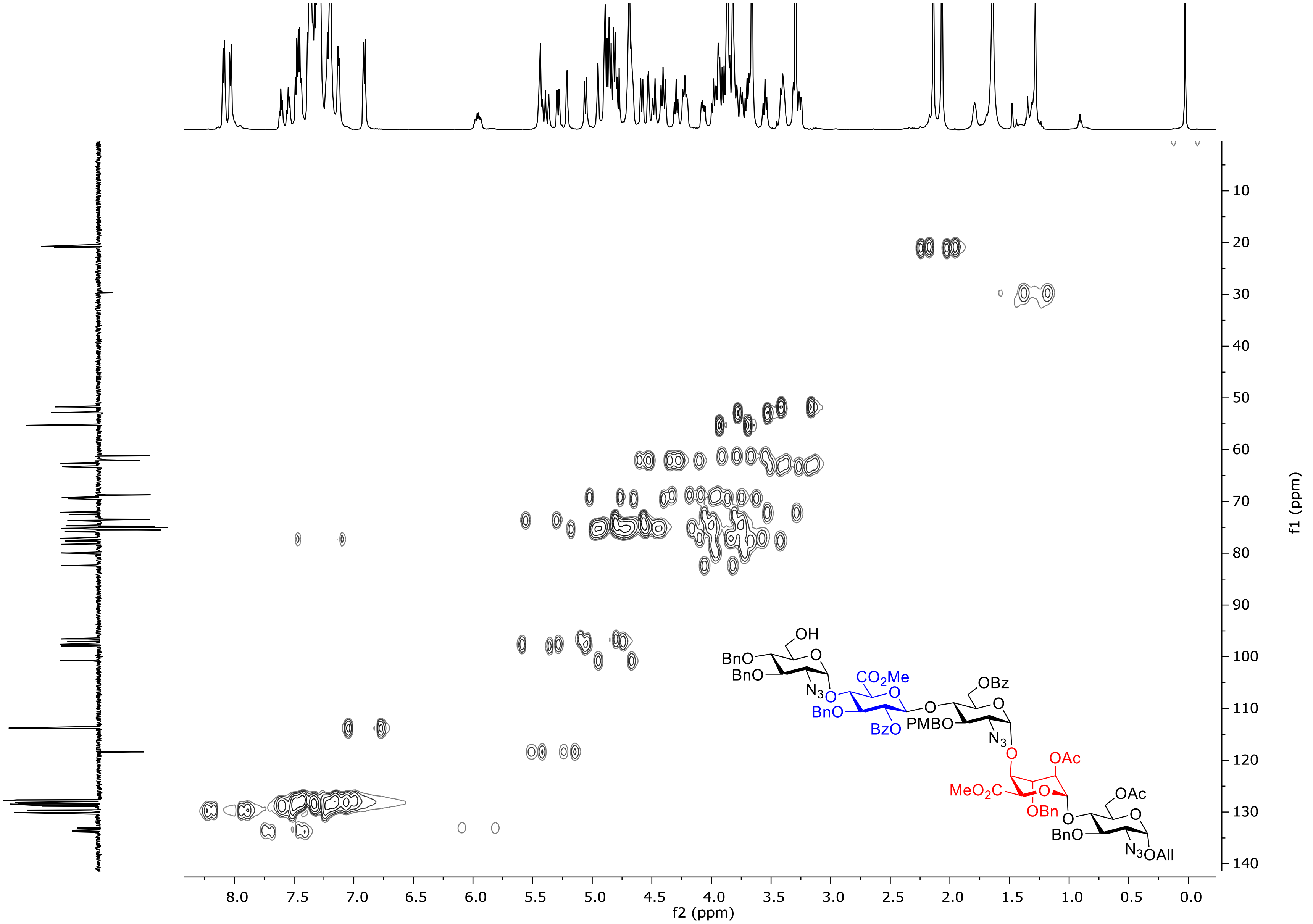

Supplementary Figure S103i. Coupled HSQC NMR Spectrum (600.40, 150.99 MHz, $CDCl_3$) of Compound 52 (Sugar region expanded)

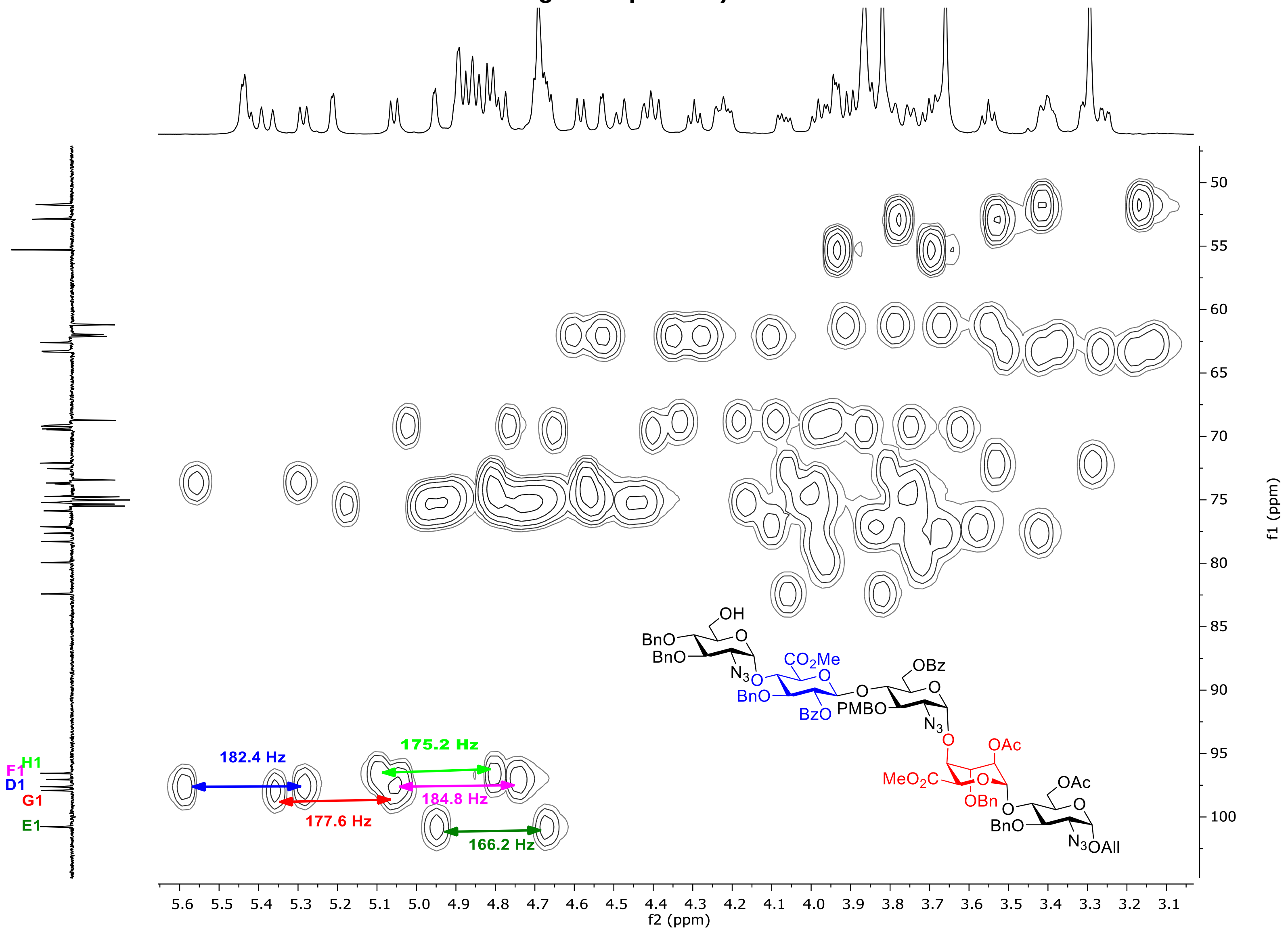

Supplementary Figure S103j. HMBC NMR Spectrum (600.40, 150.99 MHz, $CDCl_3$) of Compound 52

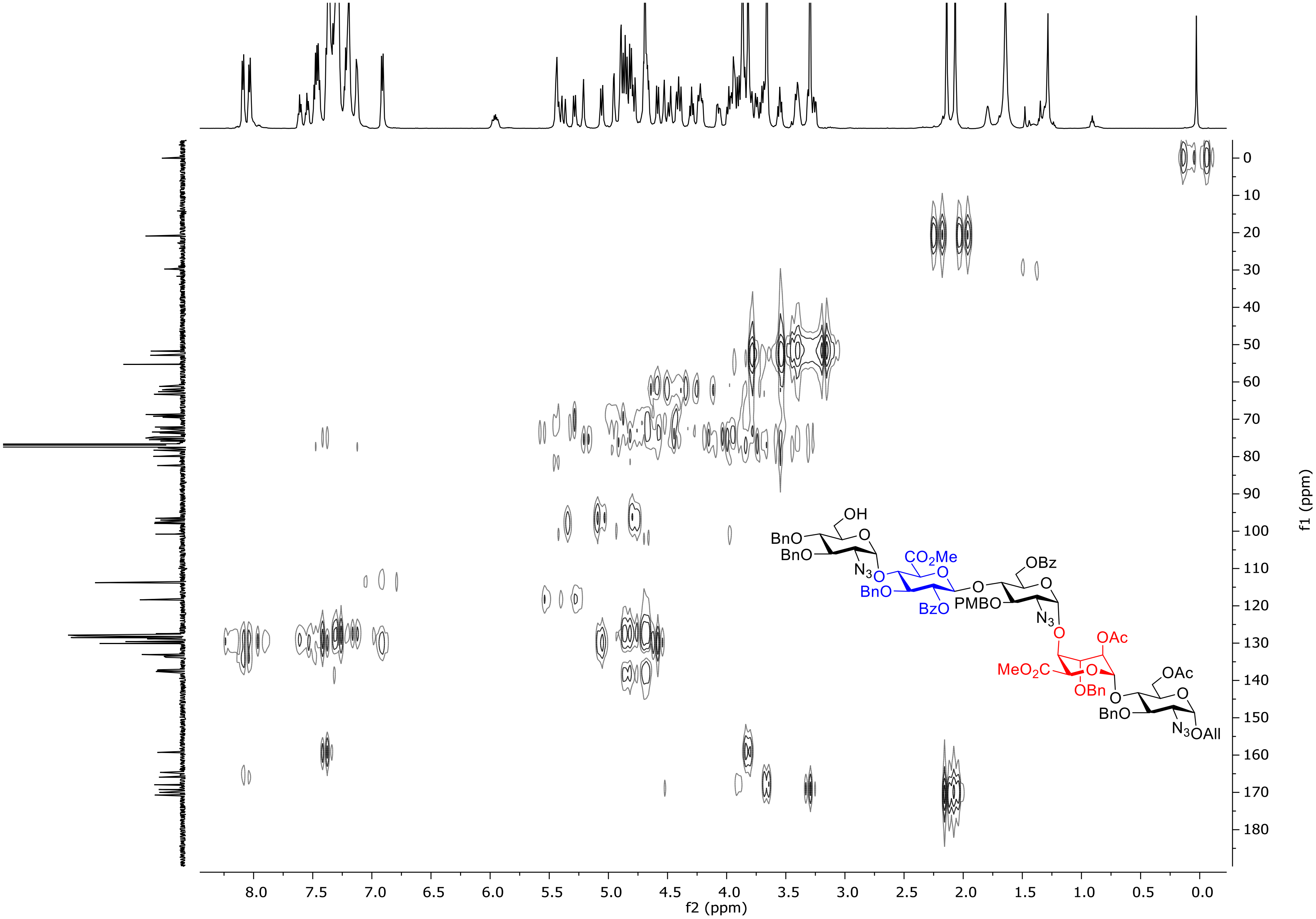

Supplementary Figure S103k. HMBC NMR Spectrum (600.40, 150.99 MHz, $CDCl_3$) of Compound 52 (Carbonyl region expanded)

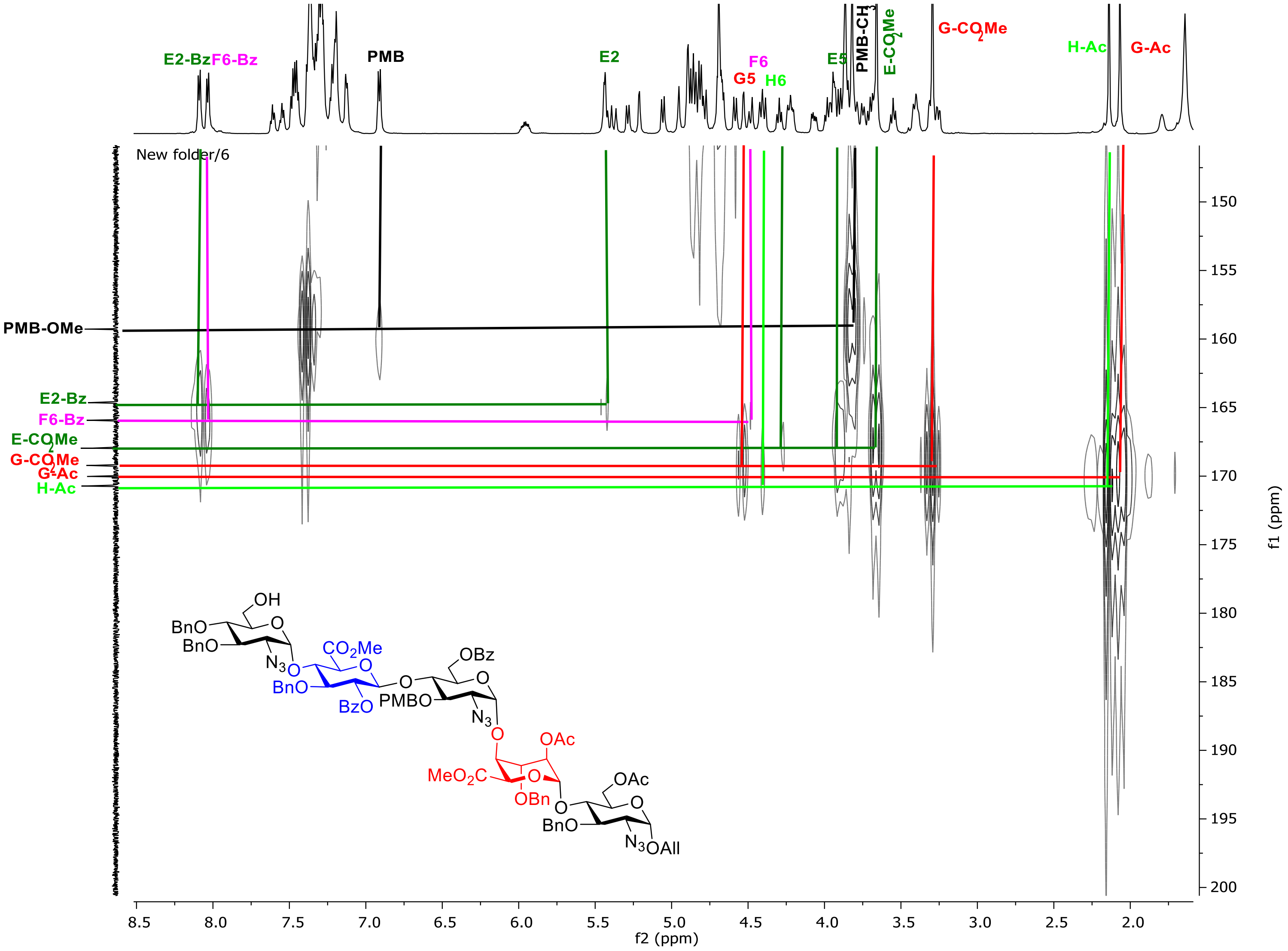

Supplementary Figure S103l. TOCSY NMR Spectrum (600.40, 600.40 MHz, $CDCl_3$) of Compound 52

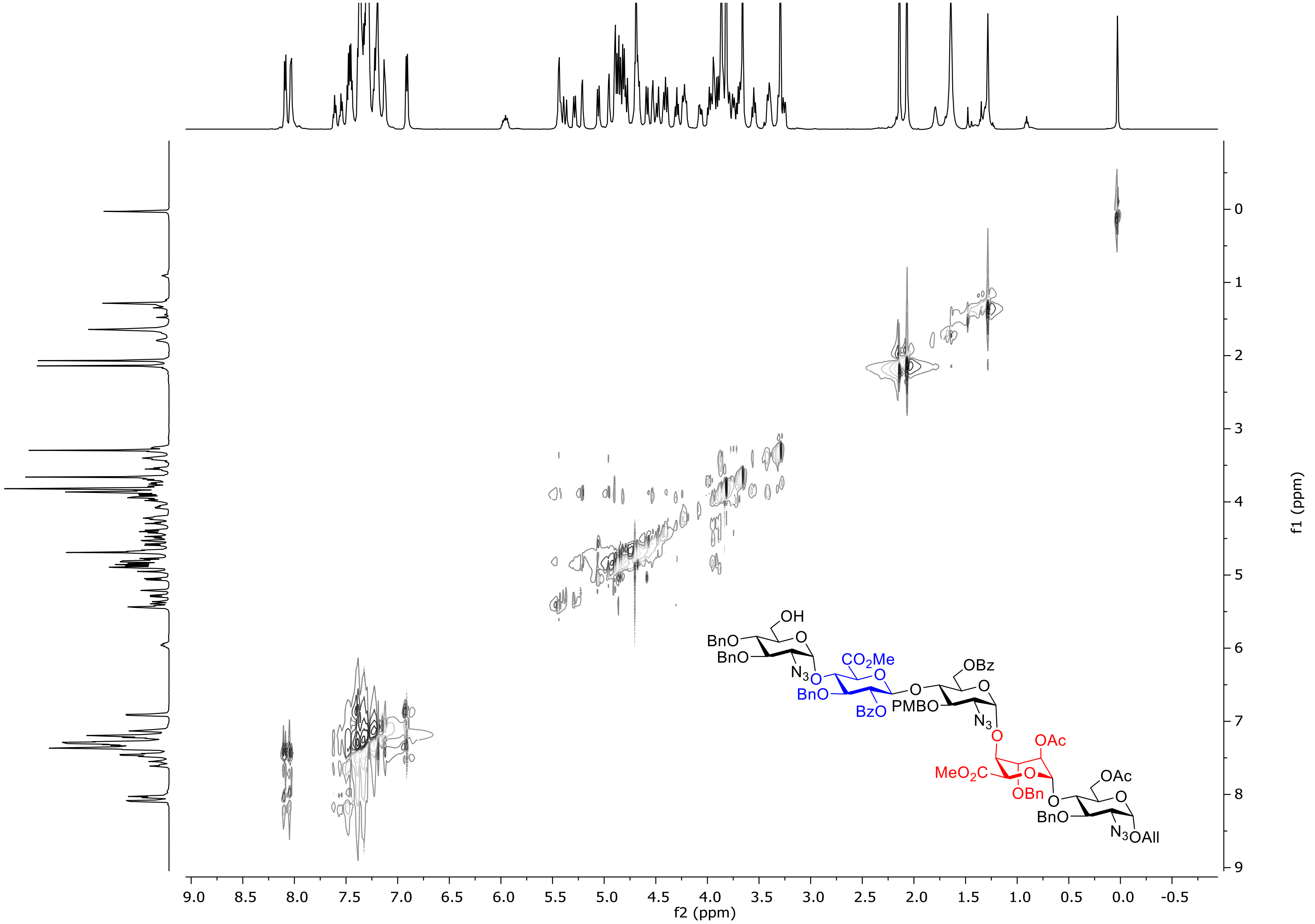

Supplementary Figure S103m. TOCSY NMR Spectrum (600.40, 600.40 MHz, $CDCl_3$) of Compound 52 (Sugar region expanded)

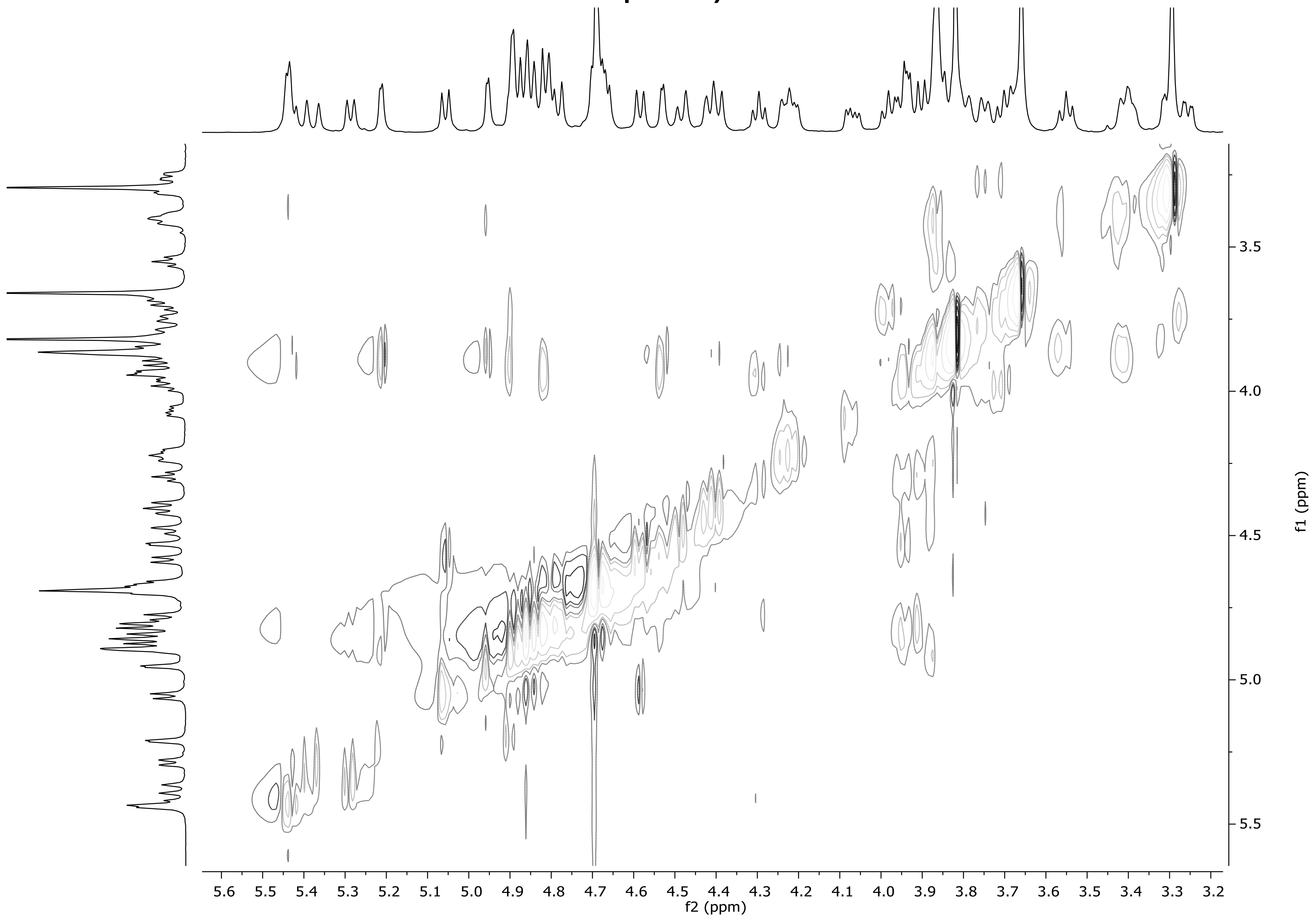

Supplementary Figure S103n. HSQC-TOCSY NMR Spectrum (600.40, 150.99 MHz, $CDCl_3$) of Compound 52

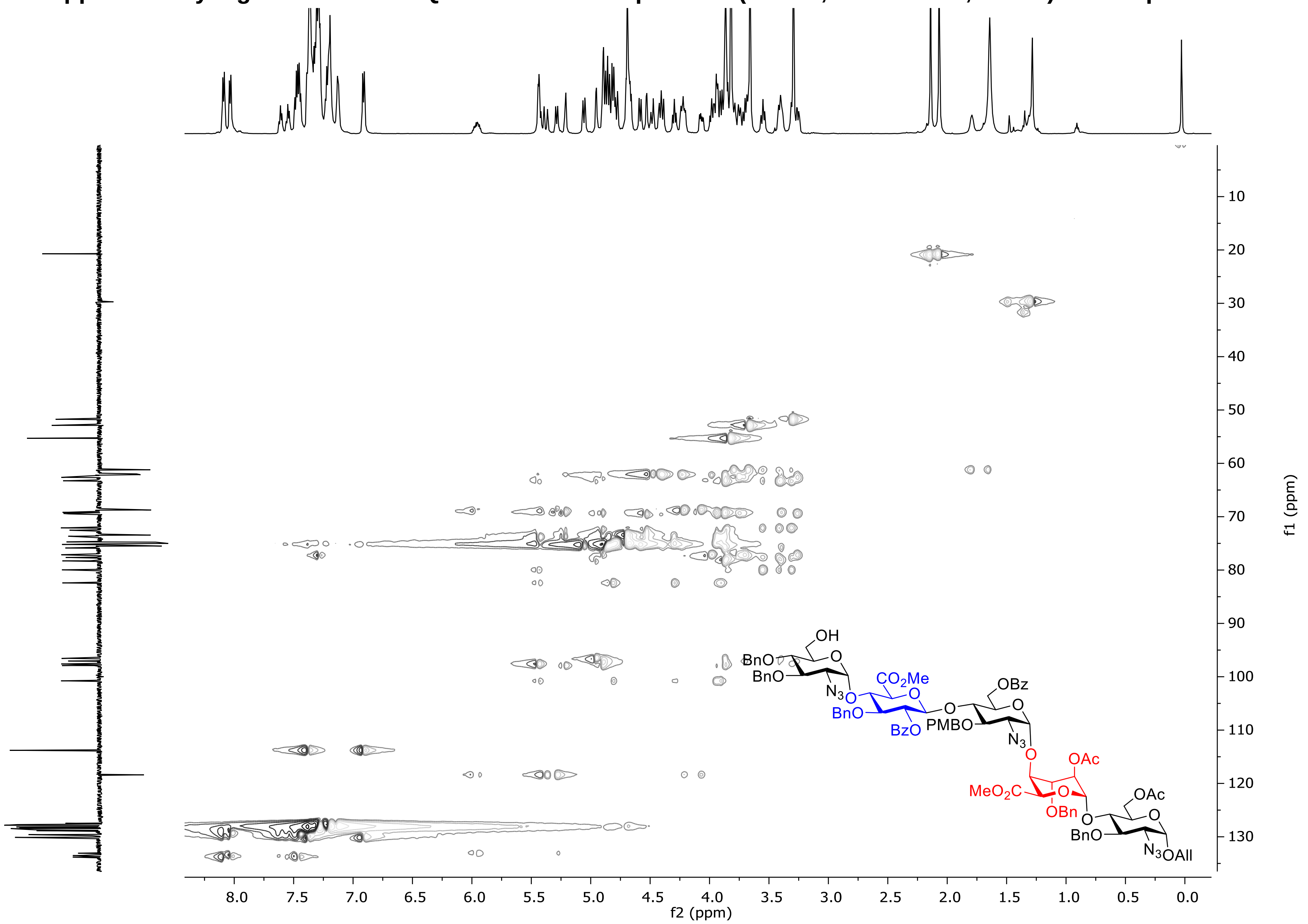

Supplementary Figure S103o. HSQC-TOCSY NMR Spectrum (600.40, 150.99 MHz, $CDCl_3$) of Compound 52 (Sugar region expanded)

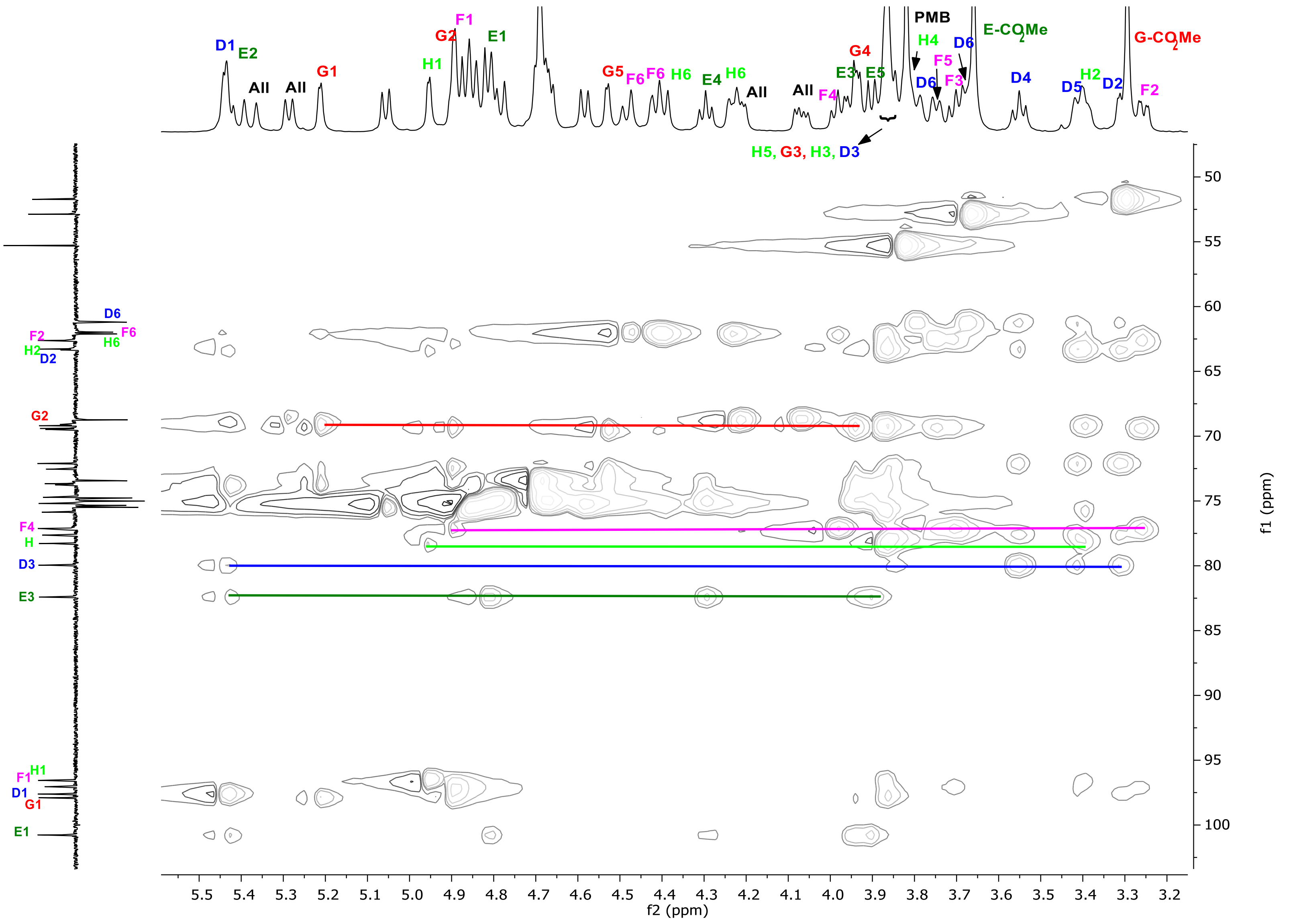

Supplementary Figure S104a. ^{1}H NMR Spectrum (600.40 MHz, $CDCl_3$) of Compound 53

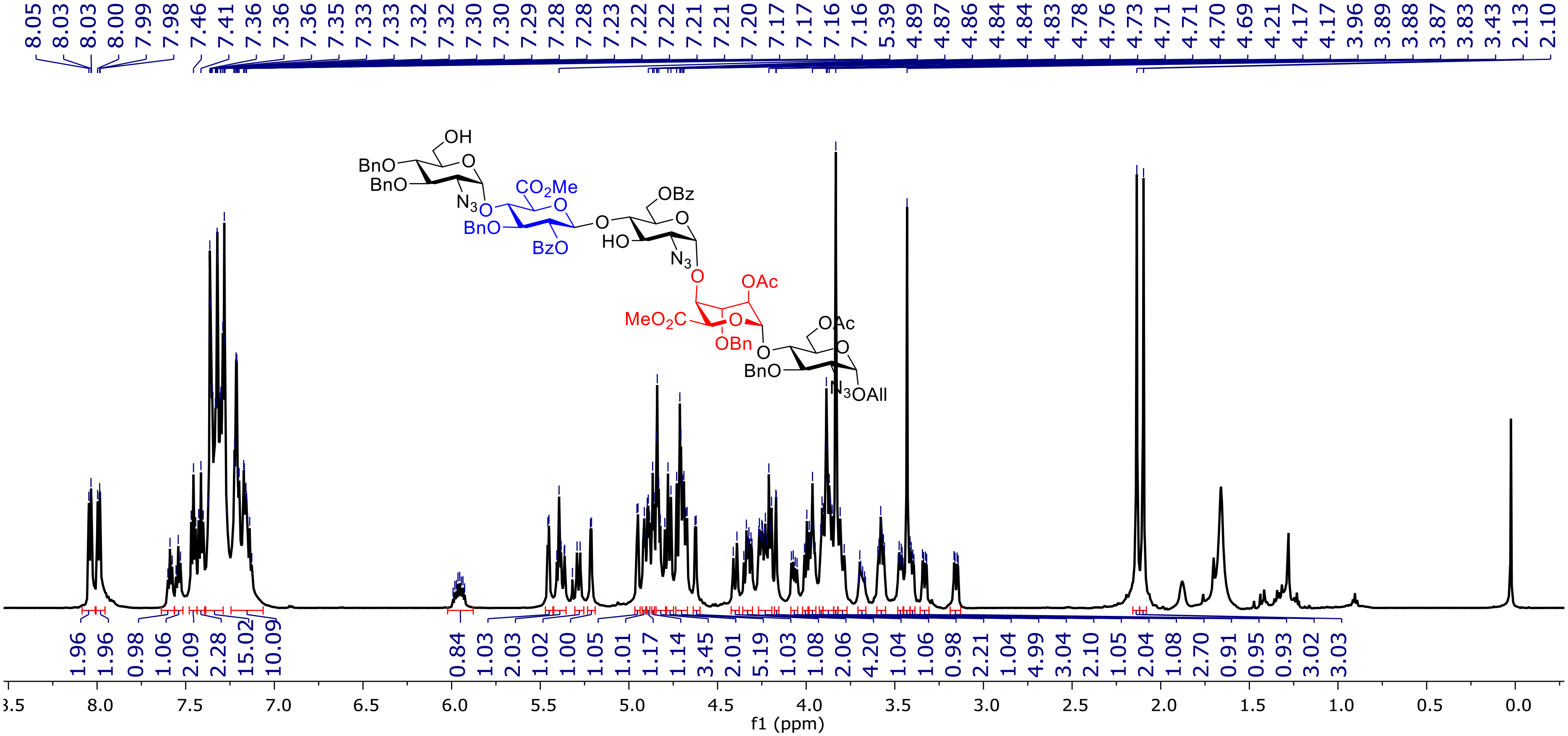

Supplementary Figure S104b. ^{13}C NMR Spectrum (150.99 MHz, $CDCl_3$) of Compound 53

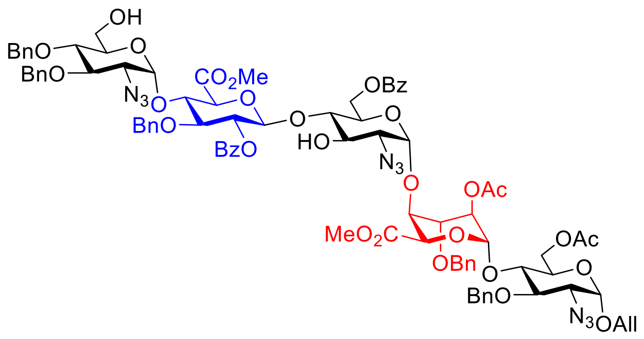

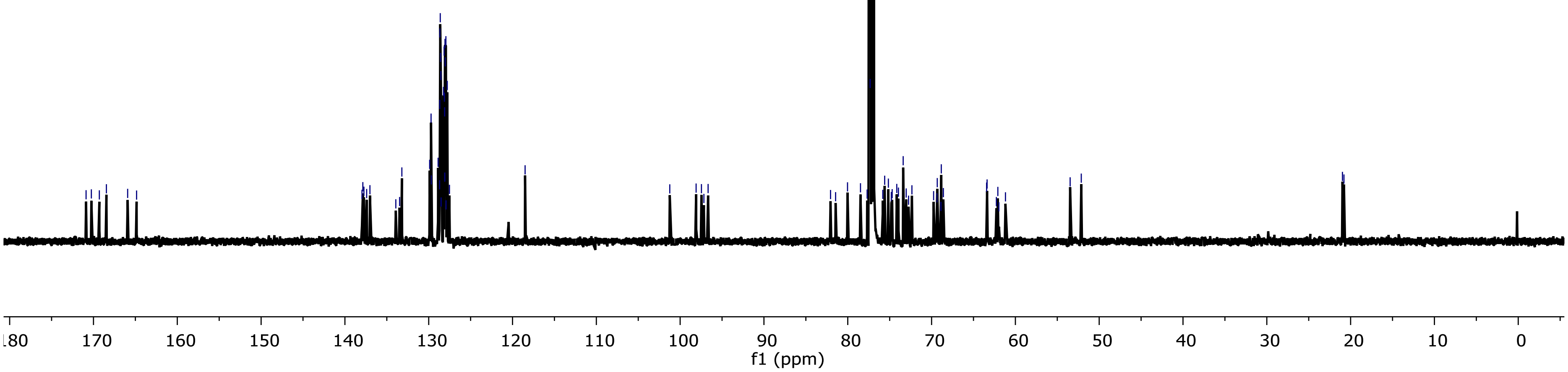

Supplementary Figure S104c. DEPT NMR Spectrum (150.97 MHz, $CDCl_3$) of Compound 53

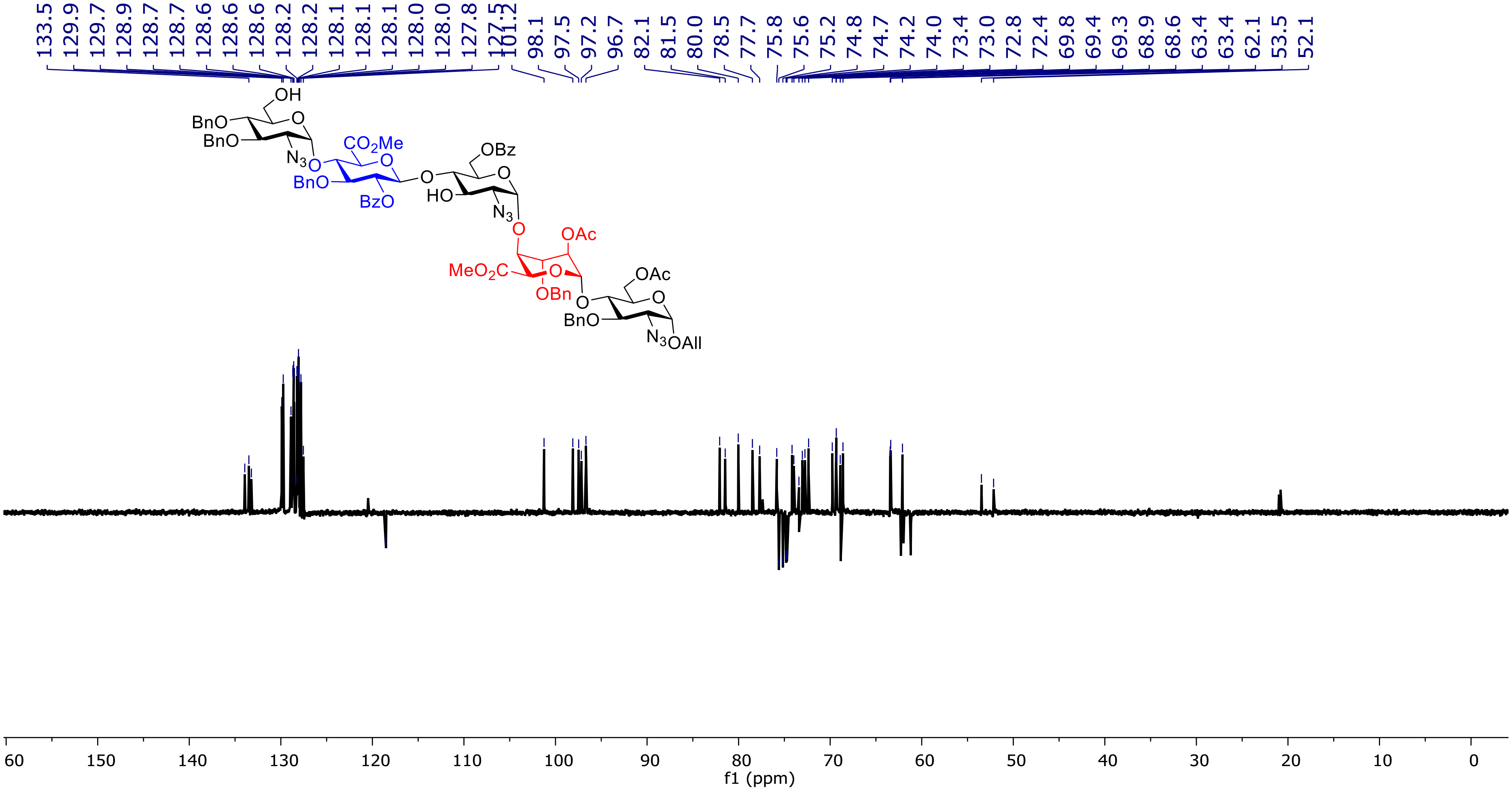

Supplementary Figure S104d. COSY NMR Spectrum (600.40, 600.40 MHz, $CDCl_3$) of Compound 53

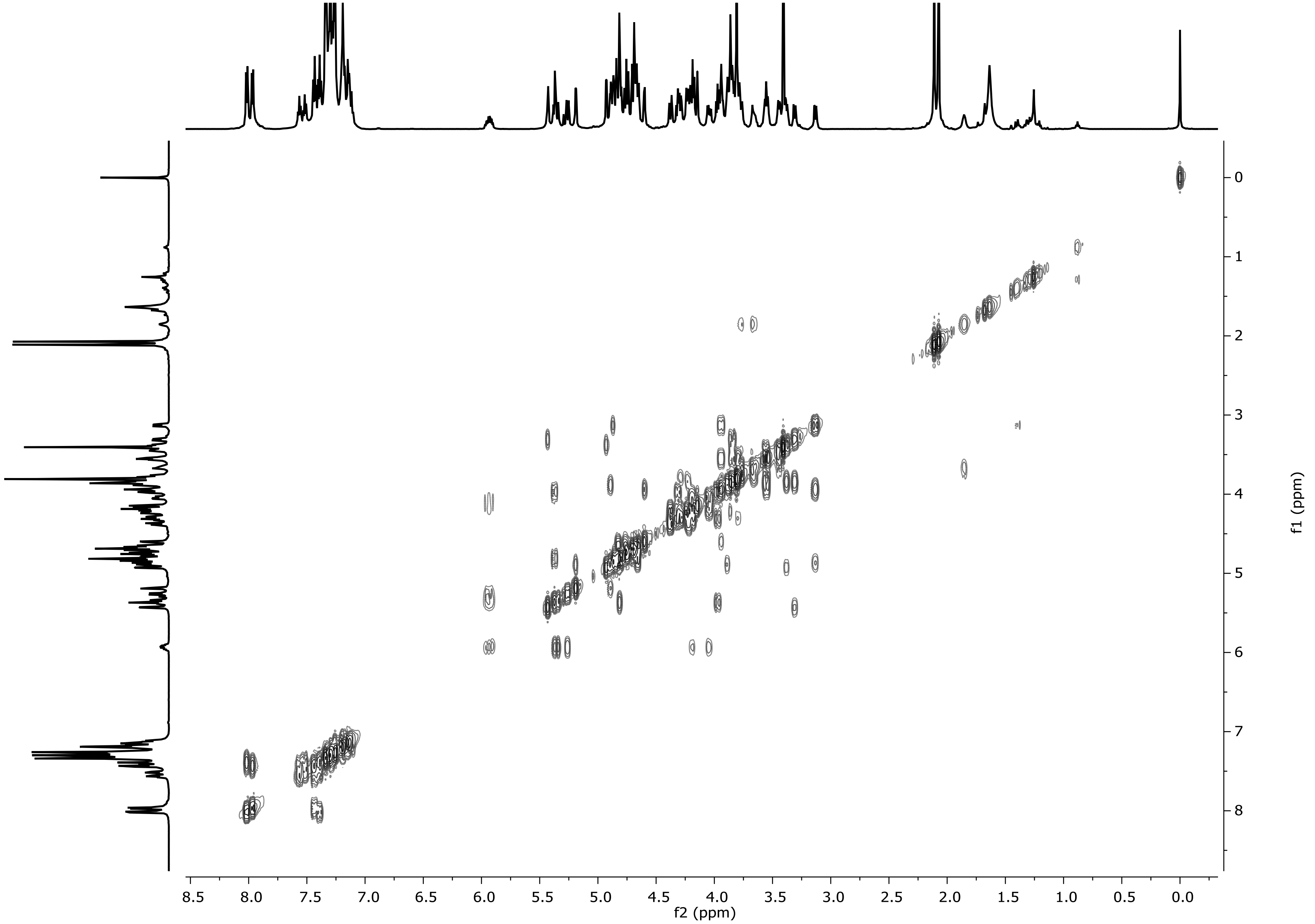

Supplementary Figure S104e. COSY NMR Spectrum (600.40, 600.40 MHz, $CDCl_3$) of Compound 53 (Sugar region expanded)

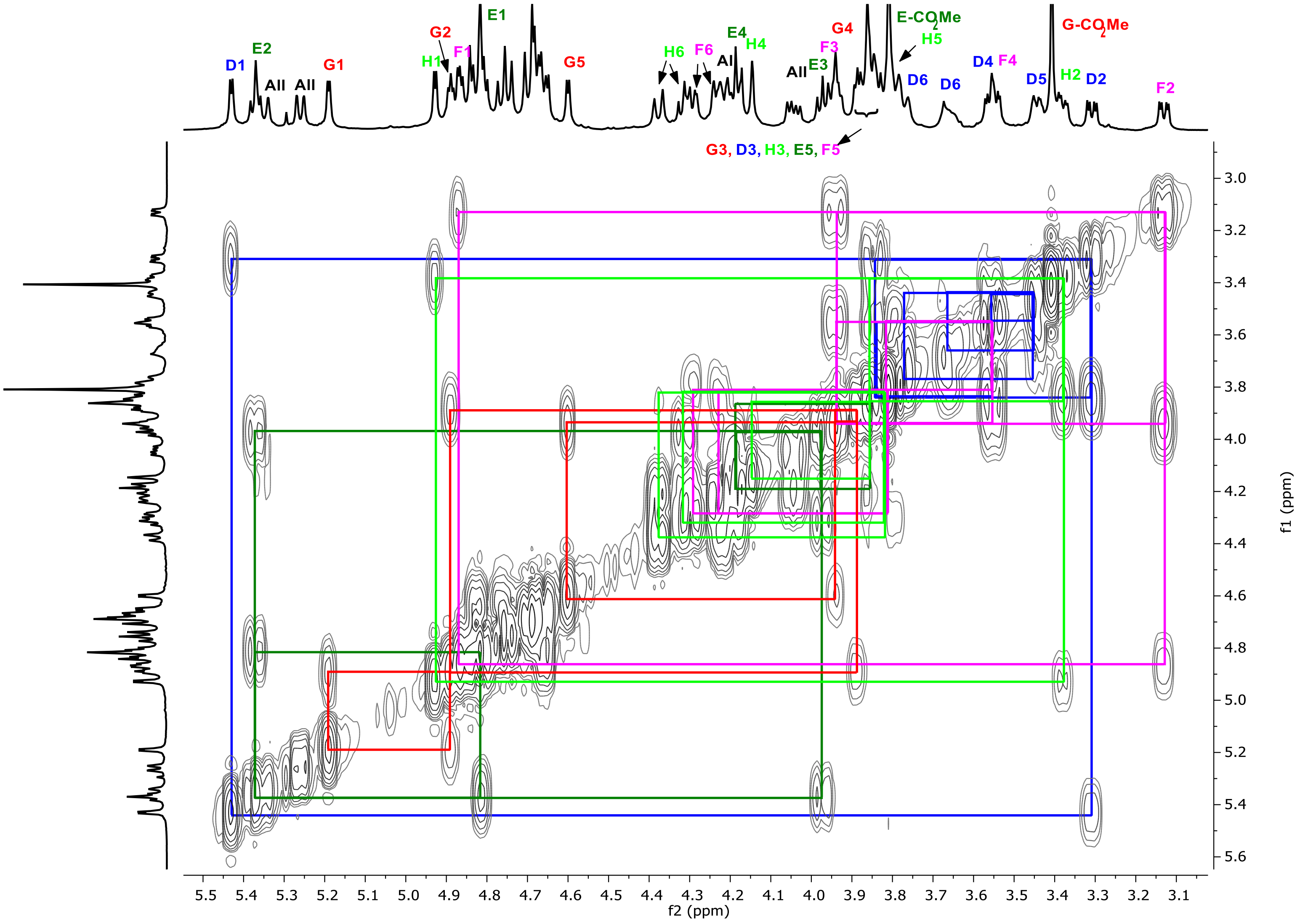

Supplementary Figure S104f. HSQC NMR Spectrum (600.40, 150.99MHz, $CDCl_3$) of Compound 53

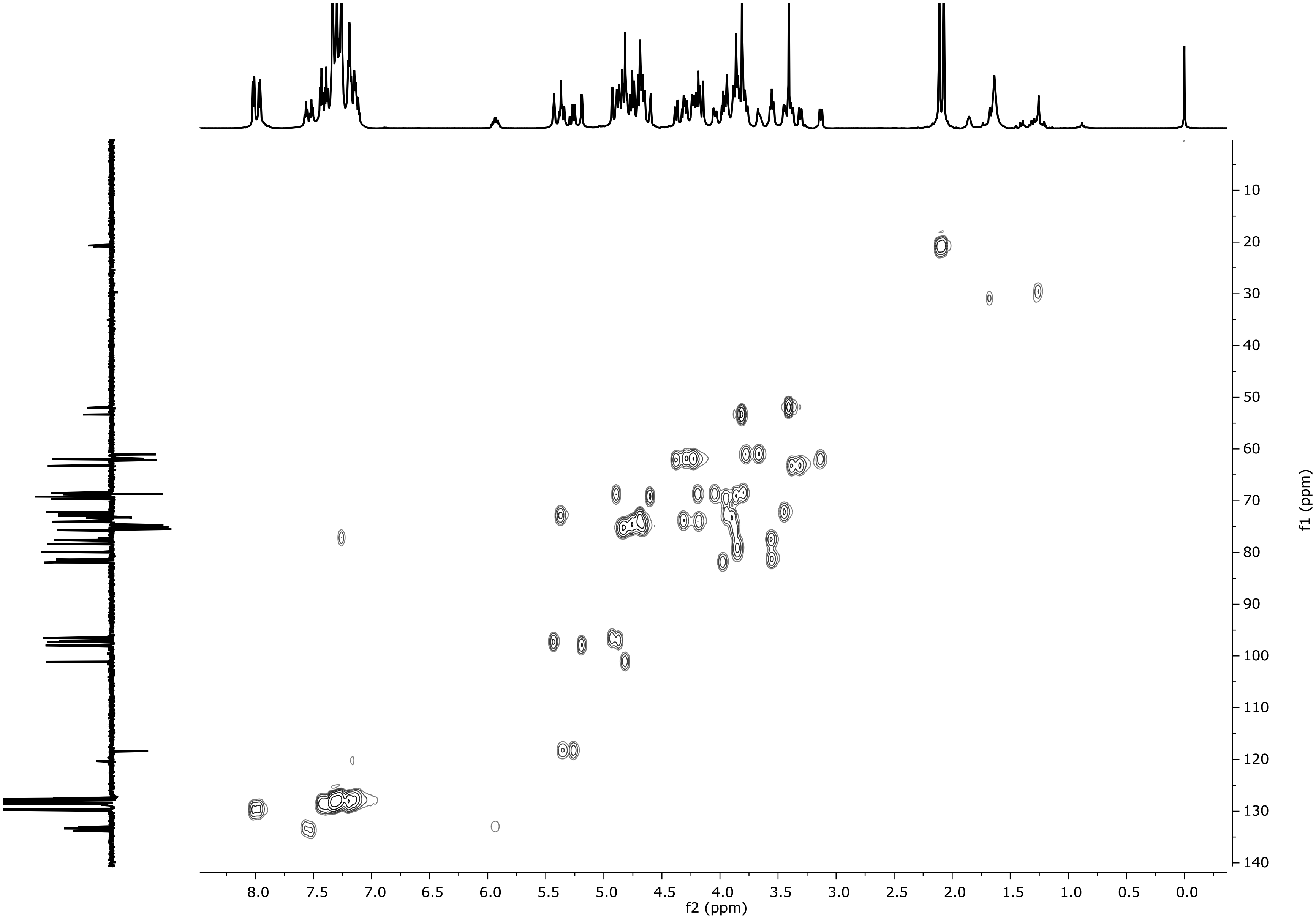

Supplementary Figure S104g. HSQC NMR Spectrum (600.40, 150.99 MHz, $CDCl_3$) of Compound 53 (Sugar region expanded)

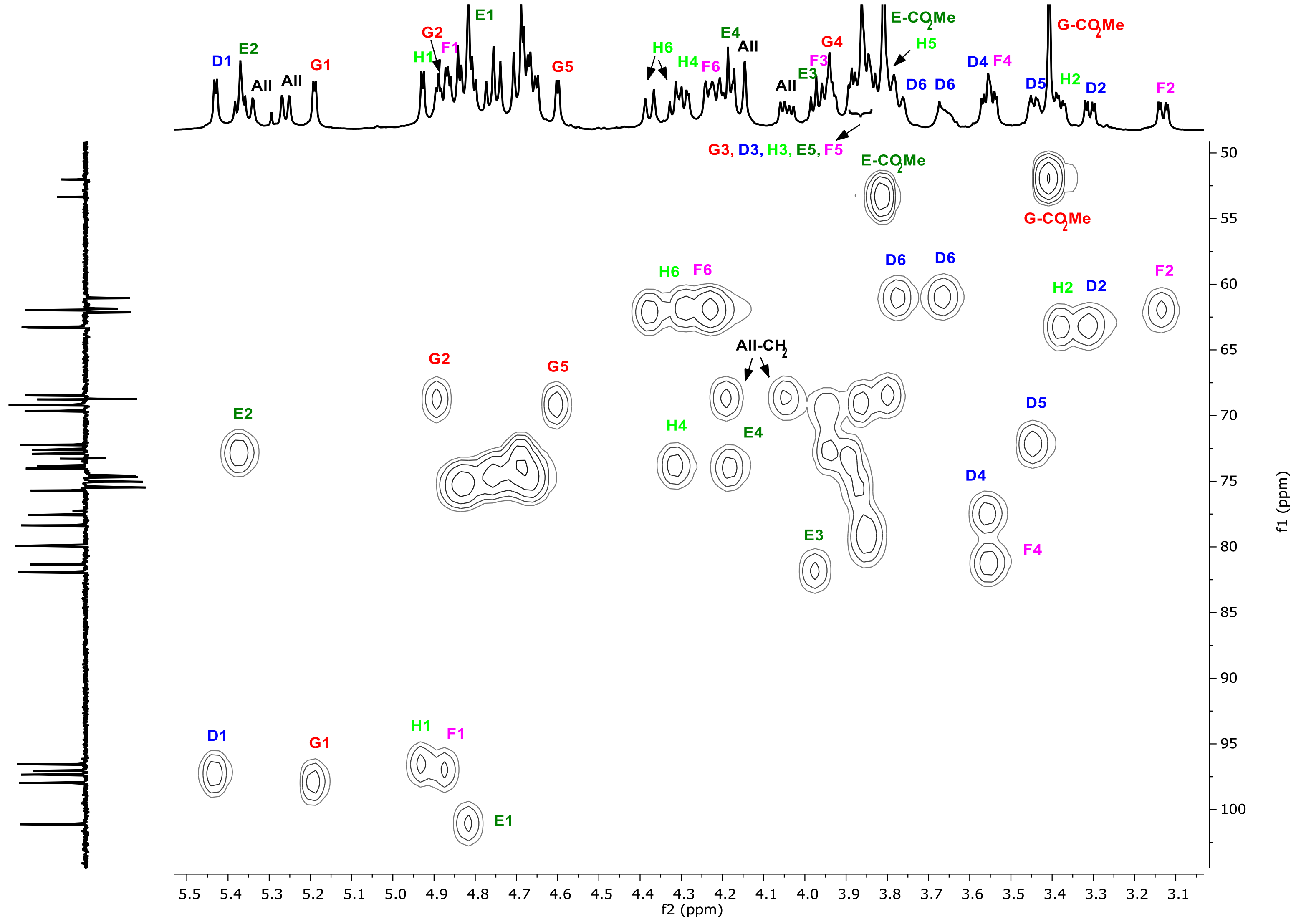

Supplementary Figure S104h. Coupled HSQC NMR Spectrum (600.40, 150.99 MHz, $CDCl_3$) of Compound 53

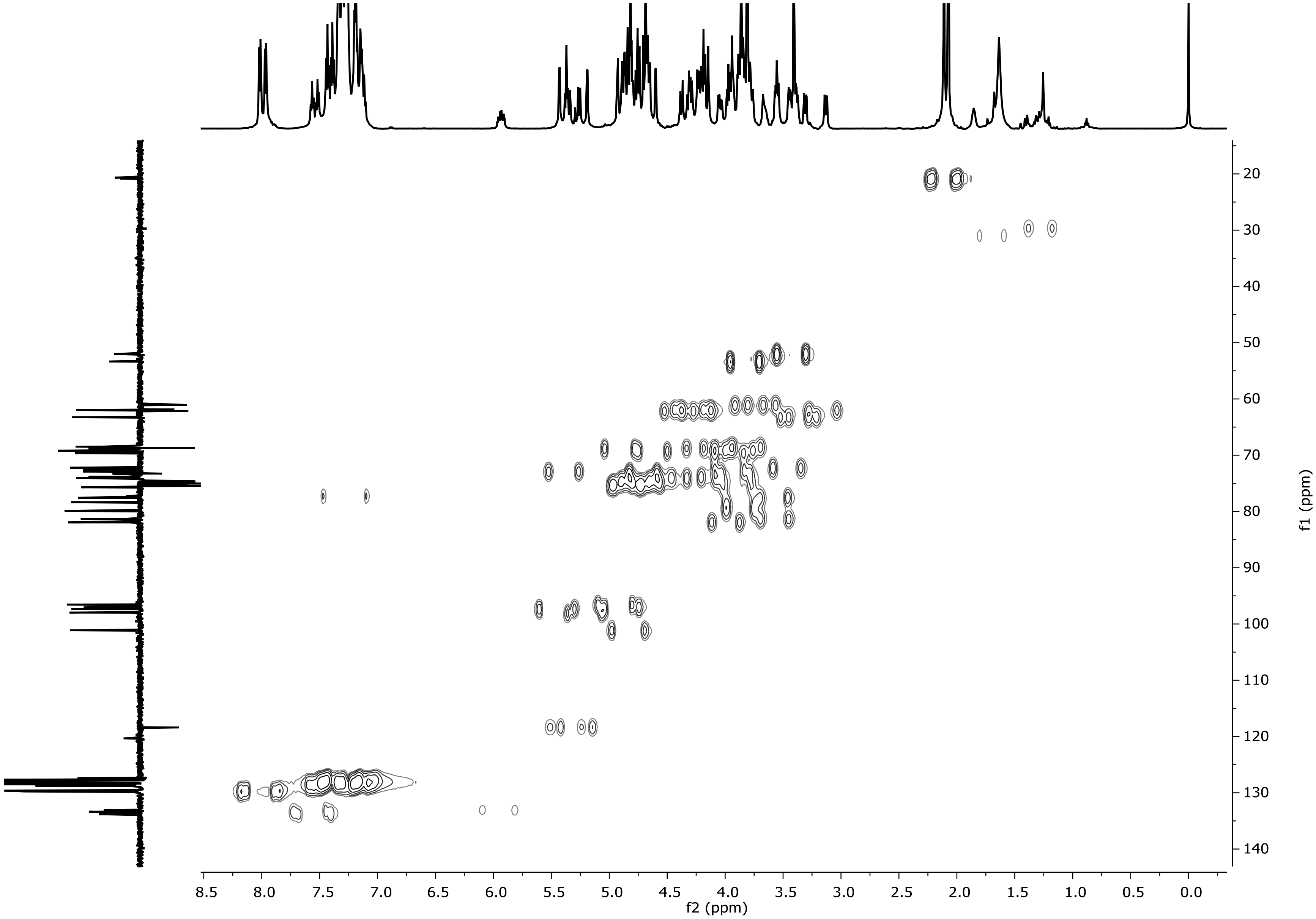

Supplementary Figure S104i. Coupled HSQC NMR Spectrum (600.40, 150.99 MHz, $CDCl_3$) of Compound 53 (Sugar region expanded)

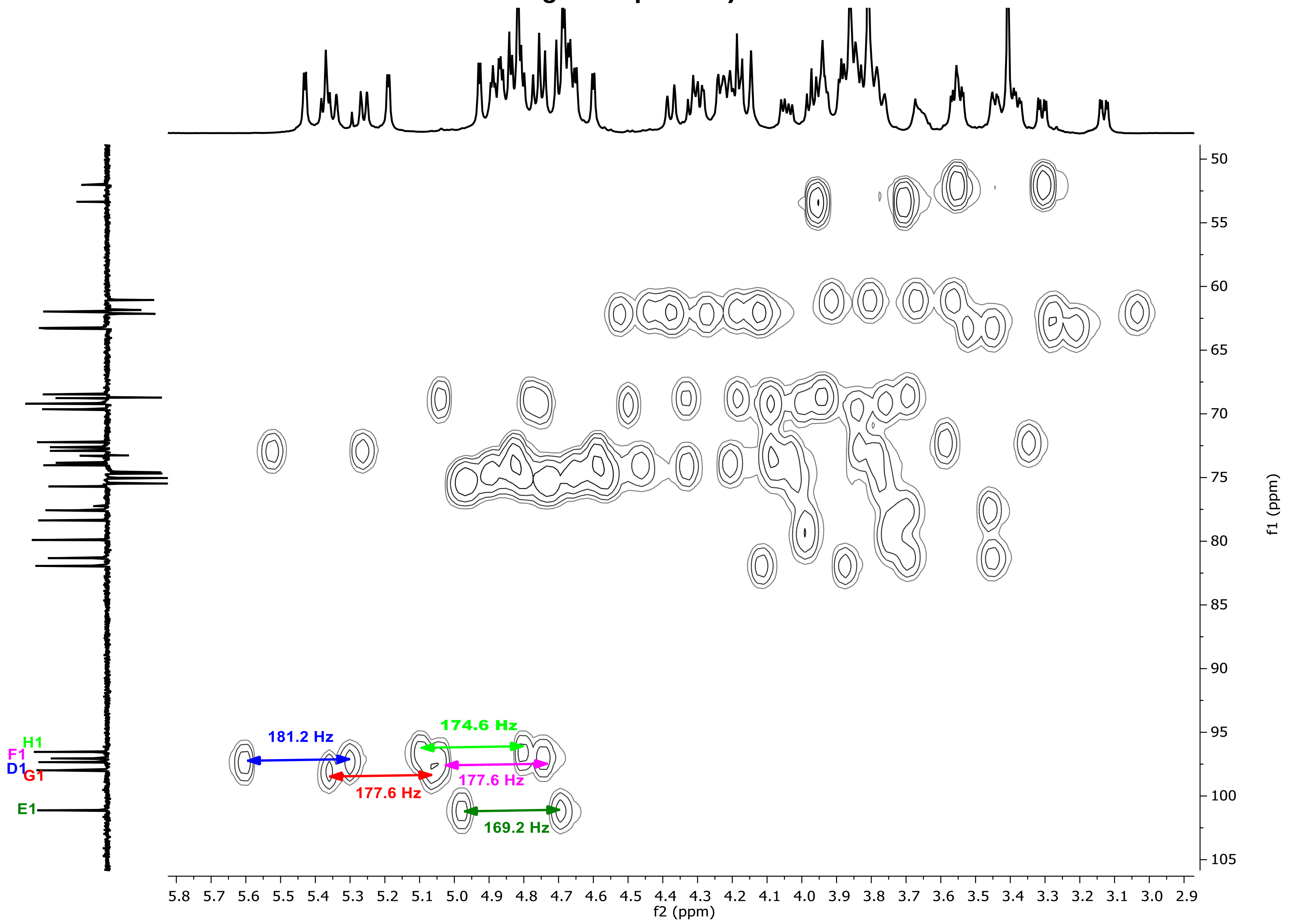

Supplementary Figure S104j. HMBC NMR Spectrum (600.40, 150.99 MHz, $CDCl_3$) of Compound 53

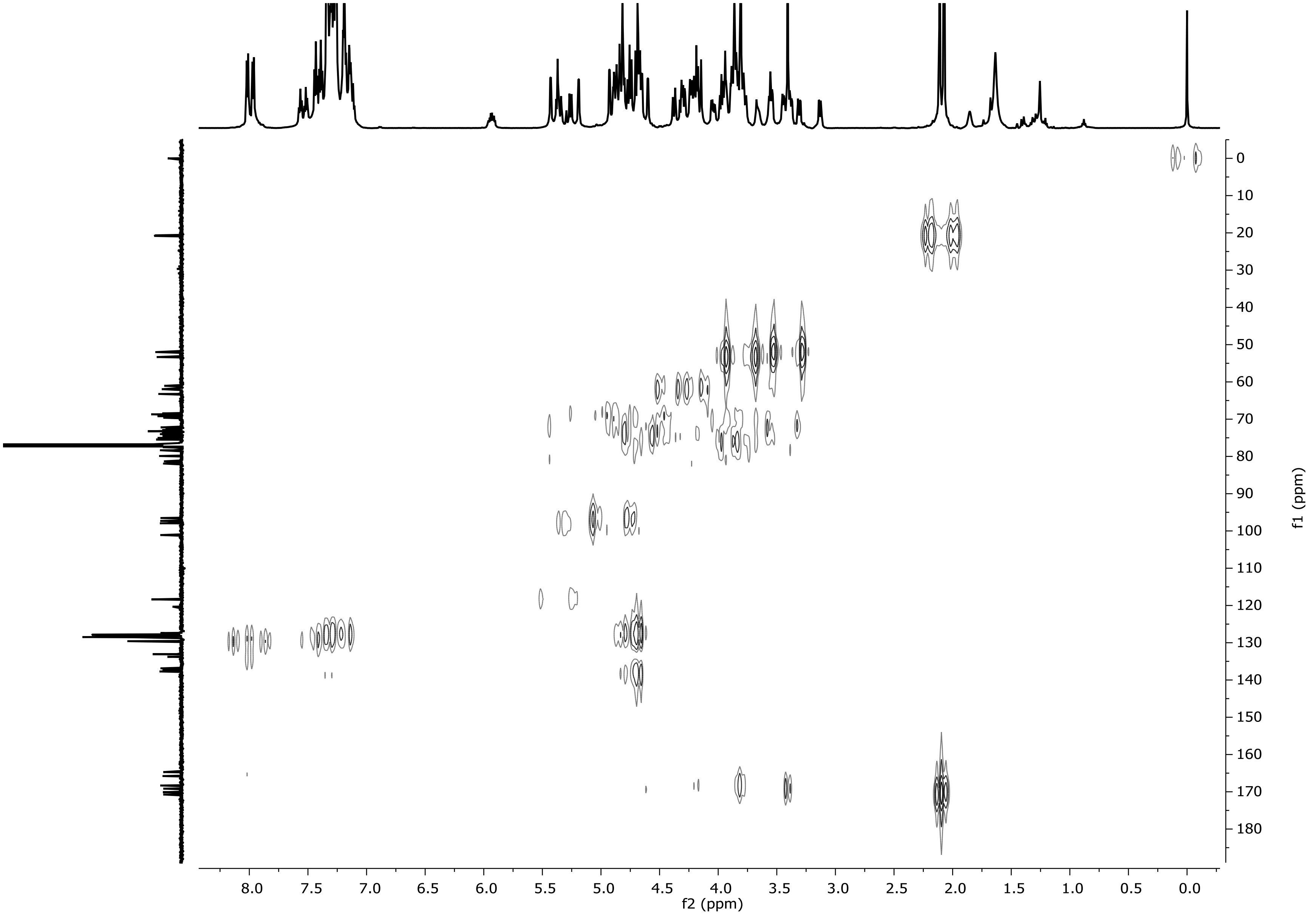

Supplementary Figure S104k. HMBC Spectrum (600.40, 150.99 MHz, $CDCl_3$) of Compound 53 (Sugar region expanded)

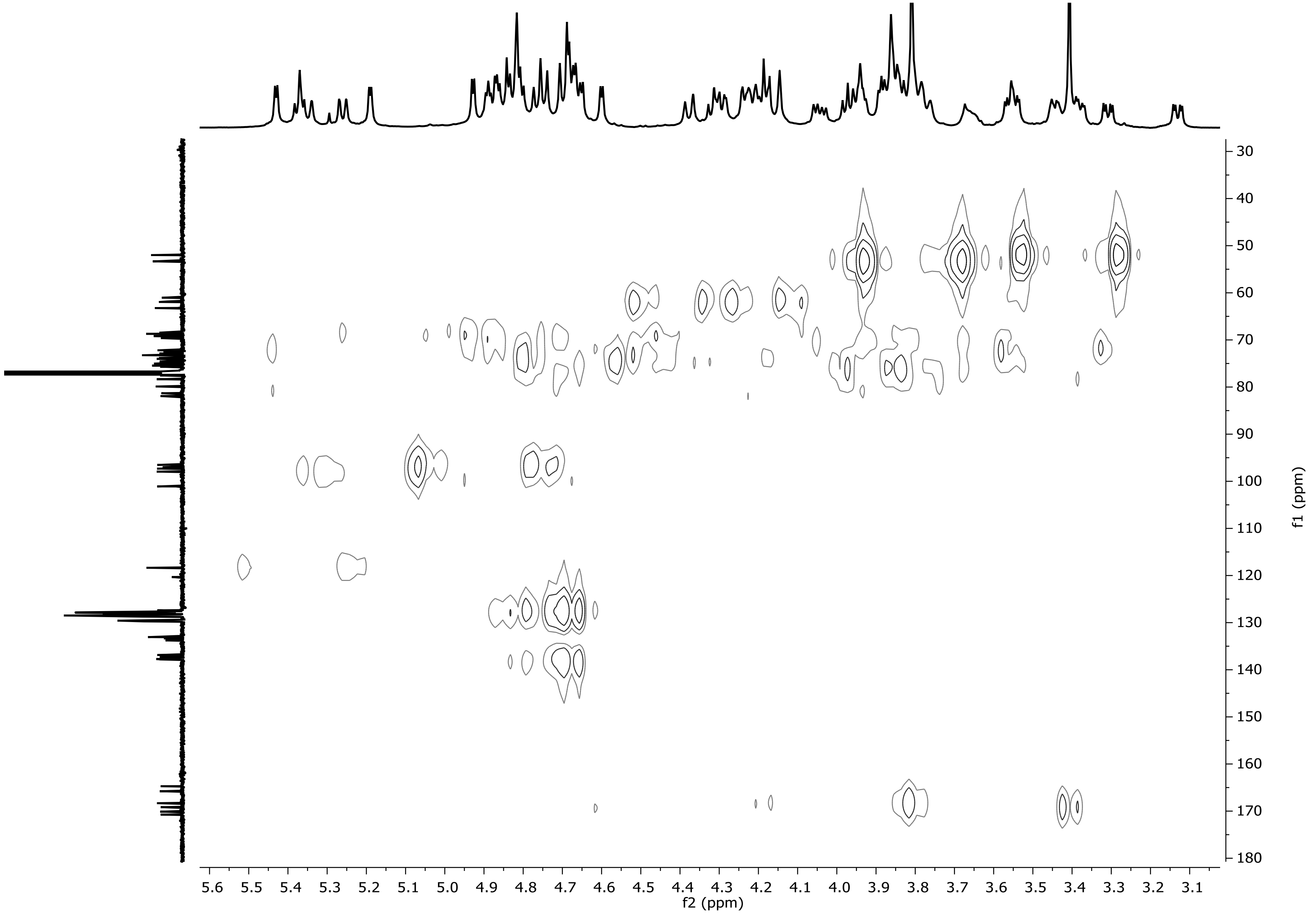

Supplementary Figure S104l. HMBC NMR Spectrum (600.40, 150.99 MHz, $CDCl_3$) of Compound GW-279-2D (Carbonyl region expanded)

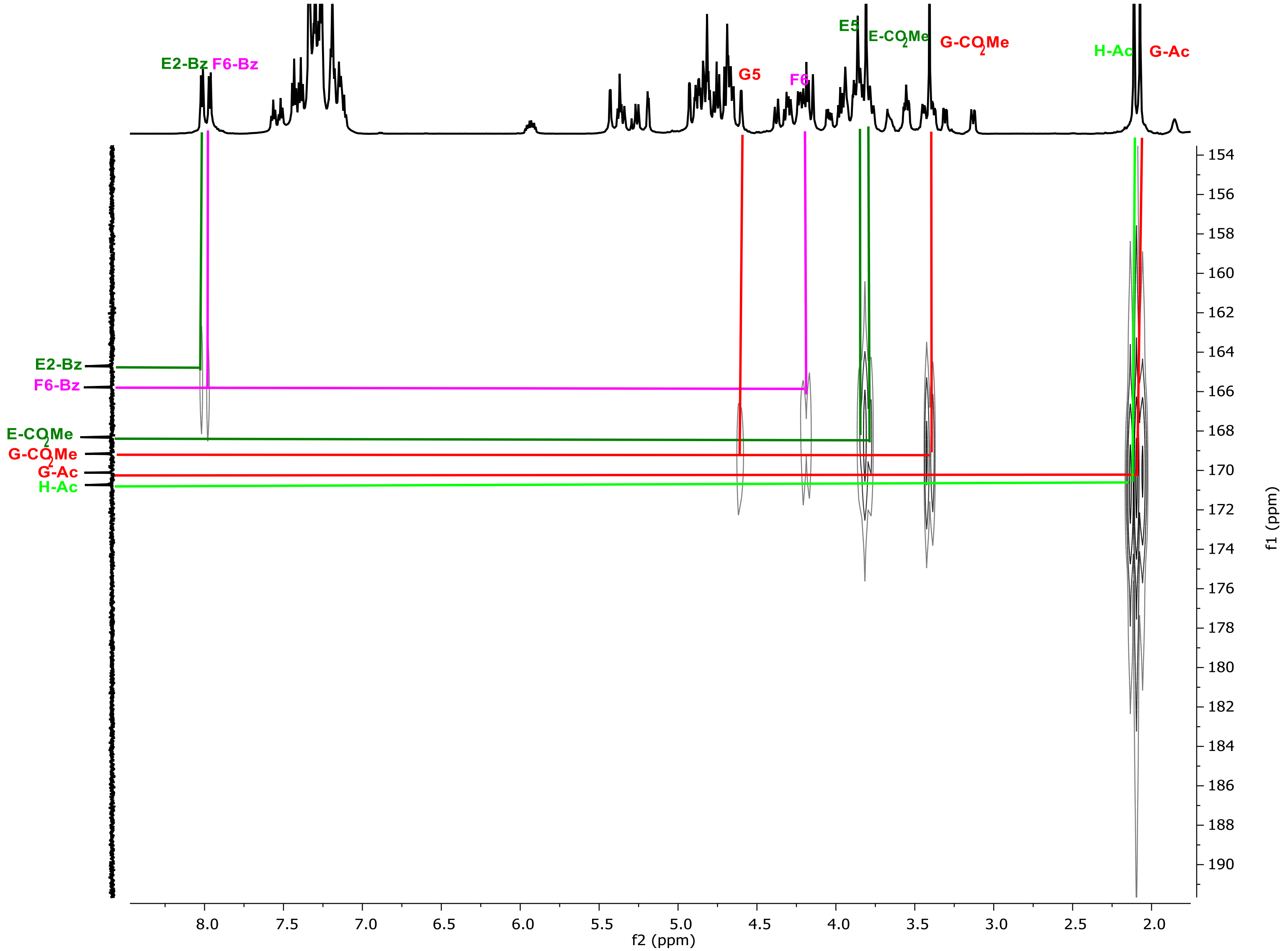

Supplementary Figure S104m. TOCSY NMR Spectrum (600.40, 600.40 MHz, $CDCl_3$) of Compound 53

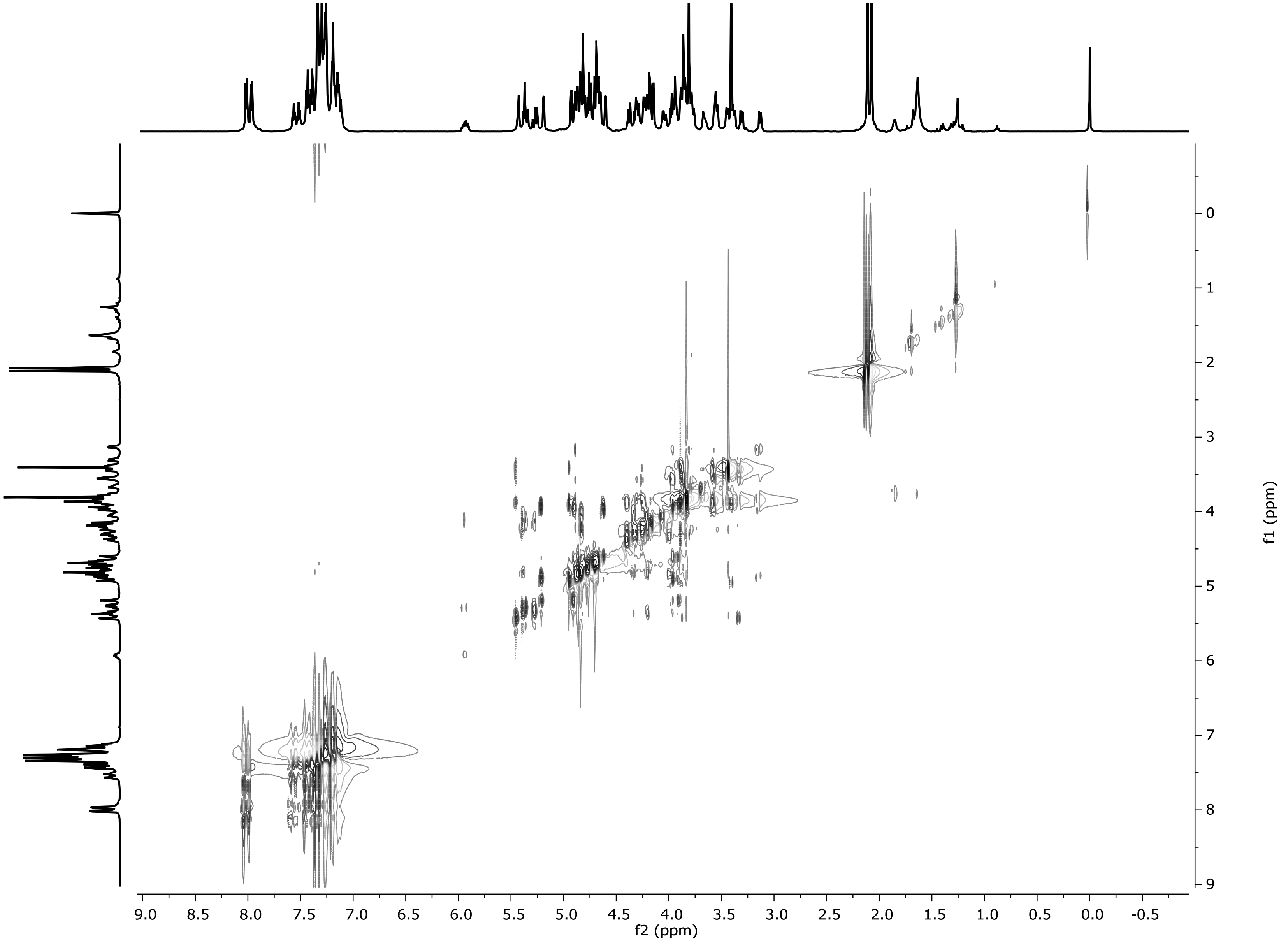

Supplementary Figure S104n. TOCSY NMR Spectrum (600.40, 600.40 MHz, $CDCl_3$) of Compound 53 (Sugar region expanded)

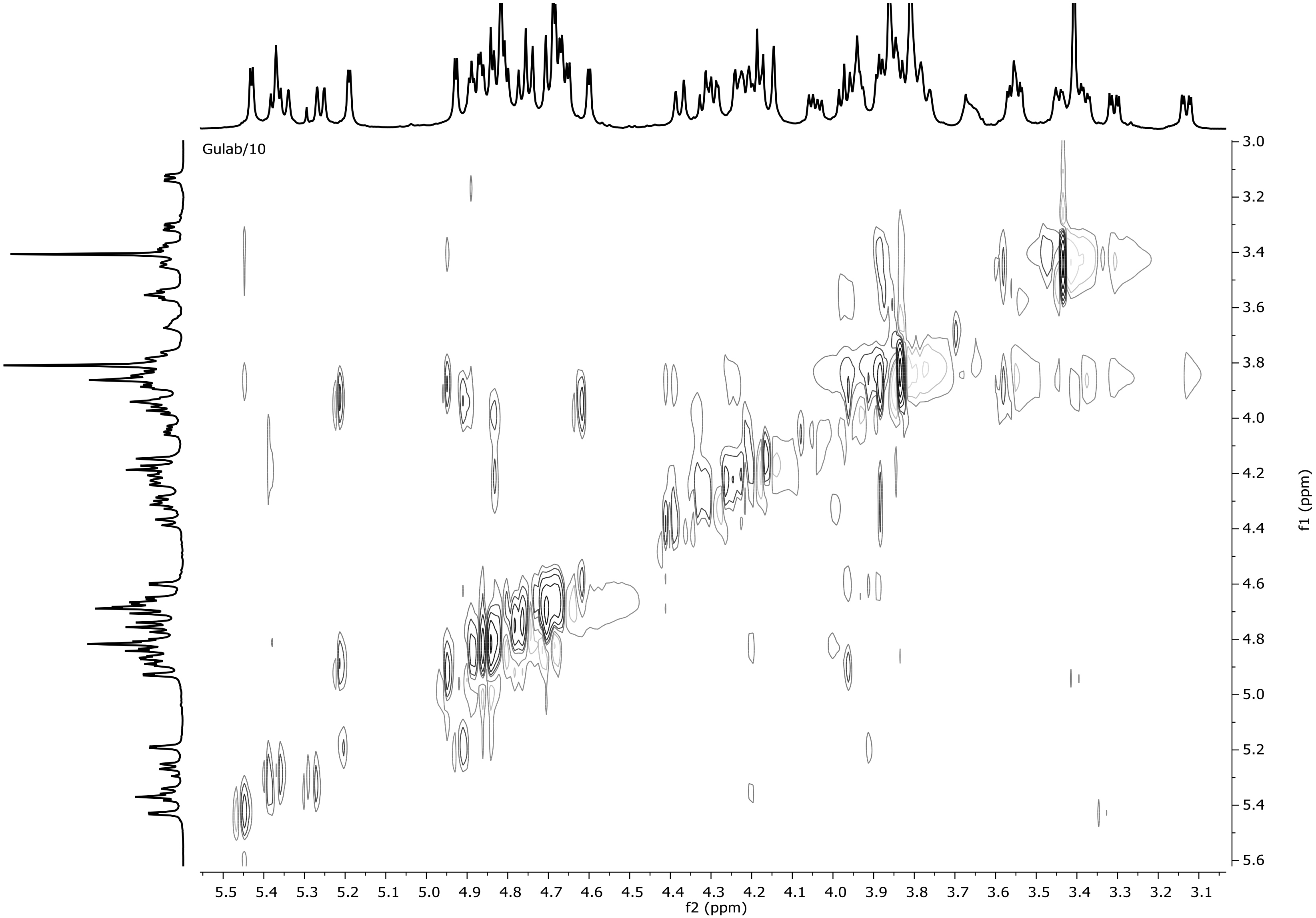

Supplementary Figure S104o. HSQC-TOCSY NMR Spectrum (600.40, 150.99 MHz, $CDCl_3$) of Compound 53

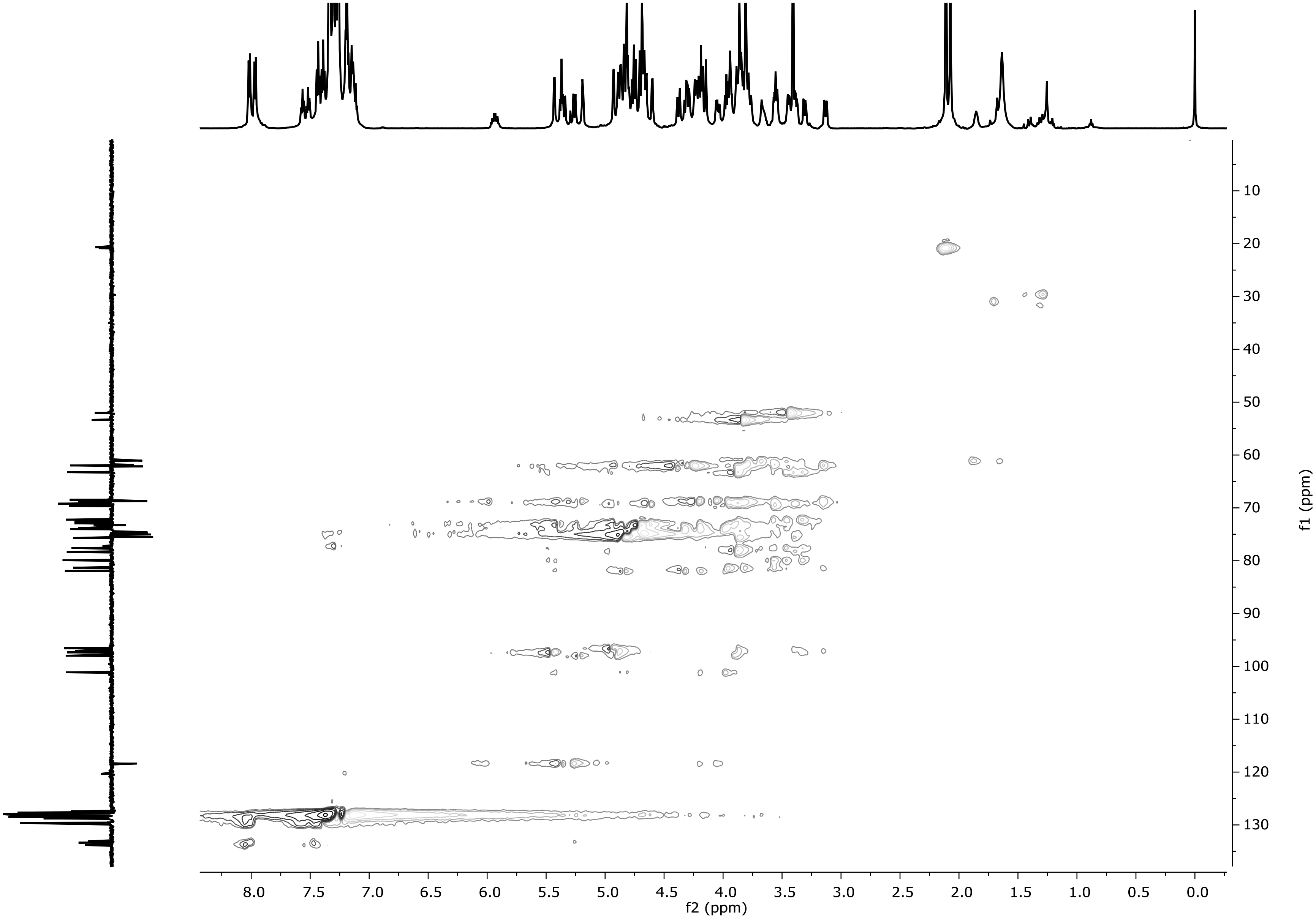

Supplementary Figure S104p. HSQC-TOCSY NMR Spectrum (600.40, 150.99 MHz, $CDCl_3$) of Compound 53 (Sugar region expanded)

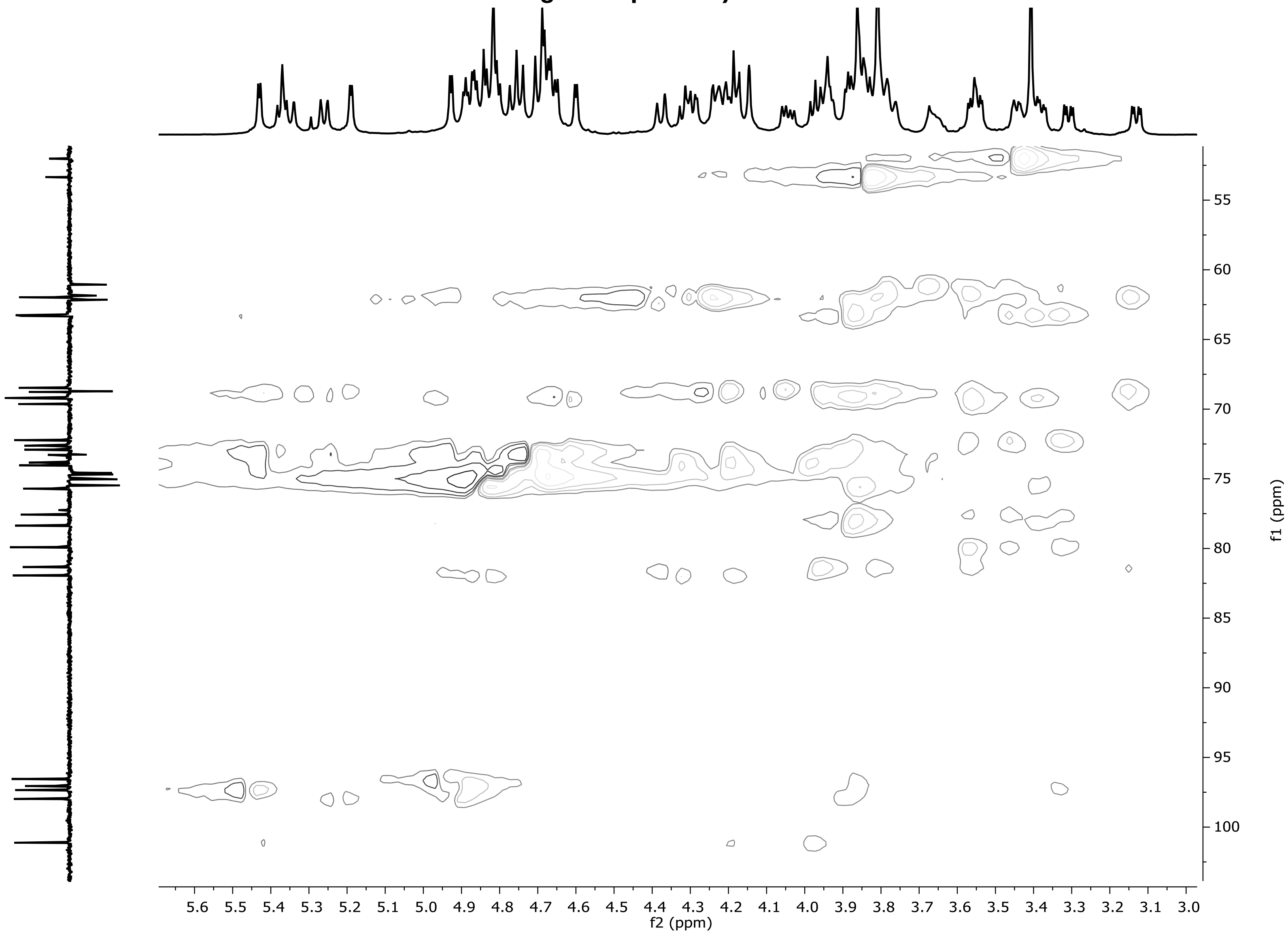

Supplement: Supplementary file 2 — Supplementary Information [file 42004_2021_452_MOESM2_ESM.pdf]
